# *Arabidopsis thaliana* LSM proteins function in mRNA splicing and degradation

**DOI:** 10.1093/nar/gkt296

**Published:** 2013-04-24

**Authors:** Anna Golisz, Pawel J. Sikorski, Katarzyna Kruszka, Joanna Kufel

**Affiliations:** Institute of Genetics and Biotechnology, Faculty of Biology, University of Warsaw, Pawinskiego 5a, 02-106 Warsaw, Poland

## Abstract

Sm-like (Lsm) proteins have been identified in all organisms and are related to RNA metabolism. Here, we report that *Arabidopsis* nuclear AtLSM8 protein, as well as AtLSM5, which localizes to both the cytoplasm and nucleus, function in pre-mRNA splicing, while AtLSM5 and the exclusively cytoplasmic AtLSM1 contribute to 5′–3′ mRNA decay. In *lsm8* and *sad1/lsm5* mutants, U6 small nuclear RNA (snRNA) was reduced and unspliced mRNA precursors accumulated, whereas mRNA stability was mainly affected in plants lacking AtLSM1 and AtLSM5. Some of the mRNAs affected in *lsm1a lsm1b* and *sad1/lsm5* plants were also substrates of the cytoplasmic 5′–3′ exonuclease AtXRN4 and of the decapping enzyme AtDCP2. Surprisingly, a subset of substrates was also stabilized in the mutant lacking AtLSM8, which supports the notion that plant mRNAs are actively degraded in the nucleus. Localization of LSM components, purification of LSM-interacting proteins as well as functional analyses strongly suggest that at least two LSM complexes with conserved activities in RNA metabolism, AtLSM1-7 and AtLSM2-8, exist also in plants.

## INTRODUCTION

The highly conserved Sm-like (Lsm) proteins exist in all kingdoms of life and have been characterized in several eukaryotic organisms, including *Saccharomyces cerevisiae*, *Trypanosoma brucei*, *Xenopus laevis* and *Homo sapiens*. In most reported cases, they participate in different aspects of RNA metabolism [reviewed in (1)]. Similar to Sm proteins, Lsm polypeptides contain specific Sm motifs, which form a characteristic Sm-fold responsible for interactions with proteins and RNA. The canonical Sm proteins form the core particles of the U1, U2, U4 and U5 spliceosomal ribonucleoproteins (RNPs), whereas the related Lsm proteins exist as at least two heptameric complexes. In yeast and humans, the nuclear Lsm2-8 is part of the U6 small nuclear ribonucleoprotein (snRNP) and is involved in pre-mRNA splicing, while cytoplasmic Lsm1-7 proteins participate in the activation of mRNA decapping by the DCP1/2 complex during mRNA decay that also involves 5′–3′ exonuclease XRN1 (2–8). Lsm1-7 proteins are often concentrated in processing bodies (P-bodies), which are cytoplasmic foci containing decapping and 5′–3′ decay factors, where non-translating stable and unstable mRNAs are sequestered [reviewed in (9)].

Additional functions of Lsm proteins include the maturation of stable RNA molecules, rRNA, tRNA and U3 small nucleolar RNA (snoRNA), and the degradation of mRNA precursors in the nucleus (10–16). Moreover, cytoplasmic Lsm proteins have been reported to contribute to mRNA degradation as decapping activators in RNA interference in *Drosophila melanogaster* and uridylation-mediated histone mRNA decay in humans (17,18). Both Lsm complexes are believed to act as chaperones that bind to RNA molecules via oligo(U) or oligo(A) tails to promote RNP formation (5,19,20).

In addition to canonical Lsm complexes, there are also alternative complexes of different composition. Lsm proteins forming the core of the *Xenopus* U8 small nuclear snRNP lack the Lsm5 component, the small nucleolar snR5 particle in yeast only contains the Lsm2-7 subunits, while Lsm1 has not been identified at all in trypanosomes (11,15,21,22). Furthermore, Lsm10/11 proteins in human cells form a part of the U7 RNP, which is involved in the maturation of histone mRNA 3′-ends (23,24).

Bioinformatic analyses of Sm domain proteins have led to the identification of novel Sm family proteins, Lsm12-16, which contain other conserved motifs in addition to the Sm-domain, often with unknown functions (25,26). The roles of these new Lsm proteins are not well characterized, although some members, including *Drosophila* Tra1 (Lsm15), *Caenorhabditis elegans* CAR-1 (Lsm13) and human RAP55 (Lsm14) proteins, have functions related to mRNA translational control [(27) and references therein] or as the yeast decapping activator Edc3 (Lsm16) are required for the formation of P-bodies (28).

Sm and Lsm proteins have also been identified in plants (25,26), but have not yet been properly characterized. A systematic survey of factors involved in pre-mRNA splicing in *Arabidopsis* revealed all Sm and Lsm homologues (29), whereas a more recent genome-wide prediction of the *Arabidopsis* Sm family identified as many as 42 Sm-related genes (30). Specific functions of most of these factors have not been demonstrated, though some of them likely participate in RNA-related processes. The initial analysis of the *Arabidopsis* mutant in *AtLSM4* suggested the involvement of this protein in pre-mRNA splicing (31), whereas DCP5, one of *Arabidopsis* homologues of LSM14, was reported to be required for mRNA decay and translational repression (32). While this work was in revision, two *Arabidopsis* LSM complexes, AtLSM1-7 and AtLSM2-8, have been described to function in mRNA degradation in the cytoplasm and pre-mRNA splicing in the nucleus, respectively (33). Interestingly, a mutant in the *SAD1* gene, the *Arabidopsis* LSM5 counterpart, exhibits hypersensitivity to the plant hormone abscisic acid (ABA), salt and drought, and displays altered expression of some stress-related genes, whereas *lsm4* plants are hypersensitive to salt and ABA (31,34). Similar phenotypes of both mutants suggest an overlapping set of substrates, which contribute to the same physiological processes.

Here, we report the functional characterization of *Arabidopsis* canonical LSM proteins, AtLSM1-8. Our data support the existence of at least two independent LSM complexes, which localize to both the nucleus and the cytoplasm, with AtLSM8 being exclusively present in the nucleus and AtLSM1 in the cytoplasm. Purification of proteins interacting with AtLSM1 and AtLSM8 supports the existence of two separate LSM complexes. Consistently, we observed that AtLSM8 bound U6 small nuclear RNA (snRNA) and its lack affected U6 stability, resulting in the accumulation of unspliced mRNA precursors, whereas plants devoid of AtLSM1 showed stabilization of a subset of mRNAs, in agreement with the function of the nuclear complex in pre-mRNA splicing and the cytoplasmic complex in mRNA turnover. Microarray data, confirmed by northern blot analyses, of mRNAs affected in *lsm1a lsm1b* and *sad1/lsm5* lines revealed that the potential substrates of the 5′-3′ decay pathway overlapped partly with those of the decapping enzyme AtDCP2 and the cytoplasmic exoribonuclease AtXRN4. In turn, the half-lives of some mRNAs were also extended in *lsm8* and *xrn3* plants, suggesting that they were degraded by the nuclear pathway, probably by the 5′–3′ exonuclease AtXRN3.

We conclude that, as in other eukaryotic organisms, plant LSM complexes participate in various aspects of nuclear and cytoplasmic RNA processes that affect specific plant metabolic pathways.

## MATERIALS AND METHODS

### Plant material and growth conditions

*Arabidopsis thaliana* wild-type ecotypes Columbia (Col-0) and WT (34) were used in this study. The following homozygous lines were selected: SALK_106536 (*lsm1a*) with a T-DNA insertion in intron I of the *AtLSM1a* gene (At1g19120); SAIL_756_305 (*lsm1b*) with a T-DNA insertion in intron IV of the *AtLSM1b* gene (At3g14080); SALK_048010 (*lsm8-1*) and SALK_025064 (*lsm8-2*), both with a T-DNA insertion in exon VII of the *AtLSM8* gene (At1g65700). The homozygous *lsm1a lsm1b* double mutant with T-DNA insertions in both *AtLSM1* genes was created by crossing *lsm1a* and *lsm1b* lines, plants were selected by growth on kanamycin and the F2 plants were genotyped by polymerase chain reaction (PCR). The number of T-DNA inserts in *lsm8* and *lsm1a lsm1b* mutants were checked by Southern hybridization. Mutant *sad1/lsm5* line in *AtLSM5* gene (At5g48870) was a kind gift of Jian-Kang Zhu (University of Arizona, USA). Seeds were surface sterilized with 30% bleach/0.02% Triton-X100 solution and grown on Murashige and Skoog (MS) (35) medium supplemented with 1% (w/v) sucrose and 0.3% phytagel, under a 16 h light/8 h dark (long-day) photoperiod. When required, plants were grown under an 8 h light/16 h dark (short-day) photoperiod.

AtLSM1-SF-TAP (2xStrep-FLAG), AtLSM5-SF-TAP and AtLSM8-SF-TAP transgenic lines expressing respective AtLSM proteins under the control of the constitutive 35S promoter from cauliflower mosaic virus (CaMV) were generated by transforming *lsm1a lsm1b*, *sad1/lsm5* and *lsm8* plants with *Agrobacterium tumefaciens* strain GV3101 carrying the pGWB502-AtLSM1-C-SF-TAP, pGWB502-AtLSM5-C-SF-TAP and pGWB502-AtLSM8-C-SF-TAP plasmids, respectively, using the floral-dip method (36). Seeds from *A. tumefaciens*-treated plants were selected on MS plates containing 50 mg/l hygromycin and hygromycin-resistant plantlets were transferred to soil.

The plant binary vector pGWB502-C-SF-TAP was obtained by cloning a C-terminal SF-TAP sequence amplified from pDEST/C-SF-TAP (37) into the BglII site of the pGWB502 (38). *AtLSM1, AtLSM5* and *AtLSM8* cDNAs were cloned into SalI and EcoRV sites of the pENTR1A vector in and these constructs were used for LR recombination reactions (Invitrogen, http://www.invitrogen.com) with the pGWB502-C-SF-TAP to generate C-terminal SF-TAP fusions. These constructs allow for the expression of SF-TAP–tagged proteins under the control of the constitutive 35S CaMV promoter.

Plasmids used in this study are listed in Supplementary Table S1.

### Yeast methods

Yeast strains are listed in Supplementary Table S1. Construction of yeast plasmids, strains and growth conditions are described in Supplemental Material.

### Sequence analysis

Sequences of LSM proteins were aligned using T-Coffee software (39). The phylogenetic analysis was performed using Phyml (40), following the removal of poorly aligned and overly divergent positions using the Gblocks program (41). The unrooted tree was drawn with Treedyn (42). Sequence identity and similarity was obtained using BLAST2 sequence search (43).

### RNA methods

Total RNA was isolated from 2-week-old seedlings using Trizol reagent (Sigma) according to the manufacturer’s instructions. Low-molecular weight RNAs were separated on 6% acrylamide/7M urea gels and transferred to a Hybond N+ membrane (GE Healthcare) by electrotransfer. High-molecular-weight RNAs were analysed on 1.1% agarose/6% formaldehyde gels and transferred to a Hybond N+ membrane by capillary elution. Northern blots were performed using γ-^32^P 5′-end-labelled oligonucleotide probes or random primed probes amplified on a cDNA template using respective primers and the DECAprime™ II labelling kit (Ambion). Quantification of northern blots was performed using a Storm 860 PhosphorImager (GE Healthcare) and ImageQuant software (Molecular Dynamics). mRNA half-life measurement experiments were carried out as described (44). Two-week-old seedlings were transferred to flasks containing a buffer (1 mM PIPES, pH 6.25, 1 mM sodium citrate, 1 mM KCl, 15 mM sucrose), and after a 30-min incubation, cordycepin (150 mg/l) was added. Total RNA samples corresponding to 0, 15, 30, 60, 90 and 120 min time points after transcriptional inhibition were extracted using Trizol reagent and analysed by either northern blot or microarrays.

Oligonucleotides used for northern hybridization, PCR reactions and for generating random primed probes are listed in Supplementary Table S2.

### Immunoprecipitation

RNA immunoprecipitation was performed as described (45) with the following modifications. Three grams of 2-week-old seedlings was cross-linked with 1% formaldehyde in phosphate buffered saline (PBS) two times for 10 min by vacuum infiltration, followed by addition of glycine to 80 mM final concentration. Plants were rinsed with water, frozen in liquid nitrogen, ground into powder using a mortar and pestle, mixed with Buffer I [10 mM Tris–HCl, pH 8.0, 10 mM MgCl_2_, 0.4 M sucrose, 5 mM β-mercaptoethanol, 1 mM PMSF, protease inhibitor cocktail (Roche), RiboLock RNase Inhibitor (ThermoFisher Scientific)], filtered through two layers of Miracloth and centrifuged at 4°C for 20 min at 5000*g*. The nuclear pellet was resuspended in 1 ml of Buffer II (10 mM Tris–HCl, pH 8.0, 10 mM MgCl_2_, 0.4 M sucrose, 1% Triton X-100, 5 mM β-mercaptoethanol, 1 mM PMSF, protease inhibitor cocktail, RiboLock RNase Inhibitor) and was centrifuged at 4°C for 10 min at 12 000*g*. The pellet was washed three times in Buffer II, resuspended in 300 µl of Buffer II, layered on top of 900 µl of Buffer III (10 mM Tris–HCl, pH 8.0, 2 mM MgCl_2_, 1.7 M sucrose, 0.15% Triton X-100, 5 mM β-mercaptoethanol, 1 mM PMSF, protease inhibitor cocktail, RiboLock RNase Inhibitor) and centrifuged at 4°C for 60 min at 16 000g. The nuclear pellet was resuspended in Nuclei Lysis Buffer (50 mM Tris–HCl, pH 8.0, 10 mM EDTA, 1% sodium dodecyl sulphate (SDS), 1 mM PMSF, protease inhibitor cocktail, RiboLock RNase Inhibitor) and sonicated twice on ice using 15 × 30 s bursts (Bioruptor). After centrifugation at 4°C for 5 min at 3000*g*, the supernatant was diluted 10-fold with ChIP Dilution Buffer (16.7 mM Tris–HCl, pH 8.0, 167 mM NaCl, 1.1% Triton X-100, 1.2 mM EDTA, 1 mM PMSF, protease inhibitor cocktail, RiboLock RNase Inhibitor). One-tenth of the diluted nuclear extract was saved as an input fraction. Immunoprecipitation on diluted nuclear extract from 40 µg of chromatin was performed using 30 µl of anti-FLAG-M2 resin (Sigma) pre-washed three times in ChIP Dilution Buffer. Samples were incubated for 3 h at 4°C on a rotating mixer, washed once for 5 min with Low Salt Wash Buffer (20 mM Tris–HCl, pH 8.0, 150 mM NaCl, 0.1% SDS, 1% Triton X-100, 2 mM EDTA, RiboLock RNase Inhibitor), once for 5 min with High Salt Wash Buffer (20 mM Tris–HCl, pH 8.0, 500 mM NaCl, 0.1% SDS, 1% Triton X-100, 2 mM EDTA, RiboLock RNase Inhibitor), once for 5 min with LiCl Wash Buffer (10 mM Tris–HCl, pH 8.0, 250 mM LiCl, 1% Igepal CA-630, 1 mM EDTA, 1% sodium deoxycholate, RiboLock RNase Inhibitor) and twice for 5 min with TE Buffer (10 mM Tris–HCl, 1 mM EDTA, RiboLock RNase Inhibitor). Bound RNA–protein complexes were eluted with 50 µl RIP Elution Buffer (10 mM Tris–HCl, 1 mM EDTA, 1% SDS, RiboLock RNase Inhibitor) at room temperature for 10 min on a rotating mixer, followed by centrifugation for 5 min at 14 000*g*. The elution was repeated in 50 µl of RIP Elution Buffer for 10 min at 65°C with gentle agitation. Both elution fractions were pooled and incubated with 1 µl of 20 mg/ml proteinase K (Bioline) for 40 min at 65°C with gentle agitation. RNA was isolated using RNeasy MinElute Cleanup Kit (Qiagen) and treated with DNase I (Invitrogen). RNA samples, 500 ng of the input fraction and 60 ng of the IP fraction, were reverse transcribed for 60 min at 55°C with random primers and SuperScript III Reverse Transcriptase (Invitrogen) and used for PCR amplification.

Immunoprecipitation of m^7^G-capped RNA was performed with 5 µg of monoclonal H20 antibodies (anti-m^7^G and TMG cap) and 10 µl of Dynabeads Protein G (Invitrogen) in IPP150 buffer (10 mM Tris, pH 7.5, 150 mM NaCl, 0.1% Nonidet P-40, RiboLock RNase Inhibitor) for 3 h at 4°C on 1 µg of total RNA from 2-week-old seedlings treated with cordycepin for 15 min. Both the unbound RNA fraction in the supernatant and the cap-bound RNAs were recovered by phenol-chloroform extraction and ethanol precipitation and analyzed by quantitative reverse-transcription PCR (qRT-PCR). The fraction of immunoprecipitated RNA was expressed relative to the supernatant plus immunoprecipitate.

### Cell fractionation and western blotting

Nuclei were isolated as described (46). Frozen 2-week-old seedlings were ground in liquid nitrogen using a mortar and pestle, and homogenized in Honda buffer (2.5% Ficoll 400, 5% Dextran T40, 0.4 M sucurose, 25 mM Tris–HCl, pH 7.4, 10 mM MgCl_2_, 10 mM β-mercaptoethanol, 0.5 mM PMSF, protease inhibitor cocktail). The homogenate was filtered through a 62 µm nylon mesh, Triton X-100 was added to a final concentration of 0.5% and incubated on ice for 15 min. An aliquot of total extract was retained for further analysis, and the remainder was centrifuged at 1500*g* for 5 min. The supernatant was saved as a cytoplasmic fraction, and the pellet was washed twice with Honda buffer, supplemented with 0.1% Triton X-100 for the first wash. Nuclei were pelleted by centrifugation at 1500*g* for 5 min and analysed by sodium dodecyl sulphate-polyacrylamide gel electrophoresis and western blotting.

Western blotting was performed using mouse monoclonal antibody anti-FLAG (M2) (Sigma-Aldrich) to detect AtLSM1-SF-TAP, AtLSM5-SF-TAP and AtLSM8-SF-TAP or specific polyclonal rabbit anti-H3 (Abcam) and anti-PEPC (Rockland) antibodies. Anti-mouse (Santa Cruz Biotechnology) or anti-rabbit (Sigma Aldrich) horseradish peroxidase-conjugated antisera were used as secondary antibodies.

### Affinity protein purification and mass spectrometry

Twenty grams of 2-week-old seedlings was cross-linked with 1% formaldehyde in PBS, two times for 10 min by vacuum infiltration, followed by addition of glycine to a final concentration of 80 mM. Seedlings were rinsed with water, frozen in liquid nitrogen, ground in a laboratory blender (Waring) with dry ice (10 × 30 s), mixed with an equal amount (w/v) of Extraction buffer (100 mM Tris–HCl, pH 8.0, 150 mM NaCl, 2 mM EDTA, 2 mM DTT, 1 mM PMSF, 0.5% Triton X-100 and protease inhibitor cocktail) and sonicated on ice using 4 × 30 s bursts (Bioruptor). The homogenate was centrifuged at 20 000*g* for 40 min at 4°C and the supernatant was further centrifuged at 35 000*g* for 1 h at 4°C. The supernatant was concentrated approximately four times by dialysis against PEG 20 000 at 4°C and then dialyzed for 3 h at 4°C in Dialysis buffer (50 mM Tris–HCl, pH 8.0, 150 mM NaCl, 1 mM EDTA, 1 mM DTT, 1 mM PMSF, 20% glycerol). The protein extract was supplemented with 250 µg/ml RNase A (Qiagen) and incubated for 3 h at 4°C with slow rotation with 200 µl anti-FLAG-M2 resin pre-washed in Wash buffer (50 mM Tris–HCl, pH 8.0, 150 mM NaCl, 1 mM EDTA, 1 mM DTT, 0.1% Triton X-100). The flow-through fraction was then separated by centrifugation for 1 min in 4°C at 1000*g*. The resin was washed 3–4 times with 2 ml of Wash buffer and twice with TBS (50 mM Tris–HCl, pH 8.0, 150 mM NaCl, 1 mM DTT) in polyprep chromatography columns (10 ml, BioRad). Bound proteins were eluted overnight by incubation with 250 µg/ml of the FLAG peptide in TBS at 4°C. The eluate was concentrated up to a final volume of 20 µl using the SpeedVac (Christ). Each purification was repeated three times.

The eluate was supplemented with 100 mM ammonium bicarbonate to a final volume of 50 µl, reduced with 10 mM DTT for 30 min at 56°C and alkylated with 50 mM iodoacetamide for 45 min in the dark at room temperature. The alkylating agent was eliminated using 50 mM DTT. The protein mixture was digested overnight with trypsin at 1:10 ratio at 37°C. The sample was then treated with trifluoroacetic acid to obtain pH <4. The sample was concentrated and desalted on a RP-C18 pre-column (Waters), and further subjected to nano-Ultra Performance Liquid Chromatography (UPLC) RP-C18 column (Waters) of a nanoAcquity UPLC system (Waters) coupled to an electrospray ionization source and LTQ Orbitrap Velos mass spectrometer (ThermoFisher Scientific). Tandem mass spectra were searched against TAIR10 non-redundant database using Mascot (Matrix Science) and filtering criteria, which provided a false discovery rate <5%. For further analysis, proteins with a score ≥50 were selected and which were present in at least two of three independent purifications.

### Microarray analysis

Genome-wide expression and mRNA stability profiles in *lsm1a lsm1b*, *lsm8* and *sad1/lsm5* mutants and their corresponding wild types [Col-0 ecotype for *lsm1a lsm1b* and *lsm8;* WT ecotype for *sad1/lsm5* (34)] were performed in biological duplicates on total RNA from seedlings treated with cordycepin (150 mg/l) using Affymetrix ATH1 Gene Chip microarrays exactly as described (47). Time points after transcriptional inhibition, based on experimental optimization, were collected at 0 and 30 min for *lsm1a lsm1b* plants; 0, 30 and 90 min for *lsm8* plants; or 0, 30 and 120 min for *sad1/lsm5* plants. RNA samples (15 µg) were used for library preparation and microarray hybridization performed at the DNA Microarrays Genomics Core Facility (EMBL, Heidelberg). The resulting raw Affymetrix expression values from replicate experiments in the form of CEL files were normalized by Robust Multichip Analysis (RMA Express software) (48,49). The RMA model logarithmically transforms probe intensities, adjusts background, performs cross-chip normalization and uses robust linear models to summarize probe-level expression values. Separate experiments were created for each mutant, and the averaged data from normalized CEL files were expressed as mutant to wild-type signals. Genes with ratios that differed by at least 10% were selected as downregulated (<0.9 values) or upregulated (>1.1 values). Affymetrix Annotations were used for assignment of gene names and gene products description. Gene Ontology functional analysis was performed using the PageMan tool [http://mapman.mpimp-golm.mpg.de/index.shtml; (50)]. Microarray results were visualized by Venn diagrams generated using http://bioinfogp.cnb.csic.es/tools/venny/index.html.

### Real-time PCR

cDNA was prepared for 60 min at 55°C with 10xRT Random Primers (Applied Biosystems) or a mix of oligo(dT)_20_ and 10xRT Random Primers using a SuperScript® III Reverse Transcriptase Kit (Sigma) on 10 µg of total RNA treated with RNase-free TURBO DNase (Ambion) for 60 min at 37°C. qPCR was carried out using LightCycler® 480 SYBR Green I Master (Roche). The tubulin (At5g62690) or actin (At2g37620) genes were used for normalization of relative expression values. All reactions were run in three independent biological replicates.

## RESULTS

### Identification of LSM proteins in *A.thaliana*

Protein BLAST (http://www.ncbi.nlm.nih.gov) searches of the *Arabidopsis* genome for homologues of yeast and human LSM proteins revealed the existence of all AtLSM1-8 protein-coding genes, with *AtLSM1*, *AtLSM3* and *AtLSM6* being duplicated (Table 1). The identified genes were identical to those previously reported (29,30).

**Table 1.** *Arabidopsis* LSM orthologs

Protein	ID	Identity (%)				Similarity (%)			
		Hs	Sc	Dm	Os	Hs	Sc	Dm	Os
AtLSM1a	At1g19120	53	42	59	76	69	68	74	87
AtLSM1b	At3g14080	53	43	57	80	71	67	74	92
AtLSM2	At1g03330	76	67	78	92	86	78	86	98
AtLSM3a	At1g21190	80	43	75	90	94	65	94	99
AtLSM3b	At1g76860	80	48	78	94	92	67	92	98
AtLSM4	At5g27720	87	40	77	79	96	58	87	82
AtLSM5	At5g48870	75	45	70	94	88	67	86	95
AtLSM6a	At2g43810	85	43	77	89/90^a^	93	52	87	95/96^a^
AtLSM6b	At3g59810	85	41	82	89/90^a^	91	52	88	91/92^a^
AtLSM7	At2g03870	55	47	61	90	79	70	82	96
AtLSM8_short	At1g65700	62	28	61	84	80	53	78	92
AtLSM8_long	At1g65700	43	26	42	58	55	49	53	64

Hs, *H.sapiens*; Sc, *S.cerevisiae*; Dm, *D. melanogaster*; Os, *Oryza sativa*.

^a^Two LSM6 proteins encoded in the *Oryza* genome.

Sequence alignment and phylogenetic comparison of eukaryotic LSM proteins showed that plant LSMs constitute a separate subgroup and individual proteins were much more similar to their human and *Drosophila* counterparts than to yeast ones (Table 1; Supplementary Figures S1 and S2). The analysis of the amino acid sequence of each AtLSM protein confirmed the presence of conserved Sm1 and Sm2 motifs (Supplementary Figure S1). The expression of individual *Arabidopsis LSM* genes in *S. cerevisiae* strains lacking corresponding Lsm proteins (4) did not compensate for the growth defects of yeast *lsm* mutants (Supplementary Figure S3). Thus, each single *Arabidopsis* LSM protein cannot functionally replace its yeast equivalent, which is consistent with the phylogenetic analysis.

**Figure 1.**
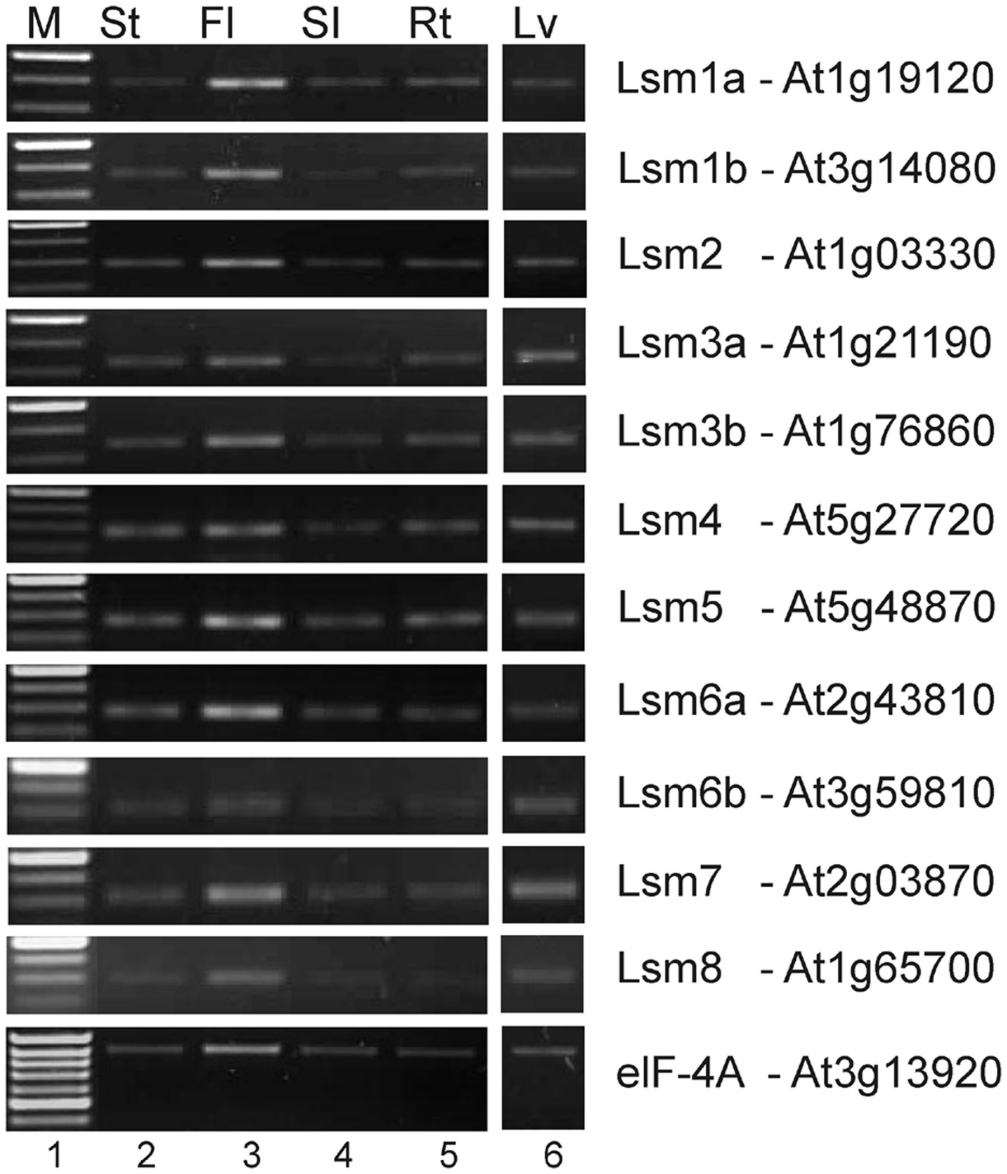
Expression of *AtLSM* genes in *A. thaliana*. Semi-quantitative RT-PCR analysis of AtLSM in leaves (Lv), stems (St), flowers (Fl), siliques (Sl) and roots (Rt). For *AtLSM8*, both short and long mRNA versions are detected. M, DNA ladder. Elongation factor elF-4A was used as a control.

We used semi-quantitative reverse-transcription PCR (RT-PCR) to detect *LSM* mRNAs in *Arabidopsis* organs and observed that all the *AtLSM* genes were expressed in leaves, flowers, stems, siliques and roots (Figure 1). The presence of *AtLSM* transcripts in every tissue tested at all developmental stages was further corroborated by checking their expression profiles using the Genevestigator tools (https://www.genevestigator.ethz.ch/at/) (51) (Supplementary Figure S4).

Two alternatively-spliced mature *AtLSM8* mRNA forms, giving rise to different open reading frames, were predicted according to the *Arabidopsis* Information Resource database (www.arabidopsis.org), with the longer one containing short predicted exons 4 and 5 encoding 43 amino acids, and the shorter one lacking these exons. Quantitative reverse-transcription PCR revealed that both forms were expressed in wild-type seedlings, albeit at a markedly different level, with the longer variant being 52-fold less abundant than the shorter one (Supplementary Figure S5). Notably, this difference was even greater in different organs (73-fold in leaves, 105-fold in flowers and 129-fold in roots; Supplementary Figure S5). Sequence alignment of the longer gene product with those of other available LSM8 proteins demonstrated a discernible mismatch, with additional residues present in this variant (see Supplementary Figure S1). This suggested that the longer mRNA, and probably also the protein it encodes, represented a rare form and hence it was not further analysed in this work. However, it may play a yet undefined role in some specific conditions.

### Subcellular localization of LSM proteins

**Figure 2.**
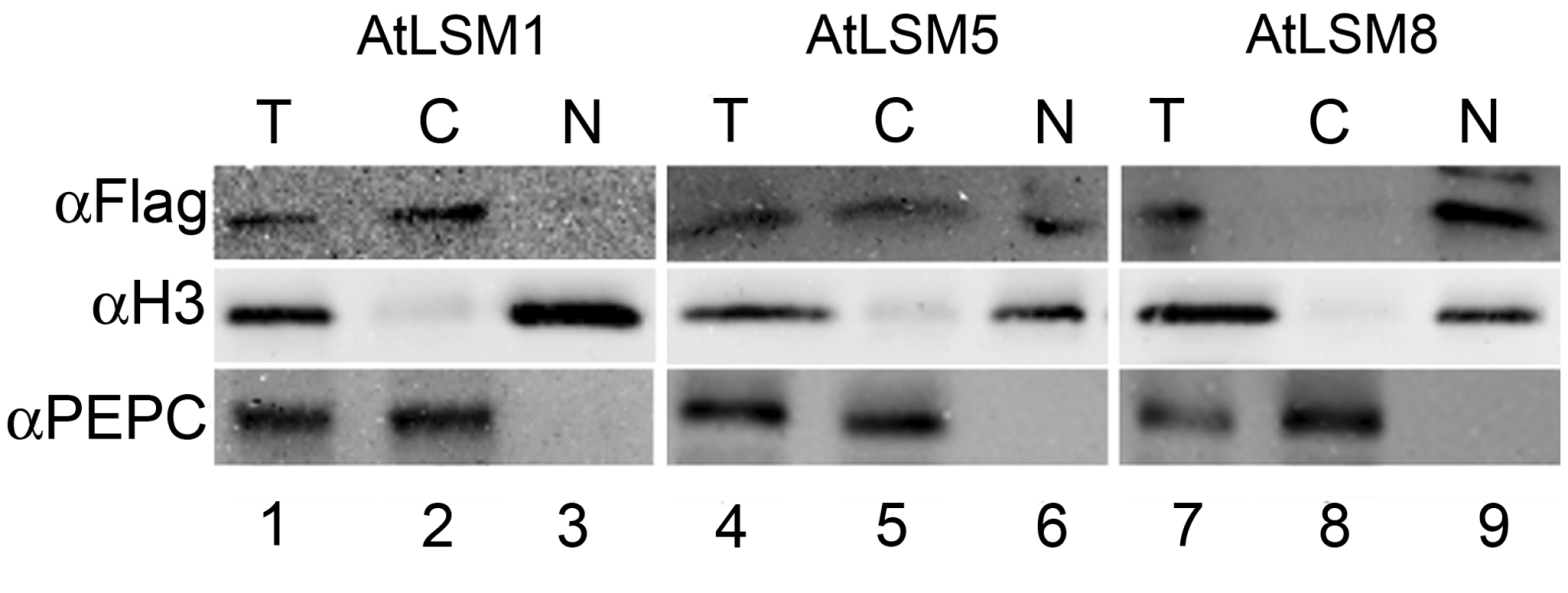
Distribution of AtLSM proteins in subcellular fractions of AtLSM1-SF-TAP, AtLSM5-SF-TAP and AtLSM8-SF-TAP transgenic lines. Nuclei and cytoplasm were separated by fractionation, and similar cell equivalents were subjected to SDS-PAGE followed by western blotting using the indicated antibodies. Histone H3 and Phosphoenolpyruvate carboxylase (PEPC) were used as nuclear and cytoplasmic markers, respectively. T, total; C, cytoplasm; N, nucleus.

Although Lsm8 determines the nuclear distribution of the yeast Lsm2-8 complex, it lacks a canonical or a functional nuclear localization signal and it is only the formation of the complex itself that enables its nuclear import or retention (52). Predictions using the WoLF PSORT server did not generate reliable data concerning the possible subcellular localization of *Arabidopsis* LSM proteins (30). To assess their intracellular distribution by fluorescence microscopy, we expressed GFP-tagged LSM constructs in tobacco or *Arabidopsis* mesophyll protoplasts. However, probably due to the low molecular weight of AtLSM polypeptides, these attempts did not give consistent results, as the fluorescent signal was diffused and evenly distributed throughout whole cells. Therefore, we used an alternative approach and examined the subcellular location of AtLSM complexes in fractionated cellular extracts from plants expressing AtLSM1-SF-TAP, AtLSM5-SF-TAP or AtLSM8-SF-TAP tagged proteins (see below). The expression of AtLSM-SF-TAP proteins was confirmed by western blotting using anti-FLAG antibodies. Detection of individual tagged proteins in the total, cytoplasmic or nuclear fraction revealed that, as in other eukaryotic organisms, AtLSM1 and AtLSM8 were localized exclusively in the cytoplasm or nucleus, respectively, while AtLSM5 was present in both compartments (Figure 2). This indicates the existence of at least two separate AtLSM complexes, cytoplasmic and nuclear, also in plants, which differ by the presence of the specific components, AtLSM1 and AtLSM8.

### Composition of AtLSM complexes

To purify and identify proteins interacting with AtLSM1, AtLSM5 or AtLSM8 components, we generated stable transgenic plants expressing tagged AtLSM1-SF-TAP, AtLSM5-SF-TAP or AtLSM8-SF-TAP proteins in *lsm1a lsm1b*, *sad1/lsm5* or *lsm8* mutants (see below), respectively. The SF-TAP tag is relatively small (4.6 kDa), which should prevent disruption of structure of LSM polypetides as well as interactions within protein complexes (37). The expression of AtLSM-SF-TAP proteins in the respective *lsm* mutants (see below) reverted their molecular phenotypes, confirming that the fusion proteins were functional (Supplementary Figure S6); however, morphological alterations of *sad1/lsm5* plants were not fully complemented by AtLSM5-SF-TAP (data not shown). SF-TAP-tagged complexes were purified from tagged lines and wild-type controls (Col-0 or WT). Proteins from whole eluate mixtures were concentrated and subjected to the Liquid chromatography-tandem mass spectrometry analysis. The highest scored hits specific for the AtLSM1, AtLSM5 and AtLSM8 proteins that were not represented in the negative control eluates from untagged Col-0 or WT plants are listed in Table 2 and Supplementary Tables S3 and S4. The results of this analysis showed that AtLSM1 interacted with all AtLSM proteins except AtLSM8, AtLSM8 with all AtLSMs except AtLSM1, whereas AtLSM5 formed complexes with all AtLSMs, including both AtLSM1 and AtLSM8. Together with the localization data, the proteomic survey of AtLSM complexes confirmed the possibility that two distinct heptameric complexes existed in the *Arabidopsis* cytoplasm and nuclei.

**Table 2.** Set of proteins present in at least two of three independent purifications that were specifically bound to anti-FLAG-M2 resin, as identified by tandem mass spectrometry following subtraction of peptides present in respective controls to account for unspecific hits

AGI no.	Description	Number of times identified	Biological process/molecular function^a^
AtLSM1a and co-purified proteins			
At1g19120	Small nuclear RNP family protein AtLSM1a	3	mRNA processing
At1g03330	Small nuclear RNP family protein AtLSM2	3	mRNA processing & mRNA splicing
At1g21190	Small nuclear RNP family protein AtLSM3a	3	mRNA processing & mRNA splicing
At1g76860	Small nuclear RNP family protein AtLSM3b	3	mRNA processing & mRNA splicing
At5g27720	Small nuclear RNP family protein AtLSM4	3	mRNA processing & mRNA splicing
At5g48870	Small nuclear RNP family protein SAD1/AtLSM5	3	mRNA processing & mRNA splicing
At2g43810	Small nuclear RNP family protein AtLSM6a	3	mRNA processing & mRNA splicing
At3g59810	Small nuclear RNP family protein AtLSM6b	3	mRNA processing & mRNA splicing
At2g03870	Small nuclear RNP family protein AtLSM7	3	mRNA processing & mRNA splicing
At3g22270	Topoisomerase II-associated protein PAT1	3	Putative role in mRNA decapping
At4g14990	Topoisomerase II-associated protein PAT1	3	Putative role in mRNA decapping
At1g79090	Topoisomerase II-associated protein PAT1	3	Putative role in mRNA decapping
At2g23350	poly(A) binding protein 4 (PABP4)	3	Response to cadmium ion/RNA binding
At1g01320	Tetratricopeptide repeat–like superfamily protein	2	mRNA modification
At1g02080	Transcription regulator	2	Putative component of the CCR4-NOT transcription complex and cytoplasmic deadenylase complex
At3g13300	Transducin/WD40 repeat-like superfamily protein VCS	2	mRNA decapping enhancer
At3g11500	Small nuclear RNP family protein, putative homologue of yeast and human SmG	2	Putative role in mRNA splicing
At2g45810	DEA(D/H)-box RNA helicase family protein	2	Nucleic acid binding/putative role in mRNA processing
At1g22760	poly(A) binding protein 3 (PABP3)	2	mRNA processing
AtLSM8 and co-purified proteins			
At1g03330	Small nuclear RNP family protein AtLSM2	3	mRNA processing & mRNA splicing
At1g21190	Small nuclear RNP family protein AtLSM3a	3	mRNA processing & mRNA splicing
At1g76860	Small nuclear RNP family protein AtLSM3b	3	mRNA processing & mRNA splicing
At5g27720	Small nuclear RNP family protein AtLSM4	3	mRNA processing & mRNA splicing
At5g48870	Small nuclear RNP family protein SAD1/AtLSM5	3	mRNA processing & mRNA splicing
At2g43810	Small nuclear RNP family protein AtLSM6a	3	mRNA processing & mRNA splicing
At3g59810	Small nuclear RNP family protein AtLSM6b	3	mRNA processing & mRNA splicing
At2g03870	Small nuclear RNP family protein AtLSM7	3	mRNA processing & mRNA splicing
At1g65700	Small nuclear RNP family protein AtLSM8	3	mRNA splicing
At4g24270	Embryo defective 140 (EMB140), putative homologue of yeast/human Prp24/SART3(p110)	3	RNA processing/putative role in mRNA splicing
At2g23350	poly(A) binding protein 4 (PABP4)	3	Response to cadmium ion/RNA binding
At3g11500	Small nuclear RNP family protein, putative homologue of yeast and human SmG	3	Putative role in mRNA splicing
At3g61240	DEA(D/H)-box RNA helicase family protein	3	Nucleotide biosynthetic process/putative role in mRNA processing
At5g07350	TUDOR-SN protein 1 (TSN1)	2	Gene silencing by RNA
At5g14140	Zinc ion binding/nucleic acid binding	2	Putative role in regulation of transcription
At1g20960	Embryo defective 1507 (EMB1507), putative homologue of yeast/human Brr2(Prp44)/SNRNP200	2	Nucleotide biosynthetic process/nucleic acid binding
At5g61780	TUDOR-SN protein 2 (TSN2)	2	Gene silencing by RNA
At1g80070	Embryo defective 14 (EMB14), putative homologue of yeast and human PRP8	2	Putative role in mRNA splicing
At3g07590	Small nuclear RNP family protein, putative homologue of yeast and human SmD1	2	Putative role in mRNA splicing
At1g01320	Tetratricopeptide repeat–like superfamily protein	2	mRNA modification
At2g25910	3′–5′ exonuclease domain-containing protein	2	Nucleobase-containing compound metabolic process/RNA binding

^a^The biological process/molecular function listed are mainly according to TAIR (http://www.arabidopsis.org/).

In addition to the components of the LSM rings, AtLSM1-SF-TAP purification revealed the presence of VARICOSE (VCS), a component of the decapping complex and a homologue of the human decapping enhancer Hedls/Ge1 (53–55), three *Arabidopsis* PAT1-like variants, homologues of yeast Pat1 and human Pat1b, which stimulate mRNA deadenylation and decapping (56–59) and a member of the NOT1 superfamily, a component of the CCR4-NOT complex that acts in both transcription regulation and mRNA deadenylation [reviewed in (60)]. The identified interactions pointed to a role of the cytoplasmic AtLSM complex in activating decapping during mRNA 5′-3′ decay.

Proteins that interact with AtLSM8 include the core components of other snRNPs, SmG and SmD1 (29), putatives homologues of the yeast and human U4/U6 recycling factor, Prp24/SART3/p110 (61,62) and Brr2/Prp44/SNRNP200 DExD/H-box helicase, the U4/U6.U5 snRNP component that catalyzes U4/U6 unwinding (61,63) and a potential plant counterpart of the yeast and human U5 snRNP-specific protein PRP8 (64). All of these proteins co-purify with the Lsm2-8 complex in yeast and humans as the U4/U6.U5 snRNP (6,62,65). These LSM8-interacting partners strongly support the role of the nuclear *Arabidopsis* LSM complex in pre-mRNA splicing.

Other proteins that were recovered in the AtLSM1-ST-TAP or AtLSM8-ST-TAP eluates, including RNA helicases, ribosomal proteins, translation factors or proteins involved in tRNA biogenesis (Supplementary Table S3), suggested an involvement of LSM proteins in different aspects of RNA metabolism, as has been previously shown in other eukaryotic organisms (10–16). Furthermore, supplementary factors present in AtLSM1 or AtLSM8 purifications (Supplementary Table S4), namely protein kinases and factors involved in the response to various stimuli (pathogen, hormones and stress), indicated a contribution of LSM proteins, possibly through their direct participation in RNA processing and decay, to plant physiological processes such as biotic and abiotic stress response and hormone signalling.

### Analysis of *Arabidopsis lsm* mutant lines

**Figure 3.**
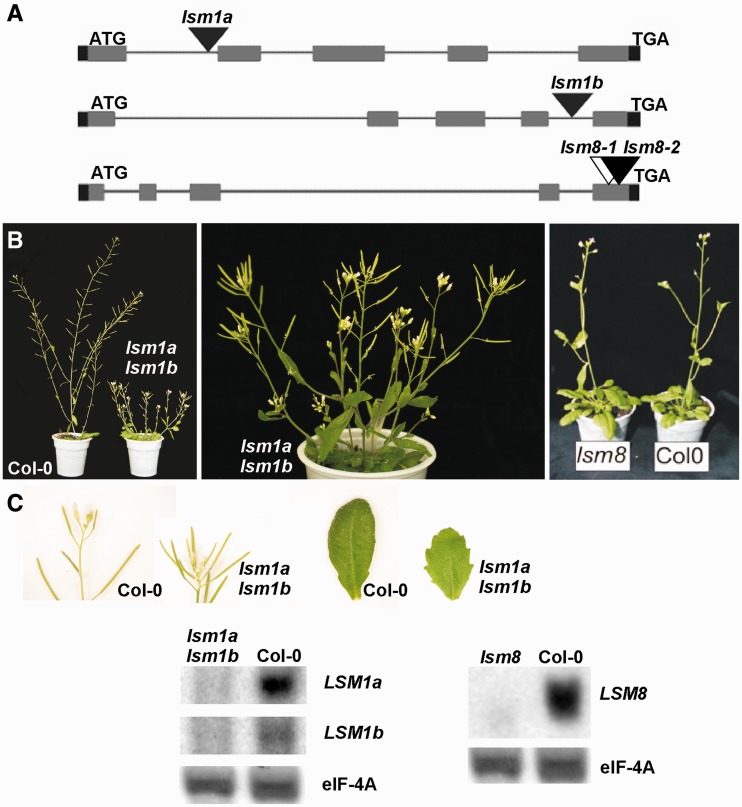
Characterization of *lsm1a lsm1b* and *lsm8* mutant lines. (**A**) Structure of the *AtLSM1a* (At1g19120), *AtLSM1b* (At3g14080) and *AtLSM8* (At1g65700) genes. Exons are represented by grey bars, the localization of T-DNA insertions are indicated. (**B**) Thirty-day-old wild-type, *lsm1a lsm1b* and *lsm8* plants. (**C**) Northern analysis of *AtLSM1a*, *AtLSM1b* and *AtLSM8* levels. elF-4A mRNA was used as a loading control.

To study the functions of LSM complexes in *Arabidopsis*, we selected *lsm1a* and *lsm1b* lines with T-DNA insertions in two *AtLSM1* genes (At1g19120 and At3g14080) and two *lsm8* lines with T-DNA insertions in the *AtLSM8* gene (At1g65700) (Figure 3A). We also used an already characterized *sad1/lsm5* mutant in the *AtLSM5* gene (34), as T-DNA insertion lines in this gene are not available and homozygous plants with a T-DNA insertion in the *AtLSM4* gene die in late development (31). Both *lsm8-1* and *lsm8-2* mutants, as well as the single *lsm1a* and *lsm1b* mutants, displayed only minor phenotypic alterations compared with wild-type plants (Figure 3B and data not shown). However, the double *lsm1a lsm1b* mutant showed a clear growth delay, which was more evident when plants were grown under short-day conditions (data not shown), and severe morphological changes including undulate or lobate leaves and larger and shortened internodes resulting in the location of siliques in the apical parts of shoots, which led to the formation of clusters, lack of apical dominance and a bushy appearance (Figure 3B). Such defects indicated pleiotropic effects of the mutation on plant growth and development.

The level of *AtLSM1a* and *AtLSM1b* in 2-week-old *lsm1a, lsm1b* and *lsm1a lsm1b* seedlings, as well as of *AtLSM8* in *lsm8-1* and *lsm8-2* seedlings was tested by northern blot (Figure 3C and data not shown). Both *AtLSM1a* and *AtLSM1b* mRNAs were virtually absent in the double *lsm1a lsm1b* mutant, whereas a residual expression of a truncated *AtLSM8* transcript was detected in *lsm8* plants. In both *lsm8* lines, the T-DNA insertion in the *AtLSM8* gene was located in the last exon, downstream of the region encoding the conserved Sm2 box. Thus, a low level of a shorter, but still functional, AtLSM8 protein could be produced. In addition, in agreement with the expression pattern at the Genevestigator website (see Supplementary Figure S4), northern hybridization confirmed that *AtLSM1a* was expressed at a higher level than *AtLSM1b*. As *lsm8-1* and *lsm8-2* mutants are comparable and have the same level of *LSM8* expression, only the *lsm8-1* was used in further analyses. Likewise, only the double *lsm1a lsm1b* mutant was examined, given that the single *lsm1a* and *lsm1b* lines did not exhibit any discernible phenotype and expressed a standard amount of *AtLSM1b* or *AtLSM1a* mRNA, respectively. In fact, the level of *AtLSM1b* was higher in the *lsm1a* mutant than in wild-type plants, so as to compensate for the absent and normally more abundant *AtLSM1a* mRNA (data not shown).

**Figure 4.**
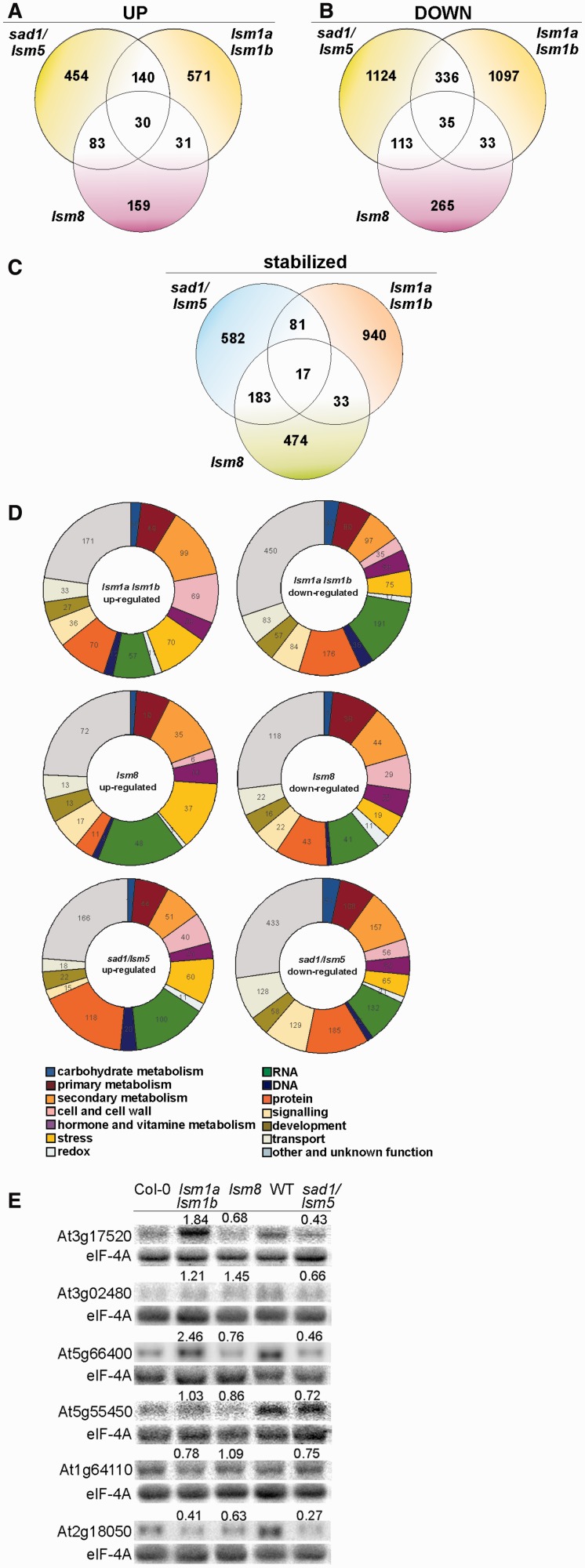
Venn diagrams representing the number of transcripts significantly (>1.5-fold) upregulated (**A**), downregulated (**B**) or stabilized upon cordycepin treatment (**C**) in *lsm1a lsm1b*, *lsm8* and *sad1/lsm5* mutants. Circular diagrams (**D**) illustrating functional classification of transcripts upregulated or downregulated in *lsm1a lsm1b*, *lsm8* and *sad1/lsm5* mutants. Results were obtained from the PageMan database (https://http://mapman.mpimpgolm.mpg.de/index.shtml). (**E**) Validation of microarray data for chosen transcripts. Northern analysis of At3g17520, At3g02480, At5g66400 (*RAB18*), At5g55450, At1g64110 (*DAA1*), At2g18050 (*HIS1-3*) in *lsm1a lsm1b*, *lsm8* and *sad1/lsm5* mutants and their respective wild types (Col-0 or WT). Numbers in parentheses represent the transcript level in the mutant relative to Col-0 or wild type, respectively. eIF-4A was used as a loading control.

To examine genome-wide mRNAs affected in the absence of AtLSM proteins, we analysed the expression and mRNA stability profiles in *lsm1a lsm1b*, *lsm8* and *sad1/lsm5* mutants using Affymetrix *Arabidopsis* ATH1 Gene-Chip microarrays. Total RNA was isolated from mutant lines and their corresponding wild-type (Col-0 or WT) seedlings at chosen time points following transcriptional inhibition by cordycepin. The number and lists of statistically upregulated or downregulated transcripts by at least 1.5-fold, as well as stabilized mRNAs (based on the analysis of two independent biological replicates) are shown in Figure 4 and Supplementary Tables S5 and S6. Functional classification of the 2315, 749 and 2273 transcripts differentially expressed in *sad1/lsm5*, *lsm8* and *lsm1a lsm1b* mutants, respectively, relative to wild-type plants demonstrated that they belonged to various categories. However, a large number of these genes encoded proteins involved in RNA biogenesis, mainly transcription, protein synthesis and turnover, primary and secondary metabolism, stress response, as well as intracellular trafficking and signalling (including hormone transduction). The lower overall number of genes affected in the *lsm8* line was consistent with its hypomorphic character. It is still noteworthy that 33% of the downregulated and 37% of the upregulated transcripts in the *lsm8* mutant overlapped with those in the *sad1/lsm5* line, while a lower number of substrates, 15% and 20%, respectively, were shared between *lsm8* and *lsm1a lsm1b*. This suggests that some changes in the *sad1/lsm5* and *lsm8* expression profiles resulted from a disturbance in the same process, probably splicing. In turn, increased expression of numerous genes in *sad1/lsm5* and *lsm1a lsm1b* plants may have been a direct effect of their stabilization due to a defect in mRNA decay. However, a relatively moderate number of common transcripts upregulated in both mutants (∼23%) suggested that either component of the cytoplasmic AtLSM complex have a partially separate pool of substrates, or, more likely, their molecular characteristics differ due to a distinct nature of the mutation in each line. It is possible that an altered and non-functional AtLSM5 protein expressed in the *sad1/lsm5* mutant was incorporated into the LSM ring, yielding a distinct set of phenotypes to those generated by absence of AtLSM1, which probably disabled the formation of the complex (34). By contrast, reduced expression of a large number of genes in *lsm1a lsm1b* plants was probably an indirect effect of a dysfunction of the cytoplasmic LSM complex.

The participation of AtLSM5 and AtLSM1 in mRNA degradation was further supported by the observation that 863 and 1071 transcripts were stabilized >1.3-fold in *sad1/lsm5* and *lsm1a lsm1b* lines, respectively, following transcriptional inhibition (Supplementary Table S6). Interestingly, 707 transcripts showed the same degree of stabilization in the *lsm8* mutant. This suggests that a subset of mRNAs undergoes 5′–3′ degradation in the nucleus.

Changes in mRNA accumulation revealed by the microarray analysis were validated by northern blot for a number of selected transcripts (Figure 4E). This analysis first of all confirmed that some mRNAs (At5g66400/*RAB18*, At5g55450, At3g17520 and At3g02480) were downregulated either in *sad1/lsm5*, in both *sad1/lsm5* and *lsm8* lines or in all mutants (At2g18050/HIS1-3), whereas expression of several mRNAs, including At4g32020, At1g20160/SBT5.2, At5g12020/HSP17.6II, At5g42380/CML37, At3g11410/AHG3 and At2g30020, was clearly increased in *lsm1a lsm1b* and *sad1/lsm5* or only in *lsm1a lsm1b* (At1g73120) plants (see Figures 6 and 7 and Supplementary Figures S7 and S8). On the other hand, some transcripts, such as At2g32150, At4g11280/ACS6, At5g62520/SRO5 and At1g55020/LOX1, were upregulated in both *lsm1a lsm1b* and *lsm8* lines (see Figure 8). The altered level of mRNAs controlled by hormone and stress stimuli or encoding proteins involved in stress or defense responses (RAB18, HIS1-3, ACS6, SRO5, At1g73120, At1g55020, embryogenesis abundant (LEA) proteins At3g17520 and At3g02480, heat shock protein HSP17.6II, protein phosphatases 2C AHG3 and At2g30020 and transcription factor WRKY33) suggest that via their role in mRNA metabolism, LSM complexes contribute to the regulation of stress- and hormone-related processes. The increased levels of the selected mRNAs in *lsm1a lsm1b* or *sad1/lsm5* lines was reverted by the stable expression of AtLSM1-SF-TAP and AtLSM5-SF-TAP, respectively, supporting the notion that this phenotype is directly related to the dysfunction of the cytoplasmic LSM complex (Supplementary Figures S6A and B).

**Figure 5.**
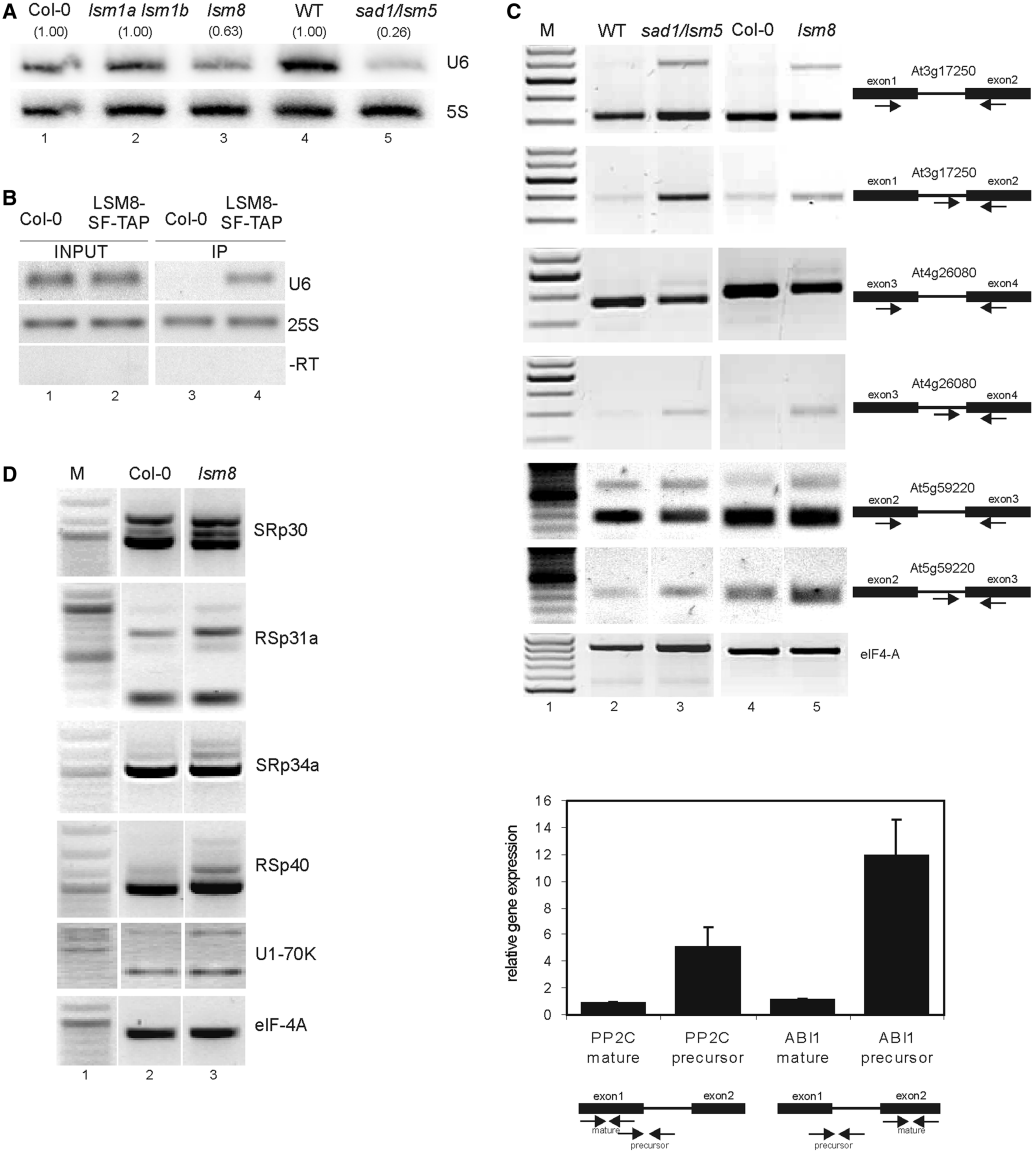
AtLSM5 and AtLSM8 are involved in pre-mRNA splicing. (**A**) Northern analysis of U6 snRNA in *lsm1a lsm1b*, *lsm8* and *sad1/lsm5* mutants and their respective wild types (Col-0 or WT). Numbers in parentheses represent the transcript level in the mutant relative to Col-0 or wild type, respectively. 5S rRNA was used as a loading control. (**B**) RNA immunoprecipitation. RNA IP was performed on nuclear extracts from *lsm8* expressing AtLSM8-SF-TAP or Col-0 plants using anti-FLAG resins. U6 snRNA and 25S rRNA were detected by semi-quantitative RT-PCR in the input and IP fractions. –RT, reverse-transcription reaction control using U6 primers. (**C**) Semi-quantitative RT-PCR and quantitative RT-PCR analyses of intron retention in *sad1/lsm5* and *lsm8* mutants and their respective wild types (Col-0 or WT). Specific primers used to detect At3g17250 (*PP2C*), At4g26080 (*ABI1*) and At5g59220 (*HAI1*) are depicted to the right of each panel. eIF-4A was used as a control. (**D**) Semi-quantitative RT-PCR for genes involved in splicing. Specific primers used to detect SRp30, SRp31a, SRp34a, RSp40, U1-70K were located in the first and last exons. eIF-4A was used as a control.

**Figure 6.**
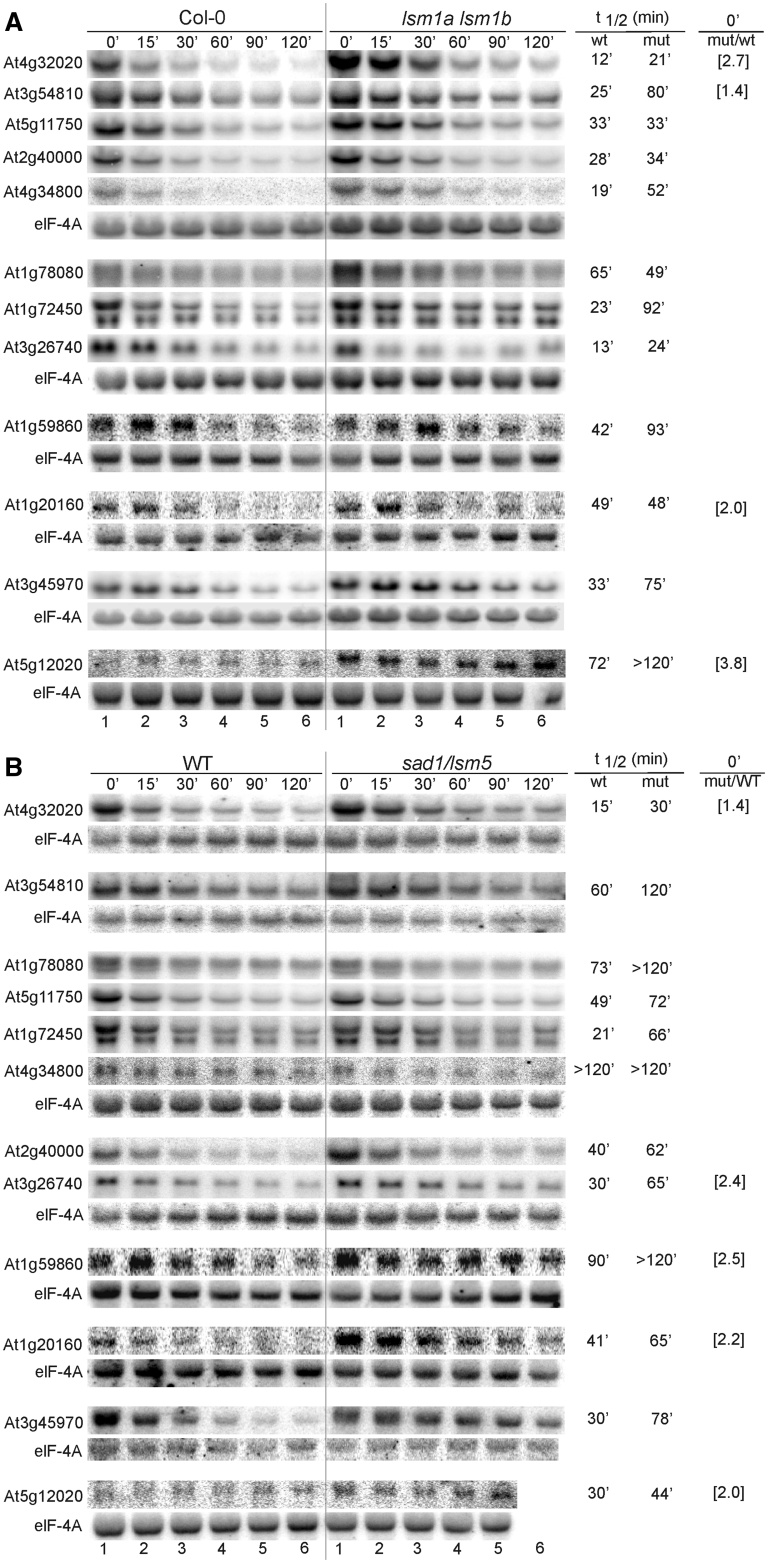
AtLSM1 and AtLSM5 affect mRNA stability. Northern analysis of mRNAs, including XRN4 or DCP2 substrates and ARE- and DST-containing transcripts, at specific time points after cordycepin treatment in *lsm1a lsm1b* (**A**) and *sad1/lsm5* (**B**) mutants and their respective wild types (Col-0 or WT). Estimated mRNA half-life (t_1/2_) is shown to the right of each panel. Numbers in parentheses represent the fold change in the transcript level of the mutant versus wild type for those mRNAs that accumulate at the steady state (at time 0’ min). elF-4A mRNA was used as control.

**Figure 7.**
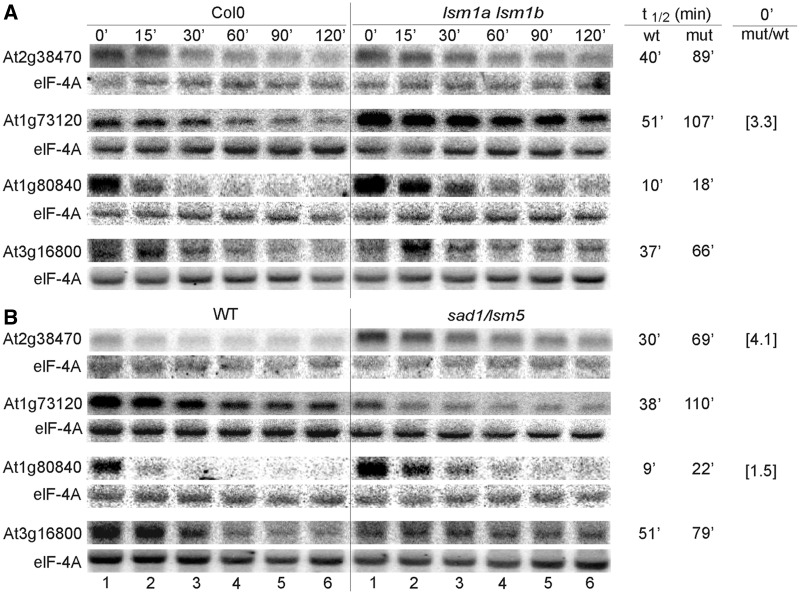
AtLSM1 and AtLSM5 affect mRNA stability. Description is as for Figure 6.

**Figure 8.**
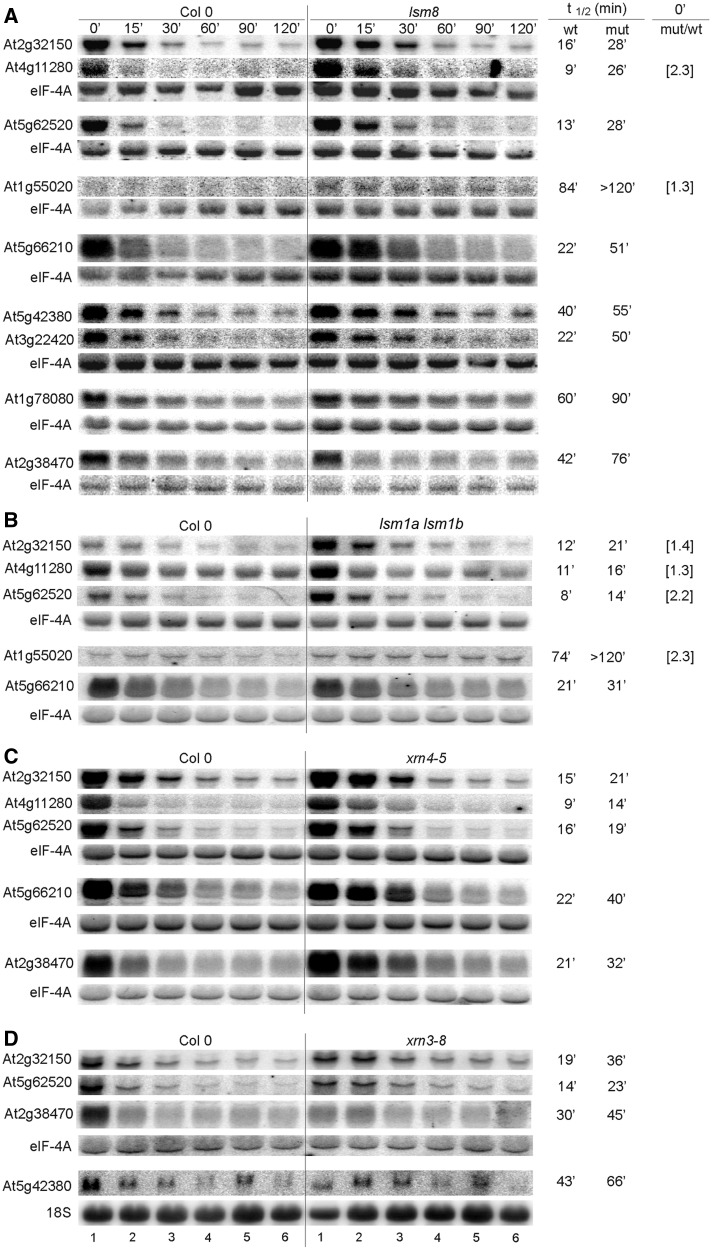
Northern analysis of mRNAs, which stability is altered in *lsm8* (**A**), *lsm1a lsm1b* (**B**), *xrn4-5* (**C**) and *xrn3-8* (**D**) mutants. Description is as for Figure 6, except that elF-4A or 18S rRNA were used as controls.

### The role of AtLSM proteins in pre-mRNA splicing

As the LSM2-8 complex in other organisms is a core component of the U6 snRNP, we tested the level of U6 snRNA in *lsm* mutants by northern analysis (Figure 5A). U6 was significantly reduced in *sad1/lsm5* and to a lesser extent in *lsm8* plants (to 0.26 and 0.63 of their wild-type level, respectively), but was not affected in the *lsm1a lsm1b* mutant. The weaker effect observed in the *lsm8* mutant again indicates that it is not a null allele and that a low level of AtLSM8 protein probably accounts for its weaker morphological and molecular phenotype. The expression of AtLSM5-SF-TAP and AtLSM8-SF-TAP proteins in *sad1/lsm5* and *lsm8* mutants, respectively, restored the wild-type levels of U6 snRNA, showing that U6 depletion was a direct consequence of the LSM complex deficiency (Supplementary Figure S6C). Moreover, RNA immunoprecipitation using nuclear extract from plants expressing tagged AtLSM8-SF-TAP, followed by semi-quantitative RT-PCR, revealed specific interaction of AtLSM8 with U6 snRNA, which was not present in the pellet from control Col-0 plants (Figure 5B).

Considering that the lack of AtLSM4 results in a splicing defect (31), we tested the level of unspliced mRNA precursors by RT-PCR. We first focused on genes encoding factors involved in the response to ABA, as both *sad1/lsm5* and *lsm4* mutants were hypersensitive to ABA (31,34). Our microarray data showed that several genes related to ABA signalling were downregulated in *sad1/lsm5* and *lsm8* lines (Supplementary Table S5). We observed a clear accumulation of intron-containing transcripts for three protein phosphatase 2C (PP2C) genes, At3g17250, At4g26080/ABI1 and At5g59220, in *sad1/lsm5* and *lsm8*, but not in *lsm1a lsm1b* plants (Figure 5C and data not shown). This result was verified by qRT-PCR, which showed a 5- and 12-fold increase of the precursor levels of At3g17250 and ABI1, respectively, in the *sad1/lsm5* mutant relative to wild type, with no change in the mature mRNA (Figure 5C). Furthermore, a survey of several splicing factor pre-mRNAs by semi-quantitative RT-PCR revealed an altered pattern of unspliced transcripts in the *lsm8* mutant, with a visible accumulation of some precursor forms of the serine-arginine–rich (SR) protein genes At1g09140/SRp30, At2g46610/RSp31a, At3g49430/SRp34a, At4g25500/RSp40 and a U1 snRNP specific component At3g50670/U1-70K (Figure 5D).

Together, these data strongly suggest that the nuclear LSM complex has a role in pre-mRNA splicing as a U6 snRNP core component required for U6 stability.

### The function of AtLSM complexes in mRNA decay

The microarray mRNA stability profiles confirmed that several transcripts could have increased half-lives in the absence of the cytoplasmic LSM components, AtLSM1 and AtLSM5, as well as in plants lacking the nuclear AtLSM8 protein (Supplementary Table S6).

To confirm the participation of the cytoplasmic LSM complex in the 5′–3′ decay pathway, we tested the stability of chosen mRNAs in *sad1/lsm5*, *lsm1a lsm1b* and *lsm8* mutants following transcriptional inhibition by cordycepin. The samples were collected at specific time points, and the RNA was analysed by northern blot. We started with potential targets of the cytoplasmic 5′–3′ exonuclease AtXRN4 and the decapping enzyme AtDCP2/TDT (44,54,55). The established half-lives of four (At4g32020, At3g54810/BME3, At1g78080/RAP2.4 and At5g11750/L19) out of the seven tested mRNAs, which were stabilized or upregulated in the *xrn4-5* line, were markedly increased in the *sad1/lsm5* mutant, whereas the two first transcripts were also stabilized in the *lsm1a lsm1b* mutant. Three (At1g59860, At5g12020/HSP17.6II and At3g45970/EXPL1) out of the ten tested putative AtDCP2 substrates were stabilized in both the *sad1/lsm5* and *lsm1a lsm1b* lines, and another (At1g20160/SBT5.2) had an extended half-life only in *sad1/lsm5* plants (Figure 6A and B). Little, if any, effect was observed for these mRNAs in the *lsm8* mutant, except for At1g78080, which had a prolonged half-life (Figure 8; Supplementary Figure S7). Additionally, some of the unstable ARE-containing (AU-rich elements) or DST-containing (downstream elements) mRNAs (66–68) (At1g72450/JAZ6, At2g40000/PRO-12, At3g26740/CCL and At4g34800) were also clearly stabilized in *sad1/lsm5* and *lsm1a lsm1b* lines (Figures 6A and B; Supplementary Figure S7).

We next tested several mRNAs from the list of stabilized transcripts established by the microarray data (see Supplementary Table S6). The half-lives of at least four (At1g73120, At1g80840/WRKY40, At3g16800/PP2C and At2g38470/WRKY33) out of 15 mRNAs were significantly longer in both *sad1/lsm5* and *lsm1a lsm1b* compared with their wild types (Figure 7). The first three were not visibly changed in *lsm8* plants (Supplementary Figure S7). In addition, we have observed that the stability of some mRNAs (At2g32150, At4g11280/ACS6, At5g62520/SRO5, At1g55020/LOX1, At5g66210/CPK28, At5g42380/CML37, At3g22420/WNK2, At1g78080/RAP2.4 and At2g38470/WRKY33) was increased in *lsm8* and *lsm1a lsm1b* or *sad1/lsm5* mutants (Figures 6, 7, 8A and B and data not shown). Interestingly, some of these transcripts were also stabilized in *xrn4-5* and *xrn3-8* but not in *xrn2-3* mutants (Figure 8C and D) and data not shown (47), which supports a notion that they are targeted for degradation in the nucleus, probably by AtXRN3, which localizes to this compartment (Sikorski and Kufel, unpublished data).

**Figure 9.**
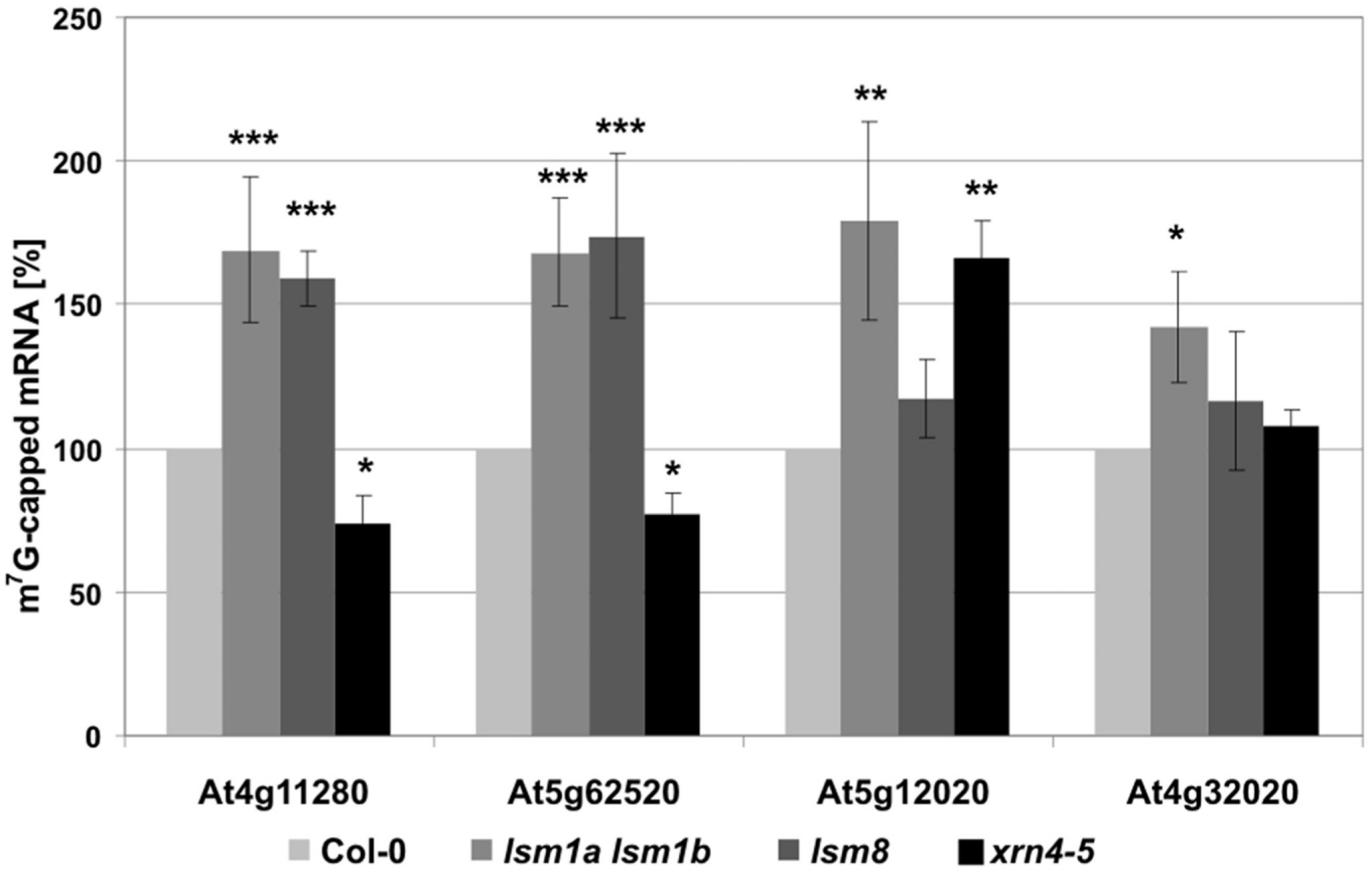
mRNAs stabilized in *lsm* mutants are 5′ capped. Immunoprecipitation of total RNAs with monoclonal anti-cap antibodies followed by qRT-PCR analysis of selected AtLSM1 (At4g11280, At5g62520, At5g12020, At4g32020), AtLSM8 (At4g11280, At5g62520) and AtXRN4 (At4g11280, At5g62520, At4g32020) substrates. Histograms represent the level of immunoprecipitated transcripts, where the value for Col-0 was arbitrarily set to 100. Values with standard deviations (±SD) were obtained from three independent experiments; *P < 0.05, **P < 0.01, ***P < 0.001.

To establish whether mRNA stabilization in *lsm* mutants resulted from a defect in their decapping, we checked the cap status of selected stabilized mRNAs by immunoprecipitation with anti-cap antibodies (H20) followed by qRT-PCR to quantify the fraction of immunoprecipitated transcripts. The levels of capped mRNAs stabilized in *lsm1a lsm1b* and *xrn4-5* (At4g32020, At4g11280/ACS6 and At5g62520/SRO5) or *lsm1a lsm1b* and *tdt-1* (At5g12020/HSP17.6II), mutants were clearly increased in *lsm1a lsm1b* but not in *xrn4-5* plants (Figure 9). This indicates that in the absence of AtLSM1, these mRNAs accumulated as capped transcripts, but in plants lacking AtXRN4, they were stabilized following cap removal by the decapping complex. In addition, mRNAs that showed stabilization in the *lsm8* mutant (At5g12020/HSP17.6II, At4g32020) were also capped (Figure 9).

These data clearly show that cytoplasmic AtLSM proteins participate in the 5′–3′ decay pathway of a fraction of cellular mRNAs. The fact that at least some of them are also direct targets of the AtDCP complex and the AtXRN4 exonuclease, and that they accumulate as capped transcripts in cells lacking AtLSM1, indicating that the LSM complex acts as a decapping activator. In addition, a subset of substrates of the 5′–3′ pathway probably undergoes degradation also in the nucleus, as they are stabilized, also in a capped form, in the absence of the nuclear AtLSM8 protein. At least some of these mRNAs are turned over by the nuclear exonuclease AtXRN3.

## DISCUSSION

The highly conserved LSM complexes have been first identified and best characterized in yeast and humans, where they participate in different aspects of sRNA metabolism, mainly pre-mRNA splicing and mRNA decay (2–8,69). Although these processes and the several proteins involved in their execution, including splicing factors, Sm proteins, deadenylases, decapping enzymes and exonucleases, have been described in plants, the contribution of LSM complexes has not been properly evaluated. In this work, we present evidence that *Arabidopsis* LSM proteins, similar to their yeast and human counterparts, form two distinct complexes: one containing AtLSM1 being exclusively located in the cytoplasm and the other with AtLSM8 present only in the nucleus. Analysis of their molecular functions confirmed their roles, already established in other organisms, in mRNA splicing and degradation, and showed that the latter was to promote mRNA decapping. In contrast to described cases, the *Arabidopsis* nuclear LSM2-LSM8 complex, in addition to its major role as a core component of the U6 snRNP, may also participate in mRNA 5′–3′ decay.

### LSM complexes in *Arabidopsis*

The existence of plant LSM proteins was predicted based on bioinformatic searches (25,26,29,30), and also partly confirmed by fortuitous identification of AtLSM5 (SAD1) and AtLSM4 and their potential role in pre-mRNA splicing (31,34).

Our systematic survey of AtLSM proteins using fractionation of cellular extracts, as well as proteomic analyses of LSM-interacting proteins, confirmed the presence of at least two distinct canonical LSM complexes in *Arabidopsis*. The number of affinity-purified LSM-interacting factors was unexpectedly low, probably due to technical difficulties, which accompany the purification of complexes from plant material. Only the addition of a cross-linking step improved the procedure’s efficiency, allowing for the isolation and identification of additional proteins that interact with the LSM ring. The existence and function of two LSM complexes was corroborated by their specific interactions with a separate set of proteins, which are either involved in pre-mRNA splicing or mRNA deadenylation, decapping and decay in the case of AtLSM8 and AtLSM1 complexes, respectively. Interestingly, the presence of AtLSM1, the decapping enhancer VCS and putative AtPAT1 and AtNOT1 homologues in one complex indicates that, as in human cells, where Pat1 was shown to interact with LSm1, Hedls/Ge1 and CCR4-NOT proteins (58,59), *Arabidopsis* PAT1 proteins may also act in connecting deadeanylation with decapping during mRNA 5′–3′ decay.

Other proteins recovered in LSM-interacting complexes, such as protein kinases or those involved in stress response and defense, in addition to the types of mRNAs affected in *lsm* mutants, including stress- and hormone-regulated transcripts, are consistent with the role of LSM complexes, and more generally of RNA metabolism, in these cellular physiological processes.

An intriguing question is whether any alternative LSM complexes exist in plants. Known examples in other organisms, mainly the yeast LSM2-7 complex that localizes to the nucleoli and associates with snR5 and the precursor to RNase P RNA (3,15), sets a precedent; however, our proteomic data did not allow for the drawing of such conclusions for plants.

### mRNA decay in the nucleus

Our microarray analyses and their validation confirmed the conserved functions of the nuclear and cytoplasmic AtLSM complexes in U6 stabilization/pre-mRNA splicing and mRNA 5′–3′ decay, respectively. As seen for the other 5′–3′ turnover factors in *Arabidopsis*, AtXRN4 and AtDCP2, the LSM1-7 complex was not responsible for general turnover of cellular mRNAs and only a limited number of transcripts were bona fide substrates of these pathways, consistent with the proposition that a bulk of plant cytoplasmic mRNAs is degraded by the exosome (44,54,55,70). In addition, our data suggested that some mRNAs may undergo 5′–3′ degradation, assisted by the LSM2-8 complex, in the nucleus as well. Lsm1 has not been identified in *T.brucei* and it is likely that only the Lsm2-8 complex exists in this organism (11,15,21,22). Consistently, the trypanosome Lsm8 was reported to be involved in mRNA degradation; however, it was also present in the cytoplasm (71). Most cases of nuclear RNA decay concern quality control mechanisms, which operate on nucleus-restricted transcripts due to processing or export defects [reviewed in (72)]. Genuine substrates of the nuclear decay pathway have also been reported in *S.cerevisiae*, mainly for the nuclear exosome component, Rrp6 (73,74). Participation of the yeast Lsm2-8 complex was implied in RNA surveillance pathways (11,15,21,22), but not in the degradation of normal cellular mRNAs. The evident stabilization of a number of transcripts in the *lsm8* mutant suggests this may be the case for the nuclear AtLSM2-8 complex in plants. It is possible that a specialized nuclear mechanism is preferred in various unusual conditions, when rapid elimination of unwanted mRNAs is required. This raises a question about the occurrence of mRNA decapping in the nucleus, especially that tested LSM8 substrates accumulated in the *lsm8* mutant as capped transcripts, suggesting that also the nuclear LSM complex facilitates decapping. The DCP1/2 complex is mainly cytoplasmic, but it was also shown to be present in the nucleus of human cells, where decapping of nascent transcripts was proposed to contribute to premature termination of transcription (75,76). Comparison of substrates common to AtLSM1 and AtXRN4 [(44); this work] implies that cytoplasmic mRNAs are eliminated by this 5′–3′ exonuclease, whereas the existence of transcripts stabilized in *lsm8* and *xrn3* lines argues that the nuclear AtXRN3 nuclease is involved in their degradation. Another atypical but conserved 5′–3′ exonuclease Rrp17/hNOL12 was identified in yeast and humans (77) and is possible that their homologue, AtNOL12 (At1g11240), contributes to the nuclear mRNA decay in *Arabidopsis*. If this is indeed the case, it would constitute a novel class of substrates for this enzyme.

We conclude that both LSM complexes participate in the 5′–3′ degradation of a subset of cellular mRNAs, but the rules dictating the selection of substrates into a specific pathway have not been identified yet.

## SUPPLEMENTARY DATA

Supplementary Data are available at NAR Online: Supplementary Tables 1–6, Supplementary Figures 1–8, Supplementary Methods and Supplementary References [4,37,38,44,78–80].

## FUNDING

Ministry of Science and Higher Education [1584/B/P01/2008/35], Foundation for Polish Science [TEAM/2008-2/] and Wellcome Trust [067504/Z/02/Z]. Experiments were carried out with the use of CePT infrastructure financed by the European Union—the European Regional Development Fund [Innovative economy 2007–13, Agreement POIG.02.02.00-14-024/08-00]. Funding for open access charge: Foundation for Polish Science [TEAM/2008-2/].

*Conflict of interest statement*. None declared.

## Supplementary Material

Supplementary Data
