# Supplementary material for: Arabidopsis thaliana LSM proteins function in mRNA splicing and degradation
Source: Nucleic Acids Res. 2013 Apr 24;41(12):6232–49. doi: 10.1093/nar/gkt296 (PMC3695525; doi:10.1093/nar/gkt296)
Supplement: Supplementary Data [file supp_41_12_6232__index.html]

Arabidopsis thaliana LSM proteins function in mRNA splicing and degradation — Arabidopsis thaliana LSM proteins function in mRNA splicing and degradation — Supplementary Data 

# *Arabidopsis thaliana* LSM proteins function in mRNA splicing and degradation

## Supplementary Data

files

**Files in this Data Supplement:**

- Supplementary Data - pdf file
- Supplementary Data - pdf file
- Supplementary Data - pdf file
- Supplementary Data - xls file
