# Supplementary material for: Arabidopsis thaliana LSM proteins function in mRNA splicing and degradation
Source: Nucleic Acids Res. 2013 Apr 24;41(12):6232–49. doi: 10.1093/nar/gkt296 (PMC3695525; doi:10.1093/nar/gkt296)
Supplement: Supplementary Data [file supp_gkt296_nar-03286-a-2012-File010.pdf]

SUPPLEMENTARY MATERIAL

Supplementary Methods

Cloning

Full-length *AtLSM1, AtLSM2, AtLSM3, AtLSM4, AtLSM5, AtLSM6, AtLSM7* and *AtLSM8* cDNAs obtained by reverse transcription PCR (RT-PCR) with total RNA isolated from *Arabidopsis* seedlings were cloned into pGEM-Teasy vector (Promega) and verified by sequencing. Then 5’ and 3’ ends of *LSM* mRNAs were characterized by 5’ rapid amplification of cDNA ends (RACE) PCR using the 5’ or 3’ RACE System (Invitrogen), respectively. The identified ends overlapped with annotated sequences (www.arabidopsis.org). *LSM* cDNAs were recloned into *SalI* and *NcoI* sites of the yeast expression vector pNOPPATAIL (constructed by Klaus Hellmuth). LSM-GFP C-terminal fusions were generated by cloning *LSM* cDNA sequences into the *NcoI* site of the ppk100 vector containing the *EGFP* gene. All plasmids were amplified using *E. coli* strain DH5α.

Yeast methods

Growth and handling of *S. cerevisiae* were carried out using standard techniques. The transformation procedure was as described (78). pNOPPATAIL-AtLSM1, pNOPPATAIL-AtLSM6, pNOPPATAIL-AtLSM7, pNOPPATAIL-AtLSM2, pNOPPATAIL-AtLSM3, pNOPPATAIL-AtLSM5 and pNOPPATAIL-AtLSM8 plasmids were introduced into yeast strains either lacking the corresponding non-essential yeast *LSM* genes (*lsm1Δ*, *lsm6Δ* or *lsm7Δ* strains) or into strains in which essential genes were placed under the control of the regulatable *GAL* promoter (strains *GAL::lsm2*, *GAL::lsm3*, *GAL::lsm5*, *GAL::lsm8* strains). The expression of *Arabidopsis LSM* mRNAs in yeast cells was confirmed by nortern blot using random-primed probes specific for each mRNA.

For yeast complementation tests, *lsm1Δ, lsm6Δ* and *lsm7Δ* strains either carrying the empty pNOPPATAIL vector as a control or plasmids expressing *AtLSM1*, *AtLSM6* or *AtLSM7* under the control of the *ADH1* promoter, were pre-grown in liquid YPD medium (1% yeast extract, 2% Bacto-peptone, 2% glucose) at the permissive temperature of 23°C, cultures were brought to equal optical density (OD_{600}~2) and cells were spotted in serial dilutions onto YPD plates and incubated either at 23°C or 37°C (non-permissive temperature) for 2-3 days. *GAL::lsm2*, *GAL::lsm3*, *GAL::lsm5* and *GAL::lsm8* strains either carrying the empty pNOPPATAIL vector or plasmids expressing *AtLSM2*, *AtLSM3*, *AtLSM5* or *AtLSM8* under the control of the *ADH1* promoter, were pre-grown at permissive conditions in liquid YPGal medium (1% yeast extract, 2% Bacto-peptone, 2% galactose) at 30°C. Cultures were brought to equal optical density (OD_{600} ~2) and cells were spotted in serial dilutions onto YPGal or YPD (non-permissive conditions) plates.

Supplementary Tables.

Supplementary Table S1. Plasmids and yeast strains used in this work.

**Yeast strains**	**Genotype**	**Reference**
BMA64	*MATα ade2-1 his3-11,15 leu2-3,112 trp1-1 ura3-1*	(79)
AEMY19	*MATα ade2-1 his3 200 leu2-3,112 trp1 1 ura3-1 LSM6*::*HIS3*	(4)
AEMY22	*MATα ade2-1 his3 200 leu2-3,112 trp1 1 ura3-1 LSM7*::*HIS3*	(4)
AEMY24	*MATα ade2-1 his3-11,15 leu2-3,112 trp1 1 ura3-1 LSM1*::*TRP1*	(4)
AEMY29	*MATα ade2-1 his3-11,15 leu2-3,112 trp1 1 ura3-1 LSM5*::*TRP1* [pACTIIst-LSM5]	(4)
AEMY30	*MATα ade2-1 his3 200 leu2-3,112 trp1 1 ura3-1 LSM2*::*HIS3* [pACTIIst-LSM2]	(4)
AEMY31	*MATα ade2-1 his3-11,15 leu2-3,112 trp1 1 ura3-1 LSM3*::*TRP1* [pBM125-GAL1-HA-LSM3]	(4)
AEMY33	*MATα ade2-1 his3 200 leu2-3,112 trp1 1 ura3-1 LSM2*::*HIS3* [pBM125-GAL1-LSM2-HA]	(4)
AEMY46	*MATα ade2-1 his3-11,15 leu2-3,112 trp1 1 ura3-1 LSM8*::*TRP1* [pBM125-GAL1-HA-LSM8]	(4)
AEMY47	*MATα ade2-1 his3-11,15 leu2-3,112 trp1 1 ura3-1 LSM5*::*TRP1* [pBM125-GAL1-HA-LSM5]	(4)

**Plasmids**	**Description**	**Reference**
pGEM-Teasy		Promega
pNOPPATAIL	yeast vector, CEN, LEU2	(80)
pGWB502	plant binary vector	(38)
pDEST/C-SF-TAP		(37)
pGWB502-C-SF-TAP	as pGWB502 but C-terminal SF-TAP cloned into BglII site	this work

Supplementary Table S2. Oligonucleotides used in this work.

**Northern blot**			
Name	Gene ID	Primer Forward	Primer Reverse
HAD	At2g32150	TTCGAAGAGATGATTTGTTTTGAG	GATGAATATTGTCCACCACCACTA
SRO5	At5g62520	TTACGTGAGAACCCAAGTCGAAGC	CGGCGAGAGATACAATCCACGGC
ACS6	At4g11280	CCTTCAAACCCGCTTGGTACGACG	GCCTGGAGACACGTTGAGCTTCAC
CPK28	At5g66210	TGTGGGAGGCGGCCTTTTTGG	GACCTGCAGGGCTTGGTGCTC
WRKY33	At2g38470	CTACTTCTGATTCTGTTGGTGACG	GGTTGTGATTACTGCTCTCATGTC
LOX1	At1g55020	GCCACTGGATGCAAACACACGC	CTCGCGCACCTCCTTCCACC
CML37	At5g42380	GAGATCAGAATCGTCAAGAAACCT	CGAATCATAACCTTACAAGCATCA
WNK2	At3g22420	CTTTACCATGATCAGTTTGAGTCG	TGTTGTTGTTTACATTTTGGGAAC
	At1g73120	GAAATCAATTCACAAGAAGAGTGC	TGGTTTCCCATTAGAACAATAGAC
WRKY40	At1g80840	TTGATGTGTGACAACTACAACGTC	AAGTAAGCTCTTGGAGATGGATTG
BCS1	At3g50930	TCAAAACTCTAGCAATGGATTCAG	TACCGTGGATCTTCTATATCGTCA
PP2C	At2g30020	GTGCTGTTATGAGTGTAGGAGGTG	GCAATGTCTACTGCTTCTTGGTTA
PP2C	At3g16800	CGTGCAATTGTTTGGGAGGTACAC	CAGCGGTGAGAGCGGTGCAG
AHG3	At3g11410	CGGTTGCATGATATAGTGAAGAAG	CAAGATCAAACACTCATCCTCATC
HAB1	At1g72770	CCGGAAGTGACATTCATGCCTCGG	ACTTCTCCTCCCCAACCCGC
L19	At5g11750	ACAAGGTTGCTTCAGAGGCATCG	CTTGAGAGCATTCACCTTGTCCC
RAP2.4	At1g78080	AGACTCTGGTGGTGAACTCATGG	CGATCTCGTACGACGGATACTTC
	At4g32020	ATGGGCGTCGCTGTTCTAAATCC	AGGCGATGTCTAGGCGAAGGATC
JAZ6	At1g72450	AGCCGGTACTTGAAGGAGAAGG	TCTTTCTTGTCCACCTCCATCG
CCL	At3g26740	CTTCTTCCTCTTCCTTCTTCAG	ATTAACTTTGGGAACTCGGGTC
HSP20	At1g59860	GCTTCTTCGGCAACAACAGGCG	AGACCCAGAGAGAACTCAAATCAAGCT
HSP17.6II	At5g12020	GGCAATGGCTGCTACACCTGC	CCATATCCCTCACGCATTCCGATTAC
SBT5.2	At1g20160	CGCTAGGGACGAGGTGAAGAGC	CCTGGTGCGGTAATGTCAGGCTG
PRO-12	At2g40000	CGATCGATAACGAGATGAGTCG	ACTCTCCCAGAAACGTCTTTGC
SAUR	At4g34800	GATTAGATTGTCGCGTGTGATC	GTTTCTTCTCTGCATGGAATCG
LTP	At5g55450	GTTTTAACAGCTGCAATAATGGTG	GATATTTATGCGAATCAGAACAGC
LEA	At3g17520	AGGAAAGTGTACGGTTTGGTTATGG	GATGTTGTCTTCGTGCTTGAGACC
RAB18	At5g66400	ATGGCGTCTTACCAGAACCGTC	AGCTAGAGCTGGATCCAGATCC
LEA	At3g02480	CAGACTAAGGTCTCTCTCTCTCTC	TTGTCCTTGACGACATCAGCAGC
DAA1	At1g64110	AGTAGGTGTGGGTTTAGGCTTGG	ACAATTCTTGAACCGAGAATGAGG
HIS1-3	At2g18050	ACAAGATCTTAAAGAAGACTCC	TCAAGCAGCGGAAGCTTTCATGG
Lsm1a	At1g19120	GAAAGCAGAGAAGGAAGAAATGC	GAACAGGCTCAACAATTTCATTCC
Lsm1b	At3g14080	GAGAGGGAAGCGAGTGAGCTGAG	GCTTAACATTTTTTTCTTAAAAAGG
Lsm8	At1g65700	ATGGGTTGCTGTCTCAGCAAGAAACC	ATGGCGGCAACTACTGGACTTGAG
tub8	At5g23860	AAGATTCGTCCCACGCGCCG	CCCGGGAAACGAAGGCAGCA
U6		AGGGGCCATGCTAATCTTCTC	

5S		GACCTCCCGGGAAGTCCTCGTGTT	
25S		GATGACCAATTGTGCGAATCAACGG	
**semi-quantitative RT-PCR**			
Name	Gene ID	Primer Forward	Primer Reverse
Lsm1a	At1g19120	CTAATCGAGATCAAGGAACTCC	ATGTCTTGGGCTGCTCCTGATG
Lsm1b	At3g14080	ATGTCGTGGGCTGGTCCTGAAG	TTAATCAAAGTCAAGAAACTCCA
Lsm2	At1g03330	ATGTTGTTCTTTTCTTACTTCAAGG	TCAGCCACCCCTAGCTTCTCTTCTAGC
Lsm3a	At1g21190	GGGAAGGTATCTCTGTCTACACG	TTTGAGTTTGAACTCAGGCTGC
Lsm3b	At1g76860	AGAAAATGTCAGTCGAGGAAGACG	TCCCAGTACACAAGAACATTTAGG
Lsm4	At5g48870	AATCTTCTGATTTAAGAAGATGC	TCAACCACGGCCGCGACCTGC
Lsm5	At5g48870	ATGGCGAACAATCCTTCACAGC	TCATTCTCCATCTTCGGGAGACCC
Lsm6a	At2g43810	ATGAGTGGAGTTGGAGAGAAAGC	CTATGCTCCATCTGACAATGTCCC
Lsm6b	At3g59810	TCTCAGTTACGTAAAGGATCGAGC	CTAGGCTCCGTCTGCTACAGTCATG
Lsm7	At2g03870	ATGTCTGGAAGAAAAGAAACGG	TTAGACAGCCTCTGCAGTAACG
Lsm8	At1g65700	ATGGCGGCAACTACTGGACTTGAG	TCAATGCACTACGGGTTTCAACGG
elf-4A	At3g13920	TCATGAGAGCTTTGATGCCATGG	GATGAGAACACGGGAGGAACCAG
ePP2C	At3g17250	ATCCTAGTAACTTTCAGATTGCC	ACACACCGTAGAAAGCCATAGGC
iPP2C	At3g17250	TCCGTACCATTCTAGATTCTCC	ACACACCGTAGAAAGCCATAGGC
eABI1	At4g26080	AGACCTGAGATGGAAGATGCTG	GCAGTTAGCGACGAAGATGTGAG
iABI1	At4g26080	TAGGGTTGTTTACAGTTTGCAG	GCAGTTAGCGACGAAGATGTGAG
ePP2C	At5g59220	TAGCTTCACGCGCATGGACATGG	GGACACGTGGGCCATCCCAG
iPP2C	At5g59220	CGAGAATTTCGTTACGTGTTGCGTTC	GGACACGTGGGCCATCCCAG
SRp30	At1g09140	CGCAAGTGTGAGGTTGAAGA	ATGCAGCCGAGACAGAGTTT
RSp31a	At2g46610	ATTAAGATAAAGATGAGGCCAGTG	TTCAACTGATAACCTGCGTTTCTC
SRp34a	At3g49430	TTGGCTTCAGACCAAATCTTC	TTCTTTTGGCCATTTTCACC
RSp40	At4g25500	ACTACGCCTGCCAAAATCAT	CACCATCATACCCACCATCA
U1-70K	At3g50670	ACTACGAATCCTCAGAGAGTAAG	TTTCATGTCACGGGTGTGCAT
L8SalI	At1g65700	ACTGTGTCGACATGGCGGCAACTACTGGACTTGAG	ACTGTGATATCCATGCACTACGGGTTTCAACGGATG
U6		GTCCCTTCGGGGACATCCGATAAAATTG	AAAATTTGGACCATTTCTCGATTTATG
25S		CAAAGGCACGTGTCGTTGGCTAAG	CGACAAAGGGCTGAATCTCAGTGG
**qRT-PCR**			
Name	Gene ID	Primer Forward	Primer Reverse
PP2C-m	At3g17250	TGGCTGCTTTACACACAGCTTT	CAACGGAGAGATGAGACTTGAC
PP2C-p	At3g17250	GCTCTTCTACTCATGGATTTGTTCT	CAAACTCAGTAACATGCTCTTCAA
ABI1-m	At4g26080	GGTGATACGTGGCTGGAGAAGT	GCAGTTAGCGACGAAGATGTGAGA
ABI1-p	At4g26080	CGACTATACCAAGATTCCTTCAAT	GCAAACTGTAAACAACCCTAATCAA

LSM8-short	At1g65700	GTAACATGGCGGCAACTACTGG	CTCCTTCCTTTGTGGAAAACACACG
LSM8-long	At1g65700	GGCCTTGTCCTACAGTTCTTTGTTTG	CCTATGTTGTCCCCTCTGATGATGTAC
UBC9	At4g27960	ACTCCTCCAGAATAAGGGCTATCCG	TTCATGTAGCGCAGGACCCGTTG
SRO5	At5g62520	CAGAGTTTGATTCTGGTGTGGATG	TCTCTTTGGACTTCGAGTTAGG
HSP17.6II	At5g12020	GAAACTTCCTCCTCCGGAACC	CACCATATCCCTCACGCATTC
ACS6	At4g11280	AGACGAGTTTATCCGCGAGAG	AAACCGGCTTTGGCCTTTAAC
	At4g32020	TCGTAATCCTAGCGCATGTCC	GCGCTACAGGAGGAGAAGAAG
**Amplification**			
Name		Primer Forward	Primer Reverse
C-SF-TAP		GCCAGCTGGAGCCACCCTCAG	TCATTTATCATCATCATCTTTATAATCCTCTCCGC
LSM1SalI/ LSM1EcoRV		TGTGTCGACATGTCTTGGGCTGCTCCTGATG	CTGTGATATCCATCGAGATCAAGGAACTCCATTC
LSM5SalI/ LSM5EcoRV		ACTGTGTCGACATGGCGAACAATCCTTCACAGC	ACTGTGATATCCTTCTCCATCTTCGGGAGACCC
LSM8SalI/ LSM8EcoRV		ACTGTGTCGACATGGCGGCAACTACTGGACTTGAG	ACTGTGATATCCATGCACTACGGGTTTCAACGGATG

At3g45970 (expansin family protein, EXPL1) and At3g54810 (zinc finger GATA type family protein) mRNAs were detected using ESTs 108O17XP and 110D19XP probes, respectively (44).

Supplementary Table S3

Supplementary Table S3A

AtLSM1a and co-purified proteins

AGI no.	Description	1st purification score	1st purification peptides no.	2nd purification score	2nd purification peptides no.	3rd purification score	3rd purification peptides no.
AT1G19120	Small nuclear ribonucleoprotein family protein AtLSM1a	8231	8	15330	7	10357	9
AT1G03330	Small nuclear ribonucleoprotein family protein AtLSM2	4553	9	6234	11	5274	10
AT3G59810	Small nuclear ribonucleoprotein family protein AtLSM6b	143	4	457	5	2802	7
AT2G43810	Small nuclear ribonucleoprotein family protein AtLSM6a	213	5	291	5	2635	8
AT1G54270	EIF4A-2	251	8	869	10	1465	15
AT2G03870	Small nuclear ribonucleoprotein family protein AtLSM7	263	8	565	7	801	7
AT5G48870	Small nuclear ribonucleoprotein family protein SAD1/AtLSM5	205	4	528	4	684	5
AT3G22270	Topoisomerase II-associated protein PAT1	335	7	369	5	676	16
AT4G14990	Topoisomerase II-associated protein PAT1	105	5	506	8	464	11
AT1G76860	Small nuclear ribonucleoprotein family protein AtLSM3b	211	4	498	4	491	4
AT1G79090	Topoisomerase II-associated protein PAT1	146	5	50	1	449	11
AT5G27720	Small nuclear ribonucleoprotein family protein AtLSM4	253	7	230	7	386	7
AT1G01320	Tetratricopeptide repeat (TPR)-like superfamily protein	-	-	207	3	367	8
AT1G50200	ALATS, Alanyl-tRNA synthetase	-	-	95	3	298	12
AT1G21190	Small nuclear ribonucleoprotein family protein AtLSM3a	127	4	179	3	260	4
AT2G23350	poly(A) binding protein 4 (PABP4)	76	4	60	2	248	6
AT5G20920	EIF2 BETA, eukaryotic translation initiation factor 2 beta subunit	77	1	-	-	245	4
AT2G43030	Ribosomal protein L3 family protein	81	2	110	5	215	6
AT4G13780	methionine--tRNA ligase, putative / methionyl-tRNA synthetase, putative / MetRS, putative	67	1	-	-	214	6
AT1G02080	Transcription regulators	-	-	58	1	186	2
AT1G67430	Ribosomal protein L22p/L17e family protein	-	-	98	3	163	2
AT1G14610	TWN2, VALRS, valyl-tRNA synthetase / valine--tRNA ligase (VALRS)	-	-	127	4	148	6
AT3G11510	Ribosomal protein S11 family protein	89	3	97	2	143	2
AT2G27710	60S acidic ribosomal protein family	101	1	135	2	45	1
AT3G13300	VCS, Transducin/WD40 repeat-like superfamily protein	-	-	69	1	132	4
AT5G02960	Ribosomal protein S12/S23 family protein	-	-	70	1	122	2
AT3G11500	Small nuclear ribonucleoprotein family protein, putative homolog of yeast and human SmG	56	1	-	-	91	2
AT3G02560	Ribosomal protein S7e family protein	-	-	90	1	82	1
AT2G37220	RNA-binding (RRM/RBD/RNP motifs) family protein	59	2	27	1	90	2
AT2G45810	DEA(D/H)-box RNA helicase family protein	24	1	55	1	88	3
AT1G22760	PAB3, poly(A) binding protein 3	50	3	43	1	78	3
AT3G25520	ATL5, PGY3, OLI5, RPL5A, ribosomal protein L5	60	1	-	-	58	1

Supplementary Table S3B

AtLSM5 and co-purified proteins

AGI no.	Description	score	peptides no.
AT1G03330	Small nuclear ribonucleoprotein family protein AtLSM2	4544	9
AT1G65700	Small nuclear ribonucleoprotein family protein AtLSM8	1356	6

AT2G03870	Small nuclear ribonucleoprotein family protein AtLSM7	855	8
AT3G14080	Small nuclear ribonucleoprotein family protein AtLSM1b	794	6
AT5G48870	Small nuclear ribonucleoprotein family protein SAD1/AtLSM5	628	5
AT1G19120	Small nuclear ribonucleoprotein family protein AtLSM1a	616	3
AT1G76860	Small nuclear ribonucleoprotein family protein AtLSM3b	483	4
AT2G43810	Small nuclear ribonucleoprotein family protein AtLSM6a	433	6
AT5G27720	Small nuclear ribonucleoprotein family protein AtLSM4	315	7
AT3G59810	Small nuclear ribonucleoprotein family protein AtLSM6b	215	4
AT1G21190	Small nuclear ribonucleoprotein family protein AtLSM3a	175	3
ATCG00800	structural constituent of ribosome	130	2
AT2G18110	Translation elongation factor EF1B/ribosomal protein S6 family protein	128	3
AT5G20290	Ribosomal protein S8e family protein	81	1
ATCG00770	RPS8, ribosomal protein S8	80	1
AT4G20440	small nuclear ribonucleoprotein associated protein B	79	1
AT5G15200	Ribosomal protein S4	78	1
AT5G39740	RPL5B, ribosomal protein L5 B	71	1
AT3G23145	zinc ion binding;isoleucine-tRNA ligases;ATP binding;aminoacyl-tRNA ligases;nucleotide binding	69	2
AT3G62840	Small nuclear ribonucleoprotein family protein	66	1
AT5G62300	Ribosomal protein S10p/S20e family protein	64	1
AT2G37270	ATRPS5B, RPS5B, ribosomal protein 5B	61	1
AT1G72730	DEA(D/H)-box RNA helicase family protein	58	2
AT3G28020	BEST Arabidopsis thaliana protein match is: DNA binding;ATP binding	55	1
AT5G22750	RAD5, DNA/RNA helicase protein	53	2

Supplementary Table S3C

AtLSM8 and co-purified proteins

		**1st purification**		**2nd purification**		**3rd purification**	
**AGI no.**	**Description**	**score**	**peptides no.**	**score**	**peptides no.**	**score**	**peptides no.**
AT1G65700	Small nuclear ribonucleoprotein family protein AtLSM8	3221	7	8224	6	10002	6
AT1G03330	Small nuclear ribonucleoprotein family protein AtLSM2	272	9	2072	11	2136	9
AT4G24270	Embryo defective 140 (EMB140), putative homolog of yeast/human Prp24/SART3(p110)	745	22	1303	27	1435	28
AT1G54270	EIF4A-2	453	6	1165	12	777	13
AT2G43810	Small nuclear ribonucleoprotein family protein AtLSM6a	578	5	1090	7	702	7
AT3G59810	Small nuclear ribonucleoprotein family protein AtLSM6b	341	6	765	6	951	5
AT1G72730	DEA(D/H)-box RNA helicase family protein	453	5	923	11	763	11
AT2G03870	Small nuclear ribonucleoprotein family protein AtLSM7	659	10	650	5	427	5
AT1G76860	Small nuclear ribonucleoprotein family protein AtLSM3b	571	8	247	4	178	4
AT5G48870	Small nuclear ribonucleoprotein family protein SAD1/AtLSM5	418	6	444	4	434	4
AT5G27720	Small nuclear ribonucleoprotein family protein AtLSM4	322	7	227	7	240	6
AT5G07350	Tudor1, AtTudor1, TSN1, TUDOR-SN protein 1	46	2	201	7	266	8
AT5G14140	zinc ion binding;nucleic acid binding;zinc ion binding	94	1	230	1	-	-
AT1G20960	Embryo defective 1507 (EMB1507), putative homolog of yeast/human Brr2(Prp44)/SNRNP200	-	-	214	2	222	5
AT1G67430	Ribosomal protein L22p/L17e family protein	-	-	214	2	88	2
AT1G50200	ALATS, Alanyl-tRNA synthetase	45	1	198	10	121	5
AT2G01250	Ribosomal protein L30/L7 family protein	-	-	193	5	90	4

AT5G61780	Tudor2, AtTudor2, TSN2, TUDOR-SN protein 2	46	2	135	8	189	5
AT2G37220	RNA-binding (RRM/RBD/RNP motifs) family protein	172	3	91	1	36	1
AT1G21190	Small nuclear ribonucleoprotein family protein	75	4	160	3	106	3
AT3G10090	Nucleic acid-binding, OB-fold-like protein	108	1	157	1	28	1
AT2G23350	PAB4, PABP4, poly(A) binding protein 4	67	2	152	4	103	2
AT5G20920	EIF2 BETA, eukaryotic translation initiation factor 2 beta subunit	-	-	150	5	89	3
AT4G11420	EIF3A, ATEIF3A-1, EIF3A-1, ATTIF3A1, TIF3A1, eukaryotic translation initiation factor 3A	-	-	145	5	116	2
AT2G18110	Translation elongation factor EF1B/ribosomal protein S6 family protein	141	4	124	4	116	3
ATCG00770	RPS8, ribosomal protein S8	-	-	55	1	126	1
AT1G80070	Embryo defective 14 (EMB14), putative homolog of yeast and human PRP8	-	-	121	4	56	3
AT3G25520	ATL5, PGY3, OLI5, RPL5A, ribosomal protein L5	59	1	115	4	75	1
AT1G29965	Ribosomal protein L18ae/LX family protein	-	-	109	2	51	2
AT2G43030	Ribosomal protein L3 family protein	34	1	108	5	69	3
AT1G18540	Ribosomal protein L6 family protein	-	-	105	2	68	4
AT1G74060	Ribosomal protein L6 family protein	-	-	105	4	68	3
AT3G11500	Small nuclear ribonucleoprotein family protein	100	2	68	1	62	1
AT3G07590	Small nuclear ribonucleoprotein family protein, putative homolog of yeast and human SmD1	99	1	30	1	54	1
AT1G75350	emb2184, Ribosomal protein L31	90	1	93	1	56	1
AT1G01320	Tetratricopeptide repeat (TPR)-like superfamily protein	-	-	83	3	96	3
AT4G13780	methionine--tRNA ligase, putative / methionyl-tRNA synthetase, putative / MetRS, putative	-	-	92	2	75	2
AT2G25910	3'-5' exonuclease domain-containing protein / K homology domain-containing protein / KH domain-containing protein	-	-	80	1	92	1
AT3G61240	DEA(D/H)-box RNA helicase family protein	51	1	58	1	84	2
AT3G27850	RPL12-C, ribosomal protein L12-C	80	3	55	2	27	1
AT1G57860	Translation protein SH3-like family protein	62	1	67	3	-	-
AT2G19730	Ribosomal L28e protein family	-	-	61	2	62	1

Supplementary Table 3. Set of proteins involved in RNA turnover co-purified with AtLSM1-SF-TAP (A), AtLSM5-SF-TAP (B) and AtLSM8-SF-TAP (C). Listed proteins were present in at least two of three independent purifications (A and B) that were specifically bound to anti-FLAG-M2 resin, as identified by tandem mass spectrometry following subtraction of peptides present in respective controls to account for unspecific hits. In the case of AtLSM5-SF-TAP, the purification was repeated only once due to partial complementation of morphological phenotypes of the *sad1/lsm5* line expressing AtLSM5-SF-TAP.

Supplementary Table S4
The list of all proteins bound to anti-Flag affinity resins identified by tandem mass spectrometry.
This Table is available as a separate xls file.

Supplementary Table S5
Affymetrix microarray data. The list of all transcripts with changed expression in *lsm1a lsm1b*, *lsm8* and *sad1/lsm5* mutants compared to their corresponding wild-types (Col-0 or WT). This Table is available as a separate pdf file.

Supplementary Table S6
Affymetrix microarray data. The list of all transcripts stabilized more than 1.3 fold in *lsm1a lsm1b*, *lsm8* and *sad1/lsm5* mutants following transcriptional inhibition. This Table is available as a separate pdf file.

Supplementary Figures

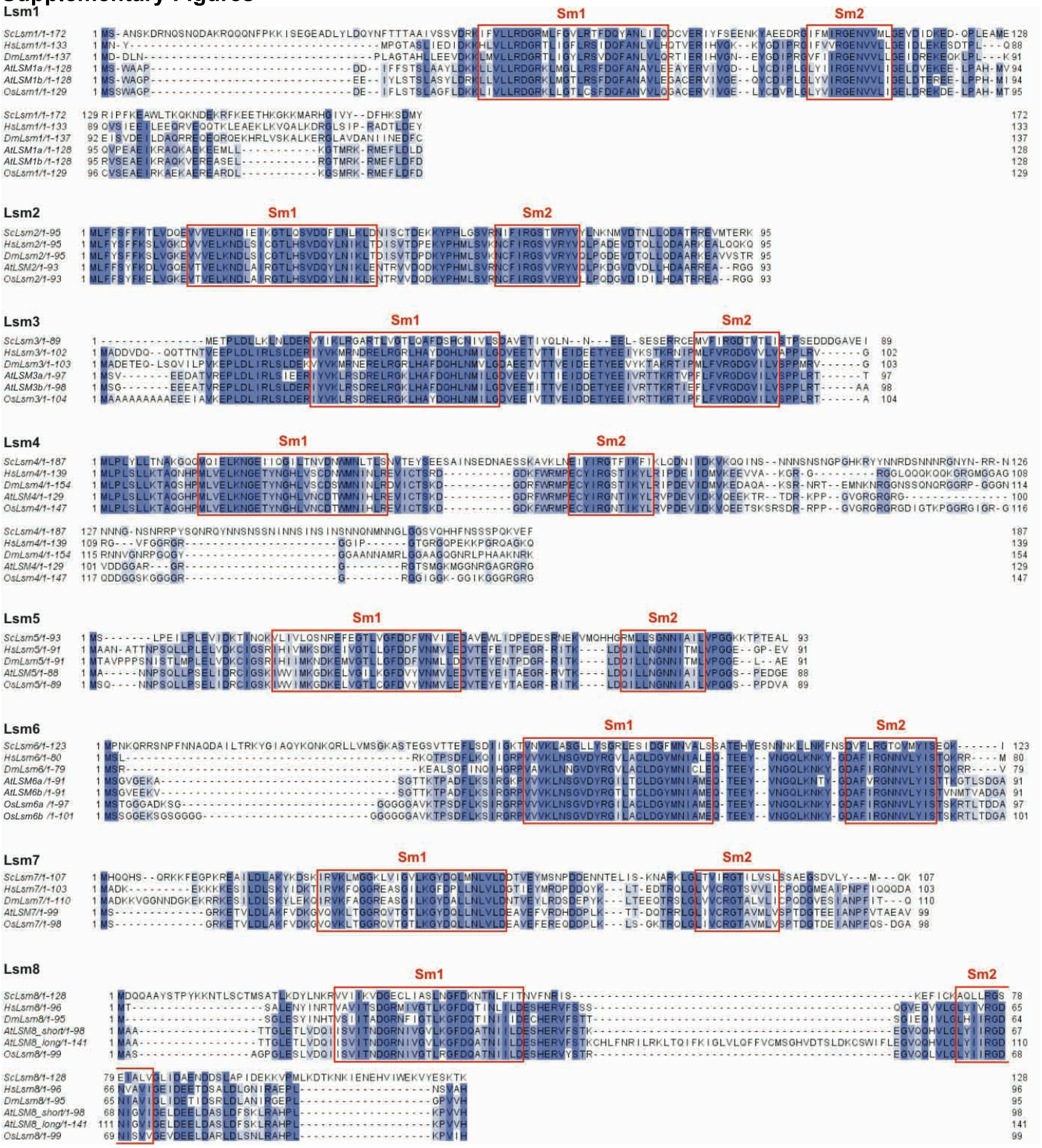

Supplementary Figure S1.

Sequence alignment of LSM proteins. Sm motives 1 and 2 are indicated by red rectangles. Identical amino acids are marked in blue and their number is reflected by the color intensity. (Sc) *Saccharomyces cerevisiae,* (Hs) *Homo sapiens,* (Dm) *Drosophila melanogaster,* (At) *Arabidobsis thaliana,* (Os) *Oryza sativa*. Accession numbers of LSM1-8 proteins: (Sc) YJL124C, YBL026W, YLR438C-A, YER112W, YER146W, YDR378C, YNL147W, YJR022W (www.yeastgenome.org); (Hs) NP_055277, NP_067000, NP_055278, NP_036453, NP_036454, NP_009011, NP_057283, NP_057284 (www.ncbi.nlm.nih.gov/genbank); (Dm) FBpp0071553, FBpp0075686, FBpp0083893, FBpp0293054, FBpp0076756, FBpp0071449, FBpp0080488, FBpp0072674 (http://flybase.org); (At) At1g19120, At3g14080, At1g03330, At1g21190, At1g76860, At5g27720, At5g48870, At2g43810, At3g59810, At2g03870, At1g65700 (www.arabidopsis.org); (Os) Os04g0445800, Os08g0154700, Os01g0866700, Os01g0256900, Os05g0389300, Os04g0388900, Os02g0510100, Os08g0177700, Os05g0594900 (http://rapdb.dna.affrc.go.jp).

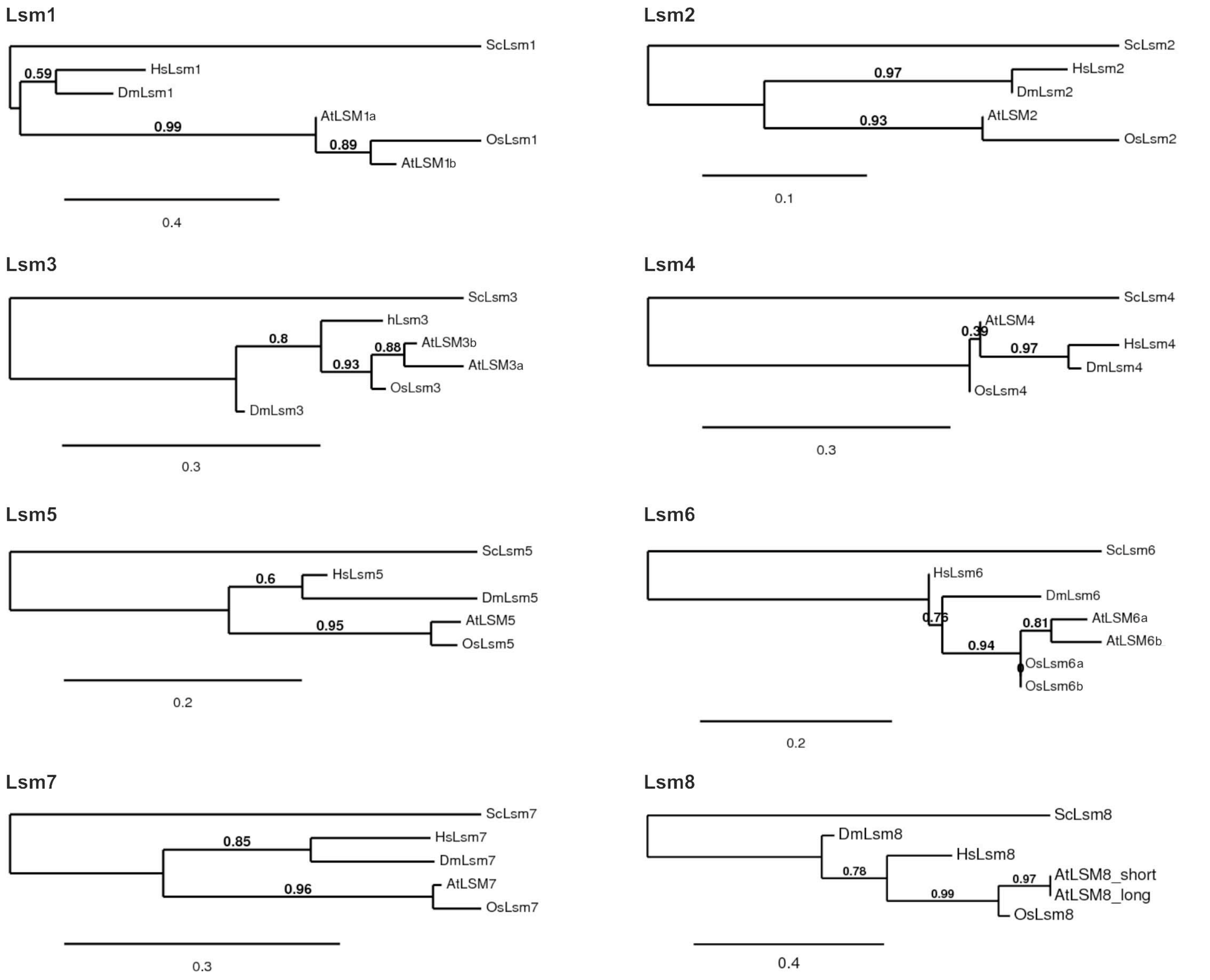

Supplementary Figure S2.
Phylogenetic analysis of LSM proteins presented as an unrooted maximum-likelihood trees. Bootstrap values are indicated along the branches. The scale bar shows the evolutionary distance (amino acid substitutions per site).

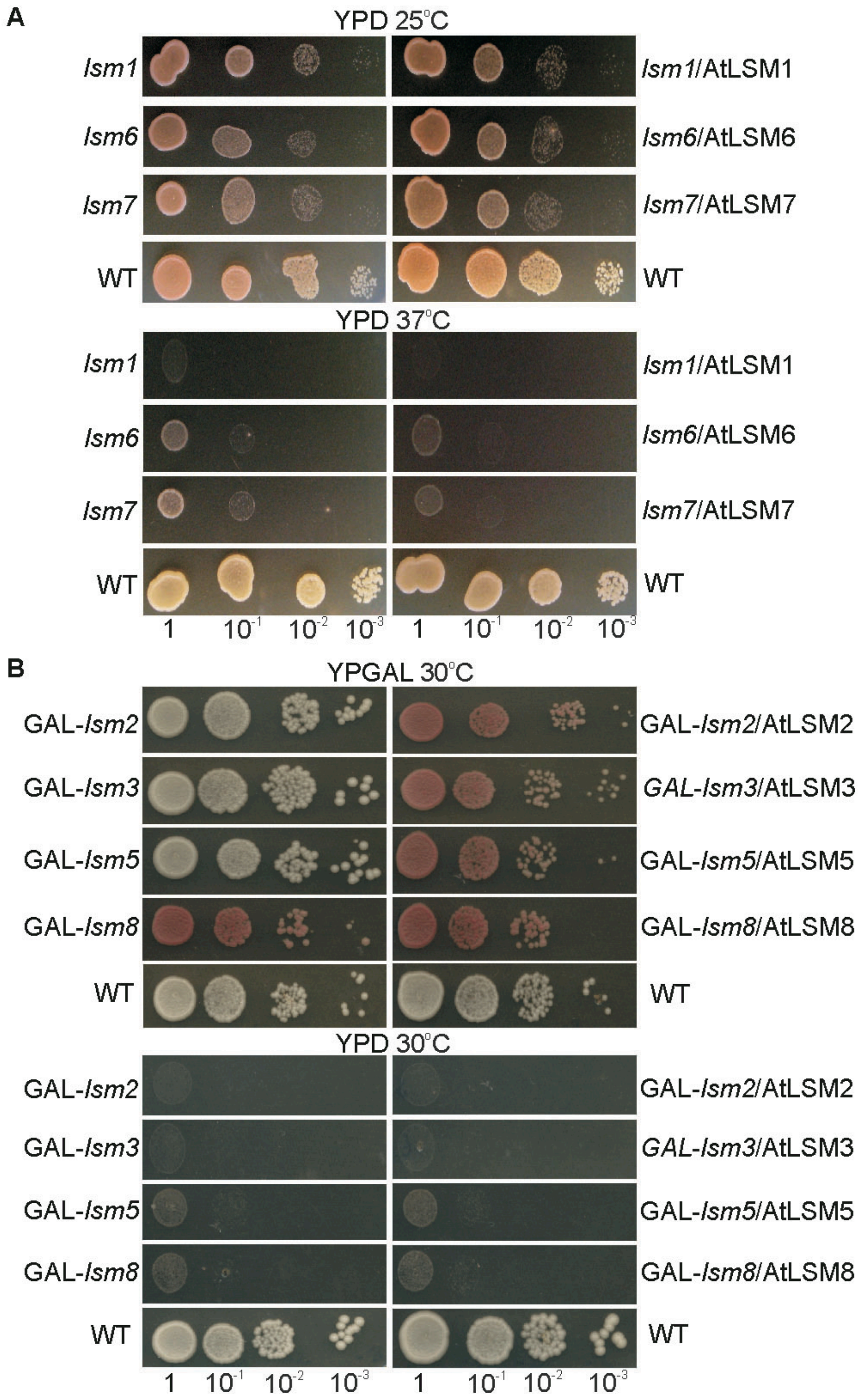

Supplementary Figure S3.
Complementation tests of the growth defects of *GAL::lsm2*, *GAL::lsm3*, *GAL::lsm5* and *GAL::lsm8* strains or temperature sensitive phenotypes of *lsm1Δ, lsm6Δ* and *lsm7Δ* strains by respective AtLSM proteins expressed under the control of the constitutive *ADH1* promoter from a low copy pNOPPATAIL vector. (A) *lsmΔ, lsm6Δ* and *lsm7Δ* strains, either carrying the empty vector (left panel) or plasmids expressing *AtLSM1*, *AtLSM6* or *AtLSM7* (right panel) were pre-grown at 23˚C, spotted in serial dilutions and incubated either at 23˚C or 37˚C. (B) *GAL::lsm2*, *GAL::lsm3*, *GAL::lsm5*, *GAL::lsm8* strains either carrying the empty vector (left panel) or plasmids expressing *AtLSM2*, *AtLSM3*, *AtLSM5* or *AtLSM8* (right panel) were pre-grown in YPGal medium at 30˚C, and spotted in serial dilutions onto YPGal or YPD plates.

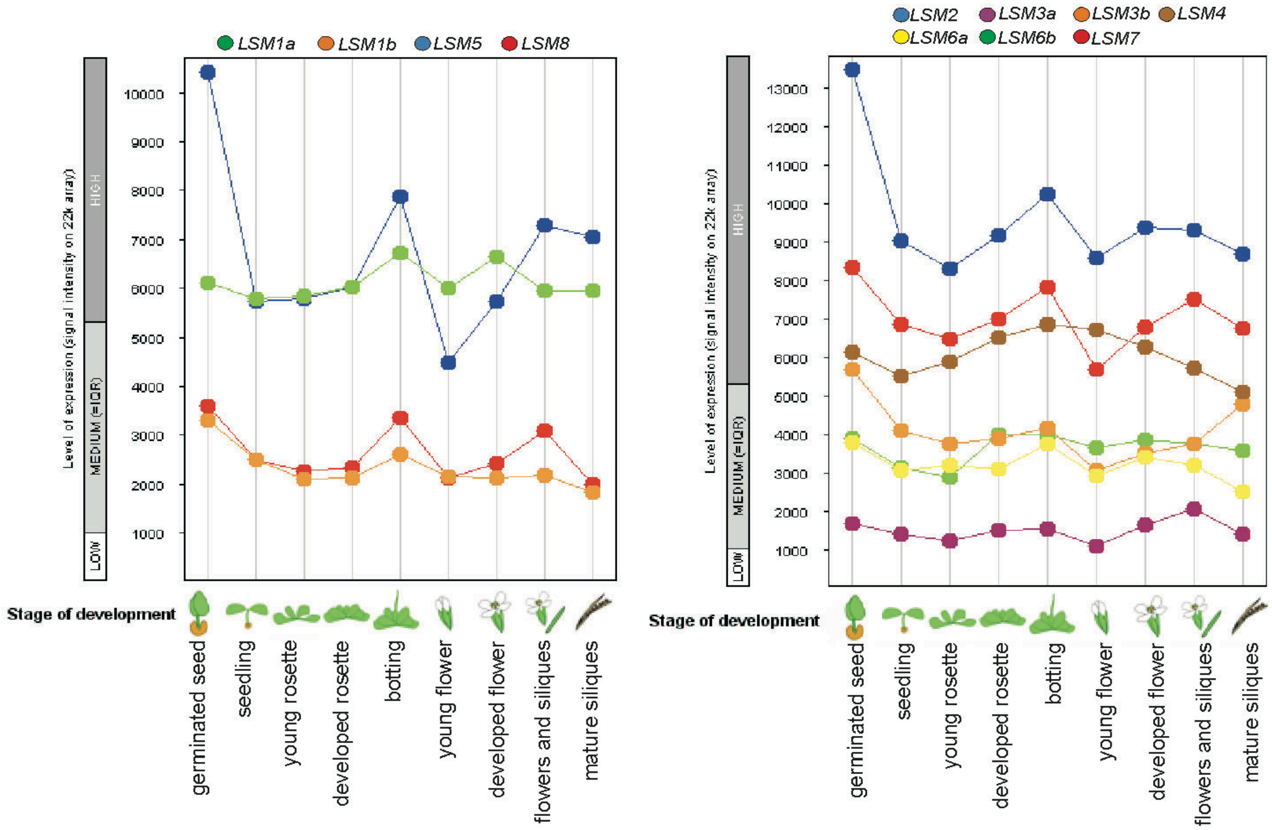

Supplementary Figure S4. Microarray expression profiles of *AtLSM* mRNAs according to https://genvestigator.ethz.ch/at/.
(A) LSM1a – At1g19120, LSM1b – At3g14080, LSM5 – At5g48870, LSM8 – At1g65700. (B) LSM2 – At1g03330, LSM3a – Atg1g21190, LSM3b – At1g76860, LSM4 – At5g27720, LSM6a – At2g43810, LSM6b – At3g59810, LSM7 – At2g03870.

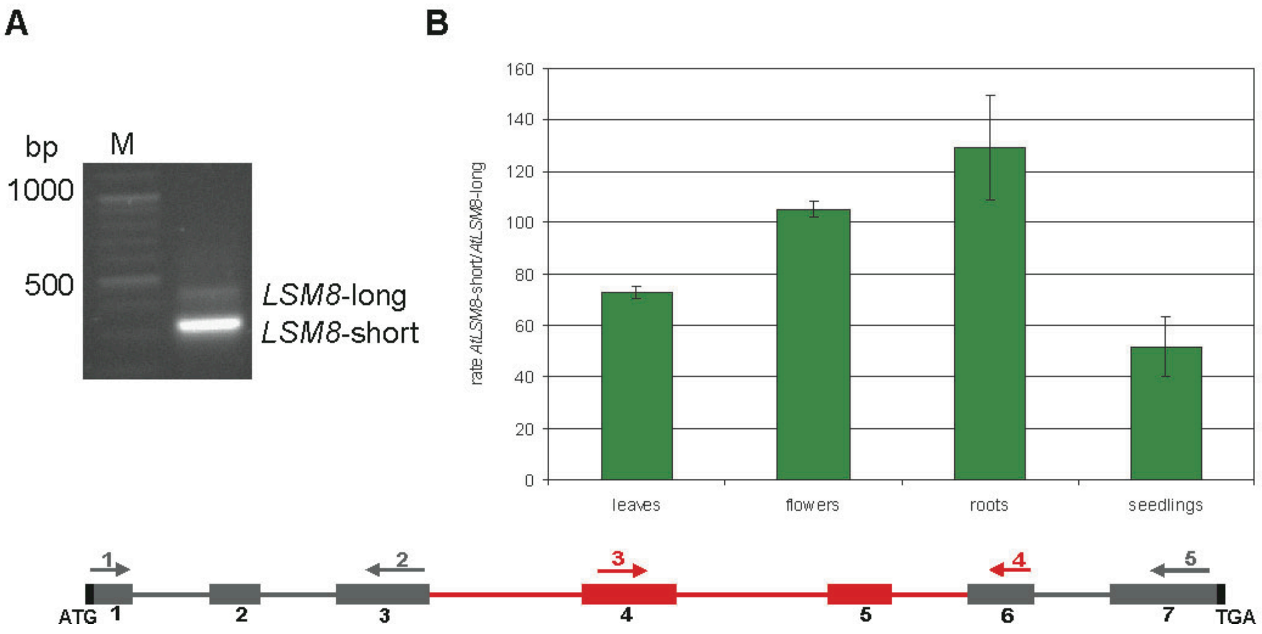

Supplementary Figure S5.

Identification of *AtLSM8-long* and *AtLSM8-short* splice variants. (A) Semi-quantitative RT-PCR on total RNA extracted from 2-week-old Col-0 seedlings. (B) Relative levels of two splicing *AtLSM8* forms assessed by quantitative RT-PCR in different organs. Total RNA from 4-week-old leaves, flowers, roots and 2-week-old seedlings was used. Black bars represent standard deviation. Specific primers 1 and 5 used to detect splice variants in RT-PCR (A) and 1 and 2 or 3 and 4 pairs used in qRT-PCR (B) are depicted on the *AtLSM8* gene structure.

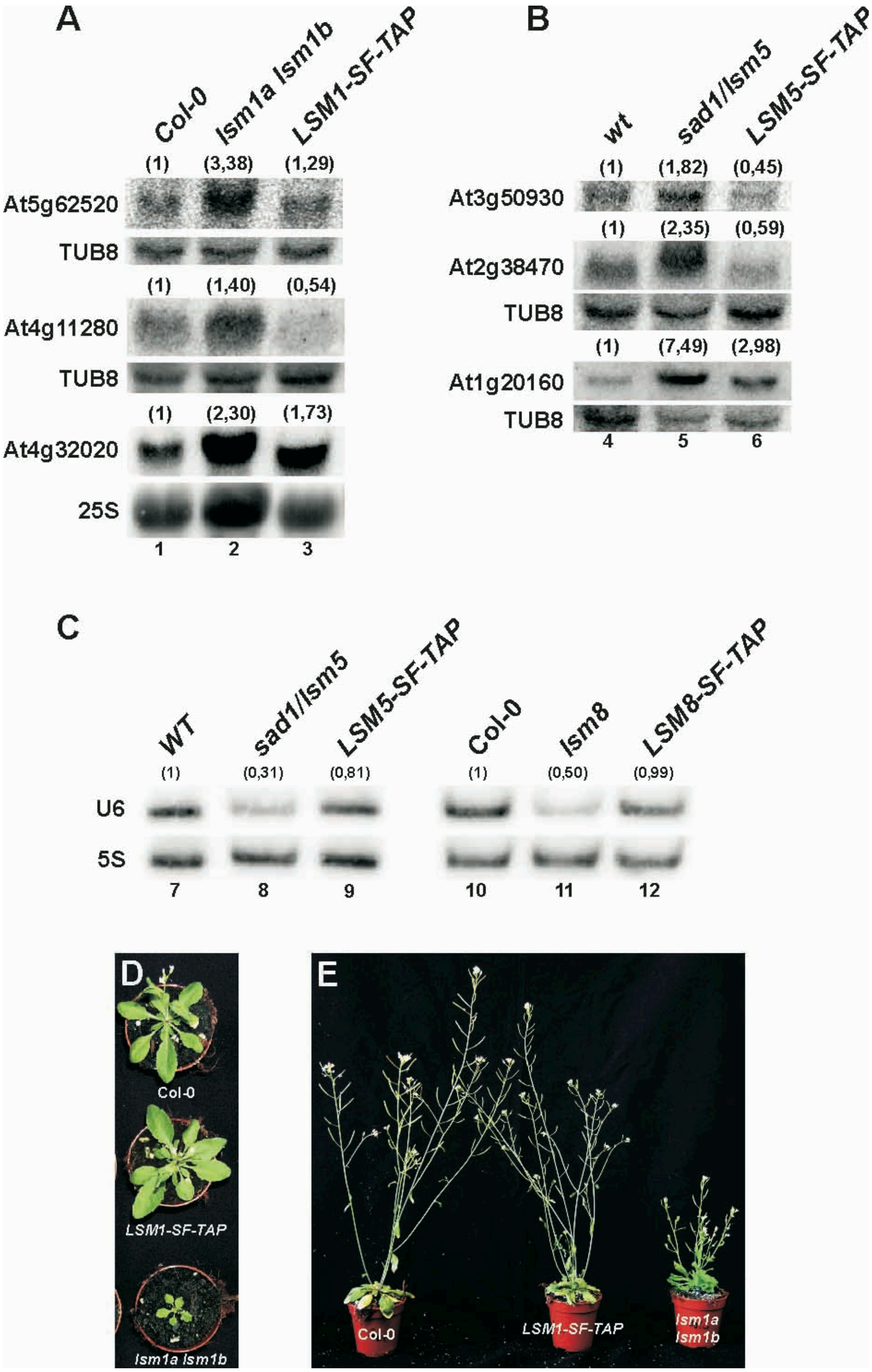

Supplementary Figure S6.

(A-C) Complementation of *sad1/lsm5*, *lsm8* and *lsm1a lsm1b* molecular phenotypes by the ectopic expression of AtLSM5, AtLSM8 or AtLSM1, respectively. Northern analyses of At5g62520 (*SRO5*), At4g11280 (*ACS6*) and At4g32020 mRNAs in Col-0, *lsm1a lsm1b* and *lsm1a lsm1b* expressing AtLSM1-SF-TAP (A); At3g50930 (*BCS1*), At2g38470 (*WRKY33*) and At1g20160 (*AtSBT5.2*) mRNAs in wild-type (WT), *sad1/lsm5* and *sad1/lsm5* expressing AtLSM5-SF-TAP (B); U6 snRNA Col-0, *lsm8*, *lsm8* expressing AtLSM1-SF-TAP, *sad1/lsm5* and *sad1/lsm5* expressing AtLSM5-SF-TAP (C). Numbers in parentheses represent the transcript level in the mutant relative to Col-0 or wild-type, respectively. Tubulin (*TUB8*) (A, B) and 25S rRNA (A) or 5S rRNA (C) were used as a loading control. (D-E) Expression of AtLSM1-SF-TAP reverts morphological phenotypes of the *lsm1a lsm1b* mutant line.

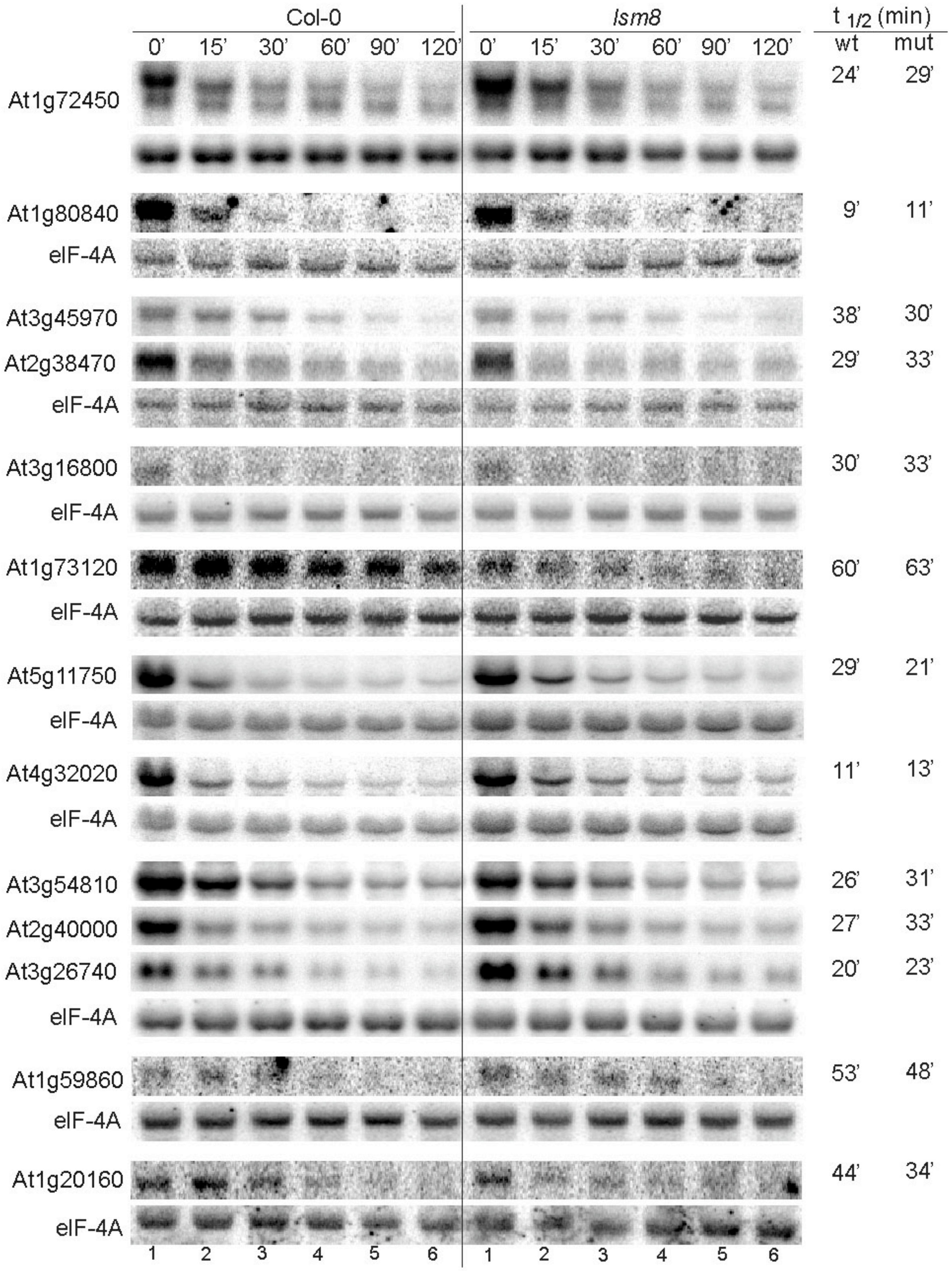

Supplementary Figure S7

Northern analysis of mRNAs at specific time-points after cordycepin treatment in the *lsm8* mutant and Col-0 plants. Estimated mRNA half-life ($t_{1/2}$) is shown to the right of each panel. eIF-4A mRNA was used as control.

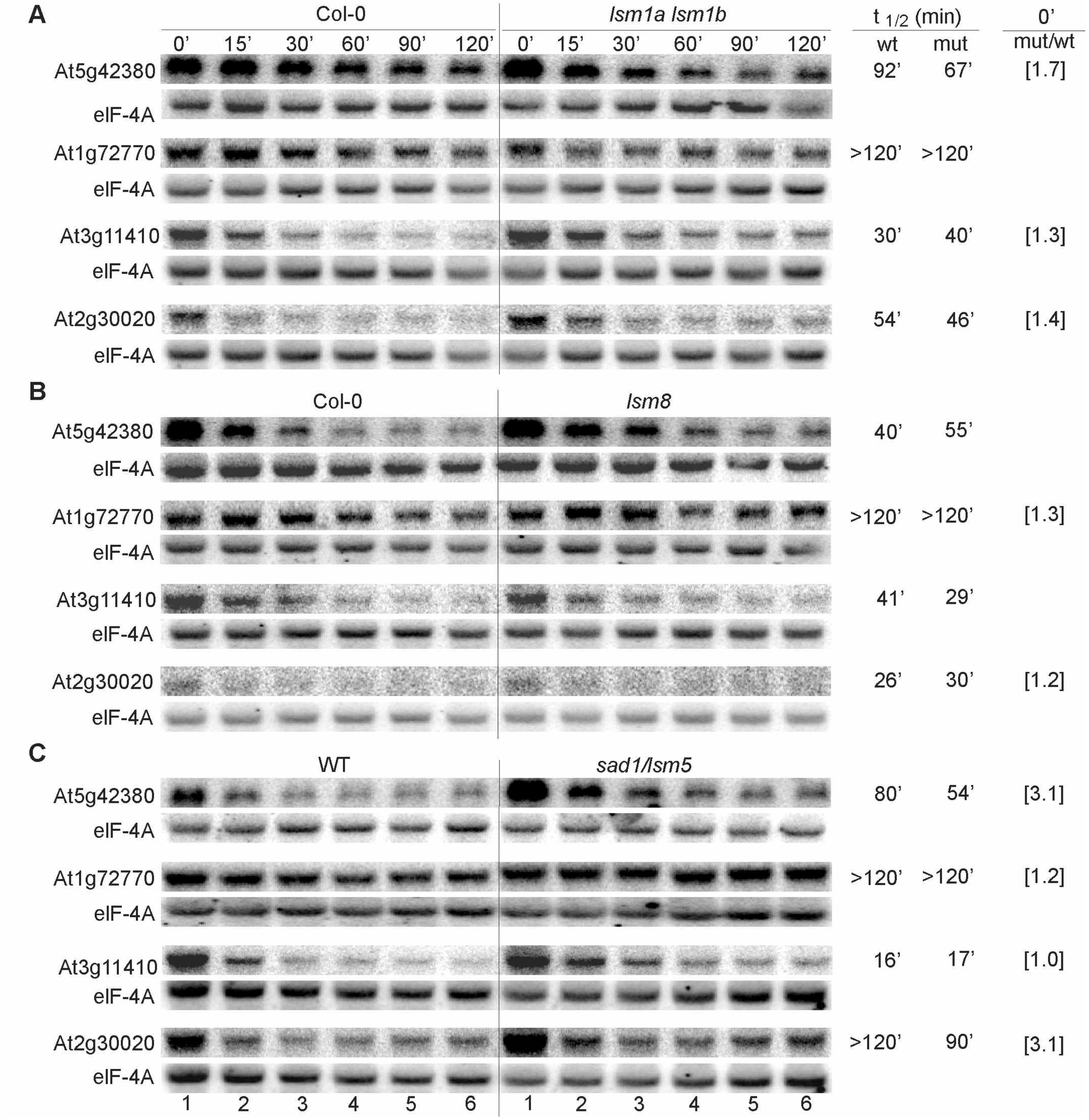

Supplementary Figure S8

Northern analysis of chosen mRNAs, which stability is not significantly altered in *lsm8, lsm1a lsm1b* and *sad1/lsm5* mutants. Respective wild-types (Col-0 or WT) were used as controls. Estimated mRNA half-life ($t_{1/2}$) is shown to the right of each panel. Numbers in parentheses represent the fold change in the transcript level of the mutant versus wild-type for mRNAs which accumulate at the steady state (0' min). eIF-4A mRNA was used as a loading control.

REFERENCES

4. Mayes, A.E., Verdone, L., Legrain, P. and Beggs, J.D. (1999) Characterization of Sm-like proteins in yeast and their association with U6 snRNA. *EMBO J.*, **18**, 4321-4331.

37. Gloeckner, C.J., Boldt, K., Schumacher, A., Roepman, R. and Ueffing, M. (2007) A novel tandem affinity purification strategy for the efficient isolation and characterisation of native protein complexes. *Proteomics*, **7**, 4228-4234.

38. Nakagawa, T., Ishiguro, S. and Kimura, T. (2009) Gateway vectors for plant transformation. *Plant Biotech.*, **26**, 275–284.

44. Souret, F.F., Kastenmayer, J.P. and Green, P.J. (2004) AtXRN4 degrades mRNA in *Arabidopsis* and its substrates include selected miRNA targets. *Mol. Cell*, **15**, 173-183.

78. Gietz, D., St Jean, A., Woods, R.A. and Schiestl, R.H. (1992) Improved method for high efficient transformation of intact yeast cells. *Nucleic Acids Res.*, **20**, 1425.

79. Baudin, A., Ozier-Kalogeropoulos, O., Denouel, A., Lacroute, F., and Cullin, C. (1993) A simple and efficient method for direct gene deletion in *Saccharomyces cerevisiae*. *Nucleic Acids Res.*, **21**, 3329-3330.

80. Lau, D., Künzler, M., Braunwarth, A., Hellmuth, K., Podtelejnikov, A., Mann, M. and Hurt, E. (2000) Purification of protein A-tagged yeast ran reveals association with a novel karyopherin beta family member, Pdr6p. *J. Biol. Chem.*, **275**, 467-471.
