# Supplementary material for: Arabidopsis thaliana LSM proteins function in mRNA splicing and degradation
Source: Nucleic Acids Res. 2013 Apr 24;41(12):6232–49. doi: 10.1093/nar/gkt296 (PMC3695525; doi:10.1093/nar/gkt296)
Supplement: Supplementary Data [file supp_gkt296_nar-03286-a-2012-File011.pdf]

Probesets	Transcript ID(Array Design)	sad1/ lsm5 natural m/wt 0min	sad1/ lsm5 natural m/wt 30min	sad1/ lsm5 natural m/wt 120min	lsm1a lsm1b natural m/wt 0min	lsm1a lsm1b natural m/wt 30min	lsm8 natural m/wt 0min	lsm8 natural m/wt 30min	lsm8 natural m/wt 90min	Gene Title
261585_at	At1g01010	0,79	0,81	0,74	1,13	1,31	1,23	0,97	1,12	no apical meristem (NAM) family protein
261568_at	At1g01030	0,85	0,96	0,96	1,06	1,17	1,06	0,98	1,06	DNA-binding protein, putative
261584_at	At1g01040	0,80	0,66	0,85	0,88	1,01	0,92	0,94	0,94	DEAD/DEAH box helicase carpel factory / CAF
261579_at	At1g01050	1,21	1,16	1,11	1,22	1,42	1,10	1,03	1,07	inorganic pyrophosphatase, putative (soluble)/ PPase, putative
261569_at	At1g01060	1,80	1,55	1,46	1,51	1,20	1,41	1,47	1,31	myb family transcription factor
261576_at	At1g01070	1,08	0,97	0,93	1,34	1,15	1,04	0,98	1,15	nodulin MtN21 family protein
261577_at	At1g01080	0,69	0,61	0,67	0,75	0,85	0,98	0,87	0,78	33 kDa ribonucleoprotein, chloroplast, putative/RNA-binding protein cp33
261583_at	At1g01090	0,96	1,01	0,91	1,05	1,07	1,04	0,91	0,99	pyruvate dehydrogenase E1 component alpha subunit, chloroplast
261578_at	At1g01100	1,31	1,21	1,14	1,31	1,20	0,95	1,03	0,93	60S acidic ribosomal protein P1 (RPP1A)
261580_at	At1g01110	0,91	0,93	1,04	1,10	1,11	0,83	1,00	1,03	expressed protein
261570_at	At1g01120	0,91	0,78	0,92	0,90	0,77	1,09	0,94	0,98	fatty acid elongase 3-ketoacyl-CoA synthase 1 (KCS1)
261575_at	At1g01130	0,98	0,88	0,93	1,20	0,96	1,01	0,89	0,99	expressed protein
261581_at	At1g01140	0,62	1,05	2,50	0,87	1,02	1,13	1,44	1,81	CBL-interacting protein kinase 9 (CIPK9)
261571_at	At1g01150	0,96	1,01	1,01	1,03	1,05	0,98	1,03	1,00	expressed protein
261582_at	At1g01160	1,15	1,03	1,20	0,99	0,96	1,05	1,13	1,41	SSXT protein-related / transcription co-activator-related
261572_at	At1g01170	1,04	0,96	0,96	0,97	1,17	0,99	0,90	0,93	ozone-responsive stress-related protein, putative
261573_at	At1g01180	1,03	0,96	1,03	0,88	1,05	1,12	0,94	0,98	expressed protein
261574_at	At1g01190	1,42	2,12	1,36	1,53	1,22	1,06	1,00	0,98	cytochrome P450, putative
261042_at	At1g01200	0,86	0,85	0,88	1,00	1,25	0,84	0,89	1,16	Ras-related GTP-binding protein, putative
261043_at	At1g01220	0,83	0,78	0,87	0,72	0,67	1,13	1,04	1,12	GHMP kinase-related
261025_at	At1g01225	0,90	0,80	1,04	1,04	0,99	0,86	0,91	0,90	NC domain-containing protein-related
261057_at	At1g01230	0,91	0,96	0,89	1,11	1,01	0,94	0,74	0,85	ORMDL family protein
261026_at	At1g01240	0,65	0,92	1,05	0,62	0,76	1,05	1,09	1,08	expressed protein
261059_at	At1g01250	0,74	0,90	0,87	1,05	1,24	0,85	0,81	0,97	AP2 domain-containing transcription factor, putative
261050_at	At1g01260	0,78	0,80	1,05	1,05	1,15	0,85	0,95	1,15	basic helix-loop-helix (bHLH) family protein
261051_at	At1g01280	0,91	0,96	1,00	1,04	1,15	1,02	0,97	1,01	cytochrome P450 family protein
261044_at	At1g01290	1,03	1,13	1,02	0,96	0,88	0,95	1,00	0,92	molybdopterin biosynthesis CNX3 protein
261055_at	At1g01300	1,10	1,01	0,98	1,33	1,32	0,88	0,74	0,74	aspartyl protease family protein
261045_at	At1g01310	0,97	1,00	0,97	1,03	0,98	0,91	0,96	0,99	allergen V5/Tpx-1-related family protein
261053_at	At1g01320	0,97	0,78	0,69	0,71	0,57	1,16	1,09	1,23	tetratricopeptide repeat (TPR)-containing protein
261054_at	At1g01330	0,89	0,81	0,67	0,75	0,67	1,00	0,89	1,19	tetratricopeptide repeat (TPR)-containing protein
261027_at	At1g01340	1,06	0,83	0,86	0,98	0,85	1,12	0,85	1,04	cyclic nucleotide-regulated ion channel (CNGC10) (ACBK1)
261056_at	At1g01360	1,39	1,44	1,13	1,32	1,07	1,18	1,39	1,28	expressed protein
261058_at	At1g01370	1,03	1,03	1,28	1,22	1,55	0,86	1,09	1,02	centromeric histone H3 HTR12 (HTR12)
257490_x_at	At1g01380	0,96	0,99	1,12	0,91	0,95	0,94	1,05	1,10	myb family transcription factor
261046_at	At1g01390	1,05	0,97	1,14	0,92	1,04	1,05	1,02	1,04	UDP-glucoronosyl/UDP-glucosyl transferase family protein
261047_at	At1g01410	1,07	1,06	1,02	0,93	1,15	0,95	0,96	1,17	hypothetical protein
261048_at	At1g01420	0,96	0,84	0,88	1,06	1,06	1,00	0,93	1,15	UDP-glucoronosyl/UDP-glucosyl transferase family protein
261049_at	At1g01430	0,95	1,09	1,11	0,90	0,87	0,90	0,84	0,91	expressed protein
261052_at	At1g01440	1,01	0,91	1,12	0,77	0,63	1,03	1,03	1,17	extra-large G-protein-related

259435_at	At1g01450	0,92	1,01	1,12	0,96	0,93	1,01	0,95	0,96	protein kinase-related
259425_at	At1g01460	1,02	1,10	1,11	1,02	1,13	0,95	0,92	0,90	phosphatidylinositol-4-phosphate 5-kinase family protein
259426_at	At1g01470	0,82	0,86	1,28	1,55	1,25	1,02	0,98	0,95	late embryogenesis abundant protein, putative / LEA protein, putative
259439_at	At1g01480	0,96	0,97	1,05	1,03	0,90	1,24	1,26	1,20	1-aminocyclopropane-1-carboxylate synthase2/ACC synthase2
259434_at	At1g01490	1,59	2,27	2,04	1,34	1,14	1,12	1,27	1,43	heavy-metal-associated domain-containing protein
259436_at	At1g01500	0,80	0,86	0,90	0,81	1,05	0,79	0,80	0,75	expressed protein
259438_at	At1g01510	1,20	1,39	1,43	1,20	1,17	0,95	0,89	0,85	C-terminal binding protein (ANGUSTIFOLIA)
259432_at	At1g01520	0,94	1,09	1,09	0,85	0,97	0,90	0,94	1,13	myb family transcription factor
259427_at	At1g01530	0,96	0,94	1,07	1,05	0,97	0,97	0,98	1,06	MADS-box protein (AGL28)
259437_at	At1g01540	1,07	0,97	1,07	0,79	1,02	1,03	1,04	1,08	protein kinase family protein
259440_at	At1g01550	0,90	1,05	1,47	0,99	0,94	1,08	1,24	1,65	expressed protein
259428_at	At1g01560	0,50	0,58	1,24	1,03	1,10	1,23	1,47	1,78	mitogen-activated protein kinase, putative / MAPK, putative (MPK11)
259433_at	At1g01570	0,79	0,70	1,17	1,13	1,13	1,44	1,67	1,69	fringe-related protein
259429_at	At1g01600	1,04	1,22	0,90	1,15	1,40	0,99	1,08	0,85	cytochrome P450, putative
259430_at	At1g01610	1,34	1,10	1,16	1,28	0,95	1,02	0,90	1,00	phospholipid/glycerol acyltransferase family protein
259431_at	At1g01620	1,30	1,31	1,43	1,20	1,34	0,89	0,95	0,88	plasma membrane intrinsic protein 1C (PIP1C) / aquaporin PIP1.3
261563_at	At1g01630	1,31	1,05	1,01	1,68	1,37	0,95	0,82	0,90	SEC14 cytosolic factor, putative / phosphoglyceride transfer protein
261586_at	At1g01640	1,41	1,36	1,26	1,56	1,30	1,09	1,12	1,32	speckle-type POZ protein-related
257484_at	At1g01650	0,48	0,66	0,67	0,91	0,98	0,92	1,00	1,00	protease-associated (PA) domain-containing protein
261587_at	At1g01660	1,03	1,01	1,05	1,15	1,11	0,96	1,10	0,99	U-box domain-containing protein
261588_at	At1g01670	1,01	0,96	1,05	1,11	0,98	0,91	0,92	0,81	U-box domain-containing protein
261589_at	At1g01690	0,97	1,04	0,94	0,98	0,96	1,01	0,90	1,22	---
261590_at	At1g01700	1,01	1,06	1,02	0,93	1,04	0,91	1,01	1,09	expressed protein
261560_at	At1g01710	0,76	0,72	0,75	0,90	0,93	0,89	0,96	0,89	acyl-CoA thioesterase family protein
261564_at	At1g01720	0,95	1,77	1,05	0,55	0,34	1,24	1,40	1,43	no apical meristem (NAM) family protein
261535_at	At1g01725	1,08	1,06	1,00	1,07	1,19	1,14	0,92	0,98	expressed protein
261561_at	At1g01730	1,15	1,02	1,30	1,38	1,79	1,05	0,82	0,92	expressed protein
261591_at	At1g01740	0,99	0,92	0,88	0,76	0,87	1,01	0,99	1,01	protein kinase family protein
261562_at	At1g01750	1,40	0,91	0,84	1,64	1,74	1,01	1,12	0,95	actin-depolymerizing factor, putative
261592_at	At1g01760	0,98	1,09	1,19	0,82	1,20	1,22	1,14	0,96	adenosine-deaminase family / editase family
261558_at	At1g01770	1,34	1,60	2,09	0,82	0,65	1,07	1,17	1,38	expressed protein
261559_at	At1g01780	1,08	1,19	1,06	0,98	0,89	1,08	1,35	1,25	LIM domain-containing protein
261536_at	At1g01790	1,12	1,20	1,08	0,91	0,85	1,24	1,16	1,20	K+ efflux antiporter, putative (KEA1)
261537_at	At1g01800	1,21	1,05	1,05	1,05	1,04	0,96	0,99	0,97	short-chain dehydrogenase/reductase (SDR) family protein
261534_at	At1g01820	0,73	0,72	0,81	0,88	0,83	0,94	0,84	0,97	peroxisomal biogenesis factor 11 family protein / PEX11 family protein
261538_at	At1g01830	1,07	1,05	1,14	1,12	1,14	1,03	0,96	0,96	armadillo/beta-catenin repeat family protein
261539_at	At1g01840	0,98	1,13	0,92	0,95	1,05	0,92	0,87	1,18	expressed protein
261652_at	At1g01860	1,33	1,42	1,70	0,96	0,88	1,06	1,17	1,05	dimethyladenosine transferase (PFC1)
261653_at	At1g01900	1,55	1,34	1,19	1,45	1,15	0,73	0,68	0,70	subtilase family protein
261627_at	At1g01910	1,02	0,91	1,05	0,82	0,94	0,93	0,87	0,84	anion-transporting ATPase, putative
261654_at	At1g01920	0,91	0,77	0,64	0,87	1,14	0,70	0,73	0,73	SET domain-containing protein
261625_at	At1g01930	1,00	1,05	1,30	0,80	0,99	1,21	1,02	1,15	zinc finger protein-related
261655_at	At1g01940	1,58	1,21	1,08	1,24	1,27	1,08	0,98	1,02	peptidyl-prolyl cis-trans isomerase cyclophilin-type family protein
261656_at	At1g01950	0,70	0,54	0,74	0,91	1,02	0,98	0,92	1,25	armadillo/beta-catenin repeat family protein /kinesin motor family protein
261621_at	At1g01960	0,81	0,76	0,79	0,93	1,00	0,92	0,86	0,88	guanine nucleotide exchange family protein
261622_at	At1g01970	0,89	0,85	1,04	0,79	0,79	1,08	1,01	1,01	pentatricopeptide (PPR) repeat-containing protein

261623_at	At1g01980	0,93	0,93	1,07	0,99	1,15	0,93	0,97	0,92	FAD-binding domain-containing protein
261626_at	At1g01990	0,81	0,84	0,90	0,73	0,82	0,99	0,88	0,81	expressed protein
261624_at	At1g02000	0,76	0,62	0,57	1,14	1,23	0,84	0,76	0,91	NAD-dependent epimerase/dehydratase family protein
264169_at	At1g02020	1,34	1,23	1,46	0,82	0,90	1,05	1,03	0,87	nitroreductase family protein
264175_at	At1g02050	0,88	0,89	1,08	0,65	0,90	0,79	1,09	0,95	chalcone and stilbene synthase family protein
264167_at	At1g02060	0,99	0,85	1,02	0,62	0,68	0,86	0,91	0,98	pentatricopeptide (PPR) repeat-containing protein
264151_at	At1g02070	0,96	0,98	0,95	0,94	1,01	0,98	0,97	1,02	expressed protein
264168_at	At1g02080	0,99	0,85	0,80	0,84	0,79	0,95	1,02	0,94	transcriptional regulator-related
264150_at	At1g02090	1,34	1,15	1,17	1,07	1,08	1,12	0,89	1,02	COP9 signalosome complex subunit 7ii / CSN complex subunit 7ii (CSN7) (
264171_at	At1g02100	0,89	0,98	0,87	1,02	1,16	0,84	0,94	0,91	leucine carboxyl methyltransferase family protein
264176_at	At1g02110	0,92	0,96	0,94	0,69	0,64	0,99	0,90	0,81	proline-rich family protein
264172_at	At1g02120	0,77	0,81	0,84	0,85	0,95	0,89	1,05	1,13	GRAM domain-containing protein-related
264122_at	At1g02130	1,12	1,10	1,17	1,27	1,25	1,05	0,94	1,06	Ras-related protein (ARA-5) / small GTP-binding protein, putative
264152_at	At1g02140	1,34	1,11	1,03	1,24	1,20	1,06	0,94	0,96	mago nashi family protein
264177_at	At1g02150	1,04	0,95	1,26	0,70	0,78	0,97	0,87	0,81	pentatricopeptide (PPR) repeat-containing protein
264173_at	At1g02160	1,38	1,09	1,07	1,19	1,14	1,05	0,92	0,82	expressed protein
264178_at	At1g02170	0,79	0,75	0,87	0,98	1,11	0,95	0,93	0,88	latex-abundant family protein (AMC1) / caspase family protein
264179_at	At1g02180	0,98	1,18	1,49	1,36	1,27	0,84	0,94	1,00	ferredoxin-related
264180_at	At1g02190	0,96	1,15	0,96	0,93	1,19	1,25	1,01	0,83	CER1 protein, putative
264147_at	At1g02200	0,98	0,79	0,69	1,55	1,16	1,80	1,49	1,63	CER1 protein
264146_at	At1g02205	0,93	0,81	0,68	1,74	1,14	1,76	1,44	1,68	CER1 protein
264148_at	At1g02220	1,15	1,16	1,47	2,05	1,55	1,08	1,44	1,14	no apical meristem (NAM) family protein
264174_s_at	At1g02230	0,86	1,06	1,10	1,03	1,05	1,05	1,11	0,83	no apical meristem (NAM) family protein
264149_at	At1g02250	1,03	1,09	0,92	0,96	1,09	1,01	0,89	0,98	no apical meristem (NAM) family protein
264170_at	At1g02260	0,80	0,85	0,68	0,74	0,86	1,09	0,97	0,90	transmembrane protein, putative
264123_at	At1g02270	0,66	0,77	1,34	0,89	1,14	1,07	1,06	1,07	endonuclease/exonuclease/phosphatase family protein / calcium-binding El
264121_at	At1g02280	1,19	0,98	0,99	0,74	0,86	0,97	0,94	0,87	GTP-binding protein (TOC33)
259441_at	At1g02300	0,40	0,46	0,55	0,61	0,74	1,66	1,88	1,70	cathepsin B-like cysteine protease, putative
259416_at	At1g02305	1,06	1,08	1,03	1,06	1,03	1,00	1,04	1,03	cathepsin B-like cysteine protease, putative
259442_at	At1g02310	0,70	1,13	0,93	0,86	0,76	1,28	1,62	1,12	glycosyl hydrolase family protein 5 / cellulase family protein / (1-4)-beta-ma
259413_at	At1g02320	0,99	1,05	1,07	0,96	1,03	0,99	1,05	0,94	hypothetical protein
259415_at	At1g02330	1,48	1,34	1,54	0,83	1,33	1,22	1,27	1,62	expressed protein
259417_at	At1g02340	1,36	1,21	1,23	0,64	0,78	1,11	1,34	1,23	long hypocotyl in far-red 1 (HFR1) / reduced phytochrome signaling (REP1)
259443_at	At1g02360	1,06	1,21	1,45	1,53	1,42	1,35	1,12	1,09	chitinase, putative
259444_at	At1g02370	1,09	0,84	0,81	0,88	0,81	0,94	0,80	0,86	pentatricopeptide (PPR) repeat-containing protein
259418_at	At1g02390	0,42	1,10	0,96	0,50	0,55	1,85	1,17	0,99	phospholipid/glycerol acyltransferase family protein
259445_at	At1g02400	1,02	1,78	1,29	1,17	1,27	1,28	1,63	1,06	gibberellin 2-oxidase, putative / GA2-oxidase, putative
259446_at	At1g02410	1,08	0,99	0,98	0,94	1,03	0,99	1,11	1,14	cytochrome c oxidase assembly protein CtaG / Cox11 family
259414_at	At1g02420	0,92	0,98	0,90	0,95	0,82	0,91	0,99	1,04	pentatricopeptide (PPR) repeat-containing protein
259447_s_at	At1g02430	0,98	1,09	1,10	1,25	1,04	1,03	1,09	1,06	ADP-ribosylation factor, putative
260904_at	At1g02450	0,86	1,02	1,06	0,98	1,02	1,14	1,01	1,11	NPR1/NIM1-interacting protein 1 (NIMIN-1)
260903_at	At1g02460	1,04	0,97	1,07	1,14	1,40	1,10	1,04	1,19	glycoside hydrolase family 28 protein / polygalacturonase (pectinase) family
260933_at	At1g02470	1,04	1,22	1,32	0,99	0,86	1,02	0,84	0,97	expressed protein
260916_at	At1g02475	0,90	0,78	1,01	0,89	0,81	0,94	0,89	0,87	expressed protein
260911_at	At1g02490	0,99	0,97	0,99	1,09	1,16	1,06	0,96	0,87	hypothetical protein
260913_at	At1g02500	1,12	1,02	0,97	0,88	0,85	0,87	0,84	0,91	S-adenosylmethionine synthetase 1 (SAM1)

260932_s_at	At1g02530	1,30	1,25	1,12	0,81	0,79	1,20	1,34	1,29 multidrug resistance P-glycoprotein, putative
260906_at	At1g02540	0,95	1,17	0,96	0,89	1,30	1,09	0,96	1,10 hypothetical protein
260931_at	At1g02550	1,05	1,02	1,05	1,02	1,11	0,99	1,16	0,92 invertase/pectin methylesterase inhibitor family protein
260912_at	At1g02560	0,84	0,80	0,81	0,92	1,03	1,03	1,00	1,07 ATP-dependent Clp protease proteolytic subunit (ClpP1)
260907_at	At1g02570	0,90	0,97	0,92	0,99	1,05	1,06	0,96	0,71 expressed protein
260908_at	At1g02580	1,07	1,05	1,15	1,06	1,08	1,14	0,97	0,96 maternal embryogenesis control protein / MEDEA (MEA)
260930_at	At1g02620	0,96	1,21	1,02	0,91	1,20	1,13	0,95	0,86 GTP-binding protein (SAR1A)
260914_at	At1g02640	0,86	1,03	1,21	0,81	0,88	0,84	0,95	0,95 glycosyl hydrolase family 3 protein
260915_at	At1g02660	0,91	1,04	1,61	0,81	0,92	1,16	1,36	1,35 lipase class 3 family protein
260909_at	At1g02670	0,84	0,78	0,91	0,97	0,93	0,92	1,20	1,60 DNA repair protein, putative
260929_at	At1g02680	1,29	1,24	1,67	0,96	1,04	1,15	1,14	1,17 transcription initiation factor IID (TFIID) 18 kDa subunit (TAFII-18) family pro
260910_at	At1g02690	1,26	1,20	1,01	1,14	1,12	0,90	0,90	0,83 importin alpha-2 subunit, putative
260917_at	At1g02700	0,66	0,82	0,82	0,70	0,95	1,07	0,91	0,84 expressed protein
260905_at	At1g02710	1,02	1,00	0,96	0,90	1,14	1,00	0,90	1,15 glycine-rich protein
260928_at	At1g02720	0,82	0,91	0,81	1,04	0,98	0,82	0,89	0,93 glycosyl transferase family 8 protein
262109_at	At1g02730	1,40	1,21	1,14	1,20	1,41	0,73	0,75	0,73 cellulose synthase family protein
262108_at	At1g02740	0,94	0,80	0,99	0,96	0,96	1,14	1,02	0,90 MRG family protein
262107_at	At1g02750	1,15	0,93	1,33	1,14	1,35	1,03	1,11	1,24 drought-responsive family protein
262117_at	At1g02780	1,40	1,18	1,12	1,33	1,33	1,02	1,01	1,05 60S ribosomal protein L19 (RPL19A)
262122_at	At1g02790	0,99	1,15	1,04	1,03	1,02	1,05	0,98	0,98 exopolygalacturonase / galacturan 1,4-alpha-galacturonidase (PGA3) / pec
262121_at	At1g02800	0,98	0,99	1,03	1,11	1,08	1,03	1,00	1,05 endo-1,4-beta-glucanase / cellulase (CEL2)
262105_at	At1g02810	0,78	0,95	0,89	1,56	1,23	0,81	1,03	0,87 pectinesterase family protein
262115_at	At1g02813	1,01	0,99	1,03	0,94	1,04	1,09	0,95	1,12 expressed protein
262116_at	At1g02816	0,76	0,69	0,74	1,16	1,12	0,80	0,83	0,77 expressed protein
262113_at	At1g02820	1,21	1,12	1,41	3,07	3,06	1,52	1,53	1,68 late embryogenesis abundant 3 family protein / LEA3 family protein
262132_at	At1g02830	0,97	1,07	1,16	0,98	1,07	1,00	1,02	0,95 60S ribosomal protein L22 (RPL22A)
262110_at	At1g02840	1,15	1,10	1,18	1,07	1,15	0,98	0,98	1,17 pre-mRNA splicing factor SF2 (SF2) / SR1 protein
262118_at	At1g02850	0,37	0,30	0,37	0,61	0,65	0,80	0,85	0,92 glycosyl hydrolase family 1 protein
262114_at	At1g02860	0,87	0,88	1,19	1,11	0,69	1,04	1,29	1,38 SPX (SYG1/Pho81/XPR1) domain-containing protein / zinc finger (C3HC4-t
262112_at	At1g02870	1,78	1,43	1,44	1,28	1,13	0,94	0,89	0,92 expressed protein
262130_at	At1g02890	0,90	0,79	0,86	0,80	0,75	1,07	1,09	1,27 AAA-type ATPase family protein
262131_at	At1g02900	1,27	1,01	0,97	1,92	1,36	0,89	1,01	0,82 rapid alkalinization factor (RALF) family protein
262104_at	At1g02910	0,87	0,80	0,74	0,76	0,65	1,04	1,08	0,89 tetratricopeptide repeat (TPR)-containing protein
262119_s_at	At1g02930	1,18	1,21	1,09	0,69	0,66	1,38	0,93	0,91 glutathione S-transferase, putative
262103_at	At1g02940	1,00	0,92	1,03	0,96	0,94	1,00	0,96	0,96 glutathione S-transferase, putative
262120_at	At1g02950	0,97	0,97	0,62	1,32	1,37	0,81	0,73	0,73 glutathione S-transferase, putative
262111_at	At1g02960	0,97	0,85	0,79	0,81	0,82	0,98	1,12	1,25 expressed protein
262106_at	At1g02970	1,16	1,36	1,06	1,33	1,15	1,08	1,07	0,88 protein kinase, putative
262102_at	At1g02980	1,08	1,06	1,00	0,85	1,06	0,97	0,98	1,09 cullin family protein
263170_at	At1g03000	0,97	1,05	1,12	0,87	0,93	0,90	0,95	0,86 AAA-type ATPase family protein
263169_at	At1g03010	0,98	1,02	1,06	0,95	1,08	0,93	0,87	0,95 phototropic-responsive NPH3 family protein
263168_at	At1g03020	1,30	0,84	0,95	0,77	0,91	1,17	1,24	1,09 glutaredoxin family protein
263167_at	At1g03030	0,79	0,78	0,66	1,06	0,93	0,82	1,00	0,92 phosphoribulokinase/uridine kinase family protein
263117_at	At1g03040	1,25	1,32	1,29	1,00	1,06	0,99	1,14	0,93 basic helix-loop-helix (bHLH) family protein
263166_at	At1g03050	0,94	0,92	1,10	1,00	1,01	1,06	1,04	0,89 epsin N-terminal homology (ENTH) domain-containing protein / clathrin ass
263115_at	At1g03055	1,04	1,11	1,27	0,93	0,96	1,19	1,35	1,47 expressed protein

263165_at	At1g03060	0,79	0,75	0,78	0,71	0,66	0,88	0,96	1,06	WD-40 repeat family protein / beige-related
263164_at	At1g03070	1,13	1,28	1,03	1,19	1,10	1,20	1,06	1,07	expressed protein
263112_at	At1g03080	1,12	0,87	1,09	1,35	1,44	1,09	1,31	1,34	kinase interacting family protein
263118_at	At1g03090	1,15	1,79	2,10	0,88	0,87	1,04	1,22	1,44	methylcrotonyl-CoA carboxylase alpha chain, mitochondrial / 3-methylcroto
263143_at	At1g03100	0,96	0,96	1,15	0,98	0,97	1,14	1,01	1,14	pentatricopeptide (PPR) repeat-containing protein
263119_at	At1g03110	1,25	1,03	0,88	1,08	1,03	0,95	0,87	1,01	transducin family protein / WD-40 repeat family protein
263114_at	At1g03130	0,94	0,93	1,20	0,78	0,89	0,85	0,71	0,73	photosystem I reaction center subunit II, chloroplast, putative / photosysten
263116_s_at	At1g03140	1,12	1,11	1,44	0,91	1,05	1,04	0,93	1,02	splicing factor Prp18 family protein
263113_at	At1g03150	1,54	1,35	1,52	1,19	1,18	1,02	1,03	1,05	GCN5-related N-acetyltransferase (GNAT) family protein
263163_at	At1g03160	0,64	0,69	0,72	0,81	0,67	0,97	0,92	0,90	GTP-binding protein-related
264363_at	At1g03170	0,87	1,06	0,82	0,97	1,09	1,04	0,91	1,01	expressed protein
264358_at	At1g03180	1,02	1,14	0,92	1,08	1,08	1,04	0,99	0,89	hypothetical protein
264356_at	At1g03190	0,86	0,73	1,00	0,91	0,87	0,95	0,90	0,80	DNA repair protein / transcription factor protein (UVH6)
264355_at	At1g03210	0,79	0,87	0,80	1,13	0,99	0,70	0,83	0,95	phenazine biosynthesis PhzC/PhzF family protein
264365_s_at	At1g03220	0,80	0,75	0,70	0,86	0,87	0,93	0,81	0,82	extracellular dermal glycoprotein, putative / EDGP, putative
264354_s_at	At1g03240	0,99	0,86	1,00	1,01	0,88	0,97	1,22	1,19	expressed protein
264366_at	At1g03250	1,62	1,21	1,52	1,10	1,01	1,26	1,16	1,16	expressed protein
264353_at	At1g03260	0,74	0,69	0,74	0,82	0,96	0,96	0,82	0,78	expressed protein
264352_at	At1g03270	0,95	0,84	0,90	0,92	1,08	1,02	0,84	0,99	expressed protein
264368_at	At1g03280	1,14	1,03	1,08	0,92	0,94	1,15	1,17	1,20	transcription initiation factor IIE (TFIIE) alpha subunit family protein / genera
264362_at	At1g03290	0,84	0,83	1,15	0,82	0,89	0,98	1,08	1,43	expressed protein
264361_at	At1g03300	0,99	1,02	1,03	0,92	0,94	0,88	0,93	1,03	agenet domain-containing protein
264360_at	At1g03310	0,72	0,59	0,57	0,77	0,71	0,93	0,84	0,77	isoamylase, putative / starch debranching enzyme, putative
264359_at	At1g03320	0,94	1,09	0,89	0,83	0,93	0,97	0,93	1,18	hypothetical protein
264364_at	At1g03330	1,60	1,44	1,25	1,44	1,81	1,02	1,02	1,09	small nuclear ribonucleoprotein D, putative / snRNP core SM-like protein, p
264367_at	At1g03350	0,83	0,76	0,76	0,88	1,32	0,97	0,84	0,85	BSD domain-containing protein
264357_at	At1g03360	1,29	1,26	1,06	0,95	0,97	0,99	0,87	1,01	exonuclease family protein
264351_at	At1g03370	0,71	0,74	1,05	0,90	0,84	0,98	1,10	1,35	C2 domain-containing protein / GRAM domain-containing protein
264828_at	At1g03380	0,99	1,47	1,81	0,69	0,77	1,21	1,33	1,15	expressed protein
264827_at	At1g03390	1,03	0,98	0,90	0,93	0,97	0,99	1,08	1,06	transferase family protein
264843_at	At1g03400	1,02	1,13	1,34	0,90	0,94	1,31	1,11	1,38	2-oxoglutarate-dependent dioxygenase, putative
264826_at	At1g03410	0,82	0,76	0,71	0,81	0,92	0,87	0,79	0,89	2-oxoglutarate-dependent dioxygenase, putative
264824_at	At1g03420	0,92	1,04	0,67	0,76	0,70	0,74	0,76	0,67	expressed protein
264838_at	At1g03430	1,24	1,21	1,04	0,80	0,79	1,03	1,12	1,17	two-component phosphorelay mediator, putative
264840_at	At1g03440	0,75	0,83	0,98	0,79	1,01	0,76	0,89	0,94	leucine-rich repeat family protein
264823_at	At1g03445	0,90	1,12	1,00	1,01	0,97	1,05	1,04	0,94	kelch repeat-containing protein / serine/threonine phosphoesterase family p
264822_at	At1g03457	0,99	0,70	1,14	0,78	0,91	0,89	1,14	1,41	RNA-binding protein, putative
264821_at	At1g03470	0,86	1,06	1,06	0,94	0,87	1,13	1,08	1,03	kinase interacting family protein
264820_at	At1g03475	0,93	0,83	0,90	0,87	0,85	1,00	1,00	0,94	coproporphyrinogen III oxidase, putative / coproporphyrinogenase, putative
264829_at	At1g03490	1,10	0,98	1,19	1,11	1,09	1,03	1,05	1,14	no apical meristem (NAM) family protein
265091_s_at	At1g03495	0,96	1,06	1,02	1,14	1,11	0,93	1,01	1,09	pseudogene, transferase family
264819_at	At1g03510	0,78	0,84	0,98	1,01	0,86	0,79	0,87	1,01	pentatricopeptide (PPR) repeat-containing protein
264844_at	At1g03520	1,00	0,86	1,16	0,82	0,75	0,92	1,41	2,00	glycosyltransferase family 14 protein / core-2/I-branching enzyme family pro
264818_at	At1g03530	1,15	1,13	0,80	0,90	1,02	0,91	0,90	1,14	expressed protein
264817_at	At1g03540	0,92	1,07	1,01	1,02	0,88	0,98	1,05	0,81	pentatricopeptide (PPR) repeat-containing protein
264835_at	At1g03550	1,10	1,18	1,08	1,04	0,91	1,05	1,20	1,22	secretory carrier membrane protein (SCAMP) family protein

264816_at	At1g03560	0,97	0,80	0,82	0,90	0,85	1,11	0,76	1,09	pentatricopeptide (PPR) repeat-containing protein
264833_at	At1g03590	1,33	1,33	1,00	1,18	1,06	1,06	1,04	1,01	protein phosphatase 2C family protein / PP2C family protein
264837_at	At1g03600	1,22	1,18	1,34	1,07	1,13	1,03	1,01	0,97	photosystem II family protein
264836_at	At1g03610	1,09	1,57	1,40	0,94	0,69	0,78	0,96	1,06	expressed protein
264815_at	At1g03620	1,26	1,31	1,59	1,24	1,15	1,09	1,70	1,87	phagocytosis and cell motility protein ELMO1-related
264839_at	At1g03630	1,32	1,25	1,41	0,73	0,73	1,44	1,28	1,23	protochlorophyllide reductase C, chloroplast / PCR C / NADPH-protochloro
264832_at	At1g03660	1,26	1,20	1,29	1,22	1,18	1,08	0,94	1,00	expressed protein
264845_at	At1g03675	0,85	0,83	0,91	0,95	0,90	1,06	0,91	0,90	thioredoxin M-type 1, chloroplast (TRX-M1)
264831_at	At1g03687	1,14	1,13	1,24	0,91	0,87	0,97	1,06	0,71	DTW domain-containing protein
264842_at	At1g03700	0,97	0,97	1,02	0,93	0,99	1,03	1,16	0,97	integral membrane family protein
264830_at	At1g03710	1,31	1,37	1,06	0,88	1,08	0,89	1,04	0,88	expressed protein
264825_at	At1g03720	1,03	0,91	0,77	1,04	1,21	1,15	1,00	0,87	cathepsin-related
264834_at	At1g03730	0,92	0,98	0,80	1,14	1,00	0,98	1,06	1,06	expressed protein
264841_at	At1g03740	0,76	0,84	1,09	0,85	0,90	0,96	1,07	1,62	protein kinase family protein
265088_at	At1g03750	0,97	1,07	1,03	0,88	1,02	1,16	1,03	0,88	helicase, putative
265087_at	At1g03760	1,34	1,08	1,33	0,77	0,96	1,03	0,97	0,86	prefoldin subunit family protein
265086_at	At1g03770	1,37	1,37	1,40	1,44	1,62	0,94	1,02	1,18	zinc finger (C3HC4-type RING finger) family protein
265085_at	At1g03780	1,06	1,04	1,01	1,27	1,18	0,88	0,95	1,08	targeting protein-related
265084_at	At1g03790	0,75	1,16	1,06	0,97	1,07	0,98	1,07	0,86	zinc finger (CCCH-type) family protein
265083_at	At1g03820	1,49	1,56	1,28	2,47	1,51	0,85	0,89	0,84	expressed protein
265082_at	At1g03830	0,98	1,14	1,19	1,05	0,91	1,01	1,05	1,17	guanylate-binding family protein
265081_at	At1g03840	1,02	1,07	1,04	0,95	0,92	1,11	0,97	0,91	zinc finger (C2H2 type) family protein
265067_at	At1g03850	0,88	1,11	1,13	1,03	1,13	1,43	1,58	1,75	glutaredoxin family protein
265037_at	At1g03860	1,49	1,35	1,23	0,99	1,06	0,96	0,91	1,01	prohibitin, putative
265066_at	At1g03870	1,17	0,92	0,85	1,10	1,13	0,77	0,61	0,63	fasciclin-like arabinogalactan-protein (FLA9)
265095_at	At1g03880	0,98	0,94	0,17	0,95	0,76	1,05	1,00	1,21	12S seed storage protein (CRB)
265094_at	At1g03890	0,88	1,05	1,00	0,93	0,93	1,03	0,95	1,06	cupin family protein
265043_at	At1g03900	0,98	0,97	1,03	1,09	1,05	1,00	0,89	0,92	expressed protein
265093_at	At1g03905	1,01	1,00	1,17	1,29	1,12	0,93	1,23	0,90	ABC transporter family protein
265092_at	At1g03910	1,15	1,42	1,86	1,05	1,13	1,07	1,15	1,31	expressed protein
265038_at	At1g03920	1,09	1,00	1,09	0,99	0,82	1,01	0,95	1,29	protein kinase, putative
265045_s_at	At1g03930	1,07	0,89	0,94	0,93	1,02	1,21	1,08	1,19	protein kinase (ADK1)
265044_at	At1g03950	0,88	0,85	0,90	1,13	1,00	1,20	1,20	1,32	SNF7 family protein
265090_at	At1g03960	1,13	1,15	1,18	1,01	0,93	1,05	0,89	0,99	calcium-binding EF hand family protein
265040_at	At1g03970	0,70	0,78	0,73	0,88	0,78	1,07	1,22	1,14	G-box binding factor 4 (GBF4)
265065_at	At1g03980	1,06	1,25	1,13	1,15	1,04	0,99	1,06	0,94	phytochelatin synthase 2 (PCS2)
265099_at	At1g03990	1,04	1,14	1,04	1,41	1,06	1,09	1,27	0,95	alcohol oxidase-related
265039_at	At1g04000	0,94	0,99	1,05	0,76	1,11	1,16	0,97	1,06	expressed protein
265098_at	At1g04010	1,07	1,01	0,95	0,96	1,22	0,97	0,89	0,92	lecithin:cholesterol acyltransferase family protein / LACT family protein
265097_at	At1g04020	1,19	1,13	1,21	1,22	1,21	1,13	0,96	1,06	zinc finger (C3HC4-type RING finger) family protein / BRCT domain-contai
265096_at	At1g04030	1,07	1,09	1,06	1,19	1,07	0,97	1,01	1,06	expressed protein
265042_at	At1g04040	1,45	1,12	1,09	1,73	1,57	0,92	0,72	0,83	acid phosphatase class B family protein
257404_at	At1g04050	0,87	0,84	0,91	1,05	1,20	0,90	0,91	0,97	SET domain-containing protein / suppressor of variegation related 1 (SUVR
265089_at	At1g04060	0,87	0,99	0,99	0,98	1,05	0,80	0,93	1,02	SET domain-containing protein / suppressor of variegation related 1 (SUVR
265041_at	At1g04070	1,08	0,92	1,04	1,27	1,33	1,00	0,98	1,04	expressed protein
265036_at	At1g04080	1,36	1,16	1,32	0,98	0,95	1,09	1,13	1,20	hydroxyproline-rich glycoprotein family protein

264320_at	At1g04090	0,95	1,01	1,03	0,92	1,22	0,95	1,04	1,10	expressed protein
264328_at	At1g04100	1,06	1,14	1,06	0,96	1,00	0,93	1,10	1,15	auxin-responsive protein / indoleacetic acid-induced protein 10 (IAA10)
264319_at	At1g04110	1,12	1,00	1,06	1,38	1,50	0,88	0,69	0,84	subtilase family protein
264330_at	At1g04120	0,87	1,00	0,88	1,31	1,06	1,03	1,05	1,06	ABC transporter family protein
264331_at	At1g04130	1,17	1,06	1,35	1,59	1,87	1,00	0,88	0,91	tetratricopeptide repeat (TPR)-containing protein
264329_at	At1g04140	0,89	0,96	0,87	0,90	0,82	0,88	0,89	0,88	transducin family protein / WD-40 repeat family protein
264325_at	At1g04150	1,06	0,94	1,04	1,03	0,99	0,95	1,04	1,13	C2 domain-containing protein
264324_at	At1g04160	0,97	0,84	0,90	1,15	1,21	0,91	0,94	0,83	myosin family protein
264327_at	At1g04170	1,33	1,24	1,12	0,91	1,00	0,99	0,89	0,84	eukaryotic translation initiation factor 2 subunit 3, putative / eIF2S3, putative
264323_at	At1g04180	1,15	1,09	1,02	1,05	1,12	1,02	0,93	1,15	flavin-containing monooxygenase family protein / FMO family protein
264322_at	At1g04190	1,35	1,05	1,07	1,26	1,24	1,15	1,04	1,08	tetratricopeptide repeat (TPR)-containing protein
264321_at	At1g04200	0,85	0,78	0,94	1,01	0,92	0,96	0,99	1,19	expressed protein
264318_at	At1g04220	1,08	1,38	1,04	1,10	0,90	1,06	1,43	1,03	beta-ketoacyl-CoA synthase, putative
264326_at	At1g04230	0,93	0,77	1,03	0,93	1,41	0,91	0,89	0,98	expressed protein
263656_at	At1g04240	1,41	1,58	2,11	0,86	0,76	1,24	1,71	1,56	auxin-responsive protein / indoleacetic acid-induced protein 3 (IAA3)
263664_at	At1g04250	1,70	1,45	1,21	1,41	1,03	1,43	1,21	1,09	auxin-responsive protein / indoleacetic acid-induced protein 17 (IAA17)
263660_at	At1g04260	0,95	0,89	0,80	1,33	1,01	0,92	0,78	0,96	prenylated rab acceptor (PRA1) family protein
263667_at	At1g04270	1,58	1,28	1,21	1,29	1,22	1,03	0,97	0,97	40S ribosomal protein S15 (RPS15A)
263661_at	At1g04290	1,15	1,17	1,01	1,74	1,68	0,98	0,90	0,90	thioesterase family protein
263654_at	At1g04300	1,00	1,18	1,33	0,93	1,04	1,09	1,20	1,46	meprin and TRAF homology domain-containing protein / MATH domain-cor
263653_at	At1g04310	0,86	1,10	1,25	0,78	1,19	0,88	1,09	1,03	ethylene receptor-related
263652_at	At1g04330	0,93	0,99	0,98	0,90	0,96	1,06	0,91	0,97	expressed protein
263651_at	At1g04340	1,23	1,04	0,97	1,15	1,20	0,97	0,90	0,93	lesion inducing protein-related
263668_at	At1g04350	0,61	0,58	0,63	1,10	1,16	0,97	0,94	0,98	2-oxoglutarate-dependent dioxygenase, putative
263650_at	At1g04360	0,96	1,10	0,89	0,92	1,03	0,97	1,03	0,95	zinc finger (C3HC4-type RING finger) family protein
263649_at	At1g04380	1,04	0,96	1,01	1,07	1,01	1,03	0,91	1,02	2-oxoglutarate-dependent dioxygenase, putative
263648_at	At1g04390	0,93	0,84	1,06	0,95	0,91	1,01	1,09	1,11	expressed protein
263669_at	At1g04400	1,12	1,22	1,49	0,87	0,93	0,95	1,05	1,19	cryptochrome 2 apoprotein (CRY2) / blue light photoreceptor (PHH1)
263663_at	At1g04410	0,83	0,83	0,87	1,07	1,14	0,89	0,87	0,90	malate dehydrogenase, cytosolic, putative
263678_at	At1g04420	0,83	0,75	0,74	0,79	0,79	1,01	1,02	1,04	aldo/keto reductase family protein
263662_at	At1g04430	1,03	0,85	0,82	1,07	0,97	0,95	0,95	1,03	dehydration-responsive protein-related
263657_at	At1g04440	1,12	1,18	1,65	1,37	1,36	1,07	1,15	1,49	casein kinase, putative
263659_at	At1g04470	1,09	1,05	1,01	1,03	1,04	1,08	0,95	0,95	expressed protein
263665_at	At1g04480	1,53	1,36	1,22	1,03	0,94	1,05	1,04	1,06	60S ribosomal protein L23 (RPL23A)
263658_at	At1g04490	0,93	1,04	1,08	0,96	0,95	1,19	1,04	0,96	expressed protein
263655_at	At1g04500	0,96	0,97	0,99	1,03	1,07	1,02	0,96	1,01	zinc finger CONSTANS-related
263666_at	At1g04510	1,01	0,94	0,94	0,94	0,99	1,03	0,96	0,83	transducin family protein / WD-40 repeat family protein
263677_at	At1g04520	1,16	1,05	0,93	1,25	0,97	0,96	0,91	0,98	33 kDa secretory protein-related
264609_at	At1g04530	1,28	1,21	1,47	1,35	1,15	1,42	1,21	1,14	expressed protein
264601_at	At1g04540	0,93	0,99	0,81	0,96	1,30	0,98	0,82	0,85	C2 domain-containing protein
264605_at	At1g04550	1,47	1,81	1,59	1,00	0,84	1,33	1,16	1,39	auxin-responsive protein / indoleacetic acid-induced protein 12 (IAA12)
264612_at	At1g04560	0,23	0,24	0,15	0,89	0,88	0,99	0,91	0,82	AWPM-19-like membrane family protein
264614_s_at	At1g04580	0,92	0,97	1,18	0,89	1,18	0,91	0,96	1,32	aldehyde oxidase, putative
264615_at	At1g04590	1,19	1,50	1,40	1,14	1,34	1,14	1,18	1,29	expressed protein
264599_at	At1g04600	0,84	1,03	1,03	1,07	1,09	0,99	0,96	0,76	myosin, putative
264598_at	At1g04610	1,17	1,16	1,11	1,13	1,07	0,99	0,88	0,98	flavin-containing monooxygenase / FMO (YUCCA3)

264597_at	At1g04620	0,68	0,63	0,72	0,68	0,64	1,51	1,19	1,20 coenzyme F420 hydrogenase family / dehydrogenase, beta subunit family
264596_at	At1g04630	0,97	0,97	0,89	0,93	1,05	1,10	1,17	1,10 pseudogene, similar to Unknown protein
264613_at	At1g04640	0,79	0,74	0,93	0,87	1,02	0,93	0,93	0,92 biotin/lipoate A/B protein ligase family protein
264610_at	At1g04645	1,03	0,99	1,09	0,91	0,98	1,05	0,94	0,94 self-incompatibility protein-related
264604_at	At1g04650	1,07	1,10	1,21	0,93	0,84	0,98	1,06	0,92 hypothetical protein
264606_at	At1g04660	0,87	0,96	0,89	1,08	1,02	0,87	1,07	0,99 glycine-rich protein
264603_at	At1g04670	0,91	1,06	0,97	0,98	1,10	0,95	0,98	1,29 expressed protein
264611_at	At1g04680	0,80	0,96	1,13	1,27	1,42	0,69	0,62	0,63 pectate lyase family protein
264607_at	At1g04690	0,79	0,83	0,89	1,18	0,99	0,85	0,88	0,94 potassium channel protein, putative
264602_at	At1g04700	1,05	0,87	1,01	1,06	0,99	1,01	0,82	1,05 protein kinase family protein
264608_at	At1g04710	0,96	0,90	0,83	0,92	0,77	1,08	1,06	1,11 acetyl-CoA C-acyltransferase, putative / 3-ketoacyl-CoA thiolase, putative
264600_at	At1g04730	1,07	1,15	1,28	1,01	1,25	1,14	0,97	1,07 AAA-type ATPase family protein
264595_at	At1g04750	1,02	0,95	1,01	1,18	1,33	0,95	0,84	0,86 synaptobrevin family protein
261178_at	At1g04760	1,02	1,00	1,04	1,19	0,98	0,84	0,92	0,80 synaptobrevin family protein
261177_at	At1g04770	1,22	1,04	0,90	0,59	0,73	0,93	1,20	1,21 male sterility MS5 family protein
261176_at	At1g04780	0,71	0,69	0,71	1,14	1,35	1,04	0,95	1,19 ankyrin repeat family protein
261127_at	At1g04790	1,10	1,55	2,09	0,85	0,96	1,05	0,94	1,08 zinc finger (C3HC4-type RING finger) family protein
261175_at	At1g04800	1,01	1,07	0,95	1,04	1,08	0,87	0,92	0,92 glycine-rich protein
261174_at	At1g04810	0,94	0,97	1,03	0,80	0,82	1,00	0,96	0,97 26S proteasome regulatory subunit, putative
261129_at	At1g04820	0,93	0,88	0,80	1,10	1,09	0,75	0,79	0,85 tubulin alpha-2/alpha-4 chain (TUA4)
261173_at	At1g04830	0,87	0,86	1,01	1,00	1,00	1,06	0,97	0,99 RabGAP/TBC domain-containing protein
261172_at	At1g04840	0,92	1,09	0,97	0,98	0,99	0,99	1,03	0,84 pentatricopeptide (PPR) repeat-containing protein
261153_at	At1g04850	0,92	0,80	0,82	1,19	1,10	1,23	1,02	1,11 ubiquitin-associated (UBA)/TS-N domain-containing protein
261128_at	At1g04860	1,06	0,93	1,20	0,80	0,78	1,15	0,97	0,99 ubiquitin-specific protease 2 (UBP2)
261130_at	At1g04870	1,37	1,36	1,34	0,94	0,87	1,02	1,09	1,10 protein arginine N-methyltransferase family protein
261171_at	At1g04880	0,98	1,13	1,12	0,94	1,05	1,10	0,93	0,92 high mobility group (HMG1/2) family protein / ARID/BRIGHT DNA-binding d
261124_at	At1g04900	0,90	0,85	0,72	1,21	0,97	0,98	0,89	0,75 expressed protein
261170_at	At1g04910	1,07	1,05	1,15	1,08	1,13	0,97	0,99	0,97 expressed protein
261169_at	At1g04920	0,77	0,87	0,94	1,02	0,96	0,95	0,87	1,05 sucrose-phosphate synthase, putative
261152_at	At1g04930	0,97	1,05	1,09	0,99	1,11	1,19	0,98	1,13 hydroxyproline-rich glycoprotein family protein
261168_at	At1g04940	1,11	1,20	1,09	0,86	1,08	0,95	0,96	0,99 tic20 family protein
261126_at	At1g04950	0,89	1,09	1,30	0,79	0,63	1,06	1,03	1,19 TATA box-binding protein-associated factor (TAF) family protein
261155_at	At1g04960	1,06	0,89	1,12	1,12	1,12	1,06	1,11	1,15 expressed protein
261154_at	At1g04970	0,89	0,83	0,83	1,17	1,27	0,84	0,83	0,91 lipid-binding serum glycoprotein family protein
261167_at	At1g04980	1,56	1,55	1,30	1,10	1,11	1,02	1,08	1,06 thioredoxin family protein
261179_at	At1g04985	1,03	0,93	0,96	1,05	1,01	1,17	1,01	1,16 expressed protein
261125_at	At1g04990	1,15	0,94	1,49	1,07	1,39	1,06	0,98	1,27 zinc finger (CCCH-type) family protein
265214_at	At1g05000	0,87	0,91	0,92	0,98	0,94	1,07	1,09	1,01 tyrosine specific protein phosphatase family protein
265194_at	At1g05010	1,01	0,93	1,37	1,14	1,00	0,89	0,81	0,79 1-aminocyclopropane-1-carboxylate oxidase / ACC oxidase / ethylene-form
265213_at	At1g05020	0,76	0,81	0,76	1,03	1,01	0,84	0,98	1,12 epsin N-terminal homology (ENTH) domain-containing protein / clathrin ass
265212_at	At1g05030	0,83	0,82	0,77	0,74	0,86	0,90	1,00	0,96 hexose transporter, putative
265215_at	At1g05040	1,09	1,10	0,96	1,02	0,94	1,01	1,02	0,87 hypothetical protein
265218_at	At1g05050	1,17	1,00	1,11	0,97	1,06	0,90	0,97	0,84 basic transcription factor 2, 44kD subunit-related
265192_at	At1g05060	1,22	1,47	1,13	0,89	0,85	1,07	0,93	0,99 expressed protein
265193_at	At1g05070	0,86	0,86	0,95	0,88	0,99	1,00	1,04	1,17 expressed protein
265217_s_at	At1g05090	1,09	1,10	1,13	1,30	1,59	0,99	0,87	1,00 dentin sialophosphoprotein-related

265216_at	At1g05100	0,77	0,91	0,86	0,98	0,97	1,20	0,96	1,04	protein kinase family protein
265191_at	At1g05120	1,10	1,11	1,35	1,18	1,23	1,08	1,06	1,12	SNF2 domain-containing protein / helicase domain-containing protein / RIN
264569_at	At1g05130	0,98	1,04	0,97	0,94	1,19	0,93	1,08	0,90	---
264584_at	At1g05140	0,85	0,67	0,83	0,60	0,61	0,84	0,95	0,77	membrane-associated zinc metalloprotease, putative
264568_at	At1g05150	1,13	1,11	1,09	1,00	1,08	0,94	0,86	0,80	calcium-binding EF hand family protein
264586_at	At1g05160	1,18	1,05	0,97	1,13	1,01	1,09	0,99	1,02	ent-kaurenoic acid hydroxylase (KAO1) / cytochrome P450 88A3, putative (
264583_at	At1g05170	1,08	1,08	1,02	0,89	0,92	1,05	1,13	0,99	galactosyltransferase family protein
264585_at	At1g05180	1,13	1,15	1,16	1,05	1,05	1,02	1,03	1,05	auxin-resistance protein AXR1 (AXR1)
264575_at	At1g05190	1,10	1,05	1,01	1,00	1,23	1,07	0,99	0,93	ribosomal protein L6 family protein
264587_at	At1g05200	0,96	1,05	1,10	0,64	0,58	1,17	1,31	1,17	glutamate receptor family protein (GLR3.4)
264579_at	At1g05205	1,15	1,11	0,95	1,31	1,47	0,88	0,91	0,98	expressed protein
264581_at	At1g05210	1,22	1,08	0,94	1,65	1,24	0,88	0,88	0,85	expressed protein
264582_at	At1g05230	1,25	1,21	1,40	0,76	0,69	1,18	1,04	1,05	homeobox-leucine zipper family protein / lipid-binding START domain-conta
264567_s_at	At1g05250	1,19	1,03	0,87	1,35	1,78	0,86	1,02	0,92	peroxidase, putative
264577_at	At1g05260	0,96	1,05	0,80	1,89	1,31	1,11	0,95	0,96	peroxidase 3 (PER3) (P3) / rare cold-inducible protein (RCI3A) (PRC)
264566_at	At1g05270	0,81	0,77	1,10	0,87	1,02	0,91	0,97	0,93	TraB family protein
264565_at	At1g05280	1,05	1,05	0,95	1,24	1,19	0,92	1,01	1,04	fringe-related protein
264564_at	At1g05290	1,00	0,99	1,09	1,10	1,18	1,01	1,09	0,96	hypothetical protein
264574_at	At1g05300	1,04	1,10	1,42	1,01	1,02	0,86	0,61	0,73	metal transporter, putative (ZIP5)
264573_at	At1g05310	0,90	0,97	1,11	0,98	1,31	0,90	0,87	1,01	pectinesterase family protein
264572_at	At1g05320	1,15	1,19	1,57	0,89	0,74	1,21	1,37	1,31	myosin-related
264571_at	At1g05330	0,98	0,91	0,93	0,98	1,03	0,91	0,95	0,98	expressed protein
264580_at	At1g05340	0,79	1,08	1,30	1,03	1,00	1,60	1,66	1,87	expressed protein
264570_at	At1g05350	0,99	0,86	0,80	0,97	1,18	0,80	0,94	0,86	thiF family protein
264576_at	At1g05360	1,21	0,96	1,04	0,93	0,85	1,24	1,21	1,07	expressed protein
264578_at	At1g05370	1,03	1,07	0,81	1,12	0,97	1,01	0,91	1,06	expressed protein
261383_at	At1g05380	0,80	0,84	1,08	0,91	1,01	0,98	0,88	0,86	PHD finger transcription factor, putative
261388_at	At1g05385	0,68	0,67	0,70	0,94	0,94	0,98	0,86	0,77	photosystem II 11 kDa protein-related
261380_at	At1g05400	1,03	1,11	1,10	0,84	1,15	1,00	1,18	0,91	hypothetical protein
261387_at	At1g05410	1,19	1,33	1,40	0,83	1,07	1,02	0,98	1,04	expressed protein
261386_at	At1g05430	1,02	0,78	0,99	0,89	1,01	0,98	0,87	0,80	expressed protein
261384_at	At1g05440	1,12	0,92	1,15	1,05	1,06	1,05	0,97	0,97	expressed protein
261385_at	At1g05450	1,09	1,03	1,03	0,97	1,15	0,89	1,03	0,96	protease inhibitor/seed storage/lipid transfer protein (LTP)-related
261381_at	At1g05460	1,01	1,25	1,13	0,81	0,91	0,90	1,16	1,24	RNA helicase SDE3 (SDE3)
261382_at	At1g05470	0,91	1,19	0,86	0,92	0,87	0,85	1,22	0,97	endonuclease/exonuclease/phosphatase family protein
263204_at	At1g05480	1,16	1,24	1,29	0,97	1,03	1,20	1,04	0,91	SNF2 domain-containing protein / helicase domain-containing protein
263203_at	At1g05490	1,01	1,05	1,01	0,95	1,15	1,05	1,05	0,87	C protein immunoglobulin-A-binding beta antigen-related
263205_at	At1g05500	1,21	1,09	1,03	1,05	0,92	1,07	0,90	0,99	C2 domain-containing protein
263175_at	At1g05510	0,16	0,36	0,64	0,54	0,66	1,09	1,14	1,10	expressed protein
263185_at	At1g05520	0,89	0,83	0,80	1,03	0,88	0,92	0,85	0,75	transport protein, putative
263176_at	At1g05530	0,99	0,96	0,96	0,99	1,00	0,98	1,02	1,01	UDP-glucoronosyl/UDP-glucosyl transferase family protein
263177_at	At1g05540	1,16	1,04	1,19	1,30	1,25	0,94	0,96	0,92	expressed protein
263178_at	At1g05550	1,00	1,06	1,17	1,09	0,92	1,01	1,02	1,03	expressed protein
263184_at	At1g05560	0,31	0,29	0,58	0,40	0,40	0,65	0,95	0,98	UDP-glucose transferase (UGT75B2)
263183_at	At1g05570	0,94	0,96	0,98	1,16	0,91	1,34	1,24	1,23	callose synthase 1 (CALS1) / 1,3-beta-glucan synthase 1
263182_at	At1g05575	0,96	1,31	0,83	0,93	0,87	1,15	1,47	0,82	expressed protein

263233_at	At1g05580	0,86	1,04	1,01	1,00	0,98	1,06	0,93	0,96	hypothetical protein
263199_at	At1g05590	0,77	1,00	1,09	1,20	1,09	0,96	0,91	0,92	glycosyl hydrolase family 20 protein
263200_at	At1g05600	1,09	1,25	1,12	0,83	0,86	0,89	1,01	0,98	pentatricopeptide (PPR) repeat-containing protein
263201_at	At1g05610	1,08	0,99	1,04	1,00	0,94	1,09	1,12	1,11	glucose-1-phosphate adenylyltransferase, putative / ADP-glucose pyrophos
263180_at	At1g05620	0,79	0,74	0,66	1,26	1,30	0,84	0,80	0,73	inosine-uridine preferring nucleoside hydrolase family protein
263202_at	At1g05630	0,86	0,81	1,05	0,89	0,99	0,87	0,92	1,14	endonuclease/exonuclease/phosphatase family protein
263229_s_at	At1g05650	1,03	0,93	0,98	1,17	1,18	1,09	1,02	0,99	polygalacturonase, putative / pectinase, putative
263230_at	At1g05670	1,43	1,30	0,98	0,77	0,96	1,31	1,56	1,08	UDP-glucoronosyl/UDP-glucosyl transferase family protein
263231_at	At1g05680	0,42	0,28	0,55	0,29	0,38	0,91	0,93	0,99	UDP-glucoronosyl/UDP-glucosyl transferase family protein
257451_at	At1g05690	1,04	1,25	1,33	0,82	0,96	1,10	1,04	0,82	TAZ zinc finger family protein / BTB/POZ domain-containing protein
263232_at	At1g05700	0,94	0,99	0,95	0,96	0,67	1,09	1,18	1,20	leucine-rich repeat protein kinase, putative
263179_at	At1g05710	0,98	0,88	0,91	1,61	1,46	1,23	1,11	1,30	ethylene-responsive protein, putative
263181_at	At1g05720	1,39	1,29	1,20	1,40	1,64	0,99	0,86	0,89	selenoprotein family protein
257452_at	At1g05740	1,04	1,07	1,14	0,83	1,07	1,08	0,98	0,95	hypothetical protein
261310_at	At1g05750	0,91	1,01	1,07	0,73	0,80	0,96	0,93	1,27	pentatricopeptide (PPR) repeat-containing protein
261256_at	At1g05760	1,39	1,25	0,98	1,24	1,23	0,82	1,01	0,92	jacalin lectin family protein (RTM1)
261311_at	At1g05770	1,07	0,98	0,97	0,96	1,27	0,84	0,88	0,71	jacalin lectin family protein
261312_at	At1g05790	0,75	0,77	1,05	0,75	0,81	1,01	0,95	1,17	lipase class 3 family protein
261278_at	At1g05800	1,04	1,01	1,19	1,06	1,00	0,92	1,01	1,07	lipase class 3 family protein
261254_at	At1g05805	1,14	1,03	1,13	0,93	0,81	0,89	0,89	1,06	basic helix-loop-helix (bHLH) family protein
261252_at	At1g05810	1,35	1,37	1,36	1,18	1,33	0,84	0,67	0,80	Ras-related protein (ARA-1) (ARA) / small GTP-binding protein, putative
261253_at	At1g05840	0,90	1,00	1,32	0,88	0,73	1,04	1,19	1,59	aspartyl protease family protein
261279_at	At1g05850	1,08	0,99	0,95	1,04	1,08	0,81	0,79	0,86	chitinase-like protein 1 (CTL1)
261280_at	At1g05860	1,20	1,17	1,07	0,99	1,14	0,98	1,24	0,95	expressed protein
261249_at	At1g05880	1,07	1,13	1,20	1,17	1,08	1,20	1,06	0,76	expressed protein
261250_at	At1g05890	0,92	1,14	1,57	0,84	0,85	1,12	1,15	1,32	zinc finger protein-related
261255_at	At1g05900	0,96	0,93	0,89	0,87	0,81	1,15	1,15	1,06	endonuclease-related
261257_at	At1g05910	0,86	0,77	0,90	0,96	1,05	0,93	0,89	0,95	cell division cycle protein 48-related / CDC48-related
261251_at	At1g05920	0,97	1,03	0,88	0,91	1,01	1,04	1,01	0,89	hypothetical protein
260927_at	At1g05940	0,70	0,66	0,67	0,86	0,82	0,92	0,83	0,86	amino acid permease family protein
260945_at	At1g05950	1,45	1,30	1,17	1,10	1,07	1,07	1,03	1,13	expressed protein
260961_at	At1g05960	1,01	1,01	0,98	0,83	0,96	0,88	0,85	0,81	expressed protein
260960_at	At1g05990	1,06	0,97	0,93	1,04	1,00	1,16	0,90	0,87	calcium-binding protein, putative
260955_at	At1g06000	0,85	0,64	0,57	1,26	1,25	0,91	0,79	0,76	UDP-glucoronosyl/UDP-glucosyl transferase family protein
260946_at	At1g06010	0,93	0,89	0,92	1,17	1,10	0,95	0,89	0,86	expressed protein
260947_at	At1g06020	0,97	0,93	0,82	0,97	1,00	0,96	0,94	1,03	pfkB-type carbohydrate kinase family protein
260956_at	At1g06040	1,33	1,63	1,92	1,03	0,86	1,22	1,21	1,34	zinc finger (B-box type) family protein / salt-tolerance protein (STO)
260959_at	At1g06050	1,36	1,26	1,32	1,12	0,97	1,20	1,25	1,31	expressed protein
260958_at	At1g06060	1,12	1,25	1,62	0,88	0,98	1,19	0,98	1,12	RanBPM-related
260953_at	At1g06070	1,03	1,10	1,18	0,95	0,97	0,94	0,94	1,21	bZIP transcription factor, putative (bZIP69)
260957_at	At1g06080	1,03	1,29	1,25	1,28	1,16	1,07	1,17	0,94	delta 9 desaturase (ADS1)
260948_at	At1g06100	1,03	0,94	1,10	1,00	0,93	1,24	1,17	1,03	fatty acid desaturase family protein
260949_at	At1g06110	0,85	1,01	1,15	0,86	0,91	0,90	0,88	0,82	F-box family protein
260950_s_at	At1g06120	1,48	1,17	1,07	2,28	2,05	1,17	1,36	1,28	fatty acid desaturase family protein
260954_at	At1g06130	0,79	0,68	0,67	0,91	1,09	0,85	1,04	0,95	hydroxyacylglutathione hydrolase, putative / glyoxalase II, putative
260952_at	At1g06140	0,92	1,15	0,94	0,90	1,09	1,04	1,02	1,12	pentatricopeptide (PPR) repeat-containing protein

260951_at	At1g06150	0,92	1,01	1,00	1,06	0,98	1,03	1,06	0,96 pentatricopeptide (PPR) repeat-containing protein
260783_at	At1g06160	0,65	0,81	0,96	0,77	1,01	0,73	0,93	0,93 ethylene-responsive factor, putative
257491_at	At1g06170	1,00	1,00	1,05	0,93	1,06	1,01	0,90	1,03 basic helix-loop-helix (bHLH) family protein
260784_at	At1g06180	0,78	0,90	0,87	0,93	1,11	1,04	0,86	0,98 myb family transcription factor
260793_at	At1g06190	1,13	0,97	0,99	0,68	0,86	1,05	1,09	0,97 expressed protein
260785_at	At1g06200	1,10	1,07	1,06	0,84	0,86	1,02	1,06	1,01 expressed protein
260794_at	At1g06210	1,28	1,24	1,62	1,01	1,58	0,99	1,10	1,14 VHS domain-containing protein / GAT domain-containing protein
260786_s_at	At1g06220	1,51	1,25	1,17	0,87	0,91	1,12	1,05	0,91 elongation factor Tu family protein
260795_at	At1g06225	1,09	0,95	1,10	1,11	1,02	1,04	1,05	1,15 Clavata3 / ESR-Related-3 (CLE3)
260787_at	At1g06230	0,93	0,98	0,99	1,00	1,08	1,09	1,01	1,17 DNA-binding bromodomain-containing protein
260790_at	At1g06240	0,80	0,91	0,80	0,68	0,64	1,01	1,06	0,96 expressed protein
260791_at	At1g06250	1,00	1,07	0,96	0,98	1,17	1,10	1,00	0,86 lipase class 3 family protein
260788_at	At1g06260	1,00	1,09	0,98	1,06	1,23	0,98	1,09	0,77 cysteine proteinase, putative
260792_at	At1g06270	0,93	0,99	0,95	0,86	0,87	1,08	0,92	1,07 pentatricopeptide (PPR) repeat-containing protein
260789_s_at	At1g06290	0,81	0,70	0,85	0,97	1,04	0,98	0,94	1,02 acyl-CoA oxidase (ACX3)
259419_at	At1g06310	0,99	1,05	0,96	1,06	1,20	0,96	0,91	1,05 acyl-CoA oxidase, putative
259389_at	At1g06320	1,08	0,95	0,95	1,06	1,50	1,20	0,88	1,04 hypothetical protein
259390_at	At1g06340	1,05	1,02	1,06	0,93	1,07	1,04	1,17	0,91 agenet domain-containing protein
259391_s_at	At1g06350	1,21	0,97	1,45	0,90	0,61	1,20	1,24	1,35 fatty acid desaturase family protein
259392_at	At1g06380	0,98	1,05	0,91	1,16	0,99	0,88	1,05	0,91 ribosomal protein-related
259396_at	At1g06390	0,93	0,94	1,09	0,91	0,82	1,01	1,01	1,14 shaggy-related protein kinase iota / ASK-iota (ASK9) (GSK1)
259395_at	At1g06400	0,97	0,96	1,17	1,04	0,96	1,05	1,15	1,20 Ras-related GTP-binding protein (ARA-2)
259393_at	At1g06410	0,74	0,76	1,02	0,98	0,92	1,02	1,09	1,07 glycosyl transferase family 20 protein / trehalose-phosphatase family protei
259394_at	At1g06420	1,00	0,94	0,93	0,99	1,10	0,98	0,96	0,98 expressed protein
262626_at	At1g06430	1,02	0,92	1,04	0,76	0,75	1,18	1,18	1,36 FtsH protease, putative
262625_at	At1g06440	0,91	0,81	0,89	0,84	0,95	0,91	1,18	0,89 expressed protein
262624_at	At1g06450	2,02	1,56	1,32	1,47	1,24	1,26	1,22	1,09 CCR4-NOT transcription complex protein, putative
262629_at	At1g06460	0,57	0,63	0,73	0,65	0,63	0,95	0,84	0,85 31.2 kDa small heat shock family protein / hsp20 family protein
262623_at	At1g06470	0,78	0,61	0,62	0,86	0,88	0,87	1,04	0,89 phosphate translocator-related
262628_at	At1g06490	0,89	1,12	1,01	0,95	0,87	1,08	1,01	1,07 glycosyl transferase family 48 protein
262631_at	At1g06500	1,00	1,00	1,01	1,12	1,22	1,04	0,88	0,81 expressed protein
262622_at	At1g06510	0,85	0,75	0,72	0,72	0,78	1,03	0,99	0,92 expressed protein
262630_at	At1g06520	1,00	1,04	1,08	1,07	1,30	0,96	0,95	0,89 phospholipid/glycerol acyltransferase family protein
262621_at	At1g06530	1,06	1,01	1,02	0,97	0,91	1,02	1,00	1,07 myosin heavy chain-related
262620_at	At1g06540	1,00	0,96	0,95	0,89	1,21	1,08	1,13	0,84 expressed protein
262619_at	At1g06550	1,06	1,02	0,90	1,18	1,33	1,22	1,19	1,41 enoyl-CoA hydratase/isomerase family protein
262618_at	At1g06560	1,44	1,48	1,49	0,97	0,98	0,84	1,05	1,19 NOL1/NOP2/sun family protein
262635_at	At1g06570	1,17	1,58	1,69	0,93	0,77	1,07	1,00	1,25 4-hydroxyphenylpyruvate dioxygenase (HPD)
262627_at	At1g06580	0,89	0,85	1,08	0,83	1,07	0,95	0,92	0,95 pentatricopeptide (PPR) repeat-containing protein
262617_at	At1g06590	1,02	1,10	1,00	0,94	0,92	1,06	0,97	0,92 expressed protein
262616_at	At1g06620	0,74	0,57	0,64	0,89	0,78	0,98	1,08	0,96 2-oxoglutarate-dependent dioxygenase, putative
262633_at	At1g06630	1,16	1,03	1,17	1,11	1,05	0,93	1,11	1,21 F-box family protein
262637_at	At1g06640	0,93	0,85	1,04	0,95	0,95	0,91	0,98	0,99 2-oxoglutarate-dependent dioxygenase, putative
262638_at	At1g06650	1,18	1,29	1,55	1,00	0,97	1,17	1,09	1,08 2-oxoglutarate-dependent dioxygenase, putative
262636_at	At1g06670	1,17	1,23	1,14	1,00	0,88	1,16	1,17	1,15 DEIH-box RNA/DNA helicase
262632_at	At1g06680	1,03	0,96	1,06	0,97	0,96	0,97	0,96	1,00 photosystem II oxygen-evolving complex 23 (OEC23)

262634_at	At1g06690	0,76	0,78	0,61	1,03	1,05	1,28	1,17	1,23	aldo/keto reductase family protein
260835_at	At1g06700	1,02	0,89	0,95	1,01	1,03	0,89	0,92	0,99	serine/threonine protein kinase, putative
260826_at	At1g06710	1,04	0,93	1,02	0,91	1,26	0,92	0,95	0,85	pentatricopeptide (PPR) repeat-containing protein
260824_at	At1g06720	1,06	0,99	0,88	0,72	0,65	1,07	1,16	1,37	expressed protein
260834_at	At1g06730	0,98	0,88	0,92	0,96	0,97	0,95	1,02	0,82	pfkB-type carbohydrate kinase family protein
260827_at	At1g06740	0,84	0,94	1,02	0,88	0,88	0,88	1,08	1,34	Mutator-like transposase family
260828_at	At1g06750	1,00	0,91	0,94	0,90	1,03	1,01	1,05	1,12	hypothetical protein
260830_at	At1g06760	0,84	0,98	1,03	0,75	1,15	0,84	0,88	0,88	histone H1, putative
260823_at	At1g06770	1,02	1,03	0,96	0,93	0,75	0,86	0,92	1,10	zinc finger (C3HC4-type RING finger) family protein
260832_at	At1g06780	1,01	0,77	0,78	0,97	1,14	1,03	0,93	0,81	glycosyl transferase family 8 protein
260822_at	At1g06790	1,14	1,27	1,35	1,26	1,14	0,90	0,99	0,96	RNA polymerase Rpb7 N-terminal domain-containing protein
260833_at	At1g06800	0,76	0,89	1,00	1,10	0,92	0,89	0,92	1,02	lipase class 3 family protein
260825_at	At1g06810	0,95	1,02	0,89	0,94	0,98	1,01	1,05	0,95	hypothetical protein
260821_at	At1g06820	1,00	0,92	0,82	1,08	0,82	0,89	0,93	0,90	carotenoid isomerase, putative
260831_at	At1g06830	1,41	1,27	1,24	1,59	1,10	1,02	0,92	1,00	glutaredoxin family protein
260820_at	At1g06840	0,77	0,85	0,84	0,90	0,97	0,95	0,91	1,03	leucine-rich repeat transmembrane protein kinase, putative
260819_at	At1g06850	0,80	0,76	0,90	0,99	0,91	0,77	0,92	0,83	bZIP transcription factor, putative
260829_s_at	At1g06870	0,82	0,75	1,01	0,74	0,77	0,91	1,03	1,04	signal peptidase, putative
260818_at	At1g06890	0,94	0,81	0,93	1,28	1,27	0,83	0,76	0,84	transporter-related
260817_at	At1g06900	1,01	0,76	0,92	0,75	0,91	0,93	0,90	0,89	peptidase M16 family protein / insulinase family protein
260816_at	At1g06930	1,03	1,10	0,99	0,94	1,07	0,86	0,88	1,09	expressed protein
260815_at	At1g06950	1,05	0,96	0,97	0,75	0,77	1,18	1,01	1,09	chloroplast inner envelope protein-related
256052_at	At1g06960	1,51	1,15	1,02	0,97	0,89	1,36	1,33	1,19	small nuclear ribonucleoprotein U2B, putative / spliceosomal protein, putati
256051_at	At1g06970	1,08	1,01	1,03	0,86	1,24	1,07	1,01	0,98	cation/hydrogen exchanger, putative (CHX14)
256066_at	At1g06980	0,97	1,02	0,96	0,88	0,90	1,09	1,07	1,05	expressed protein
256059_at	At1g06990	0,92	0,83	1,04	1,03	1,10	0,91	0,86	1,00	GDSL-motif lipase/hydrolase family protein
256050_at	At1g07000	0,66	0,65	0,81	0,78	0,92	1,11	1,03	1,12	exocyst subunit EXO70 family protein
256049_at	At1g07010	1,50	1,28	1,11	1,21	0,79	1,53	1,36	1,29	calcineurin-like phosphoesterase family protein
256064_at	At1g07020	0,89	0,86	0,80	1,14	1,52	0,97	0,86	0,76	expressed protein
256048_at	At1g07025	0,93	0,97	1,00	0,85	0,99	0,98	0,96	0,98	mitochondrial substrate carrier family protein
256055_at	At1g07030	0,80	0,74	0,70	0,91	0,85	0,91	0,84	0,79	mitochondrial substrate carrier family protein
256061_at	At1g07040	0,76	0,90	1,30	0,90	0,87	0,87	0,81	0,87	expressed protein
256060_at	At1g07050	0,71	0,79	0,73	0,21	0,32	1,09	1,03	0,93	CONSTANS-like protein-related
256047_at	At1g07060	1,03	0,96	1,06	1,04	1,07	0,91	0,89	0,87	expressed protein
256065_at	At1g07070	1,24	1,00	0,99	1,25	1,05	0,99	0,95	0,87	60S ribosomal protein L35a (RPL35aA)
256034_at	At1g07080	0,93	0,93	0,91	1,02	0,97	0,93	0,86	0,90	gamma interferon responsive lysosomal thiol reductase family protein / GIL
256062_at	At1g07090	0,78	0,85	0,98	0,95	1,00	0,82	0,93	0,83	expressed protein
256036_at	At1g07110	1,04	1,11	1,01	0,89	0,90	1,23	1,28	1,21	fructose-6-phosphate 2-kinase / fructose-2,6-bisphosphatase (F2KP)
256054_at	At1g07120	0,93	1,00	1,01	0,95	1,02	0,91	1,07	1,01	expressed protein
256063_at	At1g07130	0,90	0,82	0,74	0,98	0,98	0,96	0,85	0,97	OB-fold nucleic acid binding domain-containing protein
256046_at	At1g07135	0,66	0,99	1,13	0,91	0,85	0,95	1,42	1,04	glycine-rich protein
256035_at	At1g07140	1,03	0,92	0,89	1,01	0,98	0,85	0,91	0,89	Ran-binding protein 1a (RanBP1a)
256045_at	At1g07150	0,74	0,81	0,85	0,98	0,84	0,56	1,01	0,91	protein kinase family protein
256044_at	At1g07160	0,81	0,97	0,92	0,74	0,82	1,26	1,47	1,28	protein phosphatase 2C, putative / PP2C, putative
256067_at	At1g07170	1,41	1,33	1,45	0,96	0,99	0,99	1,06	1,29	expressed protein
256057_at	At1g07180	2,31	2,27	1,97	1,20	0,91	1,57	1,48	1,35	pyridine nucleotide-disulphide oxidoreductase family protein

256056_at	At1g07190	1,00	1,00	0,96	1,00	1,09	1,01	0,98	1,02 hypothetical protein
256043_at	At1g07210	1,49	1,41	1,36	1,22	1,07	0,99	0,88	0,89 30S ribosomal protein S18 family
256042_at	At1g07220	1,10	0,83	0,97	1,06	1,25	0,97	1,03	0,89 expressed protein
256041_at	At1g07230	1,01	1,02	1,06	0,85	1,07	1,07	1,01	0,93 phosphoesterase family protein
256058_at	At1g07240	0,74	0,64	0,68	0,91	1,02	0,84	0,76	0,84 UDP-glucoronosyl/UDP-glucosyl transferase family protein
256033_at	At1g07250	0,77	0,92	0,90	0,93	0,81	0,84	0,80	0,88 UDP-glucoronosyl/UDP-glucosyl transferase family protein
256053_at	At1g07260	0,88	1,21	0,84	1,11	1,21	0,79	0,99	0,77 UDP-glucoronosyl/UDP-glucosyl transferase family protein
256040_at	At1g07270	0,96	0,95	0,99	0,93	0,88	1,07	1,05	1,10 cell division control protein CDC6b, putative (CDC6b)
261075_at	At1g07280	0,83	1,20	1,41	0,60	0,67	1,00	1,06	1,28 expressed protein
261074_at	At1g07290	0,98	1,16	1,13	1,02	1,00	1,08	0,93	0,81 nucleotide-sugar transporter family protein
261073_at	At1g07300	0,85	0,57	0,55	1,26	1,32	1,22	0,89	0,99 josephin protein-related
261083_at	At1g07310	0,85	0,94	1,07	1,02	1,02	1,02	0,90	0,98 C2 domain-containing protein
261078_at	At1g07320	1,02	1,00	0,98	0,80	0,86	0,97	1,00	0,96 50S ribosomal protein L4, chloroplast (CL4)
257489_at	At1g07330	1,00	0,98	1,00	0,89	1,14	0,97	0,91	0,93 hypothetical protein
261072_at	At1g07340	0,91	1,06	1,02	1,00	1,00	1,00	0,94	0,81 hexose transporter, putative
261081_at	At1g07350	1,08	1,44	1,34	0,92	1,31	1,15	1,19	1,59 transformer serine/arginine-rich ribonucleoprotein, putative
261082_at	At1g07360	1,16	1,17	1,36	1,12	1,43	1,10	0,87	0,89 zinc finger (CCCH-type) family protein / RNA recognition motif (RRM)-conta
261080_at	At1g07370	1,83	1,64	1,48	1,80	1,54	0,99	0,94	0,93 proliferating cell nuclear antigen 1 (PCNA1)
261071_at	At1g07380	0,94	1,10	1,05	0,92	0,89	0,93	1,07	1,20 ceramidase family protein
261070_at	At1g07390	1,31	1,08	1,10	0,89	1,05	1,11	0,90	0,81 leucine-rich repeat family protein
261069_at	At1g07410	1,11	1,05	1,24	1,11	1,49	0,97	1,00	0,94 Ras-related GTP-binding protein, putative
261076_at	At1g07420	0,94	0,93	1,06	1,21	1,17	1,06	0,91	0,94 sterol 4-alpha-methyl-oxidase 2 (SMO2)
261077_at	At1g07430	0,89	0,92	1,04	0,92	0,99	0,92	0,99	0,95 protein phosphatase 2C, putative / PP2C, putative
261084_at	At1g07440	1,52	1,66	1,92	0,76	0,71	1,38	1,51	1,56 tropinone reductase, putative / tropine dehydrogenase, putative
261068_at	At1g07450	1,49	1,40	1,27	1,08	0,98	1,21	1,13	0,97 tropinone reductase, putative / tropine dehydrogenase, putative
261067_at	At1g07460	0,95	1,00	0,98	1,09	1,07	0,91	0,87	1,10 legume lectin family protein
261079_s_at	At1g07470	0,98	0,86	1,07	0,82	0,96	1,24	1,04	1,12 transcription factor IIA large subunit, putative / TFIIA large subunit, putative
261066_at	At1g07490	1,05	1,02	0,93	1,09	0,99	0,97	0,97	1,00 protein coding
261065_at	At1g07500	1,26	1,25	1,06	1,26	0,98	1,14	1,20	1,19 expressed protein
261064_at	At1g07510	0,93	0,81	0,89	1,10	1,01	1,01	1,10	1,21 FtsH protease, putative
261063_at	At1g07520	0,90	1,05	0,89	0,86	1,06	1,15	1,08	1,03 scarecrow transcription factor family protein
261062_at	At1g07530	0,80	0,96	1,12	0,78	0,98	1,01	1,14	1,08 scarecrow-like transcription factor 14 (SCL14)
261061_at	At1g07540	1,00	0,95	1,03	1,10	1,18	0,89	1,09	0,95 telomere-binding protein, putative
261091_at	At1g07550	0,88	1,07	1,04	0,99	0,99	1,07	1,08	0,97 leucine-rich repeat protein kinase, putative
261090_at	At1g07560	0,89	0,95	1,12	0,99	0,96	1,08	1,16	0,80 leucine-rich repeat protein kinase, putative
261089_at	At1g07570	1,56	1,48	1,89	1,05	1,11	1,16	1,22	1,15 protein kinase (APK1a)
261088_at	At1g07590	1,11	1,03	0,87	1,14	1,05	1,01	0,94	1,05 pentatricopeptide (PPR) repeat-containing protein
261438_at	At1g07600	1,08	1,04	0,99	1,20	1,18	1,03	1,05	0,99 pentatricopeptide (PPR) repeat-containing protein
261410_at	At1g07610	1,29	1,31	1,21	3,06	2,74	1,10	1,08	1,02 metallothionein-like protein 1C (MT-1C)
261435_at	At1g07620	1,29	1,22	1,18	0,82	0,74	0,96	0,89	0,96 GTP1/OBG family protein
261413_at	At1g07630	0,58	0,60	0,54	0,89	0,87	0,91	0,68	1,08 protein phosphatase 2C family protein / PP2C family protein
261409_at	At1g07640	0,86	0,90	0,80	0,99	1,05	0,85	0,89	1,01 Dof-type zinc finger domain-containing protein
261434_at	At1g07650	0,76	0,73	0,89	0,90	1,01	0,82	0,83	0,90 leucine-rich repeat transmembrane protein kinase, putative
261433_s_at	At1g07670	0,99	0,94	1,17	0,98	1,09	0,95	0,99	1,03 calcium-transporting ATPase 4, endoplasmic reticulum-type (ECA4)
261432_at	At1g07680	1,04	0,87	0,89	0,85	1,00	0,95	1,00	0,84 hypothetical protein
261466_at	At1g07690	0,83	0,84	0,84	0,83	0,69	1,09	1,25	0,84 hypothetical protein

261417_at	At1g07700	1,09	0,92	1,03	1,02	1,13	1,07	1,01	0,99	thioredoxin family protein
261465_at	At1g07705	1,29	1,71	2,39	0,87	0,93	1,05	1,46	1,27	NOT2/NOT3/NOT5 family protein
261420_at	At1g07720	0,74	0,73	0,67	0,88	1,06	0,65	0,59	0,65	beta-ketoacyl-CoA synthase family protein
261464_at	At1g07730	0,90	0,99	1,07	0,93	1,01	0,90	1,00	0,94	disease resistance-responsive family protein
261463_at	At1g07740	1,03	0,90	0,86	0,84	0,65	0,97	1,02	0,93	pentatricopeptide (PPR) repeat-containing protein
261437_at	At1g07745	1,07	0,99	1,05	0,85	0,90	1,11	1,18	1,14	DNA repair family protein
261415_at	At1g07750	0,89	0,79	0,74	1,37	1,32	0,96	0,93	0,97	cupin family protein
261416_at	At1g07770	1,45	1,31	1,22	1,22	1,14	0,96	0,96	0,91	40S ribosomal protein S15A (RPS15aA)
261411_at	At1g07790	0,84	0,73	0,69	1,15	1,10	0,95	0,94	0,92	histone H2B, putative
261414_at	At1g07795	0,89	0,87	1,20	1,14	1,08	0,87	1,03	0,93	expressed protein
261408_s_at	At1g07820	0,86	0,77	0,80	1,02	1,06	1,00	0,89	0,87	histone H4
261418_at	At1g07830	1,00	1,05	1,08	1,15	1,16	0,90	1,00	0,76	ribosomal protein L29 family protein
261419_at	At1g07840	1,08	0,87	0,97	0,88	0,84	0,98	1,03	1,13	leucine zipper factor-related
261462_at	At1g07850	1,13	0,96	1,05	1,00	1,07	0,88	1,06	1,04	fringe-related protein
261461_at	At1g07860	0,86	0,82	1,12	0,96	1,02	1,05	0,89	0,96	expressed protein
261436_at	At1g07870	0,62	0,81	1,10	1,12	1,15	1,00	1,09	0,97	protein kinase family protein
261460_at	At1g07880	1,16	1,15	1,00	1,31	1,09	1,04	0,93	0,73	mitogen-activated protein kinase, putative / MAPK, putative (MPK13)
261412_at	At1g07890	0,92	0,84	0,95	1,06	1,02	0,84	0,87	0,90	L-ascorbate peroxidase 1, cytosolic (APX1)
260677_at	At1g07910	0,94	1,10	1,10	0,92	1,10	0,95	0,94	1,07	expressed protein
257495_at	At1g07960	1,51	1,20	1,09	1,29	1,11	1,16	1,14	1,02	thioredoxin family protein
260678_at	At1g07970	1,11	1,24	1,14	0,97	0,94	1,06	0,93	0,99	expressed protein
260622_at	At1g07980	1,01	1,07	0,91	0,86	1,13	1,06	0,96	1,05	histone-like transcription factor (CBF/NF-Y) family protein
260679_at	At1g07990	1,00	1,01	1,05	0,88	1,11	1,19	1,12	1,02	SIT4 phosphatase-associated family protein
260680_s_at	At1g08000	1,02	1,08	1,22	1,13	0,85	1,03	1,06	0,99	zinc finger (GATA type) family protein
260681_at	At1g08020	1,13	1,06	1,05	0,93	1,02	1,09	1,15	1,09	expressed protein
260647_at	At1g08030	1,14	1,17	1,66	0,81	1,09	1,14	1,23	1,23	expressed protein
260626_at	At1g08040	0,90	0,87	0,98	0,92	0,90	0,99	1,13	1,38	expressed protein
260648_at	At1g08050	1,14	0,99	1,26	1,93	2,46	1,21	1,01	1,02	zinc finger (C3HC4-type RING finger) family protein
260625_at	At1g08060	1,09	1,13	1,31	0,94	0,87	1,14	1,08	1,14	MOM1
260621_at	At1g08065	0,94	1,07	1,06	0,96	1,17	1,03	1,11	1,13	carbonic anhydrase family protein
260620_at	At1g08070	1,04	1,15	1,18	0,92	0,93	1,14	1,05	1,17	pentatricopeptide (PPR) repeat-containing protein
260649_at	At1g08080	0,94	0,93	1,07	0,95	1,03	1,06	0,98	0,94	carbonic anhydrase family protein
260623_at	At1g08090	0,94	0,99	0,87	1,02	1,07	1,06	0,99	0,76	high-affinity nitrate transporter (ACH1)
260624_at	At1g08100	0,92	0,97	0,96	1,12	1,03	0,97	0,99	0,86	high-affinity nitrate transporter (ACH2)
260619_at	At1g08110	1,22	1,25	1,16	0,88	0,88	1,14	1,12	1,15	lactoylglutathione lyase, putative / glyoxalase I, putative
261810_at	At1g08130	1,46	1,12	1,13	0,90	0,87	1,11	1,14	1,07	DNA ligase / polydeoxyribonucleotide synthase (ATP)
261816_at	At1g08150	1,01	0,96	1,01	0,94	1,08	1,03	1,09	1,06	cation/hydrogen exchanger (CHX6b)
261755_at	At1g08170	1,05	0,94	0,89	0,88	0,92	0,93	0,95	1,02	histone H2B family protein
261817_at	At1g08180	0,98	1,06	1,11	0,90	0,82	0,98	0,99	0,83	expressed protein
261783_at	At1g08190	0,95	0,90	0,86	0,77	0,90	0,95	0,96	0,91	vacuolar assembly protein, putative (VPS41)
261757_at	At1g08210	1,17	1,07	1,25	0,78	0,88	0,93	0,91	1,02	aspartyl protease family protein
261784_at	At1g08220	1,02	0,96	0,98	1,13	1,10	0,95	0,82	1,00	expressed protein
261785_at	At1g08230	0,47	0,64	0,91	1,26	1,09	0,79	0,84	0,79	amino acid transporter family protein
261758_at	At1g08250	0,71	0,89	1,03	1,20	1,38	0,93	1,00	0,82	prephenate dehydratase family protein
261811_at	At1g08260	0,98	0,98	1,01	1,03	1,05	0,97	1,21	1,08	DNA-directed DNA polymerase epsilon catalytic subunit, putative
261812_at	At1g08270	1,04	1,01	1,10	1,12	1,08	1,16	1,11	0,94	expressed protein

261813_at	At1g08280	0,94	0,77	0,62	0,98	0,94	0,90	0,70	0,67	glycosyl transferase family 29 protein / sialyltransferase family protein
261814_at	At1g08310	1,51	0,95	1,13	1,92	1,70	1,20	1,34	1,38	esterase/lipase/thioesterase family protein
261756_at	At1g08320	0,58	0,67	0,67	0,86	1,08	0,93	1,11	1,34	armadillo/beta-catenin repeat family protein
261815_at	At1g08325	1,03	1,12	1,00	0,89	1,03	1,28	1,07	1,11	bZIP family transcription factor
261809_at	At1g08340	1,07	1,03	0,99	1,03	1,25	0,93	0,99	1,16	rac GTPase activating protein, putative
261747_at	At1g08350	0,94	0,92	0,76	1,36	0,85	0,77	0,82	0,83	endomembrane protein 70 family protein
261694_at	At1g08360	1,53	1,42	1,29	1,17	1,15	1,07	1,04	1,05	60S ribosomal protein L10A (RPL10aA)
261693_at	At1g08370	1,00	1,14	1,44	1,19	0,91	1,12	0,88	1,20	hydroxyproline-rich glycoprotein family protein
261746_at	At1g08380	0,94	0,94	0,98	0,92	0,92	1,02	0,97	0,97	expressed protein
261742_at	At1g08390	1,08	1,17	1,18	1,48	1,39	0,97	0,97	1,10	expressed protein
261743_s_at	At1g08420	0,80	0,69	0,82	0,99	1,10	1,11	0,98	1,07	kelch repeat-containing protein / serine/threonine phosphoesterase family p
257481_at	At1g08430	0,89	0,84	0,78	1,26	0,96	1,00	1,10	1,06	expressed protein
261692_at	At1g08450	0,79	0,69	0,66	1,03	0,97	0,93	0,99	0,92	calreticulin 3 (CRT3)
261720_at	At1g08460	0,99	0,98	1,04	0,76	0,91	0,93	0,91	0,89	histone deacetylase family protein (HDA8)
261696_at	At1g08470	0,85	0,88	0,90	1,21	1,03	0,92	0,88	0,91	strictosidine synthase family protein
261721_at	At1g08480	1,22	1,09	0,89	1,26	1,22	0,94	0,90	0,90	expressed protein
261744_at	At1g08490	0,90	0,95	0,85	0,87	0,74	1,12	1,00	0,98	cysteine desulfurase, putative
261745_at	At1g08500	0,89	0,85	0,95	1,34	1,28	0,74	0,80	0,90	plastocyanin-like domain-containing protein
261722_at	At1g08510	0,88	0,84	0,80	1,08	0,96	0,85	0,87	0,97	acyl-(acyl carrier protein) thioesterase / acyl-ACP thioesterase / oleoyl-(acyl
261695_at	At1g08520	0,83	0,84	0,87	0,78	0,82	1,06	1,04	1,10	magnesium-chelatase subunit chlD, chloroplast, putative / Mg-protoporphyr
264781_at	At1g08540	1,19	1,12	1,63	0,83	0,93	1,25	1,14	1,13	RNA polymerase sigma subunit SigB (sigB) / sigma factor 2 (SIG2)
264799_at	At1g08550	0,92	0,87	1,27	0,69	0,78	1,32	1,29	1,45	violaxanthin de-epoxidase precursor, putative (AVDE1)
264802_at	At1g08560	1,47	1,42	1,30	1,50	1,67	0,98	0,87	0,72	syntaxin-related protein KNOLLE (KN) / syntaxin 111 (SYP111)
264779_at	At1g08570	1,23	1,40	1,34	1,39	1,27	1,04	1,10	1,19	thioredoxin family protein
264803_at	At1g08580	1,61	1,24	1,10	1,25	1,27	0,99	0,95	1,07	expressed protein
264804_at	At1g08590	0,95	1,04	1,04	0,93	1,08	0,94	1,07	0,98	CLAVATA1 receptor kinase (CLV1)
264805_at	At1g08600	1,13	1,32	1,78	0,84	1,05	1,24	1,33	1,57	SNF2 domain-containing protein / helicase domain-containing protein
264806_at	At1g08610	0,98	0,71	0,96	0,78	0,78	0,96	0,82	0,90	pentatricopeptide (PPR) repeat-containing protein
264792_at	At1g08620	0,71	0,92	1,32	0,97	1,26	1,06	1,18	1,26	transcription factor jumonji (jmj) family protein / zinc finger (C5HC2 type) far
264777_at	At1g08630	0,86	1,24	2,00	0,78	0,78	0,69	0,69	0,79	L-allo-threonine aldolase-related
264811_at	At1g08640	0,94	0,90	0,84	0,82	0,82	0,97	0,94	0,92	expressed protein
264783_at	At1g08650	0,99	0,97	0,81	0,93	1,00	1,02	1,00	0,70	phosphoenolpyruvate carboxylase kinase
264793_at	At1g08660	0,87	0,83	0,80	0,87	0,98	0,77	0,88	0,84	glycosyl transferase family 29 protein / sialyltransferase family protein
264794_at	At1g08670	1,04	1,07	0,96	0,96	0,97	0,93	0,94	0,90	epsin N-terminal homology (ENTH) domain-containing protein / clathrin ass
264795_at	At1g08680	1,06	0,94	1,05	0,88	1,04	0,85	0,85	1,02	ARF GAP-like zinc finger-containing protein ZiGA4 (ZIGA4)
264796_at	At1g08690	0,92	0,94	1,02	0,91	0,91	0,82	0,98	1,06	ARF GAP-like zinc finger-containing protein ZiGA4 (ZIGA4)
264807_at	At1g08700	0,99	1,18	0,97	1,26	1,23	0,88	0,93	0,77	presenilin family protein
264797_at	At1g08710	0,82	0,72	0,89	0,86	1,00	0,91	0,99	1,02	F-box family protein
264780_at	At1g08720	0,72	1,03	2,14	0,81	0,72	1,02	1,22	1,76	mitogen-activated protein kinase kinase kinase (MAPKKK) (EDR1)
264798_at	At1g08730	0,92	1,07	0,84	1,08	0,88	1,03	1,01	0,99	myosin heavy chain (PCR43)
264810_at	At1g08750	0,98	0,95	0,95	0,93	1,06	0,99	1,04	0,99	GPI-anchor transamidase, putative
264808_at	At1g08770	0,82	0,70	0,67	0,99	0,81	0,84	0,74	0,74	prenylated rab acceptor (PRA1) family protein
264778_at	At1g08780	1,21	0,94	1,06	1,11	1,24	1,07	1,01	1,11	prefoldin, putative
264800_at	At1g08800	1,09	0,87	0,96	1,93	2,24	1,46	1,12	1,20	expressed protein
264782_at	At1g08810	1,09	1,02	0,91	0,89	1,17	1,02	0,81	0,76	myb family transcription factor (MYB60)
264809_at	At1g08830	0,75	0,71	0,65	1,10	1,11	0,85	0,80	0,88	superoxide dismutase (Cu-Zn) (SODCC) / copper/zinc superoxide dismutas

264801_at	At1g08840	1,08	0,93	0,94	0,84	0,78	0,86	0,96	0,72	DNA replication helicase, putative
264646_at	At1g08860	0,82	0,79	0,90	0,91	0,68	1,18	1,38	1,11	copine, putative
264651_at	At1g08880	1,17	1,35	1,33	1,33	1,69	0,89	0,96	1,05	histone H2A, putative
264654_s_at	At1g08900	0,74	0,83	0,85	0,86	0,76	1,06	1,08	1,16	sugar transporter family protein
264652_at	At1g08920	0,75	1,24	1,26	1,43	1,10	1,01	2,03	2,13	sugar transporter, putative
264624_at	At1g08930	0,87	0,90	1,02	0,90	0,86	1,01	1,03	1,10	early-responsive to dehydration stress protein (ERD6) / sugar transporter fa
264645_at	At1g08940	1,11	1,22	1,50	0,91	1,25	1,09	1,10	1,33	phosphoglycerate/bisphosphoglycerate mutase family protein
264644_at	At1g08960	0,86	0,86	0,87	0,77	0,86	0,95	0,90	1,02	cation exchanger, putative (CAX11)
264650_at	At1g08970	0,92	0,97	0,96	1,07	0,97	0,95	0,91	1,09	CCAAT-box binding transcription factor Hap5a, putative
264653_at	At1g08980	0,68	0,64	0,75	0,75	0,86	0,91	0,94	0,99	amidase family protein
264643_at	At1g08990	1,03	1,01	1,09	0,98	0,97	0,97	1,02	1,06	glycogenin glucosyltransferase (glycogenin)-related
264623_at	At1g09000	0,94	1,02	0,96	1,18	1,35	0,93	1,13	1,04	NPK1-related protein kinase, putative (ANP1)
264656_at	At1g09010	0,72	0,82	1,13	0,93	0,78	0,97	0,85	1,01	glycoside hydrolase family 2 protein
264625_at	At1g09020	0,85	0,81	0,94	1,21	1,09	1,01	0,84	0,83	protein kinase, putative
264642_at	At1g09030	0,96	0,97	0,92	0,96	1,15	1,01	1,03	1,07	histone-like transcription factor (CBF/NF-Y) family protein
264649_at	At1g09060	0,83	0,95	1,31	0,95	0,83	0,97	1,27	1,56	transcription factor jumonji (jmjC) domain-containing protein
264655_at	At1g09070	0,87	1,04	1,40	0,97	0,97	1,06	1,19	1,71	C2 domain-containing protein / src2-like protein, putative
264648_at	At1g09080	1,18	0,96	0,96	1,09	0,91	1,40	1,23	1,12	luminal binding protein 3 (BiP-3) (BP3)
264647_at	At1g09090	1,00	0,99	1,00	1,02	1,06	1,12	0,96	1,02	respiratory burst oxidase protein B (RbohB) / NADPH oxidase
264657_at	At1g09100	0,88	0,90	0,89	0,81	0,93	0,93	0,95	0,96	26S protease regulatory subunit 6A, putative
264641_at	At1g09130	0,87	0,85	0,85	1,06	0,98	0,98	0,99	0,98	ATP-dependent Clp protease proteolytic subunit, putative
264255_at	At1g09140	1,27	1,35	1,56	0,90	1,05	1,14	1,26	1,36	SF2/ASF-like splicing modulator (SRP30)
264254_at	At1g09150	1,28	1,07	1,03	1,16	1,04	0,99	0,94	0,98	pseudouridine synthase and archaeosine transglycosylase (PUA) domain-c
264263_at	At1g09155	0,96	0,97	1,02	1,01	1,00	0,97	0,98	0,92	SKP1 interacting partner 3-related
264266_at	At1g09160	0,78	0,69	0,78	1,11	0,99	0,94	0,91	0,92	protein phosphatase 2C-related / PP2C-related
264253_at	At1g09170	0,94	1,06	1,20	1,05	0,96	1,18	1,05	1,02	kinesin motor protein-related
264252_at	At1g09180	1,27	1,26	1,34	1,21	1,27	1,14	1,01	0,77	GTP-binding protein, putative
264251_at	At1g09190	1,05	0,98	1,16	0,94	0,99	0,95	0,94	1,01	pentatricopeptide (PPR) repeat-containing protein
264262_at	At1g09200	1,36	1,32	1,64	1,90	2,17	0,98	0,88	0,67	histone H3
264260_at	At1g09210	1,20	1,07	0,98	1,20	1,19	0,96	0,92	1,04	calreticulin 2 (CRT2)
264258_at	At1g09220	1,03	0,85	1,07	0,98	0,85	0,93	1,00	0,99	pentatricopeptide (PPR) repeat-containing protein
264257_at	At1g09230	1,15	1,23	1,38	0,82	0,89	0,86	1,07	1,04	RNA recognition motif (RRM)-containing protein
264261_at	At1g09240	1,16	1,23	1,24	1,01	1,13	0,70	0,62	0,57	nicotianamine synthase, putative
264264_at	At1g09250	0,77	0,80	0,87	1,04	1,11	0,76	0,96	0,85	expressed protein
264256_at	At1g09270	1,05	1,07	0,98	1,17	1,20	0,89	0,89	0,82	importin alpha-1 subunit, putative (IMPA4)
264265_at	At1g09280	1,12	1,17	1,16	1,02	0,82	0,99	0,96	1,15	expressed protein
264259_at	At1g09290	0,84	0,79	0,77	1,11	0,84	0,92	0,89	0,93	expressed protein
263707_at	At1g09300	0,68	0,65	0,55	0,88	0,99	0,71	0,86	0,71	metallopeptidase M24 family protein
263709_at	At1g09310	1,60	1,40	1,52	1,78	1,72	0,99	0,88	0,93	expressed protein
263708_at	At1g09320	0,91	0,88	1,19	0,88	1,11	1,03	0,87	1,12	agenet domain-containing protein
263710_at	At1g09330	0,95	0,90	0,95	1,10	1,16	0,92	0,85	0,84	expressed protein
263676_at	At1g09340	1,16	1,09	1,15	0,98	1,06	1,31	1,23	1,14	expressed protein
264511_at	At1g09350	0,89	1,10	1,12	0,93	0,93	0,97	1,05	1,10	galactinol synthase, putative
264555_at	At1g09360	1,13	1,03	1,02	0,97	1,10	1,20	0,89	1,05	invertase/pectin methylesterase inhibitor family protein
264500_at	At1g09370	1,00	0,99	0,95	0,89	0,98	1,06	0,99	0,96	pectinesterase inhibitor domain-containing protein
264505_at	At1g09380	0,62	0,73	0,55	0,81	0,94	1,11	0,94	1,21	integral membrane family protein / nodulin MtN21-related

264501_at	At1g09390	0,78	0,63	0,70	0,88	1,06	0,90	0,95	1,00	GDSL-motif lipase/hydrolase family protein
264502_at	At1g09400	0,94	0,92	0,92	0,96	1,01	0,93	0,97	0,94	12-oxophytodienoate reductase, putative
264503_at	At1g09410	1,07	0,96	1,01	0,93	0,90	1,02	1,03	1,03	pentatricopeptide (PPR) repeat-containing protein
264507_at	At1g09415	0,69	0,66	0,71	0,77	1,00	0,96	0,77	0,81	NPR1/NIM1-interacting protein 3 (NIMIN-3)
264513_at	At1g09420	0,66	0,71	0,66	1,03	1,04	0,98	1,18	1,20	glucose-6-phosphate 1-dehydrogenase, putative / G6PD, putative
264504_at	At1g09430	0,93	0,87	0,91	1,03	1,09	0,90	0,86	0,85	ATP-citrate synthase (ATP-citrate (pro-S-)-lyase/citrate cleavage enzyme),
264549_at	At1g09440	1,00	1,06	1,11	0,78	0,85	1,14	1,16	1,07	protein kinase family protein
264550_at	At1g09450	1,12	1,04	1,21	0,96	1,06	0,85	0,90	0,93	haspin-related
264551_at	At1g09460	1,10	1,29	1,37	1,10	1,53	0,69	1,01	0,96	glucan endo-1,3-beta-glucosidase-related
264552_at	At1g09470	1,09	1,05	1,08	1,20	1,03	0,98	0,99	1,28	expressed protein
264553_s_at	At1g09480	0,80	0,73	0,86	0,95	1,06	1,03	1,06	0,99	cinnamyl-alcohol dehydrogenase family / CAD family
264514_at	At1g09500	0,53	0,45	0,51	1,01	0,99	1,43	1,35	1,07	cinnamyl-alcohol dehydrogenase family / CAD family
264554_at	At1g09510	1,00	0,89	1,06	1,02	1,11	1,03	0,98	0,95	cinnamyl-alcohol dehydrogenase family / CAD family
264509_at	At1g09520	0,82	0,91	1,08	0,79	0,77	0,95	0,74	0,85	expressed protein
264510_at	At1g09530	0,93	1,16	1,33	0,91	1,12	0,84	0,76	0,96	phytochrome interacting factor 3 (PIF3)
264556_at	At1g09540	1,01	1,11	0,93	0,92	1,06	0,95	0,90	0,93	myb family transcription factor (MYB61)
264557_at	At1g09550	0,89	1,07	1,10	1,06	1,04	1,11	0,90	0,95	pectinacetylesterase, putative
264506_at	At1g09560	0,93	0,99	0,78	1,47	1,17	1,12	0,94	0,89	germin-like protein (GLP4) (GLP5)
264508_at	At1g09570	0,82	0,92	1,10	0,79	0,77	0,93	0,97	0,99	phytochrome A (PHYA)
264512_at	At1g09575	0,68	0,97	0,93	0,93	1,35	0,91	1,16	0,86	expressed protein
264558_at	At1g09600	1,03	1,03	1,06	0,96	1,02	1,05	1,00	1,00	protein kinase family protein
264559_at	At1g09610	1,02	0,97	1,02	1,02	1,29	1,05	0,97	0,98	expressed protein
264705_at	At1g09620	1,17	1,01	1,00	0,90	0,93	0,99	0,88	0,93	tRNA synthetase class I (I, L, M and V) family protein
264669_at	At1g09630	1,17	0,96	1,02	1,42	1,53	1,01	0,84	0,93	Ras-related GTP-binding protein, putative
264670_s_at	At1g09640	1,19	1,08	0,99	1,02	1,06	0,99	1,03	0,98	elongation factor 1B-gamma, putative / eEF-1B gamma, putative
264664_at	At1g09660	1,06	0,98	0,91	0,95	0,90	1,09	1,11	1,19	KH domain-containing quaking protein, putative
264665_at	At1g09670	0,98	0,93	1,02	0,77	0,88	0,93	1,12	1,17	KH domain-containing quaking protein, putative
264666_at	At1g09680	0,95	1,03	1,03	0,91	0,89	0,98	1,01	0,90	pentatricopeptide (PPR) repeat-containing protein
264679_s_at	At1g09690	1,49	1,23	1,11	1,23	1,13	1,08	1,03	0,94	60S ribosomal protein L21 (RPL21A)
264677_at	At1g09700	1,07	0,91	0,94	0,94	1,02	1,02	1,06	0,92	double-stranded RNA-binding domain (DsRBD)-containing protein
264706_at	At1g09720	0,96	0,94	1,03	0,99	1,12	1,03	1,02	1,07	kinase interacting family protein
264707_at	At1g09730	0,97	1,06	1,11	0,93	0,93	1,19	1,06	1,31	Ulp1 protease family protein
264708_at	At1g09740	0,76	0,80	0,80	0,89	1,05	0,84	0,89	0,96	ethylene-responsive protein, putative
264672_at	At1g09750	0,91	1,03	1,25	0,66	0,90	0,64	0,54	0,59	chloroplast nucleoid DNA-binding protein-related
264678_at	At1g09760	1,45	1,53	1,58	1,22	1,32	1,02	1,07	1,25	U2 small nuclear ribonucleoprotein A, putative
264709_at	At1g09770	1,12	1,08	1,17	1,00	1,00	1,13	1,02	1,24	myb family transcription factor
264668_at	At1g09780	1,22	0,91	1,00	1,96	1,57	1,01	0,97	0,99	2,3-biphosphoglycerate-independent phosphoglycerate mutase, putative / p
264710_at	At1g09790	0,90	1,06	0,96	1,03	1,16	1,17	1,11	1,06	phytochelatin synthetase-related
264673_at	At1g09795	0,69	0,59	0,59	0,64	0,90	0,84	0,97	0,96	ATP phosphoribosyl transferase 2 (ATP-PRT2)
264711_at	At1g09800	0,83	0,97	0,98	0,98	1,19	0,87	0,90	0,90	tRNA pseudouridine synthase family protein
264712_at	At1g09810	0,95	0,87	1,02	1,05	1,02	1,00	0,91	0,83	expressed protein
264674_at	At1g09815	1,32	1,11	1,08	1,17	1,21	1,04	0,91	1,04	DNA polymerase delta subunit 4 family
264713_at	At1g09820	1,01	0,89	0,97	1,10	0,97	1,09	0,88	0,96	pentatricopeptide (PPR) repeat-containing protein
264675_at	At1g09830	1,16	1,01	0,97	1,00	0,81	0,92	0,91	0,74	phosphoribosylamine--glycine ligase (PUR2)
264686_at	At1g09840	1,03	0,93	0,81	1,15	1,02	1,00	1,04	0,99	shaggy-related protein kinase kappa / ASK-kappa (ASK10)
264687_at	At1g09850	0,90	0,89	0,86	0,96	0,87	1,00	0,98	1,03	cysteine protease, papain-like (XBCP3)

264676_at	At1g09870	0,95	0,82	0,80	1,19	1,28	0,88	0,85	0,77 histidine acid phosphatase family protein
264688_at	At1g09890	1,27	1,04	1,01	1,07	1,07	0,86	0,90	1,02 expressed protein
264689_at	At1g09900	0,79	0,69	0,84	0,88	0,79	0,84	0,81	0,86 pentatricopeptide (PPR) repeat-containing protein
264658_at	At1g09910	0,68	0,72	0,86	1,19	1,29	0,92	0,89	1,14 expressed protein
264671_at	At1g09920	1,02	0,99	1,33	0,97	1,10	1,02	1,03	1,38 TRAF-type zinc finger-related
264659_at	At1g09930	1,01	1,16	1,17	0,98	1,03	1,06	1,17	1,22 oligopeptide transporter OPT family protein
264660_at	At1g09940	0,66	0,73	0,99	0,85	0,71	1,10	1,26	1,31 glutamyl-tRNA reductase 2 / GluTR (HEMA2)
264661_at	At1g09950	0,80	0,94	0,93	1,16	1,36	0,89	1,03	0,91 transcription factor-related
264662_at	At1g09960	0,79	0,75	0,74	0,80	0,83	0,94	1,07	0,95 sucrose transporter / sucrose-proton symporter (SUT4)
264663_at	At1g09970	0,74	0,73	0,83	0,80	0,75	0,95	0,93	0,91 leucine-rich repeat transmembrane protein kinase, putative
264667_s_at	At1g09980	1,02	0,90	0,90	0,96	0,97	0,91	1,02	0,91 expressed protein
264518_at	At1g09990	1,03	0,78	0,76	0,86	0,97	0,88	0,94	0,96 expressed protein
264519_at	At1g10000	0,96	1,01	1,12	0,82	0,88	1,08	1,03	0,79 expressed protein
264520_at	At1g10010	0,99	1,03	1,02	1,18	0,98	0,86	1,12	1,00 amino acid permease, putative
264521_at	At1g10020	0,99	1,14	0,96	0,80	1,21	0,80	0,99	1,32 expressed protein
264523_at	At1g10030	1,29	1,30	1,00	1,35	1,14	1,01	0,90	0,87 integral membrane family protein
264522_at	At1g10050	1,02	1,01	0,93	0,95	0,76	0,94	1,18	1,03 glycosyl hydrolase family 10 protein / carbohydrate-binding domain-containi
264525_at	At1g10060	0,76	0,93	1,31	0,72	0,70	0,99	1,31	1,37 branched-chain amino acid aminotransferase 1 / branched-chain amino aci
264524_at	At1g10070	0,79	0,98	1,41	0,84	0,77	0,67	0,77	0,89 branched-chain amino acid aminotransferase 2 / branched-chain amino aci
264515_at	At1g10080	0,67	0,69	0,89	0,95	0,95	0,97	0,93	0,98 expressed protein
264516_at	At1g10090	0,62	0,78	0,82	0,90	0,94	0,91	0,93	0,96 expressed protein
257417_at	At1g10110	1,12	0,94	1,02	1,07	0,95	1,00	1,06	0,97 F-box family protein
264517_at	At1g10120	1,27	1,43	1,62	1,19	0,92	1,29	1,45	1,21 basic helix-loop-helix (bHLH) family protein
264526_at	At1g10130	0,96	0,85	0,63	0,94	1,03	0,79	0,86	0,88 calcium-transporting ATPase 3, endoplasmic reticulum-type (ACA6) (ECA3
264467_at	At1g10140	0,90	0,88	1,11	0,99	0,96	0,85	0,99	1,27 expressed protein
264463_at	At1g10150	1,06	1,05	1,05	1,02	1,21	1,17	1,08	1,04 expressed protein
264459_at	At1g10160	0,93	0,98	0,99	0,98	1,09	0,82	0,96	0,97 non-LTR retrotransposon family (LINE)
264460_at	At1g10170	0,78	1,02	1,20	0,68	0,88	1,28	1,05	1,13 NF-X1 type zinc finger family protein
264407_at	At1g10180	1,14	1,16	1,24	0,95	1,17	0,89	0,92	0,76 expressed protein
264462_at	At1g10200	0,94	0,96	1,01	1,12	1,02	0,86	0,89	0,94 transcription factor LIM, putative
264405_at	At1g10210	1,06	1,09	1,34	1,17	1,14	1,04	1,02	1,31 mitogen-activated protein kinase, putative / MAPK, putative (MPK1)
264465_at	At1g10230	1,24	1,09	0,91	1,18	1,56	0,93	0,81	0,70 E3 ubiquitin ligase SCF complex subunit SKP1/ASK1 (At18), putative
264408_at	At1g10240	0,97	1,01	1,11	0,86	0,95	1,11	0,97	1,00 far-red impaired responsive protein, putative
264450_s_at	At1g10250	0,82	0,81	0,97	0,90	0,90	1,04	1,08	0,96 expressed protein
264451_s_at	At1g10260	0,94	0,98	0,90	0,96	0,93	1,05	0,97	1,14 ---
264452_at	At1g10270	1,00	0,87	0,76	1,01	1,12	1,00	0,81	0,86 pentatricopeptide (PPR) repeat-containing protein
264406_at	At1g10290	0,99	0,86	0,96	1,09	1,26	0,91	0,86	0,85 dynamin-like protein 6 (ADL6)
264453_at	At1g10300	1,01	0,99	0,94	1,03	1,17	0,95	0,99	0,90 GTP-binding protein-related
264468_at	At1g10310	1,00	0,98	0,92	0,88	0,99	1,11	1,02	1,06 short-chain dehydrogenase/reductase (SDR) family protein
264454_at	At1g10320	0,95	1,03	0,94	0,73	0,99	1,40	1,21	1,19 U2 snRNP auxiliary factor-related
264455_at	At1g10330	0,94	1,08	0,94	1,13	1,09	1,09	1,07	0,97 pentatricopeptide (PPR) repeat-containing protein
264434_at	At1g10340	0,72	0,96	1,34	1,15	1,07	1,05	1,05	0,89 ankyrin repeat family protein
264464_at	At1g10350	0,72	0,93	0,88	1,05	1,16	0,83	0,94	0,90 DNAJ heat shock protein, putative
264435_at	At1g10360	0,90	0,93	1,13	0,93	0,92	1,05	1,09	1,10 glutathione S-transferase, putative
264436_at	At1g10370	1,42	1,12	1,14	1,48	1,37	1,49	1,11	0,96 glutathione S-transferase, putative (ERD9)
264466_at	At1g10380	1,21	1,35	1,21	1,33	1,33	0,97	0,91	0,82 expressed protein

264456_at	At1g10390	0,90	0,85	0,96	0,89	0,85	0,95	1,09	0,95	nucleoporin family protein
264457_at	At1g10400	0,83	0,87	1,07	1,05	1,17	1,13	0,97	1,44	UDP-glucoronosyl/UDP-glucosyl transferase family protein
264458_at	At1g10410	0,98	0,87	1,24	1,15	1,03	1,10	1,15	1,44	expressed protein
263234_at	At1g10420	0,99	1,03	1,04	1,06	0,93	0,98	0,92	1,06	hypothetical protein
263235_at	At1g10430	1,11	0,88	1,14	1,03	1,20	1,03	0,85	1,10	serine/threonine protein phosphatase PP2A-1 catalytic subunit (PP2A1)
263211_at	At1g10460	0,95	1,12	1,07	0,87	1,11	0,88	0,93	0,94	germin-like protein (GLP7)
263236_at	At1g10470	1,03	1,07	1,20	0,70	0,85	1,31	1,37	1,26	two-component responsive regulator / response regulator 4 (ARR4)
263208_at	At1g10480	0,98	1,07	0,96	1,61	1,32	0,91	1,35	1,21	zinc finger (C2H2 type) family protein
263255_at	At1g10490	1,13	1,21	1,21	0,84	0,82	0,92	0,96	1,00	expressed protein
263256_at	At1g10500	0,89	0,80	0,80	0,80	0,87	1,09	0,99	1,04	hesB-like domain-containing protein
263212_at	At1g10510	1,03	0,87	0,73	0,69	0,73	1,04	1,13	1,08	leucine-rich repeat family protein
263257_at	At1g10520	0,97	1,02	1,14	1,15	1,05	1,04	0,93	1,01	DNA polymerase lambda (POLL)
263209_at	At1g10522	1,37	1,19	1,09	0,83	0,79	0,94	0,88	0,95	expressed protein
257450_at	At1g10530	1,02	1,06	1,10	0,85	0,99	1,00	0,98	0,89	expressed protein
263258_at	At1g10540	0,80	0,88	0,79	1,17	1,10	1,06	0,97	1,09	xanthine/uracil permease family protein
263207_at	At1g10550	1,11	0,94	0,65	0,82	0,74	0,78	0,67	0,61	xyloglucan:xyloglucosyl transferase, putative / xyloglucan endotransglycosy
263259_at	At1g10560	0,91	1,00	0,90	1,01	1,10	0,99	0,88	0,88	armadillo/beta-catenin repeat family protein / U-box domain-containing prot
263260_at	At1g10570	0,89	0,50	0,57	0,77	0,77	0,93	1,18	1,28	Ulp1 protease family protein
263261_at	At1g10580	0,92	0,83	0,97	0,85	0,92	1,01	0,87	1,02	transducin family protein / WD-40 repeat family protein
263210_at	At1g10585	0,47	0,57	0,96	0,69	0,79	1,45	0,96	0,96	expressed protein
263206_at	At1g10590	1,10	0,93	0,90	0,99	1,05	0,96	0,96	0,90	DNA-binding protein-related
263237_at	At1g10610	0,75	0,70	0,80	0,74	0,78	0,91	0,82	1,12	basic helix-loop-helix (bHLH) family protein
261830_at	At1g10620	1,06	1,01	1,02	1,06	1,18	0,95	1,03	1,00	protein kinase family protein
261831_at	At1g10630	1,02	0,84	0,82	1,33	1,34	0,84	0,83	0,87	ADP-ribosylation factor, putative
261834_at	At1g10640	0,58	0,97	0,99	0,89	0,96	1,23	1,19	1,05	polygalacturonase, putative / pectinase, putative
261832_at	At1g10650	0,86	1,02	1,05	1,41	1,01	1,02	1,35	1,31	expressed protein
257477_at	At1g10660	0,92	0,87	0,81	0,95	0,83	1,06	0,98	0,83	expressed protein
261833_at	At1g10670	0,90	0,85	0,82	1,11	1,03	0,87	0,90	0,89	expressed protein
261829_at	At1g10680	1,08	1,09	0,96	0,92	1,09	1,01	1,04	0,93	P-glycoprotein, putative
262788_at	At1g10690	0,91	1,02	1,02	0,98	0,92	0,98	0,93	1,13	expressed protein
262762_at	At1g10700	0,70	0,71	0,75	0,83	0,89	0,82	0,78	0,84	ribose-phosphate pyrophosphokinase 3 / phosphoribosyl diphosphate synth
262792_at	At1g10710	1,05	1,01	1,03	1,02	0,93	0,86	0,86	1,07	hypothetical protein
262761_at	At1g10720	0,89	0,75	0,85	1,05	1,00	1,06	1,04	1,02	BSD domain-containing protein
262787_at	At1g10730	0,97	0,86	1,14	1,00	0,93	1,03	1,06	1,29	clathrin adaptor complexes medium subunit family protein
262786_at	At1g10740	1,42	1,39	1,61	1,24	1,18	1,14	1,09	1,68	expressed protein
262785_at	At1g10750	1,16	1,17	0,99	0,90	1,12	1,07	0,96	0,85	expressed protein
262784_at	At1g10760	0,51	0,55	0,63	0,65	0,62	0,97	1,01	1,10	starch excess protein (SEX1)
262760_at	At1g10770	1,10	0,95	0,97	1,02	0,99	1,12	1,10	1,01	invertase/pectin methylesterase inhibitor family protein
262758_at	At1g10780	1,15	1,13	1,00	1,05	1,26	0,99	0,76	0,86	F-box family protein
262759_at	At1g10800	0,98	0,99	1,23	1,01	1,08	1,00	1,01	1,11	hypothetical protein
262789_at	At1g10810	1,12	0,98	1,14	1,03	1,28	0,95	0,95	1,12	aldo/keto reductase family protein
262791_at	At1g10830	0,74	0,69	0,75	0,84	0,87	0,97	0,86	0,85	sodium symporter-related
262790_at	At1g10840	1,11	0,97	1,04	1,01	0,97	0,96	0,99	0,97	eukaryotic translation initiation factor 3 subunit 3 / eIF-3 gamma / eIF3h (TIF
262783_at	At1g10850	1,11	1,10	1,07	0,99	1,50	0,76	0,75	0,78	pseudogene, leucine-rich repeat transmembrane protein kinase, putative
260469_at	At1g10870	0,97	0,93	0,93	1,08	0,98	0,87	0,96	1,00	ARF GTPase-activating domain-containing protein
260473_at	At1g10880	0,96	1,04	1,16	1,15	0,99	1,16	0,93	1,03	expressed protein

260467_at	At1g10890	0,88	0,94	1,02	0,95	1,11	0,87	0,99	1,10	F-box family protein
260466_at	At1g10900	0,93	1,05	1,33	1,11	1,17	1,12	1,34	1,35	phosphatidylinositol-4-phosphate 5-kinase family protein
260465_at	At1g10910	0,93	0,87	0,84	0,70	0,56	1,07	0,93	1,10	pentatricopeptide (PPR) repeat-containing protein
260464_at	At1g10920	0,88	0,99	1,12	0,74	0,96	1,11	1,02	1,05	disease resistance protein (CC-NBS-LRR class), putative
260463_at	At1g10930	0,98	1,03	0,93	0,97	0,84	1,11	0,97	0,81	DNA helicase (RECQl4A)
260480_at	At1g10940	0,74	0,63	0,60	1,05	0,91	0,87	0,92	1,00	serine/threonine protein kinase, putative
260482_at	At1g10950	1,08	0,87	0,80	1,30	1,06	0,84	0,77	0,79	endomembrane protein 70, putative
260481_at	At1g10960	1,21	1,17	1,14	1,34	1,26	0,94	0,85	0,78	ferredoxin, chloroplast, putative
260462_at	At1g10970	0,80	0,87	0,71	1,34	1,35	0,73	0,74	0,72	metal transporter, putative (ZIP4)
260461_at	At1g10980	1,00	1,13	0,91	0,95	1,04	0,96	0,90	0,84	expressed protein
260472_at	At1g10990	0,75	0,86	0,78	0,93	1,09	0,61	0,59	0,57	expressed protein
260483_at	At1g11000	0,88	0,96	1,09	1,01	0,87	1,00	1,36	1,40	seven transmembrane MLO family protein / MLO-like protein 4 (MLO4)
260479_at	At1g11020	0,82	0,79	0,87	1,13	0,96	0,94	0,93	0,83	zinc finger (C3HC4-type RING finger) family protein
260478_at	At1g11040	0,89	0,89	1,00	0,94	1,05	1,02	0,88	0,90	DNAJ chaperone C-terminal domain-containing protein
260477_at	At1g11050	0,74	0,93	0,90	0,77	0,65	1,05	1,10	1,08	protein kinase family protein
260476_at	At1g11060	0,93	1,07	1,17	1,09	0,84	1,09	1,00	1,18	expressed protein
260471_at	At1g11070	0,85	1,00	1,04	0,86	0,93	1,15	1,03	1,02	hydroxyproline-rich glycoprotein family protein
260475_at	At1g11080	1,38	1,63	2,11	0,79	1,04	1,26	1,24	1,26	serine carboxypeptidase S10 family protein
260474_at	At1g11090	0,79	0,93	1,07	0,97	0,92	0,93	1,17	0,93	hydrolase, alpha/beta fold family protein
260468_at	At1g11100	1,03	0,97	1,07	0,92	1,12	1,03	1,05	1,21	SNF2 domain-containing protein / helicase domain-containing protein / zinc
260470_at	At1g11120	1,06	1,06	1,12	0,99	0,96	0,90	0,92	1,04	expressed protein
262479_at	At1g11130	0,90	0,89	0,92	0,94	1,00	0,90	0,96	1,14	leucine-rich repeat family protein / protein kinase family protein
262451_at	At1g11140	0,98	1,19	1,22	0,85	0,85	0,97	1,18	1,12	leucine-rich repeat family protein / protein kinase family protein
262449_at	At1g11160	0,95	0,98	0,96	0,94	0,99	1,08	0,98	0,97	WD-40 repeat family protein / katanin p80 subunit, putative
262478_at	At1g11170	0,70	0,83	0,93	0,73	1,00	1,10	1,13	1,03	expressed protein
262454_at	At1g11190	1,22	1,32	1,32	1,35	1,36	0,96	1,24	1,54	bifunctional nuclease (BFN1)
262457_at	At1g11200	0,84	0,70	0,78	1,14	1,07	0,89	0,84	0,76	expressed protein
262452_at	At1g11210	0,78	1,21	0,98	0,58	0,86	0,81	1,27	0,80	expressed protein
262477_at	At1g11220	0,97	1,06	1,21	1,05	1,39	0,90	0,94	0,94	expressed protein
262453_at	At1g11240	0,99	1,01	1,01	0,93	1,09	1,07	1,11	1,08	expressed protein
262511_at	At1g11250	0,92	1,09	1,17	0,93	1,10	1,07	0,95	1,05	syntaxin, putative (SYP125)
262456_at	At1g11260	1,05	1,09	1,30	0,89	0,78	1,06	1,04	1,14	glucose transporter (STP1)
262510_at	At1g11270	1,37	1,26	1,43	1,00	1,09	0,98	0,97	1,21	F-box family protein
262458_at	At1g11280	0,93	0,90	1,05	0,84	1,04	1,13	0,98	1,00	S-locus protein kinase, putative
262509_at	At1g11290	0,73	0,84	1,13	0,57	0,68	0,95	0,96	0,99	pentatricopeptide (PPR) repeat-containing protein
262508_at	At1g11300	0,71	0,77	0,69	0,86	0,96	0,89	0,90	0,87	S-locus lectin protein kinase family protein
262455_at	At1g11310	0,76	1,03	1,34	0,75	0,78	1,07	1,23	1,51	seven transmembrane MLO family protein / MLO-like protein 2 (MLO2)
262450_at	At1g11320	0,91	1,04	1,09	0,81	1,07	0,95	0,93	0,91	expressed protein
262507_at	At1g11330	0,80	0,70	0,74	0,73	0,65	0,86	1,04	0,98	S-locus lectin protein kinase family protein
262480_at	At1g11340	0,82	0,93	0,98	0,76	0,84	1,01	0,94	1,10	S-locus lectin protein kinase family protein
261873_at	At1g11350	1,05	1,01	1,30	0,43	0,50	1,15	1,23	1,39	S-locus lectin protein kinase family protein
261828_at	At1g11360	0,69	0,68	0,74	0,87	0,91	0,87	0,75	0,81	universal stress protein (USP) family protein
261849_at	At1g11370	0,82	0,90	0,94	0,89	1,03	1,02	0,99	0,94	pectinesterase family protein
261822_at	At1g11380	1,41	1,34	1,39	0,88	1,06	0,79	1,25	0,94	expressed protein
261818_at	At1g11390	0,92	0,80	1,22	0,83	1,05	1,03	0,92	0,78	ABC1 family protein
261823_at	At1g11400	1,20	1,02	1,18	1,06	1,58	1,15	1,07	1,18	expressed protein

261819_at	At1g11410	0,75	0,77	1,07	0,82	0,70	1,11	1,04	1,34	S-locus protein kinase, putative
261820_at	At1g11420	1,04	1,13	1,14	0,99	1,02	0,91	1,02	0,86	agenet domain-containing protein
261824_at	At1g11430	0,95	0,93	0,91	0,93	0,86	0,95	0,96	0,87	plastid developmental protein DAG, putative
261871_at	At1g11440	0,93	1,02	1,01	1,08	0,77	0,75	0,89	1,01	expressed protein
261868_s_at	At1g11460	0,95	1,16	0,87	1,24	0,94	1,23	1,41	1,07	nodulin MtN21 family protein
261869_at	At1g11470	0,98	1,06	1,15	0,93	1,00	1,03	0,98	1,29	hypothetical protein
261827_at	At1g11480	1,04	1,18	1,26	1,01	1,30	1,04	0,91	1,21	eukaryotic translation initiation factor-related
261872_s_at	At1g11520	0,70	0,64	0,59	1,00	1,09	0,93	0,84	0,92	pliceosome associated protein-related
261821_at	At1g11530	0,75	0,81	0,82	0,70	0,66	1,02	0,88	0,95	thioredoxin family protein
261846_at	At1g11540	1,01	1,11	1,01	0,87	1,05	0,99	1,08	0,96	expressed protein
261825_at	At1g11545	1,00	0,85	0,78	0,98	1,00	0,68	0,63	0,56	xyloglucan:xyloglucosyl transferase, putative / xyloglucan endotransglycosy
261847_at	At1g11560	1,07	0,89	0,87	1,01	1,07	1,01	1,04	1,02	OST3/OST6 family protein
261870_at	At1g11570	1,15	1,16	1,05	1,28	1,24	1,00	0,89	1,01	nuclear transport factor 2 (NTF2), putative
261826_at	At1g11580	1,03	0,77	0,76	1,66	1,21	0,88	0,91	0,90	pectin methylesterase, putative
261848_at	At1g11590	1,04	0,95	1,00	0,99	1,03	0,93	0,96	1,07	pectin methylesterase, putative
262819_at	At1g11600	1,14	1,28	1,07	1,38	1,41	0,93	1,07	0,93	cytochrome P450, putative
262815_at	At1g11610	1,03	1,04	1,12	0,97	1,04	0,96	0,98	1,00	cytochrome P450, putative
262822_at	At1g11630	0,80	0,82	0,99	0,91	0,80	0,94	1,03	0,81	pentatricopeptide (PPR) repeat-containing protein
262824_at	At1g11650	0,96	0,92	0,97	0,95	0,91	0,96	0,96	1,00	RNA-binding protein 45 (RBP45), putative
262814_at	At1g11660	1,37	0,95	1,09	1,00	1,23	1,05	1,06	0,97	heat shock protein, putative
262813_at	At1g11670	0,58	0,87	0,76	0,87	0,88	0,90	1,37	1,28	MATE efflux family protein
262820_at	At1g11680	0,97	0,96	0,82	1,21	1,19	0,88	0,80	0,74	obtusifoliol 14-demethylase (CYP51)
262812_at	At1g11690	0,96	0,88	0,95	1,01	0,94	1,03	1,06	1,05	hypothetical protein
262811_at	At1g11700	0,70	0,75	0,94	0,88	0,76	0,90	1,04	0,84	expressed protein
262810_at	At1g11710	1,05	1,07	1,03	0,99	0,98	0,95	0,86	0,80	pentatricopeptide (PPR) repeat-containing protein
262809_at	At1g11720	0,89	0,85	0,85	0,68	0,64	1,10	1,13	1,04	starch synthase, putative
262808_at	At1g11730	0,88	1,04	0,86	1,13	1,00	0,85	0,91	0,96	galactosyltransferase family protein
262807_at	At1g11740	0,96	1,04	0,98	1,03	0,95	0,79	0,94	1,15	ankyrin repeat family protein
262823_at	At1g11750	0,97	0,95	0,84	0,97	0,89	0,93	0,96	0,99	ATP-dependent Clp protease proteolytic subunit (ClpP)
262818_at	At1g11760	0,87	1,11	1,06	1,03	1,00	0,96	0,99	0,74	expressed protein
262817_at	At1g11770	0,90	0,97	1,02	0,99	0,98	0,95	0,91	1,13	FAD-binding domain-containing protein
262816_at	At1g11780	0,86	0,73	0,82	1,09	0,86	0,90	0,75	0,72	oxidoreductase, 2OG-Fe(II) oxygenase family protein
262825_at	At1g11790	0,65	0,74	1,08	0,99	0,95	0,96	0,92	0,91	prephenate dehydratase family protein
262821_at	At1g11800	1,03	0,97	0,87	0,94	1,11	0,89	1,00	1,08	endonuclease/exonuclease/phosphatase family protein
264397_at	At1g11820	1,08	0,94	0,88	0,77	0,89	0,84	0,90	0,85	glycosyl hydrolase family 17 protein
264372_at	At1g11840	1,00	0,94	0,82	1,31	1,15	0,94	0,99	1,04	lactoylglutathione lyase, putative / glyoxalase I, putative
264343_at	At1g11850	1,14	1,16	1,10	1,46	1,32	0,78	0,93	1,05	expressed protein
264394_at	At1g11860	1,07	1,01	0,88	1,10	0,75	1,17	1,07	1,03	aminomethyltransferase, putative
264350_at	At1g11870	0,75	0,71	0,65	0,89	0,81	1,04	0,92	1,02	seryl-tRNA synthetase, putative / serine--tRNA ligase, putative
264393_at	At1g11880	0,90	0,92	1,02	1,07	0,77	0,97	1,03	0,63	expressed protein
264373_at	At1g11890	1,08	0,86	0,97	1,19	1,15	1,07	1,00	1,10	vesicle transport protein SEC22, putative
264392_at	At1g11900	0,94	0,88	0,89	0,88	0,83	1,01	0,98	1,14	pentatricopeptide (PPR) repeat-containing protein
264344_at	At1g11910	0,87	1,07	1,11	1,27	1,46	0,92	1,01	1,04	aspartyl protease family protein
264345_at	At1g11915	0,97	1,08	1,03	0,98	1,07	0,94	0,94	0,94	expressed protein
264391_at	At1g11920	1,01	0,98	0,97	0,99	1,05	0,93	1,00	0,94	pectate lyase family protein
264349_at	At1g11930	0,70	0,68	0,68	1,03	1,03	1,00	0,95	0,98	alanine racemase family protein

257422_at	At1g11940	0,87	0,75	0,56	0,97	0,94	0,94	0,95	1,01	expressed protein
264390_at	At1g11950	0,85	0,63	1,26	0,82	0,75	0,96	1,09	1,02	transcription factor jumonji (jmjC) domain-containing protein
264389_at	At1g11960	0,63	0,81	1,53	1,04	0,92	1,01	1,37	2,10	early-responsive to dehydration protein-related / ERD protein-related
264388_at	At1g11970	1,01	0,94	1,03	1,03	0,97	1,00	1,09	0,92	hypothetical protein
264387_at	At1g11990	1,04	1,10	1,02	1,11	1,07	0,93	0,97	0,99	expressed protein
264386_at	At1g12000	1,41	1,21	1,01	1,46	1,38	0,87	0,88	0,82	pyrophosphate--fructose-6-phosphate 1-phosphotransferase beta subunit, p
264346_at	At1g12010	1,71	1,33	1,73	2,00	2,28	0,85	1,00	1,00	1-aminocyclopropane-1-carboxylate oxidase, putative / ACC oxidase, putati
264385_at	At1g12020	1,07	0,84	0,82	0,96	1,03	1,03	0,97	1,06	expressed protein
257421_at	At1g12030	1,13	1,11	1,01	0,83	0,83	1,76	1,10	1,11	expressed protein
264347_at	At1g12040	1,04	1,07	0,90	1,05	0,97	1,02	1,00	0,92	leucine-rich repeat family protein / extensin family protein (LRX1)
264396_at	At1g12050	0,99	1,08	1,10	1,14	1,16	1,02	1,04	1,00	fumarylacetoacetase, putative
264395_at	At1g12070	1,10	1,20	0,97	1,45	1,35	0,95	1,10	1,22	Rho GDP-dissociation inhibitor family protein
264342_at	At1g12080	3,38	3,06	2,35	8,64	10,27	0,69	0,62	0,58	expressed protein
264371_at	At1g12090	0,98	0,98	0,93	0,93	0,98	0,96	0,83	0,82	protease inhibitor/seed storage/lipid transfer protein (LTP) family protein
264370_at	At1g12100	0,98	0,94	1,05	1,11	0,96	0,95	0,92	0,84	protease inhibitor/seed storage/lipid transfer protein (LTP) family protein
264348_at	At1g12110	0,80	0,90	0,79	1,01	0,93	0,78	0,66	0,61	nitrate/chlorate transporter (NRT1.1) (CHL1)
260995_at	At1g12120	0,95	0,96	1,01	0,80	0,89	1,19	1,25	1,91	expressed protein
260994_at	At1g12130	0,55	0,59	0,29	0,94	0,97	0,98	0,94	1,11	flavin-containing monooxygenase family protein / FMO family protein
260993_at	At1g12140	0,94	0,90	1,11	1,09	1,17	1,17	1,13	1,03	flavin-containing monooxygenase family protein / FMO family protein
260992_at	At1g12150	0,98	0,88	0,92	0,91	0,99	0,93	1,03	0,93	expressed protein
260991_at	At1g12160	0,87	1,03	0,86	1,09	1,20	0,93	0,96	1,35	flavin-containing monooxygenase family protein / FMO family protein
260990_at	At1g12180	0,99	1,00	0,96	0,98	0,92	1,05	0,93	1,01	expressed protein
261024_at	At1g12190	1,00	0,99	0,86	1,03	1,23	0,96	1,00	1,02	F-box family protein
261023_at	At1g12200	1,19	1,50	1,27	0,68	0,82	1,11	1,15	1,34	flavin-containing monooxygenase family protein / FMO family protein
260996_at	At1g12210	0,92	1,03	1,13	0,87	0,93	0,97	1,11	1,10	disease resistance protein (CC-NBS-LRR class), putative
260966_at	At1g12220	0,97	1,12	0,90	0,76	0,92	1,08	0,96	1,09	disease resistance protein RPS5 (CC-NBS-LRR class) / resistance to Pseu
260967_at	At1g12230	0,91	0,83	0,78	0,97	1,02	0,89	0,94	0,88	transaldolase, putative
260969_at	At1g12240	2,17	2,07	2,19	0,84	0,78	1,22	1,18	1,11	beta-fructosidase (BFRUCT4) / beta-fructofuranosidase / invertase, vacuola
260968_at	At1g12250	0,90	0,94	0,95	0,74	0,67	1,39	1,28	1,30	thylakoid lumenal protein-related
261022_at	At1g12260	1,05	1,08	0,94	0,96	1,24	1,15	0,92	0,91	no apical meristem (NAM) family protein
259519_at	At1g12270	1,36	1,08	1,10	1,14	1,24	0,99	1,03	1,04	stress-inducible protein, putative
259535_at	At1g12280	0,89	0,78	0,96	0,77	1,23	1,02	0,95	0,94	disease resistance protein (CC-NBS-LRR class), putative
259534_at	At1g12290	0,75	0,72	0,82	0,79	1,07	0,90	1,01	0,75	disease resistance protein (CC-NBS-LRR class), putative
259538_at	At1g12310	1,25	1,13	1,08	1,54	1,50	0,90	0,89	0,92	calmodulin, putative
259520_at	At1g12320	0,93	0,97	1,03	1,04	0,99	1,11	0,97	1,12	expressed protein
259528_at	At1g12330	0,93	0,90	1,02	0,90	0,82	0,91	0,79	0,95	expressed protein
257513_s_at	At1g12340	1,16	0,96	0,95	1,19	1,12	1,02	0,94	0,89	cornichon family protein
259512_at	At1g12360	0,86	0,79	0,87	1,05	0,98	1,02	1,12	1,28	cytokinesis-related Sec1 protein (KEULE)
259537_at	At1g12370	1,34	1,35	1,14	1,00	1,05	1,14	1,04	1,07	type II CPD photolyase PHR1 (PHR1)
259529_at	At1g12400	0,96	0,80	0,89	0,99	1,11	1,01	0,87	0,74	expressed protein
259521_at	At1g12410	0,96	0,84	0,80	0,93	0,98	1,02	0,97	1,00	ATP-dependent Clp protease proteolytic subunit (ClpP2)
259513_at	At1g12430	0,76	0,83	0,82	0,81	0,86	0,89	0,93	1,00	armadillo/beta-catenin repeat family protein / kinesin motor family protein
259530_at	At1g12450	1,01	1,21	1,02	1,27	0,96	0,98	1,12	1,26	expressed protein
259531_at	At1g12460	0,94	1,05	0,83	0,86	1,12	0,90	0,90	0,72	leucine-rich repeat transmembrane protein kinase, putative
259532_at	At1g12470	0,86	0,76	0,87	1,06	0,89	0,82	0,90	0,94	Pep3/Vps18/deep orange family protein
259514_at	At1g12480	1,04	1,17	1,35	0,76	1,02	1,20	1,05	1,48	C4-dicarboxylate transporter/malic acid transport family protein

259522_at	At1g12490	0,98	0,96	1,10	0,98	1,01	1,08	0,99	0,92	F-box family protein-related
259523_at	At1g12500	0,88	0,88	0,78	0,99	1,00	0,85	0,79	0,87	phosphate translocator-related
259511_at	At1g12520	0,86	0,95	0,89	0,51	0,54	1,14	1,16	1,41	superoxide dismutase copper chaperone, putative
259533_at	At1g12530	1,34	1,10	1,24	0,95	1,17	1,08	1,21	1,24	hypothetical protein
259524_at	At1g12550	0,86	0,91	0,96	1,25	1,06	0,93	0,91	0,85	oxidoreductase family protein
259525_at	At1g12560	0,90	1,13	0,84	1,01	0,85	0,86	1,05	1,10	expansin, putative (EXP7)
259526_at	At1g12570	0,99	1,03	0,91	0,99	0,97	1,06	1,01	1,14	glucose-methanol-choline (GMC) oxidoreductase family protein
259484_at	At1g12580	0,87	0,98	1,34	0,96	0,89	0,94	1,19	1,38	protein kinase family protein
259527_at	At1g12600	0,88	0,87	1,21	1,52	1,16	1,05	1,03	1,17	hypothetical protein
255937_at	At1g12610	1,07	1,66	1,40	0,78	0,73	1,12	2,02	1,84	DRE-binding protein, putative / CRT/DRE-binding factor, putative
255938_at	At1g12620	0,90	0,89	0,93	0,92	0,80	0,85	1,03	1,08	pentatricopeptide (PPR) repeat-containing protein
255927_at	At1g12630	0,98	1,19	0,98	1,04	1,15	0,98	1,15	1,07	AP2 domain-containing protein
255928_at	At1g12640	1,00	0,94	0,95	1,09	0,95	0,84	0,88	0,85	membrane bound O-acyl transferase (MBOAT) family protein
255929_at	At1g12650	0,98	0,92	0,92	1,00	1,17	1,21	1,08	0,94	expressed protein
255930_at	At1g12660	1,01	1,04	0,96	0,88	1,00	1,10	1,11	1,16	hypothetical protein
255936_at	At1g12680	1,11	1,14	1,08	0,91	0,95	1,16	1,38	1,36	protein kinase family protein
255935_at	At1g12700	1,00	0,64	1,02	0,93	1,02	1,16	1,00	0,87	helicase domain-containing protein / pentatricopeptide (PPR) repeat-contai
255931_at	At1g12710	0,88	1,20	1,18	0,79	1,21	0,86	1,46	0,94	F-box family protein / SKP1 interacting partner 3-related
255932_at	At1g12720	0,91	0,97	0,99	0,92	0,93	0,95	1,09	1,06	---
255939_at	At1g12730	1,07	1,21	1,24	0,63	0,59	1,08	1,06	0,93	cell division cycle protein-related
255934_at	At1g12740	1,06	0,99	1,06	1,15	1,03	0,97	1,08	1,07	cytochrome P450 family protein
255933_at	At1g12750	0,65	0,63	0,61	0,83	1,00	0,73	0,71	0,77	rhomboid family protein
261194_at	At1g12770	1,03	0,88	1,01	1,07	0,89	0,85	0,87	1,01	DEAD/DEAH box helicase family protein / pentatricopeptide (PPR) repeat-c
261211_at	At1g12780	1,00	1,03	1,28	0,88	0,89	1,04	1,08	1,15	UDP-glucose 4-epimerase / UDP-galactose 4-epimerase / Galactowaldena
261205_at	At1g12790	1,01	0,98	1,01	0,72	0,87	0,69	0,69	0,70	expressed protein
261206_at	At1g12800	1,07	1,13	1,06	0,90	0,89	1,28	1,33	1,42	S1 RNA-binding domain-containing protein
261209_at	At1g12810	1,19	1,17	1,01	1,16	1,31	0,97	0,87	0,90	proline-rich family protein
261195_at	At1g12820	1,16	0,92	0,83	0,91	1,47	0,98	0,79	0,97	transport inhibitor response protein, putative
261207_at	At1g12830	1,05	1,19	1,22	1,21	1,53	1,01	1,00	1,03	expressed protein
261210_at	At1g12840	0,85	0,86	0,80	0,91	1,05	0,85	0,85	0,86	vacuolar ATP synthase subunit C (VATC) / V-ATPase C subunit / vacuolar
261203_at	At1g12845	0,87	0,94	0,73	0,70	0,74	0,63	0,56	0,57	expressed protein
261201_at	At1g12850	1,00	0,86	0,69	1,08	0,99	0,88	0,72	0,75	phosphoglycerate/bisphosphoglycerate mutase family protein
261196_at	At1g12860	1,28	1,27	1,21	0,87	0,95	1,05	0,84	1,05	basic helix-loop-helix (bHLH) family protein / F-box family protein
261212_at	At1g12880	1,23	1,12	1,49	0,91	0,80	1,25	1,71	1,75	MutT/nudix family protein
261197_at	At1g12900	1,05	1,04	1,16	0,82	0,88	1,17	1,06	1,11	glyceraldehyde 3-phosphate dehydrogenase, chloroplast, putative / NADP-
261202_at	At1g12910	0,94	0,91	1,22	0,86	1,02	1,00	0,91	1,03	flower pigmentation protein (AN11)
261208_at	At1g12930	1,17	1,18	1,37	0,86	0,90	0,99	1,05	0,92	importin-related
261198_at	At1g12940	0,99	1,11	1,04	1,06	1,24	1,00	0,89	0,96	high-affinity nitrate transporter, putative
261199_at	At1g12950	0,99	1,14	1,27	1,13	1,03	1,19	1,66	2,19	MATE efflux family protein
261200_at	At1g12960	0,96	1,00	1,04	1,03	0,89	0,94	1,00	1,05	60S ribosomal protein L27A (RPL27aA)
261213_at	At1g12970	1,06	0,93	1,20	1,08	0,92	1,16	0,90	0,93	leucine-rich repeat family protein
262768_at	At1g12990	0,81	1,01	0,92	0,96	0,75	0,93	0,96	1,09	glycosyl transferase family 17 protein
262775_at	At1g13000	0,75	0,72	0,70	1,12	0,87	0,82	0,83	0,98	expressed protein
262776_at	At1g13020	0,89	1,00	1,01	0,96	1,05	0,94	0,87	0,83	eukaryotic translation initiation factor, putative (EIF4B5)
262777_at	At1g13030	1,16	1,02	1,17	0,98	0,91	0,98	0,94	1,15	sphere organelles protein-related
257465_at	At1g13040	1,08	1,05	1,18	0,93	1,01	1,00	0,94	1,13	pentatricopeptide (PPR) repeat-containing protein

262778_at	At1g13050	1,02	0,94	0,94	1,01	1,01	1,03	0,85	0,91	expressed protein
262781_s_at	At1g13060	1,02	0,95	0,95	1,13	1,05	0,92	0,98	1,01	20S proteasome beta subunit E, putative
262779_at	At1g13070	0,99	1,02	1,63	0,96	1,05	1,17	1,08	1,39	---
262826_at	At1g13080	1,06	1,12	0,97	1,09	0,80	1,58	1,53	1,42	cytochrome P450 family protein
262780_at	At1g13090	1,22	1,03	1,30	0,91	0,97	1,10	1,16	1,23	cytochrome P450 71B28, putative (CYP71B28)
262827_at	At1g13100	1,15	1,22	0,90	0,81	0,96	1,00	1,20	0,89	cytochrome P450 71B29, putative (CYP71B29)
262793_at	At1g13110	1,38	1,23	1,32	0,72	1,07	1,25	1,21	2,33	cytochrome P450 71B7 (CYP71B7)
262794_at	At1g13120	0,93	0,93	1,00	1,06	0,83	0,98	1,05	1,18	expressed protein
262795_at	At1g13130	0,96	1,07	0,87	1,19	1,31	0,93	1,11	1,24	glycosyl hydrolase family 5 protein / cellulase family protein
262764_at	At1g13140	1,01	0,96	0,93	1,05	0,96	1,02	0,98	1,10	cytochrome P450 family protein
262765_at	At1g13150	1,07	0,92	1,01	1,04	1,02	0,92	1,00	0,93	cytochrome P450, putative
262766_at	At1g13160	0,94	0,88	0,84	0,90	1,11	1,05	0,89	1,01	SDA1 family protein
262767_at	At1g13170	1,01	0,83	0,88	0,96	1,17	1,09	0,88	0,99	oxysterol-binding family protein
262769_at	At1g13180	1,05	1,09	1,09	1,17	1,02	0,88	0,95	1,08	actin-related protein 3 (ARP3)
262770_at	At1g13190	0,65	0,76	0,87	1,09	0,94	0,95	0,88	1,02	RNA recognition motif (RRM)-containing protein
262782_at	At1g13195	0,82	1,10	1,12	0,93	0,98	0,92	1,15	1,34	zinc finger (C3HC4-type RING finger) family protein
262771_at	At1g13200	0,90	0,99	0,84	0,94	1,14	0,95	0,86	1,01	F-box family protein
262772_at	At1g13210	0,85	0,96	1,27	0,81	0,71	1,02	1,26	1,63	haloacid dehalogenase-like hydrolase family protein
262773_at	At1g13220	1,40	0,97	1,13	0,94	0,74	1,37	1,66	1,25	nuclear matrix constituent protein-related
262774_at	At1g13230	0,96	0,99	0,93	0,99	1,02	1,00	1,01	0,84	leucine-rich repeat family protein
259358_at	At1g13250	0,84	1,09	0,81	0,91	1,14	0,63	0,72	0,80	glycosyl transferase family 8 protein
259364_at	At1g13260	0,75	0,88	0,91	0,70	1,00	1,02	1,17	1,30	DNA-binding protein RAV1 (RAV1)
259363_at	At1g13270	0,91	0,73	0,80	0,69	0,65	0,89	0,97	1,00	metallopeptidase M24 family protein
259366_at	At1g13280	0,91	0,82	0,89	1,28	1,36	0,88	0,73	0,76	allene oxide cyclase family protein
257515_at	At1g13290	0,92	1,10	0,99	1,05	1,06	0,96	1,08	1,13	zinc finger (C2H2 type) family protein
259365_at	At1g13300	0,94	1,00	0,84	1,26	0,86	0,98	1,14	1,35	myb family transcription factor
259360_at	At1g13310	0,97	0,92	1,09	1,00	1,02	1,09	1,06	1,21	expressed protein
259407_at	At1g13320	1,06	1,02	0,99	0,99	0,97	0,99	0,88	0,88	serine/threonine protein phosphatase 2A (PP2A) 65 kDa regulatory subunit
259409_at	At1g13330	1,76	1,62	1,83	0,96	0,97	1,21	1,03	0,97	expressed protein
259410_at	At1g13340	0,92	1,10	0,95	0,98	0,92	1,40	1,40	1,52	expressed protein
259362_s_at	At1g13350	1,47	1,74	2,12	0,79	0,60	1,13	1,97	1,98	protein kinase family protein
259387_at	At1g13370	0,95	0,96	1,08	1,04	1,12	1,06	1,05	0,98	histone H3, putative
259357_at	At1g13380	0,98	0,95	0,96	0,95	0,94	0,95	0,92	0,81	expressed protein
259386_at	At1g13400	1,07	1,05	1,05	0,86	1,21	0,91	1,10	0,98	zinc finger (C2H2 type) family protein
259411_at	At1g13410	0,92	1,03	1,00	0,83	0,87	0,93	0,96	1,07	pentatricopeptide (PPR) repeat-containing protein
259388_at	At1g13420	1,10	1,17	0,97	1,25	1,13	0,95	1,02	0,97	sulfotransferase family protein
259361_at	At1g13440	1,04	1,04	1,01	1,31	1,25	0,97	0,92	0,90	glyceraldehyde 3-phosphate dehydrogenase, cytosolic, putative / NAD-depe
259412_at	At1g13450	0,99	1,04	1,01	0,92	1,03	0,94	0,97	1,09	DNA binding protein GT-1
259359_at	At1g13460	1,06	0,86	0,82	0,90	0,87	0,90	0,86	0,78	serine/threonine protein phosphatase 2A (PP2A) regulatory subunit B', puta
259385_at	At1g13470	0,87	0,97	1,01	1,01	1,12	1,17	1,08	1,02	expressed protein
256103_at	At1g13540	0,96	1,00	1,01	1,04	1,03	1,04	0,93	1,03	expressed protein
256134_at	At1g13560	0,69	0,65	0,69	0,78	0,89	0,98	1,02	0,99	aminoalcoholphosphotransferase (AAPT1)
256133_at	At1g13570	1,09	1,20	1,17	0,68	0,94	0,96	1,00	1,04	F-box family protein
256157_at	At1g13580	0,90	0,84	0,75	1,06	0,77	0,88	0,86	0,85	longevity-assurance (LAG1) family protein
256158_at	At1g13590	0,92	1,01	0,76	1,06	1,12	1,11	1,16	1,14	phytosulfokines-related
256131_at	At1g13600	0,96	0,94	1,07	0,95	1,13	0,99	1,01	0,91	bZIP transcription factor family protein

256132_at	At1g13610	0,91	1,08	1,00	0,99	1,12	1,00	1,05	1,09	expressed protein
256095_at	At1g13630	1,06	0,97	1,06	1,00	0,97	0,99	1,06	1,00	pentatricopeptide (PPR) repeat-containing protein
256071_at	At1g13640	1,14	1,07	1,07	0,82	1,00	0,98	0,96	0,98	phosphatidylinositol 3- and 4-kinase family protein
256096_at	At1g13650	1,14	1,05	1,17	0,50	0,53	1,02	1,06	1,08	expressed protein
256097_at	At1g13670	1,22	0,98	1,07	1,39	1,19	0,99	0,77	0,91	expressed protein
256102_at	At1g13680	0,97	0,90	0,95	0,96	1,03	1,04	1,01	0,98	expressed protein
256068_at	At1g13690	1,25	1,31	1,48	1,00	1,13	0,97	1,00	1,04	RNA recognition motif (RRM)-containing protein
256098_at	At1g13700	0,82	1,10	1,27	1,08	0,97	1,17	1,21	1,21	glucosamine/galactosamine-6-phosphate isomerase family protein
256099_at	At1g13710	2,07	1,77	1,64	0,84	0,90	1,03	1,08	0,92	cytochrome P450 family protein
256070_at	At1g13730	1,35	1,28	1,36	0,96	0,95	1,23	1,23	1,10	nuclear transport factor 2 (NTF2) family protein / RNA recognition motif (RR
256069_at	At1g13740	0,92	1,20	1,05	1,03	1,05	0,81	1,01	0,92	expressed protein
256100_at	At1g13750	0,80	0,82	0,67	1,73	1,50	1,52	1,17	1,23	calcineurin-like phosphoesterase family protein
256101_at	At1g13760	0,95	0,91	0,96	0,93	0,98	0,96	0,99	0,79	hypothetical protein
259448_at	At1g13790	0,96	0,61	0,86	0,92	0,79	0,91	1,16	1,26	XH/XS domain-containing protein / XS zinc finger domain-containing protei
259449_at	At1g13800	0,93	0,97	0,94	0,96	0,97	0,94	0,96	1,12	pentatricopeptide (PPR) repeat-containing protein
259422_at	At1g13810	1,31	1,13	1,13	0,80	0,76	1,01	1,04	0,77	expressed protein
259424_at	At1g13830	0,96	1,04	1,08	1,01	0,83	0,93	1,01	1,05	beta-1,3-glucanase-related
259450_at	At1g13870	1,02	0,87	0,96	1,02	0,95	0,96	0,87	0,81	expressed protein
259423_at	At1g13880	0,86	1,08	1,01	0,92	1,00	0,95	1,23	1,16	ELM2 domain-containing protein
259451_at	At1g13890	1,04	0,76	1,03	0,98	0,87	0,94	0,95	1,11	SNAP25 homologous protein, putative / synaptosomal-associated protein S
259420_at	At1g13900	0,97	0,73	0,82	1,10	1,20	1,02	0,78	0,83	calcineurin-like phosphoesterase family protein
259421_at	At1g13910	1,11	1,32	1,20	0,98	0,86	1,07	1,01	1,29	leucine-rich repeat family protein
262662_at	At1g13920	0,96	0,94	0,96	0,98	1,16	0,76	0,92	1,24	remorin family protein
262609_at	At1g13930	0,63	0,54	0,53	1,02	1,05	0,91	0,84	0,83	expressed protein
262663_at	At1g13940	0,87	1,37	0,87	0,66	0,76	1,01	0,95	1,12	expressed protein
262615_at	At1g13950	0,85	0,87	0,65	1,05	1,19	1,04	0,86	0,94	eukaryotic translation initiation factor 5A-1 / eIF-5A 1
262613_at	At1g13960	0,90	0,89	0,79	0,93	1,08	1,13	0,95	1,11	WRKY family transcription factor
262664_at	At1g13970	1,01	1,03	1,00	1,02	1,08	1,10	1,04	1,07	expressed protein
262614_at	At1g13980	1,01	0,96	1,10	1,02	0,86	0,85	0,79	0,85	pattern formation protein (EMB30) (GNOM)
262607_at	At1g13990	0,62	0,61	0,77	0,73	0,76	0,85	0,96	1,00	expressed protein
262660_at	At1g14000	0,89	0,75	0,79	1,06	1,13	1,01	0,92	0,89	protein kinase family protein / ankyrin repeat family protein
262647_at	At1g14020	0,88	0,85	1,08	0,97	1,03	1,01	0,92	0,80	expressed protein
262648_at	At1g14030	0,50	0,36	0,36	0,61	0,63	0,87	0,89	0,77	ribulose-1,5 bisphosphate carboxylase oxygenase large subunit N-methyltr
262649_at	At1g14040	1,32	2,16	1,65	0,86	0,64	1,30	1,46	1,18	EXS family protein / ERD1/XPR1/SYG1 family protein
262611_at	At1g14050	1,04	0,99	0,99	1,16	1,31	0,99	0,92	0,78	expressed protein
262665_at	At1g14070	0,85	0,99	1,13	0,88	1,18	0,95	0,92	0,91	xyloglucan fucosyltransferase, putative (FUT7)
262666_at	At1g14080	0,99	1,05	0,86	1,82	1,44	0,95	0,91	1,07	xyloglucan fucosyltransferase, putative (FUT6)
262650_at	At1g14090	1,03	1,02	0,97	0,90	0,82	1,03	1,12	0,89	pseudogene, pentatricopeptide (PPR) repeat-containing protein
262651_at	At1g14100	0,80	0,79	0,97	0,97	1,15	0,99	0,94	0,96	xyloglucan fucosyltransferase family protein
262652_at	At1g14110	0,96	1,09	1,04	1,02	1,12	0,90	0,90	0,99	---
262608_at	At1g14120	0,83	0,86	0,74	1,01	0,64	0,60	0,90	1,18	2-oxoglutarate-dependent dioxygenase, putative
262653_at	At1g14130	0,64	0,65	1,01	1,00	0,97	0,84	0,93	0,86	2-oxoglutarate-dependent dioxygenase, putative
262610_at	At1g14140	0,67	0,68	0,85	0,97	1,09	0,89	0,77	0,93	mitochondrial substrate carrier family protein
262612_at	At1g14150	0,86	0,84	0,80	0,64	0,56	1,28	1,12	1,23	oxygen evolving enhancer 3 (PsbQ) family protein
262654_at	At1g14180	1,07	1,06	1,05	1,37	1,27	0,84	0,89	1,03	expressed protein
262655_s_at	At1g14190	0,99	1,01	1,01	0,93	0,90	1,05	1,02	1,10	glucose-methanol-choline (GMC) oxidoreductase family protein

262656_at	At1g14200	1,03	1,04	0,96	1,22	1,36	1,08	1,00	0,95 zinc finger (C3HC4-type RING finger) family protein
262657_at	At1g14210	1,14	0,86	0,79	1,85	1,52	0,71	0,75	0,73 ribonuclease T2 family protein
262658_at	At1g14220	0,97	0,84	0,84	1,15	1,22	0,97	0,94	1,02 ribonuclease T2 family protein
262659_at	At1g14240	0,93	0,80	0,86	1,38	1,17	0,90	0,84	0,93 nucleoside phosphatase family protein / GDA1/CD39 family protein
262661_s_at	At1g14250	0,46	0,51	0,37	1,52	0,99	1,01	0,81	0,80 nucleoside phosphatase family protein / GDA1/CD39 family protein
261481_at	At1g14260	0,98	1,01	1,16	0,98	1,29	1,12	1,01	0,83 zinc finger (C3HC4-type RING finger) family protein
261483_at	At1g14270	1,25	1,11	1,01	0,72	0,77	1,15	1,06	1,00 CAAX amino terminal protease family protein
261480_at	At1g14280	1,53	0,94	1,05	1,27	1,22	1,09	0,75	0,87 phytochrome kinase, putative
261492_at	At1g14290	0,88	1,04	1,20	1,29	1,47	0,72	0,65	0,75 acid phosphatase, putative
261524_at	At1g14300	1,45	1,38	1,33	1,04	0,86	0,96	0,87	0,94 expressed protein
261477_at	At1g14310	0,98	0,87	0,81	0,85	0,84	1,01	0,96	0,89 haloacid dehalogenase-like hydrolase family protein
261490_at	At1g14320	1,26	1,13	1,06	1,15	1,04	0,99	0,99	0,90 60S ribosomal protein L10 (RPL10A) / Wilm's tumor suppressor protein-rela
261525_at	At1g14330	0,75	0,69	0,68	1,21	1,19	0,91	0,91	1,13 kelch repeat-containing F-box family protein
261487_at	At1g14340	1,29	1,92	2,45	0,96	0,98	1,44	1,58	1,36 RNA recognition motif (RRM)-containing protein
261488_at	At1g14345	0,88	0,84	1,07	0,93	0,97	1,04	0,94	0,92 expressed protein
261491_at	At1g14350	1,26	1,59	1,39	2,32	2,68	0,82	0,93	1,00 myb family transcription factor (MYB124)
261485_at	At1g14360	1,17	1,13	1,11	1,76	1,40	1,04	1,11	1,19 UDP-galactose/UDP-glucose transporter, putative
261526_at	At1g14370	0,72	1,13	1,16	1,21	1,24	1,03	1,31	1,32 protein kinase (APK2a)
261479_at	At1g14380	0,91	1,00	1,13	1,07	1,19	0,83	1,02	1,21 calmodulin-binding family protein
261527_at	At1g14390	0,92	0,99	1,13	0,98	1,03	0,96	1,04	1,18 leucine-rich repeat transmembrane protein kinase, putative
261484_at	At1g14400	1,00	0,96	0,99	1,18	1,20	0,93	0,97	0,97 ubiquitin-conjugating enzyme 1 (UBC1)
261493_at	At1g14410	1,01	1,01	0,85	0,90	0,81	0,94	0,93	0,90 DNA-binding protein-related
261528_at	At1g14420	0,97	0,96	0,92	0,93	1,11	1,03	0,95	1,11 pectate lyase family protein
261529_at	At1g14430	1,04	1,07	1,13	1,00	1,02	1,09	0,98	1,04 glyoxal oxidase-related
261502_at	At1g14440	0,97	1,01	0,82	0,86	1,13	0,71	0,84	0,84 zinc finger homeobox family protein / ZF-HD homeobox family protein
261489_at	At1g14450	1,55	1,25	1,15	1,41	1,27	1,14	0,91	0,97 expressed protein
261471_at	At1g14460	0,97	1,02	0,90	0,76	0,89	0,88	0,94	1,05 DNA polymerase-related
261472_at	At1g14470	1,04	1,10	1,04	0,98	1,02	1,05	0,94	0,99 pentatricopeptide (PPR) repeat-containing protein
261476_at	At1g14480	1,91	2,08	1,65	1,54	1,25	0,83	1,24	1,07 ankyrin repeat family protein
261473_at	At1g14490	0,95	1,12	1,10	0,85	1,09	1,09	0,94	1,01 DNA-binding protein-related
261486_at	At1g14510	1,02	1,04	1,29	1,27	1,24	0,96	0,89	1,02 PHD finger family protein
261478_at	At1g14520	0,95	1,02	1,15	0,95	1,02	1,08	1,04	1,06 oxygenase-related
261482_at	At1g14530	0,92	0,95	1,04	1,00	0,92	0,93	1,01	1,32 tobamovirus multiplication protein 3, putative / TOM3, putative (THH1)
261474_at	At1g14540	1,27	1,84	1,18	0,86	0,88	2,26	2,13	2,14 anionic peroxidase, putative
261475_at	At1g14550	1,04	1,52	1,05	1,34	1,05	1,28	1,17	1,21 anionic peroxidase, putative
260777_at	At1g14560	1,00	0,81	0,75	0,92	0,79	1,16	1,10	1,07 mitochondrial substrate carrier family protein
260782_at	At1g14570	0,90	0,89	1,19	0,86	0,82	0,99	0,95	1,10 UBX domain-containing protein
260776_at	At1g14580	0,60	0,58	0,48	0,52	0,55	0,82	0,85	0,66 zinc finger (C2H2 type) family protein
260780_at	At1g14610	1,32	1,27	1,25	0,93	0,99	1,17	1,19	1,26 valyl-tRNA synthetase / valine--tRNA ligase (VALRS)
260781_at	At1g14620	1,36	1,23	1,19	1,12	1,05	1,04	0,98	0,89 expressed protein
260778_at	At1g14640	1,25	1,07	1,19	1,00	1,09	1,23	1,20	1,00 SWAP (Suppressor-of-White-APricot)/surp domain-containing protein
260779_at	At1g14650	1,29	1,03	1,27	1,11	1,02	1,01	1,04	1,03 SWAP (Suppressor-of-White-APricot)/surp domain-containing protein / ubiq
262835_at	At1g14660	0,92	0,86	1,10	0,75	0,99	1,06	1,20	1,05 sodium proton exchanger, putative (NHX8)
262846_at	At1g14670	0,99	0,99	0,94	1,36	1,13	0,89	0,79	0,78 endomembrane protein 70, putative
262836_at	At1g14680	1,01	0,83	0,95	1,01	0,86	0,99	1,11	1,05 hypothetical protein
262848_at	At1g14685	1,09	0,88	1,01	0,92	1,10	1,03	0,99	1,10 expressed protein

262843_at	At1g14687	0,84	0,95	0,89	1,09	1,08	0,76	0,92	0,96	zinc finger homeobox family protein / ZF-HD homeobox family protein
262834_at	At1g14690	1,08	1,06	1,04	1,08	0,93	0,93	0,83	1,06	microtubule associated protein (MAP65/ASE1) family protein
262830_at	At1g14700	1,20	1,23	1,55	0,97	1,03	1,07	1,14	1,09	purple acid phosphatase, putative
262849_at	At1g14710	1,23	1,01	1,05	1,04	1,11	1,03	1,02	1,09	hydroxyproline-rich glycoprotein family protein
262842_at	At1g14720	1,00	1,06	1,47	0,92	0,87	0,92	1,02	1,31	xyloglucan:xyloglucosyl transferase / xyloglucan endotransglycosylase / en
262831_at	At1g14730	0,87	0,83	0,73	1,10	0,92	0,98	1,18	1,22	cytochrome B561 family
262845_at	At1g14740	0,84	0,89	1,08	0,79	0,80	0,96	0,91	1,16	expressed protein
262833_at	At1g14750	1,04	1,08	1,04	0,93	0,92	0,89	0,94	0,98	cyclin, putative (SDS)
262886_at	At1g14760	1,04	0,93	0,96	0,92	0,98	0,94	0,90	0,96	expressed protein
262839_at	At1g14770	0,96	0,91	1,07	0,84	0,91	0,91	0,91	1,05	expressed protein
262887_at	At1g14780	0,78	0,75	0,78	0,96	0,70	1,08	1,25	1,28	expressed protein
262888_at	At1g14790	0,98	1,07	1,24	0,91	1,06	1,02	1,06	1,10	RNA-dependent RNA polymerase, putative
262889_at	At1g14800	0,97	1,08	0,96	1,02	1,03	1,04	0,95	1,03	hypothetical protein
262841_at	At1g14810	1,04	0,88	0,91	0,88	0,86	0,91	0,88	0,87	semialdehyde dehydrogenase family protein
257463_at	At1g14820	0,96	0,72	0,81	0,87	1,00	1,16	0,97	1,12	SEC14 cytosolic factor family protein / phosphoglyceride transfer family pro
262837_at	At1g14830	0,92	0,77	0,77	1,26	1,28	0,88	0,88	0,85	dynamin-like protein C (DL1C)
262847_at	At1g14840	1,17	1,09	0,96	0,89	0,92	1,16	1,03	1,10	expressed protein
262890_at	At1g14850	1,17	1,09	1,10	0,90	0,86	0,99	0,96	0,85	non-repetitive/WGA-negative nucleoporin family protein
262832_s_at	At1g14870	0,95	1,13	1,44	1,36	1,33	1,17	1,19	1,40	expressed protein
262844_at	At1g14890	0,98	0,96	1,01	0,74	1,11	1,03	1,29	1,37	invertase/pectin methylesterase inhibitor family protein
262840_at	At1g14900	0,91	1,02	1,06	1,11	1,25	1,03	1,09	1,12	high-mobility-group protein / HMG-I/Y protein
262856_at	At1g14910	0,92	0,94	0,95	0,91	0,92	0,90	0,82	0,91	epsin N-terminal homology (ENTH) domain-containing protein
262850_at	At1g14920	0,86	0,78	0,87	0,90	0,83	0,87	0,74	0,99	gibberellin response modulator (GAI) (RGA2) / gibberellin-responsive modu
262857_at	At1g14930	0,81	0,62	0,42	0,70	1,09	1,05	0,82	1,06	major latex protein-related / MLP-related
262858_at	At1g14940	0,90	0,94	0,73	1,01	0,97	0,92	0,97	0,90	major latex protein-related / MLP-related
262828_at	At1g14950	0,79	0,89	0,81	0,96	0,83	1,03	0,95	0,80	major latex protein-related / MLP-related
262838_at	At1g14960	0,69	0,66	0,42	2,23	1,85	0,93	0,94	1,04	major latex protein-related / MLP-related
262829_at	At1g14970	1,10	1,08	0,96	0,94	0,91	1,16	1,10	0,92	expressed protein
260714_at	At1g14980	1,68	1,44	1,35	1,58	1,61	0,99	0,92	0,91	10 kDa chaperonin (CPN10)
260738_at	At1g14990	0,97	1,02	0,77	1,03	1,08	0,87	0,91	0,85	expressed protein
260739_at	At1g15000	0,52	0,47	0,63	0,71	0,83	0,64	0,63	0,66	serine carboxypeptidase S10 family protein
260744_at	At1g15010	0,83	0,73	1,02	0,74	0,85	1,21	1,10	1,19	expressed protein
260740_at	At1g15020	0,83	0,88	0,84	0,80	0,86	0,89	0,93	0,89	thioredoxin family protein
260743_at	At1g15040	1,05	1,02	0,85	1,05	0,94	1,05	0,92	0,96	---
260741_at	At1g15045	1,30	2,27	1,54	1,29	1,51	1,17	1,28	1,13	glutamine amidotransferase-related
260742_at	At1g15050	0,96	0,96	1,04	0,98	1,01	1,11	1,01	0,96	auxin-responsive AUX/IAA family protein
262604_at	At1g15060	0,82	0,88	0,88	0,96	0,88	0,84	0,90	1,10	expressed protein
262596_at	At1g15080	0,93	0,95	0,94	0,96	1,10	0,88	1,20	0,96	phosphatidic acid phosphatase family protein / PAP2 family protein
262590_at	At1g15100	0,83	0,99	0,77	1,82	1,58	0,91	1,10	1,22	zinc finger (C3HC4-type RING finger) family protein
262583_at	At1g15110	0,84	0,96	0,99	0,99	1,04	0,98	0,99	1,08	phosphatidyl serine synthase family protein
262593_at	At1g15120	1,31	1,08	0,89	1,14	1,14	0,92	0,87	0,87	ubiquinol-cytochrome C reductase complex 7.8 kDa protein, putative / mitoc
262588_at	At1g15130	0,92	0,89	0,98	0,90	1,00	0,94	0,95	1,05	hydroxyproline-rich glycoprotein family protein
262572_at	At1g15140	0,86	0,77	0,78	0,87	0,82	1,07	0,95	0,96	oxidoreductase NAD-binding domain-containing protein
262589_s_at	At1g15150	0,96	1,00	0,89	0,98	0,92	1,05	0,99	0,84	MATE efflux family protein
262605_at	At1g15170	0,88	0,77	0,74	0,96	0,88	0,92	0,92	0,79	MATE efflux family protein
262569_at	At1g15180	0,69	0,67	0,64	0,70	0,73	0,84	0,78	0,68	MATE efflux family protein

262606_at	At1g15190	0,92	0,94	0,89	1,04	1,06	1,00	1,05	0,89	hypothetical protein
262570_at	At1g15200	1,28	1,13	1,10	0,91	0,75	1,20	1,50	1,54	protein-protein interaction regulator family protein
262575_at	At1g15210	0,94	0,89	0,74	1,40	1,19	1,14	1,44	1,40	ABC transporter family protein
262591_at	At1g15220	1,06	0,99	1,03	0,89	1,10	1,12	0,93	1,08	cytochrome c biogenesis protein family
262574_at	At1g15230	0,99	1,02	1,03	1,01	0,98	1,24	1,00	1,18	expressed protein
262594_at	At1g15250	1,39	1,19	1,49	1,17	0,92	0,94	0,93	0,95	60S ribosomal protein L37 (RPL37A)
262598_at	At1g15260	1,17	0,96	1,06	0,97	1,12	1,07	1,20	0,83	expressed protein
262602_at	At1g15270	1,89	1,66	1,41	1,44	1,35	1,10	1,08	0,97	expressed protein
262576_at	At1g15280	1,17	1,19	1,42	0,83	0,93	1,13	1,29	1,24	glycine-rich protein
262577_at	At1g15290	1,09	1,06	1,43	0,73	0,70	1,03	1,25	1,37	tetratricopeptide repeat (TPR)-containing protein
262578_at	At1g15300	0,92	0,89	1,01	0,92	0,90	1,13	1,01	1,09	hAT-like transposase family (hobo/Ac/Tam3)
262601_at	At1g15310	0,97	1,15	1,03	1,07	1,09	1,13	1,06	1,08	signal recognition particle 54 kDa protein 1 / SRP54 (SRP-54) (SRP-54A)
262579_at	At1g15320	0,95	0,93	0,92	0,88	1,04	0,94	0,95	0,84	hypothetical protein
262580_at	At1g15330	0,92	1,15	1,16	1,02	1,53	0,95	0,95	1,04	CBS domain-containing protein
262600_at	At1g15340	1,11	1,15	1,07	0,82	0,82	1,30	1,38	1,56	methyl-CpG-binding domain-containing protein
262599_at	At1g15350	0,84	0,82	0,87	0,97	1,20	0,82	0,81	0,97	expressed protein
262595_at	At1g15360	1,01	0,94	0,99	0,82	1,16	0,96	1,09	1,19	AP2 domain-containing transcription factor family protein
262581_at	At1g15370	1,15	0,93	0,97	1,16	1,17	1,09	0,96	0,98	expressed protein
262603_at	At1g15380	1,35	1,56	1,59	0,86	0,97	0,98	1,12	1,14	lactoylglutathione lyase family protein / glyoxalase I family protein
262573_at	At1g15390	1,24	1,15	1,03	0,96	0,83	0,88	0,97	0,88	peptide deformylase, mitochondrial / polypeptide deformylase 1A (PDF1A)
262592_at	At1g15400	0,92	1,00	1,37	1,04	1,22	0,95	0,92	1,22	expressed protein
262582_at	At1g15410	0,79	0,83	0,64	0,78	0,82	0,82	0,84	0,91	aspartate-glutamate racemase family
262544_at	At1g15425	1,24	0,94	0,86	0,97	0,89	1,03	0,90	0,85	expressed protein
262571_at	At1g15430	0,88	0,94	1,02	0,87	0,89	0,95	1,11	1,28	expressed protein
262584_at	At1g15440	1,39	1,14	1,30	0,87	0,84	0,92	0,87	0,75	transducin family protein / WD-40 repeat family protein
262585_at	At1g15460	0,97	1,07	1,16	0,93	0,99	1,23	1,40	0,95	anion exchange family protein
262597_at	At1g15470	1,05	0,87	0,88	1,00	0,92	1,01	0,88	0,94	transducin family protein / WD-40 repeat family protein
262586_at	At1g15480	0,95	0,77	0,95	0,95	1,10	0,95	0,79	0,82	DNA-binding protein, putative
262587_at	At1g15490	1,02	0,86	0,73	0,90	0,96	0,89	0,92	0,94	hydrolase, alpha/beta fold family protein
261767_s_at	At1g15500	1,25	1,16	1,15	0,87	0,82	1,01	1,03	0,95	chloroplast ADP, ATP carrier protein, putative / ADP, ATP translocase, puta
261762_at	At1g15510	0,85	0,75	0,93	0,64	0,53	1,04	0,91	0,82	pentatricopeptide (PPR) repeat-containing protein
261763_at	At1g15520	0,98	1,12	1,08	0,99	0,94	1,45	1,48	1,58	ABC transporter family protein
261764_at	At1g15530	0,92	1,03	0,97	0,83	1,11	1,01	1,06	1,04	receptor lectin kinase, putative
261768_at	At1g15550	1,00	1,12	1,02	1,10	0,97	0,99	1,05	1,03	gibberellin 3-beta-dioxygenase / gibberellin 3 beta-hydroxylase (GA4)
261765_at	At1g15570	1,08	1,11	0,91	1,23	1,14	0,84	0,83	0,86	cyclin, putative
261766_at	At1g15580	1,12	1,07	1,12	1,06	0,97	1,10	1,03	0,96	auxin-responsive protein / indoleacetic acid-induced protein 5 (IAA5) / auxir
261759_at	At1g15590	0,99	0,95	1,04	1,05	1,17	1,06	1,03	1,03	hypothetical protein
261760_at	At1g15600	0,93	1,08	1,10	0,99	1,18	1,02	1,20	0,85	hypothetical protein
257480_at	At1g15640	0,82	0,95	0,80	1,39	1,47	0,95	1,12	1,04	hypothetical protein
261761_at	At1g15660	1,32	1,24	1,05	1,13	1,08	0,93	1,09	0,92	expressed protein
259502_at	At1g15670	0,48	0,85	0,99	1,10	1,04	0,70	0,90	1,12	kelch repeat-containing F-box family protein
259504_at	At1g15690	1,04	1,06	1,06	0,83	0,82	1,03	0,99	1,00	pyrophosphate-energized vacuolar membrane proton pump / pyrophosphat
259485_at	At1g15700	1,00	0,86	0,97	1,27	0,94	0,91	0,92	0,84	ATP synthase gamma chain 2, chloroplast (ATPC2)
259486_at	At1g15710	0,80	0,73	0,67	0,93	0,92	0,92	0,69	0,65	prephenate dehydrogenase family protein
259487_at	At1g15720	1,01	1,08	0,90	1,13	1,10	1,14	0,94	0,97	myb family transcription factor
259499_at	At1g15730	0,98	0,92	1,15	0,95	0,86	1,19	1,22	1,10	PRLI-interacting factor L, putative

259500_at	At1g15740	1,11	2,19	2,44	1,06	0,99	1,00	1,40	1,33	leucine-rich repeat family protein
259501_at	At1g15750	1,02	1,01	1,11	0,99	0,89	1,02	1,01	1,13	WD-40 repeat family protein
259488_at	At1g15780	1,08	0,98	0,90	0,90	0,75	0,88	0,97	1,02	expressed protein
259489_at	At1g15790	1,23	1,12	1,67	1,16	1,13	1,55	1,35	1,51	expressed protein
259490_at	At1g15800	0,92	0,92	0,99	0,79	0,97	0,91	0,99	0,99	expressed protein
259505_at	At1g15810	1,35	1,42	1,41	1,29	1,44	1,03	1,03	1,05	ribosomal protein S15 family protein
259491_at	At1g15820	1,00	1,06	1,15	0,95	0,91	0,97	0,95	0,93	chlorophyll A-B binding protein, chloroplast (LHCB6)
259492_at	At1g15830	0,87	0,88	0,94	0,97	1,11	0,98	1,06	1,14	expressed protein
259493_at	At1g15840	0,85	0,94	0,93	0,82	1,01	1,02	0,96	1,10	expressed protein
259494_at	At1g15850	1,02	0,98	0,90	1,01	1,11	1,10	1,00	0,85	transducin family protein / WD-40 repeat family protein
259497_at	At1g15860	1,22	1,00	1,04	0,97	1,01	1,00	1,02	0,92	expressed protein
259503_at	At1g15870	1,08	1,15	1,16	1,19	1,40	0,85	0,90	1,01	mitochondrial glycoprotein family protein / MAM33 family protein
259498_at	At1g15880	0,85	0,74	0,82	0,81	0,97	0,95	0,92	1,10	Golgi SNARE 11 protein
259495_at	At1g15890	1,00	0,99	0,89	0,73	0,72	1,05	1,15	1,18	disease resistance protein (CC-NBS-LRR class), putative
259496_at	At1g15900	1,03	0,85	0,88	1,06	1,12	1,03	0,84	0,99	hypothetical protein
261837_s_at	At1g15910	1,06	1,00	1,02	0,79	0,98	1,01	0,96	0,92	XH/XS domain-containing protein / XS zinc finger domain-containing protei
261841_at	At1g15920	0,83	0,67	0,67	1,08	0,93	0,98	0,85	0,86	CCR4-NOT transcription complex protein, putative
261789_at	At1g15930	1,41	1,31	1,16	1,36	1,17	0,96	0,98	1,01	40S ribosomal protein S12 (RPS12A)
261844_at	At1g15940	1,41	1,29	1,44	0,86	0,87	1,30	1,72	1,53	expressed protein
261792_at	At1g15950	1,32	1,32	1,47	1,40	1,19	1,04	1,09	1,16	cinnamoyl-CoA reductase, putative
261845_at	At1g15960	1,22	1,36	1,45	0,90	0,86	0,78	0,78	0,88	NRAMP metal ion transporter 6, putative (NRAMP6)
261788_at	At1g15980	1,22	1,15	1,33	0,90	0,71	1,39	1,36	1,36	expressed protein
261786_at	At1g15990	0,97	0,96	0,98	0,95	0,97	0,90	0,97	1,14	cyclic nucleotide-regulated ion channel, putative (CNGC7)
261790_at	At1g16000	1,50	1,21	1,11	1,62	1,50	1,00	0,90	0,80	expressed protein
261795_at	At1g16010	0,88	0,83	1,18	0,92	0,99	1,03	1,00	1,05	magnesium transporter CorA-like family protein (MRS2-1)
261787_at	At1g16020	0,98	0,87	1,01	1,03	1,16	0,96	0,94	0,98	expressed protein
261838_at	At1g16030	1,34	1,27	1,29	1,05	1,29	1,14	1,09	1,41	heat shock protein 70, putative / HSP70, putative
261839_at	At1g16040	1,25	0,87	0,92	1,00	1,10	0,87	1,16	1,03	expressed protein
261835_at	At1g16050	0,98	0,92	1,27	0,91	1,02	1,00	1,21	1,08	expressed protein
261794_at	At1g16060	1,23	1,05	1,18	0,88	1,04	1,04	1,04	1,07	ovule development protein, putative
261840_at	At1g16070	1,23	1,12	1,01	1,31	0,99	0,95	0,98	0,90	tubby family protein
261793_at	At1g16080	1,14	1,06	1,10	0,99	0,99	1,16	1,10	1,14	expressed protein
261836_at	At1g16090	0,79	0,93	0,90	0,91	1,01	1,12	0,96	1,14	wall-associated kinase-related
257478_at	At1g16130	0,91	1,03	0,90	0,57	0,53	1,13	1,08	0,98	wall-associated kinase, putative
257479_at	At1g16150	0,65	0,62	0,71	1,15	0,86	1,01	0,97	1,29	wall-associated kinase, putative
261842_at	At1g16160	0,92	0,95	0,87	1,08	0,97	1,08	1,01	0,90	protein kinase family protein
261791_at	At1g16170	0,91	0,86	0,91	0,99	0,78	1,03	0,94	1,20	expressed protein
261843_at	At1g16180	1,03	0,94	0,92	1,14	1,13	0,91	0,89	0,97	TMS membrane family protein / tumour differentially expressed (TDE) famil
262708_at	At1g16190	0,84	0,84	0,74	1,35	1,14	0,98	0,91	0,78	DNA repair protein RAD23, putative
262710_at	At1g16210	1,14	1,11	1,07	1,01	1,01	1,13	1,15	1,21	expressed protein
262702_at	At1g16220	1,08	1,12	0,90	1,11	1,06	1,01	1,07	0,91	protein phosphatase 2C family protein / PP2C family protein
262709_at	At1g16240	0,83	0,75	1,00	1,10	1,23	0,90	0,96	1,01	syntaxin 51 (SYP51)
262705_at	At1g16260	0,82	1,00	0,85	0,79	0,75	1,42	1,40	2,03	protein kinase family protein
262706_at	At1g16280	1,27	1,20	1,44	0,87	0,76	1,03	0,99	1,11	DEAD/DEAH box helicase, putative
262707_at	At1g16290	1,02	1,06	0,86	1,14	0,96	1,02	1,02	0,95	expressed protein
262751_at	At1g16310	1,22	1,07	0,99	1,05	0,95	1,08	0,94	0,79	cation efflux family protein

262752_at	At1g16330	1,11	1,31	1,11	1,12	1,02	1,05	1,04	0,99	cyclin family protein
262753_at	At1g16340	1,21	1,06	0,98	0,83	0,94	1,01	0,92	0,93	2-dehydro-3-deoxyphosphooctonate aldolase, putative / phospho-2-dehydr
262754_at	At1g16350	1,39	1,38	1,38	1,10	1,14	0,97	0,98	0,91	inosine-5'-monophosphate dehydrogenase, putative
262755_at	At1g16360	1,03	0,96	0,96	1,08	1,01	0,89	1,07	1,02	LEM3 (ligand-effect modulator 3) family protein / CDC50 family protein
262756_at	At1g16370	0,52	0,46	0,59	1,07	1,05	0,86	0,74	0,68	transporter-related
262757_at	At1g16380	1,09	0,99	0,96	0,98	1,02	1,03	1,14	1,04	cation/proton exchanger, putative (CHX1)
262730_at	At1g16390	1,07	1,08	1,00	1,01	1,06	0,74	0,70	1,04	organic cation transporter-related
262717_s_at	At1g16410	1,57	1,43	1,40	1,51	1,33	0,95	1,01	0,77	cytochrome P450 family protein
262731_at	At1g16420	1,07	0,90	0,91	1,10	0,88	1,15	1,05	1,03	latex-abundant protein, putative (AMC8) / caspase family protein
262714_s_at	At1g16430	1,60	1,28	1,58	1,39	1,44	0,99	0,85	0,97	surfeit locus protein 5 family protein / SURF5 family protein
262732_at	At1g16440	1,10	1,12	1,02	0,91	1,19	1,04	1,00	0,80	protein kinase, putative
262712_at	At1g16460	0,41	0,41	0,39	0,87	0,79	0,81	0,81	0,87	mercaptopyruvate sulfurtransferase (MST2) (RDH2)
262716_at	At1g16470	0,93	0,78	0,77	1,20	1,33	0,91	0,88	0,86	20S proteasome alpha subunit B (PAB1) (PRC3)
262701_at	At1g16480	1,06	1,01	0,90	0,84	0,99	1,03	0,92	1,02	---
262715_at	At1g16490	0,94	1,08	0,98	0,96	1,19	1,03	0,98	1,07	myb family transcription factor (MYB58)
262711_at	At1g16500	0,86	0,95	0,81	1,02	1,05	1,02	1,05	0,79	expressed protein
262703_at	At1g16510	0,73	0,77	1,03	0,95	1,29	0,83	1,17	0,90	auxin-responsive family protein
262713_at	At1g16520	1,24	0,93	1,17	1,44	1,57	1,08	0,99	1,00	expressed protein
262704_at	At1g16530	0,92	1,04	1,14	1,03	1,32	0,98	0,95	1,12	LOB domain protein 3 / lateral organ boundaries domain protein 3 (LBD3)
246325_at	At1g16540	1,19	1,12	0,96	0,76	0,92	1,49	1,16	1,39	molybdenum cofactor sulfurase (LOS5) (ABA3)
246320_at	At1g16560	0,95	0,93	0,88	1,15	1,08	0,95	0,86	0,92	Per1-like family protein
246351_at	At1g16570	0,92	1,05	1,02	0,87	0,97	1,13	1,02	0,99	glycosyl transferase family 1 protein
246326_at	At1g16590	0,78	0,89	1,01	1,13	1,11	0,98	0,98	1,02	mitotic spindle checkpoint protein, putative
246324_at	At1g16610	1,12	1,11	1,22	0,95	1,02	1,09	0,89	0,86	arginine/serine-rich protein, putative (SR45)
246321_at	At1g16640	1,11	0,85	0,85	1,05	1,08	0,97	0,96	0,93	transcriptional factor B3 family protein
246350_at	At1g16650	0,97	0,90	0,93	0,89	0,82	1,00	0,97	0,96	expressed protein
246327_at	At1g16670	0,64	1,22	1,12	1,15	1,15	1,02	1,12	1,13	protein kinase family protein
246322_at	At1g16680	1,27	1,65	1,38	0,78	0,87	1,12	1,16	1,19	DNAJ heat shock N-terminal domain-containing protein / S-locus protein, pu
246323_at	At1g16690	1,05	1,20	1,10	1,16	1,26	1,08	0,89	0,99	transcription factor-related
255768_at	At1g16705	0,88	0,99	0,91	0,91	1,20	0,94	0,98	0,86	p300/CBP acetyltransferase-related protein-related
255762_at	At1g16710	0,95	1,01	1,23	0,83	0,99	1,17	1,18	1,33	TAZ zinc finger family protein / zinc finger (ZZ type) family protein
255764_at	At1g16720	1,28	1,29	1,36	0,88	0,87	1,67	1,58	1,69	expressed protein
255763_at	At1g16730	1,07	1,02	1,16	1,64	1,46	1,08	1,08	1,03	expressed protein
255767_at	At1g16740	1,15	0,94	0,95	1,12	1,13	1,04	0,95	0,92	ribosomal protein L20 family protein
255766_at	At1g16750	1,11	1,02	1,00	0,95	1,03	1,01	0,89	0,94	expressed protein
255765_at	At1g16760	0,94	1,00	1,11	1,01	1,04	1,08	1,03	1,06	protein kinase family protein
255761_at	At1g16770	1,06	1,04	1,25	0,83	1,04	1,07	0,94	1,18	hypothetical protein
255760_at	At1g16780	0,77	0,77	0,72	0,74	0,64	0,90	0,90	0,97	vacuolar-type H+-translocating inorganic pyrophosphatase, putative
255759_at	At1g16790	0,95	0,73	1,19	1,02	0,81	0,88	0,88	0,80	ribosomal protein-related
255758_at	At1g16800	1,04	1,02	0,96	0,96	0,90	1,06	1,03	0,94	tRNA-splicing endonuclease positive effector-related
256113_at	At1g16810	1,15	1,09	1,08	1,12	1,15	1,19	1,10	1,10	expressed protein
256111_at	At1g16820	0,92	0,90	0,91	0,98	1,18	1,21	1,11	0,98	expressed protein
256107_at	At1g16830	1,06	1,05	0,89	1,02	1,09	0,99	0,92	0,92	pentatricopeptide (PPR) repeat-containing protein
256117_at	At1g16840	0,88	0,89	1,09	1,08	1,12	0,95	0,89	1,05	expressed protein
256114_at	At1g16850	0,56	0,58	0,50	1,25	0,84	0,91	0,82	0,65	expressed protein
256116_at	At1g16860	0,96	0,74	0,80	1,35	1,16	0,82	0,74	0,80	merozoite surface protein-related

256106_at	At1g16870	1,07	1,08	0,98	1,11	1,04	0,85	0,93	0,92	mitochondrial 28S ribosomal protein S29-related
256115_at	At1g16880	1,05	0,99	1,11	0,86	0,92	1,17	1,19	1,12	uridylyltransferase-related
256110_at	At1g16900	1,24	0,93	0,96	1,11	1,17	1,02	0,82	0,80	curculin-like (mannose-binding) lectin family protein
256105_at	At1g16910	1,00	1,20	1,00	1,03	1,06	0,98	0,90	0,89	hypothetical protein
256112_at	At1g16920	0,99	0,83	0,84	1,54	1,25	0,82	0,80	0,92	Ras-related GTP-binding protein, putative
256108_at	At1g16940	0,88	0,92	0,96	0,90	1,16	0,92	1,00	1,06	F-box family protein
256109_at	At1g16950	0,98	0,99	1,05	1,00	0,96	1,00	0,89	1,08	expressed protein
256118_at	At1g16970	0,83	1,03	1,05	1,00	1,18	1,05	1,07	1,02	Ku70-like protein
256104_at	At1g16980	1,01	1,00	0,91	0,96	1,10	1,04	0,92	0,93	alpha, alpha-trehalose-phosphate synthase, UDP-forming, putative / trehalo
262535_at	At1g17000	1,00	1,04	0,99	1,10	1,00	1,04	0,99	1,00	alpha, alpha-trehalose-phosphate synthase, UDP-forming, putative / trehalo
262527_at	At1g17010	0,99	0,94	0,89	1,04	0,84	1,00	1,00	1,02	oxidoreductase, 2OG-Fe(II) oxygenase family protein
262482_at	At1g17020	0,30	0,31	0,21	0,95	0,76	1,42	1,32	1,22	oxidoreductase, 2OG-Fe(II) oxygenase family protein
262534_at	At1g17040	0,97	0,91	1,01	0,87	1,05	0,94	1,06	1,03	transcription factor-related
262526_at	At1g17050	1,43	1,37	1,52	0,99	0,88	1,63	1,54	1,47	geranyl diphosphate synthase, putative / GPPS, putative / dimethylallyltrans
262525_at	At1g17060	0,64	0,81	0,90	1,11	0,89	0,83	1,18	1,18	cytochrome P450, putative
262524_at	At1g17070	0,95	0,96	0,97	0,98	1,18	0,96	0,95	0,92	D111/G-patch domain-containing protein
262481_at	At1g17080	0,93	0,83	0,82	1,22	0,99	0,94	0,90	0,98	expressed protein
262533_at	At1g17090	0,84	0,91	0,88	0,91	0,91	0,80	0,86	0,93	expressed protein
262536_at	At1g17100	0,97	1,15	1,24	1,07	0,98	1,26	1,11	1,00	SOUL heme-binding family protein
262523_at	At1g17110	0,95	1,17	1,38	0,88	0,94	0,99	1,15	0,95	ubiquitin-specific protease 15 (UBP15)
262522_at	At1g17120	0,86	0,81	0,79	0,87	0,79	0,89	0,76	0,77	amino acid permease family protein
262521_at	At1g17130	1,20	1,05	1,21	0,90	1,02	1,20	1,24	1,39	cell cycle control protein-related
262538_at	At1g17140	0,96	1,00	0,93	1,13	0,85	1,06	1,18	0,96	tropomyosin-related
262512_at	At1g17145	1,02	0,93	0,99	0,83	0,82	1,12	1,24	1,26	expressed protein
262520_at	At1g17150	0,88	1,01	0,97	0,95	1,06	1,02	0,94	1,01	glycoside hydrolase family 28 protein / polygalacturonase (pectinase) family
262519_at	At1g17160	0,83	0,78	0,78	1,03	1,40	0,81	0,77	0,70	pfkB-type carbohydrate kinase family protein
262518_at	At1g17170	1,80	1,37	1,11	0,68	0,69	0,78	0,83	0,81	glutathione S-transferase, putative
262517_at	At1g17180	1,50	1,31	1,36	0,86	0,89	2,75	2,44	1,59	glutathione S-transferase, putative
262516_at	At1g17190	0,92	0,82	0,85	1,08	1,09	1,08	1,43	1,58	glutathione S-transferase, putative
262539_at	At1g17200	1,09	1,22	1,34	0,82	0,82	0,96	0,82	0,83	integral membrane family protein
262532_at	At1g17210	0,17	0,19	0,19	0,70	0,61	0,93	1,05	1,25	expressed protein
262483_at	At1g17220	1,04	0,97	0,98	0,79	0,74	1,25	1,28	1,12	translation initiation factor IF-2, chloroplast, putative
262531_at	At1g17230	0,96	0,74	0,75	1,11	1,19	0,99	0,82	0,85	leucine-rich repeat family protein / protein kinase family protein
262530_at	At1g17240	0,91	1,07	1,08	1,03	0,87	1,02	0,94	0,89	leucine-rich repeat family protein
262529_at	At1g17250	0,64	0,61	0,60	0,95	1,10	0,89	0,90	0,88	leucine-rich repeat family protein
262528_at	At1g17260	1,07	1,07	0,92	0,88	1,00	0,99	1,00	1,12	ATPase 10, plasma membrane-type, putative / proton pump 10, putative / p
262537_s_at	At1g17280	0,87	0,71	0,94	1,01	1,09	0,99	1,06	1,15	ubiquitin-conjugating enzyme, putative
260847_s_at	At1g17290	0,93	0,84	0,88	0,84	0,83	0,97	0,96	0,89	alanine aminotransferase, putative
260846_at	At1g17300	0,98	1,05	0,94	1,04	1,36	1,12	1,07	1,26	expressed protein
260845_at	At1g17310	1,01	1,07	0,94	1,08	1,16	0,99	1,03	1,14	MADS-box protein (AGL100)
261029_at	At1g17330	0,87	0,78	0,89	0,98	0,91	0,86	0,79	0,70	metal-dependent phosphohydrolase HD domain-containing protein-related
261060_at	At1g17340	0,97	1,05	1,52	1,26	1,12	1,07	1,53	1,99	phosphoinositide phosphatase family protein
261087_at	At1g17350	1,02	0,71	0,79	0,99	1,13	0,97	0,92	0,89	auxin-induced-related / indole-3-acetic acid induced-related
261031_at	At1g17360	1,00	1,07	1,33	1,26	1,05	1,38	1,63	2,08	COP1-interacting protein-related
261040_at	At1g17370	1,01	0,94	0,92	1,06	1,05	1,02	1,03	0,95	oligouridylate-binding protein, putative
261033_at	At1g17380	0,70	1,54	0,97	0,89	0,89	1,12	1,64	1,50	expressed protein

261035_at	At1g17390	1,05	1,03	0,95	1,03	0,92	0,98	0,97	0,94	hypothetical protein
261030_at	At1g17410	0,81	1,00	0,97	0,98	0,88	1,06	0,98	1,12	nucleoside diphosphate kinase family protein
261037_at	At1g17420	0,62	0,77	1,22	1,02	0,95	1,18	1,22	1,34	lipoxygenase, putative
261032_at	At1g17430	0,70	0,78	1,06	0,72	0,75	0,69	0,82	0,93	hydrolase, alpha/beta fold family protein
261041_at	At1g17440	0,80	0,80	1,05	1,01	0,78	1,02	1,12	1,34	transcription initiation factor IID (TFIID) subunit A family protein
261034_s_at	At1g17450	0,99	1,01	0,87	0,93	0,74	0,96	1,15	0,62	ATP phosphoribosyltransferase -related
261039_at	At1g17455	0,99	0,96	0,96	1,03	0,96	0,82	0,84	0,99	expressed protein
261086_at	At1g17460	0,84	0,98	1,24	0,72	0,95	0,80	0,87	1,10	myb family transcription factor
261036_at	At1g17470	1,21	1,03	0,99	0,94	0,96	0,92	0,90	0,96	developmentally regulated GTP-binding protein (DRG1)
261085_at	At1g17480	1,09	0,84	1,16	1,05	1,07	0,85	1,05	0,90	calmodulin-binding family protein
261038_at	At1g17490	1,07	0,81	0,98	1,10	1,29	1,09	0,91	0,98	expressed protein
260731_at	At1g17500	0,82	0,77	0,77	1,14	1,02	1,00	0,91	0,92	haloacid dehalogenase-like hydrolase family protein
260682_at	At1g17510	1,15	0,93	0,89	1,03	1,19	1,01	0,85	0,73	expressed protein
260732_at	At1g17520	0,97	0,87	1,04	0,91	0,76	1,12	0,96	0,96	DNA-binding protein, putative
260687_at	At1g17530	0,95	0,95	1,18	1,52	1,57	0,80	0,86	0,79	mitochondrial import inner membrane translocase subunit Tim17/Tim22/Tim
260737_at	At1g17540	0,97	0,94	0,95	1,08	0,95	0,84	1,07	1,06	protein kinase-related
260710_at	At1g17545	0,99	0,93	1,00	0,96	0,84	0,91	1,00	0,93	protein phosphatase 2C-related / PP2C-related
260712_at	At1g17550	1,07	1,01	1,01	0,96	1,22	1,07	0,98	0,93	protein phosphatase 2C-related / PP2C-related
260683_at	At1g17560	1,34	1,13	0,99	1,91	1,48	0,94	0,90	0,81	ribosomal protein L14 family protein
260711_at	At1g17580	0,95	0,81	1,27	0,93	1,02	1,07	0,97	1,26	myosin, putative
260684_at	At1g17590	1,01	0,72	1,03	0,85	0,76	0,83	0,92	1,00	CCAAT-binding transcription factor (CBF-B/NF-YA) family protein
260734_at	At1g17600	0,83	0,95	1,10	0,83	0,86	1,05	1,03	0,99	disease resistance protein (TIR-NBS-LRR class), putative
260735_at	At1g17610	0,86	0,83	0,93	0,95	0,94	1,16	0,97	1,02	disease resistance protein-related
260713_at	At1g17615	1,02	1,00	1,10	1,11	1,10	1,01	1,13	1,11	disease resistance protein (TIR-NBS class), putative
260686_at	At1g17620	0,72	0,72	0,80	1,07	1,18	0,91	0,97	1,06	expressed protein
260736_at	At1g17630	1,12	0,85	0,88	0,76	0,83	1,13	1,04	0,86	pentatricopeptide (PPR) repeat-containing protein
260733_at	At1g17640	0,92	0,93	1,27	0,85	0,94	1,01	0,97	0,81	RNA recognition motif (RRM)-containing protein
260685_at	At1g17650	1,13	0,81	0,79	0,94	0,88	1,31	1,39	1,35	6-phosphogluconate dehydrogenase NAD-binding domain-containing prote
260688_at	At1g17665	0,68	0,84	1,08	0,60	0,82	0,88	0,94	0,79	expressed protein
259397_at	At1g17680	0,82	0,99	1,50	1,07	1,10	0,94	0,93	1,32	transcription factor-related
259406_at	At1g17690	1,15	1,02	1,00	0,84	0,76	1,05	1,09	1,00	expressed protein
259398_at	At1g17700	1,01	1,15	1,01	0,90	1,01	0,87	0,91	0,83	prenylated rab acceptor (PRA1) family protein
259399_at	At1g17710	1,28	1,36	1,22	2,12	1,49	0,89	0,95	0,95	expressed protein
259404_at	At1g17720	0,98	0,88	0,86	1,16	1,06	1,00	0,98	0,97	serine/threonine protein phosphatase 2A (PP2A) 55 kDa regulatory subunit
259402_at	At1g17730	1,17	0,96	0,88	1,12	1,16	1,10	1,10	1,01	SNF7 family protein
259405_at	At1g17740	1,04	0,94	0,87	1,11	1,00	1,03	1,09	0,99	D-3-phosphoglycerate dehydrogenase / 3-PGDH
259403_at	At1g17745	0,82	0,85	0,80	0,81	0,62	1,28	1,33	1,19	D-3-phosphoglycerate dehydrogenase / 3-PGDH
259400_at	At1g17750	0,95	0,92	0,68	1,32	0,94	1,22	1,25	1,18	leucine-rich repeat transmembrane protein kinase, putative
259401_at	At1g17760	1,22	1,20	1,47	1,24	0,96	1,05	1,00	1,00	suppressor of forked protein family protein / SUF family protein
255906_at	At1g17790	0,93	1,13	1,12	0,87	0,93	1,06	1,17	0,98	DNA-binding bromodomain-containing protein
255896_at	At1g17800	1,02	1,02	0,90	1,02	1,14	1,18	0,92	0,96	plastocyanin-like domain-containing protein
255905_at	At1g17810	0,35	0,51	0,57	0,57	0,52	0,99	1,09	1,03	major intrinsic family protein / MIP family protein
255900_at	At1g17830	0,69	0,81	0,76	0,75	0,88	0,88	1,04	0,79	expressed protein
255889_at	At1g17840	1,47	1,95	1,97	1,06	0,86	1,13	1,13	1,24	ABC transporter family protein
255890_at	At1g17850	1,15	1,34	1,56	0,71	0,72	1,21	1,19	1,51	expressed protein
255904_at	At1g17860	1,19	1,05	1,07	1,18	1,29	0,93	0,92	0,91	trypsin and protease inhibitor family protein / Kunitz family protein

255891_at	At1g17870	1,40	1,12	0,96	0,79	0,92	1,15	1,24	1,10	expressed protein
255902_at	At1g17880	1,39	1,18	1,12	0,96	1,03	1,07	1,02	1,01	nascent polypeptide-associated complex (NAC) domain-containing protein /
255901_at	At1g17890	1,14	0,84	0,87	1,13	1,44	0,97	0,84	0,82	GDP-4-keto-6-deoxy-D-mannose-3,5-epimerase-4-reductase, putative
255897_at	At1g17900	1,00	0,94	0,98	1,02	1,04	0,97	0,98	0,97	hypothetical protein
255892_at	At1g17910	0,97	1,07	1,01	0,89	1,02	1,03	1,03	1,08	wall-associated kinase, putative
255907_at	At1g17920	1,12	1,06	1,07	0,96	0,96	0,84	0,89	0,95	homeobox-leucine zipper family protein / lipid-binding START domain-conta
255898_at	At1g17930	1,04	1,21	1,29	1,05	1,29	0,92	1,15	0,89	expressed protein
255903_at	At1g17950	1,13	1,01	1,04	1,01	1,09	0,99	1,08	0,98	myb family transcription factor (MYB52)
255893_at	At1g17960	2,69	2,40	3,00	0,89	0,88	1,34	1,65	2,39	threonyl-tRNA synthetase, putative / threonine--tRNA ligase, putative
255899_at	At1g17970	1,18	1,26	1,77	0,82	0,59	1,03	1,07	1,39	zinc finger (C3HC4-type RING finger) family protein
255894_at	At1g17980	0,89	0,83	0,96	1,20	1,33	1,05	1,13	1,22	nucleotidyltransferase family protein
255895_at	At1g17990	0,47	0,57	0,68	0,81	0,74	0,95	1,43	1,80	12-oxophytodienoate reductase, putative
255908_s_at	At1g18010	0,48	0,83	0,90	0,69	0,93	1,00	0,99	0,99	expressed protein
255910_at	At1g18030	0,90	0,94	1,02	0,70	0,75	1,10	1,41	1,46	protein phosphatase 2C, putative / PP2C, putative
255909_at	At1g18040	1,15	0,94	1,07	1,03	1,13	0,92	0,85	0,86	cell division protein kinase, putative
256126_at	At1g18050	1,09	1,01	1,01	1,17	1,20	1,00	1,07	1,05	SWAP (Suppressor-of-White-APricot)/surp domain-containing protein
256076_at	At1g18060	1,02	0,95	0,93	0,89	0,92	1,21	1,14	1,14	expressed protein
256119_at	At1g18070	1,05	0,99	0,84	1,21	0,95	1,02	1,03	0,98	EF-1-alpha-related GTP-binding protein, putative
256072_at	At1g18080	1,28	1,24	1,13	0,99	0,93	0,95	0,96	0,94	WD-40 repeat family protein / auxin-dependent protein (ARCA) / guanine nu
256077_at	At1g18090	1,14	0,91	1,11	0,79	0,98	1,16	1,26	1,27	exonuclease, putative
256073_at	At1g18100	0,60	0,54	0,37	1,66	1,80	1,18	0,93	0,98	mother of FT and TF1 protein (MFT)
256120_at	At1g18130	0,99	0,92	1,02	1,04	1,05	1,09	1,04	1,10	tRNA synthetase-related / tRNA ligase-related
256128_at	At1g18140	0,92	1,11	1,03	1,01	1,09	1,12	1,03	1,04	laccase family protein / diphenol oxidase family protein
256075_at	At1g18150	0,99	0,83	0,82	1,28	1,28	0,89	0,87	0,79	mitogen-activated protein kinase, putative / MAPK, putative (MPK8)
256121_at	At1g18160	0,96	0,86	0,73	0,77	1,03	1,02	1,03	0,91	protein kinase family protein
256130_at	At1g18170	0,82	0,75	0,81	0,59	0,78	1,12	1,04	1,03	immunophilin / FKBP-type peptidyl-prolyl cis-trans isomerase family protein
256122_at	At1g18180	1,18	1,09	1,01	1,11	1,34	1,04	0,82	1,11	expressed protein
256123_at	At1g18190	0,89	0,95	1,45	1,05	0,92	1,10	1,23	1,19	expressed protein
256127_at	At1g18200	0,96	1,02	1,07	1,05	1,05	0,86	1,03	0,91	Ras-related GTP-binding family protein
256129_at	At1g18210	0,91	0,99	1,30	0,95	1,16	1,06	1,05	1,01	calcium-binding protein, putative
256124_at	At1g18220	1,04	0,98	1,02	0,97	0,98	1,03	1,01	1,04	hypothetical protein
256125_at	At1g18250	1,95	1,52	1,56	2,33	2,60	0,83	0,77	0,75	thaumatin, putative
256074_at	At1g18260	0,86	0,95	1,00	0,92	0,93	0,95	1,01	1,02	suppressor of lin-12-like protein-related / sel-1 protein-related
261674_at	At1g18270	0,99	1,08	1,03	1,10	0,92	0,99	0,97	1,03	ketose-bisphosphate aldolase class-II family protein
261673_at	At1g18280	1,11	1,31	1,53	0,84	1,06	1,09	1,02	0,98	protease inhibitor/seed storage/lipid transfer protein (LTP) family protein
261675_at	At1g18290	0,98	1,10	0,93	1,53	1,55	1,05	1,01	0,99	hypothetical protein
261665_at	At1g18310	1,12	0,99	0,93	0,96	0,89	1,11	1,02	1,03	glycosyl hydrolase family 81 protein
261664_s_at	At1g18320	1,14	1,05	0,81	1,22	1,24	0,90	1,00	0,87	mitochondrial import inner membrane translocase subunit Tim17/Tim22/Tim
261663_at	At1g18330	2,01	1,63	1,41	0,88	1,01	1,49	1,95	2,03	myb family transcription factor
261671_at	At1g18340	0,91	1,05	0,85	0,99	0,88	1,06	1,02	0,95	basal transcription factor complex subunit-related
261662_at	At1g18350	1,03	1,01	1,13	0,93	1,18	0,79	0,88	0,82	mitogen-activated protein kinase kinase (MAPKK), putative (MKK7)
261661_at	At1g18360	0,87	0,91	1,10	0,74	0,72	1,13	1,21	1,07	hydrolase, alpha/beta fold family protein
261660_at	At1g18370	1,55	1,41	1,45	1,33	1,50	1,12	0,99	0,80	kinesin motor family protein (NACK1)
261719_at	At1g18380	0,65	0,76	1,04	0,82	1,02	0,99	0,87	1,06	expressed protein
261718_at	At1g18390	0,66	0,76	0,96	0,72	0,78	0,94	0,99	1,37	protein kinase family protein
261717_at	At1g18400	1,89	1,57	2,17	0,69	0,84	1,00	0,86	1,01	basic helix-loop-helix (bHLH) family protein

261716_at	At1g18410	1,05	0,99	1,18	0,86	0,96	0,87	0,96	1,11	kinesin motor protein-related
261666_at	At1g18440	1,39	1,26	1,23	1,00	0,97	1,16	1,02	0,99	peptidyl-tRNA hydrolase family protein
261672_at	At1g18450	1,13	1,10	1,11	0,91	0,91	0,95	1,03	1,13	actin-related protein 4 (ARP4)
261667_at	At1g18460	1,53	2,13	2,13	1,07	0,92	1,17	1,10	1,34	lipase family protein
261677_at	At1g18470	0,72	0,79	0,96	0,74	0,68	1,01	1,16	1,25	zinc finger (C3HC4-type RING finger) family protein
261676_at	At1g18480	0,90	0,88	0,98	0,92	0,89	0,89	0,85	0,96	calcineurin-like phosphoesterase family protein
261715_at	At1g18485	0,89	0,77	0,98	0,68	0,71	1,14	0,99	0,91	pentatricopeptide (PPR) repeat-containing protein
261669_at	At1g18490	1,32	1,54	1,70	0,74	0,82	1,10	1,09	0,93	expressed protein
261668_at	At1g18500	1,03	0,83	0,85	0,87	0,92	0,87	0,87	0,83	2-isopropylmalate synthase, putative
261714_at	At1g18510	0,96	1,02	1,06	0,99	1,13	1,07	0,88	0,97	hypothetical protein
261670_at	At1g18520	1,06	0,90	1,07	1,08	1,04	1,04	1,09	1,49	senescence-associated family protein
255772_at	At1g18530	1,06	1,05	1,06	1,01	0,96	1,12	0,98	1,05	calmodulin, putative
255776_at	At1g18540	1,35	1,10	1,09	1,25	1,18	1,01	0,93	0,99	60S ribosomal protein L6 (RPL6A)
255771_at	At1g18550	1,00	0,95	1,00	0,95	1,02	0,97	1,06	0,98	kinesin motor protein-related
255770_at	At1g18560	0,93	0,95	1,03	1,03	1,02	0,96	0,98	0,95	hAT dimerisation domain-containing protein / BED zinc finger domain-conta
255753_at	At1g18570	1,15	1,36	1,27	0,89	0,80	1,01	1,33	1,33	myb family transcription factor (MYB51)
255780_at	At1g18580	0,98	0,83	0,75	0,99	1,23	0,83	0,75	0,76	glycosyltransferase family protein 8
255773_at	At1g18590	1,10	1,27	1,03	1,32	1,18	0,85	0,95	0,96	sulfotransferase family protein
255775_at	At1g18600	0,91	0,98	0,96	1,10	1,06	1,14	0,94	0,87	rhomboid family protein
255769_at	At1g18610	1,09	1,03	1,14	0,90	0,92	0,94	1,00	1,10	kelch repeat-containing protein
255774_at	At1g18620	1,04	1,33	1,24	0,68	0,49	1,07	0,95	1,09	expressed protein
255777_at	At1g18630	1,28	1,14	1,01	1,27	0,98	0,87	0,88	0,92	glycine-rich RNA-binding protein, putative
255778_at	At1g18640	0,92	0,79	0,73	0,99	1,01	0,82	0,84	0,80	3-phosphoserine phosphatase (PSP)
255779_at	At1g18650	1,05	1,17	1,23	1,25	1,23	1,02	0,96	1,01	glycosyl hydrolase family protein 17
261376_at	At1g18660	1,11	0,98	1,01	0,84	0,90	1,34	1,38	1,37	zinc finger (C3HC4-type RING finger) family protein
261427_at	At1g18670	1,03	1,15	0,98	0,85	1,17	0,97	1,09	0,74	protein kinase family protein
261426_at	At1g18680	0,93	0,80	0,76	0,94	1,08	0,81	0,92	0,94	HNH endonuclease domain-containing protein
261404_at	At1g18690	0,80	0,77	0,81	0,86	0,96	1,00	1,07	0,90	galactosyl transferase GMA12/MNN10 family protein
261424_at	At1g18700	1,00	0,93	0,88	0,92	0,95	1,20	1,08	1,01	DNAJ heat shock N-terminal domain-containing protein
261431_at	At1g18710	0,26	0,30	0,80	0,52	0,68	1,58	1,20	0,83	myb family transcription factor (MYB47)
261379_at	At1g18720	0,83	0,75	0,79	1,09	1,02	0,94	0,92	0,92	expressed protein
261422_at	At1g18730	0,90	0,71	0,75	0,87	0,87	1,19	1,14	1,11	expressed protein
261405_at	At1g18740	0,72	0,94	0,65	0,82	0,74	0,93	1,12	0,99	expressed protein
261423_at	At1g18750	0,82	0,76	0,98	0,81	0,82	1,01	0,98	1,06	MADS-box protein (AGL65)
261406_at	At1g18800	1,37	1,14	0,91	1,12	1,05	0,94	0,97	0,82	nucleosome assembly protein (NAP) family protein
261407_at	At1g18810	1,38	1,01	1,25	0,78	0,82	1,13	0,99	1,09	phytochrome kinase substrate-related
261430_at	At1g18830	1,10	1,26	1,03	0,91	1,01	0,90	1,00	0,99	transducin family protein / WD-40 repeat family protein
261421_at	At1g18840	0,99	0,86	0,99	1,30	1,30	0,84	0,69	0,73	calmodulin-binding family protein
261377_at	At1g18850	1,18	0,99	1,05	1,19	1,00	0,85	0,81	0,86	expressed protein
261429_at	At1g18860	1,18	1,13	1,42	1,11	1,15	1,01	0,99	1,19	WRKY family transcription factor
261428_at	At1g18870	0,74	0,83	0,76	0,66	0,59	0,93	1,13	0,97	isochorismate synthase, putative / isochorismate mutase, putative
261425_at	At1g18880	0,98	1,23	1,12	0,98	1,52	0,99	1,28	1,25	proton-dependent oligopeptide transport (POT) family protein
261378_at	At1g18890	0,78	0,77	0,90	1,00	0,95	1,01	1,03	1,28	calcium-dependent protein kinase 1 (CDPK1)
259461_at	At1g18900	0,78	0,79	0,83	1,04	0,97	0,91	0,90	0,82	pentatricopeptide (PPR) repeat-containing protein
259472_at	At1g18910	0,83	0,82	0,68	0,98	1,05	1,18	1,07	1,18	zinc finger (C3HC4-type RING finger) family protein
259462_at	At1g18940	0,89	0,98	0,91	0,97	0,97	0,96	1,03	0,96	nodulin family protein

259463_at	At1g18950	1,14	1,41	1,91	0,84	0,99	1,03	1,11	1,11	aminoacyl-tRNA synthetase family
259481_at	At1g18970	2,68	3,66	2,08	1,14	0,85	1,36	1,42	1,32	germin-like protein (GLP1) (GLP4)
259478_at	At1g18980	1,31	1,53	1,11	1,95	1,26	1,62	1,42	1,23	germin-like protein, putative
259464_at	At1g18990	1,20	1,11	1,09	0,91	0,97	0,97	1,08	1,09	expressed protein
259476_at	At1g19000	0,79	1,03	1,07	0,88	1,00	0,92	0,96	0,95	myb family transcription factor
259480_at	At1g19010	0,90	1,00	1,16	0,99	1,15	1,10	1,24	0,97	expressed protein
259479_at	At1g19020	0,89	0,76	0,92	0,89	0,98	1,05	1,09	1,33	expressed protein
259473_at	At1g19025	0,83	0,91	0,80	0,71	0,86	1,01	0,96	0,97	DNA cross-link repair protein-related
259465_at	At1g19030	0,97	1,03	1,06	1,05	1,08	0,90	0,96	1,07	hypothetical protein
259471_at	At1g19040	0,90	1,00	0,99	0,93	1,06	0,97	0,91	0,91	hypothetical protein
259466_at	At1g19050	1,41	2,04	1,53	0,76	1,01	1,18	1,64	1,11	two-component responsive regulator / response regulator 7 (ARR7)
259467_at	At1g19060	0,99	1,06	0,96	1,02	1,04	1,03	0,97	1,02	hypothetical protein
259468_at	At1g19080	1,20	1,20	1,32	1,02	1,15	1,16	1,38	1,69	expressed protein
259482_at	At1g19090	1,04	1,00	1,01	0,95	1,22	0,98	1,01	0,86	serine/threonine protein kinase (RKF2)
259469_at	At1g19100	0,94	1,06	1,02	0,91	0,89	1,08	0,96	1,05	ATP-binding region, ATPase-like domain-containing protein-related
259470_at	At1g19110	0,89	0,94	1,02	1,06	0,93	0,96	1,03	1,09	inter-alpha-trypsin inhibitor heavy chain-related
259477_at	At1g19120	0,86	0,95	0,99	0,27	0,22	0,89	0,91	0,93	small nuclear ribonucleoprotein, putative / snRNP, putative / Sm protein, pu
259474_at	At1g19130	0,76	0,67	0,62	1,21	1,17	0,81	0,80	0,83	expressed protein
259475_at	At1g19140	1,19	1,19	1,09	1,06	1,08	1,20	1,14	1,09	expressed protein
256015_at	At1g19150	0,94	1,05	1,24	0,67	0,64	1,30	1,27	1,26	chlorophyll A-B binding protein, putative / LHCI type II, putative
256037_at	At1g19160	0,93	0,97	1,04	0,90	0,89	0,89	1,01	0,99	F-box family protein-related
256038_at	At1g19170	1,11	0,91	0,89	1,08	0,99	0,86	0,83	0,87	glycoside hydrolase family 28 protein / polygalacturonase (pectinase) family
256017_at	At1g19180	0,96	1,42	1,23	1,09	1,11	1,20	1,57	1,59	expressed protein
256039_at	At1g19190	0,99	1,03	0,99	1,03	1,08	1,23	1,15	1,04	expressed protein
256014_at	At1g19200	0,85	0,93	0,90	1,38	1,11	1,03	0,91	1,03	senescence-associated protein-related
256009_at	At1g19210	0,76	0,95	0,95	1,07	0,93	1,72	1,05	1,09	AP2 domain-containing transcription factor, putative
256010_at	At1g19220	0,99	1,05	1,07	0,85	0,99	1,02	1,10	1,23	transcriptional factor B3 family protein / auxin-responsive factor AUX/IAA-re
256011_at	At1g19230	1,01	1,08	1,09	1,12	0,98	1,01	1,05	1,13	respiratory burst oxidase protein E (RbohE) / NADPH oxidase
256016_at	At1g19240	1,22	1,22	1,31	1,19	1,04	0,99	0,88	0,95	expressed protein
256012_at	At1g19250	1,24	1,58	2,05	3,42	2,45	2,70	2,28	3,47	flavin-containing monooxygenase family protein / FMO family protein
256013_at	At1g19270	0,79	0,96	1,00	1,03	0,99	1,16	1,55	1,29	ubiquitin interaction motif-containing protein / LIM domain-containing protei
260654_at	At1g19290	0,96	0,98	1,04	0,84	0,84	0,95	0,93	1,07	pentatricopeptide (PPR) repeat-containing protein
260666_at	At1g19300	0,57	0,53	0,57	0,76	0,92	0,71	0,71	0,91	glycosyl transferase family 8 protein
260671_at	At1g19310	0,76	0,86	0,93	0,89	0,97	0,86	0,91	1,18	zinc finger (C3HC4-type RING finger) family protein
260655_at	At1g19320	1,00	0,77	0,81	0,89	1,05	0,83	0,70	0,63	pathogenesis-related thaumatin family protein
260673_at	At1g19330	0,69	0,87	1,19	0,77	0,74	1,04	1,23	0,94	expressed protein
260669_at	At1g19340	1,47	1,45	1,26	0,78	0,86	1,34	1,29	1,34	methyltransferase MT-A70 family protein
260665_at	At1g19360	1,11	0,79	0,81	1,31	1,12	0,92	0,83	0,86	expressed protein
260674_at	At1g19370	0,62	0,59	0,59	0,87	1,09	0,72	0,81	0,73	expressed protein
260656_at	At1g19380	0,84	1,14	0,83	0,92	1,18	1,06	0,99	1,08	expressed protein
260657_at	At1g19390	0,97	1,04	0,96	0,95	0,95	0,95	0,98	1,09	wall-associated kinase, putative
260658_at	At1g19410	1,00	0,92	1,08	0,98	1,08	0,93	1,06	0,94	hypothetical protein
260675_at	At1g19430	0,66	0,64	0,67	0,95	1,10	0,74	0,80	0,79	dehydration-responsive protein-related
260667_at	At1g19440	1,36	1,06	1,12	1,51	1,61	1,05	0,77	0,84	very-long-chain fatty acid condensing enzyme, putative
260676_at	At1g19450	1,42	1,07	1,10	1,19	1,25	1,35	1,39	1,29	integral membrane protein, putative / sugar transporter family protein
260659_s_at	At1g19470	0,91	1,04	1,00	0,95	1,18	1,04	1,03	1,07	kelch repeat-containing F-box family protein

260672_at	At1g19480	1,08	1,21	0,94	1,08	1,07	1,07	0,96	0,95	HhH-GPD base excision DNA repair family protein
260660_at	At1g19485	1,37	1,40	1,37	0,80	0,81	1,32	1,58	2,38	AT hook motif-containing protein
260661_at	At1g19500	0,99	1,07	0,90	0,97	1,24	0,99	1,19	0,91	hypothetical protein
260664_at	At1g19510	1,27	1,53	1,13	1,17	1,01	1,14	1,05	1,04	myb family transcription factor
260670_at	At1g19520	1,16	1,33	1,03	1,25	1,04	0,83	0,94	0,78	expressed protein
260663_at	At1g19525	1,17	1,18	1,18	1,02	1,23	0,85	0,78	0,73	pentatricopeptide (PPR) repeat-containing protein
260668_at	At1g19530	0,84	0,81	1,19	0,80	1,24	0,66	0,75	0,81	expressed protein
260662_at	At1g19540	0,69	0,65	0,55	0,90	0,82	0,63	0,63	0,56	isoflavone reductase, putative
261149_s_at	At1g19570	0,80	0,69	0,74	1,82	1,57	0,79	0,84	0,83	dehydroascorbate reductase, putative
261148_at	At1g19580	1,03	0,85	0,80	1,24	1,13	0,99	0,99	0,93	bacterial transferase hexapeptide repeat-containing protein
261136_at	At1g19600	1,10	1,02	1,08	0,85	0,84	1,01	1,13	1,06	pfkB-type carbohydrate kinase family protein
261135_at	At1g19610	0,79	0,74	0,94	1,14	0,78	0,78	0,86	0,83	plant defensin-fusion protein, putative (PDF1.4)
261146_at	At1g19620	0,87	0,93	1,10	1,12	1,01	0,86	1,02	0,94	expressed protein
261134_at	At1g19630	0,97	1,03	0,89	0,86	0,84	1,17	1,10	1,14	cytochrome P450 family protein
261150_at	At1g19640	0,99	1,05	1,03	1,09	1,14	0,98	0,92	0,99	S-adenosyl-L-methionine:jasmonic acid carboxyl methyltransferase (JMT)
261151_at	At1g19650	0,69	0,74	0,69	1,01	0,75	1,00	0,93	1,05	SEC14 cytosolic factor, putative / phosphoglyceride transfer protein, putativ
261144_s_at	At1g19660	0,88	1,13	1,42	0,80	0,82	1,12	1,40	1,66	wound-responsive family protein
255786_at	At1g19670	0,74	0,59	1,10	0,74	0,81	0,86	0,83	0,62	coronatine-responsive protein / coronatine-induced protein 1 (CORI1)
261140_at	At1g19680	0,96	1,06	1,14	0,84	0,83	1,04	1,03	1,23	expressed protein
261147_at	At1g19690	0,83	1,10	1,00	0,92	1,05	1,02	1,06	1,22	expressed protein
261139_at	At1g19700	1,34	1,58	1,61	0,72	0,85	1,49	1,22	1,33	homeobox-leucine zipper family protein
261138_at	At1g19710	1,16	1,24	1,14	1,34	1,27	1,45	1,23	0,94	glycosyl transferase family 1 protein
261133_at	At1g19720	2,34	2,26	2,18	0,69	0,82	1,56	1,92	1,49	jacalin lectin family protein
261145_at	At1g19730	1,27	1,43	1,34	1,42	1,34	1,00	1,10	0,94	thioredoxin H-type 4 (TRX-H-4) (GREN)
261141_at	At1g19740	0,86	0,87	1,01	0,62	0,64	1,05	0,89	0,83	ATP-dependent protease La (LON) domain-containing protein
261143_at	At1g19770	0,85	0,90	1,09	0,93	0,71	1,04	1,35	1,52	purine permease-related
261142_at	At1g19780	1,00	1,01	1,01	1,17	0,91	0,92	0,94	0,89	cyclic nucleotide-regulated ion channel, putative (CNGC8)
261132_at	At1g19800	1,07	0,98	1,04	0,81	0,77	0,93	0,99	1,03	expressed protein
261137_at	At1g19830	0,99	1,09	0,91	0,99	1,15	1,00	0,90	1,00	auxin-responsive protein, putative
261131_at	At1g19835	1,14	1,11	1,05	1,05	1,29	1,10	1,15	1,62	expressed protein
255782_at	At1g19850	1,36	1,18	0,94	0,98	0,96	0,80	0,98	1,03	transcription factor MONOPTEROS (MP) / auxin-responsive protein (IAA24
255784_at	At1g19860	1,17	1,30	1,49	0,89	0,94	1,07	1,14	1,06	zinc finger (CCCH-type) family protein
255783_at	At1g19870	0,98	1,10	1,17	0,89	1,05	0,99	1,02	1,24	calmodulin-binding family protein
255781_at	At1g19880	1,13	0,91	0,97	0,95	1,07	1,01	1,04	1,09	regulator of chromosome condensation (RCC1) family protein
255815_at	At1g19890	1,04	1,05	0,85	1,08	1,12	0,98	0,92	0,98	histone H3, putative
255814_at	At1g19900	0,86	0,93	0,93	1,11	1,05	1,08	0,95	0,98	glyoxal oxidase-related
255785_at	At1g19920	1,25	1,10	1,10	1,35	1,04	1,15	1,03	0,99	sulfate adenylyltransferase 2 / ATP-sulfurylase 2 (ASA1) (MET3-1) (APS2)
255813_at	At1g19930	1,07	1,00	1,13	0,94	1,08	0,90	1,09	1,04	kelch repeat-containing F-box family protein
255756_at	At1g19940	0,95	1,00	1,16	1,18	1,22	0,90	0,72	0,90	glycosyl hydrolase family 9 protein
261223_at	At1g19950	1,02	1,07	1,09	1,32	1,51	0,92	0,85	0,93	abscisic acid-responsive HVA22 family protein
261221_at	At1g19960	0,43	0,83	1,13	1,68	0,96	1,00	1,17	1,12	expressed protein
261220_at	At1g19970	0,93	0,88	0,86	1,25	1,23	0,78	0,94	0,86	ER lumen protein retaining receptor family protein
261219_at	At1g19980	1,10	0,91	0,87	0,93	1,02	0,97	0,95	1,10	cytomatrix protein-related
261230_at	At1g20010	0,95	0,90	0,94	0,68	0,71	0,90	0,90	0,85	tubulin beta-5 chain (TUB5)
261218_at	At1g20020	0,82	0,79	0,82	0,79	0,70	1,10	1,20	1,27	ferredoxin--NADP(+) reductase, putative / adrenodoxin reductase, putative
261248_at	At1g20030	0,85	0,87	0,85	0,87	0,89	1,07	0,91	1,11	pathogenesis-related thaumatin family protein

261228_at	At1g20050	1,03	0,91	0,84	0,99	1,04	0,95	0,86	0,90 C-8,7 sterol isomerase
261247_at	At1g20070	2,15	3,28	1,41	28,84	21,44	1,53	1,19	1,18 expressed protein
261246_at	At1g20080	0,87	1,18	1,02	0,95	1,18	1,07	1,03	1,04 C2 domain-containing protein
261229_at	At1g20090	1,07	0,88	1,01	1,44	1,24	0,96	0,97	1,03 Rac-like GTP-binding protein (ARAC4) / Rho-like GTP-binding protein (ROI
261225_at	At1g20100	0,98	1,36	1,51	0,82	1,00	1,25	1,62	1,20 expressed protein
261231_at	At1g20110	0,81	0,78	0,81	1,00	0,99	0,94	0,86	0,86 zinc finger (FYVE type) family protein
261222_at	At1g20120	1,05	1,07	1,51	2,06	1,75	0,94	1,19	1,12 family II extracellular lipase, putative
261245_at	At1g20130	1,05	1,05	1,05	1,05	0,90	0,97	0,96	1,10 family II extracellular lipase, putative
261244_at	At1g20150	1,08	1,01	1,02	1,05	1,16	1,03	0,91	0,84 subtilase family protein
261224_at	At1g20160	2,47	1,80	1,82	3,43	3,43	0,93	1,10	0,95 subtilase family protein
261243_at	At1g20180	1,16	1,15	1,05	0,95	0,99	0,95	1,17	1,12 hypothetical protein
261226_at	At1g20190	1,57	1,67	1,23	1,03	1,15	0,64	0,87	0,91 expansin, putative (EXP11)
261227_at	At1g20200	1,14	1,03	1,14	1,08	1,17	0,99	0,94	0,93 26S proteasome regulatory subunit S3, putative (RPN3)
261232_at	At1g20220	1,20	1,13	1,18	1,37	1,45	0,89	1,02	1,10 expressed protein
261277_at	At1g20230	1,00	1,17	1,31	0,82	0,94	1,08	0,95	1,16 pentatricopeptide (PPR) repeat-containing protein
255883_at	At1g20270	0,82	0,74	0,80	0,91	1,03	0,89	0,94	0,84 oxidoreductase, 2OG-Fe(II) oxygenase family protein
255882_x_at	At1g20280	1,04	1,01	0,90	1,09	1,02	0,86	0,88	0,80 homeobox-leucine zipper protein-related
255914_at	At1g20290	1,08	1,01	1,00	1,00	1,08	1,03	1,01	0,90 hypothetical protein
255888_at	At1g20300	0,76	0,68	0,87	0,69	0,79	0,86	0,76	0,69 pentatricopeptide (PPR) repeat-containing protein
255884_at	At1g20310	0,68	0,75	0,71	0,78	1,09	1,19	0,89	1,08 expressed protein
255885_at	At1g20330	0,99	0,86	0,90	1,12	1,12	0,81	0,75	0,77 S-adenosyl-methionine-sterol-C-methyltransferase
255886_at	At1g20340	1,06	1,01	0,97	1,03	0,93	1,04	0,94	0,97 plastocyanin
255941_at	At1g20350	0,86	0,69	0,80	1,08	1,20	0,84	0,72	0,75 mitochondrial import inner membrane translocase subunit Tim17, putative
255887_at	At1g20370	1,22	0,86	1,20	0,82	0,82	1,00	0,82	0,89 tRNA pseudouridine synthase family protein
255940_at	At1g20380	1,26	1,32	1,08	0,92	1,02	1,08	1,08	1,09 prolyl oligopeptidase, putative / prolyl endopeptidase, putative / post-proline
259573_at	At1g20390	0,92	1,17	0,97	0,42	0,42	0,91	1,07	0,88 gypsy-like retrotransposon family
259572_at	At1g20400	0,96	0,95	0,89	1,10	1,05	0,98	0,95	0,95 myosin heavy chain-related
259571_at	At1g20410	1,19	0,99	1,52	0,86	0,93	0,94	0,95	0,87 expressed protein
259515_at	At1g20430	1,34	1,31	1,25	1,37	1,41	0,93	0,93	0,86 expressed protein
259570_at	At1g20440	0,98	0,93	0,99	1,51	1,30	1,17	1,11	1,13 dehydrin (COR47)
259516_at	At1g20450	0,82	0,82	1,30	1,25	1,19	1,09	1,09	1,24 dehydrin (ERD10)
259569_at	At1g20480	1,05	1,08	0,96	1,00	1,22	1,04	0,85	1,15 4-coumarate--CoA ligase family protein / 4-coumaroyl-CoA synthase family
259568_at	At1g20490	0,76	0,73	0,73	0,92	0,78	0,94	1,06	1,07 AMP-dependent synthetase and ligase family protein
259567_at	At1g20500	0,94	0,95	0,95	1,00	1,06	1,19	1,04	0,91 4-coumarate--CoA ligase family / 4-coumaroyl-CoA synthase family
259518_at	At1g20510	0,65	0,72	1,28	1,07	1,03	1,04	1,16	1,15 4-coumarate--CoA ligase family protein / 4-coumaroyl-CoA synthase family
259566_at	At1g20520	1,31	1,14	0,91	1,11	0,99	1,01	1,08	0,87 expressed protein
259565_at	At1g20530	0,94	1,02	0,98	1,05	1,02	1,09	1,04	1,03 hypothetical protein
259564_at	At1g20540	0,81	0,82	0,79	0,90	0,95	0,91	0,97	0,90 transducin family protein / WD-40 repeat family protein
259539_at	At1g20550	0,86	1,01	1,12	0,95	0,99	1,01	0,93	0,84 expressed protein
259545_at	At1g20560	0,63	0,59	0,73	0,76	0,90	0,88	0,77	0,72 AMP-dependent synthetase and ligase family protein
259542_at	At1g20575	1,16	1,09	1,09	1,19	1,18	0,95	0,99	1,07 dolichyl-phosphate beta-D-mannosyltransferase, putative / dolichol-phospha
259543_at	At1g20580	1,27	1,16	1,04	1,13	1,08	1,11	0,98	0,90 small nuclear ribonucleoprotein, putative / snRNP, putative / Sm protein, pu
259563_s_at	At1g20590	1,53	1,47	1,58	1,85	1,34	0,92	1,06	0,96 cyclin, putative
259544_at	At1g20620	0,72	0,77	1,03	0,67	0,71	0,67	0,63	0,67 catalase 3 (SEN2)
259517_at	At1g20630	0,67	0,60	0,71	0,82	0,96	0,80	0,78	0,86 catalase 1
259540_at	At1g20640	1,24	1,08	1,04	0,88	1,20	1,08	0,79	0,93 RWP-RK domain-containing protein

259541_at	At1g20650	1,20	1,82	1,73	0,79	0,56	1,26	1,26	1,55	protein kinase family protein
256078_at	At1g20670	0,97	1,15	1,00	0,97	1,29	1,07	1,00	1,06	DNA-binding bromodomain-containing protein
256079_at	At1g20680	0,96	0,98	1,06	0,92	0,87	0,85	0,99	1,06	expressed protein
256080_at	At1g20690	1,05	0,97	1,02	0,89	0,89	1,13	0,89	0,94	expressed protein
256091_at	At1g20693	0,74	0,63	0,66	1,02	1,15	0,95	0,87	0,84	high mobility group protein beta1 (HMGbeta1) / HMG protein beta1
256092_at	At1g20696	0,80	0,76	0,77	0,94	1,10	0,99	0,98	0,95	high mobility group protein beta2 (HMGbeta2) / HMG protein beta2
256081_at	At1g20700	1,03	0,91	0,91	1,02	1,09	0,99	0,98	1,12	homeobox-leucine zipper family protein
256082_at	At1g20720	0,99	1,06	1,03	1,04	1,09	1,02	1,15	0,91	helicase-related
256083_at	At1g20730	0,94	0,91	1,08	1,00	1,10	1,07	0,84	1,08	expressed protein
256084_at	At1g20750	1,07	0,98	1,02	0,93	1,15	0,95	0,95	1,02	helicase-related
256085_at	At1g20760	0,74	0,85	1,05	0,96	0,91	1,04	0,80	0,91	calcium-binding EF hand family protein
256086_at	At1g20770	0,52	0,50	0,50	1,17	1,19	0,81	0,72	0,70	expressed protein
256094_at	At1g20780	0,78	0,74	0,79	0,95	1,01	0,99	0,91	0,92	armadillo/beta-catenin repeat protein-related / U-box domain-containing pro
256087_at	At1g20800	0,97	0,92	0,84	1,10	1,00	0,96	0,96	0,89	F-box family protein
256088_at	At1g20810	0,80	0,84	0,80	0,68	0,81	1,00	0,96	0,92	immunophilin / FKBP-type peptidyl-prolyl cis-trans isomerase family protein
256090_at	At1g20816	1,17	1,03	0,75	0,87	1,00	1,11	0,93	0,93	expressed protein
256093_at	At1g20823	0,68	1,05	1,00	1,18	0,75	1,25	1,51	1,42	zinc finger (C3HC4-type RING finger) family protein
256089_at	At1g20830	1,13	1,22	1,13	0,88	0,83	1,23	0,96	1,14	expressed protein
262797_at	At1g20840	0,92	0,90	0,83	0,82	0,72	0,95	0,85	0,84	transporter-related
262796_at	At1g20850	1,78	1,69	1,45	1,36	1,24	1,13	1,17	1,25	cysteine endopeptidase, papain-type (XCP2)
262855_at	At1g20860	0,95	0,95	1,18	1,15	0,89	0,96	0,93	1,01	phosphate transporter family protein
262854_at	At1g20870	1,13	1,10	0,98	1,05	0,97	0,93	1,10	1,29	expressed protein
262804_at	At1g20880	0,91	1,09	1,07	0,77	0,90	1,05	0,91	1,19	RNA recognition motif (RRM)-containing protein
262853_at	At1g20890	0,85	0,79	0,84	0,95	0,77	0,73	0,70	0,95	expressed protein
262805_at	At1g20900	1,07	1,03	1,16	1,12	1,10	0,87	0,97	0,88	DNA-binding protein-related
262852_at	At1g20910	0,93	1,06	1,23	0,92	0,95	1,14	1,07	1,00	ARID/BRIGHT DNA-binding domain-containing protein
262851_at	At1g20920	1,69	1,48	1,52	0,87	0,89	1,30	1,29	1,54	DEAD box RNA helicase, putative
262802_at	At1g20930	1,27	1,21	1,27	2,06	1,90	0,95	0,89	0,80	cell division control protein, putative
257464_at	At1g20940	1,02	0,97	0,97	1,03	1,00	1,11	1,01	0,99	F-box family protein
262806_at	At1g20950	0,92	0,83	0,81	1,32	1,12	0,84	0,87	0,92	pyrophosphate--fructose-6-phosphate 1-phosphotransferase-related / pyrop
262800_at	At1g20960	1,36	1,05	1,12	1,13	0,99	1,08	1,03	0,90	U5 small nuclear ribonucleoprotein helicase, putative
262799_at	At1g20970	1,02	0,93	1,00	1,16	1,10	0,88	1,09	1,07	adhesin-related
262798_at	At1g20980	0,89	0,84	0,89	1,14	1,13	0,89	0,90	0,87	SPL1-Related2 protein (SPL1R2)
262803_at	At1g21000	0,78	1,20	1,07	0,64	0,56	1,15	1,61	1,72	zinc-binding family protein
262801_at	At1g21010	0,74	0,75	0,78	0,88	0,97	1,01	0,97	1,23	expressed protein
261452_s_at	At1g21020	0,95	1,06	1,04	1,00	1,08	0,97	0,93	0,85	Ulp1 protease family protein
261456_at	At1g21050	1,00	1,06	0,94	1,26	1,38	0,76	0,85	1,00	expressed protein
261451_at	At1g21060	1,04	1,07	0,98	0,90	1,01	1,04	0,98	1,04	expressed protein
261457_at	At1g21065	0,91	0,81	0,82	1,03	1,11	0,97	0,97	0,91	expressed protein
261455_at	At1g21070	0,75	0,71	0,72	1,29	1,23	0,80	0,88	0,79	transporter-related
261458_at	At1g21080	0,82	0,76	0,81	1,25	1,21	0,95	0,90	1,00	DNAJ heat shock N-terminal domain-containing protein
261454_at	At1g21090	0,74	1,05	0,97	1,07	1,01	0,90	1,01	1,12	hydroxyproline-rich glycoprotein family protein
261459_at	At1g21100	1,03	1,40	1,59	0,33	0,24	0,99	0,99	0,90	O-methyltransferase, putative
261450_s_at	At1g21110	1,50	1,83	1,99	0,61	0,40	1,38	0,95	0,81	O-methyltransferase, putative
261449_at	At1g21120	1,02	1,27	1,56	0,62	0,58	1,38	1,13	0,93	O-methyltransferase, putative
261453_at	At1g21130	1,02	1,07	1,28	0,52	0,36	0,87	0,83	0,99	O-methyltransferase, putative

261448_at	At1g21140	0,96	1,09	1,12	1,02	0,81	0,97	1,23	1,62	nodulin, putative
261447_at	At1g21160	0,81	0,85	0,96	0,95	0,86	1,10	1,10	1,07	eukaryotic translation initiation factor 2 family protein / eIF-2 family protein
261446_at	At1g21170	0,99	0,79	0,80	1,16	1,16	0,92	0,76	1,00	expressed protein
259551_at	At1g21190	1,83	1,93	1,72	2,29	2,20	0,97	0,98	0,83	small nuclear ribonucleoprotein, putative / snRNP, putative / Sm protein, pu
259562_at	At1g21200	0,89	0,95	0,65	1,07	1,05	0,98	0,82	0,61	expressed protein
259557_at	At1g21210	0,95	0,73	1,12	1,01	1,00	0,98	0,93	1,24	wall-associated kinase 4
259554_at	At1g21220	1,00	1,28	1,00	0,92	1,11	1,04	1,00	0,85	copia-like retrotransposon family
259558_at	At1g21230	1,09	1,07	1,06	0,94	1,03	0,98	1,05	1,12	wall-associated kinase, putative
259559_at	At1g21240	1,02	1,06	1,01	0,93	0,98	1,01	1,18	1,12	wall-associated kinase, putative
259561_at	At1g21250	0,45	0,42	0,65	1,03	0,91	3,58	2,22	2,33	wall-associated kinase 1 (WAK1)
259560_at	At1g21270	0,69	0,80	1,04	0,83	0,96	0,98	1,08	1,16	wall-associated kinase 2 (WAK2)
259555_at	At1g21290	0,97	1,03	1,01	0,99	1,22	0,98	0,92	1,01	---
259556_at	At1g21300	0,90	0,94	1,07	1,06	1,23	1,05	0,91	0,88	---
259553_x_at	At1g21310	1,23	1,43	0,89	1,23	1,49	1,29	1,40	1,23	proline-rich extensin-like family protein
259552_at	At1g21320	0,98	1,02	0,95	1,13	0,93	0,89	0,99	1,04	RNA recognition motif (RRM)-containing protein
260925_at	At1g21340	0,96	0,99	1,03	1,12	1,09	0,86	0,95	1,12	Dof-type zinc finger domain-containing protein
260872_at	At1g21350	0,74	0,75	0,72	0,86	0,74	0,94	0,92	0,84	expressed protein
260926_at	At1g21360	1,23	1,12	1,13	0,86	1,04	0,93	1,06	0,95	expressed protein
260899_at	At1g21370	0,93	0,96	0,84	1,11	1,11	1,02	0,89	0,96	expressed protein
260880_at	At1g21380	0,85	0,86	1,21	1,32	1,28	1,09	1,03	1,22	VHS domain-containing protein / GAT domain-containing protein
260874_at	At1g21390	0,94	0,98	1,11	0,83	0,88	1,08	1,12	0,93	expressed protein
260900_s_at	At1g21400	1,31	1,38	2,19	0,86	0,82	0,92	1,01	1,10	2-oxoisovalerate dehydrogenase, putative / 3-methyl-2-oxobutanoate dehyc
260875_at	At1g21410	0,95	1,33	1,66	0,91	0,79	1,18	1,53	1,29	F-box family protein
260901_at	At1g21430	1,00	1,03	1,00	0,91	0,97	0,93	1,01	1,10	flavin-containing monooxygenase family protein / FMO family protein
260902_at	At1g21440	1,08	1,05	1,01	1,20	1,07	0,87	0,87	0,80	mutase family protein
260878_at	At1g21450	0,82	1,11	1,48	0,95	0,99	1,05	1,38	1,31	scarecrow-like transcription factor 1 (SCL1)
260876_at	At1g21460	1,29	1,10	1,30	1,48	1,23	1,16	1,07	1,08	nodulin MtN3 family protein
260879_at	At1g21480	1,03	1,15	1,00	0,83	0,83	1,04	0,94	1,04	exostosin family protein
260877_at	At1g21500	0,98	0,99	1,01	0,90	0,95	0,99	0,90	0,91	expressed protein
260918_at	At1g21510	0,93	1,04	1,33	1,20	1,08	0,90	1,07	0,81	hypothetical protein
260919_at	At1g21520	1,08	0,93	0,77	0,77	0,92	1,09	0,99	0,87	expressed protein
260920_at	At1g21530	1,02	1,13	1,16	0,94	0,77	0,99	1,11	0,91	AMP-binding protein, putative
260921_at	At1g21540	0,96	0,91	0,96	1,03	1,06	0,87	0,69	0,88	AMP-binding protein, putative
260881_at	At1g21550	1,35	1,62	1,16	2,04	1,60	1,96	1,80	1,38	calcium-binding protein, putative
260922_at	At1g21560	1,17	1,02	1,18	1,09	1,13	0,99	1,12	0,91	expressed protein
260923_at	At1g21570	0,90	0,80	0,95	0,81	0,86	1,01	1,17	1,33	zinc finger (CCCH-type) family protein
260873_at	At1g21580	0,90	0,72	0,98	0,86	0,92	1,28	1,16	1,01	hydroxyproline-rich glycoprotein family protein
260924_at	At1g21590	1,90	2,53	2,11	0,76	0,67	1,31	1,34	1,07	protein kinase family protein
262502_at	At1g21600	0,89	0,96	1,09	0,60	0,72	1,11	0,97	0,97	expressed protein
262487_at	At1g21610	1,23	1,64	2,00	0,90	1,00	1,15	1,21	1,21	wound-responsive family protein
262492_at	At1g21630	0,96	0,91	0,97	1,01	1,19	0,92	0,86	0,95	calcium-binding EF hand family protein
262506_at	At1g21640	0,94	1,01	1,24	0,79	0,79	1,13	1,11	1,21	ATP-NAD kinase family protein
262491_at	At1g21650	1,27	0,97	1,03	0,78	0,65	1,21	1,16	1,01	preprotein translocase secA family protein
262493_at	At1g21660	1,02	1,00	1,24	1,00	1,22	1,13	0,95	0,98	expressed protein
262503_at	At1g21670	0,57	0,48	0,63	1,00	1,22	0,69	0,63	0,65	expressed protein
262505_at	At1g21680	0,51	0,50	0,59	0,49	0,40	0,81	0,81	0,98	expressed protein

262501_at	At1g21690	1,27	1,25	1,36	1,04	0,88	1,04	1,03	1,07	replication factor C 37 kDa, putative
262484_at	At1g21700	1,06	1,17	1,16	0,96	1,18	1,13	0,91	1,07	SWIRM domain-containing protein / DNA-binding family protein
262498_at	At1g21710	1,23	1,27	1,17	1,11	1,02	1,00	0,91	0,90	8-oxoguanine-DNA glycosylase (OGG1)
262497_at	At1g21720	1,07	1,08	1,00	1,29	1,21	0,97	0,93	0,85	20S proteasome beta subunit C1 (PBC1) (PRCT)
262485_at	At1g21730	1,08	1,04	1,16	0,99	0,96	1,17	1,19	1,41	kinesin-related protein (MKRP1)
262486_at	At1g21740	0,91	0,95	0,89	0,95	1,22	1,05	1,05	1,02	expressed protein
262504_at	At1g21750	1,11	0,96	0,87	1,28	1,34	0,88	0,86	0,91	protein disulfide isomerase, putative
262500_at	At1g21760	1,09	1,17	1,44	1,06	0,94	1,10	1,00	1,02	F-box family protein
262499_at	At1g21770	1,10	0,87	0,83	1,12	1,36	1,01	1,01	0,97	expressed protein
262495_at	At1g21780	0,79	0,76	0,96	0,90	1,17	0,92	1,05	1,15	BTB/POZ domain-containing protein
262496_at	At1g21790	1,15	1,06	1,18	0,91	0,97	1,15	1,16	1,11	expressed protein
262494_at	At1g21810	1,20	1,00	1,06	1,11	1,12	1,01	1,01	1,04	expressed protein
262488_at	At1g21820	1,50	1,66	1,64	0,73	1,03	0,98	1,07	1,09	expressed protein
262489_at	At1g21830	1,18	1,18	1,12	1,07	1,30	0,87	0,99	1,06	expressed protein
262490_at	At1g21840	1,12	0,91	1,04	1,24	1,37	1,09	0,78	0,83	expressed protein
260848_at	At1g21850	0,97	0,97	0,91	1,04	1,05	1,16	0,93	0,79	multi-copper oxidase type I family protein
260849_at	At1g21860	0,97	1,03	0,95	0,96	1,03	0,98	0,97	1,00	multi-copper oxidase type I family protein
260850_at	At1g21870	0,90	1,00	1,08	0,98	0,87	0,94	1,09	0,85	glucose-6-phosphate/phosphate translocator-related
260857_at	At1g21880	0,95	0,88	0,91	0,92	1,07	0,79	0,75	0,74	peptidoglycan-binding LysM domain-containing protein
260851_at	At1g21890	1,03	1,02	1,05	1,07	0,99	0,97	0,84	1,21	nodulin MtN21 family protein
260852_at	At1g21900	1,03	0,88	0,87	1,19	0,99	1,02	0,89	0,92	emp24/gp25L/p24 family protein
260856_at	At1g21910	1,20	1,51	1,20	1,17	1,24	1,19	1,59	1,44	AP2 domain-containing transcription factor family protein
260855_at	At1g21920	1,05	1,13	0,90	0,74	0,75	0,99	1,41	1,19	MORN (Membrane Occupation and Recognition Nexus) repeat-containing p
260853_at	At1g21950	0,94	0,99	1,08	0,95	1,21	0,90	0,93	0,78	hypothetical protein
260854_at	At1g21970	0,98	1,03	1,02	1,05	0,95	1,02	0,94	0,93	CCAAT-box binding transcription factor (LEC1)
255959_at	At1g21980	1,00	0,94	0,96	1,20	1,03	1,11	1,26	1,51	1-phosphatidylinositol-4-phosphate 5-kinase, putative / PIP kinase, putative
255976_at	At1g22010	0,88	0,88	1,15	1,09	1,18	0,99	0,91	1,03	hypothetical protein
255956_at	At1g22015	0,93	0,92	1,23	1,00	1,00	0,93	1,04	1,02	galactosyltransferase family protein
255946_at	At1g22020	1,16	1,11	1,25	0,93	0,93	0,90	0,97	1,01	glycine hydroxymethyltransferase, putative / serine hydroxymethyltransfera
255955_at	At1g22030	0,96	0,90	0,70	1,14	1,09	1,10	0,92	0,67	expressed protein
255947_at	At1g22040	1,26	1,18	1,18	0,90	1,09	1,07	1,04	0,80	kelch repeat-containing F-box family protein
255951_at	At1g22050	1,16	0,89	0,83	0,92	0,84	1,14	1,15	1,18	ubiquitin family protein
255948_at	At1g22060	1,01	0,89	1,00	0,79	0,80	1,13	1,02	1,32	expressed protein
255953_at	At1g22070	1,08	1,10	1,17	0,99	1,10	1,02	0,99	1,17	bZIP family transcription factor (TGA3)
255949_at	At1g22080	1,00	1,02	1,14	1,13	1,03	0,95	0,99	0,90	hypothetical protein
255954_at	At1g22090	1,01	0,91	1,20	1,10	0,99	1,03	1,05	0,80	expressed protein
255950_at	At1g22110	1,00	0,89	0,93	1,17	1,08	0,96	1,03	0,93	expressed protein
255952_at	At1g22130	0,97	1,09	1,05	0,95	0,94	1,01	1,02	1,21	MADS-box family protein
255960_at	At1g22140	1,31	0,99	0,93	1,10	1,17	1,21	1,12	1,00	expressed protein
255958_at	At1g22150	1,07	1,06	0,85	1,06	1,05	1,03	0,96	1,00	sulfate transporter (Sultr1;3)
255957_at	At1g22160	1,41	1,54	1,31	0,98	1,26	1,26	1,00	1,09	senescence-associated protein-related
255924_at	At1g22170	0,98	0,86	0,79	1,21	1,08	0,90	0,86	0,87	phosphoglycerate/bisphosphoglycerate mutase family protein
255923_at	At1g22180	0,65	0,65	0,83	1,01	1,06	1,08	1,15	1,57	SEC14 cytosolic factor family protein / phosphoglyceride transfer family pro
255926_at	At1g22190	1,41	1,35	1,13	1,77	1,01	1,27	1,86	1,79	AP2 domain-containing transcription factor, putative
255925_at	At1g22200	1,05	0,91	0,83	0,94	0,82	0,90	0,99	0,86	expressed protein
255922_at	At1g22210	0,96	1,07	1,08	1,12	1,00	0,91	1,01	1,07	trehalose-6-phosphate phosphatase, putative

255921_at	At1g22240	0,89	1,03	1,00	1,15	1,06	0,91	1,10	1,21	pumilio/Puf RNA-binding domain-containing protein
255968_at	At1g22270	1,24	0,96	0,83	1,17	1,29	0,87	0,86	0,73	expressed protein
255964_at	At1g22275	1,02	0,98	0,87	0,99	1,25	0,94	0,90	1,13	expressed protein
255967_at	At1g22280	0,72	0,65	0,91	0,85	0,89	0,92	1,09	1,35	protein phosphatase 2C, putative / PP2C, putative
255965_at	At1g22290	1,16	1,05	1,03	1,06	1,18	0,92	0,88	0,91	14-3-3 protein GF14, putative (GRF10)
255966_at	At1g22300	1,04	0,95	0,93	1,43	1,35	0,91	0,87	0,93	14-3-3 protein GF14 epsilon (GRF10)
255963_at	At1g22310	0,68	0,45	0,96	0,69	0,99	1,08	1,06	1,20	DNA-binding protein-related
255969_at	At1g22330	1,42	1,09	0,86	1,05	1,14	0,84	1,00	0,98	RNA recognition motif (RRM)-containing protein
255962_at	At1g22335	1,66	1,09	0,91	0,82	1,25	0,86	1,00	0,91	expressed protein
255961_at	At1g22340	0,87	0,95	1,00	0,94	1,12	1,01	1,04	1,07	UDP-glucoronosyl/UDP-glucosyl transferase family protein
255942_at	At1g22360	0,76	0,73	0,86	1,04	1,00	1,09	1,29	1,48	UDP-glucoronosyl/UDP-glucosyl transferase family protein
255943_at	At1g22370	0,65	0,94	1,12	0,61	0,66	1,02	1,05	0,94	UDP-glucoronosyl/UDP-glucosyl transferase family protein
261923_at	At1g22380	0,73	0,77	0,83	0,91	0,94	1,16	1,01	1,00	UDP-glucoronosyl/UDP-glucosyl transferase family protein
261934_at	At1g22400	0,50	0,53	0,76	0,80	0,95	0,99	0,78	0,78	UDP-glucoronosyl/UDP-glucosyl transferase family protein
261933_at	At1g22410	1,24	0,99	1,05	1,49	1,16	1,02	1,01	1,08	2-dehydro-3-deoxyphosphoheptonate aldolase, putative / 3-deoxy-D-arabin
261932_at	At1g22420	1,04	0,99	0,97	0,91	1,08	0,95	0,90	0,85	hydroxyproline-rich glycoprotein family protein
261931_at	At1g22430	1,14	1,07	1,41	0,66	0,71	1,20	1,33	1,55	alcohol dehydrogenase, putative
261930_at	At1g22440	0,96	1,00	0,85	1,63	1,45	1,22	1,54	1,27	alcohol dehydrogenase, putative
261939_at	At1g22450	1,07	1,02	0,92	1,22	1,21	1,01	0,90	0,97	cytochrome c oxidase subunit 6b, putative (COX6b)
261929_at	At1g22460	1,03	0,97	0,99	1,00	1,02	1,02	1,02	0,99	expressed protein
261928_at	At1g22480	0,50	0,51	0,61	1,07	1,13	0,96	0,91	0,90	plastocyanin-like domain-containing protein
261941_at	At1g22490	0,65	0,70	0,89	0,77	0,91	0,98	1,16	1,03	basic helix-loop-helix (bHLH) family protein
261927_at	At1g22500	0,58	0,58	0,56	1,06	1,15	0,65	0,63	0,76	zinc finger (C3HC4-type RING finger) family protein
261938_at	At1g22510	0,62	0,67	0,95	0,88	0,95	0,82	0,98	1,18	zinc finger (C3HC4-type RING finger) family protein
261940_at	At1g22520	1,06	1,01	0,87	1,06	1,17	0,86	0,92	0,86	expressed protein
261926_at	At1g22530	0,88	0,80	0,81	0,92	0,84	1,13	1,12	1,21	SEC14 cytosolic factor family protein / phosphoglyceride transfer family pro
261925_at	At1g22540	0,73	0,74	0,92	0,96	0,93	0,80	0,89	0,90	proton-dependent oligopeptide transport (POT) family protein
261924_at	At1g22550	0,74	1,01	1,21	0,94	0,84	0,82	1,22	1,34	proton-dependent oligopeptide transport (POT) family protein
261937_at	At1g22570	0,68	0,75	1,20	0,86	0,80	1,03	1,37	1,55	proton-dependent oligopeptide transport (POT) family protein
261942_at	At1g22590	0,82	0,76	0,73	1,13	1,11	0,96	0,89	1,01	MADS-box family protein
261936_at	At1g22600	0,87	1,01	1,01	0,99	0,96	1,01	1,01	0,82	hypothetical protein
261935_at	At1g22610	0,97	0,93	0,94	1,08	1,40	0,94	0,79	0,81	C2 domain-containing protein
264197_at	At1g22620	0,82	0,82	0,86	0,85	0,90	0,97	0,94	0,97	phosphoinositide phosphatase family protein
264201_at	At1g22630	1,36	1,36	1,67	0,91	0,83	1,22	1,19	1,31	expressed protein
264210_at	At1g22640	1,87	1,29	1,15	0,68	0,83	0,92	1,12	1,02	myb family transcription factor (MYB4)
264200_at	At1g22650	0,74	0,80	1,08	0,85	0,69	0,78	0,66	0,82	beta-fructofuranosidase, putative / invertase, putative / saccharase, putative
264212_at	At1g22660	1,11	1,21	1,14	0,81	0,73	1,05	1,03	1,16	tRNA-nucleotidyltransferase, putative / tRNA adenylyltransferase, putative
264196_at	At1g22670	1,03	1,13	1,03	0,90	0,99	0,94	0,95	0,98	protease-associated zinc finger (C3HC4-type RING finger) family protein
264195_at	At1g22690	0,58	0,54	0,47	0,79	0,79	0,68	0,72	0,74	gibberellin-responsive protein, putative
264199_at	At1g22700	0,89	0,78	0,90	0,73	0,83	1,00	0,95	0,86	tetratricopeptide repeat (TPR)-containing protein
264204_at	At1g22710	1,22	2,12	3,36	1,42	1,56	0,94	1,08	1,15	sucrose transporter / sucrose-proton symporter (SUC2)
264194_at	At1g22720	0,96	1,07	0,93	1,08	1,06	1,03	0,92	1,03	wall-associated kinase, putative
264206_at	At1g22730	0,93	0,82	0,79	0,86	1,00	1,07	0,97	0,93	MA3 domain-containing protein
264209_at	At1g22740	0,87	0,74	0,73	0,68	0,68	0,76	0,74	0,85	Ras-related protein (RAB7) / AtRab75 / small GTP-binding protein, putative
264207_at	At1g22750	1,00	0,97	0,90	1,03	0,88	1,12	0,99	1,10	expressed protein
264208_at	At1g22760	0,98	0,93	0,92	1,06	1,00	1,03	0,98	0,85	polyadenylate-binding protein 3 (PABP3)

264211_at	At1g22770	0,80	1,04	1,32	0,66	0,61	0,89	0,95	0,98	gigantea protein (GI)
264203_at	At1g22780	1,39	1,19	1,16	1,23	1,40	1,00	0,98	0,92	40S ribosomal protein S18 (RPS18A)
264205_at	At1g22790	0,88	0,95	1,04	1,19	1,04	0,85	0,81	0,84	expressed protein
264198_at	At1g22800	0,94	0,92	1,17	1,25	1,08	1,08	0,94	0,90	expressed protein
264202_at	At1g22810	0,57	0,80	0,77	0,74	0,76	1,38	1,15	1,05	AP2 domain-containing transcription factor, putative
264749_at	At1g22830	0,79	0,84	1,07	0,82	0,89	1,26	1,60	2,09	pentatricopeptide (PPR) repeat-containing protein
264727_at	At1g22840	1,31	1,07	0,99	1,20	1,16	1,00	0,98	1,00	cytochrome c, putative
264728_at	At1g22850	1,09	1,12	1,11	1,01	0,84	1,19	1,15	1,24	expressed protein
264776_at	At1g22860	1,05	1,06	1,11	0,98	0,94	1,14	1,11	1,27	TGF beta receptor associated protein-related
264750_at	At1g22870	0,89	0,84	1,25	0,89	1,02	0,95	1,35	1,19	protein kinase family protein
264775_at	At1g22880	1,21	1,07	1,19	1,12	1,19	0,97	0,95	0,90	glycosyl hydrolase family 9 protein
264725_at	At1g22885	1,11	0,95	0,93	1,23	1,12	0,91	0,82	0,83	expressed protein
264774_at	At1g22890	0,78	0,71	0,71	0,85	0,63	0,91	0,75	0,82	expressed protein
264773_at	At1g22900	1,27	0,88	0,69	1,06	0,99	1,06	1,00	0,95	disease resistance-responsive family protein
257413_at	At1g22910	1,29	1,42	1,41	1,03	1,06	0,95	0,96	1,06	RNA recognition motif (RRM)-containing protein
264724_at	At1g22920	1,13	0,93	1,07	1,07	1,12	0,94	0,83	0,91	COP9 signalosome subunit 5B / CSN subunit 5B (CSN5B) / c-JUN coactiva
264772_at	At1g22930	0,74	1,11	1,67	0,86	0,75	1,12	1,44	1,70	T-complex protein 11
264771_at	At1g22940	0,97	0,97	1,02	0,86	0,93	1,11	0,98	1,04	thiamin biosynthesis protein, putative
264723_at	At1g22960	0,95	0,87	0,97	0,94	0,81	0,97	0,80	1,20	pentatricopeptide (PPR) repeat-containing protein
264722_at	At1g22970	0,91	0,78	0,95	0,95	1,33	0,81	0,83	0,98	expressed protein
257412_at	At1g22980	1,10	1,24	1,30	1,00	1,01	1,02	1,19	1,00	expressed protein
264726_at	At1g22985	0,82	1,27	1,21	0,86	0,90	1,23	1,45	1,31	AP2 domain-containing transcription factor, putative
264729_at	At1g22990	1,04	1,12	1,22	1,21	1,29	1,40	1,58	1,62	heavy-metal-associated domain-containing protein / copper chaperone (CC
264721_at	At1g23000	0,91	1,05	0,90	0,84	1,22	0,86	0,94	0,87	heavy-metal-associated domain-containing protein
264752_at	At1g23010	0,81	0,91	0,99	1,32	1,11	1,17	1,04	0,95	multi-copper oxidase type I family protein
264751_at	At1g23020	1,54	1,56	1,68	1,65	1,55	2,00	1,62	1,06	ferric-chelate reductase, putative
264770_at	At1g23030	1,70	2,13	1,84	1,38	1,73	0,97	1,35	1,48	armadillo/beta-catenin repeat family protein / U-box domain-containing prot
264894_at	At1g23040	1,10	0,91	0,79	1,92	1,32	1,00	0,87	0,88	hydroxyproline-rich glycoprotein family protein
264889_at	At1g23050	0,89	0,85	0,89	1,10	0,87	0,86	0,74	0,91	hydroxyproline-rich glycoprotein family protein
264902_at	At1g23060	0,97	1,08	1,10	0,89	1,06	0,96	1,05	0,81	expressed protein
264888_at	At1g23070	0,81	0,86	0,77	0,96	0,87	1,12	1,02	1,02	hypothetical protein
264900_at	At1g23080	1,10	1,57	1,63	0,63	0,63	1,13	1,03	1,22	auxin efflux carrier protein, putative
264901_at	At1g23090	1,42	1,16	1,43	1,03	1,10	0,60	0,66	0,56	sulfate transporter, putative
264895_at	At1g23100	1,31	1,28	1,08	1,14	0,97	0,98	0,92	0,77	10 kDa chaperonin, putative
264887_at	At1g23120	1,54	1,52	1,72	1,42	1,49	0,77	0,92	0,98	major latex protein-related / MLP-related
264899_at	At1g23130	0,70	0,69	0,69	0,99	1,10	0,76	0,81	0,80	Bet v I allergen family protein
264893_at	At1g23140	1,51	1,29	0,88	1,78	1,45	1,56	1,11	0,93	C2 domain-containing protein
264892_at	At1g23160	1,03	1,09	1,07	1,06	0,97	1,00	0,94	0,90	auxin-responsive GH3 family protein
264890_at	At1g23180	1,23	1,05	1,14	0,87	0,81	1,25	0,90	0,94	armadillo/beta-catenin repeat family protein
264903_at	At1g23190	0,75	0,73	0,74	1,23	1,28	0,85	0,89	0,80	phosphoglucomutase, cytoplasmic, putative / glucose phosphomutase, puta
264891_at	At1g23200	0,77	0,90	0,64	0,84	0,83	0,73	0,72	0,78	pectinesterase family protein
264898_at	At1g23205	0,81	0,81	0,79	1,41	1,18	0,94	0,79	0,79	invertase/pectin methylesterase inhibitor family protein
264896_at	At1g23210	0,96	1,11	0,98	0,97	1,10	1,09	1,04	1,06	glycosyl hydrolase family 9 protein
264897_at	At1g23220	1,12	1,01	1,00	1,23	1,37	0,91	0,86	0,94	dynein light chain type 1 family protein
263037_at	At1g23230	1,09	1,07	1,01	1,04	0,87	1,08	1,06	1,10	expressed protein
262987_at	At1g23240	0,98	1,05	1,09	1,06	1,02	0,92	0,98	0,97	caleosin-related family protein

263011_at	At1g23250	1,05	0,96	1,25	1,10	1,13	0,94	0,93	0,99	caleosin-related
262990_at	At1g23260	1,16	1,00	1,06	1,02	1,10	1,04	1,05	1,05	ubiquitin-conjugating enzyme family protein
263038_at	At1g23270	0,98	1,04	0,96	1,06	1,09	0,96	0,93	0,99	hypothetical protein
263039_at	At1g23280	1,22	1,09	1,01	1,03	0,91	1,18	1,10	1,17	MAK16 protein-related
262985_s_at	At1g23290	1,25	1,16	1,09	1,16	1,13	1,01	0,94	0,95	60S ribosomal protein L27A (RPL27aB)
263040_at	At1g23300	0,94	0,93	1,02	1,07	1,04	0,91	1,06	0,97	MATE efflux family protein
262988_at	At1g23310	1,01	0,92	0,98	0,99	1,00	1,14	1,05	1,09	glutamate:glyoxylate aminotransferase 1 (GGT1)
263041_at	At1g23320	1,03	1,10	0,98	0,87	1,05	1,10	1,07	0,90	alliinase family protein
263010_at	At1g23330	0,93	0,86	0,97	0,96	0,77	1,09	1,04	1,17	expressed protein
263042_at	At1g23340	0,99	0,91	0,92	0,91	0,95	0,91	0,82	1,13	expressed protein
263043_at	At1g23350	0,82	1,04	0,89	1,05	0,98	0,96	1,03	1,13	invertase/pectin methylesterase inhibitor family protein
263044_at	At1g23360	1,06	0,97	0,91	0,93	0,77	1,01	1,19	1,09	UbiE/COQ5 methyltransferase family protein
263012_at	At1g23370	1,08	1,02	1,00	0,86	1,03	0,97	0,99	0,88	homeobox transcription factor (KNAT6)
263013_at	At1g23380	1,16	1,08	1,08	0,90	0,92	1,13	0,89	0,86	homeobox transcription factor (KNAT6)
262986_at	At1g23390	1,08	1,35	1,09	0,91	0,57	0,98	1,33	1,30	kelch repeat-containing F-box family protein
263014_at	At1g23400	1,12	1,24	1,60	0,71	0,58	1,31	1,15	1,61	expressed protein
263016_at	At1g23410	1,58	1,18	0,87	1,59	1,30	1,16	1,17	0,87	ubiquitin extension protein, putative / 40S ribosomal protein S27A (RPS27a
262989_at	At1g23420	0,90	0,98	1,05	1,17	1,01	1,01	1,04	0,78	inner no outer protein (INO)
263015_at	At1g23430	0,72	0,73	0,75	1,01	0,90	1,00	0,97	1,06	pyrrolidone-carboxylate peptidase family protein
262422_at	At1g23440	0,78	0,83	0,77	0,98	1,00	0,98	0,97	1,01	pyrrolidone-carboxylate peptidase family protein
262423_at	At1g23450	1,00	0,88	1,28	1,02	0,84	1,06	1,01	1,02	---
265174_s_at	At1g23460	1,45	1,16	1,24	1,18	0,98	1,02	0,96	1,04	polygalacturonase, putative / pectinase, putative
265175_at	At1g23480	1,13	1,03	1,22	1,14	0,82	0,86	1,06	1,19	glycosyl transferase family 2 protein
265187_at	At1g23490	0,81	0,68	0,73	0,90	1,03	0,88	0,91	0,91	ADP-ribosylation factor
265181_at	At1g23510	1,01	1,07	1,03	1,02	0,97	0,98	0,94	0,97	expressed protein
265176_at	At1g23520	0,96	0,93	1,06	1,00	1,00	1,11	1,06	0,92	expressed protein
265173_at	At1g23530	1,26	1,21	1,13	1,21	1,18	0,98	0,99	0,93	expressed protein
265178_at	At1g23540	0,97	1,07	1,09	0,82	1,08	1,05	0,98	1,13	protein kinase family protein
257401_at	At1g23550	0,98	0,96	1,01	0,90	1,00	1,18	1,22	1,12	expressed protein
265186_at	At1g23560	1,29	1,22	1,14	1,07	0,96	1,04	1,24	0,99	expressed protein
257402_at	At1g23570	0,96	0,89	1,04	0,96	1,10	0,96	1,04	0,98	expressed protein
265163_at	At1g23580	1,03	0,99	1,00	0,91	1,14	1,00	1,08	1,36	expressed protein
265180_at	At1g23590	0,90	1,09	1,12	0,92	1,02	1,05	0,94	0,99	expressed protein
265164_at	At1g23600	0,99	1,06	1,18	1,04	1,02	1,02	1,00	0,97	expressed protein
265165_at	At1g23610	1,00	1,08	0,97	0,97	1,01	1,10	0,94	1,05	hypothetical protein
265177_at	At1g23630	0,98	0,97	1,11	0,91	1,00	0,95	0,96	0,98	expressed protein
265166_at	At1g23640	1,04	0,98	1,05	1,06	1,04	1,00	1,17	0,92	---
265179_at	At1g23650	0,96	0,96	0,89	0,91	1,00	1,13	1,12	1,11	expressed protein
265185_at	At1g23670	0,99	1,05	1,12	1,05	1,23	0,97	1,07	0,91	expressed protein
265167_at	At1g23680	0,99	1,00	0,94	0,96	0,98	0,92	0,93	0,93	hypothetical protein
257399_at	At1g23690	1,09	0,99	0,96	0,94	1,01	1,16	0,96	1,10	expressed protein
265168_at	At1g23700	1,21	0,93	1,06	0,85	0,96	1,06	0,94	0,95	protein kinase family protein
265184_at	At1g23710	0,79	0,87	0,68	0,63	0,62	1,09	1,43	1,37	expressed protein
265169_x_at	At1g23720	1,10	1,14	1,00	1,39	1,30	1,12	1,08	1,04	proline-rich extensin-like family protein
265170_at	At1g23730	1,36	1,07	1,37	1,00	0,93	1,12	0,96	1,11	carbonic anhydrase, putative / carbonate dehydratase, putative
265182_at	At1g23740	1,15	1,29	1,62	1,31	1,32	1,45	1,37	1,33	oxidoreductase, zinc-binding dehydrogenase family protein

265183_at	At1g23750	0,79	0,68	0,62	1,33	1,50	0,87	0,92	0,85	DNA-binding protein-related
265131_at	At1g23760	1,28	1,38	1,24	1,59	1,49	0,96	1,12	1,22	BURP domain-containing protein / polygalacturonase, putative
265190_at	At1g23780	0,99	1,04	1,03	1,01	1,07	0,96	0,89	0,94	F-box family protein
265171_at	At1g23790	1,00	1,22	0,97	1,34	1,10	0,93	0,89	0,76	expressed protein
265188_at	At1g23800	1,20	0,90	0,90	1,97	1,44	1,02	1,25	1,22	aldehyde dehydrogenase, mitochondrial (ALDH3)
257400_s_at	At1g23810	1,00	0,95	0,93	1,07	1,16	0,90	0,99	0,93	paired amphipathic helix repeat-containing protein
265172_at	At1g23820	1,03	0,97	0,91	1,02	1,01	0,95	0,97	0,96	spermidine synthase 1 (SPDSYN1) / putrescine aminopropyltransferase 1
265132_at	At1g23830	0,52	0,63	0,75	1,05	1,19	0,79	0,87	0,89	expressed protein
265189_at	At1g23840	0,56	0,68	1,08	0,92	1,08	0,85	1,04	1,02	expressed protein
263032_at	At1g23850	0,65	0,67	0,69	0,89	0,87	0,75	0,90	0,84	expressed protein
263035_at	At1g23860	1,07	1,13	1,17	1,13	1,11	0,96	0,97	1,04	splicing factor RSZp21 (RSZP21) / 9G8-like SR protein (SRZ21)
263019_at	At1g23870	1,18	1,67	2,09	0,95	1,10	1,05	1,31	1,54	glycosyl transferase family 20 protein / trehalose-phosphatase family protei
263020_at	At1g23880	0,95	1,01	0,94	0,98	0,87	1,13	0,97	1,02	NHL repeat-containing protein
263036_at	At1g23890	0,92	1,22	1,21	1,25	0,96	0,94	0,90	0,95	NHL repeat-containing protein
263021_at	At1g23910	1,10	1,05	1,00	1,00	1,03	1,01	1,04	0,93	hypothetical protein
263022_s_at	At1g23940	1,06	0,90	0,94	0,94	0,99	1,12	0,93	0,95	gamma-adaptin, putative
263033_s_at	At1g23950	1,51	1,26	1,48	1,08	1,03	0,95	1,00	0,96	expressed protein
263023_at	At1g23960	1,12	1,06	1,03	0,88	1,08	0,98	0,92	0,95	expressed protein
263024_at	At1g23980	0,90	0,97	0,94	0,97	0,95	0,99	1,02	0,92	zinc finger (C3HC4-type RING finger) family protein
263025_at	At1g23990	0,99	1,01	0,93	0,98	1,07	1,02	0,92	0,93	---
263026_at	At1g24000	1,05	0,95	0,94	1,01	1,00	1,05	1,01	0,94	Bet v I allergen family protein
263027_at	At1g24010	1,03	1,02	1,01	1,00	1,10	1,02	0,99	1,00	expressed protein
263034_at	At1g24020	1,24	1,19	1,46	1,99	2,06	0,67	0,58	0,61	Bet v I allergen family protein
263028_at	At1g24030	1,16	1,30	1,32	1,15	1,37	1,02	1,36	1,23	protein kinase family protein
257459_at	At1g24040	0,96	1,16	1,10	0,69	0,66	0,98	0,99	0,98	GCN5-related N-acetyltransferase (GNAT) family protein
263029_at	At1g24050	1,22	1,15	1,25	1,30	1,35	1,01	0,93	0,93	expressed protein
263030_at	At1g24060	0,97	1,03	0,95	1,03	1,01	1,22	1,02	0,95	expressed protein
263031_at	At1g24070	0,95	1,02	1,05	1,08	1,10	0,97	0,88	0,73	glycosyl transferase family 2 protein
264868_at	At1g24090	1,23	1,36	1,41	1,08	1,29	0,98	0,81	1,06	RNase H domain-containing protein
264873_at	At1g24100	0,82	0,90	0,94	1,07	0,97	0,96	1,17	1,05	UDP-glucoronosyl/UDP-glucosyl transferase family protein
264863_at	At1g24110	0,99	0,97	0,96	1,06	1,03	0,92	1,03	0,96	peroxidase, putative
264865_at	At1g24120	1,03	0,98	1,41	0,92	0,90	1,00	0,96	1,40	DNAJ heat shock protein, putative
264866_at	At1g24140	0,88	0,67	0,66	1,12	1,05	1,13	1,30	1,35	matrixin family protein
264867_at	At1g24150	0,78	0,61	0,60	1,01	1,12	0,90	1,07	1,25	formin homology 2 domain-containing protein / FH2 domain-containing prot
264857_at	At1g24170	1,01	0,83	0,64	1,07	1,20	0,97	0,92	0,94	glycosyl transferase family 8 protein
264871_at	At1g24180	1,15	0,84	0,86	1,50	1,32	0,92	0,94	0,91	pyruvate dehydrogenase E1 component alpha subunit, mitochondrial, putat
264858_at	At1g24190	0,98	1,01	1,37	0,84	0,86	0,98	1,04	1,47	paired amphipathic helix repeat-containing protein
264870_at	At1g24210	1,14	1,05	0,97	1,04	1,04	0,94	0,96	0,94	paired amphipathic helix repeat-containing protein
264874_at	At1g24240	0,94	0,97	1,07	1,07	0,89	0,97	0,78	0,66	ribosomal protein L19 family protein
264872_at	At1g24260	1,90	1,70	1,80	1,17	1,24	0,75	0,85	0,61	MADS-box protein (AGL9)
264859_at	At1g24280	0,70	0,90	1,08	1,08	1,14	0,83	0,99	0,86	glucose-6-phosphate 1-dehydrogenase, putative / G6PD, putative
264860_at	At1g24290	0,96	0,99	0,90	1,04	1,13	0,96	1,10	0,87	AAA-type ATPase family protein
264864_at	At1g24310	1,10	1,01	1,13	0,94	0,86	1,00	0,92	0,75	expressed protein
264861_at	At1g24320	0,98	0,98	0,96	1,19	1,03	0,96	0,99	1,15	alpha-glucosidase, putative
264862_at	At1g24330	0,57	1,63	1,29	0,89	0,78	0,96	1,35	0,92	armadillo/beta-catenin repeat family protein / U-box domain-containing fami
257410_at	At1g24340	0,90	0,90	0,87	0,78	0,80	1,08	1,00	0,94	monooxygenase family protein

264869_at	At1g24350	1,01	0,91	0,95	0,88	0,82	1,27	1,12	1,03	expressed protein
265026_at	At1g24360	0,95	0,91	0,78	1,10	0,98	0,95	0,93	0,92	3-oxoacyl-(acyl-carrier protein) reductase, chloroplast / 3-ketoacyl-acyl carri
265017_at	At1g24390	0,98	0,97	0,94	1,03	1,05	1,06	0,91	1,03	hypothetical protein
265002_at	At1g24400	0,98	1,13	1,02	1,53	2,38	0,95	1,06	0,88	lysine and histidine specific transporter, putative
265016_at	At1g24420	1,00	0,98	1,08	1,01	1,07	1,01	1,11	0,97	transferase family protein
265014_at	At1g24430	0,92	1,33	1,06	0,85	1,05	1,10	1,11	1,14	transferase family protein
265023_at	At1g24440	1,24	1,28	0,99	0,92	0,89	1,19	1,30	1,03	expressed protein
265027_at	At1g24450	1,07	1,11	1,04	0,96	0,92	0,99	0,85	0,78	ribonuclease III family protein
265013_at	At1g24460	0,86	0,80	0,76	0,97	1,04	1,01	1,04	1,17	myosin-related
265012_at	At1g24470	1,01	1,00	1,14	1,13	0,97	0,78	1,00	1,01	short-chain dehydrogenase/reductase (SDR) family protein
265011_at	At1g24490	0,91	0,91	0,96	0,86	0,85	0,88	1,03	0,93	60 kDa inner membrane family protein
265010_at	At1g24510	1,33	1,17	1,08	1,24	1,00	0,99	1,04	0,93	T-complex protein 1 epsilon subunit, putative / TCP-1-epsilon, putative / cha
265022_at	At1g24520	0,93	0,97	1,12	0,88	0,96	1,06	0,92	0,84	anther-specific protein agp1
265028_at	At1g24530	1,31	1,48	1,04	1,16	1,37	0,92	1,02	0,84	transducin family protein / WD-40 repeat family protein
265020_at	At1g24540	0,87	0,98	1,21	0,95	1,24	0,99	1,07	1,02	cytochrome P450, putative
265019_at	At1g24560	1,04	1,07	1,22	0,85	0,94	1,11	1,09	1,54	expressed protein
265025_at	At1g24575	2,31	1,86	1,32	1,24	1,50	0,95	0,92	0,81	expressed protein
265024_at	At1g24600	0,97	1,13	1,05	0,99	1,30	0,95	1,00	0,96	expressed protein
265021_at	At1g24610	1,19	1,27	1,31	0,95	0,97	1,05	1,07	0,94	SET domain-containing protein
257405_at	At1g24620	0,98	1,03	0,95	0,97	1,06	1,04	0,97	1,18	polcalcin, putative / calcium-binding pollen allergen, putative
265029_at	At1g24625	0,80	0,83	0,88	0,88	0,95	1,07	1,02	1,00	zinc finger (C2H2 type) family protein (ZFP7)
265015_at	At1g24640	0,92	1,02	0,98	0,93	0,83	1,03	0,94	0,77	---
265009_at	At1g24650	0,99	0,98	1,01	0,89	1,03	0,96	1,01	0,89	leucine-rich repeat family protein / protein kinase family protein
245647_at	At1g24678	1,02	1,02	0,95	0,98	0,89	0,93	1,06	1,22	expressed protein
245649_at	At1g24706	1,04	1,14	1,17	0,83	0,94	1,23	1,17	1,26	expressed protein
245650_at	At1g24735	0,84	1,06	0,83	0,93	0,90	1,02	1,00	1,01	caffeoyl-CoA 3-O-methyltransferase, putative
245645_at	At1g24764	0,98	1,17	1,51	1,14	1,15	1,01	0,97	1,15	expressed protein
245651_s_at	At1g24793	0,92	1,07	0,91	1,16	1,20	0,96	0,86	0,87	UDP-3-0-acyl N-acetylglucosamine deacetylase family protein / F-box prote
245648_at	At1g24938	1,05	0,97	1,00	1,00	1,01	1,09	1,03	0,91	hypothetical protein
245638_s_at	At1g24996	1,02	1,06	1,11	0,85	0,97	0,70	0,56	0,60	expressed protein
245646_x_at	At1g25025	1,00	1,09	1,09	0,98	1,22	0,89	0,93	0,84	hypothetical protein
245637_at	At1g25230	1,19	1,23	1,54	1,15	1,04	0,77	0,94	1,14	purple acid phosphatase family protein
245636_at	At1g25240	1,01	1,04	1,23	1,03	0,91	1,04	1,16	0,97	epsin N-terminal homology (ENTH) domain-containing protein / clathrin ass
245635_at	At1g25250	0,91	0,67	0,84	0,90	0,79	0,93	1,00	0,81	zinc finger (C2H2 type) family protein
245639_at	At1g25260	1,50	1,26	1,25	1,19	1,26	0,97	1,01	1,02	acidic ribosomal protein P0-related
245634_at	At1g25270	0,94	1,04	0,95	0,97	1,03	1,05	0,90	1,11	nodulin MtN21 family protein
245642_at	At1g25275	0,70	0,81	0,94	0,71	0,79	0,79	0,76	0,78	expressed protein
245633_at	At1g25280	1,01	0,98	0,95	0,88	1,16	0,86	0,89	0,89	F-box family protein / tubby family protein
245632_at	At1g25290	0,83	0,99	0,78	0,97	0,72	0,84	0,96	0,91	rhomboid family protein
245644_at	At1g25320	0,76	0,87	1,06	0,86	1,08	0,80	0,72	0,88	leucine-rich repeat transmembrane protein kinase, putative
245640_at	At1g25330	1,10	0,96	1,02	0,98	0,93	0,99	1,04	0,98	basic helix-loop-helix (bHLH) family protein
245643_at	At1g25340	0,98	0,97	1,07	1,06	1,06	0,98	0,93	0,96	myb family transcription factor (MYB116)
245631_at	At1g25350	1,08	0,91	0,95	1,05	1,00	0,96	1,01	0,96	glutamine-tRNA ligase, putative / glutaminyl-tRNA synthetase, putative / Glı
245630_at	At1g25360	1,07	1,04	1,15	0,90	1,06	1,11	0,96	0,93	pentatricopeptide (PPR) repeat-containing protein
245641_at	At1g25370	0,71	0,68	0,77	1,59	1,74	1,04	0,88	1,00	expressed protein
255743_at	At1g25375	1,07	0,98	1,04	0,79	0,80	1,03	0,99	1,02	metallo-beta-lactamase family protein

255736_at	At1g25380	0,87	0,83	0,83	0,94	0,85	0,92	0,96	0,97	mitochondrial substrate carrier family protein
255740_at	At1g25390	0,70	0,85	0,65	0,73	0,94	1,03	1,15	0,85	protein kinase family protein
255733_at	At1g25400	0,99	1,21	0,73	0,71	1,00	1,13	1,10	1,00	expressed protein
255741_at	At1g25410	0,97	1,01	0,91	0,92	1,05	1,09	1,01	1,02	adenylate isopentenyltransferase 6 / adenylate dimethylallyltransferase / cy
255737_at	At1g25420	0,89	0,96	0,89	0,97	0,99	1,04	1,12	1,16	expressed protein
255732_at	At1g25450	0,88	0,73	0,70	1,04	0,92	1,02	0,93	0,91	very-long-chain fatty acid condensing enzyme, putative
255730_at	At1g25460	0,92	1,04	0,99	1,21	1,19	1,02	0,94	1,24	oxidoreductase family protein
255729_at	At1g25470	0,96	0,92	1,25	1,10	0,87	0,81	1,12	1,01	AP2 domain-containing transcription factor, putative
255731_at	At1g25490	0,91	0,84	0,93	0,98	1,00	0,94	0,92	0,95	serine/threonine protein phosphatase 2A (PP2A) regulatory subunit A (RCN
255728_at	At1g25500	0,66	0,97	0,82	0,86	0,62	0,79	0,92	0,64	choline transporter-related
255727_at	At1g25510	0,95	1,00	0,83	1,18	1,35	0,81	0,83	0,73	aspartyl protease family protein
255735_at	At1g25520	1,11	1,07	0,94	1,08	0,82	1,11	1,13	0,98	expressed protein
255726_at	At1g25530	0,72	0,91	0,80	1,54	1,36	0,87	1,02	0,93	lysine and histidine specific transporter, putative
255725_at	At1g25540	0,95	0,96	1,21	0,87	1,08	1,09	1,08	1,34	phytochrome and flowering time regulatory protein (PFT1)
255734_at	At1g25550	1,26	1,72	1,16	0,70	0,82	1,18	1,25	1,61	myb family transcription factor
255742_at	At1g25560	0,67	0,80	0,89	0,80	0,84	1,01	1,00	0,94	AP2 domain-containing transcription factor, putative
255738_at	At1g25570	0,95	1,00	0,89	0,95	0,93	0,86	0,93	1,00	leucine-rich repeat protein-related
255739_at	At1g25580	0,87	0,90	0,91	1,07	1,06	0,83	0,96	1,11	no apical meristem (NAM) family protein
245751_s_at	At1g25988	0,89	0,86	1,36	1,06	1,19	1,15	1,18	1,25	cell cycle control protein-related
245815_at	At1g26090	0,63	0,62	0,65	0,87	0,96	0,93	0,90	0,84	hypothetical protein
245818_at	At1g26100	0,97	0,97	1,13	0,92	1,07	0,95	0,89	1,10	cytochrome B561 family protein
245822_at	At1g26110	1,01	0,92	0,94	0,88	0,95	0,95	0,93	0,89	expressed protein
245846_at	At1g26130	0,90	1,07	0,80	0,89	0,70	1,20	0,90	0,93	haloacid dehalogenase-like hydrolase family protein
245845_at	At1g26150	1,01	0,97	1,13	0,77	0,80	1,22	1,54	1,57	protein kinase family protein
245817_at	At1g26160	1,00	0,79	0,94	0,89	1,08	0,94	0,98	0,79	metal-dependent phosphohydrolase HD domain-containing protein
245844_at	At1g26170	0,84	0,74	0,73	0,77	0,88	1,04	1,03	0,96	importin beta-2 subunit family protein
245843_at	At1g26180	0,82	0,91	1,13	0,94	0,90	0,98	1,03	1,10	expressed protein
245878_at	At1g26190	0,92	0,97	0,95	0,88	1,03	0,87	0,88	0,87	phosphoribulokinase/uridine kinase family protein
245816_at	At1g26210	0,74	0,70	0,88	0,95	0,90	0,96	0,82	0,92	expressed protein
245877_at	At1g26220	1,03	1,05	0,93	1,01	1,07	1,00	1,11	1,06	GCN5-related N-acetyltransferase (GNAT) family protein
245876_at	At1g26230	0,55	0,44	0,32	0,82	0,86	0,94	0,80	0,77	chaperonin, putative
245875_at	At1g26240	1,30	1,31	1,58	0,87	0,93	1,22	1,08	1,11	proline-rich extensin-like family protein
245874_at	At1g26250	1,85	2,28	1,87	0,62	0,55	1,26	1,81	1,30	proline-rich extensin, putative
245873_at	At1g26260	0,79	0,88	0,79	0,91	1,19	0,95	1,04	0,92	basic helix-loop-helix (bHLH) family protein
245821_at	At1g26270	1,03	1,10	0,93	1,04	1,42	0,96	0,89	0,90	phosphatidylinositol 3- and 4-kinase family protein
245872_at	At1g26280	1,11	0,85	0,86	0,93	1,18	1,14	0,91	1,25	BSD domain-containing protein
245871_at	At1g26290	0,93	0,99	0,88	1,13	1,14	0,92	0,86	1,02	hypothetical protein
245870_at	At1g26300	1,17	0,94	0,93	1,10	1,13	0,94	0,90	1,07	BSD domain-containing protein
245819_at	At1g26310	1,01	1,15	1,10	0,95	1,16	1,02	1,00	0,90	MADS-box protein, putative
245820_at	At1g26320	0,94	0,95	1,28	0,95	0,99	0,95	0,98	1,09	NADP-dependent oxidoreductase, putative
245869_at	At1g26330	0,99	1,13	0,91	1,05	1,04	1,04	0,86	1,05	hypothetical protein
261011_at	At1g26340	1,16	0,81	0,91	1,10	1,11	0,97	0,81	0,94	cytochrome b5, putative
261010_at	At1g26350	0,98	1,12	1,04	0,91	0,94	1,00	1,01	0,84	hypothetical protein
261009_at	At1g26360	1,08	0,89	0,99	0,96	1,04	1,13	0,94	0,81	hydrolase, alpha/beta fold family protein
261008_at	At1g26370	1,01	1,24	1,10	1,01	0,96	0,97	0,97	0,85	RNA helicase, putative
261021_at	At1g26380	1,99	2,48	2,94	0,69	0,81	2,43	1,64	1,46	FAD-binding domain-containing protein

261020_at	At1g26390	1,45	1,93	1,46	0,67	0,61	1,66	1,61	2,16	FAD-binding domain-containing protein
261007_at	At1g26400	0,95	1,03	0,83	0,98	0,99	0,88	0,98	1,12	FAD-binding domain-containing protein
261006_at	At1g26410	1,03	1,32	1,42	0,68	0,74	1,39	0,98	1,01	FAD-binding domain-containing protein
261005_at	At1g26420	1,06	1,52	0,99	0,95	0,86	1,06	1,19	1,12	FAD-binding domain-containing protein
261013_at	At1g26440	0,59	0,61	0,64	0,86	0,69	0,79	0,94	0,97	expressed protein
261004_at	At1g26450	1,37	1,05	1,09	1,44	1,81	0,85	0,74	0,68	beta-1,3-glucanase-related
261014_at	At1g26460	1,17	1,04	1,02	0,97	1,08	1,00	0,94	0,87	pentatricopeptide (PPR) repeat-containing protein
261019_at	At1g26470	1,26	1,08	1,08	1,04	1,20	0,97	0,95	0,94	expressed protein
261015_at	At1g26480	1,04	1,09	0,99	0,96	1,07	0,87	1,07	1,09	14-3-3 protein GF14 iota (GRF12)
261003_at	At1g26500	0,86	0,99	0,86	0,97	0,90	1,16	1,06	1,00	pentatricopeptide (PPR) repeat-containing protein
261002_at	At1g26520	0,98	1,11	0,94	0,88	0,91	1,01	0,90	1,00	cobalamin synthesis/P47K family protein
261001_at	At1g26530	1,14	0,99	1,04	1,05	1,03	1,12	0,98	1,13	expressed protein
261000_at	At1g26540	1,16	1,30	1,18	1,16	1,28	0,88	0,97	0,75	agenet domain-containing protein
261018_at	At1g26550	1,20	1,17	1,09	1,28	1,36	1,02	0,94	0,92	peptidyl-prolyl cis-trans isomerase PPIC-type family protein
261016_at	At1g26560	1,24	1,20	1,62	0,80	0,79	1,00	1,18	1,31	glycosyl hydrolase family 1 protein
261017_at	At1g26570	1,17	0,89	1,03	1,14	1,04	0,90	0,85	0,80	UDP-glucose 6-dehydrogenase, putative
260999_at	At1g26580	0,80	0,71	0,78	0,81	0,89	1,03	1,12	1,09	expressed protein
260998_at	At1g26590	1,07	0,94	0,99	0,91	1,16	0,99	0,98	0,93	zinc finger (C2H2 type) family protein
261012_at	At1g26600	0,83	0,79	1,01	1,10	1,18	0,95	0,78	0,81	CLE9, putative
260997_at	At1g26610	1,04	0,96	1,13	1,01	0,98	0,97	1,01	0,83	zinc finger (C2H2 type) family protein
261028_at	At1g26620	0,94	1,15	1,07	0,88	0,98	0,92	0,90	0,91	expressed protein
261270_at	At1g26630	1,34	1,19	1,09	1,25	1,36	0,96	0,96	1,02	eukaryotic translation initiation factor 5A, putative / eIF-5A, putative
261258_at	At1g26640	0,93	0,86	0,77	1,02	1,03	0,92	0,96	0,88	aspartate/glutamate/uridylate kinase family protein
261273_at	At1g26650	0,88	0,96	0,87	1,08	1,23	0,80	0,75	0,59	expressed protein
261259_at	At1g26660	1,17	1,09	1,05	1,09	0,96	1,10	1,02	1,16	c-myc binding protein, putative / prefoldin, putative
261272_at	At1g26665	0,87	0,89	1,09	1,12	1,26	0,90	0,95	0,94	expressed protein
261276_at	At1g26670	0,89	0,90	0,98	0,98	0,95	0,88	0,94	1,07	vesical transport v-SNARE 12 (VTI12) / vesicle soluble NSF attachment prc
261260_at	At1g26680	0,89	1,09	1,00	1,01	1,27	0,95	1,12	1,04	transcriptional factor B3 family protein
261269_at	At1g26690	0,99	0,91	1,06	1,82	1,83	0,96	0,95	0,98	emp24/gp25L/p24 family protein
261275_at	At1g26700	1,06	1,01	1,08	1,20	1,16	1,05	1,00	1,00	seven transmembrane MLO family protein / MLO-like protein 14 (MLO14)
261264_at	At1g26710	0,95	1,04	0,96	0,93	0,95	0,98	0,99	0,96	expressed protein
261261_at	At1g26730	0,83	0,89	0,97	1,31	0,83	1,18	1,35	1,32	EXS family protein / ERD1/XPR1/SYG1 family protein
261268_at	At1g26740	1,34	1,32	1,27	1,30	1,20	0,91	0,85	0,93	expressed protein
261267_at	At1g26750	1,15	1,02	0,85	1,09	1,02	0,98	0,94	0,95	expressed protein
261262_at	At1g26760	1,13	1,09	0,82	0,98	1,00	1,10	0,92	1,02	SET domain-containing protein
261266_at	At1g26770	1,41	1,43	1,23	1,35	1,24	1,16	1,11	0,88	expansin, putative (EXP10)
261274_at	At1g26780	1,05	1,13	1,03	1,04	1,17	1,09	1,00	1,26	myb family transcription factor (MYB117)
261263_at	At1g26790	0,83	1,04	1,04	0,99	1,09	0,97	0,94	0,99	Dof-type zinc finger domain-containing protein
261271_at	At1g26795	0,99	0,92	0,93	1,06	0,94	1,08	1,10	0,88	self-incompatibility protein-related
261265_at	At1g26800	1,32	0,99	1,12	1,23	0,95	1,04	0,95	1,18	zinc finger (C3HC4-type RING finger) family protein
263689_at	At1g26820	1,31	1,39	1,25	2,05	1,77	1,14	1,04	1,22	ribonuclease 3 (RNS3)
263685_at	At1g26830	1,01	0,94	1,01	1,08	1,13	1,01	0,95	1,16	cullin, putative
263681_at	At1g26840	1,19	0,83	0,96	1,17	1,13	1,08	0,92	1,01	origin recognition complex subunit 6 family protein (ORC6)
263692_at	At1g26850	0,95	0,90	0,85	1,06	1,11	0,96	0,92	0,89	dehydration-responsive family protein
263682_at	At1g26860	0,98	0,99	1,03	1,04	0,88	0,99	0,94	0,99	hypothetical protein
263683_at	At1g26870	1,01	1,14	0,95	0,99	1,10	0,94	1,00	0,91	no apical meristem (NAM) family protein

263691_at	At1g26880	1,41	1,06	1,06	1,23	1,06	1,03	0,97	0,98	60S ribosomal protein L34 (RPL34A)
263684_at	At1g26900	0,83	0,94	1,09	1,05	1,01	0,93	1,00	0,99	pentatricopeptide (PPR) repeat-containing protein
263686_at	At1g26910	1,11	1,03	1,08	1,34	1,23	0,96	0,91	0,84	60S ribosomal protein L10 (RPL10B)
263688_at	At1g26920	0,86	0,90	1,19	0,92	1,02	0,91	0,90	1,17	expressed protein
263680_at	At1g26930	0,69	0,60	1,04	1,03	0,87	0,78	0,93	0,68	kelch repeat-containing F-box family protein
263687_at	At1g26940	0,84	0,60	0,47	1,16	1,07	0,85	0,79	0,82	peptidyl-prolyl cis-trans isomerase cyclophilin-type family protein
263690_at	At1g26960	1,04	1,02	1,10	1,19	1,41	0,90	0,96	1,06	homeobox-leucine zipper protein, putative / HD-ZIP transcription factor, put
265003_at	At1g26970	0,99	1,07	1,06	0,87	1,34	0,99	1,14	0,79	protein kinase, putative
265004_at	At1g26990	1,02	0,97	0,94	1,10	1,08	1,03	0,87	0,99	---
264984_at	At1g27000	1,06	0,97	0,88	1,10	1,09	0,99	1,01	1,01	bZIP family transcription factor
264987_at	At1g27030	0,77	0,68	0,59	1,16	0,95	0,89	0,79	0,81	expressed protein
264973_at	At1g27040	0,98	1,03	0,94	0,99	1,10	1,15	0,92	0,77	nitrate transporter, putative
264974_at	At1g27050	0,98	0,98	0,85	1,03	0,93	0,89	0,86	0,87	homeobox-leucine zipper family protein
257406_at	At1g27060	1,15	1,00	0,98	0,80	0,81	1,02	0,93	1,07	regulator of chromosome condensation (RCC1) family protein
264975_at	At1g27070	0,93	0,93	1,30	0,93	0,86	1,15	0,97	1,26	5'-AMP-activated protein kinase-related
264976_at	At1g27080	1,02	1,03	1,05	0,98	0,99	1,08	1,02	1,09	proton-dependent oligopeptide transport (POT) family protein
264977_at	At1g27090	1,16	1,07	1,45	0,98	0,83	0,98	0,99	1,08	glycine-rich protein
257407_at	At1g27100	0,85	1,13	1,49	0,74	0,63	1,18	1,28	1,71	expressed protein
264978_at	At1g27120	0,99	1,37	0,84	1,01	0,99	1,28	0,99	1,06	galactosyltransferase family protein
264986_at	At1g27130	0,97	0,91	0,97	0,96	0,85	0,77	0,76	0,78	glutathione S-transferase, putative
264988_at	At1g27140	0,90	0,87	0,87	0,87	0,99	0,99	0,96	0,93	glutathione S-transferase, putative
264985_at	At1g27150	0,93	1,06	1,10	0,93	0,95	1,10	1,03	0,95	expressed protein
264981_at	At1g27160	0,92	1,03	1,14	0,92	0,91	0,88	0,98	1,04	valyl-tRNA synthetase / valine--tRNA ligase-related
264979_s_at	At1g27170	1,02	0,82	0,90	0,81	0,99	0,92	1,12	0,88	disease resistance protein (TIR-NBS-LRR class), putative
264980_at	At1g27190	0,70	0,70	0,90	0,89	0,98	0,83	0,80	0,91	leucine-rich repeat transmembrane protein kinase, putative
264989_at	At1g27200	0,77	0,83	0,90	0,81	0,81	0,99	0,99	1,25	expressed protein
264990_at	At1g27210	1,19	0,94	0,83	0,79	0,94	0,90	0,93	0,86	expressed protein
264982_at	At1g27220	0,93	0,88	1,03	1,08	1,05	0,91	1,06	1,13	paired amphipathic helix repeat-containing protein
264983_at	At1g27240	0,97	1,12	1,02	1,09	1,03	0,96	0,94	1,00	paired amphipathic helix repeat-containing protein
264496_at	At1g27285	0,96	1,03	0,99	0,86	1,17	0,87	0,97	0,89	---
264445_at	At1g27290	0,88	0,96	0,98	1,03	0,73	0,95	1,03	1,20	expressed protein
264447_at	At1g27300	0,94	1,01	1,09	1,35	1,18	1,00	1,07	1,25	expressed protein
264446_at	At1g27310	1,49	1,31	1,15	1,16	1,34	1,06	1,01	1,04	nuclear transport factor 2 (NTF2), putative
264448_at	At1g27320	0,98	1,13	1,50	0,69	0,71	1,22	1,35	1,58	histidine kinase (AHK3)
264488_s_at	At1g27330	1,08	0,93	0,78	1,13	1,17	0,94	0,93	0,95	expressed protein
264440_at	At1g27340	1,07	0,99	1,10	0,76	0,94	0,97	0,93	0,88	F-box family protein
264444_at	At1g27360	0,96	0,99	1,14	0,92	1,24	1,08	0,96	1,34	squamosa promoter-binding protein-like 11 (SPL11)
264489_at	At1g27370	1,75	1,42	1,19	0,77	0,96	1,13	1,13	1,33	squamosa promoter-binding protein-like 10 (SPL10)
264495_at	At1g27380	0,97	0,89	0,94	1,03	0,93	1,00	0,96	1,20	p21-rho-binding domain-containing protein
264443_at	At1g27385	1,12	0,96	0,95	0,82	0,77	0,98	0,99	1,04	expressed protein
264490_at	At1g27390	1,38	1,07	1,08	1,11	1,02	1,02	1,00	0,96	mitochondrial import receptor subunit TOM20-2 (TOM20-2)
264438_at	At1g27400	1,41	1,25	1,10	1,23	1,19	1,09	1,05	1,02	60S ribosomal protein L17 (RPL17A)
264491_at	At1g27410	0,96	0,87	1,21	0,89	0,84	0,98	0,97	1,08	DNA cross-link repair protein-related
264492_at	At1g27430	0,75	0,81	1,00	0,70	0,91	1,06	1,09	0,86	GYF domain-containing protein
264441_at	At1g27435	1,72	1,37	1,25	1,34	1,34	1,07	1,05	1,07	expressed protein
264493_at	At1g27440	0,94	1,04	0,80	0,80	0,98	0,92	0,94	1,07	exostosin family protein

264439_at	At1g27450	1,05	1,00	0,96	1,21	1,10	0,96	0,85	1,00	adenine phosphoribosyltransferase 1 (APT1)
264449_at	At1g27460	0,90	0,82	1,13	0,90	0,85	0,89	0,73	0,84	calmodulin-binding protein
264494_at	At1g27470	0,94	0,90	1,06	0,91	0,91	1,00	0,99	1,23	Clone asmbl_1702 unknown mRNA sequence
264442_at	At1g27480	0,91	0,80	1,10	0,81	0,72	1,26	1,10	1,17	lecithin:cholesterol acyltransferase family protein / LACT family protein
264437_at	At1g27510	0,97	1,04	1,21	0,88	0,86	0,99	1,18	1,09	expressed protein
262299_at	At1g27520	0,62	0,71	0,83	0,90	1,00	0,85	0,91	1,03	glycoside hydrolase family 47 protein
262298_at	At1g27530	1,28	1,08	1,01	1,08	1,03	0,89	0,96	0,97	expressed protein
262319_s_at	At1g27540	1,04	1,00	1,00	1,32	0,83	1,01	0,87	0,81	F-box family protein
262320_at	At1g27550	1,03	1,00	0,94	1,04	0,99	0,94	1,00	1,09	F-box family protein
262321_at	At1g27570	1,04	0,97	0,95	0,94	1,23	0,87	1,01	0,97	phosphatidylinositol 3- and 4-kinase family protein
262322_at	At1g27590	1,25	1,19	1,47	0,76	0,83	1,01	1,27	1,23	expressed protein
262292_at	At1g27595	0,78	0,87	1,06	0,88	0,86	0,88	0,91	1,24	expressed protein
262297_at	At1g27600	1,03	0,83	1,26	1,03	1,27	0,99	0,97	1,41	glycosyl transferase family 43 protein
262294_at	At1g27610	0,94	0,95	0,94	0,98	0,97	1,05	1,14	0,98	hypothetical protein
262318_at	At1g27620	0,90	1,11	1,09	1,13	1,05	0,90	0,94	1,01	transferase family protein
262296_at	At1g27630	1,10	1,06	1,34	0,88	0,78	0,99	1,07	1,33	cyclin family protein
262293_at	At1g27640	0,94	1,01	1,00	0,80	1,05	0,99	1,09	0,94	expressed protein
262295_at	At1g27650	1,06	1,09	1,16	0,91	1,02	0,89	0,95	0,93	U2 snRNP auxiliary factor small subunit, putative
261641_at	At1g27670	1,04	0,93	0,94	1,21	1,18	0,94	0,94	1,09	expressed protein
261642_at	At1g27680	1,13	1,02	1,18	0,94	1,00	1,04	1,06	1,01	glucose-1-phosphate adenylyltransferase large subunit 2 (APL2) / ADP-gluc
261646_at	At1g27690	0,98	1,42	1,00	0,83	1,27	0,99	0,97	0,98	expressed protein
261649_at	At1g27700	1,02	0,84	0,90	1,15	1,11	1,06	0,99	1,15	expressed protein
261643_at	At1g27720	0,97	1,04	0,96	0,83	0,94	1,14	1,07	1,01	transcription initiation factor IID (TFIID) component TAF4 family protein
261648_at	At1g27730	0,95	1,16	1,08	0,90	0,47	1,38	1,82	1,67	zinc finger (C2H2 type) family protein (ZAT10) / salt-tolerance zinc finger pr
261647_at	At1g27740	1,04	1,07	0,93	1,12	1,09	1,17	0,99	0,94	basic helix-loop-helix (bHLH) family protein
261651_at	At1g27760	0,94	0,76	0,87	1,15	1,21	1,05	1,06	1,01	interferon-related developmental regulator family protein / IFRD protein fam
261650_at	At1g27770	0,86	1,26	2,05	0,78	0,81	1,31	1,78	1,61	calcium-transporting ATPase 1, plasma membrane-type / Ca(2+)-ATPase is
261645_at	At1g27790	0,96	0,97	1,01	0,94	1,00	1,11	1,02	1,01	hypothetical protein
257482_x_at	At1g27820	1,14	0,80	0,71	0,84	0,88	0,99	0,93	1,04	CCR4-NOT transcription complex protein, putative
261644_s_at	At1g27830	1,02	0,96	1,17	0,83	0,62	1,17	1,30	1,49	transducin family protein / WD-40 repeat family protein
245764_s_at	At1g27840	0,94	0,96	1,10	0,77	0,85	1,07	1,18	1,16	transducin family protein / WD-40 repeat family protein
245763_at	At1g27850	0,47	0,57	0,56	0,89	0,92	1,04	1,05	0,94	expressed protein
245762_at	At1g27880	1,09	1,13	1,09	1,03	1,09	1,06	1,07	0,90	ATP-dependent DNA helicase, putative
259597_at	At1g27900	0,97	0,83	0,89	1,07	1,09	0,96	1,00	0,92	RNA helicase, putative
259605_at	At1g27910	1,10	1,43	1,75	1,45	1,29	1,06	1,35	1,57	U-box domain-containing protein
259606_at	At1g27920	0,99	1,04	1,00	0,94	1,03	1,16	1,35	1,20	microtubule associated protein (MAP65/ASE1) family protein
259588_at	At1g27930	0,90	1,00	0,88	1,25	1,42	0,89	0,75	0,83	expressed protein
259607_at	At1g27940	0,93	0,98	0,96	0,99	0,95	1,21	1,20	1,26	multidrug resistance P-glycoprotein, putative
259592_at	At1g27950	1,08	0,92	0,88	1,17	1,00	0,95	0,85	0,82	lipid transfer protein-related
259608_at	At1g27960	0,96	0,81	1,33	0,94	0,93	1,03	0,88	0,93	expressed protein
259593_at	At1g27970	1,14	1,06	1,02	1,54	1,59	0,93	0,76	0,79	nuclear transport factor 2 (NTF2), putative
259598_at	At1g27980	0,75	0,82	0,80	1,18	1,27	0,92	0,76	0,90	pyridoxal-dependent decarboxylase family protein
259578_at	At1g27990	0,46	0,61	0,91	0,67	0,75	1,01	0,96	0,91	expressed protein
259579_at	At1g28010	1,32	1,16	1,25	1,25	1,00	1,25	1,37	1,43	multidrug resistance P-glycoprotein, putative
259580_at	At1g28030	0,86	1,01	0,89	1,00	1,37	0,99	0,93	0,86	oxidoreductase, 2OG-Fe(II) oxygenase family protein
259581_at	At1g28040	0,91	0,63	0,68	1,06	1,06	1,21	0,86	0,84	zinc finger (C3HC4-type RING finger) family protein

259595_at	At1g28050	0,58	0,91	0,85	0,78	1,09	0,85	0,98	1,04	zinc finger (B-box type) family protein
259582_at	At1g28060	3,01	2,70	3,11	0,82	0,84	1,87	1,94	2,19	small nuclear ribonucleoprotein family protein / snRNP family protein
259583_at	At1g28070	0,90	0,81	0,77	1,01	1,01	0,88	0,95	1,04	expressed protein
259584_at	At1g28080	0,91	1,07	1,02	0,91	1,15	1,03	0,90	0,84	expressed protein
259585_at	At1g28090	1,18	1,14	1,03	0,98	0,97	0,96	1,01	0,98	polynucleotide adenylyltransferase family protein
259586_at	At1g28100	0,84	0,92	0,90	0,83	0,96	1,03	1,22	1,15	expressed protein
259599_at	At1g28110	0,94	1,04	0,99	1,12	1,20	0,80	0,82	0,87	serine carboxypeptidase S10 family protein
259587_at	At1g28120	1,04	1,13	1,32	1,00	0,98	1,14	1,05	1,15	expressed protein
259596_at	At1g28130	1,17	1,36	1,10	1,33	1,72	1,06	0,91	0,92	auxin-responsive GH3 family protein
259589_at	At1g28135	0,98	1,04	0,97	0,92	1,17	1,04	0,81	0,90	expressed protein
259594_at	At1g28140	0,68	0,63	0,67	0,75	0,77	1,00	1,00	1,06	expressed protein
259591_at	At1g28150	0,91	1,00	1,24	0,98	0,85	1,05	0,96	0,99	expressed protein
259590_at	At1g28160	1,00	1,13	1,06	0,97	1,15	1,06	0,94	1,03	ethylene-responsive element-binding family protein
245663_at	At1g28170	1,04	1,12	1,14	1,08	1,03	0,90	0,97	1,03	sulfotransferase family protein
245662_at	At1g28190	0,82	0,93	0,98	0,86	0,86	0,95	1,00	1,01	expressed protein
245667_at	At1g28200	0,89	0,81	0,91	1,09	1,02	0,93	0,85	0,98	GRAM domain-containing protein / ABA-responsive protein-related
245670_at	At1g28210	1,09	0,87	1,04	0,90	1,01	0,98	0,88	0,89	DNAJ heat shock protein, putative
245661_at	At1g28220	0,92	1,01	1,05	1,12	1,32	0,98	0,94	0,84	purine permease, putative
245671_at	At1g28230	0,97	0,89	0,94	1,08	0,77	0,82	1,01	1,03	purine permease (PUP1)
245660_at	At1g28240	0,77	0,70	0,70	0,80	1,00	0,97	0,93	1,04	expressed protein
245665_at	At1g28250	1,18	1,24	1,21	1,35	1,21	0,86	1,01	1,02	expressed protein
245659_at	At1g28260	0,65	1,22	1,13	0,65	0,78	1,00	0,94	1,02	expressed protein
245658_at	At1g28270	0,97	0,98	1,01	1,08	1,06	0,98	0,99	0,86	rapid alkalinization factor (RALF) family protein
245666_at	At1g28280	0,90	0,87	0,73	1,10	0,94	0,78	0,73	0,82	VQ motif-containing protein
245688_at	At1g28290	1,39	1,50	1,04	1,95	1,29	1,00	1,03	1,00	pollen Ole e 1 allergen and extensin family protein
245669_at	At1g28300	0,98	1,06	0,95	1,04	0,95	1,21	0,90	1,21	transcriptional factor B3 family protein / leafy cotyledon 2 (LEC2)
245687_at	At1g28320	1,39	1,54	1,88	0,82	0,79	1,27	1,23	1,11	protease-related
245664_at	At1g28327	1,01	1,00	1,01	1,03	1,12	0,97	1,09	0,98	hypothetical protein
245668_at	At1g28330	1,04	1,76	1,72	0,66	0,72	0,92	1,17	1,19	dormancy-associated protein, putative (DRM1)
261469_at	At1g28340	0,83	0,86	0,91	0,86	0,95	0,93	0,89	0,91	leucine-rich repeat family protein
261496_at	At1g28360	0,85	1,18	1,02	0,82	1,02	0,93	0,90	0,92	ERF domain protein 12 (ERF12)
261470_at	At1g28370	0,91	1,40	1,28	1,34	1,57	1,03	1,28	1,09	ERF domain protein 11 (ERF11)
261442_at	At1g28375	0,91	0,98	0,88	1,03	0,97	0,97	0,94	0,90	expressed protein
261445_at	At1g28380	0,66	0,70	1,08	0,89	0,88	0,93	1,06	1,34	expressed protein
261501_at	At1g28390	0,96	1,04	0,91	1,33	1,11	1,00	0,73	0,75	protein kinase family protein
261439_at	At1g28395	1,46	1,20	0,97	1,03	1,02	0,99	0,86	0,79	expressed protein
261500_at	At1g28400	2,73	2,18	1,51	2,58	2,18	0,97	1,20	1,21	expressed protein
261495_at	At1g28410	1,03	1,00	0,93	0,93	1,07	0,89	0,83	0,91	expressed protein
261494_at	At1g28420	0,99	1,05	1,30	0,93	1,10	1,15	1,09	1,22	homeobox transcription factor, putative
261499_at	At1g28430	0,94	1,09	0,98	0,97	1,06	0,91	0,83	1,00	cytochrome P450, putative
261498_at	At1g28440	0,69	0,77	0,89	0,79	0,93	0,73	0,88	1,31	leucine-rich repeat transmembrane protein kinase, putative
261497_x_at	At1g28460	0,91	0,97	1,04	0,97	1,02	1,11	0,79	0,96	MADS-box family protein
261441_at	At1g28470	1,26	1,18	1,10	0,87	1,07	1,05	1,23	1,29	no apical meristem (NAM) family protein
261443_at	At1g28480	0,81	1,10	0,77	1,12	0,88	1,80	1,49	1,29	glutaredoxin family protein
261444_at	At1g28490	1,12	0,82	0,98	1,02	1,14	1,01	1,05	0,99	syntaxin 61 (SYP61) / osmotic stess-sensitive mutant 1 (OSM1)
261468_s_at	At1g28500	1,04	0,96	1,08	0,99	1,06	1,01	0,99	0,93	expressed protein

261440_at	At1g28510	1,13	1,02	0,97	1,20	1,49	0,98	0,96	0,94 expressed protein
261467_at	At1g28520	1,44	1,90	2,30	1,37	1,73	0,95	1,06	1,55 expressed protein
262738_at	At1g28530	0,80	0,76	0,93	0,69	0,57	0,89	0,95	0,70 expressed protein
262747_at	At1g28540	1,31	1,36	1,61	1,02	0,76	1,31	1,12	1,32 expressed protein
262742_at	At1g28550	1,01	1,03	0,92	1,00	1,22	1,02	0,95	0,96 Ras-related GTP-binding protein, putative
262737_at	At1g28560	1,17	1,24	1,04	1,09	0,78	0,99	1,03	0,90 snRNA activating complex family protein
262736_at	At1g28570	0,86	0,82	0,76	1,34	1,25	1,20	1,18	1,41 GDSL-motif lipase, putative
262749_at	At1g28580	0,98	1,03	0,87	1,15	1,17	1,14	1,08	1,03 GDSL-motif lipase, putative
262740_at	At1g28590	0,99	0,98	0,89	0,98	1,04	0,97	1,04	0,97 lipase, putative
262745_at	At1g28600	0,74	0,58	0,58	1,08	0,98	1,05	0,91	0,96 lipase, putative
262748_at	At1g28610	0,67	0,60	0,57	1,02	1,01	1,11	1,16	1,00 GDSL-motif lipase, putative
262735_at	At1g28630	1,01	1,09	0,93	0,92	0,90	0,97	1,00	1,07 expressed protein
262734_at	At1g28640	0,93	0,97	0,99	0,95	1,02	0,98	0,94	0,91 GDSL-motif lipase, putative
262739_at	At1g28650	0,89	0,76	0,77	0,90	0,85	1,05	0,96	0,89 lipase, putative
262733_s_at	At1g28670	1,20	1,31	1,36	0,90	0,94	0,97	0,90	0,85 lipase, putative
262744_at	At1g28680	1,19	1,42	1,61	1,61	1,43	0,96	1,19	1,29 transferase family protein
262763_at	At1g28690	0,98	1,08	1,13	0,95	0,97	0,99	0,90	0,80 pentatricopeptide (PPR) repeat-containing protein
262750_at	At1g28710	1,48	1,32	1,39	0,83	0,98	1,17	1,21	1,41 expressed protein
262741_at	At1g28760	0,83	0,71	0,74	1,09	0,96	0,98	0,79	0,85 expressed protein
262746_at	At1g28960	0,87	0,92	0,78	0,93	0,88	0,84	0,93	0,73 MutT/nudix family protein
262743_at	At1g29020	1,10	1,04	0,90	1,20	1,07	1,02	1,01	1,06 calcium-binding EF hand family protein
260871_at	At1g29040	0,82	0,80	0,78	1,30	1,38	0,93	0,74	0,85 expressed protein
260840_at	At1g29050	1,08	1,03	1,25	2,23	1,77	0,81	1,04	1,06 expressed protein
260843_at	At1g29060	0,99	0,67	0,80	0,98	0,93	0,94	0,86	0,92 expressed protein
260898_at	At1g29070	0,97	0,85	0,85	0,81	0,75	0,90	0,93	0,83 ribosomal protein L34 family protein
260891_at	At1g29080	0,95	1,11	1,03	0,97	0,98	1,06	0,91	1,24 peptidase C1A papain family protein
260890_at	At1g29090	0,60	0,67	0,49	0,50	0,65	0,89	1,27	1,19 peptidase C1A papain family protein
260892_at	At1g29110	0,97	1,17	1,07	0,98	1,24	0,96	1,13	1,17 cysteine proteinase, putative
260889_at	At1g29130	0,81	0,86	0,94	0,84	0,99	1,01	0,97	1,01 expressed protein
260888_at	At1g29140	1,03	1,14	1,16	1,04	1,03	1,04	1,06	0,95 pollen Ole e 1 allergen and extensin family protein
260842_at	At1g29150	1,13	1,07	1,12	1,20	1,24	1,01	0,93	0,89 26S proteasome regulatory subunit, putative (RPN6)
260887_at	At1g29160	0,93	1,20	1,10	1,13	1,33	0,99	1,04	0,95 Dof-type zinc finger domain-containing protein
260893_at	At1g29180	0,95	1,10	0,90	1,03	0,77	1,08	1,11	0,97 DC1 domain-containing protein
260841_at	At1g29195	0,95	0,87	0,84	1,38	1,00	1,02	0,94	1,00 expressed protein
260886_at	At1g29200	0,96	1,11	1,08	0,93	1,24	0,91	0,92	0,97 hypothetical protein
260894_at	At1g29220	1,27	1,23	1,96	1,13	1,03	1,27	1,20	1,33 transcriptional regulator family protein
260885_at	At1g29230	0,97	0,96	0,92	1,10	1,06	0,99	1,01	1,09 CBL-interacting protein kinase 18 (CIPK18)
260884_at	At1g29240	0,90	1,03	1,02	0,95	1,09	0,99	1,05	0,92 expressed protein
260895_at	At1g29250	1,40	1,17	1,00	1,30	1,15	1,03	0,95	0,93 expressed protein
260844_at	At1g29260	1,29	1,36	1,43	1,09	1,16	1,03	0,93	0,85 peroxisomal targeting signal type 2 receptor (PEX7)
260883_at	At1g29270	0,98	0,95	0,90	1,17	1,26	1,25	0,92	0,72 expressed protein
260882_at	At1g29280	1,07	1,23	1,03	1,27	1,11	0,97	1,40	1,60 WRKY family transcription factor
260896_at	At1g29310	1,09	0,95	0,89	1,35	1,29	0,85	0,86	0,81 protein transport protein sec61, putative
260897_at	At1g29330	0,69	0,50	0,45	1,41	1,17	0,81	0,85	0,74 ER lumen protein retaining receptor (ERD2) / HDEL receptor
259826_at	At1g29340	0,79	0,79	0,85	0,98	0,92	1,07	1,15	1,20 armadillo/beta-catenin repeat family protein / U-box domain-containing prot
259767_s_at	At1g29350	1,01	0,97	1,20	0,90	1,21	0,93	0,99	1,15 expressed protein

259768_at	At1g29390	0,58	0,56	0,59	0,95	1,04	0,74	0,82	0,78	stress-responsive protein, putative
259789_at	At1g29395	0,51	0,52	0,63	0,78	0,80	0,70	0,64	0,51	stress-responsive protein, putative
259769_at	At1g29400	1,00	1,12	1,38	1,02	0,98	1,11	1,17	1,27	RNA recognition motif (RRM)-containing protein
259770_s_at	At1g29410	1,39	1,43	1,51	1,13	0,88	0,75	0,90	0,81	phosphoribosylanthranilate isomerase 1 (PAI1)
259790_s_at	At1g29430	1,80	1,06	1,10	1,03	0,99	1,28	1,01	1,09	auxin-responsive family protein
257506_at	At1g29440	1,05	0,98	0,92	0,99	1,00	1,12	1,11	1,07	auxin-responsive family protein
259784_at	At1g29450	1,02	1,04	0,97	0,93	1,10	1,01	0,98	1,03	auxin-responsive protein, putative
259787_at	At1g29460	0,96	0,94	1,08	1,05	1,00	1,07	1,00	0,89	auxin-responsive protein, putative
259771_at	At1g29470	0,90	0,77	0,77	1,06	1,08	0,96	1,04	1,16	dehydration-responsive protein-related
259772_at	At1g29480	0,97	1,07	1,00	0,94	0,97	1,05	1,16	1,19	hypothetical protein
259785_at	At1g29490	0,89	1,05	1,01	1,10	0,99	0,93	0,98	0,94	auxin-responsive family protein
259773_at	At1g29500	1,32	1,12	0,96	1,14	0,90	1,03	0,83	0,81	auxin-responsive protein, putative
259783_at	At1g29510	1,37	1,10	1,00	1,20	1,08	1,03	1,03	0,95	auxin-responsive protein, putative
259774_at	At1g29520	1,10	1,01	0,87	1,57	1,41	0,93	0,96	1,05	AWPM-19-like membrane family protein
259775_at	At1g29530	1,07	1,25	1,50	0,88	0,89	1,14	1,30	1,21	expressed protein
259776_s_at	At1g29550	1,14	1,13	1,03	1,01	0,95	1,06	1,06	1,07	eukaryotic translation initiation factor 4E, putative / eIF-4E, putative / eIF4E
259777_at	At1g29570	0,96	1,01	0,96	0,99	1,12	0,99	1,04	0,93	zinc finger protein-related
257507_at	At1g29600	1,00	1,05	0,86	0,99	0,95	1,07	1,04	1,08	zinc finger (CCCH-type) family protein
259778_at	At1g29610	1,04	0,94	1,07	0,97	1,04	0,94	1,03	1,09	hypothetical protein
259779_s_at	At1g29620	1,04	0,93	0,93	0,87	0,97	1,05	0,98	0,90	hypothetical protein
259780_at	At1g29630	1,04	1,06	0,98	1,07	0,90	0,97	0,92	0,70	exonuclease, putative
259781_at	At1g29650	1,13	1,15	1,06	1,09	1,10	0,95	0,98	0,85	---
259786_at	At1g29660	1,16	1,08	1,27	1,05	0,73	0,94	0,86	1,06	GDSL-motif lipase/hydrolase family protein
259788_at	At1g29670	0,83	0,84	1,06	0,88	0,99	0,93	0,87	0,86	GDSL-motif lipase/hydrolase family protein
259782_at	At1g29680	0,73	0,71	0,94	0,85	1,14	0,99	0,82	1,28	expressed protein
259792_at	At1g29690	0,79	1,05	1,72	0,89	1,07	1,08	1,42	1,37	expressed protein
259791_at	At1g29700	0,90	0,82	0,82	0,76	0,67	1,39	1,24	1,46	expressed protein
246632_at	At1g29710	0,98	1,04	0,89	1,12	1,00	0,97	1,05	1,15	pentatricopeptide (PPR) repeat-containing protein
246633_at	At1g29720	1,09	0,98	0,87	0,74	0,68	1,16	1,20	1,37	protein kinase family protein
255996_s_at	At1g29730	0,92	1,03	0,99	1,02	1,01	0,92	0,98	1,15	leucine-rich repeat transmembrane protein kinase, putative
255995_at	At1g29750	0,90	0,96	0,86	0,83	1,02	0,91	1,01	1,20	leucine-rich repeat transmembrane protein kinase, putative / serine/threonir
255994_at	At1g29760	0,80	1,01	1,00	1,04	0,92	1,02	1,18	1,40	expressed protein
255993_at	At1g29770	0,89	0,81	0,87	1,04	1,03	0,98	0,90	1,01	NLI interacting factor (NIF) family protein
255998_at	At1g29780	0,94	0,85	1,05	1,02	1,07	1,01	0,98	1,07	NLI interacting factor (NIF) family protein
255992_at	At1g29790	0,86	0,98	0,92	0,88	1,00	1,02	0,97	0,97	expressed protein
255991_at	At1g29820	0,93	0,98	0,93	1,03	1,38	0,82	1,07	0,89	expressed protein
256001_at	At1g29850	1,22	0,92	0,94	1,29	1,29	0,98	1,01	1,17	double-stranded DNA-binding family protein
255999_at	At1g29860	0,97	0,88	1,15	0,97	1,03	0,94	0,93	0,94	WRKY family transcription factor
255990_at	At1g29870	0,95	0,91	0,99	1,00	0,83	0,96	0,99	0,94	tRNA synthetase class II (G, H, P and S) family protein
256000_at	At1g29880	1,19	1,10	1,20	0,93	0,94	1,12	1,02	1,01	glycyl-tRNA synthetase / glycine--tRNA ligase
256002_at	At1g29900	1,36	1,16	1,23	0,80	0,85	0,93	0,90	0,86	carbamoyl-phosphate synthase family protein
255997_s_at	At1g29910	1,00	0,93	0,93	0,87	0,80	1,04	0,98	0,90	chlorophyll A-B binding protein 2, chloroplast / LHCII type I CAB-2 / CAB-14
260049_at	At1g29940	1,32	1,22	1,22	0,68	0,71	0,97	1,12	1,15	DNA-directed RNA polymerase family protein
260027_at	At1g29950	0,76	0,73	0,66	1,13	0,89	0,86	0,95	0,94	expressed protein
260050_at	At1g29960	0,99	1,03	0,86	0,96	1,02	0,98	1,10	1,03	MADS-box family protein
260026_at	At1g29970	0,86	0,84	0,80	0,94	0,96	0,96	1,02	1,19	expressed protein

260028_at	At1g29980	1,00	0,78	0,76	1,54	1,27	0,83	0,81	0,76	expressed protein
260020_at	At1g29990	1,57	1,38	1,22	0,98	1,06	1,20	1,15	1,06	prefoldin, putative
260029_at	At1g30000	0,89	0,89	0,84	1,02	1,06	1,06	0,97	1,02	glycoside hydrolase family 47 protein
260021_at	At1g30010	0,81	0,87	0,99	0,94	0,78	0,90	1,05	1,06	intron maturase, type II family protein
260022_at	At1g30020	0,94	0,94	0,90	0,90	1,11	1,18	1,02	0,84	expressed protein
260023_at	At1g30040	1,31	1,88	1,32	0,93	1,38	1,17	1,05	0,93	gibberellin 2-oxidase / GA2-oxidase (GA2OX2)
260025_at	At1g30070	2,00	1,76	1,69	1,53	1,79	1,10	1,21	1,19	SGS domain-containing protein
260024_at	At1g30080	1,09	1,10	0,92	1,09	1,65	1,00	1,07	0,89	glycosyl hydrolase family 17 protein
256161_at	At1g30090	1,19	1,21	1,52	0,79	1,03	0,96	1,05	1,09	kelch repeat-containing F-box family protein
256190_at	At1g30100	1,02	0,97	0,93	1,00	0,94	1,07	1,01	1,15	9-cis-epoxycarotenoid dioxygenase, putative / neoxanthin cleavage enzyme
256192_at	At1g30110	0,92	0,89	0,63	1,03	0,95	0,93	0,91	0,74	diadenosine 5',5'''-P1,P4-tetraphosphate hydrolase, putative
256160_at	At1g30120	1,07	0,94	0,89	1,02	0,90	0,96	0,87	0,88	pyruvate dehydrogenase E1 component beta subunit, chloroplast
256191_at	At1g30130	0,78	0,73	0,92	0,89	0,93	0,93	0,92	0,91	expressed protein
256159_at	At1g30135	0,87	3,10	1,73	1,38	1,28	1,30	2,03	1,72	expressed protein
256189_at	At1g30140	1,01	1,04	1,05	0,90	0,94	0,98	0,96	0,92	hypothetical protein
257588_x_at	At1g30150	0,99	1,09	1,08	1,01	0,90	1,00	0,98	0,79	---
256188_at	At1g30160	0,96	1,00	0,98	1,17	0,99	0,97	0,91	1,19	expressed protein
256193_at	At1g30200	0,87	0,93	1,04	0,88	0,84	0,89	1,02	1,13	F-box family protein
245774_at	At1g30210	0,82	0,80	0,73	0,73	0,97	0,84	0,77	0,80	TCP family transcription factor, putative
245769_at	At1g30220	1,04	1,11	1,01	1,00	1,12	1,00	1,02	1,02	sugar transporter family protein
245770_at	At1g30240	1,16	1,05	1,45	0,87	0,79	0,94	0,91	0,90	PELP1-related
245771_at	At1g30250	0,88	1,00	1,00	0,96	0,96	0,89	1,03	0,99	expressed protein
245776_at	At1g30260	0,72	0,79	0,90	0,73	0,90	1,15	1,11	0,83	expressed protein
245775_at	At1g30270	0,88	0,87	0,86	1,00	1,00	1,04	1,06	1,18	CBL-interacting protein kinase 23 (CIPK23)
245773_at	At1g30290	1,05	1,07	1,00	1,01	1,07	1,08	0,94	0,94	---
245772_at	At1g30300	0,99	0,88	0,90	1,12	1,07	0,96	0,85	0,91	expressed protein
256311_at	At1g30330	1,10	0,98	1,37	0,91	1,02	0,95	0,95	1,04	auxin-responsive factor (ARF6)
256307_at	At1g30350	0,98	1,00	1,19	1,02	0,97	1,07	1,01	0,92	pectate lyase family protein
256310_at	At1g30360	0,81	0,77	0,91	0,87	0,87	1,05	1,05	1,11	early-responsive to dehydration stress protein (ERD4)
256306_at	At1g30370	0,95	1,02	0,95	0,66	1,11	1,26	1,06	0,97	lipase class 3 family protein
256309_at	At1g30380	0,98	0,98	0,97	1,05	0,98	1,03	0,99	0,98	photosystem I reaction center subunit psaK, chloroplast, putative / photosys
256305_at	At1g30400	0,80	0,80	0,81	0,81	0,87	1,16	1,13	1,05	glutathione S-conjugate ABC transporter (MRP1)
256308_s_at	At1g30410	0,82	0,76	0,86	0,75	0,78	0,84	0,97	0,94	ATP-binding cassette transport protein, putative
261796_at	At1g30440	1,02	1,03	1,18	0,86	0,91	1,01	0,99	1,01	phototropic-responsive NPH3 family protein
261797_at	At1g30450	0,93	0,92	0,99	0,89	1,02	1,03	1,00	1,08	cation-chloride cotransporter, putative
261798_at	At1g30460	0,85	0,89	0,88	0,88	0,85	0,96	0,93	0,93	zinc finger (CCCH-type) family protein / YT521-B-like family protein
261799_at	At1g30470	0,95	1,03	0,90	1,09	1,11	0,94	1,05	0,81	protein coding
261808_at	At1g30480	1,43	1,37	1,84	0,88	0,89	1,28	1,15	1,21	DNA-damage-repair/toleration protein, chloroplast (DRT111)
261800_at	At1g30490	1,22	1,03	1,06	1,04	0,94	1,07	1,10	1,05	homeobox-leucine zipper transcription factor (HB-9)
261803_at	At1g30500	1,00	0,98	1,00	0,63	0,79	1,09	0,95	0,98	CCAAT-binding transcription factor (CBF-B/NF-YA) family protein
261806_at	At1g30510	0,90	0,84	0,75	1,91	1,61	0,76	0,91	0,72	ferredoxin--NADP(+) reductase, putative / adrenodoxin reductase, putative
261807_at	At1g30515	0,91	1,11	0,86	0,96	0,99	0,93	0,96	1,01	expressed protein
261801_at	At1g30520	1,11	1,41	1,58	0,78	0,71	1,10	1,07	1,27	acyl-activating enzyme 14 (AAE14)
261804_at	At1g30530	0,97	0,87	0,97	0,91	0,89	0,93	0,91	0,81	UDP-glucoronosyl/UDP-glucosyl transferase family protein
261805_at	At1g30540	0,92	1,00	1,03	0,98	0,95	0,86	0,76	0,92	ATPase, BadF/BadG/BcrA/BcrD-type family
261802_at	At1g30550	1,30	1,33	1,57	1,01	0,95	0,93	0,96	0,87	expressed protein

263213_at	At1g30560	0,93	0,94	1,05	0,96	0,91	1,01	0,96	0,83	transporter, putative
263218_at	At1g30570	0,71	0,83	0,90	0,76	0,81	1,03	0,78	0,85	protein kinase family protein
263224_at	At1g30580	1,24	1,06	1,03	1,11	1,22	0,94	0,98	0,91	expressed protein
263219_at	At1g30600	1,08	1,29	1,01	1,21	1,31	0,80	0,84	0,94	subtilase family protein
263220_at	At1g30610	1,01	0,96	0,97	0,69	0,63	1,18	1,08	1,45	pentatricopeptide (PPR) repeat-containing protein
263221_at	At1g30620	0,59	0,50	0,60	1,03	1,17	0,91	0,88	0,92	UDP-D-xylose 4-epimerase, putative (MUR4)
263223_at	At1g30630	1,22	1,02	1,09	1,29	1,35	0,98	0,99	0,88	coatomer protein epsilon subunit family protein / COPE family protein
263222_at	At1g30640	0,73	0,68	0,64	1,12	1,25	1,14	1,20	1,44	protein kinase, putative
263225_at	At1g30650	1,22	1,23	0,90	0,94	0,97	1,15	1,14	1,10	WRKY family transcription factor
263214_at	At1g30660	1,05	1,13	0,91	1,07	0,88	0,96	1,10	1,14	toprim domain-containing protein
263226_at	At1g30690	1,20	0,92	1,05	0,97	0,90	0,99	1,03	0,97	SEC14 cytosolic factor family protein / phosphoglyceride transfer family pro
263228_at	At1g30700	1,48	1,29	0,85	1,15	1,00	1,84	1,45	1,36	FAD-binding domain-containing protein
263215_at	At1g30710	1,05	1,12	0,94	1,03	0,99	1,04	0,97	1,15	FAD-binding domain-containing protein
263216_s_at	At1g30720	1,17	1,04	0,98	0,90	0,81	1,12	0,83	0,72	FAD-binding domain-containing protein
263217_at	At1g30740	1,03	1,06	0,96	1,00	1,07	1,03	0,95	0,88	FAD-binding domain-containing protein
263227_at	At1g30750	1,65	1,78	1,36	1,82	1,51	1,13	1,23	1,60	expressed protein
264527_at	At1g30760	1,22	0,96	0,94	1,31	1,13	1,21	1,68	1,69	FAD-binding domain-containing protein
264530_at	At1g30780	0,97	0,89	0,89	1,05	0,94	0,97	1,05	0,70	---
264531_at	At1g30790	0,91	0,93	1,01	0,93	0,99	0,92	0,97	1,06	F-box family protein
264499_at	At1g30795	0,91	0,96	1,13	0,97	0,98	0,95	1,00	1,07	hydroxyproline-rich glycoprotein family protein
257419_at	At1g30800	1,00	0,93	1,00	1,23	1,11	0,92	0,89	0,72	expressed protein
264528_at	At1g30810	0,55	0,85	1,04	0,70	0,62	0,97	1,12	0,98	transcription factor jumonji (jmj) family protein / zinc finger (C5HC2 type) fa
264529_at	At1g30820	1,14	1,28	1,40	0,64	0,77	1,05	1,19	1,22	CTP synthase, putative / UTP--ammonia ligase, putative
264498_at	At1g30825	1,05	1,08	1,04	1,10	0,93	0,92	0,79	0,91	actin-related protein 2/3 complex 34kDa subunit family / arp2/3 complex 34
264497_at	At1g30840	0,89	0,89	0,89	1,10	1,04	0,95	0,98	1,06	purine permease-related
257418_at	At1g30850	0,92	1,04	1,00	1,04	1,33	1,03	0,94	0,90	hypothetical protein
265128_at	At1g30860	0,83	1,07	0,99	0,75	1,01	0,92	1,00	0,89	expressed protein
265102_at	At1g30870	1,12	1,03	0,87	1,88	1,37	1,08	1,03	0,89	cationic peroxidase, putative
265101_at	At1g30880	1,71	1,51	1,55	1,31	1,41	1,11	1,09	1,19	expressed protein
265130_at	At1g30890	0,83	0,81	0,78	0,95	1,01	0,89	0,90	0,82	integral membrane HRF1 family protein
265161_at	At1g30900	1,13	1,26	1,00	1,56	1,44	1,20	1,29	1,43	vacuolar sorting receptor, putative
265162_at	At1g30910	0,80	0,81	0,83	0,98	0,93	0,78	0,87	0,82	molybdenum cofactor sulfurase family protein
265152_s_at	At1g30940	0,91	0,94	0,94	1,06	1,06	1,04	1,07	1,02	---
265153_at	At1g30950	0,92	1,06	1,01	1,05	0,96	0,94	1,00	1,03	unusual floral organ (UFO) / F-box family protein (FBX1)
265154_at	At1g30960	1,15	1,04	0,92	0,80	0,98	0,93	0,89	1,28	GTP-binding protein (ERG)
265129_at	At1g30970	1,23	1,16	1,13	0,97	1,19	1,09	0,95	1,00	zinc finger (C2H2 type) family protein
265155_at	At1g30990	1,24	1,40	1,15	1,29	1,26	0,98	1,06	0,90	major latex protein-related / MLP-related
265156_at	At1g31000	0,98	0,99	0,94	1,08	1,05	1,02	1,02	1,03	F-box family protein
265100_at	At1g31010	1,19	1,03	1,09	0,80	1,07	0,98	0,93	1,02	expressed protein
265104_at	At1g31020	1,15	1,07	0,93	0,96	0,99	0,97	0,93	0,82	thioredoxin o (TRXO2)
265157_at	At1g31030	0,98	1,16	1,05	0,95	1,09	1,02	1,00	0,99	---
265158_at	At1g31040	0,94	1,03	0,92	0,97	1,10	1,03	0,86	1,07	zinc-binding protein-related
265160_at	At1g31050	1,12	1,23	1,16	0,88	1,10	1,02	1,18	1,08	expressed protein
265159_at	At1g31060	1,12	1,07	1,21	1,03	1,00	1,00	0,97	1,07	expressed protein
265103_at	At1g31070	0,86	0,80	0,90	0,82	0,94	0,98	1,07	0,92	UDP-N-acetylglucosamine pyrophosphorylase-related
263697_at	At1g31080	1,00	1,00	1,07	0,99	0,98	1,15	1,11	1,11	F-box family protein

263698_at	At1g31100	1,01	1,13	0,95	0,99	1,09	0,92	0,92	0,73	---
263699_at	At1g31120	0,68	0,82	0,92	0,87	0,73	0,86	1,18	1,24	potassium transporter family protein
263704_at	At1g31130	0,90	0,92	0,85	1,04	0,97	1,02	1,17	1,10	expressed protein
263700_at	At1g31150	1,04	0,97	0,96	0,96	1,01	1,06	1,02	0,95	expressed protein
263701_at	At1g31160	1,00	0,92	0,74	0,96	0,86	0,99	0,97	1,02	zinc-binding protein, putative / protein kinase C inhibitor, putative
263703_at	At1g31170	0,89	0,82	0,93	0,83	0,83	1,09	1,06	1,03	parB-like nuclease domain-containing protein
263706_s_at	At1g31180	1,43	1,29	1,53	1,14	0,86	1,09	1,05	0,95	3-isopropylmalate dehydrogenase, chloroplast, putative
263705_at	At1g31190	1,03	1,17	1,20	0,79	0,71	1,42	1,35	1,08	inositol monophosphatase family protein
263693_at	At1g31200	1,24	1,44	1,20	1,14	1,38	1,23	1,05	1,00	expressed protein
263694_at	At1g31210	0,90	0,98	0,98	1,01	1,11	1,03	1,06	1,02	---
263695_at	At1g31220	0,76	0,82	1,11	0,83	0,81	1,09	0,95	0,88	phosphoribosylglycinamide formyltransferase
263696_at	At1g31230	1,11	0,84	0,84	0,95	0,78	0,94	0,93	0,84	bifunctional aspartate kinase/homoserine dehydrogenase / AK-HSDH
263702_at	At1g31240	0,97	1,14	0,89	1,01	1,09	0,87	0,87	0,94	expressed protein
262545_at	At1g31250	0,97	1,02	0,91	1,04	1,13	1,04	1,10	1,08	proline-rich family protein
262546_at	At1g31260	0,99	1,07	1,02	0,96	1,11	0,89	1,09	0,94	metal transporter, putative (ZIP10)
262547_at	At1g31270	0,97	0,92	1,00	0,92	0,94	0,97	0,97	1,24	hypothetical protein
262548_at	At1g31280	0,91	1,02	1,00	0,92	1,10	1,07	1,06	1,00	PAZ domain-containing protein / piwi domain-containing protein
262549_at	At1g31290	2,46	2,05	1,23	0,82	0,78	1,84	2,11	1,50	PAZ domain-containing protein / piwi domain-containing protein
262559_at	At1g31300	1,07	0,99	0,86	1,15	0,93	1,01	0,97	1,07	expressed protein
262550_at	At1g31310	0,97	1,15	0,95	1,13	1,07	1,14	0,90	1,28	hydroxyproline-rich glycoprotein family protein
257467_at	At1g31320	1,08	1,01	0,93	1,02	1,28	0,98	1,03	0,81	LOB domain protein 4 / lateral organ boundaries domain protein 4 (LBD4)
262557_at	At1g31330	0,95	0,96	0,99	0,97	0,84	0,86	0,91	0,89	photosystem I reaction center subunit III family protein
262558_at	At1g31335	1,12	1,08	1,12	1,26	1,62	0,77	0,74	0,99	expressed protein
262551_at	At1g31340	1,52	1,25	1,19	1,10	1,05	1,11	1,04	1,05	ubiquitin family protein
262552_at	At1g31350	0,93	0,85	0,94	0,97	0,92	0,89	0,86	0,81	F-box family protein
262553_at	At1g31360	0,94	0,97	1,05	0,93	0,81	0,94	1,14	0,95	DNA helicase, putative (RECQl2)
262556_at	At1g31370	1,01	0,92	0,99	0,99	1,14	0,94	1,04	0,91	expressed protein
262554_at	At1g31380	1,00	1,04	0,92	1,00	1,06	1,12	1,01	0,95	hypothetical protein
262555_at	At1g31400	1,00	1,00	0,92	1,05	1,00	0,97	1,18	0,91	meprin and TRAF homology domain-containing protein / MATH domain-cor
256483_at	At1g31410	0,88	0,99	0,84	1,01	0,85	1,01	0,88	0,95	putrescine-binding periplasmic protein-related
256482_at	At1g31420	0,93	0,96	0,84	0,79	0,88	1,00	0,90	0,92	leucine-rich repeat transmembrane protein kinase, putative
256484_at	At1g31430	0,90	0,98	0,95	0,89	1,02	1,13	1,03	1,29	pentatricopeptide (PPR) repeat-containing protein
256485_at	At1g31440	1,03	1,01	1,08	1,15	1,00	1,11	1,10	1,06	SH3 domain-containing protein 1 (SH3P1)
256486_at	At1g31450	1,01	0,98	1,05	0,84	0,95	1,05	0,95	0,94	aspartyl protease family protein
256490_at	At1g31460	0,82	0,84	0,90	1,28	1,07	1,00	0,76	1,04	expressed protein
256488_at	At1g31470	1,06	1,03	1,19	1,16	1,18	1,01	1,03	0,99	nodulin-related
256513_at	At1g31480	1,05	1,01	0,94	0,80	0,99	1,02	1,29	1,02	shoot gravitropism 2 (SGR2)
256492_at	At1g31490	1,02	1,04	1,08	1,08	0,94	1,07	0,95	1,03	transferase family protein
256491_at	At1g31500	1,08	0,83	0,87	0,72	0,85	1,08	1,02	0,99	endonuclease/exonuclease/phosphatase family protein
256496_at	At1g31510	0,95	1,01	1,09	1,10	0,95	1,01	1,04	0,97	F-box family protein-related
256494_at	At1g31520	1,06	0,88	1,03	0,93	0,96	1,09	0,89	0,99	hypothetical protein
256495_at	At1g31530	0,99	0,95	1,00	0,98	1,07	0,90	0,99	1,10	endonuclease/exonuclease/phosphatase family protein
256487_at	At1g31540	0,95	0,92	0,92	0,93	0,87	1,49	1,21	1,11	disease resistance protein (TIR-NBS-LRR class), putative
256489_at	At1g31550	0,81	0,83	0,80	0,96	0,84	0,78	0,74	0,62	GDSL-motif lipase, putative
256497_at	At1g31580	0,95	0,96	1,07	1,65	1,07	1,54	1,22	1,42	expressed protein
256493_at	At1g31600	1,03	1,19	1,15	1,00	0,77	1,20	1,18	1,23	oxidoreductase, 2OG-Fe(II) oxygenase family protein

246579_at	At1g31620	0,91	0,92	0,97	0,99	0,90	0,98	0,94	1,02	hypothetical protein
246578_at	At1g31630	1,01	0,99	0,98	1,03	0,91	1,11	1,03	1,08	MADS-box family protein
246577_at	At1g31640	0,94	0,98	1,01	0,93	0,97	0,86	0,88	1,03	MADS-box protein-related
246576_at	At1g31650	1,32	2,11	1,36	0,92	0,95	1,22	1,07	1,04	expressed protein
246575_at	At1g31660	1,27	1,26	1,34	1,01	0,88	0,96	0,97	0,89	bystin family
246574_at	At1g31670	0,99	1,03	1,01	1,17	0,94	1,18	0,85	1,06	copper amine oxidase, putative
246573_at	At1g31680	0,95	0,91	0,99	1,10	1,09	0,99	0,94	0,97	copper amine oxidase family protein
246603_at	At1g31690	0,98	0,98	0,93	0,97	1,30	0,83	0,85	0,79	copper amine oxidase, putative
246602_at	At1g31700	1,45	1,21	1,10	1,66	1,19	0,89	0,93	0,85	copper amine oxidase, putative
246601_at	At1g31710	1,49	1,33	1,11	1,35	0,97	0,94	0,91	0,77	copper amine oxidase, putative
246635_at	At1g31720	1,04	1,05	1,09	1,15	1,03	0,85	0,99	1,14	expressed protein
246634_at	At1g31730	0,97	0,92	0,94	1,05	0,99	1,00	0,90	0,86	epsilon-adaptin, putative
246582_at	At1g31750	0,95	1,13	1,02	0,96	0,99	1,05	0,99	1,01	proline-rich family protein
246581_at	At1g31760	1,06	1,10	1,19	1,12	1,15	0,87	0,94	0,87	SWIB complex BAF60b domain-containing protein
246580_at	At1g31770	1,21	0,87	0,86	1,64	1,36	1,13	1,39	1,31	ABC transporter family protein
246263_at	At1g31780	1,26	1,23	1,27	1,10	1,14	1,03	0,79	0,76	conserved oligomeric Golgi complex component-related / COG complex co
246262_at	At1g31790	1,12	0,86	0,92	0,82	0,69	1,02	1,02	0,89	pentatricopeptide (PPR) repeat-containing protein
246268_at	At1g31800	1,12	1,18	0,94	0,92	0,80	1,30	1,26	1,23	cytochrome P450 family protein
246261_at	At1g31810	1,12	0,93	1,02	0,64	0,62	1,29	1,11	0,95	formin homology 2 domain-containing protein / FH2 domain-containing prot
246267_at	At1g31812	1,07	0,94	0,92	1,11	1,08	0,95	0,87	0,90	acyl-CoA binding protein / ACBP
246264_at	At1g31814	1,01	1,13	0,93	0,99	1,04	1,10	0,92	1,12	expressed protein
246266_at	At1g31817	1,13	1,04	0,97	0,80	0,98	1,17	1,02	0,77	chloroplast 30S ribosomal protein S11, putative
246260_at	At1g31820	0,84	0,91	0,91	0,74	0,72	1,16	1,18	0,89	amino acid permease family protein
246259_at	At1g31830	0,96	0,80	0,74	0,93	1,13	0,84	0,72	0,61	amino acid permease family protein
246258_at	At1g31840	1,04	1,16	0,85	0,88	0,92	1,06	0,95	0,96	pentatricopeptide (PPR) repeat-containing protein
246288_at	At1g31850	0,73	0,62	0,64	0,87	0,75	0,78	0,73	0,83	dehydration-responsive protein, putative
246265_at	At1g31860	1,03	0,89	0,96	0,92	0,83	1,08	0,96	0,70	histidine biosynthesis bifunctional protein (HISIE)
246287_at	At1g31870	1,36	1,22	1,28	0,95	1,07	1,24	1,13	1,65	expressed protein
246286_at	At1g31910	0,99	1,02	1,04	0,80	0,74	1,03	1,02	0,98	GHMP kinase family protein
246313_at	At1g31920	0,94	1,14	0,99	0,87	0,87	1,09	0,87	1,21	pentatricopeptide (PPR) repeat-containing protein
246312_at	At1g31930	0,93	0,92	0,97	0,85	1,10	0,93	0,99	1,19	extra-large guanine nucleotide binding protein, putative / G-protein, putative
255721_at	At1g31940	0,87	0,82	1,13	0,88	0,96	1,11	1,05	0,89	expressed protein
255751_at	At1g31950	0,96	0,99	1,10	1,29	1,05	0,97	1,11	1,05	terpene synthase/cyclase family protein
255750_at	At1g31960	0,97	0,97	1,09	0,95	1,07	1,00	1,01	1,06	hypothetical protein
255749_at	At1g31970	1,38	1,29	1,19	0,81	0,84	1,14	1,13	1,14	DEAD/DEAH box helicase, putative
255748_at	At1g32000	1,01	0,89	1,07	1,07	0,86	1,02	0,98	0,83	hypothetical protein
255747_s_at	At1g32010	1,01	0,82	0,78	1,07	1,05	1,06	1,03	0,97	myosin heavy chain-related
255746_at	At1g32020	1,06	1,08	1,05	0,91	0,90	0,98	0,94	0,93	F-box family protein
255745_at	At1g32030	0,91	1,00	0,99	0,95	1,05	1,02	0,94	0,97	expressed protein
255744_at	At1g32040	0,87	0,97	1,00	0,97	1,29	1,00	0,99	0,81	hypothetical protein
255752_at	At1g32050	0,90	0,75	0,81	1,23	1,22	0,85	0,86	0,90	secretory carrier membrane protein (SCAMP) family protein
255720_at	At1g32060	1,17	1,00	1,13	0,84	0,94	1,24	1,16	1,07	phosphoribulokinase (PRK) / phosphopentokinase
255718_at	At1g32070	1,02	0,92	0,92	0,87	0,87	1,09	1,00	0,95	GCN5-related N-acetyltransferase (GNAT) family protein / nuclear shuttle ir
255719_at	At1g32080	0,95	0,97	0,91	0,77	0,77	1,22	1,11	1,09	membrane protein, putative
245789_at	At1g32090	0,90	1,11	1,23	0,90	0,94	0,93	1,01	1,11	early-responsive to dehydration protein-related / ERD protein-related
245792_at	At1g32100	1,66	1,61	1,63	1,62	1,49	0,99	0,93	1,02	pinoresinol-lariciresinol reductase, putative

245788_at	At1g32120	0,85	1,22	0,92	1,34	0,94	0,97	0,83	0,95	expressed protein
245787_at	At1g32130	1,13	1,06	1,24	1,07	1,30	1,02	1,05	1,11	IWS1 C-terminus family protein
245786_at	At1g32150	0,88	1,09	1,19	0,97	1,19	0,95	1,04	1,04	bZIP transcription factor family protein
245795_at	At1g32160	0,83	0,94	0,99	0,74	0,78	1,09	0,92	0,88	expressed protein
245794_at	At1g32170	1,18	1,26	0,99	1,37	1,44	1,11	1,32	1,34	xyloglucan:xyloglucosyl transferase, putative / xyloglucan endotransglycosy
245785_at	At1g32180	1,07	1,02	1,06	1,07	0,94	1,06	0,99	1,08	cellulose synthase family protein
245784_at	At1g32190	0,86	0,96	1,02	0,82	0,91	0,85	0,87	0,78	expressed protein
245790_at	At1g32200	0,92	0,76	0,71	0,69	0,74	0,96	1,03	0,87	glycerol-3-phosphate acyltransferase, chloroplast (ATS1)
245791_at	At1g32210	1,12	1,18	0,91	1,17	0,93	0,94	0,83	0,83	defender against cell death 1 (DAD1)
245793_at	At1g32220	1,07	1,06	0,88	0,99	0,88	1,31	1,10	1,15	expressed protein
245796_at	At1g32230	1,05	1,11	1,90	0,95	0,96	1,20	1,32	1,97	WWE domain-containing protein / ceo protein, putative (CEO)
245758_at	At1g32240	1,11	1,11	0,95	0,86	1,02	1,01	1,01	0,95	myb family transcription factor (KAN2)
260702_at	At1g32250	1,01	1,00	0,98	1,07	1,27	0,98	0,94	1,08	calmodulin, putative
260700_at	At1g32260	1,10	1,17	1,13	1,11	1,26	0,95	0,82	0,95	expressed protein
260703_at	At1g32270	0,93	1,02	1,04	0,99	0,94	0,94	1,18	1,07	syntaxin, putative
260689_at	At1g32290	0,88	1,01	0,98	0,98	1,07	0,99	1,16	0,98	hypothetical protein
260698_at	At1g32300	1,09	1,01	1,04	0,93	1,05	0,97	1,07	1,08	FAD-binding domain-containing protein
260708_at	At1g32310	1,05	0,90	0,71	1,20	1,07	1,07	0,77	0,75	expressed protein
260699_at	At1g32320	0,89	1,02	1,00	1,04	1,12	1,07	0,85	0,83	mitogen-activated protein kinase kinase (MAPKK), putative (MKK10)
260701_at	At1g32330	1,04	0,89	0,85	1,09	1,15	0,98	0,84	1,00	heat shock transcription factor family protein
260690_at	At1g32340	0,93	0,77	0,82	0,84	0,86	1,03	0,97	1,01	zinc finger (C3HC4-type RING finger) family protein
260706_at	At1g32350	1,23	1,23	1,36	0,99	1,17	1,18	1,31	1,31	alternative oxidase, putative
260652_at	At1g32360	0,83	0,97	1,08	0,88	0,84	0,88	1,09	1,27	zinc finger (CCCH-type) family protein
260650_at	At1g32370	1,13	1,15	1,14	1,08	1,17	0,87	1,07	1,13	tobamovirus multiplication protein 2B (TOM2B)
257494_at	At1g32375	0,96	1,03	0,90	1,03	1,25	0,79	0,93	0,99	F-box family protein
260691_s_at	At1g32390	0,88	0,89	0,84	1,06	0,96	0,96	1,00	0,98	hypothetical protein
260705_at	At1g32400	0,85	0,84	0,87	1,25	1,19	0,91	0,85	0,92	senescence-associated family protein
260707_s_at	At1g32410	0,86	0,78	0,72	1,10	0,87	0,89	0,84	0,92	vacuolar protein sorting 55 family protein / VPS55 family protein
260692_at	At1g32430	1,11	1,00	0,90	0,90	1,00	1,04	0,93	0,81	F-box family protein
260653_at	At1g32440	0,99	0,79	0,92	1,04	1,14	1,05	0,97	0,88	pyruvate kinase, putative
260693_at	At1g32450	0,49	0,82	0,88	1,46	1,63	0,45	0,67	0,81	proton-dependent oligopeptide transport (POT) family protein
260651_at	At1g32460	1,05	0,95	1,31	1,02	1,23	1,01	1,01	1,23	expressed protein
260704_at	At1g32470	1,23	1,12	1,14	1,04	1,20	1,10	1,06	1,18	glycine cleavage system H protein, mitochondrial, putative
260694_at	At1g32480	0,91	1,06	1,06	0,93	0,97	1,18	1,03	0,95	isocitrate/isopropylmalate dehydrogenase family protein
260695_at	At1g32490	1,08	1,26	1,31	0,87	1,01	1,24	1,19	1,37	RNA helicase, putative
260709_at	At1g32500	1,16	0,98	1,06	0,90	0,95	0,95	0,93	0,88	ATP-binding-cassette transporter, putative
260696_at	At1g32520	1,26	1,14	1,40	0,90	0,65	1,16	1,09	1,22	expressed protein
260697_at	At1g32530	1,11	1,43	1,62	0,89	0,92	1,18	1,42	0,93	zinc finger (C3HC4-type RING finger) family protein
256469_at	At1g32540	1,23	1,46	1,94	0,69	0,54	0,77	0,83	0,87	zinc finger protein, putative
256468_at	At1g32550	1,05	0,97	0,98	0,73	0,80	1,15	1,09	1,11	ferredoxin family protein
256464_at	At1g32560	0,27	0,35	0,37	0,85	1,11	0,97	0,92	0,82	late embryogenesis abundant group 1 domain-containing protein / LEA grou
256465_at	At1g32570	1,05	1,02	1,01	1,03	0,98	0,93	0,85	1,05	hypothetical protein
256466_at	At1g32580	1,43	1,27	1,28	0,93	0,91	0,97	0,87	0,85	plastid developmental protein DAG, putative
256467_at	At1g32590	0,93	0,99	0,96	0,95	1,08	1,11	1,00	0,99	---
261704_at	At1g32600	1,02	0,95	1,01	0,99	0,93	1,03	0,97	1,13	F-box family protein-related
261697_at	At1g32610	0,93	0,98	0,93	0,83	1,15	1,03	0,99	0,78	hydroxyproline-rich glycoprotein family protein

261698_at	At1g32630	0,83	0,88	1,03	1,05	1,10	0,99	1,03	1,02	expressed protein
261713_at	At1g32640	0,66	0,89	0,79	0,71	0,86	0,89	0,97	0,99	basic helix-loop-helix (bHLH) protein (RAP-1)
261707_at	At1g32650	0,94	1,02	0,94	0,99	1,14	1,01	0,98	1,06	hypothetical protein
261699_at	At1g32660	0,87	1,04	1,07	0,99	1,07	1,00	1,07	1,02	F-box family protein
261705_at	At1g32670	0,93	1,09	1,13	1,10	0,90	0,95	0,99	1,19	hypothetical protein
261706_at	At1g32680	0,97	0,95	1,03	1,06	0,97	1,05	0,95	0,98	hypothetical protein
261700_at	At1g32690	0,88	0,86	1,02	1,49	1,49	0,66	0,93	1,05	expressed protein
261711_at	At1g32700	1,40	1,54	1,37	0,84	1,11	1,04	1,10	1,37	zinc-binding family protein
261710_at	At1g32730	0,91	1,10	1,01	0,90	0,93	1,06	1,13	1,18	expressed protein
261708_at	At1g32740	0,98	0,91	1,14	0,94	1,00	0,97	0,92	1,10	expressed protein
261701_at	At1g32750	0,83	0,96	0,70	1,11	0,98	1,20	1,09	1,02	HAC13 protein (HAC13)
261702_at	At1g32760	1,02	0,90	0,97	1,04	0,82	0,97	1,03	1,10	glutaredoxin family protein
261703_at	At1g32770	1,15	1,03	1,08	0,95	1,21	1,02	1,03	0,92	no apical meristem (NAM) family protein
261712_at	At1g32780	1,05	1,02	1,13	1,02	1,02	0,94	1,26	1,28	alcohol dehydrogenase, putative
261709_at	At1g32790	0,80	0,86	0,96	0,98	1,03	0,94	0,91	0,78	RNA-binding protein, putative
261233_at	At1g32800	1,17	1,03	1,03	0,99	0,89	0,96	1,07	1,18	PHD finger protein-related
261238_at	At1g32810	1,11	1,09	1,18	0,84	0,78	1,29	1,31	1,34	expressed protein
261234_x_at	At1g32830	0,90	1,06	1,08	1,03	0,94	1,00	0,97	0,99	hypothetical protein
261235_x_at	At1g32840	0,83	0,97	1,10	0,97	1,12	0,87	1,22	0,77	Ulp1 protease family protein
261217_at	At1g32850	1,01	0,94	0,96	0,95	1,00	0,89	1,08	0,97	ubiquitin carboxyl-terminal hydrolase family protein
261187_at	At1g32860	1,09	0,81	0,81	1,71	1,68	1,27	1,02	0,87	glycosyl hydrolase family 17 protein
261192_at	At1g32870	1,06	1,14	1,14	0,94	1,02	1,23	1,20	1,13	no apical meristem (NAM) family protein
261236_at	At1g32880	0,95	0,99	1,12	0,97	1,03	1,01	1,03	1,15	importin alpha-1 subunit, putative
261191_at	At1g32900	1,06	0,86	0,83	1,57	0,94	1,43	1,24	1,06	starch synthase, putative
261193_at	At1g32920	0,96	1,00	1,09	0,94	0,87	1,20	1,59	1,61	expressed protein
261239_at	At1g32930	1,05	0,93	0,93	1,11	0,80	0,96	0,76	1,39	galactosyltransferase family protein
261240_at	At1g32940	1,10	1,07	1,09	1,09	1,17	1,12	1,12	1,35	subtilase family protein
261241_at	At1g32950	0,97	0,98	1,01	1,05	1,24	1,14	0,93	0,79	subtilase family protein
261242_at	At1g32960	0,61	0,54	0,51	0,91	1,00	0,89	0,97	0,94	subtilase family protein
261215_at	At1g32970	0,98	1,12	0,86	1,02	1,03	1,19	1,27	0,99	subtilase family protein
261237_at	At1g32980	1,00	1,05	1,03	1,02	1,01	0,95	1,05	0,96	subtilisin-like serine protease-related
261190_at	At1g32990	1,06	0,81	0,87	0,81	0,87	1,00	0,93	1,03	ribosomal protein L11 family protein
261188_at	At1g33000	0,99	0,98	0,97	1,03	0,96	0,96	1,08	1,02	hypothetical protein
261216_at	At1g33030	0,57	0,56	0,52	0,93	1,04	0,88	0,76	0,64	O-methyltransferase family 2 protein
261189_at	At1g33040	1,26	1,09	1,06	1,29	1,16	1,05	0,97	1,11	nascent polypeptide-associated complex (NAC) domain-containing protein
261615_at	At1g33050	0,62	0,74	1,08	0,73	0,81	0,89	1,00	1,17	expressed protein
261567_at	At1g33055	1,07	1,02	0,98	0,89	1,23	0,38	0,55	0,86	expressed protein
261616_at	At1g33060	1,03	1,00	1,11	0,78	1,15	1,07	1,03	1,05	no apical meristem (NAM) family protein
261595_at	At1g33070	1,01	1,02	1,03	1,08	1,02	0,86	0,91	0,93	hypothetical protein
261596_at	At1g33080	0,70	0,81	0,91	0,82	0,89	0,90	1,00	1,05	MATE efflux family protein
261617_s_at	At1g33090	0,96	0,89	1,01	1,24	0,98	1,02	0,95	0,88	MATE efflux family protein
261618_at	At1g33110	0,94	1,04	1,18	0,70	0,81	1,24	1,37	2,05	MATE efflux family protein
261619_x_at	At1g33130	0,94	1,08	0,90	0,96	1,12	1,06	1,13	1,32	---
261620_s_at	At1g33140	1,28	1,17	1,00	1,14	0,94	1,07	0,92	0,93	60S ribosomal protein L9 (RPL90B)
261593_at	At1g33170	1,28	1,09	1,20	1,21	1,03	1,11	0,93	1,04	dehydration-responsive family protein
261565_at	At1g33220	0,87	1,00	0,97	0,99	1,04	1,02	0,86	1,06	beta-1,3-glucanase, putative

261566_at	At1g33230	0,97	0,90	1,00	1,11	1,17	0,93	1,05	1,20	expressed protein
261594_at	At1g33240	1,19	1,51	1,65	0,79	0,86	1,16	1,40	1,49	trihelix DNA-binding protein, putative
256533_at	At1g33250	1,00	1,02	0,94	0,85	1,04	0,95	1,00	0,94	fringe-related protein
256529_at	At1g33260	1,00	1,01	1,04	1,24	1,14	1,03	0,96	0,96	protein kinase family protein
256512_at	At1g33265	0,80	0,78	0,60	1,09	0,92	0,80	0,84	0,85	expressed protein
256534_at	At1g33270	0,96	0,96	0,97	1,04	0,95	0,96	0,91	0,96	patatin-related
256535_at	At1g33280	1,20	1,06	1,07	1,09	1,05	1,02	0,94	1,00	no apical meristem (NAM) family protein
256530_at	At1g33290	1,23	0,99	0,90	0,75	0,79	1,20	1,15	1,04	sporulation protein-related
256531_at	At1g33320	1,01	1,06	0,92	1,05	1,22	1,00	0,96	0,94	cystathionine gamma-synthase, chloroplast, putative / O-succinylhomoserir
256536_at	At1g33330	0,96	0,97	0,83	0,86	0,85	1,08	0,87	0,95	peptide chain release factor, putative
256537_at	At1g33340	1,38	0,97	0,80	1,77	1,16	1,43	1,06	0,99	epsin N-terminal homology (ENTH) domain-containing protein / clathrin ass
256532_at	At1g33350	0,95	0,99	1,00	0,96	0,96	0,95	0,91	0,92	pentatricopeptide (PPR) repeat-containing protein
256510_at	At1g33360	0,87	0,98	0,99	0,82	0,97	1,01	1,21	1,01	ATP-dependent Clp protease ATP-binding subunit ClpX, putative
256511_at	At1g33390	1,00	0,79	1,05	0,88	0,94	0,82	0,96	1,11	helicase domain-containing protein
256479_at	At1g33400	1,19	0,99	1,11	0,77	0,65	0,93	1,07	0,95	tetratricopeptide repeat (TPR)-containing protein
256480_at	At1g33410	0,98	0,91	0,94	0,90	0,80	0,90	1,01	1,08	expressed protein
256426_at	At1g33420	1,15	1,33	1,01	0,96	0,88	1,24	1,07	0,87	PHD finger family protein
256481_at	At1g33430	1,05	0,95	0,92	0,92	1,04	0,98	0,95	1,13	galactosyltransferase family protein
256447_at	At1g33440	1,04	1,04	1,22	0,98	0,92	0,93	0,98	0,85	proton-dependent oligopeptide transport (POT) family protein
256448_s_at	At1g33450	0,96	0,85	0,93	0,97	1,09	0,98	0,98	1,03	hypothetical protein
256449_at	At1g33460	1,00	1,10	1,03	1,10	1,04	0,99	0,98	0,82	Mutator-like transposase family
256419_at	At1g33470	1,09	1,02	1,04	1,29	1,07	0,93	0,90	0,83	RNA recognition motif (RRM)-containing protein
256420_at	At1g33480	0,93	1,05	1,11	0,78	1,04	1,05	1,05	1,27	zinc finger (C3HC4-type RING finger) family protein
256424_at	At1g33490	0,97	1,15	1,05	0,90	0,86	1,21	1,18	1,17	expressed protein
256421_at	At1g33500	0,95	0,99	1,05	0,93	1,08	0,98	0,99	1,02	hypothetical protein
256422_at	At1g33520	0,88	0,93	1,04	0,91	0,84	1,09	0,93	0,96	KOW domain-containing protein / D111/G-patch domain-containing protein
256423_at	At1g33540	1,02	1,08	0,97	1,01	1,00	1,01	1,03	0,88	serine carboxypeptidase S10 family protein
256425_at	At1g33560	0,99	1,25	1,54	1,33	1,25	1,46	1,28	1,32	disease resistance protein (CC-NBS-LRR class), putative
245766_at	At1g33580	1,01	0,91	0,97	0,93	1,12	0,90	1,05	1,00	---
245768_at	At1g33590	0,92	0,85	0,85	0,68	0,90	0,89	0,75	0,69	disease resistance protein-related / LRR protein-related
245765_at	At1g33600	1,07	0,58	0,61	0,62	0,79	0,93	0,65	0,68	leucine-rich repeat family protein
245767_at	At1g33610	0,99	0,90	0,97	1,03	1,21	1,08	1,12	0,98	leucine-rich repeat family protein
261994_at	At1g33640	1,02	1,00	1,02	0,92	0,99	0,95	0,97	0,88	hypothetical protein
261990_at	At1g33660	1,02	0,97	0,93	0,97	0,99	0,94	0,85	0,91	peroxidase family protein
261989_at	At1g33670	0,96	0,84	1,04	1,12	1,04	0,98	1,00	1,07	leucine-rich repeat family protein
261988_at	At1g33680	1,13	1,27	1,43	0,83	0,73	1,10	1,18	1,16	KH domain-containing protein
261992_at	At1g33690	1,40	1,40	1,41	0,91	0,93	1,21	1,13	1,21	KH domain-containing protein
261991_at	At1g33700	1,21	1,47	2,02	1,58	1,26	1,01	1,67	1,59	expressed protein
261987_at	At1g33710	0,92	1,04	1,11	0,99	1,12	1,19	1,03	0,87	expressed protein
261986_s_at	At1g33720	1,59	2,44	1,54	2,74	1,88	1,92	3,04	1,08	cytochrome P450, putative
261985_at	At1g33750	1,04	1,04	1,01	1,18	1,29	0,93	1,07	0,95	terpene synthase/cyclase family protein
261984_at	At1g33760	1,34	1,42	1,03	1,50	1,15	1,04	1,24	0,78	AP2 domain-containing transcription factor, putative
261983_at	At1g33770	0,88	0,95	1,11	1,05	0,95	1,02	1,01	1,01	protein kinase family protein
261982_at	At1g33780	0,96	1,04	1,14	0,82	0,96	0,92	0,90	0,94	expressed protein
262001_at	At1g33790	1,10	1,00	1,38	1,89	1,67	1,00	1,02	0,96	jacalin lectin family protein
261999_at	At1g33800	1,35	1,10	0,76	1,62	1,10	0,84	1,06	1,15	expressed protein

262000_at	At1g33810	1,17	0,98	0,94	0,82	1,01	0,98	0,96	0,97	expressed protein
261981_at	At1g33811	0,85	0,80	0,80	0,94	0,99	0,61	0,68	0,68	GDSL-motif lipase/hydrolase family protein
261998_at	At1g33813	1,04	0,99	1,02	0,97	1,07	0,89	1,02	0,93	---
261997_at	At1g33817	0,94	1,05	1,06	0,92	1,12	1,01	1,08	0,92	---
261980_at	At1g33820	0,98	0,94	1,16	1,02	1,06	0,96	1,04	0,99	hypothetical protein
261996_at	At1g33830	1,01	0,95	1,06	0,92	0,96	1,07	0,93	0,95	avirulence-responsive family protein / avirulence induced gene (AIG1) famil
257473_at	At1g33840	0,98	1,11	0,98	1,17	1,06	1,10	0,96	0,92	hypothetical protein
261995_at	At1g33850	0,96	1,03	1,03	0,95	1,00	1,00	1,02	1,06	---
261993_at	At1g33860	0,99	0,99	1,11	0,94	1,00	0,98	1,06	0,92	hypothetical protein
260115_at	At1g33870	1,11	1,01	0,98	1,02	0,92	1,05	1,10	1,17	avirulence-responsive protein, putative / avirulence induced gene protein, p
260114_at	At1g33880	0,98	1,00	0,90	1,03	1,06	0,93	0,90	0,86	avirulence-responsive family protein / avirulence induced gene (AIG1) famil
260123_at	At1g33890	1,05	1,13	0,99	1,10	1,04	1,01	1,06	0,88	avirulence-responsive protein, putative / avirulence induced gene protein, p
260122_at	At1g33900	0,91	0,90	0,96	0,92	1,00	1,06	1,05	1,10	avirulence-responsive protein, putative / avirulence induced gene protein, p
260121_at	At1g33910	0,94	1,00	1,03	1,05	1,05	0,82	0,96	0,98	avirulence-responsive family protein / avirulence induced gene (AIG1) famil
260120_at	At1g33920	1,00	1,09	1,05	1,01	1,06	1,07	0,99	1,07	lectin-related
260119_at	At1g33930	0,95	1,02	0,98	1,02	1,05	0,99	0,98	1,11	avirulence-responsive family protein / avirulence induced gene (AIG1) famil
260118_s_at	At1g33940	1,36	1,31	1,36	1,06	0,90	0,96	0,94	0,90	hypothetical protein
260117_at	At1g33950	1,04	0,93	0,91	0,93	1,14	1,00	0,92	1,00	avirulence-responsive family protein / avirulence induced gene (AIG1) famil
260116_at	At1g33960	0,87	0,85	0,80	1,04	1,14	1,23	1,42	1,24	avirulence-responsive protein / avirulence induced gene (AIG1)
255980_at	At1g33970	0,81	0,81	1,39	0,78	0,88	1,08	1,16	1,29	avirulence-responsive protein, putative / avirulence induced gene protein, p
255979_at	At1g33980	1,01	1,18	1,27	0,89	0,78	1,08	1,09	1,19	Smg-4/UPF3 family protein
255983_at	At1g33990	0,98	0,96	1,27	1,00	1,07	1,07	0,98	1,12	hydrolase, alpha/beta fold family protein
255982_at	At1g34000	1,10	1,10	1,11	0,93	1,02	1,16	0,99	1,10	light stress-responsive one-helix protein (OHP2)
255978_at	At1g34010	1,04	1,00	1,05	1,02	0,85	1,07	0,99	0,67	expressed protein
255981_at	At1g34020	0,94	0,98	1,02	0,91	1,06	1,00	0,90	0,86	transporter-related
255977_at	At1g34030	1,40	1,26	1,05	0,97	0,96	1,01	0,99	0,85	40S ribosomal protein S18 (RPS18B)
256008_s_at	At1g34040	0,98	0,96	0,93	1,43	1,28	1,05	1,07	1,02	alliinase family protein
256007_at	At1g34065	1,01	0,99	1,01	0,97	1,19	1,01	1,12	0,85	mitochondrial substrate carrier family protein
256006_at	At1g34070	0,94	0,92	0,96	0,98	1,12	0,99	0,87	0,97	hypothetical protein
256005_at	At1g34080	0,93	1,10	1,02	1,02	1,15	1,11	1,00	1,01	---
256032_at	At1g34090	0,92	1,15	0,97	1,01	1,00	0,99	1,00	0,88	---
256031_at	At1g34100	0,97	0,94	0,94	0,88	1,07	0,92	0,96	1,00	---
256030_at	At1g34110	0,82	1,27	1,03	0,85	0,89	1,18	0,98	1,12	leucine-rich repeat transmembrane protein kinase, putative
255984_at	At1g34120	0,96	0,80	1,08	0,86	0,88	1,08	1,07	1,22	inositol polyphosphate 5-phosphatase I (IP5PI)
256029_at	At1g34130	0,93	0,92	0,94	0,93	0,93	1,01	0,97	0,96	oligosaccharyl transferase STT3 subunit, putative
256028_at	At1g34140	0,97	1,10	0,99	0,95	0,91	1,07	0,98	1,07	polyadenylate-binding protein, putative / PABP, putative
255985_at	At1g34150	0,94	0,83	0,97	0,93	0,93	1,03	1,08	1,07	tRNA pseudouridine synthase family protein
256027_at	At1g34160	1,01	0,94	0,82	1,02	0,91	1,07	1,03	0,73	pentatricopeptide (PPR) repeat-containing protein
256026_at	At1g34170	0,95	1,06	1,00	1,00	1,21	0,91	1,01	1,02	transcriptional factor B3 family protein
262542_at	At1g34180	0,94	1,58	1,66	1,21	1,30	1,13	1,03	1,06	no apical meristem (NAM) family protein
262514_at	At1g34190	1,06	1,10	1,35	1,18	1,20	1,09	1,01	1,19	no apical meristem (NAM) family protein
262515_at	At1g34200	0,71	0,76	0,83	0,76	0,98	0,87	0,97	0,91	oxidoreductase family protein
262563_at	At1g34210	0,68	0,56	0,46	0,93	0,80	0,91	0,95	1,04	somatic embryogenesis receptor-like kinase 2 (SERK2)
262562_at	At1g34220	0,91	0,96	1,17	0,91	1,09	1,06	1,03	1,15	expressed protein
262541_at	At1g34240	0,94	0,97	1,04	0,97	1,01	0,98	0,94	1,00	---
262543_at	At1g34245	1,01	1,03	1,10	1,04	1,13	0,87	0,95	1,11	expressed protein

262540_at	At1g34260	0,56	0,63	0,65	0,93	1,07	0,86	1,10	1,11	phosphatidylinositol-4-phosphate 5-kinase family protein
262513_at	At1g34270	1,14	0,89	0,99	1,40	1,29	0,83	0,84	0,83	exostosin family protein
262560_at	At1g34280	0,89	0,99	1,01	0,98	0,88	0,85	1,19	0,93	hypothetical protein
262568_at	At1g34290	0,99	1,07	1,02	1,03	1,04	1,07	0,94	0,94	leucine-rich repeat family protein
262567_at	At1g34300	0,61	0,68	0,71	0,82	0,85	1,09	0,84	0,87	lectin protein kinase family protein
262566_at	At1g34310	1,20	1,27	1,20	1,01	1,09	1,09	0,99	0,95	transcriptional factor B3 family protein / auxin-responsive factor AUX/IAA-re
262565_at	At1g34320	0,85	0,83	0,86	0,94	0,84	0,94	0,97	1,15	expressed protein
262564_at	At1g34330	0,95	1,04	0,97	1,11	1,08	1,03	0,99	1,11	---
262561_at	At1g34340	0,79	0,80	0,86	0,81	0,87	0,84	1,02	1,02	esterase/lipase/thioesterase family protein
259933_at	At1g34350	1,07	0,87	0,89	1,28	1,06	0,96	0,80	0,75	expressed protein
259930_at	At1g34355	1,05	0,96	1,15	1,37	1,03	1,11	0,77	0,90	forkhead-associated domain-containing protein / FHA domain-containing pr
259929_at	At1g34360	1,07	0,96	1,09	1,01	1,08	0,98	0,91	1,06	translation initiation factor 3 (IF-3) family protein
259932_at	At1g34370	0,97	1,05	1,13	0,92	1,03	0,94	0,92	1,10	zinc finger (C2H2 type) family protein
259928_at	At1g34380	1,01	0,75	1,04	0,72	0,76	0,93	0,86	0,81	5'-3' exonuclease family protein
259931_at	At1g34400	0,87	0,93	0,91	1,01	1,01	0,89	0,98	0,86	hypothetical protein
261163_x_at	At1g34410	1,00	1,01	1,03	1,05	1,01	1,03	0,94	1,19	transcriptional factor B3 family protein / auxin-responsive factor AUX/IAA-re
261161_at	At1g34420	0,79	1,04	0,93	0,88	1,21	1,13	0,92	1,15	leucine-rich repeat family protein / protein kinase family protein
261165_at	At1g34430	1,01	0,95	0,87	0,98	1,03	0,81	0,82	0,82	dihydrolipoamide S-acetyltransferase, putative
261162_at	At1g34440	1,12	0,99	0,96	1,11	1,10	1,12	0,99	1,02	hypothetical protein
261159_s_at	At1g34460	1,46	1,06	1,39	1,56	1,80	1,10	1,15	1,04	cyclin, putative
261164_at	At1g34470	1,08	0,96	0,84	1,13	1,24	1,07	0,87	0,89	permease-related
261160_at	At1g34480	1,15	1,13	1,18	0,99	0,93	1,07	0,99	0,83	DC1 domain-containing protein
261158_at	At1g34500	1,01	0,96	0,98	1,08	1,04	0,95	0,89	0,89	membrane bound O-acyl transferase (MBOAT) family protein / wax synthas
261157_at	At1g34510	1,09	1,15	0,86	1,01	1,20	0,96	0,95	0,99	peroxidase, putative
261156_s_at	At1g34520	0,97	0,96	1,02	1,03	0,96	1,02	1,08	0,91	membrane bound O-acyl transferase (MBOAT) family protein / wax synthas
261186_at	At1g34530	0,95	1,07	1,00	1,01	1,03	0,96	1,10	0,94	---
261185_at	At1g34540	0,99	1,00	1,10	0,94	0,94	0,98	1,06	0,92	cytochrome P450 family protein
261184_at	At1g34545	0,94	1,15	0,98	1,17	1,09	0,89	1,03	0,87	---
261183_at	At1g34550	0,77	0,69	0,62	1,02	1,06	0,95	0,91	0,92	expressed protein
261166_s_at	At1g34570	0,95	0,82	1,08	0,79	0,90	1,05	0,99	1,01	expressed protein
261182_at	At1g34575	0,99	0,98	1,04	1,06	1,09	1,09	0,89	0,80	FAD-binding domain-containing protein
261181_at	At1g34580	0,97	0,86	0,85	1,10	0,90	0,84	1,02	0,93	monosaccharide transporter, putative
261180_at	At1g34590	1,00	0,94	0,97	1,04	1,13	1,02	1,00	1,01	hypothetical protein
261214_at	At1g34620	0,98	0,95	1,00	0,97	1,12	1,00	0,95	0,99	---
262407_at	At1g34630	0,86	0,87	1,17	0,93	0,92	0,86	0,95	1,48	expressed protein
262411_at	At1g34640	1,11	1,23	1,37	1,05	0,91	1,18	1,02	0,98	expressed protein
262405_at	At1g34650	1,03	1,13	1,07	1,04	1,14	1,10	1,03	1,14	homeobox-leucine zipper family protein / lipid-binding START domain-conta
262404_at	At1g34660	1,00	0,98	1,10	0,98	1,00	0,84	1,03	1,13	CACTA-like transposase family (En/Spm)
262406_at	At1g34670	0,97	1,10	1,03	1,03	1,14	1,01	1,00	0,82	myb family transcription factor
262403_s_at	At1g34740	1,02	1,02	0,99	1,11	0,94	0,96	0,90	1,03	Ulp1 protease family protein
262408_at	At1g34750	0,62	0,56	0,79	1,58	1,57	0,90	1,03	0,97	protein phosphatase 2C, putative / PP2C, putative
262412_at	At1g34760	0,79	0,80	0,86	0,99	1,23	0,82	0,86	0,88	14-3-3 protein GF14 omicron (GRF11)
262410_at	At1g34770	1,03	1,14	1,02	1,13	1,18	1,04	1,00	0,84	MAGE-8 antigen-related
262413_at	At1g34780	0,82	0,80	1,03	0,76	0,78	0,80	0,82	1,01	protein disulfide isomerase-related
262409_at	At1g34842	1,05	0,97	0,98	1,10	0,93	1,13	1,08	1,16	---
257343_s_at	At1g34967	1,08	0,92	0,86	0,91	1,05	0,94	1,10	1,01	---

261391_at	At1g35030	1,07	1,14	1,01	1,10	1,07	1,03	0,94	0,87	hypothetical protein
261390_at	At1g35040	0,90	1,07	1,20	1,02	0,99	0,95	1,03	1,17	hypothetical protein
261389_s_at	At1g35050	1,03	1,02	1,10	1,06	1,08	1,02	1,02	1,04	---
245757_at	At1g35140	1,07	1,02	1,22	1,03	0,97	1,21	1,45	1,71	phosphate-responsive protein, putative
245753_at	At1g35143	1,00	1,02	1,10	0,98	1,04	0,97	1,01	1,02	hypothetical protein
245752_at	At1g35150	1,04	0,96	0,98	1,16	0,93	0,95	1,02	1,10	hypothetical protein
245783_s_at	At1g35180	1,40	1,40	1,29	1,14	1,44	1,04	0,95	0,92	expressed protein
245754_at	At1g35183	0,98	0,96	1,00	0,96	1,00	1,05	1,02	0,92	expressed protein
245756_at	At1g35190	1,11	1,12	1,18	1,24	1,30	1,31	1,23	1,17	oxidoreductase, 2OG-Fe(II) oxygenase family protein
245782_at	At1g35200	0,95	1,09	0,83	1,01	0,99	0,94	1,00	1,03	---
245755_at	At1g35210	2,06	1,43	1,09	0,99	0,73	1,61	1,25	1,26	expressed protein
259600_at	At1g35220	1,02	0,88	0,87	1,09	1,25	0,94	0,82	0,85	expressed protein
259550_at	At1g35230	1,26	1,33	1,46	1,18	1,45	1,44	1,27	1,27	arabinogalactan-protein (AGP5)
257512_at	At1g35250	0,93	1,08	1,12	1,34	0,94	1,03	0,99	1,16	thioesterase family protein
259548_at	At1g35260	0,76	0,86	0,96	0,78	1,01	0,97	1,11	0,99	Bet v I allergen family protein
259601_s_at	At1g35280	1,09	0,94	0,93	0,92	0,94	1,02	1,07	1,09	---
259549_at	At1g35290	1,08	1,12	1,13	1,14	0,89	1,17	1,12	1,16	thioesterase family protein
259574_at	At1g35310	0,95	0,87	0,95	1,41	1,37	1,02	1,00	0,98	Bet v I allergen family protein
259575_at	At1g35320	0,97	0,94	0,97	1,27	1,26	0,87	0,95	0,89	expressed protein
259576_at	At1g35330	0,99	1,03	0,96	0,92	0,99	1,02	0,98	1,15	zinc finger (C3HC4-type RING finger) family protein
259577_at	At1g35340	0,96	1,03	0,95	1,00	0,78	1,25	1,20	1,50	ATP-dependent protease La (LON) domain-containing protein
259546_at	At1g35350	0,91	0,83	1,19	0,84	1,01	1,21	1,30	1,15	EXS family protein / ERD1/XPR1/SYG1 family protein
259547_at	At1g35370	1,03	1,12	1,00	0,99	0,87	0,90	1,08	1,11	---
257501_at	At1g35380	0,94	1,06	0,93	1,00	0,92	0,98	0,92	1,09	---
260104_x_at	At1g35410	1,07	0,97	0,98	1,02	0,92	0,94	1,06	0,93	hypothetical protein
260106_at	At1g35420	0,66	0,73	0,84	0,75	0,76	0,91	0,91	1,16	dienelactone hydrolase family protein
260103_at	At1g35430	0,72	0,82	1,00	1,17	0,95	0,91	0,92	0,92	expressed protein
260079_s_at	At1g35460	0,77	0,84	0,82	0,81	0,69	1,02	1,02	1,13	basic helix-loop-helix (bHLH) family protein
260102_at	At1g35470	1,09	0,99	1,02	0,80	0,96	1,06	0,91	0,86	SPla/RYanodine receptor (SPRY) domain-containing protein
260105_at	At1g35480	1,01	0,98	0,99	0,98	1,07	1,08	1,02	1,01	---
262022_at	At1g35490	1,04	0,87	1,10	0,96	1,03	0,98	0,95	1,05	bZIP family transcription factor
262023_at	At1g35500	0,96	0,95	1,07	0,94	1,01	0,99	1,14	0,98	hypothetical protein
262025_at	At1g35510	1,03	0,86	0,91	0,81	1,03	1,09	0,90	0,88	expressed protein
262016_at	At1g35513	1,10	0,95	1,05	1,06	1,23	0,91	1,00	1,06	isochorismate synthase-related / isochirismate mutase-related
262027_at	At1g35516	1,18	0,91	0,93	1,02	1,14	1,11	1,03	1,03	myb family transcription factor (MYB8)
262036_at	At1g35530	1,00	1,05	1,06	1,09	0,88	1,00	0,95	1,11	DEAD/DEAH box helicase, putative
262017_at	At1g35550	1,30	1,31	1,11	1,00	1,24	1,10	0,93	0,98	elongation factor Tu C-terminal domain-containing protein
262021_at	At1g35555	0,98	0,94	0,91	1,03	1,05	0,93	1,02	0,96	Hypothetical protein, partial cds, clone: RAFL15-01-E19
262028_at	At1g35560	1,02	1,02	1,04	1,01	1,06	1,01	0,88	1,04	TCP family transcription factor, putative
262037_at	At1g35570	0,90	1,08	0,89	1,05	1,01	1,11	0,99	0,98	hypothetical protein
262038_at	At1g35580	0,96	1,03	1,09	0,80	0,87	1,03	1,06	1,16	beta-fructofuranosidase, putative / invertase, putative / saccharase, putative
262008_s_at	At1g35590	0,97	1,08	1,03	0,90	0,96	1,13	1,04	0,98	---
262009_at	At1g35610	1,12	1,07	1,04	1,02	1,16	0,98	1,02	1,08	DC1 domain-containing protein
262010_at	At1g35612	1,27	1,29	1,17	0,86	0,62	1,13	1,16	1,19	expressed protein
262011_at	At1g35614	1,01	0,98	1,05	0,88	0,97	1,05	0,99	1,08	hypothetical protein
262018_at	At1g35617	1,01	0,85	1,09	1,11	0,90	1,06	0,99	0,92	hypothetical protein

262024_at	At1g35620	1,07	0,96	0,84	1,20	0,99	1,06	1,14	1,06	thioredoxin family protein
262012_s_at	At1g35625	0,92	0,98	0,98	1,06	0,81	1,09	0,96	1,00	protease-associated zinc finger (C3HC4-type RING finger) family protein
262013_s_at	At1g35640	0,93	1,13	1,02	1,15	0,95	0,98	1,06	1,06	Mutator-like transposase family
262020_at	At1g35647	0,92	0,98	0,99	0,97	1,02	1,01	0,90	0,96	---
262019_s_at	At1g35650	0,92	1,02	0,91	0,97	1,03	0,97	0,88	0,85	Ulp1 protease family protein
262014_at	At1g35660	1,03	1,08	1,21	0,75	0,86	1,11	0,95	0,98	expressed protein
262015_at	At1g35663	1,01	1,02	1,04	0,95	1,13	0,97	0,99	0,94	hypothetical protein
262026_at	At1g35670	1,13	1,12	1,25	0,98	1,11	1,00	0,93	0,94	calcium-dependent protein kinase 2 (CDPK2)
262029_at	At1g35680	1,03	0,91	0,86	0,78	0,83	1,03	0,94	0,90	50S ribosomal protein L21, chloroplast / CL21 (RPL21)
261339_at	At1g35710	0,96	1,08	0,92	0,91	1,07	1,92	1,35	1,19	leucine-rich repeat transmembrane protein kinase, putative
261285_at	At1g35720	0,82	0,76	0,80	1,39	1,41	0,94	0,93	0,95	annexin 1 (ANN1)
261340_at	At1g35730	1,03	1,03	1,04	1,11	0,91	1,05	1,35	1,19	pumilio/Puf RNA-binding domain-containing protein
261281_at	At1g35740	1,08	0,99	1,08	1,09	1,08	0,97	1,05	0,97	---
261282_at	At1g35750	1,02	0,99	0,97	0,87	1,16	1,01	1,11	0,91	pumilio/Puf RNA-binding domain-containing protein
261283_s_at	At1g35770	1,01	1,10	1,01	0,93	1,03	1,12	0,97	1,04	Ulp1 protease family protein
261286_at	At1g35780	1,44	1,41	1,23	1,18	1,02	1,10	1,12	1,19	expressed protein
261284_at	At1g35790	0,95	1,04	0,92	0,92	1,05	0,97	1,00	1,02	---
256312_x_at	At1g35820	1,04	1,02	0,92	1,04	1,14	0,91	0,95	1,01	hypothetical protein
256313_s_at	At1g35850	1,00	1,04	1,07	0,96	1,05	0,96	0,98	1,03	pumilio/Puf RNA-binding domain-containing protein
256314_at	At1g35860	0,93	0,96	0,90	1,01	1,14	0,87	0,97	0,96	chloroplast outer membrane protein-related
256315_at	At1g35880	1,03	0,96	1,18	0,98	1,00	0,96	0,96	1,15	hypothetical protein
256316_at	At1g35890	0,93	1,03	0,96	0,94	1,03	1,14	1,05	0,90	hypothetical protein
256317_at	At1g35900	1,12	0,94	1,16	0,93	0,98	1,02	0,90	0,92	hypothetical protein
256319_at	At1g35910	1,91	1,55	1,18	1,27	1,11	0,95	0,99	0,97	trehalose-6-phosphate phosphatase, putative
256318_s_at	At1g35940	0,98	1,00	1,07	0,97	1,03	0,93	0,95	0,97	AT hook motif-containing protein-related
260183_at	At1g35970	1,13	0,96	1,05	1,00	1,03	0,92	1,10	1,01	---
260188_at	At1g35995	0,96	0,98	0,97	0,78	1,03	1,08	1,03	0,96	---
260187_at	At1g36000	0,94	0,98	1,00	1,05	0,99	0,98	1,05	1,05	LOB domain family protein / lateral organ boundaries domain family protein
260186_at	At1g36020	0,98	0,96	1,14	0,95	1,12	0,96	1,08	0,89	hypothetical protein
260185_at	At1g36030	1,00	0,94	0,99	0,97	1,02	0,96	0,99	0,93	F-box family protein
263193_at	At1g36050	0,95	0,82	0,77	0,95	1,05	0,95	0,81	0,83	expressed protein
263194_at	At1g36060	1,02	1,01	1,09	1,30	1,25	0,86	0,87	0,98	AP2 domain-containing transcription factor, putative
263196_at	At1g36070	1,10	1,20	1,32	0,98	0,89	0,99	1,10	1,36	WD-40 repeat family protein
263188_at	At1g36095	1,02	1,02	0,95	1,06	1,06	0,97	1,02	1,04	hypothetical protein
263189_at	At1g36100	1,00	1,00	0,90	0,92	1,00	0,94	0,91	0,95	myosin heavy chain-related
263190_at	At1g36105	1,03	0,95	0,98	0,97	1,02	1,01	0,96	1,03	---
263186_at	At1g36110	1,05	0,97	1,05	0,97	1,03	1,07	1,00	1,02	---
263191_at	At1g36120	1,00	1,00	0,98	0,95	1,19	1,00	1,05	0,97	---
263187_at	At1g36130	1,05	1,09	0,97	0,96	1,03	1,14	1,00	0,97	---
263195_at	At1g36150	1,10	1,19	1,12	0,84	1,11	0,85	1,02	0,81	protease inhibitor/seed storage/lipid transfer protein (LTP) family protein
263192_at	At1g36160	1,03	0,66	0,79	0,87	0,74	0,90	0,93	0,91	acetyl-CoA carboxylase 1 (ACC1)
256459_at	At1g36180	1,96	2,44	2,02	1,06	0,92	1,04	1,13	1,06	---
256463_s_at	At1g36190	1,06	0,86	1,12	1,07	1,08	1,01	0,99	0,95	---
256462_at	At1g36230	1,02	1,15	1,02	0,94	1,00	1,01	1,05	1,03	hypothetical protein
256460_at	At1g36240	1,07	1,11	0,96	1,06	1,00	0,88	0,97	0,85	60S ribosomal protein L30 (RPL30A)
260128_at	At1g36310	1,10	1,10	1,12	1,00	1,09	1,01	0,88	0,97	expressed protein

260127_at	At1g36320	0,71	0,71	0,70	0,90	0,96	0,88	0,88	0,96	expressed protein
260124_at	At1g36340	1,01	1,17	1,01	1,08	0,93	1,15	1,08	0,96	ubiquitin-conjugating enzyme family protein
260126_at	At1g36370	1,23	1,15	1,18	0,72	0,87	1,06	1,13	1,20	glycine hydroxymethyltransferase, putative / serine hydroxymethyltransfera
260129_at	At1g36380	1,33	1,59	1,33	1,17	1,11	1,17	1,01	1,11	expressed protein
260125_at	At1g36390	1,06	0,86	0,98	0,75	0,78	1,04	1,04	0,94	co-chaperone grpE family protein
261963_at	At1g36430	1,00	0,97	1,03	0,96	1,04	1,12	0,98	0,98	---
261967_at	At1g36440	1,05	0,96	0,90	0,92	1,07	1,07	0,95	1,38	---
261966_x_at	At1g36470	0,98	0,85	0,95	1,09	1,09	1,03	0,96	0,95	---
261962_s_at	At1g36480	1,01	0,97	0,95	1,03	1,15	1,02	1,02	1,02	---
261961_at	At1g36510	1,00	1,17	0,99	0,99	0,89	0,98	1,02	0,88	hypothetical protein
261960_at	At1g36550	0,94	0,98	0,99	1,01	1,02	1,01	0,93	1,03	hypothetical protein
261965_at	At1g36560	0,96	0,97	1,01	1,05	1,06	1,01	1,07	0,98	---
261959_s_at	At1g36590	0,84	1,02	1,04	0,94	0,99	0,99	1,05	1,15	---
261964_at	At1g36600	1,01	1,10	1,16	1,11	1,03	1,09	0,95	1,04	---
256500_at	At1g36620	0,97	1,03	1,11	0,92	1,09	0,96	0,94	1,02	---
256499_at	At1g36640	1,09	1,16	1,04	1,08	1,10	1,11	1,22	1,00	expressed protein
256498_at	At1g36670	0,97	0,91	0,88	0,96	1,06	1,06	1,02	0,99	hypothetical protein
256502_at	At1g36730	0,80	0,68	0,76	0,80	0,87	0,92	0,86	0,88	eukaryotic translation initiation factor 5, putative / eIF-5, putative
256501_at	At1g36756	1,01	1,01	1,00	0,94	1,10	1,18	1,10	1,21	hypothetical protein
256194_at	At1g36810	0,95	0,90	0,88	0,99	1,08	1,02	0,95	1,06	pseudogene, hypothetical protein
256195_at	At1g36820	1,07	1,00	1,10	0,90	0,95	1,01	1,14	0,99	---
256167_at	At1g36830	1,06	0,95	1,04	0,92	1,05	0,98	0,96	1,05	---
256196_at	At1g36900	1,09	1,13	1,09	0,87	1,13	1,03	1,05	1,15	pseudogene, disease resistance protein
256197_at	At1g36910	0,99	0,98	1,03	0,97	1,07	1,06	0,96	0,97	---
256166_at	At1g36920	1,08	1,11	1,02	1,08	1,10	1,05	0,96	0,91	hypothetical protein
261292_at	At1g36940	0,94	1,20	0,99	1,03	1,16	0,81	0,94	1,07	hypothetical protein
261291_at	At1g36970	1,05	0,92	0,94	0,99	1,02	1,04	0,96	0,87	hypothetical protein
261293_at	At1g36980	0,93	0,96	0,96	0,99	1,07	1,04	0,92	0,96	expressed protein
261290_at	At1g36990	0,85	1,04	0,94	0,91	1,09	0,93	0,92	0,91	expressed protein
261289_at	At1g37000	0,90	1,07	1,01	0,97	1,02	0,93	0,99	1,17	hypothetical protein
261288_at	At1g37010	1,02	0,98	0,96	0,99	0,95	1,05	1,03	0,94	hypothetical protein
261287_at	At1g37020	0,96	1,00	1,02	1,11	1,10	0,97	0,96	0,95	Ulp1 protease family protein
261977_at	At1g37057	0,94	1,04	0,91	0,96	1,06	0,95	1,05	1,05	---
261978_at	At1g37060	0,95	0,98	1,06	1,04	1,00	1,01	1,06	0,98	---
261976_at	At1g37063	1,08	0,99	1,05	1,08	1,06	0,95	1,11	1,01	---
262034_at	At1g37080	0,95	0,95	1,07	1,03	1,05	0,91	0,94	1,08	hypothetical protein
262035_at	At1g37110	0,92	0,94	0,91	1,04	1,09	1,01	0,97	0,84	---
261979_at	At1g37130	0,62	0,71	1,01	0,66	0,52	1,09	1,19	1,59	nitrate reductase 2 (NR2)
262033_at	At1g37140	1,01	0,85	0,96	1,08	0,82	0,93	0,91	0,75	RNA-binding protein, putative
262032_at	At1g37150	1,01	0,88	0,87	0,98	1,03	1,01	1,00	0,91	holocarboxylase synthetase 2 (HCS2.d)
262031_x_at	At1g37160	0,97	1,11	0,96	0,98	0,99	0,97	0,91	0,84	---
262030_s_at	At1g37170	0,98	1,12	1,01	1,02	0,98	0,97	0,87	1,00	---
245810_at	At1g37607	1,02	1,00	0,78	0,95	1,08	1,01	1,01	0,91	expressed protein
245811_at	At1g37826	1,01	1,01	1,04	1,02	1,03	0,91	0,94	0,95	---
245812_at	At1g37900	1,03	1,02	1,02	1,00	1,07	0,96	1,04	0,94	---
245217_at	At1g38340	0,99	1,02	1,00	1,00	1,14	1,08	0,90	0,92	---

246355_at	At1g38470	1,08	1,05	0,96	0,91	1,07	1,02	1,02	1,03	---
246354_at	At1g38950	1,02	0,98	0,93	0,92	1,23	0,96	1,02	0,88	hypothetical protein
246353_s_at	At1g39190	1,00	1,01	1,00	1,02	0,83	0,93	1,02	1,00	---
246352_at	At1g39270	1,00	0,98	1,00	0,81	1,02	0,98	1,02	0,81	hypothetical protein
246382_at	At1g39350	1,10	1,00	1,05	0,95	1,02	1,07	0,99	1,06	hypothetical protein
246381_at	At1g39430	1,03	1,07	1,18	0,89	1,01	1,01	1,02	1,02	hypothetical protein
246365_at	At1g39750	1,07	1,07	0,94	0,87	0,95	1,02	0,96	1,02	---
246356_x_at	At1g40150	1,00	1,09	0,91	1,12	1,13	0,90	1,00	1,08	---
246364_at	At1g40230	1,11	1,04	0,90	1,07	1,13	1,05	1,09	0,82	hypothetical protein
246363_at	At1g40390	0,94	1,14	1,04	1,03	1,12	1,05	1,00	1,07	hypothetical protein
246357_x_at	At1g40550	1,02	1,03	0,82	1,01	1,08	0,95	0,99	1,07	hypothetical protein
246358_x_at	At1g40710	0,93	1,03	0,89	0,86	1,13	0,98	1,01	1,05	hypothetical protein
246359_x_at	At1g40871	1,07	0,98	1,02	1,04	1,26	0,92	0,99	0,93	---
246360_at	At1g40952	0,95	1,00	0,96	0,94	0,92	0,98	0,94	1,07	---
246361_s_at	At1g41114	0,92	0,99	1,03	0,96	1,08	0,89	0,98	0,91	---
246362_at	At1g41276	0,96	0,95	1,08	1,02	1,08	1,07	1,06	1,03	---
259989_at	At1g41750	1,16	0,91	0,98	0,97	1,01	0,93	1,00	0,86	hypothetical protein
260043_at	At1g41770	0,97	1,16	0,97	0,95	1,15	0,94	1,11	1,06	hypothetical protein
260016_at	At1g41795	0,94	1,06	1,01	1,00	1,04	1,03	0,95	0,99	---
259988_at	At1g41797	1,05	0,94	1,03	0,98	1,05	0,93	0,99	0,93	gypsy-like retrotransposon family (Athila)
260017_at	At1g41810	0,95	0,92	0,89	1,05	1,09	0,98	0,95	0,91	hypothetical protein
260018_at	At1g41820	0,95	0,95	0,98	1,01	0,99	1,03	0,99	0,92	hypothetical protein
260019_at	At1g41825	1,00	0,96	1,00	1,02	1,08	1,01	1,03	0,79	---
261363_at	At1g41830	1,36	1,09	0,98	2,21	1,91	0,85	0,75	0,75	multi-copper oxidase type I family protein
261357_at	At1g41860	0,99	0,96	0,94	0,99	0,94	1,05	1,18	1,09	hypothetical protein
261360_at	At1g41870	1,00	1,01	0,97	1,05	1,00	1,12	1,00	0,88	hypothetical protein
261362_s_at	At1g41880	1,47	1,27	1,07	1,24	0,92	1,10	1,06	1,01	60S ribosomal protein L35a (RPL35aB)
261359_at	At1g41900	1,05	0,92	0,94	0,98	1,00	0,93	1,11	1,01	myosin heavy chain-related
261358_s_at	At1g41920	1,00	1,07	0,95	0,97	1,02	0,97	0,93	1,00	hypothetical protein
261361_at	At1g41930	0,98	0,99	0,88	0,96	1,13	0,90	1,00	0,89	---
245835_at	At1g42190	1,00	1,06	1,07	1,01	1,01	1,04	1,07	0,84	hypothetical protein
245834_at	At1g42200	0,98	1,06	1,07	0,93	1,10	0,98	1,19	1,19	---
245833_at	At1g42220	0,94	1,11	0,94	0,96	1,07	1,00	0,96	1,10	---
245836_at	At1g42250	1,02	0,95	0,98	1,07	1,00	1,07	0,86	1,03	replication protein-related
262271_x_at	At1g42360	1,02	0,96	1,03	1,01	1,17	1,04	1,08	0,92	---
257342_at	At1g42365	0,84	1,13	1,07	0,94	0,95	1,03	1,11	0,89	---
262270_x_at	At1g42375	1,01	1,11	0,99	0,85	0,96	0,99	0,99	1,12	---
262269_at	At1g42400	0,98	0,97	0,97	1,05	0,98	1,06	0,96	0,95	hypothetical protein
262268_at	At1g42410	0,93	0,94	1,02	0,93	1,04	1,16	0,98	0,96	---
262267_at	At1g42420	0,93	1,12	0,97	0,98	1,01	0,94	1,09	0,90	---
262272_at	At1g42440	1,03	1,07	1,11	0,86	0,74	1,07	1,07	1,02	expressed protein
262266_at	At1g42450	0,99	1,06	1,06	0,86	0,99	0,97	0,95	0,95	---
262265_at	At1g42460	0,96	0,95	0,94	1,02	1,02	1,11	1,00	1,01	Ulp1 protease family protein
262264_at	At1g42470	0,76	0,74	0,88	0,77	0,77	0,99	1,17	1,32	patched family protein
256543_at	At1g42480	0,74	0,91	0,71	1,09	0,95	0,97	1,04	1,00	expressed protein
256541_at	At1g42540	0,68	0,78	0,88	0,86	0,96	1,14	1,22	1,15	glutamate receptor family protein (GLR3.3)

256542_at	At1g42550	1,29	1,57	1,91	0,89	0,99	1,28	1,45	1,53 expressed protein
256544_at	At1g42560	1,09	1,08	0,98	1,14	1,16	1,22	1,35	1,47 seven transmembrane MLO family protein / MLO-like protein 9 (MLO9)
256470_at	At1g42570	0,98	1,02	1,02	0,89	1,02	0,98	1,04	1,15 ---
256471_at	At1g42580	0,96	1,02	1,04	1,03	1,01	1,09	0,95	0,91 hypothetical protein
256472_at	At1g42610	0,95	1,05	1,02	0,96	1,02	0,91	0,99	1,04 pseudogene, hypothetical protein
256473_at	At1g42630	0,92	1,02	0,98	0,96	1,06	1,01	1,04	1,01 zinc knuckle (CCHC-type) family protein
256474_at	At1g42640	0,97	0,94	1,02	0,95	0,99	0,96	0,98	0,94 ---
256478_at	At1g42650	1,05	1,01	1,00	0,97	1,26	1,10	1,03	0,84 ---
256475_s_at	At1g42680	1,07	1,03	1,09	0,90	1,00	0,96	1,06	1,01 myosin-related
256477_at	At1g42690	1,07	1,07	0,92	1,14	1,00	0,93	1,10	1,14 pseudogene, similar to B
256476_at	At1g42697	1,08	0,88	1,07	0,97	1,07	1,02	0,98	1,09 ---
259623_at	At1g42700	1,01	1,02	1,04	1,04	1,08	1,11	0,97	0,90 hypothetical protein
259620_s_at	At1g42705	1,39	1,37	1,06	0,99	1,11	1,08	1,04	1,22 ---
259621_at	At1g42710	1,00	0,96	0,88	1,01	0,99	0,97	1,07	0,98 hypothetical protein
259627_at	At1g42960	1,05	0,85	0,88	1,06	1,06	0,93	0,91	0,90 expressed protein
259625_at	At1g42970	1,02	1,01	1,03	0,80	0,82	1,23	1,17	1,13 glyceraldehyde-3-phosphate dehydrogenase B, chloroplast (GAPB) / NADF
259619_at	At1g42980	1,05	1,00	0,80	0,94	0,91	1,06	1,25	0,84 formin homology 2 domain-containing protein / FH2 domain-containing prot
259626_at	At1g42990	0,80	1,10	1,52	0,81	0,82	1,10	1,37	1,71 bZIP transcription factor family protein
257511_at	At1g43000	0,88	1,10	0,84	0,89	1,03	1,00	1,01	1,02 zinc-binding family protein
259622_at	At1g43010	1,05	0,94	0,99	1,18	1,02	1,05	1,01	1,07 pentatricopeptide (PPR) repeat-containing protein
259624_at	At1g43020	1,12	1,06	1,10	1,13	1,00	0,99	1,03	0,89 expressed protein
255754_at	At1g43040	1,12	1,04	0,97	1,12	0,98	0,98	1,17	0,91 auxin-responsive protein, putative
255755_s_at	At1g43080	1,01	1,07	1,05	1,04	1,11	1,04	0,94	0,92 pseudogene, putative polygalacturonase
264422_at	At1g43130	0,74	0,73	0,85	1,04	1,01	0,94	0,81	0,93 expressed protein
264416_at	At1g43140	1,07	1,14	0,94	1,05	1,03	0,98	1,07	0,95 ---
264415_at	At1g43160	1,04	1,83	1,19	3,67	2,19	1,17	2,62	2,07 AP2 domain-containing protein RAP2.6 (RAP2.6)
264421_at	At1g43170	1,33	1,24	1,23	1,18	1,02	1,06	1,04	0,98 60S ribosomal protein L3 (RPL3A)
264418_at	At1g43190	1,10	1,03	1,11	0,96	1,04	1,08	0,99	1,07 polypyrimidine tract-binding protein, putative / heterogeneous nuclear ribonu
264409_s_at	At1g43200	0,96	1,16	0,97	1,00	0,97	1,13	1,07	0,92 hypothetical protein
264417_at	At1g43220	1,00	0,88	1,10	1,01	1,00	0,97	1,04	1,01 ---
264410_s_at	At1g43230	0,97	1,04	0,96	1,08	1,09	0,95	0,91	0,98 hypothetical protein
264411_at	At1g43240	1,02	0,91	0,88	0,92	0,94	1,05	0,94	0,94 ---
264412_at	At1g43260	1,03	1,04	1,06	1,00	1,05	1,03	0,96	1,09 hypothetical protein
264413_s_at	At1g43290	0,92	1,04	1,02	0,96	1,05	0,99	1,03	0,86 pseudogene, hypothetical protein
264419_s_at	At1g43310	1,11	0,97	0,88	1,32	0,95	0,97	0,94	0,94 triose phosphate/phosphate translocator-related
264420_at	At1g43320	1,02	1,00	1,01	1,05	1,03	1,11	0,94	1,19 hypothetical protein
262724_s_at	At1g43330	0,94	0,93	1,10	1,07	1,08	1,02	1,17	0,95 myb family protein-related
262721_at	At1g43560	1,19	1,14	1,05	0,90	0,83	1,18	1,15	1,03 thioredoxin family protein
262718_at	At1g43570	1,05	1,15	0,95	1,04	1,11	0,96	1,02	0,81 hypothetical protein
262725_at	At1g43580	0,76	0,82	0,75	1,08	1,12	0,76	0,81	0,70 expressed protein
262719_at	At1g43590	1,22	1,04	1,11	1,12	1,27	1,04	1,07	0,98 hypothetical protein
262720_s_at	At1g43610	0,97	0,99	1,02	0,99	0,90	1,06	0,94	0,91 NLI interacting factor (NIF) family protein
262722_at	At1g43620	0,78	0,96	0,92	0,83	0,71	1,21	1,06	1,10 UDP-glucose:sterol glucosyltransferase, putative
262723_at	At1g43630	0,88	1,09	0,91	0,95	0,96	0,99	0,99	1,06 expressed protein
262726_at	At1g43640	0,89	1,27	1,04	0,94	1,25	0,95	0,89	0,94 F-box family protein / tubby family protein
260812_at	At1g43650	0,90	0,80	0,81	1,21	1,04	0,89	1,04	1,24 integral membrane family protein / nodulin MtN21-related

260836_at	At1g43660	1,01	0,98	1,01	0,96	0,94	0,97	1,00	1,06	hypothetical protein
260837_at	At1g43670	1,10	1,09	1,17	0,61	0,75	1,35	1,32	1,34	fructose-1,6-bisphosphatase, putative / D-fructose-1,6-bisphosphate 1-phos
260838_at	At1g43680	0,93	1,10	0,91	1,11	0,98	0,91	1,00	1,17	hypothetical protein
260839_at	At1g43690	1,28	1,26	1,16	1,13	1,10	1,08	0,94	0,87	ubiquitin interaction motif-containing protein
260813_at	At1g43700	1,09	1,22	1,36	0,87	0,91	1,02	1,06	1,09	VirE2-interacting protein (VIP1)
260814_at	At1g43710	0,65	0,73	1,06	1,00	1,01	0,88	0,88	1,01	serine decarboxylase
260808_at	At1g43715	1,09	1,01	0,98	1,01	1,07	0,86	0,98	1,16	---
260809_at	At1g43730	0,97	1,02	1,08	0,76	1,15	0,96	0,97	0,99	hypothetical protein
260811_s_at	At1g43740	0,96	0,96	0,98	0,99	1,03	0,96	1,03	0,94	---
260810_s_at	At1g43745	1,04	0,94	0,98	1,07	0,98	1,05	1,00	0,87	---
260865_at	At1g43760	1,01	0,97	0,93	1,18	0,97	0,95	0,95	1,02	hypothetical protein
260858_at	At1g43770	1,17	1,04	0,89	0,87	1,03	1,04	1,12	1,16	PHD finger family protein
260859_at	At1g43780	0,99	0,82	0,71	1,11	1,00	1,07	1,06	1,06	serine carboxypeptidase S10 family protein
260867_at	At1g43790	1,67	1,60	1,37	1,31	1,35	0,91	0,91	0,88	expressed protein
260869_at	At1g43800	0,48	1,27	1,10	1,11	1,20	0,84	0,54	0,42	acyl-(acyl-carrier-protein) desaturase, putative / stearoyl-ACP desaturase, p
260860_at	At1g43810	1,01	1,03	1,06	0,99	0,83	1,03	1,08	0,84	hypothetical protein
260861_at	At1g43840	1,07	0,93	0,95	0,99	1,08	1,01	1,12	0,88	---
260862_at	At1g43850	0,93	0,95	1,29	0,82	1,03	1,01	0,97	1,39	SEUSS transcriptional co-regulator
260868_at	At1g43860	1,17	0,91	1,04	0,85	0,90	1,20	0,97	1,04	expressed protein
260863_at	At1g43870	0,97	1,03	0,96	1,01	0,98	1,03	1,03	0,96	hypothetical protein
260864_at	At1g43880	1,02	0,94	1,00	0,95	1,03	0,91	1,02	0,99	hypothetical protein
260866_at	At1g43886	0,93	0,87	1,04	0,90	1,30	0,88	0,91	0,82	---
260870_at	At1g43890	0,96	0,95	1,00	1,26	1,22	0,98	0,85	0,78	Ras-related GTP-binding protein, putative
259506_at	At1g43900	0,86	0,72	0,79	1,01	0,96	0,93	0,93	0,89	protein phosphatase 2C, putative / PP2C, putative
259507_at	At1g43910	1,93	2,00	1,98	3,96	3,39	1,27	1,39	1,15	AAA-type ATPase family protein
259508_at	At1g43920	0,90	1,00	1,00	0,96	1,07	1,00	0,92	0,78	hypothetical protein
259509_at	At1g43930	0,93	1,03	0,90	0,91	1,31	0,95	0,85	1,10	---
257514_at	At1g43940	0,97	1,09	1,05	1,08	1,07	0,96	0,97	1,05	hypothetical protein
259457_at	At1g43950	0,93	0,93	0,90	1,07	0,93	0,99	0,97	1,05	auxin-responsive factor, putative
259510_at	At1g43970	0,96	0,90	1,00	0,98	1,09	1,02	1,02	1,00	hypothetical protein
259483_at	At1g43980	0,95	0,98	1,05	0,90	1,01	1,06	0,97	0,95	pentatricopeptide (PPR) repeat-containing protein
259456_s_at	At1g43995	0,94	1,03	1,03	0,92	1,11	0,99	0,97	1,05	copia-like retrotransposon family
259460_at	At1g44000	1,19	1,27	1,34	1,00	0,98	1,26	1,27	1,34	expressed protein
259455_x_at	At1g44030	0,92	0,94	0,89	1,04	0,93	1,07	1,01	1,23	DC1 domain-containing protein
259454_at	At1g44050	0,99	0,96	0,92	1,18	1,20	0,88	1,10	0,91	DC1 domain-containing protein
259452_at	At1g44060	1,04	0,99	0,98	0,99	0,99	0,98	1,01	1,10	---
259459_at	At1g44070	0,98	1,02	0,97	0,94	1,05	0,99	1,02	0,95	---
259458_at	At1g44085	0,95	1,13	0,94	0,97	1,14	1,09	1,03	1,09	glycine-rich protein
259453_at	At1g44090	1,17	1,05	1,15	1,06	1,01	0,98	0,99	0,82	gibberellin 20-oxidase family protein
245740_at	At1g44100	0,75	0,78	0,84	1,22	0,82	0,97	1,11	1,02	amino acid permease 5, putative (AAP5)
245739_at	At1g44110	1,22	1,43	0,98	1,43	1,09	0,99	0,97	0,86	cyclin, putative
245741_at	At1g44120	0,97	0,95	0,87	0,96	1,11	1,04	1,07	0,92	C2 domain-containing protein / armadillo/beta-catenin repeat family protein
245738_at	At1g44130	1,06	1,28	1,16	1,22	1,08	1,33	0,99	0,93	nucellin protein, putative
245737_at	At1g44160	1,13	1,19	1,05	1,17	0,94	1,03	1,09	1,03	DNAJ chaperone C-terminal domain-containing protein
245742_at	At1g44170	0,99	0,90	0,95	1,17	0,97	1,05	0,88	0,86	aldehyde dehydrogenase, putative (ALDH)
261326_s_at	At1g44180	1,09	1,09	1,08	0,97	0,99	1,16	1,19	1,21	aminoacylase, putative / N-acyl-L-amino-acid amidohydrolase, putative

245246_at	At1g44222	0,92	1,10	0,88	0,85	1,19	1,02	0,94	1,00	protein coding
245212_at	At1g44254	0,94	0,94	0,90	0,94	0,88	0,96	1,10	1,18	hypothetical protein
245245_at	At1g44318	0,92	1,00	1,04	0,89	0,88	0,96	1,02	1,21	porphobilinogen synthase, putative / delta-aminolevulinic acid dehydratase,
245244_at	At1g44350	0,83	0,80	1,32	1,29	1,24	1,08	1,09	1,19	IAA-amino acid hydrolase 6, putative (ILL6) / IAA-Ala hydrolase, putative
245243_at	At1g44414	0,82	0,94	1,01	1,05	1,17	0,99	0,96	1,14	hypothetical protein
245242_at	At1g44446	1,51	1,70	1,81	0,87	1,10	1,14	1,19	1,28	chlorophyll a oxygenase (CAO) / chlorophyll b synthase
245241_at	At1g44478	0,98	0,93	0,95	0,92	1,23	0,99	1,03	1,06	hypothetical protein
245240_at	At1g44510	1,05	1,08	1,13	0,92	1,05	0,98	0,96	1,12	---
245213_at	At1g44575	1,03	0,98	0,90	0,96	0,82	1,19	1,08	1,14	photosystem II 22kDa protein, chloroplast / CP22 (PSBS)
261321_at	At1g44740	0,88	1,08	1,01	0,96	1,16	0,92	1,06	0,86	expressed protein
261322_at	At1g44750	1,06	1,09	0,92	0,99	0,96	0,94	0,95	0,92	purine permease family protein
261323_at	At1g44760	0,92	1,01	1,17	1,05	1,20	0,82	1,00	0,94	universal stress protein (USP) family protein
261324_at	At1g44770	1,06	1,14	1,26	0,88	0,99	1,18	1,23	1,05	expressed protein
261325_at	At1g44780	1,03	1,00	1,05	0,99	1,03	0,93	1,10	1,15	expressed protein
261336_at	At1g44790	0,69	0,57	0,52	1,18	1,15	1,03	0,95	0,99	ChaC-like family protein
261335_at	At1g44800	1,14	1,16	1,02	1,62	1,41	0,98	1,00	0,92	nodulin MtN21 family protein
261337_at	At1g44810	0,91	0,90	0,86	1,05	0,98	0,85	0,86	0,89	expressed protein
261327_at	At1g44830	1,48	1,20	1,11	1,04	1,22	0,82	0,72	0,87	AP2 domain-containing transcription factor TINY, putative
261328_at	At1g44835	1,05	0,83	0,96	1,02	1,23	0,99	0,77	0,83	YbaK/prolyl-tRNA synthetase family protein
261332_s_at	At1g44840	0,98	1,02	0,95	0,96	1,13	1,02	0,98	0,94	---
261329_at	At1g44890	0,88	1,02	0,85	0,85	0,81	0,91	1,00	0,85	expressed protein
261330_at	At1g44900	1,40	1,35	1,23	1,26	1,18	1,15	1,13	1,12	DNA replication licensing factor, putative
261333_at	At1g44910	1,15	0,92	1,33	0,80	0,84	1,03	1,54	1,07	FF domain-containing protein / WW domain-containing protein
261338_at	At1g44920	0,94	1,03	1,09	0,73	0,75	0,91	0,86	0,86	expressed protein
261331_at	At1g44930	0,91	0,97	1,04	1,02	0,98	1,05	1,14	1,13	---
261334_at	At1g44960	0,95	0,92	0,93	1,12	0,96	1,03	0,96	0,93	expressed protein
260941_at	At1g44970	1,56	1,49	1,37	1,37	1,10	1,03	1,07	0,80	peroxidase, putative
260962_at	At1g44980	0,94	1,04	0,97	1,01	1,13	0,99	1,06	0,89	pectinesterase family protein
260963_at	At1g44990	0,88	1,03	1,02	0,94	0,99	0,95	1,16	1,17	hypothetical protein
260940_at	At1g45000	1,15	1,03	0,96	1,16	1,11	1,04	0,95	0,97	26S proteasome regulatory complex subunit p42D, putative
260964_at	At1g45050	0,81	0,68	0,64	1,03	1,19	0,87	0,85	0,77	ubiquitin-conjugating enzyme 15 (UBC15)
257344_s_at	At1g45070	1,00	0,98	1,11	1,08	1,04	0,92	0,95	0,87	Mutator-like transposase family
260965_s_at	At1g45090	0,95	1,05	0,91	1,04	1,10	1,03	0,99	0,91	Ulp1 protease family protein
260934_at	At1g45100	0,93	1,01	1,17	1,09	1,01	1,06	1,02	1,11	polyadenylate-binding protein, putative / PABP, putative
260935_at	At1g45110	1,33	1,31	1,17	1,06	1,05	0,97	0,99	1,14	tetrapyrrole methylase family protein
260944_at	At1g45130	1,15	1,31	1,00	1,39	0,97	1,02	1,02	1,03	beta-galactosidase, putative / lactase, putative
260943_at	At1g45145	1,39	1,60	1,32	1,40	1,37	1,20	1,35	1,25	thioredoxin H-type 5 (TRX-H-5) (TOUL)
260936_at	At1g45150	0,95	0,98	0,83	0,81	0,91	1,06	1,01	1,07	expressed protein
260937_at	At1g45160	0,83	0,93	1,04	0,89	0,95	1,10	1,01	1,01	protein kinase family protein
260938_at	At1g45170	1,34	1,16	1,06	0,96	0,76	1,16	1,10	1,00	expressed protein
260939_at	At1g45180	0,95	0,76	1,14	1,20	1,03	1,01	1,20	1,21	zinc finger (C3HC4-type RING finger) family protein
260942_s_at	At1g45190	0,96	1,08	0,99	0,99	0,95	1,09	1,02	1,00	expressed protein
245797_at	At1g45261	1,04	1,07	1,39	0,75	0,68	1,31	1,12	1,11	defective chloroplasts and leaves protein-related / DCL protein-related
245806_at	At1g45474	1,01	0,94	0,91	0,90	0,91	1,17	1,07	1,09	chlorophyll A-B binding protein, putative (LHCA5)
245798_at	At1g45545	0,98	0,78	1,07	1,00	1,05	0,96	1,00	1,12	hypothetical protein
245799_at	At1g45616	0,94	0,89	0,82	0,83	0,92	0,93	1,03	0,92	leucine-rich repeat family protein

245780_at	At1g45688	1,15	1,00	1,02	1,33	1,30	0,97	1,02	1,02	expressed protein
245781_at	At1g45976	1,02	1,06	1,41	1,05	1,35	1,04	1,09	1,24	expressed protein
245800_at	At1g46264	1,12	1,30	1,02	1,37	1,64	1,05	0,86	1,11	heat shock transcription factor family protein
245805_at	At1g46336	0,98	0,97	0,98	0,99	0,98	1,04	1,01	1,04	hypothetical protein
245804_at	At1g46696	0,98	0,92	1,08	0,97	1,09	0,90	0,98	0,85	hypothetical protein
245807_at	At1g46768	0,96	1,03	1,05	0,94	1,11	1,02	0,83	0,98	AP2 domain-containing protein RAP2.1 (RAP2.1)
245802_at	At1g46840	0,95	1,08	0,89	1,08	1,07	1,00	0,96	1,10	F-box family protein
245801_at	At1g46912	1,05	1,01	0,99	0,92	1,04	1,03	1,01	1,00	F-box family protein-related
245803_at	At1g47128	0,95	1,04	1,14	0,85	0,76	1,24	1,34	1,43	cysteine proteinase (RD21A) / thiol protease
260507_at	At1g47200	1,03	0,87	0,88	1,28	1,65	0,89	0,77	0,72	MFP1 attachment factor, putative
260506_at	At1g47210	1,48	1,66	1,59	1,18	1,33	0,96	0,85	0,81	cyclin family protein
260504_at	At1g47220	1,03	1,00	0,95	1,08	0,87	1,13	1,02	1,11	cyclin, putative
260505_at	At1g47230	0,99	1,10	1,03	0,94	0,90	0,89	0,95	1,24	cyclin, putative
260509_at	At1g47240	0,95	0,81	1,00	0,95	1,07	0,97	0,92	0,85	NRAMP metal ion transporter 2, putative (NRAMP2)
260503_at	At1g47250	1,09	0,97	0,98	0,95	1,11	0,97	0,92	0,91	20S proteasome alpha subunit F2 (PAF2) (PRC2B) (PRS1)
260508_at	At1g47260	1,38	1,18	1,14	1,08	1,19	1,01	1,00	0,91	bacterial transferase hexapeptide repeat-containing protein
260502_at	At1g47270	0,87	1,01	1,02	0,84	1,13	0,97	0,85	1,05	F-box family protein / tubby family protein
261687_at	At1g47280	1,07	1,00	1,05	0,98	0,97	1,10	0,95	0,98	expressed protein
261685_at	At1g47290	0,75	0,86	0,73	1,05	0,76	0,87	0,86	0,81	3-beta hydroxysteroid dehydrogenase/isomerase family protein
261683_at	At1g47300	1,03	1,00	1,02	1,04	0,98	0,95	1,00	0,98	F-box family protein
261686_at	At1g47310	0,95	0,94	1,03	1,01	1,04	0,97	0,97	0,97	expressed protein
261682_at	At1g47330	0,88	1,02	1,00	0,98	1,23	0,94	0,92	1,15	expressed protein
261681_at	At1g47340	0,84	0,90	0,97	0,90	0,78	0,96	0,81	0,99	F-box family protein
261680_at	At1g47350	0,98	0,90	0,99	0,91	0,92	0,93	1,03	1,03	F-box family protein-related
261679_at	At1g47360	0,95	1,02	1,04	1,02	1,01	1,00	1,15	0,92	---
261678_at	At1g47370	0,94	0,95	0,89	1,11	1,08	0,96	0,98	0,99	Toll-Interleukin-Resistance (TIR) domain-containing protein
261688_at	At1g47380	0,78	0,65	0,91	1,00	1,23	0,92	0,99	1,16	protein phosphatase 2C-related / PP2C-related
261684_at	At1g47400	1,09	1,72	1,45	1,38	1,20	4,51	4,10	4,04	expressed protein
262438_at	At1g47410	1,20	1,36	1,27	1,64	1,20	1,10	1,12	1,07	expressed protein
262442_at	At1g47420	1,13	1,09	1,02	1,31	1,16	1,03	0,93	0,95	expressed protein
262437_at	At1g47465	0,97	0,95	1,07	0,92	1,07	1,08	0,90	0,98	---
257468_at	At1g47470	0,96	1,07	1,01	0,93	1,09	0,96	1,02	1,05	hypothetical protein
262444_at	At1g47480	0,91	0,85	0,88	1,31	1,25	0,89	1,05	0,85	expressed protein
262445_at	At1g47485	1,12	1,06	0,97	0,98	1,07	0,95	0,93	0,89	expressed protein
262433_s_at	At1g47500	1,11	1,02	1,20	0,92	0,82	1,07	1,07	1,08	RNA-binding protein 47 (RBP47), putative
262429_at	At1g47520	0,88	0,90	0,99	1,04	0,95	1,12	0,99	0,98	---
262432_at	At1g47530	0,90	1,04	0,84	0,76	0,69	1,20	1,26	1,15	ripening-responsive protein, putative
262431_at	At1g47540	0,88	0,94	0,31	1,11	1,07	0,82	1,03	0,98	trypsin inhibitor, putative
262430_s_at	At1g47550	1,02	0,93	0,92	0,97	1,04	0,99	1,01	0,92	expressed protein
262428_at	At1g47570	1,25	1,38	1,61	1,00	0,90	1,18	1,31	1,54	zinc finger (C3HC4-type RING finger) family protein
262427_s_at	At1g47600	1,61	1,43	1,06	3,42	2,86	1,24	1,52	1,46	glycosyl hydrolase family 1 protein
262436_at	At1g47610	0,95	1,03	1,04	0,99	1,16	1,04	1,18	1,10	transducin family protein / WD-40 repeat family protein
262435_at	At1g47620	0,96	0,93	0,98	1,04	0,98	1,06	1,00	1,15	cytochrome P450, putative
262426_s_at	At1g47630	1,04	1,00	0,94	1,21	1,20	0,93	0,95	0,84	hypothetical protein
262443_at	At1g47655	0,97	0,97	1,05	1,09	1,23	0,96	0,94	0,94	Dof-type zinc finger domain-containing protein
262425_at	At1g47660	0,96	1,01	0,98	0,91	1,01	1,04	0,91	0,95	hypothetical protein

262434_at	At1g47670	1,12	1,13	1,00	1,13	0,80	0,88	0,91	0,77	amino acid transporter family protein
262424_at	At1g47680	0,99	1,00	1,00	0,98	1,01	1,00	1,04	0,94	hypothetical protein
262440_at	At1g47710	0,88	0,88	0,99	1,10	1,33	0,89	0,88	0,85	serpin, putative / serine protease inhibitor, putative
262441_at	At1g47720	0,90	0,96	1,09	0,98	0,96	0,89	0,91	0,89	expressed protein
262439_at	At1g47730	0,95	1,01	1,09	0,98	1,05	1,16	1,03	0,91	F-box family protein
261740_at	At1g47740	0,94	0,96	0,93	1,20	0,92	0,96	0,88	0,95	expressed protein
261739_at	At1g47750	0,83	0,80	0,90	1,13	1,58	0,93	0,98	0,72	peroxisomal biogenesis factor 11 family protein / PEX11 family protein
261735_at	At1g47765	0,93	1,04	0,90	0,90	1,10	0,97	1,14	1,11	F-box family protein
261732_at	At1g47770	0,94	1,05	0,97	0,85	1,07	1,09	1,07	1,21	hypothetical protein
261731_s_at	At1g47780	1,00	0,92	1,01	0,89	1,06	0,95	0,95	0,94	acyl-protein thioesterase-related
261734_at	At1g47790	0,97	0,98	1,00	1,03	0,91	0,98	1,02	1,04	F-box family protein
261736_at	At1g47810	1,05	0,94	0,99	0,95	1,00	0,88	0,92	0,98	F-box family protein
261730_s_at	At1g47816	1,00	1,01	1,00	0,95	1,04	1,01	1,04	1,00	---
261738_s_at	At1g47820	0,96	0,96	0,92	1,13	1,24	1,21	0,97	0,89	hypothetical protein
261733_at	At1g47830	1,12	0,87	0,84	1,19	1,27	0,91	0,92	0,68	clathrin coat assembly protein, putative
261729_s_at	At1g47840	1,00	0,79	0,62	1,10	0,89	0,91	1,03	0,93	hexokinase, putative
261741_at	At1g47870	1,02	0,89	0,98	0,97	1,29	0,83	0,90	0,77	E2F transcription factor-2 (E2F2) / transcription factor E2Fc (E2Fc)
261737_at	At1g47885	1,06	1,06	1,09	1,17	1,05	0,98	0,94	1,03	leucine-rich repeat family protein
259805_at	At1g47890	0,93	0,91	0,94	0,88	0,90	0,90	1,01	0,86	disease resistance family protein
259806_at	At1g47900	1,08	1,00	0,95	1,12	1,17	1,07	1,03	0,99	expressed protein
259807_at	At1g47920	1,15	1,10	1,12	0,95	0,92	1,04	0,99	0,84	syntaxin-related family protein
257505_at	At1g47940	0,90	1,18	0,86	0,83	0,91	0,93	1,03	1,30	hypothetical protein
259616_at	At1g47960	0,79	0,75	0,70	1,04	0,91	0,75	0,79	0,69	invertase/pectin methylesterase inhibitor family protein
259617_at	At1g47970	1,00	0,85	0,95	0,88	0,88	0,94	0,91	1,12	expressed protein
259615_at	At1g47980	0,39	0,51	0,62	0,77	0,72	0,99	0,95	1,09	expressed protein
259614_at	At1g47990	1,07	0,99	1,03	1,11	0,98	1,14	0,99	1,01	gibberellin 2-oxidase, putative / GA2-oxidase, putative
259618_at	At1g48000	1,17	1,17	1,34	1,33	1,31	1,21	1,25	1,29	myb family transcription factor
259613_at	At1g48010	0,99	0,89	1,03	1,01	0,99	1,08	0,98	0,92	invertase/pectin methylesterase inhibitor family protein
260730_at	At1g48030	0,62	0,61	0,64	0,84	0,94	0,95	0,88	0,84	dihydrolipoamide dehydrogenase 1, mitochondrial / lipoamide dehydrogena
260722_at	At1g48040	0,88	0,72	1,01	1,07	0,76	0,99	0,85	1,01	protein phosphatase 2C-related / PP2C-related
260729_at	At1g48050	1,01	0,97	1,09	0,78	0,99	1,20	1,11	1,14	Ku80 family protein
260721_at	At1g48060	0,96	1,00	0,98	0,99	1,08	0,96	0,94	1,00	F-box family protein
260723_at	At1g48070	0,99	1,06	0,90	1,01	0,84	0,94	1,00	1,06	expressed protein
260719_at	At1g48090	0,73	0,75	0,60	0,78	0,79	1,12	1,16	1,10	C2 domain-containing protein
260720_at	At1g48095	0,98	0,94	1,09	1,03	0,92	0,98	0,90	0,83	hypothetical protein
260727_at	At1g48100	0,95	0,96	0,91	0,96	1,13	0,95	0,90	1,00	glycoside hydrolase family 28 protein / polygalacturonase (pectinase) famil[illegible]
260718_at	At1g48110	1,11	1,14	1,31	1,37	1,63	1,05	0,92	0,90	expressed protein
260717_at	At1g48120	0,84	1,08	0,92	0,87	0,85	1,16	0,94	1,19	calcineurin-like phosphoesterase family protein
260716_at	At1g48130	0,04	0,04	0,05	0,52	0,46	0,99	0,97	1,13	peroxiredoxin (PER1) / rehydrin, putative
260724_at	At1g48140	1,10	1,01	0,86	1,09	1,04	1,01	0,95	0,95	dolichol-phosphate mannosyltransferase-related
260726_at	At1g48160	1,08	0,89	1,00	1,20	1,28	0,99	0,85	0,84	signal recognition particle 19 kDa protein, putative / SRP19, putative
260725_at	At1g48170	1,15	0,94	1,05	1,20	1,22	0,96	0,88	0,94	expressed protein
257493_at	At1g48180	1,07	0,90	0,94	1,02	1,03	0,96	1,00	0,96	expressed protein
260715_at	At1g48200	0,79	0,88	0,89	1,14	0,95	0,87	0,91	0,74	expressed protein
260728_at	At1g48210	0,87	0,82	0,70	1,06	1,02	0,81	0,82	0,86	serine/threonine protein kinase, putative
262240_at	At1g48220	0,94	1,00	1,05	1,00	1,15	0,97	0,90	0,90	serine/threonine protein kinase, putative

262239_at	At1g48230	0,94	0,87	0,86	0,93	0,91	0,95	0,90	0,95	phosphate translocator-related
262245_at	At1g48240	1,05	0,90	0,92	0,91	1,11	0,88	0,94	0,93	novel plant SNARE 12 (NPSN12)
262244_at	At1g48260	1,23	1,51	1,30	0,90	1,29	1,06	1,14	0,85	CBL-interacting protein kinase 17 (CIPK17)
262234_at	At1g48270	0,91	1,04	0,98	1,12	1,08	0,88	0,90	1,28	G protein coupled receptor-related
262250_at	At1g48280	0,93	0,98	1,08	0,82	0,99	0,91	0,87	1,05	hydroxyproline-rich glycoprotein family protein
262243_at	At1g48290	0,97	1,02	1,04	0,95	1,08	0,90	0,93	0,91	expressed protein
262238_at	At1g48300	1,12	1,44	1,47	0,93	0,82	1,48	1,61	2,29	expressed protein
262233_at	At1g48310	0,84	0,93	1,09	0,86	0,91	0,88	0,86	0,77	SNF2 domain-containing protein / helicase domain-containing protein
262237_at	At1g48320	0,91	1,20	1,03	1,13	1,38	1,10	1,19	0,77	thioesterase family protein
262236_at	At1g48330	0,95	0,97	1,00	0,93	1,06	1,18	1,16	1,04	expressed protein
262235_at	At1g48350	0,93	0,87	0,83	0,85	0,91	1,04	0,97	1,01	ribosomal protein L18 family protein
262242_at	At1g48360	0,83	0,88	0,97	0,73	1,03	0,84	0,95	1,01	expressed protein
262248_at	At1g48370	0,92	1,15	1,03	0,85	1,16	0,99	1,10	1,20	oligopeptide transporter OPT family protein
262249_at	At1g48380	1,12	1,12	1,33	0,91	1,02	0,98	0,95	1,05	root hair initiation protein root hairless 1 (RHL1)
262241_at	At1g48390	1,02	0,97	1,09	0,94	1,03	0,95	1,09	0,96	syntaxin-related family protein
262246_at	At1g48410	0,86	0,89	0,99	1,14	1,11	0,90	0,90	0,85	argonaute protein (AGO1)
262247_at	At1g48420	0,89	0,88	0,87	0,97	0,97	0,95	0,97	0,99	desulfhydrase family
261294_at	At1g48430	0,52	0,45	0,38	0,81	0,85	0,72	0,76	0,66	dihydroxyacetone kinase family protein
261304_at	At1g48440	0,96	0,88	0,81	1,13	1,00	0,93	0,96	0,92	expressed protein
261295_at	At1g48450	1,02	0,99	1,03	0,77	0,76	0,97	1,04	1,04	expressed protein
261296_at	At1g48460	0,97	0,93	0,80	0,60	0,46	0,87	0,86	0,74	expressed protein
261305_at	At1g48470	0,80	0,80	0,58	0,82	0,92	1,03	0,98	0,93	glutamine synthetase, putative
261308_at	At1g48480	1,12	1,07	0,95	1,23	1,19	0,78	0,72	0,80	leucine-rich repeat transmembrane protein kinase, putative
261297_at	At1g48500	0,97	1,10	1,01	0,94	1,21	1,08	1,06	1,10	expressed protein
261298_at	At1g48510	1,01	0,97	0,90	1,09	1,31	0,99	1,13	1,38	cytochrome c oxidase assembly protein surfeit-related
261307_at	At1g48520	0,98	0,81	0,82	0,84	0,98	1,04	1,00	0,97	glutamyl-tRNA(Gln) amidotransferase B family protein
261299_at	At1g48550	1,15	1,16	1,20	0,91	1,11	1,07	0,92	1,03	vacuolar protein sorting-associated protein 26 family protein / VPS26 family
261300_at	At1g48560	0,95	0,85	1,00	0,94	1,13	1,08	0,94	0,99	expressed protein
261301_at	At1g48570	1,21	1,15	1,13	0,57	0,63	0,79	0,82	0,83	zinc finger (Ran-binding) family protein
261302_at	At1g48580	1,30	1,14	1,10	1,14	0,99	0,97	1,06	1,06	expressed protein
261309_at	At1g48600	0,93	0,87	0,78	1,39	1,50	0,85	0,93	0,89	phosphoethanolamine N-methyltransferase 2, putative (NMT2)
261306_at	At1g48610	1,09	0,84	0,78	0,99	1,01	0,99	0,92	0,97	AT hook motif-containing protein
261303_at	At1g48620	0,78	0,97	0,98	0,83	0,85	0,87	0,93	0,98	histone H1/H5 family protein
256144_at	At1g48630	1,27	1,13	1,05	1,13	0,81	1,03	1,02	0,90	guanine nucleotide-binding family protein / activated protein kinase C recep
256141_at	At1g48640	0,97	0,94	0,80	0,85	1,08	0,97	0,92	0,95	lysine and histidine specific transporter, putative
256140_at	At1g48650	1,33	1,23	1,54	0,83	0,70	1,30	1,33	1,51	helicase domain-containing protein
256139_at	At1g48660	1,02	1,03	1,04	0,94	0,93	1,03	1,05	0,80	auxin-responsive GH3 family protein
256138_at	At1g48670	1,03	1,05	0,98	0,97	0,99	1,13	1,10	0,97	auxin-responsive GH3 family protein
256137_at	At1g48690	1,00	1,16	1,16	1,02	1,22	1,08	0,93	1,33	auxin-responsive GH3 family protein
256136_s_at	At1g48720	0,99	1,01	1,06	0,97	1,03	1,01	0,98	0,90	copia-like retrotransposon family
256135_at	At1g48730	0,99	0,95	0,99	1,01	1,11	0,91	1,08	0,86	expressed protein
256145_at	At1g48750	1,39	1,56	1,41	1,88	2,07	0,97	1,00	1,05	protease inhibitor/seed storage/lipid transfer protein (LTP) family protein
256146_at	At1g48760	0,93	0,79	0,82	1,00	1,31	1,01	0,78	0,89	delta-adaptin, putative
256165_at	At1g48780	1,02	0,76	0,90	0,99	1,17	0,98	0,79	1,07	hypothetical protein
256142_at	At1g48790	1,00	0,95	1,09	1,16	1,17	1,06	0,94	1,07	mov34 family protein
256164_at	At1g48800	0,98	1,00	0,92	1,00	1,08	1,07	1,15	0,93	terpene synthase/cyclase family protein

256163_at	At1g48820	0,94	0,98	0,94	0,99	1,06	1,15	1,01	0,94	terpene synthase/cyclase family protein
256143_at	At1g48830	1,27	1,09	1,07	1,26	1,16	0,96	0,97	0,92	40S ribosomal protein S7 (RPS7A)
245831_at	At1g48840	0,94	0,95	1,23	0,74	0,70	1,09	0,96	0,96	expressed protein
245832_at	At1g48850	1,09	0,97	0,91	1,05	1,07	1,08	1,05	1,05	chorismate synthase, putative / 5-enolpyruvylshikimate-3-phosphate phospl
246627_s_at	At1g48860	0,88	0,79	0,75	0,95	0,86	0,98	0,98	1,00	3-phosphoshikimate 1-carboxyvinyltransferase, putative / 5-enolpyruvylshik
246626_at	At1g48870	1,05	1,02	0,88	0,91	1,10	0,90	0,91	1,06	WD-40 repeat family protein
246625_at	At1g48880	0,99	1,17	0,97	0,99	1,14	0,96	1,03	0,95	hypothetical protein
246628_at	At1g48900	1,24	1,04	1,17	1,02	1,00	1,13	1,06	1,07	signal recognition particle 54 kDa protein 3 / SRP54 (SRP-54C)
246624_at	At1g48910	0,98	0,96	1,06	0,86	0,91	1,05	1,09	1,10	flavin-containing monooxygenase family protein / FMO family protein
246623_at	At1g48920	1,04	0,98	1,12	0,77	0,68	0,95	0,99	0,91	nucleolin, putative
260758_at	At1g48930	1,07	1,15	1,06	1,10	1,17	1,00	0,98	1,02	endo-1,4-beta-glucanase, putative / cellulase, putative
260757_at	At1g48940	1,00	0,99	0,96	0,87	1,03	0,97	0,95	1,10	plastocyanin-like domain-containing protein
260764_at	At1g48950	1,15	0,97	0,85	0,95	1,08	1,00	0,76	0,73	expressed protein
260766_at	At1g48960	0,75	0,97	1,06	0,99	1,39	0,92	0,97	1,25	universal stress protein (USP) family protein
260756_at	At1g48970	0,84	0,77	0,93	0,88	0,86	0,88	0,83	1,24	eukaryotic translation initiation factor 2B family protein / eIF-2B family prote
260755_at	At1g48980	1,09	0,79	1,09	0,94	0,91	1,17	0,99	1,17	hypothetical protein
260754_at	At1g49000	0,70	0,97	1,02	1,41	1,28	0,98	1,04	1,18	expressed protein
260769_at	At1g49010	0,86	0,94	0,95	0,72	0,76	1,09	0,97	0,91	myb family transcription factor
260762_at	At1g49015	0,98	0,92	0,95	1,21	0,99	0,82	0,93	0,96	eukaryotic translation initiation factor-related
260752_at	At1g49030	0,87	1,03	0,98	1,06	1,12	1,01	0,99	1,02	expressed protein
260751_at	At1g49040	0,95	0,82	0,76	1,08	0,94	0,88	0,92	0,89	stomatal cytokinesis defective / SCD1 protein (SCD1)
260772_at	At1g49050	0,84	0,87	0,91	0,94	1,04	0,95	1,07	1,15	aspartyl protease family protein
260750_at	At1g49100	0,93	1,04	1,00	0,99	0,95	1,03	0,97	0,98	leucine-rich repeat protein kinase, putative
260749_at	At1g49110	1,03	1,07	1,07	0,83	1,06	1,04	1,10	1,04	hypothetical protein
260767_s_at	At1g49140	1,27	1,16	1,12	1,44	1,07	0,97	0,99	0,95	NADH-ubiquinone oxidoreductase-related
260761_at	At1g49150	0,87	0,97	1,01	0,82	0,94	0,96	0,94	0,91	hypothetical protein
260771_at	At1g49160	1,30	1,40	1,91	0,85	0,95	1,21	1,38	1,20	protein kinase family protein
260760_at	At1g49170	0,93	1,05	1,22	1,05	1,30	0,80	0,83	1,02	expressed protein
260759_at	At1g49180	1,34	1,89	1,17	0,80	0,77	1,18	1,14	1,79	protein kinase family protein
257492_at	At1g49190	1,05	1,08	0,95	0,91	1,18	1,08	1,04	1,16	two-component responsive regulator family protein / response regulator fan
260770_at	At1g49200	1,00	1,01	0,98	1,07	0,86	0,97	0,94	1,05	zinc finger (C3HC4-type RING finger) family protein
260748_at	At1g49210	1,07	0,85	1,01	0,93	1,04	0,97	1,01	0,85	zinc finger (C3HC4-type RING finger) family protein
260763_at	At1g49220	0,97	1,08	0,90	0,94	1,01	0,90	0,88	1,13	zinc finger (C3HC4-type RING finger) family protein
260753_at	At1g49230	0,56	0,76	0,89	0,96	1,00	0,95	1,39	1,88	zinc finger (C3HC4-type RING finger) family protein
260765_at	At1g49240	0,93	0,90	0,88	0,89	0,88	0,76	0,81	0,85	actin 8 (ACT8)
260768_at	At1g49245	0,84	0,87	0,73	1,35	1,18	0,71	0,65	0,75	expressed protein
262447_at	At1g49250	1,03	1,01	0,95	0,95	0,97	1,02	0,94	0,86	ATP dependent DNA ligase family protein
262390_at	At1g49260	1,06	1,01	1,10	1,06	1,06	1,02	0,98	0,88	hypothetical protein
262389_at	At1g49270	0,86	1,00	1,00	0,94	1,12	1,03	1,08	0,93	protein kinase family protein
257469_at	At1g49290	0,93	0,99	0,97	1,05	0,97	1,03	1,00	1,14	expressed protein
262401_at	At1g49300	0,81	0,74	0,83	1,01	1,24	1,01	0,95	0,92	Ras-related GTP-binding protein, putative
262446_at	At1g49310	1,15	1,37	1,32	0,96	0,95	1,13	1,08	1,02	expressed protein
262388_at	At1g49320	1,85	1,66	1,42	1,89	1,64	0,99	1,15	0,87	BURP domain-containing protein
262387_s_at	At1g49340	0,87	0,83	0,72	0,91	0,83	1,00	0,86	0,82	phosphatidylinositol 3- and 4-kinase family protein
262398_at	At1g49350	0,95	0,84	0,88	0,81	1,02	0,81	0,86	0,83	pfkB-type carbohydrate kinase family protein
262386_at	At1g49370	0,87	0,93	0,94	0,95	0,98	1,13	1,04	0,96	---

262397_at	At1g49380	0,93	0,96	0,96	0,69	0,61	1,38	1,19	1,37	cytochrome c biogenesis protein family
262416_at	At1g49390	0,89	0,97	0,95	0,95	1,22	1,01	1,05	1,29	oxidoreductase, 2OG-Fe(II) oxygenase family protein
262415_at	At1g49400	1,03	0,98	1,00	1,01	1,07	1,11	0,93	0,89	ribosomal protein S17 family protein
262402_at	At1g49410	1,93	1,44	1,15	1,42	1,33	0,99	0,94	0,87	expressed protein
262414_at	At1g49430	1,13	1,32	1,44	0,94	0,97	0,98	0,95	0,89	long-chain-fatty-acid--CoA ligase / long-chain acyl-CoA synthetase
262448_at	At1g49450	0,82	1,06	0,91	0,76	0,82	1,27	1,04	0,91	transducin family protein / WD-40 repeat family protein
262396_at	At1g49470	0,64	0,93	0,97	0,94	1,07	0,87	0,86	1,03	expressed protein
262400_at	At1g49480	0,89	1,00	1,13	1,05	0,88	0,90	0,99	1,17	transcriptional factor B3 family protein
262393_at	At1g49490	1,09	1,01	1,04	0,89	1,01	1,19	0,95	1,00	leucine-rich repeat family protein / extensin family protein
262399_at	At1g49500	0,63	0,79	0,74	0,67	0,44	0,97	1,25	1,45	expressed protein
262394_at	At1g49510	0,93	1,04	1,24	0,85	0,99	0,95	0,82	0,82	expressed protein
262392_at	At1g49520	0,88	0,91	0,91	0,83	0,84	0,84	1,19	1,08	SWIB complex BAF60b domain-containing protein
262391_at	At1g49530	0,95	1,13	0,90	0,94	0,76	0,93	1,18	1,25	geranylgeranyl pyrophosphate synthase (GGPS6) / GGPP synthetase / farr
262395_at	At1g49540	0,90	1,04	1,01	0,88	0,77	0,76	0,86	1,20	transducin family protein / WD-40 repeat family protein
261610_at	At1g49560	1,11	0,85	0,82	0,83	1,05	1,17	0,89	0,98	myb family transcription factor
261606_at	At1g49570	2,39	2,76	2,15	2,78	1,98	2,13	2,02	1,47	peroxidase, putative
261605_at	At1g49580	0,84	0,97	1,14	0,94	0,83	1,04	0,97	0,91	calcium-dependent protein kinase, putative / CDPK, putative
261604_at	At1g49590	1,24	1,36	1,48	1,03	1,06	1,07	1,20	1,28	formin-binding protein-related
261603_at	At1g49600	1,02	1,12	1,03	0,84	0,81	0,90	0,98	0,92	RNA-binding protein 47 (RBP47), putative
257483_at	At1g49620	0,97	0,84	0,82	0,75	1,06	0,92	0,92	0,78	kip-related protein 7 (KRP7) / cyclin-dependent kinase inhibitor 7 (ICK7)
261602_at	At1g49630	0,81	0,84	0,72	0,88	0,70	1,01	0,92	0,87	peptidase M16 family protein / insulinase family protein
261608_at	At1g49650	0,62	0,64	0,86	0,78	0,84	0,90	0,78	0,75	cell death associated protein-related
261607_at	At1g49660	1,07	1,06	1,12	1,25	1,60	0,58	0,77	0,97	expressed protein
261601_at	At1g49670	0,78	0,73	0,70	0,82	0,86	0,89	0,82	0,82	ARP protein (REF)
261600_x_at	At1g49680	0,92	0,97	0,97	0,98	1,03	1,03	0,99	0,95	hypothetical protein
261599_at	At1g49700	0,97	1,04	1,02	1,04	1,10	1,04	1,00	0,75	expressed protein
261612_at	At1g49710	1,26	1,26	1,08	1,06	1,21	0,98	0,81	1,12	fucosyltransferase-like protein, putative / FucT2, putative / FucTB, putative
261613_at	At1g49720	0,81	0,96	0,96	0,70	0,75	1,05	1,06	1,13	ABA-responsive element-binding protein / abscisic acid responsive element
261611_at	At1g49730	0,87	1,03	1,32	1,08	1,16	0,92	0,89	0,90	protein kinase family protein
261609_at	At1g49740	1,17	1,08	1,15	0,94	0,99	0,99	1,27	1,19	expressed protein
261598_at	At1g49750	0,64	0,61	0,59	1,01	0,92	1,01	0,69	0,70	leucine-rich repeat family protein
261614_at	At1g49760	1,09	1,08	1,04	0,95	0,89	1,05	0,97	0,87	polyadenylate-binding protein, putative / PABP, putative
261597_at	At1g49780	0,85	0,97	0,91	0,73	0,93	0,80	1,03	0,96	U-box domain-containing protein
259808_at	At1g49790	0,95	1,05	0,98	0,94	1,03	1,03	0,90	1,09	F-box family protein-related
259809_at	At1g49800	0,95	1,08	1,04	1,13	1,00	0,99	0,95	1,15	hypothetical protein
259810_at	At1g49810	1,00	0,99	0,94	0,92	1,23	1,01	1,00	0,96	sodium hydrogen antiporter, putative
259819_at	At1g49820	1,10	1,05	1,10	1,21	1,25	0,88	0,91	0,82	5-methylthioribose kinase family
259811_at	At1g49830	1,13	1,30	1,14	0,89	1,06	0,87	1,22	0,99	ethylene-responsive protein -related
259812_at	At1g49840	0,94	1,92	1,60	0,76	0,73	0,89	1,33	1,08	expressed protein
259816_at	At1g49850	1,02	1,02	1,30	1,09	1,06	1,07	1,03	1,08	zinc finger (C3HC4-type RING finger) family protein
259813_at	At1g49860	1,06	1,02	0,97	1,44	1,83	1,22	0,95	0,84	glutathione S-transferase, putative
259815_at	At1g49870	1,08	1,05	0,97	0,97	0,99	1,16	1,06	1,03	expressed protein
259817_at	At1g49880	1,14	1,05	1,16	1,11	0,87	0,96	1,05	0,93	Erv1/Alr family protein
259818_at	At1g49890	0,83	1,04	1,27	0,87	1,08	0,81	1,02	0,86	expressed protein
259814_at	At1g49900	0,96	1,08	0,93	0,93	1,14	0,97	1,01	0,98	zinc finger (C2H2 type) family protein
245814_at	At1g49910	1,07	1,14	1,34	1,15	0,99	1,14	0,92	0,69	WD-40 repeat family protein / mitotic checkpoint protein, putative

245813_at	At1g49920	0,99	1,11	1,23	0,98	1,02	1,05	1,02	0,94	zinc finger protein-related
261633_at	At1g49930	1,01	1,12	1,08	0,83	0,72	1,28	1,10	1,01	hypothetical protein
261631_at	At1g49940	0,89	1,02	1,08	0,98	1,01	1,11	1,02	1,11	expressed protein
261637_at	At1g49950	1,05	0,96	0,86	1,04	1,15	1,29	1,18	1,19	DNA-binding protein, putative
261640_at	At1g49960	1,19	1,16	0,99	1,05	1,02	0,98	0,99	1,02	xanthine/uracil permease family protein
261634_at	At1g49970	0,94	0,87	0,83	0,88	1,00	1,17	1,20	1,40	ATP-dependent Clp protease proteolytic subunit (ClpR1) (nClpP5)
261638_at	At1g49975	1,34	1,16	0,93	0,80	1,09	0,99	0,89	0,84	expressed protein
261629_at	At1g49980	0,89	1,12	1,14	0,79	0,78	1,10	1,04	1,35	UMUC-like DNA repair family protein
261628_at	At1g50000	1,08	0,99	1,01	0,99	0,95	1,15	0,90	1,09	hypothetical protein
261639_at	At1g50010	1,03	1,00	0,84	0,94	0,91	0,83	0,84	0,87	tubulin alpha-2/alpha-4 chain (TUA2)
261635_at	At1g50020	1,05	1,13	1,24	0,91	0,96	1,13	1,06	1,06	expressed protein
261659_at	At1g50030	0,97	0,94	1,15	0,77	0,84	1,12	1,10	1,15	target of rapamycin protein (TOR)
261658_at	At1g50040	1,08	1,43	1,34	0,93	1,24	0,94	1,03	1,03	expressed protein
261657_at	At1g50050	1,64	1,53	1,74	1,07	1,17	0,91	1,01	1,11	pathogenesis-related protein, putative
261691_at	At1g50060	4,29	3,77	2,51	4,87	5,09	1,15	1,38	1,25	pathogenesis-related protein, putative
261630_at	At1g50080	0,99	0,93	0,99	0,86	1,02	1,03	1,02	0,92	hypothetical protein
261690_at	At1g50090	0,97	0,93	1,08	1,21	1,31	1,10	1,09	1,01	aminotransferase class IV family protein
261636_at	At1g50110	1,02	1,05	0,89	1,06	1,06	0,97	0,95	0,82	branched-chain amino acid aminotransferase 6 / branched-chain amino aci
261632_at	At1g50120	0,95	0,73	0,93	0,98	0,98	1,04	0,98	0,97	expressed protein
261689_at	At1g50140	1,00	0,91	0,86	0,87	0,93	1,07	1,06	0,94	AAA-type ATPase family protein
262471_at	At1g50150	0,91	0,92	1,01	0,97	0,78	1,08	1,06	0,86	hypothetical protein
262472_at	At1g50160	1,00	1,06	0,92	0,99	0,95	0,99	0,91	0,90	hypothetical protein
262417_at	At1g50170	0,83	0,91	1,09	0,97	1,00	0,90	0,90	0,83	expressed protein
262470_at	At1g50180	0,98	1,05	0,99	1,12	1,05	1,07	1,04	1,08	disease resistance protein (CC-NBS-LRR class), putative
262469_s_at	At1g50190	1,05	0,92	1,07	0,98	1,09	1,00	1,06	1,07	pseudogene, CHP-rich zinc finger protein, putative
262468_at	At1g50200	1,08	0,99	1,03	0,84	0,86	0,98	1,08	1,06	aminoacyl-tRNA synthetase family protein
262474_at	At1g50230	0,98	1,09	1,07	1,05	1,00	0,96	0,91	0,80	protein kinase family protein
262467_at	At1g50240	1,21	0,86	1,15	1,13	1,14	0,94	0,85	0,69	armadillo/beta-catenin repeat family protein
262473_at	At1g50250	0,95	0,88	0,87	0,75	0,75	1,19	1,24	1,24	cell division protein ftsH homolog 1, chloroplast (FTSH1) (FTSH)
262466_at	At1g50260	0,81	0,91	0,88	0,81	1,00	0,98	1,01	1,06	C2 domain-containing protein
262465_at	At1g50270	1,05	1,07	0,97	0,81	0,90	0,97	1,14	0,77	pentatricopeptide (PPR) repeat-containing protein
262464_at	At1g50280	1,02	1,00	0,95	0,96	0,94	1,27	1,01	0,96	phototropic-responsive NPH3 family protein
262421_at	At1g50290	0,75	0,60	0,62	1,20	0,85	1,05	0,92	0,82	expressed protein
262420_at	At1g50300	1,21	1,25	1,03	1,02	0,99	0,97	1,05	0,95	zinc finger (Ran-binding) family protein / RNA recognition motif (RRM)-conta
262463_at	At1g50310	0,92	1,04	0,84	0,97	1,11	1,01	0,97	0,89	monosaccharide transporter (STP9)
262418_at	At1g50320	1,26	1,05	1,03	0,94	0,80	1,07	1,08	1,14	thioredoxin x
262475_at	At1g50340	1,04	1,00	0,98	1,01	1,00	1,01	0,96	1,18	invertase/pectin methylesterase inhibitor family protein
262462_at	At1g50350	1,01	1,03	0,96	1,02	1,13	1,01	1,02	1,03	hypothetical protein
262461_at	At1g50360	0,76	0,73	0,89	0,78	0,79	1,01	1,03	1,06	myosin family protein
262476_at	At1g50370	0,97	0,90	1,05	1,23	1,23	0,97	0,91	0,93	serine/threonine protein phosphatase, putative
262419_at	At1g50380	0,98	0,95	0,95	0,94	0,74	1,06	1,04	0,98	prolyl oligopeptidase family protein
262460_s_at	At1g50390	1,07	0,99	1,02	0,95	1,02	1,08	1,15	1,22	pfkB-type carbohydrate kinase family protein
262459_at	At1g50400	1,09	1,10	0,96	1,19	0,74	1,06	1,20	1,11	porin family protein
261862_at	At1g50410	1,17	1,14	1,39	0,86	0,88	1,13	1,43	1,40	SNF2 domain-containing protein / helicase domain-containing protein / RIN
261866_at	At1g50420	1,29	1,24	1,12	1,48	1,55	0,96	0,84	0,99	scarecrow-like transcription factor 3 (SCL3)
261865_at	At1g50430	0,88	0,81	0,77	1,17	0,94	0,85	0,81	0,81	7-dehydrocholesterol reductase / 7-DHC reductase / sterol delta-7-reductas

261852_at	At1g50440	1,19	1,17	1,06	0,88	0,87	0,94	0,93	1,01	zinc finger (C3HC4-type RING finger) family protein
261861_at	At1g50450	1,40	1,29	1,10	0,86	0,83	1,26	1,42	1,47	expressed protein
261851_at	At1g50460	0,79	0,73	1,04	0,92	1,13	0,91	0,95	1,31	hexokinase, putative
261850_at	At1g50470	0,96	1,06	1,00	0,91	0,94	1,07	1,04	0,85	hypothetical protein
261864_s_at	At1g50480	0,90	0,73	0,93	0,83	0,84	0,86	0,83	0,79	formate--tetrahydrofolate ligase / 10-formyltetrahydrofolate synthetase (THF
261859_at	At1g50490	1,50	1,16	1,11	1,44	1,28	0,87	0,86	0,93	ubiquitin-conjugating enzyme 20 (UBC20)
261880_at	At1g50500	1,07	1,10	1,00	0,97	1,01	1,17	1,20	1,36	membrane trafficking VPS53 family protein
261855_at	At1g50510	1,00	1,09	1,10	1,15	0,93	0,95	1,02	1,10	indigoidine synthase A family protein
261879_at	At1g50520	0,99	1,00	0,70	0,97	1,14	1,04	0,97	1,11	cytochrome P450 family protein
261856_at	At1g50530	1,02	1,07	0,95	0,94	1,09	1,05	1,04	0,97	hypothetical protein
261878_at	At1g50560	1,01	0,91	0,91	1,07	1,00	1,06	1,09	1,28	cytochrome P450, putative
261858_at	At1g50570	0,93	1,07	1,13	1,06	1,21	0,91	1,01	1,35	C2 domain-containing protein
261867_at	At1g50575	1,02	0,79	0,81	0,89	0,85	0,92	0,93	0,79	lysine decarboxylase family protein
261877_at	At1g50580	1,00	0,91	0,80	1,10	0,97	1,01	1,11	1,04	glycosyltransferase family protein
261876_at	At1g50590	1,11	1,14	1,05	0,84	0,76	1,06	1,18	1,03	pirin, putative
261860_at	At1g50600	0,96	1,04	1,04	1,02	0,91	0,98	1,39	1,44	scarecrow-like transcription factor 5 (SCL5)
261875_at	At1g50610	1,03	1,02	0,96	1,04	1,00	1,18	1,02	0,88	leucine-rich repeat transmembrane protein kinase, putative
261857_at	At1g50620	1,20	1,26	1,38	0,90	0,99	1,20	1,08	1,28	PHD finger family protein
261863_at	At1g50630	0,77	0,74	0,74	1,03	0,93	0,74	0,94	0,81	expressed protein
261874_at	At1g50640	1,04	0,97	0,75	1,52	1,37	0,93	0,89	1,11	ethylene-responsive element-binding factor 3 (ERF3)
261908_at	At1g50650	0,82	0,96	0,93	0,97	1,11	0,95	0,99	0,84	stigma-specific Stig1 family protein
261853_at	At1g50660	0,93	0,99	1,11	0,94	1,16	1,00	0,96	0,88	expressed protein
261854_at	At1g50670	0,94	0,87	0,84	1,29	1,16	1,06	1,00	1,01	OTU-like cysteine protease family protein
257581_s_at	At1g50680	0,99	0,98	1,05	0,94	1,01	1,03	0,87	1,05	AP2 domain-containing transcription factor, putative
256405_at	At1g50700	0,89	0,72	0,77	1,00	1,03	1,11	0,98	0,97	calcium-dependent protein kinase, putative / CDPK, putative
256406_at	At1g50710	0,89	0,92	0,97	0,99	0,98	0,97	0,90	0,90	expressed protein
257582_at	At1g50720	1,01	0,96	1,04	1,06	1,05	1,09	1,08	0,90	stigma-specific Stig1 family protein
246630_at	At1g50730	1,30	1,15	1,08	0,97	0,94	1,15	1,10	1,06	expressed protein
246631_at	At1g50740	0,75	0,86	0,97	0,95	1,05	0,86	1,16	1,40	expressed protein
246629_at	At1g50750	0,92	1,00	0,98	1,05	1,05	1,20	0,96	1,06	expressed protein
257586_at	At1g50760	1,03	1,04	0,99	0,93	1,13	1,01	1,06	0,81	hypothetical protein
256202_s_at	At1g50780	0,94	0,99	0,99	1,01	0,91	0,96	0,91	0,95	MADS-box protein (AGL74)
256203_at	At1g50800	0,90	1,05	0,99	1,04	1,05	1,00	1,02	1,12	hypothetical protein
256204_at	At1g50840	0,97	0,91	0,99	0,65	0,61	1,13	1,13	1,07	polI-like DNA polymerase, putative
256205_at	At1g50890	1,12	1,17	0,98	0,79	1,01	1,00	0,89	1,02	expressed protein
256215_at	At1g50900	0,92	0,87	1,00	0,78	0,84	0,97	0,95	0,96	expressed protein
256206_at	At1g50910	0,94	1,02	1,01	0,93	1,12	1,00	0,91	0,98	expressed protein
256207_at	At1g50920	1,25	1,14	1,18	0,83	0,85	1,12	1,07	1,13	GTP-binding protein-related
256208_at	At1g50930	0,89	1,06	0,92	1,03	1,03	0,99	1,05	0,97	hypothetical protein
256209_at	At1g50940	1,02	1,12	1,34	0,78	1,10	1,02	1,03	1,02	electron transfer flavoprotein alpha subunit family protein
256210_at	At1g50950	1,00	1,00	0,92	0,99	0,94	0,97	0,93	0,90	thioredoxin-related
256211_at	At1g50960	1,04	1,01	1,04	1,13	1,00	1,01	0,98	0,96	gibberellin 20-oxidase-related
256212_at	At1g50970	1,12	1,18	1,07	1,04	0,90	1,02	1,12	1,51	membrane trafficking VPS53 family protein
256213_at	At1g50990	0,97	1,09	1,11	0,91	0,99	1,02	0,91	0,79	protein kinase-related
256214_x_at	At1g51000	0,98	0,98	0,97	1,02	1,03	1,01	1,05	0,97	expressed protein
245750_at	At1g51060	0,97	1,01	1,18	1,24	1,48	1,03	1,02	0,98	histone H2A, putative

245746_at	At1g51070	1,12	1,02	0,93	1,22	1,31	0,97	1,03	1,04	basic helix-loop-helix (bHLH) family protein
245743_at	At1g51080	1,16	1,04	1,05	0,76	0,89	1,26	1,05	0,96	expressed protein
245749_at	At1g51090	0,80	1,36	2,32	1,00	1,52	1,56	1,68	2,13	heavy-metal-associated domain-containing protein
245747_at	At1g51100	1,00	1,03	1,11	0,87	0,89	0,95	0,99	0,82	expressed protein
245744_at	At1g51110	0,69	0,60	0,59	1,00	0,98	1,00	1,05	1,07	plastid-lipid associated protein PAP / fibrillin family protein
245745_at	At1g51115	0,64	0,58	0,53	0,76	0,90	1,02	1,18	1,17	plastid-lipid associated protein PAP / fibrillin family protein
245748_at	At1g51140	0,93	0,92	0,86	1,12	1,16	1,12	1,12	1,10	basic helix-loop-helix (bHLH) family protein
265148_at	At1g51160	1,10	1,01	1,05	1,12	1,31	0,92	0,89	0,93	synbindin, putative
265144_at	At1g51170	0,92	0,92	0,99	0,93	1,06	1,20	1,01	0,96	protein kinase family protein
265145_at	At1g51190	0,94	1,03	1,12	0,91	1,13	0,98	1,09	1,06	ovule development protein, putative
265150_at	At1g51200	1,09	0,93	0,92	0,91	0,97	0,93	0,95	0,95	zinc finger (AN1-like) family protein
265146_at	At1g51210	0,86	0,92	1,04	1,00	1,03	1,01	1,02	0,95	UDP-glucoronosyl/UDP-glucosyl transferase family protein
265133_s_at	At1g51250	0,97	0,99	1,03	1,03	1,05	1,01	0,88	1,11	hypothetical protein
265134_at	At1g51260	0,99	0,98	1,04	1,01	1,04	1,06	0,83	0,98	acyl-CoA:1-acylglycerol-3-phosphate acyltransferase, putative
265135_at	At1g51270	0,96	1,06	1,12	1,31	1,41	1,18	1,43	1,29	vesicle-associated membrane protein, putative / VAMP, putative
265136_at	At1g51280	1,03	1,10	0,99	1,42	1,19	1,28	1,77	1,85	disease resistance protein (TIR class), putative
265137_at	At1g51290	1,01	1,04	1,04	0,99	1,12	1,08	0,98	0,89	F-box family protein (FBX10)
265138_at	At1g51300	1,10	1,04	1,06	1,07	0,98	1,00	0,81	1,02	acyl-protein thioesterase-related
265139_at	At1g51310	1,22	1,14	1,03	0,81	0,69	0,99	0,99	0,98	tRNA methyl transferase family protein
265140_at	At1g51320	1,02	0,94	0,92	1,02	0,98	1,01	0,97	0,88	F-box family protein (FBX11)
265151_at	At1g51340	1,11	1,22	1,20	1,08	1,06	0,89	0,80	1,14	MATE efflux family protein
265141_at	At1g51350	1,01	1,07	1,19	0,91	0,99	1,06	0,98	0,92	armadillo/beta-catenin repeat family protein
265142_at	At1g51360	1,13	1,00	0,93	1,10	1,15	0,99	0,80	0,95	expressed protein
265147_at	At1g51380	1,86	1,53	1,63	1,11	1,03	1,08	1,07	0,99	eukaryotic translation initiation factor 4A, putative / eIF-4A, putative
265143_at	At1g51390	0,97	0,98	0,96	1,12	1,03	0,74	0,93	0,90	nitrogen fixation NifU-like family protein
265149_at	At1g51400	0,90	1,05	1,31	1,08	1,16	0,95	0,89	1,03	photosystem II 5 kD protein
260518_at	At1g51410	0,97	0,93	0,90	0,97	0,94	0,97	1,03	0,92	cinnamyl-alcohol dehydrogenase, putative (CAD)
260517_at	At1g51420	1,32	1,13	1,35	1,16	1,39	1,11	1,11	1,29	sucrose-phosphatase, putative
257497_at	At1g51430	0,92	0,92	1,01	0,86	1,07	0,90	0,91	1,13	expressed protein
260491_at	At1g51440	0,96	0,99	0,85	1,25	1,23	0,99	0,93	0,95	lipase class 3 family protein
260516_at	At1g51450	0,91	0,99	0,87	0,99	1,29	0,92	1,00	0,85	SPla/RYanodine receptor (SPRY) domain-containing protein
260515_at	At1g51460	1,05	1,24	1,17	0,86	1,04	1,04	1,08	1,06	ABC transporter family protein
260514_at	At1g51480	0,98	1,00	1,07	1,08	1,06	1,02	1,02	1,02	disease resistance protein (CC-NBS-LRR class), putative
260513_at	At1g51490	1,04	1,06	0,93	1,05	1,03	1,04	0,90	0,97	glycosyl hydrolase family 1 protein
260490_at	At1g51500	1,18	1,46	1,57	0,78	0,81	1,24	1,17	1,24	ABC transporter family protein
260487_at	At1g51510	1,01	0,96	1,02	0,99	1,07	0,95	0,94	0,98	RNA-binding protein, putative
260512_at	At1g51520	0,93	1,07	1,02	0,89	0,98	1,04	1,10	1,02	expressed protein
260519_at	At1g51540	0,89	0,89	1,03	1,07	1,34	0,93	0,94	0,87	kelch repeat-containing protein
260486_at	At1g51550	0,98	0,96	1,03	0,81	0,94	0,71	0,76	1,01	F-box family protein
260511_at	At1g51570	1,04	1,05	1,04	0,99	1,00	0,83	0,73	0,77	C2 domain-containing protein
260510_at	At1g51580	1,09	1,03	1,11	0,78	0,99	0,99	0,90	0,94	KH domain-containing protein
260520_at	At1g51590	1,10	0,92	0,82	1,00	1,09	0,94	0,92	0,71	mannosyl-oligosaccharide 1,2-alpha-mannosidase, putative
260488_at	At1g51600	0,98	1,03	1,05	1,02	1,02	0,96	0,96	0,80	zinc finger (GATA type) family protein
260489_at	At1g51610	0,85	0,84	1,05	0,80	0,93	0,98	0,91	0,89	cation efflux family protein / metal tolerance protein, putative (MTPc4)
256177_at	At1g51620	0,99	0,70	0,71	0,82	0,82	1,00	1,14	1,10	protein kinase family protein
256176_at	At1g51640	1,00	0,98	0,94	1,01	1,09	1,08	1,09	0,94	exocyst subunit EXO70 family protein

256184_at	At1g51650	1,46	1,25	1,06	1,34	1,39	1,04	1,05	0,93	ATP synthase epsilon chain, mitochondrial
256183_at	At1g51660	0,77	0,61	0,74	1,13	1,05	0,87	0,75	0,80	mitogen-activated protein kinase kinase (MAPKK), putative (MKK4)
256175_at	At1g51670	0,92	0,56	0,55	0,88	0,92	1,04	0,79	1,13	expressed protein
256186_at	At1g51680	1,25	1,28	1,34	1,17	1,02	1,32	1,53	1,35	4-coumarate--CoA ligase 1 / 4-coumaroyl-CoA synthase 1 (4CL1)
256187_at	At1g51690	0,99	1,11	1,12	0,91	0,74	0,99	1,10	1,22	serine/threonine protein phosphatase 2A (PP2A) 55 kDa regulatory subunit
256185_at	At1g51700	1,03	1,19	0,98	1,11	1,32	1,13	1,19	1,04	Dof-type zinc finger domain-containing protein (ADOF1)
256179_at	At1g51710	0,92	0,87	0,89	0,98	1,11	0,97	0,99	0,99	ubiquitin-specific protease 6, putative (UBP6)
256174_at	At1g51720	1,09	0,99	1,38	0,79	0,83	1,07	1,28	1,41	glutamate dehydrogenase, putative
256173_at	At1g51730	1,07	1,10	1,17	1,04	1,24	0,96	1,00	1,06	RWD domain-containing protein
256182_at	At1g51740	1,11	0,89	0,89	1,24	1,04	1,00	0,91	0,87	syntaxin 81 (SYP81)
256172_at	At1g51745	0,97	1,05	1,00	0,90	0,95	0,93	0,93	0,84	expressed protein
256171_s_at	At1g51750	0,86	0,93	1,14	1,10	0,95	1,04	0,97	1,17	---
256178_s_at	At1g51780	0,71	0,54	0,84	1,05	1,41	0,85	0,85	0,89	IAA-amino acid hydrolase 3 / IAA-Ala hydrolase 3 (IAR3)
256170_at	At1g51790	1,01	0,93	0,75	1,04	1,26	1,01	0,79	0,86	leucine-rich repeat protein kinase, putative
256169_at	At1g51800	1,03	0,99	1,38	1,84	1,63	1,08	0,69	0,86	leucine-rich repeat protein kinase, putative
256168_at	At1g51805	0,91	0,84	1,65	0,49	0,35	1,19	1,27	1,78	leucine-rich repeat protein kinase, putative
256180_at	At1g51810	0,93	0,97	1,16	1,09	1,13	1,01	0,98	1,10	leucine-rich repeat protein kinase, putative
256181_at	At1g51820	1,00	1,04	1,00	1,49	1,82	1,00	0,72	1,00	leucine-rich repeat protein kinase, putative
246375_at	At1g51830	0,81	0,94	0,69	1,11	0,88	1,09	1,19	1,19	leucine-rich repeat protein kinase, putative
246374_at	At1g51840	0,79	0,86	0,83	0,98	0,89	1,04	1,15	0,96	protein kinase-related
246366_at	At1g51850	0,95	0,82	0,79	1,49	1,16	1,07	1,08	1,25	leucine-rich repeat protein kinase, putative
246373_at	At1g51860	0,94	0,87	0,80	1,21	1,38	1,00	0,88	0,92	leucine-rich repeat protein kinase, putative
246367_at	At1g51880	1,02	0,96	1,10	0,98	1,14	0,98	1,06	0,94	leucine-rich repeat protein kinase, putative
246368_at	At1g51890	1,57	1,45	1,47	1,71	0,96	1,58	1,16	1,40	leucine-rich repeat protein kinase, putative
246372_at	At1g51900	1,05	1,05	0,96	1,07	0,99	1,11	1,03	0,97	hypothetical protein
246369_at	At1g51910	0,99	1,00	1,01	0,98	1,12	0,98	1,01	0,95	protein kinase family protein
246349_at	At1g51915	0,97	0,98	0,96	1,04	1,08	1,02	0,92	1,03	cryptdin protein-related
246370_at	At1g51920	0,90	0,89	0,77	1,07	1,06	1,06	1,17	1,29	expressed protein
246371_at	At1g51940	0,84	0,78	0,82	0,67	0,72	0,86	0,87	0,94	protein kinase family protein / peptidoglycan-binding LysM domain-containi
246376_at	At1g51950	0,91	1,06	0,95	0,99	1,10	0,86	1,06	1,02	auxin-responsive protein / indoleacetic acid-induced protein 18 (IAA18)
265047_at	At1g51960	1,02	1,10	1,07	0,96	1,12	0,93	0,96	0,93	calmodulin-binding family protein
265056_at	At1g51980	1,05	0,92	0,89	1,17	1,06	0,94	0,94	0,95	mitochondrial processing peptidase alpha subunit, putative
265052_at	At1g51990	0,95	1,23	1,31	0,99	1,12	1,02	1,01	0,81	O-methyltransferase family 2 protein
265053_at	At1g52000	0,67	0,67	1,04	1,02	0,93	1,05	0,91	0,90	jacalin lectin family protein
265054_x_at	At1g52010	1,06	0,99	0,98	1,01	0,97	1,13	1,10	0,92	Mutator-like transposase family
265055_at	At1g52020	1,05	0,99	1,08	1,04	1,09	1,05	1,00	0,98	pseudogene, Ulp1 protease family
265058_s_at	At1g52040	0,90	0,91	0,95	0,75	0,83	0,82	0,77	0,58	myrosinase-binding protein, putative (F-ATMBP)
265048_at	At1g52050	1,30	1,06	0,88	2,71	2,09	0,99	1,09	1,21	jacalin lectin family protein
265049_at	At1g52060	2,13	1,77	1,38	2,92	2,58	1,20	1,38	1,50	jacalin lectin family protein
265050_at	At1g52070	1,48	1,13	0,77	3,40	1,65	1,20	1,71	1,54	jacalin lectin family protein
265059_at	At1g52080	0,85	0,98	0,87	1,03	1,11	1,10	1,12	1,26	actin binding protein family
265046_s_at	At1g52090	1,00	1,00	0,97	1,00	1,03	1,00	1,05	1,03	Mutator-like transposase family
265051_at	At1g52100	1,67	1,56	1,66	0,80	0,87	1,06	1,23	1,16	jacalin lectin family protein
265057_at	At1g52140	1,27	1,67	1,08	1,41	1,32	0,96	1,21	1,03	expressed protein
265060_at	At1g52150	1,64	1,52	1,32	1,14	1,30	0,89	1,05	0,93	homeobox-leucine zipper family protein / lipid-binding START domain-conta
259835_at	At1g52160	0,96	0,99	0,98	0,82	0,80	1,03	0,84	0,78	metallo-beta-lactamase family protein

259837_at	At1g52180	1,14	1,20	1,09	1,04	1,16	0,83	0,98	0,92	major intrinsic family protein / MIP family protein
259839_at	At1g52190	1,16	1,21	1,36	0,66	0,63	0,69	0,73	0,75	proton-dependent oligopeptide transport (POT) family protein
259841_at	At1g52200	0,87	0,86	0,97	1,14	1,06	0,86	0,60	0,65	expressed protein
259838_at	At1g52220	1,03	1,07	1,06	0,89	0,90	0,94	0,96	0,92	expressed protein
259840_at	At1g52230	1,06	0,99	1,05	0,98	0,98	0,99	0,93	0,97	photosystem I reaction center subunit VI, chloroplast, putative / PSI-H, puta
259836_at	At1g52240	0,95	1,04	1,01	1,03	1,11	1,00	1,11	0,98	expressed protein
257504_at	At1g52250	0,98	1,15	0,89	1,08	1,30	0,95	1,03	0,87	dynein light chain type 1 family protein
259637_at	At1g52260	0,94	0,88	0,76	0,79	0,98	0,93	0,93	1,01	thioredoxin family protein
259611_at	At1g52280	0,68	0,48	0,57	1,13	1,02	0,96	0,92	0,89	Ras-related GTP-binding protein, putative
259671_at	At1g52290	0,73	0,55	0,75	0,94	0,85	0,63	0,70	0,97	protein kinase family protein
259612_at	At1g52300	1,39	1,22	1,17	1,23	1,17	0,98	1,02	0,99	60S ribosomal protein L37 (RPL37B)
259670_at	At1g52310	1,12	0,97	1,46	0,94	0,95	1,02	0,94	1,18	protein kinase family protein / C-type lectin domain-containing protein
259610_at	At1g52320	0,79	0,91	0,91	0,71	0,85	0,93	0,89	1,10	expressed protein
259669_at	At1g52340	1,27	1,16	1,14	1,27	1,02	1,03	1,01	1,02	short-chain dehydrogenase/reductase (SDR) family protein
259638_at	At1g52360	0,93	0,79	0,89	1,13	0,91	0,90	0,98	0,89	coatomer protein complex, subunit beta 2 (beta prime), putative
259639_at	At1g52380	1,13	0,96	0,93	1,31	1,03	1,04	0,94	0,96	Ran-binding protein 1 domain-containing protein / RanBP1 domain-containi
259668_at	At1g52390	0,94	0,99	1,05	1,13	1,01	1,03	1,04	0,92	hypothetical protein
259640_at	At1g52400	1,40	0,95	0,88	0,84	0,75	0,82	0,79	0,87	glycosyl hydrolase family 1 protein / beta-glucosidase, putative (BG1)
259609_at	At1g52410	0,76	0,67	0,49	1,22	0,96	0,83	0,88	0,97	caldesmon-related
262155_at	At1g52420	0,89	0,78	0,79	0,91	1,01	1,09	0,97	0,95	glycosyl transferase family 1 protein
262152_s_at	At1g52450	1,16	1,16	0,99	0,98	0,97	1,17	1,20	1,07	ubiquitin carboxyl-terminal hydrolase-related
262141_s_at	At1g52460	1,00	0,96	1,11	0,94	1,04	0,90	0,99	0,98	hypothetical protein
262140_at	At1g52470	0,99	1,10	1,06	1,14	0,95	1,05	1,05	0,89	hypothetical protein
262129_at	At1g52500	1,32	1,09	1,07	1,02	0,89	1,16	1,09	0,93	formamidopyrimidine-DNA glycolase family protein / mutM, putative (MMH-:
262151_at	At1g52510	0,88	0,79	0,84	0,98	0,89	0,95	0,94	0,98	hydrolase, alpha/beta fold family protein
262150_at	At1g52520	1,05	0,87	1,01	0,95	1,16	1,06	1,10	0,93	far-red impaired responsive protein, putative
262149_at	At1g52530	1,04	1,12	0,92	0,87	1,02	1,04	1,00	0,94	expressed protein
262158_at	At1g52540	1,01	1,09	1,12	1,09	0,93	0,94	1,09	1,25	protein kinase, putative
262127_at	At1g52550	0,95	1,13	1,07	0,85	0,67	1,21	1,13	1,02	expressed protein
262148_at	At1g52560	1,05	1,04	0,97	1,17	1,03	1,15	1,32	1,28	26.5 kDa class I small heat shock protein-like (HSP26.5-P)
262147_at	At1g52570	0,95	1,02	0,89	0,92	1,01	0,98	0,99	0,81	phospholipase D alpha 2 / PLD alpha 2 (PLDALPHA2) (PLD2) / choline phc
262146_at	At1g52580	0,97	0,95	1,08	0,90	1,08	1,05	1,03	1,00	rhomboid family protein
262160_at	At1g52590	1,16	0,98	0,89	1,11	0,96	1,20	1,11	1,03	expressed protein
262161_at	At1g52600	1,19	0,96	0,88	1,30	1,24	1,00	0,96	0,95	signal peptidase, putative
262139_at	At1g52610	0,97	1,01	0,91	0,89	0,99	0,92	1,02	1,06	Mutator-like transposase family
262144_at	At1g52620	0,93	1,00	0,86	0,98	0,96	1,00	1,01	1,09	pentatricopeptide (PPR) repeat-containing protein
262143_at	At1g52630	0,89	0,98	0,81	0,95	1,02	0,95	0,87	0,83	expressed protein
262142_at	At1g52640	1,03	1,13	1,06	0,82	1,00	1,04	0,85	0,94	pentatricopeptide (PPR) repeat-containing protein
262138_at	At1g52660	0,92	1,13	1,00	0,96	0,95	1,07	1,09	0,86	disease resistance protein, putative
262157_at	At1g52670	0,86	0,71	0,71	0,94	0,93	0,97	0,83	0,86	biotin/lipoyl attachment domain-containing protein
262156_at	At1g52680	0,96	0,94	0,90	0,88	1,27	1,13	1,03	1,09	late embryogenesis abundant protein-related / LEA protein-related
262128_at	At1g52690	0,18	0,17	0,14	2,03	1,88	1,13	1,14	1,00	late embryogenesis abundant protein, putative / LEA protein, putative
262154_at	At1g52700	1,45	1,16	1,03	1,25	1,28	1,29	1,30	1,20	phospholipase/carboxylesterase family protein
262153_at	At1g52710	0,77	0,55	0,71	0,89	1,21	1,02	1,01	1,14	cytochrome c oxidase-related
262159_at	At1g52720	1,00	1,08	1,10	1,89	2,01	0,89	0,88	0,86	expressed protein
262145_at	At1g52730	1,18	0,93	0,91	1,05	0,99	1,07	1,02	1,02	transducin family protein / WD-40 repeat family protein

260154_at	At1g52740	1,10	1,09	1,12	1,17	1,09	0,96	1,00	1,05	histone H2A, putative
260153_at	At1g52760	1,37	1,22	1,68	1,48	1,28	0,97	1,20	1,19	esterase/lipase/thioesterase family protein
260146_at	At1g52770	1,06	0,94	0,87	0,77	0,94	1,11	0,93	0,85	phototropic-responsive NPH3 family protein
260147_at	At1g52790	1,00	1,00	0,96	1,07	1,07	1,04	0,89	0,91	oxidoreductase, 2OG-Fe(II) oxygenase family protein
260148_at	At1g52800	1,00	1,03	1,00	1,05	1,02	1,05	0,94	1,07	oxidoreductase, 2OG-Fe(II) oxygenase family protein
260149_at	At1g52810	1,04	1,02	0,99	1,04	0,94	1,00	0,93	1,12	2-oxoglutarate-dependent dioxygenase-related
260150_at	At1g52820	1,05	1,02	1,18	1,03	1,08	0,99	0,99	0,95	2-oxoglutarate-dependent dioxygenase, putative
260152_at	At1g52830	1,14	1,30	1,17	0,96	1,11	1,22	1,19	1,17	auxin-responsive protein / indoleacetic acid-induced protein 6 (IAA6)
260202_at	At1g52840	1,00	1,02	0,98	1,07	1,09	1,02	1,09	0,97	hypothetical protein
260155_at	At1g52870	1,21	1,24	1,28	0,58	0,55	1,52	1,74	1,78	peroxisomal membrane protein-related
260156_at	At1g52880	0,74	0,87	0,83	0,82	1,17	0,95	0,81	1,23	no apical meristem (NAM) family protein
260203_at	At1g52890	0,88	1,77	1,31	1,86	1,33	1,00	1,43	1,31	no apical meristem (NAM) family protein
260204_at	At1g52900	1,02	1,14	1,10	1,00	0,97	1,04	1,09	1,06	disease resistance protein (TIR class), putative
260151_at	At1g52910	1,08	1,08	0,89	1,47	1,19	0,86	0,87	0,81	expressed protein
260145_at	At1g52920	1,05	1,01	0,92	1,08	1,08	1,00	1,08	0,93	lanthionine synthetase C-like family protein
260157_at	At1g52930	1,29	1,01	0,93	1,22	1,16	0,99	0,98	0,90	brix domain-containing protein
261341_s_at	At1g52940	0,92	1,09	0,98	1,10	1,01	0,92	1,05	1,10	calcineurin-like phosphoesterase family protein
261342_at	At1g52950	0,96	1,12	1,05	0,96	1,01	1,05	0,96	1,02	replication protein-related
261313_at	At1g52970	1,00	1,02	1,12	1,01	0,90	1,06	1,09	0,84	hypothetical protein
261314_at	At1g52980	1,12	0,99	1,13	1,07	1,03	1,02	0,97	1,04	GTP-binding family protein
261374_at	At1g52990	1,12	1,00	0,94	1,24	1,15	1,11	0,92	1,16	thioredoxin family protein
261373_at	At1g53000	0,93	0,85	0,76	1,10	1,14	0,76	0,85	0,86	cytidylyltransferase family
261372_at	At1g53010	0,99	1,09	1,00	0,95	1,08	1,09	0,94	0,95	zinc finger (C3HC4-type RING finger) family protein
261371_at	At1g53020	1,05	0,97	1,09	1,12	1,06	0,96	0,86	1,07	ubiquitin-conjugating enzyme family protein
261317_at	At1g53030	1,21	1,16	1,08	1,44	1,75	1,10	1,03	0,77	cytochrome c oxidase copper chaperone family protein
261318_at	At1g53035	0,91	1,10	1,07	0,79	0,91	1,01	0,71	0,60	expressed protein
261370_at	At1g53050	0,97	0,84	0,89	0,81	0,82	0,99	0,97	1,14	protein kinase family protein
261369_at	At1g53060	1,02	1,06	1,09	0,94	1,10	0,78	0,87	0,91	legume lectin family protein
261368_at	At1g53070	1,51	1,32	1,23	1,83	1,62	0,94	0,83	0,81	legume lectin family protein
261367_at	At1g53080	1,13	1,03	0,91	0,96	0,83	0,91	0,87	0,82	legume lectin family protein
261319_at	At1g53090	1,25	1,73	1,90	0,72	0,74	1,34	1,55	1,23	WD-40 repeat family protein / phytochrome A-related
261366_at	At1g53100	0,73	0,95	0,88	0,74	0,78	0,99	0,99	1,00	glycosyltransferase family 14 protein / core-2/I-branching enzyme family prc
261365_at	At1g53110	0,97	0,93	1,00	1,04	0,87	0,93	1,05	1,01	expressed protein
261320_at	At1g53120	1,00	0,84	0,83	0,78	0,69	1,15	1,04	0,99	RNA-binding S4 domain-containing protein
261364_at	At1g53140	1,12	1,16	1,24	1,33	1,29	0,92	0,97	0,82	dynamin family protein
261375_at	At1g53160	1,00	1,08	1,20	0,97	0,96	0,88	1,13	0,97	squamosa promoter-binding protein-like 4 (SPL4)
261316_at	At1g53165	0,90	0,84	0,98	0,99	1,08	1,02	1,10	1,51	protein kinase, putative
261315_at	At1g53170	0,80	0,97	0,98	0,76	0,70	1,01	1,00	0,85	ethylene-responsive element-binding factor 8 / ERF transcription factor 8 (E
260639_at	At1g53180	1,23	1,37	1,05	1,04	1,47	1,08	1,66	1,70	expressed protein
260641_at	At1g53200	0,91	1,08	0,99	0,84	0,85	0,99	1,06	1,20	expressed protein
260587_at	At1g53210	0,88	0,81	0,83	1,12	0,95	1,00	1,03	0,98	sodium/calcium exchanger family protein / calcium-binding EF hand family [illegible]
260618_at	At1g53230	0,88	0,86	0,82	0,74	0,86	0,90	0,79	0,79	TCP family transcription factor 3 (TCP3)
260615_at	At1g53240	1,08	0,92	0,86	1,05	0,89	1,00	0,91	0,88	malate dehydrogenase (NAD), mitochondrial
260642_at	At1g53260	0,94	0,91	0,91	0,99	1,15	1,10	1,00	0,96	hypothetical protein
260643_at	At1g53270	1,11	1,09	1,02	0,95	0,99	1,03	0,93	0,80	ABC transporter family protein
260616_at	At1g53280	0,95	0,90	0,84	0,85	0,97	1,03	1,10	1,08	DJ-1 family protein

260644_at	At1g53290	0,83	0,86	0,99	0,87	1,02	0,82	0,85	0,92	galactosyltransferase family protein
260645_at	At1g53300	0,92	0,88	0,90	1,03	1,14	0,86	0,89	1,02	thioredoxin family protein
260590_at	At1g53310	1,04	0,95	1,05	1,46	1,49	1,08	1,06	0,95	phosphoenolpyruvate carboxylase, putative / PEP carboxylase, putative (PI
260588_at	At1g53320	0,94	0,92	0,92	0,89	0,83	0,92	0,90	1,07	F-box family protein / tubby family protein (TULP7)
260591_at	At1g53325	0,88	0,94	0,94	0,95	1,02	1,02	0,99	1,04	F-box family protein-related
260646_at	At1g53340	1,07	0,79	0,96	1,11	1,18	1,06	0,90	0,99	DC1 domain-containing protein
260617_at	At1g53345	0,92	0,97	0,98	1,03	0,95	0,86	1,08	1,07	expressed protein
260640_at	At1g53350	1,10	0,94	0,92	0,69	0,90	1,04	1,09	0,98	disease resistance protein (CC-NBS-LRR class), putative
260612_at	At1g53360	1,01	1,05	0,98	1,05	1,04	1,00	1,01	1,06	F-box family protein-related
260613_at	At1g53380	1,03	1,20	0,91	1,18	1,05	0,98	0,91	0,91	expressed protein
260614_at	At1g53390	0,92	1,03	0,93	1,04	0,88	0,94	0,85	0,82	ABC transporter family protein
260589_at	At1g53400	1,20	0,99	1,11	0,95	1,36	1,10	1,01	1,07	expressed protein
260977_at	At1g53420	0,96	0,98	0,95	1,02	1,00	0,95	1,05	0,98	serine/threonine protein kinase-related
260975_at	At1g53430	0,81	0,81	1,67	0,72	0,68	1,03	1,45	2,36	leucine-rich repeat family protein / protein kinase family protein
260974_at	At1g53440	1,11	1,06	2,69	0,84	1,04	1,01	1,59	2,68	leucine-rich repeat family protein / protein kinase family protein
260989_at	At1g53450	1,19	1,45	1,45	0,59	0,59	0,91	1,27	0,75	expressed protein
260981_at	At1g53460	1,53	1,70	2,62	1,11	1,33	1,04	1,15	1,32	expressed protein
260973_at	At1g53490	0,99	0,92	0,81	1,04	1,21	1,25	1,09	1,08	bZIP protein
260985_at	At1g53500	0,97	0,86	1,00	1,29	1,35	0,98	0,85	1,06	NAD-dependent epimerase/dehydratase family protein
260979_at	At1g53510	1,17	1,19	1,29	0,89	1,01	1,14	1,15	1,20	mitogen-activated protein kinase, putative / MAPK, putative (MPK18)
260982_at	At1g53520	0,89	0,79	0,70	0,90	0,88	0,73	0,73	0,65	chalcone-flavanone isomerase-related
260972_at	At1g53530	1,12	1,09	0,90	1,18	1,08	1,09	0,82	0,70	signal peptidase I family protein
260978_at	At1g53540	3,19	3,04	3,51	1,25	1,95	1,30	1,37	1,23	17.6 kDa class I small heat shock protein (HSP17.6C-CI) (AA 1-156)
260983_at	At1g53560	0,67	0,67	0,71	1,15	0,95	1,00	0,86	0,98	expressed protein
260988_at	At1g53570	0,89	0,89	1,15	1,15	0,97	0,99	1,14	1,22	mitogen-activated protein kinase kinase kinase (MAPKKK), putative (MAP3
260986_at	At1g53580	0,82	0,96	1,01	0,94	0,87	0,98	0,98	1,13	hydroxyacylglutathione hydrolase, putative / glyoxalase II, putative
260987_at	At1g53590	0,94	0,85	1,17	0,97	0,88	1,13	1,28	1,57	C2 domain-containing protein
260980_at	At1g53600	0,86	1,02	0,97	0,92	0,98	0,97	1,04	1,07	pentatricopeptide (PPR) repeat-containing protein
260971_at	At1g53620	0,93	0,97	1,10	1,02	1,10	1,08	1,08	1,07	glycine-rich protein
260970_at	At1g53640	0,98	0,95	1,11	0,97	1,07	1,05	1,06	1,07	hypothetical protein
260984_at	At1g53645	1,18	1,06	1,06	1,07	1,11	0,97	0,91	0,84	hydroxyproline-rich glycoprotein family protein
260976_at	At1g53650	0,97	1,07	1,15	0,93	0,89	0,99	0,91	1,05	RNA-binding protein, putative
259963_at	At1g53660	1,06	1,14	0,97	1,03	1,06	1,06	0,95	0,95	phosphate translocator-related
259965_at	At1g53670	1,21	1,21	1,07	1,10	0,90	1,14	1,05	1,00	transcription factor-related
259964_at	At1g53680	0,92	0,89	0,85	1,07	0,97	0,96	0,92	1,12	glutathione S-transferase, putative
259962_at	At1g53690	1,04	1,09	0,98	1,10	1,05	1,04	1,19	1,17	DNA-directed RNA polymerases I, II, and III 7 kDa subunit, putative
259961_at	At1g53700	1,02	1,02	1,02	0,98	1,19	0,88	0,98	1,01	protein kinase, putative
259960_at	At1g53710	0,82	0,92	0,81	0,95	0,89	0,99	0,83	1,15	calcineurin-like phosphoesterase family protein
259959_at	At1g53720	1,21	1,11	1,12	0,86	1,05	1,09	1,01	1,00	cyclophilin-RNA interacting protein, putative
259958_at	At1g53730	1,07	1,03	1,07	1,15	1,20	0,92	0,97	1,10	leucine-rich repeat transmembrane protein kinase, putative
262227_s_at	At1g53750	1,02	0,97	1,03	0,98	0,97	1,03	1,05	1,08	26S proteasome AAA-ATPase subunit (RPT1a)
262251_at	At1g53760	0,96	0,90	0,98	1,08	0,92	0,94	0,92	0,91	expressed protein
257470_at	At1g53770	0,86	0,91	0,88	1,07	1,29	0,87	0,80	1,16	expressed protein
262255_at	At1g53790	1,21	1,01	1,11	1,24	1,20	1,04	1,10	1,20	F-box family protein
262199_at	At1g53800	0,93	0,95	1,04	0,72	0,86	1,20	1,02	1,35	expressed protein
262256_at	At1g53810	0,95	0,95	0,93	1,07	1,10	0,97	0,97	0,93	---

262252_at	At1g53820	1,03	1,14	1,01	0,93	1,10	0,95	1,07	0,95	zinc finger (C3HC4-type RING finger) family protein
262198_at	At1g53830	1,48	1,14	1,17	1,39	1,13	1,14	1,01	1,10	pectinesterase family protein
262225_at	At1g53840	0,77	0,78	1,01	1,00	1,00	1,01	0,99	1,06	pectinesterase family protein
262258_at	At1g53850	0,91	0,84	0,87	1,24	1,07	0,92	0,90	0,91	20S proteasome alpha subunit E1 (PAE1)
262257_at	At1g53860	0,84	1,00	0,93	0,94	0,96	1,06	1,07	0,98	remorin family protein
262259_s_at	At1g53870	0,65	1,32	1,43	0,80	0,78	1,42	1,22	1,09	expressed protein
262253_s_at	At1g53880	1,48	1,26	1,33	1,06	1,00	1,02	1,00	1,08	eukaryotic translation initiation factor 2B family protein / eIF-2B family prote
262226_at	At1g53885	0,60	1,16	0,63	0,80	0,94	0,87	0,79	0,76	senescence-associated protein-related
262197_at	At1g53910	0,77	0,66	0,81	0,86	0,84	0,70	0,67	0,67	AP2 domain-containing protein RAP2.12 (RAP2.12)
262254_at	At1g53920	0,65	0,68	0,54	0,98	0,73	0,99	0,79	0,81	GDSL-motif lipase/hydrolase family protein
263197_at	At1g53930	1,01	0,98	0,91	1,07	1,21	1,01	0,96	1,22	ubiquitin family protein
263146_at	At1g53940	0,94	0,87	1,01	0,91	1,29	1,02	0,99	0,92	GDSL-motif lipase/hydrolase family protein
263148_at	At1g53950	1,07	0,96	1,01	0,93	1,16	1,01	0,91	1,12	ubiquitin family protein
263149_at	At1g53970	0,96	0,96	1,03	1,08	1,06	0,99	0,95	1,02	hypothetical protein
263147_at	At1g53980	1,02	1,01	0,95	0,95	1,13	0,90	1,08	0,90	polyubiquitin-related
263198_at	At1g53990	1,01	1,03	1,12	0,82	0,70	1,09	1,19	1,25	GDSL-motif lipase/hydrolase family protein
263153_s_at	At1g54010	1,21	0,89	0,78	1,29	1,27	0,78	0,78	0,71	myrosinase-associated protein, putative
263161_at	At1g54020	1,06	1,01	0,96	0,98	0,91	1,05	0,76	0,70	myrosinase-associated protein, putative
263156_at	At1g54030	1,08	0,84	0,83	1,33	1,06	1,01	0,94	0,93	GDSL-motif lipase, putative
263174_at	At1g54040	1,30	1,19	1,15	1,33	1,17	0,88	1,02	0,84	kelch repeat-containing protein
263150_at	At1g54050	3,46	3,16	1,73	1,55	1,70	1,17	0,97	0,94	17.4 kDa class III heat shock protein (HSP17.4-CIII)
263152_at	At1g54060	0,73	0,72	0,91	0,90	1,09	0,87	0,85	0,82	expressed protein
263144_at	At1g54070	0,99	0,86	0,94	0,97	1,05	0,99	0,95	0,92	dormancy/auxin associated protein-related
263160_at	At1g54080	0,99	0,88	0,84	1,06	1,08	0,84	0,89	1,04	oligouridylate-binding protein, putative
263145_at	At1g54090	0,73	0,60	0,74	1,00	1,00	0,79	0,71	0,76	exocyst subunit EXO70 family protein
263157_at	At1g54100	0,59	0,61	0,69	0,99	0,84	0,87	0,91	0,95	aldehyde dehydrogenase, putative / antiquitin, putative
263154_at	At1g54110	1,33	1,42	1,56	0,85	1,02	1,23	1,01	1,19	cation exchanger, putative (CAX10)
263171_at	At1g54115	1,07	0,88	0,88	0,98	1,03	0,93	0,99	0,88	cation exchanger, putative
263151_at	At1g54120	0,83	0,94	0,87	1,06	1,36	0,96	0,98	1,02	expressed protein
263159_at	At1g54130	1,04	0,99	0,86	0,77	0,84	1,03	1,09	1,24	RelA/SpoT protein, putative (RSH3)
263155_at	At1g54140	1,08	1,19	1,50	1,04	1,17	1,15	1,07	1,04	transcription initiation factor IID (TFIID) 31 kDa subunit (TAFII-31) family pro
263162_at	At1g54150	0,88	0,83	1,15	1,07	1,02	1,04	1,04	1,47	zinc finger (C3HC4-type RING finger) family protein
263158_at	At1g54160	0,80	0,78	0,98	0,86	1,18	0,90	0,72	0,89	CCAAT-binding transcription factor (CBF-B/NF-YA) family protein
263172_at	At1g54170	1,08	0,90	1,10	0,89	1,03	1,07	1,10	1,66	ataxin-2-related
263173_at	At1g54180	1,02	1,20	1,02	1,00	1,17	0,94	1,04	0,89	expressed protein
262991_at	At1g54190	0,89	1,03	0,90	1,01	1,05	1,04	0,97	0,89	zinc finger protein-related
263002_at	At1g54200	1,10	1,11	1,06	1,17	0,92	1,19	0,98	1,07	expressed protein
262992_at	At1g54210	1,16	1,19	1,30	0,85	0,93	1,30	1,30	1,40	autophagy 12a (APG12a)
262963_at	At1g54220	0,97	0,96	0,70	1,15	0,92	1,07	0,96	1,01	dihydrolipoamide S-acetyltransferase, putative
262993_at	At1g54230	0,99	1,00	0,99	1,13	0,98	1,01	1,04	0,90	hypothetical protein
263006_at	At1g54240	0,91	0,99	1,01	0,98	1,01	0,93	1,06	1,04	hypothetical protein
262957_s_at	At1g54250	1,51	1,27	1,19	1,37	1,20	1,00	1,01	1,01	DNA-directed RNA polymerase I, II, and III, putative
263007_at	At1g54260	1,08	0,85	0,82	1,16	1,10	1,03	1,11	1,34	histone H1/H5 family protein
262956_at	At1g54270	1,01	0,89	0,90	1,00	0,93	0,97	0,96	1,01	eukaryotic translation initiation factor 4A-2 / eIF-4A-2
262998_at	At1g54280	0,96	0,82	0,92	0,99	1,04	1,15	1,07	0,97	haloacid dehalogenase-like hydrolase family protein
262959_at	At1g54290	0,90	0,90	0,93	1,03	1,14	1,04	0,98	0,97	eukaryotic translation initiation factor SUI1, putative

262965_at	At1g54310	0,92	1,01	0,99	0,74	0,81	1,21	0,92	0,98	expressed protein
262960_at	At1g54320	0,78	0,76	0,78	1,00	1,10	0,96	0,92	0,84	LEM3 (ligand-effect modulator 3) family protein / CDC50 family protein
262999_at	At1g54330	0,98	0,91	1,03	0,99	1,02	0,98	0,93	1,03	no apical meristem (NAM) family protein
262962_at	At1g54340	0,98	0,98	1,01	0,96	1,33	1,06	0,97	1,04	isocitrate dehydrogenase, putative / NADP+ isocitrate dehydrogenase, puta
263000_at	At1g54350	1,04	1,00	1,17	0,79	0,87	1,25	1,26	1,38	ABC transporter family protein
263001_at	At1g54360	0,95	1,04	0,93	0,75	0,92	1,04	1,07	0,94	expressed protein
263009_at	At1g54370	0,76	0,66	0,63	0,95	0,82	0,94	0,94	1,01	sodium proton exchanger, putative (NHX5)
262964_at	At1g54380	1,09	0,93	0,83	0,95	0,99	0,97	0,92	0,96	spliceosome protein-related
262982_at	At1g54390	1,02	0,87	0,93	1,10	0,98	0,97	1,02	1,21	PHD finger protein-related
262958_at	At1g54410	1,09	1,08	1,15	1,20	1,34	1,18	1,19	1,34	dehydrin family protein
262994_at	At1g54420	0,93	0,96	0,98	0,97	1,06	1,06	0,96	1,08	hypothetical protein
262995_s_at	At1g54430	0,97	1,00	1,00	0,91	1,09	0,96	1,05	0,97	hypothetical protein
262996_at	At1g54440	0,94	0,93	1,36	0,76	0,86	1,09	0,98	1,04	3'-5' exonuclease domain-containing protein / helicase and RNase D C-tern
263003_at	At1g54450	1,09	0,90	1,00	1,07	1,18	0,92	0,95	0,84	calcium-binding EF-hand family protein
262984_at	At1g54460	1,21	0,97	1,10	1,42	1,59	1,10	1,05	1,21	expressed protein
263008_at	At1g54470	0,95	1,05	0,97	1,00	0,99	0,95	1,04	1,00	hypothetical protein
262997_at	At1g54480	0,91	1,14	0,95	0,99	1,03	0,98	1,02	1,03	leucine-rich repeat family protein
262961_at	At1g54490	0,87	0,81	1,08	0,83	0,92	0,97	1,06	1,01	5'-3' exoribonuclease (XRN4)
262954_at	At1g54500	1,18	1,24	1,17	0,99	0,78	1,13	0,88	0,82	rubredoxin family protein
263004_at	At1g54510	1,16	1,23	1,63	0,91	0,97	0,95	1,33	1,10	protein kinase family protein
262955_at	At1g54520	0,86	0,79	0,86	0,67	0,72	1,18	1,16	1,19	expressed protein
263005_at	At1g54540	1,11	1,27	0,96	1,24	0,98	1,09	0,96	0,97	hypothetical protein
262983_at	At1g54550	0,81	1,00	1,00	0,99	1,04	1,00	0,97	0,88	F-box family protein
264235_at	At1g54560	0,87	0,97	1,15	1,08	1,23	0,95	1,06	1,06	myosin, putative
264186_at	At1g54570	0,77	0,69	0,69	0,78	0,77	1,15	1,09	1,03	esterase/lipase/thioesterase family protein
264193_at	At1g54610	1,08	1,20	1,04	1,07	0,99	1,05	1,03	1,12	protein kinase family protein
264189_s_at	At1g54630	1,01	0,81	0,76	1,22	1,38	0,83	0,83	0,80	acyl carrier protein, chloroplast, putative / ACP, putative
264242_at	At1g54640	0,99	1,03	0,93	0,98	1,05	0,99	1,01	0,98	F-box family protein-related
264243_at	At1g54650	1,30	1,34	1,72	1,19	0,97	0,96	1,08	1,14	expressed protein
264236_at	At1g54680	0,77	0,77	1,06	0,84	0,98	0,92	0,95	1,08	expressed protein
264188_at	At1g54690	1,32	1,21	1,29	1,48	1,65	1,00	0,94	0,91	histone H2A, putative
264237_at	At1g54700	1,01	0,94	0,95	1,02	1,07	1,05	0,95	0,93	hypothetical protein
264192_at	At1g54710	1,05	1,04	0,82	0,84	1,00	1,14	0,98	0,87	expressed protein
264234_at	At1g54720	0,94	1,08	1,05	0,98	1,02	0,89	1,04	1,17	early-responsive to dehydration protein-related / ERD protein-related
264191_at	At1g54730	0,92	0,90	1,12	0,85	0,77	1,07	1,33	1,32	sugar transporter, putative
264238_at	At1g54740	1,62	1,81	1,60	1,07	0,94	1,13	1,10	1,70	expressed protein
264239_at	At1g54770	0,96	0,88	0,92	0,83	1,10	0,93	0,94	0,84	expressed protein
264185_at	At1g54780	1,12	1,07	1,15	1,09	0,89	1,08	1,00	1,01	thylakoid lumen 18.3 kDa protein
264184_at	At1g54790	1,24	1,03	1,03	1,33	1,49	0,95	0,91	0,89	GDSL-motif lipase/hydrolase family protein
264240_at	At1g54820	0,93	1,12	1,17	0,52	0,58	1,21	1,24	1,09	protein kinase family protein
264190_at	At1g54830	1,27	1,33	1,21	1,01	1,04	1,06	0,95	1,15	CCAAT-box binding transcription factor Hap5a, putative
264241_at	At1g54840	0,94	0,99	0,94	1,08	1,08	1,00	0,96	1,05	expressed protein
257426_at	At1g54850	1,20	1,13	1,09	0,85	0,72	0,99	0,90	0,86	expressed protein
264187_at	At1g54860	0,87	0,98	0,86	0,90	0,93	0,85	1,08	1,14	expressed protein
256354_at	At1g54870	0,33	0,38	0,26	0,82	0,50	0,99	1,05	0,98	short-chain dehydrogenase/reductase (SDR) family protein
256348_at	At1g54880	0,93	1,09	0,99	0,97	1,04	0,99	1,05	1,08	hypothetical protein

256349_at	At1g54890	1,06	1,11	1,10	1,43	1,38	1,14	1,35	0,83	late embryogenesis abundant protein-related / LEA protein-related
256323_at	At1g54920	0,69	0,69	0,59	0,75	0,77	0,95	0,76	0,76	expressed protein
256347_at	At1g54923	1,01	1,05	1,01	1,15	1,03	1,01	1,13	0,91	hypothetical protein
256346_s_at	At1g54926	1,00	1,02	1,03	1,02	1,02	0,99	0,88	1,09	hypothetical protein
256350_at	At1g54940	0,96	0,97	1,02	1,02	0,97	1,15	1,18	0,87	glycogenin glucosyltransferase (glycogenin)-related
257584_at	At1g54955	0,96	0,97	1,17	1,00	1,02	1,08	1,03	1,00	hypothetical protein
256351_at	At1g54960	1,10	1,13	1,15	0,98	1,33	0,84	0,91	1,23	NPK1-related protein kinase, putative (ANP2)
256352_at	At1g54970	1,12	1,03	0,98	1,16	1,26	0,96	0,87	1,03	proline-rich family protein
256322_at	At1g54990	0,84	0,87	0,72	0,85	1,00	0,91	0,90	0,84	expressed protein
256353_at	At1g55000	0,90	1,03	1,05	0,78	0,90	1,02	1,03	1,19	peptidoglycan-binding LysM domain-containing protein
256321_at	At1g55020	1,66	1,79	1,69	1,11	1,20	1,68	2,69	3,98	lipoxygenase (LOX1)
256345_at	At1g55030	0,91	0,98	0,97	0,95	1,02	1,03	0,96	0,97	F-box family protein
256355_at	At1g55040	0,99	0,97	0,91	0,91	0,92	0,90	1,07	1,05	zinc finger (Ran-binding) family protein
257589_at	At1g55050	1,06	0,95	0,97	0,95	1,11	0,97	1,01	1,05	expressed protein
256153_at	At1g55060	0,92	1,05	1,02	0,88	1,05	1,06	0,98	1,00	polyubiquitin (UBQ12)
256147_at	At1g55080	1,07	0,89	0,96	0,93	1,10	1,21	1,09	1,20	expressed protein
256148_at	At1g55090	0,92	0,92	1,01	0,84	0,82	0,96	0,94	1,04	carbon-nitrogen hydrolase family protein
256149_at	At1g55110	0,83	1,16	0,97	1,04	1,20	1,23	1,16	1,20	zinc finger (C2H2 type) family protein
256150_at	At1g55120	0,89	0,86	0,74	1,13	0,86	0,78	0,89	0,69	beta-fructosidase, putative / beta-fructofuranosidase, putative
256151_at	At1g55130	0,98	0,89	0,96	0,95	0,92	0,94	0,85	0,83	endomembrane protein 70, putative
256152_at	At1g55150	1,13	1,05	1,26	0,93	0,98	1,06	0,99	1,13	DEAD box RNA helicase, putative (RH20)
259665_at	At1g55160	1,02	0,90	0,79	1,14	1,15	0,93	0,85	0,93	expressed protein
259659_at	At1g55170	0,94	1,00	0,87	1,00	0,79	1,00	0,95	0,81	expressed protein
259657_at	At1g55180	1,07	1,26	1,13	1,08	1,18	1,05	1,03	0,96	phospholipase D, putative (PLDEPSILON)
259663_at	At1g55190	1,04	0,83	0,69	0,95	1,08	0,88	0,78	0,78	prenylated rab acceptor (PRA1) family protein
259656_at	At1g55200	0,98	0,89	0,99	0,89	1,02	1,12	1,01	1,01	protein kinase family protein
259655_at	At1g55210	1,17	0,98	0,79	2,07	1,87	0,86	0,81	0,85	disease resistance response protein-related/ dirigent protein-related
259654_at	At1g55220	1,02	1,01	0,95	0,89	1,14	1,09	0,89	0,89	hypothetical protein
259653_at	At1g55240	1,00	1,14	0,97	0,94	1,10	1,43	1,69	1,76	expressed protein
259652_at	At1g55250	1,17	1,08	1,25	1,00	0,99	1,03	1,07	1,14	expressed protein
259662_at	At1g55255	1,23	1,39	1,56	0,94	1,04	1,19	1,34	1,69	zinc finger (C3HC4-type RING finger) family protein
259660_at	At1g55260	1,57	1,46	1,46	1,64	1,65	0,99	0,80	0,81	protease inhibitor/seed storage/lipid transfer protein (LTP) family protein
259661_at	At1g55265	0,76	0,77	0,80	2,23	2,02	0,70	0,66	0,65	expressed protein
259667_at	At1g55270	0,75	1,06	1,25	0,93	0,96	1,11	1,22	0,93	kelch repeat-containing F-box family protein
259651_at	At1g55280	0,93	1,28	1,33	0,75	0,84	1,02	0,91	0,93	expressed protein
259650_at	At1g55290	0,94	0,93	1,22	1,08	1,26	1,09	0,92	0,98	oxidoreductase, 2OG-Fe(II) oxygenase family protein
259649_at	At1g55300	1,39	1,43	1,30	1,56	1,40	1,09	0,94	1,11	TATA-binding protein-associated factor TAFII55 family protein
259666_at	At1g55310	1,03	1,34	1,26	1,08	0,82	0,87	1,10	1,07	SC35-like splicing factor, 33 kD (SCL33)
259648_at	At1g55315	1,00	1,07	0,89	0,89	1,17	0,87	0,96	0,96	---
259647_at	At1g55320	1,11	1,22	1,74	0,99	0,93	1,17	1,27	1,24	expressed protein
259664_at	At1g55330	1,09	1,00	0,96	1,12	1,10	0,96	0,96	0,90	arabinogalactan-protein (AGP21)
259646_at	At1g55350	0,93	1,01	1,48	0,70	0,70	1,00	1,18	1,37	calpain-type cysteine protease family
257510_at	At1g55360	0,83	0,86	0,82	1,63	1,59	0,98	0,81	0,78	expressed protein
259658_at	At1g55370	0,95	1,05	1,06	1,04	1,05	1,08	1,09	1,22	expressed protein
265126_s_at	At1g55380	1,09	0,97	0,96	1,02	0,91	1,14	1,03	1,08	DC1 domain-containing protein
256162_at	At1g55390	1,03	1,05	0,90	1,20	1,05	1,09	0,90	0,93	DC1 domain-containing protein

265125_at	At1g55410	1,01	1,02	1,10	0,83	0,94	1,04	1,03	1,11	---
265124_at	At1g55430	1,22	1,28	0,96	1,01	1,14	1,10	1,01	1,01	DC1 domain-containing protein
265123_at	At1g55440	0,98	1,00	0,94	1,09	0,88	0,97	0,98	1,22	DC1 domain-containing protein
265075_at	At1g55450	0,73	0,88	2,00	0,77	0,87	1,04	1,10	1,54	embryo-abundant protein-related
265079_at	At1g55460	1,00	0,84	0,89	0,80	0,88	0,96	0,95	1,45	Kin17 DNA-binding protein-related
265073_at	At1g55480	1,15	1,17	1,15	0,94	0,87	1,44	1,32	1,31	expressed protein
265076_at	At1g55490	0,98	0,87	0,88	0,71	0,66	1,11	1,13	1,14	RuBisCO subunit binding-protein beta subunit, chloroplast / 60 kDa chapero
265078_at	At1g55500	0,76	0,60	0,70	0,79	0,77	1,21	0,91	0,92	expressed protein
265070_at	At1g55510	1,02	1,32	1,61	0,87	0,77	1,07	1,21	1,45	2-oxoisovalerate dehydrogenase, putative / 3-methyl-2-oxobutanoate dehyd
265071_at	At1g55520	1,67	1,22	1,55	1,21	1,43	1,07	1,13	1,22	transcription initiation factor IID-2 (TFIID-2) / TATA-box factor 2 / TATA seq
265077_at	At1g55530	1,10	1,20	1,16	1,21	1,12	1,13	1,07	1,14	zinc finger (C3HC4-type RING finger) family protein
265074_at	At1g55540	0,92	0,87	0,94	1,11	0,83	0,99	0,98	0,72	proline-rich family protein
265069_at	At1g55545	0,90	1,14	1,06	0,94	1,07	0,99	0,95	0,79	nucleoporin-related
265068_at	At1g55550	0,91	1,07	0,97	0,96	1,26	0,99	0,99	0,97	kinesin motor protein-related
265127_at	At1g55560	1,01	0,95	0,95	0,95	1,15	1,01	1,04	1,02	multi-copper oxidase type I family protein
265080_at	At1g55570	1,04	0,92	0,96	0,91	1,12	1,06	1,04	0,97	multi-copper oxidase type I family protein
265072_at	At1g55580	0,96	1,09	1,13	1,04	1,13	1,11	0,96	1,05	scarecrow transcription factor family protein
264539_at	At1g55590	0,75	0,64	0,90	1,04	1,02	0,90	1,01	1,17	F-box family protein
264538_at	At1g55600	0,89	1,06	0,87	1,00	1,02	0,98	0,92	1,05	WRKY family transcription factor
264537_at	At1g55610	0,89	0,95	0,87	0,80	0,86	1,30	1,03	1,04	protein kinase family protein
264547_at	At1g55620	0,85	0,72	0,94	1,07	1,33	1,11	0,77	0,77	voltage-gated chloride channel family protein
264540_at	At1g55630	0,91	0,92	0,96	1,11	0,88	1,04	0,97	0,94	pentatricopeptide (PPR) repeat-containing protein
264536_at	At1g55640	0,94	1,24	0,94	1,10	1,07	0,98	0,93	0,94	prenylated rab acceptor (PRA1) family protein
264541_at	At1g55660	1,01	0,98	1,00	1,08	1,04	1,12	1,08	0,91	F-box family protein
264545_at	At1g55670	0,90	0,94	0,91	1,00	0,92	0,97	0,93	0,92	photosystem I reaction center subunit V, chloroplast, putative / PSI-G, putat
264548_at	At1g55680	1,13	1,23	1,38	0,99	1,05	1,06	1,02	1,02	WD-40 repeat family protein
264535_at	At1g55690	0,89	1,03	1,01	0,93	1,07	0,99	0,96	1,08	SEC14 cytosolic factor family protein / phosphoglyceride transfer family pro
264534_at	At1g55700	0,85	0,99	1,13	0,94	1,00	1,07	0,98	1,22	DC1 domain-containing protein
264533_s_at	At1g55730	0,89	0,84	0,85	1,03	1,15	0,85	0,99	0,94	calcium exchanger, putative
264532_at	At1g55740	0,93	1,09	1,02	0,96	0,77	1,20	1,15	1,00	alkaline alpha galactosidase, putative
264563_s_at	At1g55750	1,01	0,99	0,98	1,14	1,12	1,30	1,36	1,25	transcription factor-related
264562_at	At1g55760	1,30	1,21	1,00	1,34	1,39	0,93	0,89	0,92	BTB/POZ domain-containing protein
264543_at	At1g55780	1,07	1,24	1,11	1,26	1,06	1,05	0,96	1,16	heavy-metal-associated domain-containing protein
264544_s_at	At1g55800	1,06	0,93	1,09	1,01	1,12	1,01	0,99	1,07	hypothetical protein
264546_at	At1g55805	1,26	1,04	1,13	1,18	1,14	0,95	0,82	0,80	BolA-like family protein
264561_at	At1g55810	0,98	1,09	1,03	0,73	1,01	0,86	0,96	0,87	uracil phosphoribosyltransferase, putative / UMP pyrophosphorylase, putati
264560_at	At1g55820	0,98	1,00	1,12	1,07	0,96	0,87	0,97	1,19	hydroxyproline-rich glycoprotein family protein
264542_at	At1g55830	0,91	0,80	0,92	0,82	1,30	1,19	0,77	0,89	expressed protein
260604_at	At1g55840	0,94	1,02	1,28	1,12	1,39	0,96	0,98	1,10	SEC14 cytosolic factor (SEC14) / phosphoglyceride transfer protein
260592_at	At1g55850	0,58	0,74	0,98	0,65	0,49	0,84	1,03	1,36	cellulose synthase family protein
260593_at	At1g55870	0,97	1,02	1,09	1,04	1,08	1,01	0,96	0,98	CAF1 family ribonuclease
260594_at	At1g55880	0,88	0,89	0,87	1,11	0,99	1,01	0,97	0,89	pyridoxal-5'-phosphate-dependent enzyme, beta family protein
260595_at	At1g55890	1,43	1,30	1,40	0,91	0,96	0,93	0,90	0,78	pentatricopeptide (PPR) repeat-containing protein
260596_at	At1g55900	1,32	1,04	1,14	1,00	1,01	1,05	0,86	0,94	NLI interacting factor (NIF) family protein
260601_at	At1g55910	0,88	0,92	1,10	1,18	1,18	1,19	1,01	1,03	metal transporter, putative (ZIP11)
260597_at	At1g55915	0,93	0,91	0,98	0,94	0,92	0,91	0,88	1,20	expressed protein

260602_at	At1g55920	0,91	1,15	1,27	0,95	0,97	0,89	1,03	1,13	serine O-acetyltransferase, putative
260598_at	At1g55930	1,02	0,92	0,87	0,80	0,78	1,10	1,18	1,22	CBS domain-containing protein / transporter associated domain-containing
260599_at	At1g55940	0,98	1,04	0,98	0,94	1,03	1,22	1,01	1,02	cytochrome P450, putative
260600_at	At1g55950	0,94	1,07	0,98	0,96	1,14	0,92	1,00	0,98	hypothetical protein
260603_at	At1g55960	1,49	1,49	1,45	1,26	1,20	1,58	1,47	1,45	expressed protein
262090_at	At1g55970	0,94	0,99	1,19	0,81	1,00	0,94	0,88	0,84	histone acetyltransferase 4 (HAC4)
262089_s_at	At1g55980	0,62	0,67	0,61	0,89	0,81	0,89	0,87	0,84	expressed protein
262097_at	At1g55990	0,90	0,98	0,99	1,02	0,97	1,10	0,99	0,82	glycine-rich protein
262096_at	At1g56010	0,97	1,32	1,35	1,45	1,84	1,03	0,94	0,91	transcription activator NAC1 (NAC1)
262088_at	At1g56020	0,90	1,02	0,92	1,13	1,48	0,88	1,05	0,98	expressed protein
262087_at	At1g56030	0,93	1,08	1,02	1,07	1,08	0,96	0,98	0,98	MIF4G domain-containing protein / U-box domain-containing protein
257472_at	At1g56040	1,02	0,99	1,05	1,10	1,16	1,04	0,89	1,07	U-box domain-containing protein
262086_at	At1g56050	1,02	0,95	0,84	0,61	0,72	1,18	1,09	1,10	GTP-binding protein-related
262085_at	At1g56060	0,97	0,90	1,14	0,78	0,75	1,19	1,44	1,79	expressed protein
262064_at	At1g56075	1,26	1,12	1,06	0,97	0,83	0,99	1,03	0,95	elongation factor 2, putative / EF-2, putative
262084_at	At1g56080	1,10	1,03	0,95	1,34	1,29	0,94	1,09	0,91	expressed protein
262095_at	At1g56090	0,91	1,05	1,12	0,95	1,29	1,02	0,99	1,10	tetratricopeptide repeat (TPR)-containing protein
262083_at	At1g56100	0,94	0,98	1,02	1,01	1,03	0,95	1,08	1,10	pectinesterase inhibitor domain-containing protein
262094_at	At1g56110	1,56	1,47	1,20	1,28	1,10	1,15	1,16	1,21	nucleolar protein Nop56, putative
262082_s_at	At1g56120	0,79	0,96	1,61	0,96	0,80	1,11	1,73	2,39	leucine-rich repeat family protein / protein kinase family protein
262093_at	At1g56145	1,00	0,94	0,88	0,72	0,95	1,04	1,35	1,36	leucine-rich repeat family protein / protein kinase family protein
262092_at	At1g56150	1,55	1,36	1,20	1,24	1,18	1,20	1,04	0,92	auxin-responsive family protein
262091_at	At1g56160	1,05	1,01	1,16	1,05	1,13	1,08	0,92	1,03	myb family transcription factor (MYB72)
262098_at	At1g56170	1,31	1,34	1,24	1,54	1,40	1,59	1,27	1,09	transcription factor, putative
262065_at	At1g56180	0,90	0,95	0,98	0,91	1,29	1,13	0,99	1,10	expressed protein
256228_at	At1g56190	0,70	0,71	0,68	0,86	0,79	1,17	1,08	1,14	phosphoglycerate kinase, putative
256223_at	At1g56200	1,16	1,16	1,27	1,13	1,13	0,99	1,02	1,02	expressed protein
256222_at	At1g56210	1,04	0,96	1,11	1,04	1,11	1,23	1,06	1,16	copper chaperone (CCH)-related
256225_at	At1g56220	1,06	1,85	2,47	0,68	0,72	0,87	1,01	1,25	dormancy/auxin associated family protein
256220_at	At1g56230	1,16	1,42	1,20	0,96	0,85	1,03	1,24	1,16	expressed protein
256219_at	At1g56260	0,96	0,87	1,06	0,85	0,98	0,82	0,94	1,01	expressed protein
256218_at	At1g56270	0,99	1,05	1,01	1,16	1,04	1,10	1,10	0,92	hypothetical protein
256226_at	At1g56280	0,72	0,71	0,74	0,84	0,92	0,81	0,80	0,97	drought-responsive family protein
256227_at	At1g56290	0,99	1,23	1,07	0,94	1,03	1,03	1,01	1,15	CwfJ-like family protein
256221_at	At1g56300	0,82	0,90	1,30	0,93	0,94	1,04	0,92	1,14	DNAJ heat shock N-terminal domain-containing protein
257587_at	At1g56310	1,04	1,25	1,19	0,70	0,92	0,90	1,17	1,21	3'-5' exonuclease domain-containing protein
256217_at	At1g56320	1,10	1,35	1,06	1,02	1,28	0,90	0,84	0,98	expressed protein
256224_at	At1g56330	1,27	1,08	1,05	1,33	1,31	1,00	0,94	0,93	GTP-binding protein (SAR1B)
256216_at	At1g56340	1,42	1,24	1,13	1,38	1,31	0,91	1,02	1,02	calreticulin 1 (CRT1)
259636_at	At1g56345	0,84	0,74	1,18	1,01	1,01	0,98	0,97	1,22	pseudouridine synthase family protein
259630_at	At1g56350	1,34	1,02	1,21	1,17	1,05	1,22	0,96	1,03	peptide chain release factor, putative
259635_at	At1g56360	1,02	0,94	0,91	1,08	1,02	1,02	1,19	0,91	calcineurin-like phosphoesterase family protein
259634_at	At1g56380	1,10	1,16	1,05	0,97	1,14	1,01	0,99	1,19	mitochondrial transcription termination factor family protein / mTERF family
259631_at	At1g56410	0,98	1,03	1,05	0,87	0,85	1,02	0,89	1,27	heat shock cognate 70 kDa protein, putative / HSC70, putative / HSP70, pu
259632_at	At1g56430	2,53	2,74	1,38	2,56	2,24	1,94	1,20	0,96	nicotianamine synthase, putative
259628_at	At1g56440	1,21	1,32	1,13	1,15	1,02	0,95	0,98	0,93	serine/threonine protein phosphatase-related

259604_at	At1g56450	0,98	0,91	0,95	1,18	1,16	1,01	0,87	0,91	20S proteasome beta subunit G1 (PBG1) (PRCH)
259603_at	At1g56500	0,72	0,74	0,72	1,05	1,05	0,91	0,82	0,87	haloacid dehalogenase-like hydrolase family protein
259633_at	At1g56505	0,96	0,93	0,87	0,96	1,09	1,05	0,96	1,00	haloacid dehalogenase-like hydrolase family protein
259629_at	At1g56510	0,65	0,79	1,69	0,94	0,63	1,33	2,36	2,02	disease resistance protein (TIR-NBS-LRR class), putative
259602_at	At1g56520	0,99	1,32	1,19	0,91	1,08	1,28	1,07	1,08	disease resistance protein (TIR-NBS-LRR class), putative
245655_at	At1g56530	1,06	0,86	0,99	1,06	1,11	1,10	0,96	1,02	hydroxyproline-rich glycoprotein family protein
245654_at	At1g56540	1,04	1,03	0,92	0,96	0,88	1,59	1,46	1,17	disease resistance protein (TIR-NBS-LRR class), putative
245681_at	At1g56560	1,14	1,05	1,22	1,06	0,94	1,26	1,04	1,13	beta-fructofuranosidase, putative / invertase, putative / saccharase, putative
245680_at	At1g56570	0,96	1,05	1,23	0,79	0,98	0,97	1,12	1,07	pentatricopeptide (PPR) repeat-containing protein
245629_at	At1g56580	1,68	1,50	1,33	1,60	1,47	1,13	0,99	0,93	expressed protein
245679_at	At1g56590	1,38	1,18	1,35	1,07	1,13	0,97	0,88	0,88	clathrin adaptor complexes medium subunit family protein
245627_at	At1g56600	0,86	1,00	0,97	1,18	1,34	1,12	0,99	0,95	galactinol synthase, putative
245678_at	At1g56610	1,50	1,41	1,22	1,05	1,06	1,12	0,96	0,94	syntaxin-related family protein
245656_at	At1g56620	0,95	1,11	0,91	1,07	1,01	1,03	0,94	0,92	pectinesterase inhibitor domain-containing protein
245628_at	At1g56650	0,87	1,07	1,08	0,96	1,11	1,05	0,92	0,84	myb family transcription factor (MYB75)
245677_at	At1g56660	2,54	1,97	0,94	1,07	0,84	2,44	3,03	1,55	expressed protein
245676_at	At1g56670	0,50	0,50	0,88	0,96	1,24	0,81	1,17	1,17	GDSL-motif lipase/hydrolase family protein
245675_at	At1g56675	0,98	1,06	1,04	1,07	1,03	1,07	0,94	1,06	---
245674_at	At1g56680	0,93	1,01	0,97	1,47	1,36	1,20	1,01	0,94	glycoside hydrolase family 19 protein
245673_at	At1g56690	0,97	0,96	1,03	0,83	0,96	0,99	1,01	0,83	pentatricopeptide (PPR) repeat-containing protein
245626_at	At1g56700	1,16	1,26	1,48	1,58	1,43	0,76	0,99	1,06	pyrrolidone-carboxylate peptidase family protein
245672_at	At1g56710	0,90	0,94	1,13	0,88	1,00	1,05	1,14	0,86	glycoside hydrolase family 28 protein / polygalacturonase (pectinase) family
245657_at	At1g56720	0,83	0,86	0,95	0,93	0,89	0,99	0,99	0,81	protein kinase family protein
246400_at	At1g57540	1,13	1,13	1,06	1,20	1,17	0,90	0,88	0,92	expressed protein
246377_at	At1g57550	0,98	0,89	0,87	1,19	1,14	0,99	1,10	1,03	hydrophobic protein, putative / low temperature and salt responsive protein,
246401_at	At1g57560	1,08	1,04	1,08	1,08	1,10	1,02	0,99	1,03	myb family transcription factor (MYB50)
246402_at	At1g57570	0,94	0,83	0,97	0,99	1,15	0,97	1,03	0,98	jacalin lectin family protein
246403_at	At1g57590	0,83	0,70	0,80	1,20	1,21	0,68	0,83	0,81	pectinacetylesterase, putative
246404_at	At1g57600	0,98	0,96	0,94	0,93	1,01	1,01	1,08	0,96	membrane bound O-acyl transferase (MBOAT) family protein
246378_at	At1g57620	1,09	0,92	0,86	1,22	1,30	0,87	0,84	0,72	emp24/gp25L/p24 family protein
246405_at	At1g57630	0,97	1,01	1,06	2,18	1,54	1,29	0,91	0,95	disease resistance protein (TIR class), putative
246406_at	At1g57650	0,95	0,95	1,05	1,07	1,01	1,14	1,00	0,93	disease resistance protein (NBS-LRR class), putative
246379_s_at	At1g57660	1,34	1,12	1,06	1,09	1,20	0,96	1,04	0,94	60S ribosomal protein L21 (RPL21E)
246407_at	At1g57670	0,95	1,00	1,28	0,89	1,18	0,76	1,01	1,16	Toll-Interleukin-Resistance (TIR) domain-containing protein
246408_at	At1g57680	0,90	0,85	0,85	0,94	0,76	0,90	0,99	1,04	expressed protein
246409_at	At1g57700	0,88	0,98	0,83	0,97	1,10	0,94	0,99	0,80	protein kinase family protein
246380_at	At1g57750	1,03	1,08	1,02	0,95	1,15	1,06	1,03	0,97	cytochrome P450, putative
246410_at	At1g57760	0,94	0,91	0,91	1,19	1,28	0,97	0,89	0,99	expressed protein
246411_at	At1g57770	0,42	0,41	0,44	0,77	0,91	0,93	0,84	0,86	amine oxidase family
245829_at	At1g57780	1,03	0,97	1,03	1,00	1,05	0,96	0,98	0,99	heavy-metal-associated domain-containing protein
245830_at	At1g57790	0,78	0,91	0,87	1,00	1,17	0,89	0,93	1,18	F-box family protein
245828_at	At1g57820	1,68	1,54	1,39	1,88	1,94	1,09	0,89	0,82	zinc finger (C3HC4-type RING finger) family protein
245827_at	At1g57830	0,93	1,03	1,08	0,92	1,09	0,88	1,04	0,89	Toll-Interleukin-Resistance (TIR) domain-containing protein
245826_at	At1g57850	0,98	1,01	1,05	0,91	0,89	1,13	1,08	1,06	Toll-Interleukin-Resistance (TIR) domain-containing protein
245825_at	At1g57870	0,89	0,95	0,86	1,08	1,06	1,02	0,98	0,95	shaggy-related protein kinase kappa, putative / ASK-kappa, putative
245823_at	At1g57906	0,93	0,98	1,07	1,09	1,00	0,95	1,00	1,04	hypothetical protein

245824_at	At1g57943	0,85	0,86	0,93	0,90	1,05	0,83	1,01	0,96	purine permease-related
245866_s_at	At1g57990	0,93	0,88	0,93	0,78	0,70	0,91	1,15	1,39	purine permease-related
245865_at	At1g58025	1,27	1,48	1,44	1,01	1,08	0,88	1,00	1,06	DNA-binding bromodomain-containing protein
245868_at	At1g58032	0,67	0,70	0,72	0,75	0,70	0,96	1,07	1,11	amino acid permease family protein
245863_s_at	At1g58050	0,92	0,83	0,78	0,85	0,85	1,00	0,94	1,01	helicase domain-containing protein
245864_at	At1g58070	1,25	1,23	0,95	1,11	1,36	1,00	0,93	0,86	expressed protein
245867_at	At1g58080	1,10	0,98	0,95	1,14	1,16	0,85	0,89	0,82	ATP phosphoribosyl transferase 1 (ATP-PRT1)
246398_at	At1g58100	0,91	0,93	1,07	0,86	1,24	1,00	0,92	0,73	TCP family transcription factor, putative
246399_at	At1g58110	1,09	1,00	1,26	1,02	1,26	1,01	0,92	1,19	bZIP family transcription factor
246392_at	At1g58120	1,07	0,94	0,90	0,83	1,11	0,99	0,92	0,88	expressed protein
246393_at	At1g58150	1,01	1,09	1,03	0,84	0,92	0,75	0,81	0,87	hypothetical protein
246394_at	At1g58160	1,01	1,04	0,97	0,96	0,96	0,93	0,86	0,87	jacalin lectin family protein
246395_at	At1g58170	1,00	1,14	1,03	1,15	1,50	1,18	1,91	1,22	disease resistance-responsive protein-related / dirigent protein-related
246396_at	At1g58180	1,47	1,43	1,57	0,84	1,03	1,19	1,40	1,72	carbonic anhydrase family protein / carbonate dehydratase family protein
246397_at	At1g58190	0,95	0,97	0,95	1,10	0,96	0,92	0,97	0,93	leucine-rich repeat family protein
256229_at	At1g58200	1,17	1,33	2,44	0,93	1,19	1,11	1,33	1,75	mechanosensitive ion channel domain-containing protein / MS ion channel
256200_at	At1g58210	1,01	0,94	0,99	0,93	1,07	1,09	0,93	1,21	kinase interacting family protein
256198_at	At1g58220	0,88	0,92	1,14	0,72	0,93	1,04	0,97	1,37	myb family transcription factor
256201_at	At1g58230	0,94	0,82	1,05	0,75	0,90	0,96	1,01	1,10	WD-40 repeat family protein / beige-related
256199_at	At1g58250	1,28	0,81	1,09	0,67	0,66	1,21	1,08	1,34	SABRE, putative
256019_at	At1g58260	1,05	1,01	1,00	1,01	1,08	0,96	1,00	0,82	cytochrome P450-related
256021_at	At1g58270	0,71	0,61	0,77	1,12	1,00	0,91	1,00	1,01	meprin and TRAF homology domain-containing protein / MATH domain-cor
256020_at	At1g58290	0,99	0,96	1,24	0,61	0,76	0,73	0,78	0,85	glutamyl-tRNA reductase 1 / GluTR (HEMA1)
256018_at	At1g58300	1,03	0,98	0,95	1,04	1,07	1,01	1,08	1,08	heme oxygenase, putative
256023_at	At1g58330	0,97	0,93	0,98	1,10	1,09	1,17	1,07	0,98	transcription factor-related
256024_at	At1g58340	0,82	0,92	1,12	0,95	0,91	1,14	1,04	1,26	MATE efflux protein-related
256022_at	At1g58360	0,53	0,56	0,66	0,98	1,01	0,93	1,00	0,99	amino acid permease I (AAP1)
256025_at	At1g58370	1,80	1,62	1,46	1,88	1,67	0,71	0,81	1,17	glycosyl hydrolase family 10 protein / carbohydrate-binding domain-containi
245841_s_at	At1g58380	1,35	1,21	1,18	1,10	0,99	1,01	0,99	0,98	40S ribosomal protein S2 (RPS2A)
245839_at	At1g58390	0,94	0,97	1,19	0,93	1,04	0,94	0,93	1,00	disease resistance protein (CC-NBS-LRR class), putative
245838_at	At1g58410	0,89	1,06	1,02	1,04	0,92	1,18	1,14	1,10	disease resistance protein (CC-NBS-LRR class), putative
245840_at	At1g58420	0,80	0,55	0,65	1,21	1,26	1,02	0,88	0,86	expressed protein
245842_at	At1g58430	0,92	0,97	1,01	0,99	1,00	0,94	1,08	0,75	GDSL-motif lipase/hydrolase family protein
245809_at	At1g58440	0,85	0,76	0,75	1,22	1,03	0,92	0,75	0,86	squalene monooxygenase, putative / squalene epoxidase, putative
245837_at	At1g58450	1,01	1,00	1,00	1,02	0,99	1,04	1,03	1,08	peptidyl-prolyl cis-trans isomerase FKBP-type family protein
245808_at	At1g58470	0,97	0,90	1,16	1,17	1,26	1,02	1,18	0,97	RNA-binding protein (XF41)
245218_s_at	At1g58842	0,91	0,88	1,15	0,55	0,51	1,36	2,03	1,71	disease resistance protein (CC-NBS-LRR class), putative / PRM1 homolog,
245219_at	At1g59124	1,07	1,05	1,03	0,52	0,47	1,40	1,73	2,13	disease resistance protein (CC-NBS-LRR class), putative / PRM1 homolog,
245220_at	At1g59171	1,05	1,06	0,98	1,10	1,05	0,99	0,86	1,00	hypothetical protein
245221_s_at	At1g59265	0,96	0,97	1,13	1,01	1,04	1,00	0,94	0,95	---
262099_s_at	At1g59500	1,99	2,23	1,97	1,00	1,12	1,46	1,22	1,06	auxin-responsive GH3 family protein
262071_at	At1g59510	0,96	1,06	0,94	0,98	0,88	1,28	0,99	0,85	expressed protein
262078_at	At1g59520	0,97	0,97	1,04	1,19	0,96	0,93	1,00	0,99	expressed protein (CW7)
257471_at	At1g59530	0,90	0,86	1,01	0,92	1,02	1,06	1,03	0,96	bZIP transcription factor family protein
262101_at	At1g59535	0,92	0,95	0,91	1,03	1,05	1,03	0,96	1,12	hypothetical protein
262081_at	At1g59540	1,23	1,17	1,23	1,33	0,98	1,13	1,22	1,08	kinesin motor protein-related

262100_s_at	At1g59550	1,03	1,13	1,08	1,10	1,08	0,96	0,99	0,90	UBX domain-containing protein
262075_at	At1g59560	1,19	1,24	1,21	0,99	1,04	1,01	0,90	0,98	expressed protein
262076_at	At1g59580	0,86	0,82	0,89	1,25	1,26	0,84	0,76	0,84	mitogen-activated protein kinase, putative / MAPK, putative (MPK2)
262072_at	At1g59590	1,02	1,09	0,97	0,87	0,99	1,36	1,11	1,26	expressed protein
262079_at	At1g59600	1,21	1,04	1,06	0,92	0,94	0,88	0,96	0,86	expressed protein
262077_at	At1g59610	1,13	0,88	0,90	1,00	1,04	0,94	0,98	0,99	dynamin-like protein, putative (ADL3)
262126_at	At1g59620	0,67	0,93	1,17	0,58	1,06	1,23	1,16	1,20	disease resistance protein (CC-NBS class), putative
262123_at	At1g59630	0,91	1,04	1,08	0,95	0,98	1,00	0,98	0,95	F-box family protein-related
262073_at	At1g59640	0,80	0,98	0,94	1,00	0,93	0,97	0,97	0,91	basic helix-loop-helix (bHLH) family protein
262080_at	At1g59650	0,96	1,02	1,07	1,33	1,17	0,94	0,84	0,93	expressed protein
262124_at	At1g59660	1,07	0,90	1,04	1,09	1,06	1,12	0,90	0,76	nucleoporin family protein
262074_at	At1g59670	0,88	0,88	0,81	1,01	1,04	1,09	1,08	1,36	glutathione S-transferase, putative
262125_at	At1g59680	0,93	0,98	1,06	1,03	1,03	1,07	0,95	1,14	F-box family protein
262916_at	At1g59700	0,86	0,80	1,21	1,02	0,85	0,88	0,96	0,97	glutathione S-transferase, putative
262910_at	At1g59710	0,67	0,75	0,95	1,26	1,34	0,90	0,84	0,73	expressed protein
262907_at	At1g59720	0,89	0,98	0,96	0,61	0,77	1,03	1,07	1,00	pentatricopeptide (PPR) repeat-containing protein
262904_at	At1g59725	0,94	1,04	0,95	1,08	1,10	1,02	0,87	0,93	DNAJ heat shock protein, putative
262905_at	At1g59730	1,01	0,76	0,94	1,21	1,34	1,02	1,29	1,27	thioredoxin, putative
262912_at	At1g59740	0,94	1,44	1,27	1,87	1,53	1,02	1,28	1,12	proton-dependent oligopeptide transport (POT) family protein
262914_at	At1g59750	0,83	0,72	1,02	0,65	0,85	1,07	0,87	0,96	auxin-responsive factor (ARF1)
262906_at	At1g59760	1,18	1,04	1,14	0,79	0,83	0,98	0,91	0,95	ATP-dependent RNA helicase, putative
262894_at	At1g59780	1,03	1,10	1,16	0,96	1,12	1,00	0,94	1,02	disease resistance protein (CC-NBS-LRR class), putative
262893_at	At1g59790	0,99	1,03	0,94	0,97	1,06	1,03	0,94	1,02	cullin-related
262895_at	At1g59800	1,10	1,01	0,88	0,97	1,15	1,12	1,02	1,06	cullin-related
262896_at	At1g59820	0,77	0,79	1,20	1,04	0,96	1,07	1,31	1,57	haloacid dehalogenase-like hydrolase family protein
262909_at	At1g59830	0,94	0,91	1,30	0,88	0,90	1,01	0,82	1,07	serine/threonine protein phosphatase PP2A-2 catalytic subunit (PP2A2)
262897_at	At1g59840	0,72	0,65	0,59	0,75	0,55	1,00	0,90	0,85	expressed protein
262898_at	At1g59850	0,99	0,96	1,05	1,04	1,12	1,11	0,92	0,85	expressed protein
262911_s_at	At1g59860	3,08	2,10	1,70	2,13	2,13	1,51	1,17	1,37	17.8 kDa class I heat shock protein (HSP17.8-CI)
262899_at	At1g59870	0,85	0,69	0,78	0,78	0,71	0,97	0,87	0,88	ABC transporter family protein
262900_at	At1g59890	0,88	0,91	1,02	0,83	0,90	1,00	1,00	1,07	paired amphipathic helix repeat-containing protein
262908_at	At1g59900	0,90	0,91	0,96	0,89	1,01	0,93	0,90	0,90	pyruvate dehydrogenase E1 component alpha subunit, mitochondrial (PDH
262901_at	At1g59910	0,70	1,04	1,55	0,81	0,85	1,18	1,64	1,68	formin homology 2 domain-containing protein / FH2 domain-containing prot
262902_x_at	At1g59930	0,94	1,07	0,99	1,02	1,08	0,95	0,93	0,98	hypothetical protein
262915_at	At1g59940	1,04	0,99	1,08	1,18	1,05	0,96	1,07	1,07	two-component responsive regulator / response regulator 3 (ARR3)
262903_at	At1g59950	0,97	0,90	0,89	1,05	0,97	1,01	0,95	0,93	aldo/keto reductase, putative
262913_at	At1g59960	0,78	0,76	0,78	1,26	1,26	0,67	0,62	0,64	aldo/keto reductase, putative
263731_at	At1g59970	0,59	0,62	0,64	1,08	1,22	0,74	0,62	0,98	matrixin family protein
263732_at	At1g59980	1,02	0,90	0,99	0,85	1,01	0,88	0,90	0,82	DNAJ heat shock N-terminal domain-containing protein
263679_at	At1g59990	1,17	0,98	0,91	0,69	0,63	0,95	0,95	0,95	DEAD/DEAH box helicase, putative (RH22)
263736_at	At1g60000	1,09	0,91	1,02	0,70	0,61	1,01	0,99	0,93	29 kDa ribonucleoprotein, chloroplast, putative / RNA-binding protein cp29,
263737_at	At1g60010	1,11	1,01	1,37	0,82	0,86	0,85	0,94	1,27	expressed protein
263733_at	At1g60020	1,02	0,95	1,22	1,03	1,07	1,02	0,94	0,92	---
263734_at	At1g60030	1,15	1,38	1,16	1,30	2,50	0,90	1,04	1,18	xanthine/uracil permease family protein
263735_s_at	At1g60040	0,90	1,01	0,99	1,23	1,10	1,10	0,97	0,85	MADS-box family protein
263738_at	At1g60060	1,10	1,14	1,02	1,02	1,11	0,97	1,16	1,04	expressed protein

263728_at	At1g60070	0,85	0,79	0,81	1,13	1,16	0,80	0,86	0,94	gamma-adaptin, putative
263729_at	At1g60080	1,36	0,98	1,20	0,97	0,86	1,00	0,97	0,91	3' exoribonuclease family domain 1-containing protein
263730_at	At1g60090	0,78	0,83	0,77	0,91	1,02	1,05	1,00	1,06	glycosyl hydrolase family 1 protein
264278_at	At1g60130	1,06	1,05	0,98	0,95	1,03	1,09	1,04	1,07	jacalin lectin family protein
264246_at	At1g60140	0,69	1,24	1,63	0,74	0,99	0,94	1,09	1,30	glycosyl transferase family 20 protein / trehalose-phosphatase family protei
264247_at	At1g60160	0,81	0,88	1,09	0,64	0,65	1,09	1,06	0,88	potassium transporter family protein
264221_s_at	At1g60170	1,41	1,24	1,41	1,01	1,17	1,02	1,03	1,15	pre-mRNA processing ribonucleoprotein binding region-containing protein
264216_at	At1g60180	0,96	1,03	1,06	0,98	1,00	0,95	0,96	1,01	hypothetical protein
264217_at	At1g60190	1,09	1,30	1,23	2,04	1,10	1,69	1,18	1,29	armadillo/beta-catenin repeat family protein / U-box domain-containing prot
264218_at	At1g60200	1,12	1,11	1,49	0,89	0,93	1,23	1,67	2,00	splicing factor PWI domain-containing protein / RNA recognition motif (RRM
264267_at	At1g60210	1,11	1,37	1,22	0,96	0,97	1,37	1,26	1,61	splicing factor PWI domain-containing protein / RNA recognition motif (RRM
264268_at	At1g60220	1,01	0,88	0,89	1,05	1,17	0,97	1,01	0,87	Ulp1 protease family protein
264222_at	At1g60230	0,97	0,90	0,89	0,73	0,72	1,10	0,85	0,86	radical SAM domain-containing protein
264269_at	At1g60240	1,06	1,00	1,14	0,99	0,89	1,01	0,95	1,01	apical meristem formation protein-related
264270_at	At1g60260	0,89	0,82	0,88	1,07	0,66	0,86	1,03	1,02	Full-length cDNA Complete sequence from clone GSLTLS85ZG04 of Adult
264271_at	At1g60270	0,91	0,89	0,80	1,43	1,05	0,93	1,47	1,39	pseudogene, glycosyl hydrolase family 1
264272_at	At1g60280	0,94	1,03	1,02	0,99	1,05	1,07	1,00	1,00	no apical meristem (NAM) protein-related
264273_s_at	At1g60300	0,96	0,99	1,09	0,91	1,11	0,96	0,98	0,84	apical meristem formation protein-related
264274_at	At1g60320	0,92	1,02	1,22	0,93	1,05	1,01	1,04	1,07	Toll-Interleukin-Resistance (TIR) domain-containing protein
264275_at	At1g60350	0,95	0,94	0,99	0,91	1,05	0,96	0,93	1,07	no apical meristem (NAM) protein-related
264276_at	At1g60380	0,96	1,04	1,01	0,94	1,02	0,98	0,95	0,92	apical meristem formation protein-related
264277_at	At1g60390	1,04	1,08	1,26	1,09	1,29	0,86	0,90	0,84	BURP domain-containing protein / polygalacturonase, putative
257425_at	At1g60400	1,01	1,00	0,97	0,90	1,16	0,97	1,09	0,80	F-box family protein
264219_at	At1g60420	0,92	0,92	0,79	1,06	1,16	0,90	0,92	0,89	DC1 domain-containing protein
264220_at	At1g60430	0,97	1,10	1,20	1,06	1,14	1,04	0,81	0,92	ARP2/3 complex 21 kDa subunit family
264244_at	At1g60440	0,94	0,83	0,84	1,09	0,92	0,87	0,81	1,04	eukaryotic pantothenate kinase family protein
264245_at	At1g60450	0,98	0,99	1,02	0,97	1,20	0,98	0,90	1,07	galactinol synthase, putative
264964_at	At1g60460	0,87	0,97	0,94	0,85	0,98	0,96	1,01	0,95	hypothetical protein
264940_at	At1g60470	1,23	1,06	1,04	1,12	0,99	1,24	1,58	1,05	galactinol synthase, putative
264927_at	At1g60490	0,84	0,93	0,98	0,92	1,10	1,01	0,99	1,05	phosphatidylinositol 3-kinase (PI3K)
264917_at	At1g60500	0,98	1,12	1,04	0,99	1,11	0,99	0,94	0,90	dynamin family protein
264918_at	At1g60530	1,00	1,01	0,97	1,12	0,92	1,04	0,93	1,02	dynamin family protein
264919_at	At1g60540	1,01	0,98	0,97	1,01	0,99	1,10	0,94	1,17	dynamin family protein
264920_at	At1g60550	1,10	1,04	1,01	1,04	0,86	1,20	1,05	1,04	naphthoate synthase, putative / dihydroxynaphthoic acid synthetase, putativ
264965_at	At1g60560	1,29	1,49	1,50	0,92	0,88	1,00	1,06	1,00	SWIM zinc finger family protein
264966_at	At1g60570	0,99	1,12	1,14	0,88	1,09	0,95	1,17	1,18	kelch repeat-containing F-box family protein
264931_at	At1g60590	0,78	0,87	1,05	0,88	0,76	1,71	1,62	1,18	polygalacturonase, putative / pectinase, putative
264963_at	At1g60600	1,01	1,01	0,84	0,82	0,63	0,93	0,92	0,96	UbiA prenyltransferase family protein
264938_at	At1g60610	0,85	0,93	1,01	0,99	1,12	0,85	0,85	0,87	expressed protein
264924_at	At1g60620	1,11	1,20	1,16	0,90	1,09	1,07	1,07	1,18	DNA-directed RNA polymerase, putative
264939_at	At1g60630	0,82	0,96	0,98	1,06	1,24	1,01	0,86	0,98	leucine-rich repeat family protein
264921_at	At1g60650	1,07	1,32	1,49	1,01	1,12	0,95	0,97	1,07	glycine-rich RNA-binding protein, putative
264926_at	At1g60660	1,30	1,06	0,88	1,31	1,34	0,84	0,74	0,64	cytochrome b5 domain-containing protein
264925_at	At1g60670	1,53	1,53	1,45	0,86	1,19	0,91	1,03	1,46	expressed protein
264941_at	At1g60680	0,82	0,85	0,84	1,29	1,21	1,10	1,08	1,10	aldo/keto reductase family protein
264911_at	At1g60690	1,01	1,12	1,19	0,89	0,96	1,11	0,98	0,99	aldo/keto reductase family protein

264928_at	At1g60710	0,96	0,78	0,84	1,19	1,07	0,98	1,05	0,97	aldo/keto reductase family protein
264929_at	At1g60730	0,73	0,77	0,76	0,87	0,80	1,10	1,06	1,00	aldo/keto reductase family protein
264923_s_at	At1g60740	1,09	1,00	1,01	0,65	0,68	0,56	0,70	0,82	peroxiredoxin type 2, putative
264912_at	At1g60750	1,03	0,99	0,89	0,92	1,03	0,96	0,90	1,07	---
264913_at	At1g60770	1,48	1,06	1,17	1,06	1,07	1,05	0,87	0,87	pentatricopeptide (PPR) repeat-containing protein
264914_at	At1g60780	1,00	0,85	0,92	1,35	1,18	0,98	0,91	0,94	clathrin adaptor complexes medium subunit family protein
264915_at	At1g60790	0,95	0,99	1,09	0,99	1,17	0,96	1,01	1,06	expressed protein
264930_at	At1g60800	0,85	1,32	1,18	0,68	0,66	1,11	1,32	1,53	leucine-rich repeat family protein / protein kinase family protein
264916_at	At1g60810	0,82	0,67	0,61	1,28	1,27	0,77	0,85	0,73	ATP citrate-lyase -related
264922_s_at	At1g60830	1,21	1,19	2,01	0,86	0,97	1,19	1,54	1,57	U2 snRNP auxiliary factor large subunit, putative
259908_at	At1g60850	1,13	1,10	1,12	1,05	0,90	0,94	0,94	0,87	DNA-directed RNA polymerase, putative
259907_at	At1g60860	1,10	1,22	1,41	0,89	1,10	1,14	1,35	1,28	ARF GTPase-activating domain-containing protein
259909_at	At1g60870	1,24	1,07	1,20	1,21	1,34	1,05	0,86	0,85	expressed protein
259721_at	At1g60890	0,72	0,83	1,10	0,85	0,80	0,96	0,98	0,93	phosphatidylinositol-4-phosphate 5-kinase family protein
259722_at	At1g60930	0,98	0,91	0,95	0,86	0,89	0,88	0,83	0,98	DNA helicase, putative
259724_at	At1g60940	1,01	0,87	0,85	0,93	1,11	1,05	0,94	0,93	serine/threonine protein kinase, putative
259727_at	At1g60950	1,04	1,00	1,01	0,68	0,73	0,89	0,81	0,77	ferredoxin, chloroplast (PETF)
259723_at	At1g60960	1,01	1,07	1,18	0,77	0,96	0,91	1,01	1,40	metal transporter, putative (IRT3)
259728_at	At1g60970	0,92	0,92	0,85	0,92	0,95	1,01	0,93	0,85	clathrin adaptor complex small chain family protein
259714_at	At1g60980	0,98	1,09	0,97	1,06	0,97	0,96	1,02	0,95	gibberellin 20-oxidase, putative
259726_at	At1g60985	1,01	1,04	0,97	0,93	1,10	0,99	1,04	1,00	expressed protein
259715_at	At1g60990	1,30	1,16	1,20	0,86	0,70	1,03	1,15	1,28	glycine cleavage T family protein / aminomethyl transferase family protein
259716_at	At1g61000	1,06	0,84	0,90	0,95	0,95	0,99	0,98	0,90	Nuf2 family protein
259717_at	At1g61010	1,29	1,46	1,78	0,97	1,35	0,92	0,98	1,03	cleavage and polyadenylation specificity factor, putative
259718_at	At1g61040	0,89	0,74	0,76	0,83	1,01	1,01	0,83	1,00	plus-3 domain-containing protein
259725_at	At1g61065	0,56	1,01	1,03	0,91	0,81	0,75	1,08	1,10	expressed protein
259719_at	At1g61070	0,96	1,04	0,96	0,95	1,05	0,96	1,02	1,05	plant defensin-fusion protein, putative (PDF2.4)
259720_at	At1g61080	0,92	0,92	0,99	0,90	1,01	1,08	1,09	1,04	proline-rich family protein
264934_at	At1g61090	1,01	1,02	1,06	0,99	1,05	1,11	1,00	1,03	hypothetical protein
264910_at	At1g61100	1,00	0,98	1,25	1,06	1,01	0,96	1,37	1,77	disease resistance protein (TIR class), putative
264882_at	At1g61110	0,93	1,10	1,08	0,91	1,07	1,12	1,18	0,97	no apical meristem (NAM) family protein
264886_at	At1g61120	0,86	0,52	1,05	1,55	1,29	0,76	0,90	1,21	terpene synthase/cyclase family protein
264937_at	At1g61130	1,03	0,99	0,91	1,03	1,11	0,99	1,02	0,68	serine carboxypeptidase S10 family protein
264936_at	At1g61140	0,69	0,79	0,93	1,23	1,35	1,01	1,08	0,86	SNF2 domain-containing protein / helicase domain-containing protein / zinc
264935_at	At1g61150	1,17	1,01	1,12	1,21	1,15	1,06	0,97	1,07	expressed protein
264933_at	At1g61160	0,94	1,03	1,10	0,92	1,06	1,02	1,05	1,08	expressed protein
264884_at	At1g61170	0,90	0,75	0,86	0,83	1,00	0,76	0,93	0,80	expressed protein
264885_s_at	At1g61180	1,21	1,01	1,17	0,85	0,76	1,34	1,12	0,99	disease resistance protein (CC-NBS-LRR class), putative
264880_at	At1g61210	1,28	1,20	1,00	0,98	1,17	0,88	1,09	0,96	WD-40 repeat family protein / katanin p80 subunit, putative
264932_at	At1g61240	0,82	0,84	1,01	0,93	0,85	0,99	1,10	0,78	expressed protein
264883_s_at	At1g61250	0,84	0,76	0,77	1,01	0,86	0,90	0,85	0,85	secretory carrier membrane protein (SCAMP) family protein
264879_at	At1g61260	0,75	0,87	0,71	0,90	1,03	1,01	1,13	0,96	expressed protein
264881_s_at	At1g61270	1,01	1,05	1,03	0,99	0,97	0,97	0,91	0,95	lysine and histidine specific transporter, putative
264761_at	At1g61280	0,90	0,85	0,91	1,14	0,91	0,93	0,92	1,03	expressed protein
264760_at	At1g61290	0,96	0,92	0,96	0,91	1,11	1,01	0,99	1,10	syntaxin, putative (SYP124)
257411_s_at	At1g61320	1,00	0,94	0,96	0,95	1,10	0,89	0,89	0,90	hypothetical protein

264758_at	At1g61340	1,16	2,09	1,27	0,97	0,78	1,51	2,63	2,13	F-box family protein
264769_at	At1g61350	0,73	0,82	0,80	0,77	0,88	0,77	0,83	0,84	armadillo/beta-catenin repeat family protein
264757_at	At1g61360	0,82	0,94	1,05	0,70	0,77	0,97	1,13	1,22	S-locus lectin protein kinase family protein
264756_at	At1g61370	1,03	0,91	0,91	0,90	0,97	1,34	1,44	1,38	S-locus lectin protein kinase family protein
264767_at	At1g61380	0,56	0,55	0,79	0,77	1,01	0,87	0,79	0,78	S-locus protein kinase, putative
264755_at	At1g61390	0,93	0,93	0,99	0,92	0,99	0,87	0,97	1,08	S-locus protein kinase, putative
264754_at	At1g61400	1,26	1,12	1,15	0,95	0,91	1,22	1,18	1,55	S-locus protein kinase, putative
264768_at	At1g61410	0,96	0,97	0,97	0,92	1,01	0,96	1,02	1,00	tolA protein-related
264766_at	At1g61420	1,24	0,98	1,35	0,89	0,95	1,07	1,07	1,18	S-locus lectin protein kinase family protein
264765_at	At1g61430	1,04	0,95	0,98	0,95	1,11	0,97	0,98	1,05	S-locus protein kinase, putative
264764_at	At1g61440	1,02	1,07	1,02	1,01	1,00	0,98	0,99	0,99	S-locus protein kinase, putative
264763_at	At1g61450	1,18	1,22	1,09	1,54	1,30	0,90	0,86	0,79	expressed protein
264762_at	At1g61460	0,93	0,97	0,96	0,92	0,89	0,99	1,12	0,97	S-locus protein kinase, putative
264759_at	At1g61480	1,00	1,04	1,13	1,00	0,92	1,06	1,03	0,97	S-locus protein kinase, putative
264753_at	At1g61490	1,07	1,06	1,21	0,88	0,86	1,05	1,13	1,15	S-locus protein kinase, putative
265063_at	At1g61500	1,09	0,96	1,06	0,84	0,60	1,10	1,36	1,32	S-locus protein kinase, putative
265033_at	At1g61520	0,95	0,91	1,01	0,83	0,92	1,00	1,00	0,97	chlorophyll A-B binding protein / LHCI type III (LHCA3.1)
265062_at	At1g61550	0,97	0,97	0,92	1,06	1,08	1,02	1,01	1,04	S-locus protein kinase, putative
265008_at	At1g61560	0,64	0,77	0,92	1,02	0,99	0,82	1,32	1,23	seven transmembrane MLO family protein / MLO-like protein 6 (MLO6)
265007_s_at	At1g61566	0,99	0,90	1,01	1,05	0,89	1,09	1,07	0,98	rapid alkalinization factor (RALF) family protein
265006_at	At1g61570	1,70	1,29	1,04	1,48	1,37	1,00	0,96	0,81	mitochondrial import inner membrane translocase (TIM13)
265032_at	At1g61580	0,97	1,05	0,89	1,19	1,22	0,58	0,71	0,65	60S ribosomal protein L3 (RPL3B)
265031_at	At1g61590	1,12	1,09	0,93	0,92	1,06	1,21	1,11	1,35	protein kinase, putative
265030_at	At1g61610	1,04	0,94	1,08	1,66	1,27	0,99	1,01	0,99	S-locus lectin protein kinase family protein
265035_at	At1g61620	0,98	1,01	1,16	1,04	1,13	1,08	1,03	1,19	expressed protein
265064_at	At1g61630	0,90	1,01	1,00	1,01	1,18	0,95	0,93	0,96	equilibrative nucleoside transporter, putative (ENT7)
265061_at	At1g61640	1,09	0,86	1,03	0,93	0,98	0,79	0,68	0,79	ABC1 family protein
265034_at	At1g61660	1,42	2,91	1,42	1,03	1,26	1,04	1,22	1,19	basic helix-loop-helix (bHLH) family protein
265005_at	At1g61667	0,98	0,97	0,78	1,06	1,17	0,97	0,74	0,64	expressed protein
264429_at	At1g61670	0,98	1,01	1,05	0,96	0,89	0,89	0,99	0,92	expressed protein
264430_at	At1g61680	0,95	1,04	1,06	1,06	1,33	0,94	1,02	1,07	terpene synthase/cyclase family protein
264423_at	At1g61690	0,73	0,84	1,07	0,93	1,15	1,04	1,01	1,14	tetratricopeptide repeat (TPR)-containing protein
264431_at	At1g61700	0,91	0,99	0,99	0,97	1,18	0,85	0,98	0,92	DNA-directed RNA polymerase II, putative (RPB10)
264432_at	At1g61710	1,05	1,06	0,94	0,96	0,96	0,94	1,10	1,04	DC1 domain-containing protein
264401_at	At1g61720	1,05	0,93	1,40	1,19	0,98	0,98	1,04	0,91	dihydroflavonol 4-reductase (dihydrokaempferol 4-reductase) family (BAN)
264398_at	At1g61730	0,79	0,69	0,81	0,91	0,84	0,85	0,89	0,99	DNA-binding storekeeper protein-related
264424_at	At1g61740	1,63	1,29	1,22	1,39	1,20	1,08	1,11	1,12	expressed protein
264425_at	At1g61750	1,11	1,19	1,05	1,11	1,00	1,18	1,07	1,28	expressed protein
264426_at	At1g61760	1,09	1,08	1,08	1,19	0,91	0,96	0,93	1,11	harpin-induced protein-related / HIN1-related / harpin-responsive protein-re
264427_at	At1g61770	1,12	1,03	0,86	1,22	1,09	1,04	0,96	0,97	DNAJ heat shock N-terminal domain-containing protein
264399_at	At1g61780	1,19	1,06	0,98	1,05	0,90	1,17	1,07	0,95	postsynaptic protein-related
264428_at	At1g61790	1,21	1,20	0,98	1,24	0,96	0,98	0,86	0,85	OST3/OST6 family protein
264400_at	At1g61800	0,84	0,65	0,54	0,29	0,54	1,67	1,06	1,11	glucose-6-phosphate/phosphate translocator, putative
264433_at	At1g61810	0,85	0,96	0,94	1,06	1,55	0,92	1,14	0,99	glycosyl hydrolase family 1 protein
264280_at	At1g61820	1,38	1,64	1,89	1,06	0,89	1,49	3,01	2,44	glycosyl hydrolase family 1 protein
264281_at	At1g61830	1,03	1,02	1,02	1,02	0,93	0,97	1,00	1,01	---

264282_at	At1g61840	0,93	1,00	0,85	1,42	1,00	1,24	0,98	0,94	DC1 domain-containing protein
264283_at	At1g61850	1,07	1,06	1,15	0,84	1,03	1,19	1,02	1,16	patatin family protein
264284_at	At1g61860	1,03	0,88	0,89	0,92	0,89	0,90	0,84	1,02	protein kinase, putative
264286_at	At1g61870	1,02	0,79	0,76	1,23	1,07	0,94	0,85	0,83	pentatricopeptide (PPR) repeat-containing protein
264289_at	At1g61890	0,71	0,79	0,88	1,01	0,80	1,14	1,22	1,34	MATE efflux family protein
264307_at	At1g61900	0,87	0,83	0,78	1,06	0,80	0,93	0,82	0,86	expressed protein
264332_at	At1g61920	0,99	1,14	0,91	0,84	1,03	0,91	0,96	0,98	hypothetical protein
264287_at	At1g61930	1,06	0,95	0,89	1,02	1,09	0,98	0,87	1,18	expressed protein
264333_at	At1g61940	0,98	1,02	1,14	0,83	1,06	0,95	0,98	1,21	F-box family protein / tubby family protein
264334_at	At1g61950	1,01	1,07	1,03	0,98	1,01	0,94	1,03	0,97	calcium-dependent protein kinase, putative / CDPK, putative
264308_at	At1g61990	0,98	0,96	0,85	0,84	0,97	0,97	0,86	0,81	mitochondrial transcription termination factor-related / mTERF-related
257423_at	At1g62010	0,92	1,00	0,99	1,00	1,04	0,93	0,94	0,83	mitochondrial transcription termination factor-related / mTERF-related
264309_at	At1g62020	0,95	0,91	0,94	0,96	1,14	0,95	0,84	0,91	coatomer protein complex, subunit alpha, putative
264310_at	At1g62030	0,99	1,04	1,20	0,94	0,93	0,97	1,05	1,26	DC1 domain-containing protein
264285_at	At1g62040	0,98	0,94	1,04	0,95	1,05	0,99	1,05	0,96	autophagy 8c (APG8c)
264288_at	At1g62045	1,17	0,99	1,01	1,02	1,25	0,87	0,95	0,72	expressed protein
264744_at	At1g62050	1,00	1,01	1,07	0,64	0,94	0,88	0,84	1,09	ankyrin repeat protein-related
264735_s_at	At1g62060	1,06	0,78	1,04	0,86	1,02	1,16	0,87	1,03	expressed protein
264740_at	At1g62070	0,96	1,02	0,95	0,98	0,95	1,05	0,90	0,99	expressed protein
264730_at	At1g62090	1,21	1,05	1,00	1,22	1,09	0,96	0,88	0,96	mitochondrial transcription termination factor family protein / mTERF family
264743_at	At1g62100	0,98	1,08	1,02	0,98	1,00	1,21	1,01	0,92	---
257414_at	At1g62110	0,99	0,91	0,90	0,96	1,18	0,96	1,00	1,13	mitochondrial transcription termination factor family protein / mTERF family
264742_at	At1g62130	0,94	1,03	0,98	0,74	0,90	1,06	1,19	1,17	AAA-type ATPase family protein
264731_at	At1g62150	0,82	0,87	1,18	1,00	0,93	0,92	0,86	0,94	mitochondrial transcription termination factor-related / mTERF-related
264732_at	At1g62160	1,05	1,05	0,98	0,96	1,00	0,91	1,02	1,07	---
264733_at	At1g62170	1,06	1,03	0,95	0,98	1,19	1,00	1,01	1,05	serpin family protein / serine protease inhibitor family protein
264745_at	At1g62180	1,62	1,43	1,36	0,72	0,86	0,90	0,95	1,01	5'-adenylylsulfate reductase 2, chloroplast (APR2) (APSR) / adenosine 5'-p
264736_at	At1g62200	0,93	1,01	0,92	0,90	0,98	0,87	0,87	0,85	proton-dependent oligopeptide transport (POT) family protein
264737_at	At1g62210	1,09	1,06	1,11	0,91	0,96	1,02	1,06	0,92	expressed protein
264738_at	At1g62250	0,86	0,76	0,86	1,14	1,07	1,09	1,15	1,13	expressed protein
264739_at	At1g62260	1,05	1,04	1,19	1,07	0,92	0,91	1,06	1,02	pentatricopeptide (PPR) repeat-containing protein
264734_at	At1g62280	1,08	1,19	1,08	1,57	1,12	0,79	0,87	0,92	C4-dicarboxylate transporter/malic acid transport family protein
264741_at	At1g62290	0,32	0,31	0,29	0,81	0,73	0,76	0,74	0,67	aspartyl protease family protein
264746_at	At1g62300	0,49	0,60	1,00	0,85	0,93	0,90	1,34	1,31	WRKY family transcription factor
260627_at	At1g62310	0,72	0,76	1,03	0,83	0,80	1,12	1,17	1,09	transcription factor jumonji (jmjC) domain-containing protein
260628_at	At1g62320	0,97	0,96	0,90	1,15	1,00	0,92	1,26	1,05	early-responsive to dehydration protein-related / ERD protein-related
260629_at	At1g62330	1,02	0,98	0,98	1,16	1,09	0,82	0,73	0,78	expressed protein
260630_at	At1g62340	1,04	1,02	0,78	1,05	1,14	0,82	1,02	0,82	subtilisin-like serine protease / abnormal leaf shape1 (ALE1)
260631_at	At1g62350	1,06	1,10	0,95	0,94	0,91	1,14	0,99	1,02	pentatricopeptide (PPR) repeat-containing protein
260632_at	At1g62360	1,19	1,40	1,61	1,13	0,99	1,06	0,94	0,68	homeobox protein SHOOT MERISTEMLESS (STM)
260637_at	At1g62380	1,33	1,31	1,59	1,13	0,98	0,88	0,88	0,95	1-aminocyclopropane-1-carboxylate oxidase, putative / ACC oxidase, putati
260638_at	At1g62390	1,42	1,25	1,08	0,97	0,99	1,02	1,06	1,06	octicosapeptide/Phox/Bem1p (PB1) domain-containing protein / tetratricope
260633_at	At1g62400	0,81	0,78	0,63	0,83	0,79	0,99	0,98	0,99	protein kinase, putative
260634_at	At1g62410	0,93	1,01	0,96	0,90	0,96	0,91	1,02	0,93	MIF4G domain-containing protein
260635_at	At1g62420	0,83	0,94	0,81	1,12	0,89	0,86	1,02	1,05	expressed protein
260636_at	At1g62430	0,74	0,83	0,77	0,88	0,77	1,12	1,02	1,14	phosphatidate cytidylyltransferase / CDP-diglyceride synthetase (CDS1)

265114_at	At1g62440	1,01	1,21	1,05	1,19	1,37	1,28	0,90	0,96	leucine-rich repeat family protein / extensin family protein
265115_at	At1g62450	0,96	1,21	1,07	0,90	0,89	0,85	0,97	1,09	Rho GDP-dissociation inhibitor family protein
265113_at	At1g62460	1,08	1,04	1,05	1,15	1,00	0,95	0,94	0,92	---
265116_at	At1g62480	2,78	2,48	2,11	5,24	5,23	0,83	0,76	0,82	vacuolar calcium-binding protein-related
265110_at	At1g62490	0,95	1,06	1,04	0,96	1,19	1,05	0,98	1,00	mitochondrial transcription termination factor-related / mTERF-related
265117_at	At1g62500	1,10	1,01	1,05	1,23	1,37	0,69	0,51	0,47	protease inhibitor/seed storage/lipid transfer protein (LTP) family protein
265111_at	At1g62510	3,04	3,19	1,91	1,55	1,83	1,07	0,90	0,92	protease inhibitor/seed storage/lipid transfer protein (LTP) family protein
257403_at	At1g62520	0,99	1,12	0,97	1,11	1,12	0,91	0,85	1,04	expressed protein
265112_at	At1g62530	0,98	1,02	1,01	1,03	1,01	1,06	1,08	0,90	hypothetical protein
265122_at	At1g62540	0,78	0,77	0,91	1,40	1,04	1,04	1,04	1,03	flavin-containing monooxygenase family protein / FMO family protein
265121_at	At1g62560	1,25	1,26	1,20	1,26	1,14	1,12	1,02	0,68	flavin-containing monooxygenase family protein / FMO family protein
265119_at	At1g62570	1,10	0,92	1,10	0,67	0,58	0,97	1,26	1,18	flavin-containing monooxygenase family protein / FMO family protein
265106_s_at	At1g62590	0,87	0,84	0,98	0,99	1,03	0,88	1,04	1,04	pentatricopeptide (PPR) repeat-containing protein
265107_s_at	At1g62610	0,96	0,89	0,90	1,01	1,13	0,87	1,02	1,11	short-chain dehydrogenase/reductase (SDR) family protein
265108_s_at	At1g62620	0,98	0,99	0,87	0,90	0,99	0,98	0,99	0,97	flavin-containing monooxygenase family protein / FMO family protein
265109_s_at	At1g62630	0,95	1,12	1,29	0,44	0,52	1,20	1,07	1,11	disease resistance protein (CC-NBS-LRR class), putative
265120_at	At1g62640	0,92	0,86	0,87	1,12	1,18	0,97	0,88	0,76	3-oxoacyl-(acyl-carrier-protein) synthase III, chloroplast / beta-ketoacyl-ACF
265118_at	At1g62660	1,18	1,05	0,84	1,33	1,19	1,13	1,16	0,99	beta-fructosidase (BFRUCT3) / beta-fructofuranosidase / invertase, vacuola
262687_at	At1g62670	1,00	0,99	1,02	1,12	1,06	0,93	0,99	1,12	pentatricopeptide (PPR) repeat-containing protein
262688_at	At1g62680	1,01	0,96	1,03	0,98	0,90	0,94	0,85	1,06	pentatricopeptide (PPR) repeat-containing protein
262642_at	At1g62690	1,16	1,00	0,95	0,93	1,00	1,01	0,93	1,02	expressed protein
262689_at	At1g62700	0,98	1,00	0,93	0,96	0,99	0,89	0,93	0,89	no apical meristem (NAM) family protein
262644_at	At1g62710	0,20	0,19	0,11	0,49	0,48	1,26	1,21	1,28	vacuolar processing enzyme beta / beta-VPE
262690_at	At1g62720	1,01	0,89	1,01	0,91	1,05	1,05	0,92	1,02	pentatricopeptide (PPR) repeat-containing protein
262641_at	At1g62730	1,13	1,20	1,11	0,94	1,00	0,99	0,89	0,84	expressed protein
262691_at	At1g62740	1,38	1,11	1,17	1,37	1,22	1,12	1,11	1,23	stress-inducible protein, putative
262645_at	At1g62750	1,28	1,20	1,18	0,65	0,69	1,58	1,45	1,49	elongation factor Tu family protein
262692_at	At1g62760	1,02	0,83	1,07	1,03	0,93	1,04	1,04	0,96	---
262640_at	At1g62763	1,02	0,99	1,12	0,91	1,09	1,18	0,96	1,02	invertase/pectin methylesterase inhibitor family protein
262643_at	At1g62770	0,86	1,09	0,96	0,95	1,19	0,92	0,92	0,99	invertase/pectin methylesterase inhibitor family protein
262693_at	At1g62780	1,01	0,73	0,59	0,56	0,39	1,33	1,15	1,08	expressed protein
262694_at	At1g62790	0,98	0,81	0,69	1,39	1,19	0,84	0,77	0,80	protease inhibitor/seed storage/lipid transfer protein (LTP) family protein
262646_at	At1g62800	0,79	0,72	0,89	1,49	1,15	1,21	1,21	1,17	aspartate aminotransferase, cytoplasmic isozyme 2 / transaminase A (ASP
262667_at	At1g62810	0,95	0,84	0,73	1,10	1,22	1,12	1,06	1,14	copper amine oxidase, putative
262639_at	At1g62820	1,02	1,00	1,08	1,13	1,48	0,86	0,80	0,87	calmodulin, putative
262668_at	At1g62830	1,06	1,09	0,85	0,98	0,89	0,67	0,92	0,73	amine oxidase family protein / SWIRM domain-containing protein
257466_at	At1g62840	0,77	1,00	1,00	1,27	1,33	1,25	1,36	1,32	expressed protein
262669_at	At1g62850	1,09	0,98	1,01	0,93	0,94	1,13	1,14	0,97	expressed protein
261123_at	At1g62860	0,96	0,92	0,92	1,08	1,03	1,10	0,96	0,91	---
261092_at	At1g62870	0,92	1,10	0,96	1,06	0,94	1,01	0,98	0,94	expressed protein
261102_at	At1g62880	1,25	1,45	1,37	1,31	0,90	1,00	0,99	0,84	cornichon family protein
261093_at	At1g62890	1,02	0,99	1,24	0,88	1,08	1,11	1,13	1,27	hypothetical protein
261103_s_at	At1g62900	1,00	0,99	1,02	0,89	0,96	1,24	0,98	0,97	O-methyltransferase, putative
261104_at	At1g62910	1,18	1,00	1,17	1,02	1,03	1,12	0,95	1,17	pentatricopeptide (PPR) repeat-containing protein
261094_at	At1g62920	1,03	1,14	1,05	0,85	1,05	1,03	0,94	0,91	expressed protein
261095_at	At1g62930	0,92	0,98	0,93	0,97	1,07	1,09	0,94	0,88	pentatricopeptide (PPR) repeat-containing protein

261096_at	At1g62940	0,88	1,10	0,99	1,07	1,02	1,03	0,90	1,21	4-coumarate--CoA ligase family protein / 4-coumaroyl-CoA synthase family
261097_at	At1g62950	1,08	1,10	1,12	1,03	1,13	0,95	1,14	0,97	leucine-rich repeat transmembrane protein kinase, putative
261108_at	At1g62960	1,09	1,30	1,51	0,93	0,94	1,27	1,12	1,29	1-aminocyclopropane-1-carboxylate synthase, putative / ACC synthase, pu
261098_at	At1g62970	0,85	0,98	0,97	0,84	1,10	0,83	0,97	1,09	DNAJ heat shock N-terminal domain-containing protein
261099_at	At1g62980	0,98	0,99	0,84	1,15	1,02	1,10	0,92	0,90	expansin, putative (EXP18)
261106_at	At1g62990	0,93	1,25	1,01	1,08	0,82	0,99	1,55	1,36	homeodomain transcription factor (KNAT7)
261105_at	At1g63000	1,05	0,85	0,90	1,36	1,21	0,93	0,92	0,93	expressed protein
261107_at	At1g63010	0,85	0,85	0,82	0,80	0,86	0,91	0,99	0,91	SPX (SYG1/Pho81/XPR1) domain-containing protein
261100_at	At1g63020	1,18	1,05	1,07	0,92	1,10	0,90	0,93	0,92	DNA-directed RNA polymerase alpha subunit family protein
261101_at	At1g63030	0,38	0,66	0,93	0,61	0,75	0,64	0,95	0,94	AP2 domain-containing transcription factor, putative
259682_at	At1g63040	0,87	1,12	0,92	1,20	0,94	1,30	1,09	1,00	pseudogene, DRE binding protein
259683_at	At1g63050	1,17	1,25	1,09	1,10	0,91	1,21	0,98	0,96	membrane bound O-acyl transferase (MBOAT) family protein
259693_at	At1g63060	1,08	0,97	0,92	0,95	1,16	0,97	1,12	1,03	expressed protein
259684_at	At1g63070	0,95	0,94	0,98	0,99	0,94	1,09	1,04	1,05	pentatricopeptide (PPR) repeat-containing protein
259692_at	At1g63080	1,04	0,94	1,02	1,02	0,94	1,02	1,03	1,02	pentatricopeptide (PPR) repeat-containing protein
259685_at	At1g63090	0,81	1,69	0,76	0,96	0,73	1,17	1,46	1,58	F-box family protein / SKP1 interacting partner 3-related
259686_at	At1g63100	1,02	0,97	1,03	1,04	1,19	0,87	0,96	0,70	scarecrow transcription factor family protein
259687_at	At1g63110	0,90	0,88	0,76	0,84	0,90	0,85	0,75	0,75	cell division cycle protein-related
259688_at	At1g63120	1,05	0,96	0,99	1,09	0,99	0,95	0,87	1,06	rhomboid family protein
259689_x_at	At1g63130	1,06	0,86	1,05	0,91	0,96	0,91	0,85	1,26	pentatricopeptide (PPR) repeat-containing protein
259696_at	At1g63150	0,96	0,95	0,99	0,99	1,06	1,05	1,11	0,98	pentatricopeptide (PPR) repeat-containing protein
259690_at	At1g63160	1,38	1,34	1,23	1,02	0,96	0,94	0,90	0,93	replication factor C 40 kDa, putative
259695_at	At1g63170	0,92	0,96	0,94	0,90	0,87	1,10	0,91	0,95	zinc finger (C3HC4-type RING finger) family protein
259694_at	At1g63180	1,22	1,25	1,46	1,12	1,02	0,99	1,13	1,10	UDP-glucose 4-epimerase, putative / UDP-galactose 4-epimerase, putative
257509_at	At1g63190	1,05	1,01	1,05	0,97	1,09	1,03	0,96	0,96	hypothetical protein
259691_at	At1g63200	0,93	1,08	1,04	0,95	1,20	1,13	1,08	0,83	hypothetical protein
260084_at	At1g63210	0,99	1,05	1,14	1,01	1,14	1,04	0,96	1,05	hypothetical protein
260083_at	At1g63220	1,04	1,11	0,83	1,61	1,31	0,78	0,63	0,63	C2 domain-containing protein
260086_at	At1g63240	0,54	0,60	0,70	0,88	0,86	0,93	0,95	0,98	expressed protein
260109_at	At1g63260	0,79	0,67	0,66	0,84	0,80	0,77	0,84	0,92	senescence-associated family protein
260108_at	At1g63280	0,99	0,98	1,05	0,97	1,06	1,00	1,02	0,92	serpin-related / serine protease inhibitor-related
260085_at	At1g63290	1,15	0,95	0,93	1,04	1,06	0,97	0,87	0,87	ribulose-phosphate 3-epimerase, cytosolic, putative / pentose-5-phosphate
260113_at	At1g63300	1,08	1,13	1,09	0,92	0,97	1,10	1,03	1,15	expressed protein
260112_at	At1g63310	1,41	1,22	0,94	0,96	1,42	0,98	0,98	0,85	expressed protein
260111_at	At1g63320	0,97	1,01	1,12	1,05	1,03	1,07	1,11	1,02	pentatricopeptide (PPR) repeat-containing protein
265105_s_at	At1g63340	0,96	0,98	0,93	1,00	0,99	0,79	0,93	1,03	flavin-containing monooxygenase family protein / FMO family protein
260110_at	At1g63350	0,95	1,21	1,00	1,13	1,21	1,02	0,81	1,14	disease resistance protein (CC-NBS-LRR class), putative
261556_s_at	At1g63390	0,77	0,98	1,16	1,14	1,08	0,97	0,87	0,91	flavin-containing monooxygenase family protein / FMO family protein
261554_at	At1g63400	0,91	0,95	1,09	1,00	1,06	0,95	1,08	1,01	pentatricopeptide (PPR) repeat-containing protein
261553_at	At1g63420	1,10	0,98	0,95	0,83	0,87	1,04	1,21	1,08	expressed protein
261552_at	At1g63430	0,93	0,95	1,01	0,80	1,00	1,04	1,06	1,20	leucine-rich repeat transmembrane protein kinase, putative
261551_at	At1g63440	1,01	1,26	1,09	1,31	1,37	1,02	1,14	1,10	copper-exporting ATPase, putative / responsive-to-antagonist 1, putative / c
261550_at	At1g63450	1,03	1,08	0,91	1,10	1,05	0,99	0,91	1,08	exostosin family protein
261530_at	At1g63460	0,74	0,63	0,64	1,16	1,20	0,84	0,76	0,60	glutathione peroxidase, putative
261549_at	At1g63470	0,87	1,12	0,98	1,05	1,20	0,94	0,99	1,20	DNA-binding family protein
261548_at	At1g63480	0,83	1,09	1,02	0,75	0,87	0,95	0,96	0,95	DNA-binding family protein

261531_at	At1g63490	1,18	1,03	1,15	0,89	0,65	1,09	1,12	1,34 transcription factor jumonji (jmjC) domain-containing protein
261547_at	At1g63500	0,93	0,75	0,88	1,10	1,08	0,91	0,94	1,11 protein kinase-related
261546_at	At1g63520	1,06	0,98	1,12	1,01	1,07	0,98	0,93	0,91 expressed protein
261545_at	At1g63530	0,78	0,72	0,49	1,33	1,18	0,63	0,74	0,54 hypothetical protein
261544_at	At1g63540	0,78	0,60	0,62	1,04	1,04	0,98	1,07	0,93 hydroxyproline-rich glycoprotein family protein
261543_at	At1g63550	0,98	1,10	1,02	1,00	1,00	0,89	0,89	1,04 hypothetical protein
261542_at	At1g63560	0,91	1,00	1,11	1,00	1,05	1,04	0,93	1,28 receptor-like protein kinase-related
257485_at	At1g63580	0,95	1,13	1,08	1,03	1,20	0,90	0,98	0,94 protein kinase-related
257486_at	At1g63590	1,12	1,02	1,25	0,89	1,26	1,05	0,90	1,05 receptor-like protein kinase-related
261541_at	At1g63600	1,08	1,08	1,08	1,01	1,08	1,04	0,88	0,88 protein kinase-related
261540_at	At1g63610	1,13	1,00	0,99	0,99	1,16	1,10	1,03	1,09 expressed protein
261555_s_at	At1g63630	1,03	0,85	0,81	0,88	0,96	0,94	0,82	0,96 pentatricopeptide (PPR) repeat-containing protein
261557_at	At1g63640	0,83	0,75	0,76	1,24	0,91	0,98	1,05	0,82 kinesin motor protein-related
260242_at	At1g63650	0,98	0,97	0,99	0,83	1,12	0,95	1,02	1,09 basic helix-loop-helix (bHLH) family protein
260294_at	At1g63660	1,65	1,46	1,33	0,90	0,90	1,20	1,03	0,99 GMP synthase (glutamine-hydrolyzing), putative / glutamine amidotransfera
260293_at	At1g63670	0,94	0,97	1,13	0,76	1,13	1,03	1,33	1,13 expressed protein
260292_at	At1g63680	0,94	0,87	0,98	0,61	0,49	1,05	0,91	0,86 Mur ligase family protein
260271_at	At1g63690	0,80	0,77	0,83	0,92	0,84	0,94	0,96	0,90 protease-associated (PA) domain-containing protein
260291_at	At1g63700	0,83	0,95	1,23	1,01	1,08	0,79	1,11	1,37 protein kinase, putative
260241_at	At1g63710	1,08	0,89	0,94	0,90	1,08	0,97	1,10	1,02 cytochrome P450, putative
260243_at	At1g63720	0,94	1,18	0,91	0,85	1,05	1,26	1,11	1,28 expressed protein
260270_at	At1g63730	0,95	0,91	1,12	1,00	0,90	0,95	0,96	1,09 disease resistance protein (TIR-NBS-LRR class), putative
260269_at	At1g63740	0,65	0,84	1,12	0,80	0,78	0,95	1,00	1,01 disease resistance protein (TIR-NBS-LRR class), putative
260296_at	At1g63750	1,03	1,27	1,15	0,97	0,74	1,33	1,24	1,28 disease resistance protein (TIR-NBS-LRR class), putative
260295_at	At1g63770	0,94	0,81	0,83	0,99	1,05	1,09	1,06	1,02 peptidase M1 family protein
260323_at	At1g63780	1,65	1,46	1,30	1,26	1,18	1,15	0,94	0,90 brix domain-containing protein
260317_at	At1g63800	0,93	1,18	1,19	0,92	0,97	1,14	1,20	1,22 ubiquitin-conjugating enzyme 5 (UBC5)
260316_at	At1g63810	1,15	1,02	1,02	0,83	0,78	0,88	0,95	1,01 nucleolar RNA-associated family protein / Nrap family protein
260315_at	At1g63820	0,94	0,96	0,93	0,75	1,11	0,95	0,97	0,98 hypothetical protein
260314_at	At1g63830	0,65	0,54	0,56	0,92	0,89	0,85	0,93	1,00 proline-rich family protein
260327_at	At1g63840	0,84	1,09	0,64	1,26	1,12	1,10	1,01	0,82 zinc finger (C3HC4-type RING finger) family protein
257499_at	At1g63850	0,91	1,10	0,97	0,96	0,91	0,79	0,93	1,06 PRLI-interacting factor-related
260313_at	At1g63870	0,76	0,97	0,92	1,09	1,08	1,10	0,92	0,86 disease resistance protein (TIR-NBS-LRR class), putative
260312_at	At1g63880	1,09	1,16	2,09	0,77	0,51	1,23	1,94	1,52 disease resistance protein (TIR-NBS-LRR class), putative
260322_at	At1g63890	1,10	1,07	1,27	1,01	1,16	1,08	1,07	1,06 zinc finger (C3HC4-type RING finger) family protein
260321_at	At1g63900	1,11	1,09	1,25	0,99	0,86	1,09	0,99	1,00 zinc finger (C3HC4-type RING finger) family protein
260326_at	At1g63910	0,98	1,13	1,08	1,05	1,16	0,96	1,18	1,11 myb family transcription factor (MYB103)
260320_at	At1g63930	0,88	1,11	0,89	0,87	1,11	0,84	1,11	1,03 expressed protein
260325_at	At1g63940	0,69	0,66	0,65	1,17	1,10	0,81	0,82	0,84 monodehydroascorbate reductase, putative
260319_at	At1g63950	0,93	0,96	1,04	0,98	1,06	0,93	1,05	1,03 heavy-metal-associated domain-containing protein
260318_at	At1g63960	1,08	0,93	1,05	1,03	0,89	1,08	1,06	1,10 hypothetical protein
260324_at	At1g63970	1,04	0,92	0,91	0,95	0,90	0,98	0,88	0,94 2C-methyl-D-erythritol 2,4-cyclodiphosphate synthase, putative
262346_at	At1g63980	1,54	1,41	1,46	1,12	1,35	1,06	1,09	1,37 D111/G-patch domain-containing protein
262335_at	At1g63990	0,95	1,09	0,91	1,18	1,00	1,06	0,97	1,06 DNA topoisomerase VIA, putative (SPO11-2)
262339_at	At1g64000	0,95	0,99	1,07	1,06	0,97	1,03	1,03	1,07 WRKY family transcription factor
262334_at	At1g64010	0,88	1,06	0,89	0,97	1,19	0,96	1,15	0,83 serpin, putative / serine protease inhibitor, putative

262333_at	At1g64020	0,94	0,93	1,01	1,07	0,91	0,87	0,87	0,85 serpin-related / serine protease inhibitor-related
262332_at	At1g64030	0,97	1,04	1,00	1,07	1,08	1,10	0,97	0,94 serpin family protein / serine protease inhibitor family protein
262343_at	At1g64040	0,96	0,94	0,91	1,06	0,91	0,95	0,94	0,94 serine/threonine protein phosphatase PP1 isozyme 3 (TOPP3) / phosphopr
262331_at	At1g64050	1,04	0,96	1,27	0,77	0,75	0,98	0,89	0,90 expressed protein
262344_at	At1g64060	1,02	0,95	0,93	1,09	1,19	1,11	1,59	1,34 respiratory burst oxidase protein F (RbohF) (RbohAp108) / NADPH oxidase
262330_at	At1g64070	0,91	1,12	0,87	1,17	1,13	1,06	0,95	1,02 disease resistance protein (TIR-NBS-LRR class), putative
262329_at	At1g64090	1,04	0,98	0,89	1,20	1,08	0,91	0,82	0,90 reticulon family protein (RTNLB3)
262328_at	At1g64100	0,93	0,82	1,06	0,91	1,16	1,13	0,90	0,77 pentatricopeptide (PPR) repeat-containing protein
262340_at	At1g64105	0,97	0,90	0,78	1,02	1,22	0,95	0,93	0,91 no apical meristem (NAM) family protein
262347_at	At1g64110	0,45	0,41	0,32	0,83	0,79	1,06	1,13	1,21 AAA-type ATPase family protein
262327_at	At1g64130	0,93	1,01	0,99	0,91	1,08	1,06	0,98	1,12 hypothetical protein
262326_at	At1g64140	0,88	0,82	0,81	0,84	0,79	0,99	0,93	0,99 expressed protein
262342_at	At1g64150	0,80	0,67	0,62	0,71	0,65	1,14	1,09	1,13 expressed protein
262325_at	At1g64160	1,57	1,28	0,82	1,14	1,34	1,53	1,61	1,56 disease resistance-responsive family protein / dirigent family protein
262324_at	At1g64170	0,97	1,28	0,99	0,93	1,06	0,90	0,87	0,94 cation/hydrogen exchanger, putative (CHX16)
262345_at	At1g64180	1,04	1,10	1,01	0,83	1,03	1,01	1,05	0,97 intracellular protein transport protein USO1-related
262338_at	At1g64185	1,18	0,95	0,94	1,06	1,05	0,89	0,89	1,03 lactoylglutathione lyase family protein / glyoxalase I family protein
262323_at	At1g64190	0,99	0,99	1,04	1,35	1,44	0,77	0,84	0,73 6-phosphogluconate dehydrogenase family protein
262354_at	At1g64200	1,24	1,02	0,95	1,08	0,98	0,98	0,98	0,94 vacuolar ATP synthase subunit E, putative / V-ATPase E subunit, putative /
262353_at	At1g64210	0,94	1,00	1,09	0,95	0,86	1,09	1,16	1,13 leucine-rich repeat transmembrane protein kinase, putative
262336_at	At1g64220	1,13	1,05	1,08	1,28	1,34	0,99	0,84	0,92 preprotein translocase-related
262341_at	At1g64230	1,07	0,89	0,94	1,04	1,14	0,90	0,97	1,00 ubiquitin-conjugating enzyme, putative
262352_at	At1g64250	0,96	0,92	1,06	0,91	1,03	0,92	1,07	1,06 ---
262337_at	At1g64260	1,04	1,05	1,14	0,98	1,01	0,97	0,97	1,05 zinc finger protein-related
259796_at	At1g64270	0,87	1,00	1,17	0,98	0,83	0,96	1,02	0,95 Mutator-like transposase family
259764_at	At1g64280	0,93	1,08	1,25	1,19	1,51	1,07	1,13	0,89 regulatory protein (NPR1)
259795_at	At1g64290	1,00	1,04	1,11	1,03	1,04	0,98	1,07	0,84 F-box protein-related
259740_at	At1g64300	1,05	0,92	0,90	1,03	1,05	0,90	0,90	1,05 protein kinase family protein
259798_at	At1g64310	1,08	1,11	1,23	0,94	0,93	0,98	0,89	1,06 pentatricopeptide (PPR) repeat-containing protein
257508_at	At1g64320	1,01	0,93	0,95	0,99	1,10	1,01	0,98	0,92 myosin heavy chain-related
259794_at	At1g64330	0,99	0,68	0,98	1,25	1,11	1,12	1,13	1,20 myosin heavy chain-related
259739_at	At1g64350	0,99	1,00	0,94	0,87	0,74	0,97	0,91	1,00 transducin family protein / WD-40 repeat family protein
259738_at	At1g64355	1,10	1,12	1,36	1,01	1,03	1,32	1,03	1,27 expressed protein
259766_at	At1g64360	0,51	0,42	0,38	1,50	1,24	0,53	0,50	0,49 expressed protein
259765_at	At1g64370	1,22	1,20	1,23	1,22	1,28	0,82	0,86	0,85 expressed protein
259793_at	At1g64380	0,80	1,02	0,64	0,90	1,75	1,16	0,90	0,79 AP2 domain-containing transcription factor, putative
259736_at	At1g64390	1,36	1,89	1,05	1,16	1,44	0,88	0,91	0,79 endo-1,4-beta-glucanase, putative / cellulase, putative
259737_at	At1g64400	1,95	2,11	1,82	1,07	1,06	1,24	1,38	1,41 long-chain-fatty-acid--CoA ligase, putative / long-chain acyl-CoA synthetase
259735_at	At1g64405	0,93	1,23	0,84	1,11	1,35	1,08	1,04	1,06 expressed protein
259797_at	At1g64410	1,00	0,98	0,94	0,95	1,08	0,91	0,99	0,98 hypothetical protein
261952_at	At1g64430	0,75	0,72	0,65	0,70	0,68	1,01	0,94	0,88 expressed protein
261953_at	At1g64440	1,25	1,17	1,21	0,88	0,89	1,22	1,20	1,12 UDP-glucose 4-epimerase, putative / UDP-galactose 4-epimerase, putative
262002_at	At1g64450	0,88	0,88	0,87	1,16	1,23	0,82	0,86	0,96 proline-rich family protein
262003_at	At1g64460	1,24	1,18	1,01	1,00	1,12	0,78	1,01	0,77 phosphatidylinositol 3- and 4-kinase family protein
261947_at	At1g64470	1,39	1,10	0,88	1,34	1,09	0,88	1,01	1,02 ubiquitin family protein
262004_at	At1g64480	0,97	0,99	1,12	1,06	1,37	1,02	1,13	1,05 calcineurin B-like protein 8 (CBL8)

261951_at	At1g64490	1,89	1,27	1,55	1,41	1,55	1,05	0,91	0,88	expressed protein
261958_at	At1g64500	1,94	1,36	1,11	1,08	0,98	1,38	1,12	1,32	glutaredoxin family protein
261954_at	At1g64510	1,10	1,00	0,88	0,93	0,80	1,01	0,97	0,98	ribosomal protein S6 family protein
261955_at	At1g64520	1,19	1,11	1,16	1,02	1,14	1,04	0,91	0,85	26S proteasome regulatory subunit, putative (RPN12)
261945_at	At1g64530	0,84	0,94	0,98	1,03	1,07	1,16	1,01	1,03	RWP-RK domain-containing protein
262005_at	At1g64550	1,07	1,06	1,04	1,00	0,85	1,07	0,96	0,95	ABC transporter family protein
261946_at	At1g64560	0,94	0,87	1,00	0,92	0,83	1,00	1,02	1,31	---
262006_at	At1g64570	0,83	0,78	0,75	0,88	0,88	1,17	1,10	1,69	expressed protein
262007_at	At1g64580	0,95	0,91	0,80	1,02	1,13	0,93	0,92	0,94	pentatricopeptide (PPR) repeat-containing protein
261956_at	At1g64590	1,05	1,69	0,95	0,94	0,72	1,19	1,32	1,31	short-chain dehydrogenase/reductase (SDR) family protein
261972_at	At1g64600	1,37	1,61	1,58	0,95	1,12	1,01	1,06	1,29	expressed protein
261973_at	At1g64610	0,80	1,00	1,36	1,16	1,18	1,07	1,47	1,73	WD-40 repeat family protein
261950_at	At1g64620	0,87	1,06	1,00	2,07	1,55	0,69	0,90	1,16	Dof-type zinc finger domain-containing protein
261974_at	At1g64630	1,01	0,97	1,13	1,00	0,90	1,06	0,97	1,22	protein kinase family protein
261975_at	At1g64640	0,90	0,82	0,79	0,94	1,06	0,83	0,57	0,73	plastocyanin-like domain-containing protein
261944_at	At1g64650	0,89	0,95	0,72	1,23	0,94	0,82	0,75	0,72	expressed protein
261957_at	At1g64660	0,64	0,64	1,11	0,85	0,84	0,88	1,17	1,33	Cys/Met metabolism pyridoxal-phosphate-dependent enzyme family proteir
261949_at	At1g64670	1,13	1,19	1,56	1,31	1,63	0,88	0,81	0,83	hydrolase, alpha/beta fold family protein
261948_at	At1g64680	1,26	1,14	1,14	0,74	0,74	1,41	1,21	1,34	expressed protein
262872_at	At1g64690	0,92	1,03	0,99	1,01	1,10	1,03	1,20	0,88	expressed protein
262873_at	At1g64700	1,19	1,10	1,12	1,19	1,23	1,10	0,93	0,91	expressed protein
262870_at	At1g64710	1,00	1,17	1,30	0,77	0,81	1,07	1,21	1,13	alcohol dehydrogenase, putative
262884_at	At1g64720	1,06	1,16	1,21	0,86	0,91	1,05	1,08	1,29	expressed protein
262885_at	At1g64740	0,79	0,74	0,73	1,07	1,12	1,08	0,87	0,91	tubulin alpha-1 chain (TUA1)
262876_at	At1g64750	1,41	1,14	0,85	1,28	1,38	0,97	0,92	0,81	DSS1/SEM1 family protein
262878_at	At1g64770	1,03	1,03	1,04	0,90	0,82	1,20	1,16	1,11	expressed protein
262883_at	At1g64780	0,84	0,84	0,99	1,78	1,42	0,96	0,86	0,95	ammonium transporter 1, member 2 (AMT1.2)
262859_at	At1g64790	1,24	1,08	0,95	0,90	0,77	1,15	1,12	1,05	translational activator family protein
262917_at	At1g64800	1,09	0,95	1,02	1,05	1,12	0,86	1,06	0,94	expressed protein
262860_at	At1g64810	0,71	0,86	0,85	0,98	0,94	1,03	0,94	1,05	expressed protein
262861_at	At1g64820	1,04	0,82	0,89	0,96	1,12	0,95	0,83	0,88	MATE efflux family protein
262862_at	At1g64830	1,09	1,07	1,04	1,04	1,10	1,05	1,01	0,96	aspartyl protease family protein
262877_at	At1g64850	1,10	1,15	1,21	1,05	1,03	1,15	1,12	1,09	calcium-binding EF hand family protein
262879_at	At1g64860	1,43	1,69	2,32	0,43	0,55	1,28	1,28	1,32	RNA polymerase sigma subunit SigA (sigA) / sigma factor 1 (SIG1)
262880_at	At1g64880	1,25	1,03	0,93	1,02	0,96	1,05	1,07	1,00	ribosomal protein S5 family protein
262881_at	At1g64890	0,62	0,69	0,75	1,01	1,05	0,80	0,81	0,81	integral membrane transporter family protein
262882_at	At1g64900	0,64	0,66	0,62	0,63	0,76	0,70	0,70	0,68	cytochrome P450, putative
262863_at	At1g64910	0,97	0,99	1,02	0,97	1,03	1,02	1,06	0,95	glycosyltransferase family protein
262864_at	At1g64920	0,95	1,03	0,80	1,19	1,07	1,01	1,11	1,01	glycosyltransferase family protein
262865_at	At1g64930	0,88	0,99	0,87	1,04	0,95	0,94	0,98	1,08	cytochrome P450, putative
262866_at	At1g64940	1,12	0,91	1,00	1,01	1,04	0,98	0,97	1,04	cytochrome P450, putative
266155_at	At1g64950	0,64	0,57	0,92	0,86	0,97	0,85	0,75	0,73	cytochrome P450, putative
262867_at	At1g64960	0,87	1,06	0,95	0,84	0,97	1,06	0,90	1,13	expressed protein
262875_at	At1g64970	0,85	0,86	0,73	0,99	0,83	1,37	1,23	0,93	expressed protein
262868_at	At1g64980	1,23	1,10	1,33	1,21	1,00	1,20	1,23	1,24	expressed protein
262918_at	At1g65000	0,83	0,71	0,82	0,88	1,18	0,83	0,83	1,02	expressed protein

262871_at	At1g65010	1,22	0,90	0,92	1,32	0,98	1,05	1,14	1,07	expressed protein
262874_at	At1g65020	1,20	1,22	1,18	0,98	1,03	1,14	0,99	0,88	expressed protein
261904_at	At1g65040	1,13	1,12	0,80	1,13	0,85	1,01	0,93	0,83	zinc finger (C3HC4-type RING finger) family protein
261907_at	At1g65060	1,08	0,98	1,01	1,16	1,08	1,13	1,12	0,76	4-coumarate--CoA ligase 3 / 4-coumaroyl-CoA synthase 3 (4CL3)
261905_at	At1g65070	1,03	1,01	1,39	0,70	0,74	1,38	1,39	1,25	DNA mismatch repair MutS family protein
261906_at	At1g65080	0,97	1,02	0,85	0,89	1,03	0,90	1,01	0,88	OXA1 family protein
263138_at	At1g65090	0,74	0,82	0,52	0,82	1,02	0,90	0,97	1,16	expressed protein
263139_at	At1g65110	0,99	1,05	1,04	0,96	0,99	1,12	0,96	0,88	ubiquitin carboxyl-terminal hydrolase-related
263140_at	At1g65120	1,11	0,96	0,94	0,96	0,90	1,15	1,06	1,05	ubiquitin carboxyl-terminal hydrolase-related
257453_at	At1g65130	0,93	0,97	1,04	0,99	0,87	0,89	0,99	1,01	ubiquitin carboxyl-terminal hydrolase-related
257454_at	At1g65170	0,95	1,01	1,03	1,02	1,01	0,94	1,00	1,01	ubiquitin carboxyl-terminal hydrolase family protein
263109_at	At1g65180	0,98	0,96	1,06	1,02	1,15	0,99	0,98	1,05	DC1 domain-containing protein
263111_s_at	At1g65190	1,01	0,83	0,86	0,74	0,69	1,12	0,90	0,93	protein kinase family protein
263141_at	At1g65210	0,97	0,97	1,07	1,06	1,08	0,99	0,94	0,98	hypothetical protein
263110_at	At1g65220	0,89	0,93	0,86	0,75	0,92	1,05	0,98	0,94	eIF4-gamma/eIF5/eIF2-epsilon domain-containing protein
263142_at	At1g65230	0,94	0,87	0,87	0,75	0,72	1,29	1,08	1,01	expressed protein
263108_at	At1g65240	1,17	1,08	0,88	0,84	1,26	1,04	0,83	0,67	aspartyl protease family protein
264158_at	At1g65260	0,99	0,90	0,93	0,98	1,05	0,95	1,02	1,06	PspA/IM30 family protein
264159_at	At1g65270	1,14	1,13	0,94	1,28	1,21	1,00	0,86	0,85	expressed protein
264156_at	At1g65280	1,01	1,06	1,47	0,97	0,95	1,11	1,15	1,24	DNAJ heat shock N-terminal domain-containing protein
264162_at	At1g65290	1,45	1,28	1,08	1,39	1,32	1,02	1,02	0,99	acyl carrier family protein / ACP family protein
264164_at	At1g65295	1,33	1,23	1,15	1,06	0,92	0,80	0,83	0,75	expressed protein
264157_at	At1g65310	1,54	1,47	1,18	0,88	1,05	1,18	1,01	0,92	xyloglucan:xyloglucosyl transferase, putative / xyloglucan endotransglycosy
264214_s_at	At1g65330	0,87	0,90	0,88	1,05	1,15	0,88	1,03	0,94	MADS-box protein (AGL38)
264215_at	At1g65340	1,03	0,99	0,97	1,00	1,03	0,88	1,01	1,11	cytochrome P450, putative
264181_at	At1g65350	0,82	0,88	0,84	0,73	0,83	1,05	1,03	0,96	polyubiquitin, putative
264182_at	At1g65360	0,91	1,20	1,02	1,00	1,08	1,06	1,04	0,93	MADS-box protein (AGL23)
264166_at	At1g65370	1,35	1,25	1,27	1,58	1,75	0,87	0,94	0,83	meprin and TRAF homology domain-containing protein / MATH domain-cor
264183_at	At1g65380	0,88	0,80	0,92	1,32	1,22	0,93	0,85	0,79	receptor-like protein CLAVATA2 (CLV2)
264153_at	At1g65390	0,92	1,12	1,68	0,69	0,71	1,35	1,10	1,06	disease resistance protein (TIR class), putative
264213_at	At1g65400	0,89	1,02	2,40	0,64	0,79	1,37	1,06	1,00	disease resistance protein (TIR class), putative
264165_at	At1g65410	0,79	0,73	0,88	0,90	0,90	0,97	1,05	1,09	ABC transporter family protein
264161_at	At1g65420	0,84	0,98	1,12	0,78	0,83	1,12	0,95	0,83	expressed protein
264154_at	At1g65430	0,85	0,88	0,81	0,91	1,03	0,98	0,94	0,92	zinc finger protein-related
264155_at	At1g65440	1,07	0,94	0,97	0,90	1,20	1,02	1,20	1,22	glycine-rich protein
264163_at	At1g65445	1,02	0,98	0,93	0,98	1,06	1,19	0,93	1,07	transferase-related
264160_at	At1g65450	1,01	0,98	0,91	0,90	0,92	1,45	1,07	1,45	transferase family protein
264630_at	At1g65470	1,17	1,15	1,13	1,09	0,97	1,12	1,01	1,15	chromatin assembly factor-1 (FASCIATA1) (FAS1)
264638_at	At1g65480	1,20	1,42	1,86	4,75	5,43	1,11	0,67	0,62	flowering locus T protein (FT)
264636_at	At1g65490	0,48	0,52	0,67	1,02	1,02	1,28	0,92	0,82	expressed protein
264635_at	At1g65500	0,75	0,69	0,65	1,38	1,41	1,84	1,41	1,34	expressed protein
264680_at	At1g65510	0,69	0,64	0,69	1,26	1,22	0,91	0,93	0,86	expressed protein
264627_at	At1g65520	0,85	0,87	1,07	0,97	1,01	0,87	0,68	0,79	enoyl-CoA hydratase/isomerase family protein
264628_at	At1g65530	1,33	1,09	1,10	0,91	0,90	1,07	1,07	1,23	calcium-binding EF hand family protein
264629_at	At1g65540	1,17	0,96	1,03	1,08	1,28	1,05	1,02	1,03	calcium-binding EF hand family protein
264637_at	At1g65560	0,81	0,73	1,11	0,97	1,01	1,13	1,06	1,17	allyl alcohol dehydrogenase, putative

264682_at	At1g65570	0,94	0,96	0,97	1,76	1,48	1,05	1,08	0,96	polygalacturonase, putative / pectinase, putative
264683_at	At1g65580	1,07	1,13	1,42	1,12	1,44	1,18	1,30	1,30	endonuclease/exonuclease/phosphatase family protein
264684_at	At1g65590	1,16	1,22	1,03	1,03	0,95	1,28	1,30	1,32	glycosyl hydrolase family 20 protein
264685_at	At1g65610	0,77	0,87	0,71	1,28	1,27	1,22	0,86	1,01	endo-1,4-beta-glucanase, putative / cellulase, putative
264626_at	At1g65620	0,99	1,02	0,97	1,02	0,99	1,04	0,92	1,05	LOB domain protein 6 / lateral organ boundaries domain protein 6 (LBD6) /
264631_at	At1g65630	0,94	0,97	0,97	0,95	0,95	1,02	1,02	1,16	DegP protease, putative
264632_at	At1g65640	0,97	1,07	1,00	0,93	1,08	0,94	0,95	0,90	DegP protease, putative
264639_at	At1g65650	1,32	1,23	1,33	1,26	1,15	1,13	1,17	1,19	ubiquitin carboxyl-terminal hydrolase family 1 protein
264633_at	At1g65660	1,01	0,89	1,07	0,85	0,86	1,09	1,27	1,60	zinc knuckle (CCHC-type) family protein
264634_at	At1g65670	0,97	0,96	0,97	1,00	0,97	1,01	1,04	1,12	cytochrome P450 family protein
264640_at	At1g65680	0,99	1,20	0,95	0,97	1,00	1,05	1,14	0,95	beta-expansin, putative (EXBP2)
262930_at	At1g65690	1,00	1,21	0,97	1,06	0,88	9,90	4,57	2,21	harpin-induced protein-related / HIN1-related / harpin-responsive protein-re
262931_at	At1g65700	1,17	0,96	0,91	0,75	0,84	0,26	0,27	0,27	small nuclear ribonucleoprotein, putative / snRNP, putative / Sm protein, pu
262929_at	At1g65720	1,12	1,00	0,95	1,17	1,18	0,90	0,87	0,85	expressed protein
262925_at	At1g65730	0,75	0,69	0,67	0,97	1,03	1,08	1,13	1,09	oligopeptide transporter OPT family protein
257462_at	At1g65740	0,94	1,06	0,97	0,93	0,93	1,09	0,94	0,88	F-box family protein
262928_at	At1g65760	1,02	0,95	0,95	1,00	1,04	0,93	0,92	1,06	F-box family protein
262926_s_at	At1g65790	0,74	0,79	1,13	0,80	0,69	1,67	1,60	1,26	S-receptor protein kinase, putative
262927_at	At1g65810	0,91	0,94	1,06	0,98	1,02	0,96	1,04	1,18	tRNA-splicing endonuclease positive effector-related
262932_at	At1g65820	1,08	1,01	0,89	1,23	0,95	1,10	0,95	0,99	microsomal glutathione s-transferase, putative
262933_at	At1g65840	1,02	0,82	0,80	1,10	1,07	1,25	0,96	0,94	amine oxidase family protein
261968_at	At1g65850	0,93	1,01	1,01	1,02	0,93	1,04	0,99	0,95	disease resistance protein (TIR-NBS-LRR class), putative
261913_at	At1g65860	1,36	1,43	1,83	1,81	1,30	1,07	0,92	0,93	flavin-containing monooxygenase family protein / FMO family protein
261914_at	At1g65870	0,94	0,92	0,94	1,76	1,19	1,40	0,98	1,08	disease resistance-responsive family protein
261915_at	At1g65880	0,99	0,97	0,99	1,05	1,12	0,86	1,08	0,94	AMP-dependent synthetase and ligase family protein
261922_at	At1g65890	0,82	0,83	1,11	0,96	1,17	0,96	0,96	1,19	acyl-activating enzyme 12 (AAE12)
261921_at	At1g65900	1,04	0,88	0,92	0,98	0,97	0,96	0,99	0,65	expressed protein
261916_at	At1g65910	1,01	1,13	1,02	1,19	0,99	1,16	1,03	1,14	no apical meristem (NAM) family protein
261917_at	At1g65920	0,98	0,89	0,88	0,92	0,95	1,05	1,12	1,07	regulator of chromosome condensation (RCC1) family protein / zinc finger p
261920_at	At1g65930	1,14	1,07	1,11	1,15	1,11	1,06	1,12	1,05	isocitrate dehydrogenase, putative / NADP+ isocitrate dehydrogenase, puta
261918_at	At1g65940	0,92	0,85	1,01	1,01	1,01	0,93	1,04	0,97	---
261969_at	At1g65950	1,14	1,09	1,12	0,88	0,72	1,20	1,28	1,39	ABC1 family protein
261970_at	At1g65960	0,95	0,90	0,92	1,08	1,11	0,88	0,95	0,98	glutamate decarboxylase 2 (GAD 2)
261919_at	At1g65980	0,95	0,91	0,78	1,27	1,15	0,90	0,91	0,99	peroxiredoxin type 2, putative
261971_at	At1g65990	1,03	1,10	0,91	1,11	0,97	1,03	0,84	1,03	type 2 peroxiredoxin-related / thiol specific antioxidant / mal allergen family
261912_s_at	At1g66000	0,94	1,03	0,98	1,06	1,09	1,02	0,99	1,05	expressed protein
256515_at	At1g66020	1,08	0,98	1,04	1,01	0,89	1,11	1,04	1,03	terpene synthase/cyclase family protein
256520_at	At1g66030	1,04	0,97	1,02	1,00	0,96	1,01	1,00	1,16	cytochrome P450-related
256523_at	At1g66070	1,13	0,91	0,85	1,00	0,97	1,10	0,97	1,13	translation initiation factor-related
256518_at	At1g66080	1,13	1,11	1,03	1,26	1,22	0,92	0,92	0,94	expressed protein
256526_at	At1g66090	1,53	3,12	2,14	0,60	0,61	2,00	1,97	1,74	disease resistance protein (TIR-NBS class), putative
256527_at	At1g66100	1,17	1,00	0,92	0,89	0,63	1,48	1,08	1,07	thionin, putative
256519_at	At1g66110	1,01	1,04	0,74	0,99	1,06	1,03	0,99	0,91	hypothetical protein
256521_at	At1g66120	1,04	0,91	1,01	0,97	1,13	1,09	0,97	1,13	acyl-activating enzyme 11 (AAE11)
256514_at	At1g66130	1,42	1,52	1,97	0,96	0,75	1,45	1,35	1,16	oxidoreductase N-terminal domain-containing protein
256528_at	At1g66140	1,06	0,99	0,91	1,02	0,89	0,99	1,10	1,28	zinc finger (C2H2 type) family protein

256516_at	At1g66150	1,04	0,91	1,06	0,77	0,83	1,04	0,97	1,18	leucine-rich repeat protein kinase, putative (TMK1)
256522_at	At1g66160	1,05	1,35	0,82	0,86	1,26	1,17	1,21	1,18	U-box domain-containing protein
256517_at	At1g66170	0,97	1,01	0,94	0,99	1,03	0,87	1,02	1,00	PHD finger family protein (MMD1)
256525_at	At1g66180	0,81	0,90	0,77	0,80	0,70	0,85	0,85	1,04	aspartyl protease family protein
256524_at	At1g66200	1,05	1,04	1,15	1,07	0,95	1,11	1,08	1,18	glutamine synthetase, putative
259820_at	At1g66210	1,06	1,02	1,01	0,99	1,11	1,01	1,03	1,13	subtilase family protein
259821_at	At1g66220	0,87	1,05	0,94	0,91	0,98	0,97	0,87	0,82	subtilase family protein
259822_at	At1g66230	1,31	1,27	1,13	0,97	1,08	1,05	1,11	1,10	myb family transcription factor (MYB20)
259824_at	At1g66240	1,06	0,95	0,92	1,36	1,39	0,99	0,93	1,01	copper homeostasis factor, putative / copper chaperone, putative (CCH)
259823_at	At1g66250	1,01	1,08	1,05	1,33	1,09	0,94	1,02	0,90	glycosyl hydrolase family 17 protein
259825_at	At1g66260	0,87	0,82	1,03	1,05	1,00	0,96	1,03	1,12	RNA and export factor-binding protein, putative
260130_s_at	At1g66280	1,05	0,96	0,73	2,10	1,81	1,21	1,29	1,20	beta-glucosidase (PSR3.2)
260131_at	At1g66310	0,94	0,96	1,03	1,05	1,14	1,05	1,05	0,97	F-box family protein
260137_at	At1g66330	1,64	2,08	2,50	0,88	0,98	1,77	1,99	2,30	senescence-associated family protein
260133_at	At1g66340	0,94	1,02	1,00	0,98	1,09	1,11	1,00	1,05	ethylene receptor 1 (ETR1)
260141_at	At1g66350	0,87	0,83	0,83	0,96	0,79	0,57	0,61	0,78	gibberellin regulatory protein (RGL1)
260136_at	At1g66360	1,02	1,06	1,22	1,04	1,10	0,96	0,90	0,91	C2 domain-containing protein
260134_at	At1g66370	1,00	1,05	0,85	0,94	1,01	1,02	1,11	0,88	myb family transcription factor (MYB113)
260139_at	At1g66380	0,98	1,00	1,04	0,93	0,90	1,10	0,97	1,10	myb family transcription factor (MYB114)
260140_at	At1g66390	0,94	1,08	0,87	1,15	1,10	0,99	0,92	1,32	myb family transcription factor, putative / production of anthocyanin pigmen
260135_at	At1g66400	1,19	1,20	0,90	0,75	0,76	1,40	1,05	0,80	calmodulin-related protein, putative
260138_at	At1g66410	1,02	0,89	0,87	1,25	1,31	0,95	0,93	1,02	calmodulin-1/4 (CAM4)
260107_at	At1g66430	0,55	0,56	0,48	0,88	0,84	0,84	0,86	0,86	pfkB-type carbohydrate kinase family protein
256360_at	At1g66440	1,20	1,19	1,10	1,11	1,21	1,11	0,93	1,00	DC1 domain-containing protein
256362_at	At1g66450	0,99	0,95	0,87	1,07	1,00	0,97	0,99	1,07	DC1 domain-containing protein
256359_at	At1g66460	1,04	1,03	1,04	1,09	0,94	0,97	1,03	0,95	protein kinase family protein
256358_at	At1g66470	1,00	1,08	0,98	1,08	1,21	1,00	1,10	0,95	basic helix-loop-helix (bHLH) family protein
257583_at	At1g66480	0,78	1,07	1,16	1,13	1,05	0,96	1,39	1,37	expressed protein
256357_at	At1g66490	0,99	1,04	0,99	1,01	1,04	1,02	1,06	1,05	F-box family protein
256356_s_at	At1g66500	1,77	0,92	0,98	0,54	0,97	1,84	1,33	1,70	zinc finger (C2H2-type) family protein
256363_at	At1g66510	0,93	1,06	1,43	1,12	0,95	0,90	1,08	1,15	AAR2 protein family
256361_at	At1g66520	0,87	0,92	0,88	0,88	0,82	1,06	0,90	0,92	formyltransferase, putative
256365_at	At1g66530	1,06	0,94	0,90	1,08	1,06	1,07	0,99	0,99	arginyl-tRNA synthetase, putative / arginine--tRNA ligase, putative
256386_at	At1g66540	1,31	1,65	2,19	0,90	0,81	1,19	1,25	1,15	cytochrome P450, putative
256364_at	At1g66550	1,00	0,99	0,93	0,91	1,36	1,23	0,89	1,38	WRKY family transcription factor
256407_at	At1g66570	0,87	0,91	1,07	0,98	1,08	1,15	0,99	0,92	sucrose transporter, putative / sucrose-proton symporter, putative
256385_at	At1g66580	0,86	0,87	0,95	1,13	1,03	0,99	0,96	0,99	60S ribosomal protein L10 (RPL10C)
256408_at	At1g66610	0,94	0,98	1,02	0,95	1,34	0,91	0,90	1,03	seven in absentia (SINA) protein, putative
256409_at	At1g66620	1,21	1,13	1,26	1,56	1,04	0,91	0,90	0,80	seven in absentia (SINA) protein, putative
256410_at	At1g66630	0,87	0,97	0,92	0,93	1,13	1,08	0,99	1,01	seven in absentia (SINA) family protein
260132_s_at	At1g66640	1,00	1,00	1,05	0,92	1,19	0,98	0,91	0,98	F-box family protein
256377_at	At1g66645	0,99	1,07	1,08	1,15	1,45	0,98	0,79	1,37	seven in absentia (SINA) protein, putative
256384_at	At1g66660	1,04	1,00	0,98	0,95	0,96	0,99	0,93	1,12	seven in absentia (SINA) protein, putative
256411_at	At1g66670	1,07	1,00	1,08	0,90	0,98	1,20	1,08	1,12	ATP-dependent Clp protease proteolytic subunit (ClpP3)
256380_at	At1g66680	0,97	0,94	1,01	1,05	0,97	0,98	0,93	1,00	S locus-linked protein, putative
256376_s_at	At1g66690	1,66	2,07	1,47	0,65	0,78	1,07	1,02	0,92	S-adenosyl-L-methionine:carboxyl methyltransferase family protein

256375_at	At1g66720	0,95	0,98	0,96	1,04	1,05	0,97	0,94	0,86	S-adenosyl-L-methionine:carboxyl methyltransferase family protein
256374_at	At1g66730	1,03	1,02	0,98	1,02	0,99	1,04	1,11	1,32	ATP dependent DNA ligase family protein
256373_at	At1g66740	1,16	1,09	1,45	1,15	1,10	1,01	0,90	1,16	ASF1-like anti-silencing protein, putative
256372_at	At1g66750	1,79	1,60	1,99	1,02	1,15	1,11	1,05	1,17	cell division protein kinase, putative
256324_at	At1g66760	0,55	0,60	0,69	0,90	0,88	0,96	1,21	1,15	MATE efflux family protein
256371_at	At1g66770	0,98	1,10	0,92	1,13	1,03	1,12	0,95	1,05	nodulin MtN3 family protein
256370_at	At1g66780	1,02	0,92	0,96	0,91	1,09	0,97	1,01	0,99	MATE efflux family protein
256369_at	At1g66790	1,04	1,02	0,97	1,02	1,05	0,91	1,08	0,99	hypothetical protein
256368_at	At1g66800	0,83	0,70	0,60	1,38	1,28	0,87	1,01	0,98	cinnamyl-alcohol dehydrogenase family / CAD family
256367_at	At1g66810	1,09	1,17	0,99	1,05	0,99	1,00	0,98	1,07	zinc finger (CCCH-type) family protein
256383_at	At1g66820	0,80	0,65	0,63	0,79	0,81	0,99	0,75	0,67	glycine-rich protein
256378_at	At1g66830	1,21	1,12	1,06	0,89	1,04	1,16	0,90	0,94	leucine-rich repeat transmembrane protein kinase, putative
256379_at	At1g66840	1,16	1,09	1,08	0,78	0,84	1,29	1,09	1,11	expressed protein
256381_at	At1g66850	0,93	1,02	0,91	0,85	1,30	0,89	0,86	1,11	protease inhibitor/seed storage/lipid transfer protein (LTP) family protein
256382_at	At1g66860	0,74	0,89	1,70	0,96	1,05	0,98	0,97	0,91	expressed protein
256366_at	At1g66880	1,14	1,29	1,47	1,60	1,38	1,26	1,22	1,04	serine/threonine protein kinase family protein
245761_at	At1g66890	0,97	1,06	1,07	0,80	0,82	0,79	0,72	0,91	expressed protein
245759_at	At1g66900	0,98	1,20	1,14	1,13	1,08	1,02	0,98	0,89	expressed protein
245760_s_at	At1g66920	0,95	1,08	0,98	0,70	0,91	1,20	0,99	0,96	protein kinase, putative
255911_at	At1g66930	0,98	0,95	0,91	1,00	1,10	1,00	1,01	1,00	serine/threonine protein kinase family protein
255856_at	At1g66940	1,23	0,99	0,90	1,20	1,02	0,75	0,70	0,72	protein kinase-related
255855_at	At1g66950	0,90	0,91	0,96	0,90	0,89	0,94	1,05	0,83	ABC transporter family protein
255912_at	At1g66960	0,92	1,03	1,01	1,00	1,08	1,05	1,19	1,10	lupeol synthase, putative / 2,3-oxidosqualene-triterpenoid cyclase, putative
255852_at	At1g66970	0,96	0,84	0,77	0,73	0,80	1,31	1,16	1,11	glycerophosphoryl diester phosphodiesterase family protein
255913_at	At1g66980	1,10	1,17	1,97	0,79	0,73	1,40	1,59	1,70	protein kinase family protein / glycerophosphoryl diester phosphodiesterase
255879_at	At1g67000	0,99	0,96	1,02	0,88	0,94	0,97	1,06	1,15	protein kinase family protein
255853_at	At1g67020	0,95	1,00	0,94	0,93	0,96	1,01	1,01	1,08	hypothetical protein
255858_at	At1g67030	0,98	0,98	0,94	0,92	1,00	1,06	1,18	1,06	zinc finger (C2H2 type) family protein (ZFP6)
255851_at	At1g67040	1,14	1,13	0,94	1,10	1,10	0,97	1,03	0,92	expressed protein
255854_at	At1g67050	0,94	1,03	1,00	0,75	0,80	0,88	0,99	0,76	expressed protein
255880_at	At1g67060	0,79	0,73	0,57	1,03	1,13	0,87	0,91	0,92	expressed protein
255881_at	At1g67070	1,38	1,40	1,46	0,88	1,08	0,98	1,10	1,07	phosphomannose isomerase, putative (DIN9)
255857_at	At1g67080	1,02	0,93	1,01	1,08	1,04	1,00	0,97	0,93	expressed protein
264474_s_at	At1g67090	1,03	0,93	1,00	0,96	1,03	1,01	1,00	0,97	ribulose bisphosphate carboxylase small chain 1A / RuBisCO small subunit
264469_at	At1g67100	0,73	0,86	0,98	0,86	0,95	0,91	0,98	0,84	LOB domain protein 40 / lateral organ boundaries domain protein 40 (LBD4
264470_at	At1g67110	1,09	0,97	1,04	1,50	1,64	1,03	1,44	1,55	cytochrome P450, putative
264471_at	At1g67120	1,04	0,85	1,08	0,43	0,56	1,10	1,28	1,31	midasin-related
264472_at	At1g67140	0,74	0,82	0,82	0,79	0,71	1,12	1,15	1,00	expressed protein
264473_at	At1g67180	1,09	0,97	1,03	1,08	1,17	0,81	0,84	0,99	zinc finger (C3HC4-type RING finger) family protein / BRCT domain-contair
264971_at	At1g67210	0,93	1,03	0,98	0,85	0,99	0,94	0,99	0,99	proline-rich spliceosome-associated (PSP) family protein / zinc knuckle (CC
264997_at	At1g67220	1,20	1,29	1,23	0,85	1,16	0,97	1,10	0,80	zinc finger protein-related
264996_at	At1g67230	0,94	0,90	1,20	0,77	0,84	1,07	1,37	1,84	expressed protein
264995_at	At1g67240	0,98	1,04	0,89	0,98	1,04	1,06	0,97	0,91	---
264967_at	At1g67250	1,35	1,14	1,03	1,20	1,16	0,96	0,97	0,99	proteasome maturation factor UMP1 family protein
264994_at	At1g67270	0,94	1,00	0,97	0,97	1,03	0,99	1,00	1,00	hypothetical protein
264970_at	At1g67280	0,93	0,93	0,90	0,83	0,83	1,07	1,15	1,07	lactoylglutathione lyase, putative / glyoxalase I, putative

264993_at	At1g67290	1,00	1,05	0,94	1,03	1,11	0,96	0,92	0,88	glyoxal oxidase-related
264992_at	At1g67300	0,56	0,65	0,63	0,81	0,82	0,93	0,94	0,85	hexose transporter, putative
264999_at	At1g67310	0,91	1,12	1,46	1,00	0,94	0,98	1,18	1,45	calmodulin-binding protein
264969_at	At1g67320	1,02	0,94	1,06	1,20	1,10	0,94	0,91	0,76	DNA primase, large subunit family
264998_at	At1g67330	0,90	1,00	0,80	2,21	2,29	0,90	0,89	0,80	expressed protein
264942_at	At1g67340	2,16	1,67	1,62	1,10	0,99	0,96	1,22	1,12	zinc finger (MYND type) family protein / F-box family protein
265001_at	At1g67350	1,13	1,00	0,88	1,26	1,21	0,88	0,94	0,90	expressed protein
264968_at	At1g67360	0,87	0,91	0,94	1,65	1,64	1,18	1,16	1,20	rubber elongation factor (REF) family protein
264972_at	At1g67370	0,84	0,92	0,78	0,90	1,10	0,94	0,97	0,96	meiotic asynaptic mutant 1 (ASY1)
265000_at	At1g67410	1,08	1,12	1,20	0,88	1,00	0,87	0,97	1,15	exostosin family protein
264225_at	At1g67420	0,90	0,93	0,90	1,17	0,92	1,14	1,00	1,00	24 kDa vacuolar protein, putative
264233_at	At1g67430	1,28	1,10	1,08	1,30	1,24	0,93	0,97	0,92	60S ribosomal protein L17 (RPL17B)
264224_at	At1g67440	1,27	1,23	1,34	0,81	0,64	1,16	0,95	1,01	expressed protein
264231_at	At1g67450	0,98	1,08	1,51	1,12	0,98	1,00	0,91	0,87	F-box family protein
264230_at	At1g67460	0,99	1,04	1,11	1,02	0,86	1,09	1,16	1,42	hypothetical protein
264232_at	At1g67470	0,87	0,91	0,85	1,06	1,32	0,84	0,84	0,95	protein kinase family protein
264229_at	At1g67480	1,15	1,43	1,49	0,90	1,08	1,01	0,94	0,91	kelch repeat-containing F-box family protein
264228_at	At1g67490	0,96	0,93	0,88	1,02	0,86	1,20	1,04	1,29	alpha-glucosidase I (GCS1) / KNOPF (KNF)
264227_at	At1g67500	0,87	1,05	1,04	0,96	1,11	1,07	0,95	0,96	DNA polymerase family B protein
264226_at	At1g67510	0,72	0,81	0,95	0,79	1,03	0,86	0,88	0,80	leucine-rich repeat family protein
264223_s_at	At1g67520	0,62	0,82	0,81	0,82	0,62	1,47	1,60	1,38	lectin protein kinase family protein
260194_at	At1g67530	1,03	1,55	1,61	0,81	1,02	1,19	1,54	2,32	armadillo/beta-catenin repeat family protein / U-box domain-containing fami
260195_at	At1g67540	0,86	0,97	0,99	0,93	1,07	0,93	0,99	0,91	expressed protein
260189_at	At1g67550	0,81	0,73	0,68	0,71	0,78	1,02	1,00	0,95	urease, putative / urea amidohydrolase, putative
260190_at	At1g67560	0,75	0,74	0,90	1,00	0,81	0,88	1,00	1,11	lipoxygenase family protein
260196_at	At1g67570	0,95	1,01	0,91	1,08	1,20	1,02	1,08	1,11	expressed protein
260191_at	At1g67580	0,98	1,05	1,18	0,84	0,82	1,04	1,22	1,16	protein kinase family protein
260199_at	At1g67590	1,05	1,03	0,96	0,98	1,08	0,92	0,99	1,08	remorin family protein
260201_at	At1g67600	0,90	0,99	0,94	1,05	0,99	1,07	1,03	0,92	expressed protein
260200_at	At1g67620	1,11	1,10	1,00	1,11	1,43	0,99	0,89	0,89	expressed protein
260197_at	At1g67623	0,99	0,93	1,02	0,89	0,96	1,01	1,01	1,08	F-box family protein
260192_at	At1g67630	1,42	1,56	1,60	1,11	1,41	1,05	1,06	0,92	DNA polymerase alpha subunit B family
260198_at	At1g67635	0,92	1,11	1,04	1,02	1,08	1,04	0,98	1,04	hypothetical protein
260193_at	At1g67640	0,92	0,98	1,12	0,85	1,06	1,08	1,01	1,06	lysine and histidine specific transporter, putative
245188_at	At1g67660	0,87	0,94	1,08	0,70	0,83	1,03	1,01	1,04	expressed protein
245189_at	At1g67670	1,00	0,95	0,90	0,93	1,00	0,97	1,11	0,90	hypothetical protein
245187_s_at	At1g67680	1,17	1,05	0,94	1,13	1,30	1,12	0,97	0,96	expressed protein
245190_at	At1g67690	0,95	0,98	1,13	0,98	0,92	1,05	0,89	0,82	peptidase M3 family protein / thimet oligopeptidase family protein
245198_at	At1g67700	1,04	0,96	1,00	0,87	0,92	1,23	1,21	1,18	expressed protein
245186_at	At1g67710	1,05	1,02	1,01	0,74	0,99	1,02	1,16	1,01	two-component responsive regulator family protein / response regulator fam
245202_at	At1g67720	0,89	0,93	1,01	0,88	0,89	0,94	1,23	1,26	leucine-rich repeat family protein / protein kinase family protein
245199_at	At1g67730	1,02	1,02	1,01	1,12	1,04	0,93	0,93	0,99	b-keto acyl reductase, putative (GLOSSY8)
245195_at	At1g67740	1,10	1,13	1,09	1,06	1,04	1,04	0,94	1,04	photosystem II core complex proteins psbY, chloroplast (PSBY) / L-arginine
245196_at	At1g67750	2,25	1,87	1,46	2,49	1,34	0,98	0,98	1,02	pectate lyase family protein
245185_at	At1g67760	1,17	1,10	1,06	1,19	1,39	0,87	0,94	0,98	T-complex protein 1 epsilon subunit, putative / TCP-1-epsilon, putative / cha
245191_at	At1g67770	1,05	0,87	0,92	0,87	0,81	1,11	1,04	0,90	RNA-binding protein, putative

245192_at	At1g67780	1,01	0,96	1,06	1,03	1,12	1,05	0,99	0,86	hypothetical protein
245184_at	At1g67790	0,90	0,94	1,11	0,91	0,97	0,97	0,96	0,93	expressed protein
245197_at	At1g67800	0,93	1,00	1,00	1,04	0,89	1,15	1,18	1,19	copine-related
245193_at	At1g67810	0,72	0,70	1,07	0,94	1,04	0,75	0,83	0,74	Fe-S metabolism associated domain-containing protein
245194_at	At1g67820	0,99	1,05	0,90	0,83	0,92	0,95	0,86	1,04	protein phosphatase 2C, putative / PP2C, putative
245215_at	At1g67830	0,78	1,02	1,31	1,20	1,26	0,96	0,81	0,93	GDSL-motif lipase/hydrolase family protein
245201_at	At1g67840	1,09	1,08	1,30	0,74	0,67	1,36	1,41	1,76	ATP-binding region, ATPase-like domain-containing protein
245200_at	At1g67850	0,82	0,79	1,02	1,54	1,24	1,07	1,20	1,12	expressed protein
245214_at	At1g67855	1,02	0,93	0,94	0,89	1,31	0,97	1,04	0,81	hypothetical protein
260004_at	At1g67860	0,78	0,67	0,66	1,91	1,48	0,63	0,74	0,72	expressed protein
260012_at	At1g67865	0,95	0,83	0,74	2,95	3,18	0,73	0,78	0,84	expressed protein
260007_at	At1g67870	0,80	0,78	0,72	0,94	0,90	0,67	0,88	0,90	glycine-rich protein
259997_at	At1g67880	0,82	1,06	1,18	0,86	0,86	1,07	1,33	1,30	glycosyl transferase family 17 protein
259957_at	At1g67890	0,88	0,76	1,29	0,85	0,82	1,27	1,18	1,47	protein kinase family protein
259996_at	At1g67910	1,49	1,94	1,59	1,20	1,15	0,99	0,85	1,00	expressed protein
260005_at	At1g67920	1,15	1,40	1,28	1,04	1,01	1,75	1,40	1,34	expressed protein
259995_at	At1g67930	0,95	0,84	0,91	1,17	1,23	1,13	0,79	0,70	Golgi transport complex protein-related
260002_at	At1g67940	1,35	1,60	1,04	1,96	1,62	1,03	0,91	1,21	ABC transporter family protein
260009_at	At1g67950	0,93	0,87	0,92	1,41	1,30	0,99	0,85	0,93	RNA recognition motif (RRM)-containing protein
259993_at	At1g67960	0,85	0,85	1,01	0,73	0,81	1,06	0,99	0,82	expressed protein
259992_at	At1g67970	0,87	1,36	1,14	0,78	0,94	0,90	1,14	1,03	heat shock factor protein, putative (HSF5) / heat shock transcription factor,
260015_at	At1g67980	1,31	1,79	1,45	0,40	0,42	1,59	1,88	1,66	caffeoyl-CoA 3-O-methyltransferase, putative
260001_at	At1g67990	0,96	0,98	0,83	1,01	1,04	0,96	0,90	0,93	caffeoyl-CoA 3-O-methyltransferase, putative
260006_at	At1g68000	0,99	0,83	0,98	1,18	0,89	0,98	0,81	0,96	CDP-diacylglycerol--inositol 3-phosphatidyltransferase / phosphatidylinosito
260014_at	At1g68010	1,14	1,08	1,19	0,83	0,93	1,24	1,26	1,24	glycerate dehydrogenase / NADH-dependent hydroxypyruvate reductase
260010_at	At1g68020	0,86	0,93	0,96	0,77	0,85	0,99	0,92	0,79	glycosyl transferase family 20 protein / trehalose-phosphatase family protei
259991_at	At1g68040	0,87	1,07	1,01	1,06	1,06	1,05	0,96	1,19	S-adenosyl-L-methionine:carboxyl methyltransferase family protein
259990_s_at	At1g68050	0,45	0,69	0,83	0,55	0,82	0,76	1,00	0,99	F-box family protein (FKF1) / adagio 3 (ADO3)
260000_at	At1g68060	0,86	0,79	0,95	0,96	1,04	1,02	0,97	1,11	expressed protein
260008_at	At1g68070	1,02	0,93	1,31	0,93	1,05	0,92	0,88	0,77	zinc finger (C3HC4-type RING finger) family protein
259999_at	At1g68080	0,96	1,15	1,07	0,92	0,92	1,28	1,33	1,21	expressed protein
260013_at	At1g68090	0,94	1,02	1,04	1,04	1,07	1,08	1,01	1,00	annexin 5 (ANN5)
260003_at	At1g68100	0,78	0,78	0,75	0,81	0,83	0,78	0,90	0,84	IAA-alanine resistance protein 1, putative
260011_at	At1g68110	0,85	0,95	0,94	0,89	0,82	0,87	0,89	1,10	epsin N-terminal homology (ENTH) domain-containing protein / clathrin ass
259998_at	At1g68120	1,19	1,05	1,17	1,06	1,10	0,78	1,06	1,12	expressed protein
259994_at	At1g68130	1,01	1,23	1,04	0,92	0,94	0,97	0,86	0,86	zinc finger (C2H2 type) family protein
260436_at	At1g68140	0,92	1,29	1,46	0,87	1,06	0,98	1,09	1,43	expressed protein
260432_at	At1g68150	1,06	1,11	1,08	1,30	1,28	1,09	1,12	1,07	WRKY family transcription factor
260440_at	At1g68160	1,47	1,33	1,14	0,97	1,04	1,14	0,99	1,13	expressed protein
260433_at	At1g68170	0,98	1,02	0,95	0,91	0,97	0,99	1,05	0,87	nodulin MtN21 family protein
260443_at	At1g68185	1,23	1,05	1,16	0,82	0,93	1,07	0,93	0,95	ubiquitin-related
260431_at	At1g68190	1,43	2,06	1,93	0,78	0,61	1,52	1,80	1,77	zinc finger (B-box type) family protein
260430_at	At1g68200	1,16	0,96	1,22	1,00	1,19	1,10	1,00	0,92	zinc finger (CCCH-type) family protein
260442_at	At1g68220	0,63	0,62	0,61	1,06	0,86	0,80	0,78	0,85	expressed protein
260460_at	At1g68230	1,13	1,00	0,75	1,05	1,16	1,03	1,09	1,06	reticulon family protein (RTNLB14)
260459_at	At1g68240	0,91	1,04	1,02	0,86	1,13	1,21	1,15	0,84	hypothetical protein

260458_at	At1g68250	0,96	1,09	0,89	0,69	0,77	0,96	1,10	0,97	expressed protein
260441_at	At1g68260	0,95	0,86	0,77	1,03	0,97	0,85	0,82	0,73	thioesterase family protein
260485_at	At1g68270	0,99	1,00	1,03	0,95	1,09	0,96	1,04	1,05	AMP-dependent synthetase and ligase family protein
260438_at	At1g68290	1,16	1,05	1,11	0,98	0,81	1,01	0,91	0,82	bifunctional nuclease, putative
260444_at	At1g68300	1,32	1,08	0,98	1,08	0,89	1,00	0,93	0,88	universal stress protein (USP) family protein
260435_at	At1g68320	1,22	1,14	1,10	0,99	1,12	0,95	0,97	0,97	myb family transcription factor (MYB62)
260434_at	At1g68330	0,93	1,02	0,83	0,86	1,04	0,88	0,80	0,90	expressed protein
260439_at	At1g68340	0,67	0,97	1,16	1,08	1,31	1,14	1,11	1,05	expressed protein
260484_at	At1g68360	0,89	0,97	0,92	1,10	0,95	0,96	1,02	1,13	zinc finger protein-related
260445_at	At1g68370	1,04	0,85	0,89	1,06	0,99	0,99	0,96	1,03	gravity-responsive protein / altered response to gravity protein (ARG1)
260437_at	At1g68380	1,02	0,98	1,18	0,97	1,01	1,04	0,91	0,90	expressed protein
259858_at	At1g68400	1,09	0,86	0,90	1,04	1,19	0,75	0,88	1,05	leucine-rich repeat transmembrane protein kinase, putative
259859_at	At1g68410	0,78	0,77	0,95	1,22	1,17	0,98	1,07	1,33	protein phosphatase 2C-related / PP2C-related
259855_at	At1g68420	0,97	1,01	0,94	0,95	0,96	1,08	1,15	0,98	asparaginyl-tRNA synthetase-related
259857_at	At1g68430	0,98	1,05	1,08	1,01	1,06	0,99	0,92	0,69	expressed protein
259856_at	At1g68440	1,02	1,12	1,01	0,88	0,84	1,13	1,21	1,10	expressed protein
260261_at	At1g68450	1,09	1,38	1,07	1,05	1,06	0,96	1,12	1,05	VQ motif-containing protein
260240_at	At1g68460	1,04	1,06	1,00	1,00	1,15	0,97	0,97	1,00	adenylate isopentenyltransferase 1 / cytokinin synthase (IPT1)
260262_at	At1g68470	0,90	0,67	0,78	1,11	1,25	0,78	0,88	0,96	exostosin family protein
260263_at	At1g68480	0,97	0,89	0,93	0,98	1,39	1,09	0,91	1,03	zinc finger (C2H2 type) family protein
260268_at	At1g68490	0,86	0,86	0,72	0,84	1,21	0,83	0,79	0,72	expressed protein
260264_at	At1g68500	0,64	0,69	0,82	1,11	0,99	1,03	1,12	1,13	expressed protein
260265_at	At1g68510	0,97	1,04	1,08	1,00	1,18	1,00	0,93	1,00	LOB domain protein 42 / lateral organ boundaries domain protein 42 (LBD4
260266_at	At1g68520	1,27	1,26	1,62	0,53	0,68	1,02	1,14	1,23	zinc finger (B-box type) family protein
260267_at	At1g68530	0,95	1,06	1,14	1,07	0,89	0,84	0,79	0,89	very-long-chain fatty acid condensing enzyme (CUT1)
260260_at	At1g68540	0,57	0,58	0,67	0,80	0,91	0,86	0,94	0,92	oxidoreductase family protein
260209_at	At1g68550	0,73	0,70	0,81	0,85	0,81	0,67	0,84	1,12	AP2 domain-containing transcription factor, putative
262230_at	At1g68560	1,35	1,13	1,14	1,07	1,07	0,79	0,72	0,76	alpha-xylosidase (XYL1)
262281_at	At1g68570	1,19	0,89	0,86	0,92	1,02	0,74	0,69	0,90	proton-dependent oligopeptide transport (POT) family protein
262280_at	At1g68580	0,92	1,49	1,17	0,69	0,86	1,01	1,45	1,32	agenet domain-containing protein / bromo-adjacent homology (BAH) domai
262286_at	At1g68585	1,17	0,67	0,49	1,22	1,21	0,84	0,81	0,88	expressed protein
262283_at	At1g68590	1,16	0,92	1,02	0,89	0,94	1,11	0,94	0,99	plastid-specific 30S ribosomal protein 3, putative / PSRP-3, putative
262232_at	At1g68600	0,77	0,76	0,79	0,86	0,83	0,71	0,80	0,70	expressed protein
262282_at	At1g68610	0,92	1,06	0,91	0,96	1,02	1,16	1,06	0,96	hypothetical protein
262229_at	At1g68620	0,80	1,05	1,23	0,59	0,91	1,31	1,36	1,21	expressed protein
262279_at	At1g68630	1,01	1,20	0,97	1,04	1,02	1,06	1,02	0,94	expressed protein
262278_at	At1g68640	1,12	1,12	1,13	0,95	0,98	0,96	1,09	1,19	bZIP family transcription factor (PERIANTHIA)
262277_at	At1g68650	1,00	1,07	1,28	1,00	0,84	0,64	0,64	0,60	expressed protein
262287_at	At1g68660	1,28	1,46	1,30	0,99	0,91	1,32	1,30	1,28	expressed protein
262284_at	At1g68670	1,05	1,32	0,94	0,82	0,75	1,00	0,96	0,93	myb family transcription factor
262285_at	At1g68680	1,25	0,94	0,81	0,87	1,17	1,07	0,86	0,74	expressed protein
262228_at	At1g68690	0,65	0,55	0,67	1,01	0,89	1,06	1,36	1,49	pseudogene, protein kinase family
262276_at	At1g68700	1,03	0,92	0,99	1,00	1,00	1,07	0,88	0,88	hypothetical protein
262275_at	At1g68710	1,04	1,19	1,00	0,87	0,98	1,15	1,16	1,21	haloacid dehalogenase-like hydrolase family protein
262274_at	At1g68720	1,03	0,88	1,07	0,98	0,86	0,99	1,10	1,06	cytidine/deoxycytidylate deaminase family protein
262273_at	At1g68730	0,97	1,01	0,89	0,73	0,92	0,93	1,20	1,04	zinc finger (DNL type) family protein

262231_at	At1g68740	0,98	1,29	1,32	1,38	1,20	1,07	1,06	1,12	EXS family protein / ERD1/XPR1/SYG1 family protein
260032_at	At1g68750	1,08	0,91	0,90	1,06	0,99	0,91	1,08	1,17	phosphoenolpyruvate carboxylase family protein / PEP carboxylase family p
260033_at	At1g68760	0,93	0,70	0,97	1,40	1,47	0,94	0,87	0,92	MutT/nudix family protein
260040_at	At1g68765	0,98	1,02	1,11	1,05	0,98	0,89	1,06	1,12	expressed protein
260041_at	At1g68780	1,00	0,94	0,97	0,63	1,00	1,18	0,92	0,93	leucine-rich repeat family protein
260031_at	At1g68790	1,07	1,00	1,07	0,98	1,03	1,31	1,48	1,74	expressed protein
260039_at	At1g68795	1,03	1,06	1,00	1,18	1,10	0,96	1,03	1,00	CLE12
260034_at	At1g68810	0,98	0,93	1,05	1,29	1,30	0,79	0,96	0,94	basic helix-loop-helix (bHLH) family protein
260042_at	At1g68820	1,01	1,12	1,09	1,03	0,94	0,99	0,97	1,02	membrane protein, putative
260036_at	At1g68830	1,05	1,12	1,16	0,78	0,84	1,30	1,18	1,20	protein kinase family protein
260037_at	At1g68840	0,79	0,92	1,24	0,81	0,90	1,12	1,17	1,32	DNA-binding protein RAV2 (RAV2) / AP2 domain-containing protein RAP2.8
260035_at	At1g68850	1,22	1,52	1,21	1,02	1,05	1,05	1,00	0,96	peroxidase, putative
260038_at	At1g68875	1,08	1,02	1,05	1,07	0,97	1,03	1,02	1,04	expressed protein
260030_at	At1g68880	0,93	0,88	0,99	1,00	1,07	0,83	0,89	0,95	bZIP transcription factor family protein
259643_at	At1g68890	1,14	1,32	1,32	0,79	0,66	1,03	1,01	1,00	menaquinone biosynthesis protein-related
259644_at	At1g68910	0,88	0,82	0,89	1,00	0,82	1,03	1,14	1,01	expressed protein
259697_at	At1g68920	0,77	0,84	0,83	0,83	1,01	1,04	0,92	1,03	basic helix-loop-helix (bHLH) family protein
259698_at	At1g68930	1,07	1,14	1,07	0,76	1,15	0,81	0,90	0,95	pentatricopeptide (PPR) repeat-containing protein
259699_at	At1g68940	1,06	1,07	1,06	0,99	1,02	1,00	1,08	1,00	armadillo/beta-catenin repeat protein-related / U-box domain-containing pro
259700_at	At1g68980	1,01	0,77	0,84	0,87	0,87	0,97	0,96	0,78	pentatricopeptide (PPR) repeat-containing protein
259672_at	At1g68990	1,30	1,45	1,24	0,82	0,67	1,17	0,94	1,36	DNA-directed RNA polymerase, mitochondrial (RPOMT)
259645_at	At1g69010	1,04	1,16	1,16	0,95	1,06	0,91	0,91	1,01	basic helix-loop-helix (bHLH) family protein
259641_at	At1g69020	0,84	0,82	0,83	1,14	1,07	0,88	1,04	1,15	prolyl oligopeptidase family protein
259642_at	At1g69030	0,77	0,66	0,62	1,38	1,43	0,92	0,82	0,86	BSD domain-containing protein
257516_at	At1g69040	1,09	1,14	0,95	1,10	1,12	0,81	0,89	0,66	ACT domain containing protein (ACR4)
259370_at	At1g69050	0,84	1,10	1,18	0,87	1,19	1,00	0,91	0,87	expressed protein
259367_at	At1g69070	1,17	1,02	0,96	1,00	1,00	1,23	1,04	0,96	expressed protein
259371_at	At1g69080	1,00	0,82	1,01	1,13	0,89	0,84	1,10	0,86	universal stress protein (USP) family protein
259368_at	At1g69100	0,92	1,04	0,99	1,09	0,93	1,05	1,02	0,96	aspartyl protease family protein
259372_at	At1g69120	1,03	1,13	1,00	0,99	1,09	1,13	0,98	1,04	floral homeotic protein APETALA1 (AP1) / agamous-like MADS box protein
259369_s_at	At1g69150	0,90	0,97	0,77	1,03	1,00	1,08	0,93	0,89	DC1 domain-containing protein
259373_at	At1g69160	0,86	0,93	1,01	0,97	1,27	0,93	1,01	1,01	expressed protein
260341_at	At1g69170	1,25	1,61	1,24	1,09	0,99	1,02	1,04	1,11	squamosa promoter-binding protein-like 6 (SPL6)
260355_at	At1g69180	0,95	1,02	0,84	0,97	1,12	1,01	0,98	1,12	transcription factor CRC (CRABS CLAW)
260340_at	At1g69190	1,03	0,96	0,92	0,96	1,21	1,07	1,02	0,83	dihydropterin pyrophosphokinase, putative / dihydropteroate synthase, puta
260343_at	At1g69200	1,11	0,90	0,97	0,70	0,65	0,93	0,89	0,95	pfkB-type carbohydrate kinase family protein
260359_at	At1g69210	1,20	1,06	1,29	0,87	0,97	1,21	0,93	1,16	expressed protein
260339_at	At1g69220	0,80	0,75	0,85	0,86	0,93	0,96	0,93	0,89	serine/threonine protein kinase, putative
260353_at	At1g69230	1,04	1,15	0,91	1,11	1,20	0,76	0,86	0,71	expressed protein
260344_at	At1g69240	1,02	1,02	1,16	1,10	1,17	1,00	1,06	0,89	hydrolase, alpha/beta fold family protein
260338_at	At1g69250	1,22	0,94	0,93	1,08	1,05	1,02	0,88	0,89	nuclear transport factor 2 (NTF2) family protein / RNA recognition motif (RR
260357_at	At1g69260	0,79	1,31	1,19	1,32	1,22	0,88	1,22	1,51	expressed protein
260345_at	At1g69270	0,68	0,68	0,94	0,88	0,82	0,91	1,06	0,85	leucine-rich repeat family protein / protein kinase family protein
260342_at	At1g69280	0,92	0,90	0,94	0,81	0,77	0,87	0,83	0,95	expressed protein
260352_at	At1g69295	1,10	1,04	0,99	1,27	1,26	0,99	0,93	1,06	beta-1,3-glucanase-related
260337_at	At1g69310	1,11	1,05	1,17	1,65	1,73	1,13	1,14	1,42	WRKY family transcription factor

260346_at	At1g69320	1,06	1,17	1,11	1,00	1,26	0,98	0,97	0,87	remorin family protein
260354_at	At1g69330	1,06	1,06	1,12	1,25	1,19	0,96	0,96	0,82	zinc finger (C3HC4-type RING finger) family protein
260358_at	At1g69340	0,97	0,91	1,08	0,88	1,09	0,93	0,90	0,92	appr-1-p processing enzyme family protein
260348_at	At1g69350	0,97	0,85	0,95	0,87	0,91	0,86	0,97	0,94	pentatricopeptide (PPR) repeat-containing protein
260361_at	At1g69360	0,82	1,04	0,84	0,90	0,95	0,85	1,05	1,10	expressed protein
260360_at	At1g69370	1,02	1,17	1,31	1,42	1,32	0,87	1,00	1,01	chorismate mutase, putative
260351_at	At1g69380	1,17	1,07	1,45	0,89	0,82	0,96	1,05	1,00	expressed protein
260356_at	At1g69390	0,95	0,92	1,01	0,99	0,92	1,03	0,98	1,10	chloroplast division protein, putative (MinE1)
260349_at	At1g69400	0,92	0,93	0,97	1,04	0,99	0,95	0,86	1,07	transducin family protein / WD-40 repeat family protein
260350_at	At1g69410	0,86	0,77	0,73	1,31	1,32	0,87	0,92	0,97	eukaryotic translation initiation factor 5A, putative / eIF-5A, putative
260347_at	At1g69420	1,05	0,93	0,93	0,91	0,86	0,89	0,83	0,80	zinc finger (DHHC type) family protein
256292_at	At1g69430	1,01	0,94	0,90	1,21	1,05	1,15	0,84	0,87	hypothetical protein
256293_at	At1g69440	1,01	1,07	0,96	1,04	1,04	1,05	0,96	1,09	PAZ domain-containing protein / piwi domain-containing protein
256294_at	At1g69450	0,75	0,71	0,87	1,13	1,03	1,10	1,09	1,10	early-responsive to dehydration protein-related / ERD protein-related
256295_at	At1g69470	0,95	1,02	1,00	0,95	1,05	0,97	1,01	1,16	hypothetical protein
256296_at	At1g69480	0,37	0,80	0,79	0,90	1,07	1,10	1,22	1,14	EXS family protein / ERD1/XPR1/SYG1 family protein
256300_at	At1g69490	1,33	1,23	0,99	0,83	1,02	1,07	1,03	1,18	no apical meristem (NAM) family protein
256297_at	At1g69500	1,05	0,97	0,97	0,98	1,00	0,92	1,06	1,02	cytochrome P450 family protein
256301_at	At1g69510	1,11	1,03	1,06	1,03	1,04	0,89	0,95	1,00	expressed protein
256304_at	At1g69523	0,79	0,88	1,07	0,58	0,60	1,19	1,14	0,97	UbiE/COQ5 methyltransferase family protein
256302_at	At1g69526	1,01	1,07	0,71	0,76	0,90	1,13	1,39	1,38	UbiE/COQ5 methyltransferase family protein
256299_at	At1g69530	3,44	1,79	0,99	0,52	0,48	2,19	1,51	2,09	expansin, putative (EXP1)
256298_at	At1g69540	0,96	1,00	1,04	1,04	1,05	0,94	0,93	0,91	---
256303_at	At1g69550	1,02	0,98	1,00	0,89	0,77	0,90	1,03	1,04	disease resistance protein (TIR-NBS class), putative
259833_at	At1g69560	1,07	0,97	1,05	1,04	1,15	0,98	1,14	1,05	myb family transcription factor (MYB105)
259834_at	At1g69570	1,12	1,31	1,13	1,12	1,02	0,97	1,20	1,18	Dof-type zinc finger domain-containing protein
259832_at	At1g69580	1,30	1,10	0,86	1,14	0,93	1,16	1,66	1,08	myb family transcription factor
259831_at	At1g69600	1,01	0,95	0,88	1,04	1,11	1,09	1,10	1,14	zinc finger homeobox family protein / ZF-HD homeobox family protein
260420_at	At1g69610	0,91	0,91	0,91	1,02	1,03	1,12	1,06	1,03	expressed protein
260369_at	At1g69620	1,49	1,25	1,32	1,54	1,43	1,09	0,98	1,11	60S ribosomal protein L34 (RPL34B)
260422_at	At1g69630	0,94	1,03	1,03	1,04	1,11	1,04	0,97	0,93	F-box family protein
260421_at	At1g69640	0,85	0,73	0,71	1,09	0,97	0,93	0,82	0,83	acid phosphatase, putative
257498_at	At1g69660	0,99	0,99	0,93	0,96	0,90	0,97	1,03	1,01	meprin and TRAF homology domain-containing protein / MATH domain-cor
260416_at	At1g69670	1,17	0,94	1,20	0,77	0,82	0,97	1,23	1,43	cullin, putative
260397_at	At1g69680	1,00	0,93	0,97	0,97	1,04	0,92	0,94	0,88	expressed protein
260371_at	At1g69690	0,78	0,82	0,94	0,92	0,85	0,82	0,80	0,73	TCP family transcription factor, putative
260368_at	At1g69700	0,85	0,89	0,91	1,44	1,44	0,80	0,71	0,65	ABA-responsive protein (HVA22c)
260396_at	At1g69720	1,07	1,01	0,91	1,09	1,07	1,09	1,12	0,82	heme oxygenase 3 (HO3)
260419_at	At1g69730	1,11	0,96	0,91	1,30	1,02	1,30	1,17	1,09	protein kinase family protein
260370_at	At1g69740	0,66	0,68	0,68	0,85	0,92	0,97	0,94	0,94	porphobilinogen synthase, putative / delta-aminolevulinic acid dehydratase,
260418_s_at	At1g69750	1,12	0,82	0,82	0,96	0,80	0,94	0,97	0,96	cox19 family protein
260367_at	At1g69760	0,68	0,70	0,82	0,76	1,01	1,00	1,00	1,14	expressed protein
260417_at	At1g69770	1,10	1,08	1,00	1,15	1,13	0,98	1,04	0,86	chromomethylase 3 (CMT3)
260395_at	At1g69780	1,19	1,26	0,93	1,02	1,02	0,78	0,88	0,96	homeobox-leucine zipper protein 13 (HB-13) / HD-ZIP transcription factor 1
260415_at	At1g69790	0,73	1,07	0,98	0,82	0,90	1,08	1,24	1,07	protein kinase, putative
260413_at	At1g69800	0,86	1,01	1,44	0,92	0,85	0,90	0,94	0,92	CBS domain-containing protein

260403_at	At1g69810	1,11	0,99	0,91	1,04	1,49	0,98	1,37	1,73	WRKY family transcription factor
260402_at	At1g69820	1,09	1,01	1,02	1,02	1,03	1,01	1,13	1,23	gamma-glutamyltranspeptidase family protein
260412_at	At1g69830	0,63	0,62	0,67	0,74	0,73	0,94	0,92	0,87	alpha-amylase, putative / 1,4-alpha-D-glucan glucanohydrolase, putative
260401_at	At1g69840	1,00	0,84	0,99	1,00	1,03	1,04	1,20	1,27	band 7 family protein
260414_at	At1g69850	0,87	1,31	1,30	1,12	1,25	0,82	0,89	1,11	nitrate transporter (NTL1)
260400_at	At1g69860	0,96	1,14	0,92	1,10	0,98	0,97	0,95	0,95	proton-dependent oligopeptide transport (POT) family protein
260410_at	At1g69870	0,70	0,67	0,61	1,13	1,26	0,98	0,77	0,76	proton-dependent oligopeptide transport (POT) family protein
260408_at	At1g69880	0,76	0,59	0,47	1,03	1,60	2,53	1,28	1,46	thioredoxin, putative
260411_at	At1g69890	0,69	1,15	1,06	0,55	0,57	0,91	1,33	1,46	expressed protein
260407_at	At1g69910	0,66	0,76	0,73	0,82	0,89	0,76	0,94	1,15	protein kinase family protein
260406_at	At1g69920	0,86	0,86	0,98	1,30	0,88	1,30	0,97	1,13	glutathione S-transferase, putative
260405_at	At1g69930	0,50	0,54	0,55	1,56	1,39	1,09	1,00	1,21	glutathione S-transferase, putative
260409_at	At1g69935	0,80	0,68	0,87	0,80	0,85	0,87	0,82	0,93	expressed protein
260404_at	At1g69950	1,04	0,95	1,24	1,03	1,16	0,83	0,88	0,98	---
264703_at	At1g69960	1,08	0,99	0,99	1,16	1,20	0,98	0,84	0,76	serine/threonine protein phosphatase PP2A-5 catalytic subunit (PP2A5)
264693_at	At1g69970	0,97	1,07	1,00	0,91	0,84	0,88	0,89	0,93	CLE26, putative
264699_at	At1g69980	0,90	0,73	0,85	0,96	1,22	0,96	0,92	1,02	expressed protein
264714_at	At1g69990	0,99	0,90	1,02	1,12	0,98	1,03	1,08	0,93	leucine-rich repeat transmembrane protein kinase, putative
264692_at	At1g70000	1,29	1,25	1,17	1,31	1,16	1,20	1,14	0,92	DNA-binding family protein
257415_at	At1g70040	1,04	0,98	0,90	0,98	1,18	1,00	0,91	1,07	hypothetical protein
264690_at	At1g70060	0,85	0,71	1,02	0,93	1,21	1,00	0,90	1,04	paired amphipathic helix repeat-containing protein
264748_at	At1g70070	0,88	0,90	0,88	0,83	0,70	1,02	1,02	0,98	DEAD/DEAH box helicase, putative
264720_at	At1g70080	1,03	0,96	0,79	1,04	0,95	1,14	1,00	1,01	terpene synthase/cyclase family protein
264704_at	At1g70090	0,72	0,56	0,49	1,03	0,87	0,94	0,99	0,96	glycosyl transferase family 8 protein
264700_at	At1g70100	1,21	1,14	1,64	0,73	0,93	1,46	1,27	1,36	expressed protein
264719_at	At1g70110	1,03	1,07	1,11	0,92	1,12	0,94	0,91	0,96	lectin protein kinase family protein
264718_at	At1g70130	1,04	1,13	1,00	1,44	1,27	1,24	1,50	0,96	lectin protein kinase, putative
264717_at	At1g70140	0,64	0,78	0,73	1,20	1,32	0,93	0,87	0,95	formin homology 2 domain-containing protein / FH2 domain-containing prot
264747_at	At1g70150	0,99	0,90	1,08	0,72	1,10	0,75	0,92	1,10	zinc finger (MYND type) family protein
264701_at	At1g70160	0,73	0,70	0,80	0,77	0,87	0,87	0,81	0,82	expressed protein
264716_at	At1g70170	1,25	1,33	1,28	1,65	1,18	1,17	1,22	1,23	matrixin family protein
264715_at	At1g70180	0,89	1,07	1,12	1,06	1,03	0,86	0,91	1,12	sterile alpha motif (SAM) domain-containing protein
264702_at	At1g70190	1,21	1,09	1,17	1,34	1,21	0,93	0,74	0,73	ribosomal protein L12 family protein
264698_at	At1g70200	1,05	0,90	1,17	0,89	0,94	1,18	1,09	1,11	RNA recognition motif (RRM)-containing protein
264697_at	At1g70210	1,32	1,52	1,38	0,91	0,95	0,79	0,93	0,86	cyclin delta-1 (CYCD1)
264696_at	At1g70230	1,08	0,90	0,80	0,94	1,05	0,79	0,79	0,76	expressed protein
264695_at	At1g70240	1,21	1,17	1,16	1,37	1,02	1,02	1,07	0,84	receptor serine/threonine kinase, putative
264694_at	At1g70250	1,44	1,37	1,20	1,17	1,09	1,15	1,11	1,02	receptor serine/threonine kinase, putative
264341_at	At1g70270	0,93	1,03	1,01	1,14	1,12	1,04	1,19	0,85	expressed protein
264340_at	At1g70280	1,26	1,11	1,41	0,83	0,90	0,86	0,92	1,13	NHL repeat-containing protein
264339_at	At1g70290	1,43	2,24	2,42	0,83	0,80	1,13	1,74	1,82	trehalose-6-phosphate synthase, putative
264338_at	At1g70300	1,11	1,25	0,86	0,96	0,93	1,00	0,99	0,83	potassium transporter, putative
264317_at	At1g70310	0,96	0,86	0,90	0,86	0,89	1,03	0,99	1,11	spermidine synthase 2 (SPDSYN2) / putrescine aminopropyltransferase 2
264335_s_at	At1g70320	0,81	0,84	0,91	0,71	0,74	1,00	1,15	1,10	ubiquitin-protein ligase 1 (UPL1)
264316_at	At1g70330	0,70	0,61	0,63	1,12	1,09	0,77	0,75	0,75	equilibrative nucleoside transporter family protein
264337_at	At1g70350	1,26	1,21	1,01	1,16	1,04	0,99	0,92	0,78	expressed protein

264336_at	At1g70360	0,82	0,85	1,07	0,82	0,80	0,85	1,03	0,94	F-box protein-related
264315_at	At1g70370	0,71	0,60	0,59	1,02	1,01	0,76	0,66	0,62	BURP domain-containing protein / polygalacturonase, putative
264311_at	At1g70400	0,97	1,01	1,00	1,15	0,99	1,00	1,07	1,09	hypothetical protein
264313_at	At1g70410	1,20	1,14	1,26	1,10	1,13	1,04	1,14	1,29	carbonic anhydrase, putative / carbonate dehydratase, putative
264314_at	At1g70420	0,57	1,03	0,98	0,52	0,48	1,19	1,05	1,03	expressed protein
264369_at	At1g70430	1,23	1,22	1,39	0,93	0,93	1,29	1,26	0,90	protein kinase family protein
264312_at	At1g70450	1,02	0,98	0,93	0,87	1,07	0,96	0,97	1,11	protein kinase family protein
260366_at	At1g70460	1,05	0,99	0,98	1,06	1,14	1,06	0,99	0,91	protein kinase, putative
260332_at	At1g70470	1,09	1,09	0,95	1,37	1,01	1,00	0,89	1,12	expressed protein
260304_at	At1g70480	0,78	0,87	1,21	1,13	1,16	1,04	1,04	1,21	expressed protein
260305_at	At1g70490	1,18	1,01	0,93	1,09	1,17	0,92	0,90	0,92	ADP-ribosylation factor, putative
260333_at	At1g70500	1,15	1,01	1,06	1,31	1,48	0,90	1,04	1,21	polygalacturonase, putative / pectinase, putative
260334_at	At1g70510	1,00	1,01	1,06	0,96	0,92	1,10	0,96	1,11	homeobox protein knotted-1 like 2 (KNAT2) (K1)
260303_at	At1g70520	0,69	0,79	0,81	0,64	0,75	0,92	1,11	1,37	protein kinase family protein
260362_at	At1g70530	0,63	0,95	1,19	0,68	0,60	1,05	1,38	1,38	protein kinase family protein
260306_at	At1g70540	0,91	1,04	1,12	0,89	0,94	1,24	0,97	1,08	invertase/pectin methylesterase inhibitor family protein
260363_at	At1g70550	1,11	1,25	1,03	1,15	0,90	1,19	1,17	1,26	expressed protein
260364_at	At1g70560	1,26	1,23	1,20	0,85	1,07	1,16	0,99	1,10	alliinase C-terminal domain-containing protein
260311_at	At1g70570	0,91	0,88	0,94	0,79	0,79	1,06	1,07	1,05	anthranilate phosphoribosyltransferase, putative
260309_at	At1g70580	0,87	0,81	0,82	1,12	0,97	1,29	1,19	1,19	glutamate:glyoxylate aminotransferase 2 (GGT2)
260310_at	At1g70590	0,97	1,58	1,67	1,08	1,20	1,20	1,36	1,61	F-box family protein
260308_at	At1g70610	1,14	1,10	1,61	1,37	1,64	1,16	1,26	1,60	ABC transporter (TAP1)
260307_at	At1g70620	1,20	1,18	1,53	0,73	0,93	1,03	1,26	1,25	cyclin-related
260365_at	At1g70630	0,90	0,87	1,10	1,12	1,05	0,92	1,02	1,03	expressed protein
260177_at	At1g70650	1,07	0,91	1,03	0,80	0,87	0,87	1,04	0,95	zinc finger (Ran-binding) family protein
260180_at	At1g70660	1,37	1,42	1,57	1,41	1,42	1,13	1,20	1,36	ubiquitin-conjugating enzyme family protein
260208_s_at	At1g70670	1,54	1,34	1,17	1,07	0,96	0,91	1,01	0,98	caleosin-related family protein
260179_at	At1g70690	0,93	0,83	0,62	1,04	0,98	0,98	1,01	0,84	kinase-related
260205_at	At1g70700	0,92	1,16	1,85	1,26	1,07	0,83	0,95	1,01	expressed protein
260181_at	At1g70710	1,38	1,12	1,08	0,97	0,96	0,80	0,78	0,89	endo-1,4-beta-glucanase (EGASE) / cellulase
260178_at	At1g70720	1,04	0,91	0,98	0,99	1,08	1,08	0,97	1,15	invertase/pectin methylesterase inhibitor family protein
260207_at	At1g70730	1,02	0,91	0,92	0,88	0,91	1,15	1,05	0,94	phosphoglucomutase, cytoplasmic, putative / glucose phosphomutase, puta
260206_at	At1g70740	0,73	1,24	1,10	0,71	0,58	1,11	1,49	1,33	protein kinase family protein
260182_at	At1g70750	0,83	1,07	0,87	1,01	0,75	0,82	1,15	1,04	expressed protein
262288_at	At1g70760	1,25	1,28	1,39	0,79	0,95	1,33	1,20	1,36	inorganic carbon transport protein-related
262289_at	At1g70770	1,02	0,86	0,94	1,07	0,94	1,00	0,94	0,98	expressed protein
262262_at	At1g70780	0,54	0,51	0,49	0,59	0,58	0,86	0,98	0,89	expressed protein
262291_at	At1g70790	0,61	0,59	0,64	0,78	0,78	1,01	1,12	1,01	C2 domain-containing protein
262314_at	At1g70810	0,79	0,70	0,92	1,00	1,05	1,07	0,99	0,89	C2 domain-containing protein
262309_at	At1g70820	0,74	0,84	1,27	0,54	0,53	1,18	1,38	1,76	phosphoglucomutase, putative / glucose phosphomutase, putative
262312_at	At1g70830	1,32	1,19	1,24	0,76	0,67	0,98	0,97	0,87	Bet v I allergen family protein
262310_at	At1g70840	0,86	1,16	0,79	0,77	0,88	1,03	0,92	1,04	Bet v I allergen family protein
262260_at	At1g70850	0,55	0,58	0,59	0,59	0,55	0,74	0,99	0,99	Bet v I allergen family protein
262300_at	At1g70870	0,88	1,09	0,98	0,94	1,04	1,16	0,92	0,91	major latex protein-related / MLP-related
262301_at	At1g70880	0,84	0,87	0,97	0,86	1,08	1,01	1,03	0,83	Bet v I allergen family protein
262304_at	At1g70890	0,88	0,79	0,80	0,95	0,88	0,68	0,66	0,67	major latex protein-related / MLP-related

262261_at	At1g70895	1,19	1,13	1,03	1,13	0,83	1,23	1,04	1,12 CLE17, putative
262313_at	At1g70900	0,86	0,81	0,93	0,75	0,66	1,04	0,99	1,09 expressed protein
262302_at	At1g70910	0,95	1,01	1,04	0,94	0,93	1,00	0,97	1,05 zinc finger (C3HC4-type RING finger) family protein
262311_at	At1g70920	0,92	0,86	1,04	0,89	1,03	1,03	0,98	1,03 homeobox-leucine zipper protein, putative / HD-ZIP transcription factor, put
262303_at	At1g70930	0,99	0,92	1,03	1,15	0,97	1,11	1,05	1,03 homeobox-leucine zipper protein, putative / HD-ZIP transcription factor, put
262263_at	At1g70940	1,53	1,67	2,35	0,77	0,73	1,37	1,27	1,27 auxin transport protein, putative (PIN3)
262305_at	At1g70950	1,26	1,12	0,96	1,19	1,26	0,96	1,07	1,14 expressed protein
262306_s_at	At1g70980	1,32	1,07	1,15	0,89	1,00	0,96	0,98	0,88 asparaginyl-tRNA synthetase, cytoplasmic, putative / asparagine-tRNA liga
262290_at	At1g70985	1,03	1,13	0,93	1,33	1,24	1,00	0,79	1,05 hydroxyproline-rich glycoprotein family protein
262315_at	At1g70990	1,06	1,11	1,05	1,46	1,34	0,78	0,90	0,92 proline-rich family protein
262307_at	At1g71000	1,60	1,37	1,18	1,46	0,95	1,18	0,86	0,94 DNAJ heat shock N-terminal domain-containing protein
262308_at	At1g71010	1,10	1,42	1,34	1,22	1,19	1,03	1,01	1,03 phosphatidylinositol-4-phosphate 5-kinase family protein
259741_at	At1g71020	1,41	1,38	1,07	1,13	1,33	0,69	0,77	1,01 armadillo/beta-catenin repeat family protein / U-box domain-containing prot
259751_at	At1g71030	1,68	2,05	1,51	0,72	0,57	0,95	1,17	1,34 myb family transcription factor
259752_at	At1g71040	1,00	0,92	1,04	1,11	1,22	1,02	1,01	0,97 multi-copper oxidase type I family protein
259753_at	At1g71050	0,92	1,08	1,05	1,54	1,21	0,83	0,87	0,80 heavy-metal-associated domain-containing protein / copper chaperone (CC
259746_at	At1g71060	1,13	1,04	1,04	0,87	0,92	1,10	1,00	1,00 pentatricopeptide (PPR) repeat-containing protein
259755_at	At1g71070	0,95	0,90	0,90	1,03	1,30	0,90	0,76	0,77 glycosyltransferase family 14 protein / core-2/I-branching enzyme family pro
259756_at	At1g71080	1,16	1,04	1,14	1,11	1,10	0,98	1,02	1,03 expressed protein
259754_at	At1g71090	0,81	0,82	0,71	0,93	0,72	0,92	0,88	0,85 auxin efflux carrier family protein
259749_at	At1g71100	1,06	0,89	1,02	1,11	1,12	0,87	0,94	0,90 ribose 5-phosphate isomerase-related
259742_at	At1g71120	0,90	1,02	0,85	0,92	1,14	1,16	0,97	1,00 GDSL-motif lipase/hydrolase family protein
259750_at	At1g71130	1,05	0,99	1,01	1,05	1,06	1,08	1,08	1,30 AP2 domain-containing transcription factor, putative
259743_at	At1g71140	0,92	0,97	0,98	0,70	0,63	1,15	1,16	1,15 MATE efflux family protein
259744_at	At1g71160	1,01	1,06	1,06	1,12	0,90	1,03	1,01	1,29 beta-ketoacyl-CoA synthase family protein
259747_at	At1g71170	0,83	0,89	0,99	1,31	1,49	0,70	0,77	1,02 6-phosphogluconate dehydrogenase NAD-binding domain-containing prote
259748_at	At1g71180	0,87	1,04	1,05	1,03	1,02	0,81	0,89	1,02 6-phosphogluconate dehydrogenase NAD-binding domain-containing prote
259745_at	At1g71190	0,75	0,85	0,81	0,98	0,98	0,89	0,97	1,15 expressed protein
259898_at	At1g71200	1,31	1,03	1,19	1,23	1,15	1,20	1,11	1,00 basic helix-loop-helix (bHLH) family protein
259899_at	At1g71210	1,06	0,83	0,82	0,95	1,04	1,06	1,02	1,03 pentatricopeptide (PPR) repeat-containing protein
259936_at	At1g71220	1,01	1,00	0,81	0,97	0,84	1,09	1,07	0,94 UDP-glucose:glycoprotein glucosyltransferase, putative
259900_at	At1g71230	1,15	0,98	0,93	1,49	0,97	1,37	0,84	0,86 COP9 signalosome subunit 5A / CSN subunit 5A (CSN5A) / c-JUN coactiva
259935_at	At1g71250	0,62	0,61	0,51	0,94	1,06	1,08	0,83	0,85 GDSL-motif lipase/hydrolase family protein
259942_at	At1g71260	1,52	1,41	1,30	0,88	0,95	0,97	1,00	0,95 expressed protein
259941_s_at	At1g71280	1,32	1,42	1,39	0,93	1,13	1,22	1,06	1,07 DEAD/DEAH box helicase, putative
259940_at	At1g71290	0,97	1,03	1,02	1,04	1,03	1,01	0,99	1,02 F-box family protein-related
259939_s_at	At1g71300	0,92	1,00	0,88	1,05	1,26	0,92	1,04	1,02 Vps52/Sac2 family protein
259938_at	At1g71310	1,01	1,09	1,45	1,16	1,34	0,94	0,99	1,27 expressed protein
259937_s_at	At1g71330	1,35	1,10	1,15	0,85	0,82	1,35	1,25	1,33 ABC transporter family protein
259934_at	At1g71340	1,07	0,80	0,79	0,83	0,80	0,95	1,18	1,31 glycerophosphoryl diester phosphodiesterase family protein
259948_at	At1g71350	1,00	0,97	0,94	0,96	0,89	0,96	0,95	0,71 eukaryotic translation initiation factor SUI1 family protein
259897_at	At1g71380	1,02	1,12	0,97	1,11	1,11	1,08	0,93	0,97 glycosyl hydrolase family 9 protein
259893_at	At1g71390	0,96	1,07	0,99	1,10	0,88	1,02	1,07	0,85 disease resistance family protein / LRR family protein
259952_at	At1g71400	1,74	1,35	1,29	0,74	0,70	2,02	1,55	1,47 disease resistance family protein / LRR family protein
259950_at	At1g71410	0,79	0,92	1,17	0,89	0,91	1,09	0,86	0,92 protein kinase family protein
259949_at	At1g71420	0,94	0,99	0,86	0,90	0,76	1,00	1,01	0,82 pentatricopeptide (PPR) repeat-containing protein

259894_at	At1g71430	1,09	1,15	1,06	1,14	1,36	0,91	0,91	0,78 expressed protein
259895_at	At1g71440	1,05	0,94	1,18	1,13	0,99	0,95	0,90	0,91 tubulin folding cofactor E / Pfifferling (PFI)
259946_at	At1g71450	1,09	1,02	1,21	0,97	0,84	0,83	0,94	1,02 AP2 domain-containing transcription factor, putative
259945_at	At1g71460	1,16	1,17	0,96	0,65	0,60	0,88	0,87	0,86 pentatricopeptide (PPR) repeat-containing protein
259944_at	At1g71470	0,94	1,09	1,03	1,02	1,02	1,01	0,93	1,12 hypothetical protein
259943_at	At1g71480	1,12	1,05	1,03	0,86	0,96	1,41	1,29	1,24 nuclear transport factor 2 (NTF2) family protein
259951_at	At1g71490	0,97	0,95	0,99	0,97	1,01	0,95	1,03	1,11 pentatricopeptide (PPR) repeat-containing protein
259896_at	At1g71500	1,02	0,91	1,00	0,92	1,09	1,07	1,02	0,99 Rieske (2Fe-2S) domain-containing protein
259947_at	At1g71530	0,98	0,92	0,93	0,94	1,06	1,04	1,09	1,08 protein kinase family protein
261532_at	At1g71680	0,91	1,05	0,98	1,26	1,24	0,95	1,09	1,04 lysine and histidine specific transporter, putative
261533_at	At1g71690	1,03	1,11	1,16	0,86	1,17	1,19	0,91	1,25 expressed protein
261503_at	At1g71691	1,00	1,07	0,90	0,94	0,96	1,03	0,84	0,94 GDSL-motif lipase/hydrolase family protein
261504_at	At1g71692	1,13	1,33	1,15	1,25	1,21	0,93	1,17	1,25 MADS-box protein (AGL12)
261518_at	At1g71695	1,05	0,94	1,00	1,11	1,22	0,80	0,69	0,71 peroxidase 12 (PER12) (P12) (PRXR6)
261505_at	At1g71696	0,86	0,83	0,76	0,92	1,06	0,99	0,94	0,92 carboxypeptidase D, putative
261506_at	At1g71697	0,79	0,70	0,68	1,00	1,13	1,13	1,13	1,13 choline kinase, putative
261522_at	At1g71710	0,82	0,71	0,82	0,99	0,86	0,99	1,00	1,05 inositol polyphosphate 5-phosphatase, putative
261507_at	At1g71720	0,98	0,92	0,95	0,80	0,82	1,14	1,15	1,15 S1 RNA-binding domain-containing protein
261508_at	At1g71730	1,24	1,13	1,21	0,99	1,22	0,96	0,92	0,84 expressed protein
261509_at	At1g71740	1,32	1,37	1,27	1,03	1,05	1,04	1,07	1,04 hypothetical protein
261516_at	At1g71750	1,09	0,93	0,98	1,12	1,08	1,00	0,97	0,86 phosphoribosyltransferase family protein
261510_at	At1g71760	0,99	1,00	1,08	0,99	1,02	1,05	1,14	1,22 hypothetical protein
261511_at	At1g71770	0,95	1,15	1,21	0,85	1,19	1,04	0,97	0,86 polyadenylate-binding protein 5 (PABP5)
261517_at	At1g71780	1,35	1,18	1,11	1,47	1,53	0,94	0,81	0,85 expressed protein
261512_at	At1g71790	0,78	0,96	0,89	1,11	1,22	0,92	0,85	0,93 F-actin capping protein beta subunit family protein
261515_at	At1g71800	0,89	1,00	0,85	1,05	1,13	0,96	0,87	0,99 cleavage stimulation factor, putative
261519_at	At1g71810	1,31	1,43	1,22	0,67	0,75	1,30	1,50	1,24 ABC1 family protein
261520_at	At1g71820	0,89	0,82	0,89	0,87	0,96	0,95	0,92	0,92 expressed protein
261521_at	At1g71830	1,07	1,06	0,85	1,02	1,22	0,96	0,92	0,78 leucine-rich repeat family protein / protein kinase family protein
261513_at	At1g71840	0,93	0,81	0,84	0,90	1,00	0,86	0,85	0,84 transducin family protein / WD-40 repeat family protein
257487_at	At1g71850	1,07	1,00	0,85	1,04	1,28	0,88	0,89	0,72 expressed protein
261523_at	At1g71860	1,00	1,08	1,31	1,02	1,09	0,93	1,07	1,17 protein tyrosine phosphatase 1 (PTP1)
261514_at	At1g71870	0,88	0,88	0,93	1,03	0,87	0,89	0,97	0,75 MATE efflux family protein
260143_at	At1g71880	1,32	1,03	1,28	1,74	1,54	1,05	0,99	1,07 sucrose transporter / sucrose-proton symporter (SUC1)
260170_at	At1g71890	1,04	0,90	0,86	1,10	1,16	0,91	0,83	0,70 sucrose transporter / sucrose-proton symporter (SUC5)
260142_at	At1g71900	1,08	1,18	1,34	1,06	1,23	1,12	1,01	1,05 expressed protein
260171_at	At1g71910	0,72	0,98	1,12	0,96	1,14	1,04	0,97	0,84 expressed protein
260172_s_at	At1g71920	1,29	1,17	1,12	1,08	1,01	1,13	1,11	1,00 histidinol-phosphate aminotransferase, putative
260173_at	At1g71930	1,06	1,03	1,12	1,18	1,17	1,11	1,08	0,99 no apical meristem (NAM) family protein
260174_at	At1g71940	0,86	0,93	0,90	1,36	1,74	0,93	0,88	0,94 expressed protein
260176_at	At1g71950	0,95	0,90	0,95	1,18	1,21	1,17	0,99	1,05 expressed protein
260144_at	At1g71960	0,86	0,96	1,08	1,06	0,89	0,91	1,16	1,18 ABC transporter family protein
260167_at	At1g71970	1,03	0,90	0,82	1,00	0,84	0,76	0,91	1,06 expressed protein
260168_at	At1g71980	1,15	1,20	1,16	0,87	0,90	1,24	1,02	1,10 protease-associated zinc finger (C3HC4-type RING finger) family protein
260169_at	At1g71990	1,02	1,01	1,05	1,18	1,05	0,98	0,85	0,86 alpha-(1,4)-fucosyltransferase / galactoside 3(4)-L-fucosyltransferase (FUT
260175_at	At1g72000	0,93	0,97	1,20	0,97	0,99	1,03	1,07	1,13 beta-fructofuranosidase, putative / invertase, putative / saccharase, putative

256342_at	At1g72020	1,40	1,37	1,16	1,24	1,41	1,02	0,88	1,00	expressed protein
256336_at	At1g72030	1,54	2,07	2,58	1,13	1,33	1,36	1,61	1,60	GCN5-related N-acetyltransferase (GNAT) family protein
256341_at	At1g72040	1,13	1,09	1,04	0,91	0,90	0,98	0,92	0,94	deoxynucleoside kinase family
256344_at	At1g72050	0,97	1,01	1,12	0,74	0,82	1,01	1,02	0,98	zinc finger (C2H2 type) family protein
256337_at	At1g72060	0,94	0,99	1,74	0,97	1,24	0,99	0,69	0,67	expressed protein
256340_at	At1g72070	1,04	0,93	0,96	1,10	1,13	1,46	1,44	1,05	DNAJ heat shock N-terminal domain-containing protein
256339_at	At1g72080	0,90	1,16	0,93	1,05	1,08	1,12	0,98	0,80	hypothetical protein
256343_at	At1g72090	1,06	1,16	1,10	0,93	0,77	1,07	0,98	1,12	radical SAM domain-containing protein / TRAM domain-containing protein
256338_at	At1g72100	0,59	0,93	0,93	0,73	0,70	1,12	0,94	0,97	late embryogenesis abundant domain-containing protein / LEA domain-con
256335_at	At1g72110	0,89	1,05	1,03	1,02	1,10	1,18	0,87	0,97	expressed protein
256334_at	At1g72120	0,98	0,83	0,90	0,98	0,97	0,95	0,89	1,22	proton-dependent oligopeptide transport (POT) family protein
259829_at	At1g72130	1,06	1,06	1,29	1,17	0,92	1,05	1,02	1,02	proton-dependent oligopeptide transport (POT) family protein
259846_at	At1g72140	0,93	1,03	0,89	1,37	0,88	0,74	0,98	1,06	proton-dependent oligopeptide transport (POT) family protein
259803_at	At1g72150	1,06	0,94	1,07	0,90	0,90	1,19	1,03	1,19	SEC14 cytosolic factor family protein / phosphoglyceride transfer family pro
259804_at	At1g72160	0,93	0,90	1,12	1,03	0,99	1,05	1,00	1,10	SEC14 cytosolic factor family protein / phosphoglyceride transfer family pro
259847_at	At1g72170	1,54	1,32	1,29	1,07	1,16	0,92	0,98	1,09	expressed protein
259800_at	At1g72175	0,81	0,74	0,90	1,26	1,30	0,92	0,77	0,77	zinc finger (C3HC4-type RING finger) family protein
259848_at	At1g72180	1,05	1,13	1,65	0,87	1,20	0,92	0,90	0,99	leucine-rich repeat transmembrane protein kinase, putative
259849_at	At1g72190	0,74	0,92	1,03	0,95	0,86	0,95	0,98	0,88	oxidoreductase family protein
259854_at	At1g72200	0,98	1,13	0,92	1,16	0,94	1,04	1,02	0,90	zinc finger (C3HC4-type RING finger) family protein
259828_at	At1g72220	0,97	1,05	0,90	0,97	1,04	1,00	1,02	0,99	zinc finger (C3HC4-type RING finger) family protein
259801_at	At1g72230	1,10	1,12	1,01	2,41	1,51	0,68	0,74	0,84	plastocyanin-like domain-containing protein
259850_at	At1g72240	1,31	1,36	1,00	1,02	0,99	1,16	1,15	0,97	expressed protein
259851_at	At1g72250	1,13	1,28	1,34	1,33	1,12	0,99	0,91	1,13	kinesin motor protein-related
259802_at	At1g72260	0,92	1,11	1,04	0,92	0,91	0,95	1,02	1,24	thionin (THI2.1)
259827_at	At1g72270	1,14	1,00	0,96	1,07	0,97	0,89	1,06	1,00	expressed protein
259852_at	At1g72280	0,61	0,45	0,66	0,77	0,89	1,02	0,84	0,93	endoplasmic reticulum oxidoreductin 1 (ERO1) family protein
259799_at	At1g72290	0,94	1,04	0,93	1,20	1,14	1,10	0,94	0,96	trypsin and protease inhibitor family protein / Kunitz family protein
259853_at	At1g72300	0,77	0,66	0,55	0,78	0,91	0,92	0,98	0,98	leucine-rich repeat transmembrane protein kinase, putative
260454_at	At1g72310	0,82	1,02	0,95	0,90	0,99	0,89	0,90	0,99	zinc finger (C3HC4-type RING finger) family protein (ATL3)
260398_at	At1g72320	0,95	0,85	1,00	0,81	0,92	0,95	0,89	0,95	pumilio/Puf RNA-binding domain-containing protein
260428_at	At1g72340	1,25	1,09	1,57	1,00	1,12	1,03	0,88	0,94	eukaryotic translation initiation factor 2B family protein / eIF-2B family prote
260452_at	At1g72350	0,99	1,03	1,09	0,96	1,04	0,86	1,11	1,28	MADS-box protein (AGL60)
260451_at	At1g72360	0,58	0,76	0,91	1,03	1,23	0,76	1,04	1,36	ethylene-responsive element-binding protein, putative
260426_at	At1g72370	1,11	1,09	0,95	1,05	0,92	0,95	0,96	0,99	40S ribosomal protein SA (RPSaA)
260450_at	At1g72380	0,93	1,01	0,92	1,05	1,03	1,05	1,03	0,99	expressed protein
260449_at	At1g72390	0,95	0,94	1,06	0,82	1,09	1,03	0,98	0,98	expressed protein
260448_at	At1g72400	0,94	1,00	1,11	0,86	0,96	1,01	1,00	1,03	COP1-interacting protein-related
260447_at	At1g72410	0,90	0,88	0,98	1,00	0,89	0,99	1,06	0,98	COP1-interacting protein-related
260446_at	At1g72420	0,97	0,69	0,65	0,65	0,84	0,81	0,96	1,01	chaperone-related
260427_at	At1g72430	1,32	1,26	1,23	1,13	1,34	0,89	0,76	0,75	auxin-responsive protein-related
260425_at	At1g72440	1,19	0,83	0,87	0,76	0,65	1,03	1,14	1,24	CCAAT-box-binding transcription factor-related
260429_at	At1g72450	0,74	1,05	1,31	1,03	0,80	1,05	1,33	1,23	expressed protein
260424_at	At1g72460	0,92	1,00	1,01	0,99	1,15	1,11	0,96	1,01	leucine-rich repeat transmembrane protein kinase, putative
260423_at	At1g72470	0,89	0,59	0,71	1,22	0,99	0,77	0,77	0,55	exocyst subunit EXO70 family protein
260457_at	At1g72480	0,76	0,68	0,77	1,06	0,87	0,93	0,82	0,73	expressed protein

260456_at	At1g72490	1,00	1,00	0,92	1,01	1,08	1,04	0,96	0,96	hypothetical protein
260455_at	At1g72500	1,54	1,77	2,01	0,88	0,80	1,10	1,46	1,64	inter-alpha-trypsin inhibitor heavy chain-related
260453_s_at	At1g72510	1,00	1,16	0,71	1,05	0,88	1,16	2,09	2,32	expressed protein
260399_at	At1g72520	1,00	1,31	1,73	0,84	0,63	1,31	1,73	1,64	lipoxygenase, putative
257503_at	At1g72530	0,92	0,99	0,96	0,88	1,10	0,98	0,88	0,98	plastid developmental protein DAG, putative
259921_at	At1g72540	0,92	1,09	0,95	0,86	0,97	0,98	1,01	1,09	protein kinase, putative
259920_at	At1g72550	1,15	0,99	1,01	1,07	0,90	1,02	1,00	1,14	tRNA synthetase beta subunit family protein
259919_at	At1g72560	1,17	1,03	1,10	0,87	0,89	1,10	0,99	1,13	tRNA export mediator exportin-t, putative (PAUSED)
259918_at	At1g72570	0,99	1,03	0,97	1,01	0,95	0,94	0,94	0,90	ovule development protein, putative
259917_at	At1g72580	1,03	1,03	1,13	1,02	0,87	1,01	0,94	0,97	hypothetical protein
259916_at	At1g72600	1,01	1,06	1,09	0,99	0,99	1,03	0,93	1,01	hydroxyproline-rich glycoprotein family protein
259892_at	At1g72610	1,17	1,04	0,99	0,85	0,65	1,10	0,92	0,93	germin-like protein (GER1)
259863_at	At1g72630	1,09	0,90	1,20	1,16	1,20	0,86	0,83	0,74	expressed protein
259914_at	At1g72640	1,06	1,04	0,71	0,82	0,84	0,99	0,89	0,91	expressed protein
259862_at	At1g72650	1,11	1,18	1,50	0,75	0,68	1,05	1,29	1,40	myb family transcription factor
259913_at	At1g72660	1,27	1,16	1,33	0,91	1,17	1,06	0,95	1,12	developmentally regulated GTP-binding protein, putative
259912_at	At1g72670	1,04	1,37	1,19	1,27	1,00	0,98	1,06	1,02	calmodulin-binding family protein
259911_at	At1g72680	0,51	0,51	0,67	0,87	0,93	0,97	1,05	1,18	cinnamyl-alcohol dehydrogenase, putative
259910_at	At1g72700	0,75	0,81	1,06	0,89	0,78	0,98	1,46	2,00	haloacid dehalogenase-like hydrolase family protein
259865_at	At1g72710	0,83	0,83	0,90	1,12	1,23	0,95	0,88	1,03	casein kinase, putative
259891_at	At1g72730	1,12	1,20	0,85	1,41	1,24	1,05	0,89	0,77	eukaryotic translation initiation factor 4A, putative / eIF-4A, putative
259924_at	At1g72740	0,99	0,79	0,85	0,98	1,12	1,04	0,93	0,95	DNA-binding family protein / histone H1/H5 family protein
259890_at	At1g72750	0,80	0,81	0,77	1,06	1,08	0,86	0,88	0,84	mitochondrial import inner membrane translocase subunit Tim17/Tim22/Tim
259923_at	At1g72760	0,93	0,97	1,17	0,94	0,98	0,98	0,90	0,92	protein kinase family protein
259922_at	At1g72770	1,17	1,14	1,38	1,14	1,47	1,02	1,17	1,11	protein phosphatase 2C P2C-HA / PP2C P2C-HA (P2C-HA)
259915_at	At1g72790	0,89	0,76	0,54	0,97	0,91	0,93	0,93	0,91	hydroxyproline-rich glycoprotein family protein
259864_at	At1g72800	0,58	0,51	0,80	1,05	0,86	0,82	0,95	0,87	nuM1-related
262380_at	At1g72810	1,41	1,53	1,77	1,14	1,43	0,83	0,86	0,64	threonine synthase, putative
262355_at	At1g72820	0,88	1,08	1,02	0,71	0,68	0,82	0,98	1,01	mitochondrial substrate carrier family protein
262378_at	At1g72830	0,95	0,93	0,96	1,20	1,18	0,78	0,81	1,27	CCAAT-binding transcription factor (CBF-B/NF-YA) family protein
262362_at	At1g72840	0,96	1,03	0,97	0,99	1,05	1,08	1,16	1,16	disease resistance protein (TIR-NBS-LRR class), putative
262363_at	At1g72850	0,94	1,03	1,01	0,96	0,95	1,08	0,93	0,86	disease resistance protein (TIR-NBS class), putative
262364_at	At1g72860	1,08	1,14	0,96	1,06	1,20	0,92	0,87	0,97	disease resistance protein (TIR-NBS-LRR class), putative
262365_at	At1g72870	0,94	1,04	0,95	0,96	1,07	1,10	0,96	0,86	disease resistance protein (TIR-NBS class), putative
262366_at	At1g72890	1,00	1,11	0,83	0,88	0,76	1,28	1,42	1,28	disease resistance protein (TIR-NBS class), putative
262381_at	At1g72900	0,87	0,64	0,56	0,65	0,61	1,03	1,13	1,29	disease resistance protein (TIR-NBS class), putative
262382_at	At1g72920	0,89	1,38	1,95	1,06	0,64	1,19	1,78	1,14	disease resistance protein (TIR-NBS class), putative
262374_s_at	At1g72930	0,74	0,66	0,83	0,64	0,70	1,20	0,96	0,93	disease resistance protein (TIR-NBS class), putative
262383_at	At1g72940	0,95	1,20	1,29	0,88	0,59	1,11	1,32	1,22	disease resistance protein (TIR-NBS class), putative
262384_at	At1g72950	0,76	1,08	0,94	0,77	1,02	1,03	1,09	1,04	disease resistance protein (TIR-NBS class), putative
262385_at	At1g72960	0,98	0,92	0,90	0,99	0,95	0,95	1,08	1,06	root hair defective 3 GTP-binding (RHD3) family protein
262376_at	At1g72970	1,11	0,90	0,80	1,23	1,03	0,90	0,80	0,74	glucose-methanol-choline (GMC) oxidoreductase family protein
262351_at	At1g72990	1,30	1,20	1,02	1,03	0,97	0,94	1,04	1,07	glycosyl hydrolase family 35 protein
262356_at	At1g73000	1,06	1,07	1,03	1,02	1,04	1,04	1,04	1,29	hypothetical protein
262369_at	At1g73010	2,20	2,79	2,66	3,05	2,81	1,21	1,28	1,23	expressed protein
262379_at	At1g73020	0,87	0,82	0,95	1,35	1,00	1,02	0,97	0,98	expressed protein

262367_at	At1g73030	0,93	0,89	0,93	1,15	1,17	1,08	0,96	0,99	SNF7 family protein
262357_at	At1g73040	0,76	0,71	0,73	1,59	1,61	0,98	0,99	1,14	jacalin lectin family protein
262358_at	At1g73050	0,95	0,95	0,81	1,12	1,22	0,84	0,90	0,95	(R)-mandelonitrile lyase, putative / (R)-oxynitrilase, putative
262368_at	At1g73060	0,42	0,40	0,37	0,85	0,85	0,80	0,74	0,89	leucine-rich repeat family protein
262359_at	At1g73070	1,28	1,05	1,08	0,74	1,10	0,98	1,16	1,34	expressed protein
262360_at	At1g73080	0,92	0,97	1,12	0,83	0,84	1,26	1,41	1,37	leucine-rich repeat transmembrane protein kinase, putative
262370_at	At1g73090	1,00	0,88	0,93	0,87	0,93	1,08	1,07	0,97	expressed protein
262375_at	At1g73100	0,95	1,04	0,96	0,90	1,00	0,96	0,93	0,91	SET domain-containing protein (SUVH3)
262377_at	At1g73110	1,02	0,88	0,91	0,82	0,79	1,16	1,05	1,07	ribulose bisphosphate carboxylase/oxygenase activase, putative / RuBisCC
262373_at	At1g73120	0,59	0,65	0,78	1,52	1,56	0,36	0,34	0,45	expressed protein
262372_at	At1g73130	1,19	1,10	1,02	1,21	1,27	1,25	1,34	1,09	expressed protein
262361_at	At1g73150	0,79	1,08	0,90	1,09	1,07	0,91	0,97	1,06	DNA-binding bromodomain-containing protein
262371_at	At1g73160	0,99	1,06	1,03	1,01	1,14	1,09	0,97	0,85	glycosyl transferase family 1 protein
260089_at	At1g73170	1,10	0,95	1,08	0,66	0,69	1,13	1,15	1,11	expressed protein
260100_at	At1g73177	1,04	0,87	0,83	1,05	0,95	0,93	0,98	0,71	expressed protein
260099_at	At1g73180	1,00	0,79	0,79	0,81	0,91	0,93	1,00	0,92	eukaryotic translation initiation factor-related
260088_at	At1g73190	0,05	0,06	0,05	0,63	0,60	1,12	1,08	0,85	tonoplast intrinsic protein, alpha / alpha-TIP (TIP3.1)
260087_at	At1g73200	0,86	0,76	1,15	1,07	1,01	0,99	1,06	1,46	expressed protein
260097_at	At1g73220	0,33	0,44	0,45	0,56	0,74	0,97	1,15	1,05	sugar transporter family protein
260095_at	At1g73230	1,40	1,23	1,16	1,25	1,47	1,09	0,96	1,02	nascent polypeptide-associated complex (NAC) domain-containing protein
260096_at	At1g73240	1,03	0,82	0,91	1,10	1,17	0,82	0,77	0,71	expressed protein
260094_at	At1g73250	0,96	0,95	1,07	1,14	0,98	0,96	0,92	1,06	GDP-4-keto-6-deoxy-D-mannose-3,5-epimerase-4-reductase (GER1)
260101_at	At1g73260	1,78	2,31	1,57	2,23	1,60	1,20	1,13	1,13	trypsin and protease inhibitor family protein / Kunitz family protein
260093_at	At1g73270	0,99	1,02	0,91	0,91	1,04	1,12	1,07	0,97	serine carboxypeptidase S10 family protein
260092_at	At1g73280	1,02	1,00	0,92	0,99	0,87	0,96	0,98	1,04	serine carboxypeptidase S10 family protein
260091_at	At1g73290	1,01	0,97	0,89	1,00	0,97	0,96	1,01	1,07	serine carboxypeptidase S10 family protein
257500_s_at	At1g73300	0,89	0,89	0,97	1,03	1,13	0,96	1,19	1,01	serine carboxypeptidase S10 family protein
260090_at	At1g73310	1,06	1,12	0,86	1,05	0,94	1,06	1,09	1,13	serine carboxypeptidase S10 family protein
260098_at	At1g73320	1,20	1,39	1,18	0,85	0,93	1,21	1,17	1,26	expressed protein
245736_at	At1g73330	0,48	0,55	0,49	0,75	0,61	0,79	0,86	0,89	protease inhibitor, putative (DR4)
245728_at	At1g73340	1,14	1,07	0,98	1,05	0,94	1,01	1,04	1,07	cytochrome P450 family protein
245727_at	At1g73350	0,96	1,21	1,10	0,79	1,06	1,17	1,09	1,22	expressed protein
245726_at	At1g73360	0,91	0,92	0,99	0,80	1,04	0,97	1,00	0,96	homeobox-leucine zipper family protein / lipid-binding START domain-conta
245725_at	At1g73370	1,19	0,96	0,79	1,16	1,11	0,88	1,02	1,01	sucrose synthase, putative / sucrose-UDP glucosyltransferase, putative
245733_at	At1g73380	0,65	0,67	0,76	0,87	1,13	1,08	0,89	1,20	expressed protein
245724_at	At1g73390	1,03	1,10	1,06	1,08	0,97	1,07	1,00	1,15	expressed protein
245723_at	At1g73400	1,08	1,03	0,86	0,93	1,06	1,01	0,95	0,83	pentatricopeptide (PPR) repeat-containing protein
245735_at	At1g73410	0,93	0,99	1,02	1,06	0,96	1,04	1,10	0,98	myb family transcription factor (MYB54)
245722_at	At1g73430	1,07	0,97	0,97	0,76	0,77	1,11	1,10	0,98	sec34-like family protein
245732_at	At1g73440	0,94	0,81	0,85	0,98	0,93	1,12	0,92	0,90	calmodulin-related
245721_s_at	At1g73460	1,14	1,05	1,17	0,83	0,98	1,16	1,14	1,12	protein kinase, putative
245730_at	At1g73470	0,48	0,47	0,50	0,73	0,89	0,78	0,81	0,87	expressed protein
245734_at	At1g73480	0,74	1,12	1,91	1,13	1,14	0,97	1,18	1,15	hydrolase, alpha/beta fold family protein
245729_at	At1g73490	1,20	1,01	1,19	1,05	1,05	0,96	0,98	1,23	RNA recognition motif (RRM)-containing protein
245731_at	At1g73500	0,71	1,01	1,08	0,91	1,04	0,79	1,00	1,05	mitogen-activated protein kinase kinase (MAPKK), putative (MKK9)
245779_at	At1g73510	1,04	0,92	1,10	0,60	0,71	1,19	0,95	1,13	hypothetical protein

245778_at	At1g73530	1,06	0,91	0,79	0,83	0,69	1,05	1,00	0,97	RNA recognition motif (RRM)-containing protein
245777_at	At1g73540	1,60	2,31	1,92	0,81	0,71	2,34	3,56	2,82	MutT/nudix family protein
259844_at	At1g73560	0,93	1,13	1,17	1,03	1,08	1,11	1,04	1,05	protease inhibitor/seed storage/lipid transfer protein (LTP) family protein
259843_at	At1g73570	0,79	0,95	1,06	0,75	1,10	0,96	0,90	0,97	suppressor of lin-12-like protein-related / sel-1 protein-related
259845_at	At1g73590	1,09	1,11	1,04	1,08	1,13	1,20	0,96	0,92	auxin efflux carrier protein, putative (PIN1)
259842_at	At1g73600	0,65	0,62	0,74	1,80	1,59	0,67	0,82	0,86	phosphoethanolamine N-methyltransferase 3, putative (NMT3)
260066_at	At1g73610	0,95	0,99	0,96	1,27	0,98	1,03	1,08	0,78	GDSL-motif lipase/hydrolase family protein
260077_at	At1g73620	1,47	1,58	0,92	2,55	1,34	0,89	0,91	0,80	thaumatin-like protein, putative / pathogenesis-related protein, putative
260076_at	At1g73630	0,87	0,96	0,98	1,30	1,15	0,85	0,89	0,85	calcium-binding protein, putative
260074_at	At1g73640	0,72	0,60	0,85	1,40	1,14	1,01	0,84	1,13	Ras-related GTP-binding family protein
260072_at	At1g73650	1,02	0,94	0,88	1,21	1,09	1,33	1,23	1,15	expressed protein
260044_at	At1g73655	0,89	0,89	1,01	0,74	0,71	1,12	1,02	1,02	immunophilin / FKBP-type peptidyl-prolyl cis-trans isomerase family protein
260073_at	At1g73660	1,28	1,39	1,23	1,04	0,91	1,11	1,11	1,31	protein kinase family protein
260045_at	At1g73670	0,95	0,84	0,83	1,05	0,98	1,15	0,95	1,18	mitogen-activated protein kinase, putative / MAPK, putative (MPK15)
260060_at	At1g73680	0,78	1,04	1,63	1,09	1,07	1,19	1,39	1,28	pathogen-responsive alpha-dioxygenase, putative
260061_at	At1g73690	0,99	1,08	1,00	0,99	1,04	0,99	0,87	0,81	cell division protein kinase, putative
260075_at	At1g73700	0,99	0,95	1,04	0,75	0,77	1,34	1,32	1,12	MATE efflux family protein
260062_at	At1g73710	0,92	0,94	0,99	0,72	0,84	0,99	0,96	1,06	pentatricopeptide (PPR) repeat-containing protein
260063_at	At1g73720	1,03	1,04	1,22	0,88	0,94	1,07	1,00	0,88	transducin family protein / WD-40 repeat family protein
260064_at	At1g73730	1,00	1,16	1,12	0,91	0,92	1,20	1,05	1,05	ethylene-insensitive3-like3 (EIL3)
260047_at	At1g73740	0,85	0,93	1,04	0,91	0,78	0,78	0,78	0,90	glycosyl transferase family 28 protein
260048_at	At1g73750	1,08	1,47	1,85	1,00	0,85	1,47	1,60	1,71	expressed protein
260065_at	At1g73760	0,81	1,22	1,61	0,76	0,86	0,93	0,79	0,91	zinc finger (C3HC4-type RING finger) family protein
260067_at	At1g73780	1,07	1,05	1,11	1,14	1,18	1,05	1,02	0,86	protease inhibitor/seed storage/lipid transfer protein (LTP) family protein
260078_at	At1g73790	0,92	0,86	1,01	1,22	1,53	0,79	0,84	1,06	expressed protein
260046_at	At1g73800	1,18	1,97	1,25	1,62	1,17	1,90	1,38	1,49	calmodulin-binding protein
260068_at	At1g73805	1,10	1,36	1,09	1,62	1,09	1,30	1,21	1,08	calmodulin-binding protein
260069_at	At1g73820	0,92	0,98	1,00	1,01	1,22	0,85	0,94	0,87	Ssu72-like family protein
260070_at	At1g73830	4,58	2,32	1,47	1,24	1,25	1,05	0,93	1,64	basic helix-loop-helix (bHLH) family protein
260071_at	At1g73840	1,13	1,07	1,29	0,94	1,09	1,00	0,92	0,99	hydroxyproline-rich glycoprotein family protein
260382_at	At1g73850	0,98	0,97	1,01	1,01	1,01	0,85	0,94	1,18	expressed protein
260381_at	At1g73860	1,01	1,05	1,11	0,99	1,10	1,04	0,94	0,98	kinesin motor protein-related
260380_at	At1g73870	1,61	1,66	1,18	1,10	1,29	1,14	0,99	1,37	zinc finger (B-box type) family protein
260379_at	At1g73880	0,84	0,88	1,09	0,81	1,03	0,86	0,97	1,04	expressed protein
260378_at	At1g73910	0,95	1,16	1,05	1,03	1,07	0,93	1,04	1,04	actin-related protein 5 (ARP5)
260393_at	At1g73920	0,96	1,19	1,42	0,91	0,93	1,12	1,22	1,20	lipase family protein
260377_at	At1g73930	1,02	0,83	0,74	1,12	1,13	1,05	0,88	0,92	expressed protein
260390_at	At1g73940	1,30	1,17	1,01	1,22	1,31	1,03	0,94	0,78	expressed protein
260375_at	At1g73950	1,20	1,32	1,23	1,07	1,08	1,01	1,09	0,98	zinc finger (C3HC4-type RING finger) family protein
260374_at	At1g73960	0,93	0,79	0,93	0,80	1,00	1,05	1,12	1,22	expressed protein
260373_at	At1g73970	0,88	0,86	0,84	0,82	0,81	1,11	1,04	1,13	expressed protein
260372_at	At1g73980	0,59	0,72	0,83	0,87	0,88	0,92	0,99	1,02	phosphoribulokinase/uridine kinase family protein
260336_at	At1g73990	0,97	1,00	0,86	1,02	0,74	1,21	1,19	1,16	peptidase U7 family protein
260335_at	At1g74000	1,06	1,03	0,99	1,19	1,31	1,02	1,03	0,92	strictosidine synthase family protein
260386_at	At1g74010	0,93	0,85	0,71	1,23	1,28	1,07	0,92	0,76	strictosidine synthase family protein
260391_at	At1g74020	0,89	0,68	0,68	1,18	1,32	0,91	0,89	0,93	strictosidine synthase family protein

260392_at	At1g74030	1,27	1,22	1,01	1,74	1,51	1,04	0,89	0,89	enolase, putative
260384_at	At1g74040	0,84	0,74	0,68	0,73	0,60	0,97	0,92	0,88	2-isopropylmalate synthase 1 (IMS1)
260389_at	At1g74055	1,36	1,19	0,94	1,24	1,38	0,92	0,96	0,83	expressed protein
260383_s_at	At1g74060	1,25	1,21	1,11	1,08	1,09	0,95	0,97	0,91	60S ribosomal protein L6 (RPL6C)
260388_at	At1g74070	1,23	1,33	1,47	0,99	1,04	1,18	1,00	1,13	peptidyl-prolyl cis-trans isomerase cyclophilin-type family protein
260394_at	At1g74080	1,07	1,00	0,97	1,07	0,99	1,04	1,14	1,08	myb family transcription factor (MYB122)
260385_at	At1g74090	1,18	1,28	1,40	1,07	0,95	0,90	0,88	0,96	sulfotransferase family protein
260387_at	At1g74100	0,84	0,99	1,44	1,36	0,94	0,92	0,97	0,96	sulfotransferase family protein
260376_at	At1g74110	0,96	1,00	0,78	0,89	0,93	0,97	0,99	1,31	cytochrome P450 family protein
259906_at	At1g74120	0,86	0,88	1,01	0,80	1,24	0,98	0,92	0,99	mitochondrial transcription termination factor-related / mTERF-related
259905_s_at	At1g74140	1,03	0,97	1,09	0,92	1,22	0,93	1,10	0,89	rhomboid family protein
259904_at	At1g74150	0,86	1,00	0,95	0,92	0,94	0,98	0,96	0,94	kelch repeat-containing protein
259903_at	At1g74160	0,70	0,89	0,85	0,72	0,88	1,03	1,28	1,09	expressed protein
259902_at	At1g74170	1,36	1,03	1,35	0,95	0,80	1,11	1,16	1,07	leucine-rich repeat family protein
259901_at	At1g74180	0,89	0,84	1,02	0,93	0,73	1,07	1,01	1,16	leucine-rich repeat family protein
260253_at	At1g74190	0,92	1,00	0,96	0,93	0,99	1,03	1,04	0,91	leucine-rich repeat family protein
260247_at	At1g74200	1,04	0,93	0,99	0,86	1,04	1,08	1,17	1,07	leucine-rich repeat family protein
260254_at	At1g74210	1,14	0,93	0,83	2,65	2,35	1,20	1,06	1,18	glycerophosphoryl diester phosphodiesterase family protein
260246_at	At1g74220	1,00	1,06	0,99	0,91	1,00	1,12	0,91	1,14	expressed protein
260245_at	At1g74230	1,18	1,17	1,00	1,18	0,93	0,94	0,93	0,91	glycine-rich RNA-binding protein
260252_at	At1g74240	1,40	1,28	1,10	0,95	0,97	0,90	0,97	0,91	mitochondrial substrate carrier family protein
260251_at	At1g74250	1,09	0,89	0,88	0,66	0,86	1,19	1,30	1,80	DNAJ heat shock N-terminal domain-containing protein
260250_at	At1g74260	1,27	1,01	0,92	0,81	0,69	0,95	0,94	0,93	AIR synthase-related family protein
260258_at	At1g74270	1,63	1,32	1,16	1,29	1,36	1,05	0,99	1,02	60S ribosomal protein L35a (RPL35aC)
260249_s_at	At1g74290	0,63	1,02	0,97	1,00	0,83	0,97	1,04	0,75	hydrolase, alpha/beta fold family protein
260259_at	At1g74300	1,03	0,89	1,02	1,11	1,14	1,05	0,93	0,87	esterase/lipase/thioesterase family protein
260248_at	At1g74310	2,48	1,97	1,54	1,40	1,64	1,28	1,24	1,64	heat shock protein 101 (HSP101)
260244_at	At1g74320	1,07	0,98	0,82	0,83	0,97	0,91	0,84	0,82	choline kinase, putative
260255_at	At1g74330	0,82	1,14	1,10	1,02	1,04	1,16	1,24	1,02	protein kinase, putative
260257_at	At1g74340	1,10	0,98	0,85	1,20	1,22	1,01	0,88	0,95	dolichol phosphate-mannose biosynthesis regulatory protein-related
260256_at	At1g74350	1,08	1,19	1,03	0,95	0,96	1,03	0,98	0,85	intron maturase, type II family protein
260239_at	At1g74360	0,94	0,88	0,92	0,99	0,88	1,04	1,08	0,98	leucine-rich repeat transmembrane protein kinase, putative
260229_at	At1g74370	0,88	1,05	1,05	1,02	1,27	1,04	0,89	1,13	zinc finger (C3HC4-type RING finger) family protein
260222_at	At1g74380	0,96	0,98	0,93	1,06	1,03	1,06	0,97	1,04	galactosyl transferase GMA12/MNN10 family protein
260223_at	At1g74390	1,08	1,27	1,20	0,85	0,83	0,80	1,12	1,08	exonuclease family protein
260224_at	At1g74400	1,12	0,88	1,12	0,84	0,86	0,84	1,28	1,20	pentatricopeptide (PPR) repeat-containing protein
260231_at	At1g74410	0,98	1,06	1,15	0,71	0,84	1,09	1,15	1,36	zinc finger (C3HC4-type RING finger) family protein
260210_at	At1g74420	0,88	1,01	1,08	1,00	0,93	0,75	0,95	0,93	xyloglucan fucosyltransferase, putative (FUT3)
260237_at	At1g74430	0,46	0,96	1,18	0,78	1,23	0,79	1,06	1,02	myb family transcription factor (MYB95)
260211_at	At1g74440	0,87	0,88	0,78	1,04	0,71	1,16	1,32	1,00	expressed protein
260227_at	At1g74450	0,72	0,96	0,68	0,77	0,73	0,99	1,05	0,98	expressed protein
260234_at	At1g74460	1,08	1,19	1,08	1,29	1,28	0,99	1,04	0,82	GDSL-motif lipase/hydrolase family protein
260236_at	At1g74470	1,18	1,17	1,28	0,95	0,75	1,05	0,99	1,04	geranylgeranyl reductase
260212_at	At1g74480	0,88	0,85	1,06	1,10	1,26	1,06	0,81	0,65	RWP-RK domain-containing protein
260213_at	At1g74490	0,94	1,07	1,03	0,85	1,01	0,96	1,00	0,96	protein kinase, putative
260230_at	At1g74500	1,23	0,98	0,96	2,05	1,41	1,05	1,05	1,15	bHLH family protein

260214_at	At1g74510	1,04	1,06	1,07	0,98	1,04	0,94	0,99	1,02	kelch repeat-containing F-box family protein
260238_at	At1g74520	0,79	0,76	0,82	1,16	1,21	0,77	0,79	0,85	ABA-responsive protein (HVA22a)
260215_at	At1g74530	1,02	0,97	1,02	1,19	1,17	1,06	0,94	0,94	expressed protein
260228_at	At1g74540	0,95	0,96	0,94	1,07	1,02	1,05	1,02	1,12	cytochrome P450, putative
260233_at	At1g74550	0,95	0,87	1,07	0,96	1,02	0,96	0,86	0,81	cytochrome P450, putative
260235_at	At1g74560	1,75	1,50	1,36	1,18	1,23	1,09	1,04	1,09	nucleosome assembly protein (NAP) family protein
260216_at	At1g74580	1,03	1,06	1,08	1,02	0,90	0,81	0,97	0,85	pentatricopeptide (PPR) repeat-containing protein
260225_at	At1g74590	2,32	4,03	2,36	1,85	1,54	1,87	1,76	1,13	glutathione S-transferase, putative
260217_at	At1g74600	0,97	1,08	1,10	0,79	0,99	1,03	1,04	0,88	pentatricopeptide (PPR) repeat-containing protein
260218_at	At1g74620	0,97	0,98	0,96	0,94	1,09	1,10	0,94	1,08	zinc finger (C3HC4-type RING finger) family protein
260219_at	At1g74630	1,02	0,89	0,97	0,90	0,94	1,03	1,02	0,99	pentatricopeptide (PPR) repeat-containing protein
260232_at	At1g74640	0,82	0,80	1,02	0,77	0,82	1,09	0,91	0,91	expressed protein
260220_at	At1g74650	1,02	0,77	0,89	0,99	1,13	0,96	0,69	0,96	myb family transcription factor (cY13)
260226_at	At1g74660	1,07	1,00	0,77	0,91	0,83	0,80	1,07	1,04	zinc finger homeobox family protein / ZF-HD homeobox family protein
260221_at	At1g74670	1,47	1,52	1,83	0,52	0,37	1,49	1,77	1,77	gibberellin-responsive protein, putative
262223_at	At1g74680	0,70	0,64	0,68	1,04	1,01	0,91	0,75	0,92	exostosin family protein
262210_at	At1g74690	1,04	0,98	0,91	1,35	1,26	0,80	0,89	0,84	calmodulin-binding family protein
262222_at	At1g74700	1,07	0,84	0,83	1,00	0,90	0,92	0,86	0,99	RNase Z
262177_at	At1g74710	0,85	1,13	1,43	0,72	0,56	1,50	1,70	1,32	isochorismate synthase 1 (ICS1) / isochorismate mutase
262209_at	At1g74720	0,76	0,77	0,88	0,74	0,71	0,95	0,78	0,66	C2 domain-containing protein
262168_at	At1g74730	1,00	0,91	0,94	0,85	0,78	1,19	1,02	1,07	expressed protein
262220_at	At1g74740	1,09	0,72	0,98	1,01	1,12	1,10	1,04	0,92	calcium-dependent protein kinase, putative / CDPK, putative
262219_at	At1g74750	0,77	0,79	0,83	0,82	0,90	0,93	1,03	1,04	pentatricopeptide (PPR) repeat-containing protein
262218_at	At1g74760	1,82	2,26	1,62	1,46	0,97	1,88	1,83	1,33	zinc finger (C3HC4-type RING finger) family protein
262217_at	At1g74770	1,04	1,23	1,04	1,04	1,13	1,18	1,18	1,08	expressed protein
262216_at	At1g74780	0,74	0,79	0,79	0,70	0,73	0,92	0,85	0,79	nodulin family protein
262215_at	At1g74790	0,98	0,84	0,80	1,10	1,09	0,83	0,74	0,77	expressed protein
262208_at	At1g74800	0,94	1,02	0,90	0,88	0,86	1,06	0,85	0,91	galactosyltransferase family protein
259953_at	At1g74810	1,01	1,19	1,16	1,08	1,29	0,86	1,35	1,29	anion exchange family protein
262214_at	At1g74820	0,93	1,13	0,92	0,95	0,91	1,00	0,96	0,94	cupin family protein
262166_at	At1g74840	0,96	1,05	0,86	0,82	0,89	0,85	1,11	1,19	myb family transcription factor
262207_at	At1g74850	0,96	1,00	1,16	0,70	0,54	0,97	0,96	1,11	pentatricopeptide (PPR) repeat-containing protein
262169_at	At1g74860	0,89	0,90	0,87	1,18	1,21	1,01	0,97	0,99	expressed protein
262213_at	At1g74870	0,89	0,90	0,85	0,98	1,02	1,00	0,94	0,87	expressed protein
262175_at	At1g74880	0,99	0,99	0,90	0,89	0,77	1,15	1,04	1,02	expressed protein
262212_at	At1g74890	1,00	1,05	1,00	0,90	1,08	0,95	1,06	0,97	two-component responsive regulator / response regulator 15 (ARR15)
262224_at	At1g74900	1,04	1,11	1,10	1,03	1,07	0,95	1,04	0,96	pentatricopeptide (PPR) repeat-containing protein
262174_at	At1g74910	1,25	1,29	1,56	0,93	1,05	1,03	1,04	1,02	ADP-glucose pyrophosphorylase family protein
262173_at	At1g74920	0,85	0,76	0,72	0,94	0,87	0,84	0,89	0,94	betaine-aldehyde dehydrogenase, putative
262211_at	At1g74930	0,90	1,03	0,85	1,00	0,57	1,64	1,22	1,10	AP2 domain-containing transcription factor, putative
262170_at	At1g74940	1,03	0,72	0,63	1,05	1,30	0,66	0,64	0,64	senescence-associated protein-related
262171_at	At1g74950	0,70	0,92	0,99	0,95	0,94	0,97	1,08	1,17	expressed protein
262176_at	At1g74960	0,82	0,83	0,89	0,79	0,85	0,93	0,93	0,99	3-ketoacyl-ACP synthase, putative
262172_at	At1g74970	1,05	0,97	0,83	0,89	0,92	1,00	1,00	0,95	ribosomal protein S9 (RPS9)
262167_at	At1g74990	0,97	0,95	0,92	0,96	1,01	0,87	1,01	1,03	zinc finger (C3HC4-type RING finger) family protein
262221_at	At1g75010	0,85	0,84	0,75	0,73	0,78	1,02	0,95	0,99	MORN (Membrane Occupation and Recognition Nexus) repeat-containing [illegible]

262165_at	At1g75020	0,89	1,13	0,96	0,91	1,07	1,08	1,35	1,06	phospholipid/glycerol acyltransferase family protein
259987_at	At1g75030	0,91	0,67	0,81	0,97	1,38	0,96	0,92	0,85	pathogenesis-related thaumatin family protein
259925_at	At1g75040	0,75	0,72	0,65	0,76	0,49	1,50	1,15	1,29	pathogenesis-related protein 5 (PR-5)
259986_at	At1g75050	0,94	0,96	1,03	1,01	1,10	1,03	1,08	0,98	thaumatin-like protein, putative / pathogenesis-related protein, putative
259955_s_at	At1g75080	0,78	0,81	1,05	0,87	0,86	0,87	0,96	1,10	brassinosteroid signalling positive regulator, putative
259926_at	At1g75090	1,06	1,23	1,05	0,96	0,96	1,07	1,26	1,18	methyladenine glycosylase family protein
259927_at	At1g75100	1,68	2,03	2,13	1,46	1,44	1,76	1,75	1,66	expressed protein
259956_at	At1g75110	1,19	1,15	1,04	1,04	1,10	0,95	1,04	0,86	expressed protein
259954_at	At1g75130	1,02	0,95	1,15	0,79	0,91	0,86	0,86	0,92	cytochrome P450 family protein
256508_at	At1g75140	0,82	0,94	0,91	0,93	0,99	0,85	0,81	0,89	expressed protein
256507_at	At1g75150	1,07	1,11	0,94	1,12	1,10	0,97	1,03	0,98	expressed protein
256506_at	At1g75160	0,86	1,04	0,89	0,96	1,00	0,99	1,02	0,91	expressed protein
256451_s_at	At1g75170	1,23	0,96	1,29	1,10	1,10	1,11	1,26	1,26	SEC14 cytosolic factor family protein / phosphoglyceride transfer family pro
256456_at	At1g75180	1,15	1,49	1,71	0,79	0,83	0,82	0,98	1,28	expressed protein
256455_at	At1g75190	1,00	0,96	1,10	0,63	0,71	0,99	0,85	0,94	expressed protein
256505_at	At1g75200	1,13	1,07	1,23	0,97	1,13	1,04	1,05	0,99	flavodoxin family protein / radical SAM domain-containing protein
256504_at	At1g75210	1,01	0,87	0,96	0,87	0,89	1,04	1,09	1,10	5' nucleotidase family protein
256458_at	At1g75220	0,80	0,79	0,80	0,97	0,94	0,93	1,00	0,96	integral membrane protein, putative
256457_at	At1g75230	0,58	1,12	1,05	1,03	1,19	0,91	1,21	1,26	HhH-GPD base excision DNA repair family protein
256452_at	At1g75240	1,16	1,12	1,16	0,68	0,85	1,00	0,97	1,04	zinc finger homeobox family protein / ZF-HD homeobox family protein
256503_at	At1g75250	0,92	0,94	1,06	1,23	1,42	0,73	0,99	1,03	myb family transcription factor
256453_at	At1g75270	1,03	1,05	1,10	0,96	1,23	0,91	0,90	0,85	dehydroascorbate reductase, putative
256454_at	At1g75280	1,09	1,08	1,38	1,33	1,33	0,98	1,01	1,09	isoflavone reductase, putative
256450_at	At1g75290	1,12	0,97	1,00	1,07	1,20	1,03	1,08	1,01	isoflavone reductase, putative
256509_at	At1g75300	0,80	0,78	1,00	0,91	1,15	0,97	1,06	0,93	isoflavone reductase, putative
261117_at	At1g75310	0,86	0,78	1,01	0,96	0,91	1,26	1,60	2,15	DNAJ heat shock N-terminal domain-containing protein
261122_at	At1g75330	1,25	1,15	1,07	0,84	0,82	0,95	0,93	0,89	ornithine carbamoyltransferase, chloroplast / ornithine transcarbamylase / O
261121_at	At1g75340	1,08	1,22	1,35	0,80	0,93	1,03	0,94	0,91	zinc finger (CCCH-type) family protein
261119_at	At1g75350	0,93	0,85	0,84	0,90	0,97	0,96	0,98	0,92	ribosomal protein L31 family protein
261115_at	At1g75360	1,05	1,19	1,00	0,96	1,12	1,07	0,97	0,99	hypothetical protein
261116_at	At1g75370	0,87	0,95	1,29	0,85	0,92	0,95	1,00	1,07	SEC14 cytosolic factor, putative / phosphatidylinositol transfer-like protein, p
261114_at	At1g75390	0,86	0,78	0,70	1,38	1,23	0,98	0,93	0,82	bZIP transcription factor family protein
261113_at	At1g75400	0,91	0,84	1,12	0,88	0,89	0,94	1,10	0,98	expressed protein
261120_at	At1g75410	1,28	1,31	1,69	0,73	0,67	1,45	1,35	1,64	BEL1-like homeodomain 3 protein (BLH3)
261112_at	At1g75420	0,78	0,85	0,90	0,82	1,03	0,94	0,86	0,85	glycosyl transferase family 1 protein
261111_at	At1g75430	0,96	1,03	0,96	0,96	0,95	1,08	0,92	1,17	homeodomain-containing protein
261110_at	At1g75440	1,03	1,04	0,99	1,06	1,16	1,12	1,18	1,15	ubiquitin-conjugating enzyme 16 (UBC16)
261109_at	At1g75450	1,02	1,20	1,48	0,97	0,91	1,00	1,16	1,08	FAD-binding domain-containing protein / cytokinin oxidase family protein
261118_at	At1g75460	1,00	1,10	1,11	0,71	0,64	1,05	0,95	0,89	ATP-dependent protease La (LON) domain-containing protein
257461_at	At1g75470	0,99	0,93	0,91	0,87	0,96	0,99	0,93	1,16	purine permease-related
262977_at	At1g75490	1,00	1,06	1,00	1,10	1,15	1,12	1,06	1,00	DRE-binding transcription factor, putative
262951_at	At1g75500	1,25	1,46	1,29	1,38	0,82	0,74	0,76	0,85	nodulin MtN21 family protein
262950_at	At1g75510	1,22	1,10	1,18	1,07	1,02	1,12	1,05	1,07	transcription initiation factor IIF beta subunit (TFIIF-beta) family protein
262976_at	At1g75520	1,00	1,10	1,17	0,97	1,18	0,96	1,06	1,00	lateral root primordium (LRP) protein-related
262975_at	At1g75540	1,23	1,06	1,18	1,33	1,04	0,88	1,12	1,29	zinc finger (B-box type) family protein
262974_at	At1g75550	1,04	1,00	1,09	1,11	1,20	1,23	0,89	0,90	glycine-rich protein

262948_at	At1g75560	1,01	0,85	0,86	0,81	0,89	0,86	0,82	0,86	zinc knuckle (CCHC-type) family protein
257460_at	At1g75580	0,90	0,94	0,98	1,02	0,99	1,06	1,03	0,77	auxin-responsive protein, putative
262981_at	At1g75590	0,89	0,97	1,12	0,97	1,16	1,01	1,06	1,03	auxin-responsive family protein
262973_at	At1g75600	1,04	0,96	0,92	0,96	1,04	1,00	1,00	1,09	histone H3.2, putative
262979_s_at	At1g75610	1,03	0,99	1,02	1,05	0,96	1,01	0,98	1,00	histone H3, putative
262972_at	At1g75620	1,05	1,13	1,19	0,99	1,18	1,06	0,91	0,77	glyoxal oxidase-related
262924_s_at	At1g75630	1,08	0,95	0,92	1,06	0,96	1,00	0,99	0,89	vacuolar ATP synthase 16 kDa proteolipid subunit 2 / V-ATPase 16 kDa prc
262971_at	At1g75640	0,94	1,05	1,07	1,02	1,10	0,84	0,94	0,96	leucine-rich repeat family protein / protein kinase family protein
262923_at	At1g75660	0,99	0,99	0,97	0,89	0,87	0,92	0,85	1,12	5'-3' exoribonuclease (XRN3)
262953_at	At1g75670	1,23	0,80	0,92	1,06	1,06	1,01	0,83	1,08	expressed protein
262980_at	At1g75680	0,91	0,81	0,81	1,23	1,18	0,82	0,72	0,76	glycosyl hydrolase family 9 protein
262970_at	At1g75690	1,10	1,01	1,03	0,90	0,96	1,13	0,97	0,92	chaperone protein dnaJ-related
262969_at	At1g75710	0,99	1,42	1,05	1,53	1,20	0,91	1,06	1,14	zinc finger (C2H2 type) family protein
262968_at	At1g75720	0,97	0,90	0,88	1,14	1,06	0,87	0,89	1,18	hypothetical protein
262967_at	At1g75730	0,74	0,95	1,06	0,84	1,13	1,06	1,03	1,33	expressed protein
262947_at	At1g75750	1,20	1,05	0,98	1,24	1,11	1,04	1,05	1,02	gibberellin-regulated protein 1 (GASA1) / gibberellin-responsive protein 1
262966_at	At1g75760	1,09	0,97	0,86	0,93	1,00	0,94	0,85	0,90	ER lumen protein retaining receptor family protein
262952_at	At1g75770	1,19	1,16	1,22	0,97	0,99	1,10	0,98	1,03	expressed protein
262978_at	At1g75780	1,38	1,31	1,31	1,32	1,22	0,70	0,65	0,62	tubulin beta-1 chain (TUB1)
262949_at	At1g75790	1,03	0,94	1,01	0,92	0,98	1,01	1,00	0,96	multi-copper oxidase type I family protein
262727_at	At1g75800	0,95	1,49	1,47	0,73	0,79	0,81	1,07	1,33	pathogenesis-related thaumatin family protein
262678_at	At1g75810	0,73	1,07	0,96	0,95	0,90	1,16	1,11	1,10	expressed protein
262728_at	At1g75820	0,98	0,99	0,83	0,87	0,74	0,83	0,83	0,70	CLAVATA1 receptor kinase (CLV1)
262679_at	At1g75830	0,98	0,81	0,88	0,59	0,54	1,09	1,02	0,85	plant defensin-fusion protein, putative (PDF1.1)
262729_at	At1g75840	1,11	0,89	0,98	1,19	1,01	1,03	0,86	0,98	Rac-like GTP-binding protein (ARAC5) / Rho-like GTP-binding protein (ROF
262695_at	At1g75850	0,80	0,70	0,72	0,89	1,11	1,00	1,02	1,06	vacuolar protein sorting-associated protein 35 family protein / VPS35 family
262677_at	At1g75860	1,37	2,03	2,56	1,20	0,90	1,37	1,96	2,21	expressed protein
262696_at	At1g75870	0,94	1,20	1,02	1,01	1,08	1,16	0,90	0,94	hypothetical protein
262680_at	At1g75880	0,55	0,63	0,65	1,22	0,95	0,65	0,49	0,43	family II extracellular lipase 1 (EXL1)
262681_at	At1g75890	0,98	1,01	1,04	1,03	0,96	0,92	0,99	0,99	family II extracellular lipase 2 (EXL2)
262682_at	At1g75900	1,06	0,88	1,03	1,65	1,12	0,89	0,83	0,85	family II extracellular lipase 3 (EXL3)
262674_at	At1g75910	0,97	1,03	1,16	0,98	0,95	1,04	1,04	1,00	family II extracellular lipase 4 (EXL4)
262683_at	At1g75920	1,00	0,90	0,92	1,16	0,96	1,06	0,99	0,98	family II extracellular lipase 5 (EXL5)
262675_at	At1g75930	0,88	1,05	0,94	0,93	1,07	1,00	0,98	1,01	family II extracellular lipase 6 (EXL6)
262697_at	At1g75940	1,03	0,96	1,08	0,97	1,01	1,03	1,07	0,90	glycosyl hydrolase family 1 protein / anther-specific protein ATA27
262676_at	At1g75950	1,11	1,03	1,05	1,02	1,03	0,94	0,96	1,01	E3 ubiquitin ligase SCF complex subunit SKP1/ASK1 (At1)
262698_at	At1g75960	0,73	0,61	1,17	0,98	0,96	0,53	0,73	1,14	AMP-binding protein, putative
262699_at	At1g75980	1,19	1,06	1,13	1,06	1,11	1,08	1,05	1,10	expressed protein
262686_at	At1g75990	1,03	0,85	1,21	1,12	1,30	1,00	0,81	0,98	26S proteasome regulatory subunit S3, putative (RPN3)
262685_at	At1g76010	1,26	1,34	1,33	0,82	0,95	0,98	1,05	1,07	expressed protein
262700_at	At1g76020	0,59	0,69	0,51	1,01	0,98	0,73	0,55	0,51	expressed protein
262684_s_at	At1g76030	0,85	0,76	0,74	0,79	0,81	0,86	0,93	0,92	vacuolar ATP synthase subunit B, putative / V-ATPase B subunit, putative /
262670_s_at	At1g76035	1,01	1,13	1,10	0,94	1,12	1,07	1,11	1,28	bacterial IS-element
262671_at	At1g76040	0,69	0,70	0,72	1,04	0,95	0,99	1,06	1,07	calcium-dependent protein kinase, putative / CDPK, putative
262672_at	At1g76050	0,99	1,02	1,22	0,81	0,91	1,08	1,06	1,08	pseudouridine synthase family protein
262673_at	At1g76060	0,88	0,88	0,73	1,22	1,12	0,88	0,86	0,86	complex 1 family protein / LVR family protein

261748_at	At1g76070	1,07	0,82	0,83	1,00	1,15	1,13	0,98	0,96	expressed protein
261751_at	At1g76080	1,03	1,02	1,20	0,98	0,99	0,91	0,87	0,84	thioredoxin family protein
261727_at	At1g76090	0,93	0,85	0,86	0,82	0,79	0,88	0,74	0,78	S-adenosyl-methionine-sterol-C-methyltransferase
261769_at	At1g76100	1,09	1,01	1,12	0,93	0,98	0,99	0,92	0,98	plastocyanin
261782_at	At1g76110	1,46	1,83	4,09	0,60	0,80	1,51	1,36	1,27	high mobility group (HMG1/2) family protein / ARID/BRIGHT DNA-binding d
261750_at	At1g76120	1,02	0,80	0,90	0,86	0,80	0,91	0,90	1,01	tRNA pseudouridine synthase family protein
261754_at	At1g76130	0,84	0,68	0,81	1,80	1,35	0,97	0,82	0,77	alpha-amylase, putative / 1,4-alpha-D-glucan glucanohydrolase, putative
261770_at	At1g76140	0,96	0,86	0,92	1,03	1,04	1,00	0,94	0,97	prolyl oligopeptidase, putative / prolyl endopeptidase, putative / post-proline
261771_at	At1g76150	0,72	0,76	0,81	1,02	1,11	0,92	0,85	0,89	maoC-like dehydratase domain-containing protein
261728_at	At1g76160	1,09	0,86	0,92	1,43	1,23	0,91	0,87	0,90	multi-copper oxidase type I family protein
267397_at	At1g76170	1,18	0,99	1,18	0,77	0,88	1,17	1,22	1,37	expressed protein
261749_at	At1g76180	1,01	0,93	0,96	1,16	1,20	1,04	1,00	1,11	dehydrin (ERD14)
261724_at	At1g76185	0,92	1,17	0,98	0,96	1,24	0,91	0,89	1,09	expressed protein
261776_at	At1g76190	0,97	1,03	1,18	1,18	1,00	0,95	0,99	1,14	auxin-responsive family protein
261723_at	At1g76200	1,23	1,13	1,01	1,27	1,31	0,93	0,92	0,92	expressed protein
261777_at	At1g76210	1,55	1,27	1,14	0,89	1,01	2,03	1,07	1,12	expressed protein
261778_at	At1g76220	0,97	1,04	0,92	0,99	0,95	0,99	1,07	0,86	hypothetical protein
261779_at	At1g76230	1,01	1,04	1,02	1,07	1,08	1,13	1,13	1,09	expressed protein
261772_at	At1g76240	1,91	1,63	1,53	1,55	1,30	0,95	0,89	1,12	expressed protein
261773_at	At1g76250	0,94	1,07	1,06	1,06	0,99	1,07	1,16	0,96	expressed protein
261774_at	At1g76260	0,76	0,68	0,72	1,03	1,18	0,80	0,85	0,66	transducin family protein / WD-40 repeat family protein
261726_at	At1g76270	1,07	0,89	1,36	1,26	1,11	0,97	1,04	1,26	expressed protein
261775_at	At1g76280	1,11	1,07	1,00	1,07	0,99	1,11	0,87	1,12	pentatricopeptide (PPR) repeat-containing protein
261752_at	At1g76290	0,97	1,00	1,00	0,91	1,17	1,05	1,00	0,92	AMP-dependent synthetase and ligase family protein
261725_at	At1g76300	1,41	1,31	1,14	0,89	0,96	1,03	1,03	1,04	small nuclear ribonucleoprotein D3, putative / snRNP core protein D3, putat
261780_at	At1g76310	1,46	1,38	1,36	1,30	1,27	0,84	0,88	1,01	cyclin, putative
261781_at	At1g76320	1,26	1,39	1,34	0,94	0,95	1,05	1,11	1,34	far-red impaired responsive protein, putative
261753_at	At1g76340	0,93	0,93	0,87	1,00	1,00	0,96	0,98	0,91	integral membrane family protein
259888_at	At1g76350	0,76	0,91	0,90	0,94	0,94	1,07	1,00	0,94	RWP-RK domain-containing protein
259887_at	At1g76360	0,68	0,80	0,95	0,86	1,06	1,14	1,48	1,62	protein kinase, putative
259886_at	At1g76370	1,00	1,16	0,89	1,07	1,02	1,01	1,01	0,85	protein kinase, putative
259885_at	At1g76380	0,72	0,69	0,79	0,84	1,06	1,02	1,04	1,02	DNA-binding bromodomain-containing protein
259884_at	At1g76390	0,77	0,87	1,44	0,99	1,14	0,97	1,08	0,98	armadillo/beta-catenin repeat family protein / U-box domain-containing prot
259883_at	At1g76400	1,07	0,89	1,00	1,16	1,00	0,88	0,88	0,91	ribophorin I family protein
259889_at	At1g76405	1,52	0,99	1,25	0,87	1,20	0,96	0,93	1,06	expressed protein
259982_at	At1g76410	1,01	1,02	1,23	0,95	1,09	0,82	1,28	1,81	zinc finger (C3HC4-type RING finger) family protein
259972_at	At1g76420	0,98	1,05	0,99	1,16	1,07	0,86	1,00	0,99	no apical meristem (NAM) family protein
259974_at	At1g76430	0,89	0,88	0,98	0,95	0,89	0,91	1,08	1,20	phosphate transporter family protein
259981_at	At1g76450	0,81	0,70	0,69	0,91	0,82	1,04	0,99	0,98	oxygen-evolving complex-related
259984_at	At1g76460	0,79	0,89	0,87	0,88	0,86	0,81	0,82	0,86	RNA recognition motif (RRM)-containing protein
259975_at	At1g76470	0,89	1,01	0,94	1,15	0,93	1,26	1,13	1,19	cinnamoyl-CoA reductase family
259983_at	At1g76490	0,85	0,91	0,81	0,76	0,74	0,91	0,82	0,90	3-hydroxy-3-methylglutaryl-CoA reductase 1 / HMG-CoA reductase 1 (HMG
259966_at	At1g76500	0,88	0,91	0,88	1,28	1,11	1,17	1,02	0,91	DNA-binding family protein
259967_at	At1g76510	0,98	0,95	0,98	0,71	0,83	1,05	1,12	1,23	ARID/BRIGHT DNA-binding domain-containing protein
259980_at	At1g76520	0,81	0,90	1,02	0,81	0,79	1,02	1,06	1,11	auxin efflux carrier family protein
259968_at	At1g76530	0,74	0,84	0,79	0,56	0,87	1,16	1,04	0,91	auxin efflux carrier family protein

259978_at	At1g76540	1,44	1,18	1,23	1,80	1,73	0,86	0,87	0,80	cell division control protein, putative
259969_at	At1g76550	1,05	1,00	0,98	1,42	1,26	0,89	0,89	0,87	pyrophosphate--fructose-6-phosphate 1-phosphotransferase alpha subunit,
259976_at	At1g76560	1,09	1,16	1,35	1,34	1,50	0,95	0,89	0,91	CP12 domain-containing protein
259970_at	At1g76570	1,59	1,44	1,49	0,84	0,79	1,58	1,54	1,94	chlorophyll A-B binding family protein
259971_at	At1g76580	0,75	0,71	0,97	0,92	0,96	1,03	1,14	1,38	SPL1-Related3 protein (SPL1R3)
259977_at	At1g76590	1,10	1,10	1,10	0,73	0,53	1,21	1,37	1,33	zinc-binding family protein
259979_at	At1g76600	0,83	0,98	1,00	0,76	0,71	1,27	1,13	1,27	expressed protein
259985_at	At1g76620	0,93	1,05	1,06	1,24	1,11	0,98	1,03	1,08	expressed protein
259973_at	At1g76630	1,16	1,10	1,41	0,93	0,77	1,30	1,05	1,26	tetratricopeptide repeat (TPR)-containing protein
259866_at	At1g76640	0,98	1,56	0,81	1,90	1,73	0,92	1,32	0,99	calmodulin-related protein, putative
259879_at	At1g76650	0,80	1,00	0,80	0,80	0,67	1,05	1,49	1,48	calcium-binding EF hand family protein
259874_at	At1g76660	0,77	0,63	0,82	1,14	0,95	0,96	0,76	1,12	expressed protein
259882_at	At1g76670	0,78	0,74	0,72	1,08	1,03	0,82	0,72	0,79	transporter-related
259875_s_at	At1g76690	0,79	0,89	1,56	0,69	0,82	0,90	0,97	1,09	12-oxophytodienoate reductase (OPR1)
259876_at	At1g76700	0,86	0,84	1,64	0,95	1,09	1,14	1,45	1,65	DNAJ heat shock N-terminal domain-containing protein
259877_at	At1g76710	1,18	1,15	1,49	1,12	0,79	0,98	1,04	1,19	SET domain-containing protein (ASHH1)
259873_s_at	At1g76720	0,96	0,93	1,11	0,90	0,96	1,01	1,15	1,14	eukaryotic translation initiation factor 2 family protein / eIF-2 family protein
259880_at	At1g76730	0,84	0,97	0,90	0,76	0,80	1,12	1,15	1,11	5-formyltetrahydrofolate cyclo-ligase family protein
259867_at	At1g76740	1,13	1,08	1,05	1,04	1,07	0,87	1,02	1,06	expressed protein
259868_at	At1g76760	1,28	1,16	0,98	0,98	1,02	0,99	1,15	1,24	thioredoxin family protein
259869_at	At1g76770	0,98	1,02	0,99	1,05	1,05	1,11	0,92	1,03	heat shock protein-related
259870_at	At1g76780	1,06	0,80	1,02	1,00	1,03	1,01	1,02	0,92	expressed protein
259878_at	At1g76790	0,59	0,59	0,63	1,11	0,93	0,81	1,01	1,07	O-methyltransferase family 2 protein
259871_at	At1g76800	1,18	1,11	0,98	1,23	1,42	0,86	0,96	0,87	nodulin, putative
259872_at	At1g76810	0,94	0,90	1,03	0,77	0,79	1,11	1,13	1,10	eukaryotic translation initiation factor 2 family protein / eIF-2 family protein
259881_at	At1g76820	0,93	0,88	0,97	0,85	0,71	0,96	1,04	0,93	expressed protein
256329_at	At1g76850	0,91	0,89	0,95	0,88	1,17	1,06	1,03	0,99	expressed protein
256333_at	At1g76860	1,32	1,40	1,33	1,01	1,26	1,07	0,91	0,78	small nuclear ribonucleoprotein, putative / snRNP, putative / Sm protein, pu
256330_at	At1g76870	0,99	1,01	0,93	0,86	0,98	0,81	1,00	0,98	hypothetical protein
256331_at	At1g76880	0,79	0,74	0,63	1,00	1,10	0,99	0,92	1,11	trihelix DNA-binding protein, putative
256332_at	At1g76890	1,41	1,34	1,08	1,11	1,25	1,21	1,31	0,91	trihelix DNA-binding protein / GT-2 factor (GT2)
264952_at	At1g76900	1,26	1,77	1,93	1,09	1,28	1,12	1,67	1,60	F-box family protein / tubby family protein
264943_at	At1g76910	1,10	1,07	0,94	1,01	0,90	1,05	1,10	1,09	hypothetical protein
264955_at	At1g76920	0,57	0,63	0,73	0,87	1,08	0,86	0,68	0,65	F-box family protein (FBX3)
264960_at	At1g76930	1,59	1,53	1,25	1,28	0,95	1,65	1,18	0,92	proline-rich extensin-like family protein
264944_at	At1g76940	0,90	1,06	1,17	1,17	1,05	1,09	0,97	1,03	RNA recognition motif (RRM)-containing protein
264961_at	At1g76950	0,87	0,84	0,80	1,07	1,11	0,98	0,84	0,89	zinc finger protein (PRAF1) / regulator of chromosome condensation (RCC1
264958_at	At1g76960	0,94	0,95	0,94	1,21	1,23	1,46	1,06	1,16	expressed protein
264951_at	At1g76970	0,60	0,72	1,17	1,08	0,95	0,95	1,21	1,51	VHS domain-containing protein / GAT domain-containing protein
264956_at	At1g76990	1,16	0,93	1,01	1,01	1,04	1,10	1,16	1,29	ACT domain containing protein
264957_at	At1g77000	0,89	1,34	1,43	0,76	0,85	0,98	1,34	1,35	F-box family protein
264946_at	At1g77010	0,93	0,98	0,97	0,70	0,85	0,94	0,95	1,11	pentatricopeptide (PPR) repeat-containing protein
264947_at	At1g77020	0,90	0,72	0,66	1,17	0,89	0,89	1,06	0,92	DNAJ heat shock N-terminal domain-containing protein
264948_at	At1g77050	1,21	1,12	1,07	1,04	1,20	0,89	1,05	1,00	DEAD/DEAH box helicase, putative
264954_at	At1g77060	0,80	0,73	0,93	0,86	1,00	0,86	0,96	0,80	mutase family protein
264945_at	At1g77070	1,03	0,97	0,90	1,14	0,83	0,82	1,01	0,83	MADS-box protein AGL27-II (AGL27) / MADS affecting flowering 1 (MAF1)

264949_at	At1g77080	1,01	0,93	0,97	1,08	0,88	1,06	1,02	0,99	MADS-box protein AGL27-II (AGL27) / MADS affecting flowering 1 (MAF1)
264959_at	At1g77090	0,92	0,84	1,02	0,82	0,80	1,22	1,09	1,09	thylakoid lumenal 29.8 kDa protein
264950_at	At1g77100	1,03	1,00	0,96	0,92	0,99	1,05	0,90	1,05	peroxidase, putative
264962_at	At1g77110	0,98	1,32	1,00	0,89	0,85	0,97	0,89	0,96	auxin transport protein, putative
264953_at	At1g77120	0,28	0,29	0,33	1,91	1,97	0,94	0,69	0,55	alcohol dehydrogenase (ADH)
264476_at	At1g77130	0,90	0,79	0,60	0,64	0,76	1,00	0,94	0,80	glycogenin glucosyltransferase (glycogenin)-related
264487_at	At1g77140	1,06	0,93	1,05	0,96	1,30	1,04	0,86	1,09	vacuolar protein sorting protein 45, putative / VPS45p, putative
264475_s_at	At1g77170	1,15	0,75	0,84	0,82	0,90	1,01	0,99	1,13	expressed protein
264486_at	At1g77180	1,13	1,09	1,27	0,92	0,98	1,08	1,07	1,15	chromatin protein family
264481_at	At1g77200	0,92	0,90	1,07	1,00	1,15	0,92	0,85	0,80	AP2 domain-containing transcription factor TINY, putative
264482_at	At1g77210	0,69	1,15	1,51	0,70	0,51	0,67	0,99	1,00	sugar transporter, putative
264485_at	At1g77220	0,85	0,84	0,98	0,97	0,93	0,94	1,21	1,37	expressed protein
264483_at	At1g77230	0,84	0,73	0,91	0,99	1,17	1,00	0,91	0,93	tetratricopeptide repeat (TPR)-containing protein
264477_at	At1g77240	1,09	1,00	1,16	0,93	1,10	1,15	1,10	1,09	AMP-binding protein, putative
257420_at	At1g77250	0,86	0,91	1,40	0,96	0,96	1,08	1,36	1,46	PHD finger family protein
264484_at	At1g77260	0,95	0,84	0,72	0,92	1,03	0,93	0,95	0,94	dehydration-responsive protein-related
264478_at	At1g77270	0,95	1,04	1,04	0,94	1,06	0,80	1,07	1,15	expressed protein
264479_at	At1g77280	1,36	1,51	1,20	0,95	0,92	1,05	1,01	1,01	protein kinase family protein
264480_at	At1g77300	0,92	0,92	1,09	1,02	0,93	1,08	1,05	1,18	SET domain-containing protein
246413_at	At1g77310	1,16	1,06	0,97	0,91	1,02	0,96	0,94	1,00	wound-responsive protein, putative
246390_at	At1g77330	1,47	1,21	1,07	3,15	2,48	1,00	0,97	1,14	1-aminocyclopropane-1-carboxylate oxidase, putative / ACC oxidase, putati
246414_at	At1g77340	0,93	0,97	1,12	1,03	1,06	1,04	0,98	0,98	pentatricopeptide (PPR) repeat-containing protein
246391_at	At1g77350	1,16	1,15	1,13	1,11	1,17	0,98	0,98	0,97	expressed protein
246383_at	At1g77360	0,91	0,95	1,02	1,10	0,88	0,90	1,21	0,94	pentatricopeptide (PPR) repeat-containing protein
246384_at	At1g77370	0,95	0,92	0,83	0,98	0,99	0,87	0,89	0,83	glutaredoxin, putative
246389_at	At1g77380	1,43	0,99	1,42	1,25	1,08	1,46	1,55	1,67	amino acid carrier, putative / amino acid permease, putative
246385_at	At1g77390	0,79	0,79	0,75	0,93	1,09	0,98	1,16	1,17	cyclin, putative
246387_at	At1g77400	0,98	1,04	1,05	1,00	1,11	1,11	0,89	1,02	expressed protein
246388_at	At1g77405	0,96	1,06	1,04	0,98	0,93	1,05	0,99	0,99	pentatricopeptide (PPR) repeat-containing protein
246386_at	At1g77410	0,87	0,93	0,90	0,92	1,09	0,96	0,91	0,83	beta-galactosidase, putative / lactase, putative
259708_at	At1g77420	0,86	0,97	0,86	1,15	0,91	1,01	0,93	0,85	hydrolase, alpha/beta fold family protein
259712_at	At1g77440	1,15	1,09	1,07	1,25	1,20	0,91	0,91	0,92	20S proteasome beta subunit C (PBC2)
259705_at	At1g77450	0,70	1,06	1,21	0,53	0,48	1,17	1,70	1,79	no apical meristem (NAM) family protein
259731_at	At1g77460	0,85	1,28	1,30	0,82	1,11	0,99	1,00	1,24	C2 domain-containing protein / armadillo/beta-catenin repeat family protein
259732_at	At1g77470	1,14	1,24	1,25	0,94	0,96	1,11	1,08	0,98	replication factor C 36 kDA, putative
259733_at	At1g77480	0,96	0,84	0,95	0,62	0,69	0,99	0,97	1,10	nucellin protein, putative
259707_at	At1g77490	0,69	0,65	0,54	0,81	0,91	1,16	1,04	1,01	L-ascorbate peroxidase, thylakoid-bound (tAPX)
259734_at	At1g77500	0,74	0,74	0,79	0,99	0,96	1,09	1,40	1,25	expressed protein
259757_at	At1g77510	0,90	0,86	0,79	0,97	0,84	1,11	0,98	0,93	protein disulfide isomerase, putative
259758_s_at	At1g77530	0,91	0,97	0,95	1,12	0,79	0,78	0,99	0,90	O-methyltransferase family 2 protein
259706_at	At1g77540	1,21	0,98	1,11	1,29	1,49	0,90	0,79	0,77	expressed protein
259759_at	At1g77550	1,18	1,02	1,10	0,91	0,66	0,93	1,08	1,13	tubulin-tyrosine ligase family protein
259711_at	At1g77570	1,03	0,98	1,13	0,93	0,97	1,13	1,02	1,08	heat shock transcription factor family protein
259760_at	At1g77580	0,87	0,92	1,08	0,77	0,98	1,12	1,04	0,79	myosin heavy chain-related
259761_at	At1g77590	0,82	0,78	0,74	0,86	0,98	0,69	0,65	0,63	long-chain-fatty-acid--CoA ligase family protein / long-chain acyl-CoA synth
259762_at	At1g77600	0,94	0,98	0,95	0,82	0,90	1,11	1,16	1,11	expressed protein

259713_at	At1g77610	0,95	0,94	0,76	1,21	1,09	1,02	0,89	0,83	glucose-6-phosphate/phosphate translocator-related
259763_at	At1g77630	1,14	0,84	0,78	0,96	1,02	0,86	0,92	0,93	peptidoglycan-binding LysM domain-containing protein
259729_at	At1g77640	1,20	1,00	0,69	0,91	0,83	1,12	1,11	0,93	AP2 domain-containing transcription factor, putative
259709_at	At1g77655	0,99	1,09	1,05	1,06	1,01	1,03	1,04	1,10	expressed protein
259730_at	At1g77660	1,42	1,36	1,04	1,02	0,97	1,06	1,05	0,99	MORN (Membrane Occupation and Recognition Nexus) repeat-containing p
259710_at	At1g77670	1,17	0,97	0,89	1,01	1,03	1,05	0,99	0,95	aminotransferase class I and II family protein
259704_at	At1g77680	1,47	1,44	1,36	1,82	2,15	1,02	1,22	1,27	ribonuclease II family protein
259680_at	At1g77690	1,17	1,24	0,92	1,06	0,89	0,85	0,73	0,73	amino acid permease, putative
259674_at	At1g77700	1,08	1,15	1,04	1,11	1,08	0,96	1,06	1,07	pathogenesis-related thaumatin family protein
259675_at	At1g77710	1,11	1,07	1,00	1,18	1,34	0,99	0,89	0,84	expressed protein
259679_at	At1g77720	0,94	1,02	1,03	1,11	1,22	0,82	0,81	0,97	protein kinase family protein
259676_at	At1g77730	1,00	0,88	1,12	1,00	1,15	0,98	0,97	1,02	pleckstrin homology (PH) domain-containing protein
259677_at	At1g77740	0,99	1,06	1,25	0,70	0,94	0,92	1,20	1,28	1-phosphatidylinositol-4-phosphate 5-kinase, putative / PIP kinase, putative
259678_at	At1g77750	1,42	1,24	1,18	1,20	1,57	0,83	0,74	0,74	30S ribosomal protein S13, chloroplast, putative
259681_at	At1g77760	0,66	0,83	1,08	0,77	0,74	0,86	0,90	1,01	nitrate reductase 1 (NR1)
259701_at	At1g77770	0,93	0,74	0,77	0,98	1,10	0,80	0,71	0,72	expressed protein
259702_at	At1g77780	0,99	0,99	1,01	0,91	1,05	0,91	0,89	0,87	glycosyl hydrolase family 17 protein
259703_at	At1g77790	0,91	1,08	1,04	0,89	0,99	0,99	0,94	0,98	glycosyl hydrolase family 17 protein
259673_at	At1g77800	0,82	0,90	0,82	0,77	0,67	1,14	1,13	1,01	PHD finger family protein
262188_at	At1g77810	0,99	1,11	0,96	0,89	0,85	1,05	1,09	0,87	galactosyltransferase family protein
262191_at	At1g77820	1,05	1,02	0,94	1,06	1,03	1,08	1,07	0,88	---
262192_at	At1g77830	0,92	0,99	0,97	0,96	0,90	1,06	0,93	1,09	zinc finger (C3HC4-type RING finger) family protein
262193_at	At1g77840	0,76	0,60	0,63	1,04	1,11	0,83	0,75	0,81	eukaryotic translation initiation factor 5, putative / eIF-5, putative
262136_at	At1g77850	0,73	0,57	0,70	0,81	1,31	1,04	0,86	0,94	transcriptional factor B3 family protein
262178_at	At1g77860	1,01	1,00	1,22	0,96	0,97	1,00	1,05	1,09	rhomboid family protein
262196_at	At1g77870	1,20	1,12	1,30	0,89	1,07	1,01	0,99	0,83	expressed protein
262182_at	At1g77890	0,97	1,08	1,16	1,00	0,95	0,94	0,94	0,98	expressed protein
262183_at	At1g77900	0,85	1,07	1,46	1,09	1,09	1,20	1,26	1,08	expressed protein
262184_at	At1g77910	1,02	0,95	1,03	1,09	1,08	1,03	0,94	0,98	hypothetical protein
262137_at	At1g77920	1,02	1,00	1,06	1,09	1,21	1,12	1,27	1,29	bZIP family transcription factor
262194_at	At1g77930	0,93	0,97	1,14	0,92	1,00	1,05	1,16	1,29	DNAJ heat shock N-terminal domain-containing protein
262163_at	At1g77940	1,41	1,13	1,07	1,30	1,17	1,01	0,93	0,90	60S ribosomal protein L30 (RPL30B)
262185_at	At1g77950	1,05	1,00	0,95	0,94	0,95	1,13	1,02	0,99	MADS-box family protein
262186_at	At1g77960	0,97	0,97	0,99	1,01	1,08	1,09	0,94	1,07	hypothetical protein
262187_at	At1g77970	0,96	1,10	0,91	1,22	1,03	1,06	1,15	0,97	hydroxyproline-rich glycoprotein family protein
262179_at	At1g77980	0,86	1,09	1,06	0,96	0,99	1,05	1,03	1,25	MADS-box family protein
262134_at	At1g77990	1,10	1,44	0,97	1,04	0,73	0,97	1,25	1,06	sulfate transporter
262133_at	At1g78000	1,13	1,45	0,80	0,73	0,77	2,03	1,83	1,88	sulfate transporter (Sultr1;2)
262189_at	At1g78010	1,17	1,12	1,04	0,93	0,66	1,06	1,04	1,06	tRNA modification GTPase, putative
262162_at	At1g78020	1,78	1,59	1,23	1,44	1,31	1,21	1,05	1,38	senescence-associated protein-related
262190_at	At1g78030	0,90	0,93	1,10	1,04	1,27	1,02	1,04	0,80	expressed protein
262195_at	At1g78040	1,06	0,95	0,95	1,29	1,28	1,03	0,97	0,97	pollen Ole e 1 allergen and extensin family protein
262180_at	At1g78050	1,17	1,13	0,90	1,38	1,31	0,95	1,07	1,15	phosphoglycerate/bisphosphoglycerate mutase family protein
262181_at	At1g78060	1,05	0,92	0,85	1,44	1,32	0,95	0,81	0,77	glycosyl hydrolase family 3 protein
262164_at	At1g78070	0,59	1,70	3,74	0,72	1,14	1,22	3,02	3,19	WD-40 repeat family protein
262135_at	At1g78080	0,99	1,02	0,86	1,05	0,88	1,10	1,30	1,29	AP2 domain-containing transcription factor RAP2.4

260059_at	At1g78090	1,34	1,10	1,00	0,97	1,01	1,00	0,93	1,15	trehalose-6-phosphate phosphatase (TPPB)
260058_at	At1g78100	0,82	0,84	0,96	1,17	1,10	1,16	0,95	1,04	F-box family protein
257502_at	At1g78110	1,16	0,98	1,02	0,95	0,82	0,93	0,97	1,15	expressed protein
260053_at	At1g78120	1,28	1,14	1,12	0,99	1,03	1,04	1,07	1,18	tetratricopeptide repeat (TPR)-containing protein
260054_at	At1g78130	0,79	0,96	1,01	0,98	1,23	0,84	0,91	0,85	transporter-related
260056_at	At1g78140	1,10	0,97	0,99	1,02	0,98	1,16	1,06	0,98	methyltransferase-related
260055_at	At1g78150	1,29	1,29	1,36	0,92	1,10	1,02	0,99	1,11	expressed protein
260080_at	At1g78160	1,03	1,02	0,96	1,04	0,90	0,99	0,99	1,00	pumilio/Puf RNA-binding domain-containing protein
260081_at	At1g78170	1,24	1,20	1,00	1,00	1,31	0,86	0,77	0,69	expressed protein
260082_at	At1g78180	1,01	0,98	1,11	0,89	0,83	0,79	0,97	1,28	mitochondrial substrate carrier family protein
260057_at	At1g78200	1,15	1,16	1,45	1,05	0,77	1,25	1,40	1,48	protein phosphatase 2C, putative / PP2C, putative
260051_at	At1g78210	1,01	0,95	1,15	1,49	1,39	0,87	0,80	0,79	hydrolase, alpha/beta fold family protein
260052_at	At1g78220	1,01	1,04	1,02	0,98	1,15	0,94	1,12	1,03	14-3-3 protein GF14 pi (GRF13)
260800_at	At1g78240	0,97	0,98	1,41	1,09	1,36	1,00	1,06	1,20	dehydration-responsive protein-related
260806_at	At1g78260	1,25	1,11	1,23	0,91	1,16	0,93	0,83	0,89	RNA recognition motif (RRM)-containing protein
260799_at	At1g78270	0,39	0,52	0,90	0,74	1,16	0,63	0,63	1,01	UDP-glucose glucosyltransferase, putative
260798_at	At1g78280	0,56	0,85	1,49	1,08	0,90	1,12	1,48	1,65	transcription factor jumonji (jmjC) domain-containing protein
260774_at	At1g78290	3,67	3,56	3,40	1,01	1,02	2,77	4,10	4,06	serine/threonine protein kinase, putative
260775_at	At1g78300	1,11	0,94	0,88	1,38	1,25	0,84	0,82	0,87	14-3-3 protein GF14 omega (GRF2)
260807_at	At1g78310	0,82	1,07	0,95	0,98	1,09	0,84	0,86	1,04	VQ motif-containing protein
260805_at	At1g78320	1,02	1,17	0,87	1,09	1,09	0,91	0,94	0,82	glutathione S-transferase, putative
260803_at	At1g78340	1,69	2,39	1,43	1,08	0,60	1,90	1,62	1,31	glutathione S-transferase, putative
260796_at	At1g78360	1,02	1,01	1,12	0,94	0,98	0,90	0,95	1,00	glutathione S-transferase, putative
260745_at	At1g78370	1,33	1,00	0,98	1,35	1,02	0,89	0,76	0,77	glutathione S-transferase, putative
260746_at	At1g78380	0,77	0,68	0,70	0,74	0,84	0,82	0,86	0,79	glutathione S-transferase, putative
260797_at	At1g78390	1,08	0,89	1,00	1,08	1,10	1,11	1,02	1,04	9-cis-epoxycarotenoid dioxygenase, putative / neoxanthin cleavage enzyme
260802_at	At1g78400	0,96	1,04	1,04	0,98	1,04	0,97	0,96	1,01	glycoside hydrolase family 28 protein / polygalacturonase (pectinase) family
260804_at	At1g78410	0,95	0,96	1,01	0,81	1,05	1,03	0,99	1,06	VQ motif-containing protein
260747_at	At1g78420	0,93	0,98	1,09	0,90	1,08	1,08	1,25	1,39	expressed protein
260801_at	At1g78430	1,41	1,11	0,97	1,40	1,21	1,01	1,02	0,77	tropomyosin-related
260773_at	At1g78440	0,93	1,02	1,01	0,98	1,00	1,00	1,07	1,01	gibberellin 2-oxidase / GA2-oxidase (GA2OX1)
263133_at	At1g78450	1,61	1,74	1,60	0,88	1,12	1,32	0,93	0,87	SOUL heme-binding family protein
263126_at	At1g78460	0,85	1,30	1,26	0,57	0,55	0,72	0,72	0,71	SOUL heme-binding family protein
263125_at	At1g78470	1,05	1,00	0,96	0,95	1,01	1,04	1,04	1,15	hypothetical protein
263124_at	At1g78480	1,10	1,14	1,26	0,93	1,00	0,97	1,06	0,96	prenyltransferase/squalene oxidase repeat-containing protein
263120_at	At1g78490	0,92	0,87	0,88	1,17	1,08	0,82	0,87	0,66	cytochrome P450 family protein
263123_at	At1g78500	1,01	0,98	1,07	0,98	1,07	0,94	1,03	0,98	pentacyclic triterpene synthase, putative
263122_at	At1g78510	1,85	2,17	2,61	1,10	1,02	2,03	1,75	1,62	solanesyl diphosphate synthase (SPS)
263121_at	At1g78530	0,89	0,83	1,00	1,00	1,05	0,76	0,83	0,89	protein kinase family protein
263135_at	At1g78550	0,83	0,94	0,85	0,98	0,82	0,72	0,96	0,98	oxidoreductase, 2OG-Fe(II) oxygenase family protein
263132_at	At1g78560	0,69	0,58	0,55	0,71	0,75	0,77	0,70	0,71	bile acid:sodium symporter family protein
263134_at	At1g78570	0,86	0,63	0,75	1,51	1,17	0,85	0,77	0,82	NAD-dependent epimerase/dehydratase family protein
263136_at	At1g78580	1,17	1,17	1,09	1,02	0,79	1,18	1,02	1,29	alpha, alpha-trehalose-phosphate synthase, UDP-forming, putative / trehalc
263128_at	At1g78600	1,13	1,39	1,83	0,69	0,65	1,02	0,96	1,13	zinc finger (B-box type) family protein
263127_at	At1g78610	0,80	0,73	1,19	0,95	0,86	1,10	0,96	1,32	mechanosensitive ion channel domain-containing protein / MS ion channel
263129_at	At1g78620	0,68	0,72	0,79	0,93	0,95	0,96	0,88	0,86	integral membrane family protein

263131_at	At1g78630	0,91	0,78	0,80	0,85	0,95	0,91	0,95	0,99	ribosomal protein L13 family protein
263130_at	At1g78650	1,04	1,03	1,17	1,07	0,98	1,12	1,01	1,01	expressed protein
263137_at	At1g78660	0,87	0,66	0,62	0,98	1,12	0,81	0,98	0,80	gamma-glutamyl hydrolase, putative / gamma-Glu-X carboxypeptidase, put
264300_at	At1g78670	0,65	0,78	0,99	0,87	0,99	0,87	0,84	0,94	gamma-glutamyl hydrolase, putative / gamma-Glu-X carboxypeptidase, put
264250_at	At1g78680	0,85	0,72	0,80	0,74	0,79	0,86	0,93	0,98	gamma-glutamyl hydrolase (GGH1) / gamma-Glu-X carboxypeptidase / cor
264298_at	At1g78690	1,09	1,44	1,43	1,00	1,07	1,06	1,08	1,10	phospholipid/glycerol acyltransferase family protein
264248_at	At1g78700	0,92	0,93	1,04	0,82	0,89	0,84	0,88	1,06	brassinosteroid signalling positive regulator-related
264297_at	At1g78710	0,93	1,03	0,98	1,00	1,03	0,91	0,90	0,90	expressed protein
264296_at	At1g78720	0,92	1,08	1,13	1,43	1,21	0,99	0,97	0,92	protein transport protein sec61, putative
264295_at	At1g78740	0,91	1,11	0,94	1,19	0,94	0,99	0,99	0,79	hypothetical protein
264294_at	At1g78750	1,00	0,93	1,13	1,09	1,00	0,91	1,12	1,07	F-box family protein
264293_at	At1g78770	0,99	0,92	1,00	1,23	1,30	0,92	1,02	0,97	cell division cycle family protein
264301_at	At1g78780	0,86	0,86	0,98	1,13	1,26	0,88	0,96	1,00	pathogenesis-related family protein
264292_at	At1g78790	0,75	0,88	0,81	1,14	0,93	0,76	0,91	0,83	expressed protein
264291_at	At1g78800	0,80	0,76	0,77	1,01	1,07	0,98	0,76	1,00	glycosyl transferase family 1 protein
264290_at	At1g78810	0,94	1,03	1,20	0,99	0,99	1,15	1,06	1,17	expressed protein
264305_at	At1g78815	1,02	1,00	1,02	0,95	0,96	0,89	0,85	0,77	expressed protein
264279_s_at	At1g78820	0,91	0,74	0,74	0,80	1,06	0,79	0,81	0,65	curculin-like (mannose-binding) lectin family protein / PAN domain-containir
257424_at	At1g78840	1,06	1,08	1,14	1,14	0,98	1,08	0,91	0,74	F-box family protein
264299_s_at	At1g78860	0,89	0,86	0,92	0,85	0,94	0,89	0,73	0,67	curculin-like (mannose-binding) lectin family protein
264303_s_at	At1g78870	1,09	0,97	0,93	1,25	1,20	1,10	1,01	0,96	ubiquitin-conjugating enzyme, putative
264306_at	At1g78890	1,21	1,62	2,09	1,16	1,31	0,99	1,06	1,10	expressed protein
264304_at	At1g78895	1,09	0,98	0,97	0,96	0,92	0,94	0,83	1,03	expressed protein
264302_at	At1g78900	0,85	0,75	0,78	0,81	0,89	0,91	0,91	0,87	vacuolar ATP synthase catalytic subunit A / V-ATPase A subunit / vacuolar
264249_at	At1g78920	1,02	1,13	0,94	0,90	0,94	0,99	1,00	0,99	vacuolar-type H+-translocating inorganic pyrophosphatase (AVPL1)
264142_at	At1g78930	1,09	1,18	1,24	0,91	0,98	0,96	0,90	1,09	mitochondrial transcription termination factor-related / mTERF-related
264139_at	At1g78940	0,88	0,92	0,92	0,96	1,09	1,05	1,00	1,08	protein kinase family protein
264138_at	At1g78950	0,92	0,99	1,08	0,91	0,90	1,16	1,02	1,05	beta-amyrin synthase, putative
264137_at	At1g78960	1,18	1,08	1,28	0,74	0,83	1,08	1,34	1,56	lupeol synthase, putative / 2,3-oxidosqualene-triterpenoid cyclase, putative
264100_at	At1g78970	1,34	1,28	1,43	0,92	0,91	1,18	1,22	1,08	lupeol synthase (LUP1) / 2,3-oxidosqualene-triterpenoid cyclase
264136_at	At1g78980	1,04	1,06	1,19	1,01	1,10	1,00	1,00	1,14	leucine-rich repeat transmembrane protein kinase, putative
257428_at	At1g78990	1,16	1,12	1,27	0,78	0,95	1,06	1,02	1,12	transferase family protein
264096_at	At1g78995	1,56	2,09	2,03	0,90	1,03	1,30	1,32	1,29	expressed protein
264101_at	At1g79000	1,21	1,26	1,46	0,69	0,70	1,05	1,10	1,18	p300/CBP acetyltransferase-related protein 2 (PCAT2)
264097_s_at	At1g79010	1,16	0,99	0,94	0,97	1,15	0,96	0,94	0,94	NADH-ubiquinone oxidoreductase 23 kDa subunit, mitochondrial, putative
264135_at	At1g79030	1,07	1,06	1,12	0,91	1,12	1,15	0,99	0,93	DNAJ heat shock N-terminal domain-containing protein / S-locus protein, pu
264092_at	At1g79040	1,04	1,00	1,02	0,99	1,07	0,98	0,98	0,97	photosystem II 10 kDa polypeptide
264099_at	At1g79050	1,21	1,10	1,12	0,82	0,90	1,11	1,03	1,00	DNA repair protein recA
257427_at	At1g79060	1,07	1,08	1,08	1,07	0,98	1,01	0,93	0,82	expressed protein
264134_at	At1g79070	0,93	0,80	1,02	0,93	1,04	0,80	0,82	0,93	SNARE-associated protein-related
264133_at	At1g79080	1,03	0,77	1,13	0,83	1,01	1,07	0,86	0,77	pentatricopeptide (PPR) repeat-containing protein
264141_at	At1g79090	0,99	1,01	1,21	0,90	0,96	0,99	0,92	0,85	expressed protein
264132_at	At1g79100	0,91	0,99	1,00	0,99	0,94	1,10	0,95	1,18	arginine/serine-rich protein-related
264091_at	At1g79110	0,87	1,13	1,04	1,01	1,03	0,87	0,98	1,13	expressed protein
264118_at	At1g79140	1,51	1,24	1,14	1,14	1,28	1,06	1,06	1,36	expressed protein
264131_at	At1g79150	1,22	1,13	1,04	1,08	1,27	1,09	1,07	1,39	expressed protein

264130_at	At1g79160	1,04	1,11	0,97	1,11	1,27	0,94	0,92	1,29	expressed protein
264129_at	At1g79170	0,98	0,86	0,77	0,93	0,96	1,09	1,08	1,29	hypothetical protein
264119_at	At1g79180	0,91	1,10	1,02	1,11	1,01	0,93	1,02	1,03	myb family transcription factor (MYB63)
264128_at	At1g79190	1,08	1,05	0,93	1,03	0,74	1,00	0,92	0,90	expressed protein
264094_at	At1g79200	1,08	1,07	1,08	1,07	1,21	1,00	1,29	1,26	expressed protein
264140_at	At1g79210	0,91	0,85	0,83	1,22	1,15	0,91	0,81	0,83	20S proteasome alpha subunit B, putative
264093_at	At1g79220	0,92	1,12	0,81	1,13	1,02	0,79	0,87	1,02	mitochondrial transcription termination factor family protein / mTERF family
264095_at	At1g79230	1,13	1,06	1,02	1,01	1,07	1,10	0,95	0,99	mercaptopyruvate sulfurtransferase (MST1) (RDH1)
264127_at	At1g79250	0,97	0,86	0,95	0,99	1,24	1,03	1,01	0,98	protein kinase, putative
264098_at	At1g79260	1,15	0,90	0,92	1,03	1,06	1,01	0,88	0,80	expressed protein
264102_at	At1g79270	1,48	1,21	1,70	1,06	0,94	1,44	1,98	2,11	expressed protein
264126_at	At1g79280	1,16	1,05	1,28	0,86	0,75	1,26	1,26	1,59	expressed protein
264145_at	At1g79310	0,87	0,87	1,06	0,82	0,72	1,04	2,38	2,30	latex-abundant protein, putative (AMC4) / caspase family protein
264144_at	At1g79320	1,07	1,00	1,02	1,03	0,93	1,01	1,12	1,01	latex abundant protein, putative (AMC5) / caspase family protein
264143_at	At1g79330	0,95	1,01	1,04	0,91	0,96	1,04	1,00	1,19	latex-abundant protein, putative (AMC6) / caspase family protein
264120_at	At1g79340	0,99	0,86	0,96	1,21	1,17	0,96	0,79	0,93	latex-abundant protein, putative (AMC7) / caspase family protein
264125_at	At1g79350	1,05	1,01	1,05	0,78	0,89	1,22	1,24	1,32	DNA-binding protein, putative
264124_at	At1g79360	1,78	1,51	1,20	0,86	0,83	0,97	0,92	1,10	transporter-related
264090_at	At1g79370	0,90	1,22	0,96	0,96	1,07	1,04	0,95	0,98	cytochrome P450 family protein
262919_at	At1g79380	0,63	0,67	0,79	0,91	0,89	0,98	1,23	1,53	copine-related
262946_at	At1g79390	1,66	1,44	1,21	1,29	1,08	0,93	1,00	0,89	expressed protein
262936_at	At1g79400	0,89	0,98	1,00	1,07	1,05	0,94	0,91	0,98	cation/proton exchanger, putative (CHX2)
262935_at	At1g79410	0,66	0,88	0,80	0,60	0,88	0,98	1,16	0,85	transporter-related
262922_at	At1g79420	1,04	1,09	1,17	1,16	1,00	1,03	0,90	1,30	expressed protein
262921_at	At1g79430	1,03	1,18	0,95	1,03	1,17	1,10	1,06	1,05	myb family transcription factor-related
262892_at	At1g79440	0,53	0,59	0,59	0,77	0,82	0,76	0,81	0,81	succinate-semialdehyde dehydrogenase (SSADH1)
262942_at	At1g79450	0,93	0,94	0,50	0,96	0,98	1,21	1,06	0,84	LEM3 (ligand-effect modulator 3) family protein / CDC50 family protein
262891_at	At1g79460	0,79	0,95	0,82	1,17	0,88	0,87	0,92	0,97	ent-kaurene synthase / ent-kaurene synthetase B (KS) (GA2)
262943_at	At1g79470	1,24	1,23	1,17	1,08	1,35	1,05	0,95	0,99	inosine-5'-monophosphate dehydrogenase
262941_at	At1g79490	1,12	1,09	1,14	1,10	1,01	0,94	0,82	0,88	pentatricopeptide (PPR) repeat-containing protein
262920_at	At1g79500	0,82	0,78	0,80	1,01	1,19	0,80	0,85	0,74	2-dehydro-3-deoxyphosphooctonate aldolase / phospho-2-dehydro-3-deox
262945_at	At1g79510	0,92	0,95	1,15	0,78	0,78	1,36	1,23	1,30	expressed protein
262940_at	At1g79520	0,97	1,05	0,83	1,02	0,90	1,36	1,81	1,94	cation efflux family protein
262939_s_at	At1g79530	1,13	1,16	0,83	1,19	1,13	1,10	1,12	0,99	glyceraldehyde 3-phosphate dehydrogenase, cytosolic, putative / NAD-dep
262938_at	At1g79540	0,72	0,73	0,86	0,87	1,05	0,97	0,82	1,01	pentatricopeptide (PPR) repeat-containing protein
262944_at	At1g79550	1,47	1,49	1,26	1,87	1,90	1,02	1,02	1,04	phosphoglycerate kinase, putative
262937_at	At1g79560	0,96	0,85	0,77	0,77	0,71	1,04	1,03	0,99	FtsH protease, putative
262934_s_at	At1g79570	1,10	0,95	0,96	1,30	1,22	0,96	1,03	1,02	protein kinase family protein
261393_at	At1g79580	1,09	1,09	1,03	0,97	1,06	0,95	1,00	1,15	no apical meristem (NAM) family protein
261403_at	At1g79590	1,09	0,94	1,00	1,07	1,18	0,97	0,86	0,89	syntaxin 52 (SYP52)
261353_at	At1g79600	0,81	0,83	0,89	0,80	0,75	1,11	0,93	0,94	ABC1 family protein
261398_at	At1g79610	1,15	0,87	0,76	1,06	0,95	1,12	1,41	1,41	sodium proton exchanger, putative (NHX6)
261349_at	At1g79615	0,98	0,87	0,97	1,16	1,06	0,98	1,02	0,96	sodium proton exchanger, putative (NHX6)
261399_at	At1g79620	1,21	1,37	1,25	1,20	1,03	1,03	1,04	1,11	leucine-rich repeat transmembrane protein kinase, putative
261400_at	At1g79630	1,08	0,98	0,99	0,81	0,98	1,09	0,95	1,01	protein phosphatase 2C family protein / PP2C family protein
261401_at	At1g79640	0,95	0,93	1,11	1,10	1,03	0,82	0,87	1,09	protein kinase family protein

261352_at	At1g79650	0,95	0,83	0,86	0,98	1,20	0,93	1,03	1,02	DNA repair protein RAD23, putative
261356_at	At1g79660	1,06	0,99	1,35	1,63	1,25	1,00	1,03	1,13	expressed protein
261402_at	At1g79670	0,85	0,95	0,99	0,65	0,68	0,89	0,85	0,96	wall-associated kinase, putative
261394_at	At1g79680	0,70	0,64	0,70	1,18	1,08	0,90	0,81	1,20	wall-associated kinase, putative
261354_at	At1g79690	0,85	0,83	0,80	1,02	1,04	0,80	0,82	0,84	MutT/nudix family protein
261395_at	At1g79700	1,75	2,84	3,54	0,92	0,76	1,18	1,28	1,87	ovule development protein, putative
261344_at	At1g79710	0,96	1,01	1,09	0,95	1,11	0,90	1,01	1,21	integral membrane transporter family protein
261346_at	At1g79720	1,14	1,19	1,37	1,45	1,13	0,90	0,71	0,60	aspartyl protease family protein
261347_at	At1g79730	1,01	1,00	1,30	0,98	0,86	1,27	1,02	1,30	hydroxyproline-rich glycoprotein family protein
261355_at	At1g79750	0,90	0,83	0,75	0,67	0,62	0,92	0,91	0,90	malate oxidoreductase, putative
261345_at	At1g79760	0,97	0,97	0,99	0,96	1,11	0,97	1,00	1,16	expressed protein
261350_at	At1g79770	1,39	1,63	1,30	0,82	1,03	1,36	1,61	1,18	expressed protein
261392_at	At1g79780	0,96	1,01	0,98	0,87	1,01	1,07	0,86	0,87	integral membrane protein, putative
261351_at	At1g79790	0,48	0,41	0,41	0,80	1,05	0,70	0,68	0,66	haloacid dehalogenase-like hydrolase family protein
261396_at	At1g79800	0,98	0,99	1,11	1,01	1,13	1,04	1,11	1,04	plastocyanin-like domain-containing protein
261348_at	At1g79810	1,01	1,12	1,10	0,81	0,89	0,92	1,06	1,09	Pex2/Pex12 N-terminal domain-containing protein / zinc finger (C3HC4-type
261397_at	At1g79820	1,02	0,87	0,97	1,39	1,37	0,92	0,98	1,08	hexose transporter, putative
260162_at	At1g79830	0,84	0,89	0,87	0,79	0,87	0,93	0,93	1,01	expressed protein
260166_at	At1g79840	0,96	1,03	1,09	0,78	0,87	0,99	0,95	1,29	homeobox-leucine zipper protein 10 (HB-10) / HD-ZIP transcription factor 1
260165_at	At1g79850	1,09	1,12	1,06	1,15	1,23	1,00	0,89	0,84	30S ribosomal protein S17, chloroplast / CS17 (RPS17)
260161_at	At1g79860	0,89	0,99	1,01	1,03	1,22	0,99	0,97	1,03	expressed protein
260164_at	At1g79870	1,02	0,99	1,22	0,99	1,04	1,02	0,88	1,02	oxidoreductase family protein
260160_at	At1g79880	1,19	1,23	1,36	1,01	1,04	1,10	1,15	1,30	La domain-containing protein
260159_at	At1g79890	0,96	0,99	1,01	0,92	1,00	1,08	1,15	1,14	helicase-related
260163_at	At1g79900	0,94	1,07	1,04	0,96	1,01	1,02	1,01	0,92	mitochondrial substrate carrier family protein
260158_at	At1g79910	0,95	0,96	1,02	0,93	0,91	0,84	1,11	0,99	expressed protein
262054_s_at	At1g79920	1,08	0,94	1,03	1,12	1,34	0,97	0,96	0,95	heat shock protein 70, putative / HSP70, putative
262053_at	At1g79940	0,93	0,80	0,80	0,95	1,09	0,91	0,88	0,95	DNAJ heat shock N-terminal domain-containing protein / sec63 domain-cor
262066_at	At1g79950	1,24	1,28	1,54	0,87	0,68	1,09	1,31	1,62	helicase-related
262046_at	At1g79960	0,81	1,06	0,95	0,89	1,05	1,01	0,93	0,87	ovate protein-related
262055_at	At1g79970	0,55	0,73	0,88	0,91	0,94	0,82	0,83	0,74	expressed protein
262058_at	At1g79975	0,93	0,86	1,00	1,00	0,99	1,00	1,00	1,13	expressed protein
262051_at	At1g79990	0,92	0,76	0,68	1,15	0,98	0,87	0,91	0,95	coatomer protein complex, subunit beta 2 (beta prime), putative
262063_at	At1g80000	0,78	0,83	0,93	0,95	0,94	0,90	0,95	0,86	expressed protein
262070_at	At1g80010	0,89	0,96	1,14	0,99	1,01	1,14	1,00	0,99	far-red impaired responsive protein, putative
262052_at	At1g80020	0,79	0,74	0,78	0,85	0,95	1,06	0,94	0,94	---
262059_at	At1g80030	0,81	0,72	0,75	0,81	0,93	1,13	1,01	1,05	DNAJ heat shock protein, putative
262057_at	At1g80040	0,98	0,84	1,13	1,28	1,18	1,02	1,03	1,25	expressed protein
262039_at	At1g80050	1,44	1,20	1,37	1,15	1,20	0,92	0,81	0,99	adenine phosphoribosyltransferase 2 (APT2)
262067_at	At1g80060	1,13	1,02	0,90	0,86	1,08	0,84	0,84	0,85	expressed protein
262068_at	At1g80070	1,18	1,17	1,17	1,06	0,89	1,11	1,07	1,13	splicing factor, putative
262040_at	At1g80080	0,97	1,03	1,03	1,09	0,99	0,75	0,90	0,92	leucine-rich repeat family protein
262069_at	At1g80090	0,96	1,03	1,01	0,74	0,84	0,96	0,95	0,93	CBS domain-containing protein
262041_at	At1g80100	0,95	0,98	1,19	1,03	0,91	1,10	1,00	0,93	phosphotransfer family protein
262061_at	At1g80110	0,85	1,13	1,43	1,18	1,19	0,84	0,94	0,92	expressed protein
262050_at	At1g80130	0,66	0,65	0,68	0,63	0,77	1,16	0,96	0,77	expressed protein

262042_at	At1g80140	0,90	0,90	1,04	0,98	1,10	0,94	1,01	0,97	glycoside hydrolase family 28 protein / polygalacturonase (pectinase) family
262047_at	At1g80160	0,68	0,95	0,94	0,98	0,81	1,09	0,98	1,04	lactoylglutathione lyase family protein / glyoxalase I family protein
262060_at	At1g80170	0,93	0,95	0,83	1,10	1,12	0,98	1,29	1,01	polygalacturonase, putative / pectinase, putative
262049_at	At1g80180	1,29	1,16	1,21	0,93	0,82	1,12	0,98	1,20	expressed protein
262043_at	At1g80190	1,18	1,26	1,44	0,95	1,27	1,04	1,07	1,08	expressed protein
262044_s_at	At1g80210	0,86	0,92	1,00	0,81	0,97	1,06	1,09	1,02	expressed protein
262048_at	At1g80230	1,09	1,02	0,98	1,11	1,04	0,87	0,95	0,93	cytochrome c oxidase family protein
262045_at	At1g80240	0,98	0,97	1,20	2,95	2,74	0,97	0,99	0,83	expressed protein
262056_at	At1g80245	1,28	0,93	1,15	0,95	0,94	0,94	0,94	0,94	expressed protein
262062_s_at	At1g80260	1,11	0,94	1,04	1,03	0,93	1,06	1,12	0,98	tubulin family protein
260331_at	At1g80270	1,79	1,63	1,48	1,32	1,35	1,03	1,07	0,93	DNA-binding protein, putative
260297_at	At1g80280	1,55	1,18	0,69	0,90	0,81	1,07	0,72	0,71	hydrolase, alpha/beta fold family protein
260301_at	At1g80290	0,81	0,72	0,72	1,03	1,34	0,96	0,78	0,78	glycosyltransferase family protein 47
260302_at	At1g80310	0,66	0,76	0,84	0,64	0,62	0,72	0,86	0,95	expressed protein
260298_at	At1g80320	0,78	1,04	0,96	0,82	0,97	0,87	0,81	0,92	oxidoreductase, 2OG-Fe(II) oxygenase family protein
260299_at	At1g80330	1,02	1,05	1,05	0,97	1,13	1,10	0,88	0,94	gibberellin 3-beta-dioxygenase, putative / gibberellin 3 beta-hydroxylase, pu
260300_at	At1g80340	0,89	1,03	0,90	0,97	1,33	1,05	0,86	1,13	gibberellin 3-beta-dioxygenase / gibberellin 3 beta-hydroxylase (GA4H)
260289_at	At1g80350	1,07	1,03	1,09	1,05	1,01	1,06	0,91	0,84	katanin 1 (KTN1)
260328_at	At1g80360	0,82	0,83	0,79	0,92	0,85	0,77	0,80	0,74	aminotransferase class I and II family protein
260329_at	At1g80370	1,23	1,22	1,06	1,21	1,51	0,92	0,87	1,12	cyclin, putative
260284_at	At1g80380	0,87	0,89	0,85	0,67	0,69	0,98	1,05	1,05	phosphoribulokinase/uridine kinase-related
260330_at	At1g80400	0,96	0,94	0,96	0,76	0,87	0,90	1,00	0,94	zinc finger (C3HC4-type RING finger) family protein
260282_at	At1g80410	1,34	1,30	1,30	1,16	1,14	0,98	0,98	1,00	acetyltransferase-related
260279_at	At1g80420	0,76	0,87	1,00	0,85	0,96	0,82	0,96	0,97	DNA repair protein, putative (XRCC1)
260287_at	At1g80440	0,96	1,23	1,06	0,64	0,63	0,83	1,22	1,37	kelch repeat-containing F-box family protein
260276_at	At1g80450	0,62	0,94	0,85	0,96	1,06	0,93	0,92	1,16	VQ motif-containing protein
260274_at	At1g80460	1,05	1,13	1,18	1,13	1,24	1,02	0,95	0,84	glycerol kinase, putative
260283_at	At1g80480	0,74	0,68	0,79	0,65	0,76	1,07	1,03	1,09	PRLI-interacting factor L, putative
260281_at	At1g80500	1,24	0,99	0,94	1,23	1,06	0,98	1,02	1,02	expressed protein
260290_at	At1g80510	0,90	0,85	0,81	1,00	0,93	0,94	0,90	0,90	amino acid transporter family protein
260277_at	At1g80520	1,01	1,13	0,99	1,13	1,20	0,99	0,94	1,16	expressed protein
260288_at	At1g80530	0,90	0,76	0,73	0,93	1,08	0,86	0,84	0,77	nodulin family protein
260273_at	At1g80550	0,84	0,97	1,00	0,81	0,70	1,06	1,07	1,40	pentatricopeptide (PPR) repeat-containing protein
260285_at	At1g80560	0,84	0,80	0,70	0,74	0,76	0,76	0,83	0,84	3-isopropylmalate dehydrogenase, chloroplast, putative
260272_at	At1g80570	0,98	0,93	1,29	1,03	0,93	0,99	0,99	1,05	F-box family protein (FBL14)
260280_at	At1g80580	1,01	1,01	0,87	0,88	1,04	1,12	0,96	1,05	ethylene-responsive element-binding family protein
260278_at	At1g80590	0,88	0,95	1,13	0,93	1,23	1,00	0,80	1,24	WRKY family transcription factor
260286_at	At1g80600	1,10	1,04	0,97	0,90	0,88	0,93	0,93	0,96	acetylornithine aminotransferase, mitochondrial, putative / acetylornithine tr
260275_at	At1g80610	0,93	1,00	1,15	0,92	0,92	1,06	1,08	1,23	expressed protein
259861_at	At1g80615	1,07	0,94	0,99	1,09	1,38	1,04	0,91	0,89	ribosomal protein S15 family protein
259830_at	At1g80630	0,78	0,70	0,76	0,77	1,15	0,87	1,19	1,12	leucine-rich repeat family protein
259860_at	At1g80640	0,99	1,00	0,86	0,87	0,83	1,02	1,11	0,98	protein kinase family protein
261943_at	At1g80660	0,94	1,08	1,20	1,01	1,10	1,06	1,13	1,02	ATPase 9, plasma membrane-type, putative / proton pump 9, putative / prot
261896_at	At1g80670	1,28	1,10	1,16	0,96	1,01	0,87	0,86	0,99	transducin family protein / WD-40 repeat family protein
261909_at	At1g80680	1,14	1,16	1,30	0,86	0,86	1,01	0,99	0,91	nucleoporin family protein
261893_at	At1g80690	1,17	1,12	0,75	1,10	1,12	0,88	0,99	1,04	expressed protein

261886_s_at	At1g80700	0,93	0,93	0,98	1,04	1,05	0,97	1,31	1,33	expressed protein
261898_at	At1g80720	1,53	1,30	1,42	1,22	1,26	0,98	0,76	0,73	mitochondrial glycoprotein family protein / MAM33 family protein
261910_at	At1g80730	0,91	1,07	1,04	1,01	1,07	0,95	0,96	1,02	zinc finger (C2H2 type) family protein (ZFP1)
261903_at	At1g80740	0,91	0,98	1,12	0,90	1,18	0,88	1,00	1,08	chromomethylase 1 (CMT1)
261911_at	At1g80750	1,46	1,34	1,26	0,88	0,92	1,07	1,11	1,04	60S ribosomal protein L7 (RPL7A)
261881_at	At1g80760	1,43	1,12	1,11	1,32	1,19	0,78	0,85	0,78	major intrinsic family protein / MIP family protein
261882_at	At1g80770	1,07	0,89	0,98	0,76	0,78	1,02	0,95	1,06	expressed protein
261887_at	At1g80780	0,89	0,91	0,92	1,01	0,95	0,87	0,92	1,06	CCR4-NOT transcription complex protein, putative
261891_at	At1g80790	1,00	1,04	1,18	0,89	0,94	1,03	1,26	1,58	XH/XS domain-containing protein / XS zinc finger domain-containing protei
261888_at	At1g80800	1,16	1,08	1,13	0,91	0,86	1,17	1,44	1,28	pseudogene, 40S ribosomal protein S12 (RPS12B)
261889_at	At1g80810	1,06	1,08	1,07	0,93	0,88	1,14	1,23	1,26	expressed protein
261899_at	At1g80820	0,99	1,18	1,03	1,52	1,65	1,29	1,53	1,48	cinnamoyl-CoA reductase, putative
261895_at	At1g80830	1,10	1,50	1,39	0,78	0,77	0,99	1,24	1,08	NRAMP metal ion transporter 1 (NRAMP1)
261892_at	At1g80840	1,06	4,38	2,35	0,89	0,55	1,37	2,66	2,10	WRKY family transcription factor
257474_at	At1g80850	1,45	1,52	1,52	0,80	0,93	1,21	1,01	1,10	methyladenine glycosylase family protein
261902_at	At1g80860	0,97	0,98	0,91	0,93	0,97	0,90	0,95	0,85	expressed protein
261883_at	At1g80870	0,86	0,80	0,86	0,79	1,03	1,01	1,00	1,10	protein kinase family protein
257475_at	At1g80880	0,96	1,14	1,15	0,76	0,94	0,90	0,94	1,11	pentatricopeptide (PPR) repeat-containing protein
261894_at	At1g80900	1,03	1,15	1,44	1,01	0,90	1,16	1,20	1,26	magnesium transporter CorA-like family protein (MGT1) (MRS2)
261884_at	At1g80910	1,13	0,84	1,08	1,13	1,35	0,97	0,85	0,89	expressed protein
261901_at	At1g80920	1,61	1,96	1,61	0,78	0,90	1,10	1,28	1,38	DNAJ heat shock N-terminal domain-containing protein
261885_at	At1g80930	1,10	1,03	1,10	0,93	0,86	0,99	0,94	1,02	MIF4G domain-containing protein / MA3 domain-containing protein
261900_at	At1g80940	1,21	1,35	1,33	1,45	1,83	1,22	1,12	1,20	expressed protein
261897_at	At1g80950	1,12	1,06	1,08	1,06	0,87	1,08	0,87	1,03	phospholipid/glycerol acyltransferase family protein
257476_at	At1g80960	0,96	0,86	0,92	1,14	0,91	1,43	1,19	1,17	F-box protein-related
261890_at	At1g80970	0,84	0,96	1,14	0,91	0,98	1,01	0,98	0,88	XH domain-containing protein
262203_at	At2g01060	0,93	1,02	1,46	0,98	1,10	0,98	1,07	1,21	myb family transcription factor
262200_at	At2g01070	1,19	1,19	0,96	1,04	0,93	0,95	0,91	0,97	expressed protein
262205_at	At2g01080	0,85	0,84	0,94	1,08	1,46	0,82	0,75	0,79	expressed protein
262206_at	At2g01090	1,44	1,17	1,03	1,06	0,98	1,00	1,03	1,04	ubiquinol-cytochrome C reductase complex 7.8 kDa protein, putative / mitoc
262204_at	At2g01100	0,78	0,92	1,09	0,88	1,14	1,15	1,25	1,46	expressed protein
262202_at	At2g01110	0,89	0,89	1,02	0,76	0,67	1,15	1,05	1,12	thylakoid membrane formation protein / cpTatC (APG2)
262201_at	At2g01120	0,97	0,87	0,60	0,97	0,91	0,84	0,80	0,71	origin recognition complex subunit 4, putative (ORC4)
265731_at	At2g01130	1,11	1,10	1,13	1,00	1,07	1,12	1,18	1,01	helicase domain-containing protein
265735_at	At2g01140	1,12	0,96	0,93	1,04	1,05	0,97	0,97	1,00	fructose-bisphosphate aldolase, putative
265740_at	At2g01150	0,91	1,10	0,92	1,48	2,02	0,90	0,92	1,11	zinc finger (C3HC4-type RING finger) family protein
265790_at	At2g01170	0,59	0,68	0,61	0,74	0,78	0,82	0,91	1,01	amino acid permease family protein
265737_at	At2g01180	0,66	0,70	0,81	0,84	0,79	1,02	1,27	1,39	phosphatidic acid phosphatase family protein / PAP2 family protein
265733_at	At2g01190	0,71	0,73	0,68	1,03	0,88	0,86	0,80	0,93	octicosapeptide/Phox/Bem1p (PB1) domain-containing protein
265788_at	At2g01200	0,88	0,92	1,07	1,01	0,63	1,01	0,87	0,97	auxin-responsive AUX/IAA family protein
265789_at	At2g01210	0,92	0,86	1,12	1,07	0,88	0,82	0,84	0,83	leucine-rich repeat transmembrane protein kinase, putative
265793_at	At2g01220	0,94	0,79	0,84	0,93	1,01	0,91	0,95	0,97	expressed protein
265760_at	At2g01230	0,84	0,81	0,75	0,77	0,78	0,93	1,08	0,93	expressed protein
265762_at	At2g01240	0,96	1,12	1,16	0,97	1,05	0,88	1,08	1,11	reticulon family protein (RTNLB15)
265736_at	At2g01250	1,33	1,19	1,02	1,21	1,23	1,03	0,94	1,06	60S ribosomal protein L7 (RPL7B)
265734_at	At2g01260	0,93	1,00	0,99	0,92	0,92	1,04	0,80	0,76	expressed protein

265739_at	At2g01270	0,98	0,90	0,98	0,88	0,81	0,94	1,02	1,06	thioredoxin family protein
265791_at	At2g01280	0,92	1,08	0,98	0,96	1,15	1,05	0,92	1,18	transcription factor IIB (TFIIB) family protein
265742_at	At2g01290	0,64	0,57	0,60	0,90	0,77	0,94	0,86	0,94	expressed protein
265732_at	At2g01300	0,59	0,66	0,60	1,10	0,93	0,95	1,07	1,25	expressed protein
265794_at	At2g01310	0,91	0,88	0,91	0,96	1,00	1,10	1,07	1,07	expressed protein
265741_at	At2g01320	0,88	0,98	0,97	0,55	0,52	1,21	1,35	1,41	ABC transporter family protein
265761_at	At2g01330	1,11	1,17	1,16	1,05	0,97	0,97	1,06	0,97	transducin family protein / WD-40 repeat family protein
265738_at	At2g01350	1,00	0,89	0,88	0,93	0,88	1,05	1,10	1,28	quinolinate phosphoribosyl transferase family protein
265792_at	At2g01380	0,87	1,02	0,92	0,84	0,93	0,92	1,09	1,13	pentatricopeptide (PPR) repeat-containing protein
266339_s_at	At2g01390	0,98	0,99	1,05	0,85	0,93	1,02	0,92	0,89	pentatricopeptide (PPR) repeat-containing protein
266355_at	At2g01400	1,29	1,17	1,30	1,18	1,33	1,15	1,21	1,18	expressed protein
266345_at	At2g01410	0,77	0,94	0,98	1,28	1,54	0,85	0,88	0,97	hypothetical protein
266300_at	At2g01420	1,11	2,07	1,84	0,88	0,65	1,38	1,18	1,60	auxin transport protein, putative
266346_at	At2g01430	1,00	1,04	1,02	0,95	1,11	1,17	1,00	1,10	homeobox-leucine zipper protein 17 (HB-17) / HD-ZIP transcription factor 1
266347_at	At2g01440	0,85	0,89	0,86	0,91	0,96	0,96	0,92	0,94	ATP-dependent DNA helicase, putative
266348_at	At2g01450	0,77	0,71	0,85	0,89	1,03	0,93	1,11	1,22	mitogen-activated protein kinase, putative / MAPK, putative (MPK17)
266349_at	At2g01460	1,44	1,55	1,64	0,79	0,83	1,08	1,53	1,32	phosphoribulokinase/uridine kinase family protein
266350_at	At2g01470	1,01	0,88	0,94	0,92	0,90	1,11	1,06	0,90	St12p protein (ST12p) / SEC12p protein, putative
266340_at	At2g01480	1,02	0,95	0,98	0,95	0,95	1,26	1,17	1,48	expressed protein
266351_at	At2g01490	0,70	0,72	0,76	0,90	1,06	0,87	0,87	0,89	phytanoyl-CoA dioxygenase (PhyH) family protein
266354_at	At2g01500	1,03	1,00	1,11	0,91	1,07	0,98	0,99	1,13	homeobox-leucine zipper transcription factor family protein
266341_at	At2g01510	0,97	1,01	0,91	0,84	0,81	1,03	0,96	0,89	pentatricopeptide (PPR) repeat-containing protein
266353_at	At2g01520	0,56	0,59	0,62	0,92	0,73	0,54	0,79	0,79	major latex protein-related / MLP-related
266330_at	At2g01530	0,67	0,68	0,76	1,39	1,23	0,70	0,91	1,05	major latex protein-related / MLP-related
266342_at	At2g01540	0,62	0,57	0,53	1,25	1,26	0,90	0,80	0,83	C2 domain-containing protein
266343_at	At2g01550	1,09	0,95	0,96	0,94	1,14	0,91	1,08	0,86	---
266331_at	At2g01570	0,85	0,78	0,73	0,71	0,72	0,74	0,91	0,87	gibberellin response modulator (RGA1) / gibberellin-responsive modulator
266344_at	At2g01580	0,83	1,00	1,02	0,91	1,02	1,13	1,04	1,04	expressed protein
266329_at	At2g01590	1,00	0,91	0,91	0,87	0,78	1,16	1,01	1,07	expressed protein
266328_at	At2g01600	1,02	0,94	1,02	1,08	1,06	0,93	0,93	1,02	epsin N-terminal homology (ENTH) domain-containing protein
266352_at	At2g01610	0,83	0,81	0,81	1,18	1,05	0,98	0,94	0,90	invertase/pectin methylesterase inhibitor family protein
265867_at	At2g01620	0,87	1,44	1,40	0,86	0,73	0,98	1,02	1,14	expressed protein
265873_at	At2g01630	1,00	1,24	1,03	1,08	1,16	0,94	0,84	0,81	glycosyl hydrolase family 17 protein / beta-1,3-glucanase, putative
265874_at	At2g01640	1,27	1,06	1,01	1,04	1,00	1,07	0,96	0,92	expressed protein
265868_at	At2g01650	0,80	0,75	0,87	0,96	1,00	1,02	0,89	0,96	zinc finger (C2H2 type) family protein
265870_at	At2g01660	1,25	0,98	0,95	1,17	1,10	0,94	1,02	0,89	33 kDa secretory protein-related
265872_at	At2g01670	1,30	2,50	1,66	0,85	0,72	1,72	2,43	1,76	MutT/nudix family protein
265871_at	At2g01680	0,88	1,02	0,78	0,93	0,85	0,97	1,09	0,92	ankyrin repeat family protein
265875_at	At2g01690	1,24	1,16	1,70	1,21	1,37	1,12	1,05	1,21	expressed protein
265859_at	At2g01700	1,01	1,06	1,00	0,97	1,01	0,95	1,05	0,85	hypothetical protein
265866_at	At2g01710	0,99	0,91	0,89	1,36	1,06	0,82	0,90	0,83	DNAJ heat shock N-terminal domain-containing protein
265858_at	At2g01720	1,09	1,01	0,95	1,22	0,99	1,12	1,01	0,98	ribophorin I family protein
265888_at	At2g01730	0,82	0,85	0,99	1,08	0,87	0,91	0,94	0,99	zinc finger (C3HC4-type RING finger) family protein
265865_at	At2g01740	1,06	1,03	1,02	0,97	0,89	0,86	1,09	0,91	pentatricopeptide (PPR) repeat-containing protein
265864_at	At2g01750	0,94	1,11	1,07	0,99	0,89	0,99	1,04	1,05	expressed protein
265869_at	At2g01760	1,05	0,99	0,89	0,88	1,18	0,94	0,79	1,04	two-component responsive regulator family protein / response regulator fan

265863_at	At2g01770	1,04	1,09	1,08	1,12	1,10	1,11	0,98	0,89	integral membrane family protein
265862_at	At2g01780	0,93	0,99	1,02	1,12	0,95	1,13	1,07	0,80	S-locus glycoprotein, putative
265861_at	At2g01790	1,02	0,97	1,02	1,05	0,94	0,98	0,88	0,98	meprin and TRAF homology domain-containing protein / MATH domain-cor
257385_at	At2g01800	0,98	1,06	1,02	0,94	1,04	1,02	1,14	0,97	COP1-interacting protein-related
265860_at	At2g01810	1,10	1,11	0,94	0,91	1,09	0,89	1,00	0,91	PHD finger family protein
263590_at	At2g01820	1,06	1,13	0,96	1,00	1,00	0,94	0,80	0,86	leucine-rich repeat protein kinase, putative
263599_at	At2g01830	1,16	1,03	1,24	1,02	1,01	1,07	1,28	1,10	histidine kinase (AHK4) (WOL)
263592_at	At2g01840	1,04	1,03	0,99	0,94	1,06	0,85	0,98	1,03	---
263598_at	At2g01850	1,03	0,98	1,21	1,07	1,20	1,06	1,08	1,30	xyloglucan:xyloglucosyl transferase / xyloglucan endotransglycosylase / en
263593_at	At2g01860	2,03	1,37	1,10	0,69	0,49	1,33	1,26	1,09	pentatricopeptide (PPR) repeat-containing protein
263597_at	At2g01870	0,75	0,82	1,06	0,84	0,66	1,09	0,90	1,12	expressed protein
263594_at	At2g01880	0,96	0,80	0,76	1,37	1,52	0,95	1,62	1,55	purple acid phosphatase (PAP7)
263595_at	At2g01890	1,45	1,27	0,99	0,90	0,79	2,44	2,84	2,64	purple acid phosphatase, putative
263596_at	At2g01900	0,94	1,04	1,02	0,95	1,10	1,14	0,98	1,02	endonuclease/exonuclease/phosphatase family protein
263591_at	At2g01910	0,86	0,83	1,09	0,95	1,27	0,95	1,07	1,27	microtubule associated protein (MAP65/ASE1) family protein
263304_at	At2g01920	1,01	0,95	0,91	0,94	0,99	1,15	1,07	1,03	epsin N-terminal homology (ENTH) domain-containing protein / clathrin ass
263305_at	At2g01930	1,01	0,72	1,08	0,85	1,00	0,97	0,98	1,51	expressed protein
265249_at	At2g01940	1,00	1,12	0,98	0,84	1,04	1,05	0,90	1,37	zinc finger (C2H2 type) family protein
265250_at	At2g01950	0,92	0,91	0,76	0,95	0,90	0,78	0,78	1,05	leucine-rich repeat transmembrane protein kinase, putative
265251_at	At2g01960	1,05	1,20	1,00	1,04	1,02	0,89	0,91	0,88	expressed protein
265222_at	At2g01970	1,03	0,92	0,84	1,13	0,96	0,92	0,83	0,87	endomembrane protein 70, putative
265252_at	At2g01980	0,92	1,04	1,12	0,94	1,12	1,19	1,13	0,96	sodium proton exchanger, putative (NHX7) (SOS1)
257398_at	At2g01990	1,54	1,35	1,09	0,80	1,28	1,00	0,89	1,07	expressed protein
265221_s_at	At2g02010	1,46	1,00	1,20	1,11	1,15	1,28	1,15	1,09	glutamate decarboxylase, putative
265253_at	At2g02020	1,29	1,14	1,32	0,85	0,81	0,87	1,06	1,03	proton-dependent oligopeptide transport (POT) family protein
265220_at	At2g02040	0,85	0,91	0,98	1,09	1,10	0,84	0,82	0,86	peptide transporter (PTR2-B) / oligopeptide transporter 1-1, putative (OPT1
265219_at	At2g02050	1,58	1,17	1,09	1,29	1,01	1,09	0,99	1,07	NADH-ubiquinone oxidoreductase B18 subunit, putative
266111_at	At2g02060	0,91	1,00	0,94	1,01	0,96	1,07	0,91	0,76	calcium-dependent protein kinase-related / CDPK-related
266120_at	At2g02070	1,05	0,99	1,12	0,81	0,83	0,90	0,92	1,04	zinc finger (C2H2 type) family protein
266110_at	At2g02080	0,81	0,85	1,01	0,72	0,72	1,01	0,95	1,24	zinc finger (C2H2 type) family protein
266122_at	At2g02090	0,91	0,86	1,25	1,17	1,09	1,02	1,10	1,38	SNF2 domain-containing protein / helicase domain-containing protein
266119_at	At2g02100	1,14	1,06	1,00	1,46	1,38	0,86	0,83	0,86	plant defensin-fusion protein, putative (PDF2.2)
266141_at	At2g02120	1,66	1,64	0,74	5,69	4,64	0,76	0,81	1,02	plant defensin-fusion protein, putative (PDF2.1)
266118_at	At2g02130	1,81	1,44	1,23	1,49	1,26	0,80	0,83	0,85	plant defensin-fusion protein, putative (PDF2.3)
266115_at	At2g02140	0,85	1,05	1,02	1,04	0,92	1,17	0,99	1,09	plant defensin-fusion protein, putative (PDF2.6)
266114_at	At2g02150	0,92	1,01	0,93	0,86	1,09	1,01	1,19	1,10	pentatricopeptide (PPR) repeat-containing protein
266121_at	At2g02160	1,07	1,02	1,16	0,91	1,12	1,04	1,05	1,41	zinc finger (CCCH-type) family protein
266117_at	At2g02170	0,90	0,90	0,91	0,91	0,88	0,92	1,06	1,28	remorin family protein
266116_at	At2g02180	0,89	0,79	0,81	1,09	1,01	0,86	0,87	0,87	tobamovirus multiplication protein 3 (TOM3)
266113_x_at	At2g02200	0,97	1,08	1,06	1,00	1,12	1,04	1,14	1,04	pseudogene, hypothetical protein
266112_x_at	At2g02210	0,97	0,96	1,10	1,00	0,97	1,00	1,05	0,93	pseudogene, Ulp1 protease family
266231_at	At2g02220	0,62	0,56	0,70	0,82	0,73	0,90	0,96	0,94	leucine-rich repeat transmembrane protein kinase, putative
266203_at	At2g02230	1,05	1,25	1,09	0,79	0,96	1,21	1,06	0,98	F-box family protein / SKP1 interacting partner 3-related
266176_at	At2g02250	1,05	0,99	0,96	1,14	1,03	0,95	1,00	1,01	F-box family protein / SKP1 interacting partner 3-related
266177_at	At2g02270	0,97	0,95	1,08	1,07	1,01	1,04	1,02	0,94	pseudogene, F-box protein (SKP1 interacting partner 3-related)
266178_at	At2g02280	1,05	1,03	1,05	0,89	1,02	0,92	1,01	1,16	hypothetical protein

257378_s_at	At2g02290	1,04	1,10	0,99	0,96	1,04	0,97	0,92	0,89	NLI interacting factor (NIF) family protein
266179_at	At2g02300	0,96	0,98	0,92	0,97	1,07	1,09	1,09	0,93	F-box family protein / SKP1 interacting partner 3-related
266232_at	At2g02310	0,87	1,38	1,04	1,54	1,25	0,91	1,24	0,98	F-box family protein / SKP1 interacting partner 3-related
266233_at	At2g02340	0,91	0,84	1,05	0,95	1,15	0,99	0,97	1,10	F-box family protein / SKP1 interacting partner 3-related
266234_at	At2g02350	0,84	0,83	1,13	0,99	0,89	0,92	1,00	1,09	SKP1 interacting partner 3 (SKIP3)
266235_at	At2g02360	0,77	0,88	1,27	0,92	0,91	0,91	0,80	0,79	F-box family protein / SKP1 interacting partner 3-related
266182_at	At2g02370	0,64	0,95	0,92	0,85	0,75	0,84	1,09	1,51	expressed protein
266236_at	At2g02380	1,24	1,01	0,92	1,03	1,24	0,96	0,99	1,02	glutathione S-transferase, putative
266181_at	At2g02390	0,79	0,77	0,74	1,12	1,04	1,01	0,96	1,12	glutathione S-transferase zeta 1 (GSTZ1) (GST18)
266202_at	At2g02400	1,05	1,24	1,27	1,37	1,25	0,99	0,97	1,00	cinnamoyl-CoA reductase family
266204_at	At2g02410	0,78	1,01	0,95	0,72	0,74	1,02	1,26	1,28	expressed protein
266173_at	At2g02420	0,89	0,94	0,95	0,93	0,98	0,98	1,04	1,16	expressed protein
266174_at	At2g02440	0,94	0,97	1,04	0,94	1,02	1,04	1,00	1,14	hypothetical protein
266175_at	At2g02450	0,84	1,22	1,07	0,87	1,11	0,89	0,96	1,04	no apical meristem (NAM) family protein
266180_at	At2g02470	1,11	1,04	1,14	1,09	0,91	0,98	1,21	1,16	PHD finger family protein
267221_at	At2g02480	0,97	0,94	0,96	0,99	1,33	1,15	0,84	1,35	DNA polymerase-related
267241_at	At2g02490	1,05	1,01	1,01	1,06	1,06	1,13	0,98	1,03	Clone asmbl_4776 unknown mRNA sequence
267220_at	At2g02500	0,77	0,63	0,68	1,10	1,17	0,92	0,96	0,88	expressed protein
267239_at	At2g02510	1,22	0,97	0,91	1,37	1,42	0,91	0,80	0,83	expressed protein
267218_at	At2g02515	0,91	1,12	1,12	0,89	1,06	0,85	1,08	1,07	expressed protein
267273_at	At2g02520	0,96	0,97	1,08	1,12	0,95	1,01	1,07	1,01	hypothetical protein
267272_at	At2g02530	1,06	0,97	0,93	1,05	1,05	1,02	1,00	0,87	---
267271_at	At2g02540	1,15	1,13	0,99	1,08	1,20	0,94	0,80	0,80	zinc finger homeobox family protein / ZF-HD homeobox family protein
267270_at	At2g02550	1,01	1,03	1,04	1,07	1,10	0,94	1,03	1,05	exonuclease family protein
267269_at	At2g02560	1,11	1,07	1,06	1,06	0,90	1,07	1,00	0,91	TIP120 protein, putative
267268_at	At2g02570	1,06	0,98	1,09	0,85	0,90	1,16	1,38	1,63	expressed protein
267267_at	At2g02580	1,00	0,91	0,88	1,06	1,00	0,88	1,03	0,87	cytochrome P450 family protein
267219_at	At2g02590	0,88	1,17	1,14	0,84	0,68	0,82	0,84	0,73	expressed protein
267217_at	At2g02610	0,93	1,10	1,03	0,99	0,97	1,06	1,01	0,96	DC1 domain-containing protein
267216_at	At2g02620	1,04	1,08	0,96	1,03	0,92	0,98	1,06	1,23	DC1 domain-containing protein / PHD finger protein-related
267215_at	At2g02630	0,96	1,01	0,89	0,91	1,03	0,94	1,12	0,97	DC1 domain-containing protein
267245_at	At2g02640	0,97	0,99	1,03	0,99	1,00	0,96	1,04	1,09	DC1 domain-containing protein
267244_at	At2g02650	1,00	1,03	0,93	0,99	0,95	0,91	1,08	0,98	reverse transcriptase-related
267243_at	At2g02660	1,00	1,00	1,02	1,02	1,03	1,00	1,09	0,99	hypothetical protein
267242_at	At2g02670	1,01	0,94	0,96	1,02	1,10	1,05	0,98	0,86	---
267240_at	At2g02680	0,99	1,01	0,91	1,41	1,37	1,09	1,41	1,04	DC1 domain-containing protein
267479_at	At2g02690	0,93	1,04	0,83	1,06	1,04	0,93	0,96	1,00	hypothetical protein
267478_at	At2g02700	0,93	0,92	0,88	1,07	1,08	0,96	1,03	0,87	DC1 domain-containing protein
267477_at	At2g02710	0,92	1,34	1,97	0,88	0,76	1,12	1,54	2,04	PAC motif-containing protein
267476_at	At2g02720	1,05	0,87	0,96	0,92	1,02	1,06	1,01	0,85	pectate lyase family protein
267475_at	At2g02730	0,98	0,83	0,79	0,94	0,74	1,09	1,07	0,99	expressed protein
267474_at	At2g02740	1,17	0,92	0,99	0,82	0,79	1,01	1,03	0,96	transcription factor, putative
267473_at	At2g02750	1,01	0,88	0,90	0,85	1,02	0,91	0,95	0,94	pentatricopeptide (PPR) repeat-containing protein
267484_at	At2g02760	0,93	0,87	0,85	1,14	1,14	0,92	0,92	0,84	ubiquitin-conjugating enzyme 2 (UBC2)
267482_s_at	At2g02770	1,06	0,93	1,04	0,89	0,89	0,86	1,01	0,95	COP1-interacting protein-related
267481_at	At2g02780	0,67	0,72	0,77	1,20	1,32	0,92	0,93	0,87	leucine-rich repeat transmembrane protein kinase, putative

267486_at	At2g02800	0,91	0,92	1,04	1,16	1,14	0,94	1,02	1,20	protein kinase (APK2b)
267483_at	At2g02810	1,22	1,24	1,51	1,06	1,05	1,05	1,22	1,20	UDP-galactose/UDP-glucose transporter
267485_at	At2g02820	1,06	1,06	0,91	1,44	1,14	0,94	0,98	0,87	myb family transcription factor (MYB88)
267480_at	At2g02830	1,07	1,07	1,13	1,00	0,98	1,00	0,93	1,21	---
267472_at	At2g02850	0,40	0,40	0,37	0,77	1,11	0,85	1,11	1,10	plastocyanin-like domain-containing protein / plantacyanin, putative
266792_at	At2g02860	0,92	0,86	0,80	0,98	0,98	0,96	0,98	1,09	sucrose transporter / sucrose-proton symporter (SUC3)
266747_at	At2g02870	0,78	0,76	0,76	0,79	0,97	0,91	0,96	0,88	kelch repeat-containing F-box family protein
266796_at	At2g02880	1,18	1,09	1,29	0,90	0,91	0,97	1,01	0,78	mucin-related
266746_s_at	At2g02930	0,86	0,77	0,79	0,86	0,75	0,98	0,81	0,88	glutathione S-transferase, putative
266745_at	At2g02950	2,15	1,38	0,88	1,20	1,10	0,79	0,86	0,80	phytochrome kinase substrate 1 (PKS1)
266791_at	At2g02960	0,86	0,77	0,81	1,04	1,11	0,87	0,97	1,08	zinc finger (C3HC4-type RING finger) family protein
266744_at	At2g02970	1,00	0,81	0,88	0,96	0,98	0,96	0,86	0,90	nucleoside phosphatase family protein / GDA1/CD39 family protein
266794_at	At2g02980	0,94	0,84	0,97	0,92	0,87	1,05	1,07	0,96	pentatricopeptide (PPR) repeat-containing protein
266743_at	At2g02990	0,92	1,19	0,65	1,80	1,47	1,03	0,82	0,66	ribonuclease 1 (RNS1)
266771_s_at	At2g03010	0,97	0,97	0,94	0,99	1,07	0,94	1,05	1,07	expressed protein
266772_s_at	At2g03020	0,99	0,88	0,75	1,48	1,19	1,11	0,92	0,81	heat shock protein-related
257366_s_at	At2g03040	0,97	1,09	0,99	1,05	0,99	1,06	0,93	0,81	transmembrane protein-related
266742_at	At2g03050	0,69	0,85	1,23	1,03	1,15	0,89	0,80	0,71	mitochondrial transcription termination factor-related / mTERF-related
266793_at	At2g03060	0,92	0,90	0,89	0,75	0,84	0,99	0,91	0,91	MADS-box family protein
266795_at	At2g03070	1,63	1,56	2,08	1,41	1,61	1,40	1,23	1,50	expressed protein
266769_s_at	At2g03080	1,02	1,01	1,07	1,06	1,03	1,08	1,07	1,04	---
266770_at	At2g03090	1,15	1,56	0,95	1,76	1,55	1,04	1,04	1,13	expansin, putative (EXP15)
266730_at	At2g03110	0,89	0,97	1,24	0,97	1,04	1,10	0,99	1,10	KH domain-containing protein
266709_at	At2g03120	1,08	1,09	0,93	1,18	1,05	0,93	0,90	0,82	signal peptide peptidase family protein
266729_at	At2g03130	0,87	0,80	1,04	1,08	1,11	0,91	1,04	0,93	ribosomal protein L12 family protein
266728_at	At2g03140	0,94	1,15	1,19	0,91	0,72	1,30	1,31	1,45	CAAX amino terminal protease family protein
266727_at	At2g03150	1,21	1,19	1,07	0,89	0,80	1,20	1,51	1,66	ATP/GTP-binding protein family
266726_at	At2g03160	0,97	1,02	0,91	0,96	1,09	1,00	1,00	1,03	E3 ubiquitin ligase SCF complex subunit SKP1/ASK1 (At19), putative
266725_at	At2g03170	0,99	1,11	1,13	0,78	1,08	1,13	0,96	0,82	E3 ubiquitin ligase SCF complex subunit SKP1/ASK1 (At14), putative
266724_at	At2g03180	0,91	1,01	0,93	1,20	1,12	1,13	1,04	0,81	hypothetical protein
266723_at	At2g03190	1,05	0,98	0,97	0,91	1,00	0,88	0,84	1,17	E3 ubiquitin ligase SCF complex subunit SKP1/ASK1 (At16), putative
266708_at	At2g03200	1,06	1,36	1,01	0,90	0,90	1,00	1,09	0,99	aspartyl protease family protein
266722_at	At2g03210	0,92	1,03	1,02	0,89	1,07	1,03	0,89	0,89	xyloglucan fucosyltransferase, putative (FUT2)
266721_at	At2g03220	0,98	1,05	0,95	1,20	1,41	0,89	0,82	0,79	galactoside 2-alpha-L-fucosyltransferase / xyloglucan alpha-(1,2)-fucosyltra
266732_at	At2g03240	0,61	1,10	1,02	1,00	0,89	1,08	1,55	1,73	EXS family protein / ERD1/XPR1/SYG1 family protein
266731_at	At2g03260	0,99	1,10	1,07	1,09	1,01	1,10	0,98	1,06	EXS family protein / ERD1/XPR1/SYG1 family protein
266679_at	At2g03270	0,95	1,00	1,06	0,97	0,86	0,97	1,01	1,11	DNA-binding protein, putative
266733_at	At2g03280	1,14	1,02	1,12	1,00	1,04	0,88	1,32	0,98	expressed protein
266741_s_at	At2g03300	0,92	1,01	1,03	0,99	1,14	0,90	0,90	0,97	Toll-Interleukin-Resistance (TIR) domain-containing protein
266707_at	At2g03310	0,95	1,15	1,02	0,66	0,55	1,10	1,25	1,13	expressed protein
266706_at	At2g03320	0,97	1,06	1,04	0,82	1,07	1,02	1,01	0,96	hypothetical protein
265712_s_at	At2g03330	0,80	0,89	0,85	1,03	0,83	0,99	0,89	0,85	expressed protein
265718_at	At2g03340	0,89	1,03	1,13	0,87	0,79	1,20	1,04	1,02	WRKY family transcription factor
265716_at	At2g03350	1,05	0,89	0,86	1,08	1,22	0,93	0,73	0,83	expressed protein
265711_at	At2g03360	0,99	0,99	0,98	0,90	0,95	0,94	0,99	0,82	hypothetical protein
265710_at	At2g03370	0,99	1,06	1,17	0,94	1,05	1,04	0,94	0,98	hypothetical protein

265708_at	At2g03380	0,90	0,95	1,07	0,88	1,00	0,93	1,03	1,01	pentatricopeptide (PPR) repeat-containing protein
265707_at	At2g03390	0,94	0,96	1,16	0,74	0,68	1,15	1,11	1,25	uvrB/uvrC motif-containing protein
265706_at	At2g03400	0,96	0,95	1,18	0,73	0,61	1,13	1,08	1,37	uvrB/uvrC motif-containing protein
265705_at	At2g03410	1,03	0,94	1,02	0,93	1,08	0,99	0,93	1,03	Mo25 family protein
265704_at	At2g03420	0,75	0,69	0,92	0,81	0,83	1,20	1,11	1,21	expressed protein
265703_at	At2g03430	1,19	1,14	1,08	1,13	0,98	1,08	1,02	1,11	ankyrin repeat family protein
265702_at	At2g03450	0,96	0,95	1,05	1,07	0,88	1,08	1,01	0,73	purple acid phosphatase (PAP9)
265701_at	At2g03460	0,98	0,99	1,02	1,01	0,92	0,99	1,01	1,18	kelch repeat-containing protein
265700_at	At2g03470	0,78	0,87	0,62	0,98	1,04	0,98	0,97	0,91	myb family transcription factor / ELM2 domain-containing protein
265715_s_at	At2g03480	1,40	0,76	0,70	0,91	0,90	1,09	0,79	0,81	dehydration-responsive protein-related
265719_at	At2g03500	1,00	1,18	1,15	0,85	1,07	1,04	0,98	1,03	myb family transcription factor
265717_at	At2g03510	1,11	0,99	1,06	1,26	1,30	1,10	1,02	1,06	band 7 family protein
265714_at	At2g03520	0,98	1,04	0,97	0,82	0,89	0,91	1,07	1,11	expressed protein
265713_at	At2g03530	1,06	1,10	1,20	1,07	0,86	0,97	1,42	1,57	F-box family protein-related
265709_at	At2g03540	0,87	0,89	1,14	0,89	0,95	1,07	1,17	1,57	expressed protein
265699_at	At2g03550	0,47	0,73	1,04	0,70	0,60	0,78	0,79	1,01	expressed protein
264058_at	At2g03560	0,97	0,88	1,12	0,83	1,03	0,95	0,95	0,89	F-box family protein (FBX7)
264033_at	At2g03580	0,97	1,07	1,12	0,88	0,98	0,91	0,98	1,27	F-box family protein-related
264034_s_at	At2g03600	0,88	1,11	0,86	1,10	1,01	1,07	1,05	1,02	expressed protein
264035_at	At2g03630	1,12	0,97	0,98	0,92	1,10	1,12	0,91	0,98	hypothetical protein
264027_at	At2g03670	1,13	0,99	1,01	1,05	0,98	1,01	0,88	0,95	AAA-type ATPase family protein
264028_at	At2g03680	1,13	1,09	1,01	1,27	1,28	0,98	0,88	0,87	expressed protein
264038_at	At2g03690	0,76	0,82	0,98	1,10	1,19	0,88	0,90	0,90	coenzyme Q biosynthesis Coq4 family protein / ubiquinone biosynthesis Co
264041_at	At2g03710	0,98	1,00	1,07	0,83	0,76	1,56	1,46	1,33	MADS-box protein (AGL3)
264029_at	At2g03720	1,06	0,91	1,00	1,05	1,04	0,95	0,97	1,11	universal stress protein (USP) family protein
264040_at	At2g03730	1,81	1,76	1,34	0,58	0,62	1,16	1,39	1,11	ACT domain-containing protein (ACR5)
264039_at	At2g03740	0,90	0,97	1,01	1,12	1,15	1,02	0,93	0,91	late embryogenesis abundant domain-containing protein / LEA domain-cont
264037_at	At2g03750	1,11	1,08	1,21	0,90	0,74	0,76	0,98	0,97	sulfotransferase family protein
264042_at	At2g03760	0,83	0,57	0,83	0,63	0,55	1,31	1,06	1,13	steroid sulfotransferase, putative
264030_at	At2g03770	0,97	0,94	0,88	0,93	1,03	0,93	0,92	0,86	sulfotransferase family protein
264031_at	At2g03780	0,83	0,77	0,83	0,84	0,75	0,88	0,98	0,96	translin family protein
264032_at	At2g03800	0,79	0,74	0,76	1,04	1,14	1,01	1,02	1,01	expressed protein
264036_at	At2g03810	0,93	0,99	1,01	0,96	1,15	1,03	0,98	0,97	18S pre-ribosomal assembly protein gar2-related
263334_at	At2g03820	1,17	1,02	0,98	0,76	0,79	0,93	0,85	0,87	nonsense-mediated mRNA decay NMD3 family protein
263360_at	At2g03830	1,03	0,94	0,92	1,01	1,16	1,08	0,94	1,03	expressed protein
263361_at	At2g03840	1,05	1,12	0,99	0,89	1,07	0,93	1,13	0,94	senescence-associated family protein
263363_at	At2g03850	0,92	1,02	1,05	1,05	1,05	1,05	1,01	0,98	late embryogenesis abundant domain-containing protein / LEA domain-cont
263332_at	At2g03870	1,36	1,32	1,12	1,27	1,16	1,10	1,06	1,01	small nuclear ribonucleoprotein, putative / snRNP, putative / Sm protein, pu
263362_at	At2g03880	1,12	1,05	1,12	0,87	1,07	1,09	0,91	0,96	pentatricopeptide (PPR) repeat-containing protein
263333_at	At2g03890	0,94	1,32	1,14	0,87	1,15	1,02	0,93	1,01	phosphatidylinositol 3- and 4-kinase family protein
263482_at	At2g03980	0,81	0,92	1,17	0,93	1,05	0,91	0,79	0,81	GDSL-motif lipase/hydrolase family protein
263479_x_at	At2g04000	1,05	0,96	0,99	1,07	0,94	1,08	1,00	1,06	expressed protein
257441_at	At2g04020	0,98	1,02	1,00	0,94	1,01	1,09	0,99	1,02	GDSL-motif lipase/hydrolase family protein
263481_at	At2g04025	1,14	1,02	1,03	1,21	1,21	1,06	1,08	0,99	expressed protein
263483_at	At2g04030	1,23	1,03	1,09	0,75	0,75	1,09	1,04	1,15	heat shock protein, putative
263480_at	At2g04032	1,03	0,96	0,97	0,99	0,99	1,21	1,20	1,20	metal transporter, putative (ZIP7)

263407_at	At2g04038	1,04	1,03	0,94	1,09	1,06	1,05	1,03	1,01	bZIP transcription factor family protein
263410_at	At2g04039	0,79	0,71	0,73	1,08	1,00	1,20	1,11	1,15	expressed protein
263403_at	At2g04040	1,10	0,93	1,00	0,98	1,02	1,19	1,07	1,09	MATE efflux family protein
263402_at	At2g04050	3,64	2,85	3,48	0,60	0,58	2,71	2,49	2,66	MATE efflux family protein
263408_at	At2g04060	0,99	0,94	1,13	1,02	1,11	0,89	0,91	0,89	beta-galactosidase, putative / lactase, putative
263409_at	At2g04063	1,02	0,95	1,03	0,88	1,00	1,13	0,99	1,04	glycine-rich protein
263401_at	At2g04070	1,00	1,02	0,85	0,95	0,91	1,28	1,28	1,02	MATE efflux family protein
263405_s_at	At2g04080	0,74	0,71	0,65	0,92	0,97	0,83	0,79	0,62	MATE efflux protein-related
263404_s_at	At2g04100	1,40	1,24	1,11	1,46	1,22	1,09	1,15	1,16	MATE efflux family protein
263406_at	At2g04160	1,47	1,05	0,91	0,90	0,78	0,89	0,89	0,86	subtilisin-like protease (AIR3)
263051_s_at	At2g04210	1,02	1,01	1,01	1,02	1,00	1,05	1,02	0,89	CACTA-like transposase family (Ptta/En/Spm)
263324_at	At2g04220	1,00	0,94	1,00	1,16	0,98	1,02	1,11	1,16	hypothetical protein
257447_at	At2g04230	1,04	0,90	0,83	1,06	1,07	1,31	1,27	1,39	F-box family protein
263325_at	At2g04240	0,96	0,97	0,92	0,63	0,91	1,07	1,03	0,95	zinc finger (C3HC4-type RING finger) family protein
263322_at	At2g04270	1,58	1,78	1,74	0,71	0,75	1,32	1,15	0,92	glycoside hydrolase starch-binding domain-containing protein
263326_at	At2g04280	0,88	0,80	0,73	0,90	1,04	0,75	0,72	0,77	expressed protein
263323_at	At2g04300	0,96	1,04	0,97	0,99	0,93	0,93	1,01	0,99	leucine-rich repeat protein kinase, putative
263806_at	At2g04305	0,95	0,90	1,08	1,21	1,22	0,98	1,01	1,21	magnesium transporter CorA-like protein-related
263860_at	At2g04330	0,95	1,00	0,95	1,04	1,10	1,12	0,97	1,13	hypothetical protein
263808_at	At2g04340	1,28	1,08	1,27	1,04	1,23	1,11	0,90	0,90	expressed protein
263811_at	At2g04350	0,88	0,88	0,84	0,99	1,01	1,25	1,12	1,13	long-chain-fatty-acid--CoA ligase family protein / long-chain acyl-CoA synth
263859_at	At2g04360	1,11	1,27	1,24	0,89	0,98	1,06	1,03	0,98	expressed protein
263858_at	At2g04370	0,97	1,04	1,01	0,94	1,12	1,06	0,98	0,94	hypothetical protein
263857_at	At2g04380	0,98	1,06	1,02	0,97	0,97	1,00	1,02	1,00	expressed protein
263807_at	At2g04400	1,02	0,91	0,96	0,96	1,03	1,09	1,05	1,08	indole-3-glycerol phosphate synthase (IGPS)
263856_at	At2g04410	1,11	0,98	0,86	0,92	0,84	1,04	0,98	0,96	expressed protein
263855_at	At2g04420	1,03	0,85	0,91	0,94	1,12	0,94	0,98	1,10	expressed protein
263854_at	At2g04430	0,87	0,96	0,96	1,45	1,58	1,21	0,96	1,11	MutT/nudix family protein
263853_at	At2g04440	1,03	1,16	1,06	0,93	1,03	0,94	0,92	1,12	NUDIX/mutT hydrolase family protein
263852_at	At2g04450	0,80	1,03	0,76	1,11	1,10	1,25	1,17	1,03	MutT/nudix family protein
263851_at	At2g04460	1,42	1,32	1,10	1,22	1,36	0,84	0,95	0,80	expressed protein
263850_at	At2g04480	1,12	1,00	0,96	0,88	1,08	0,89	1,04	0,94	hypothetical protein
263849_at	At2g04490	1,04	0,91	1,24	0,93	1,34	1,14	1,15	1,17	---
263837_at	At2g04500	1,14	1,16	1,15	1,09	0,98	1,06	1,06	1,54	DC1 domain-containing protein
263810_at	At2g04520	1,05	0,87	0,87	1,00	0,94	1,03	1,00	0,92	eukaryotic translation initiation factor 1A, putative / eIF-1A, putative / eIF-4C
263864_at	At2g04530	1,04	0,95	0,90	0,81	0,76	1,18	1,11	1,32	RNase Z
263863_at	At2g04540	1,38	1,53	1,68	0,78	0,70	1,21	1,31	1,30	3-oxoacyl-(acyl-carrier-protein) synthase II, putative
263862_at	At2g04550	1,19	1,22	1,73	0,99	1,17	1,04	1,07	1,35	dual specificity protein phosphatase family protein
263861_at	At2g04560	0,93	1,21	1,23	1,08	1,05	1,04	1,21	1,02	glycotransferase family protein 19
263809_at	At2g04570	1,17	1,03	1,11	1,64	1,73	0,81	0,62	0,74	GDSL-motif lipase/hydrolase family protein
263848_at	At2g04580	1,08	1,00	1,08	1,06	0,99	1,04	0,97	0,83	hypothetical protein
263055_at	At2g04600	0,99	1,02	0,93	1,02	1,03	0,97	1,07	1,02	hypothetical protein
263054_at	At2g04620	0,65	0,67	0,75	1,01	0,92	0,94	0,80	0,81	cation efflux family protein
263621_at	At2g04630	1,29	0,98	0,91	0,95	0,90	1,09	1,14	0,89	DNA-directed RNA polymerase II, putative
263620_at	At2g04640	0,95	1,00	0,94	1,08	0,99	1,00	0,93	0,94	---
263619_at	At2g04650	0,95	0,85	0,86	0,99	1,23	0,99	0,71	0,98	ADP-glucose pyrophosphorylase family protein

263618_at	At2g04660	1,01	0,97	1,00	1,04	0,83	1,06	1,05	1,10	E3 ubiquitin ligase, putative
263617_at	At2g04670	0,99	1,04	0,95	0,98	1,07	0,87	0,95	1,02	---
263627_at	At2g04675	0,98	1,02	0,95	1,00	0,94	0,99	1,03	0,97	expressed protein
263616_at	At2g04680	0,94	0,94	0,96	1,03	0,98	0,91	1,02	1,03	DC1 domain-containing protein
263647_at	At2g04690	1,09	1,50	1,43	0,79	0,73	1,17	1,10	1,35	cellular repressor of E1A-stimulated genes (CREG) family
263624_at	At2g04700	0,95	0,83	0,81	0,90	0,80	1,01	1,03	1,04	ferredoxin thioredoxin reductase catalytic beta chain family protein
263646_at	At2g04710	1,08	1,00	0,98	0,97	0,98	0,99	0,92	0,96	---
263645_at	At2g04720	1,00	1,05	0,95	0,97	0,96	1,03	1,01	1,09	---
263644_at	At2g04730	1,04	1,02	0,91	1,07	1,11	1,08	0,98	0,97	---
263643_at	At2g04740	1,04	0,87	0,97	0,95	1,03	0,95	0,94	1,02	ankyrin repeat family protein
263642_at	At2g04750	0,98	1,05	0,99	0,99	1,14	1,06	1,00	1,07	fimbrin-like protein, putative
263641_at	At2g04760	1,00	1,01	0,99	1,12	0,93	1,03	1,02	0,92	---
263675_x_at	At2g04770	1,04	1,05	1,06	0,85	0,93	1,00	0,96	1,11	---
263628_at	At2g04780	1,02	0,97	0,82	1,68	1,70	0,81	0,74	0,73	fasciclin-like arabinogalactan-protein (FLA7)
263674_at	At2g04790	1,25	1,53	1,39	0,82	0,72	1,34	1,09	1,29	expressed protein
263632_at	At2g04795	1,04	1,20	0,97	1,85	1,45	1,04	1,41	1,39	expressed protein
263673_at	At2g04800	0,98	0,96	1,11	1,37	1,49	0,89	0,98	0,90	expressed protein
263672_at	At2g04820	1,00	0,97	1,06	1,14	0,95	1,01	0,88	1,22	---
263671_at	At2g04830	1,07	1,04	1,03	0,99	1,14	0,93	0,93	0,90	F-box family protein
263670_at	At2g04840	1,00	0,88	1,07	0,96	1,10	0,96	1,00	1,01	F-box family protein
263630_at	At2g04845	1,04	0,98	0,87	1,34	1,18	0,98	0,93	0,94	GCN5-related N-acetyltransferase (GNAT) family protein
263629_at	At2g04850	1,28	1,17	1,59	1,72	1,08	0,91	1,09	1,08	auxin-responsive protein-related
263623_at	At2g04860	1,03	1,04	1,06	0,90	1,08	1,06	1,06	0,97	pentatricopeptide (PPR) repeat-containing protein
263622_at	At2g04870	1,08	1,02	1,03	1,00	1,06	1,00	0,96	0,90	hypothetical protein
263626_at	At2g04880	1,29	1,69	1,79	0,88	1,10	1,22	1,48	1,47	WRKY family transcription factor (ZAP1)
263633_at	At2g04890	0,83	1,00	0,84	0,93	1,12	0,79	0,84	0,91	scarecrow-like transcription factor 21 (SCL21)
263631_at	At2g04900	1,11	1,01	0,82	1,28	1,01	0,78	0,85	0,88	expressed protein
263625_at	At2g04920	1,04	1,09	0,93	0,92	1,04	1,00	1,02	1,24	F-box family protein (FBX9)
263344_at	At2g04940	0,87	0,88	1,07	0,65	0,77	0,99	0,94	0,94	scramblase-related
263335_x_at	At2g04970	1,01	1,12	0,96	0,97	1,01	1,06	1,07	0,95	hypothetical protein
263336_x_at	At2g04980	1,01	0,97	1,09	0,93	0,98	0,99	1,15	0,97	hypothetical protein
263337_at	At2g04990	0,92	1,01	1,05	1,02	1,07	0,92	1,02	0,97	pseudogene, hypothetical protein
263338_at	At2g05000	0,94	1,13	1,02	0,99	1,12	1,09	1,07	1,11	hypothetical protein
263339_at	At2g05010	0,96	1,05	0,95	0,99	1,02	1,03	1,00	1,02	---
263340_at	At2g05020	1,13	1,02	1,13	0,92	1,06	1,05	1,02	1,09	---
263341_at	At2g05030	0,99	0,99	1,05	0,92	0,99	0,99	0,94	0,88	hypothetical protein
263345_s_at	At2g05070	1,17	1,26	1,96	1,07	1,01	1,09	1,01	1,17	chlorophyll A-B binding protein / LHCII type II (LHCB2.2)
263342_at	At2g05080	1,03	0,99	0,93	0,95	0,99	1,04	0,91	0,89	hypothetical protein
263343_at	At2g05090	0,97	0,97	0,96	0,98	1,02	0,93	1,08	1,00	hypothetical protein
263104_at	At2g05120	1,06	1,05	0,99	0,83	0,87	1,04	1,01	0,91	expressed protein
263106_at	At2g05160	0,74	1,31	1,07	0,76	0,83	0,91	1,12	1,03	zinc finger (CCCH-type) family protein / RNA recognition motif (RRM)-conta
263050_at	At2g05170	1,07	1,08	1,41	0,95	1,12	0,93	0,83	0,89	vacuolar protein sorting 11 family protein / VPS11 family protein
263107_at	At2g05180	0,99	0,97	0,94	0,86	0,97	0,91	1,07	0,85	cytochrome P450 family protein
263099_at	At2g05200	1,07	1,05	1,00	1,09	0,90	0,92	1,09	0,94	---
263100_at	At2g05210	0,96	0,95	1,16	0,84	0,87	0,94	1,11	0,92	expressed protein
263101_s_at	At2g05230	1,01	0,91	0,85	1,17	1,09	0,98	0,92	1,08	DNAJ heat shock N-terminal domain-containing protein

263049_at	At2g05260	0,98	1,00	1,14	1,11	1,12	1,07	1,05	1,01	lipase class 3 family protein
263102_at	At2g05270	1,04	0,90	0,91	0,94	0,92	1,06	0,97	0,84	expressed protein
263103_at	At2g05290	0,91	1,01	1,02	0,92	1,04	1,03	0,90	1,06	expressed protein
263048_s_at	At2g05310	0,95	0,97	1,18	0,81	0,87	1,15	1,10	0,97	expressed protein
263105_at	At2g05320	0,71	0,74	0,82	1,06	0,92	0,81	0,75	0,78	beta-1,2-N-acetylglucosaminyltransferase II
263078_at	At2g05350	0,93	1,07	0,98	1,03	1,07	0,91	0,88	1,13	hypothetical protein
263045_at	At2g05370	1,00	0,86	0,99	0,98	1,35	0,98	0,94	1,00	expressed protein
263046_at	At2g05380	0,85	0,79	0,80	1,12	1,25	0,83	0,88	0,86	glycine-rich protein (GRP3S)
263079_at	At2g05390	1,15	0,92	1,00	0,95	1,21	0,98	1,10	1,02	---
257458_at	At2g05400	0,91	1,01	1,02	0,96	1,11	1,08	1,03	0,87	meprin and TRAF homology domain-containing protein / MATH domain-cor
257457_at	At2g05430	1,04	1,02	0,85	0,95	0,98	0,91	1,00	0,96	expressed protein
257394_at	At2g05450	1,06	0,95	1,05	1,00	0,98	1,07	1,02	1,00	pseudogene, Ulp1 protease family
265564_s_at	At2g05460	1,07	1,13	0,95	1,11	0,95	1,01	0,95	1,06	expressed protein
265563_s_at	At2g05470	1,01	1,02	1,06	1,03	1,01	0,93	1,09	1,03	pseudogene, hypothetical protein
265562_at	At2g05500	0,97	0,97	1,05	0,95	1,08	1,03	1,04	1,07	expressed protein
265561_s_at	At2g05510	1,19	1,36	1,28	3,08	2,56	1,06	1,90	1,62	glycine-rich protein
265560_at	At2g05520	0,88	0,81	0,81	1,09	1,04	0,79	0,84	0,88	glycine-rich protein (GRP)
265559_at	At2g05530	0,99	0,83	0,96	1,10	1,02	0,94	0,90	0,96	glycine-rich protein
265511_at	At2g05540	0,71	0,83	0,91	0,89	0,70	1,13	1,34	1,37	glycine-rich protein
265558_at	At2g05550	1,04	0,98	0,94	0,96	1,11	0,99	0,99	1,16	---
265568_s_at	At2g05560	1,01	1,03	1,03	0,98	0,88	0,98	1,11	1,10	hypothetical protein
265567_at	At2g05580	0,87	0,87	0,83	0,96	1,07	0,94	0,97	1,27	pseudogene, glycine-rich protein
265512_at	At2g05590	1,23	1,12	1,19	1,09	1,23	1,11	1,07	1,17	expressed protein
265566_at	At2g05600	0,85	1,03	0,95	1,00	1,13	1,02	0,98	0,83	hypothetical protein
265565_at	At2g05610	0,99	0,98	1,05	0,96	0,95	0,98	0,89	1,00	---
265569_at	At2g05620	1,29	1,22	1,23	1,18	1,18	1,28	1,20	1,24	expressed protein
265510_at	At2g05630	1,25	1,00	1,07	0,95	0,96	1,09	1,01	0,89	autophagy 8d (APG8d)
265557_at	At2g05640	1,03	1,01	1,19	0,96	1,08	0,90	0,95	0,94	---
263346_at	At2g05650	0,92	0,94	0,98	0,95	1,01	1,00	0,93	1,01	---
263348_at	At2g05710	0,83	0,78	0,80	0,74	0,85	0,76	0,78	0,75	aconitate hydratase, cytoplasmic, putative / citrate hydro-lyase/aconitase, p
263347_at	At2g05720	0,87	1,15	1,39	0,86	0,90	0,88	0,97	1,01	transducin family protein / WD-40 repeat family protein
263315_at	At2g05755	0,95	0,90	0,87	1,07	0,78	0,98	0,84	0,72	integral membrane family protein
263314_at	At2g05760	1,07	1,25	1,22	1,01	0,83	1,02	1,07	0,83	xanthine/uracil permease family protein
265377_at	At2g05790	1,12	1,06	1,05	1,15	1,01	0,82	0,85	0,74	glycosyl hydrolase family 17 protein
265376_at	At2g05810	0,79	0,92	1,34	1,10	1,26	0,81	0,97	1,05	armadillo/beta-catenin repeat family protein
266027_at	At2g05820	0,99	1,02	1,03	1,03	1,03	1,06	0,99	1,00	---
266033_at	At2g05830	0,95	0,97	0,96	0,89	0,88	1,02	1,01	1,02	eukaryotic translation initiation factor 2B family protein / eIF-2B family prote
266036_s_at	At2g05840	1,02	0,86	0,91	0,93	1,07	0,98	0,97	1,00	20S proteasome alpha subunit A2 (PAA2)
266029_at	At2g05850	1,06	1,12	1,03	1,01	1,12	1,08	0,99	1,16	serine carboxypeptidase S10 family protein
266030_x_at	At2g05870	0,99	1,10	0,94	1,00	1,10	0,97	0,98	0,95	cytochrome P-450 aromatase-related
266031_at	At2g05880	1,03	0,95	1,01	1,02	1,02	1,12	1,04	0,94	replication protein-related
266032_x_at	At2g05890	0,96	1,08	1,00	1,00	1,02	1,10	0,92	1,01	hypothetical protein
266020_at	At2g05900	1,01	1,06	1,01	0,97	0,86	0,98	1,06	0,99	SET domain-containing protein / YDG/SRA domain-containing protein
266021_at	At2g05910	1,40	1,29	1,42	0,95	1,06	1,22	1,20	1,19	expressed protein
266022_at	At2g05920	0,86	0,88	0,98	1,01	1,02	0,86	0,91	0,87	subtilase family protein
266023_at	At2g05930	0,89	0,95	0,91	0,94	1,12	1,03	0,94	1,04	---

266037_at	At2g05940	1,06	1,68	1,13	0,91	1,03	1,47	1,46	1,30	protein kinase, putative
266024_at	At2g05950	0,97	0,98	1,07	0,92	0,92	0,90	1,02	0,99	hypothetical protein
266025_at	At2g05960	0,94	1,03	1,04	0,98	1,12	1,02	1,02	0,94	---
266026_at	At2g05980	0,98	1,05	1,00	0,98	0,97	0,87	0,95	1,00	---
266035_at	At2g05990	0,92	0,88	0,71	0,87	0,95	0,85	0,84	0,79	enoyl-(acyl-carrier protein) reductase (NADH), chloroplast, putative / NADH
266028_at	At2g06000	1,08	1,02	0,91	1,06	1,04	0,95	0,95	0,87	pentatricopeptide (PPR) repeat-containing protein
266034_at	At2g06005	1,02	1,08	0,99	0,83	1,11	0,90	1,15	1,00	expressed protein
265521_at	At2g06010	1,16	1,15	1,46	0,59	0,66	1,16	1,16	1,04	expressed protein
265520_at	At2g06020	1,00	0,92	0,99	0,85	1,03	1,09	0,94	0,88	myb family transcription factor
265519_at	At2g06030	1,20	0,98	1,19	0,90	1,03	0,82	0,97	0,93	expressed protein
265518_at	At2g06040	1,24	1,06	1,09	0,84	0,99	0,98	1,00	0,92	expressed protein
265530_at	At2g06050	0,71	0,90	1,78	1,16	1,26	0,93	1,03	1,11	12-oxophytodienoate reductase (OPR3) / delayed dehiscence1 (DDE1)
265517_at	At2g06090	0,95	1,06	1,00	1,02	0,95	1,02	0,89	1,05	self-incompatibility protein-related
265516_at	At2g06110	1,00	1,04	0,94	0,93	1,05	0,93	1,04	1,02	---
265515_at	At2g06120	0,90	0,95	0,98	0,97	1,04	0,98	1,07	1,04	hypothetical protein
265514_s_at	At2g06130	0,88	0,91	0,86	0,96	1,02	0,99	1,01	1,09	---
265513_at	At2g06140	1,10	0,93	0,99	0,99	0,95	1,12	0,96	0,91	hypothetical protein
265527_at	At2g06150	0,96	0,99	0,93	1,01	1,02	1,00	0,93	0,98	---
265526_x_at	At2g06160	1,04	0,96	1,08	0,84	1,12	0,98	0,99	1,02	---
265525_at	At2g06170	0,99	1,02	1,14	0,99	1,08	0,96	1,01	1,00	---
265524_at	At2g06180	0,87	1,02	1,16	1,02	1,02	0,97	0,95	0,97	---
265523_at	At2g06190	0,96	0,96	1,20	0,96	1,14	1,10	1,02	0,95	gypsy-like retrotransposon family
265531_at	At2g06200	0,95	1,05	1,14	0,93	1,26	1,10	1,03	0,96	expressed protein
265522_at	At2g06210	0,98	1,06	1,26	0,90	0,91	0,91	1,08	1,11	phosphoprotein-related
265528_at	At2g06220	0,97	0,84	0,93	1,06	1,03	1,04	1,11	1,01	---
265529_at	At2g06230	0,93	1,01	0,96	0,87	1,09	0,83	1,10	1,00	hypothetical protein
263772_at	At2g06255	0,94	1,01	1,05	1,16	1,45	0,96	0,95	1,05	expressed protein
263771_at	At2g06260	0,95	1,10	0,98	1,07	1,24	0,98	1,07	1,27	---
263801_at	At2g06310	1,00	0,96	1,07	0,94	0,97	0,84	0,97	1,09	---
263767_at	At2g06320	0,94	1,01	0,96	0,98	0,98	1,04	1,03	1,05	---
263768_x_at	At2g06330	0,86	0,92	1,09	1,00	0,88	1,00	0,95	0,87	---
263769_at	At2g06390	0,99	0,99	1,05	0,99	1,23	1,08	0,93	0,95	hypothetical protein
263770_at	At2g06410	1,12	0,97	0,88	0,99	0,93	1,06	0,82	0,98	---
263254_x_at	At2g06430	1,00	1,14	1,09	1,04	1,07	1,01	1,01	0,92	Ulp1 protease family protein
265369_s_at	At2g06470	0,93	0,97	1,02	1,00	0,82	1,04	0,96	1,18	---
265370_s_at	At2g06480	0,96	1,02	0,94	0,98	0,94	0,94	0,85	1,00	gypsy-like retrotransposon family
265371_at	At2g06490	1,00	0,92	0,97	0,93	1,01	0,97	1,14	1,01	CACTA-like transposase family (Ptta/En/Spm)
265372_at	At2g06500	1,07	1,02	0,95	0,93	1,12	1,02	1,06	0,99	hAT dimerisation domain-containing protein / transposase-related
265373_at	At2g06510	1,13	0,91	1,06	0,96	0,77	0,95	1,10	0,88	replication protein, putative
265374_at	At2g06520	0,98	0,88	0,85	0,91	0,82	0,99	0,90	0,86	membrane protein, putative
265375_at	At2g06530	0,91	0,93	1,20	0,93	1,14	1,11	1,06	1,23	SNF7 family protein
265746_at	At2g06630	1,01	1,02	0,95	1,04	0,98	1,17	1,04	0,84	hypothetical protein
265745_at	At2g06640	1,00	0,97	0,98	1,03	0,85	1,03	1,03	0,94	pseudogene, hypothetical protein
265747_s_at	At2g06645	0,92	1,03	1,01	1,10	1,02	1,13	0,95	1,10	hypothetical protein
265744_at	At2g06660	1,05	1,07	1,09	0,99	1,15	0,94	0,96	0,85	---
257390_x_at	At2g06700	1,04	1,10	0,97	1,11	0,98	1,00	1,07	1,02	hypothetical protein

265743_s_at	At2g06730	0,94	0,97	0,98	0,95	1,21	1,03	0,99	1,04	CACTA-like transposase family (Ptta/En/Spm)
266211_at	At2g06820	0,92	1,03	1,01	0,91	0,84	1,08	0,90	0,93	expressed protein
266212_at	At2g06840	1,02	0,99	1,08	1,00	1,09	1,09	0,95	0,88	---
266215_at	At2g06850	1,23	1,22	1,07	1,22	1,26	0,95	0,85	0,81	xyloglucan:xyloglucosyl transferase / xyloglucan endotransglycosylase / en
266213_s_at	At2g06870	0,98	0,95	1,08	0,90	0,90	1,03	1,05	1,06	---
266214_at	At2g06890	0,94	1,11	1,05	1,07	1,04	0,98	0,98	1,05	gypsy-like retrotransposon family
266500_at	At2g06925	0,45	0,40	0,42	0,75	0,61	0,70	0,68	0,67	phospholipase A2 family protein
266497_at	At2g06950	1,02	0,91	1,08	0,95	1,18	0,95	1,01	0,91	---
266498_at	At2g06960	0,95	0,92	0,96	0,92	1,08	1,00	0,87	0,79	oxidoreductase, 2OG-Fe(II) oxygenase family protein
266499_at	At2g06970	1,01	0,99	1,03	1,03	1,24	1,07	0,99	1,06	---
266501_at	At2g06990	1,17	1,20	1,08	0,92	0,83	1,18	1,11	0,97	HUA enhancer 2 (HEN2) / DExH-box RNA helicase, putative
266490_at	At2g07000	1,04	1,09	1,03	1,02	0,97	1,08	0,91	1,04	expressed protein
266491_at	At2g07010	0,95	0,89	1,11	0,99	1,01	0,88	0,93	1,09	---
266492_at	At2g07020	1,00	1,08	0,94	1,02	1,01	1,03	1,12	0,92	protein kinase family protein
266493_at	At2g07030	0,89	1,10	0,93	1,14	0,94	1,00	0,98	0,85	---
266494_at	At2g07040	0,88	0,99	0,96	0,90	0,96	0,93	0,88	0,92	leucine-rich repeat transmembrane protein kinase, putative
266495_at	At2g07050	0,82	0,81	0,74	0,95	1,01	0,82	0,85	0,79	cycloartenol synthase (CAS1) / 2,3-epoxysqualene--cycloartenol cyclase / (
266496_at	At2g07070	1,00	0,95	0,99	1,13	1,16	0,95	1,00	1,14	---
266432_s_at	At2g07080	1,06	0,93	1,01	0,95	1,03	1,02	1,00	0,95	---
266433_at	At2g07110	0,96	0,94	1,02	0,95	0,97	0,97	0,96	1,03	hypothetical protein
266434_s_at	At2g07120	0,98	1,07	1,01	0,98	1,05	0,91	0,93	0,88	F-box family protein-related
266435_s_at	At2g07130	1,00	1,00	1,06	1,00	1,01	1,05	0,99	0,98	pseudogene, hypothetical protein
266426_x_at	At2g07140	0,94	1,06	1,02	1,14	0,88	1,05	1,10	1,01	F-box family protein
266427_at	At2g07170	1,12	1,18	1,39	1,14	1,14	0,81	0,96	0,86	expressed protein
266428_at	At2g07180	1,16	1,32	1,60	0,87	1,09	0,93	0,94	1,18	protein kinase, putative
266429_at	At2g07190	1,02	0,92	0,95	0,99	1,02	1,03	0,93	0,95	hypothetical protein
266430_at	At2g07200	0,87	1,05	1,24	0,87	0,95	1,02	1,17	1,05	---
266431_at	At2g07230	0,91	1,00	0,98	0,93	1,07	1,02	0,91	1,16	---
265774_at	At2g07240	1,00	1,00	1,02	1,18	1,10	1,17	0,93	1,19	Ulp1 protease family protein
265784_at	At2g07280	0,97	1,09	1,06	0,96	0,93	0,94	0,77	0,87	hypothetical protein
265785_at	At2g07290	1,08	1,08	1,03	0,95	1,00	1,11	0,84	1,00	hypothetical protein
265786_at	At2g07300	1,01	0,97	1,03	0,99	0,94	0,97	0,98	1,13	hypothetical protein
265787_at	At2g07320	1,00	0,98	0,97	1,02	0,92	1,01	1,17	0,87	SWIM zinc finger family protein
265775_at	At2g07330	0,96	0,94	1,06	1,09	1,05	1,09	0,97	1,15	---
265776_at	At2g07340	1,34	1,02	0,77	0,99	1,11	1,07	1,01	0,86	prefoldin-related KE2 family protein
265777_at	At2g07350	1,30	1,01	0,91	1,10	1,13	1,00	1,09	1,07	prefoldin-related KE2 family protein
265778_at	At2g07360	0,98	0,94	0,91	1,06	1,08	0,95	0,86	0,90	SH3 domain-containing protein
265779_at	At2g07370	0,87	0,90	0,87	1,12	0,91	0,90	0,91	0,76	SH3 domain-containing protein
265780_at	At2g07380	1,01	0,86	1,11	0,94	0,99	1,11	0,96	1,06	Mutator-like transposase family
265781_at	At2g07430	1,05	0,96	0,92	0,92	1,02	1,03	0,96	1,02	---
265782_at	At2g07440	1,07	0,98	1,03	0,99	1,08	1,08	0,86	1,03	two-component responsive regulator-related / response regulator protein-re
265783_at	At2g07450	0,96	1,07	1,00	1,03	1,18	0,96	0,97	0,89	---
265556_at	At2g07490	0,93	0,99	1,03	1,00	1,03	0,83	1,05	1,09	---
265555_at	At2g07500	0,96	1,05	0,93	0,96	0,90	0,99	1,04	1,02	---
265554_at	At2g07505	0,98	1,07	1,07	1,01	1,07	1,15	0,93	1,02	hypothetical protein
265550_at	At2g07510	0,97	1,15	0,96	0,79	1,01	1,13	0,98	1,06	pseudogene, hypothetical protein

265549_at	At2g07520	0,94	0,98	0,97	0,98	1,06	1,02	0,89	1,05	hypothetical protein
265553_at	At2g07550	0,94	1,09	0,92	0,96	0,99	1,02	1,06	1,03	---
265552_at	At2g07560	0,97	1,07	1,00	1,03	1,11	1,06	0,95	1,02	ATPase, plasma membrane-type, putative / proton pump, putative
265548_s_at	At2g07620	1,01	1,02	1,02	1,01	0,95	0,98	1,01	1,02	pseudogene, similar to putative helicase
265551_at	At2g07630	0,99	0,99	1,00	0,88	1,00	1,02	1,00	0,95	hypothetical protein
263058_at	At2g07650	1,08	1,13	0,98	0,97	1,08	0,87	0,96	1,11	---
263059_at	At2g07670	0,96	1,13	0,95	1,15	1,12	0,98	0,98	1,20	---
263500_s_at	At2g07672	0,92	0,90	1,02	1,01	0,93	1,02	0,87	0,95	hypothetical protein
263501_s_at	At2g07673	0,95	0,86	1,06	1,02	1,06	0,90	1,01	1,19	hypothetical protein
263502_s_at	At2g07675	0,83	0,70	0,66	0,58	0,63	0,73	0,74	0,81	ribosomal protein S12 mitochondrial family protein
263503_s_at	At2g07676	0,94	1,03	1,00	0,90	0,93	0,87	0,86	0,98	expressed protein
263504_s_at	At2g07677	0,82	0,95	1,03	0,89	1,01	0,95	1,01	0,95	---
266038_at	At2g07680	1,57	1,55	1,99	0,94	1,00	1,02	1,21	1,04	ABC transporter family protein
263510_s_at	At2g07681	0,91	0,90	0,91	0,91	1,02	0,98	1,00	1,06	cytochrome c biogenesis protein, putative
263506_s_at	At2g07683	0,95	0,91	0,92	1,08	1,04	0,96	1,04	0,85	---
263507_s_at	At2g07684	0,90	0,92	1,00	0,85	0,97	0,96	1,12	0,88	---
263508_s_at	At2g07685	0,95	0,99	0,92	0,94	1,00	0,94	1,13	0,85	---
263509_s_at	At2g07687	0,52	0,53	0,67	0,80	0,60	0,80	0,77	0,64	cytochrome c oxidase subunit 3
266959_at	At2g07690	1,66	1,65	1,57	1,32	1,06	1,16	1,10	1,20	minichromosome maintenance family protein / MCM family protein
265240_s_at	At2g07691	1,02	0,99	1,09	0,93	1,20	0,99	1,09	1,06	hypothetical protein
265239_s_at	At2g07692	0,98	0,94	0,96	1,20	1,11	0,99	1,01	1,01	hypothetical protein
265241_at	At2g07693	0,94	1,01	1,03	0,95	0,97	0,96	0,95	1,06	---
265227_s_at	At2g07695	0,87	0,94	0,87	1,02	1,05	1,00	0,92	1,18	cytochrome c oxidase subunit II, putative
265238_s_at	At2g07696	0,86	0,87	0,99	1,00	0,90	0,85	1,01	1,03	ribosomal protein S7 family protein
265228_s_at	At2g07698	0,70	0,72	0,77	0,74	0,74	0,75	0,83	0,86	ATP synthase alpha chain, mitochondrial, putative
266960_at	At2g07700	0,95	0,97	0,94	0,96	1,09	0,98	1,02	0,97	---
265229_s_at	At2g07701	0,65	0,97	1,11	0,93	1,16	0,90	0,81	1,09	expressed protein
266013_s_at	At2g07702	0,79	1,00	1,07	1,02	0,96	1,02	1,04	0,95	expressed protein
265242_at	At2g07705	0,86	1,04	1,01	0,93	0,86	0,99	0,91	0,94	hypothetical protein
265237_s_at	At2g07706	0,99	0,96	0,93	0,95	1,10	0,91	0,91	0,94	expressed protein
265230_s_at	At2g07707	0,98	0,90	0,83	0,66	0,77	0,94	0,97	0,87	hypothetical protein
265231_s_at	At2g07713	0,96	0,94	0,98	1,08	1,07	1,08	1,05	1,08	expressed protein
265236_s_at	At2g07714	1,01	0,90	0,90	1,08	0,93	1,01	1,07	0,84	transcription factor-related
265232_s_at	At2g07715	0,81	0,84	0,81	0,77	0,96	0,86	0,94	1,03	ribosomal protein L2, putative
265233_s_at	At2g07718	0,64	0,69	0,52	0,78	0,69	0,85	0,77	0,73	cytochrome b, putative
265235_s_at	At2g07719	0,92	0,91	0,99	1,35	0,96	0,91	1,03	0,97	expressed protein
266961_at	At2g07720	0,99	0,97	1,04	0,99	1,00	1,13	1,01	1,04	---
265234_at	At2g07721	1,05	0,87	0,90	0,86	0,93	0,85	0,92	1,13	hypothetical protein
266014_s_at	At2g07722	0,88	0,95	1,09	0,58	0,91	0,70	0,95	0,91	hypothetical protein
266043_at	At2g07724	0,99	1,00	1,08	1,02	1,16	0,96	0,89	0,84	expressed protein
266044_s_at	At2g07725	0,87	0,79	0,76	0,81	0,65	0,83	0,82	0,93	60S ribosomal protein L5 (RPL5)
266045_s_at	At2g07727	0,81	0,71	0,93	0,78	0,92	0,85	0,81	0,60	cytochrome b (MTCYB) (COB) (CYTB)
266046_at	At2g07728	0,91	0,96	1,24	0,84	0,87	0,98	0,98	1,14	expressed protein
266931_at	At2g07730	1,07	1,03	0,91	1,00	1,07	0,91	0,89	1,18	---
266042_s_at	At2g07734	1,09	1,01	1,03	1,18	1,19	0,96	0,83	0,87	ribosomal protein S4 (RPS4)
266041_s_at	At2g07736	0,99	0,98	0,85	0,96	1,02	1,01	0,94	1,28	---

266040_at	At2g07738	1,06	1,09	0,95	1,05	1,04	1,03	1,05	0,99	hypothetical protein
266039_s_at	At2g07739	0,92	0,88	1,01	0,93	0,84	0,86	1,03	0,98	expressed protein
257361_at	At2g07740	0,83	1,14	1,05	1,04	1,10	1,00	0,93	0,93	zinc knuckle (CCHC-type) family protein
266012_s_at	At2g07741	0,57	0,56	0,69	0,79	0,85	0,79	0,64	0,81	pseudogene, similar to ATPase subunit 6
266932_s_at	At2g07750	1,46	1,22	1,30	0,87	1,00	1,05	1,05	1,17	DEAD box RNA helicase, putative
266933_at	At2g07760	1,00	0,90	1,02	0,96	1,09	0,93	1,01	0,99	zinc knuckle (CCHC-type) family protein
263057_at	At2g09840	0,99	1,02	1,06	0,96	1,05	0,92	1,01	1,12	hypothetical protein
263820_at	At2g09910	0,93	1,10	1,08	0,90	1,15	0,96	1,09	0,86	hypothetical protein
263812_at	At2g09960	1,00	0,94	1,00	1,10	1,02	0,95	1,04	1,05	expressed protein
263821_s_at	At2g09990	1,30	1,31	1,24	1,25	1,32	1,00	0,98	1,02	40S ribosomal protein S16 (RPS16A)
263813_s_at	At2g10000	0,96	1,15	0,99	1,07	1,18	0,99	1,08	0,94	---
263814_at	At2g10010	1,04	0,92	1,03	1,01	1,13	0,94	1,02	0,98	---
263815_at	At2g10020	0,87	0,98	0,97	0,89	0,99	1,02	1,12	1,04	hypothetical protein
263816_at	At2g10050	0,88	0,92	0,97	1,02	0,96	1,09	0,98	1,11	zinc knuckle (CCHC-type) family protein
263817_at	At2g10070	0,95	1,04	1,02	1,03	1,15	1,03	0,96	0,80	expressed protein
263818_at	At2g10090	1,09	1,04	1,06	1,00	1,01	1,06	1,06	0,96	hypothetical protein
263819_x_at	At2g10140	0,90	1,13	0,98	0,95	0,97	1,09	1,04	1,25	---
263311_at	At2g10340	0,96	1,08	1,02	0,97	1,08	1,12	0,86	1,04	hypothetical protein
263309_at	At2g10370	1,02	1,03	0,98	0,99	1,03	1,03	0,96	0,98	hypothetical protein
263308_at	At2g10380	0,97	0,90	0,96	1,00	1,03	0,98	0,96	1,03	hypothetical protein
257445_x_at	At2g10390	0,96	0,98	1,02	0,89	1,25	1,02	1,04	0,98	hypothetical protein
257341_at	At2g10400	0,97	1,00	0,96	1,01	1,13	0,98	1,01	0,99	---
257446_at	At2g10440	1,02	0,92	0,97	1,08	0,99	0,93	1,00	0,95	hypothetical protein
263313_at	At2g10450	0,96	1,07	0,95	0,95	1,03	0,91	0,95	0,96	14-3-3 protein, putative / grf15, putative
263312_at	At2g10460	1,06	0,94	0,99	1,05	1,00	1,04	0,97	1,08	hypothetical protein
263394_at	At2g10465	0,94	0,93	0,97	1,01	1,11	1,00	0,98	0,97	replication protein-related
263392_at	At2g10490	0,98	0,96	1,07	0,95	1,10	1,04	1,03	1,12	---
263393_at	At2g10500	0,98	0,97	1,03	1,05	1,03	0,96	0,98	0,98	hypothetical protein
265749_at	At2g10610	0,95	1,02	1,03	0,86	0,96	1,01	1,07	0,91	---
265748_at	At2g10620	1,03	1,04	0,96	0,97	1,04	1,01	1,01	0,93	---
265755_at	At2g10630	1,01	1,00	1,00	1,05	0,98	1,01	1,08	0,95	---
265754_x_at	At2g10640	0,90	1,03	0,95	0,96	1,11	1,11	0,97	0,98	---
265753_at	At2g10650	1,01	0,96	1,03	0,93	0,98	1,00	0,97	1,12	---
265752_at	At2g10660	0,97	1,05	1,01	1,01	1,01	0,97	1,01	0,95	---
265751_at	At2g10690	1,04	0,97	1,05	0,90	1,04	0,98	0,99	0,86	---
265750_x_at	At2g10740	1,02	1,08	1,10	0,94	1,00	1,14	0,93	0,95	---
264072_at	At2g10750	0,93	1,06	1,04	1,10	0,98	0,98	0,92	0,95	MRNA sequence
257340_at	At2g10760	1,08	1,03	1,09	0,94	1,01	0,98	1,02	0,96	---
264074_at	At2g10780	0,99	1,05	0,99	0,96	0,99	0,99	1,00	1,04	---
264073_at	At2g10840	1,03	1,03	1,04	1,00	1,05	1,05	0,99	0,97	pseudogene, hypothetical protein
264075_at	At2g10850	0,99	0,95	1,03	0,92	1,04	1,08	0,86	1,06	hypothetical protein
263294_at	At2g10870	1,07	1,03	0,96	0,90	0,84	0,97	1,25	1,12	hypothetical protein
263293_x_at	At2g10880	1,03	0,95	0,91	0,92	1,30	1,00	0,97	0,80	hypothetical protein
263292_at	At2g10900	0,96	1,05	0,96	1,06	1,00	1,19	1,00	0,98	hypothetical protein
263291_at	At2g10920	0,98	0,85	0,85	0,91	0,98	1,09	1,10	1,09	hypothetical protein
263290_at	At2g10930	1,01	1,06	0,93	0,87	1,11	0,83	0,77	0,95	expressed protein

265400_at	At2g10940	1,32	1,22	1,05	0,81	0,83	0,93	0,81	0,82	protease inhibitor/seed storage/lipid transfer protein (LTP) family protein
265402_at	At2g10950	1,04	0,97	0,95	1,07	1,05	1,01	0,99	0,94	BSD domain-containing protein
265401_at	At2g10970	1,07	0,89	1,04	0,91	0,99	0,98	1,04	0,88	invertase/pectin methylesterase inhibitor family protein
265398_at	At2g11000	1,21	0,99	1,12	0,88	0,95	1,14	1,04	0,99	natC N(alpha)-terminal acetyltransferase, Mak10 subunit family protein
265399_at	At2g11010	0,97	1,05	1,07	1,03	1,09	0,91	0,93	1,00	hypothetical protein
265397_at	At2g11090	0,95	0,92	0,97	0,90	0,99	1,10	1,08	0,98	expressed protein
265969_at	At2g11110	0,99	0,99	1,02	0,99	1,12	0,92	0,98	0,94	---
265976_at	At2g11120	0,98	0,94	1,03	0,95	1,07	1,03	0,94	0,93	---
265977_at	At2g11130	0,96	0,90	1,06	0,99	1,15	0,98	1,08	1,12	---
265978_at	At2g11140	0,88	0,98	0,97	1,04	1,15	1,15	0,94	0,94	---
265979_at	At2g11150	0,99	1,02	0,98	1,00	0,98	0,90	0,94	1,09	pseudogene, hypothetical protein
265980_at	At2g11160	0,98	0,93	0,96	1,01	1,10	0,99	0,94	1,06	pseudogene, hypothetical protein
265981_s_at	At2g11170	0,92	1,07	0,81	0,94	0,98	0,93	1,05	1,21	hypothetical protein
265982_at	At2g11190	1,04	1,08	1,03	1,10	0,83	1,11	0,92	0,97	---
265970_at	At2g11210	1,06	0,96	0,92	0,92	1,18	1,10	1,11	0,99	---
265971_at	At2g11220	0,98	1,07	0,99	0,99	1,05	0,98	1,02	1,04	---
265972_at	At2g11230	1,02	0,99	0,95	1,02	1,00	1,01	1,03	0,95	---
265973_at	At2g11240	1,00	1,08	0,95	0,97	1,18	0,90	0,80	0,87	---
265974_at	At2g11260	1,00	0,95	1,04	0,93	0,98	0,83	0,95	0,98	Hypothetical protein, complete cds, clone: RAFL16-43-P18
265975_at	At2g11270	1,01	1,02	1,03	0,99	1,07	1,05	1,10	1,28	citrate synthase-related
265533_at	At2g11360	0,92	1,02	0,96	1,05	0,96	0,90	1,02	0,98	hypothetical protein
265532_at	At2g11370	0,90	1,05	1,10	1,11	1,22	1,00	1,07	0,97	hypothetical protein
265534_at	At2g11390	0,97	1,00	0,99	0,95	0,97	0,99	1,02	1,05	---
263271_s_at	At2g11480	0,97	0,95	1,09	1,00	1,05	0,91	1,11	1,02	Ulp1 protease family protein
263270_at	At2g11490	0,88	1,05	1,05	0,94	1,03	1,05	1,11	1,03	hypothetical protein
263274_at	At2g11520	0,91	0,86	0,94	1,00	1,00	0,92	1,00	1,01	protein kinase family protein
263269_at	At2g11540	1,15	1,01	0,96	0,98	1,02	1,02	1,00	1,02	---
263300_at	At2g11560	0,92	1,04	0,94	0,98	1,10	0,92	1,00	1,02	---
263273_x_at	At2g11580	0,98	1,00	0,98	0,94	0,92	0,94	0,96	1,00	---
263299_s_at	At2g11600	1,06	1,11	0,95	1,14	1,18	1,08	0,97	1,04	hypothetical protein
263272_at	At2g11650	1,02	0,95	0,95	0,89	1,19	1,02	0,93	1,02	---
263389_at	At2g11680	0,98	1,02	1,04	1,10	1,07	1,03	0,98	1,03	---
263388_at	At2g11690	1,00	0,99	1,06	0,98	0,97	0,93	0,92	0,94	CACTA-like transposase family (Tnp1/En/Spm)
263391_at	At2g11810	1,01	1,12	0,91	1,65	1,03	1,78	1,64	1,19	1,2-diacylglycerol 3-beta-galactosyltransferase, putative / monogalactosyldi
263390_at	At2g11830	0,90	1,00	1,03	1,02	1,00	1,04	1,01	0,98	hypothetical protein
263637_at	At2g11890	1,00	0,83	0,80	0,99	0,95	1,02	0,83	0,90	expressed protein
263636_at	At2g11910	1,19	1,06	1,00	0,95	1,02	0,88	0,93	0,87	expressed protein
263635_at	At2g11920	0,96	1,08	1,00	0,91	0,97	1,11	0,97	1,08	---
263634_at	At2g11940	0,94	1,06	0,98	1,02	1,06	0,99	1,05	1,06	---
266152_s_at	At2g12050	1,39	1,20	1,04	1,03	1,03	0,95	1,02	0,98	pseudogene, embryogenesis protein-related
257379_x_at	At2g12150	0,95	1,03	1,21	0,94	1,30	1,02	1,06	0,97	Mutator-like transposase family
266144_at	At2g12170	0,94	1,00	0,98	1,06	0,98	1,04	0,99	0,96	expressed protein
266154_at	At2g12190	1,06	0,89	0,98	1,29	0,97	1,09	0,90	0,85	cytochrome P450, putative
266145_at	At2g12210	1,02	0,96	1,01	1,02	0,97	0,99	1,00	0,98	---
266146_at	At2g12220	0,96	1,01	1,08	1,01	1,11	1,01	0,94	0,94	---
266147_at	At2g12230	1,11	0,98	0,98	1,02	1,03	1,01	1,00	1,06	pseudogene, C-1-tetrahydrofolate synthase

266148_x_at	At2g12240	0,87	1,08	0,93	0,84	0,88	1,21	0,99	0,90	---
266149_x_at	At2g12250	0,78	1,01	1,08	1,12	1,15	0,99	0,96	1,00	---
266150_s_at	At2g12290	1,19	1,33	1,09	0,91	0,87	1,31	1,01	0,97	hypothetical protein
266151_x_at	At2g12300	0,93	1,07	0,93	1,04	0,98	0,96	1,05	1,01	---
266153_at	At2g12320	1,02	0,99	0,88	0,95	1,15	0,86	0,94	0,97	hypothetical protein
263512_at	At2g12350	0,97	1,03	1,06	1,08	1,03	1,00	1,07	0,99	---
263513_at	At2g12400	0,67	0,58	0,55	1,16	1,19	1,02	1,17	1,28	expressed protein
263511_at	At2g12420	1,09	1,01	0,96	1,01	1,15	0,92	0,99	0,95	CACTA-like transposase family (Ptta/En/Spm)
263306_at	At2g12480	0,96	1,21	1,13	1,04	0,85	1,04	1,02	0,94	serine carboxypeptidase S10 family protein
263307_at	At2g12520	1,00	1,13	1,01	1,03	0,90	1,03	0,95	1,01	hypothetical protein
257444_at	At2g12550	1,07	1,10	1,08	0,79	1,10	1,07	1,12	0,99	ubiquitin-associated (UBA)/TS-N domain-containing protein
263080_at	At2g12610	0,98	0,92	0,92	0,96	0,92	1,14	1,03	1,07	expressed protein
263056_at	At2g12720	0,99	0,93	0,93	1,06	0,98	1,11	0,98	1,03	---
263965_at	At2g12870	0,96	1,01	1,08	1,04	0,97	1,00	1,00	1,08	---
263964_at	At2g12880	0,95	1,03	1,03	0,99	1,09	1,04	0,99	1,08	zinc knuckle (CCHC-type) family protein
263994_at	At2g12900	0,97	1,00	1,03	1,03	1,35	1,05	1,01	0,94	hypothetical protein
263993_at	At2g12910	0,98	1,12	1,01	1,19	1,09	1,13	0,92	1,06	---
263992_at	At2g12920	1,04	1,06	0,96	0,97	1,13	1,00	1,00	0,98	---
263969_at	At2g12940	0,95	1,04	1,03	1,06	1,12	1,02	1,08	0,91	expressed protein
263968_s_at	At2g12980	0,93	0,99	1,04	1,00	1,01	1,05	0,90	0,89	---
263967_at	At2g12990	1,05	1,05	0,94	0,95	1,05	0,99	1,03	0,91	---
263966_s_at	At2g13000	0,96	0,99	0,99	1,13	1,06	0,86	0,90	0,98	---
263991_at	At2g13020	1,03	1,00	1,07	1,03	1,07	1,03	1,05	0,87	---
265758_at	At2g13070	0,95	0,95	0,97	0,95	1,06	0,90	1,07	0,92	hypothetical protein
265759_at	At2g13100	0,98	1,09	1,11	1,08	0,94	1,01	1,04	1,07	glycerol-3-phosphate transporter, putative / glycerol 3-phosphate permease
265756_at	At2g13150	1,00	1,01	0,98	0,93	0,98	1,18	1,01	0,91	expressed protein
265757_x_at	At2g13160	0,90	1,06	0,92	0,90	1,10	0,85	0,97	1,11	---
265362_at	At2g13210	0,90	0,99	1,00	0,95	1,05	1,01	1,02	0,90	---
265361_at	At2g13230	1,09	0,96	1,07	1,08	1,10	1,01	0,95	0,93	---
265360_s_at	At2g13250	0,94	1,04	1,12	0,95	1,09	1,00	1,00	1,17	---
265367_at	At2g13270	0,96	1,00	1,06	0,98	0,99	0,97	0,97	1,11	hypothetical protein
265366_at	At2g13290	0,82	0,74	0,77	1,28	1,07	0,91	0,73	0,83	glycosyl transferase family 17 protein
265365_at	At2g13310	1,01	1,00	1,04	1,00	1,01	1,05	1,02	1,03	---
265364_at	At2g13330	0,99	1,06	0,93	1,13	1,04	1,04	0,98	1,08	---
265363_at	At2g13335	1,00	1,02	0,95	1,00	1,10	1,01	0,99	0,72	---
265368_at	At2g13350	0,89	1,10	1,03	0,95	1,03	1,00	0,86	0,79	C2 domain-containing protein
263350_at	At2g13360	0,98	1,05	1,18	0,83	0,87	1,07	1,09	1,09	serine-glyoxylate aminotransferase-related
263349_at	At2g13370	0,96	1,17	1,56	0,77	0,82	1,11	1,46	1,34	chromodomain-helicase-DNA-binding family protein / CHD family protein
263052_at	At2g13430	0,95	0,97	0,96	0,91	1,15	1,05	1,00	0,95	hypothetical protein
263053_at	At2g13440	0,89	0,64	0,57	0,73	1,00	0,98	1,07	0,95	glucose-inhibited division family A protein
263723_at	At2g13500	1,01	0,98	0,94	0,95	1,03	1,04	0,88	1,02	hypothetical protein
263724_at	At2g13510	0,96	1,08	0,79	1,09	1,19	0,96	0,82	0,78	hypothetical protein
263727_at	At2g13540	1,21	1,11	1,14	0,85	0,82	1,03	1,03	1,06	mRNA cap-binding protein (ABH1)
263725_at	At2g13550	0,99	1,02	1,02	0,90	1,09	1,08	0,98	0,96	expressed protein
263717_at	At2g13560	0,97	0,84	0,76	1,03	1,07	0,97	0,90	0,83	malate oxidoreductase, putative
263718_at	At2g13570	0,96	0,91	0,97	0,98	0,89	1,03	0,86	0,90	CCAAT-box binding transcription factor, putative

263719_at	At2g13600	1,00	1,07	0,95	0,96	1,04	1,08	0,94	0,99	pentatricopeptide (PPR) repeat-containing protein
263726_at	At2g13610	0,97	0,91	0,87	1,03	1,11	0,62	0,61	0,68	ABC transporter family protein
263720_at	At2g13620	0,93	0,98	0,85	1,08	0,94	1,09	0,90	0,96	cation/hydrogen exchanger, putative (CHX15)
263721_at	At2g13630	0,91	1,06	0,95	1,09	0,98	0,98	1,05	1,19	F-box family protein-related
263722_at	At2g13650	0,90	0,83	0,90	1,13	0,91	0,91	0,87	0,99	GDP-mannose transporter (GONST1)
264103_at	At2g13660	1,02	1,00	1,03	0,95	1,06	1,03	1,04	0,97	expressed protein
264108_at	At2g13670	1,08	1,00	1,00	1,00	0,96	1,01	0,91	0,97	glycosyl transferase family 48 protein
264112_at	At2g13675	0,90	1,11	0,96	0,86	1,16	1,01	0,99	0,89	glycosyl transferase family 48 protein
264111_at	At2g13690	0,87	0,94	1,14	0,69	0,96	0,89	0,88	0,93	PRLI-interacting factor, putative
264109_at	At2g13720	0,98	0,95	0,94	0,98	1,07	0,88	1,06	0,97	hypothetical protein
264110_at	At2g13730	0,96	0,94	0,96	0,94	1,05	0,99	1,09	0,96	hypothetical protein
264104_at	At2g13750	1,02	0,97	0,95	0,94	1,11	1,12	1,12	0,92	CACTA-like transposase family (Ptta/En/Spm)
264105_x_at	At2g13760	1,07	1,06	1,05	0,98	1,11	1,04	1,00	0,93	hypothetical protein
265018_s_at	At2g13770	0,97	0,89	0,99	0,91	1,02	0,96	0,95	1,00	hypothetical protein
264106_at	At2g13780	0,92	0,82	0,87	0,95	1,00	1,05	1,07	1,01	---
264107_s_at	At2g13790	0,63	0,65	0,84	0,91	0,99	1,02	1,10	1,23	leucine-rich repeat family protein / protein kinase family protein
265658_at	At2g13810	1,10	1,23	1,21	1,50	1,09	1,31	1,08	1,26	aminotransferase class I and II family protein
265656_at	At2g13820	1,45	1,21	0,95	2,57	2,19	0,83	0,79	0,79	protease inhibitor/seed storage/lipid transfer protein (LTP) family protein
265657_at	At2g13840	1,00	1,03	1,12	0,91	1,31	0,97	0,87	0,97	PHP domain-containing protein
265654_s_at	At2g13870	0,94	1,00	1,05	1,04	1,15	1,06	1,06	0,98	---
265653_at	At2g13880	1,01	1,04	1,01	1,05	1,11	1,01	0,93	0,92	---
265652_x_at	At2g13890	1,00	0,95	0,98	1,09	1,38	1,01	0,96	0,76	pseudogene, zinc knuckle (CCHC type) protein family
265651_at	At2g13900	0,97	0,97	1,01	0,96	1,17	0,99	1,03	1,01	DC1 domain-containing protein
265655_at	At2g13920	0,93	0,99	0,87	1,02	0,92	1,00	1,09	0,93	---
265298_s_at	At2g13930	0,97	0,99	0,96	0,99	0,97	0,85	1,05	0,82	---
265299_at	At2g13940	0,86	1,02	0,96	1,00	0,95	1,12	1,15	0,94	---
265300_at	At2g13950	0,97	0,92	1,09	1,00	0,93	1,03	1,14	1,02	DC1 domain-containing protein
265301_s_at	At2g13960	0,95	0,99	0,93	0,91	1,05	1,02	1,29	1,01	myb family transcription factor (MYB3R5)
265302_at	At2g13970	0,97	1,06	1,12	1,02	1,20	1,14	0,92	1,09	---
265292_s_at	At2g14010	0,91	1,02	0,96	0,99	1,06	0,89	0,96	0,99	hypothetical protein
265293_at	At2g14020	0,92	1,01	1,05	1,00	1,10	0,99	1,03	0,95	hypothetical protein
265294_at	At2g14030	1,02	1,12	1,07	1,01	1,11	1,00	1,14	0,92	---
265303_at	At2g14040	0,93	1,03	0,89	0,91	1,06	0,94	1,01	0,97	---
265295_at	At2g14050	1,12	1,06	1,11	0,89	1,14	1,07	0,93	0,85	minichromosome maintenance family protein / MCM family protein
265296_at	At2g14060	1,07	1,03	1,13	0,85	1,03	0,97	0,96	1,03	S-adenosyl-L-methionine:carboxyl methyltransferase family protein
265297_at	At2g14080	0,86	1,19	0,86	0,57	0,40	1,57	1,23	1,14	disease resistance protein (TIR-NBS-LRR class), putative
263282_at	At2g14095	0,95	1,04	1,13	1,07	1,04	1,33	1,15	1,06	expressed protein
263276_at	At2g14100	0,94	0,86	0,70	1,18	1,11	0,97	1,06	1,04	cytochrome P450 family protein
263277_at	At2g14110	1,01	1,07	1,03	0,84	0,98	1,11	0,93	0,99	expressed protein
263278_at	At2g14120	0,93	0,86	0,89	0,85	1,07	1,04	0,94	1,20	dynamin-like protein 2b (ADL2b)
263279_x_at	At2g14130	1,00	1,04	0,97	1,00	0,97	1,07	1,03	0,86	Ulp1 protease family protein
263280_x_at	At2g14140	0,98	1,14	0,99	0,98	0,98	1,05	1,05	1,18	hypothetical protein
263281_at	At2g14160	1,04	1,04	0,97	1,01	0,85	0,99	1,12	1,35	RNA recognition motif (RRM)-containing protein
263275_at	At2g14170	0,97	0,98	1,18	1,09	0,98	0,95	1,03	1,07	methylmalonate-semialdehyde dehydrogenase, putative
263295_at	At2g14210	1,01	1,00	0,97	1,03	1,10	0,98	0,98	0,93	MADS-box protein (ANR1)
265600_at	At2g14230	1,03	0,97	0,98	1,13	1,02	1,02	1,08	1,08	---

265599_at	At2g14250	0,80	1,13	0,93	0,83	0,98	1,18	1,40	1,54	ankyrin repeat family protein
265575_at	At2g14260	0,98	0,95	0,98	0,80	0,94	1,09	1,12	1,25	proline iminopeptidase
265598_at	At2g14270	0,91	0,97	0,99	0,98	1,09	1,05	0,96	0,99	pseudogene, protein phosphatase 2C
265632_at	At2g14290	0,94	1,06	0,94	1,24	1,09	0,97	0,98	0,94	F-box family protein
265631_at	At2g14300	0,98	1,01	0,96	0,94	0,96	0,95	0,96	0,90	pseudogene, similar to putative helicase
265574_at	At2g14330	0,95	0,98	1,02	1,02	0,86	1,11	0,99	1,03	hypothetical protein
265604_s_at	At2g14350	0,99	1,02	0,91	0,99	1,19	1,07	0,99	0,85	expressed protein
265603_at	At2g14360	1,01	1,01	0,98	0,95	1,14	0,98	0,94	1,21	protein coding
265602_at	At2g14380	1,05	0,86	0,94	1,02	1,07	0,96	0,96	0,94	---
265601_at	At2g14390	1,13	1,04	1,10	0,91	0,93	0,97	1,17	1,22	hypothetical protein
265835_at	At2g14400	1,02	1,00	0,88	0,94	0,88	0,98	1,01	1,11	---
265834_at	At2g14410	1,01	1,02	0,97	0,99	0,93	0,99	0,97	0,81	---
265833_at	At2g14420	0,93	1,11	1,07	0,95	0,91	1,13	1,04	0,69	---
257388_at	At2g14430	1,01	1,01	0,87	0,93	1,03	1,00	0,95	0,74	---
265832_at	At2g14450	0,87	1,16	1,10	0,94	0,96	0,92	1,01	1,03	hypothetical protein
265831_at	At2g14460	0,96	0,98	0,99	1,12	1,34	1,00	1,00	1,05	expressed protein
265830_at	At2g14470	0,98	1,02	0,96	1,03	1,14	0,98	0,99	1,00	pseudogene, similar to putative helicase
265829_at	At2g14490	0,94	0,98	0,86	1,00	0,97	0,94	0,99	0,94	---
257387_s_at	At2g14510	1,00	1,04	1,04	0,87	0,91	1,08	1,05	1,12	leucine-rich repeat protein kinase, putative
265828_at	At2g14520	0,95	0,84	0,98	0,82	1,04	1,03	0,93	0,77	CBS domain-containing protein
265840_at	At2g14530	0,66	0,68	0,67	0,91	0,95	0,82	0,86	0,85	expressed protein
265839_at	At2g14540	1,08	0,91	1,13	0,93	1,17	1,03	0,98	0,98	serpin family protein / serine protease inhibitor family protein
265838_at	At2g14550	0,99	1,10	1,02	0,95	1,04	0,88	1,01	1,25	---
265837_at	At2g14560	0,46	0,51	1,04	1,03	1,10	3,34	2,09	1,10	expressed protein
265836_at	At2g14570	1,02	0,98	1,08	1,14	1,11	1,03	0,88	0,89	SWIM zinc finger family protein
266383_at	At2g14580	0,86	0,85	0,74	1,09	1,43	1,20	1,01	1,06	pathogenesis-related protein, putative
266377_at	At2g14590	1,01	0,95	1,10	1,00	1,09	1,01	0,93	0,98	hypothetical protein
266382_at	At2g14595	0,99	1,03	1,08	0,89	0,95	1,06	0,82	1,01	---
266385_at	At2g14610	0,11	0,27	0,34	1,68	1,25	14,85	8,47	24,67	pathogenesis-related protein 1 (PR-1)
266376_at	At2g14620	0,97	1,04	0,83	1,07	1,03	0,85	0,95	1,13	xyloglucan:xyloglucosyl transferase, putative / xyloglucan endotransglycosy
266375_at	At2g14630	0,94	1,05	1,04	1,04	1,03	1,08	1,26	1,00	hypothetical protein
266374_at	At2g14640	1,04	0,96	1,06	1,10	0,97	1,08	0,93	1,01	---
266373_x_at	At2g14650	1,09	0,99	0,89	0,92	1,21	0,96	1,03	1,45	gypsy-like retrotransposon family
266384_at	At2g14660	0,99	1,01	1,09	0,85	0,98	0,98	1,05	0,77	expressed protein
266381_at	At2g14670	0,93	1,01	1,06	1,13	1,00	0,95	1,05	0,98	sucrose transporter, putative / sucrose-proton symporter, putative
266380_at	At2g14680	1,06	1,05	1,28	1,03	1,08	1,12	1,58	1,44	myosin heavy chain-related
266379_at	At2g14690	1,02	0,98	1,02	0,92	1,09	1,02	1,02	1,00	glycosyl hydrolase family 10 protein
266378_at	At2g14700	0,93	0,96	1,08	0,93	1,06	0,96	0,85	1,01	hypothetical protein
267107_at	At2g14730	1,03	1,04	1,05	0,98	1,23	1,08	0,98	0,87	hypothetical protein
267106_s_at	At2g14740	0,99	0,93	0,95	1,33	1,36	1,07	0,91	0,95	vacuolar sorting receptor, putative
267112_at	At2g14750	1,07	1,10	1,48	1,27	1,12	0,93	0,96	0,85	adenylylsulfate kinase 1 (AKN1)
267105_at	At2g14760	1,01	1,07	1,05	0,91	1,13	1,01	1,02	1,07	basic helix-loop-helix protein / bHLH protein
267111_s_at	At2g14770	0,98	1,01	0,89	1,12	1,06	0,88	0,94	0,88	hypothetical protein
267110_at	At2g14800	1,00	1,05	1,03	0,83	0,89	1,12	0,89	0,89	hypothetical protein
267109_at	At2g14810	0,98	0,99	0,91	0,92	0,97	1,13	1,00	1,14	hypothetical protein
267108_at	At2g14830	0,92	1,01	1,07	0,98	1,08	1,00	0,96	0,87	expressed protein

267114_at	At2g14835	0,92	0,88	0,82	1,00	1,04	0,98	0,80	0,88	zinc finger (C3HC4-type RING finger) family protein
267113_at	At2g14850	0,85	0,95	1,07	1,06	0,85	1,01	1,08	1,01	expressed protein
266612_at	At2g14860	1,04	1,12	1,23	1,01	0,96	1,05	0,73	0,89	peroxisomal membrane protein 22 kDa, putative
266587_at	At2g14880	1,16	0,99	0,93	0,99	1,15	1,07	0,97	0,98	SWIB complex BAF60b domain-containing protein
266588_at	At2g14890	1,00	1,00	0,88	1,08	1,02	0,84	0,82	0,85	arabinogalactan-protein (AGP9)
266613_at	At2g14900	1,29	1,08	1,01	2,43	2,51	0,74	0,62	0,59	gibberellin-regulated family protein
266614_at	At2g14910	0,89	0,98	0,97	0,66	0,68	1,02	1,04	1,16	expressed protein
266584_s_at	At2g14920	0,94	0,96	1,02	1,13	0,90	0,96	1,15	1,13	sulfotransferase family protein
266585_at	At2g14930	0,93	1,03	0,96	1,03	1,10	0,92	0,97	1,05	---
266610_at	At2g14950	1,02	0,98	0,97	1,10	1,03	0,96	0,93	0,95	---
266611_at	At2g14960	0,89	1,01	0,90	1,03	1,03	0,90	1,02	1,05	auxin-responsive GH3 family protein
265890_s_at	At2g14980	0,91	0,97	1,03	1,00	0,94	0,98	0,93	1,16	---
265895_at	At2g15000	1,82	1,67	1,27	1,30	1,00	0,96	1,01	0,75	expressed protein
265891_at	At2g15010	0,43	0,42	0,16	0,43	0,38	1,11	0,99	1,03	thionin, putative
265892_at	At2g15020	1,04	1,11	0,98	0,89	0,94	1,15	1,15	1,08	expressed protein
265893_at	At2g15040	1,00	0,85	0,90	0,93	0,95	0,98	0,98	1,23	pseudogene, disease resistance protein-related
265894_at	At2g15050	0,90	1,03	0,97	0,84	0,64	0,87	0,84	0,97	lipid transfer protein, putative
265914_at	At2g15060	0,90	1,06	1,17	0,98	0,89	1,20	0,97	0,97	---
265915_at	At2g15070	1,09	0,93	0,92	1,05	1,03	0,84	1,10	0,99	---
265917_at	At2g15080	2,25	1,85	1,73	1,28	1,54	2,66	1,39	1,47	disease resistance family protein
265918_at	At2g15090	0,86	0,87	1,10	0,78	0,91	0,82	0,59	0,63	fatty acid elongase, putative
265919_at	At2g15100	1,02	1,03	1,01	1,05	1,04	1,02	0,96	1,02	---
257384_at	At2g15110	0,96	1,00	1,01	0,83	1,00	1,13	0,95	0,80	---
265920_s_at	At2g15120	0,90	1,27	1,02	1,05	0,77	1,46	1,67	1,38	pseudogene, disease-resistance family protein / fatty acid elongase-related
265889_at	At2g15130	1,00	0,88	0,92	1,02	0,99	1,08	0,95	0,92	plant basic secretory protein (BSP) family protein
263358_x_at	At2g15140	0,87	1,01	0,96	0,97	1,10	0,98	0,94	0,98	Ulp1 protease family protein
263357_s_at	At2g15150	1,06	1,01	0,97	0,96	1,00	0,91	1,03	0,94	---
263302_at	At2g15190	0,99	0,99	1,05	0,98	0,99	1,08	1,00	0,98	pseudogene, Ulp1 protease family
263301_x_at	At2g15200	1,00	0,94	0,99	1,12	0,95	1,03	1,07	0,92	hypothetical protein
263359_at	At2g15230	0,87	1,04	1,06	1,02	1,00	1,20	1,11	1,34	lipase family protein
263303_at	At2g15240	0,95	0,97	0,72	1,18	0,89	0,97	0,86	0,83	UNC-50 family protein
263329_at	At2g15260	1,07	1,05	0,87	1,03	0,95	0,97	0,92	1,09	zinc finger (C3HC4-type RING finger) family protein
263331_at	At2g15270	1,31	1,23	1,40	0,98	1,03	1,20	1,27	1,32	expressed protein
263328_at	At2g15280	1,04	0,98	1,21	1,08	0,83	0,95	0,87	1,12	reticulon family protein (RTNLB10)
263298_at	At2g15290	1,07	1,03	0,83	1,22	0,93	0,93	0,95	1,02	expressed protein
263327_at	At2g15300	1,03	0,94	0,93	0,99	1,00	1,08	1,05	0,98	leucine-rich repeat transmembrane protein kinase, putative
263297_at	At2g15310	0,84	1,02	1,32	1,44	1,79	1,01	1,18	1,46	ADP-ribosylation factor, putative
263330_at	At2g15320	0,65	0,62	0,62	1,40	1,50	0,94	0,73	0,67	leucine-rich repeat family protein
257438_at	At2g15325	0,99	1,06	0,98	1,00	0,96	1,08	0,90	0,84	protease inhibitor/seed storage/lipid transfer protein (LTP) family protein
263566_at	At2g15340	0,87	1,04	1,04	0,89	0,91	0,80	0,90	0,93	glycine-rich protein
263561_at	At2g15360	0,94	1,02	0,95	0,99	1,00	0,98	1,01	1,08	hypothetical protein
263560_s_at	At2g15370	1,06	1,11	1,01	0,96	1,04	0,92	1,05	1,10	xyloglucan fucosyltransferase, putative (FUT10)
263559_at	At2g15380	0,97	1,01	1,05	0,98	1,14	0,88	0,89	0,93	---
263565_at	At2g15390	0,92	0,83	0,70	1,12	1,08	1,01	0,90	0,90	xyloglucan fucosyltransferase, putative (FUT4)
263564_s_at	At2g15400	1,26	1,16	1,25	1,02	1,22	1,03	1,01	1,09	DNA-directed RNA polymerase II 36 kDa polypeptide B / RNA polymerase
263563_at	At2g15410	1,02	1,00	0,93	1,08	1,07	1,19	0,94	1,01	---

263562_at	At2g15420	0,93	1,22	1,05	1,01	1,14	1,03	0,95	0,87 myosin heavy chain-related
263567_at	At2g15440	0,88	0,90	0,94	0,94	0,92	0,87	0,90	1,12 expressed protein
265490_s_at	At2g15470	1,02	0,95	0,96	1,01	1,02	0,97	0,96	1,12 glycoside hydrolase family 28 protein / polygalacturonase (pectinase) famil
265499_at	At2g15480	0,66	0,65	1,04	0,66	0,77	1,00	0,89	1,28 UDP-glucoronosyl/UDP-glucosyl transferase family protein
265501_at	At2g15490	1,02	0,94	1,10	0,72	0,65	1,24	1,23	1,00 UDP-glucoronosyl/UDP-glucosyl transferase family protein
265502_at	At2g15500	1,01	1,05	1,01	0,94	1,13	1,00	1,04	0,82 hypothetical protein
265503_at	At2g15510	0,97	1,07	1,00	1,11	1,08	0,99	0,95	0,91 ---
265504_at	At2g15520	0,96	1,06	1,07	1,03	1,01	0,96	0,97	0,97 zinc finger protein, putative
265505_at	At2g15530	0,95	1,08	0,97	1,02	0,97	1,00	0,84	0,96 zinc finger (C3HC4-type RING finger) family protein
265473_at	At2g15535	1,03	0,99	1,03	0,99	0,97	1,00	1,02	1,02 SLR1 binding pollen coat protein-related
265506_at	At2g15540	0,96	1,06	1,03	1,09	1,03	1,01	0,88	0,91 ---
265485_at	At2g15550	0,96	0,91	0,89	1,02	1,12	0,89	1,00	0,94 hypothetical protein
265486_at	At2g15560	3,75	3,28	3,60	1,01	0,86	0,95	1,01	1,06 expressed protein
265476_at	At2g15570	0,84	0,97	1,09	0,98	1,02	0,96	1,00	1,09 thioredoxin M-type 3, chloroplast (TRX-M3)
265472_at	At2g15580	1,07	1,49	1,72	0,97	0,98	0,98	1,07	1,39 zinc finger (C3HC4-type RING finger) family protein
265487_at	At2g15600	0,99	1,00	1,04	1,01	0,98	1,04	1,03	0,97 hypothetical protein
265488_at	At2g15610	1,01	1,12	1,05	0,90	1,11	1,01	1,01	1,02 expressed protein
265475_at	At2g15620	0,47	0,46	0,63	0,96	0,93	0,57	0,60	0,51 ferredoxin--nitrite reductase, putative
257395_at	At2g15630	1,13	0,89	0,91	0,80	1,01	1,00	0,98	0,93 pentatricopeptide (PPR) repeat-containing protein
265489_at	At2g15640	0,95	1,12	0,98	1,01	0,95	0,96	0,96	1,29 F-box family protein
265491_s_at	At2g15650	1,03	0,99	1,14	0,92	1,01	1,07	1,15	1,05 ---
265492_at	At2g15660	0,98	0,92	1,04	0,97	1,16	1,01	0,99	0,99 hypothetical protein
265493_at	At2g15670	1,00	0,98	0,98	0,92	1,27	0,99	1,04	0,89 hypothetical protein
265494_at	At2g15680	1,40	1,42	1,07	0,94	1,04	0,82	0,84	0,89 calmodulin-related protein, putative
265474_at	At2g15690	1,30	1,31	1,34	1,10	1,23	0,98	0,90	0,90 pentatricopeptide (PPR) repeat-containing protein
265495_at	At2g15695	1,11	1,23	1,26	0,61	0,90	1,03	1,11	1,43 expressed protein
265496_at	At2g15700	1,01	0,96	1,01	1,11	1,07	0,98	1,02	0,93 ---
265497_at	At2g15720	0,97	1,09	1,00	0,86	1,14	1,04	1,01	1,00 ---
265498_at	At2g15740	1,01	1,04	0,83	1,07	1,00	1,05	0,96	0,97 zinc finger (C2H2 type) family protein
265500_at	At2g15750	0,97	1,09	0,93	1,04	1,10	1,01	1,10	0,82 hypothetical protein
265479_at	At2g15760	0,92	1,12	0,99	1,40	1,01	0,92	0,99	1,13 calmodulin-binding protein
265482_at	At2g15780	0,98	1,16	0,88	1,09	1,19	1,14	1,05	1,24 glycine-rich protein
265483_at	At2g15790	1,23	1,10	0,80	1,07	1,03	1,02	1,11	0,96 peptidyl-prolyl cis-trans isomerase / cyclophilin-40 (CYP40) / rotamase
265541_at	At2g15800	1,02	0,97	1,06	0,90	1,08	1,17	1,12	1,00 expressed protein
265540_at	At2g15810	1,01	1,01	0,96	1,05	1,18	1,11	1,05	0,91 ---
265484_at	At2g15820	1,02	0,81	1,00	0,89	0,94	1,06	1,12	1,08 pentatricopeptide (PPR) repeat-containing protein
265539_at	At2g15830	0,67	0,97	0,95	0,71	0,97	0,80	0,84	0,91 expressed protein
265538_at	At2g15860	1,15	1,22	1,46	1,18	1,21	1,08	1,10	0,96 expressed protein
265537_at	At2g15870	0,97	1,01	1,12	0,87	1,25	1,01	1,01	1,09 ---
265536_at	At2g15880	1,32	1,26	1,10	0,43	0,55	1,26	1,18	1,09 leucine-rich repeat family protein / extensin family protein
265478_at	At2g15890	0,76	0,87	1,18	0,73	0,66	0,89	0,84	1,08 expressed protein
265535_at	At2g15900	0,72	0,84	1,04	0,71	0,79	0,97	0,79	1,22 phox (PX) domain-containing protein
265509_at	At2g15920	0,93	1,09	0,97	0,94	0,97	0,95	0,91	0,97 ---
265508_at	At2g15930	1,02	1,03	0,98	0,94	0,95	1,06	0,99	1,10 hypothetical protein
265507_s_at	At2g15940	2,72	2,29	1,72	1,05	1,01	1,03	0,87	1,12 ---
265481_at	At2g15960	0,82	0,93	1,01	0,91	0,97	0,92	1,05	1,24 expressed protein

265480_at	At2g15970	0,77	0,88	1,01	0,94	0,98	0,89	0,78	0,72	cold-acclimation protein, putative (FL3-5A3)
263089_at	At2g16000	1,12	1,05	0,87	0,98	1,04	1,05	0,97	0,87	---
263098_at	At2g16005	0,79	0,81	0,60	3,71	2,17	0,67	0,65	0,80	MD-2-related lipid recognition domain-containing protein / ML domain-conta
263093_at	At2g16010	0,97	1,04	0,89	0,96	0,98	0,96	1,08	1,18	hypothetical protein
263094_at	At2g16020	0,94	1,02	1,08	0,96	1,06	1,09	1,05	1,17	hypothetical protein
263095_at	At2g16040	0,96	1,03	1,02	0,92	1,01	1,00	1,05	1,05	hAT dimerisation domain-containing protein / transposase-related
263096_at	At2g16060	0,87	0,89	0,97	2,27	3,04	0,86	0,52	0,63	non-symbiotic hemoglobin 1 (HB1) (GLB1)
263097_at	At2g16070	1,14	1,25	1,31	1,23	1,20	1,03	0,96	1,12	expressed protein
263085_at	At2g16090	1,04	1,23	1,44	0,96	0,98	1,08	0,91	0,97	zinc finger protein-related
257455_s_at	At2g16100	1,04	0,93	0,97	0,92	1,08	1,03	0,97	0,98	hypothetical protein
263086_at	At2g16110	0,96	1,06	1,03	1,09	0,93	0,92	0,82	0,97	---
263087_s_at	At2g16120	0,93	0,92	1,13	1,03	1,08	1,08	1,06	1,05	mannitol transporter, putative
263088_at	At2g16160	0,98	0,96	0,96	0,94	1,03	1,04	1,02	1,04	hypothetical protein
263090_at	At2g16190	0,98	0,94	1,07	0,96	1,10	1,01	1,05	1,19	hypothetical protein
263091_at	At2g16200	1,07	0,97	1,06	1,04	1,11	1,02	1,01	1,02	hypothetical protein
263092_at	At2g16210	1,00	1,00	0,93	1,00	1,11	0,99	0,97	0,87	transcriptional factor B3 family protein
263610_at	At2g16230	0,96	1,03	1,11	1,03	1,00	0,95	0,96	0,84	glycosyl hydrolase family 17 protein
263609_at	At2g16250	0,94	0,84	0,95	1,04	0,93	0,96	1,06	1,07	leucine-rich repeat transmembrane protein kinase, putative
263608_at	At2g16260	0,89	0,99	0,99	1,00	0,99	0,96	1,03	1,07	---
263607_at	At2g16270	1,28	1,15	1,25	1,05	1,10	1,00	0,97	0,78	expressed protein
263606_at	At2g16280	1,23	0,95	0,84	0,89	0,79	0,94	0,89	0,97	very-long-chain fatty acid condensing enzyme, putative
257437_s_at	At2g16290	0,97	1,04	1,13	1,09	1,16	0,99	0,95	0,90	F-box family protein
263604_s_at	At2g16330	0,97	1,05	1,13	1,12	1,20	0,87	0,94	0,95	hypothetical protein
263603_at	At2g16340	0,95	1,14	1,03	0,90	0,99	0,89	1,18	0,92	hypothetical protein
263556_at	At2g16350	1,03	1,21	2,03	0,80	0,94	1,20	1,25	1,58	F-box family protein
263602_at	At2g16360	1,01	1,03	1,01	1,03	1,10	0,96	1,11	0,87	40S ribosomal protein S25 (RPS25A)
263601_s_at	At2g16370	1,14	1,09	1,23	0,84	0,71	0,99	1,06	1,15	bifunctional dihydrofolate reductase-thymidylate synthase 1 / DHFR-TS (TH
263558_at	At2g16380	0,75	0,80	0,77	0,65	0,85	0,97	1,09	1,21	SEC14 cytosolic factor family protein / phosphoglyceride transfer family pro
263600_at	At2g16390	0,69	0,95	0,89	0,81	0,97	0,91	0,84	0,99	SNF2 domain-containing protein / helicase domain-containing protein
263557_at	At2g16400	1,48	1,90	1,59	0,91	0,62	1,41	1,32	1,25	homeodomain-containing protein
263554_at	At2g16410	1,07	1,13	1,00	1,00	1,21	1,06	1,04	0,79	hypothetical protein
263553_at	At2g16430	1,12	1,02	0,98	1,41	1,18	1,06	1,03	0,96	purple acid phosphatase (PAP10)
263612_at	At2g16440	1,93	1,79	1,84	1,51	1,27	1,17	1,07	1,02	DNA replication licensing factor, putative
263611_at	At2g16450	1,07	0,98	0,89	0,99	0,97	0,99	1,07	1,15	F-box family protein
263555_at	At2g16460	1,43	1,36	1,79	1,58	2,32	0,88	0,92	1,03	expressed protein
263605_at	At2g16480	0,98	0,89	0,92	1,01	1,12	0,91	1,17	1,12	SWIB complex BAF60b domain-containing protein / plus-3 domain-containi
263241_at	At2g16500	1,06	0,90	0,80	1,01	1,07	0,86	0,97	1,01	arginine decarboxylase 1 (SPE1) (ARGDC)
263267_at	At2g16510	0,86	0,86	0,85	0,92	0,97	0,98	0,90	0,92	vacuolar ATP synthase 16 kDa proteolipid subunit 5 / V-ATPase 16 kDa pro
263266_at	At2g16520	0,96	0,96	0,97	0,93	1,03	0,98	0,96	0,98	hypothetical protein
263240_s_at	At2g16530	0,84	1,00	0,84	0,98	1,00	0,91	0,98	1,34	3-oxo-5-alpha-steroid 4-dehydrogenase family protein / steroid 5-alpha-redu
263239_at	At2g16570	1,06	1,07	0,98	1,41	0,89	0,91	1,12	1,12	amidophosphoribosyltransferase / glutamine phosphoribosylpyrophosphate
263238_at	At2g16580	1,00	1,02	1,03	0,98	1,07	0,96	0,96	0,90	auxin-responsive protein, putative
263268_at	At2g16590	0,99	1,03	1,11	0,97	1,28	0,98	0,81	0,85	---
265356_at	At2g16595	0,80	0,73	0,66	1,18	1,01	0,97	0,72	0,91	translocon-associated protein (TRAP), putative
265352_at	At2g16600	0,88	0,88	0,99	1,27	1,51	0,88	0,87	0,85	peptidyl-prolyl cis-trans isomerase, cytosolic / cyclophilin / rotamase (ROC3
265406_at	At2g16610	1,03	0,98	1,01	0,95	1,00	0,98	0,92	0,97	---

265410_at	At2g16620	0,91	0,97	0,98	0,92	1,01	1,06	1,02	0,94	protein kinase-related
265411_at	At2g16630	0,79	0,77	0,80	1,26	1,22	0,74	0,66	0,74	proline-rich family protein
265412_at	At2g16640	0,93	1,06	1,61	0,81	0,85	0,98	1,09	0,99	chloroplast outer membrane protein, putative
265413_s_at	At2g16650	0,80	0,88	1,05	0,62	0,69	0,88	1,09	0,84	expressed protein
265414_at	At2g16660	0,31	0,41	0,50	0,62	0,59	1,26	1,47	1,75	nodulin family protein
265380_at	At2g16670	0,98	0,96	0,87	0,91	0,96	0,98	1,00	0,90	---
265381_at	At2g16680	0,75	1,06	1,16	0,97	1,10	1,00	0,92	0,89	---
265403_at	At2g16690	0,98	1,06	0,99	0,91	1,13	0,97	1,07	0,92	hypothetical protein
265354_at	At2g16700	0,65	0,84	1,07	1,08	1,16	0,98	1,16	1,31	actin-depolymerizing factor 5 (ADF5)
265358_at	At2g16710	1,08	0,82	0,84	0,94	0,83	0,91	0,84	0,79	hesB-like domain-containing protein
265359_at	At2g16720	1,19	1,04	0,90	1,01	1,12	1,15	1,26	0,94	myb family transcription factor
265404_at	At2g16730	0,85	0,92	0,92	0,91	0,94	0,89	1,01	1,04	glycosyl hydrolase family 35 protein
265357_at	At2g16740	1,22	1,35	1,22	1,43	1,37	0,92	0,97	0,94	ubiquitin-conjugating enzyme, putative
265405_at	At2g16750	0,88	0,94	0,98	1,38	1,29	0,91	0,98	1,28	protein kinase family protein
265355_at	At2g16760	1,03	1,08	1,27	1,10	1,21	1,14	0,88	1,09	expressed protein
265383_at	At2g16780	1,37	1,25	1,14	1,13	1,25	1,06	0,95	0,87	WD-40 repeat protein (MSI2)
265382_at	At2g16790	1,50	1,21	1,16	1,46	1,55	1,18	1,40	1,30	shikimate kinase family protein
265353_at	At2g16800	1,10	1,12	1,20	0,92	0,95	0,94	0,94	0,94	high-affinity nickel-transport family protein
265407_at	At2g16810	0,97	1,05	1,03	1,00	1,23	0,92	0,93	0,99	F-box family protein (FBX8)
265408_at	At2g16820	0,95	0,92	1,05	1,05	1,02	1,06	1,00	1,02	hypothetical protein
265409_at	At2g16830	1,01	1,01	0,96	0,92	1,04	1,04	1,00	1,09	pseudogene, hypothetical protein
266531_at	At2g16835	0,97	1,11	1,02	0,92	0,99	0,96	1,05	1,01	water channel protein, putative
266527_at	At2g16840	1,02	1,13	0,94	0,96	0,92	1,03	0,91	1,06	---
266533_s_at	At2g16850	1,19	1,22	1,23	1,05	0,99	0,99	1,02	0,98	plasma membrane intrinsic protein, putative
266537_at	At2g16860	1,27	1,03	1,43	1,03	1,06	1,48	1,43	1,52	GCIP-interacting family protein
266528_at	At2g16870	0,96	1,01	1,02	0,94	1,17	1,04	1,11	1,08	disease resistance protein (TIR-NBS-LRR class), putative
266529_at	At2g16880	0,99	0,91	0,88	0,89	1,05	0,96	0,88	0,97	pentatricopeptide (PPR) repeat-containing protein
266532_at	At2g16890	1,14	1,18	1,13	1,04	0,92	1,14	1,28	1,24	UDP-glucoronosyl/UDP-glucosyl transferase family protein
266536_at	At2g16900	1,08	0,79	0,83	1,08	1,06	1,03	1,13	1,13	expressed protein
266530_at	At2g16910	0,99	1,05	1,18	1,03	1,08	0,88	1,03	0,99	basic helix-loop-helix (bHLH) family protein
266522_at	At2g16920	0,93	1,06	1,26	0,90	1,08	1,11	0,89	0,94	ubiquitin-conjugating enzyme family protein
266535_s_at	At2g16930	0,98	0,89	0,94	1,18	1,23	0,87	0,87	0,85	ribosomal protein L27 family protein
266534_at	At2g16940	1,44	1,37	1,38	0,90	0,83	1,26	1,32	1,23	RNA recognition motif (RRM)-containing protein
266523_at	At2g16950	0,86	0,92	0,98	0,85	0,97	1,00	0,98	0,98	importin beta-2 subunit family protein
266524_at	At2g16960	0,94	0,98	0,95	1,01	1,04	0,98	0,92	0,93	importin beta-2 subunit family protein
266525_at	At2g16970	0,87	0,75	0,74	1,03	1,15	0,82	0,98	1,23	expressed protein
266526_at	At2g16980	0,90	0,96	1,02	0,93	0,99	1,01	1,06	0,94	expressed protein
263574_at	At2g16990	0,79	0,73	0,50	0,97	0,94	0,75	0,84	0,85	expressed protein
257439_at	At2g17000	0,89	1,00	0,90	0,98	1,04	0,91	1,00	0,93	mechanosensitive ion channel domain-containing protein / MS ion channel
263578_at	At2g17020	1,17	1,23	1,03	0,88	1,02	1,09	0,89	0,98	F-box family protein (FBL10)
263579_at	At2g17030	1,39	1,43	1,03	1,23	1,01	0,91	1,19	1,11	F-box family protein
263550_at	At2g17033	0,85	0,87	0,83	0,74	0,74	0,92	0,85	0,78	pentatricopeptide (PPR) repeat-containing protein
263584_at	At2g17040	1,37	1,77	0,97	1,16	1,10	1,23	1,18	0,87	no apical meristem (NAM) family protein
263571_at	At2g17050	0,94	0,98	1,03	0,98	0,92	1,14	1,08	0,87	disease resistance protein (TIR-NBS-LRR class), putative
263572_at	At2g17060	0,95	1,05	1,10	0,86	0,92	0,93	0,99	1,03	disease resistance protein (TIR-NBS-LRR class), putative
263575_at	At2g17070	1,67	1,19	1,10	1,01	1,14	0,99	1,04	1,00	expressed protein

263576_at	At2g17080	1,24	1,03	0,94	1,16	1,05	0,92	0,99	1,02	hypothetical protein
263577_at	At2g17090	0,92	1,01	0,86	1,01	0,96	1,12	1,05	0,92	protein kinase family protein
263551_at	At2g17110	0,87	0,93	0,97	0,69	0,85	0,76	1,06	1,29	expressed protein
263582_at	At2g17120	0,55	0,51	0,47	0,86	0,70	0,93	0,85	0,84	peptidoglycan-binding LysM domain-containing protein
263583_at	At2g17130	0,86	0,83	0,74	1,18	1,01	0,99	0,87	0,84	isocitrate dehydrogenase subunit 2 / NAD+ isocitrate dehydrogenase subur
263580_at	At2g17140	1,05	1,08	1,10	0,97	1,01	1,05	1,10	0,94	pentatricopeptide (PPR) repeat-containing protein
263573_at	At2g17150	0,85	0,78	0,86	0,89	0,76	1,21	1,02	1,13	RWP-RK domain-containing protein
263581_at	At2g17160	0,96	1,01	0,99	0,93	1,03	1,08	0,99	1,01	protein kinase-related
263416_at	At2g17170	0,99	0,94	0,97	0,98	1,07	1,01	1,02	0,96	protein kinase family protein
263417_at	At2g17180	1,00	1,10	1,05	0,95	1,07	1,00	1,00	0,81	zinc finger (C2H2 type) family protein
263422_s_at	At2g17200	1,02	0,97	0,87	1,07	0,93	1,09	0,99	1,01	ubiquitin family protein
263418_at	At2g17210	1,01	0,99	1,00	0,96	0,93	1,04	0,97	1,01	pentatricopeptide (PPR) repeat-containing protein
263419_at	At2g17220	0,75	0,81	1,03	1,02	1,07	0,98	1,11	1,35	protein kinase, putative
263421_at	At2g17230	0,92	0,89	0,75	0,99	1,13	0,83	0,86	0,83	phosphate-responsive 1 family protein
263420_at	At2g17240	1,04	0,85	0,73	0,99	0,99	1,06	0,90	0,91	expressed protein
263415_at	At2g17250	1,12	1,12	1,22	0,83	0,91	1,11	1,01	0,82	expressed protein
264853_at	At2g17260	0,87	0,97	0,90	0,98	1,03	1,03	1,01	0,89	glutamate receptor family protein (GLR3.1) (GLR2)
264855_at	At2g17265	0,85	0,83	0,78	0,96	1,02	0,80	0,72	0,59	homoserine kinase (HSK)
264906_at	At2g17270	0,97	1,05	0,80	1,05	1,05	0,92	0,96	0,88	mitochondrial substrate carrier family protein
264907_at	At2g17280	0,54	0,44	0,57	0,82	0,89	0,93	0,83	0,75	phosphoglycerate/bisphosphoglycerate mutase family protein
264851_at	At2g17290	0,84	0,68	0,71	1,24	1,27	1,06	0,97	0,96	calcium-dependent protein kinase isoform 6 (CPK6)
264909_at	At2g17300	0,67	0,64	0,80	0,74	0,92	0,92	0,91	0,86	expressed protein
257408_at	At2g17310	0,95	1,08	1,00	1,03	1,01	1,01	0,95	1,17	F-box family protein
264877_at	At2g17330	1,00	0,98	1,03	0,89	0,98	0,93	0,99	1,11	pseudogene, obtusifoliol 14-alpha demethylase, putative
264850_at	At2g17340	0,74	0,74	0,76	1,01	1,01	1,08	1,14	1,13	pantothenate kinase-related
264848_at	At2g17350	1,02	1,30	1,53	1,07	1,00	1,02	1,07	1,13	expressed protein
264849_at	At2g17360	1,37	1,12	1,08	1,05	1,11	1,01	0,99	0,89	40S ribosomal protein S4 (RPS4A)
264856_at	At2g17370	0,91	0,85	0,86	1,05	0,94	0,89	0,88	0,76	3-hydroxy-3-methylglutaryl-CoA reductase 2 / HMG-CoA reductase 2 (HMG
264847_at	At2g17380	1,33	0,95	0,99	1,30	1,35	0,92	0,89	0,81	clathrin assembly protein AP19
264875_at	At2g17390	1,10	1,04	1,04	0,99	1,00	0,99	1,01	0,98	ankyrin repeat family protein
264876_at	At2g17400	1,03	1,00	0,99	1,00	0,82	1,09	0,98	1,22	ARID/BRIGHT DNA-binding domain-containing protein
264878_at	At2g17410	1,02	0,98	1,29	0,89	1,01	1,08	1,19	1,43	ARID/BRIGHT DNA-binding domain-containing protein
264904_s_at	At2g17420	1,03	0,99	0,92	1,11	0,97	1,01	1,03	0,93	thioredoxin reductase 2 / NADPH-dependent thioredoxin reductase 2 (NTR:
264905_at	At2g17430	0,95	0,68	1,35	1,14	1,26	0,94	1,06	1,30	seven transmembrane MLO family protein / MLO-like protein 7 (MLO7)
264908_at	At2g17440	0,95	0,93	0,80	0,89	1,11	0,88	0,99	1,16	leucine-rich repeat family protein
264854_at	At2g17450	0,85	0,96	0,94	1,04	1,03	0,75	0,84	1,10	zinc finger (C3HC4-type RING finger) family protein
257409_at	At2g17470	0,84	1,09	1,01	0,94	1,05	0,91	1,01	0,85	expressed protein
264852_at	At2g17480	0,78	0,67	0,84	1,15	1,02	0,97	1,08	1,20	seven transmembrane MLO family protein / MLO-like protein 8 (MLO8)
263071_at	At2g17490	1,02	0,93	0,90	0,93	0,95	0,97	1,00	0,91	---
263073_at	At2g17500	0,67	0,69	0,64	0,88	0,69	0,77	0,86	0,92	auxin efflux carrier family protein
263077_at	At2g17510	0,91	0,99	1,14	0,83	0,76	1,06	0,90	0,95	ribonuclease II family protein
263076_at	At2g17520	0,76	0,72	0,87	1,00	1,06	0,96	0,95	1,22	protein kinase family protein / Ire1 homolog-2 (IRE1-2)
263072_at	At2g17530	1,18	0,94	1,05	1,07	0,97	1,04	1,10	1,17	protein kinase family protein
263066_at	At2g17540	0,94	0,93	1,09	0,96	0,82	0,93	0,95	1,05	expressed protein
263067_at	At2g17550	0,77	0,95	1,15	0,80	0,85	0,94	1,09	1,50	expressed protein
263074_at	At2g17560	1,16	0,89	0,86	1,53	1,41	1,10	0,95	0,93	high mobility group protein gamma (HMGgamma) / HMG protein gamma

263075_at	At2g17570	1,24	1,03	1,23	1,10	0,99	0,91	0,94	0,96 undecaprenyl pyrophosphate synthetase family protein / UPP synthetase fa
263068_at	At2g17580	1,01	0,89	1,03	0,87	0,90	0,90	0,80	1,05 polynucleotide adenylyltransferase family protein
263069_at	At2g17590	1,11	0,97	0,85	1,04	1,36	0,84	1,01	1,05 DC1 domain-containing protein
263070_at	At2g17600	1,00	0,99	1,00	0,98	0,98	0,95	0,90	1,02 DC1 domain-containing protein
263018_at	At2g17610	0,95	1,12	1,03	0,90	1,08	0,95	0,92	0,99 ---
263017_at	At2g17620	1,07	1,12	0,91	1,02	1,16	1,11	1,01	0,76 cyclin, putative (CYC2a)
263047_at	At2g17630	0,96	0,84	0,68	1,10	1,10	0,88	0,83	0,78 phosphoserine aminotransferase, putative
264594_at	At2g17640	1,29	1,54	1,73	0,95	1,09	1,33	1,52	1,60 serine O-acetyltransferase, putative (SAT-106)
264589_at	At2g17650	0,91	0,89	0,93	0,84	0,89	1,04	0,87	1,03 AMP-dependent synthetase and ligase family protein
264617_at	At2g17660	0,86	0,86	0,84	0,89	0,70	1,39	1,62	1,05 nitrate-responsive NOI protein, putative
264591_at	At2g17670	1,17	1,02	1,00	1,07	1,24	0,90	0,87	0,94 pentatricopeptide (PPR) repeat-containing protein
264618_at	At2g17680	0,96	1,02	1,06	1,07	0,94	1,14	0,98	0,84 expressed protein
264621_at	At2g17700	1,23	1,19	1,46	1,12	1,16	0,99	1,06	1,12 protein kinase family protein
264590_at	At2g17710	1,42	1,03	1,07	1,15	0,92	1,04	1,11	1,16 expressed protein
264592_at	At2g17720	0,94	0,73	0,71	1,57	1,35	1,01	0,94	0,97 oxidoreductase, 2OG-Fe(II) oxygenase family protein
264588_at	At2g17730	0,94	1,09	1,04	0,70	0,82	1,00	0,97	1,25 zinc finger (C3HC4-type RING finger) family protein
264616_at	At2g17740	1,23	0,92	1,00	2,66	1,81	0,88	0,72	0,95 DC1 domain-containing protein
257416_at	At2g17750	0,96	0,96	0,91	1,00	1,08	0,95	1,03	0,99 hypothetical protein
264619_at	At2g17760	0,73	0,74	0,61	1,02	1,16	0,96	0,83	0,80 aspartyl protease family protein
264620_at	At2g17770	0,95	0,97	0,95	1,18	1,00	1,06	0,83	0,96 ABA-responsive element binding protein, putative
264622_at	At2g17790	1,09	0,95	0,94	1,18	1,07	1,00	0,97	0,95 vacuolar protein sorting-associated protein 35 family protein / VPS35 family
264593_at	At2g17800	0,96	0,89	0,81	1,10	1,01	0,84	0,91	0,94 Rac-like GTP-binding protein (ARAC1) (ATGP2)
264790_at	At2g17820	1,01	1,28	1,68	0,58	0,76	1,58	1,47	1,83 histidine kinase 1
264784_s_at	At2g17830	0,98	1,07	0,91	1,21	1,13	0,99	1,09	0,87 F-box family protein
264787_at	At2g17840	0,71	0,80	1,23	0,93	0,91	1,11	1,15	1,14 senescence/dehydration-associated protein-related (ERD7)
264791_at	At2g17845	1,01	0,95	1,01	0,93	1,11	1,03	1,07	1,04 short-chain dehydrogenase/reductase (SDR) family protein
264846_at	At2g17850	0,82	1,10	1,71	1,20	1,35	0,68	0,51	0,48 senescence-associated family protein
264812_at	At2g17860	1,02	0,80	0,97	1,08	1,33	1,15	0,94	1,04 pathogenesis-related thaumatin family protein
264789_at	At2g17870	0,95	0,84	0,98	1,03	0,94	0,92	1,05	0,98 cold-shock DNA-binding family protein
264788_at	At2g17880	0,85	1,02	0,93	0,64	0,63	0,78	0,86	0,93 DNAJ heat shock protein, putative
264813_at	At2g17890	1,10	0,96	0,98	0,93	1,00	1,03	1,06	1,01 calcium-dependent protein kinase family protein / CDPK family protein
264814_at	At2g17900	1,16	1,16	1,23	0,87	1,14	1,00	0,96	0,89 zinc finger (MYND type) family protein / SET domain-containing protein
264785_at	At2g17910	1,00	1,09	1,01	0,91	0,98	0,98	1,09	1,04 ---
264786_at	At2g17920	0,97	1,04	0,85	0,97	1,01	0,88	0,99	1,04 expressed protein
265814_at	At2g17930	0,87	0,89	1,02	0,88	0,83	1,02	1,05	1,08 FAT domain-containing protein / phosphatidylinositol 3- and 4-kinase family
265820_at	At2g17940	0,97	1,07	1,07	0,89	1,17	1,04	1,08	1,34 expressed protein
265821_at	At2g17950	0,98	1,02	1,10	1,00	1,07	0,97	0,81	0,94 homeodomain transcription factor (WUSCHEL)
265808_at	At2g17960	0,99	1,08	1,00	0,91	1,06	1,01	1,05	0,79 hypothetical protein
257389_at	At2g17970	1,39	1,46	1,46	0,81	1,00	1,24	1,05	1,11 oxidoreductase, 2OG-Fe(II) oxygenase family protein
265819_at	At2g17972	1,04	1,02	1,17	0,98	0,87	1,24	0,91	0,92 expressed protein
265815_at	At2g17975	1,11	1,20	2,33	0,86	0,73	0,87	1,25	1,60 zinc finger (Ran-binding) family protein
265822_at	At2g17980	0,95	0,81	0,81	1,15	1,27	0,91	0,77	0,90 sec1 family protein
265807_at	At2g17990	1,01	1,28	1,47	1,08	1,30	1,01	1,15	1,24 expressed protein
265806_at	At2g18010	0,97	1,09	1,05	0,97	1,24	0,99	1,00	0,93 auxin-responsive family protein
265805_s_at	At2g18020	1,22	1,12	1,05	1,11	0,97	0,95	0,98	0,88 60S ribosomal protein L8 (RPL8A)
265804_at	At2g18030	0,98	0,85	0,66	1,19	0,95	0,98	0,84	0,76 peptide methionine sulfoxide reductase family protein

265818_at	At2g18040	1,40	1,14	1,15	1,29	1,34	1,18	1,14	1,10	peptidyl-prolyl cis-trans isomerase (PIN1) / cyclophilin / rotamase
265817_at	At2g18050	0,19	0,36	0,44	0,18	0,32	1,29	1,22	1,00	histone H1-3 (HIS1-3)
265813_at	At2g18060	1,11	1,14	1,03	1,01	1,12	1,00	0,98	1,00	no apical meristem (NAM) family protein
265812_at	At2g18070	1,01	1,04	0,95	1,03	1,11	0,99	1,04	0,98	hypothetical protein
265811_at	At2g18080	1,04	1,05	1,09	1,01	0,94	1,14	1,10	0,98	serine carboxypeptidase S28 family protein
265810_at	At2g18090	0,84	1,00	0,96	0,82	0,95	1,03	1,17	1,10	PHD finger family protein / SWIB complex BAF60b domain-containing prote
265809_at	At2g18100	0,88	0,93	0,80	0,90	0,86	0,79	0,98	0,91	expressed protein
265816_s_at	At2g18110	1,27	1,07	1,03	1,08	1,15	0,98	0,98	1,00	elongation factor 1-beta / EF-1-beta
265803_at	At2g18115	0,98	1,01	1,10	1,05	0,94	1,23	1,09	1,13	---
257456_at	At2g18120	1,11	0,99	1,06	0,82	1,07	0,97	1,10	0,93	lateral root primordium (LRP) protein-related
263063_s_at	At2g18140	1,02	1,08	1,21	3,43	2,69	0,91	0,75	0,82	peroxidase, putative
263064_at	At2g18160	1,07	0,98	0,89	0,89	0,98	0,78	0,79	0,94	bZIP transcription factor family protein
263065_at	At2g18170	0,60	0,66	0,80	0,82	0,95	0,82	0,74	0,77	mitogen-activated protein kinase, putative / MAPK, putative (MPK7)
263062_at	At2g18180	1,00	0,97	1,23	0,95	1,00	1,00	0,97	0,79	SEC14 cytosolic factor, putative / phosphoglyceride transfer protein, putativ
263061_at	At2g18190	1,27	1,20	1,16	1,05	1,04	1,12	1,02	1,00	AAA-type ATPase family protein
265328_at	At2g18200	1,03	0,94	0,96	1,03	1,11	0,97	1,02	0,86	hypothetical protein
265327_at	At2g18210	1,20	1,14	0,78	0,68	0,82	1,17	1,36	1,02	expressed protein
265326_at	At2g18220	1,31	0,90	0,98	0,86	0,93	1,17	1,02	1,29	expressed protein
265339_at	At2g18230	1,23	1,13	0,94	1,71	1,60	1,11	1,14	0,98	inorganic pyrophosphatase (soluble) (PPA) / pyrophosphate phospho-hydro
265325_at	At2g18240	0,96	0,93	0,80	1,19	0,89	1,23	1,06	0,80	RER1 protein, putative
265335_at	At2g18245	1,00	1,01	1,20	1,01	1,14	0,84	0,81	0,80	expressed protein
265324_at	At2g18250	1,04	1,06	1,07	0,91	0,87	0,92	0,85	1,22	cytidylyltransferase domain-containing protein
265323_at	At2g18260	1,04	1,11	1,15	1,13	1,00	1,03	1,06	1,06	syntaxin-related protein, putative (SYP112)
265322_at	At2g18270	0,94	0,96	1,04	0,99	1,01	0,98	0,93	0,96	expressed protein
265321_at	At2g18280	0,73	0,87	0,91	0,56	0,55	0,88	0,97	1,08	tubby-like protein 2 (TULP2)
265336_at	At2g18290	1,43	1,33	1,40	1,00	0,94	1,20	1,08	1,08	anaphase-promoting complex, subunit 10 family / APC10 family
265342_at	At2g18300	5,60	5,32	2,60	0,84	0,72	1,95	1,48	1,16	basic helix-loop-helix (bHLH) family protein
265320_at	At2g18320	0,91	1,08	1,01	1,00	1,14	1,07	0,84	0,88	hypothetical protein
265340_at	At2g18330	1,59	1,48	1,50	0,90	1,15	1,19	1,07	0,99	AAA-type ATPase family protein
265379_at	At2g18340	1,03	0,96	1,25	1,02	1,02	0,99	1,07	1,08	late embryogenesis abundant domain-containing protein / LEA domain-cont
265333_at	At2g18350	1,22	0,94	1,05	1,37	1,13	1,03	0,91	0,91	zinc finger homeobox family protein / ZF-HD homeobox family protein
265341_at	At2g18360	1,82	1,52	1,79	1,33	1,10	1,14	0,93	1,01	hydrolase, alpha/beta fold family protein
265334_at	At2g18370	1,14	1,69	1,07	0,81	0,62	1,03	1,50	1,84	protease inhibitor/seed storage/lipid transfer protein (LTP) family protein
265378_at	At2g18380	1,09	1,21	1,06	1,00	1,21	1,06	0,91	0,76	zinc finger (GATA type) family protein
265337_at	At2g18390	1,24	1,08	1,18	0,95	1,04	1,02	1,02	0,98	ADP-ribosylation factor-like protein 2 (ARL2)
265338_at	At2g18400	1,27	1,04	0,99	1,33	1,11	1,00	1,00	0,91	ribosomal protein L6 family protein
265332_at	At2g18410	1,12	1,15	1,16	1,18	0,80	0,95	0,89	0,75	expressed protein
265331_at	At2g18420	1,11	0,98	0,96	0,91	1,08	1,07	0,91	1,06	gibberellin-responsive protein, putative
265330_at	At2g18440	0,91	1,01	1,17	0,59	0,52	0,92	1,48	2,22	expressed protein
265329_at	At2g18450	0,94	0,84	0,94	1,17	1,40	1,01	0,97	0,80	succinate dehydrogenase (ubiquinone) flavoprotein subunit, mitochondrial,
265923_at	At2g18470	0,95	1,04	1,04	0,97	1,08	1,08	1,01	0,94	protein kinase family protein
265922_at	At2g18480	1,19	0,97	0,90	0,92	0,96	0,84	1,21	0,65	mannitol transporter, putative
265921_at	At2g18490	0,83	1,01	0,90	0,96	0,91	1,04	1,01	0,93	zinc finger (C2H2 type) family protein
265951_at	At2g18500	0,95	1,02	1,15	0,86	1,32	0,96	1,08	1,12	ovate family protein
265930_at	At2g18510	1,35	1,40	1,55	1,05	1,40	1,12	0,97	1,05	pre-mRNA splicing factor, putative
265931_at	At2g18520	1,26	1,06	1,09	1,15	1,20	0,92	0,98	0,82	pentatricopeptide (PPR) repeat-containing protein

265950_s_at	At2g18530	1,04	1,11	0,97	1,06	1,07	0,95	1,20	1,06	protein kinase family protein
265949_at	At2g18540	0,98	0,97	0,89	0,90	1,27	1,19	0,93	0,97	cupin family protein
265983_at	At2g18550	0,95	0,93	1,18	1,10	0,88	0,85	0,95	1,03	homeobox-leucine zipper family protein
265929_s_at	At2g18570	0,98	1,00	0,98	0,99	1,21	0,90	0,99	1,20	UDP-glucoronosyl/UDP-glucosyl transferase family protein
265928_at	At2g18580	0,97	0,98	0,99	0,93	1,08	0,93	1,04	1,12	---
265927_at	At2g18590	0,95	1,12	0,98	1,03	1,09	0,98	1,04	1,11	transporter-related
265926_at	At2g18600	1,33	1,17	1,11	1,21	1,35	1,35	1,30	1,06	RUB1-conjugating enzyme, putative
265925_at	At2g18610	1,02	1,14	1,03	0,97	1,12	0,96	0,96	1,06	hypothetical protein
265924_at	At2g18620	1,03	0,99	0,91	0,97	0,99	1,08	1,04	1,08	geranylgeranyl pyrophosphate synthase, putative / GGPP synthetase, puta
266060_at	At2g18630	0,88	1,02	0,96	1,37	1,57	0,90	1,12	1,16	expressed protein
266068_at	At2g18640	0,90	0,95	0,95	0,96	1,14	1,05	0,97	0,96	geranylgeranyl pyrophosphate synthase, putative / GGPP synthetase, puta
266069_at	At2g18650	1,00	1,03	1,01	0,97	0,95	0,94	0,93	0,97	zinc finger (C3HC4-type RING finger) family protein
266070_at	At2g18660	0,59	0,58	0,54	0,82	0,63	5,19	4,28	8,59	expansin family protein (EXPR3)
266016_at	At2g18670	0,96	0,97	0,90	0,81	0,79	0,93	1,11	1,13	zinc finger (C3HC4-type RING finger) family protein
266071_at	At2g18680	0,78	0,69	0,75	0,74	0,85	1,16	0,84	1,05	expressed protein
266017_at	At2g18690	1,13	1,02	0,80	1,29	1,03	1,20	0,89	0,88	expressed protein
266072_at	At2g18700	1,24	1,59	1,95	0,61	0,61	1,27	1,61	1,87	glycosyl transferase family 20 protein / trehalose-phosphatase family protei
266018_at	At2g18710	1,07	1,06	1,01	0,86	0,74	1,01	0,99	0,96	preprotein translocase secY subunit, chloroplast (CpSecY)
266061_at	At2g18720	1,04	1,13	1,11	0,96	1,15	0,90	1,02	0,99	eukaryotic translation initiation factor 2 subunit 3, putative / eIF2S3, putative
266062_at	At2g18730	0,89	0,75	0,91	0,99	0,98	0,99	1,06	1,43	diacylglycerol kinase, putative
266074_at	At2g18740	1,37	1,04	0,95	0,82	1,00	1,18	1,07	0,90	small nuclear ribonucleoprotein E, putative / snRNP-E, putative / Sm protei
266019_at	At2g18750	1,04	1,05	1,32	1,06	0,99	0,98	1,02	1,18	calmodulin-binding protein
266063_at	At2g18760	1,00	0,95	0,88	0,97	1,02	0,92	0,95	0,94	SNF2 domain-containing protein / helicase domain-containing protein
266073_at	At2g18770	0,98	1,04	1,00	0,93	1,02	1,03	0,90	0,97	expressed protein
266064_at	At2g18780	1,03	1,09	1,18	1,06	0,95	0,94	1,00	0,98	F-box family protein
266065_at	At2g18790	1,35	1,46	1,73	0,91	0,81	1,40	1,39	1,37	phytochrome B (PHYB)
266066_at	At2g18800	0,96	0,98	0,97	1,10	1,17	1,09	1,14	1,01	xyloglucan:xyloglucosyl transferase, putative / xyloglucan endotransglycosy
266067_s_at	At2g18810	0,91	0,96	1,09	0,91	1,03	1,02	1,06	0,86	hypothetical protein
266943_at	At2g18820	1,00	1,04	0,95	1,03	1,05	1,08	0,88	0,94	---
266947_at	At2g18830	0,95	1,03	0,97	1,01	1,04	0,98	1,02	0,85	hypothetical protein
266949_s_at	At2g18840	0,86	0,76	0,84	0,90	0,86	0,97	0,92	0,81	integral membrane Yip1 family protein
266948_at	At2g18850	1,07	0,86	1,06	0,82	0,70	1,02	0,89	0,99	SET domain-containing protein
266944_at	At2g18870	1,02	0,91	1,13	0,95	0,93	0,99	1,02	1,08	expressed protein
266945_at	At2g18880	1,03	0,92	1,07	0,87	0,93	1,15	1,02	1,15	fibronectin type III domain-containing protein
266946_at	At2g18890	0,88	1,22	1,01	0,87	0,92	1,09	1,02	0,87	protein kinase family protein
266934_at	At2g18900	1,18	1,02	1,08	0,79	0,79	1,00	1,06	0,91	transducin family protein / WD-40 repeat family protein
266950_at	At2g18910	0,96	0,70	0,64	1,36	1,23	0,85	0,76	0,79	hydroxyproline-rich glycoprotein family protein
266935_at	At2g18915	0,82	1,00	0,95	0,98	1,04	0,87	0,95	1,02	F-box family protein / LOV kelch protein 2 (LKP2) / adagio 2 (ADO2)
266936_at	At2g18920	0,98	0,92	0,85	0,87	1,07	0,92	1,06	1,11	hypothetical protein
266937_at	At2g18930	0,93	1,02	1,07	0,97	0,98	1,03	0,99	0,86	hypothetical protein
266951_at	At2g18940	0,86	0,80	0,81	0,70	0,52	0,95	0,90	0,97	pentatricopeptide (PPR) repeat-containing protein
266938_at	At2g18950	0,77	0,75	0,71	0,92	0,77	0,99	0,95	0,94	homogentisate phytylprenyltransferase family protein (HPT1) / tocopherol p
266939_at	At2g18960	0,95	0,88	0,91	0,74	0,71	1,01	1,02	1,01	ATPase 1, plasma membrane-type, putative / proton pump 1, putative / prot
266940_at	At2g18970	0,95	1,24	1,01	0,98	1,01	1,03	0,97	1,05	expressed protein
266941_at	At2g18980	0,79	0,96	0,45	1,64	1,41	1,22	1,22	1,21	peroxidase, putative
266942_at	At2g18990	0,94	0,92	0,90	1,09	0,87	1,02	0,94	0,79	expressed protein

267443_at	At2g19000	0,95	1,01	0,91	0,85	1,09	1,00	1,04	0,81 expressed protein
267466_at	At2g19010	0,86	1,01	1,01	1,00	1,00	1,01	0,81	0,77 GDSL-motif lipase/hydrolase family protein
257350_x_at	At2g19040	0,90	1,02	0,84	1,04	1,12	0,99	0,97	1,17 rapid alkalinization factor (RALF) family protein
267438_at	At2g19050	0,98	1,02	1,04	1,00	0,93	1,04	1,13	0,84 GDSL-motif lipase/hydrolase family protein
267439_at	At2g19060	0,87	0,94	0,72	0,81	1,06	1,00	0,91	1,09 GDSL-motif lipase/hydrolase family protein
267440_at	At2g19070	0,88	0,91	1,25	0,97	0,91	0,98	0,94	1,16 transferase family protein
267442_at	At2g19080	1,17	1,08	1,25	1,14	1,06	0,94	1,03	1,01 metaxin-related
267487_at	At2g19100	0,95	1,04	1,07	0,83	0,97	0,97	0,99	0,91 ---
267488_at	At2g19110	0,96	1,13	1,00	0,82	0,74	1,03	1,30	1,07 ATPase E1-E2 type family protein / haloacid dehalogenase-like hydrolase fa
267489_s_at	At2g19120	0,75	1,02	1,24	0,68	0,55	1,27	1,65	1,71 tRNA-splicing endonuclease positive effector-related
267490_at	At2g19130	0,61	0,81	0,73	0,80	0,89	1,02	0,80	1,13 S-locus lectin protein kinase family protein
267491_at	At2g19140	0,88	1,08	1,02	1,09	1,01	0,94	1,01	1,08 ---
267464_at	At2g19150	0,92	1,14	0,94	0,94	1,10	1,06	1,07	1,00 pectinesterase family protein
267465_at	At2g19170	1,00	0,92	0,71	0,80	0,94	0,88	0,77	0,90 subtilase family protein
267444_at	At2g19180	1,57	1,62	1,56	1,39	1,19	1,06	1,00	1,16 expressed protein
267436_at	At2g19190	0,83	1,00	1,27	1,71	1,23	1,10	1,01	1,20 light-responsive receptor protein kinase / senescence-responsive receptor-l
267437_at	At2g19200	0,98	1,05	0,97	0,97	1,25	0,94	1,09	1,08 expressed protein
267441_at	At2g19210	1,17	0,94	1,11	1,01	1,08	1,02	0,97	0,97 leucine-rich repeat protein kinase, putative
267328_at	At2g19230	0,95	0,90	1,06	0,95	1,00	0,89	0,99	0,95 leucine-rich repeat protein kinase, putative
267329_at	At2g19250	1,10	0,91	0,99	0,99	0,97	0,98	1,02	1,18 pseudogene, similar to Magnesium-chelatase subunit chlD, chloroplast prec
267330_at	At2g19270	1,30	1,03	1,29	1,07	1,09	0,95	0,95	0,93 expressed protein
267331_at	At2g19280	1,06	0,96	1,16	0,98	0,98	0,99	1,01	1,01 pentatricopeptide (PPR) repeat-containing protein
267332_at	At2g19290	1,01	1,02	1,02	0,99	1,17	0,87	1,04	0,98 expressed protein
267320_at	At2g19300	0,95	0,91	0,94	0,96	1,08	1,04	0,97	1,01 hypothetical protein
267336_at	At2g19310	1,11	1,18	1,32	0,84	1,10	0,93	0,85	0,75 expressed protein
267321_at	At2g19320	0,78	0,71	0,81	0,80	0,96	1,02	0,96	1,03 expressed protein
267322_at	At2g19330	1,06	1,00	0,95	0,89	0,98	1,04	0,94	1,11 leucine-rich repeat family protein
267323_at	At2g19340	1,22	1,08	1,04	1,18	1,37	1,01	0,84	0,84 membrane protein, putative
267278_at	At2g19350	0,98	0,96	0,99	0,79	0,81	0,97	0,89	1,14 expressed protein
267324_at	At2g19360	0,93	0,97	1,02	0,97	1,01	0,95	0,97	0,88 hypothetical protein
267325_at	At2g19370	0,94	0,98	0,94	0,86	1,02	1,01	0,97	0,96 ---
267326_at	At2g19380	1,01	1,01	1,01	0,95	1,09	1,12	1,08	1,10 RNA recognition motif (RRM)-containing protein
267309_at	At2g19385	1,15	0,96	0,93	0,92	0,97	1,17	0,98	0,85 expressed protein
267282_at	At2g19390	0,88	0,96	1,04	0,92	0,89	1,15	0,97	1,20 expressed protein
267281_at	At2g19400	1,00	0,92	0,87	0,93	1,00	1,01	0,98	0,88 protein kinase, putative
267327_at	At2g19410	0,90	1,01	0,85	0,97	0,91	0,92	0,97	0,97 protein kinase family protein
267334_at	At2g19420	1,05	1,05	1,04	1,10	1,09	1,00	1,03	1,07 hypothetical protein
267333_at	At2g19430	0,94	1,00	1,05	0,97	0,92	0,94	0,90	0,97 transducin family protein / WD-40 repeat family protein
267335_s_at	At2g19440	0,81	0,67	0,84	0,91	0,86	1,02	0,91	1,02 glycosyl hydrolase family 17 protein
267280_at	At2g19450	0,59	0,57	0,64	0,94	0,87	0,78	0,77	0,90 diacylglycerol O-acyltransferase / acyl CoA:diacylglycerol acyltransferase (D
267279_at	At2g19460	1,01	0,85	0,88	1,42	1,56	0,71	0,74	1,05 expressed protein
265942_at	At2g19470	1,08	1,03	1,17	0,76	1,09	1,16	1,09	1,18 casein kinase, putative
265940_at	At2g19480	1,47	1,15	1,03	0,92	0,99	1,03	1,03	1,10 nucleosome assembly protein (NAP), putative
265945_at	At2g19500	1,04	1,11	1,21	0,91	1,09	0,97	0,99	0,94 FAD-binding domain-containing protein / cytokinin oxidase family protein
265932_at	At2g19510	0,97	0,98	1,02	1,03	1,08	1,07	1,01	0,92 LOB domain family protein / lateral organ boundaries domain family protein
265946_s_at	At2g19520	1,13	0,98	0,99	0,90	0,89	0,97	1,02	1,01 WD-40 repeat family protein

265947_at	At2g19540	1,08	1,14	1,09	0,94	0,89	1,02	1,04	1,04 transducin family protein / WD-40 repeat family protein
265933_at	At2g19550	1,02	0,88	0,97	0,84	1,03	1,05	1,07	1,13 esterase/lipase/thioesterase family protein
265934_at	At2g19560	1,05	1,15	1,37	1,11	0,63	1,03	1,08	1,18 proteasome protein-related
265943_at	At2g19570	1,00	1,00	0,90	1,89	1,77	0,94	0,93	0,89 cytidine deaminase (CDD) / cytidine aminohydrolase
265935_at	At2g19580	0,98	1,12	1,20	1,23	1,52	1,12	1,05	1,02 senescence-associated protein-related
265948_at	At2g19590	1,01	1,06	1,07	1,77	1,68	0,80	0,73	0,84 1-aminocyclopropane-1-carboxylate oxidase, putative / ACC oxidase, putati
265936_at	At2g19600	1,20	1,24	1,22	1,02	0,97	0,94	1,02	1,09 K+ efflux antiporter, putative (KEA4)
265937_at	At2g19610	1,01	1,18	0,97	1,00	1,15	1,09	0,86	0,83 zinc finger (C3HC4-type RING finger) family protein
265938_at	At2g19620	1,05	1,07	1,55	1,10	1,12	1,07	1,18	1,38 Ndr family protein
257383_at	At2g19630	1,01	0,96	0,94	0,98	1,12	0,89	1,01	1,26 F-box family protein
265944_at	At2g19640	0,78	0,84	0,97	0,98	1,15	0,90	0,90	0,93 SET domain-containing protein
265939_at	At2g19650	1,10	1,04	1,00	0,94	0,93	1,09	0,98	0,88 DC1 domain-containing protein
266688_at	At2g19660	0,96	1,02	0,96	0,99	1,21	0,84	0,93	0,96 DC1 domain-containing protein
266687_at	At2g19670	1,82	1,83	1,61	1,17	1,64	1,02	0,98	1,05 protein arginine N-methyltransferase, putative
266696_at	At2g19680	1,16	0,93	0,95	1,13	1,09	1,02	0,89	0,85 mitochondrial ATP synthase g subunit family protein
266686_at	At2g19700	0,99	1,03	0,95	1,01	0,98	1,09	0,86	0,80 expressed protein
266685_at	At2g19710	0,91	0,91	1,00	1,24	1,07	1,26	1,35	1,57 expressed protein
266684_at	At2g19720	1,41	1,17	1,15	1,35	1,12	1,01	0,92	0,78 40S ribosomal protein S15A (RPS15aB)
266699_at	At2g19730	1,52	1,26	1,09	1,43	1,34	0,91	0,97	0,93 60S ribosomal protein L28 (RPL28A)
266700_at	At2g19740	2,11	1,71	1,59	1,46	1,67	1,16	1,15	1,18 60S ribosomal protein L31 (RPL31A)
266705_at	At2g19750	1,57	1,31	1,12	1,34	1,32	1,02	0,92	0,81 40S ribosomal protein S30 (RPS30A)
266701_at	At2g19760	1,22	0,95	0,85	1,33	1,29	0,86	0,88	0,83 profilin 1 (PRO1) (PFN1) (PRF1) / allergen Ara t 8
266697_at	At2g19770	0,92	0,93	0,98	0,99	1,03	0,97	1,10	1,24 profilin 4 (PRO4) (PFN4)
266682_at	At2g19780	0,87	0,82	0,72	1,10	1,10	0,86	1,18	0,87 leucine-rich repeat family protein / extensin family protein
266694_at	At2g19790	1,06	0,90	0,99	1,12	1,31	1,05	0,94	1,02 clathrin adaptor complex small chain family protein
266693_at	At2g19800	1,43	1,53	2,01	0,82	0,93	0,98	1,12	1,96 expressed protein
266695_at	At2g19810	0,96	1,17	1,09	0,79	0,81	0,80	0,85	1,16 zinc finger (CCCH-type) family protein
266698_at	At2g19830	1,13	0,91	0,96	0,90	1,11	1,05	0,98	1,02 SNF7 family protein
266681_at	At2g19840	0,94	1,00	1,06	1,04	0,99	0,98	0,97	1,10 ---
266680_s_at	At2g19850	1,08	1,01	1,15	1,11	1,03	1,02	1,03	0,95 hypothetical protein
266702_at	At2g19860	0,95	0,89	0,97	0,81	0,86	1,03	1,02	0,96 hexokinase 2 (HXK2)
266703_at	At2g19880	1,26	1,06	1,00	1,01	1,17	0,90	0,85	0,92 ceramide glucosyltransferase, putative
266691_at	At2g19890	0,95	0,87	0,90	0,88	1,25	1,07	0,89	1,13 expressed protein
266690_at	At2g19900	0,43	0,35	0,29	0,78	0,71	1,00	1,09	0,97 malate oxidoreductase, putative
266689_at	At2g19930	0,72	0,73	0,69	0,81	0,58	1,04	1,05	1,04 RNA-dependent RNA polymerase family protein
266704_at	At2g19940	0,81	0,74	0,65	1,06	0,83	0,78	0,76	0,83 semialdehyde dehydrogenase family protein
266683_at	At2g19950	1,21	0,92	1,24	1,02	1,08	1,10	1,06	1,48 expressed protein
266692_at	At2g19960	1,04	1,10	1,08	1,08	1,02	1,02	1,07	0,99 hAT dimerisation domain-containing protein / transposase-related
265588_at	At2g19970	3,09	3,19	2,38	4,70	5,54	1,48	1,14	1,13 pathogenesis-related protein, putative
265587_at	At2g19980	1,19	1,02	0,79	0,88	0,97	0,85	0,98	1,10 allergen V5/Tpx-1-related family protein
265586_at	At2g19990	1,05	1,22	0,99	1,67	1,44	1,30	1,26	0,77 pathogenesis-related protein 1 (PR-1)
265585_at	At2g20000	1,14	1,21	1,34	1,02	1,00	1,08	1,03	1,06 cell division cycle family protein / CDC family protein
265583_at	At2g20010	0,78	0,71	0,77	0,96	1,01	0,92	0,92	1,01 expressed protein
265596_at	At2g20020	0,96	0,72	0,79	0,56	0,49	1,08	1,02	0,94 expressed protein
265582_at	At2g20030	1,10	1,05	1,14	0,95	1,03	0,98	0,92	0,80 zinc finger (C3HC4-type RING finger) family protein
265581_at	At2g20040	1,08	1,24	1,43	0,75	1,01	1,10	1,20	1,20 protein kinase, putative

265580_at	At2g20050	1,13	1,20	1,34	0,93	1,03	1,13	1,08	1,29	protein phosphatase 2C, putative / PP2C, putative
265594_at	At2g20060	1,49	1,26	1,09	1,01	1,04	1,04	1,06	1,04	ribosomal protein L4 family protein
265579_at	At2g20070	0,89	0,93	1,05	1,04	1,11	0,92	0,99	0,97	hypothetical protein
257393_at	At2g20080	0,93	1,08	1,02	0,98	0,98	0,91	0,99	1,10	expressed protein
265578_at	At2g20090	1,17	1,11	1,13	0,95	1,09	1,05	0,95	0,97	ethylene-responsive family protein
265577_at	At2g20100	1,07	1,03	1,17	1,03	1,01	1,06	1,13	1,09	ethylene-responsive family protein
265592_at	At2g20110	0,94	0,96	1,09	1,01	1,08	0,90	1,05	0,87	tesmin/TSO1-like CXC domain-containing protein
265593_s_at	At2g20130	0,78	0,66	0,92	0,96	1,08	0,83	0,85	1,00	expressed protein
265595_at	At2g20140	1,10	0,98	1,01	1,18	0,95	1,03	0,92	0,88	26S protease regulatory complex subunit 4, putative
265597_at	At2g20145	1,89	2,45	2,67	1,31	1,24	1,24	1,71	1,48	expressed protein
265591_at	At2g20150	0,90	0,84	0,95	0,91	1,27	1,11	0,99	1,11	expressed protein
265590_at	At2g20160	1,00	1,00	1,17	0,90	1,24	0,99	1,03	1,25	E3 ubiquitin ligase SCF complex subunit SKP1/ASK1 (At17), putative
265589_at	At2g20170	0,98	1,00	0,97	0,88	1,13	1,00	0,94	1,02	hypothetical protein
265584_at	At2g20180	1,33	1,22	1,08	0,57	0,86	1,04	1,04	1,46	basic helix-loop-helix (bHLH) family protein
265576_at	At2g20190	0,88	0,86	0,93	0,94	0,97	0,90	0,94	0,85	CLIP-associating protein (CLASP) -related
265315_at	At2g20200	0,94	0,97	0,94	1,14	1,24	1,02	0,85	0,86	CLIP-associating protein (CLASP) -related
265314_at	At2g20210	1,03	1,15	1,10	0,75	0,90	1,15	0,98	1,03	leucine-rich repeat family protein
265313_at	At2g20220	0,98	1,14	1,02	0,85	1,04	0,96	1,12	0,94	leucine-rich repeat family protein
265284_at	At2g20230	0,96	0,93	0,93	1,06	0,99	0,98	0,86	0,90	expressed protein
265312_at	At2g20240	0,87	1,01	0,98	0,91	1,11	0,98	0,97	0,86	expressed protein
265311_at	At2g20250	1,12	1,09	1,11	0,83	0,87	1,08	0,87	0,98	hypothetical protein
265287_at	At2g20260	0,89	0,84	0,86	0,80	0,94	0,95	0,96	0,96	photosystem I reaction center subunit IV, chloroplast, putative / PSI-E, puta
265288_at	At2g20270	1,18	0,94	0,82	0,88	0,95	0,97	0,92	0,98	glutaredoxin family protein
265310_at	At2g20280	1,01	1,01	0,96	1,01	0,96	1,12	1,03	1,22	zinc finger (CCCH-type) family protein
265309_at	At2g20290	0,73	0,88	1,01	0,90	1,11	1,02	0,99	1,14	myosin, putative
265308_at	At2g20300	1,01	1,18	1,11	0,94	0,98	0,98	0,95	0,92	protein kinase family protein
265307_at	At2g20310	1,29	1,05	1,19	0,85	0,97	0,96	1,10	0,95	expressed protein
265306_at	At2g20320	0,97	0,84	0,89	1,14	1,00	1,08	0,96	1,00	DENN (AEX-3) domain-containing protein
265259_at	At2g20330	1,17	1,15	1,14	0,79	0,90	1,01	0,98	1,04	transducin family protein / WD-40 repeat family protein
265305_at	At2g20340	0,91	0,78	0,64	0,78	0,67	1,14	1,28	1,13	tyrosine decarboxylase, putative
265304_at	At2g20350	0,88	1,00	1,03	0,88	0,95	1,07	0,92	0,84	AP2 domain-containing transcription factor, putative
265286_at	At2g20360	1,08	0,99	0,94	1,30	1,16	0,91	0,93	0,91	expressed protein
265283_at	At2g20370	1,10	1,00	0,91	1,15	1,12	1,18	0,94	0,93	exostosin family protein
265282_at	At2g20380	1,11	1,04	0,97	1,00	0,90	1,03	1,05	0,94	kelch repeat-containing F-box family protein
265258_at	At2g20390	1,31	1,08	0,92	1,04	0,83	0,92	0,95	0,93	expressed protein
265316_at	At2g20400	1,01	0,97	1,31	0,90	0,97	0,98	0,94	0,99	myb family transcription factor
265285_at	At2g20410	0,97	0,87	0,79	0,92	0,92	0,98	0,96	1,14	activating signal cointegrator-related
265257_at	At2g20420	1,50	1,25	1,14	1,16	1,15	1,18	1,10	1,07	succinyl-CoA ligase (GDP-forming) beta-chain, mitochondrial, putative / suc
257397_at	At2g20430	0,96	0,97	1,20	0,86	0,88	0,92	0,83	0,92	p21-rho-binding domain-containing protein
263366_at	At2g20440	1,12	1,11	1,11	1,00	0,99	1,02	1,19	1,10	RabGAP/TBC domain-containing protein
263372_at	At2g20450	1,37	1,06	1,13	1,01	1,09	1,07	0,98	0,98	60S ribosomal protein L14 (RPL14A)
263367_at	At2g20460	0,96	0,93	0,98	1,02	0,95	0,88	0,95	1,15	---
263368_at	At2g20470	0,86	0,97	1,14	0,89	1,30	0,93	1,07	1,03	protein kinase, putative
263369_at	At2g20480	1,11	1,05	1,05	0,91	1,05	1,15	0,91	0,90	expressed protein
263371_at	At2g20490	1,91	1,57	1,47	1,61	1,65	1,09	1,03	0,97	nucleolar RNA-binding Nop10p family protein
263370_at	At2g20500	0,95	0,89	0,96	0,87	1,16	1,16	1,09	0,94	expressed protein

263395_at	At2g20510	1,10	1,20	0,95	0,99	1,04	0,95	0,95	0,93	mitochondrial import inner membrane translocase subunit TIM44, putative
263373_at	At2g20515	1,18	1,21	0,94	1,37	1,27	1,04	0,93	1,08	expressed protein
263376_at	At2g20520	1,09	1,07	0,93	1,20	1,09	1,12	0,98	1,21	fasciclin-like arabinogalactan-protein (FLA6)
263375_s_at	At2g20530	1,38	1,23	1,14	1,05	0,99	1,00	0,98	0,92	prohibitin, putative
263364_at	At2g20540	1,01	1,03	0,96	1,02	0,99	1,17	1,09	1,09	pentatricopeptide (PPR) repeat-containing protein
263365_at	At2g20550	1,27	1,21	0,95	1,23	1,15	1,04	0,92	0,96	DNAJ chaperone C-terminal domain-containing protein
263374_at	At2g20560	2,03	2,30	2,02	1,86	1,54	1,42	1,44	1,38	DNAJ heat shock family protein
263715_at	At2g20570	0,90	0,89	1,09	0,62	0,78	0,99	0,79	0,79	golden2-like transcription factor (GLK1)
263716_at	At2g20580	1,02	0,98	1,05	1,13	1,06	0,90	0,95	0,94	26S proteasome regulatory subunit S2 (RPN1)
263712_at	At2g20585	1,20	1,12	0,90	0,98	1,25	0,88	0,83	0,78	expressed protein
263713_at	At2g20595	0,96	0,90	0,99	0,94	0,97	1,01	0,84	1,14	expressed protein
263714_at	At2g20610	1,18	1,02	1,24	1,05	1,01	0,91	0,90	0,87	aminotransferase, putative
265421_s_at	At2g20616	0,97	0,81	1,02	0,93	0,89	0,95	0,93	0,81	hypothetical protein
263741_at	At2g20620	1,03	1,16	1,14	0,88	1,06	1,00	1,13	1,15	expressed protein
263742_at	At2g20625	0,90	0,93	1,22	1,01	0,85	1,18	1,85	2,13	hypothetical protein
263711_at	At2g20630	0,65	0,60	0,75	0,91	0,95	1,11	1,29	1,42	protein phosphatase 2C, putative / PP2C, putative
265432_at	At2g20650	0,92	1,04	0,93	0,76	0,88	0,89	0,97	1,37	zinc finger (C3HC4-type RING finger) family protein
263740_at	At2g20660	1,07	1,10	0,95	0,96	1,23	0,89	1,01	0,81	rapid alkalinization factor (RALF) family protein
265387_at	At2g20670	2,50	2,82	1,82	0,51	0,34	1,21	2,36	2,15	expressed protein
265431_at	At2g20680	1,02	1,15	0,87	0,85	0,87	0,73	0,81	1,09	glycosyl hydrolase family 5 protein / cellulase family protein
265389_at	At2g20690	0,89	0,69	0,67	0,75	0,74	0,94	0,91	0,82	lumazine-binding family protein
265430_at	At2g20700	0,98	1,26	1,13	1,05	0,99	1,21	1,03	0,86	expressed protein
265429_at	At2g20710	1,15	1,01	1,17	1,13	1,10	1,11	0,92	1,12	pentatricopeptide (PPR) repeat-containing protein
265428_at	At2g20720	0,95	1,04	1,26	0,93	1,06	1,07	1,00	0,83	pentatricopeptide (PPR) repeat-containing protein
265394_at	At2g20725	0,74	0,72	0,81	0,70	0,75	0,79	0,84	0,81	CAAX amino terminal protease family protein
265427_at	At2g20730	0,83	0,80	0,80	0,90	0,92	0,81	0,94	0,94	expressed protein
265426_at	At2g20740	0,83	0,87	0,89	0,87	0,94	0,94	0,91	1,02	expressed protein
265443_at	At2g20750	1,09	1,02	0,92	1,07	1,09	1,04	1,17	0,97	beta-expansin, putative (EXPB1)
265384_at	At2g20760	1,09	0,93	0,91	1,17	1,23	1,00	0,93	1,01	expressed protein
265425_at	At2g20770	0,76	0,74	0,73	0,98	0,96	1,00	0,84	0,87	lanthionine synthetase C-like family protein
265424_at	At2g20780	0,62	0,62	0,63	1,00	1,03	0,77	0,79	0,88	mannitol transporter, putative
265423_at	At2g20790	0,86	0,88	0,83	0,89	0,94	0,91	0,94	0,82	expressed protein
265422_at	At2g20800	1,25	1,47	1,21	1,00	1,49	1,04	1,09	0,93	pyridine nucleotide-disulphide oxidoreductase family protein
265434_at	At2g20805	0,98	0,97	1,03	0,96	0,89	1,03	1,02	0,98	hypothetical protein
265391_at	At2g20810	0,95	0,92	0,85	1,14	1,05	0,91	0,91	0,97	glycosyl transferase family 8 protein
265390_at	At2g20820	1,19	1,07	0,87	1,27	1,29	0,98	0,91	0,93	expressed protein
265393_at	At2g20830	0,73	0,65	0,66	0,98	0,90	1,12	0,97	1,04	expressed protein
265419_at	At2g20840	0,74	0,63	0,88	1,28	1,19	0,94	0,95	1,08	secretory carrier membrane protein (SCAMP) family protein
265395_at	At2g20850	0,74	0,81	1,22	0,96	1,06	0,83	1,02	1,29	leucine-rich repeat protein kinase, putative
265392_at	At2g20860	1,07	1,00	1,04	0,90	1,09	1,02	0,97	0,92	lipoic acid synthase (LIP1)
265441_at	At2g20870	1,07	0,92	1,10	0,97	1,02	1,19	1,07	0,94	cell wall protein precursor, putative
257396_at	At2g20875	0,90	1,00	0,99	1,19	1,22	0,94	0,89	0,78	hypothetical protein
265418_at	At2g20880	0,85	0,96	0,92	1,45	1,24	1,08	1,23	0,83	AP2 domain-containing transcription factor, putative
265415_at	At2g20890	0,94	0,88	0,90	0,92	0,83	0,99	0,97	0,95	expressed protein
265385_at	At2g20900	0,96	0,97	1,12	1,11	1,01	0,96	1,08	1,11	diacylglycerol kinase, putative
265417_at	At2g20920	0,88	0,96	1,08	0,82	0,81	1,20	1,22	1,34	expressed protein

265386_at	At2g20930	1,11	0,96	0,95	1,34	1,20	0,86	0,83	0,88 expressed protein
265442_at	At2g20940	1,25	1,15	1,19	1,27	1,71	0,86	0,83	0,85 expressed protein
265433_at	At2g20950	0,91	0,98	1,07	0,82	0,81	1,12	1,24	1,21 expressed protein
265440_at	At2g20960	0,57	0,62	1,02	0,77	0,89	1,04	1,12	1,08 expressed protein
265438_at	At2g20970	1,00	1,03	0,88	0,95	1,11	0,98	0,95	0,91 hypothetical protein
265437_at	At2g20980	1,11	1,26	0,99	1,19	1,11	1,01	1,05	1,20 expressed protein
265388_s_at	At2g20990	1,13	0,91	0,87	1,00	0,88	0,94	0,99	0,94 C2 domain-containing protein (sytA)
265436_at	At2g21000	1,06	0,96	0,90	0,95	1,05	1,02	1,09	0,89 ---
265435_s_at	At2g21020	1,06	1,26	0,92	1,17	0,97	0,94	0,97	0,73 major intrinsic family protein / MIP family protein
265420_s_at	At2g21030	1,02	1,02	0,92	0,88	1,00	0,99	0,94	1,17 expressed protein
265396_at	At2g21040	1,00	0,89	1,13	0,91	1,06	0,90	0,96	0,77 C2 domain-containing protein
265439_at	At2g21045	1,24	1,18	0,88	1,24	1,10	1,25	1,41	1,18 senescence-associated family protein
264025_at	At2g21050	1,25	1,26	1,16	1,28	1,19	0,90	0,99	0,88 amino acid permease, putative
264026_at	At2g21060	1,05	1,08	1,03	1,25	1,08	0,78	1,16	0,92 cold-shock DNA-binding family protein / glycine-rich protein (GRP2)
264013_at	At2g21070	0,85	1,00	1,00	0,90	1,18	0,82	0,69	0,85 expressed protein
264012_at	At2g21080	0,79	1,16	1,12	0,71	0,89	1,01	1,14	1,04 expressed protein
264011_at	At2g21090	0,98	0,95	0,80	0,78	0,96	0,86	0,89	0,78 pentatricopeptide (PPR) repeat-containing protein
264010_at	At2g21100	1,05	1,16	1,16	1,14	1,22	1,06	1,09	1,17 disease resistance-responsive protein-related / dirigent protein-related
264009_at	At2g21110	0,93	1,02	1,14	0,90	0,99	1,14	0,93	0,93 disease resistance-responsive family protein
264008_at	At2g21120	0,83	0,86	1,20	1,16	1,01	1,05	1,26	1,35 expressed protein
264019_at	At2g21130	0,78	0,69	0,63	0,91	0,89	1,00	0,89	0,86 peptidyl-prolyl cis-trans isomerase / cyclophilin (CYP2) / rotamase
264007_at	At2g21140	1,01	0,88	0,76	1,97	1,94	0,88	0,76	0,70 hydroxyproline-rich glycoprotein family protein
264015_at	At2g21150	1,10	1,07	1,04	0,91	1,01	1,07	1,07	1,04 XAP5 family protein
264020_at	At2g21160	1,13	1,03	0,85	1,45	1,22	1,02	0,89	0,92 translocon-associated protein alpha (TRAP alpha) family protein
264018_at	At2g21170	0,88	0,82	0,81	0,71	0,78	0,94	0,97	0,97 triosephosphate isomerase, chloroplast, putative
264024_at	At2g21180	0,95	1,26	1,30	1,15	1,40	0,93	0,94	1,08 expressed protein
264022_at	At2g21185	0,99	0,95	1,05	0,98	0,80	1,08	1,12	1,04 expressed protein
264017_s_at	At2g21190	1,05	1,00	0,90	1,28	1,05	0,96	0,93	0,98 ER lumen protein retaining receptor family protein
264023_at	At2g21195	0,96	1,07	1,25	0,95	1,01	0,89	0,95	0,81 expressed protein
264021_at	At2g21200	1,01	0,94	0,94	0,95	1,07	0,99	0,86	0,89 auxin-responsive protein, putative
264014_at	At2g21210	0,79	0,88	0,95	0,41	0,60	0,98	1,05	1,09 auxin-responsive protein, putative
264016_at	At2g21220	1,06	1,02	1,19	1,10	1,31	1,00	0,86	0,81 auxin-responsive protein, putative
263414_at	At2g21230	0,94	0,95	1,15	0,91	1,19	0,92	0,95	1,10 bZIP family transcription factor
263413_at	At2g21240	0,86	1,01	1,38	1,02	0,94	0,96	0,91	1,13 expressed protein
263758_s_at	At2g21260	0,98	0,95	1,04	0,94	1,03	0,90	0,96	0,88 mannose 6-phosphate reductase (NADPH-dependent), putative
263756_at	At2g21270	0,97	0,92	1,07	1,11	1,14	0,94	1,00	0,86 ubiquitin fusion degradation UFD1 family protein
263760_at	At2g21280	1,16	1,00	0,88	0,84	0,92	1,07	1,09	1,09 expressed protein
263759_at	At2g21290	1,31	1,30	1,26	1,36	1,41	1,01	0,96	1,02 expressed protein
263751_at	At2g21300	1,22	1,35	1,40	1,01	1,18	0,96	1,02	1,09 kinesin motor family protein
263752_at	At2g21310	1,01	0,94	1,06	0,96	1,08	1,07	1,10	1,02 ---
263739_at	At2g21320	2,13	2,23	1,49	1,62	1,37	2,26	1,61	1,27 zinc finger (B-box type) family protein
263761_at	At2g21330	1,45	1,26	1,26	0,75	0,71	1,27	1,21	1,14 fructose-bisphosphate aldolase, putative
263755_at	At2g21340	0,84	0,84	0,79	0,67	0,60	1,02	0,97	1,02 enhanced disease susceptibility protein, putative / salicylic acid induction de
263773_at	At2g21370	0,99	0,92	0,81	0,89	0,87	0,97	0,94	1,01 xylulose kinase, putative
263762_at	At2g21380	0,87	0,75	0,87	0,79	0,73	1,03	1,13	1,20 kinesin motor protein-related
263763_at	At2g21385	0,98	0,92	0,91	0,78	0,84	1,19	1,12	1,19 expressed protein

263743_at	At2g21390	0,95	0,84	0,83	1,14	1,10	0,84	0,80	0,82	coatomer protein complex, subunit alpha, putative
263764_at	At2g21410	0,81	0,82	0,81	0,94	0,87	0,96	0,94	0,80	vacuolar proton ATPase, putative
263744_at	At2g21420	0,98	1,00	1,12	0,87	1,09	1,00	0,97	0,83	zinc finger protein-related
263757_at	At2g21430	1,15	1,05	1,07	0,95	0,92	0,95	0,93	0,96	cysteine proteinase A494, putative / thiol protease, putative
263766_at	At2g21440	1,20	1,07	1,35	0,68	0,77	1,10	1,13	1,26	RNA recognition motif (RRM)-containing protein
263745_at	At2g21450	1,06	0,98	0,97	0,97	1,07	1,05	1,08	0,96	SNF2 domain-containing protein / helicase domain-containing protein
263746_at	At2g21460	0,95	0,94	1,05	1,03	1,07	0,98	1,10	1,03	---
263747_at	At2g21470	1,18	1,04	1,09	1,07	0,97	1,20	1,03	1,19	SUMO activating enzyme 2 (SAE2)
263748_at	At2g21480	0,95	0,94	0,99	1,15	1,14	1,07	0,96	0,99	protein kinase family protein
263753_at	At2g21490	0,66	0,61	0,97	0,81	1,07	1,03	0,99	1,34	dehydrin family protein
263754_at	At2g21510	0,54	0,72	1,29	1,51	1,34	0,75	1,19	1,42	DNAJ heat shock N-terminal domain-containing protein
263749_at	At2g21520	1,08	0,99	1,06	0,98	1,05	1,18	1,13	1,19	SEC14 cytosolic factor, putative / phosphoglyceride transfer protein, putativ
263750_at	At2g21530	1,25	1,23	1,69	0,96	0,87	1,08	1,00	1,03	forkhead-associated domain-containing protein / FHA domain-containing pr
263765_at	At2g21540	0,94	0,72	0,68	1,29	1,27	0,62	0,61	0,73	SEC14 cytosolic factor, putative / phosphoglyceride transfer protein, putativ
263546_at	At2g21550	1,09	0,94	0,59	1,15	0,95	1,07	0,86	0,83	bifunctional dihydrofolate reductase-thymidylate synthase, putative / DHFR-
263545_at	At2g21560	1,06	1,05	1,05	1,11	1,11	1,02	1,02	1,07	expressed protein
263519_at	At2g21580	1,33	1,06	0,96	1,38	1,24	0,90	0,99	0,86	40S ribosomal protein S25 (RPS25B)
263544_at	At2g21590	1,05	0,91	0,98	0,95	0,92	1,03	1,22	1,10	glucose-1-phosphate adenylyltransferase large subunit, putative / ADP-gluc
263516_at	At2g21600	1,23	1,07	0,99	1,14	1,07	0,96	0,95	0,95	RER1B protein
263543_at	At2g21610	1,09	1,04	1,01	1,09	1,44	0,96	0,94	0,81	pectinesterase family protein
263517_at	At2g21620	0,66	0,56	0,60	1,02	0,99	0,77	0,78	0,85	universal stress protein (USP) family protein / responsive to dessication pro
263542_at	At2g21630	1,03	1,02	1,45	1,00	0,85	0,87	0,87	0,96	transport protein, putative
263515_at	At2g21640	1,91	1,73	1,55	1,46	1,51	1,71	1,41	1,43	expressed protein
263549_at	At2g21650	0,97	1,03	1,05	0,99	1,17	0,94	0,90	0,96	myb family transcription factor
263518_at	At2g21655	0,99	1,14	0,99	0,98	0,92	1,05	1,02	0,88	expressed protein
263548_at	At2g21660	0,72	0,71	0,82	0,80	0,79	0,90	0,97	0,98	glycine-rich RNA-binding protein (GRP7)
263547_at	At2g21680	0,93	0,98	0,97	0,95	1,17	0,83	0,98	1,18	kelch repeat-containing F-box family protein
263925_at	At2g21690	1,01	1,04	0,92	0,99	1,04	0,99	1,00	1,13	RNA-binding protein, putative
263899_at	At2g21710	0,94	1,19	1,13	0,73	0,79	0,90	0,81	0,79	mitochondrial transcription termination factor-related / mTERF-related
257434_at	At2g21740	0,98	1,05	1,11	1,03	1,26	0,91	1,02	1,04	hypothetical protein
263872_at	At2g21770	0,92	0,99	0,98	0,92	1,06	1,00	0,99	0,94	cellulose synthase, catalytic subunit, putative
263877_at	At2g21780	0,92	0,93	1,12	1,02	1,07	0,84	1,16	1,02	expressed protein
263882_at	At2g21790	1,59	1,34	1,32	1,14	1,11	0,91	0,99	0,98	ribonucleoside-diphosphate reductase small chain, putative / ribonucleotide
263926_at	At2g21800	1,07	0,99	1,11	0,92	1,09	1,08	1,03	0,87	expressed protein
263881_at	At2g21820	0,42	0,57	0,67	0,81	0,91	1,01	1,02	0,88	expressed protein
263883_at	At2g21830	0,88	1,01	0,90	0,90	0,94	0,76	0,93	0,86	DC1 domain-containing protein
257432_at	At2g21850	1,02	1,15	1,00	0,90	1,07	1,25	1,01	1,09	DC1 domain-containing protein
263873_at	At2g21860	1,41	1,23	1,04	0,65	0,61	1,16	0,94	0,93	violaxanthin de-epoxidase-related
263874_at	At2g21870	1,13	1,02	0,98	1,14	1,22	0,96	0,98	1,07	expressed protein
263876_at	At2g21880	0,60	0,61	0,85	0,96	1,00	0,77	0,78	0,94	Ras-related GTP-binding protein, putative
263927_s_at	At2g21890	1,20	1,36	1,83	1,25	1,09	0,82	1,09	1,21	mannitol dehydrogenase, putative
263893_at	At2g21900	0,93	1,15	1,11	1,09	1,31	1,08	0,97	1,11	WRKY family transcription factor
263894_at	At2g21910	0,96	0,96	0,91	0,99	1,07	0,95	0,94	0,89	cytochrome P450, putative
263895_at	At2g21920	0,98	0,90	0,96	1,00	0,91	1,03	0,93	0,88	hypothetical protein
263896_at	At2g21930	1,02	1,13	0,87	1,23	1,10	1,04	1,00	0,95	F-box family protein
263897_at	At2g21940	0,97	0,99	1,22	1,21	1,03	1,08	1,06	1,41	shikimate kinase, putative

263898_at	At2g21950	0,64	0,82	0,80	0,74	0,74	0,85	0,91	1,08	SKP1 interacting partner 6 (SKIP6)
263880_at	At2g21960	0,81	0,79	0,76	1,12	1,01	1,02	0,88	1,00	expressed protein
263875_at	At2g21970	1,60	1,76	2,44	1,65	1,91	1,53	1,26	1,30	stress enhanced protein 2 (SEP2)
257433_at	At2g21990	0,97	1,02	1,20	1,10	0,86	0,94	0,92	0,87	expressed protein
263869_at	At2g22000	1,21	1,00	1,06	1,07	1,00	1,00	1,01	0,69	expressed protein
263870_at	At2g22010	0,73	0,84	0,85	0,83	0,97	0,91	1,21	1,04	zinc finger (C3HC4-type RING finger) family protein
263871_at	At2g22020	0,70	0,79	0,80	1,07	0,96	0,92	1,25	1,17	zinc finger (C3HC4-type RING finger) family protein
263879_at	At2g22030	1,02	1,01	0,88	1,06	1,05	1,12	0,95	1,23	kelch repeat-containing F-box family protein
263878_s_at	At2g22040	1,09	0,89	0,79	1,02	1,02	0,91	1,10	1,05	transducin family protein / WD-40 repeat family protein
257443_at	At2g22050	0,98	1,01	1,15	0,96	0,94	0,94	1,15	0,87	kelch repeat-containing F-box family protein
263354_at	At2g22060	0,87	1,14	0,93	1,06	1,05	1,09	0,94	1,01	expressed protein
263353_at	At2g22070	1,09	1,04	1,18	0,96	1,09	1,01	1,03	1,02	pentatricopeptide (PPR) repeat-containing protein
263352_at	At2g22080	1,03	0,96	1,33	1,40	1,16	0,91	1,14	0,98	expressed protein
263356_at	At2g22090	0,95	0,94	0,98	1,01	1,06	0,89	0,91	1,00	UBP1 interacting protein 1a (UBA1a)
263355_at	At2g22100	1,04	1,02	0,87	1,08	1,01	1,15	0,96	0,88	RNA recognition motif (RRM)-containing protein
263351_at	At2g22120	0,99	1,05	1,10	1,07	1,02	1,01	0,88	0,90	zinc finger (C3HC4-type RING finger) family protein
263456_at	At2g22125	1,00	0,82	0,93	1,07	1,06	0,88	0,82	0,88	C2 domain-containing protein
263454_at	At2g22160	0,92	1,09	1,06	0,96	0,97	1,03	1,02	1,01	cysteine endopeptidase-related
263431_at	At2g22170	0,80	0,67	0,59	1,55	1,56	0,66	0,61	0,68	lipid-associated family protein
263453_at	At2g22180	1,03	0,99	0,95	1,03	0,99	1,14	1,03	1,07	hydroxyproline-rich glycoprotein family protein
263452_at	At2g22190	0,91	1,46	1,36	1,08	0,86	1,01	1,03	0,98	trehalose-6-phosphate phosphatase, putative
263486_at	At2g22200	1,05	1,19	1,32	0,91	0,85	0,98	1,22	0,84	AP2 domain-containing transcription factor
263432_at	At2g22230	0,90	0,82	0,75	1,02	1,12	0,87	0,85	0,82	beta-hydroxyacyl-ACP dehydratase, putative
263433_at	At2g22240	1,76	1,74	1,56	1,20	1,06	1,22	1,19	1,10	inositol-3-phosphate synthase isozyme 2 / myo-inositol-1-phosphate syntha
263429_at	At2g22250	1,13	0,98	0,99	1,18	0,78	1,10	1,02	0,84	aminotransferase class I and II family protein
263427_at	At2g22260	1,20	1,22	1,14	1,06	1,04	1,06	1,02	0,90	oxidoreductase, 2OG-Fe(II) oxygenase family protein
263430_at	At2g22270	1,00	1,05	1,01	0,96	1,20	0,95	0,95	0,86	expressed protein
263458_at	At2g22290	1,04	1,02	1,07	0,94	1,18	0,89	0,97	0,90	Ras-related GTP-binding protein, putative
263457_at	At2g22300	0,86	1,01	1,58	0,94	0,65	1,15	1,52	1,97	ethylene-responsive calmodulin-binding protein, putative (SR1)
263428_at	At2g22310	1,16	1,34	1,02	0,79	0,99	1,01	0,86	1,52	ubiquitin-specific protease 4 (UBP4)
263455_at	At2g22320	0,94	1,00	1,01	0,97	1,00	1,05	0,93	1,20	hypothetical protein
264052_at	At2g22330	0,78	1,15	0,78	0,90	0,82	0,84	1,10	0,90	cytochrome P450, putative
264051_at	At2g22340	0,95	0,98	1,02	0,96	0,93	0,96	0,94	1,18	hypothetical protein
264050_at	At2g22350	0,90	1,30	0,98	0,97	0,94	1,03	1,02	0,99	RNase H domain-containing protein
264002_at	At2g22360	1,10	0,97	1,14	0,90	0,92	1,23	0,93	0,99	DNAJ heat shock family protein
263999_at	At2g22370	0,99	0,96	0,80	0,88	0,92	0,94	0,91	0,84	expressed protein
264049_at	At2g22390	0,88	0,97	0,93	1,05	1,16	0,81	1,10	0,96	---
264048_at	At2g22400	0,99	0,87	0,86	0,79	0,88	0,97	0,95	0,98	NOL1/NOP2/sun family protein
264047_at	At2g22410	1,03	0,92	0,91	1,05	0,92	0,91	0,94	1,01	pentatricopeptide (PPR) repeat-containing protein
264001_at	At2g22420	0,94	1,28	0,98	1,32	1,04	1,02	1,26	0,91	peroxidase 17 (PER17) (P17)
264004_at	At2g22425	1,11	1,07	0,96	1,40	1,37	0,85	0,85	0,84	expressed protein
264006_at	At2g22430	1,26	1,59	1,28	0,88	1,33	0,91	1,12	1,39	homeobox-leucine zipper protein 6 (HB-6) / HD-ZIP transcription factor 6
264046_at	At2g22440	1,08	1,00	1,05	1,03	1,13	0,96	1,00	0,90	hypothetical protein
264045_at	At2g22450	0,64	0,62	0,66	0,66	0,73	0,86	0,86	0,81	riboflavin biosynthesis protein, putative
264005_at	At2g22470	0,78	0,68	0,57	1,06	0,91	1,23	1,08	1,08	arabinogalactan-protein (AGP2)
264003_at	At2g22475	0,87	0,73	1,06	1,38	1,05	0,80	0,86	1,04	GRAM domain-containing protein / ABA-responsive protein-related

264044_at	At2g22480	0,90	0,92	0,99	1,10	1,29	1,01	0,93	0,90 phosphofructokinase family protein
264043_at	At2g22490	0,87	0,66	0,68	1,00	1,01	0,75	0,75	0,79 cyclin delta-2 (CYCD2)
264000_at	At2g22500	0,71	0,76	0,66	1,09	1,13	1,02	1,03	1,00 mitochondrial substrate carrier family protein
263998_at	At2g22510	0,93	0,84	0,67	1,21	0,93	1,07	1,00	1,05 hydroxyproline-rich glycoprotein family protein
263997_at	At2g22520	0,99	1,09	1,00	0,98	1,01	1,11	0,99	0,96 hypothetical protein
263996_at	At2g22530	0,99	1,16	1,17	1,00	0,98	0,95	1,01	0,91 phosphatidylinositolglycan-related
263995_at	At2g22540	1,02	1,26	1,47	0,83	1,02	1,20	1,13	1,23 short vegetative phase protein (SVP)
264054_at	At2g22550	0,99	1,29	1,48	0,76	0,78	1,17	1,31	1,21 short vegetative phase protein (SVP)
264053_at	At2g22560	0,79	0,76	0,65	1,12	1,17	0,87	1,06	1,05 kinase interacting protein-related
265343_at	At2g22570	0,87	1,03	1,06	0,85	1,05	0,87	0,86	0,89 isochorismatase hydrolase family protein
265290_at	At2g22590	0,94	1,02	1,11	1,10	1,16	0,88	1,04	0,86 glycosyltransferase family protein
265348_at	At2g22600	0,93	1,00	0,94	0,93	0,98	0,96	1,16	0,87 KH domain-containing protein
265349_at	At2g22610	1,11	1,17	1,03	1,38	1,20	1,09	0,93	1,09 kinesin motor protein-related
265350_at	At2g22620	0,95	1,05	1,03	1,11	1,01	1,01	0,90	1,25 expressed protein
265351_at	At2g22630	0,95	0,95	0,94	1,12	1,05	0,94	1,00	1,03 MADS-box protein (AGL17)
265317_at	At2g22640	0,94	1,05	0,98	0,97	0,94	0,93	0,98	0,98 BRICK1, putative
265318_at	At2g22650	1,30	1,47	1,47	0,80	0,67	1,24	1,32	1,14 FAD-dependent oxidoreductase family protein
265344_at	At2g22660	0,72	0,99	1,35	0,77	0,95	0,95	0,95	1,10 glycine-rich protein
265319_at	At2g22670	1,07	0,99	0,86	0,82	0,82	0,93	0,96	0,95 auxin-responsive protein / indoleacetic acid-induced protein 8 (IAA8)
265345_at	At2g22680	0,96	0,85	1,14	1,06	1,21	0,88	1,13	1,17 zinc finger (C3HC4-type RING finger) family protein
265289_at	At2g22690	1,05	0,91	0,97	1,10	1,34	1,02	1,06	1,20 expressed protein
265346_at	At2g22700	0,97	1,16	1,06	0,95	1,10	1,12	0,96	1,26 ---
265291_at	At2g22720	1,27	1,15	1,33	0,94	1,11	1,11	1,03	0,96 expressed protein
265347_at	At2g22740	0,82	0,71	0,81	0,85	0,89	0,99	1,02	0,93 SET domain-containing protein (SUVH6)
266454_at	At2g22750	1,01	1,05	1,04	0,91	1,16	0,90	1,05	1,00 basic helix-loop-helix (bHLH) family protein
266455_at	At2g22760	1,91	2,57	0,96	0,74	0,81	2,09	1,85	1,40 basic helix-loop-helix (bHLH) family protein
266456_at	At2g22770	0,61	0,90	0,62	0,87	0,94	0,56	0,89	1,05 basic helix-loop-helix (bHLH) family protein
266457_at	At2g22780	0,74	0,70	0,70	1,05	1,11	0,88	0,90	0,91 malate dehydrogenase, glyoxysomal, putative
266824_at	At2g22800	1,12	1,15	1,13	0,83	0,92	0,97	1,16	0,87 homeobox-leucine zipper protein 9 (HAT9) / HD-ZIP protein 9
266830_at	At2g22810	0,97	1,07	1,36	0,98	0,97	1,03	0,98	0,97 1-aminocyclopropane-1-carboxylate synthase 4 / ACC synthase 4 (ACS4)
266831_at	At2g22830	0,91	1,13	0,96	0,77	0,75	1,05	1,08	0,97 squalene monooxygenase, putative / squalene epoxidase, putative
266797_at	At2g22840	1,31	1,32	1,43	1,13	1,23	1,07	0,84	0,95 expressed protein
266798_at	At2g22850	0,93	0,91	0,71	1,15	0,91	1,06	1,23	0,98 bZIP transcription factor family protein
266799_at	At2g22860	1,12	1,50	1,51	1,05	1,08	1,29	1,76	1,52 phytosulfokines 2 (PSK2)
266801_at	At2g22870	1,18	1,32	1,72	0,84	0,65	0,91	1,00	1,05 expressed protein
266800_at	At2g22880	1,27	1,96	1,46	1,52	1,81	1,21	1,18	1,33 VQ motif-containing protein
266825_at	At2g22890	0,71	0,82	0,81	1,09	0,83	0,83	0,79	0,81 expressed protein
266802_at	At2g22900	0,67	0,59	0,60	1,20	1,27	0,77	0,70	0,66 galactosyl transferase GMA12/MNN10 family protein
266826_at	At2g22910	0,52	0,51	0,54	1,00	1,07	0,87	0,94	0,78 GCN5-related N-acetyltransferase (GNAT) family protein / amino acid kinas
266827_at	At2g22920	0,69	0,56	0,47	0,89	1,06	0,90	0,99	0,94 serine carboxypeptidase S10 family protein
266828_at	At2g22930	0,95	0,96	0,91	0,93	1,04	0,79	1,03	1,07 glycosyltransferase family protein
266829_at	At2g22940	0,97	1,08	0,98	0,89	1,11	0,99	0,96	1,08 expressed protein
267255_at	At2g22950	0,97	0,99	1,02	0,91	1,07	0,97	1,11	1,06 calcium-transporting ATPase, plasma membrane-type, putative / Ca2+-ATF
267253_at	At2g22960	0,83	0,70	0,74	0,85	1,06	1,01	1,10	1,13 serine carboxypeptidase S10 family protein
267264_at	At2g22970	0,54	0,50	0,44	0,96	0,74	0,87	0,76	0,81 serine carboxypeptidase S10 family protein
267265_at	At2g22980	0,59	0,63	0,72	0,75	0,84	1,10	1,73	1,89 serine carboxypeptidase S10 family protein

267262_at	At2g22990	0,90	0,86	0,81	1,07	1,07	1,08	1,07	1,09	sinapoylglucose:malate sinapoyltransferase (SNG1)
267256_s_at	At2g23000	2,55	1,49	1,52	1,23	1,15	1,40	1,55	1,50	serine carboxypeptidase S10 family protein
267254_at	At2g23030	0,58	0,95	1,28	0,85	0,58	1,24	1,17	1,24	protein kinase, putative
267249_at	At2g23040	1,06	0,93	0,96	0,92	0,89	0,92	0,98	1,05	expressed protein
257353_at	At2g23050	1,06	1,04	0,89	1,09	1,05	0,88	0,89	0,93	phototropic-responsive NPH3 family protein
267250_at	At2g23060	1,00	0,94	1,01	0,92	0,99	0,98	0,92	0,95	GCN5-related N-acetyltransferase (GNAT) family protein
267251_at	At2g23070	0,98	0,87	0,90	0,84	0,92	0,92	0,93	1,00	casein kinase II alpha chain, putative
267257_at	At2g23080	1,01	1,00	1,28	1,05	1,09	1,23	1,21	1,22	casein kinase II alpha chain, putative
267259_at	At2g23090	1,55	1,22	1,19	1,57	1,66	0,98	0,92	0,89	expressed protein
267252_at	At2g23100	1,04	1,03	1,15	0,90	1,20	0,96	0,97	1,02	DC1 domain-containing protein
267263_at	At2g23110	0,89	0,93	0,82	1,38	1,65	1,72	1,20	1,49	expressed protein
267261_at	At2g23120	1,02	0,97	0,88	1,00	1,17	1,10	1,06	1,01	expressed protein
267260_at	At2g23130	1,43	1,36	1,60	1,32	1,32	1,05	0,80	0,80	arabinogalactan-protein (AGP17)
267258_at	At2g23140	0,94	0,91	1,08	0,97	0,91	0,91	1,04	1,05	armadillo/beta-catenin repeat family protein / U-box domain-containing prot
267266_at	At2g23150	0,88	0,83	0,88	1,28	1,18	0,83	0,77	0,95	NRAMP metal ion transporter 3 (NRAMP3)
245077_at	At2g23160	1,04	0,95	1,04	0,91	1,07	0,90	0,96	0,96	F-box family protein
245076_at	At2g23170	1,42	1,79	1,68	0,72	0,68	1,50	1,51	1,81	auxin-responsive GH3 family protein
245075_at	At2g23180	1,17	1,00	1,11	0,88	0,95	0,90	1,03	0,91	cytochrome P450, putative
245074_at	At2g23200	0,68	0,82	0,93	1,02	0,95	0,83	0,88	1,11	protein kinase family protein
245073_at	At2g23210	1,02	1,10	1,00	1,05	1,00	1,09	1,00	0,95	UDP-glucoronosyl/UDP-glucosyl transferase family protein
245072_s_at	At2g23220	0,97	0,98	0,97	0,96	0,94	1,08	0,95	0,95	cytochrome P450, putative
245071_at	At2g23230	1,00	1,01	1,03	0,90	1,05	0,98	1,00	1,14	terpene synthase/cyclase family protein
245070_at	At2g23240	0,57	0,60	0,38	0,98	1,01	1,00	0,95	0,88	plant EC metallothionein-like family 15 protein
245069_at	At2g23250	1,06	1,04	0,93	1,08	0,92	0,94	1,10	1,03	UDP-glucoronosyl/UDP-glucosyl transferase family protein
245068_at	At2g23260	0,93	1,00	1,01	1,03	1,00	1,02	0,99	1,18	UDP-glucoronosyl/UDP-glucosyl transferase family protein
245082_at	At2g23270	1,14	1,28	1,19	0,96	0,93	1,25	0,99	1,13	expressed protein
245081_at	At2g23280	0,98	1,18	0,99	0,90	0,94	0,93	1,04	0,82	---
245084_at	At2g23290	0,78	0,93	0,84	0,70	0,77	1,14	1,08	0,88	myb family transcription factor
245080_at	At2g23300	0,88	0,95	0,74	0,94	1,02	0,98	0,88	1,20	leucine-rich repeat transmembrane protein kinase, putative
245083_at	At2g23310	0,94	0,85	0,91	0,92	1,06	0,92	0,86	0,88	RER1C protein
245051_at	At2g23320	0,78	1,39	1,29	0,84	0,98	1,01	1,03	1,18	WRKY family transcription factor
245079_at	At2g23330	0,95	1,05	0,94	0,86	1,11	1,09	1,03	1,21	---
245078_at	At2g23340	0,84	0,76	0,63	1,36	1,09	1,19	1,17	0,82	AP2 domain-containing transcription factor, putative
245085_at	At2g23350	1,26	1,07	0,96	0,73	0,72	0,91	0,96	0,93	polyadenylate-binding protein, putative / PABP, putative
267182_at	At2g23360	1,01	1,02	1,14	0,96	1,09	1,24	0,97	0,88	transport protein-related
267129_at	At2g23380	1,03	1,08	1,00	0,99	1,08	1,09	0,93	1,24	curly leaf protein (CURLY LEAF) / polycomb-group protein
267130_at	At2g23390	1,22	1,25	1,34	0,71	0,79	1,45	1,44	1,49	expressed protein
267131_at	At2g23400	0,97	0,90	0,94	0,96	0,93	1,02	0,90	0,90	dehydrodolichyl diphosphate synthase, putative / DEDOL-PP synthase, put
267137_at	At2g23410	1,01	1,06	0,97	1,02	1,07	0,89	0,97	1,09	dehydrodolichyl diphosphate synthase / DEDOL-PP synthase (DPS)
267132_at	At2g23420	1,12	1,17	1,60	1,02	0,92	1,28	1,68	1,86	nicotinate phosphoribosyltransferase family protein / NAPRTase family prot
267135_at	At2g23430	1,05	1,08	0,88	0,86	1,00	1,18	1,01	0,99	kip-related protein 1 (KRP1) / cyclin-dependent kinase inhibitor 1 (ICK1)
267133_at	At2g23440	0,89	1,02	0,94	1,00	0,93	0,97	0,98	0,83	expressed protein
267134_at	At2g23450	0,75	0,88	0,99	0,95	0,89	1,00	1,01	1,06	protein kinase family protein
267136_at	At2g23460	1,13	1,18	1,13	0,98	0,96	0,86	0,99	0,92	extra-large guanine nucleotide binding protein / G-protein (XLG)
267148_at	At2g23470	0,91	1,00	1,05	0,77	0,79	1,16	1,11	1,16	expressed protein
257354_x_at	At2g23480	1,07	1,03	1,06	0,95	1,15	1,06	1,16	0,87	hypothetical protein

267149_s_at	At2g23500	0,99	0,99	0,95	1,02	0,98	0,96	0,97	0,77	---
267150_at	At2g23510	0,90	1,01	0,99	0,91	1,24	1,00	1,07	0,77	transferase family protein
267120_at	At2g23530	1,10	1,02	1,00	1,20	0,98	1,00	0,95	0,86	expressed protein
267121_at	At2g23540	1,21	1,50	1,46	1,43	1,32	1,21	1,57	1,51	GDSL-motif lipase/hydrolase family protein
267122_at	At2g23550	0,94	1,06	0,89	0,95	1,10	1,08	0,96	1,08	hydrolase, alpha/beta fold family protein
267123_at	At2g23560	0,70	0,95	1,06	1,18	1,18	0,98	1,17	0,95	hydrolase, alpha/beta fold family protein
267124_at	At2g23570	1,02	1,08	1,24	1,10	1,20	0,95	0,96	1,13	hydrolase, alpha/beta fold family protein
267125_at	At2g23580	1,05	1,03	1,03	1,01	0,95	1,06	1,01	1,03	hydrolase, alpha/beta fold family protein
267126_s_at	At2g23590	1,39	1,11	1,43	0,78	0,75	1,35	1,38	1,33	hydrolase, alpha/beta fold family protein
267127_at	At2g23610	1,26	1,14	1,08	0,98	0,98	0,83	0,74	0,70	esterase, putative
267128_at	At2g23620	0,54	0,51	0,43	1,42	1,49	1,04	0,94	0,99	esterase, putative
267287_at	At2g23630	1,16	1,33	1,20	1,66	1,48	0,97	1,10	0,99	multi-copper oxidase type I family protein
267286_at	At2g23640	0,61	0,77	0,81	0,93	0,97	1,02	0,91	1,00	reticulon family protein (RTNLB13)
267296_at	At2g23660	0,95	1,06	0,99	0,90	1,05	1,01	1,00	0,92	LOB domain family protein / lateral organ boundaries domain family protein
267294_at	At2g23670	0,96	0,78	0,77	0,85	0,88	0,96	0,82	0,68	expressed protein
267288_at	At2g23680	0,92	1,04	1,16	1,44	1,39	0,82	1,00	1,20	stress-responsive protein, putative
267285_at	At2g23690	0,82	1,09	1,16	0,95	0,97	0,79	1,09	0,93	expressed protein
267284_at	At2g23700	1,12	1,06	0,93	1,13	1,24	1,06	0,85	1,02	expressed protein
267283_at	At2g23720	1,03	0,99	1,00	1,00	1,01	1,01	0,95	1,21	---
267292_at	At2g23730	0,98	0,89	0,96	0,86	0,85	1,00	1,04	1,13	expressed protein
267291_at	At2g23740	0,92	0,94	1,09	0,91	0,79	0,94	1,08	0,96	zinc finger (C2H2 type) family protein
267290_at	At2g23750	1,04	0,79	0,99	0,77	0,64	1,10	0,89	1,40	SET domain-containing protein
267298_at	At2g23760	0,81	0,98	1,19	0,66	0,69	1,31	1,14	1,25	BEL1-like homeobox 4 protein (BLH4)
267289_at	At2g23770	0,99	0,93	0,86	0,90	1,24	1,10	1,04	0,90	protein kinase family protein / peptidoglycan-binding LysM domain-containi
267297_at	At2g23780	1,03	1,02	1,02	1,16	0,92	0,95	1,04	1,04	zinc finger (C3HC4-type RING finger) family protein
267295_at	At2g23800	1,03	0,97	1,10	0,92	1,12	1,08	0,96	1,14	geranylgeranyl pyrophosphate synthase (GGPS2) (GGPS5) / GGPP synthe
267293_at	At2g23810	0,91	1,13	1,25	0,90	0,74	1,13	1,65	1,96	senescence-associated family protein
266571_at	At2g23830	0,93	0,99	0,96	0,99	1,00	1,01	0,98	0,91	vesicle-associated membrane protein, putative / VAMP, putative
266572_at	At2g23840	1,04	1,40	2,14	0,68	0,47	1,40	1,50	1,77	HNH endonuclease domain-containing protein
266573_at	At2g23860	0,96	0,98	1,01	0,99	0,98	1,00	0,96	1,00	pseudogene, similar to VAP27
266574_at	At2g23890	1,43	1,47	1,57	1,01	0,97	1,33	1,27	1,17	5' nucleotidase family protein
266558_at	At2g23900	0,90	1,09	1,13	0,99	1,08	0,98	0,93	1,02	glycoside hydrolase family 28 protein / polygalacturonase (pectinase) family
266578_at	At2g23910	0,53	0,45	0,51	0,70	0,46	0,82	0,76	0,79	cinnamoyl-CoA reductase-related
266559_at	At2g23920	1,03	1,12	1,00	1,05	1,00	1,02	1,06	1,01	hypothetical protein
266579_at	At2g23930	1,66	1,32	1,15	1,10	1,11	0,95	1,01	0,89	small nuclear ribonucleoprotein G, putative / snRNP-G, putative / Sm protei
266576_at	At2g23940	1,09	0,95	0,89	0,88	1,10	0,98	0,92	0,90	expressed protein
266577_at	At2g23945	0,99	1,05	1,13	0,97	0,96	0,85	0,97	0,92	chloroplast nucleoid DNA-binding protein-related
266560_at	At2g23950	0,92	1,10	1,14	0,81	0,83	1,01	1,14	1,22	leucine-rich repeat family protein / protein kinase family protein
266561_at	At2g23960	1,05	1,08	1,08	1,23	1,17	0,89	1,01	1,15	defense-related protein, putative
266562_at	At2g23970	0,89	0,94	0,88	0,91	1,04	0,98	1,09	0,96	defense-related protein, putative
266520_at	At2g23980	0,95	0,77	0,69	1,04	0,83	0,97	1,05	1,14	cyclic nucleotide-regulated ion channel / cyclic nucleotide-gated channel (C
266563_at	At2g23990	1,01	0,98	1,01	1,04	0,83	0,97	1,01	1,12	plastocyanin-like domain-containing protein
266564_at	At2g24000	0,95	0,99	1,07	0,92	1,27	1,02	1,03	0,92	serine carboxypeptidase S10 family protein
266565_at	At2g24010	1,04	1,01	1,02	0,95	1,13	0,83	1,01	0,94	serine carboxypeptidase S10 family protein
266521_at	At2g24020	1,14	0,95	0,80	1,09	1,08	1,04	0,95	1,02	expressed protein
266566_at	At2g24040	0,83	0,79	0,85	1,30	1,05	0,76	0,76	0,71	hydrophobic protein, putative / low temperature and salt responsive protein,

266567_at	At2g24050	1,15	0,81	0,81	0,95	0,73	1,13	0,82	0,70	MIF4G domain-containing protein / MA3 domain-containing protein
266575_at	At2g24060	1,23	1,00	0,90	0,71	0,82	1,11	1,23	1,18	translation initiation factor 3 (IF-3) family protein
266568_at	At2g24070	0,93	1,09	0,89	1,10	1,26	0,94	1,07	0,98	expressed protein
266569_at	At2g24080	1,00	0,89	1,08	0,86	1,06	0,96	0,94	1,21	F-box family protein-related
266570_at	At2g24090	1,15	1,01	0,95	0,94	0,80	1,05	0,95	0,83	ribosomal protein L35 family protein
265999_at	At2g24100	0,95	2,02	4,29	0,93	1,01	1,37	2,28	3,80	expressed protein
265991_at	At2g24120	0,95	1,24	1,15	0,61	0,55	0,84	1,18	1,12	DNA-directed RNA polymerase, chloroplast (RPOPT)
265992_at	At2g24130	1,02	1,23	1,19	0,97	0,79	1,35	1,25	1,09	leucine-rich repeat transmembrane protein kinase, putative
265995_at	At2g24140	0,99	1,05	0,97	0,99	0,95	1,01	1,05	1,07	expressed protein
266001_at	At2g24150	1,07	1,37	1,17	1,05	0,95	0,97	0,84	0,94	expressed protein
265993_at	At2g24160	0,92	0,89	0,88	0,59	0,81	1,04	0,99	1,13	pseudogene, leucine rich repeat protein family
265994_at	At2g24170	0,93	0,83	0,66	1,27	0,95	0,70	0,73	0,69	endomembrane protein 70, putative
266000_at	At2g24180	0,97	1,21	1,16	0,91	0,85	1,24	1,22	1,18	cytochrome P450 family protein
266015_at	At2g24190	1,15	1,23	1,09	1,38	1,38	1,30	1,54	1,40	short-chain dehydrogenase/reductase (SDR) family protein
265996_at	At2g24200	0,94	0,91	0,95	1,01	1,05	0,93	0,93	0,98	cytosol aminopeptidase
265984_at	At2g24210	0,98	1,00	1,07	1,08	1,07	1,22	1,01	0,86	myrcene/ocimene synthase (TPS10)
265985_at	At2g24220	0,88	0,89	1,02	1,07	1,01	0,72	0,93	0,92	purine permease-related
265986_at	At2g24230	1,02	0,93	0,93	0,80	1,06	1,10	1,01	0,99	leucine-rich repeat transmembrane protein kinase, putative
265987_at	At2g24240	0,77	0,91	0,84	0,91	0,79	1,00	1,00	1,00	potassium channel tetramerisation domain-containing protein
265997_at	At2g24250	1,11	1,04	1,08	1,03	0,92	0,88	0,94	1,00	F-box family protein
265988_at	At2g24255	1,10	0,97	0,97	1,02	1,07	0,94	0,94	1,02	hypothetical protein
265989_at	At2g24260	1,15	1,22	1,25	1,24	1,19	1,08	1,14	0,91	basic helix-loop-helix (bHLH) family protein
265998_at	At2g24270	0,96	0,95	1,05	0,94	0,91	1,13	1,08	1,09	NADP-dependent glyceraldehyde-3-phosphate dehydrogenase, putative
265990_at	At2g24280	0,98	0,92	0,94	1,18	1,17	1,26	1,51	1,36	serine carboxypeptidase S28 family protein
265663_at	At2g24290	1,08	1,21	1,19	1,00	1,21	0,87	0,79	0,90	expressed protein
265688_at	At2g24300	1,03	0,91	0,91	1,29	1,04	0,99	0,90	1,02	calmodulin-binding protein
265689_at	At2g24310	1,08	0,96	0,95	1,02	1,02	1,00	1,01	0,83	expressed protein
265690_at	At2g24320	0,98	0,56	1,38	0,85	0,98	1,08	1,03	1,21	hypothetical protein
265691_at	At2g24330	0,92	0,83	1,38	1,10	1,10	1,01	1,07	1,42	expressed protein
265692_at	At2g24340	0,96	1,02	1,05	1,04	1,11	0,95	1,02	0,99	hypothetical protein
265693_at	At2g24350	1,11	0,99	1,08	0,99	1,30	1,01	0,91	0,91	RNA recognition motif (RRM)-containing protein
265661_at	At2g24360	1,02	0,90	1,00	1,02	1,08	1,02	0,97	1,00	serine/threonine/tyrosine kinase, putative
265681_at	At2g24370	0,96	1,09	1,00	1,10	1,16	0,96	0,87	0,94	protein kinase family protein
265682_at	At2g24390	1,00	0,89	0,84	1,08	1,08	1,09	0,95	0,89	avirulence-responsive protein-related / avirulence induced gene (AIG) prote
265683_at	At2g24400	0,91	1,04	1,02	0,91	0,93	1,12	0,96	1,07	auxin-responsive protein, putative / small auxin up RNA (SAUR_D)
265684_at	At2g24410	0,90	0,96	1,06	1,01	0,89	0,99	0,98	0,91	hypothetical protein
265664_at	At2g24420	0,98	1,02	0,98	0,82	0,80	1,10	1,03	1,06	DNA repair ATPase-related
265685_at	At2g24430	1,02	1,26	1,14	0,85	1,17	1,07	1,11	1,12	no apical meristem (NAM) family protein
265694_at	At2g24440	1,08	0,89	0,85	1,01	0,80	1,06	0,99	0,97	expressed protein
257392_at	At2g24450	0,97	0,85	0,93	1,01	1,28	0,98	1,06	0,91	fasciclin-like arabinogalactan family protein
265686_at	At2g24460	0,92	1,08	1,03	1,01	1,10	0,99	0,92	1,12	expressed protein
265687_s_at	At2g24470	1,01	0,95	1,01	1,12	1,04	1,08	1,00	1,05	hypothetical protein
265695_at	At2g24490	1,09	0,99	1,05	1,27	1,32	0,92	1,07	1,19	replication protein, putative
265662_at	At2g24500	0,85	0,85	1,09	0,92	0,93	0,92	0,89	0,82	zinc finger (C2H2 type) family protein
263791_at	At2g24520	0,97	0,75	0,69	0,78	0,81	0,94	0,88	0,86	ATPase, plasma membrane-type, putative / proton pump, putative
263790_at	At2g24530	0,86	1,00	1,02	0,87	0,93	0,96	1,27	0,90	expressed protein

263796_at	At2g24540	0,87	0,96	0,97	1,22	1,15	1,06	1,00	1,20	kelch repeat-containing F-box family protein
263799_at	At2g24550	1,07	1,39	1,21	0,84	0,78	1,25	1,69	1,51	expressed protein
263789_at	At2g24560	0,96	0,93	0,93	1,01	1,10	1,36	1,19	0,97	GDSL-motif lipase/hydrolase family protein
263797_at	At2g24570	1,10	0,96	0,68	0,87	1,23	1,00	0,88	1,06	WRKY family transcription factor
263788_at	At2g24580	1,37	1,11	1,10	1,20	0,95	0,93	0,87	0,93	sarcosine oxidase family protein
257435_at	At2g24590	1,80	1,68	1,47	1,15	0,93	1,18	1,15	1,12	splicing factor, putative
263800_at	At2g24600	0,87	0,79	1,24	0,84	0,96	1,08	1,01	1,06	ankyrin repeat family protein
263795_at	At2g24610	0,88	1,06	0,99	1,25	1,16	1,02	0,94	0,96	cyclic nucleotide-regulated ion channel, putative (CNGC14)
263794_at	At2g24620	0,97	1,01	0,96	1,01	1,14	0,98	1,11	0,90	S-locus glycoprotein family protein
263793_at	At2g24630	0,89	0,97	0,78	0,67	1,01	0,87	0,98	1,10	glycosyl transferase family 2 protein
263798_at	At2g24640	1,02	1,03	0,94	1,02	1,34	0,94	0,96	1,11	ubiquitin carboxyl-terminal hydrolase family protein / zinc finger (MYND type
257436_s_at	At2g24650	0,83	0,91	0,89	0,91	1,04	0,94	0,86	1,05	transcriptional factor B3 family protein
263792_at	At2g24660	1,00	1,12	1,07	0,96	1,18	0,98	0,89	0,90	---
263316_s_at	At2g24710	1,01	0,97	0,94	0,87	1,06	1,09	1,14	0,97	glutamate receptor family protein (GLR2.3)
263317_s_at	At2g24740	0,87	1,26	1,02	1,11	0,97	1,02	1,00	1,16	SET domain-containing protein (SUVH7)
263318_at	At2g24762	0,94	0,93	0,96	1,29	1,13	1,00	0,89	1,04	expressed protein
263529_at	At2g24765	1,33	1,09	1,05	1,06	1,13	1,11	1,11	1,05	ADP-ribosylation factor 3 (ARF3)
263540_at	At2g24780	1,00	1,01	1,02	0,98	0,96	1,07	1,03	1,10	hypothetical protein
263537_at	At2g24790	0,90	1,01	0,94	0,96	0,97	1,05	1,05	1,24	zinc finger (B-box type) family protein
263528_at	At2g24800	0,92	0,92	1,05	0,93	0,90	1,03	0,94	1,00	peroxidase, putative
263527_at	At2g24810	1,03	1,00	1,07	1,02	1,03	1,05	0,97	0,77	pathogenesis-related thaumatin family protein
263533_at	At2g24820	0,76	0,68	1,01	0,77	0,68	0,94	0,87	0,93	Rieske (2Fe-2S) domain-containing protein
263526_at	At2g24830	1,11	1,21	1,17	1,08	1,03	1,11	1,16	1,32	zinc finger (CCCH-type) family protein / D111/G-patch domain-containing p
263525_at	At2g24840	1,00	1,00	1,05	0,89	1,31	0,92	0,88	0,89	MADS-box family protein
263539_at	At2g24850	0,80	0,75	0,88	1,42	1,38	1,75	1,61	1,76	aminotransferase, putative
263541_at	At2g24860	0,91	0,84	0,72	0,94	0,87	0,95	0,77	0,84	chaperone protein dnaJ-related
257440_at	At2g24880	1,06	0,92	0,97	1,01	0,92	0,99	1,08	0,97	self-incompatibility protein-related
263524_x_at	At2g24920	0,93	0,95	1,16	1,07	1,00	0,98	0,97	1,27	expressed protein
263523_at	At2g24930	0,97	0,95	1,00	1,09	1,03	0,98	0,94	0,98	Ulp1 protease family protein
263534_at	At2g24940	1,34	1,18	1,17	1,81	1,69	0,91	0,87	0,97	cytochrome b5 domain-containing protein
263522_at	At2g24950	0,90	1,14	0,95	1,01	1,12	1,07	1,12	1,15	hypothetical protein
263521_at	At2g24960	1,13	1,15	0,94	0,93	0,84	1,07	1,12	1,31	expressed protein
263535_at	At2g24970	1,24	0,99	0,90	1,15	1,08	0,94	1,00	0,83	expressed protein
263552_x_at	At2g24980	1,26	1,27	0,89	1,17	1,08	1,12	1,01	1,01	proline-rich extensin-like family protein
263536_at	At2g25000	1,18	1,30	1,05	1,49	1,30	1,12	1,17	1,25	WRKY family transcription factor
263538_at	At2g25010	1,08	1,09	1,22	0,92	0,99	0,98	1,04	1,16	expressed protein
263530_at	At2g25030	1,01	0,96	1,11	0,97	1,02	0,98	1,08	1,00	---
264377_at	At2g25060	1,33	1,15	1,16	1,61	1,24	0,86	0,87	0,82	plastocyanin-like domain-containing protein
264376_at	At2g25070	1,26	1,44	1,75	1,04	1,02	1,17	1,20	1,14	protein phosphatase 2C, putative / PP2C, putative
264383_at	At2g25080	1,19	1,18	1,26	1,12	1,22	1,16	1,05	1,01	phospholipid hydroperoxide glutathione peroxidase, chloroplast / PHGPx (G
264375_at	At2g25090	2,26	1,78	1,27	0,95	0,95	1,07	1,21	1,16	CBL-interacting protein kinase 16 (CIPK16)
264381_at	At2g25100	1,05	1,18	0,97	0,99	0,95	0,87	0,89	1,10	ribonuclease HII family protein
264382_at	At2g25110	1,24	1,25	1,30	1,16	1,25	0,96	0,98	1,02	MIR domain-containing protein
264402_at	At2g25140	1,37	1,63	1,35	1,12	1,16	1,14	1,21	1,26	heat shock protein 100, putative / HSP100, putative / heat shock protein clp
264403_at	At2g25150	0,98	0,97	0,95	0,99	1,03	1,06	0,90	0,98	transferase family protein
264404_at	At2g25160	1,02	1,36	1,12	1,36	0,99	0,92	1,00	0,88	cytochrome P450, putative

264384_at	At2g25170	1,27	1,06	1,17	1,05	0,95	1,23	1,20	1,48 chromatin remodeling factor CHD3 (PICKLE)
264374_at	At2g25180	1,04	0,99	0,93	1,02	1,04	1,08	1,04	0,99 two-component responsive regulator family protein / response regulator fam
264380_at	At2g25190	0,91	0,79	1,01	1,02	1,15	0,93	0,93	0,99 expressed protein
264379_at	At2g25200	0,68	1,05	0,94	0,84	0,63	0,96	1,13	0,88 expressed protein
263585_at	At2g25210	1,34	1,18	1,04	1,52	1,48	0,92	0,94	0,96 60S ribosomal protein L39 (RPL39A)
264378_at	At2g25220	1,10	1,00	1,42	1,11	0,93	1,11	1,14	1,03 protein kinase family protein
263615_at	At2g25230	1,03	1,08	1,10	0,93	1,02	0,99	0,97	0,92 myb family transcription factor (MYB100)
263614_at	At2g25240	0,99	1,11	1,09	1,06	0,91	0,91	0,99	0,94 serpin, putative / serine protease inhibitor, putative
263613_at	At2g25250	1,02	1,83	1,66	1,44	1,85	1,01	1,09	1,24 expressed protein
263640_at	At2g25270	0,89	1,04	1,00	1,32	1,33	0,81	0,90	0,86 expressed protein
263589_at	At2g25280	1,09	1,13	0,96	1,11	1,17	0,83	0,91	0,87 expressed protein
263639_at	At2g25300	1,03	1,06	1,13	0,89	0,70	1,12	1,11	1,21 galactosyltransferase family protein
263638_at	At2g25310	1,20	1,17	0,95	0,86	0,87	0,97	0,87	0,79 expressed protein
263588_s_at	At2g25330	0,96	1,14	1,10	0,92	0,95	1,02	0,99	0,96 meprin and TRAF homology domain-containing protein / MATH domain-cor
263587_at	At2g25340	1,02	0,98	0,96	1,04	1,03	0,81	1,03	0,88 synaptobrevin family protein
263586_at	At2g25350	0,99	0,95	0,96	0,81	1,12	0,99	1,20	0,78 phox (PX) domain-containing protein
265614_at	At2g25355	1,46	1,44	1,51	1,01	1,11	1,09	0,95	0,99 exonuclease-related
265635_at	At2g25360	1,00	0,95	0,93	0,93	0,92	0,99	1,02	1,00 zinc finger protein-related
265607_at	At2g25370	1,01	1,03	1,04	1,10	0,91	0,96	1,08	1,01 zinc finger protein-related
265608_at	At2g25380	0,96	1,01	0,96	0,86	1,02	1,14	1,03	0,97 ---
265612_at	At2g25390	1,01	0,94	1,06	1,07	1,00	1,01	1,12	1,11 Protein coding
265609_at	At2g25420	0,86	0,84	1,07	0,89	0,93	0,88	0,95	0,82 transducin family protein / WD-40 repeat family protein
265610_at	At2g25430	0,89	0,87	1,03	0,96	1,07	0,90	0,86	0,99 epsin N-terminal homology (ENTH) domain-containing protein
265659_at	At2g25440	1,07	1,00	0,79	0,88	0,96	1,00	1,06	0,93 leucine-rich repeat family protein
265615_at	At2g25450	0,56	0,64	0,88	1,04	1,06	1,09	1,01	1,02 2-oxoglutarate-dependent dioxygenase, putative
265618_at	At2g25460	0,81	1,29	1,16	0,64	0,57	1,74	1,72	1,32 expressed protein
265660_at	At2g25470	1,00	1,04	0,99	1,09	1,02	1,11	0,97	0,77 leucine-rich repeat family protein
265633_at	At2g25490	0,82	1,13	0,85	0,77	0,72	0,81	1,11	1,17 F-box family protein (FBL6)
265616_at	At2g25500	0,94	1,09	1,13	1,03	0,89	0,94	1,03	1,12 expressed protein
265611_at	At2g25510	0,75	0,67	0,71	2,91	2,12	3,85	3,17	3,05 expressed protein
265617_at	At2g25520	0,76	0,65	0,58	1,19	1,10	0,81	0,67	0,68 phosphate translocator-related
265634_at	At2g25530	1,14	1,33	0,94	0,96	0,96	1,22	1,17	0,82 AFG1-like ATPase family protein
265605_at	At2g25540	0,91	0,99	1,00	1,01	1,22	1,04	0,98	0,90 cellulose synthase, catalytic subunit, putative
265606_s_at	At2g25550	0,95	1,07	1,02	1,02	1,00	0,95	1,10	1,16 non-LTR retrotransposon family (LINE)
265613_at	At2g25560	0,94	1,02	1,18	0,87	0,95	0,91	1,02	0,94 DNAJ heat shock N-terminal domain-containing protein
265906_at	At2g25565	1,02	0,95	1,07	0,80	0,98	1,28	0,91	0,97 hypothetical protein
265912_at	At2g25570	1,30	1,35	1,36	0,92	1,15	1,14	1,14	1,20 expressed protein
265901_at	At2g25580	0,98	0,94	0,97	0,89	0,93	0,97	1,01	0,88 pentatricopeptide (PPR) repeat-containing protein
265902_at	At2g25590	0,89	0,88	1,49	1,06	1,00	0,86	0,99	1,18 agenet domain-containing protein
265903_at	At2g25600	1,02	1,02	0,84	0,92	1,00	0,97	0,90	0,83 potassium channel protein, putative
265910_at	At2g25610	1,01	0,90	0,85	1,08	1,03	1,06	0,94	0,94 H+-transporting two-sector ATPase, C subunit family protein
265886_at	At2g25620	0,83	0,90	0,86	0,92	0,65	1,06	0,94	0,97 protein phosphatase 2C, putative / PP2C, putative
265913_at	At2g25625	1,18	1,30	1,32	0,96	1,09	1,03	1,16	0,90 expressed protein
265904_at	At2g25630	1,03	1,09	1,06	1,13	1,01	1,17	0,88	0,94 glycosyl hydrolase family 1 protein
265905_at	At2g25640	1,19	0,97	0,94	1,05	1,14	1,04	0,91	1,27 transcription elongation factor-related
265907_at	At2g25650	1,00	1,04	0,98	0,92	1,04	1,06	0,92	1,28 DNA-binding storekeeper protein-related

265896_at	At2g25660	0,94	0,90	0,91	0,80	0,80	1,10	1,20	1,11	expressed protein
265911_at	At2g25670	0,89	0,79	0,89	1,13	1,17	1,04	1,07	0,95	expressed protein
265897_at	At2g25680	1,11	0,97	0,91	1,50	1,23	0,89	0,91	0,81	expressed protein
265898_at	At2g25690	0,97	1,00	1,26	1,88	1,80	0,90	1,28	1,25	senescence-associated protein-related
265899_s_at	At2g25700	1,50	1,31	1,53	1,24	1,19	1,24	1,05	1,23	E3 ubiquitin ligase SCF complex subunit, putative
265887_at	At2g25710	1,18	1,21	1,38	0,96	0,92	1,22	1,22	1,21	holocarboxylase synthetase 1 (HCS1)
265909_at	At2g25720	1,19	0,83	1,09	1,18	1,18	0,78	1,23	1,04	expressed protein
265900_at	At2g25730	0,68	0,63	0,65	0,75	0,80	1,05	1,06	0,96	expressed protein
266658_at	At2g25735	0,84	0,69	0,72	0,85	0,91	0,88	1,03	0,97	expressed protein
266653_at	At2g25740	0,97	0,95	1,24	0,87	1,07	1,01	1,04	1,09	ATP-dependent protease La (LON) domain-containing protein
266652_at	At2g25750	0,59	0,55	0,65	0,96	1,00	0,81	0,82	0,91	protein kinase family protein
266651_at	At2g25760	0,57	0,62	0,66	0,92	0,99	0,67	0,80	0,88	protein kinase family protein
257367_at	At2g25780	1,01	1,07	0,95	0,80	0,94	0,96	1,03	0,95	hypothetical protein
266663_at	At2g25790	1,00	1,11	0,94	1,20	0,94	1,02	1,03	1,30	leucine-rich repeat transmembrane protein kinase, putative
266650_at	At2g25800	0,90	0,77	1,01	0,92	0,90	0,92	0,92	1,07	expressed protein
266649_at	At2g25810	1,26	1,17	1,13	1,35	1,09	0,92	1,01	1,10	tonoplast intrinsic protein, putative
266661_at	At2g25820	1,00	1,11	0,97	1,11	0,89	1,07	1,05	0,83	transcription factor, putative
266662_at	At2g25830	1,26	1,00	0,81	0,98	1,01	1,20	1,10	1,03	YebC-related
266648_at	At2g25840	0,89	0,78	0,69	0,71	0,72	1,01	0,99	1,02	tRNA synthetase class I (W and Y) family protein
266659_at	At2g25850	0,65	0,56	0,64	0,89	1,03	0,83	0,97	0,97	nucleotidyltransferase family protein
266647_at	At2g25860	0,96	0,95	0,88	0,99	0,81	1,08	1,04	0,89	haloacid dehalogenase-like hydrolase family protein
266678_at	At2g25870	0,95	0,96	0,83	0,80	0,75	1,07	1,12	1,12	haloacid dehalogenase-like hydrolase family protein
266655_at	At2g25880	1,03	1,15	1,25	1,41	1,33	0,95	0,91	1,25	serine/threonine protein kinase, putative
266654_at	At2g25890	0,15	0,18	0,27	1,02	0,86	1,11	1,14	0,98	glycine-rich protein / oleosin
266656_at	At2g25900	0,91	1,34	1,11	0,88	0,65	0,64	1,12	1,09	zinc finger (CCCH-type) family protein
266657_at	At2g25910	1,32	1,38	1,24	0,96	0,97	1,17	1,17	1,33	3'-5' exonuclease domain-containing protein / K homology domain-containir
266660_at	At2g25920	0,97	0,93	0,86	0,85	1,05	0,96	1,05	0,93	expressed protein
266839_at	At2g25930	0,73	0,85	1,14	0,61	0,70	1,14	1,01	1,61	hydroxyproline-rich glycoprotein family protein
266849_at	At2g25940	1,26	1,44	1,40	1,03	0,88	1,09	1,00	1,09	vacuolar processing enzyme alpha / alpha-VPE
266848_at	At2g25950	0,72	0,78	0,77	0,84	0,80	0,85	0,95	0,67	expressed protein
266846_at	At2g25970	1,07	0,95	0,95	0,97	0,95	0,94	0,99	1,06	KH domain-containing protein
266838_at	At2g25980	0,85	0,76	0,54	1,27	1,08	0,90	0,99	1,01	jacalin lectin family protein
266837_x_at	At2g25990	1,08	1,00	1,03	1,03	1,02	1,11	1,16	0,90	hypothetical protein
266836_at	At2g26000	0,94	0,99	1,10	0,93	1,05	1,25	1,03	1,19	zinc finger (C3HC4-type RING finger) family protein
257365_x_at	At2g26020	1,20	0,85	1,39	1,02	0,77	0,67	0,51	0,44	plant defensin-fusion protein, putative (PDF1.2b)
266895_at	At2g26040	1,34	1,10	0,94	0,98	1,06	1,07	0,96	0,88	Bet v I allergen family protein
266894_at	At2g26050	0,88	1,10	0,94	0,97	1,11	1,07	0,97	0,85	hypothetical protein
266847_at	At2g26060	0,97	0,90	0,97	0,92	0,85	1,08	0,93	0,86	transducin family protein / WD-40 repeat family protein
266893_at	At2g26070	0,78	1,00	1,15	1,01	1,12	0,94	0,89	0,90	expressed protein
266892_at	At2g26080	1,58	1,46	1,42	0,98	0,89	1,58	1,51	1,35	glycine dehydrogenase (decarboxylating), putative / glycine decarboxylase,
266845_at	At2g26110	0,91	0,89	0,80	1,17	1,34	0,93	0,83	0,91	expressed protein
266844_at	At2g26120	1,02	1,05	1,02	1,07	1,13	0,85	0,94	0,85	glycine-rich protein
266843_at	At2g26130	0,95	0,91	0,98	1,11	1,10	0,97	0,98	0,94	zinc finger (C3HC4-type RING finger) family protein
266842_at	At2g26140	1,19	1,06	1,05	1,17	0,87	1,03	0,93	0,89	FtsH protease, putative
266841_at	At2g26150	1,61	1,55	1,05	1,07	1,10	1,26	1,07	1,06	heat shock transcription factor family protein
266840_at	At2g26160	0,97	1,05	0,93	1,14	1,07	1,03	0,92	1,10	F-box family protein

267380_at	At2g26170	1,26	0,99	0,85	1,46	1,58	1,07	1,01	0,95	thromboxane-A synthase, putative / cytochrome P450 family protein
267402_at	At2g26180	1,11	1,28	1,10	1,29	1,29	0,95	0,86	0,79	calmodulin-binding family protein
267381_at	At2g26190	0,89	2,12	1,40	0,71	0,93	1,22	1,30	1,32	calmodulin-binding family protein
267378_at	At2g26200	1,13	1,25	1,29	0,89	0,76	0,99	1,03	0,99	expressed protein
267401_at	At2g26210	0,97	0,92	0,90	1,06	1,20	1,12	1,00	1,11	ankyrin repeat family protein
267374_at	At2g26230	1,14	1,14	1,17	0,94	1,16	0,96	0,95	0,92	uricase / urate oxidase / nodulin 35, putative
267400_at	At2g26240	0,91	0,90	0,76	1,16	0,93	0,84	0,82	0,83	expressed protein
267377_at	At2g26250	0,99	1,00	1,28	0,91	0,87	0,85	0,83	1,01	beta-ketoacyl-CoA synthase family (FIDDLEHEAD) (FDH)
267434_at	At2g26260	1,02	0,84	0,90	1,00	1,07	0,90	0,87	0,85	3-beta hydroxysteroid dehydrogenase/isomerase family protein
267373_at	At2g26280	0,97	0,90	0,93	0,96	0,89	1,11	1,11	1,04	smr (Small MutS Related) domain-containing protein
267372_at	At2g26290	1,32	1,77	1,38	1,30	1,26	1,02	1,47	1,68	protein kinase, putative
267375_at	At2g26300	0,90	0,90	1,12	1,01	1,08	0,90	1,03	1,10	guanine nucleotide binding protein (G-protein) alpha-1 subunit / GP-alpha-1
267403_at	At2g26320	0,99	0,90	0,96	1,12	0,96	1,01	0,97	0,88	MADS-box protein (AGL33)
267376_at	At2g26330	0,74	0,79	0,96	0,93	0,95	0,80	0,80	0,83	leucine-rich repeat protein kinase, putative (ERECTA)
267379_at	At2g26340	0,91	0,87	0,88	0,94	0,97	1,27	1,11	1,16	expressed protein
267433_at	At2g26350	1,13	1,23	1,22	0,96	0,98	1,06	1,31	1,12	zinc-binding peroxisomal integral membrane protein (PEX10)
245031_at	At2g26360	0,97	0,93	0,94	1,11	1,10	0,90	1,21	0,86	mitochondrial substrate carrier family protein
245033_at	At2g26380	1,18	1,14	0,90	1,22	1,15	1,21	1,56	1,38	disease resistance protein-related / LRR protein-related
245034_at	At2g26390	0,75	0,71	0,76	1,28	1,18	1,03	1,23	1,07	serpin, putative / serine protease inhibitor, putative
245035_at	At2g26400	0,88	0,95	0,86	1,82	1,39	1,11	1,18	1,04	acireductone dioxygenase (ARD/ARD') family protein
245036_at	At2g26410	0,92	1,01	0,96	0,98	0,94	0,94	1,02	0,96	calmodulin-binding family protein
245037_at	At2g26420	0,98	1,01	0,97	1,08	0,95	0,99	1,01	0,91	1-phosphatidylinositol-4-phosphate 5-kinase, putative / PIP kinase, putative
245043_at	At2g26430	1,12	1,06	1,40	0,85	0,85	1,06	0,96	1,04	ania-6a type cyclin (RCY1)
245052_at	At2g26440	0,75	0,63	0,65	1,90	2,03	0,76	0,60	0,61	pectinesterase family protein
245053_at	At2g26450	1,04	0,91	1,03	1,02	1,01	1,01	0,93	1,18	pectinesterase family protein
245054_at	At2g26460	1,35	1,08	0,98	1,00	0,97	1,21	0,86	1,18	RED family protein
245055_at	At2g26470	0,93	0,97	0,97	0,69	0,72	0,87	0,92	1,42	expressed protein
245056_at	At2g26480	1,10	1,13	0,94	1,08	1,22	1,11	0,99	1,24	UDP-glucoronosyl/UDP-glucosyl transferase family protein
245057_at	At2g26490	1,02	0,96	1,02	0,92	1,04	0,97	1,06	1,07	transducin family protein / WD-40 repeat family protein
245044_at	At2g26500	1,05	1,05	1,01	0,91	0,93	0,93	0,92	0,88	cytochrome b6f complex subunit (petM), putative
245046_at	At2g26510	0,78	0,83	0,89	0,94	0,93	0,98	0,98	1,07	xanthine/uracil permease family protein
245040_at	At2g26520	0,93	1,09	1,16	1,10	1,01	0,98	1,11	0,89	expressed protein
245041_at	At2g26530	0,94	1,05	0,98	0,73	0,77	1,16	1,31	1,44	expressed protein
245042_at	At2g26540	0,65	0,66	0,73	0,90	0,98	0,94	0,92	0,96	uroporphyrinogen-III synthase family protein
245027_at	At2g26550	1,09	1,20	1,42	0,84	0,68	1,02	1,18	1,17	heme oxygenase 2 (HO2)
245038_at	At2g26560	0,84	0,90	1,47	0,73	0,59	0,93	0,81	1,13	patatin, putative
245028_at	At2g26570	1,16	1,37	1,67	0,91	1,06	1,14	1,08	1,19	expressed protein
245029_at	At2g26580	1,28	1,06	1,17	0,95	0,96	1,00	0,89	0,96	plant-specific transcription factor YABBY family protein
245045_at	At2g26590	1,04	0,89	1,05	0,98	1,19	1,03	1,08	1,08	adhesion regulating molecule family
245039_at	At2g26600	1,03	0,94	0,90	1,44	1,36	1,23	1,17	1,12	glycosyl hydrolase family 17 protein
245030_at	At2g26620	0,92	1,03	1,04	1,06	0,95	1,05	0,95	0,97	glycoside hydrolase family 28 protein / polygalacturonase (pectinase) family
245032_at	At2g26630	1,01	1,02	0,88	0,98	1,14	1,05	1,02	1,12	transposase IS4 family protein
267606_at	At2g26640	0,92	0,94	0,75	1,11	1,07	0,89	0,77	0,89	beta-ketoacyl-CoA synthase, putative
267610_at	At2g26650	0,83	0,88	0,84	0,72	0,84	0,91	1,01	1,24	potassium channel protein 1 (AKT1)
267611_at	At2g26660	1,53	1,25	1,20	0,98	1,10	1,08	0,98	0,97	SPX (SYG1/Pho81/XPR1) domain-containing protein
267617_at	At2g26670	0,86	0,83	0,84	0,99	0,90	0,99	0,92	0,90	heme oxygenase 1 (HO1) (HY1)

267616_at	At2g26680	0,86	0,74	0,77	1,12	1,26	0,92	0,73	0,59	expressed protein
267612_at	At2g26690	1,50	1,64	1,82	1,44	1,23	1,10	1,11	1,10	nitrate transporter (NTP2)
267613_at	At2g26700	1,20	1,04	0,98	1,00	1,00	0,91	1,06	1,12	protein kinase family protein
267614_at	At2g26710	1,04	1,17	1,16	0,72	1,08	1,68	1,32	1,32	cytochrome P450, putative
267615_at	At2g26720	0,98	1,01	1,05	1,05	1,07	0,90	0,88	0,94	plastocyanin-like domain-containing protein / mavicyanin, putative
267619_at	At2g26730	1,16	0,82	0,79	0,88	0,68	0,86	0,68	0,69	leucine-rich repeat transmembrane protein kinase, putative
267607_s_at	At2g26740	0,82	0,83	0,92	0,99	1,07	0,86	0,82	0,86	epoxide hydrolase, soluble (sEH)
267618_at	At2g26760	1,11	1,02	1,24	2,14	1,75	0,96	0,76	1,03	cyclin, putative
267608_at	At2g26770	1,04	0,98	1,25	0,96	0,93	1,02	1,13	1,13	plectin-related
267609_at	At2g26780	0,79	0,75	0,84	0,81	0,76	0,99	0,94	0,92	expressed protein
266853_at	At2g26790	1,20	0,88	0,81	1,02	0,88	0,86	0,92	0,84	pentatricopeptide (PPR) repeat-containing protein
266864_at	At2g26800	1,41	1,35	1,46	1,14	1,05	1,22	1,25	1,47	hydroxymethylglutaryl-CoA lyase, putative / 3-hydroxy-3-methylglutarate-Co
266852_at	At2g26810	1,02	1,01	0,86	1,17	0,92	1,04	0,98	1,17	expressed protein
266851_at	At2g26820	1,03	1,08	0,94	1,04	1,04	0,98	1,15	1,09	avirulence-responsive family protein / avirulence induced gene (AIG1) famil
266861_at	At2g26830	1,37	0,87	0,96	0,90	0,92	1,04	0,94	0,87	choline/ethanolamine kinase family protein
266850_at	At2g26850	1,00	0,94	0,98	0,96	0,95	1,04	0,99	0,99	F-box family protein
266860_at	At2g26870	1,18	1,41	1,18	1,48	1,48	1,20	1,05	1,00	phosphoesterase family protein
266859_at	At2g26880	0,99	1,08	1,03	0,96	1,18	0,96	1,12	0,98	MADS-box family protein
266858_at	At2g26890	0,84	0,84	0,81	0,89	0,86	1,01	1,06	0,89	DNAJ heat shock N-terminal domain-containing protein
266857_at	At2g26900	0,82	0,76	0,84	0,92	0,87	0,89	0,88	0,82	bile acid:sodium symporter family protein
266856_at	At2g26910	0,94	1,14	1,09	0,76	0,80	1,04	1,03	1,10	ABC transporter family protein
266855_at	At2g26920	1,09	1,01	0,91	0,92	0,97	0,90	1,05	0,94	ubiquitin-associated (UBA)/TS-N domain-containing protein
266863_at	At2g26930	0,89	0,86	0,76	0,74	0,88	1,04	1,05	1,00	4-diphosphocytidyl-2-C-methyl-D-erythritol kinase (CMK) (CDPMEK)
266854_at	At2g26940	0,96	1,21	1,02	1,00	1,04	1,02	1,05	1,14	zinc finger (C2H2 type) family protein
266862_at	At2g26950	0,92	0,94	1,05	1,06	0,94	1,15	0,93	1,01	myb family transcription factor
266301_at	At2g26960	0,95	0,98	1,08	0,96	1,17	1,06	0,86	0,92	myb family transcription factor (MYB81)
266306_at	At2g26970	1,04	1,07	1,25	0,86	0,97	1,14	1,07	0,99	exonuclease family protein
266313_at	At2g26980	0,92	1,50	1,69	0,57	0,51	1,34	1,43	1,39	CBL-interacting protein kinase 3 (CIPK3)
266310_at	At2g26990	1,38	1,26	1,72	1,11	1,17	1,14	1,11	1,38	COP9 signalosome complex subunit 2 / CSN complex subunit 2 (CSN2)
266307_at	At2g27000	0,89	0,92	0,82	0,98	0,96	0,95	0,94	0,86	cytochrome P450 family protein
266308_at	At2g27010	1,00	1,13	1,04	0,93	0,98	1,03	1,04	1,02	cytochrome P450 family protein
266312_at	At2g27020	0,95	0,90	0,87	1,12	1,06	0,98	0,97	0,92	20S proteasome alpha subunit G (PAG1) (PRC8)
266317_at	At2g27030	1,09	0,91	0,88	1,37	1,19	0,93	0,97	0,87	calmodulin-2/3/5 (CAM5) (TCH1)
266314_at	At2g27040	0,93	1,04	1,13	0,86	1,07	0,88	0,96	0,94	PAZ domain-containing protein / piwi domain-containing protein
266302_at	At2g27050	1,24	1,56	1,67	1,04	1,01	1,16	1,10	1,30	ethylene-insensitive3-like1 (EIL1)
266303_at	At2g27060	1,12	1,07	1,18	0,70	0,83	1,18	1,06	0,98	leucine-rich repeat transmembrane protein kinase, putative
266316_at	At2g27080	0,99	0,88	0,86	1,04	0,80	0,97	1,19	1,15	harpin-induced protein-related / HIN1-related / harpin-responsive protein-re
266304_at	At2g27090	0,87	0,93	1,08	0,81	0,82	1,09	1,16	1,05	expressed protein
266315_at	At2g27100	1,08	1,06	1,12	0,90	0,99	1,13	1,00	1,00	C2H2 zinc-finger protein SERRATE (SE)
266318_at	At2g27110	0,97	0,97	1,24	0,77	0,82	0,96	0,86	1,13	far-red impaired responsive protein, putative
266305_at	At2g27120	0,99	1,04	1,06	1,00	1,01	1,05	0,97	0,89	DNA-directed DNA polymerase epsilon catalytic subunit, putative
266311_at	At2g27130	0,98	0,87	0,94	1,14	1,15	0,91	0,86	0,80	protease inhibitor/seed storage/lipid transfer protein (LTP) family protein
266309_at	At2g27140	1,06	1,12	0,80	1,38	1,49	0,87	1,02	0,89	heat shock family protein
263570_at	At2g27150	0,77	0,77	0,95	0,81	0,70	0,98	0,99	1,11	aldehyde oxidase 3 (AAO3)
263568_at	At2g27160	0,97	1,01	0,96	1,04	1,07	1,04	1,00	1,08	hypothetical protein
263569_at	At2g27170	1,15	0,92	1,14	0,79	0,79	1,22	1,27	1,81	structural maintenance of chromosomes (SMC) family protein

263084_at	At2g27180	0,90	1,00	1,05	0,92	0,87	0,95	1,00	0,84	expressed protein
263083_at	At2g27190	1,15	0,96	1,01	0,99	1,10	1,14	1,20	1,19	iron(III)-zinc(II) purple acid phosphatase (PAP12)
263082_at	At2g27200	0,88	1,01	1,00	0,81	1,12	1,09	1,00	1,05	GTP-binding family protein
263081_at	At2g27220	1,00	1,14	1,04	0,97	0,96	1,00	1,02	1,03	homeodomain-containing protein
265629_at	At2g27230	0,93	0,90	0,92	1,14	0,97	1,00	1,00	0,95	transcription factor-related
265625_at	At2g27240	0,99	1,01	1,07	1,03	1,00	0,93	0,90	0,98	expressed protein
265624_at	At2g27250	0,94	1,05	1,10	1,01	1,10	0,96	1,07	1,14	CLAVATA3
265626_at	At2g27260	0,79	0,90	1,04	0,86	1,10	0,98	1,00	1,05	expressed protein
265623_at	At2g27270	0,80	0,75	1,15	0,93	0,98	0,97	1,10	1,20	expressed protein
265622_at	At2g27280	0,85	0,93	1,05	0,87	1,07	1,12	1,02	0,86	hypothetical protein
265627_at	At2g27285	1,40	1,49	2,05	1,13	1,20	1,49	1,29	1,32	expressed protein
265628_at	At2g27290	1,10	1,04	0,95	0,99	0,86	1,13	1,12	1,14	expressed protein
265621_at	At2g27300	0,95	1,07	1,16	0,93	1,01	0,95	1,02	1,21	no apical meristem (NAM) family protein
265620_at	At2g27310	0,77	0,93	0,95	1,02	1,03	1,19	1,17	1,06	F-box family protein
265619_at	At2g27320	1,05	1,00	0,95	1,03	1,07	0,98	0,96	1,01	hypothetical protein
265641_at	At2g27330	0,98	0,83	0,73	0,86	0,97	0,88	0,86	0,79	RNA recognition motif (RRM)-containing protein
265638_at	At2g27340	0,84	0,77	0,81	0,96	1,10	0,80	0,96	1,01	hypothetical protein
265630_at	At2g27350	1,04	1,11	1,09	0,97	0,83	1,29	1,16	1,10	OTU-like cysteine protease family protein
265646_at	At2g27360	1,45	1,03	1,05	1,58	1,42	1,03	0,94	0,83	lipase, putative
265645_at	At2g27370	0,96	1,19	0,99	1,21	1,41	1,24	1,10	0,98	integral membrane family protein
265639_at	At2g27375	1,01	0,99	1,06	0,92	1,08	1,04	0,97	0,95	hypothetical protein
265644_at	At2g27380	0,92	0,99	0,80	0,63	0,63	0,94	0,97	0,99	proline-rich family protein
265643_at	At2g27390	1,00	0,92	1,04	0,83	1,03	1,16	0,95	0,94	proline-rich family protein
265640_at	At2g27395	1,17	1,05	0,95	1,00	0,98	1,09	0,97	1,27	cysteine protease-related
265647_at	At2g27410	0,89	1,03	0,96	0,86	1,15	1,01	1,00	0,97	hypothetical protein
265665_at	At2g27420	2,28	1,28	1,10	1,11	0,96	1,13	1,01	1,02	cysteine proteinase, putative
265666_at	At2g27440	0,93	0,98	1,01	0,84	1,05	1,17	1,04	1,00	---
265642_at	At2g27450	1,14	1,05	0,98	1,25	1,20	0,97	0,96	1,02	carbon-nitrogen hydrolase family protein
265650_at	At2g27460	1,18	1,11	1,41	1,10	0,88	1,10	0,97	1,16	sec23/sec24 transport family protein
265667_at	At2g27470	1,18	1,11	0,99	0,97	1,16	1,08	1,11	1,17	CCAAT-box binding transcription factor subunit HAP3-related
265636_at	At2g27480	1,63	1,72	2,14	0,88	0,95	1,29	1,36	1,37	calcium-binding EF hand family protein
265637_at	At2g27490	0,89	0,79	0,93	1,42	1,32	0,82	0,97	0,89	dephospho-CoA kinase family
265648_at	At2g27500	0,77	0,73	0,79	0,89	0,90	1,04	1,40	1,32	glycosyl hydrolase family 17 protein
265649_at	At2g27510	0,95	0,89	0,89	1,32	1,19	0,93	0,98	0,88	ferredoxin, putative
266252_at	At2g27520	1,03	0,93	1,11	0,94	1,02	0,96	1,03	1,00	F-box family protein
266210_at	At2g27530	1,11	1,00	0,92	0,97	0,98	0,96	0,90	0,88	60S ribosomal protein L10A (RPL10aB)
266251_s_at	At2g27540	0,95	0,97	1,03	1,00	0,90	0,98	0,98	0,98	hypothetical protein
266209_at	At2g27550	2,27	2,55	1,84	2,56	2,39	1,59	1,66	1,67	centroradialis protein, putative (CEN)
266263_at	At2g27570	0,93	1,02	1,01	0,91	1,17	0,95	1,12	0,98	sulfotransferase family protein
266261_at	At2g27580	0,93	0,87	0,81	1,15	1,26	1,15	1,05	1,01	zinc finger (AN1-like) family protein
266262_at	At2g27590	1,13	1,23	1,38	1,06	1,02	0,93	0,95	1,08	expressed protein
266260_at	At2g27600	1,04	0,91	0,95	1,05	1,20	0,99	0,96	1,05	AAA-type ATPase family protein / vacuolar sorting protein-related
266255_at	At2g27610	1,02	1,02	0,98	0,93	0,80	1,09	0,89	0,73	pentatricopeptide (PPR) repeat-containing protein
266249_at	At2g27620	1,01	0,97	1,00	1,01	1,03	1,10	0,99	0,95	ubiquitin carboxyl-terminal hydrolase-related
266248_at	At2g27650	1,00	0,96	1,07	0,92	0,94	0,95	0,89	1,07	ubiquitin carboxyl-terminal hydrolase-related
266247_at	At2g27660	0,63	0,77	0,67	1,32	1,37	1,29	0,97	1,00	DC1 domain-containing protein

266207_at	At2g27680	0,89	0,83	0,87	0,74	0,75	1,13	1,10	1,13	aldo/keto reductase family protein
266246_at	At2g27690	0,66	0,92	0,86	0,92	0,93	0,96	1,23	1,19	cytochrome P450, putative
266245_at	At2g27700	1,06	0,98	0,99	1,05	0,99	1,01	1,04	1,01	eukaryotic translation initiation factor 2 family protein / eIF-2 family protein
266256_at	At2g27710	1,38	1,07	0,99	1,27	1,17	1,03	0,87	0,95	60S acidic ribosomal protein P2 (RPP2B)
266258_at	At2g27720	1,45	1,28	1,16	1,34	1,06	1,06	1,01	0,99	60S acidic ribosomal protein P2 (RPP2A)
266206_at	At2g27730	1,13	0,98	0,88	1,44	1,43	0,90	0,97	0,99	expressed protein
266244_at	At2g27740	0,98	1,17	1,01	0,97	0,96	0,93	1,09	1,13	expressed protein
266243_at	At2g27750	1,01	0,98	1,05	1,01	0,98	1,00	0,93	0,86	nucleolar matrix protein-related
266208_at	At2g27760	1,09	1,14	1,18	1,07	1,13	1,22	1,10	0,96	tRNA isopentenyltransferase 2 / IPP transferase 2 (IPT2)
266264_at	At2g27775	1,47	1,26	0,90	0,86	0,76	0,76	0,84	0,76	expressed protein
266242_at	At2g27790	1,17	1,00	0,98	0,93	0,92	1,01	1,07	1,09	expressed protein
266241_s_at	At2g27800	0,86	0,95	1,09	0,78	0,95	0,99	1,03	1,08	pentatricopeptide (PPR) repeat-containing protein
266254_at	At2g27810	0,89	0,74	0,70	0,82	0,86	0,98	0,82	0,78	xanthine/uracil permease family protein
266257_at	At2g27820	0,85	0,94	0,92	1,23	1,12	0,87	0,75	0,81	prephenate dehydratase family protein
266259_at	At2g27830	1,02	1,30	1,30	0,96	0,91	1,01	1,20	1,36	expressed protein
266253_at	At2g27840	1,07	0,81	0,75	1,17	0,85	0,92	1,00	0,88	histone deacetylase-related / HD-related
266205_s_at	At2g27860	0,96	0,91	0,88	1,11	1,07	0,90	0,96	0,88	expressed protein
266250_at	At2g27870	0,95	0,83	0,91	0,98	1,01	0,87	0,97	1,14	hypothetical protein
264066_at	At2g27880	1,11	1,12	1,05	0,90	0,91	1,02	0,94	0,78	argonaute protein, putative / AGO, putative
264065_at	At2g27890	0,90	0,87	1,02	1,13	0,96	1,03	0,83	0,90	expressed protein
264064_at	At2g27900	0,82	0,94	0,98	1,14	0,92	0,97	0,84	0,96	expressed protein
264063_at	At2g27910	0,79	0,81	0,87	0,84	0,94	1,07	0,96	1,16	expressed protein
264071_at	At2g27920	1,09	0,94	0,78	1,13	0,98	1,17	0,96	0,73	serine carboxypeptidase S10 family protein
257429_at	At2g27940	1,08	0,98	0,92	0,97	1,01	1,03	1,00	0,84	zinc finger (C3HC4-type RING finger) family protein
264062_at	At2g27950	1,04	0,93	1,20	0,80	0,78	0,90	1,05	1,00	expressed protein
264070_at	At2g27960	1,04	1,00	0,89	1,55	1,30	1,00	0,86	0,83	cyclin-dependent kinase / CDK (CKS1)
264061_at	At2g27970	1,57	1,49	1,24	2,27	2,19	0,86	0,78	0,76	cyclin-dependent kinase, putative / CDK, putative
264060_at	At2g27980	1,00	1,16	1,04	0,72	0,73	1,13	1,12	1,10	expressed protein
264068_at	At2g27990	1,03	1,21	1,05	0,97	1,26	0,96	1,03	1,10	homeodomain-containing protein
264069_at	At2g28000	1,36	1,18	1,11	0,75	0,78	1,07	1,05	0,96	RuBisCO subunit binding-protein alpha subunit, chloroplast / 60 kDa chape
264067_x_at	At2g28010	1,00	1,03	1,01	0,92	1,01	0,83	1,05	0,98	aspartyl protease family protein
266159_at	At2g28030	0,93	0,82	0,92	0,99	0,89	1,03	0,98	1,15	aspartyl protease family protein
266164_at	At2g28050	0,94	0,99	1,12	0,87	0,95	1,08	0,99	0,84	pentatricopeptide (PPR) repeat-containing protein
266138_at	At2g28060	1,41	1,01	1,11	0,98	0,99	1,16	1,08	0,97	protein kinase-related
266158_at	At2g28070	1,08	1,24	1,72	0,81	0,77	0,97	1,31	1,43	ABC transporter family protein
266166_at	At2g28080	1,18	0,99	0,86	1,60	1,55	0,87	0,98	0,91	glycosyltransferase family protein
266139_at	At2g28085	0,68	0,92	1,03	0,94	1,13	0,62	0,94	1,07	auxin-responsive family protein
257380_at	At2g28090	0,94	0,95	1,04	0,97	0,89	0,92	0,97	0,84	heavy-metal-associated domain-containing protein
266157_at	At2g28100	0,87	0,74	0,68	1,23	1,15	0,88	0,74	0,68	glycosyl hydrolase family 29 / alpha-L-fucosidase, putative
266156_at	At2g28110	1,91	1,93	1,72	1,25	0,92	1,37	2,08	1,51	exostosin family protein
266140_at	At2g28120	0,64	0,66	0,71	1,07	1,04	0,65	0,68	0,84	nodulin family protein
266163_at	At2g28130	0,88	0,76	0,90	0,90	0,85	0,78	0,76	0,85	expressed protein
266162_at	At2g28160	1,09	1,01	0,97	1,09	1,06	1,01	0,91	1,06	basic helix-loop-helix (bHLH) family protein
266161_at	At2g28170	1,02	1,06	1,01	1,05	1,15	1,04	0,88	0,89	cation/hydrogen exchanger, putative (CHX7)
266160_at	At2g28180	0,96	1,06	1,02	0,98	0,95	0,98	1,01	0,96	cation/hydrogen exchanger, putative (CHX8)
266165_at	At2g28190	0,70	0,62	0,54	0,62	0,58	1,01	0,99	1,11	superoxide dismutase (Cu-Zn), chloroplast (SODCP) / copper/zinc superoxi

265573_at	At2g28200	1,39	0,92	1,07	1,22	0,97	0,94	0,89	1,08	zinc finger (C2H2 type) family protein
265572_at	At2g28210	0,98	1,07	0,91	0,97	0,96	1,05	1,14	0,94	carbonic anhydrase family protein
265571_s_at	At2g28230	1,36	1,05	0,93	1,05	0,99	1,06	1,15	1,07	hypothetical protein
265546_at	At2g28240	1,08	0,97	1,20	0,84	0,95	0,98	1,02	1,05	hydroxyproline-rich glycoprotein family protein
265545_at	At2g28250	0,96	1,01	0,94	1,14	1,05	1,13	0,98	1,13	protein kinase family protein
265544_at	At2g28260	0,93	0,81	0,59	1,14	1,19	0,96	1,17	0,97	cyclic nucleotide-regulated ion channel, putative (CNGC15)
265543_at	At2g28270	0,94	0,95	1,11	1,01	1,05	0,92	1,05	0,87	DC1 domain-containing protein
265542_at	At2g28290	0,96	0,92	0,79	0,92	0,88	1,00	1,03	1,04	chromatin remodeling protein, putative (SYD)
265547_at	At2g28305	0,91	0,91	1,22	1,37	1,73	1,07	1,59	1,24	expressed protein
265570_at	At2g28310	1,23	1,17	1,25	0,86	1,09	1,09	1,22	1,27	expressed protein
265273_at	At2g28320	1,29	1,18	0,97	0,89	1,04	1,32	1,37	1,44	pleckstrin homology (PH) domain-containing protein / lipid-binding START d
265278_at	At2g28330	1,04	1,03	1,09	1,09	1,29	1,06	1,08	1,27	expressed protein
265272_at	At2g28350	0,91	0,76	0,69	0,99	0,77	0,92	0,92	1,05	auxin-responsive factor (ARF10)
265280_at	At2g28355	0,98	1,04	1,00	0,89	1,01	1,09	1,00	0,92	expressed protein
265271_at	At2g28360	0,98	0,93	1,09	0,95	1,05	1,35	1,08	1,15	SIT4 phosphatase-associated family protein
265281_at	At2g28370	1,02	0,96	0,91	0,88	0,86	1,06	1,02	0,96	expressed protein
265254_at	At2g28380	0,88	0,94	0,83	1,03	1,16	0,94	0,85	0,81	double-stranded RNA-binding domain (DsRBD)-containing protein
265256_at	At2g28390	1,01	0,89	1,26	0,83	1,06	0,96	0,92	0,92	SAND family protein
265276_at	At2g28400	0,58	0,56	0,71	1,26	1,16	1,01	1,19	0,98	expressed protein
265277_at	At2g28410	0,88	0,87	0,91	0,88	1,07	0,90	0,77	0,82	expressed protein
265255_at	At2g28420	1,00	1,01	1,00	0,87	1,01	0,88	0,93	1,29	lactoylglutathione lyase family protein / glyoxalase I family protein
265226_at	At2g28430	1,19	0,85	0,83	0,92	1,03	0,85	0,90	0,73	expressed protein
265275_at	At2g28440	0,88	1,15	1,02	0,99	1,31	0,85	1,10	0,92	proline-rich family protein
265274_at	At2g28450	1,15	1,19	1,24	0,77	0,91	1,07	0,88	0,93	zinc finger (CCCH-type) family protein
265279_at	At2g28460	1,04	0,87	0,93	1,03	1,15	0,88	0,87	1,04	DC1 domain-containing protein
264078_at	At2g28470	0,61	0,62	0,62	0,92	1,07	0,69	0,79	0,84	beta-galactosidase, putative / lactase, putative
264079_at	At2g28490	0,04	0,04	0,03	0,37	0,29	0,93	1,10	1,14	cupin family protein
264056_at	At2g28510	1,23	1,06	1,00	1,60	1,56	0,91	0,95	1,00	Dof-type zinc finger domain-containing protein
264080_at	At2g28520	1,12	1,22	1,21	0,87	0,89	1,13	0,98	0,88	vacuolar proton ATPase, putative
264081_at	At2g28530	1,10	1,28	1,60	1,02	0,96	1,20	1,17	1,51	RNA recognition motif (RRM)-containing protein
264076_at	At2g28540	1,17	1,23	1,88	0,86	1,00	1,39	1,58	2,38	expressed protein
264057_at	At2g28550	1,07	1,06	1,12	0,93	1,01	1,22	1,24	1,21	AP2 domain-containing transcription factor RAP2.7 (RAP2.7)
264077_at	At2g28560	1,07	1,14	0,96	1,04	0,99	1,12	0,91	0,83	expressed protein
264082_at	At2g28570	1,66	1,41	1,17	2,13	2,42	0,84	0,86	0,67	expressed protein
257430_at	At2g28580	0,97	1,08	0,92	0,95	0,91	1,02	1,09	0,76	hypothetical protein
264055_at	At2g28590	1,01	1,10	0,95	1,25	0,85	1,02	1,13	0,99	protein kinase family protein
263435_at	At2g28600	0,97	0,84	0,86	0,92	0,79	0,91	0,81	0,72	expressed protein
263442_at	At2g28605	1,07	0,95	0,98	0,94	0,84	0,95	0,82	0,82	expressed protein
263434_at	At2g28610	1,02	1,02	1,06	1,10	1,12	0,89	0,91	0,95	homeobox-leucine zipper transcription factor (PRESSED FLOWER)
263441_at	At2g28620	1,23	1,06	1,07	1,34	1,36	0,97	0,92	1,01	kinesin motor protein-related
263443_at	At2g28630	0,75	0,76	0,60	0,83	0,65	0,75	0,64	0,52	beta-ketoacyl-CoA synthase family protein
263440_at	At2g28640	0,98	1,07	1,11	1,08	1,21	0,98	0,98	0,96	exocyst subunit EXO70 family protein
263439_at	At2g28650	0,85	1,02	0,82	1,19	1,15	1,09	0,95	0,87	exocyst subunit EXO70 family protein
263438_at	At2g28660	1,08	1,06	1,43	1,29	1,70	0,95	0,97	1,33	copper-binding family protein
263437_at	At2g28670	1,35	1,24	0,93	1,65	1,28	1,10	1,15	1,15	disease resistance-responsive family protein / fibroin-related
257442_at	At2g28680	0,90	1,05	0,87	1,08	0,98	1,12	1,01	1,22	cupin family protein

263436_at	At2g28690	0,85	0,94	0,90	1,10	1,13	0,96	0,97	0,86	expressed protein
263444_at	At2g28700	1,02	1,04	0,98	1,15	1,01	1,00	0,94	1,00	MADS-box protein-related
263411_at	At2g28710	0,97	1,04	1,11	0,95	0,91	1,04	0,85	1,05	zinc finger (C2H2 type) family protein
263412_at	At2g28720	0,62	0,59	0,67	0,54	0,65	1,06	0,88	1,15	histone H2B, putative
266226_at	At2g28740	1,13	1,04	0,87	1,11	1,52	0,93	0,90	0,84	histone H4
266220_at	At2g28755	0,99	1,09	0,99	1,05	1,07	1,00	0,98	0,90	UDP-D-glucuronate carboxy-lyase-related
266221_at	At2g28760	1,26	1,23	0,98	1,74	1,38	0,80	0,94	0,94	NAD-dependent epimerase/dehydratase family protein
266222_at	At2g28780	0,91	0,97	0,99	1,00	1,08	0,99	1,01	1,00	expressed protein
266223_at	At2g28790	1,59	1,50	1,24	2,58	1,83	1,00	0,76	0,70	osmotin-like protein, putative
266224_at	At2g28800	0,98	0,89	0,94	0,95	0,82	1,10	1,04	1,13	chloroplast membrane protein (ALBINO3)
266216_at	At2g28810	1,24	1,25	1,09	0,81	0,89	1,02	1,08	1,28	Dof-type zinc finger domain-containing protein
266217_at	At2g28820	0,88	0,88	0,82	0,82	0,91	1,17	1,04	1,19	ribosomal protein L16 family protein
266230_at	At2g28830	0,81	0,85	1,09	0,63	0,68	0,72	1,03	1,11	armadillo/beta-catenin repeat family protein / U-box domain-containing prot
266229_at	At2g28840	0,75	0,92	1,03	0,55	0,54	0,86	0,84	0,99	ankyrin repeat family protein
266218_s_at	At2g28850	0,92	1,10	0,89	1,00	1,04	0,97	1,03	0,98	cytochrome P450 family protein
266227_at	At2g28870	1,02	1,05	0,87	1,14	0,93	0,98	0,97	1,02	expressed protein
266219_at	At2g28880	0,94	1,04	0,93	0,98	0,94	0,94	1,20	0,79	para-aminobenzoate (PABA) synthase family protein
257377_at	At2g28890	0,56	0,61	0,57	0,95	0,96	0,90	0,85	0,95	protein phosphatase 2C family protein / PP2C family protein
266225_at	At2g28900	0,49	0,50	0,53	0,72	0,86	0,90	0,92	0,96	mitochondrial import inner membrane translocase subunit Tim17/Tim22/Tim
266228_at	At2g28910	0,98	0,92	1,01	0,85	0,94	0,98	0,95	1,05	CAX-interacting protein 4 (CAXIP4)
266803_at	At2g28930	1,59	2,29	2,39	1,07	1,05	1,33	1,68	1,72	protein kinase (APK1b)
266781_at	At2g28940	0,90	1,00	1,13	0,84	0,70	1,17	1,06	1,12	protein kinase family protein
266790_at	At2g28950	1,33	1,35	1,26	1,15	1,17	0,89	0,73	0,72	expansin, putative (EXP6)
266784_at	At2g28960	1,12	1,09	0,89	1,09	0,77	1,17	0,97	1,06	leucine-rich repeat protein kinase, putative
266785_at	At2g28970	1,05	0,98	1,21	0,99	0,88	1,08	0,97	0,97	leucine-rich repeat protein kinase, putative
266786_at	At2g28980	0,93	1,08	1,06	0,97	1,07	0,89	1,14	0,99	---
266787_at	At2g28990	1,01	1,01	1,00	1,13	0,99	1,06	0,94	0,92	leucine-rich repeat protein kinase, putative
266788_at	At2g29000	1,03	1,13	1,06	1,06	1,06	1,00	0,95	0,97	leucine-rich repeat family protein / protein kinase family protein
266789_at	At2g29020	1,04	1,19	1,10	1,03	1,07	0,89	0,89	0,83	Rab5-interacting family protein
266773_at	At2g29040	0,96	0,95	0,95	1,03	1,08	0,98	0,95	1,09	exostosin family protein
266774_at	At2g29050	1,08	0,92	1,01	0,95	1,31	0,76	0,89	0,98	rhomboid family protein
266775_at	At2g29060	0,68	0,82	0,91	0,86	0,74	1,27	1,03	1,25	scarecrow transcription factor family protein
266776_at	At2g29070	1,16	0,99	1,09	1,07	1,00	1,10	1,08	1,19	ubiquitin fusion degradation UFD1 family protein
266777_at	At2g29080	1,11	1,13	1,06	1,10	1,08	1,01	0,98	0,92	FtsH protease, putative
266778_at	At2g29090	0,88	1,01	1,21	0,87	1,13	1,05	1,13	1,11	cytochrome P450 family protein
266779_at	At2g29100	1,03	0,92	0,93	0,80	1,00	1,01	1,14	1,09	glutamate receptor family protein (GLR2.9)
266780_at	At2g29110	0,85	1,00	0,92	1,08	1,13	1,11	0,98	1,16	glutamate receptor family protein (GLR2.8) (GLUR9)
266782_at	At2g29120	0,94	0,96	1,10	1,53	1,59	1,30	0,96	0,97	glutamate receptor family protein (GLR2.7)
266783_at	At2g29130	0,91	1,10	1,10	1,00	1,00	1,06	1,04	1,14	laccase, putative / diphenol oxidase, putative
266288_s_at	At2g29140	1,06	1,17	1,29	0,92	0,79	1,23	1,23	1,13	pumilio/Puf RNA-binding domain-containing protein
266287_at	At2g29150	0,93	1,05	0,99	1,04	1,04	0,94	1,05	0,87	tropinone reductase, putative / tropine dehydrogenase, putative
266286_at	At2g29170	0,81	0,77	0,64	0,98	0,94	0,93	0,94	0,85	short-chain dehydrogenase/reductase (SDR) family protein / tropinone redu
266285_at	At2g29180	1,21	0,97	1,02	0,87	0,82	1,23	1,07	1,01	expressed protein
266284_at	At2g29190	0,98	0,96	1,06	0,68	0,77	1,02	1,09	1,03	pumilio/Puf RNA-binding domain-containing protein
266283_at	At2g29210	1,55	1,40	1,84	0,87	1,07	1,31	1,37	1,48	splicing factor PWI domain-containing protein
266282_at	At2g29220	0,99	1,18	1,02	1,29	1,09	1,30	0,98	1,18	lectin protein kinase, putative

266281_at	At2g29250	0,94	1,02	1,02	0,97	0,97	1,13	1,00	0,90	lectin protein kinase, putative
266280_at	At2g29260	1,14	1,00	1,07	0,88	1,05	0,99	0,96	1,08	tropinone reductase, putative / tropine dehydrogenase, putative
266279_at	At2g29290	1,42	1,74	2,23	0,90	0,85	1,13	1,38	1,47	tropinone reductase, putative / tropine dehydrogenase, putative
266278_at	At2g29300	1,21	1,77	2,06	0,72	0,67	1,37	1,90	1,89	tropinone reductase, putative / tropine dehydrogenase, putative
266277_at	At2g29310	1,14	1,60	2,11	1,23	1,29	1,04	0,96	1,01	tropinone reductase, putative / tropine dehydrogenase, putative
266291_at	At2g29320	0,61	0,99	1,19	1,04	1,02	1,35	1,09	0,98	tropinone reductase, putative / tropine dehydrogenase, putative
266276_at	At2g29330	1,20	1,29	1,06	2,69	1,98	1,22	1,11	1,17	tropinone reductase, putative / tropine dehydrogenase, putative
266265_at	At2g29340	0,82	0,84	0,82	0,95	0,82	0,93	0,91	1,06	short-chain dehydrogenase/reductase (SDR) family protein
266292_at	At2g29350	1,25	1,12	1,19	0,84	0,95	1,82	1,23	1,75	tropinone reductase, putative / tropine dehydrogenase, putative
266293_at	At2g29360	1,30	1,20	1,27	0,89	0,72	1,23	1,20	1,28	tropinone reductase, putative / tropine dehydrogenase, putative
266275_at	At2g29370	0,85	0,95	1,26	1,26	1,47	1,20	1,24	0,99	tropinone reductase, putative / tropine dehydrogenase, putative
266274_at	At2g29380	0,97	0,93	0,95	0,95	0,83	0,83	1,18	0,82	protein phosphatase 2C, putative / PP2C, putative
266289_at	At2g29390	0,81	0,78	0,73	1,26	1,08	0,94	0,90	0,87	sterol 4-alpha-methyl-oxidase 1 (SMO1)
266238_at	At2g29400	1,03	1,25	1,45	0,70	0,78	0,95	1,05	1,25	serine/threonine protein phosphatase PP1 isozyme 1 (TOPP1) / phosphopr
266273_at	At2g29410	1,00	1,01	0,95	1,11	1,17	0,98	1,02	1,05	zinc transporter, putative
266296_at	At2g29420	0,56	0,63	0,88	0,79	0,72	0,73	0,90	0,81	glutathione S-transferase, putative
266271_at	At2g29440	1,13	1,26	1,03	1,72	1,43	1,35	1,65	1,62	glutathione S-transferase, putative
266299_at	At2g29450	1,18	1,24	1,55	1,33	1,55	1,25	1,10	0,98	glutathione S-transferase (103-1A)
266267_at	At2g29460	0,71	0,53	0,67	0,92	1,06	1,37	1,11	1,01	glutathione S-transferase, putative
266270_at	At2g29470	0,98	1,00	0,84	0,95	0,86	1,29	1,29	1,23	glutathione S-transferase, putative
266269_at	At2g29480	0,98	0,88	0,97	0,80	0,72	1,14	1,28	1,34	glutathione S-transferase, putative
266290_at	At2g29490	0,69	0,73	0,90	0,64	0,83	0,85	1,11	1,23	glutathione S-transferase, putative
266294_at	At2g29500	2,44	1,75	1,02	1,49	1,47	1,36	1,08	1,00	17.6 kDa class I small heat shock protein (HSP17.6B-CI)
266268_at	At2g29510	0,82	0,94	1,29	1,04	1,16	0,83	0,92	1,17	expressed protein
266239_at	At2g29530	1,70	1,50	1,21	1,43	1,33	1,07	1,11	1,05	mitochondrial import inner membrane translocase (TIM10)
266237_at	At2g29540	1,64	1,29	1,48	0,87	1,03	1,08	0,98	0,98	DNA-directed RNA polymerase I(A) and III(C) 14 kDa subunit (RPAC14)
266295_at	At2g29550	1,24	0,95	0,87	1,43	1,27	0,87	0,81	0,88	tubulin beta-7 chain (TUB7)
266266_at	At2g29560	0,91	0,88	0,88	1,21	0,99	0,95	0,90	0,89	enolase, putative
266297_at	At2g29570	1,71	1,81	1,60	1,86	1,54	0,98	0,90	0,94	proliferating cell nuclear antigen 2 (PCNA2)
266240_at	At2g29580	0,96	0,84	1,13	0,76	1,09	0,95	0,78	0,90	zinc finger (CCCH-type) family protein / RNA recognition motif (RRM)-conta
266298_at	At2g29590	1,37	1,16	0,84	1,07	0,78	1,17	0,94	1,03	thioesterase family protein
266272_at	At2g29600	1,04	1,12	1,06	1,11	0,82	0,91	1,18	1,04	kelch repeat-containing F-box family protein
266676_s_at	At2g29605	0,98	1,02	0,97	1,03	1,07	1,15	0,88	0,85	hypothetical protein
266675_s_at	At2g29610	1,01	1,03	0,93	1,03	0,99	1,06	1,02	1,00	F-box family protein
266674_at	At2g29620	0,91	1,00	1,16	1,08	1,06	1,01	0,83	0,90	expressed protein
266673_at	At2g29630	0,81	0,86	0,95	0,58	0,66	1,23	1,26	1,22	thiamine biosynthesis family protein / thiC family protein
266672_at	At2g29650	0,92	0,95	1,04	0,89	0,83	1,16	1,16	1,30	inorganic phosphate transporter, putative
266644_at	At2g29660	0,80	0,99	0,94	0,72	0,96	0,94	0,98	1,00	zinc finger (C2H2 type) family protein
266617_at	At2g29670	0,93	0,95	1,04	0,79	0,77	1,33	1,53	1,55	expressed protein
266616_at	At2g29680	1,07	1,24	1,06	1,04	0,96	1,18	1,12	1,07	cell division control protein CDC6, putative
266671_at	At2g29690	1,06	0,99	0,94	0,74	0,81	1,02	1,02	0,99	anthranilate synthase, alpha subunit, component I-2 (ASA2)
266646_at	At2g29700	0,82	0,82	1,01	1,29	1,30	0,88	0,77	0,82	pleckstrin homology (PH) domain-containing protein (PH1)
266615_s_at	At2g29720	1,05	0,93	1,01	1,01	0,89	1,23	1,15	1,14	monooxygenase family protein
266643_s_at	At2g29730	1,21	0,87	1,11	2,23	2,16	0,70	0,69	0,65	UDP-glucoronosyl/UDP-glucosyl transferase family protein
266670_at	At2g29740	0,85	1,00	0,77	1,09	0,95	0,94	0,91	0,79	UDP-glucoronosyl/UDP-glucosyl transferase family protein
266669_at	At2g29750	0,84	0,56	0,42	1,78	1,62	1,00	0,77	0,79	UDP-glucoronosyl/UDP-glucosyl transferase family protein

266668_at	At2g29760	1,06	0,82	0,66	0,76	0,58	0,83	0,91	0,69	pentatricopeptide (PPR) repeat-containing protein
266667_at	At2g29770	1,07	1,03	0,97	1,01	0,92	0,99	1,17	0,93	kelch repeat-containing F-box family protein
266666_at	At2g29780	0,94	0,98	1,03	1,05	0,92	1,02	0,91	0,88	kelch repeat-containing F-box family protein
266665_at	At2g29790	0,97	1,03	0,91	0,89	1,18	0,96	0,92	1,07	expressed protein
266664_at	At2g29800	0,85	1,03	1,11	0,89	0,88	1,01	1,13	1,10	kelch repeat-containing F-box family protein
266677_at	At2g29820	0,93	1,04	0,85	1,00	0,92	0,92	1,15	1,19	kelch repeat-containing F-box family protein
257368_at	At2g29860	0,94	0,93	1,21	1,05	1,16	0,99	1,08	0,90	kelch repeat-containing F-box family protein
266645_at	At2g29880	1,07	1,05	1,01	0,99	0,96	1,02	1,02	1,27	hypothetical protein
263485_at	At2g29890	1,04	0,94	1,13	1,08	1,04	0,91	1,12	0,97	villin 1 (VLN1)
263484_at	At2g29900	0,80	0,95	0,78	0,89	1,02	1,05	0,77	0,83	presenilin family protein
266807_at	At2g29920	0,86	1,07	0,94	0,91	1,20	1,06	0,92	0,86	expressed protein
266866_at	At2g29940	0,69	0,76	0,68	0,77	0,61	0,90	0,79	0,98	ABC transporter family protein
266804_at	At2g29960	1,47	1,18	1,11	1,64	1,50	0,96	1,03	0,94	peptidyl-prolyl cis-trans isomerase / cyclophilin (CYP5) / rotamase
266809_at	At2g29970	0,78	0,95	0,88	0,77	0,81	0,92	1,00	0,85	heat shock protein-related
266865_at	At2g29980	1,13	1,10	1,12	1,02	1,00	0,85	0,79	0,83	omega-3 fatty acid desaturase, endoplasmic reticulum (FAD3)
266835_at	At2g29990	0,56	0,64	0,57	0,83	0,85	0,89	0,84	0,81	pyridine nucleotide-disulphide oxidoreductase family protein
266808_at	At2g29995	1,63	1,47	1,27	1,30	1,26	1,01	1,06	1,00	expressed protein
266806_at	At2g30000	1,45	1,26	1,35	0,83	0,97	1,19	1,16	1,12	expressed protein
266805_at	At2g30010	0,89	0,70	0,72	0,85	0,80	0,85	0,77	0,80	expressed protein
266834_s_at	At2g30020	0,89	1,33	1,23	0,71	0,51	1,45	2,19	1,47	pseudogene, protein phosphatase 2C
266833_at	At2g30030	0,86	0,91	1,01	1,07	0,96	1,07	1,04	0,87	---
266832_at	At2g30040	0,53	0,66	0,80	0,62	0,66	0,96	1,50	1,18	protein kinase family protein
267277_at	At2g30050	1,23	1,15	1,04	1,32	1,45	0,81	0,92	0,92	transducin family protein / WD-40 repeat family protein
267306_at	At2g30060	1,35	1,19	1,14	1,48	1,41	0,88	0,91	0,86	Ran-binding protein 1b (RanBP1b)
267305_at	At2g30070	0,98	1,43	1,31	0,68	0,70	1,09	1,54	1,43	potassium transporter (KUP1)
267304_at	At2g30080	1,07	1,26	1,18	0,88	0,96	0,96	0,93	1,10	metal transporter, putative (ZIP6)
267303_at	At2g30090	1,05	1,09	1,03	0,96	1,03	1,08	1,07	0,97	GCN5-related N-acetyltransferase (GNAT) family protein
267302_at	At2g30100	1,20	1,02	1,18	1,00	1,22	1,04	1,07	0,97	ubiquitin family protein
267301_at	At2g30110	1,09	0,99	1,04	0,98	0,99	0,92	1,00	0,93	ubiquitin activating enzyme 1 (UBA1)
267248_at	At2g30120	1,00	1,04	1,14	1,00	1,11	1,10	0,88	1,23	expressed protein
267276_at	At2g30130	1,08	1,24	0,95	1,62	1,32	1,22	1,10	1,11	LOB domain protein 12 / lateral organ boundaries domain protein 12 (LBD1
267300_at	At2g30140	0,74	0,66	0,88	0,81	0,70	1,03	0,88	1,07	UDP-glucoronosyl/UDP-glucosyl transferase family protein
267299_at	At2g30150	0,92	1,10	1,01	1,03	0,63	1,08	1,03	0,82	UDP-glucoronosyl/UDP-glucosyl transferase family protein
267274_at	At2g30160	1,07	1,05	0,98	0,88	0,81	1,01	0,93	1,00	mitochondrial substrate carrier family protein
267247_at	At2g30170	1,04	1,06	0,95	0,64	0,73	1,03	1,10	0,98	expressed protein
267308_at	At2g30200	0,84	0,71	0,63	0,83	0,82	0,92	0,80	0,79	expressed protein
267307_at	At2g30210	1,24	1,27	0,97	1,51	1,14	1,12	0,98	0,89	laccase, putative / diphenol oxidase, putative
267275_at	At2g30240	0,89	1,07	1,27	0,95	1,11	0,99	0,94	1,33	cation/hydrogen exchanger, putative (CHX13)
267246_at	At2g30250	0,92	0,98	1,09	0,71	0,76	1,18	1,46	1,61	WRKY family transcription factor
255871_at	At2g30260	1,21	0,97	0,93	1,27	1,33	0,92	0,87	0,86	small nuclear ribonucleoprotein U2B, putative / spliceosomal protein, putati
255869_at	At2g30270	0,79	0,80	0,79	1,02	0,96	0,88	0,79	0,84	expressed protein
255870_at	At2g30280	1,19	1,02	1,05	1,17	0,94	1,10	1,15	0,93	expressed protein
255861_at	At2g30290	0,96	0,98	0,92	1,00	1,07	0,97	1,02	0,91	vacuolar sorting receptor, putative
255862_at	At2g30300	1,07	1,00	0,97	1,05	1,07	1,01	0,92	1,08	nodulin-related
255863_s_at	At2g30310	0,95	0,95	1,03	0,96	1,07	0,97	1,03	1,01	GDSL-motif lipase/hydrolase family protein
255864_at	At2g30320	0,88	0,65	0,67	0,72	0,93	0,97	0,94	1,01	tRNA pseudouridine synthase family protein

255865_at	At2g30330	0,68	0,45	0,60	0,92	1,18	0,92	0,77	0,71	GCN5L1 family protein
255873_at	At2g30340	0,96	1,05	1,09	0,99	1,34	1,03	1,09	1,07	LOB domain protein 13 / lateral organ boundaries domain protein 13 (LBD1
255866_at	At2g30350	1,02	0,87	1,34	0,89	0,99	1,10	0,83	1,01	endo/excinuclease amino terminal domain-containing protein
255872_at	At2g30360	0,90	0,98	0,81	1,40	1,01	0,88	1,06	1,20	CBL-interacting protein kinase 11 (CIPK11)
255867_at	At2g30370	1,11	1,19	1,02	0,95	1,17	1,15	1,05	1,02	allergen-related
255868_at	At2g30380	1,01	1,03	1,11	0,91	1,14	0,89	0,95	1,09	expressed protein
267471_at	At2g30390	0,93	0,99	1,14	0,71	0,73	1,12	1,09	1,13	ferrochelatase II
267493_at	At2g30400	0,98	0,93	1,09	1,19	0,64	0,97	1,01	1,05	ovate family protein
267494_at	At2g30410	1,19	1,02	0,92	1,19	1,19	0,95	0,93	0,96	tubulin folding cofactor A (KIESEL)
267495_at	At2g30420	0,99	1,01	0,96	0,90	0,83	1,07	1,08	0,94	myb family transcription factor
267522_at	At2g30430	0,99	1,04	1,03	0,99	1,07	1,09	0,94	0,94	hypothetical protein
267520_at	At2g30460	0,83	1,01	0,81	1,04	0,84	0,87	0,86	0,76	expressed protein
267519_at	At2g30470	0,73	0,69	0,81	0,84	1,12	1,06	0,93	1,09	transcriptional factor B3 family protein
267521_at	At2g30480	0,78	1,02	0,98	0,95	0,82	1,00	1,00	1,33	expressed protein
267470_at	At2g30490	1,19	1,19	1,35	1,20	1,14	1,18	1,18	1,13	trans-cinnamate 4-monooxygenase / cinnamic acid 4-hydroxylase (C4H) (C
267518_at	At2g30500	0,60	0,89	0,80	0,89	1,08	1,19	1,48	1,27	kinase interacting family protein
267517_at	At2g30510	1,55	2,06	2,52	0,86	0,82	1,75	1,73	1,83	signal transducer of phototropic response (RPT2)
267516_at	At2g30520	1,54	2,43	2,61	0,96	0,77	1,55	1,73	2,15	signal transducer of phototropic response (RPT2)
267468_at	At2g30530	0,95	0,85	0,89	0,92	1,11	1,02	1,03	1,11	expressed protein
267497_at	At2g30540	1,11	1,02	1,05	0,57	0,57	0,83	0,74	0,84	glutaredoxin family protein
267496_at	At2g30550	0,91	1,07	0,96	0,94	0,98	0,94	1,12	1,22	lipase class 3 family protein
267525_at	At2g30560	0,97	0,99	0,93	0,97	1,07	1,06	0,98	1,05	glycine-rich protein
267526_at	At2g30570	0,93	0,94	0,94	1,00	0,97	1,00	0,94	0,90	photosystem II reaction center W (PsbW) protein-related
267467_at	At2g30580	1,01	0,67	0,89	0,76	0,86	1,12	0,88	1,13	zinc finger (C3HC4-type RING finger) family protein
267469_at	At2g30590	0,99	1,04	1,00	1,03	1,17	0,98	0,77	0,72	WRKY family transcription factor
267524_at	At2g30600	1,16	1,44	1,70	0,75	0,62	1,08	1,35	1,27	BTB/POZ domain-containing protein
267523_at	At2g30610	1,21	1,41	1,56	0,70	0,57	1,06	1,30	1,26	BTB/POZ domain-containing protein
267492_at	At2g30620	0,99	0,98	1,29	0,78	1,06	0,89	0,87	1,20	histone H1.2
257349_at	At2g30630	0,92	0,86	0,97	0,94	1,17	0,99	0,93	0,86	expressed protein
267576_at	At2g30640	0,85	0,90	1,06	0,90	0,83	1,16	1,04	0,99	---
267571_at	At2g30650	1,04	1,00	1,08	0,96	0,97	0,91	0,95	0,84	3-hydroxyisobutyryl-coenzyme A hydrolase, putative / CoA-thioester hydrola
267572_at	At2g30660	0,97	0,96	0,96	0,89	1,07	0,94	1,10	1,11	3-hydroxyisobutyryl-coenzyme A hydrolase, putative / CoA-thioester hydrola
267573_at	At2g30670	0,62	0,57	0,29	0,96	1,06	0,88	1,01	0,82	tropinone reductase, putative / tropine dehydrogenase, putative
267574_at	At2g30680	0,98	1,17	1,10	1,02	0,95	1,17	0,97	1,03	expressed protein
267575_at	At2g30690	0,94	1,13	1,04	0,99	1,16	0,94	0,98	0,86	expressed protein
267578_at	At2g30695	0,58	0,52	0,45	1,05	0,82	0,81	0,91	0,85	expressed protein
267577_at	At2g30710	1,39	1,02	1,19	1,18	1,27	1,01	0,94	0,96	RabGAP/TBC domain-containing protein
267563_at	At2g30730	0,94	1,02	0,98	0,95	1,04	1,03	0,96	0,98	serine/threonine protein kinase, putative
267564_at	At2g30740	0,72	0,67	0,82	1,00	0,89	0,82	0,81	0,80	serine/threonine protein kinase, putative
267565_at	At2g30750	1,48	1,97	1,80	0,70	0,53	2,67	2,21	2,18	cytochrome P450 71A12, putative (CYP71A12)
267566_at	At2g30760	1,16	0,93	1,10	0,99	1,03	1,28	1,10	1,13	hypothetical protein
267567_at	At2g30770	1,42	1,10	1,15	0,91	0,82	1,44	1,71	2,30	cytochrome P450 71A13, putative (CYP71A13)
267568_at	At2g30780	0,88	1,02	0,95	0,93	1,02	1,03	0,92	0,92	pentatricopeptide (PPR) repeat-containing protein
267569_at	At2g30790	0,55	0,50	0,62	0,68	0,82	0,91	0,88	0,84	photosystem II oxygen-evolving complex 23, putative
267570_at	At2g30800	0,93	0,98	1,14	0,94	1,04	1,26	1,26	1,06	DEIH-box RNA/DNA helicase, putative
267198_at	At2g30810	0,97	0,96	0,98	0,94	1,21	0,94	1,07	0,97	gibberellin-regulated family protein

267205_at	At2g30820	0,95	1,03	1,02	0,98	1,08	1,03	1,08	0,86	expressed protein
267206_at	At2g30830	0,93	0,92	0,83	1,17	1,39	0,98	0,94	1,18	2-oxoglutarate-dependent dioxygenase, putative
267207_at	At2g30840	1,15	1,20	0,94	1,18	0,86	1,10	1,09	1,18	2-oxoglutarate-dependent dioxygenase, putative
267153_at	At2g30860	1,04	0,98	0,96	1,03	0,96	0,82	0,90	0,86	glutathione S-transferase, putative
267154_at	At2g30870	1,01	0,96	0,94	1,03	1,04	1,02	1,06	1,00	glutathione S-transferase, putative
267192_at	At2g30890	1,54	1,45	1,50	1,22	1,53	1,00	0,88	0,98	membrane protein, putative
267193_at	At2g30900	0,97	1,11	1,10	1,04	1,03	1,05	0,87	0,98	expressed protein
267194_s_at	At2g30910	0,91	0,95	0,82	1,11	1,26	0,99	0,82	0,77	transducin family protein / WD-40 repeat family protein
267210_at	At2g30920	1,33	1,22	1,17	1,22	1,11	1,00	1,01	0,97	hexaprenyldihydroxybenzoate methyltransferase
267155_at	At2g30925	0,93	0,94	0,98	1,00	1,10	1,02	1,00	0,92	hypothetical protein
267209_at	At2g30930	0,99	0,94	1,25	1,39	1,62	0,90	0,85	0,98	expressed protein
267195_at	At2g30940	0,94	0,93	1,13	1,10	1,14	1,10	1,05	0,97	protein kinase family protein
267196_at	At2g30950	0,83	0,83	0,83	0,81	0,79	1,08	1,07	1,05	FtsH protease (VAR2)
267197_at	At2g30960	0,88	0,96	1,07	1,00	1,22	1,08	1,12	0,98	expressed protein
267151_at	At2g30970	1,15	1,14	0,89	1,31	0,88	1,05	1,06	0,93	aspartate aminotransferase, mitochondrial / transaminase A (ASP1)
267208_at	At2g30980	0,95	0,95	1,21	0,94	0,96	0,94	0,91	1,08	shaggy-related protein kinase delta / ASK-delta / ASK-dzeta (ASK4)
267199_at	At2g30990	0,87	0,82	0,97	0,84	0,97	1,11	1,24	1,65	expressed protein
267200_at	At2g31000	0,77	0,76	0,89	1,30	1,45	0,81	0,86	0,95	protein kinase family protein
267201_at	At2g31010	0,78	0,71	0,76	1,17	1,17	1,06	0,91	0,95	protein kinase family protein
267202_s_at	At2g31030	1,00	0,92	0,74	0,98	1,01	1,02	1,10	0,94	oxysterol-binding family protein
267203_at	At2g31035	1,01	0,91	1,05	0,98	1,07	0,97	0,95	1,02	hypothetical protein
267152_at	At2g31040	0,70	0,72	0,86	0,78	0,89	1,07	1,16	1,31	ATP synthase protein I -related
267204_at	At2g31050	0,88	1,14	1,04	0,92	1,05	0,98	0,90	1,00	plastocyanin-like domain-containing protein
266471_at	At2g31060	1,27	1,11	1,03	0,82	1,01	1,06	0,90	0,91	elongation factor family protein
266481_at	At2g31070	0,98	0,74	0,90	0,98	0,85	1,19	0,89	0,94	TCP family transcription factor, putative
266470_at	At2g31080	0,87	0,94	0,85	1,08	1,13	0,96	0,91	0,98	---
266477_at	At2g31085	1,01	1,05	1,04	1,21	1,19	1,10	1,10	1,01	Clavata3 / ESR-Related-6 (CLE6)
266476_at	At2g31090	0,96	0,99	1,22	1,21	1,21	0,87	1,01	1,11	expressed protein
266475_at	At2g31100	1,01	0,97	0,98	1,10	1,00	1,17	1,14	1,02	lipase, putative
266474_at	At2g31110	1,55	1,45	1,83	1,51	1,44	0,99	0,86	0,87	expressed protein
266473_at	At2g31120	1,42	1,26	1,78	1,21	1,57	1,08	0,99	0,94	expressed protein
266480_at	At2g31130	1,21	1,10	0,91	0,77	1,02	1,14	1,22	1,26	expressed protein
266472_at	At2g31150	1,21	0,92	1,07	0,91	0,78	1,22	1,32	1,30	expressed protein
266479_at	At2g31160	0,98	1,13	1,25	1,11	1,23	0,96	1,06	0,76	expressed protein
266478_at	At2g31170	0,81	0,72	0,71	0,67	0,88	1,05	0,98	0,90	tRNA synthetase class I (C) family protein
266469_at	At2g31180	0,98	0,98	0,98	1,12	1,73	1,05	1,14	1,14	myb family transcription factor (MYB14)
264086_at	At2g31190	1,15	1,26	1,60	0,82	0,85	1,09	1,36	0,93	expressed protein
264089_at	At2g31200	0,91	0,83	0,82	1,30	1,20	0,90	0,96	0,88	actin-depolymerizing factor 6 (ADF6)
264117_at	At2g31210	0,96	0,92	1,09	0,94	1,03	0,95	1,08	1,14	basic helix-loop-helix (bHLH) family protein
264083_at	At2g31230	1,04	1,06	1,16	0,86	1,10	0,90	0,90	0,93	ethylene-responsive factor, putative
264084_at	At2g31240	1,26	1,14	1,09	0,98	1,20	1,04	0,93	1,02	tetratricopeptide repeat (TPR)-containing protein
264085_at	At2g31250	0,91	0,95	1,01	0,95	1,08	1,13	0,97	0,91	glutamyl-tRNA reductase, putative
264113_at	At2g31260	1,24	1,13	1,26	0,95	1,05	0,99	1,07	1,19	autophagy 9 (APG9)
264114_at	At2g31270	1,18	0,92	1,08	1,24	1,70	1,06	0,91	1,02	hydroxyproline-rich glycoprotein family protein
264115_at	At2g31290	1,12	1,04	1,09	0,83	1,05	1,04	0,96	0,83	expressed protein
264059_at	At2g31305	1,29	0,98	1,04	1,22	1,19	1,03	0,96	1,02	expressed protein

264116_at	At2g31320	1,33	1,32	1,67	0,93	0,99	1,07	1,12	1,09	poly (ADP-ribose) polymerase, putative / NAD(+) ADP-ribosyltransferase, p
264087_at	At2g31330	1,06	0,91	1,20	1,07	0,98	1,09	1,05	1,07	expressed protein
264088_at	At2g31340	1,14	0,99	1,15	1,16	1,00	0,96	0,89	0,77	expressed protein
263243_at	At2g31350	0,95	0,85	0,85	0,81	1,07	0,96	0,98	1,03	hydroxyacylglutathione hydrolase, putative / glyoxalase II, putative
263249_at	At2g31360	1,00	1,05	1,18	1,09	1,02	0,97	0,85	0,96	delta 9 desaturase (ADS2)
263253_at	At2g31370	0,93	0,93	0,85	1,50	1,29	1,10	1,01	1,03	bZIP transcription factor (POSF21)
263252_at	At2g31380	2,74	2,86	2,29	1,66	1,44	2,44	2,04	1,38	zinc finger (B-box type) family protein / salt tolerance-like protein (STH)
263250_at	At2g31390	1,15	1,08	0,92	1,79	1,50	1,07	0,92	0,96	pfkB-type carbohydrate kinase family protein
263242_at	At2g31400	0,93	0,88	0,95	0,91	0,95	1,10	1,09	1,13	pentatricopeptide (PPR) repeat-containing protein
263251_at	At2g31410	1,12	0,96	1,02	1,09	1,05	1,06	0,86	0,83	expressed protein
257449_at	At2g31420	0,98	1,05	1,03	0,97	1,02	0,89	1,02	1,07	hypothetical protein
263247_at	At2g31440	1,44	1,28	1,13	1,26	1,21	1,10	1,01	1,03	expressed protein
263248_at	At2g31450	1,12	1,08	1,37	0,85	0,82	1,12	1,16	1,53	endonuclease-related
263246_at	At2g31460	0,98	0,97	0,95	1,07	0,98	1,10	1,00	0,96	hypothetical protein
263245_at	At2g31470	0,99	1,05	0,95	1,05	0,95	1,06	1,05	0,93	F-box family protein
263244_at	At2g31480	1,01	0,98	0,92	0,97	1,08	0,97	1,09	1,00	expressed protein
263399_at	At2g31490	1,32	1,06	0,93	1,14	1,24	1,08	1,03	1,13	expressed protein
263450_at	At2g31500	0,99	0,95	1,10	1,05	1,13	0,94	1,08	1,07	calcium-dependent protein kinase, putative / CDPK, putative
263424_at	At2g31510	0,95	1,07	1,40	0,87	0,96	1,02	1,11	1,53	IBR domain-containing protein / ARIADNE-like protein ARI7 (ARI7)
263425_at	At2g31530	0,98	1,00	1,09	0,88	0,83	1,08	0,94	1,22	secY family protein
263397_s_at	At2g31550	1,06	1,08	0,99	1,05	1,15	1,11	0,89	0,90	GDSL-motif lipase/hydrolase family protein
263426_at	At2g31570	1,33	1,25	1,25	1,45	1,37	1,13	1,08	1,08	glutathione peroxidase, putative
263445_at	At2g31590	0,97	0,99	0,93	1,02	0,96	1,02	0,98	0,85	hypothetical protein
263400_s_at	At2g31610	1,36	1,08	1,08	1,13	1,13	1,02	1,01	0,92	40S ribosomal protein S3 (RPS3A)
263446_at	At2g31630	0,95	1,16	1,06	0,80	1,08	1,04	1,02	1,02	trithorax 1 (ATX-1) (TRX1)
263447_s_at	At2g31640	0,94	1,09	1,45	0,87	0,90	0,95	1,05	1,14	trithorax protein, putative / PHD finger family protein / SET domain-containi
263448_at	At2g31660	1,08	1,08	1,05	0,80	0,88	1,10	0,95	0,84	importin beta-2 subunit family protein
263449_at	At2g31670	0,90	0,82	0,84	1,08	1,07	0,90	0,77	0,79	expressed protein
263398_at	At2g31680	1,00	0,76	0,86	1,26	1,27	0,95	0,89	0,82	Ras-related GTP-binding protein, putative
263451_at	At2g31690	0,98	1,00	1,00	1,07	0,95	1,02	0,90	0,96	lipase class 3 family protein
263423_at	At2g31700	0,96	0,88	1,07	0,98	0,94	0,94	0,98	0,85	hypothetical protein
263396_at	At2g31710	1,12	0,83	0,83	1,29	1,08	0,94	0,88	0,86	expressed protein
263474_at	At2g31725	1,47	1,06	1,13	1,13	0,86	1,14	0,94	1,10	expressed protein
263467_at	At2g31730	0,82	0,73	0,83	1,52	1,39	0,81	0,83	0,82	ethylene-responsive protein, putative
263466_at	At2g31740	1,12	1,01	1,06	1,02	0,82	1,14	0,97	1,10	expressed protein
263473_at	At2g31750	1,14	1,10	1,37	1,18	1,04	1,04	0,97	1,05	UDP-glucoronosyl/UDP-glucosyl transferase family protein
263464_at	At2g31760	1,06	0,99	0,92	1,10	1,11	1,05	1,01	0,96	zinc finger protein-related
263463_at	At2g31770	1,00	0,97	0,96	1,06	1,02	1,12	1,04	1,13	zinc finger (C3HC4-type RING finger) family protein
263462_at	At2g31780	0,97	1,02	0,89	0,95	1,04	1,03	0,98	0,94	zinc finger (C3HC4-type RING finger) family protein
263477_at	At2g31790	1,53	1,71	2,06	1,12	0,95	1,10	1,23	1,08	UDP-glucoronosyl/UDP-glucosyl transferase family protein
263461_at	At2g31800	0,89	0,93	1,00	0,80	0,91	1,16	1,00	1,06	ankyrin protein kinase, putative
263460_at	At2g31810	0,70	0,71	0,74	0,84	0,72	0,99	0,99	1,03	acetolactate synthase small subunit, putative
263459_at	At2g31820	0,79	0,86	0,95	0,94	0,80	1,01	1,28	1,29	ankyrin repeat family protein
263489_at	At2g31830	0,99	1,09	0,99	0,83	1,08	1,02	1,00	1,18	endonuclease/exonuclease/phosphatase family protein
263488_at	At2g31840	1,01	0,90	1,02	0,70	0,66	0,91	0,89	0,85	expressed protein
263487_at	At2g31850	1,03	0,91	1,15	0,98	0,93	1,03	0,97	1,03	expressed protein

263514_at	At2g31860	0,98	1,04	0,93	1,00	0,93	0,96	1,06	1,13	---
263476_at	At2g31870	0,81	1,05	0,97	0,79	0,85	0,92	1,06	0,99	poly (ADP-ribose) glycohydrolase (PARG) family protein
263478_at	At2g31880	0,79	0,96	1,20	0,80	0,76	1,10	1,57	1,77	leucine-rich repeat transmembrane protein kinase, putative
263471_at	At2g31890	1,09	1,11	1,16	0,61	0,67	1,11	1,22	1,21	expressed protein
263470_at	At2g31900	0,94	1,09	0,89	1,12	1,07	1,12	0,85	0,84	myosin family protein
263469_at	At2g31910	1,01	0,92	1,02	1,04	0,95	1,01	0,99	1,13	cation/hydrogen exchanger, putative
263468_at	At2g31930	0,98	1,03	1,02	1,00	1,06	1,08	0,93	0,92	expressed protein
263465_at	At2g31940	1,06	1,07	0,98	1,29	1,07	1,01	0,86	0,95	expressed protein
263475_at	At2g31945	0,64	0,95	1,10	0,83	0,68	1,27	2,13	1,45	expressed protein
263472_at	At2g31950	0,75	0,70	0,92	0,90	1,00	0,85	0,87	0,92	molybdopterin biosynthesis protein, putative / molybdenum cofactor biosynt
265696_at	At2g31955	1,06	1,12	0,90	0,95	1,21	0,96	1,05	0,95	---
265729_at	At2g31960	0,84	0,85	0,85	0,85	0,75	1,00	1,11	1,11	glycosyl transferase family 48 protein
265678_at	At2g31970	0,94	0,80	0,85	0,63	0,75	1,19	1,29	1,17	DNA repair-recombination protein (RAD50)
265672_at	At2g31980	0,23	0,36	0,37	0,85	0,67	1,10	1,14	0,99	cysteine proteinase inhibitor-related
265728_at	At2g31990	0,80	0,94	0,92	0,92	1,27	0,98	1,18	0,92	exostosin family protein
265727_at	At2g32000	0,85	0,93	0,92	0,71	0,85	1,15	0,94	1,08	DNA topoisomerase family protein
265726_at	At2g32010	1,42	1,40	1,12	0,78	1,00	1,29	1,16	1,02	endonuclease/exonuclease/phosphatase family protein
265668_at	At2g32020	1,41	1,47	1,45	1,21	1,29	1,55	1,41	1,47	GCN5-related N-acetyltransferase (GNAT) family protein
265725_at	At2g32030	1,46	1,46	1,21	1,39	1,29	1,39	1,34	1,15	GCN5-related N-acetyltransferase (GNAT) family protein
265669_at	At2g32040	1,24	1,37	1,20	0,88	0,96	1,20	1,21	1,26	integral membrane transporter family protein
257391_at	At2g32050	0,97	1,03	0,90	0,93	0,99	0,88	1,04	0,95	cell cycle control protein-related
265671_at	At2g32060	1,31	1,08	1,02	1,27	1,34	0,97	0,96	1,01	40S ribosomal protein S12 (RPS12C)
265676_at	At2g32070	1,10	0,93	0,98	1,08	1,26	0,98	0,97	1,05	CCR4-NOT transcription complex protein, putative
265677_at	At2g32080	0,79	0,83	0,81	0,92	0,91	0,91	0,88	0,74	PUR alpha-1 protein
265673_at	At2g32090	1,09	1,18	1,25	0,98	1,04	0,95	1,04	1,02	lactoylglutathione lyase family protein / glyoxalase I family protein
265724_at	At2g32100	0,81	0,95	0,91	0,60	0,92	0,92	0,93	0,93	ovate protein-related
265675_at	At2g32120	1,50	1,65	1,48	1,03	1,11	1,19	1,23	1,07	heat shock protein 70 family protein / HSP70 family protein
265723_at	At2g32140	1,13	1,08	0,99	1,30	0,97	1,59	2,22	2,88	disease resistance protein (TIR class), putative
265680_at	At2g32150	0,99	2,71	2,71	0,92	0,92	1,16	2,33	2,90	haloacid dehalogenase-like hydrolase family protein
265698_at	At2g32160	1,01	0,98	1,14	1,31	1,77	1,36	1,40	1,43	expressed protein
265697_at	At2g32170	1,10	1,13	1,22	0,89	0,93	1,06	1,22	1,20	expressed protein
265674_at	At2g32190	0,82	0,66	0,62	1,64	1,61	1,35	1,47	1,89	expressed protein
265670_s_at	At2g32210	0,71	0,67	0,81	1,11	1,48	1,33	1,45	1,71	expressed protein
265730_at	At2g32220	0,85	0,77	0,80	0,95	0,90	0,83	0,73	0,82	60S ribosomal protein L27 (RPL27A)
265679_at	At2g32240	1,32	1,23	1,74	2,22	1,84	1,24	1,11	1,38	expressed protein
266360_at	At2g32250	0,91	0,79	1,01	1,63	1,43	0,94	0,98	1,31	far-red impaired responsive protein, putative
266359_at	At2g32260	0,97	0,93	0,88	0,85	0,82	1,08	1,05	1,07	cholinephosphate cytidylyltransferase, putative / phosphorylcholine transfer
266336_at	At2g32270	1,01	1,15	1,07	1,36	1,41	1,08	0,82	0,84	zinc transporter (ZIP3)
266358_at	At2g32280	1,34	1,40	1,26	1,64	1,57	0,88	1,03	1,06	expressed protein
266357_at	At2g32290	1,10	1,19	0,98	0,75	0,70	1,15	1,09	0,98	beta-amylase, putative / 1,4-alpha-D-glucan maltohydrolase, putative
266356_at	At2g32300	1,40	1,30	1,22	1,65	1,06	1,18	1,27	1,03	uclacyanin I
266390_at	At2g32310	0,95	0,98	0,99	0,98	1,05	1,10	1,06	0,86	expressed protein
266389_s_at	At2g32330	1,10	1,13	1,13	0,98	0,90	1,01	1,11	1,06	expressed protein
266388_at	At2g32340	0,92	0,88	0,92	1,02	0,91	1,04	1,06	1,09	TraB family protein
257376_at	At2g32350	0,95	0,94	1,31	0,87	0,99	0,96	0,97	1,10	ubiquitin family protein
266387_at	At2g32360	0,96	0,91	1,09	1,07	0,96	1,03	0,93	0,89	ubiquitin family protein

266386_at	At2g32370	0,87	1,13	0,92	0,88	1,12	0,96	0,84	1,05	homeobox-leucine zipper family protein / lipid-binding START domain-conta
266334_at	At2g32380	1,00	0,94	0,71	1,42	1,05	0,95	0,79	0,69	expressed protein
266337_at	At2g32390	0,72	0,77	0,94	0,68	0,92	0,96	0,94	1,10	glutamate receptor family protein (GLR3.5)
266338_at	At2g32400	1,16	0,98	1,05	0,82	1,00	1,02	1,23	1,34	glutamate receptor family protein (GLR3.7) (GLR5)
266333_at	At2g32410	1,09	1,12	0,98	1,00	0,92	0,97	1,13	0,94	auxin-resistance protein, putative
266332_at	At2g32420	0,77	0,83	0,85	0,98	0,98	0,88	0,90	0,97	3'-5' exonuclease domain-containing protein
266362_at	At2g32430	1,07	1,28	1,32	1,02	1,28	1,04	1,23	1,08	galactosyltransferase family protein
266335_at	At2g32440	1,07	1,12	1,35	0,74	0,80	1,17	1,20	1,13	ent-kaurenoic acid hydroxylase, putative / cytochrome P450, putative
266361_at	At2g32450	0,95	0,77	0,78	0,99	0,76	0,91	0,92	0,90	calcium-binding EF hand family protein
267087_at	At2g32460	0,97	0,91	0,85	1,06	0,86	1,00	0,87	0,84	myb family transcription factor (MYB101)
267056_at	At2g32470	0,88	1,06	1,02	0,87	0,96	1,04	0,95	0,94	F-box family protein-related
267061_at	At2g32480	0,94	0,78	0,78	0,66	0,76	1,04	0,96	0,87	membrane-associated zinc metalloprotease, putative
257356_s_at	At2g32490	0,93	0,97	1,06	1,00	0,94	0,87	1,03	0,83	3'-5' exonuclease domain-containing protein
267057_at	At2g32500	1,17	1,00	0,92	0,70	0,76	1,21	1,13	1,10	expressed protein
267058_at	At2g32510	0,90	1,06	0,85	1,14	1,09	1,09	1,15	1,17	protein kinase family protein
267059_at	At2g32520	1,18	1,23	1,13	1,09	1,07	1,10	0,98	0,89	dienelactone hydrolase family protein
267115_s_at	At2g32540	0,88	0,96	1,07	1,08	0,78	1,30	1,21	1,12	cellulose synthase family protein
267116_at	At2g32560	1,11	0,74	0,62	1,03	1,12	1,02	1,17	1,39	F-box family protein
267117_at	At2g32570	1,07	0,99	0,91	0,94	0,97	1,03	1,13	0,91	F-box family protein
267060_at	At2g32580	0,89	0,91	0,82	1,10	1,18	0,94	0,95	0,99	expressed protein
267118_at	At2g32590	1,15	1,00	1,22	1,21	1,13	1,11	1,02	0,85	barren family protein
267062_at	At2g32600	1,10	1,06	1,19	0,89	1,20	0,95	0,88	1,03	hydroxyproline-rich glycoprotein family protein
267119_at	At2g32610	0,87	1,08	0,97	1,12	1,06	0,98	1,04	1,09	cellulose synthase family protein
267085_at	At2g32620	1,04	1,17	1,00	1,11	1,10	0,95	1,12	1,06	cellulose synthase family protein
267086_at	At2g32630	0,93	0,97	1,01	0,99	1,19	1,06	1,05	0,93	pentatricopeptide (PPR) repeat-containing protein
267549_at	At2g32640	1,25	1,38	1,34	0,73	0,86	1,20	1,28	1,19	expressed protein
267553_s_at	At2g32650	1,10	0,90	1,03	0,89	0,79	0,94	0,90	0,91	expressed protein
267548_at	At2g32660	0,82	0,73	0,64	1,12	1,09	0,87	1,15	0,98	disease resistance family protein / LRR family protein
267547_at	At2g32670	0,96	1,03	0,92	0,91	1,01	0,93	0,93	0,89	synaptobrevin family protein
267546_at	At2g32680	0,99	0,93	1,14	0,89	1,09	1,08	0,97	1,10	disease resistance family protein
267545_at	At2g32690	0,93	1,03	0,84	1,22	0,86	0,82	0,87	0,76	pseudogene, glycine-rich protein
267558_at	At2g32700	0,92	0,89	0,79	1,14	1,12	0,74	0,95	0,91	WD-40 repeat family protein
267557_at	At2g32710	1,13	1,04	1,10	0,93	0,99	1,00	0,98	1,11	kip-related protein 4 (KRP4) / cyclin-dependent kinase inhibitor 4 (ICK4)
267544_at	At2g32720	1,50	1,48	1,31	1,65	1,44	0,99	0,90	1,06	cytochrome b5, putative
267543_at	At2g32730	1,02	0,98	0,98	0,90	1,01	0,96	0,95	0,92	26S proteasome regulatory subunit, putative
267542_at	At2g32740	0,98	1,00	0,94	1,03	1,09	1,00	1,01	1,06	exostosin family protein
267541_at	At2g32750	0,97	1,00	0,96	1,05	1,02	0,94	0,94	0,97	exostosin family protein
267540_at	At2g32760	0,97	0,94	1,07	1,19	1,17	1,00	0,97	1,00	expressed protein
267555_at	At2g32765	0,79	0,60	0,70	1,00	1,07	0,74	0,74	0,91	small ubiquitin-like modifier 5 (SUMO)
267552_at	At2g32770	1,03	0,88	0,82	1,07	1,13	0,89	0,97	1,20	purple acid phosphatase (PAP13)
267551_at	At2g32780	1,01	0,97	0,96	0,96	1,05	0,98	0,99	1,01	ubiquitin-specific protease 1, putative (UBP1)
267554_at	At2g32790	1,02	0,90	0,89	1,02	1,14	1,08	0,87	0,91	ubiquitin-conjugating enzyme, putative
267550_at	At2g32800	0,98	1,01	1,15	1,33	1,82	1,20	1,20	1,13	protein kinase family protein
267556_at	At2g32810	0,82	0,74	0,69	1,07	1,16	0,97	0,91	0,90	beta-galactosidase, putative / lactase, putative
267646_at	At2g32830	0,94	0,94	0,86	0,92	0,99	0,96	1,02	1,03	inorganic phosphate transporter (PHT5)
267599_at	At2g32850	0,91	0,90	0,80	1,13	1,28	0,96	0,91	0,94	protein kinase family protein

267645_at	At2g32860	0,99	0,76	0,81	0,60	0,68	0,87	0,92	1,24	glycosyl hydrolase family 1 protein
267644_s_at	At2g32880	1,40	1,37	1,53	0,99	1,11	1,00	1,13	1,05	meprin and TRAF homology domain-containing protein / MATH domain-cor
267643_at	At2g32890	1,03	0,95	0,96	1,09	1,05	0,95	1,09	1,06	hypothetical protein
267603_at	At2g32900	1,07	1,04	1,14	1,13	1,02	0,98	0,95	1,15	centromere/kinetochore protein, putative (ZW10)
267642_at	At2g32910	1,12	1,19	1,67	0,98	1,23	1,09	1,00	1,12	expressed protein
267605_at	At2g32920	1,25	1,06	0,90	1,36	1,44	0,97	1,00	1,03	thioredoxin family protein
267604_at	At2g32930	0,83	0,89	1,09	1,07	1,30	0,93	1,08	1,07	zinc finger (CCCH-type) family protein
267641_at	At2g32940	0,89	1,06	0,91	0,90	1,08	0,96	0,98	0,99	PAZ domain-containing protein / piwi domain-containing protein
267640_at	At2g32950	1,19	1,15	1,34	0,94	0,89	1,34	1,35	1,39	COP1 regulatory protein
267602_at	At2g32970	0,79	0,91	0,84	0,84	0,67	1,02	1,03	1,36	expressed protein
267601_at	At2g32980	0,95	0,88	1,19	1,25	1,21	1,00	1,03	0,96	expressed protein
267595_at	At2g32990	0,94	0,94	0,79	1,93	1,57	0,87	0,84	0,70	glycosyl hydrolase family 9 protein
267594_at	At2g33000	0,86	1,02	1,11	0,99	1,06	1,09	0,99	1,14	ubiquitin-associated (UBA)/TS-N domain-containing protein-related
267598_at	At2g33010	0,97	1,09	0,98	0,90	0,92	1,08	1,04	1,12	ubiquitin-associated (UBA)/TS-N domain-containing protein
267597_at	At2g33020	1,07	1,35	1,28	1,02	0,98	1,08	1,02	1,01	leucine-rich repeat family protein
267593_at	At2g33030	1,03	0,95	1,13	1,00	0,88	0,96	1,00	1,03	leucine-rich repeat family protein
267600_at	At2g33040	1,06	1,03	0,94	1,16	1,24	0,93	0,92	0,87	ATP synthase gamma chain, mitochondrial (ATPC)
267596_s_at	At2g33050	1,62	1,37	1,24	0,75	0,72	1,41	0,96	1,03	leucine-rich repeat family protein
245161_at	At2g33070	0,69	0,65	0,54	0,58	0,47	0,96	0,93	0,92	jacalin lectin family protein
245160_at	At2g33080	1,06	1,00	0,98	0,93	1,10	1,02	1,10	1,19	leucine-rich repeat family protein
245159_at	At2g33100	1,07	0,84	0,82	0,96	1,01	1,08	1,13	1,25	cellulose synthase family protein
245167_s_at	At2g33120	0,94	0,79	0,90	0,92	0,98	1,07	1,04	0,84	synaptobrevin-related protein / vesicle-associated membrane protein 722 (
245158_at	At2g33130	1,12	1,10	1,35	1,02	1,03	0,99	1,03	1,04	rapid alkalinization factor (RALF) family protein
245168_at	At2g33150	0,79	0,76	0,81	0,97	0,94	0,93	1,03	0,95	acetyl-CoA C-acyltransferase, putative / 3-ketoacyl-CoA thiolase, putative
245157_at	At2g33160	1,03	0,96	0,96	1,00	1,11	0,93	0,91	1,00	glycoside hydrolase family 28 protein / polygalacturonase (pectinase) famil
245166_at	At2g33170	0,82	1,03	1,28	0,62	1,00	1,03	1,37	1,10	leucine-rich repeat transmembrane protein kinase, putative
245165_at	At2g33180	0,95	0,68	0,64	0,95	0,94	1,01	0,83	0,78	expressed protein
245164_at	At2g33210	1,50	1,39	1,25	1,12	1,07	1,07	1,02	1,05	chaperonin, putative
245169_at	At2g33220	0,94	1,00	0,79	1,16	1,04	0,92	0,91	0,99	expressed protein
245163_at	At2g33230	0,97	0,98	1,03	0,93	1,17	0,96	1,03	0,89	flavin-containing monooxygenase, putative / FMO, putative
245162_at	At2g33240	0,90	1,07	0,86	1,08	1,05	0,94	1,04	1,03	myosin, putative
255793_at	At2g33250	1,16	1,40	1,39	1,29	1,19	1,07	1,02	1,28	expressed protein
255798_at	At2g33255	1,13	1,24	1,38	0,88	0,91	1,21	1,14	1,15	haloacid dehalogenase-like hydrolase family protein
255841_at	At2g33260	1,02	1,01	1,35	1,05	1,17	0,95	0,98	1,03	tryptophan/tyrosine permease family protein
255847_at	At2g33270	0,92	1,00	0,96	0,96	1,09	0,97	0,92	1,08	thioredoxin family protein
255796_at	At2g33290	1,01	0,93	1,10	1,07	0,96	0,95	0,97	0,87	SET domain-containing protein (SUVH2)
255788_at	At2g33310	0,83	1,00	0,91	0,81	0,76	1,13	1,20	1,38	auxin-responsive protein / indoleacetic acid-induced protein 13 (IAA13)
255849_at	At2g33320	1,01	1,01	1,15	1,03	1,13	0,93	1,00	0,79	C2 domain-containing protein
255817_at	At2g33330	0,96	0,80	0,75	0,94	0,89	1,03	0,86	0,75	33 kDa secretory protein-related
255830_at	At2g33340	1,28	1,31	1,22	0,80	1,00	1,02	1,01	0,99	transducin family protein / WD-40 repeat family protein
255831_at	At2g33350	1,03	1,06	1,00	1,00	1,13	0,93	1,04	0,87	hypothetical protein
255832_at	At2g33360	0,98	1,09	1,06	1,01	1,05	0,93	0,90	0,92	expressed protein
255789_at	At2g33370	1,63	1,50	1,26	1,12	1,11	1,00	1,05	0,94	60S ribosomal protein L23 (RPL23B)
255795_at	At2g33380	0,84	0,67	0,81	3,77	2,70	1,00	0,96	0,88	calcium-binding RD20 protein (RD20)
255833_at	At2g33390	0,86	0,87	0,94	0,81	0,90	0,98	1,33	0,98	expressed protein
255834_at	At2g33410	1,01	0,98	0,97	1,22	1,18	0,96	0,97	0,99	heterogeneous nuclear ribonucleoprotein, putative / hnRNP, putative

255835_at	At2g33420	1,19	1,15	0,88	0,91	1,10	1,07	0,96	1,04	expressed protein
255791_at	At2g33430	1,02	0,98	1,10	0,65	0,79	0,97	0,92	0,84	plastid developmental protein DAG, putative
255836_at	At2g33440	0,98	1,18	1,41	0,71	0,65	1,49	1,58	1,43	splicing factor family protein
255850_at	At2g33450	0,99	0,84	0,80	0,76	0,79	0,96	0,99	1,08	50S ribosomal protein L28, chloroplast (CL28)
255837_at	At2g33460	0,96	1,00	1,10	0,87	1,11	1,11	0,95	1,04	p21-rho-binding domain-containing protein
255816_at	At2g33470	1,05	0,95	0,84	1,24	1,28	1,03	0,92	0,86	glycolipid transfer protein-related
255794_at	At2g33480	1,33	1,26	1,49	0,96	1,08	1,20	1,47	1,14	no apical meristem (NAM) family protein
255838_at	At2g33490	0,99	0,95	0,94	0,89	1,27	1,00	0,92	0,97	hydroxyproline-rich glycoprotein family protein
255839_at	At2g33500	0,96	0,94	1,13	0,83	0,94	0,99	0,86	0,94	zinc finger (B-box type) family protein
255840_at	At2g33520	0,81	0,92	0,81	0,90	0,99	1,01	1,05	1,00	expressed protein
255842_at	At2g33530	1,21	1,08	1,05	1,06	1,09	1,17	1,22	1,20	serine carboxypeptidase S10 family protein
255843_at	At2g33540	0,73	0,72	1,07	0,98	1,06	1,11	0,96	1,02	CTD phosphatase-like protein 3 (CPL3)
255790_at	At2g33560	0,97	0,87	1,01	1,29	1,24	0,92	0,96	0,91	spindle checkpoint protein-related
255818_at	At2g33570	0,88	0,70	0,74	0,93	1,06	1,00	0,86	0,87	expressed protein
255844_at	At2g33580	0,49	0,63	0,79	0,76	0,50	1,33	1,20	1,32	protein kinase family protein / peptidoglycan-binding LysM domain-containi
255787_at	At2g33590	0,81	0,72	0,72	0,99	0,91	1,07	1,00	0,88	cinnamoyl-CoA reductase family
255845_at	At2g33600	0,93	0,88	0,92	1,19	1,38	1,00	0,90	0,86	cinnamoyl-CoA reductase family
255846_at	At2g33610	1,03	1,02	1,08	1,06	1,09	1,07	0,90	0,87	SWIRM domain-containing protein / DNA-binding family protein
255792_at	At2g33620	1,06	0,90	0,90	0,97	0,83	1,04	1,00	1,02	DNA-binding family protein / AT-hook protein 1 (AHP1)
255797_at	At2g33630	0,80	0,75	0,89	0,99	0,89	0,92	0,81	0,77	3-beta hydroxysteroid dehydrogenase/isomerase family protein
255848_at	At2g33640	1,06	1,01	1,09	0,92	1,13	0,98	1,12	1,12	zinc finger (DHHC type) family protein
267458_at	At2g33670	1,02	1,09	0,88	1,01	1,02	0,95	0,94	0,76	seven transmembrane MLO family protein / MLO-like protein 5 (MLO5)
267445_at	At2g33680	0,95	0,92	1,09	0,80	0,76	1,06	0,94	0,98	pentatricopeptide (PPR) repeat-containing protein
267449_at	At2g33690	0,96	0,99	0,93	0,99	1,27	1,01	0,90	0,87	late embryogenesis abundant protein, putative / LEA protein, putative
267448_at	At2g33700	1,10	1,16	0,91	0,87	1,08	1,11	1,18	1,51	protein phosphatase 2C, putative / PP2C, putative
267451_at	At2g33710	1,03	1,07	0,98	0,94	1,17	1,00	1,14	1,16	AP2 domain-containing transcription factor family protein
267450_at	At2g33720	1,02	1,06	0,87	0,98	1,15	1,05	0,93	1,12	expressed protein
267454_at	At2g33730	1,07	1,21	1,17	1,05	1,03	1,11	0,95	1,12	DEAD box RNA helicase, putative
267462_at	At2g33735	0,98	0,99	1,05	0,92	0,82	1,03	0,98	0,97	DNAJ heat shock N-terminal domain-containing protein
267405_at	At2g33740	0,83	0,85	0,81	1,00	1,02	0,90	0,93	0,94	copper-binding protein (CUTA)
267455_at	At2g33760	0,99	1,06	1,29	1,07	0,97	0,97	1,06	0,94	pentatricopeptide (PPR) repeat-containing protein
267456_at	At2g33770	1,21	1,10	1,13	1,03	1,00	1,39	1,38	1,12	ubiquitin-conjugating enzyme family protein
267457_at	At2g33790	0,87	0,70	0,56	2,72	2,46	1,33	1,86	1,74	pollen Ole e 1 allergen and extensin family protein
267435_at	At2g33800	0,97	0,92	0,91	0,94	0,94	1,01	0,96	0,98	ribosomal protein S5 family protein
267460_at	At2g33810	0,77	0,96	0,92	1,46	1,19	0,73	0,88	0,81	squamosa promoter-binding protein-like 3 (SPL3)
257351_at	At2g33820	1,05	1,29	1,24	0,70	0,91	0,99	1,15	1,03	mitochondrial substrate carrier family protein (BAC1)
267461_at	At2g33830	0,71	0,93	1,89	0,97	1,08	0,80	0,75	0,83	dormancy/auxin associated family protein
267446_s_at	At2g33840	1,07	0,92	0,86	0,84	0,83	0,98	1,01	1,03	tRNA synthetase class I (W and Y) family protein
267463_at	At2g33845	1,27	1,04	0,92	1,05	1,14	0,94	0,82	0,80	DNA-binding protein-related
267459_at	At2g33850	1,13	1,14	0,99	2,51	2,52	2,15	1,29	1,32	expressed protein
267452_at	At2g33860	0,83	0,97	1,37	0,87	0,86	0,91	0,98	1,19	auxin-responsive factor (ARF3) / ETTIN protein (ETT)
267447_at	At2g33870	0,98	1,02	0,98	1,01	0,90	0,97	0,97	0,91	Ras-related GTP-binding protein, putative
267453_at	At2g33880	0,99	1,06	0,90	0,99	1,16	0,98	1,02	1,13	expressed protein
267404_at	At2g33980	0,96	1,09	0,98	0,74	1,04	0,99	1,09	0,89	MutT/nudix family protein
257573_at	At2g33990	1,31	1,19	1,91	1,04	1,10	0,88	0,96	1,01	calmodulin-binding family protein
256712_at	At2g34020	0,98	0,94	0,98	1,10	1,26	1,00	1,12	1,36	calcium-binding EF hand family protein

256724_at	At2g34040	1,35	1,16	1,32	0,98	0,89	1,16	1,03	1,10	apoptosis inhibitory 5 (API5) family protein
256722_at	At2g34050	0,94	0,83	0,82	1,19	1,40	0,97	0,81	0,83	expressed protein
256713_at	At2g34060	0,87	0,74	0,87	0,91	1,01	0,99	0,89	0,75	peroxidase, putative
256725_at	At2g34070	0,99	0,77	0,73	1,09	1,22	0,86	0,80	0,92	expressed protein
256714_at	At2g34080	0,65	0,42	0,16	0,65	0,53	0,94	1,16	0,87	cysteine proteinase, putative
256715_at	At2g34090	0,73	0,68	0,96	0,76	0,86	0,90	1,13	1,11	expressed protein
256716_at	At2g34100	1,06	1,04	1,08	1,09	1,05	0,94	0,97	0,98	expressed protein
256717_at	At2g34110	0,96	0,93	1,04	0,94	0,97	0,89	1,10	0,97	hypothetical protein
256718_at	At2g34120	0,96	0,99	1,06	0,93	1,05	0,95	0,95	1,13	hypothetical protein
256719_at	At2g34130	0,90	1,06	1,04	0,99	1,08	0,93	1,06	0,99	---
256720_at	At2g34140	0,99	1,09	1,01	1,31	1,30	0,85	1,04	1,26	Dof-type zinc finger domain-containing protein
256721_at	At2g34150	1,15	1,20	1,17	0,89	1,00	0,96	1,10	1,04	expressed protein
256723_at	At2g34160	1,20	1,05	0,92	0,90	0,97	1,04	1,06	1,08	expressed protein
267045_at	At2g34180	0,91	1,01	0,91	0,97	0,96	1,09	0,97	0,93	CBL-interacting protein kinase 13 (CIPK13)
267006_at	At2g34190	1,06	1,05	1,05	1,03	0,98	0,96	0,94	0,98	xanthine/uracil permease family protein
267048_at	At2g34200	0,77	0,69	0,95	0,98	0,87	0,71	0,80	1,21	zinc finger (C3HC4-type RING finger) family protein
267049_at	At2g34210	0,97	1,03	1,00	1,00	0,97	1,07	1,06	1,01	KOW domain-containing transcription factor family protein
267022_at	At2g34230	1,01	0,94	1,17	0,94	1,08	1,02	0,99	1,22	hypothetical protein
267023_at	At2g34250	0,94	0,87	0,89	1,09	1,08	0,89	0,85	0,84	protein transport protein sec61, putative
267004_at	At2g34260	1,20	1,13	1,37	1,00	0,94	0,90	0,90	1,01	transducin family protein / WD-40 repeat family protein
267039_at	At2g34270	1,06	1,03	0,99	0,98	0,99	1,15	1,01	0,86	hypothetical protein
257359_x_at	At2g34290	0,87	1,05	1,00	0,90	0,93	1,03	0,97	1,06	protein kinase family protein
267040_at	At2g34300	1,16	1,02	0,87	1,60	1,60	0,97	0,88	1,07	dehydration-responsive protein-related
267000_at	At2g34310	0,93	0,90	0,96	0,82	0,78	1,23	1,17	1,23	expressed protein
267041_at	At2g34315	0,88	0,90	0,98	1,05	1,24	1,03	1,00	0,89	disease resistance protein-related
267042_at	At2g34320	0,91	1,06	1,04	0,82	0,96	0,94	1,00	0,91	hypothetical protein
267043_at	At2g34330	1,04	0,99	0,93	1,05	1,09	0,98	0,89	1,06	expressed protein
267003_at	At2g34340	0,87	0,94	0,98	0,91	1,17	1,07	1,09	0,98	expressed protein
267044_at	At2g34350	1,81	1,53	1,61	0,84	0,84	1,24	1,23	1,06	expressed protein
267046_at	At2g34360	0,95	0,83	0,91	0,97	1,06	1,06	1,00	1,03	MATE efflux family protein
267047_at	At2g34370	1,04	0,99	1,06	1,05	1,11	0,90	1,12	0,95	pentatricopeptide (PPR) repeat-containing protein
267024_s_at	At2g34390	0,86	0,94	0,96	1,05	1,14	1,08	0,67	0,83	major intrinsic family protein / MIP family protein
266998_at	At2g34400	1,03	1,05	0,99	0,86	0,87	0,98	1,03	0,78	pentatricopeptide (PPR) repeat-containing protein
266997_at	At2g34410	1,00	1,04	1,21	0,93	1,03	1,04	0,95	0,86	O-acetyltransferase family protein
267002_s_at	At2g34430	0,96	0,96	1,06	0,82	0,79	0,98	1,01	0,99	chlorophyll A-B binding protein / LHCII type I (LHB1B2)
266994_at	At2g34440	0,94	1,02	1,14	0,90	1,28	1,12	1,00	0,94	MADS-box family protein
266999_at	At2g34450	1,21	1,19	1,22	1,17	1,32	1,04	1,06	1,04	high mobility group (HMG1/2) family protein
267005_at	At2g34460	1,21	1,18	1,24	1,20	1,11	1,20	1,07	1,07	flavin reductase-related
267001_at	At2g34470	0,95	0,94	0,91	0,84	0,97	0,92	0,96	0,94	urease accessory protein (UREG)
267007_at	At2g34480	1,32	1,12	1,08	1,13	1,06	0,99	0,94	0,95	60S ribosomal protein L18A (RPL18aB)
266996_at	At2g34490	0,89	0,78	0,86	1,63	1,96	0,70	0,60	0,54	cytochrome P450 family protein
266995_at	At2g34500	0,99	1,10	0,87	0,98	0,90	1,06	0,92	0,93	cytochrome P450 family protein
266956_at	At2g34510	0,87	0,81	1,01	0,68	0,66	0,92	0,81	0,87	expressed protein
266955_at	At2g34520	1,24	1,24	1,38	1,07	1,15	0,94	0,80	0,87	ribosomal protein S14 mitochondrial family protein
266954_at	At2g34530	1,16	1,16	0,99	1,06	1,14	0,96	0,97	0,99	expressed protein
266953_at	At2g34540	1,04	0,96	0,95	1,08	1,10	1,19	1,07	1,07	expressed protein

266907_at	At2g34550	1,03	0,97	1,03	0,98	1,12	0,95	0,86	0,92	gibberellin 2-oxidase / GA2-oxidase (GA2OX3)
266952_at	At2g34555	0,93	1,12	0,94	1,00	1,15	1,25	0,98	0,94	gibberellin 2-oxidase / GA2-oxidase (GA2OX3)
266905_at	At2g34560	1,01	1,03	1,04	0,98	0,96	0,91	1,04	1,08	katanin, putative
266903_at	At2g34570	1,25	1,20	0,85	0,96	1,38	1,04	0,87	0,71	expressed protein
266902_at	At2g34580	0,97	0,85	1,12	0,95	1,12	0,99	0,98	0,99	hypothetical protein
266906_at	At2g34585	0,96	0,75	0,80	1,18	1,36	0,82	0,77	0,66	expressed protein
266904_at	At2g34590	1,08	0,97	0,99	0,87	0,85	0,88	0,87	0,84	transketolase family protein
266901_at	At2g34600	0,90	2,27	1,55	1,00	0,52	1,21	2,50	2,52	expressed protein
266900_at	At2g34610	1,00	0,97	1,00	1,05	0,90	1,02	1,03	1,07	expressed protein
266899_at	At2g34620	1,24	1,55	1,45	0,78	0,70	0,94	0,87	1,20	mitochondrial transcription termination factor-related / mTERF-related
266958_at	At2g34630	1,20	1,15	1,07	1,06	1,15	1,29	1,00	1,04	geranyl diphosphate synthase, putative / GPPS, putative / dimethylallyltrans
266957_at	At2g34640	1,01	0,77	0,80	0,68	0,70	1,18	1,17	1,33	expressed protein
266908_at	At2g34650	0,83	0,98	1,00	0,90	0,80	1,10	0,91	0,99	protein kinase PINOID (PID)
267319_at	At2g34660	0,69	0,60	0,71	0,86	0,72	1,02	1,15	1,33	glutathione S-conjugate ABC transporter (MRP2)
267311_at	At2g34670	0,91	1,15	1,24	0,99	1,23	1,22	1,09	0,84	proline-rich family protein
267310_at	At2g34680	0,95	0,97	0,89	1,15	1,04	0,91	0,98	0,88	leucine-rich repeat family protein
267340_at	At2g34690	0,95	0,94	0,85	1,03	1,13	0,86	0,90	1,02	expressed protein
267317_at	At2g34700	4,89	5,40	5,85	1,19	1,02	1,07	0,95	0,92	pollen Ole e 1 allergen and extensin family protein
267316_at	At2g34710	1,42	1,30	1,18	0,96	0,87	1,03	1,07	0,92	homeobox-leucine zipper transcription factor (HB-14)
267315_at	At2g34720	1,13	0,97	0,99	0,88	0,96	1,15	0,99	0,97	CCAAT-binding transcription factor (CBF-B/NF-YA) family protein
267314_at	At2g34730	1,16	1,12	1,09	0,84	0,97	1,01	1,16	1,25	myosin heavy chain-related
267313_at	At2g34740	0,92	0,95	0,93	1,08	0,90	0,99	1,08	0,94	protein phosphatase 2C, putative / PP2C, putative
267312_at	At2g34750	1,05	0,97	1,13	0,84	0,82	0,97	0,93	0,96	RNA polymerase I specific transcription initiation factor RRN3 family proteir
267318_at	At2g34770	0,92	0,86	1,09	1,07	0,96	1,01	1,05	1,21	fatty acid hydroxylase (FAH1)
267406_at	At2g34780	1,41	1,38	2,05	0,99	0,96	1,17	1,33	1,38	expressed protein
267414_at	At2g34790	1,28	0,96	1,13	1,59	1,36	0,99	1,20	1,06	FAD-binding domain-containing protein
267424_at	At2g34800	0,99	1,05	1,06	1,00	1,04	0,98	1,02	0,98	hypothetical protein
267425_at	At2g34810	1,05	0,70	0,72	2,71	1,57	1,10	0,83	0,73	FAD-binding domain-containing protein
267426_at	At2g34820	0,98	0,98	1,11	1,00	1,11	0,93	1,05	1,11	basic helix-loop-helix (bHLH) family protein
267427_at	At2g34830	1,13	1,02	1,04	1,00	1,09	1,17	1,09	0,84	WRKY family transcription factor
267428_at	At2g34840	1,03	1,03	1,05	0,98	0,99	0,95	1,05	0,97	coatomer protein epsilon subunit family protein / COPE family protein
267429_at	At2g34850	0,79	0,96	0,93	1,02	1,29	0,95	1,05	1,01	NAD-dependent epimerase/dehydratase family protein
267430_at	At2g34860	1,19	1,00	0,95	0,83	0,66	1,18	1,23	1,15	chaperone protein dnaJ-related
267431_at	At2g34870	0,98	0,93	1,01	0,90	1,02	0,92	0,97	0,81	hydroxyproline-rich glycoprotein family protein
267407_at	At2g34880	1,01	1,15	0,86	0,94	0,98	1,02	0,99	0,91	transcription factor jumonji (jmj) family protein / zinc finger (C5HC2 type) far
267408_at	At2g34890	0,94	0,88	1,32	0,91	1,07	1,01	0,97	0,75	CTP synthase, putative / UTP--ammonia ligase, putative
257352_at	At2g34900	1,09	0,96	1,05	1,02	0,92	1,10	1,08	1,13	DNA-binding bromodomain-containing protein
267409_at	At2g34910	1,02	1,16	1,03	1,64	1,58	1,40	1,13	1,07	expressed protein
267410_at	At2g34920	1,14	1,18	1,07	1,16	1,18	1,21	1,01	0,85	ubiquitin-protein ligase-related
267411_at	At2g34930	1,08	0,88	0,93	1,08	1,37	1,20	0,99	0,90	disease resistance family protein
267412_at	At2g34940	0,86	0,83	0,89	1,08	1,25	0,94	1,07	0,92	vacuolar sorting receptor, putative
267413_at	At2g34960	0,81	0,92	0,86	1,00	0,96	1,00	0,85	0,86	amino acid permease family protein
267415_at	At2g34970	1,06	1,09	0,88	0,90	0,92	1,11	0,78	0,74	eIF4-gamma/eIF5/eIF2-epsilon domain-containing protein
267416_at	At2g34980	1,05	1,01	1,20	1,35	1,15	0,92	0,93	0,98	phosphatidylinositolglycan synthase family protein
267417_at	At2g34990	0,98	0,98	0,96	1,04	0,79	0,93	0,83	0,86	zinc finger (C3HC4-type RING finger) family protein
267418_at	At2g35000	0,82	0,81	0,67	1,01	0,99	0,76	1,05	1,06	zinc finger (C3HC4-type RING finger) family protein

267419_at	At2g35010	1,07	1,02	0,98	1,07	1,21	1,03	0,92	0,92 thioredoxin family protein
267432_at	At2g35020	1,03	0,76	0,80	1,19	1,21	0,77	0,78	0,75 UTP--glucose-1-phosphate uridylyltransferase family protein
267420_at	At2g35030	0,96	0,82	1,12	0,83	0,92	0,95	0,83	0,83 pentatricopeptide (PPR) repeat-containing protein
267421_at	At2g35040	1,07	0,92	0,89	0,89	0,87	0,95	0,92	0,83 AICARFT/IMPCHase bienzyme family protein
267422_at	At2g35050	0,91	0,86	0,85	0,89	0,93	0,89	0,92	1,00 protein kinase family protein
267423_at	At2g35060	0,71	0,79	0,80	0,66	0,62	1,19	1,26	1,15 potassium transporter family protein
266550_s_at	At2g35070	0,99	0,93	1,00	0,99	1,01	0,99	1,05	1,19 expressed protein
266543_at	At2g35075	1,19	0,90	1,00	0,89	1,06	0,93	1,02	1,14 hypothetical protein
257370_at	At2g35080	0,95	1,06	1,36	1,06	1,04	1,06	1,08	0,84 expressed protein
266542_at	At2g35100	0,86	1,05	0,80	0,96	1,08	0,84	0,95	1,31 exostosin family protein
266541_at	At2g35110	1,09	0,98	1,18	0,95	0,83	1,02	1,02	1,02 HEM protein-related
266517_at	At2g35120	1,57	1,48	1,13	2,22	1,61	0,93	1,00	1,03 glycine cleavage system H protein, mitochondrial, putative
266549_at	At2g35150	1,02	0,92	1,11	1,04	1,05	0,99	1,07	0,94 phosphate-responsive 1 family protein
266519_at	At2g35155	0,99	1,18	1,36	1,06	0,78	1,04	0,89	0,86 expressed protein
266539_at	At2g35160	0,98	1,06	0,96	0,95	1,08	0,96	0,84	1,08 SET domain-containing protein (SUVH5)
266518_at	At2g35170	1,31	1,26	0,95	0,80	0,93	1,16	0,97	1,00 MORN (Membrane Occupation and Recognition Nexus) repeat-containing p
266489_at	At2g35190	0,88	0,77	0,72	1,45	1,45	0,75	0,74	0,76 novel plant SNARE 11 (NPSN11)
266548_at	At2g35210	1,10	1,26	1,25	1,19	1,56	1,18	1,08	1,22 human Rev interacting-like family protein / hRIP family protein
266538_at	At2g35230	0,90	0,86	0,74	0,84	0,98	0,82	0,83	1,02 VQ motif-containing protein
266551_at	At2g35260	1,15	1,42	1,83	0,64	0,42	1,24	1,38	1,67 expressed protein
266546_at	At2g35270	0,86	0,85	0,58	1,33	1,09	0,88	1,12	1,05 DNA-binding protein-related
266545_at	At2g35290	0,94	1,15	2,28	1,27	1,81	0,94	0,94	1,03 expressed protein
266544_at	At2g35300	0,09	0,10	0,07	0,85	0,78	1,14	1,35	1,13 late embryogenesis abundant group 1 domain-containing protein / LEA grou
266540_at	At2g35310	0,95	1,03	0,83	0,99	1,01	0,87	1,02	0,94 transcriptional factor B3 family protein
266547_at	At2g35320	0,97	1,08	1,46	1,01	1,09	0,91	0,92	1,29 expressed protein
266628_at	At2g35330	1,24	1,17	1,37	1,13	1,01	1,03	1,18	1,12 zinc finger (C3HC4-type RING finger) protein-related
266627_at	At2g35340	1,33	1,13	1,76	1,06	1,05	1,09	1,32	1,22 RNA helicase, putative
266626_at	At2g35360	1,02	0,86	1,05	1,03	0,99	0,80	0,72	0,66 ubiquitin family protein
266636_at	At2g35370	0,91	0,82	0,78	0,86	0,94	1,02	1,07	1,10 glycine cleavage system H protein 1, mitochondrial (GDCSH) (GCDH)
266625_at	At2g35380	1,48	1,25	1,11	1,43	1,56	1,01	1,08	1,06 peroxidase 20 (PER20) (P20)
266624_s_at	At2g35390	0,86	0,85	0,91	0,82	0,65	1,19	1,10	0,90 ribose-phosphate pyrophosphokinase 2 / phosphoribosyl diphosphate synth
266623_at	At2g35400	0,99	0,86	1,00	0,83	0,78	1,04	0,98	1,03 ribose-phosphate pyrophosphokinase 1 / phosphoribosyl diphosphate synth
266642_at	At2g35410	0,84	0,71	0,75	0,93	0,95	0,99	0,98	0,90 33 kDa ribonucleoprotein, chloroplast, putative / RNA-binding protein cp33,
266622_at	At2g35430	1,19	1,10	1,18	1,04	0,88	1,11	0,95	0,93 zinc finger (CCCH-type) family protein
266621_at	At2g35440	0,76	0,81	0,72	0,66	0,68	0,96	0,93	1,02 amidohydrolase family
266620_at	At2g35450	0,71	0,75	0,63	0,76	0,66	0,92	0,88	0,86 amidohydrolase family
266619_at	At2g35460	0,97	1,00	1,08	1,18	0,95	0,95	1,00	1,05 harpin-induced family protein / HIN1 family protein / harpin-responsive famil
266635_at	At2g35470	1,32	1,50	0,94	1,19	1,30	0,81	0,80	0,96 expressed protein
266618_at	At2g35480	0,95	1,06	1,14	0,98	0,95	0,87	0,92	0,92 expressed protein
266638_at	At2g35490	0,93	0,83	0,91	0,91	0,86	0,98	0,96	0,98 plastid-lipid associated protein PAP, putative
266608_at	At2g35500	0,94	0,96	0,97	0,81	1,00	0,98	0,95	0,93 shikimate kinase-related
266609_at	At2g35510	0,75	0,81	0,96	0,81	1,10	1,00	0,97	0,97 WWE domain-containing protein
266639_at	At2g35520	1,09	0,99	0,74	1,37	0,94	0,95	0,85	0,82 defender against cell death 2 (DAD2)
266634_at	At2g35530	1,02	1,04	1,02	0,94	1,01	1,06	1,10	1,57 bZIP transcription factor family protein
266633_at	At2g35540	0,88	0,93	1,10	0,94	0,89	0,77	0,86	0,97 DNAJ heat shock N-terminal domain-containing protein
257369_at	At2g35550	1,02	1,05	1,01	1,11	1,31	1,05	0,95	0,98 expressed protein

266632_at	At2g35560	1,01	1,03	0,82	0,95	0,97	0,98	0,97	0,93	---
266631_at	At2g35570	1,01	0,98	1,06	1,15	1,05	1,03	1,03	0,90	---
266630_at	At2g35580	1,00	1,08	1,04	1,09	0,88	0,96	0,93	1,03	serpin family protein / serine protease inhibitor family protein
266640_at	At2g35585	1,02	1,03	1,03	1,06	1,03	0,88	0,99	1,06	expressed protein
266629_at	At2g35590	1,01	1,08	0,95	1,02	1,00	0,89	0,91	1,10	---
266637_at	At2g35600	0,91	0,99	0,92	1,06	0,98	1,04	0,91	0,93	expressed protein
266641_at	At2g35605	1,62	1,38	1,18	1,69	1,61	1,10	0,93	0,86	SWIB complex BAF60b domain-containing protein
265845_at	At2g35610	0,93	0,91	0,70	1,47	1,25	1,01	0,88	0,84	expressed protein
265844_at	At2g35620	0,91	0,92	0,71	1,08	1,07	0,86	0,84	0,84	leucine-rich repeat transmembrane protein kinase, putative
265800_at	At2g35630	1,04	0,90	1,12	0,80	0,83	0,89	1,12	1,22	microtubule organization 1 protein (MOR1)
265825_at	At2g35635	1,25	1,10	1,14	1,38	1,55	0,94	0,90	0,96	ubiquitin family protein
265824_at	At2g35650	0,88	1,09	1,29	0,94	1,11	1,00	1,19	1,18	glycosyl transferase family 2 protein
265801_at	At2g35670	1,06	0,99	1,02	1,03	1,05	0,99	1,00	1,01	transcription factor, putative / fertilization-independent seed 2 protein (FIS2)
265799_at	At2g35680	0,92	0,97	1,01	0,87	1,01	0,99	1,01	1,26	dual specificity protein phosphatase family protein
265843_at	At2g35690	1,01	0,92	1,06	0,76	0,95	1,08	1,09	1,08	acyl-CoA oxidase, putative
265842_at	At2g35700	0,94	1,01	0,91	1,05	1,08	0,94	0,92	0,92	AP2 domain-containing transcription factor, putative
265841_at	At2g35710	0,69	1,20	1,50	1,00	1,02	1,05	1,70	1,72	glycogenin glucosyltransferase (glycogenin)-related
265797_at	At2g35715	0,54	1,14	1,29	0,90	1,04	0,91	1,60	1,52	glycogenin glucosyltransferase (glycogenin)-related
265850_at	At2g35720	1,22	0,94	1,01	0,92	0,98	0,88	0,85	0,80	DNAJ heat shock N-terminal domain-containing protein
265796_at	At2g35730	1,01	0,89	1,03	1,02	0,97	0,96	0,99	0,97	heavy-metal-associated domain-containing protein
265802_at	At2g35733	1,00	1,12	0,92	0,97	1,07	0,95	0,97	0,87	hypothetical protein
265849_at	At2g35736	1,23	1,09	0,79	0,93	0,89	1,08	0,91	0,84	expressed protein
265848_at	At2g35740	0,96	1,10	0,99	1,03	1,23	0,86	1,00	0,93	sugar transporter family protein
265847_at	At2g35750	0,75	0,61	0,72	0,62	0,67	0,94	0,88	0,91	expressed protein
265823_at	At2g35760	0,74	0,65	0,63	2,18	1,35	0,73	0,85	0,84	integral membrane family protein
265846_at	At2g35770	1,05	1,18	0,98	1,05	1,37	0,98	0,96	1,09	serine carboxypeptidase S10 family protein
265795_at	At2g35780	0,79	0,78	0,81	0,99	1,11	0,83	0,81	0,76	serine carboxypeptidase S10 family protein
265798_at	At2g35790	1,24	1,25	1,06	1,12	1,18	0,96	0,92	0,92	expressed protein
265826_at	At2g35795	1,33	1,07	0,93	1,23	1,14	1,06	0,98	1,00	DNAJ heat shock N-terminal domain-containing protein
263943_at	At2g35800	1,15	1,45	1,46	0,89	1,05	0,97	0,99	1,08	mitochondrial substrate carrier family protein
263952_s_at	At2g35810	1,05	0,98	1,03	0,98	1,01	1,13	1,10	1,11	expressed protein
263947_at	At2g35820	1,07	0,88	0,86	0,77	0,87	0,81	1,04	0,86	expressed protein
263954_at	At2g35840	1,18	1,04	1,11	1,01	1,13	1,23	1,31	1,34	sucrose-phosphatase 1 (SPP1)
263942_at	At2g35860	1,19	1,01	0,78	1,62	1,61	0,78	0,64	0,67	beta-Ig-H3 domain-containing protein / fasciclin domain-containing protein
263941_at	At2g35870	1,05	0,95	0,97	0,85	1,09	1,04	0,82	0,96	---
263957_at	At2g35880	0,98	0,99	1,35	0,94	0,91	1,06	1,11	1,15	expressed protein
263940_at	At2g35890	0,97	0,96	0,98	1,00	1,20	1,00	0,99	0,93	calcium-dependent protein kinase, putative / CDPK, putative
263938_at	At2g35900	1,15	1,01	0,93	1,05	1,27	0,99	0,82	0,90	expressed protein
263937_at	At2g35910	0,74	0,85	0,99	0,95	1,01	0,94	0,92	1,08	zinc finger (C3HC4-type RING finger) family protein
263936_at	At2g35920	0,97	0,84	0,97	0,84	0,70	1,03	0,82	1,06	helicase domain-containing protein
263935_at	At2g35930	1,02	1,32	1,04	1,06	1,09	1,28	1,68	1,55	U-box domain-containing protein
263956_at	At2g35940	0,86	0,78	0,90	0,90	0,79	0,92	1,13	1,24	homeodomain-containing protein
263934_at	At2g35950	0,97	1,06	1,12	1,00	1,04	0,95	0,99	0,89	expressed protein
263951_at	At2g35960	1,62	1,16	1,03	1,89	1,74	1,12	0,78	0,54	harpin-induced family protein / HIN1 family protein / harpin-responsive famil
263933_s_at	At2g35970	1,07	0,96	0,95	1,01	1,04	0,97	1,00	1,08	harpin-induced family protein / HIN1 family protein / harpin-responsive famil
263948_at	At2g35980	1,30	1,21	1,08	1,24	0,96	1,93	1,50	1,98	harpin-induced family protein (YLS9) / HIN1 family protein / harpin-responsi

263932_at	At2g35990	0,97	0,94	0,94	0,90	1,22	0,96	1,03	1,04 hypothetical protein
263946_at	At2g36000	0,83	1,12	1,09	0,75	0,73	0,80	0,88	0,70 mitochondrial transcription termination factor-related / mTERF-related
263955_at	At2g36010	1,06	0,95	1,07	0,83	1,14	1,10	0,90	1,10 E2F transcription factor-3 (E2F3)
263950_at	At2g36020	1,00	1,06	0,98	0,99	1,35	1,12	0,95	0,95 abscisic acid-responsive HVA22 family protein
263945_at	At2g36030	0,98	0,95	0,96	1,08	0,98	1,08	1,03	0,84 hypothetical protein
263944_at	At2g36040	0,98	0,95	0,94	0,95	0,96	0,96	1,16	1,03 expressed protein
263953_at	At2g36050	0,94	0,97	0,98	0,92	1,09	0,81	0,87	0,96 ovate protein-related
263949_at	At2g36060	1,12	1,04	1,02	0,93	1,00	0,90	0,90	0,99 ubiquitin-conjugating enzyme family protein
263939_at	At2g36070	1,15	1,01	0,83	0,87	0,93	1,11	0,96	1,11 mitochondrial import inner membrane translocase subunit TIM44, putative
263963_at	At2g36080	1,73	2,17	1,87	1,05	1,13	0,78	1,08	0,83 DNA-binding protein, putative
263283_at	At2g36090	0,99	0,98	0,88	1,02	1,23	0,83	1,00	1,05 F-box family protein
263284_at	At2g36100	1,24	1,19	0,81	1,68	1,34	1,17	1,37	1,32 integral membrane family protein
263285_at	At2g36120	1,20	1,54	1,22	1,45	1,36	1,03	1,50	1,43 pseudogene, glycine-rich protein
263288_at	At2g36130	1,26	0,99	1,17	1,20	1,36	0,94	0,92	0,90 peptidyl-prolyl cis-trans isomerase, putative / cyclophilin, putative / rotamas
263287_at	At2g36145	0,89	0,82	0,82	1,24	1,25	1,34	1,11	1,05 expressed protein
263286_at	At2g36160	1,32	1,26	1,16	1,44	1,17	0,98	0,97	1,00 40S ribosomal protein S14 (RPS14A)
263289_at	At2g36170	1,53	1,29	1,13	1,31	1,28	1,04	0,97	0,99 ubiquitin extension protein 2 (UBQ2) / 60S ribosomal protein L40 (RPL40A)
263903_at	At2g36180	1,01	1,00	0,95	0,86	0,98	1,04	0,98	0,90 calmodulin-related protein, putative
263905_at	At2g36190	1,00	1,04	1,08	1,04	1,18	1,02	0,83	0,94 beta-fructosidase, putative / beta-fructofuranosidase, putative
263960_at	At2g36200	1,22	1,07	1,04	1,33	1,40	0,97	0,98	1,21 kinesin motor protein-related
263959_at	At2g36210	0,94	1,02	0,82	0,99	1,09	0,88	0,95	1,10 auxin-responsive family protein
263931_at	At2g36220	0,95	1,11	0,90	0,91	0,93	1,08	1,31	1,22 expressed protein
263902_at	At2g36230	2,17	1,93	1,73	0,95	0,87	1,44	1,30	1,17 N'-5'-phosphoribosyl-formimino-5-aminoimidazole-4-carboxamide ribonucle
263958_at	At2g36240	0,92	0,90	0,97	0,77	0,79	1,01	0,98	0,89 pentatricopeptide (PPR) repeat-containing protein
263906_at	At2g36250	1,00	0,84	0,83	0,69	0,72	0,93	0,97	1,05 chloroplast division protein FtsZ (FtsZ2-1)
263907_at	At2g36270	0,67	0,63	0,80	0,92	1,00	0,91	1,03	0,93 bZIP transcription factor family protein / ABA-responsive element-binding p
263900_at	At2g36290	0,97	0,94	0,81	1,32	1,09	1,03	1,07	1,18 hydrolase, alpha/beta fold family protein
263930_at	At2g36300	0,81	0,80	0,82	1,08	1,11	0,82	0,81	0,83 integral membrane Yip1 family protein
263929_at	At2g36310	0,98	0,90	1,26	0,94	1,04	1,00	1,05	1,13 inosine-uridine preferring nucleoside hydrolase family protein
263901_at	At2g36320	0,92	0,95	0,96	1,07	0,91	1,06	1,14	0,95 zinc finger (AN1-like) family protein
263928_at	At2g36330	0,90	1,01	0,83	1,04	1,19	0,88	0,89	0,73 integral membrane protein, putative
263962_at	At2g36350	0,88	1,03	1,10	0,89	0,99	0,79	0,92	0,97 protein kinase, putative
257431_at	At2g36360	0,76	0,91	0,91	0,88	0,93	0,97	0,97	0,90 kelch repeat-containing protein
263961_at	At2g36370	1,14	1,14	1,18	0,97	0,95	0,98	1,07	1,20 F-box family protein (FBL11)
263904_at	At2g36380	0,64	0,61	0,77	0,91	0,84	1,01	0,97	1,02 ABC transporter family protein
263912_at	At2g36390	0,47	0,43	0,42	0,59	0,66	0,89	1,01	0,99 1,4-alpha-glucan branching enzyme / starch branching enzyme class II (SB
263914_at	At2g36400	1,86	1,81	1,46	1,09	1,70	0,93	0,78	0,94 expressed protein
263920_at	At2g36410	0,83	0,80	1,07	1,11	1,19	0,91	0,98	1,22 expressed protein
263915_at	At2g36430	0,94	0,93	0,85	0,59	0,39	1,04	1,01	1,02 expressed protein
263916_at	At2g36440	1,03	0,91	1,04	0,99	1,07	1,05	1,00	0,90 hypothetical protein
263917_at	At2g36450	1,09	1,17	1,04	1,21	0,99	0,96	1,25	0,94 AP2 domain-containing protein
263921_at	At2g36460	0,93	1,06	1,21	1,02	1,02	0,94	0,92	0,93 fructose-bisphosphate aldolase, putative
263919_at	At2g36470	0,95	0,97	1,05	1,03	1,02	0,88	0,85	0,98 expressed protein
263908_at	At2g36480	0,74	0,78	0,93	0,82	1,08	1,07	0,87	0,98 zinc finger (C2H2-type) family protein
263923_at	At2g36485	0,97	0,92	1,14	0,75	1,14	1,06	1,05	1,00 expressed protein
263909_at	At2g36490	0,49	0,38	0,38	1,17	1,25	0,79	0,77	0,84 HhH-GPD base excision DNA repair family protein (ROS1)

263924_at	At2g36530	1,13	1,01	0,96	1,28	1,26	1,01	0,93	0,98	enolase
263910_at	At2g36550	0,97	1,00	1,01	0,95	1,08	0,96	1,04	0,90	expressed protein
263911_at	At2g36560	0,98	1,17	1,10	0,97	1,07	0,98	1,04	0,89	DNA-binding protein-related
263913_at	At2g36570	0,97	1,02	0,85	1,53	1,20	0,87	0,82	0,64	leucine-rich repeat transmembrane protein kinase, putative
263922_s_at	At2g36580	0,93	0,74	0,88	0,94	1,02	1,01	1,00	0,83	pyruvate kinase, putative
263918_at	At2g36590	0,98	0,91	0,92	1,89	2,35	0,95	0,94	0,78	proline transporter, putative
265209_at	At2g36610	0,97	0,99	1,05	0,95	1,01	0,95	0,94	0,96	homeobox-leucine zipper family protein
265210_at	At2g36620	1,46	1,25	1,13	1,19	1,16	1,07	1,01	1,01	60S ribosomal protein L24 (RPL24A)
265203_at	At2g36630	1,49	1,67	1,45	1,12	0,89	1,33	1,20	1,12	expressed protein
265211_at	At2g36640	0,56	0,80	0,87	0,72	0,66	0,95	0,93	0,99	late embryogenesis abundant protein (ECP63) / LEA protein
265204_at	At2g36650	0,84	1,20	0,74	0,79	1,12	1,74	1,73	1,19	expressed protein
265205_at	At2g36660	0,95	1,09	1,04	1,06	0,89	0,98	0,98	0,95	polyadenylate-binding protein, putative / PABP, putative
265206_at	At2g36670	0,79	1,07	1,26	0,73	0,74	0,75	0,95	1,16	aspartyl protease family protein
265207_at	At2g36680	0,95	0,81	0,96	1,00	1,21	0,97	0,99	1,19	expressed protein
265208_at	At2g36690	0,70	0,99	1,02	1,00	0,76	0,59	0,85	1,08	oxidoreductase, 2OG-Fe(II) oxygenase family protein
265223_at	At2g36700	1,04	0,99	1,03	1,02	0,96	1,01	1,05	1,05	pectinesterase family protein
265224_at	At2g36710	0,96	0,98	0,96	1,10	1,00	0,97	1,04	1,09	pectinesterase family protein
265225_at	At2g36720	0,89	0,75	1,12	0,90	0,84	0,90	0,94	1,13	PHD finger transcription factor, putative
265195_at	At2g36730	1,06	1,00	1,07	0,90	0,75	1,00	1,13	1,23	pentatricopeptide (PPR) repeat-containing protein
265196_at	At2g36740	1,15	1,16	1,26	0,88	1,11	1,07	1,32	1,23	YL1 nuclear family protein
265197_at	At2g36750	0,41	0,70	0,90	0,62	0,73	1,25	1,18	1,22	UDP-glucoronosyl/UDP-glucosyl transferase family protein
265198_at	At2g36760	0,98	0,92	1,03	0,95	1,05	0,97	1,00	0,91	UDP-glucoronosyl/UDP-glucosyl transferase family protein
265199_s_at	At2g36770	0,66	0,86	0,91	0,83	0,94	0,90	1,01	1,00	UDP-glucoronosyl/UDP-glucosyl transferase family protein
265200_s_at	At2g36790	0,60	0,59	1,04	0,86	0,68	1,01	1,08	1,44	UDP-glucoronosyl/UDP-glucosyl transferase family protein
265201_at	At2g36810	1,18	1,27	1,28	0,98	0,80	1,12	1,20	1,03	expressed protein
265202_at	At2g36820	1,00	1,13	1,03	1,01	1,01	0,97	0,97	1,16	expressed protein
263867_at	At2g36830	1,59	1,67	1,51	5,99	5,19	0,87	0,80	0,78	major intrinsic family protein / MIP family protein
263842_at	At2g36835	1,24	1,29	1,29	0,90	0,89	1,48	1,34	1,35	expressed protein
263868_at	At2g36840	0,70	0,91	1,02	0,64	0,88	1,05	1,01	1,09	ACT domain-containing protein
263891_at	At2g36850	0,89	0,78	0,72	1,01	1,05	1,00	1,03	1,00	glycosyl transferase family 48 protein
263841_at	At2g36870	1,20	1,05	1,12	1,12	1,38	0,82	0,72	0,71	xyloglucan:xyloglucosyl transferase, putative / xyloglucan endotransglycosy
263838_at	At2g36880	1,03	0,98	1,03	1,68	1,24	0,98	0,94	0,92	S-adenosylmethionine synthetase, putative
263840_at	At2g36885	1,54	1,24	1,35	0,47	0,53	0,65	0,67	0,54	expressed protein
263892_at	At2g36890	1,07	1,06	1,07	1,03	1,23	0,97	0,93	0,95	myb family transcription factor (MYB38)
263839_at	At2g36900	1,20	1,39	1,27	1,08	0,99	1,03	1,04	1,42	Golgi SNARE protein membrin 11 (MEMB11) / Golgi SNAP receptor compl
263865_at	At2g36910	1,01	1,00	1,09	0,86	0,80	0,88	0,95	1,10	multidrug resistance P-glycoprotein (PGP1)
263884_at	At2g36920	0,99	1,14	0,92	1,00	0,81	1,13	0,96	0,97	expressed protein
263844_at	At2g36930	1,53	1,46	1,55	1,46	1,53	1,08	1,10	1,19	zinc finger (C2H2 type) family protein
263885_at	At2g36940	0,99	0,94	1,13	1,04	1,20	1,03	1,03	0,88	hypothetical protein
263866_at	At2g36950	1,10	1,58	1,52	0,78	0,97	1,23	1,16	1,49	heavy-metal-associated domain-containing protein
263886_at	At2g36960	1,01	1,02	1,18	0,90	0,96	1,05	1,04	1,28	myb family transcription factor
263847_at	At2g36970	0,80	0,89	0,84	0,80	0,75	1,11	0,95	0,90	UDP-glucoronosyl/UDP-glucosyl transferase family protein
263887_at	At2g36980	0,94	0,93	0,98	1,14	0,92	0,93	1,01	1,11	pentatricopeptide (PPR) repeat-containing protein
263846_at	At2g36990	0,95	1,08	1,59	0,61	0,58	1,22	1,11	1,24	RNA polymerase sigma subunit SigF (sigF) / sigma-like factor (SIG6)
263888_at	At2g37000	0,96	1,00	1,10	0,93	1,03	0,96	0,93	1,19	TCP family transcription factor, putative
263889_at	At2g37010	0,96	1,10	1,05	1,01	1,01	0,91	0,99	0,95	ABC transporter family protein

263843_at	At2g37020	1,27	1,11	1,33	1,06	1,00	1,04	0,97	0,96	translin family protein
263890_at	At2g37030	1,36	1,07	1,11	1,13	1,00	0,96	1,11	1,11	auxin-responsive family protein
263845_at	At2g37040	1,49	1,47	1,53	1,50	1,23	1,28	1,32	1,24	phenylalanine ammonia-lyase 1 (PAL1)
265467_at	At2g37050	1,09	1,04	1,22	0,92	0,99	0,94	1,00	1,09	leucine-rich repeat family protein / protein kinase family protein
265466_at	At2g37060	0,97	0,93	0,93	1,65	1,55	1,01	0,92	0,94	CCAAT-box binding transcription factor, putative
265465_at	At2g37070	1,07	0,90	0,99	1,10	1,10	0,82	0,86	1,02	expressed protein
265464_at	At2g37080	1,09	1,12	1,10	1,04	0,86	1,15	1,15	1,14	myosin heavy chain-related
265463_at	At2g37090	1,32	1,35	1,28	1,06	1,10	1,05	1,07	0,99	glycosyl transferase family 43 protein
265446_at	At2g37110	0,84	0,80	0,86	1,07	1,16	0,99	0,94	1,03	expressed protein
265416_at	At2g37120	1,14	1,16	1,05	1,65	1,51	0,78	0,85	0,90	DNA-binding S1FA family protein
265471_at	At2g37130	0,90	1,02	1,36	0,94	1,12	0,72	0,72	0,85	peroxidase 21 (PER21) (P21) (PRXR5)
265462_at	At2g37140	0,96	0,95	0,97	0,98	1,06	0,90	1,09	0,90	terpene synthase/cyclase-related
265470_at	At2g37150	1,01	0,89	0,80	0,91	1,17	1,16	1,03	0,82	zinc finger (C3HC4-type RING finger) family protein
265469_at	At2g37160	0,95	0,90	1,07	0,87	1,01	0,86	0,97	1,02	transducin family protein / WD-40 repeat family protein
265444_s_at	At2g37180	1,40	1,51	1,62	3,61	3,09	0,86	0,89	0,91	plasma membrane intrinsic protein 2B (PIP2B) / aquaporin PIP2.2 (PIP2.2)
265445_at	At2g37190	1,33	1,28	1,20	1,59	1,65	1,06	0,99	1,00	60S ribosomal protein L12 (RPL12A)
265468_at	At2g37210	1,01	1,30	1,25	0,80	0,88	1,35	1,05	0,96	expressed protein
265966_at	At2g37220	0,87	0,91	1,12	0,69	0,67	1,09	1,03	1,08	29 kDa ribonucleoprotein, chloroplast, putative / RNA-binding protein cp29,
265959_at	At2g37240	0,82	0,81	0,78	1,24	1,27	1,11	1,04	1,14	expressed protein
265958_at	At2g37250	0,96	0,85	0,74	0,84	0,82	1,05	0,91	0,98	adenylate kinase family protein
265954_at	At2g37260	1,10	1,11	1,06	1,04	1,05	0,97	0,98	1,04	WRKY family transcription factor (TTG2)
265963_s_at	At2g37270	1,24	1,15	1,09	1,29	1,24	0,94	0,93	0,98	40S ribosomal protein S5 (RPS5A)
265955_at	At2g37280	0,98	1,01	0,77	1,25	1,05	1,06	1,10	0,98	ABC transporter family protein
265956_at	At2g37290	0,96	0,90	1,03	1,00	0,90	1,01	0,99	1,08	RabGAP/TBC domain-containing protein
265957_at	At2g37300	1,01	1,06	1,02	1,07	1,03	1,10	0,96	0,95	expressed protein
266002_at	At2g37310	1,03	0,86	0,91	0,84	1,15	0,99	0,93	1,07	pentatricopeptide (PPR) repeat-containing protein
266003_at	At2g37320	0,98	1,08	0,89	0,88	0,94	0,98	0,93	0,95	pentatricopeptide (PPR) repeat-containing protein
266004_at	At2g37330	0,93	0,91	1,04	1,18	1,31	0,72	0,75	0,97	expressed protein
266005_at	At2g37340	1,24	1,30	1,23	1,05	1,05	0,97	0,97	1,02	splicing factor RSZ33 (RSZ33)
266006_at	At2g37360	0,87	1,05	0,94	1,01	0,92	0,90	0,97	0,73	ABC transporter family protein
266007_at	At2g37380	0,86	0,95	0,84	0,96	1,14	1,02	0,88	0,89	expressed protein
266008_at	At2g37390	1,06	0,97	1,04	1,32	1,17	1,03	0,84	0,99	heavy-metal-associated domain-containing protein
265961_at	At2g37400	1,13	0,86	0,92	0,99	0,95	0,90	0,65	0,67	chloroplast lumen common family protein
265968_at	At2g37410	1,22	1,23	1,29	1,20	1,18	0,96	0,91	0,92	mitochondrial import inner membrane translocase (TIM17)
266009_at	At2g37420	1,14	0,90	1,11	1,40	1,11	0,88	1,01	1,09	kinesin motor protein-related
266010_at	At2g37430	1,83	1,51	0,73	0,77	0,90	1,63	1,47	1,44	zinc finger (C2H2 type) family protein (ZAT11)
266011_at	At2g37440	0,94	1,04	1,05	0,96	0,94	1,16	1,06	1,21	endonuclease/exonuclease/phosphatase family protein
265967_at	At2g37450	1,04	1,22	1,60	0,64	0,60	1,21	1,31	1,39	nodulin MtN21 family protein
265962_at	At2g37460	1,32	1,51	1,31	1,40	1,07	1,00	1,23	1,07	nodulin MtN21 family protein
265960_at	At2g37470	0,72	0,65	0,82	0,85	1,01	0,96	0,99	1,14	histone H2B, putative
265952_at	At2g37480	0,92	0,91	0,93	0,87	0,78	0,84	0,96	0,83	expressed protein
265953_at	At2g37490	1,06	0,99	1,09	0,92	0,87	0,95	0,78	0,88	expressed protein
265965_at	At2g37500	1,05	1,01	0,86	0,88	0,87	1,00	0,93	0,93	arginine biosynthesis protein ArgJ family
265964_at	At2g37510	1,07	1,01	0,97	1,05	0,97	0,97	0,86	0,96	RNA-binding protein, putative
267163_at	At2g37520	1,03	1,22	1,42	0,99	0,71	1,05	1,12	1,07	PHD finger family protein
267169_at	At2g37540	0,65	0,53	0,72	0,74	0,73	1,32	1,20	1,53	short-chain dehydrogenase/reductase (SDR) family protein

267179_at	At2g37550	0,87	0,63	0,78	0,92	0,95	0,89	0,91	0,96	arabidopsis pde1 suppressor 1 protein (ASP1)
267173_at	At2g37560	1,23	1,15	1,05	1,16	1,08	0,98	0,98	0,88	origin recognition complex subunit 2 (ORC2)
267180_at	At2g37570	0,84	0,96	0,84	1,02	1,04	1,07	0,96	1,02	expressed protein
267177_at	At2g37580	0,55	0,75	0,83	1,19	1,23	0,76	0,87	1,11	zinc finger (C3HC4-type RING finger) family protein
267170_at	At2g37585	0,75	0,61	0,72	0,83	1,16	0,86	0,79	0,72	glycosyltransferase family 14 protein / core-2/I-branching enzyme family pro
267171_at	At2g37590	1,03	1,08	0,96	1,39	0,91	1,11	1,07	0,87	Dof-type zinc finger domain-containing protein
267174_at	At2g37600	1,38	1,11	1,06	0,88	0,77	0,97	0,97	0,81	60S ribosomal protein L36 (RPL36A)
267156_at	At2g37610	1,05	0,98	0,96	0,97	1,00	1,02	0,96	1,00	hypothetical protein
267175_s_at	At2g37620	1,07	0,92	0,82	1,30	1,09	0,81	0,82	0,80	actin 1 (ACT1)
267157_at	At2g37630	0,88	0,88	1,03	0,84	0,89	1,01	1,00	0,90	myb family transcription factor (MYB91)
267158_at	At2g37640	2,41	2,44	1,71	1,15	1,49	0,70	0,56	0,64	expansin, putative (EXP3)
267159_at	At2g37650	0,86	0,97	1,09	0,79	0,98	0,91	0,86	1,14	scarecrow-like transcription factor 9 (SCL9)
267172_at	At2g37660	0,84	0,77	0,76	0,78	0,80	1,04	1,00	1,08	expressed protein
267160_at	At2g37670	0,97	0,87	0,94	0,96	1,02	0,97	0,89	0,97	WD-40 repeat family protein
267161_at	At2g37680	1,09	1,03	1,03	0,98	1,01	0,74	0,89	0,96	phytochrome A specific signal transduction component (PAT3) / far-red elon
267162_s_at	At2g37690	1,81	1,66	1,43	0,97	0,91	1,02	0,97	1,02	phosphoribosylaminoimidazole carboxylase family protein / AIR carboxylase
267164_at	At2g37700	0,99	1,06	0,99	1,00	0,97	1,03	0,92	0,83	CER1 protein, putative
267165_at	At2g37710	0,43	0,52	0,57	0,77	0,85	0,76	0,67	0,84	lectin protein kinase, putative
267166_at	At2g37720	0,92	0,94	1,05	0,87	0,93	0,97	1,08	1,09	expressed protein
267176_at	At2g37730	1,06	0,96	1,01	0,98	1,07	0,96	0,98	0,94	fringe-related protein
267167_at	At2g37740	1,10	1,05	1,00	1,04	1,23	1,03	0,99	0,90	zinc finger (C2H2 type) family protein
267178_at	At2g37750	1,36	1,53	1,29	1,24	1,15	0,84	0,80	0,98	expressed protein
267181_at	At2g37760	0,71	0,60	0,71	1,08	1,25	0,92	0,93	0,87	aldo/keto reductase family protein
267168_at	At2g37770	0,39	0,32	0,62	1,35	1,19	0,83	1,02	1,05	aldo/keto reductase family protein
266088_at	At2g37780	0,86	1,00	0,97	0,88	1,09	0,98	0,99	0,94	DC1 domain-containing protein
266087_at	At2g37790	1,28	1,34	1,50	0,92	1,03	1,03	1,05	1,23	aldo/keto reductase family protein
266085_at	At2g37800	0,88	1,11	0,95	0,94	1,12	0,97	0,88	0,86	DC1 domain-containing protein
266084_at	At2g37810	0,92	0,94	1,00	0,99	0,90	1,03	0,85	0,97	CHP-rich zinc finger protein, putative
266083_at	At2g37820	0,90	1,05	1,10	0,95	1,21	0,88	0,91	1,02	DC1 domain-containing protein
266082_at	At2g37830	0,90	0,98	1,07	0,97	1,05	0,99	1,01	0,83	---
266081_at	At2g37840	1,16	1,00	1,01	0,89	1,05	0,91	0,99	1,13	protein kinase family protein
266080_at	At2g37850	1,19	1,13	1,12	0,77	0,96	1,19	1,12	1,05	protein kinase family protein
266079_at	At2g37860	1,34	1,43	1,23	1,20	0,95	1,08	0,97	1,00	expressed protein
266098_at	At2g37870	0,98	0,86	0,65	4,23	4,39	0,87	0,96	0,78	protease inhibitor/seed storage/lipid transfer protein (LTP) family protein
266092_at	At2g37880	0,93	1,10	1,11	1,01	0,93	1,07	1,07	1,00	expressed protein
266109_at	At2g37890	1,04	1,03	1,16	1,13	1,02	1,02	0,96	0,80	mitochondrial substrate carrier family protein
266108_at	At2g37900	1,14	1,06	1,08	0,92	1,13	0,97	0,81	0,77	proton-dependent oligopeptide transport (POT) family protein
266107_at	At2g37910	0,99	1,01	0,99	1,04	1,00	1,06	0,88	0,97	cation/hydrogen exchanger, putative (CHX21)
266091_at	At2g37920	0,80	0,80	0,95	0,76	0,92	0,78	0,86	0,84	expressed protein
266101_at	At2g37940	0,70	0,80	1,06	0,92	0,99	1,01	1,08	1,41	expressed protein
257381_at	At2g37950	1,00	1,04	1,01	0,98	1,01	0,87	1,16	1,10	zinc finger (C3HC4-type RING finger) family protein
266097_at	At2g37970	0,84	0,78	0,92	0,75	0,90	1,09	0,98	1,06	SOUL heme-binding family protein
266094_at	At2g37975	1,15	0,96	0,85	1,25	1,59	0,98	0,87	0,86	expressed protein
266100_at	At2g37980	0,99	0,99	0,86	0,88	0,94	0,98	1,08	0,86	expressed protein
266093_at	At2g37990	1,31	1,03	1,06	1,05	1,09	1,05	0,98	1,11	ribosome biogenesis regulatory protein (RRS1) family protein
266090_at	At2g38000	0,71	0,77	0,96	0,92	0,78	1,00	0,98	1,18	chaperone protein dnaJ-related

266089_at	At2g38010	0,74	0,60	0,78	0,92	1,08	1,07	1,04	1,03 ceramidase family protein
266096_at	At2g38020	1,11	0,98	0,93	0,96	0,76	0,99	0,99	0,86 vacuoleless1 (VCL1)
266099_at	At2g38040	0,78	0,70	0,76	0,89	0,91	0,97	0,98	1,14 acetyl co-enzyme A carboxylase carboxyltransferase alpha subunit family
266095_at	At2g38050	0,87	0,95	0,93	1,11	0,92	0,87	0,81	0,84 3-oxo-5-alpha-steroid 4-dehydrogenase, putative / steroid 5-alpha-reductas
266086_at	At2g38060	1,07	1,21	0,93	0,75	1,09	0,88	0,94	1,06 transporter-related
267094_at	At2g38080	1,51	1,35	1,68	1,07	0,90	1,23	1,50	1,61 laccase, putative / diphenol oxidase, putative
267141_at	At2g38090	1,05	1,05	1,16	1,02	0,98	0,84	0,90	0,92 myb family transcription factor
267143_at	At2g38100	1,01	1,10	1,05	1,01	1,02	0,98	1,06	1,13 proton-dependent oligopeptide transport (POT) family protein
267144_at	At2g38110	1,12	1,31	1,29	1,20	1,40	0,98	1,10	0,83 phospholipid/glycerol acyltransferase family protein
267092_at	At2g38120	1,09	1,18	1,38	0,92	0,80	0,87	0,93	1,16 amino acid permease, putative (AUX1)
267145_at	At2g38130	1,11	1,16	1,16	1,18	1,04	1,00	0,84	0,91 GCN5-related N-acetyltransferase, putative
267088_at	At2g38140	1,10	0,91	0,88	1,10	1,26	0,93	0,91	0,96 chloroplast 30S ribosomal protein S31 (PSRP4)
257355_at	At2g38150	1,04	1,04	0,99	0,93	1,04	1,05	0,86	0,80 alpha 1,4-glycosyltransferase family protein / glycosyltransferase sugar-bin
267146_at	At2g38160	1,05	1,12	1,04	1,22	1,36	0,86	0,86	0,84 expressed protein
267093_at	At2g38170	0,66	0,60	0,73	0,75	0,51	0,92	1,13	1,22 calcium exchanger (CAX1)
267096_at	At2g38180	0,67	0,78	1,03	1,05	1,03	0,96	0,93	1,12 GDSL-motif lipase/hydrolase family protein
267091_at	At2g38185	0,94	1,08	0,94	0,85	1,03	0,83	0,88	0,99 zinc finger (C3HC4-type RING finger) family protein
267138_s_at	At2g38210	1,54	1,26	1,45	0,90	0,94	1,25	1,06	1,06 ethylene-responsive protein, putative
267139_s_at	At2g38220	1,30	1,17	0,95	0,89	0,96	1,02	0,99	0,95 expressed protein
267147_at	At2g38240	0,79	0,72	0,75	1,60	1,18	1,62	1,44	1,04 oxidoreductase, 2OG-Fe(II) oxygenase family protein
267140_at	At2g38250	1,07	0,87	0,85	1,40	1,27	1,13	1,23	1,22 DNA-binding protein-related
267090_at	At2g38270	1,03	0,93	0,85	1,04	0,83	1,03	0,99	1,13 CAX-interacting protein, putative
267095_at	At2g38280	0,84	0,80	0,81	0,88	0,75	1,02	1,00	0,95 AMP deaminase, putative / myoadenylate deaminase, putative
267142_at	At2g38290	0,79	0,63	0,67	0,76	0,81	0,81	0,86	0,84 ammonium transporter 2 (AMT2)
267089_at	At2g38300	1,10	1,04	1,25	0,76	0,89	1,29	1,82	1,29 myb family transcription factor
267034_at	At2g38310	0,86	0,99	0,80	1,07	1,28	0,98	0,96	0,89 expressed protein
267037_at	At2g38320	1,03	0,99	1,07	0,99	0,93	0,78	1,05	1,30 expressed protein
267027_at	At2g38330	1,16	1,29	1,16	0,76	0,76	1,36	1,60	1,43 MATE efflux family protein
267026_at	At2g38340	1,21	1,12	0,98	1,03	0,95	1,14	1,12	1,19 AP2 domain-containing transcription factor, putative (DRE2B)
267025_at	At2g38350	1,00	1,06	1,02	0,90	1,16	1,08	0,96	0,91 hypothetical protein
267055_at	At2g38360	1,13	0,91	0,79	1,42	1,23	0,92	0,79	0,79 prenylated rab acceptor (PRA1) family protein
267054_at	At2g38370	0,94	0,97	1,15	0,88	1,07	0,89	0,92	0,86 expressed protein
267053_s_at	At2g38390	1,69	1,93	1,55	3,24	2,62	0,88	1,02	0,94 peroxidase 22 (PER22) (P22) (PRXEA) / basic peroxidase E
267035_at	At2g38400	1,12	0,98	1,04	0,67	0,75	1,14	1,20	1,25 alanine--glyoxylate aminotransferase, putative / beta-alanine-pyruvate amin
267052_at	At2g38410	1,22	1,31	1,59	0,93	0,95	1,17	1,15	1,19 VHS domain-containing protein / GAT domain-containing protein
267031_at	At2g38420	0,97	0,96	1,00	0,86	1,20	1,03	0,98	1,03 pentatricopeptide (PPR) repeat-containing protein
267030_at	At2g38440	0,84	0,94	1,11	0,95	1,02	0,88	0,89	1,05 expressed protein
267033_at	At2g38450	0,85	0,68	0,75	0,82	0,79	0,93	0,80	0,92 expressed protein
267029_at	At2g38460	0,84	0,87	0,89	0,84	0,84	1,00	0,91	1,27 iron transporter-related
267036_at	At2g38465	0,49	0,42	0,52	0,67	0,78	0,67	0,71	0,69 expressed protein
267028_at	At2g38470	1,19	4,31	3,60	0,58	0,61	1,88	3,38	2,92 WRKY family transcription factor
267038_at	At2g38480	1,05	0,94	1,02	1,13	1,21	0,89	0,99	0,87 integral membrane protein, putative
267032_at	At2g38490	1,07	1,13	1,02	0,93	0,92	1,07	0,84	1,12 CBL-interacting protein kinase 22, putative (CIPK22)
267051_at	At2g38500	1,07	0,96	0,85	1,07	1,12	0,96	0,86	0,79 expressed protein
266409_at	At2g38510	1,04	1,01	1,10	1,06	1,06	0,99	1,10	0,88 MATE efflux protein-related
266408_at	At2g38520	0,95	1,06	0,98	0,95	1,12	0,85	1,03	1,22 ---

266415_at	At2g38530	2,41	2,45	2,12	2,03	1,91	1,02	0,75	0,85	nonspecific lipid transfer protein 2 (LTP2)
266421_at	At2g38540	1,33	1,22	1,25	1,49	1,45	0,91	0,88	0,88	nonspecific lipid transfer protein 1 (LTP1)
266417_at	At2g38550	1,06	1,07	0,92	0,92	0,91	0,98	0,95	0,90	expressed protein
266407_at	At2g38560	1,12	1,28	1,51	0,96	0,87	1,10	1,07	1,04	transcription factor S-II (TFIIS) domain-containing protein
266406_at	At2g38570	1,04	1,05	1,11	1,04	0,89	1,18	1,10	1,19	expressed protein
266405_at	At2g38580	1,41	1,13	1,73	1,17	1,26	1,15	1,01	1,21	expressed protein
266404_at	At2g38590	1,10	1,01	0,98	1,03	0,94	1,03	0,94	1,01	F-box family protein
266403_at	At2g38600	1,12	1,00	1,01	0,99	0,93	1,01	0,92	1,01	acid phosphatase class B family protein
266420_at	At2g38610	1,09	0,86	0,96	1,10	1,13	0,97	0,88	0,88	KH domain-containing protein
266401_s_at	At2g38620	1,29	1,33	1,31	1,45	1,43	0,93	0,84	0,86	cell divsion control protein, putative
257375_at	At2g38640	0,80	1,08	1,00	1,46	1,28	0,94	0,91	1,16	expressed protein
266422_at	At2g38650	0,83	0,79	0,62	1,09	0,98	0,85	0,85	0,90	glycosyl transferase family 8 protein
266400_at	At2g38660	0,92	1,02	1,05	1,35	1,33	0,98	1,03	0,96	tetrahydrofolate dehydrogenase/cyclohydrolase, putative
266399_at	At2g38670	0,72	0,68	0,69	0,87	1,00	0,88	0,84	0,86	ethanolamine-phosphate cytidylyltransferase, putative / phosphorylethanola
266398_at	At2g38680	1,16	1,00	1,11	1,05	1,25	0,88	1,04	0,92	pyrimidine 5'-nucleotidase family protein / uridine monophosphate hydrolas
266397_at	At2g38690	0,93	1,03	1,04	0,98	0,99	1,09	0,94	0,90	hypothetical protein
266414_at	At2g38700	0,89	0,90	0,80	0,97	1,10	0,90	0,86	0,96	mevalonate diphosphate decarboxylase (MVD1)
266416_at	At2g38710	1,19	1,37	1,48	1,63	2,18	1,07	0,98	1,04	AMMECR1 family
266412_at	At2g38720	1,04	0,94	0,90	1,01	1,11	0,99	1,04	0,97	microtubule associated protein (MAP65/ASE1) family protein
266411_at	At2g38730	1,30	1,16	1,02	1,03	0,99	1,13	0,95	0,99	peptidyl-prolyl cis-trans isomerase, putative / cyclophilin, putative / rotamas
266413_at	At2g38740	1,12	0,87	0,81	1,57	1,31	1,49	1,19	1,22	haloacid dehalogenase-like hydrolase family protein
266418_at	At2g38750	0,62	0,52	0,61	0,91	0,69	0,62	0,76	0,83	annexin 4 (ANN4)
266419_at	At2g38760	0,65	0,55	0,65	1,07	0,69	0,82	1,16	1,71	annexin 3 (ANN3)
266410_at	At2g38770	1,34	1,19	1,30	0,83	0,74	0,95	0,97	1,09	expressed protein
266402_at	At2g38780	1,13	1,07	1,15	0,79	0,57	1,43	1,54	1,76	expressed protein
266396_at	At2g38790	0,63	1,08	0,93	0,85	0,54	1,09	1,18	1,12	expressed protein
263296_at	At2g38800	1,21	1,22	1,31	1,06	1,14	1,04	1,26	1,51	calmodulin-binding protein-related
263264_at	At2g38810	1,40	1,35	1,28	1,80	1,64	0,88	0,80	0,73	histone H2A, putative
263265_at	At2g38820	2,43	2,02	1,35	2,17	1,84	1,19	1,24	1,63	expressed protein
263262_at	At2g38830	0,98	1,05	1,05	0,99	1,07	1,06	1,16	0,89	tumor susceptibility protein-related
263263_at	At2g38840	1,12	1,06	1,13	1,13	1,08	1,01	0,88	0,81	guanylate-binding family protein
266190_at	At2g38850	0,96	1,03	1,22	0,93	1,16	1,07	0,97	1,15	guanylate-binding family protein
266167_at	At2g38860	1,09	1,15	1,30	1,12	1,21	1,09	1,00	1,00	proteaseI (pfpI)-like protein (YLS5)
266168_at	At2g38870	1,73	1,81	1,73	2,00	1,95	1,26	0,92	0,89	protease inhibitor, putative
266171_at	At2g38880	1,01	0,89	0,78	1,02	1,06	1,03	1,05	0,90	histone-like transcription factor (CBF/NF-Y) family protein
266198_at	At2g38890	0,96	0,90	0,89	1,02	1,11	0,96	0,96	1,12	expressed protein
266169_at	At2g38900	1,03	0,93	1,13	0,93	1,07	0,92	1,08	1,23	serine protease inhibitor, potato inhibitor I-type family protein
266143_at	At2g38905	0,45	0,59	0,55	0,68	0,61	0,96	1,07	1,06	hydrophobic protein, putative / low temperature and salt responsive protein,
266199_at	At2g38910	1,01	1,12	1,08	1,12	1,11	0,96	0,95	0,99	calcium-dependent protein kinase, putative / CDPK, putative
266200_at	At2g38920	0,91	0,95	0,95	0,96	0,88	0,92	0,96	0,97	SPX (SYG1/Pho81/XPR1) domain-containing protein / zinc finger (C3HC4-t
266183_at	At2g38930	1,00	0,97	0,93	1,18	0,86	0,99	0,91	0,89	phosphate transporter (PT2)
266184_s_at	At2g38940	0,66	0,95	0,64	1,48	0,99	1,01	0,95	0,84	phosphate transporter (PT2)
266185_at	At2g38950	1,06	1,15	1,32	1,05	0,89	1,07	0,96	1,29	transcription factor jumonji (jmj) family protein / zinc finger (C5HC2 type) far
266186_at	At2g38960	0,84	0,76	0,75	0,96	1,35	0,90	0,77	0,73	endoplasmic reticulum oxidoreductin 1 (ERO1) family protein
266187_at	At2g38970	0,81	0,95	1,28	0,80	1,03	0,89	0,88	1,03	zinc finger (C3HC4-type RING finger) family protein
266188_at	At2g39000	0,67	0,73	0,85	0,66	0,70	0,94	1,01	1,06	GCN5-related N-acetyltransferase (GNAT) family protein

266172_at	At2g39010	0,84	0,84	0,84	0,96	0,81	0,85	0,75	0,82	aquaporin, putative
266189_at	At2g39020	1,09	1,10	0,87	1,28	1,02	0,94	0,76	0,63	GCN5-related N-acetyltransferase (GNAT) family protein
266142_at	At2g39030	0,89	0,76	0,76	1,05	1,03	0,91	1,05	0,79	GCN5-related N-acetyltransferase (GNAT) family protein
266191_at	At2g39040	0,84	0,95	0,95	1,20	1,04	0,99	0,99	0,82	peroxidase, putative
266170_at	At2g39050	0,77	0,70	0,68	0,91	1,00	0,91	0,88	0,84	hydroxyproline-rich glycoprotein family protein
266201_at	At2g39060	0,92	1,15	1,21	0,99	1,04	0,98	1,04	0,99	nodulin MtN3 family protein
266192_at	At2g39070	0,95	0,75	0,77	0,86	0,92	1,00	0,99	0,94	expressed protein
266193_at	At2g39080	0,88	0,90	0,76	0,87	0,97	0,91	0,89	0,95	expressed protein
266194_at	At2g39090	1,11	1,10	1,20	0,91	1,02	1,04	1,07	1,05	tetratricopeptide repeat (TPR)-containing protein
266195_at	At2g39100	0,94	1,00	1,06	0,98	1,04	1,13	1,13	1,06	zinc finger (C3HC4-type RING finger) family protein
266196_at	At2g39110	1,17	0,91	1,26	1,53	1,56	0,97	1,25	1,13	protein kinase, putative
266197_at	At2g39120	1,02	0,78	1,02	0,95	0,80	1,02	1,28	1,20	expressed protein
267019_at	At2g39130	0,69	0,49	0,50	0,89	0,87	0,96	0,99	0,85	amino acid transporter family protein
267018_at	At2g39140	1,01	0,84	0,98	0,80	0,71	1,07	0,97	1,16	pseudouridine synthase family protein
267017_at	At2g39150	0,90	0,65	0,89	0,58	0,57	1,16	1,11	1,00	pseudouridine synthase family protein
267016_at	At2g39160	0,92	0,92	1,04	0,90	1,07	0,97	0,96	0,99	hypothetical protein
267014_at	At2g39170	0,96	0,94	0,98	0,82	0,97	1,03	0,96	1,12	expressed protein
267013_at	At2g39180	0,92	0,46	0,62	0,87	0,71	0,57	0,62	0,73	protein kinase family protein
266990_at	At2g39190	0,84	0,87	0,90	0,83	0,89	1,09	1,04	1,07	ABC1 family protein
266992_at	At2g39200	1,02	1,30	1,20	1,47	1,01	1,13	1,24	1,63	seven transmembrane MLO family protein / MLO-like protein 12 (MLO12)
266993_at	At2g39210	0,73	0,71	0,77	1,22	1,14	0,75	0,62	0,72	nodulin family protein
267012_at	At2g39220	1,00	0,93	0,96	0,88	1,10	0,80	1,12	1,15	patatin family protein
267011_at	At2g39230	1,04	1,14	1,03	0,97	0,98	0,95	0,88	1,02	pentatricopeptide (PPR) repeat-containing protein
257360_at	At2g39240	0,97	0,92	1,07	0,94	1,14	1,00	1,30	1,18	RNA polymerase I specific transcription initiation factor RRN3 family proteir
267010_at	At2g39250	0,76	1,06	1,13	0,94	1,07	1,03	1,09	1,03	AP2 domain-containing transcription factor, putative
267009_at	At2g39260	1,05	0,96	1,40	1,28	1,25	0,94	0,96	1,23	MIF4G domain-containing protein
266987_at	At2g39280	1,01	0,97	1,00	0,97	0,81	1,04	1,06	1,19	RabGAP/TBC domain-containing protein
266991_at	At2g39290	1,01	0,91	0,97	0,94	0,87	1,04	0,91	0,87	phosphatidylglycerolphosphate synthase (PGS1)
267021_at	At2g39300	0,92	1,03	0,99	1,05	1,01	1,00	0,98	1,00	expressed protein
266988_at	At2g39310	0,72	0,62	0,70	1,06	0,87	0,46	0,50	0,52	jacalin lectin family protein
267020_at	At2g39320	1,04	1,06	1,04	0,90	0,97	0,91	1,00	1,00	OTU-like cysteine protease family protein
266989_at	At2g39330	1,17	0,95	0,89	0,99	1,08	0,94	0,82	0,75	jacalin lectin family protein
267015_at	At2g39340	0,94	0,85	1,10	0,74	0,91	0,95	1,10	1,35	SAC3/GANP family protein
267008_at	At2g39350	1,30	1,34	0,97	1,29	0,96	0,98	0,86	0,91	ABC transporter family protein
266968_at	At2g39360	0,91	0,75	0,65	1,04	1,05	1,16	1,03	0,98	protein kinase family protein
266974_at	At2g39370	1,02	0,98	1,05	1,03	0,96	0,99	0,98	0,90	expressed protein
266975_at	At2g39380	1,07	1,00	0,97	1,42	1,05	1,00	0,98	1,12	exocyst subunit EXO70 family protein
266980_at	At2g39390	1,45	1,07	0,90	1,27	1,17	1,07	0,97	0,84	60S ribosomal protein L35 (RPL35B)
266983_at	At2g39400	1,20	2,07	2,21	0,60	0,55	1,15	1,29	1,49	hydrolase, alpha/beta fold family protein
266976_at	At2g39410	0,97	1,11	1,01	1,12	1,02	0,99	1,00	0,94	hydrolase, alpha/beta fold family protein
266977_at	At2g39420	0,43	0,39	0,52	1,01	0,79	0,84	1,09	1,23	esterase/lipase/thioesterase family protein
266978_at	At2g39430	1,00	1,14	0,93	1,54	1,39	1,09	0,96	0,97	disease resistance-responsive protein-related / dirigent protein-related
266962_at	At2g39440	0,88	0,95	1,07	1,05	0,95	0,89	1,12	1,04	expressed protein
266963_at	At2g39450	0,65	0,70	0,62	1,05	1,04	0,85	0,85	0,87	cation efflux family protein
266981_at	At2g39460	1,38	1,06	0,93	1,21	0,98	1,01	0,96	0,97	60S ribosomal protein L23A (RPL23aA)
266979_at	At2g39470	0,81	0,72	0,73	0,82	0,87	1,15	1,06	1,07	photosystem II reaction center PsbP family protein

266964_at	At2g39480	0,69	0,62	0,77	0,80	0,79	0,99	0,99	1,46	ABC transporter family protein
266982_at	At2g39500	1,31	1,29	1,10	1,17	1,30	1,03	1,04	0,91	expressed protein
266965_at	At2g39510	0,90	1,02	1,01	1,23	1,13	0,87	0,94	0,92	nodulin MtN21 family protein
266966_at	At2g39520	1,02	0,95	0,99	0,92	1,02	0,98	1,01	0,98	hypothetical protein
266967_at	At2g39530	1,33	1,71	1,06	2,94	1,95	1,54	1,19	1,37	integral membrane protein, putative
266969_at	At2g39540	1,00	1,03	1,11	0,86	1,17	0,93	1,09	0,79	gibberellin-regulated family protein
266985_at	At2g39550	0,80	0,76	0,82	1,18	1,04	1,06	0,93	0,99	geranylgeranyl transferase type I beta subunit (GGT-IB)
266970_at	At2g39560	1,06	0,93	1,09	0,96	1,11	0,97	1,00	0,77	expressed protein
266984_at	At2g39570	1,14	1,62	1,54	0,52	0,54	1,09	1,27	1,66	ACT domain-containing protein
266971_at	At2g39580	1,14	0,87	0,97	0,88	0,81	1,06	1,27	1,29	expressed protein
266972_at	At2g39590	1,03	1,10	0,98	0,96	1,01	1,05	0,94	0,98	40S ribosomal protein S15A (RPS15aC)
266973_at	At2g39620	0,91	1,03	1,02	0,98	1,03	1,05	0,97	0,88	pentatricopeptide (PPR) repeat-containing protein
266986_at	At2g39630	0,98	0,83	0,94	0,98	1,09	1,07	0,92	0,77	glycosyl transferase family 2 protein
267620_at	At2g39640	0,93	1,03	1,07	1,02	1,04	0,98	0,87	0,88	glycosyl hydrolase family 17 protein
267623_at	At2g39650	1,05	0,79	0,73	0,91	0,71	1,19	1,10	1,11	expressed protein
267624_at	At2g39660	0,84	1,34	1,22	0,88	1,01	1,08	1,26	1,59	protein kinase, putative
267562_at	At2g39670	1,31	1,29	1,56	0,62	0,70	1,09	1,11	1,23	radical SAM domain-containing protein
267621_at	At2g39680	0,98	0,95	1,00	1,11	1,03	0,87	0,95	0,96	expressed protein
267622_at	At2g39690	1,23	1,21	1,10	0,91	0,83	0,93	1,16	1,24	expressed protein
267590_at	At2g39700	1,76	1,78	1,31	1,54	1,57	0,92	1,04	1,13	expansin, putative (EXP4)
267591_at	At2g39705	1,00	0,92	1,14	0,80	1,07	0,87	0,92	1,03	expressed protein
267592_at	At2g39710	0,50	0,50	0,71	0,77	0,73	0,82	0,76	0,84	aspartyl protease family protein
245059_at	At2g39720	0,83	0,78	0,87	1,05	1,09	0,92	0,82	0,99	zinc finger (C3HC4-type RING finger) family protein
245064_at	At2g39725	1,15	1,15	1,04	1,12	1,23	1,02	0,95	0,99	complex 1 family protein / LVR family protein
245061_at	At2g39730	1,18	1,08	1,11	1,15	1,09	1,12	1,04	1,07	ribulose bisphosphate carboxylase/oxygenase activase / RuBisCO activase
245065_at	At2g39750	0,72	0,73	0,75	0,91	1,01	0,94	0,87	0,81	dehydration-responsive family protein
245062_at	At2g39760	1,19	1,12	1,35	0,92	1,03	1,04	1,05	1,01	speckle-type POZ protein-related
245060_at	At2g39770	0,93	0,78	0,92	0,93	1,09	0,84	0,84	0,95	GDP-mannose pyrophosphorylase (GMP1)
245067_at	At2g39780	0,80	0,72	0,66	1,08	1,26	1,09	0,93	0,93	ribonuclease 2 (RNS2)
245058_at	At2g39790	0,96	1,05	1,10	0,90	0,94	0,98	1,04	0,92	mitochondrial glycoprotein family protein / MAM33 family protein
245063_at	At2g39795	1,58	1,27	1,34	1,38	1,38	0,90	0,81	0,69	mitochondrial glycoprotein family protein / MAM33 family protein
245120_at	At2g39810	0,97	0,99	1,03	0,87	1,00	1,11	0,97	1,27	expressed protein
245086_at	At2g39820	1,27	1,11	1,18	1,00	1,15	0,91	0,93	1,05	eukaryotic translation initiation factor 6, putative / eIF-6, putative
245087_at	At2g39830	1,13	1,58	1,36	1,19	1,17	0,94	0,90	0,96	LIM domain-containing protein
245066_at	At2g39840	0,92	0,89	0,91	1,07	1,10	1,09	0,94	1,00	serine/threonine protein phosphatase PP1 isozyme 4 (TOPP4) / phosphopr
245088_at	At2g39850	1,07	0,94	0,83	0,84	0,65	0,84	0,79	0,82	subtilase family protein
267339_at	At2g39870	1,12	1,23	1,26	0,95	0,78	0,80	1,00	1,09	expressed protein
267354_at	At2g39880	0,93	1,03	1,11	0,91	1,06	0,92	1,04	1,05	myb family transcription factor (MYB25)
267358_at	At2g39890	0,66	0,59	0,60	0,95	0,85	0,85	0,76	0,92	proline transporter 1 (ProT1)
267355_at	At2g39900	0,78	0,75	0,83	1,05	1,09	0,79	0,77	0,77	LIM domain-containing protein
267362_at	At2g39910	0,94	0,98	0,94	0,95	1,03	0,97	1,03	0,98	expressed protein
267361_at	At2g39920	0,92	1,22	1,66	0,58	0,75	1,09	1,01	0,85	acid phosphatase class B family protein
267356_at	At2g39930	0,84	0,88	0,81	0,89	0,82	0,98	0,91	1,04	isoamylase, putative / starch debranching enzyme, putative
267346_at	At2g39940	1,09	1,30	1,28	0,98	0,80	0,93	1,01	1,14	coronatine-insensitive 1 / COI1 (FBL2)
267347_at	At2g39950	0,99	1,00	1,12	0,70	0,65	1,06	1,07	1,37	expressed protein
267348_at	At2g39960	1,03	1,03	1,12	1,12	1,24	1,17	0,96	1,00	microsomal signal peptidase 25 kDa subunit, putative (SPC25)

267363_at	At2g39970	0,90	0,80	0,72	0,89	1,01	0,86	0,88	0,88 peroxisomal membrane protein (PMP36)
267337_at	At2g39980	0,73	0,81	1,07	0,84	1,03	0,70	0,94	1,00 transferase family protein
267338_at	At2g39990	1,21	0,99	1,05	1,11	1,02	0,88	0,85	0,81 eukaryotic translation initiation factor 3 subunit 5 / eIF-3 epsilon / eIF3f (TIF
267357_at	At2g40000	1,05	1,19	1,27	0,89	0,60	1,07	1,79	2,02 expressed protein
267349_at	At2g40010	1,11	1,00	1,16	0,84	0,70	1,00	0,92	1,00 60S acidic ribosomal protein P0 (RPP0A)
267359_at	At2g40020	1,01	1,01	0,91	0,90	0,91	1,13	1,33	1,19 expressed protein
267350_at	At2g40030	0,90	0,91	0,83	0,99	0,93	0,92	0,93	0,81 DNA-directed RNA polymerase alpha subunit family protein
267351_at	At2g40040	0,74	0,87	1,04	0,93	0,99	0,92	0,71	0,86 defective chloroplasts and leaves protein-related / DCL protein-related
267352_at	At2g40050	0,96	0,96	0,99	1,06	1,18	0,83	1,06	0,97 DC1 domain-containing protein
267360_at	At2g40060	1,06	0,88	0,85	1,12	1,25	0,98	0,91	1,01 expressed protein
267353_at	At2g40070	0,95	0,91	0,94	0,91	0,97	1,03	0,81	1,08 expressed protein
267364_at	At2g40080	0,66	0,70	0,61	0,76	0,74	1,02	1,19	0,95 expressed protein
265721_at	At2g40090	1,15	0,96	0,92	0,89	0,75	1,02	0,92	0,90 ABC1 family protein
265722_at	At2g40100	1,92	1,81	1,45	1,23	0,90	0,80	0,78	0,66 chlorophyll A-B binding protein (LHCB4.3)
265720_at	At2g40110	0,93	0,74	0,75	0,81	1,03	0,72	1,00	1,26 yippee family protein
263383_at	At2g40120	1,06	0,98	0,83	0,96	0,88	0,97	0,73	1,18 protein kinase family protein
263384_at	At2g40130	0,85	1,05	1,06	0,77	1,08	1,02	0,99	1,14 heat shock protein-related
263379_at	At2g40140	1,67	2,41	1,56	0,74	0,75	1,49	2,01	1,67 zinc finger (CCCH-type) family protein
263386_at	At2g40150	0,95	1,13	0,96	0,94	0,94	0,87	1,00	0,92 expressed protein
263387_at	At2g40160	0,93	0,92	0,94	1,09	1,21	1,00	1,09	0,99 expressed protein
263385_at	At2g40170	0,43	0,67	0,66	0,65	0,62	1,12	1,07	1,07 Em-like protein GEA6 (EM6)
263378_at	At2g40180	0,94	1,13	0,90	0,99	1,00	0,84	1,63	1,45 protein phosphatase 2C, putative / PP2C, putative
263380_at	At2g40200	1,05	1,07	0,96	1,00	1,14	1,04	1,04	1,01 basic helix-loop-helix (bHLH) family protein
263381_at	At2g40210	0,90	1,03	0,98	0,96	1,09	0,97	0,95	1,07 MADS-box family protein
263377_at	At2g40220	0,86	1,09	1,09	1,17	1,03	0,98	1,10	0,99 abscisic acid-insensitive 4 (ABI4)
263382_at	At2g40230	0,83	0,97	0,94	1,17	1,37	0,95	0,88	1,26 transferase family protein
263822_at	At2g40240	1,13	1,06	1,02	1,00	1,02	1,03	0,90	0,86 pentatricopeptide (PPR) repeat-containing protein
263828_at	At2g40250	1,02	1,11	0,88	1,02	1,17	1,33	0,96	0,92 GDSL-motif lipase/hydrolase family protein
263830_at	At2g40260	0,92	0,97	1,01	1,03	0,93	1,20	1,19	1,32 myb family transcription factor
263804_at	At2g40270	0,85	1,43	1,26	0,79	0,79	1,12	1,36	1,31 protein kinase family protein
263774_at	At2g40280	0,96	0,86	0,89	1,05	1,22	1,02	0,95	0,84 dehydration-responsive family protein
263835_at	At2g40290	1,13	1,07	1,05	1,03	1,06	1,12	1,05	1,05 eukaryotic translation initiation factor 2 subunit 1, putative / eIF-2A, putative
263831_at	At2g40300	0,97	0,90	1,15	0,60	0,75	0,53	0,65	0,56 ferritin, putative
263832_at	At2g40310	0,94	1,02	1,02	1,04	1,00	0,95	1,05	1,06 glycoside hydrolase family 28 protein / polygalacturonase (pectinase) family
263833_at	At2g40313	1,09	0,99	0,95	1,24	0,88	0,99	0,91	0,97 expressed protein
263834_at	At2g40316	1,02	0,94	1,07	0,78	0,99	0,92	0,86	0,86 expressed protein
263836_at	At2g40330	1,18	0,89	0,91	1,02	1,10	0,65	0,82	0,80 Bet v I allergen family protein
263823_s_at	At2g40350	0,92	0,99	1,01	0,44	0,61	1,16	1,30	1,15 AP2 domain-containing transcription factor, putative (DRE2B)
263824_at	At2g40360	1,23	1,12	1,14	0,96	0,88	0,98	0,98	1,08 transducin family protein / WD-40 repeat family protein
263825_at	At2g40370	0,90	1,13	1,09	0,98	1,04	1,16	1,09	1,16 laccase, putative / diphenol oxidase, putative
263805_at	At2g40400	1,33	1,18	1,01	0,73	0,54	1,25	1,18	0,91 expressed protein
263826_at	At2g40410	0,89	1,00	1,39	1,06	1,15	1,04	0,97	0,91 Ca(2+)-dependent nuclease, putative
263827_at	At2g40420	1,17	1,26	1,45	0,91	0,88	1,05	0,96	1,15 amino acid transporter family protein
263802_at	At2g40430	1,12	1,01	1,12	0,92	0,99	1,17	1,09	1,22 expressed protein
263829_at	At2g40435	0,96	0,89	0,96	1,15	1,06	0,97	1,04	0,92 expressed protein
263803_at	At2g40440	0,96	0,94	0,98	1,06	1,26	0,98	1,00	0,97 BTB/POZ domain-containing protein

255877_at	At2g40460	0,82	0,70	0,87	0,76	0,66	1,08	1,50	1,45	proton-dependent oligopeptide transport (POT) family protein
255823_at	At2g40470	0,93	0,96	1,03	0,92	0,97	0,92	1,06	1,12	LOB domain protein 15 / lateral organ boundaries domain protein 15 (LBD1
255825_at	At2g40475	1,12	1,00	1,21	0,91	0,98	1,02	0,91	0,95	expressed protein
255876_at	At2g40480	0,83	0,88	0,92	1,20	1,01	0,92	0,71	0,75	expressed protein
255826_at	At2g40490	0,98	0,81	0,92	0,93	0,87	1,08	0,92	0,86	uroporphyrinogen decarboxylase, putative / UPD, putative
255875_s_at	At2g40500	0,98	0,89	1,07	0,86	1,06	0,93	0,99	1,08	protein kinase family protein
255824_at	At2g40530	0,92	0,97	1,01	1,07	1,19	0,94	1,01	0,98	expressed protein
255829_at	At2g40540	0,98	1,13	1,38	0,81	0,88	1,19	1,18	1,51	potassium transporter, putative (KT2)
255874_at	At2g40550	1,66	1,55	1,39	1,20	1,09	1,37	0,88	1,04	expressed protein
255821_at	At2g40570	0,94	0,93	0,81	0,73	0,68	1,37	1,03	1,47	initiator tRNA phosphoribosyl transferase family protein
255820_at	At2g40580	0,97	1,07	1,02	1,02	0,99	0,94	0,97	1,36	protein kinase family protein
255819_s_at	At2g40590	1,51	1,13	1,10	1,10	0,99	1,10	0,97	0,96	40S ribosomal protein S26 (RPS26A)
255827_at	At2g40600	0,91	0,79	0,82	1,04	0,91	1,02	0,98	1,09	appr-1-p processing enzyme family protein
255822_at	At2g40610	2,43	2,77	1,44	0,69	0,53	1,90	1,85	1,72	expansin, putative (EXP8)
255878_at	At2g40620	1,00	0,78	0,96	1,17	1,24	1,01	1,01	1,26	bZIP transcription factor family protein
255828_at	At2g40630	1,07	0,92	1,09	1,07	0,96	0,96	1,00	1,10	expressed protein
266054_at	At2g40640	0,99	1,08	1,16	0,77	1,06	0,91	0,86	1,19	expressed protein
266055_at	At2g40650	1,09	1,01	1,11	0,94	1,16	1,02	1,22	1,20	pre-mRNA splicing factor PRP38 family protein
266057_at	At2g40660	1,23	1,10	1,02	1,13	1,06	1,02	1,07	1,09	tRNA-binding region domain-containing protein
266078_at	At2g40670	0,98	0,99	0,97	0,96	1,05	1,09	1,12	1,15	two-component responsive regulator / response regulator 16 (ARR16)
266077_at	At2g40680	0,93	1,07	1,11	0,82	1,08	1,06	1,01	0,92	hypothetical protein
266058_at	At2g40690	0,99	1,15	0,91	0,77	0,93	1,08	1,08	1,00	NAD-dependent glycerol-3-phosphate dehydrogenase family protein
266076_at	At2g40700	1,04	1,00	1,30	0,91	0,96	1,01	1,23	1,37	DEAD/DEAH box helicase, putative (RH17)
266075_s_at	At2g40710	0,94	0,87	1,01	1,01	0,88	1,18	1,26	1,31	expressed protein
266102_at	At2g40720	1,03	0,98	1,13	0,75	0,89	1,11	1,01	0,92	pentatricopeptide (PPR) repeat-containing protein
266053_at	At2g40730	0,94	0,93	0,86	0,96	1,09	0,91	0,94	0,92	HEAT repeat-containing protein
266052_at	At2g40740	1,15	1,05	1,16	1,04	1,14	1,08	0,92	1,02	WRKY family transcription factor
257382_at	At2g40750	0,55	0,69	1,15	1,34	0,98	1,39	1,17	1,25	WRKY family transcription factor
266051_at	At2g40760	0,93	1,12	0,98	0,74	0,74	1,00	0,91	0,94	rhodanese-like domain-containing protein
266059_at	At2g40765	1,25	0,99	0,90	1,11	1,37	0,94	0,96	1,00	expressed protein
266050_at	At2g40770	0,89	0,92	1,01	0,84	0,94	0,91	1,02	1,18	SNF2 domain-containing protein / helicase domain-containing protein / zinc
266049_at	At2g40780	1,03	0,95	1,16	1,11	1,14	0,94	0,96	1,01	hypothetical protein
266048_at	At2g40790	0,97	0,91	0,99	1,06	0,96	0,94	0,99	1,10	thioredoxin family protein
266047_at	At2g40800	1,22	0,95	0,56	1,23	1,08	1,02	0,84	0,91	expressed protein
266056_at	At2g40810	0,99	1,04	0,94	0,80	0,92	1,23	1,06	1,18	WD-40 repeat protein family
245093_at	At2g40820	1,07	1,19	1,40	0,90	1,20	0,79	0,95	0,78	proline-rich family protein
245099_at	At2g40830	0,89	1,15	1,23	0,98	1,14	0,87	0,85	1,09	zinc finger (C3HC4-type RING finger) family protein
245094_at	At2g40840	0,55	0,47	0,57	0,53	0,56	0,89	0,91	0,91	glycoside hydrolase family 77 protein
245095_at	At2g40870	0,86	0,87	1,02	0,81	0,83	1,05	0,92	0,95	protein kinase family protein / protein phosphatase 2C (PP2C) family prote
245096_at	At2g40880	1,07	0,96	0,96	1,19	1,31	1,10	1,00	0,96	cysteine protease inhibitor, putative / cystatin, putative (FL3-27)
245101_at	At2g40890	1,33	1,32	1,57	1,38	1,34	1,27	1,20	1,06	cytochrome P450 98A3, putative (CYP98A3)
245090_at	At2g40900	1,44	1,39	1,23	0,92	1,01	0,80	0,91	0,91	nodulin MtN21 family protein
245091_at	At2g40910	0,92	0,95	0,89	1,04	1,04	0,96	0,92	1,03	F-box protein-related
245100_at	At2g40930	1,20	1,43	1,91	0,87	0,60	1,16	1,35	1,59	ubiquitin-specific protease 5, putative (UBP5)
245097_at	At2g40935	0,93	0,87	1,12	0,87	1,00	1,07	1,04	0,86	expressed protein
245098_at	At2g40940	1,00	0,95	1,08	1,21	1,33	0,90	0,86	0,91	ethylene response sensor / ethylene-responsive sensor (ERS)

245092_at	At2g40950	0,90	0,93	0,95	1,07	1,06	1,11	0,94	0,86 bZIP transcription factor family protein
267074_s_at	At2g40955	0,99	0,99	1,03	1,01	1,13	0,99	0,94	1,10 hypothetical protein
267078_at	At2g40960	1,05	1,19	1,24	0,59	0,53	1,37	1,31	1,44 expressed protein
267077_at	At2g40970	1,09	1,27	1,32	1,48	1,38	0,90	0,91	1,10 myb family transcription factor
267071_at	At2g40980	0,96	0,91	1,08	1,01	1,09	0,99	0,84	1,01 expressed protein
257358_at	At2g40990	1,04	1,06	0,97	1,04	1,03	1,23	0,92	1,00 zinc finger (DHHC type) family protein
267070_at	At2g41000	1,06	0,96	1,15	0,95	1,16	1,12	1,12	1,00 DNAJ heat shock N-terminal domain-containing protein
267069_at	At2g41010	0,87	0,90	0,94	0,96	0,97	0,97	0,88	1,13 VQ motif-containing protein
267068_at	At2g41020	0,85	1,08	1,55	0,94	0,96	0,91	1,13	0,96 WW domain-containing protein
267067_at	At2g41030	1,01	0,98	1,13	0,96	1,08	1,03	0,93	1,34 WW domain-containing protein
267066_at	At2g41040	1,14	1,04	0,99	0,99	0,76	1,31	1,20	0,95 methyltransferase-related
257357_at	At2g41050	1,07	1,10	1,10	0,84	1,04	1,06	0,97	1,01 PQ-loop repeat family protein / transmembrane family protein
267050_at	At2g41060	0,93	1,02	1,01	1,08	1,05	1,12	1,07	1,11 RNA recognition motif (RRM)-containing protein
267075_at	At2g41070	0,82	0,70	0,88	1,02	1,04	1,07	0,94	1,00 basic leucine zipper transcription factor (BZIP12)
267065_at	At2g41080	0,83	0,96	1,03	0,53	0,66	0,81	1,00	1,03 pentatricopeptide (PPR) repeat-containing protein
267076_at	At2g41090	0,60	0,61	0,78	1,05	0,92	1,04	0,80	0,89 calmodulin-like calcium-binding protein, 22 kDa (CaBP-22)
267083_at	At2g41100	1,09	1,05	1,38	0,98	1,04	1,02	1,11	1,14 touch-responsive protein / calmodulin-related protein 3, touch-induced (TCH
267064_at	At2g41110	0,92	0,70	0,66	1,04	1,09	0,82	0,90	0,82 calmodulin-2/3/5 (CAM2) (CAL1)
267063_at	At2g41120	0,76	0,70	0,71	0,99	1,25	1,17	1,11	0,90 expressed protein
267082_at	At2g41140	1,14	1,76	1,74	1,08	1,27	1,04	1,19	1,39 calcium-dependent protein kinase, putative / CDPK, putative
267073_at	At2g41160	0,97	1,08	1,25	1,27	1,24	1,09	1,12	1,08 ubiquitin-associated (UBA)/TS-N domain-containing protein
267072_at	At2g41170	0,99	0,98	1,22	1,26	1,01	0,85	0,99	1,17 F-box family protein
267084_at	At2g41180	1,07	1,11	0,91	0,80	1,00	0,86	0,83	1,02 sigA-binding protein-related
267080_at	At2g41190	0,84	0,89	0,95	0,84	0,99	0,94	0,97	0,71 amino acid transporter family protein
267079_at	At2g41200	0,92	0,94	0,86	1,11	1,07	0,93	0,90	0,68 expressed protein
267081_at	At2g41210	0,65	0,48	0,48	0,97	0,86	1,03	0,91	0,87 phosphatidylinositol-4-phosphate 5-kinase family protein
266365_at	At2g41220	1,04	1,14	0,97	1,07	1,09	1,18	0,98	1,08 glutamate synthase, chloroplast (GLU2) / ferredoxin-dependent glutamate s
266364_at	At2g41230	1,13	1,66	1,72	1,31	1,43	0,94	0,67	0,68 expressed protein
266363_at	At2g41250	1,89	1,87	1,41	1,19	1,04	1,92	1,39	1,51 haloacid dehalogenase-like hydrolase family protein
266393_at	At2g41260	0,03	0,02	0,01	1,45	1,32	1,11	1,07	0,89 glycine-rich protein / late embryogenesis abundant protein (M17)
266392_at	At2g41280	0,91	0,97	0,78	1,02	0,94	1,38	1,27	1,10 late embryogenesis abundant protein (M10) / LEA protein M10
266391_at	At2g41290	1,51	1,31	1,29	1,01	1,00	1,20	1,18	1,41 strictosidine synthase family protein
266425_at	At2g41300	1,18	1,12	0,91	1,01	1,07	0,97	1,02	1,16 strictosidine synthase family protein
266372_at	At2g41310	0,99	0,97	0,90	0,96	0,99	1,07	1,03	0,95 two-component responsive regulator / response reactor 3 (RR3)
266424_at	At2g41330	0,81	0,81	0,94	0,84	1,34	0,84	0,92	1,01 glutaredoxin family protein
266423_at	At2g41340	1,12	0,73	1,19	0,68	0,92	0,71	0,75	0,77 eukaryotic rpb5 RNA polymerase subunit family protein
266370_at	At2g41350	1,27	1,16	1,34	0,94	1,08	1,18	1,03	1,10 expressed protein
266369_at	At2g41370	1,02	0,89	0,94	1,26	1,13	1,04	0,93	0,94 ankyrin repeat family protein / BTB/POZ domain-containing protein
266368_at	At2g41380	0,61	0,60	0,94	0,96	1,06	0,81	0,82	0,94 embryo-abundant protein-related
266367_s_at	At2g41390	1,02	1,05	0,98	0,92	1,10	1,00	0,97	0,99 expressed protein
266371_at	At2g41410	0,96	0,97	0,93	1,36	1,72	0,87	0,81	0,84 calmodulin, putative
266366_at	At2g41420	0,86	0,68	0,72	0,96	0,95	0,95	0,86	0,96 proline-rich family protein
267104_at	At2g41430	0,96	0,91	1,01	0,91	1,10	0,99	0,98	1,02 dehydration-induced protein (ERD15)
267097_at	At2g41440	1,00	0,96	1,02	0,97	1,10	1,00	1,07	1,08 expressed protein
267098_at	At2g41450	0,96	1,00	1,06	0,98	1,16	1,12	1,04	0,80 GCN5-related N-acetyltransferase (GNAT) family protein
267099_at	At2g41460	1,09	0,98	1,10	1,21	1,04	1,09	0,98	1,37 apurinic endonuclease-redox protein / DNA-(apurinic or apyrimidinic site) ly

267100_at	At2g41470	0,91	1,01	1,16	0,93	1,12	1,09	1,06	0,88	embryo-specific protein-related
267101_at	At2g41480	0,82	0,78	1,11	1,18	1,30	1,05	1,12	1,26	peroxidase, putative
267103_at	At2g41490	0,83	0,72	0,69	1,11	1,13	0,97	0,89	0,86	UDP-GlcNAc:dolichol phosphate N-acetylglucosamine-1-phosphate transfe
267102_at	At2g41500	1,38	1,30	1,41	0,87	0,96	1,12	1,06	1,26	WD-40 repeat family protein / small nuclear ribonucleoprotein Prp4p-relatec
245108_at	At2g41510	1,06	1,09	0,95	1,27	1,09	1,06	1,02	0,94	FAD-binding domain-containing protein / cytokinin oxidase family protein
245109_at	At2g41520	0,98	1,00	1,09	0,82	1,06	1,21	0,92	1,14	DNAJ heat shock N-terminal domain-containing protein
245115_at	At2g41530	1,11	1,08	1,22	1,17	1,05	1,00	0,98	0,95	esterase, putative
245112_at	At2g41540	1,09	1,07	1,07	0,94	0,95	0,91	0,91	0,92	NAD-dependent glycerol-3-phosphate dehydrogenase family protein
245110_at	At2g41550	1,17	1,36	1,17	1,10	1,05	1,02	0,91	1,00	expressed protein
245117_at	At2g41560	0,80	0,75	0,67	0,75	0,67	0,98	0,92	0,91	calcium-transporting ATPase 4, plasma membrane-type / Ca2+-ATPase, is
245111_at	At2g41570	1,04	1,05	1,10	0,97	1,02	0,96	1,06	0,95	hypothetical protein
245102_at	At2g41580	1,10	1,05	1,03	1,02	1,02	0,95	1,05	1,06	---
245103_at	At2g41590	1,12	0,96	1,08	1,03	1,07	0,96	0,99	0,96	expressed protein
245104_at	At2g41600	1,06	1,05	1,19	0,85	0,95	1,06	0,89	0,82	expressed protein
245105_at	At2g41610	1,01	1,01	1,12	1,15	0,87	0,98	1,00	0,77	expressed protein
245116_at	At2g41620	0,98	0,96	0,95	1,05	0,96	1,02	0,84	0,83	nucleoporin interacting component family protein
245114_at	At2g41630	0,94	0,64	0,80	0,74	0,83	1,07	1,07	1,15	transcription initiation factor IIB-1 / general transcription factor TFIIB-1 (TFII
245119_at	At2g41640	1,07	1,58	1,44	0,84	0,71	1,38	2,00	1,90	expressed protein
245106_at	At2g41650	1,68	1,27	1,52	1,32	1,37	0,99	1,01	0,81	expressed protein
245113_at	At2g41660	0,80	0,98	1,02	1,11	1,17	0,95	1,02	1,16	expressed protein
245118_at	At2g41680	1,03	0,93	0,85	0,87	0,87	1,29	1,24	1,33	thioredoxin reductase, putative / NADPH-dependent thioredoxin reductase,
245107_at	At2g41690	1,00	1,06	0,99	0,96	0,95	0,87	1,02	1,07	heat shock transcription factor family protein
260496_at	At2g41700	0,81	0,76	0,81	0,82	0,80	0,95	0,99	1,06	ABC transporter family protein
260500_at	At2g41705	0,94	1,26	1,82	0,78	0,68	1,14	1,15	1,24	camphor resistance CrcB family protein
260498_at	At2g41710	0,81	1,16	1,33	0,93	1,11	1,10	0,99	0,95	ovule development protein, putative
260523_at	At2g41720	1,01	0,86	0,95	0,66	0,50	1,07	0,91	1,00	pentatricopeptide (PPR) repeat-containing protein
260522_x_at	At2g41730	1,09	0,86	1,36	0,92	1,39	1,60	1,13	1,39	expressed protein
260521_at	At2g41740	0,96	0,77	0,83	0,96	1,14	0,88	0,84	0,90	villin 2 (VLN2)
260499_at	At2g41760	0,94	1,01	0,96	0,88	1,02	1,06	1,02	0,99	expressed protein
260501_at	At2g41770	0,82	0,84	0,88	0,96	1,29	0,86	0,76	0,73	expressed protein
260555_at	At2g41780	1,25	1,18	1,10	1,25	1,56	0,95	0,98	1,01	expressed protein
260554_at	At2g41790	0,88	0,86	0,88	1,03	0,81	1,01	0,99	1,08	peptidase M16 family protein / insulinase family protein
260553_at	At2g41800	2,10	2,06	1,80	2,55	2,70	1,09	1,26	1,27	expressed protein
260495_at	At2g41810	1,00	0,97	0,91	1,01	1,09	1,04	1,14	0,95	expressed protein
260494_at	At2g41820	1,20	1,34	1,31	0,67	1,02	0,92	1,18	0,94	leucine-rich repeat transmembrane protein kinase, putative
260493_at	At2g41830	0,69	0,64	0,62	1,23	1,00	1,04	0,91	0,92	cyclin-related
260497_at	At2g41840	1,11	1,04	0,91	1,04	1,11	0,91	0,86	0,87	40S ribosomal protein S2 (RPS2C)
260492_at	At2g41850	1,22	1,20	1,30	1,13	1,34	1,09	1,14	1,23	endo-polygalacturonase, putative
267531_at	At2g41860	0,93	0,99	0,98	0,93	1,09	0,94	1,03	1,02	calcium-dependent protein kinase, putative / CDPK, putative
267538_at	At2g41870	1,15	1,24	1,43	1,37	1,76	0,87	1,00	1,21	remorin family protein
267537_at	At2g41880	0,64	0,63	0,61	1,16	1,11	0,77	0,78	0,84	guanylate kinase 1 (GK-1)
267530_at	At2g41890	0,97	0,84	0,64	1,18	0,97	0,97	1,05	1,12	curculin-like (mannose-binding) lectin family protein / PAN domain-containir
267534_at	At2g41900	0,91	0,86	0,91	0,81	1,11	1,09	0,93	0,83	zinc finger (CCCH-type) family protein
267584_at	At2g41930	1,06	0,96	1,05	1,07	1,10	1,06	0,98	1,17	protein kinase family protein
267535_at	At2g41940	0,95	0,76	0,96	0,84	1,01	0,87	0,82	1,01	zinc finger (C2H2 type) family protein
267586_at	At2g41950	1,22	1,07	1,28	0,91	0,87	0,92	0,85	0,83	expressed protein

267583_at	At2g41960	0,92	0,95	1,29	1,15	1,09	1,05	0,97	1,33 expressed protein
267582_at	At2g41970	1,08	0,94	1,08	0,84	1,08	1,10	1,02	0,95 protein kinase, putative
267581_at	At2g41980	0,94	0,67	0,65	0,89	0,87	1,09	0,79	0,69 seven in absentia (SINA) family protein
267580_at	At2g41990	1,03	0,82	1,03	0,95	0,92	0,93	0,75	0,86 expressed protein
267579_at	At2g42000	0,96	0,91	0,99	0,99	1,04	1,06	1,04	1,27 plant EC metallothionein-like family 15 protein
267536_at	At2g42010	0,81	0,64	0,82	1,10	1,07	0,98	1,06	1,21 phospholipase D beta 1 / PLD beta 1 (PLDBETA1)
267539_at	At2g42030	0,80	0,78	0,83	0,92	0,91	1,02	0,92	0,97 zinc finger (C3HC4-type RING finger) family protein
267532_at	At2g42040	1,00	0,87	1,10	1,13	1,06	0,72	0,77	0,78 expressed protein
267589_at	At2g42050	1,05	1,00	1,01	0,97	0,94	0,98	0,96	1,01 hypothetical protein
267588_at	At2g42060	1,19	1,29	0,91	0,95	0,83	1,05	0,90	0,92 CHP-rich zinc finger protein, putative
267533_at	At2g42070	0,89	0,91	0,91	0,90	0,92	0,97	0,77	0,65 MutT/nudix family protein
267587_at	At2g42080	0,92	0,78	0,88	1,01	1,33	0,91	0,88	0,67 DNAJ heat shock N-terminal domain-containing protein
267585_s_at	At2g42090	0,98	0,98	1,10	0,99	0,99	0,97	1,00	1,11 actin, putative
267634_at	At2g42100	0,99	1,07	0,88	1,01	1,26	0,91	1,10	1,01 actin, putative
267636_at	At2g42110	1,09	0,98	0,96	1,16	1,26	0,77	0,94	1,03 expressed protein
267629_at	At2g42120	1,06	1,08	1,21	1,01	1,09	0,96	0,93	0,93 DNA polymerase delta small subunit-related
267630_at	At2g42130	0,80	0,77	0,78	0,85	1,02	1,00	0,94	0,95 expressed protein
257348_at	At2g42140	1,22	1,11	1,02	1,11	1,79	1,01	1,03	1,03 VQ motif-containing protein
267631_at	At2g42150	1,19	0,97	1,07	1,11	0,92	1,11	0,95	0,91 DNA-binding bromodomain-containing protein
267632_at	At2g42160	1,03	0,94	1,15	0,92	0,93	1,27	1,13	1,23 zinc finger (ubiquitin-hydrolase) domain-containing protein
267633_at	At2g42180	0,96	1,03	1,14	0,94	0,98	1,01	0,99	1,05 expressed protein
267637_at	At2g42190	0,97	0,94	0,98	0,91	0,94	0,98	1,00	0,99 expressed protein
267639_at	At2g42200	1,12	1,07	1,07	0,97	1,06	0,89	1,10	0,96 squamosa promoter-binding protein-like 9 (SPL9)
267638_at	At2g42210	1,13	1,03	0,92	1,15	1,08	0,91	0,95	0,89 mitochondrial import inner membrane translocase subunit Tim17/Tim22/Tim
267635_at	At2g42220	0,93	0,88	0,93	0,88	0,96	1,05	0,99	0,98 rhodanese-like domain-containing protein
267625_at	At2g42240	0,93	0,92	1,01	1,04	1,07	0,86	0,89	0,89 RNA recognition motif (RRM)-containing protein
267626_at	At2g42250	0,97	0,95	1,06	0,96	0,94	1,08	1,05	0,88 cytochrome P450 family protein
267627_at	At2g42270	3,33	2,24	2,72	1,10	0,90	2,28	2,26	1,95 U5 small nuclear ribonucleoprotein helicase, putative
267628_at	At2g42280	2,65	1,62	1,08	0,96	1,22	1,82	1,85	1,21 basic helix-loop-helix (bHLH) family protein
265876_at	At2g42290	0,78	0,95	0,71	1,04	1,01	0,90	0,99	0,98 leucine-rich repeat family protein
265880_at	At2g42300	1,47	1,63	1,48	0,81	0,87	1,15	1,25	1,02 basic helix-loop-helix (bHLH) family protein
265883_at	At2g42310	1,47	1,34	1,33	1,20	1,26	0,92	0,88	1,00 expressed protein
265884_at	At2g42320	1,10	1,16	1,56	1,04	1,32	1,05	0,89	1,05 nucleolar protein gar2-related
265885_at	At2g42330	2,43	1,70	1,68	0,83	0,82	1,52	1,57	1,51 D111/G-patch domain-containing protein
265851_at	At2g42340	0,96	0,98	1,10	0,99	1,00	1,06	1,00	0,95 expressed protein
265852_at	At2g42350	0,90	1,18	0,93	1,14	1,44	0,88	0,81	0,88 zinc finger (C3HC4-type RING finger) family protein
265853_at	At2g42360	0,84	1,09	0,97	2,15	1,65	0,97	0,83	0,81 zinc finger (C3HC4-type RING finger) family protein
265854_at	At2g42370	0,86	0,93	0,92	0,98	0,83	1,04	0,99	0,97 expressed protein
265877_at	At2g42380	1,21	1,15	1,15	0,87	1,03	1,13	0,91	1,02 bZIP transcription factor family protein
265855_at	At2g42390	0,93	0,89	0,92	0,90	0,86	0,96	0,94	0,92 protein kinase C substrate, heavy chain-related
265827_at	At2g42400	1,23	1,07	1,32	1,01	1,10	0,90	1,05	1,35 expressed protein
265878_at	At2g42410	1,00	1,09	1,01	0,97	1,17	1,07	0,87	1,23 zinc finger (C2H2 type) family protein
265856_at	At2g42430	0,85	0,90	0,83	0,97	1,05	0,93	1,01	1,14 LOB domain protein 16 / lateral organ boundaries domain protein 16 (LBD1
257386_at	At2g42440	1,03	0,94	0,93	1,01	1,11	1,08	0,94	0,96 LOB domain protein 17 / lateral organ boundaries domain protein 17 (LBD1
265879_at	At2g42450	1,08	0,92	0,92	0,97	1,08	0,82	0,94	0,85 lipase class 3 family protein
265881_at	At2g42480	0,87	0,92	0,95	0,99	0,95	0,98	1,01	0,89 meprin and TRAF homology domain-containing protein / MATH domain-cor

265882_at	At2g42490	1,02	0,92	0,89	1,31	0,94	0,91	0,95	1,00	copper amine oxidase, putative
265857_s_at	At2g42500	1,17	0,99	1,06	0,99	1,05	0,94	0,96	0,94	serine/threonine protein phosphatase PP2A-4 catalytic subunit (PP2A4)
263493_at	At2g42520	0,96	0,96	1,02	0,92	0,89	0,97	0,98	0,95	DEAD box RNA helicase, putative
263495_at	At2g42530	0,28	0,20	0,19	0,50	0,44	1,11	1,28	0,94	cold-responsive protein / cold-regulated protein (cor15b)
263497_at	At2g42540	0,37	0,27	0,25	1,72	1,49	1,57	1,63	1,30	cold-responsive protein / cold-regulated protein (cor15a)
263492_at	At2g42560	0,74	0,88	1,01	0,93	0,94	0,98	0,98	0,99	late embryogenesis abundant domain-containing protein / LEA domain-cont
263496_at	At2g42570	1,17	1,26	1,00	1,22	1,40	0,93	0,96	1,09	expressed protein
263499_at	At2g42580	1,07	0,92	1,52	1,19	1,21	1,05	0,84	0,99	tetratricopeptide repeat (TPR)-containing protein
263494_at	At2g42590	0,87	0,82	0,81	0,95	1,04	0,94	0,97	0,95	14-3-3 protein GF14 mu (GRF9)
263491_at	At2g42600	0,88	0,74	0,84	0,90	0,93	1,06	1,05	1,20	phosphoenolpyruvate carboxylase, putative / PEP carboxylase, putative (PI
263498_at	At2g42610	0,90	0,81	0,84	1,11	1,11	0,66	0,54	0,56	expressed protein
263490_at	At2g42620	0,96	0,92	0,86	0,84	1,06	0,97	0,83	1,01	F-box family protein (ORE9)
263520_at	At2g42630	1,00	1,08	1,10	0,90	0,93	1,18	1,26	0,86	protein kinase family protein
263977_at	At2g42660	1,01	1,03	1,12	1,06	1,12	0,96	1,03	0,99	myb family transcription factor
263984_at	At2g42670	0,98	1,00	1,03	1,14	1,13	0,93	0,90	0,89	expressed protein
263978_at	At2g42680	1,33	1,14	1,12	1,20	1,28	0,89	0,99	0,95	ethylene-responsive transcriptional coactivator, putative
263987_at	At2g42690	1,21	1,51	1,94	0,85	0,96	1,19	1,21	1,08	lipase, putative
263976_at	At2g42700	0,88	1,00	1,10	0,90	1,08	0,98	1,12	1,09	expressed protein
263975_at	At2g42710	1,23	1,10	1,15	0,98	1,01	0,88	0,94	0,84	ribosomal protein L1 family protein
263974_at	At2g42720	1,22	1,16	1,20	1,05	1,17	1,05	0,90	0,92	F-box family protein
263973_at	At2g42740	1,42	1,41	1,33	1,60	1,54	1,22	1,04	1,13	60S ribosomal protein L11 (RPL11A)
263985_at	At2g42750	1,09	1,17	1,42	0,82	0,75	1,75	1,65	1,71	DNAJ heat shock N-terminal domain-containing protein
263972_at	At2g42760	0,64	1,03	0,53	1,52	1,06	0,81	0,88	0,85	expressed protein
263980_at	At2g42770	1,10	1,03	0,94	0,86	0,96	1,06	1,00	0,89	peroxisomal membrane 22 kDa family protein
263983_at	At2g42780	1,00	0,71	0,89	1,03	0,98	0,94	0,98	0,99	expressed protein
263986_at	At2g42790	0,59	0,60	0,64	0,96	0,97	0,91	0,91	0,93	citrate synthase, glyoxysomal, putative
263971_at	At2g42800	0,96	1,05	1,07	1,08	1,01	0,79	0,88	0,85	leucine-rich repeat family protein
263990_at	At2g42810	1,34	1,24	1,19	1,13	1,12	1,07	1,01	1,01	serine/threonine protein phosphatase, putative
263988_at	At2g42830	0,78	1,10	0,98	0,99	1,10	1,09	0,94	0,84	agamous-like MADS box protein AGL5 / floral homeodomain transcription fa
263979_at	At2g42840	1,35	1,20	1,12	1,43	1,41	0,90	0,86	0,80	protodermal factor 1 (PDF1)
263970_at	At2g42850	0,96	1,01	1,10	1,07	0,97	0,95	1,09	1,18	cytochrome P450 family protein
263982_at	At2g42860	1,06	0,94	0,91	1,00	1,08	0,99	1,02	0,96	expressed protein
263981_at	At2g42870	1,26	0,99	1,03	0,79	1,20	0,53	0,79	1,05	expressed protein
263989_at	At2g42880	0,91	0,88	0,86	1,05	0,96	0,95	0,93	1,02	mitogen-activated protein kinase, putative / MAPK, putative (MPK20)
265266_at	At2g42890	0,70	0,90	1,61	0,94	1,07	0,97	1,52	1,84	RNA recognition motif (RRM)-containing protein
265265_at	At2g42900	0,92	0,83	1,13	0,78	0,98	1,13	1,05	1,33	expressed protein
265270_at	At2g42910	1,29	1,26	1,10	1,01	0,81	0,92	0,92	0,94	ribose-phosphate pyrophosphokinase 4 / phosphoribosyl diphosphate synth
265267_at	At2g42920	1,03	1,01	1,09	0,97	0,78	1,21	1,16	1,15	pentatricopeptide (PPR) repeat-containing protein
265264_at	At2g42930	1,01	0,91	0,97	1,03	1,02	0,99	1,00	1,01	glycosyl hydrolase family protein 17
265263_at	At2g42940	0,90	1,05	1,02	1,05	0,97	1,10	0,95	1,00	DNA-binding family protein
265269_at	At2g42950	0,92	1,16	1,22	0,91	1,10	1,02	1,10	1,05	expressed protein
265268_at	At2g42960	1,01	1,06	1,11	1,03	0,96	0,88	1,13	1,03	protein kinase family protein
265262_at	At2g42980	0,96	1,07	0,92	0,94	1,04	0,94	1,03	0,99	aspartyl protease family protein
265261_at	At2g42990	0,95	0,97	1,00	0,86	1,17	0,89	0,91	0,80	GDSL-motif lipase/hydrolase family protein
265260_at	At2g43000	1,38	1,72	1,42	0,54	0,84	1,15	1,23	1,02	no apical meristem (NAM) family protein
265248_at	At2g43010	1,24	1,51	1,60	0,82	0,86	0,97	1,07	0,86	phytochrome-interacting factor 4 (PIF4) / basic helix-loop-helix protein 9 (bH

265244_at	At2g43020	0,73	0,81	0,91	0,88	1,03	0,90	1,02	1,09	amine oxidase family protein
265247_at	At2g43030	0,97	0,84	0,86	0,87	0,93	0,96	0,93	0,87	ribosomal protein L3 family protein
265243_at	At2g43040	1,10	0,91	0,88	1,03	1,04	0,89	1,02	0,94	calmodulin-binding protein
265246_at	At2g43050	1,12	1,14	0,93	0,75	1,16	0,86	0,87	0,88	pectinesterase family protein
265245_at	At2g43060	0,68	1,00	1,15	0,60	0,66	0,80	1,01	1,19	expressed protein
266449_at	At2g43080	0,75	0,67	0,71	1,10	1,08	0,91	0,86	0,81	oxidoreductase, 2OG-Fe(II) oxygenase family protein
266451_at	At2g43090	1,45	1,26	1,31	1,23	1,19	1,02	0,88	0,88	aconitase C-terminal domain-containing protein
266395_at	At2g43100	1,38	1,06	1,06	1,16	0,93	0,97	0,85	0,68	aconitase C-terminal domain-containing protein
266445_at	At2g43105	0,84	0,79	0,82	1,15	1,09	1,00	0,85	0,81	protease-associated (PA) domain-containing protein
266394_at	At2g43130	0,88	0,69	0,60	0,89	1,10	0,92	0,76	0,84	Ras-related protein (ARA-4) / small GTP-binding protein, putative
257373_at	At2g43140	1,82	1,28	2,18	1,07	0,90	1,00	1,28	1,26	basic helix-loop-helix (bHLH) family protein
266444_at	At2g43150	1,02	0,93	0,90	1,07	0,89	1,25	0,97	0,94	proline-rich extensin-like family protein
266436_at	At2g43160	0,90	0,80	0,91	0,93	1,04	0,87	0,79	0,86	epsin N-terminal homology (ENTH) domain-containing protein
266437_at	At2g43170	0,94	0,87	1,03	0,94	1,08	1,03	0,92	1,07	epsin N-terminal homology (ENTH) domain-containing protein
266438_at	At2g43180	0,85	0,84	0,90	0,95	0,74	1,24	1,03	1,10	expressed protein
266450_s_at	At2g43190	1,23	1,24	1,23	0,86	0,82	1,05	1,00	1,12	ribonuclease P family protein
266439_s_at	At2g43200	0,87	0,88	0,85	0,93	0,92	1,03	0,90	1,01	dehydration-responsive family protein
266448_s_at	At2g43210	0,92	0,89	1,22	1,09	1,06	1,08	1,09	1,17	UBX domain-containing protein
257372_at	At2g43220	1,01	0,97	1,02	1,05	1,09	1,04	0,84	1,23	DC1 domain-containing protein
266453_at	At2g43230	0,93	1,04	0,94	0,82	0,93	1,16	0,92	1,17	serine/threonine protein kinase, putative
266440_at	At2g43240	1,06	0,96	1,26	0,83	0,89	1,10	1,06	1,07	nucleotide-sugar transporter family protein
266441_at	At2g43250	1,07	1,00	0,85	1,12	1,15	0,86	0,89	1,02	expressed protein
266442_at	At2g43260	0,94	1,19	0,96	1,01	1,06	1,08	1,02	1,04	F-box family protein / S locus-related
266443_at	At2g43270	0,90	1,00	0,83	0,95	0,99	1,01	0,98	1,01	F-box family protein
257374_at	At2g43280	1,03	1,02	1,16	1,01	1,21	1,07	1,17	1,08	far-red impaired responsive family protein / FAR1 family protein
266447_at	At2g43290	0,76	0,74	0,50	0,84	0,96	1,12	1,25	1,11	calmodulin-like protein (MSS3)
266446_at	At2g43310	0,95	1,08	0,99	0,91	0,88	0,99	0,89	0,97	expressed protein
266452_at	At2g43320	0,83	0,79	1,13	1,29	1,37	1,12	1,12	1,51	expressed protein
260543_at	At2g43330	1,09	1,60	1,47	0,97	0,71	1,18	1,26	1,27	sugar transporter family protein
260545_at	At2g43350	0,96	0,82	0,72	1,11	1,11	0,94	0,89	0,94	glutathione peroxidase, putative
260548_at	At2g43360	0,95	0,99	1,08	1,02	0,92	0,84	0,78	0,79	biotin synthase (BioB) (BIO2)
260533_at	At2g43370	0,76	0,95	0,95	0,89	0,89	0,98	0,93	0,99	U1 small nuclear ribonucleoprotein 70 kDa, putative
260534_at	At2g43380	0,99	0,98	0,99	1,06	1,11	0,90	1,00	0,89	---
260535_at	At2g43390	0,97	1,02	1,03	0,94	1,01	1,07	1,02	0,89	expressed protein
260536_at	At2g43400	1,14	1,14	1,05	0,81	0,86	1,00	1,10	0,99	electron transfer flavoprotein-ubiquinone oxidoreductase family protein
260550_at	At2g43420	1,12	1,17	1,16	1,10	1,02	0,93	0,90	0,91	3-beta hydroxysteroid dehydrogenase/isomerase family protein
260552_at	At2g43430	0,97	0,75	1,16	1,31	1,24	0,90	0,99	1,22	hydroxyacylglutathione hydrolase, mitochondrial / glyoxalase II (GLX2-1)
260537_at	At2g43450	1,02	1,06	1,14	1,02	1,03	0,79	0,95	1,11	expressed protein
260538_at	At2g43460	1,56	1,25	1,11	1,17	1,26	1,01	0,93	0,96	60S ribosomal protein L38 (RPL38A)
260539_at	At2g43480	0,98	1,13	1,17	1,03	1,22	1,02	1,17	1,19	peroxidase, putative
260540_at	At2g43500	0,79	0,91	1,02	0,70	0,84	1,04	1,23	1,22	RWP-RK domain-containing protein
260551_at	At2g43510	0,87	0,87	0,76	0,51	0,47	1,68	1,39	1,28	trypsin inhibitor, putative
260546_at	At2g43520	0,42	0,45	0,41	0,61	0,63	0,96	0,96	0,85	trypsin inhibitor, putative
260541_at	At2g43530	0,60	0,49	0,54	0,97	0,83	0,96	0,87	0,77	trypsin inhibitor, putative
260549_at	At2g43535	0,88	0,88	0,86	1,05	1,13	0,69	0,73	0,67	trypsin inhibitor, putative
260544_at	At2g43540	0,96	0,88	1,03	0,82	0,72	1,00	0,83	1,27	expressed protein

260547_at	At2g43550	0,80	0,62	0,64	0,99	0,86	0,86	0,76	0,75	trypsin inhibitor, putative
260542_at	At2g43560	0,89	0,79	0,79	0,78	0,93	1,11	1,14	1,14	immunophilin / FKBP-type peptidyl-prolyl cis-trans isomerase family protein
260568_at	At2g43570	1,11	1,20	0,98	1,22	1,00	6,49	4,35	5,67	chitinase, putative
260561_at	At2g43580	1,06	1,00	0,86	1,05	1,10	1,08	1,01	0,87	chitinase, putative
260560_at	At2g43590	1,21	1,62	1,31	0,81	0,76	1,21	1,08	1,02	chitinase, putative
260558_at	At2g43600	0,97	0,95	0,98	1,37	1,38	0,98	1,35	1,19	glycoside hydrolase family 19 protein
260557_at	At2g43610	1,94	2,18	1,47	2,57	1,82	1,76	1,72	1,71	glycoside hydrolase family 19 protein
260556_at	At2g43620	2,16	2,39	2,04	4,29	2,30	1,60	0,82	0,82	chitinase, putative
260586_at	At2g43630	1,07	0,96	1,09	0,89	1,01	1,02	0,99	0,98	expressed protein
260569_at	At2g43640	1,12	1,01	0,80	1,36	1,19	0,97	0,91	0,88	signal recognition particle 14 kDa family protein / SRP14 family protein
260585_at	At2g43650	1,53	1,18	1,11	0,91	0,80	1,42	1,20	1,54	Sas10/U3 ribonucleoprotein (Utp) family protein
260584_at	At2g43660	0,94	0,91	1,06	1,04	1,03	1,04	1,06	1,17	glycosyl hydrolase family protein 17
260611_at	At2g43670	1,01	1,07	1,17	1,14	1,12	1,06	1,12	1,16	glycosyl hydrolase family protein 17
260610_at	At2g43680	0,84	0,83	0,86	0,77	0,87	0,91	0,88	1,05	calmodulin-binding family protein
260609_at	At2g43690	0,99	0,98	0,86	0,91	1,04	0,87	0,88	0,91	lectin protein kinase, putative
260607_at	At2g43700	0,84	1,04	0,81	0,99	1,03	0,98	1,12	1,00	lectin protein kinase family protein
260570_at	At2g43710	0,98	0,85	0,93	0,92	1,01	1,00	0,92	0,89	acyl-(acyl-carrier-protein) desaturase / stearoyl-ACP desaturase (SSI2)
257496_s_at	At2g43730	1,01	1,01	1,06	0,91	1,14	0,94	1,03	1,04	lectin-related
260566_at	At2g43750	0,79	0,67	0,71	0,95	0,87	0,87	0,86	0,89	cysteine synthase, chloroplast / O-acetylserine (thiol)-lyase / O-acetylserine
260572_at	At2g43760	0,81	0,91	0,93	1,14	1,00	0,88	0,91	1,04	molybdopterin biosynthesis MoaE family protein
260606_at	At2g43770	1,34	1,18	1,18	1,04	1,08	0,98	0,86	0,81	transducin family protein / WD-40 repeat family protein
260605_at	At2g43780	1,94	1,64	1,30	1,56	1,38	0,97	0,95	0,76	expressed protein
260571_at	At2g43790	0,83	0,76	0,79	0,91	0,97	0,88	0,86	0,98	mitogen-activated protein kinase, putative / MAPK, putative (MPK6)
260565_at	At2g43800	1,05	1,11	1,09	1,33	1,32	1,05	0,77	0,75	formin homology 2 domain-containing protein / FH2 domain-containing prot
260564_at	At2g43810	1,54	1,17	1,01	1,07	1,08	0,93	0,98	0,79	small nuclear ribonucleoprotein F, putative / U6 snRNA-associated Sm-like
260567_at	At2g43820	0,48	0,44	0,93	0,70	0,63	0,83	0,86	1,11	UDP-glucoronosyl/UDP-glucosyl transferase family protein
260563_at	At2g43840	0,89	1,05	1,10	0,88	1,02	0,95	1,11	1,00	UDP-glucoronosyl/UDP-glucosyl transferase family protein
260562_at	At2g43850	1,13	0,98	1,05	0,84	0,96	1,07	1,02	1,04	ankyrin protein kinase, putative (APK1)
260559_at	At2g43860	0,93	1,06	0,77	1,04	1,07	1,13	0,92	0,93	polygalacturonase, putative / pectinase, putative
260608_at	At2g43870	0,95	1,01	0,90	1,09	1,05	0,94	1,06	1,03	polygalacturonase, putative / pectinase, putative
267222_at	At2g43880	1,13	1,05	1,09	1,06	1,19	0,95	1,12	1,15	polygalacturonase, putative / pectinase, putative
267228_at	At2g43890	1,00	1,05	1,09	0,88	0,95	0,98	1,18	1,10	polygalacturonase, putative / pectinase, putative
267231_at	At2g43900	0,88	1,04	0,95	0,86	0,99	0,96	1,01	1,20	endonuclease/exonuclease/phosphatase family protein
267233_s_at	At2g43920	1,14	1,13	1,19	1,02	1,14	1,05	1,07	0,94	thiol methyltransferase, putative
267234_x_at	At2g43930	0,88	1,05	1,05	0,89	0,96	0,93	0,98	0,85	protein kinase family protein
267235_at	At2g43940	0,84	0,81	0,79	1,08	0,97	0,94	0,90	0,84	expressed protein
267185_at	At2g43950	1,13	1,02	1,15	0,91	0,93	1,05	0,99	0,99	expressed protein
267214_at	At2g43970	1,00	0,79	0,90	0,81	0,82	1,06	1,07	1,22	La domain-containing protein
267223_at	At2g43980	1,04	0,96	0,93	1,13	1,07	1,02	1,04	1,02	inositol 1,3,4-trisphosphate 5/6-kinase family protein
267224_at	At2g43990	0,92	1,03	1,11	0,93	1,07	0,97	0,86	0,95	expressed protein
267225_at	At2g44000	1,05	0,92	0,97	1,00	0,98	1,02	0,91	0,96	expressed protein
267226_at	At2g44010	0,89	1,00	0,79	1,17	1,30	1,02	0,91	0,75	expressed protein
267183_at	At2g44020	0,87	0,79	0,95	1,09	1,07	0,86	0,87	0,82	mitochondrial transcription termination factor-related / mTERF-related
267227_at	At2g44030	1,03	0,97	0,96	0,93	1,10	1,07	1,05	0,91	kelch repeat-containing F-box family protein
267237_s_at	At2g44040	1,50	1,05	0,95	1,14	1,08	0,81	0,82	0,80	dihydrodipicolinate reductase family protein
267188_at	At2g44050	1,11	1,01	0,96	1,28	1,07	0,91	0,80	0,85	6,7-dimethyl-8-ribityllumazine synthase / DMRL synthase / lumazine syntha

267212_at	At2g44060	1,00	0,91	0,85	1,40	1,36	0,99	0,92	0,96	late embryogenesis abundant family protein / LEA family protein
267211_at	At2g44065	1,32	1,17	1,11	1,28	1,42	1,03	0,92	1,05	ribosomal protein L2 family protein
267229_s_at	At2g44070	0,99	1,01	0,95	0,87	0,97	0,87	0,84	0,82	eukaryotic translation initiation factor 2B family protein / eIF-2B family prote
267230_at	At2g44080	1,54	2,00	1,27	1,28	1,21	0,81	0,85	0,94	expressed protein
267186_at	At2g44090	0,98	0,99	0,96	1,11	1,02	0,87	1,07	0,91	expressed protein
267236_at	At2g44100	1,08	1,00	0,94	1,25	1,29	0,87	0,86	0,80	Rab GDP dissociation inhibitor (GDI1)
267191_at	At2g44110	0,99	0,99	1,01	1,30	1,22	1,27	1,01	0,64	seven transmembrane MLO family protein / MLO-like protein 15 (MLO15)
267213_at	At2g44120	1,46	1,26	1,21	1,27	1,27	1,00	1,05	1,07	60S ribosomal protein L7 (RPL7C)
267238_at	At2g44130	1,10	1,00	0,96	0,95	1,11	0,99	0,99	1,07	kelch repeat-containing F-box family protein
267184_at	At2g44150	1,07	1,35	1,54	0,90	0,99	1,04	1,06	1,46	SET domain-containing protein (ASHH3)
267187_s_at	At2g44160	1,12	1,01	1,00	1,05	1,02	1,03	0,93	0,83	methylenetetrahydrofolate reductase 2 (MTHFR2)
267190_at	At2g44170	0,98	1,02	1,18	1,03	1,17	1,04	1,12	1,30	pseudogene, myristoyl-CoA:protein N-myristoyltransferase (NMT), putative
267189_at	At2g44180	0,73	0,81	0,88	1,03	0,99	0,86	0,96	0,99	methionyl aminopeptidase, putative / methionine aminopeptidase, putative /
267232_at	At2g44190	1,08	1,11	0,97	1,14	1,26	1,00	0,98	0,90	expressed protein
267399_at	At2g44195	1,04	1,02	1,00	0,96	1,19	1,18	1,03	1,07	hypothetical protein
267341_at	At2g44200	1,06	1,22	1,63	0,97	0,86	1,51	1,52	1,66	expressed protein
267367_at	At2g44210	0,80	0,73	0,75	1,08	1,01	0,88	0,87	0,85	expressed protein
267344_at	At2g44230	1,26	1,14	1,05	0,74	0,75	0,89	1,03	1,11	expressed protein
267345_at	At2g44240	1,06	1,09	0,92	1,00	1,13	1,03	0,92	1,17	expressed protein
267395_at	At2g44250	0,95	0,98	1,05	1,02	1,03	1,21	1,04	0,91	expressed protein
267343_at	At2g44260	1,12	1,18	1,19	1,18	1,46	1,08	0,97	1,04	expressed protein
267396_at	At2g44270	1,22	1,12	1,20	1,02	1,06	1,06	0,94	1,02	expressed protein
267382_at	At2g44300	1,08	0,96	1,18	1,45	1,77	1,00	0,93	1,00	lipid transfer protein-related
267366_at	At2g44310	0,64	0,57	0,59	0,97	0,97	0,90	0,72	0,73	calcium-binding EF hand family protein
267368_at	At2g44350	0,95	0,85	0,79	1,12	1,17	0,91	0,97	0,97	citrate synthase, mitochondrial, putative
267383_at	At2g44360	0,93	0,82	0,76	0,98	1,24	0,94	0,76	0,81	expressed protein
267384_at	At2g44370	0,89	1,05	0,65	1,91	1,20	1,13	1,04	0,90	DC1 domain-containing protein
267385_at	At2g44380	0,86	0,98	0,76	2,00	1,28	0,78	0,83	0,89	DC1 domain-containing protein
267369_at	At2g44420	0,92	0,96	0,93	0,87	1,19	1,03	0,91	1,07	protein N-terminal asparagine amidohydrolase family protein
267386_at	At2g44430	0,80	0,71	0,71	0,94	1,32	1,00	0,95	1,09	DNA-binding bromodomain-containing protein
267387_at	At2g44440	1,09	1,00	1,27	0,87	0,93	1,07	1,05	0,89	emsy N terminus domain-containing protein / ENT domain-containing protei
267388_at	At2g44450	0,96	0,83	0,86	1,37	1,12	0,80	0,98	0,88	glycosyl hydrolase family 1 protein
267389_at	At2g44460	1,51	1,33	1,68	0,78	0,55	0,88	0,92	1,20	glycosyl hydrolase family 1 protein
267390_at	At2g44470	1,04	0,89	1,01	0,93	0,87	1,06	1,05	1,03	Clone asmbl_7698 unknown mRNA sequence
267391_at	At2g44480	0,93	1,03	1,09	0,96	0,98	0,94	1,22	1,17	glycosyl hydrolase family 1 protein
267392_at	At2g44490	0,83	0,82	1,22	0,89	0,80	1,01	0,85	0,99	glycosyl hydrolase family 1 protein
267393_at	At2g44500	0,99	1,20	1,01	0,81	0,82	1,20	1,77	1,91	expressed protein
267371_at	At2g44510	1,12	0,94	1,04	1,10	0,85	0,92	0,76	1,24	p21Cip1-binding protein-related
267342_at	At2g44520	0,92	0,90	0,75	1,21	1,07	0,95	0,80	0,77	UbiA prenyltransferase family protein
267370_at	At2g44525	1,12	1,02	0,92	1,13	1,01	1,01	0,86	0,85	expressed protein
267365_at	At2g44530	1,17	0,99	0,95	0,81	0,83	0,97	0,93	0,98	ribose-phosphate pyrophosphokinase, putative / phosphoribosyl diphospha
267394_s_at	At2g44540	0,95	1,00	0,94	1,09	0,95	0,94	1,02	1,00	glycosyl hydrolase family 9 protein
267398_at	At2g44560	0,97	1,00	1,04	1,05	0,94	0,99	1,00	0,92	glycosyl hydrolase family 9 protein
266877_at	At2g44570	0,89	0,85	0,94	1,01	1,04	0,90	0,84	1,09	glycosyl hydrolase family 9 protein
266878_at	At2g44580	1,04	1,17	0,96	1,04	1,13	0,82	1,04	0,92	zinc finger (C3HC4-type RING finger) family protein
266879_at	At2g44590	0,97	0,95	0,91	1,21	1,01	1,02	0,97	1,02	dynamin-like protein D (DL1D)

266885_at	At2g44610	0,94	0,77	0,76	1,24	1,26	0,83	0,79	0,86	Ras-related GTP-binding protein, putative
266890_at	At2g44620	1,24	1,12	1,04	1,23	1,05	1,01	1,03	0,86	acyl carrier protein, mitochondrial / ACP / NADH-ubiquinone oxidoreductase
266868_at	At2g44630	0,95	1,04	0,95	0,92	1,02	0,98	1,08	1,01	kelch repeat-containing F-box family protein
266889_at	At2g44640	1,06	0,99	1,06	0,77	0,76	0,92	0,87	0,81	expressed protein
266887_at	At2g44650	1,08	0,95	0,96	0,95	0,98	1,06	0,98	0,90	chloroplast chaperonin 10 (cpn10)
266869_at	At2g44660	1,05	0,98	0,96	1,18	1,25	1,01	0,87	0,98	ALG6, ALG8 glycosyltransferase family protein
266882_at	At2g44670	1,28	1,05	0,83	1,78	1,24	0,83	1,06	0,94	senescence-associated protein-related
266881_at	At2g44680	1,15	1,14	1,13	1,14	1,14	0,99	0,80	1,02	casein kinase II beta chain, putative
266891_at	At2g44690	1,26	1,08	1,20	1,16	0,97	0,93	1,00	0,86	Rac-like GTP-binding protein (ARAC9)
266870_at	At2g44710	1,17	1,02	1,07	0,86	0,78	1,04	0,88	0,91	RNA recognition motif (RRM)-containing protein
266871_at	At2g44720	1,01	1,05	1,12	0,95	0,82	0,94	1,06	1,08	RNA recognition motif (RRM)-containing protein
266872_at	At2g44730	0,78	0,97	0,94	1,00	1,04	0,91	0,83	0,86	expressed protein
266873_at	At2g44740	1,09	0,99	0,97	1,11	1,09	1,06	0,96	1,26	cyclin family protein
266886_at	At2g44745	1,10	1,19	1,29	1,05	1,04	0,99	0,91	1,03	WRKY family transcription factor
266888_s_at	At2g44750	1,24	1,08	1,22	0,84	0,80	1,16	1,18	1,27	thiamin pyrophosphokinase, putative
266874_at	At2g44760	0,92	0,90	1,31	0,68	0,76	1,16	1,10	1,20	expressed protein
266880_at	At2g44770	0,98	0,96	1,00	1,18	1,22	0,94	0,84	0,86	phagocytosis and cell motility protein ELMO1-related
266884_at	At2g44790	1,31	1,49	1,27	1,19	1,03	1,10	1,05	0,79	uclacyanin II
266875_at	At2g44800	1,01	0,93	0,97	0,94	0,89	1,12	0,91	0,91	oxidoreductase, 2OG-Fe(II) oxygenase family protein
266883_at	At2g44810	0,88	0,94	0,97	0,93	1,14	0,87	0,92	1,07	defective in anther dehiscence1 (DAD1)
266876_at	At2g44820	1,12	0,91	1,05	0,99	0,81	1,11	1,09	1,19	expressed protein
266812_at	At2g44830	1,12	1,07	1,09	1,12	1,21	0,93	1,08	1,11	protein kinase, putative
266821_at	At2g44840	1,04	1,15	0,92	1,19	0,90	0,95	1,10	0,90	ethylene-responsive element-binding protein, putative
266811_at	At2g44850	0,97	0,93	0,91	0,86	0,95	0,88	0,92	0,88	expressed protein
266822_at	At2g44860	1,34	1,04	1,06	1,04	1,27	0,94	1,02	1,07	60S ribosomal protein L24, putative
266819_at	At2g44870	0,87	0,81	0,75	0,73	0,60	1,04	0,92	0,99	expressed protein
266810_at	At2g44880	1,16	1,01	1,06	0,87	0,90	0,99	1,06	1,01	pentatricopeptide (PPR) repeat-containing protein
266815_at	At2g44900	0,99	0,92	1,08	0,92	1,06	0,97	0,91	0,99	armadillo/beta-catenin repeat family protein / F-box family protein
266814_at	At2g44910	0,87	1,03	0,99	1,01	1,13	1,06	1,03	1,21	homeobox-leucine zipper protein 4 (HB-4) / HD-ZIP protein 4
266813_at	At2g44920	0,76	0,79	0,75	0,71	0,83	1,10	0,99	1,10	thylakoid lumenal 15 kDa protein, chloroplast
266823_at	At2g44930	0,99	1,08	1,11	0,92	1,02	1,08	1,09	1,03	expressed protein
266820_at	At2g44940	0,68	0,75	0,79	0,69	0,72	0,94	0,96	0,96	AP2 domain-containing transcription factor TINY, putative
266818_at	At2g44950	1,19	1,30	1,51	0,68	0,88	1,24	1,28	1,21	zinc finger (C3HC4-type RING finger) family protein
266817_at	At2g44960	1,23	1,21	1,11	0,92	1,01	1,05	0,93	1,27	zinc finger (C3HC4-type RING finger) family protein
266816_at	At2g44970	0,91	0,83	0,87	1,05	1,05	1,07	1,01	1,09	lipase-related
266130_at	At2g44980	0,87	0,87	0,91	0,90	1,00	0,82	0,92	0,83	transcription regulatory protein SNF2, putative
266129_at	At2g44990	1,01	1,06	0,99	1,06	1,04	0,99	0,92	0,90	dioxygenase-related
266128_at	At2g45000	1,01	0,99	0,93	1,02	1,03	1,00	0,98	0,76	expressed protein
266137_at	At2g45010	0,93	0,87	1,02	1,07	1,16	0,93	1,00	1,16	expressed protein
266127_s_at	At2g45030	1,54	1,40	1,26	0,93	0,94	1,09	1,12	1,17	mitochondrial elongation factor, putative
266126_at	At2g45040	1,02	1,06	0,98	0,92	1,14	1,07	1,07	1,19	matrix metalloproteinase
266125_at	At2g45050	0,99	1,07	1,01	1,13	1,13	0,98	0,92	0,88	zinc finger (GATA type) family protein
266136_at	At2g45060	1,12	0,87	1,02	1,05	1,13	1,12	0,88	0,82	expressed protein
266105_at	At2g45070	1,09	0,92	0,95	1,51	1,37	0,88	0,92	1,01	sec61beta family protein
266124_at	At2g45080	1,45	1,36	1,18	1,40	1,49	1,10	1,02	1,07	cyclin family protein
266135_at	At2g45100	0,94	0,97	1,02	1,06	1,01	0,97	1,12	1,45	transcription factor IIB (TFIIB) family protein

266134_s_at	At2g45110	0,96	1,00	1,04	0,92	1,06	1,05	1,01	0,93	beta-expansin, putative (EXBP2)
266133_at	At2g45120	1,01	0,95	1,04	0,93	0,98	0,99	0,92	0,96	zinc finger (C2H2 type) family protein
266132_at	At2g45130	1,07	1,05	0,88	1,24	1,11	0,93	0,98	0,90	SPX (SYG1/Pho81/XPR1) domain-containing protein
266103_at	At2g45140	1,01	0,96	1,01	1,12	1,08	1,02	0,89	0,88	vesicle-associated membrane protein, putative / VAMP, putative
266104_at	At2g45150	0,92	0,79	1,32	0,88	0,68	0,79	0,87	1,19	phosphatidate cytidylyltransferase family protein
266131_at	At2g45160	0,88	0,76	0,75	0,88	0,96	0,94	0,92	0,88	scarecrow transcription factor family protein
266106_at	At2g45170	1,53	1,94	1,84	0,74	1,00	1,44	1,27	1,51	autophagy 8e (APG8e)
266123_at	At2g45180	1,14	1,17	1,06	1,09	1,02	1,03	0,83	0,89	protease inhibitor/seed storage/lipid transfer protein (LTP) family protein
245138_at	At2g45190	0,87	0,88	1,12	1,15	1,08	1,05	0,98	0,99	axial regulator YABBY1 (YABBY1) / abnormal floral organs protein (AFO) /
245146_at	At2g45200	1,02	1,02	1,08	0,97	0,95	0,95	0,98	0,97	Golgi SNARE 12 protein / Golgi SNAP receptor complex member 1
245136_at	At2g45210	0,69	0,59	0,69	0,98	0,96	0,82	0,89	1,08	auxin-responsive protein-related
245148_at	At2g45220	1,14	1,46	1,66	2,12	1,02	1,59	1,42	1,55	pectinesterase family protein
245135_at	At2g45230	0,87	1,00	0,87	0,98	1,17	1,06	1,06	0,94	---
245144_at	At2g45240	1,06	0,85	0,91	1,17	1,02	0,93	0,86	0,80	methionyl aminopeptidase, putative / methionine aminopeptidase, putative /
245134_s_at	At2g45250	1,10	1,12	1,24	0,85	1,02	0,97	1,02	1,02	expressed protein
245142_at	At2g45270	0,93	0,89	0,94	0,84	0,89	1,00	0,97	0,99	glycoprotease M22 family protein
245147_at	At2g45280	0,91	0,91	0,94	1,15	0,93	0,94	0,91	0,73	DNA repair family protein
245089_at	At2g45290	1,04	1,19	0,90	1,26	0,91	1,10	1,06	0,97	transketolase, putative
245133_at	At2g45310	1,01	0,87	0,76	1,09	1,10	1,07	0,96	1,15	NAD-dependent epimerase/dehydratase family protein
245132_at	At2g45320	0,91	0,98	1,04	1,11	1,13	1,00	1,02	1,02	expressed protein
245131_s_at	At2g45330	0,87	0,98	1,08	1,15	1,08	0,91	1,02	1,00	tRNA 2'phosphotransferase, putative
245130_at	At2g45340	0,89	0,93	1,04	0,76	0,80	1,02	1,00	1,16	leucine-rich repeat transmembrane protein kinase, putative
245129_at	At2g45350	0,88	0,94	1,05	0,81	1,04	1,07	0,93	0,99	pentatricopeptide (PPR) repeat-containing protein
245128_at	At2g45380	0,99	0,95	0,97	1,05	1,04	0,98	0,95	1,15	expressed protein
245141_at	At2g45400	0,81	0,85	0,89	1,09	1,29	0,88	1,01	1,04	dihydroflavonol 4-reductase family / dihydrokaempferol 4-reductase family
245140_at	At2g45420	1,05	1,03	0,97	1,00	0,96	1,07	1,03	0,93	LOB domain protein 18 / lateral organ boundaries domain protein 18 (LBD1
245139_at	At2g45430	0,99	1,03	1,07	1,44	1,78	1,03	1,15	1,06	DNA-binding protein-related
245145_at	At2g45440	0,99	0,89	0,82	0,85	0,71	0,99	0,99	0,85	dihydrodipicolinate synthase 2 (DHDPS2)
245143_at	At2g45450	1,14	1,09	1,10	1,10	1,12	1,15	0,91	0,88	expressed protein
245137_at	At2g45460	1,16	1,36	1,21	0,85	0,97	1,07	0,91	1,09	forkhead-associated domain-containing protein / FHA domain-containing pr
251395_at	At2g45470	1,05	1,02	0,90	1,25	1,28	0,76	0,66	0,68	fasciclin-like arabinogalactan-protein (FLA8)
267529_at	At2g45490	1,31	1,36	1,47	1,47	1,30	0,93	0,78	0,92	protein kinase, putative
267499_at	At2g45500	1,01	0,85	1,18	0,97	1,06	1,04	1,17	1,11	AAA-type ATPase family protein
267500_s_at	At2g45510	0,63	0,59	0,48	1,12	1,03	0,94	1,15	1,17	cytochrome P450 family protein
267506_at	At2g45520	1,43	1,25	1,27	1,02	1,21	1,07	1,08	0,99	expressed protein
267504_at	At2g45530	1,14	1,08	1,05	1,10	1,28	0,85	0,94	0,91	zinc finger (C3HC4-type RING finger) family protein
267501_at	At2g45540	0,77	0,73	0,69	0,91	0,90	0,87	0,90	0,76	WD-40 repeat family protein / beige-related
267502_at	At2g45550	1,04	1,26	1,43	1,01	0,97	1,07	1,05	0,87	cytochrome P450 family protein
267505_at	At2g45560	0,86	1,07	1,04	1,03	0,94	0,91	0,89	1,05	cytochrome P450 family protein
267559_at	At2g45570	0,88	0,96	0,96	1,00	1,06	1,13	1,01	0,90	cytochrome P450 76C2, putative (CYP76C2) (YLS6)
267560_at	At2g45580	0,99	1,07	1,05	0,87	0,94	0,95	1,03	1,07	cytochrome P450 family protein
267561_at	At2g45590	0,73	0,77	1,04	0,68	0,84	0,85	0,84	0,96	protein kinase family protein
267503_at	At2g45600	0,81	0,95	0,98	0,77	0,90	0,86	0,83	0,92	expressed protein
267527_at	At2g45610	1,07	1,01	1,01	0,86	1,01	0,99	1,03	0,97	expressed protein
267513_at	At2g45620	1,13	1,23	1,37	0,84	0,91	1,04	1,01	1,25	nucleotidyltransferase family protein
267514_at	At2g45630	0,96	1,08	0,72	0,91	1,01	0,88	0,80	0,76	oxidoreductase family protein

267510_at	At2g45640	1,35	1,27	1,18	1,16	1,04	1,09	1,08	0,92 sin3 associated polypeptide p18 family protein
267528_at	At2g45650	0,89	1,01	1,05	0,99	1,18	0,90	1,09	0,97 MADS-box protein (AGL6)
267509_at	At2g45660	1,52	1,39	1,39	1,20	1,13	1,55	1,49	1,28 MADS-box protein (AGL20)
267511_at	At2g45670	1,20	1,11	1,06	0,99	0,93	1,11	1,05	1,03 calcineurin B subunit-related
267515_at	At2g45680	0,95	0,85	1,32	1,12	0,86	1,00	0,98	0,92 TCP family transcription factor, putative
267512_at	At2g45690	1,02	0,96	1,25	0,91	1,03	1,11	1,02	0,87 shrunken seed protein (SSE1)
267508_at	At2g45700	0,80	0,97	1,09	0,97	0,89	1,09	0,95	0,85 sterile alpha motif (SAM) domain-containing protein
267507_at	At2g45710	1,24	1,02	0,95	1,32	1,35	1,03	0,96	0,97 40S ribosomal protein S27 (RPS27A)
267498_at	At2g45720	0,99	1,04	0,95	0,87	1,01	1,11	1,21	1,04 armadillo/beta-catenin repeat family protein
266924_at	At2g45730	1,28	1,21	1,15	0,96	0,78	1,01	0,91	0,93 eukaryotic initiation factor 3 gamma subunit family protein
266925_at	At2g45740	0,98	1,02	1,21	0,76	0,72	1,15	1,18	1,17 peroxisomal biogenesis factor 11 family protein / PEX11 family protein
266920_at	At2g45750	1,09	0,91	0,92	0,98	0,82	0,91	1,03	1,02 dehydration-responsive family protein
257363_at	At2g45760	1,03	0,94	0,98	1,19	1,01	1,17	1,01	1,03 BON1-associated protein (BAP1)-related
266867_at	At2g45770	0,74	0,71	0,65	0,77	0,82	1,04	0,97	0,92 signal recognition particle receptor protein, chloroplast (FTSY)
266919_at	At2g45780	0,96	1,04	0,95	0,99	0,98	0,97	0,98	1,07 expressed protein
266928_at	At2g45790	1,03	0,98	0,95	1,09	0,91	0,98	0,97	0,88 eukaryotic phosphomannomutase family protein
266918_at	At2g45800	0,95	1,19	1,05	0,91	0,78	0,86	0,96	1,08 LIM domain-containing protein
266896_at	At2g45810	1,27	1,91	2,25	1,08	0,91	1,15	1,13	1,23 DEAD/DEAH box helicase, putative
266897_at	At2g45820	0,84	0,79	0,75	0,92	0,94	1,10	1,20	1,23 DNA-binding protein, putative
266917_at	At2g45830	0,83	0,98	0,99	1,04	1,01	0,89	1,06	1,10 expressed protein
257362_at	At2g45840	0,89	1,11	0,95	0,97	0,98	1,11	1,02	1,03 expressed protein
266929_at	At2g45850	0,90	1,06	1,37	0,61	0,74	0,94	0,93	1,05 DNA-binding family protein
266916_at	At2g45860	1,63	1,17	1,27	1,35	1,53	1,19	1,02	0,95 expressed protein
266915_at	At2g45870	0,98	1,06	1,06	1,02	1,00	0,76	0,84	0,76 expressed protein
266914_at	At2g45880	0,82	0,93	1,04	1,05	0,92	0,98	1,18	1,17 glycosyl hydrolase family 14 protein
266913_at	At2g45890	1,08	0,98	1,05	1,07	1,05	0,86	0,93	0,93 expressed protein
266912_at	At2g45900	0,98	1,12	0,95	1,07	1,06	1,01	0,96	0,87 expressed protein
266911_at	At2g45910	0,74	0,83	1,19	0,79	0,79	0,77	1,09	1,62 protein kinase family protein / U-box domain-containing protein
266910_at	At2g45920	1,19	0,87	1,25	1,11	0,97	1,39	1,31	1,66 U-box domain-containing protein
266930_at	At2g45930	0,91	1,24	1,21	0,80	0,92	1,09	1,02	0,85 expressed protein
257364_at	At2g45940	0,99	0,93	0,99	1,07	0,98	1,04	1,00	1,14 hypothetical protein
266922_s_at	At2g45950	1,31	1,24	1,25	0,84	0,92	0,94	1,05	0,92 SKP1 family protein
266927_at	At2g45960	1,19	1,24	1,55	1,53	1,47	0,99	0,92	0,99 plasma membrane intrinsic protein 1B (PIP1B) / aquaporin PIP1.2 (PIP1.2)
266921_at	At2g45970	1,21	1,24	1,15	1,12	0,92	0,89	0,99	1,15 cytochrome P450, putative
266923_at	At2g45980	0,81	0,93	1,37	0,94	1,10	1,01	1,01	1,38 expressed protein
266898_at	At2g45990	0,87	0,78	0,80	0,89	1,09	1,08	1,16	1,22 expressed protein
266926_at	At2g46000	1,03	1,10	0,88	1,20	1,16	1,19	1,04	0,94 expressed protein
266909_at	At2g46010	0,98	0,76	0,94	0,99	1,00	0,90	1,02	1,44 transcription regulatory protein SNF2, putative
266605_at	At2g46020	0,88	0,84	1,06	0,98	0,96	1,07	1,14	1,13 transcription regulatory protein SNF2, putative
266604_at	At2g46030	1,43	1,54	1,59	1,12	1,10	1,20	1,22	1,38 ubiquitin-conjugating enzyme 6 (UBC6)
266603_at	At2g46040	0,95	0,99	0,95	0,87	1,02	1,04	0,88	1,19 ARID/BRIGHT DNA-binding domain-containing protein / ELM2 domain-cont
266602_at	At2g46050	0,85	1,15	1,10	0,84	0,93	1,09	0,96	1,25 pentatricopeptide (PPR) repeat-containing protein
266601_at	At2g46060	0,84	1,13	1,02	0,88	0,81	0,91	1,05	1,09 transmembrane protein-related
266600_at	At2g46070	0,77	0,72	1,35	1,01	1,27	0,95	0,97	1,24 mitogen-activated protein kinase, putative / MAPK, putative (MPK12)
266557_at	At2g46080	1,13	1,12	1,16	1,21	1,32	1,02	1,18	1,31 expressed protein
266582_at	At2g46090	0,92	0,86	0,79	0,83	0,83	1,03	1,00	1,02 diacylglycerol kinase family protein

266599_at	At2g46100	1,07	1,17	1,58	0,89	0,90	1,21	1,40	1,72 expressed protein
266598_at	At2g46110	0,87	0,90	0,81	0,96	0,75	0,70	0,89	0,80 ketopantoate hydroxymethyltransferase family protein
266597_at	At2g46130	0,99	1,03	0,96	0,97	1,03	1,07	1,04	1,00 WRKY family transcription factor
266581_at	At2g46140	1,58	1,44	1,05	1,82	1,36	1,26	1,11	1,11 late embryogenesis abundant protein, putative / LEA protein, putative
266596_at	At2g46150	1,00	0,83	0,72	1,06	1,00	0,94	0,97	1,06 expressed protein
266553_at	At2g46170	0,96	0,91	0,87	1,14	1,02	1,00	0,86	0,83 reticulon family protein (RTNLB5)
266595_at	At2g46180	1,34	1,16	1,82	0,82	0,93	1,18	1,35	1,43 intracellular protein transport protein USO1-related
266594_at	At2g46190	0,86	1,06	1,00	1,06	1,05	0,93	0,94	1,14 hypothetical protein
266593_at	At2g46200	0,77	0,71	0,94	0,78	1,11	1,19	0,84	0,99 expressed protein
266592_at	At2g46210	1,17	0,89	1,04	0,99	1,00	0,83	0,81	0,76 delta-8 sphingolipid desaturase, putative
266583_at	At2g46220	0,96	1,05	1,14	0,81	0,69	1,09	0,98	1,01 expressed protein
266591_at	At2g46225	1,24	1,17	1,19	1,00	1,17	0,97	0,95	1,17 expressed protein
266556_at	At2g46230	1,41	1,25	1,45	1,05	1,10	1,07	1,05	1,10 expressed protein
266590_at	At2g46240	1,62	1,08	1,05	1,28	1,00	1,39	1,19	1,35 IQ domain-containing protein / BAG domain-containing protein
266589_at	At2g46250	1,27	1,43	1,16	0,60	0,53	1,24	1,39	1,09 myosin heavy chain-related
266580_at	At2g46260	0,99	1,06	1,19	0,88	1,06	1,06	1,01	0,91 BTB/POZ domain-containing protein
266555_at	At2g46270	0,75	0,85	1,08	0,91	0,97	1,16	1,46	1,51 G-box binding factor 3 (GBF3)
266554_s_at	At2g46280	1,20	1,15	0,95	1,15	1,04	0,91	0,96	1,01 eukaryotic translation initiation factor 3 subunit 2 / TGF-beta receptor intera
266607_at	At2g46300	1,03	0,99	0,95	1,04	1,25	0,97	1,01	1,15 expressed protein
266606_at	At2g46310	0,99	1,07	1,12	0,92	1,06	0,90	0,85	1,14 AP2 domain-containing transcription factor, putative
266552_at	At2g46330	1,03	1,24	1,40	0,89	0,96	0,88	1,06	1,22 arabinogalactan-protein (AGP16)
263779_at	At2g46340	1,10	1,31	1,63	0,84	0,78	1,17	1,28	1,48 phytochrome A supressor spa1 (SPA1)
263780_at	At2g46350	1,12	1,30	1,52	0,89	0,88	1,16	1,14	1,28 phytochrome A supressor spa1 (SPA1)
263781_at	At2g46360	0,99	1,00	0,95	1,11	1,19	0,92	0,91	1,14 expressed protein
263786_at	At2g46370	0,85	0,82	1,11	0,99	1,13	1,06	1,17	1,17 auxin-responsive GH3 family protein
263784_at	At2g46375	0,94	1,06	1,00	1,09	0,99	0,98	0,95	0,88 expressed protein
263782_at	At2g46380	1,02	1,19	1,07	0,89	1,00	1,12	1,08	1,00 hypothetical protein
263785_at	At2g46390	1,15	0,98	0,86	1,37	1,30	1,03	0,88	0,85 expressed protein
263783_at	At2g46400	1,36	1,86	1,33	1,10	0,85	1,59	1,68	1,31 WRKY family transcription factor
263775_at	At2g46410	0,95	0,84	0,97	1,03	1,41	1,09	1,11	0,98 myb-related protein CAPRICE (CPC)
263787_at	At2g46420	1,05	0,94	0,88	0,86	0,88	0,97	1,03	1,15 expressed protein
263776_s_at	At2g46440	1,03	1,00	1,49	0,86	1,08	1,01	1,06	1,53 cyclic nucleotide-regulated ion channel / cyclic nucleotide-gated channel (C
263777_at	At2g46450	1,21	1,31	1,09	0,99	1,06	1,33	1,68	1,36 cyclic nucleotide-regulated ion channel, putative (CNGC12)
263778_at	At2g46470	1,07	0,93	1,02	0,79	0,89	1,06	0,84	0,80 OXA1 protein, putative
265477_at	At2g46480	0,87	0,93	1,05	1,03	1,22	1,10	0,89	0,95 glycosyl transferase family 8 protein
265451_at	At2g46490	0,99	0,89	0,92	0,92	1,08	0,81	0,81	0,97 expressed protein (APS2)
265461_at	At2g46500	1,03	0,93	1,00	1,13	1,18	1,09	1,09	1,16 phosphatidylinositol 3- and 4-kinase family protein / ubiquitin family protein
265456_at	At2g46505	1,04	0,86	0,78	1,40	1,39	0,86	0,79	0,77 expressed protein
265452_at	At2g46510	0,79	0,90	0,75	1,04	0,80	1,05	1,04	0,94 basic helix-loop-helix (bHLH) family protein
265453_at	At2g46520	0,99	0,75	0,78	0,86	0,87	0,92	0,91	0,80 cellular apoptosis susceptibility protein, putative / importin-alpha re-exporter
265454_at	At2g46530	0,67	0,60	0,67	0,61	0,63	0,65	0,84	0,86 transcriptional factor B3 family protein / auxin-responsive factor AUX/IAA-re
265459_at	At2g46540	1,29	1,19	1,02	1,29	1,05	1,01	0,95	0,97 expressed protein
265457_at	At2g46550	0,76	1,11	1,16	0,74	0,59	0,97	1,06	1,24 expressed protein
265455_at	At2g46560	1,22	1,14	1,21	0,76	0,90	1,21	1,11	1,08 transducin family protein / WD-40 repeat family protein
265447_at	At2g46570	1,09	1,18	1,02	0,91	1,00	1,16	0,96	1,10 laccase family protein / diphenol oxidase family protein
265458_at	At2g46580	1,05	1,00	1,07	0,96	0,88	1,03	0,96	0,98 pyridoxine 5'-phosphate oxidase-related

265448_at	At2g46590	1,10	1,35	1,16	1,05	1,12	0,86	0,93	1,05	Dof zinc finger protein DAG2 / Dof affecting germination 2 (DAG2)
265460_at	At2g46600	1,17	1,41	1,18	1,08	1,36	1,13	1,11	1,21	calcium-binding protein, putative
265449_at	At2g46610	0,88	0,94	1,09	1,18	1,29	0,80	0,85	0,88	arginine/serine-rich splicing factor, putative
265450_at	At2g46620	0,89	0,94	0,93	1,06	1,23	0,99	0,98	1,21	AAA-type ATPase family protein
266325_at	At2g46630	1,06	1,00	0,68	1,05	1,17	1,01	0,88	0,81	---
266320_at	At2g46640	0,84	0,69	0,57	1,64	1,24	0,95	0,88	0,82	hypothetical protein
266326_at	At2g46650	0,87	0,84	1,23	1,03	1,19	0,70	0,83	0,67	cytochrome b5, putative
266321_at	At2g46660	0,96	0,96	0,95	0,90	1,04	1,00	1,04	0,94	cytochrome P450, putative
266327_at	At2g46680	0,96	0,97	1,14	0,94	1,24	1,46	1,46	1,74	homeobox-leucine zipper protein 7 (HB-7) / HD-ZIP transcription factor 7
266322_at	At2g46690	0,76	0,77	0,97	1,17	1,74	0,92	1,09	1,44	auxin-responsive family protein
266323_at	At2g46700	1,02	1,01	1,20	1,03	1,15	0,92	1,16	0,99	calcium-dependent protein kinase, putative / CDPK, putative
266324_at	At2g46710	1,40	2,73	1,76	0,82	0,87	1,14	1,33	1,01	rac GTPase activating protein, putative
266319_s_at	At2g46720	0,95	0,78	0,64	0,74	0,87	0,96	0,78	0,54	fatty acid elongase 3-ketoacyl-CoA synthase, putative
266739_at	At2g46730	1,05	1,06	1,00	1,01	1,00	0,96	1,03	0,97	pseudogene, similar to 68 kDa protein
266717_at	At2g46735	0,89	0,94	1,00	1,14	1,30	1,09	1,08	1,14	expressed protein
266711_at	At2g46740	0,59	0,50	0,40	0,80	0,63	1,13	1,36	1,15	FAD-binding domain-containing protein
266712_at	At2g46750	1,56	2,11	2,00	0,73	0,70	1,53	1,36	1,34	FAD-binding domain-containing protein
266713_at	At2g46760	1,11	1,07	0,93	1,06	0,93	0,91	1,00	0,84	FAD-binding domain-containing protein
266714_at	At2g46770	1,04	1,11	0,99	1,08	1,11	1,00	0,91	0,99	no apical meristem (NAM) family protein
266715_at	At2g46780	1,29	1,36	1,66	1,41	1,87	0,95	1,12	1,32	RNA recognition motif (RRM)-containing protein
266720_s_at	At2g46790	1,31	1,09	1,23	0,75	0,87	1,31	1,17	1,10	pseudo-response regulator, putative / timing of CAB expression 1-like prote
266718_at	At2g46800	1,03	1,18	1,25	1,08	0,89	0,89	0,93	0,93	zinc transporter (ZAT)
266716_at	At2g46820	0,96	0,91	0,91	0,93	0,93	1,00	1,00	1,02	expressed protein
266719_at	At2g46830	1,79	1,48	1,17	1,75	1,39	2,29	1,97	1,17	myb-related transcription factor (CCA1)
266740_at	At2g46840	0,95	0,98	1,06	1,02	1,05	1,02	0,99	1,03	expressed protein
266710_at	At2g46850	0,97	1,01	0,95	0,90	1,05	1,06	1,07	1,20	expressed protein
266765_at	At2g46860	1,04	0,99	0,91	1,03	0,95	0,96	0,97	0,87	inorganic pyrophosphatase, putative (soluble) / pyrophosphate phospho-hy
266760_at	At2g46870	1,12	0,92	0,83	0,76	0,78	1,06	1,10	0,88	DNA-binding protein, putative
266766_at	At2g46880	1,07	1,10	0,91	1,54	1,81	0,90	1,09	0,98	calcineurin-like phosphoesterase family protein
266734_at	At2g46900	0,95	1,05	1,32	0,88	0,94	1,00	0,95	1,15	expressed protein
266767_at	At2g46910	0,70	0,72	0,60	1,10	0,91	0,94	0,97	0,89	plastid-lipid associated protein PAP / fibrillin family protein
266759_at	At2g46915	0,99	1,00	1,02	0,88	1,04	1,05	0,95	1,00	expressed protein
266758_at	At2g46920	0,96	0,83	0,65	1,05	0,95	1,07	0,87	0,76	protein phosphatase 2C family protein / PP2C family protein
266735_at	At2g46930	0,99	0,90	0,86	1,20	1,17	0,83	0,86	0,83	pectinacetylesterase, putative
266757_at	At2g46940	1,01	0,92	0,86	1,09	0,99	1,08	0,98	0,93	expressed protein
266756_at	At2g46950	0,53	0,52	0,60	0,94	0,92	1,06	0,94	0,86	cytochrome P450 family protein
266736_at	At2g46960	1,02	1,02	0,89	1,02	0,89	1,13	0,97	1,03	cytochrome P450 family protein
266754_at	At2g46980	1,01	1,12	0,99	0,94	1,12	1,01	0,86	0,94	expressed protein
266753_at	At2g46990	1,08	1,01	1,06	0,96	1,02	1,04	1,13	1,06	auxin-responsive protein / indoleacetic acid-induced protein 20 (IAA20)
266752_at	At2g47000	1,44	1,13	1,03	0,72	0,82	1,49	1,31	1,33	multidrug resistant (MDR) ABC transporter, putative
266738_at	At2g47010	1,19	1,16	1,13	1,13	0,81	1,04	1,17	0,96	expressed protein
266751_at	At2g47020	0,88	1,10	0,95	0,98	1,39	0,74	0,85	1,16	peptide chain release factor, putative
266750_s_at	At2g47040	1,05	1,00	1,15	1,07	1,04	0,90	0,98	0,97	pectinesterase family protein
266764_at	At2g47050	0,93	1,01	1,03	0,96	1,13	1,06	1,04	0,81	invertase/pectin methylesterase inhibitor family protein
266749_at	At2g47060	0,67	0,63	1,06	0,73	0,71	0,91	1,16	1,54	serine/threonine protein kinase, putative
266748_at	At2g47070	0,82	0,77	0,95	0,78	0,88	0,88	0,93	0,95	squamosa promoter-binding protein-like 1 (SPL1)

266763_at	At2g47080	0,81	0,76	0,94	0,84	0,88	0,98	1,06	1,16	squamosa promoter-binding protein-like 1 (SPL1)
266768_s_at	At2g47110	1,50	1,32	1,31	1,35	1,35	0,99	0,99	0,98	ubiquitin extension protein 6 (UBQ6) / 40S ribosomal protein S27A (RPS27
266762_at	At2g47120	1,00	1,03	0,88	0,96	0,97	1,04	0,93	1,17	short-chain dehydrogenase/reductase (SDR) family protein
266761_at	At2g47130	0,88	0,83	0,88	1,20	1,20	1,02	0,93	1,10	short-chain dehydrogenase/reductase (SDR) family protein
266737_at	At2g47140	1,10	1,06	1,04	1,18	0,95	1,10	1,02	1,11	short-chain dehydrogenase/reductase (SDR) family protein
266755_at	At2g47150	1,06	0,91	0,76	1,02	0,92	1,04	0,80	0,94	short-chain dehydrogenase/reductase (SDR) family protein
263319_at	At2g47160	1,40	1,41	1,21	1,17	0,98	1,08	1,00	0,83	anion exchange family protein
263321_at	At2g47170	1,03	1,02	0,97	1,26	1,23	0,96	0,90	0,98	ADP-ribosylation factor 1 (ARF1)
263320_at	At2g47180	1,66	1,92	1,95	1,05	1,19	1,14	1,13	1,26	galactinol synthase, putative
260581_at	At2g47190	1,04	1,05	0,75	1,19	1,09	0,95	0,95	1,01	myb family transcription factor (MYB2)
260582_at	At2g47200	1,37	1,56	1,23	1,44	1,47	1,07	1,08	0,90	expressed protein
260583_x_at	At2g47220	1,08	1,20	1,00	1,08	0,77	1,08	1,00	0,99	3' exoribonuclease family domain 1 protein-related
260524_at	At2g47230	1,04	0,84	0,95	1,03	1,10	1,02	1,03	0,88	agenet domain-containing protein
260531_at	At2g47240	0,81	0,70	0,73	0,72	0,66	0,75	0,69	0,67	long-chain-fatty-acid--CoA ligase family protein / long-chain acyl-CoA synth
260525_at	At2g47250	1,00	0,97	0,72	0,87	0,95	0,96	0,90	0,87	RNA helicase, putative
260528_at	At2g47260	1,04	1,08	1,28	1,04	0,93	1,19	1,62	1,37	WRKY family transcription factor
260527_at	At2g47270	1,36	1,26	1,02	1,00	0,92	0,96	0,99	1,05	expressed protein
260573_at	At2g47280	0,94	1,08	1,42	1,01	1,06	1,08	0,91	1,25	pectinesterase family protein
260574_at	At2g47290	1,09	0,94	0,98	0,75	0,94	1,02	1,09	0,94	hypothetical protein
260575_at	At2g47300	1,03	1,19	1,22	0,83	0,82	0,99	1,04	1,04	hypothetical protein
260576_at	At2g47310	0,93	0,91	1,09	0,85	1,05	1,12	0,93	1,18	flowering time control protein-related / FCA gamma-related
260530_at	At2g47320	0,88	0,91	0,85	1,30	1,42	0,93	0,86	0,99	peptidyl-prolyl cis-trans isomerase cyclophilin-type family protein
260532_at	At2g47330	0,98	1,03	1,06	1,00	1,07	1,02	0,99	1,14	DEAD/DEAH box helicase, putative
260577_at	At2g47340	1,02	1,01	0,95	1,12	1,06	1,00	0,98	0,80	invertase/pectin methylesterase inhibitor family protein
260578_at	At2g47350	1,00	0,93	1,21	1,10	1,24	0,93	1,04	0,99	PAPA-1-like family protein / zinc finger (HIT type) family protein
260579_at	At2g47380	1,33	1,26	1,11	1,32	1,41	0,95	0,96	0,86	cytochrome c oxidase subunit Vc family protein / COX5C family protein
260580_at	At2g47390	0,67	0,60	0,61	0,74	0,86	1,10	1,06	1,06	expressed protein
260529_at	At2g47400	0,99	0,94	0,92	0,90	0,92	0,99	0,96	0,86	CP12 domain-containing protein
260526_at	At2g47410	0,81	0,85	1,09	0,76	0,91	1,09	1,12	1,44	transducin family protein / WD-40 repeat family protein
245122_at	At2g47420	0,91	0,91	0,89	0,85	0,89	0,91	0,79	0,84	dimethyladenosine transferase, putative
245125_at	At2g47430	0,98	1,09	0,94	1,06	1,04	0,99	0,95	0,92	cytokinin-responsive histidine kinase (CKI1)
245176_at	At2g47440	1,15	1,03	0,78	1,00	1,37	0,84	0,92	0,84	DNAJ heat shock N-terminal domain-containing protein
245123_at	At2g47450	1,18	1,19	1,60	0,91	0,99	0,94	0,90	0,90	chloroplast signal recognition particle component (CAO)
245126_at	At2g47460	0,92	0,94	0,98	0,94	1,05	0,98	0,93	1,02	myb family transcription factor (MYB12)
245175_at	At2g47470	1,20	1,05	1,00	1,28	1,34	0,92	0,98	1,03	thioredoxin family protein
245152_at	At2g47490	1,33	1,25	1,34	1,41	1,38	1,12	1,04	1,10	mitochondrial substrate carrier family protein
245174_at	At2g47500	1,06	1,04	0,98	1,39	1,24	0,94	0,91	1,00	kinesin motor protein-related
245173_at	At2g47520	1,04	1,14	1,13	0,92	1,44	1,16	0,88	0,91	AP2 domain-containing transcription factor, putative
245172_at	At2g47540	1,16	0,99	0,95	1,35	1,24	1,18	1,21	1,13	pollen Ole e 1 allergen and extensin family protein
245151_at	At2g47550	1,34	1,49	1,10	2,96	2,71	1,16	1,02	0,77	pectinesterase family protein
245171_at	At2g47560	1,17	1,09	0,98	1,25	1,09	0,87	1,04	0,90	zinc finger (C3HC4-type RING finger) family protein
245170_at	At2g47570	1,12	1,05	1,03	1,07	1,14	1,10	1,03	0,99	60S ribosomal protein L18 (RPL18A)
245124_at	At2g47580	1,44	1,24	1,17	1,09	1,11	1,16	1,10	1,09	small nuclear ribonucleoprotein U1A / spliceosomal protein U1A / U1snRNF
245150_at	At2g47590	0,99	0,97	0,95	1,06	0,96	0,92	0,95	0,99	photolyase/blue light photoreceptor (PHR2)
245127_at	At2g47600	0,63	0,58	0,73	0,65	0,66	0,83	0,80	0,79	magnesium/proton exchanger (MHX1)
245121_at	At2g47610	1,29	1,11	1,07	1,11	1,00	0,99	0,96	0,99	60S ribosomal protein L7A (RPL7aA)

245149_at	At2g47620	1,06	1,05	1,40	1,10	0,75	1,10	0,93	0,94	SWIRM domain-containing protein / DNA-binding family protein
266485_at	At2g47630	0,85	0,78	0,84	1,02	1,15	1,02	1,06	1,02	esterase/lipase/thioesterase family protein
266482_at	At2g47640	1,34	1,01	0,90	1,29	1,15	1,05	1,02	1,00	small nuclear ribonucleoprotein D2, putative / snRNP core protein D2, putat
251234_s_at	At2g47650	0,87	0,76	0,77	0,99	1,00	0,89	0,84	0,81	NAD-dependent epimerase/dehydratase family protein
266487_at	At2g47660	0,93	1,00	1,06	0,98	1,00	1,01	0,93	0,97	hypothetical protein
266488_at	At2g47670	0,94	0,88	0,93	0,90	1,15	1,08	0,94	0,82	invertase/pectin methylesterase inhibitor family protein
266511_at	At2g47680	0,99	0,83	0,82	1,01	0,98	0,82	0,76	0,69	zinc finger (CCCH type) helicase family protein
266512_at	At2g47690	1,30	1,06	1,12	1,25	1,20	0,98	0,92	0,94	NADH-ubiquinone oxidoreductase-related
266513_at	At2g47700	0,92	0,85	1,20	0,79	0,86	0,87	0,89	1,22	zinc finger (C3HC4-type RING finger) family protein
266458_at	At2g47710	1,17	1,13	1,02	1,31	1,32	0,88	0,94	0,91	universal stress protein (USP) family protein
266502_at	At2g47720	0,99	0,94	0,92	0,91	0,90	0,95	0,81	0,97	expressed protein
266461_at	At2g47730	0,77	0,77	0,87	1,09	1,03	0,94	0,87	0,90	glutathione S-transferase 6 (GST6)
266465_at	At2g47750	0,97	0,96	1,10	1,04	0,98	1,02	1,04	0,92	auxin-responsive GH3 family protein
266467_at	At2g47760	1,06	1,04	0,87	0,90	0,79	1,10	1,12	1,00	ALG3 family protein
266462_at	At2g47770	0,12	0,16	0,12	0,95	0,49	0,96	0,97	1,06	benzodiazepine receptor-related
266503_at	At2g47780	0,75	0,89	0,71	0,87	0,66	0,95	0,96	1,07	rubber elongation factor (REF) protein-related
266466_at	At2g47790	1,46	1,51	2,17	0,87	0,93	1,13	1,13	1,14	transducin family protein / WD-40 repeat family protein
266464_at	At2g47800	0,63	0,51	0,51	0,75	0,71	1,26	1,04	0,95	glutathione-conjugate transporter (MRP4)
257371_at	At2g47810	1,09	0,93	0,93	0,92	1,07	1,08	1,04	0,94	histone-like transcription factor (CBF/NF-Y) family protein
266504_at	At2g47820	0,97	1,03	0,99	1,08	1,00	0,94	1,08	0,86	expressed protein
266505_at	At2g47830	1,27	1,07	1,24	0,91	1,15	1,28	1,15	1,17	cation efflux family protein / metal tolerance protein, putative (MTPc1)
266463_at	At2g47840	1,01	0,83	0,76	0,99	1,00	0,83	0,75	0,73	tic20 protein-related
266506_at	At2g47850	0,88	0,90	1,05	1,11	1,26	0,95	1,09	0,85	zinc finger (CCCH-type) family protein
266507_at	At2g47860	0,91	0,95	1,02	1,11	1,19	0,95	0,99	1,27	phototropic-responsive NPH3 family protein
266516_at	At2g47880	1,05	1,13	0,81	0,87	0,65	1,06	0,90	0,95	glutaredoxin family protein
266514_at	At2g47890	0,48	0,55	0,75	0,66	0,57	1,01	1,11	1,15	zinc finger (B-box type) family protein
266515_at	At2g47900	1,06	1,05	1,09	1,00	1,00	0,85	0,96	1,11	F-box family protein / tubby family protein
266483_at	At2g47910	0,88	0,88	1,47	0,83	0,86	0,96	0,90	0,96	expressed protein
266508_at	At2g47920	1,09	1,30	1,11	1,05	1,00	0,99	0,98	1,01	kinase interacting family protein
266460_at	At2g47930	0,92	0,92	0,92	0,85	1,11	0,70	0,58	0,63	hydroxyproline-rich glycoprotein family protein
266509_at	At2g47940	0,94	1,03	0,94	0,76	0,76	1,16	1,11	1,04	DegP2 protease (DEGP2)
266486_at	At2g47950	1,04	0,91	0,92	2,11	1,33	1,16	1,27	1,47	expressed protein
266468_at	At2g47960	1,01	0,89	0,86	0,89	0,93	0,90	0,80	0,90	expressed protein
266459_at	At2g47970	0,98	0,99	0,99	1,06	1,11	1,02	0,89	0,93	NPL4 family protein
266484_at	At2g47980	0,92	0,86	1,05	0,96	0,89	0,93	0,92	1,01	expressed protein
266510_at	At2g47990	0,98	0,68	0,96	1,06	0,82	0,83	0,73	0,75	transducin family protein / WD-40 repeat family protein
265772_at	At2g48010	0,71	0,77	0,70	0,86	0,84	0,92	1,18	0,79	serine/threonine protein kinase (RFK3)
265768_at	At2g48020	0,91	0,90	0,92	0,81	0,75	1,11	1,29	1,17	sugar transporter, putative
265771_at	At2g48030	1,21	1,26	1,49	1,09	1,28	0,90	0,90	0,86	endonuclease/exonuclease/phosphatase family protein
265763_at	At2g48040	0,88	0,88	0,88	0,75	0,72	1,12	1,03	1,04	expressed protein
265764_at	At2g48050	0,67	0,84	0,80	0,94	0,75	1,09	1,05	0,77	expressed protein
265765_at	At2g48060	0,96	1,08	0,91	0,96	0,93	0,98	1,03	0,84	hypothetical protein
265773_at	At2g48070	1,03	1,00	0,96	0,77	0,79	0,98	0,94	0,92	expressed protein
265766_at	At2g48080	0,89	0,98	0,94	0,77	1,00	0,89	1,11	1,18	oxidoreductase, 2OG-Fe(II) oxygenase family protein
265769_at	At2g48090	1,09	1,19	1,08	1,10	1,02	1,17	1,26	0,97	expressed protein
265770_at	At2g48100	1,54	1,78	2,80	1,21	1,34	1,19	1,39	1,86	exonuclease family protein

265767_at	At2g48110	1,01	1,20	1,35	1,04	0,98	0,78	1,14	1,04	expressed protein
262316_at	At2g48120	0,98	1,02	1,27	0,87	0,84	1,24	1,32	1,20	pale cress protein (PAC)
262349_at	At2g48130	0,93	1,42	1,25	1,57	1,33	1,27	1,32	1,06	protease inhibitor/seed storage/lipid transfer protein (LTP) family protein
262317_at	At2g48140	1,17	1,26	1,15	1,29	1,15	1,05	1,20	1,18	protease inhibitor/seed storage/lipid transfer protein (LTP) family protein
262350_at	At2g48150	0,97	0,90	0,87	1,11	0,90	1,02	0,99	1,11	glutathione peroxidase, putative
262348_at	At2g48160	0,99	0,94	1,27	0,84	0,90	0,99	0,99	0,93	PWWP domain-containing protein
259263_at	At3g01010	0,87	1,02	0,94	0,99	1,15	1,02	0,91	0,83	UDP-glucose/GDP-mannose dehydrogenase family protein
258442_at	At3g01015	0,93	0,94	0,89	0,98	1,01	0,96	1,02	0,89	expressed protein
259262_at	At3g01020	1,04	1,00	1,02	0,95	1,08	0,99	0,98	0,88	iron-sulfur cluster assembly complex protein, putative
259321_at	At3g01040	0,94	0,81	0,71	0,96	1,17	1,05	1,03	1,10	glycosyl transferase family 8 protein
259275_at	At3g01060	1,75	1,73	1,81	1,10	1,21	1,82	1,45	1,22	expressed protein
259268_at	At3g01070	1,03	1,07	0,93	1,12	1,00	1,15	0,85	0,96	plastocyanin-like domain-containing protein
259320_at	At3g01080	0,96	0,88	0,98	1,03	1,18	1,11	1,05	1,08	WRKY family transcription factor
259319_at	At3g01090	1,09	1,18	1,30	1,09	1,18	1,11	1,07	1,06	Snf1-related protein kinase (KIN10) (SKIN10)
259318_at	At3g01100	1,09	1,14	1,04	0,97	1,06	1,20	1,18	1,01	early-responsive to dehydration protein-related / ERD protein-related
259317_at	At3g01110	1,04	1,29	1,19	0,84	1,19	0,97	1,03	0,83	early-responsive to dehydration protein-related / ERD protein-related
259279_at	At3g01120	1,21	1,12	1,13	1,56	1,42	0,98	0,95	1,01	cystathionine gamma-synthase, chloroplast / O-succinylhomoserine (Thiol)-
259267_at	At3g01130	1,36	1,25	1,17	1,41	1,19	1,01	0,91	1,00	expressed protein
259281_at	At3g01140	0,91	1,01	1,10	0,92	0,98	1,10	1,00	1,04	myb family transcription factor (MYB106)
259280_at	At3g01150	1,00	0,85	0,98	0,94	1,01	0,99	0,97	0,90	polypyrimidine tract-binding protein, putative / heterogeneous nuclear ribon
259278_at	At3g01160	1,19	1,11	1,02	0,90	0,93	1,11	1,24	1,00	expressed protein
259271_at	At3g01170	0,62	0,59	0,61	0,82	0,85	0,86	0,85	0,87	expressed protein
259316_at	At3g01175	1,16	0,98	0,89	0,95	0,99	0,92	0,97	0,82	expressed protein
259277_at	At3g01180	1,17	1,56	1,62	0,87	0,82	1,34	1,26	0,96	glycogen synthase, putative
259276_at	At3g01190	1,37	1,35	1,00	2,31	1,86	0,91	1,05	0,99	peroxidase 27 (PER27) (P27) (PRXR7)
259315_at	At3g01200	0,89	0,92	0,93	0,83	0,91	1,07	0,96	1,00	expressed protein
257519_at	At3g01210	1,04	1,12	1,41	1,23	1,04	1,03	1,33	1,35	RNA recognition motif (RRM)-containing protein
259274_at	At3g01220	1,10	0,93	1,13	1,35	1,32	1,10	0,94	0,93	homeobox-leucine zipper protein, putative / HD-ZIP transcription factor, put
259266_at	At3g01240	1,02	0,97	1,04	1,06	1,06	0,91	1,00	1,04	expressed protein
259265_at	At3g01250	1,01	0,96	1,02	0,89	1,02	1,10	0,98	0,86	expressed protein
259264_at	At3g01260	1,07	1,10	1,04	1,47	1,33	0,90	0,98	0,97	aldose 1-epimerase family protein
259269_at	At3g01270	1,04	1,02	1,03	1,00	1,24	0,86	1,03	0,97	pectate lyase family protein
259270_at	At3g01280	1,14	1,01	1,03	1,33	1,30	1,07	0,95	0,96	porin, putative
259272_at	At3g01290	1,26	1,23	1,13	0,91	0,92	1,11	0,97	1,07	band 7 family protein
259118_at	At3g01310	0,67	0,69	0,81	0,74	0,69	0,96	0,94	1,09	expressed protein
259117_at	At3g01320	1,06	1,26	1,21	0,66	0,79	1,27	1,51	1,61	paired amphipathic helix repeat-containing protein
257524_at	At3g01330	1,26	1,22	1,14	1,31	1,07	1,04	1,03	0,86	transcription factor, putative / E2F-like repressor E2L2 (E2L2)
259119_at	At3g01340	0,85	0,83	0,98	1,04	1,24	0,85	0,87	0,92	protein transport protein SEC13 family protein / WD-40 repeat family protei
259116_at	At3g01350	1,57	1,31	1,15	1,19	0,95	1,02	1,09	0,92	proton-dependent oligopeptide transport (POT) family protein
259115_at	At3g01360	1,23	0,95	0,81	1,43	1,11	0,98	0,81	0,79	expressed protein
258949_at	At3g01370	0,91	0,92	1,25	0,58	0,71	1,12	1,23	1,30	expressed protein
258951_at	At3g01380	1,05	1,12	0,95	0,86	0,84	1,14	1,19	1,05	phosphatidylinositolglycan class N (PIG-N) family protein
258958_at	At3g01390	1,56	1,00	0,94	1,39	1,30	0,94	0,95	0,85	vacuolar ATP synthase subunit G 1 (VATG1) / V-ATPase G subunit 1 (VAG
258954_at	At3g01400	0,73	0,86	1,07	1,19	1,49	0,87	0,94	1,09	armadillo/beta-catenin repeat family protein
258952_at	At3g01410	1,08	1,11	1,03	1,31	1,22	0,98	0,94	0,85	RNase H domain-containing protein
258957_at	At3g01420	1,29	1,54	1,09	0,86	0,75	2,14	2,11	1,82	pathogen-responsive alpha-dioxygenase, putative

258953_at	At3g01430	0,77	0,77	0,83	1,10	1,13	0,91	0,93	1,17 expressed protein
258956_at	At3g01440	1,27	1,24	1,39	0,64	0,58	1,76	1,47	1,11 oxygen evolving enhancer 3 (PsbQ) family protein
258955_s_at	At3g01450	1,01	0,97	0,96	1,09	1,03	0,94	0,85	0,91 expressed protein
258950_at	At3g01460	0,92	0,95	1,18	0,89	0,85	0,94	0,95	1,12 PHD finger family protein / methyl-CpG binding domain-containing protein
259165_at	At3g01470	1,13	1,05	0,96	1,01	1,17	0,86	0,87	0,83 homeobox-leucine zipper protein 5 (HAT5) / HD-ZIP protein 5 / HD-ZIP prot
259193_at	At3g01480	1,17	1,06	1,01	0,72	0,82	1,08	1,09	1,10 peptidyl-prolyl cis-trans isomerase, putative / cyclophilin, putative / rotamas
259163_at	At3g01490	1,45	1,74	1,94	0,85	0,72	1,13	1,14	1,40 protein kinase, putative
259161_at	At3g01500	1,23	1,20	1,09	1,02	0,92	1,22	1,15	1,14 carbonic anhydrase 1, chloroplast / carbonate dehydratase 1 (CA1)
259188_at	At3g01510	0,66	0,71	0,74	1,10	0,96	1,16	1,15	1,23 5'-AMP-activated protein kinase beta-1 subunit-related
259184_at	At3g01520	0,88	0,80	0,76	1,06	1,15	0,92	0,86	0,90 universal stress protein (USP) family protein
259187_at	At3g01530	0,94	1,10	0,97	0,91	1,17	1,13	1,17	1,12 myb family transcription factor (MYB57)
259194_at	At3g01540	0,90	0,82	0,99	0,84	0,90	0,98	1,00	1,05 DEAD box RNA helicase (DRH1)
259185_at	At3g01550	0,99	0,74	0,63	1,29	1,01	1,36	1,28	1,20 triose phosphate/phosphate translocator, putative
259175_at	At3g01560	0,92	1,00	1,03	1,00	0,99	1,00	0,93	1,19 proline-rich family protein
259167_at	At3g01570	0,29	0,32	0,16	0,62	0,61	1,00	0,97	1,00 glycine-rich protein / oleosin
259183_at	At3g01580	1,05	0,98	1,07	1,07	0,94	0,82	1,10	0,92 pentatricopeptide (PPR) repeat-containing protein
259186_at	At3g01590	1,25	1,26	1,43	1,38	1,46	1,09	1,18	1,08 aldose 1-epimerase family protein
259176_at	At3g01610	1,10	1,06	1,53	0,91	1,32	1,01	1,13	0,95 AAA-type ATPase family protein
257523_at	At3g01620	1,01	0,97	1,06	1,03	1,08	0,98	0,93	0,94 glycosyl transferase family 17 protein
259177_at	At3g01630	0,96	1,08	1,16	0,96	1,03	0,96	0,95	0,90 nodulin-related
259162_at	At3g01640	0,61	0,62	0,57	1,01	1,03	0,91	0,80	0,83 GHMP kinase family protein
259178_at	At3g01650	0,82	1,01	1,19	0,90	0,85	1,10	1,14	1,29 copine-related
259179_at	At3g01660	1,08	0,94	0,96	0,62	0,83	1,09	0,87	1,02 expressed protein
259166_at	At3g01670	1,43	1,18	0,84	0,82	0,80	0,89	0,85	0,81 expressed protein
259180_at	At3g01680	1,20	1,09	0,97	0,76	0,71	0,88	1,03	0,99 expressed protein
259181_at	At3g01690	0,88	1,04	1,27	0,65	0,82	0,99	1,04	1,33 expressed protein
259189_at	At3g01700	0,99	1,09	0,96	1,01	0,93	1,01	1,04	1,07 arabinogalactan-protein (AGP11)
259191_at	At3g01720	1,02	0,98	0,85	1,03	1,31	1,03	0,90	0,86 expressed protein
259195_at	At3g01730	1,02	0,93	1,18	1,00	1,09	0,95	1,11	1,18 expressed protein
259192_at	At3g01740	1,47	1,39	1,33	1,21	1,42	1,10	0,94	0,90 expressed protein
259182_at	At3g01750	0,86	0,96	1,06	1,19	1,02	0,88	1,07	1,06 ankyrin repeat family protein
259164_at	At3g01770	0,90	1,12	1,26	0,66	0,76	1,04	1,15	1,34 DNA-binding bromodomain-containing protein
259190_at	At3g01780	0,88	0,74	0,69	1,15	1,36	0,98	0,89	0,86 expressed protein
258995_at	At3g01790	1,39	1,23	1,07	1,34	1,23	1,02	1,06	1,04 ribosomal protein L13 family protein
258996_at	At3g01800	1,15	0,96	0,84	1,05	1,09	1,02	0,97	1,06 ribosome recycling factor family protein / ribosome releasing factor family p
258997_at	At3g01810	0,96	1,08	1,25	0,90	1,05	1,09	1,15	1,31 expressed protein
258998_at	At3g01820	0,73	0,68	0,62	0,92	0,93	0,94	0,91	0,81 adenylate kinase family protein
258947_at	At3g01830	0,63	1,07	1,83	0,91	1,23	1,32	1,46	1,59 calmodulin-related protein, putative
259004_at	At3g01840	1,06	0,91	1,10	1,04	1,15	1,01	0,88	1,04 protein kinase family protein
258999_at	At3g01850	1,13	1,16	1,01	0,83	0,89	0,98	1,13	1,06 ribulose-phosphate 3-epimerase, cytosolic, putative / pentose-5-phosphate
259000_at	At3g01860	1,44	1,50	1,52	1,21	1,15	0,79	0,93	0,91 expressed protein
257527_at	At3g01880	1,02	0,96	1,04	1,03	0,96	0,98	1,12	1,01 hypothetical protein
258974_at	At3g01890	1,30	1,41	1,47	0,77	0,92	1,17	1,18	1,18 SWIB complex BAF60b domain-containing protein
258973_at	At3g01900	0,98	0,90	0,91	1,01	1,26	0,91	0,93	1,01 cytochrome P450 family protein
258948_at	At3g01910	0,87	0,79	0,79	0,83	0,95	0,92	0,93	0,88 sulfite oxidase, putative
258972_at	At3g01920	0,79	0,78	0,74	0,85	0,90	1,08	1,05	0,93 yrdC family protein

259005_at	At3g01930	0,74	0,72	0,67	1,25	1,08	0,94	0,75	0,68	nodulin family protein
259001_at	At3g01960	1,65	1,70	1,89	0,82	0,62	0,91	1,03	0,91	expressed protein
258975_at	At3g01970	0,90	1,18	1,82	1,93	1,93	0,99	1,09	1,14	WRKY family transcription factor
258976_at	At3g01980	1,03	1,22	1,63	0,71	0,62	1,21	1,05	1,24	short-chain dehydrogenase/reductase (SDR) family protein
258971_at	At3g01990	0,99	1,01	1,07	0,88	1,02	0,96	1,21	0,96	ACT domain-containing protein (ACR6)
259002_at	At3g02000	0,96	1,13	0,95	0,98	1,11	0,93	1,05	0,90	glutaredoxin family protein
259003_at	At3g02010	1,05	0,93	0,93	0,81	0,74	1,05	1,19	1,03	pentatricopeptide (PPR) repeat-containing protein
258977_s_at	At3g02020	0,98	1,06	1,05	0,73	0,85	1,07	1,05	1,03	aspartate kinase, lysine-sensitive, putative
258856_at	At3g02040	2,21	2,07	1,10	1,37	1,29	1,03	1,07	0,92	glycerophosphoryl diester phosphodiesterase family protein
258860_at	At3g02050	0,64	0,59	0,55	0,91	1,02	0,93	0,93	0,80	potassium transporter (KUP3)
258861_at	At3g02060	1,11	1,13	1,47	0,65	0,49	1,21	1,23	1,13	DEAD/DEAH box helicase, putative
258855_at	At3g02070	0,55	0,53	0,72	0,81	0,93	0,96	0,86	1,03	OTU-like cysteine protease family protein
258858_at	At3g02080	1,27	1,20	1,17	1,36	1,32	0,98	0,94	1,00	40S ribosomal protein S19 (RPS19A)
258862_at	At3g02090	1,24	1,16	1,13	1,13	1,11	1,03	1,01	0,98	mitochondrial processing peptidase beta subunit, putative
258854_at	At3g02100	0,89	0,90	1,00	1,00	0,93	0,92	1,24	0,89	UDP-glucoronosyl/UDP-glucosyl transferase family protein
258857_at	At3g02110	1,09	1,15	1,18	0,98	1,06	0,85	0,78	0,84	serine carboxypeptidase S10 family protein
258859_at	At3g02120	1,43	1,21	1,09	1,38	1,70	0,81	0,84	0,70	hydroxyproline-rich glycoprotein family protein
257528_at	At3g02125	1,03	1,09	1,05	1,08	0,93	1,04	1,06	1,15	hypothetical protein
259074_at	At3g02130	0,65	0,64	0,71	0,66	0,57	0,82	0,97	1,12	leucine-rich repeat transmembrane protein kinase, putative
259076_at	At3g02140	0,90	0,79	1,03	0,98	0,99	1,08	1,11	1,10	expressed protein
259129_at	At3g02150	0,86	0,79	1,11	1,10	1,09	1,03	0,93	0,86	TCP family transcription factor, putative
259104_at	At3g02170	1,53	1,79	1,16	0,71	0,79	1,07	1,34	1,49	expressed protein
259131_at	At3g02180	0,90	0,90	1,05	1,03	1,05	0,86	0,78	0,78	expressed protein
259130_at	At3g02190	1,14	0,96	1,06	1,05	1,11	1,20	0,91	0,95	60S ribosomal protein L39 (RPL39B)
259123_at	At3g02200	1,31	1,23	1,25	1,17	1,12	1,01	0,95	1,09	proteasome family protein
259122_at	At3g02210	0,90	1,22	1,15	0,93	1,01	1,03	0,95	0,95	phytochelatin synthetase family protein / COBRA cell expansion protein CO
259121_at	At3g02220	1,45	1,24	1,32	0,99	0,98	1,25	1,05	1,02	expressed protein
259077_s_at	At3g02230	0,81	0,74	0,77	1,24	1,22	0,96	0,91	0,89	reversibly glycosylated polypeptide-1 (RGP1)
259120_at	At3g02240	1,00	0,90	0,81	1,22	0,66	1,35	0,97	1,00	expressed protein
259132_at	At3g02250	0,96	0,81	0,76	0,94	0,96	0,97	0,96	0,84	expressed protein
259128_at	At3g02260	0,68	0,72	0,69	0,58	0,77	0,93	1,02	0,96	auxin transport protein (BIG)
259127_at	At3g02270	1,03	1,06	1,00	1,00	1,05	0,99	0,96	1,11	eIF4-gamma/eIF5/eIF2-epsilon domain-containing protein
259126_at	At3g02280	1,03	0,87	1,22	0,73	0,83	1,00	1,06	1,22	flavodoxin family protein
259073_at	At3g02290	1,10	1,13	1,01	1,07	1,21	1,07	0,89	1,14	zinc finger (C3HC4-type RING finger) family protein
259125_at	At3g02300	1,09	0,98	1,04	0,96	0,75	0,92	1,04	0,91	regulator of chromosome condensation (RCC1) family protein
259124_at	At3g02310	1,01	1,02	0,88	0,94	1,01	0,94	0,96	1,00	developmental protein SEPALLATA2 / floral homeotic protein (AGL4) (SEP
259075_at	At3g02320	1,08	0,90	0,91	0,96	0,71	0,98	1,00	1,01	N2,N2-dimethylguanosine tRNA methyltransferase family protein
256325_at	At3g02330	1,18	0,99	0,92	0,82	0,93	0,90	1,03	0,95	pentatricopeptide (PPR) repeat-containing protein
256326_at	At3g02340	0,76	1,07	1,02	0,81	1,20	1,04	0,93	1,07	zinc finger (C3HC4-type RING finger) family protein
256327_at	At3g02350	0,89	0,75	0,85	1,15	1,41	0,90	0,85	0,81	glycosyl transferase family 8 protein
256328_at	At3g02360	0,99	1,05	1,04	1,16	1,22	1,02	0,99	0,84	6-phosphogluconate dehydrogenase family protein
258497_at	At3g02380	1,19	1,12	0,99	1,22	1,20	1,31	1,12	1,22	zinc finger protein CONSTANS-LIKE 2 (COL2)
258492_at	At3g02390	0,93	0,87	1,13	0,91	1,16	0,96	1,41	1,40	expressed protein
258476_at	At3g02400	0,99	0,92	0,99	1,07	1,06	1,18	1,01	1,14	forkhead-associated domain-containing protein / FHA domain-containing pr
258488_at	At3g02420	1,01	1,04	1,13	1,27	1,03	0,95	0,84	0,85	expressed protein
258479_at	At3g02440	1,00	1,03	1,08	1,09	0,99	1,07	1,00	0,97	expressed protein

258494_at	At3g02450	1,23	1,10	1,16	0,75	0,63	1,26	1,10	1,03 cell division protein ftsH, putative
258496_at	At3g02460	1,06	0,94	1,33	1,18	1,02	0,95	1,16	1,29 plant adhesion molecule, putative
258500_at	At3g02470	1,04	0,97	0,95	0,87	0,82	1,02	1,01	1,00 adenosylmethionine decarboxylase family protein
258498_at	At3g02480	0,14	0,14	0,09	0,81	0,96	1,31	1,44	1,11 ABA-responsive protein-related
258502_at	At3g02490	0,95	0,90	0,89	1,00	1,08	0,97	0,92	1,09 pentatricopeptide (PPR) repeat-containing protein
258503_at	At3g02500	1,10	1,14	1,14	1,12	1,03	1,12	0,97	0,90 expressed protein
258489_at	At3g02520	1,34	1,41	1,27	1,53	1,34	1,02	0,96	0,95 14-3-3 protein GF14 nu (GRF7)
258482_at	At3g02530	1,08	0,99	0,98	0,96	0,91	1,00	0,86	0,90 chaperonin, putative
258499_at	At3g02540	1,17	1,17	1,24	0,87	1,12	0,88	0,76	0,86 ubiquitin family protein
258487_at	At3g02550	1,33	1,21	1,20	1,41	1,74	0,39	0,49	0,67 LOB domain protein 41 / lateral organ boundaries domain protein 41 (LBD4
258493_at	At3g02555	1,17	1,23	1,31	0,99	1,18	0,88	0,75	1,08 expressed protein
258486_at	At3g02560	1,42	1,29	1,26	1,49	1,39	0,99	0,97	1,03 40S ribosomal protein S7 (RPS7B)
258483_at	At3g02570	1,05	0,81	1,08	0,96	0,99	1,07	0,87	0,90 phosphomannose isomerase type I family protein
258484_at	At3g02580	1,10	0,98	0,94	1,41	1,32	1,02	0,84	0,69 delta 7-sterol-C5-desaturase (STE1)
258504_at	At3g02590	1,04	1,01	0,91	1,12	0,95	0,98	1,05	0,87 delta 7-sterol-C5-desaturase, putative
258481_at	At3g02600	1,06	1,08	0,95	1,10	1,13	0,90	0,94	1,03 phosphatidic acid phosphatase family protein / PAP2 family protein
258473_s_at	At3g02610	0,91	0,82	0,87	1,11	1,49	0,91	1,00	0,85 acyl-(acyl-carrier-protein) desaturase, putative / stearoyl-ACP desaturase, p
258485_at	At3g02630	0,91	0,80	0,84	1,00	0,92	0,77	0,70	0,73 acyl-(acyl-carrier-protein) desaturase, putative / stearoyl-ACP desaturase, p
258480_at	At3g02640	1,83	1,69	1,34	1,93	1,65	0,83	0,74	0,66 expressed protein
258474_at	At3g02650	1,39	1,17	1,12	1,15	0,99	0,87	0,87	0,76 pentatricopeptide (PPR) repeat-containing protein
258475_at	At3g02660	1,25	1,18	1,29	0,96	0,84	1,15	1,02	0,98 tRNA synthetase class I (W and Y) family protein
258490_at	At3g02670	1,07	1,05	1,07	1,34	1,40	0,94	0,96	0,86 proline-rich family protein
258477_at	At3g02680	0,91	1,03	1,00	0,92	0,94	0,93	1,11	0,97 hypothetical protein
258495_at	At3g02690	0,90	0,97	0,99	0,65	0,57	1,22	1,21	1,23 integral membrane family protein
258491_at	At3g02700	1,21	1,16	1,26	1,20	1,19	0,90	0,86	0,95 NC domain-containing protein
258478_at	At3g02710	1,02	1,10	1,06	1,03	1,06	0,94	0,93	0,86 nuclear associated protein-related / NAP-related
258622_at	At3g02720	0,98	1,03	1,08	0,86	0,97	1,10	1,07	1,10 DJ-1 family protein / protease-related
258607_at	At3g02730	0,87	0,87	1,11	0,83	0,92	1,04	0,96	0,97 thioredoxin, putative
258615_at	At3g02740	1,03	1,07	1,02	0,92	0,87	1,07	0,94	0,85 aspartyl protease family protein
258602_at	At3g02750	0,86	0,89	1,29	0,97	1,11	0,99	0,89	0,98 protein phosphatase 2C family protein / PP2C family protein
258601_at	At3g02760	1,21	1,07	1,21	0,76	0,81	1,19	1,05	0,97 histidyl-tRNA synthetase, putative / histidine--tRNA ligase, putative
258614_at	At3g02770	0,96	0,83	0,91	1,28	1,24	0,81	0,86	0,96 dimethylmenaquinone methyltransferase family protein
258619_at	At3g02780	0,90	0,92	0,86	1,26	1,21	0,94	0,96	0,86 isopentenyl-diphosphate delta-isomerase II / isopentenyl diphosphate:dimet
258623_at	At3g02790	1,83	1,39	1,24	0,84	1,03	1,06	0,90	0,98 zinc finger (C2H2 type) family protein
257536_at	At3g02800	1,51	1,58	1,67	0,93	1,26	1,32	1,21	1,53 tyrosine specific protein phosphatase family protein
258600_at	At3g02810	0,95	1,12	1,02	0,99	1,04	0,94	0,89	0,98 protein kinase family protein
258630_at	At3g02820	1,10	1,20	1,16	1,08	1,21	0,94	0,99	1,05 zinc knuckle (CCHC-type) family protein
258621_at	At3g02830	1,23	1,45	1,97	1,11	1,14	1,24	1,58	1,54 zinc finger (CCCH-type) family protein
258606_at	At3g02840	0,86	1,15	0,99	0,93	0,84	1,98	2,17	1,79 immediate-early fungal elicitor family protein
258629_at	At3g02850	1,06	1,07	0,99	1,36	0,97	1,05	0,96	0,92 stelar K+ outward rectifier (SKOR) / potassium channel protein
258611_at	At3g02860	1,33	1,15	1,12	1,09	0,92	1,16	1,04	1,06 expressed protein
258613_at	At3g02870	1,22	1,16	1,13	1,48	1,06	1,20	1,07	1,05 inositol-1(or 4)-monophosphatase, putative / inositol monophosphatase, put
258610_at	At3g02875	0,75	0,68	0,86	1,03	1,09	0,89	0,96	1,03 IAA-amino acid hydrolase 1 (ILR1)
258616_at	At3g02880	0,77	0,67	0,75	1,07	1,12	1,09	0,95	0,97 leucine-rich repeat transmembrane protein kinase, putative
258618_at	At3g02885	0,91	1,19	1,01	1,46	1,02	1,04	0,96	0,98 gibberellin-regulated protein 5 (GASA5) / gibberellin-responsive protein 5
258628_at	At3g02890	1,24	1,37	1,63	0,74	0,75	1,27	1,45	1,30 PHD finger protein-related

258662_at	At3g02900	1,33	1,27	1,41	1,23	1,40	0,96	0,89	0,88 expressed protein
258609_at	At3g02910	0,61	0,55	0,54	0,78	1,02	0,97	1,05	1,08 expressed protein
258612_at	At3g02920	1,31	1,32	1,32	1,23	1,03	1,09	1,04	1,12 replication protein-related
258661_at	At3g02930	1,04	0,94	1,06	0,91	0,94	0,94	0,99	1,07 expressed protein
258620_at	At3g02940	1,01	0,93	1,24	0,94	1,11	0,99	1,00	1,04 myb family transcription factor (MYB107)
258624_at	At3g02950	1,04	1,01	0,98	1,07	1,06	1,14	0,96	1,03 expressed protein
258605_at	At3g02970	0,88	0,98	0,98	1,14	1,09	0,97	0,93	1,15 phosphate-responsive 1 family protein
258604_at	At3g02980	1,27	1,09	1,30	1,14	1,23	0,99	1,06	1,16 GCN5-related N-acetyltransferase (GNAT) family protein
258603_at	At3g02990	1,01	0,98	1,11	0,90	0,99	1,08	1,10	0,85 heat shock factor protein 2 (HSF2) / heat shock transcription factor 2 (HSTF
258617_at	At3g03000	0,72	0,63	0,51	1,06	0,94	0,79	0,65	0,55 calmodulin, putative
258608_at	At3g03020	0,47	0,48	0,45	0,59	0,72	0,73	0,79	0,69 expressed protein
257530_at	At3g03040	0,69	0,66	0,86	0,81	0,80	0,93	1,05	0,92 F-box family protein
258850_at	At3g03050	0,71	0,63	0,65	1,26	1,11	0,94	0,96	0,92 cellulose synthase family protein (CslD3)
258871_at	At3g03060	1,22	1,20	0,95	1,10	1,27	1,05	1,05	0,81 AAA-type ATPase family protein
258846_at	At3g03070	1,41	1,24	1,05	1,34	1,36	0,92	0,94	0,95 NADH-ubiquinone oxidoreductase-related
258870_at	At3g03080	0,89	0,93	0,93	0,97	0,98	0,99	0,93	0,99 NADP-dependent oxidoreductase, putative
258869_at	At3g03090	0,75	0,91	0,65	0,69	0,75	0,89	0,93	0,76 sugar transporter family protein
258847_at	At3g03100	1,14	0,93	0,92	1,27	1,30	0,99	0,95	1,03 NADH:ubiquinone oxidoreductase family protein
258868_at	At3g03110	0,67	0,67	0,84	0,95	1,02	0,80	0,84	0,91 exportin 1, putative
258876_at	At3g03120	1,10	1,08	1,10	1,29	1,16	0,97	0,84	0,98 ADP-ribosylation factor, putative
258867_at	At3g03130	1,07	1,12	0,87	1,20	1,12	0,98	0,95	1,05 expressed protein
258845_at	At3g03150	0,83	0,84	0,92	0,75	0,90	0,89	0,83	0,88 expressed protein
258875_at	At3g03160	1,01	0,93	0,81	1,27	1,14	0,90	0,82	0,85 expressed protein
258878_at	At3g03170	1,11	0,95	1,04	1,02	1,03	0,99	0,89	0,98 expressed protein
258866_at	At3g03180	0,81	0,93	1,14	0,81	0,97	0,90	0,89	1,05 Got1-like family protein
258851_at	At3g03190	0,98	1,35	1,27	1,10	1,10	1,08	1,37	0,85 glutathione S-transferase, putative
258865_at	At3g03200	1,05	1,08	1,05	0,99	1,21	0,94	1,30	1,01 no apical meristem (NAM) family protein
258864_at	At3g03210	0,74	1,09	0,88	0,98	0,88	1,03	1,04	1,12 expressed protein
258877_at	At3g03220	1,10	1,02	0,92	1,18	1,42	0,97	0,88	0,75 expansin, putative (EXP13)
258874_at	At3g03230	0,83	0,87	1,05	0,95	1,22	0,96	0,96	1,17 esterase/lipase/thioesterase family protein
258873_at	At3g03240	0,90	0,89	1,05	1,09	1,15	1,01	1,01	1,06 esterase/lipase/thioesterase family protein
258849_at	At3g03250	1,17	0,91	0,80	1,25	1,08	0,94	0,94	0,94 UTP--glucose-1-phosphate uridylyltransferase, putative / UDP-glucose pyro
258872_at	At3g03260	0,92	1,02	0,99	0,97	1,12	1,02	1,11	0,97 homeobox-leucine zipper family protein / lipid-binding START domain-conta
258879_at	At3g03270	0,86	0,85	0,88	1,24	1,57	0,81	0,70	0,72 universal stress protein (USP) family protein / early nodulin ENOD18 family
257529_at	At3g03290	0,93	0,99	1,02	0,94	1,04	1,04	1,01	0,95 universal stress protein (USP) family protein
258863_at	At3g03300	0,94	0,70	0,92	0,90	0,93	0,93	1,00	1,14 DEAD/DEAH box helicase carpel factory-related
258848_at	At3g03305	0,69	0,67	0,82	0,99	0,97	0,89	0,78	0,83 calcineurin-like phosphoesterase family protein
259057_at	At3g03310	0,84	0,85	0,80	1,16	1,01	1,08	1,13	1,04 lecithin:cholesterol acyltransferase family protein / LACT family protein
259053_at	At3g03320	1,02	0,84	1,02	1,41	1,44	1,14	1,07	0,85 expressed protein
259051_at	At3g03330	0,87	0,85	0,83	0,83	0,89	0,94	0,83	0,89 short-chain dehydrogenase/reductase (SDR) family protein
259055_at	At3g03340	1,12	1,10	1,41	0,92	0,95	1,31	1,30	1,84 LUC7 N_terminus domain-containing protein
259050_at	At3g03360	1,40	1,28	1,37	0,92	0,98	1,30	1,28	1,07 F-box family protein
259049_at	At3g03370	1,01	1,02	0,96	0,93	0,89	1,00	1,00	1,37 expressed protein
259048_at	At3g03380	0,89	0,97	0,85	0,81	0,82	0,94	0,91	1,08 DegP protease, putative
259047_at	At3g03390	0,91	0,86	0,87	0,77	0,77	1,04	0,98	1,04 DegP protease, putative
259046_at	At3g03400	1,02	1,13	0,92	0,97	0,92	1,02	1,02	1,03 calmodulin-related protein, putative

259045_at	At3g03410	0,93	0,85	0,98	1,00	1,09	0,94	1,13	0,99 calmodulin-related protein, putative
259056_at	At3g03420	1,30	1,13	1,34	1,11	1,18	0,90	0,98	0,92 Ku70-binding family protein
259044_at	At3g03430	0,97	0,92	0,98	1,09	1,18	0,93	0,94	1,05 polcalcin, putative / calcium-binding pollen allergen, putative
259043_at	At3g03440	0,96	1,01	1,04	0,97	0,99	1,15	0,96	1,23 armadillo/beta-catenin repeat family protein
259042_at	At3g03450	0,83	0,98	0,85	0,94	0,90	0,97	1,07	0,94 gibberellin response modulator, putative / gibberellin-responsive modulator,
259058_at	At3g03470	0,65	0,91	1,07	1,12	1,39	0,92	0,86	0,92 cytochrome P450, putative
259054_at	At3g03480	0,96	1,03	1,08	0,81	1,00	1,11	0,94	1,14 transferase family protein
259052_at	At3g03490	1,06	1,11	1,29	0,95	1,03	1,03	1,04	1,26 peroxisomal protein PEX19 family protein
259170_at	At3g03510	1,02	0,95	0,92	0,81	0,94	0,94	1,06	0,96 phototropic-responsive NPH3 family protein
259169_at	At3g03520	0,66	0,63	0,64	1,49	1,57	0,79	0,95	0,88 phosphoesterase family protein
259221_s_at	At3g03540	1,09	1,08	1,12	1,19	1,18	0,97	1,15	1,02 phosphoesterase family protein
259220_at	At3g03550	0,81	0,63	0,63	1,06	1,17	0,84	0,75	0,73 zinc finger (C3HC4-type RING finger) family protein
259219_at	At3g03560	1,07	0,82	0,83	1,24	1,11	1,02	1,01	1,12 expressed protein
259168_at	At3g03570	1,02	1,13	1,24	1,08	0,96	1,07	0,90	0,85 expressed protein
259218_at	At3g03580	1,05	0,98	1,03	0,95	0,92	1,11	0,98	0,88 pentatricopeptide (PPR) repeat-containing protein
259171_at	At3g03590	0,79	0,82	0,91	1,09	1,11	0,84	0,81	0,98 SWIB complex BAF60b domain-containing protein
259196_at	At3g03600	1,01	0,76	0,72	1,10	1,12	0,89	0,83	0,77 ribosomal protein S2, mitochondrial (RPS2)
259198_at	At3g03610	1,11	0,96	1,36	1,02	1,16	0,99	1,04	1,15 phagocytosis and cell motility protein ELMO1-related
259217_at	At3g03620	0,82	0,82	0,67	0,90	0,80	1,02	1,08	1,14 MATE efflux family protein
259172_at	At3g03630	1,27	1,01	1,23	0,56	0,87	1,02	0,92	0,92 cysteine synthase, chloroplast, putative / O-acetylserine (thiol)-lyase, putati
259173_at	At3g03640	1,36	1,15	0,98	1,88	1,18	1,01	0,99	0,97 glycosyl hydrolase family 1 protein
259223_at	At3g03660	0,92	0,96	1,18	0,99	1,04	0,89	1,10	0,94 homeobox-leucine zipper transcription factor family protein
259197_at	At3g03670	1,46	1,94	2,83	1,59	1,23	1,36	1,28	0,90 peroxidase, putative
259222_at	At3g03680	0,75	0,81	0,94	1,03	0,81	0,87	0,87	1,21 C2 domain-containing protein
259174_at	At3g03690	0,97	1,08	0,97	1,15	1,23	1,07	1,08	1,03 glycosyltransferase family 14 protein / core-2/I-branching enzyme family pro
259345_s_at	At3g03700	0,93	1,04	0,93	1,08	1,13	1,11	1,24	0,88 expressed protein
259344_at	At3g03710	0,90	0,90	0,99	0,82	0,79	1,18	1,11	0,89 polyribonucleotide nucleotidyltransferase, putative
259337_at	At3g03720	0,65	0,68	0,83	0,91	0,81	0,94	0,96	0,92 amino acid permease family protein
259341_at	At3g03740	0,90	0,87	0,85	1,13	1,28	0,97	0,92	0,96 speckle-type POZ protein-related
259336_at	At3g03750	0,76	1,06	0,90	0,99	1,26	1,06	0,89	0,88 SET domain-containing protein
259348_at	At3g03770	1,13	0,91	0,76	1,29	1,00	1,27	1,16	1,02 leucine-rich repeat transmembrane protein kinase, putative
259343_s_at	At3g03780	1,20	0,95	0,97	1,04	0,99	0,91	0,88	0,88 5-methyltetrahydropteroyltriglutamate--homocysteine methyltransferase, pu
259334_at	At3g03790	1,20	1,35	1,48	1,07	1,00	1,17	1,07	1,28 ankyrin repeat family protein / regulator of chromosome condensation (RCC
259338_at	At3g03800	1,00	1,04	1,08	1,04	1,11	0,98	1,08	1,09 syntaxin, putative (SYP131)
259333_at	At3g03810	1,10	1,13	1,06	0,95	0,90	1,04	1,05	0,88 expressed protein
259332_at	At3g03830	1,07	1,03	1,04	1,02	1,06	0,97	0,94	0,89 auxin-responsive protein, putative
259331_at	At3g03840	1,18	1,03	0,96	0,95	1,02	0,98	1,00	1,03 auxin-responsive protein, putative
259349_at	At3g03860	1,07	1,06	1,01	1,01	0,86	1,02	0,95	0,99 expressed protein
259340_at	At3g03870	0,82	0,85	1,00	0,67	0,87	0,91	1,06	1,27 expressed protein
257518_at	At3g03880	1,36	1,18	0,83	0,93	1,05	1,11	1,03	1,16 expressed protein
259342_at	At3g03890	0,80	0,74	0,69	0,85	1,09	1,07	0,95	1,00 expressed protein
259339_at	At3g03900	0,84	0,94	0,88	1,37	1,14	0,95	0,86	0,92 adenylylsulfate kinase, putative
259346_at	At3g03910	0,97	0,94	0,96	0,91	1,01	0,97	0,95	0,88 glutamate dehydrogenase, putative
259347_at	At3g03920	1,64	1,60	1,43	1,56	1,29	0,97	0,95	0,88 Gar1 RNA-binding region family protein
259335_s_at	At3g03930	1,17	0,98	0,97	0,91	1,23	1,12	0,94	0,93 protein kinase-related
258812_at	At3g03950	1,50	1,28	1,34	1,07	0,96	1,33	1,30	1,25 expressed protein

258816_at	At3g03960	1,31	1,18	1,10	1,19	1,16	1,02	0,99	1,07 chaperonin, putative
258810_at	At3g03970	0,96	0,99	1,07	1,03	1,06	1,22	0,97	0,99 expressed protein
258814_at	At3g03980	1,01	0,95	1,11	0,96	1,01	0,93	0,89	1,03 short-chain dehydrogenase/reductase (SDR) family protein
258811_at	At3g03990	0,96	1,41	1,31	1,34	1,30	1,02	1,25	1,32 esterase/lipase/thioesterase family protein
258815_at	At3g04000	0,58	0,55	0,67	0,84	0,94	0,74	0,87	0,74 short-chain dehydrogenase/reductase (SDR) family protein
258805_at	At3g04010	0,57	0,51	0,67	1,01	1,12	1,11	1,08	1,39 glycosyl hydrolase family 17 protein
258806_at	At3g04020	1,02	0,88	0,94	0,89	0,96	0,97	0,79	1,08 expressed protein
258807_at	At3g04030	1,21	1,18	1,03	0,79	1,06	1,15	0,94	1,04 myb family transcription factor
258808_at	At3g04050	0,87	0,94	1,14	1,06	1,02	0,93	1,03	0,82 pyruvate kinase, putative
258813_at	At3g04060	1,01	1,12	1,01	0,79	0,90	1,53	1,73	1,57 no apical meristem (NAM) family protein
258809_at	At3g04070	1,27	1,20	0,91	1,31	1,19	0,85	1,08	0,97 no apical meristem (NAM) family protein
258567_at	At3g04080	0,96	0,75	0,72	0,96	1,18	0,83	0,79	0,64 apyrase (APY1)
258584_at	At3g04090	1,03	0,97	1,03	1,30	1,20	0,96	0,84	0,94 major intrinsic family protein / MIP family protein
258583_at	At3g04100	0,89	1,06	1,04	1,01	0,92	1,03	1,06	0,95 MADS-box family protein
258566_at	At3g04110	0,81	0,63	0,83	1,09	1,14	1,06	1,12	1,01 glutamate receptor family protein (GLR1.1) (GLR1)
258588_s_at	At3g04120	1,17	1,01	0,99	1,55	1,33	0,96	0,95	0,91 glyceraldehyde-3-phosphate dehydrogenase, cytosolic (GAPC) / NAD-depe
258592_at	At3g04130	1,02	1,10	1,01	0,89	1,13	1,07	1,01	1,23 pentatricopeptide (PPR) repeat-containing protein
258582_at	At3g04150	0,91	1,07	1,06	0,97	0,99	1,00	0,97	1,05 germin-like protein, putative
258581_at	At3g04160	1,10	1,00	1,04	0,95	1,14	1,15	1,02	1,08 expressed protein
258580_at	At3g04170	1,01	1,05	0,92	0,96	1,08	1,02	1,10	0,98 germin-like protein, putative
258579_s_at	At3g04190	1,07	0,94	1,05	1,01	1,06	1,09	1,05	0,94 germin-like protein, putative
258578_at	At3g04200	1,00	0,95	0,94	0,96	0,85	0,90	1,23	0,94 germin-like protein, putative
258537_at	At3g04210	0,79	0,75	0,90	0,55	0,51	0,82	0,78	0,78 disease resistance protein (TIR-NBS class), putative
258577_at	At3g04220	1,04	1,03	1,10	1,19	1,05	1,38	1,14	1,10 disease resistance protein (TIR-NBS-LRR class), putative
258576_at	At3g04230	1,43	1,12	0,98	1,64	1,42	0,97	0,86	0,88 40S ribosomal protein S16 (RPS16B)
258575_at	At3g04240	0,83	0,82	1,01	0,93	1,00	1,05	1,13	1,16 O-linked N-acetyl glucosamine transferase, putative
258574_at	At3g04250	0,89	1,00	1,02	1,10	1,02	1,13	1,14	1,01 F-box family protein-related
258573_at	At3g04260	0,87	0,75	0,73	0,56	0,63	1,06	1,11	1,01 SAP domain-containing protein
258572_at	At3g04270	0,98	0,95	1,05	0,90	0,97	1,01	1,04	1,07 hypothetical protein
258590_at	At3g04280	0,99	0,97	0,97	1,01	1,08	1,06	0,94	1,01 two-component responsive regulator family protein / response regulator fan
258589_at	At3g04290	1,22	0,98	1,11	1,45	1,17	0,81	0,73	0,68 GDSL-motif lipase/hydrolase family protein
258587_at	At3g04310	0,92	1,04	1,00	0,73	0,84	0,91	0,92	0,98 expressed protein
258586_s_at	At3g04320	2,24	2,11	1,57	4,16	3,27	1,08	1,25	1,43 trypsin and protease inhibitor family protein / Kunitz family protein
258585_at	At3g04340	1,06	0,94	0,91	0,76	0,81	1,12	1,06	1,11 FtsH protease family protein
258565_at	At3g04350	0,86	1,18	1,02	0,83	0,86	0,87	0,86	0,92 expressed protein
258591_at	At3g04360	1,05	0,90	0,95	0,84	1,03	1,04	1,02	1,09 C2 domain-containing protein
258625_at	At3g04370	1,10	0,96	0,96	0,98	1,07	1,12	0,92	1,03 hypothetical protein
258597_x_at	At3g04390	0,90	0,90	0,94	0,92	1,06	1,00	1,10	0,84 xanthine dehydrogenase family protein
258569_at	At3g04400	1,30	1,26	1,13	1,21	1,15	0,99	0,97	0,98 60S ribosomal protein L23 (RPL23C)
258598_at	At3g04410	0,86	0,98	1,11	1,08	1,18	1,01	1,09	1,15 hypothetical protein
258571_at	At3g04420	1,19	1,18	1,04	1,05	0,98	1,08	1,08	1,09 no apical meristem (NAM) family protein
258626_at	At3g04450	0,87	0,93	0,91	0,69	0,89	1,05	1,11	0,95 myb family transcription factor
258627_at	At3g04460	1,71	1,78	2,10	1,03	0,97	1,16	1,29	1,34 Pex2/Pex12 N-terminal domain-containing protein
258568_at	At3g04470	0,98	0,90	0,99	1,00	1,03	0,99	0,82	0,86 expressed protein
258593_at	At3g04480	0,97	1,04	1,21	0,90	0,95	1,00	0,94	1,01 endoribonuclease L-PSP family protein
258594_at	At3g04490	0,99	0,81	0,80	0,97	0,77	1,02	0,90	0,86 exportin-related

258595_at	At3g04500	1,46	1,21	1,22	0,85	0,99	1,14	1,04	1,11	RNA recognition motif (RRM)-containing protein
258596_at	At3g04510	0,83	0,92	0,99	0,96	1,08	0,94	0,95	1,20	hypothetical protein
258599_at	At3g04520	1,02	1,02	1,21	1,37	1,36	1,00	1,02	0,96	threonine aldolase family protein
258570_at	At3g04530	1,02	0,99	1,09	1,02	1,01	1,08	0,95	0,91	phosphoenolpyruvate carboxylase kinase 2 (PPCK2)
258798_at	At3g04540	0,92	1,04	0,98	1,11	1,17	1,12	0,92	1,09	hypothetical protein
258800_at	At3g04550	0,66	0,62	0,73	0,64	0,73	0,95	0,84	0,85	expressed protein
258801_at	At3g04560	1,25	1,21	0,99	1,07	1,10	1,21	0,99	1,01	expressed protein
258795_at	At3g04570	0,87	0,88	0,79	1,19	1,15	1,03	1,00	1,28	DNA-binding protein-related
258818_at	At3g04580	0,99	0,68	0,66	1,19	0,94	1,01	1,07	0,76	ethylene receptor, putative (EIN4)
258819_at	At3g04590	0,98	0,99	1,02	0,77	0,86	0,98	0,97	0,93	DNA-binding family protein
258820_at	At3g04600	0,97	0,99	1,08	0,97	0,88	0,92	1,01	0,98	tRNA synthetase class I (W and Y) family protein
258789_at	At3g04605	0,94	1,03	0,97	0,98	0,79	1,11	0,95	1,03	Mutator-like transposase family
258790_at	At3g04610	1,13	1,06	1,19	0,86	0,92	1,02	1,03	1,14	KH domain-containing protein
258840_at	At3g04620	1,20	1,14	1,14	1,00	1,31	1,05	1,21	1,21	expressed protein
258796_at	At3g04630	1,36	1,18	1,47	1,32	1,59	1,09	1,13	1,11	expressed protein
258792_at	At3g04640	0,79	0,86	1,24	0,95	0,99	1,05	1,38	1,75	glycine-rich protein
258802_at	At3g04650	0,92	0,89	0,75	0,76	0,71	1,04	0,91	0,98	expressed protein
258841_at	At3g04660	0,89	1,10	1,00	0,93	1,09	1,11	1,03	1,06	F-box family protein
258803_at	At3g04670	0,93	1,01	0,90	0,98	0,98	1,12	1,01	1,04	WRKY family transcription factor
258842_at	At3g04680	0,93	0,94	0,91	0,82	1,00	0,94	0,91	0,76	pre-mRNA cleavage complex family protein
258843_at	At3g04690	1,04	1,04	0,91	0,99	0,96	1,03	1,01	0,98	protein kinase family protein
257532_at	At3g04700	1,00	0,99	1,08	0,93	0,96	1,14	0,97	0,99	expressed protein
258794_at	At3g04710	1,21	1,11	1,16	0,76	1,15	1,17	1,11	1,13	ankyrin repeat family protein
258791_at	At3g04720	0,95	1,05	1,09	1,09	1,17	0,83	0,86	1,04	hevein-like protein (HEL)
258797_at	At3g04730	1,48	1,55	1,65	0,79	0,91	1,21	1,20	1,53	auxin-responsive protein / indoleacetic acid-induced protein 16 (IAA16)
258844_at	At3g04740	1,07	0,98	1,08	0,81	0,94	0,94	1,06	1,03	expressed protein (SWP1)
258817_at	At3g04750	0,86	0,81	0,80	0,86	0,84	0,99	0,91	0,94	pentatricopeptide (PPR) repeat-containing protein
258804_at	At3g04760	0,93	0,93	0,94	0,68	0,60	0,97	1,01	0,96	pentatricopeptide (PPR) repeat-containing protein
258799_at	At3g04770	1,16	1,07	1,01	1,00	0,78	1,01	1,00	1,17	40S ribosomal protein SA (RPSaB)
258793_at	At3g04780	1,27	0,92	0,98	1,39	1,31	0,94	0,91	0,97	expressed protein
259098_at	At3g04790	1,06	0,97	0,95	0,91	0,87	1,13	1,05	1,01	ribose 5-phosphate isomerase-related
259084_at	At3g04800	1,00	0,98	0,96	1,07	1,11	0,82	0,94	1,12	mitochondrial import inner membrane translocase subunit Tim17/Tim22/Tim
259083_at	At3g04810	1,08	1,13	1,03	0,90	0,76	1,19	1,36	1,08	protein kinase, putative
259082_at	At3g04820	1,05	1,08	1,05	1,10	0,96	0,95	1,00	0,90	expressed protein
259097_at	At3g04830	1,10	0,93	0,90	1,03	1,05	1,04	0,85	0,96	expressed protein
259096_at	At3g04840	1,37	1,15	1,08	1,34	1,17	1,01	0,97	1,05	40S ribosomal protein S3A (RPS3aA)
259093_at	At3g04860	0,77	1,05	1,10	1,02	1,11	0,98	1,04	0,68	expressed protein
259092_at	At3g04870	0,99	0,93	1,00	1,00	1,05	1,19	1,14	1,01	zeta-carotene desaturase (ZDS1) / carotene 7,8-desaturase
259100_at	At3g04880	0,85	0,90	0,89	1,01	1,28	0,78	0,66	0,65	DNA-damage-repair/toleration protein (DRT102)
259091_at	At3g04890	0,63	0,55	0,48	0,79	0,77	0,64	0,65	0,66	expressed protein
257525_at	At3g04900	0,88	0,98	0,92	0,93	1,06	1,01	0,96	1,03	heavy-metal-associated domain-containing protein
259080_at	At3g04910	0,85	0,80	0,83	0,85	1,00	0,91	0,87	0,93	protein kinase family protein
259090_at	At3g04920	1,48	1,33	1,18	1,34	1,52	1,00	0,93	0,89	40S ribosomal protein S24 (RPS24A)
259094_at	At3g04940	0,94	0,94	1,19	0,95	0,96	0,90	1,04	1,17	cysteine synthase, putative / O-acetylserine (thiol)-lyase, putative / O-acetyl
259079_at	At3g04950	1,06	0,93	0,93	1,17	1,02	0,96	0,95	0,73	hypothetical protein
259089_at	At3g04960	0,91	0,86	1,04	1,19	0,96	0,97	1,10	0,99	expressed protein

259088_at	At3g04970	1,04	1,00	1,21	1,05	0,76	0,89	0,89	1,13	zinc finger (DHHC type) family protein
259087_at	At3g04980	1,05	1,00	1,06	1,16	0,97	0,98	0,98	1,14	DNAJ heat shock N-terminal domain-containing protein
259086_at	At3g04990	1,04	0,94	1,18	0,95	1,04	1,05	0,98	0,94	hypothetical protein
259085_at	At3g05000	0,96	1,01	1,03	1,20	1,03	0,93	0,87	0,83	transport protein particle (TRAPP) component Bet3 family protein
259099_at	At3g05010	0,94	1,08	0,99	0,96	0,99	1,04	0,96	1,00	transmembrane protein, putative
259095_at	At3g05020	1,02	0,76	0,67	1,33	1,50	0,74	0,68	0,59	acyl carrier protein 1, chloroplast (ACP-1)
259081_at	At3g05030	0,82	1,01	1,00	0,66	0,86	1,18	1,21	1,29	sodium proton exchanger, putative (NHX2)
259078_at	At3g05040	1,06	0,97	1,12	0,84	1,00	0,89	0,94	0,90	expressed protein
259309_at	At3g05050	1,08	0,88	0,95	1,04	1,08	1,06	1,12	1,11	protein kinase family protein
259311_at	At3g05060	1,34	1,23	1,23	1,11	1,12	1,22	1,15	0,97	SAR DNA-binding protein, putative
259305_at	At3g05070	1,35	1,07	0,98	1,13	1,24	1,12	1,08	1,12	expressed protein
259299_at	At3g05080	1,06	0,93	0,94	1,23	1,20	0,98	0,97	0,85	expressed protein
259313_at	At3g05090	0,87	1,01	1,06	0,89	0,91	1,09	0,98	1,24	transducin family protein / WD-40 repeat family protein
259300_at	At3g05100	0,85	0,72	0,85	0,90	1,18	0,90	0,76	0,80	expressed protein
259301_at	At3g05110	0,88	1,01	1,11	0,97	1,11	1,03	1,06	0,98	hypothetical protein
259302_at	At3g05120	0,89	1,17	1,02	0,93	0,69	0,71	0,83	0,96	expressed protein
259303_at	At3g05130	0,90	0,87	0,91	1,06	1,03	1,00	0,94	0,84	expressed protein
259350_at	At3g05140	1,02	0,96	0,99	0,96	1,02	1,00	1,12	1,00	protein kinase family protein
259351_at	At3g05150	1,01	1,09	1,02	1,02	1,07	0,74	0,78	0,77	sugar transporter family protein
259310_s_at	At3g05165	0,76	0,72	0,67	0,89	0,84	0,85	0,93	0,83	sugar transporter, putative
259352_at	At3g05170	1,01	0,80	0,94	1,07	1,30	0,99	0,83	1,27	phosphoglycerate/bisphosphoglycerate mutase family protein
259308_at	At3g05180	1,06	1,13	1,10	0,74	0,80	0,94	0,89	0,76	GDSL-motif lipase/hydrolase family protein
259353_at	At3g05190	0,95	0,96	0,89	1,12	0,91	1,05	1,09	0,77	aminotransferase class IV family protein
259312_at	At3g05200	0,69	0,79	0,80	0,69	0,86	0,83	0,98	0,93	zinc finger (C3HC4-type RING finger) family protein (ATL6)
259304_at	At3g05210	0,85	0,73	0,83	0,82	1,03	0,84	0,92	1,17	nucleotide repair protein, putative
259354_at	At3g05220	0,76	0,94	1,37	0,96	0,91	0,89	1,17	1,35	heavy-metal-associated domain-containing protein
259307_at	At3g05230	1,01	0,85	0,80	1,14	1,20	0,92	0,92	0,97	signal peptidase subunit family protein
259355_at	At3g05240	0,89	0,92	1,03	0,83	0,89	1,10	1,03	0,90	pentatricopeptide (PPR) repeat-containing protein
259356_at	At3g05250	0,77	0,74	0,96	0,95	1,03	0,96	0,69	0,78	zinc finger (C3HC4-type RING finger) family protein
259314_at	At3g05260	0,65	0,67	0,80	0,99	0,83	1,05	0,98	1,16	short-chain dehydrogenase/reductase (SDR) family protein
259322_at	At3g05270	1,12	1,24	1,12	0,92	1,16	1,18	1,12	1,14	expressed protein
259323_at	At3g05280	0,95	0,91	0,88	0,97	1,11	0,97	0,88	0,74	integral membrane Yip1 family protein
259306_at	At3g05290	1,09	0,91	1,09	0,83	0,93	0,97	0,99	0,97	mitochondrial substrate carrier family protein
259324_at	At3g05310	0,93	1,07	1,11	1,13	1,21	1,10	0,94	0,90	GTP-binding protein-related
259325_at	At3g05320	1,12	1,19	1,42	1,32	1,13	1,23	1,91	1,77	expressed protein
259294_at	At3g05330	1,09	0,83	0,88	0,95	1,00	1,07	1,08	0,94	cyclin family
259295_at	At3g05340	0,89	0,87	0,90	0,92	0,76	1,12	1,24	1,23	pentatricopeptide (PPR) repeat-containing protein
259296_at	At3g05350	0,81	0,79	0,79	1,00	0,94	1,05	1,10	1,19	aminopeptidase P, cytosolic, putative
259297_at	At3g05360	1,23	0,96	1,03	1,18	0,89	1,40	1,27	1,13	disease resistance family protein / LRR family protein
259298_at	At3g05370	0,85	0,61	0,71	0,44	0,58	1,29	1,24	0,99	disease resistance family protein
259134_at	At3g05390	0,94	0,98	1,00	1,02	1,17	1,15	1,20	0,92	expressed protein
259133_at	At3g05400	0,90	0,99	0,78	1,02	0,88	1,24	1,57	1,52	sugar transporter, putative
259160_at	At3g05410	1,17	1,16	1,37	0,93	0,84	0,94	0,85	0,78	expressed protein
259159_at	At3g05420	1,04	0,87	0,80	1,09	1,00	0,93	0,98	0,81	acyl-CoA binding family protein
259158_at	At3g05430	0,93	0,63	0,95	1,15	1,40	1,11	1,08	1,00	PWWP domain-containing protein
259157_at	At3g05440	0,94	1,06	0,91	0,98	0,84	1,03	1,02	0,84	C2 domain-containing protein

259107_at	At3g05460	1,06	0,97	0,92	0,93	1,01	1,00	1,15	1,05 sporozoite surface protein-related
259136_at	At3g05470	1,07	1,07	0,98	0,98	0,91	0,86	1,00	0,92 formin homology 2 domain-containing protein / FH2 domain-containing prot
259135_at	At3g05480	0,88	1,11	1,02	0,87	0,94	1,02	0,92	1,10 cell cycle checkpoint control protein family
259106_at	At3g05490	0,80	0,78	0,77	1,20	1,01	0,98	0,84	0,99 rapid alkalinization factor (RALF) family protein
259105_at	At3g05500	0,86	0,81	0,81	1,10	1,11	1,17	1,21	1,15 rubber elongation factor (REF) family protein
259113_at	At3g05510	1,42	1,15	1,29	0,81	1,07	1,06	1,02	0,98 phospholipid/glycerol acyltransferase family protein
259111_at	At3g05520	0,94	0,74	0,79	0,95	1,13	1,03	0,87	0,95 F-actin capping protein alpha subunit family protein
259114_at	At3g05530	1,26	1,18	1,12	1,11	1,19	0,99	0,98	1,07 26S proteasome AAA-ATPase subunit (RPT5a)
259108_at	At3g05540	1,01	0,87	1,00	1,03	1,08	0,98	0,92	1,03 translationally controlled tumor family protein
259112_at	At3g05560	1,18	1,13	1,08	1,16	1,07	0,97	1,04	1,09 60S ribosomal protein L22-2 (RPL22B)
259110_at	At3g05570	1,28	0,86	0,94	1,04	0,98	1,18	1,03	0,97 expressed protein
259109_at	At3g05580	0,75	0,83	0,80	1,23	1,07	0,97	1,05	1,34 serine/threonine protein phosphatase, putative
258900_at	At3g05590	1,41	1,21	1,10	1,08	0,96	1,09	1,00	0,98 60S ribosomal protein L18 (RPL18B)
258895_at	At3g05600	1,45	1,63	1,53	1,68	1,56	0,75	0,97	0,91 epoxide hydrolase, putative
258889_at	At3g05610	0,91	0,93	1,02	0,92	0,98	1,00	1,02	0,96 pectinesterase family protein
258888_at	At3g05620	1,14	1,27	1,00	1,12	1,29	1,02	1,03	1,11 pectinesterase family protein
258887_at	At3g05630	1,20	0,93	1,01	1,31	0,86	1,24	1,08	0,94 phospholipase D, putative (PLDP2)
258901_at	At3g05640	0,79	1,02	0,93	3,08	4,03	0,68	0,64	0,81 protein phosphatase 2C, putative / PP2C, putative
258894_at	At3g05650	1,01	1,38	1,20	1,07	1,07	1,20	0,86	0,96 disease resistance family protein
258893_at	At3g05660	1,21	1,08	1,06	0,99	1,22	1,39	0,83	0,90 disease resistance family protein
258892_at	At3g05670	0,81	0,90	1,01	0,83	0,88	0,96	1,37	1,34 PHD finger family protein
258899_at	At3g05675	1,12	1,07	1,13	0,92	1,13	0,98	1,06	1,07 expressed protein
258891_at	At3g05680	0,99	1,00	1,01	0,94	0,96	0,91	0,98	0,87 expressed protein
258890_at	At3g05690	0,91	0,66	0,78	1,15	1,09	0,79	0,78	0,87 CCAAT-binding transcription factor (CBF-B/NF-YA) family protein
258896_at	At3g05710	0,88	0,81	0,98	0,90	1,22	1,02	0,95	0,94 syntaxin, putative
258886_at	At3g05720	1,06	1,08	0,93	1,17	1,03	0,90	0,96	0,85 importin alpha-1 subunit, putative
258897_at	At3g05730	2,21	1,82	1,54	1,32	1,33	0,84	0,91	0,93 expressed protein
258898_at	At3g05740	1,03	0,99	0,88	1,05	0,99	1,01	0,94	0,98 DNA helicase (RECQI1)
258738_at	At3g05750	0,73	0,84	0,90	0,69	0,72	1,16	1,06	1,03 expressed protein
258749_at	At3g05760	1,08	0,99	1,07	0,85	0,97	1,11	1,03	1,16 expressed protein
258739_s_at	At3g05770	0,98	0,98	1,00	0,95	0,93	0,97	1,00	1,00 hypothetical protein
258740_at	At3g05780	0,98	1,00	1,00	0,98	1,14	0,93	0,99	1,13 Lon protease, putative
258741_at	At3g05790	1,25	1,03	1,29	1,01	1,12	0,95	0,95	1,26 Lon protease, putative
258742_at	At3g05800	0,49	0,48	0,64	0,56	0,70	0,69	0,84	0,84 expressed protein
258747_at	At3g05810	1,28	1,00	1,00	1,09	1,21	0,85	0,82	0,80 expressed protein
258732_at	At3g05820	0,93	0,99	1,21	0,91	1,17	0,98	1,11	0,99 beta-fructofuranosidase, putative / invertase, putative / saccharase, putative
258744_at	At3g05830	1,02	0,79	0,90	0,95	0,85	1,01	1,27	1,04 expressed protein
258743_s_at	At3g05840	1,12	1,15	1,64	1,04	1,11	1,06	1,13	1,24 shaggy-related protein kinase gamma / ASK-gamma (ASK3)
258733_at	At3g05850	0,95	1,07	1,02	0,95	1,17	1,06	0,84	0,72 Mutator-like transposase family
258734_at	At3g05860	1,08	1,04	1,09	0,95	1,12	0,98	0,95	0,99 MADS-box protein (AGL45)
258735_at	At3g05880	0,73	0,76	0,76	0,86	0,89	0,84	0,94	0,88 hydrophobic protein (RCI2A) / low temperature and salt responsive protein
258751_at	At3g05890	1,17	0,93	0,78	2,15	1,22	1,09	1,17	1,00 hydrophobic protein (RCI2B) / low temperature and salt responsive protein
258736_at	At3g05900	1,39	1,07	1,15	0,98	0,84	1,18	1,41	1,68 neurofilament protein-related
258750_at	At3g05910	0,92	0,75	0,74	1,13	1,17	0,92	0,79	0,90 pectinacetylesterase, putative
258745_at	At3g05920	1,04	0,85	0,95	1,05	1,34	1,07	1,01	1,22 heavy-metal-associated domain-containing protein
258748_at	At3g05930	0,85	1,04	0,96	0,93	0,91	1,07	0,98	0,89 germin-like protein (GLP8)

258737_at	At3g05940	0,90	0,93	1,18	0,95	1,03	1,02	0,90	1,05	expressed protein
258746_at	At3g05950	1,48	2,05	1,58	1,08	1,09	0,94	1,01	0,99	germin-like protein, putative
258561_at	At3g05960	0,96	1,07	1,03	0,99	1,12	1,02	0,94	0,85	sugar transporter, putative
258563_at	At3g05970	0,80	0,67	0,71	0,89	1,04	0,89	0,93	0,87	long-chain-fatty-acid--CoA ligase / long-chain acyl-CoA synthetase (LACS6
258562_at	At3g05980	0,91	0,91	1,00	0,99	0,90	0,92	0,94	0,90	expressed protein
258557_at	At3g05990	0,96	0,87	0,68	0,98	0,92	1,12	1,01	0,85	leucine-rich repeat family protein
258558_at	At3g06000	1,06	0,98	0,91	1,01	1,00	0,99	0,93	0,95	leucine-rich repeat family protein
258559_at	At3g06010	0,92	0,92	1,11	0,91	0,75	1,24	1,12	0,92	homeotic gene regulator, putative
258560_at	At3g06020	0,69	0,94	0,77	1,02	0,98	0,77	1,00	0,87	expressed protein
258471_at	At3g06030	1,13	1,00	0,91	1,19	1,54	0,87	0,87	0,85	NPK1-related protein kinase, putative (ANP3)
258470_at	At3g06035	0,99	1,01	0,99	1,85	1,80	0,91	0,96	0,91	expressed protein
258466_at	At3g06040	1,35	1,20	1,08	1,07	1,05	1,07	0,96	0,89	ribosomal protein L12 family protein
258469_at	At3g06050	0,85	0,83	0,86	1,11	1,22	0,91	0,88	0,92	alkyl hydroperoxide reductase/thiol specific antioxidant (AhpC/TSA)/mal alle
258467_at	At3g06060	1,13	1,00	0,94	1,12	1,07	1,01	0,93	1,01	short-chain dehydrogenase/reductase (SDR) family protein
258468_at	At3g06070	1,39	2,30	2,38	0,80	0,97	1,56	2,13	2,01	expressed protein
258472_at	At3g06080	0,95	1,22	1,53	0,61	0,56	1,11	1,19	1,48	expressed protein
256391_at	At3g06090	0,96	1,08	1,01	1,02	1,02	1,01	0,98	0,90	hypothetical protein
256398_at	At3g06100	0,80	0,97	0,93	0,96	0,89	0,88	0,97	1,00	major intrinsic family protein / MIP family protein
256397_at	At3g06110	1,18	1,34	1,40	0,94	1,04	1,09	1,14	1,23	dual specificity protein phosphatase family protein
256395_at	At3g06120	1,00	0,96	1,04	1,03	1,01	1,01	1,06	1,17	basic helix-loop-helix (bHLH) family protein
256402_at	At3g06130	0,81	0,93	0,95	0,94	1,21	0,75	0,85	1,30	heavy-metal-associated domain-containing protein
256400_at	At3g06140	1,03	1,00	0,99	0,85	1,12	0,90	0,87	0,80	zinc finger (C3HC4-type RING finger) family protein
256396_at	At3g06150	1,05	1,26	0,99	0,72	0,91	0,98	0,99	1,20	expressed protein
256418_at	At3g06160	0,92	0,91	1,06	0,87	0,93	1,06	1,27	0,93	transcriptional factor B3 family protein
256387_at	At3g06170	0,81	0,81	0,75	0,96	0,99	0,91	0,83	0,87	TMS membrane family protein / tumour differentially expressed (TDE) famil
256388_at	At3g06180	0,99	0,97	0,99	0,91	1,10	0,97	1,14	1,16	expressed protein
256403_at	At3g06190	0,97	1,08	1,19	0,91	0,94	1,05	1,04	1,20	speckle-type POZ protein-related
256401_at	At3g06200	1,29	1,66	1,87	1,05	1,00	1,14	1,05	0,79	guanylate kinase, putative
257580_at	At3g06210	0,75	0,59	0,81	0,77	0,75	0,81	1,07	1,25	expressed protein
256389_at	At3g06220	0,97	0,82	0,80	1,17	1,15	0,93	0,99	0,70	transcriptional factor B3 family protein
256390_at	At3g06230	1,05	0,94	0,92	0,91	1,01	1,06	0,81	1,05	mitogen-activated protein kinase kinase (MAPKK), putative (MKK8)
256399_at	At3g06240	0,75	0,72	0,87	1,07	1,22	0,97	0,90	0,86	F-box family protein
256404_at	At3g06250	1,00	1,10	1,37	1,08	1,13	1,06	0,97	0,85	far-red impaired responsive protein, putative
256392_at	At3g06260	1,08	1,05	0,95	0,90	1,04	0,84	1,01	0,86	galactinol synthase, putative
256393_at	At3g06280	0,95	0,98	1,08	1,06	1,03	0,99	1,02	1,03	hypothetical protein
256394_at	At3g06290	0,81	0,80	0,86	0,85	0,87	1,26	1,15	1,46	SAC3/GANP family protein
258852_at	At3g06300	1,01	0,95	0,87	1,30	1,26	0,93	0,83	0,84	oxidoreductase, 2OG-Fe(II) oxygenase family protein
258881_at	At3g06310	1,15	1,04	0,94	1,13	1,28	1,03	0,88	0,78	NADH-ubiquinone oxidoreductase 19 kDa subunit (NDUFA8) family protein
258882_at	At3g06330	1,15	1,38	1,37	0,88	0,89	1,25	1,00	0,98	zinc finger (C3HC4-type RING finger) family protein
258909_at	At3g06340	0,95	0,96	1,03	1,04	0,75	1,01	0,85	0,85	DNAJ heat shock N-terminal domain-containing protein
258908_at	At3g06350	1,11	1,01	1,01	1,00	0,94	1,03	0,98	0,86	dehydroquinate dehydratase, putative / shikimate dehydrogenase, putative
258914_at	At3g06360	1,08	1,07	1,05	1,03	1,21	1,11	0,99	1,01	arabinogalactan-protein (AGP27)
258907_at	At3g06370	1,04	1,00	1,09	0,93	1,24	0,94	0,96	0,97	sodium proton exchanger, putative (NHX3)
258906_at	At3g06380	1,18	1,75	2,09	0,78	0,64	1,05	1,10	1,43	F-box family protein / tubby family protein
258905_at	At3g06390	1,08	1,20	1,07	0,88	0,84	1,10	1,15	1,04	integral membrane family protein
258904_at	At3g06400	1,08	1,09	1,05	0,90	0,97	1,10	1,14	1,28	DNA-dependent ATPase, putative

258903_at	At3g06410	1,11	0,98	0,75	1,13	1,28	0,97	1,06	0,86 zinc finger (CCCH-type) family protein
258880_at	At3g06420	1,06	1,07	0,98	1,15	1,31	1,07	1,13	1,33 autophagy 8h (APG8h)
258853_at	At3g06440	0,70	0,61	0,69	0,87	1,15	0,82	0,75	0,81 galactosyltransferase family protein
258913_at	At3g06450	0,76	0,76	0,80	0,74	0,77	0,89	0,92	0,91 anion exchange family protein
258912_at	At3g06460	1,08	1,04	1,01	1,22	1,20	1,10	1,03	1,18 GNS1/SUR4 membrane family protein
258911_at	At3g06470	0,87	0,83	0,73	1,03	0,84	0,89	0,73	0,76 GNS1/SUR4 membrane family protein
258910_at	At3g06480	1,11	1,22	1,48	0,97	0,83	1,14	1,09	1,54 DEAD box RNA helicase, putative
258902_at	At3g06483	1,07	1,01	1,34	0,87	1,07	1,07	1,07	1,01 pyruvate dehydrogenase (lipoamide) kinase (PDHK)
258516_at	At3g06490	0,90	1,26	0,97	0,73	0,84	1,31	1,09	1,06 myb family transcription factor (MYB108)
258507_at	At3g06500	0,75	1,24	2,18	0,99	1,26	1,22	1,91	1,96 beta-fructofuranosidase, putative / invertase, putative / saccharase, putative
258512_at	At3g06510	0,90	1,14	1,30	0,76	0,71	1,33	1,39	1,11 glycosyl hydrolase family 1 protein
258506_at	At3g06520	0,84	0,82	1,04	0,67	0,92	1,18	1,06	0,93 agenet domain-containing protein
258505_at	At3g06530	1,20	0,94	0,73	0,69	0,67	0,95	1,01	0,87 BAP28-related
258536_at	At3g06540	1,05	1,20	1,15	1,23	1,19	1,03	1,07	1,33 GDP dissociation inhibitor family protein / Rab GTPase activator family prot
258564_at	At3g06560	0,99	0,93	0,84	0,95	1,08	0,97	1,01	1,06 poly (A) polymerase family protein
258518_at	At3g06570	0,86	0,93	0,95	0,98	1,08	1,18	0,76	0,88 kelch repeat-containing F-box family protein
258517_at	At3g06580	1,17	1,11	1,09	0,97	0,93	1,01	1,08	1,13 galactokinase (GAL1)
258511_at	At3g06590	1,24	1,18	1,43	0,96	1,52	0,79	0,84	0,94 expressed protein
258510_at	At3g06600	1,12	1,04	0,84	0,98	0,92	0,98	0,98	1,29 expressed protein
258514_at	At3g06610	1,30	1,31	1,12	1,09	1,16	1,01	0,97	1,00 DNA-binding enhancer protein-related
258509_at	At3g06620	1,09	0,91	1,43	0,90	0,89	0,93	1,09	1,09 protein kinase family protein
258513_at	At3g06630	0,98	0,96	1,03	1,00	1,04	1,06	1,01	0,99 protein kinase family protein
258508_at	At3g06640	0,96	0,88	0,98	1,02	1,01	0,95	0,96	0,92 protein kinase family protein
258515_at	At3g06650	1,03	0,90	0,90	0,93	0,97	0,89	0,88	0,85 ATP-citrate synthase, putative / ATP-citrate (pro-S-)-lyase, putative / citrate
258522_at	At3g06660	1,32	1,27	1,80	1,00	1,14	1,16	0,89	0,96 PAPA-1-like family protein / zinc finger (HIT type) family protein
258533_at	At3g06670	1,01	1,25	1,67	1,06	1,09	1,06	1,37	2,21 expressed protein
258521_at	At3g06680	1,65	1,43	1,24	1,38	1,42	1,08	0,99	0,92 60S ribosomal protein L29 (RPL29B)
258532_at	At3g06700	1,45	1,23	1,03	1,51	1,36	1,11	1,02	0,95 60S ribosomal protein L29 (RPL29A)
258520_at	At3g06710	0,88	0,99	1,25	0,95	0,86	1,09	0,86	1,01 expressed protein
258531_at	At3g06720	1,10	0,82	0,86	1,10	1,13	0,94	0,87	0,85 importin alpha-1 subunit, putative (IMPA1)
258534_at	At3g06730	1,07	0,87	0,86	0,84	0,78	0,90	0,88	0,89 thioredoxin family protein
258529_at	At3g06740	0,97	0,83	0,87	1,17	1,43	0,80	0,79	0,87 zinc finger (GATA type) family protein
258535_at	At3g06750	0,92	1,06	0,98	0,83	0,73	0,97	0,94	0,93 hydroxyproline-rich glycoprotein family protein
258519_at	At3g06760	0,94	0,96	1,15	0,77	1,00	1,21	1,25	1,27 drought-responsive family protein
258528_at	At3g06770	1,08	1,17	1,01	0,91	0,99	0,84	0,85	0,83 glycoside hydrolase family 28 protein / polygalacturonase (pectinase) family
258501_at	At3g06780	0,90	1,09	1,07	1,03	1,24	0,99	1,23	1,56 glycine-rich protein
258526_at	At3g06790	1,28	1,16	1,12	0,96	0,90	0,94	0,92	0,84 plastid developmental protein DAG, putative
258525_at	At3g06800	0,94	0,75	0,84	0,88	0,89	1,02	0,85	0,96 acyl-CoA dehydrogenase-related
258524_at	At3g06810	0,92	0,80	0,78	0,93	0,81	1,05	1,04	1,00 acyl-CoA dehydrogenase-related
258523_at	At3g06830	0,91	1,04	1,01	0,84	1,10	1,04	0,97	0,89 pectinesterase family protein
258530_at	At3g06840	1,00	1,17	1,14	1,26	1,33	0,96	0,80	1,05 expressed protein
258527_at	At3g06850	1,13	1,38	1,67	1,11	1,14	0,95	0,99	1,04 branched chain alpha-keto acid dehydrogenase E2 subunit (din3)
258555_at	At3g06860	0,84	0,75	0,75	1,02	0,90	0,95	0,94	0,88 fatty acid multifunctional protein (MFP2)
258543_at	At3g06870	1,01	0,98	0,99	1,03	0,99	1,10	0,79	1,15 proline-rich family protein
258551_at	At3g06890	0,50	0,59	0,49	1,27	1,39	0,77	0,84	0,83 expressed protein
258547_at	At3g06895	1,06	1,18	1,08	1,03	0,89	1,00	0,95	1,19 expressed protein

258548_at	At3g06910	0,95	0,82	0,92	1,00	0,81	1,01	0,99	1,08	Ulp1 protease family protein
258549_at	At3g06930	1,31	1,07	1,03	0,96	0,97	0,91	0,86	0,96	protein arginine N-methyltransferase family protein
258550_at	At3g06940	0,85	0,85	0,81	1,06	1,14	0,77	1,00	0,96	Mutator-like transposase family
258538_at	At3g06950	0,89	0,83	0,98	0,77	0,81	0,81	0,80	0,88	tRNA pseudouridine synthase family protein
258553_at	At3g06960	0,88	0,98	1,01	0,87	0,59	0,90	0,87	0,87	expressed protein
258539_at	At3g06970	0,99	0,95	0,98	0,90	0,94	0,96	1,11	1,03	RNA recognition motif (RRM)-containing protein
258554_at	At3g06980	1,07	1,04	1,38	0,74	0,84	1,11	1,01	1,03	DEAD/DEAH box helicase, putative
258540_at	At3g06990	0,88	1,05	0,99	1,11	0,99	0,85	0,89	1,01	DC1 domain-containing protein
258541_at	At3g07000	0,84	0,89	0,69	1,12	1,06	0,82	0,81	0,90	DC1 domain-containing protein
258552_at	At3g07010	1,72	2,09	2,52	1,09	1,41	0,89	0,83	0,93	pectate lyase family protein
258556_at	At3g07020	0,82	0,73	0,65	1,02	1,22	0,93	0,88	0,81	UDP-glucose:sterol glucosyltransferase (UGT80A2)
258542_at	At3g07030	1,38	1,41	1,76	1,14	1,13	1,04	1,07	1,22	---
258544_at	At3g07040	0,95	1,00	1,03	0,59	0,68	1,21	0,96	0,98	disease resistance protein RPM1 (CC-NBS-LRR class), putative
258545_at	At3g07050	1,36	1,15	1,05	1,14	0,85	0,89	1,01	1,11	GTP-binding family protein
258546_at	At3g07060	0,87	0,78	0,64	0,77	0,79	0,95	0,81	0,84	expressed protein
258832_at	At3g07070	1,00	1,01	0,93	1,02	1,06	1,08	0,93	1,17	protein kinase family protein
258831_at	At3g07080	0,90	0,82	0,80	1,00	0,92	0,93	0,83	0,89	membrane protein
258830_at	At3g07090	1,48	1,21	1,24	1,46	1,41	1,32	1,23	1,19	expressed protein
258829_at	At3g07100	0,84	0,84	0,88	0,80	0,92	0,96	0,95	0,90	protein transport protein Sec24, putative
258837_at	At3g07110	1,23	1,04	0,94	1,00	0,97	1,02	0,97	0,95	60S ribosomal protein L13A (RPL13aA)
258828_at	At3g07130	1,08	1,21	1,22	1,92	2,35	0,93	0,97	1,10	serine/threonine protein phosphatase family protein
258838_at	At3g07140	1,00	0,97	1,01	0,98	1,09	0,94	0,79	0,72	GPI transamidase component Gpi16 subunit family protein
258827_at	At3g07150	1,03	0,88	0,79	0,86	1,05	1,07	0,83	0,83	hypothetical protein
258826_at	At3g07160	0,91	0,88	0,77	0,93	0,91	1,03	1,04	1,08	glycosyl transferase family 48 protein
258839_at	At3g07170	1,03	0,84	0,84	1,16	1,16	1,13	1,06	0,96	sterile alpha motif (SAM) domain-containing protein
258825_at	At3g07180	0,92	0,82	0,70	0,97	0,99	1,01	0,92	0,96	GPI transamidase component PIG-S-related
258824_at	At3g07190	0,75	0,80	1,11	1,00	1,13	0,94	0,93	1,22	expressed protein
258823_at	At3g07200	0,79	0,96	1,03	0,91	1,08	1,04	1,11	1,03	zinc finger (C3HC4-type RING finger) family protein
258836_at	At3g07210	0,97	1,17	0,97	1,01	1,01	0,81	0,99	1,01	expressed protein
258821_at	At3g07230	1,37	1,17	1,51	1,26	1,57	1,04	0,91	0,91	wound-responsive protein-related
257531_at	At3g07240	0,90	1,01	1,08	0,97	0,99	1,10	0,95	1,10	nuclear transport factor 2 (NTF2) family protein / RNA recognition motif (RR
258835_at	At3g07250	0,96	0,96	0,91	0,90	0,93	1,03	0,96	1,09	nuclear transport factor 2 (NTF2) family protein / RNA recognition motif (RR
258822_s_at	At3g07260	1,29	1,27	1,49	1,11	0,91	1,04	0,97	1,13	transcriptional activator, putative
258834_at	At3g07270	1,16	1,05	1,35	1,18	1,10	1,05	1,04	1,18	GTP cyclohydrolase I
258833_at	At3g07280	0,63	0,55	0,71	0,95	0,80	1,00	1,32	1,44	expressed protein
259059_at	At3g07300	1,19	1,09	1,15	0,96	1,02	1,01	0,97	0,95	eukaryotic translation initiation factor 2B family protein / eIF-2B family prote
259017_at	At3g07310	1,14	1,76	1,66	0,76	0,84	1,03	0,99	0,91	expressed protein
259014_at	At3g07320	0,88	0,90	1,11	1,23	1,14	0,79	0,76	0,76	glycosyl hydrolase family 17 protein
259041_at	At3g07330	0,88	0,80	0,81	1,00	0,97	1,01	0,75	0,82	glycosyl transferase family 2 protein
259010_at	At3g07340	1,15	1,03	1,12	1,08	1,34	0,71	1,17	1,12	basic helix-loop-helix (bHLH) family protein
259015_at	At3g07350	0,64	0,91	0,89	0,87	0,66	0,71	1,14	1,05	expressed protein
259012_at	At3g07360	0,89	1,08	1,43	0,87	0,94	1,01	1,53	1,84	armadillo/beta-catenin repeat family protein / U-box domain-containing prot
259019_at	At3g07370	0,97	0,85	0,74	0,84	0,91	1,19	1,20	1,17	tetratricopeptide repeat (TPR)-containing protein / U-box domain-containing
259011_s_at	At3g07380	1,10	0,85	0,91	0,99	1,18	0,98	0,99	0,91	expressed protein
259018_at	At3g07390	0,88	0,67	0,59	0,77	0,76	0,89	0,69	0,69	auxin-responsive protein / auxin-induced protein (AIR12)
259060_at	At3g07400	0,78	0,79	0,87	0,90	0,86	0,91	1,04	1,00	lipase class 3 family protein

259061_at	At3g07410	0,90	0,88	0,77	1,61	1,33	0,75	0,76	0,57	Ras-related GTP-binding family protein
259022_at	At3g07420	1,12	1,26	0,89	1,11	1,22	0,92	0,96	1,11	asparaginyl-tRNA synthetase 2, cytoplasmic / asparagine-tRNA ligase 2 (S'
259013_at	At3g07430	1,05	0,96	0,91	0,91	0,96	0,93	0,78	0,73	YGGT family protein
259062_at	At3g07440	1,00	0,92	0,88	0,89	1,05	0,94	0,81	0,99	expressed protein
259063_at	At3g07450	0,93	1,13	0,86	0,93	0,91	0,95	0,99	0,94	protease inhibitor/seed storage/lipid transfer protein (LTP) family protein
259020_at	At3g07470	0,89	0,91	1,16	0,83	0,99	0,91	0,96	0,94	expressed protein
259016_at	At3g07480	1,12	1,08	0,95	1,38	1,26	0,94	0,83	0,85	expressed protein
259064_at	At3g07490	1,07	0,75	0,89	1,12	1,06	1,01	0,98	0,97	calcium-binding protein, putative
259023_at	At3g07510	1,01	1,08	0,83	1,43	1,69	0,72	0,67	0,56	expressed protein
259065_at	At3g07520	1,12	1,01	1,19	0,86	1,28	0,95	1,06	1,11	glutamate receptor family protein (GLR1.4)
259066_at	At3g07530	1,17	1,17	1,04	0,95	0,90	0,96	1,12	1,07	expressed protein
259021_at	At3g07540	1,70	1,65	1,59	0,89	0,93	0,82	0,91	0,94	formin homology 2 domain-containing protein / FH2 domain-containing prot
259067_at	At3g07550	0,72	0,79	0,83	1,03	1,02	0,86	0,87	1,07	F-box family protein (FBL12)
259068_at	At3g07560	0,99	1,03	1,04	1,06	1,11	0,96	0,92	0,94	glycine-rich protein
259243_at	At3g07565	0,96	1,17	1,51	0,95	1,10	1,07	1,09	1,35	expressed protein
259247_at	At3g07570	1,12	0,93	0,91	1,00	1,11	1,01	0,90	0,95	membrane protein, putative
259250_at	At3g07580	0,56	0,64	0,60	1,05	1,34	1,01	1,08	1,02	expressed protein
259225_at	At3g07590	2,88	2,16	2,14	1,19	1,11	1,88	1,64	1,60	small nuclear ribonucleoprotein D1, putative / snRNP core protein D1, putat
259251_at	At3g07600	1,19	1,60	1,18	1,44	1,32	1,09	1,21	0,95	heavy-metal-associated domain-containing protein
259252_at	At3g07610	0,59	0,54	0,74	0,82	0,93	0,92	0,83	0,98	transcription factor jumonji (jmjC) domain-containing protein
259254_at	At3g07630	1,10	0,96	0,96	0,82	0,85	1,15	0,97	0,95	prephenate dehydratase family protein
259253_at	At3g07640	0,94	1,08	1,23	0,74	0,84	0,97	0,98	1,21	expressed protein
259244_at	At3g07650	0,54	0,72	1,08	0,39	0,43	1,03	0,85	1,03	zinc finger (B-box type) family protein
259245_at	At3g07660	1,18	1,07	1,07	0,98	0,98	1,02	0,96	0,93	expressed protein
259258_at	At3g07670	1,01	0,98	1,04	0,78	0,83	1,38	1,32	1,19	SET domain-containing protein
259256_at	At3g07680	1,18	1,11	1,07	1,30	1,36	0,99	0,87	0,99	emp24/gp25L/p24 family protein
259255_at	At3g07690	0,84	0,75	0,90	1,02	0,78	0,97	0,87	1,02	NAD-dependent glycerol-3-phosphate dehydrogenase family protein
259226_at	At3g07700	0,92	0,89	0,90	0,98	1,01	1,07	1,14	1,21	ABC1 family protein
257520_at	At3g07710	1,03	0,90	0,95	1,10	1,06	1,01	1,18	1,08	hypothetical protein
259228_at	At3g07720	1,04	0,96	0,84	1,02	1,01	1,08	1,12	1,19	kelch repeat-containing protein
259246_s_at	At3g07730	0,98	0,99	1,08	0,97	0,95	1,01	1,14	0,98	expressed protein
259229_at	At3g07740	1,02	1,06	1,28	0,96	0,81	1,13	1,21	1,22	transcriptional adaptor (ADA2a)
259227_at	At3g07750	1,32	1,09	1,41	1,10	1,06	0,94	1,00	0,86	3' exoribonuclease family domain 1-containing protein
259257_at	At3g07760	0,98	0,93	1,04	1,06	1,24	0,90	1,02	1,01	expressed protein
259248_at	At3g07770	1,46	1,36	1,30	1,21	1,05	1,11	1,15	1,25	heat shock protein-related
259230_at	At3g07780	0,89	0,88	1,17	0,85	0,99	1,14	1,17	1,26	expressed protein
259249_at	At3g07790	0,91	0,95	1,10	0,90	0,93	0,99	0,85	0,92	DGCR14-related
259224_at	At3g07800	1,48	1,29	1,17	1,28	1,33	0,83	0,86	0,97	thymidine kinase, putative
258644_at	At3g07810	0,84	0,77	0,74	1,20	1,33	0,96	0,85	0,87	heterogeneous nuclear ribonucleoprotein, putative / hnRNP, putative
258639_at	At3g07820	0,92	0,94	1,01	1,04	1,06	1,02	0,96	0,97	polygalacturonase 3 (PGA3) / pectinase
258685_at	At3g07830	0,96	1,05	0,95	0,99	1,19	1,06	0,86	1,00	polygalacturonase, putative / pectinase, putative
258686_at	At3g07840	1,10	0,96	0,93	1,09	1,13	0,90	1,08	1,04	polygalacturonase, putative / pectinase, putative
258645_s_at	At3g07850	0,98	1,04	1,00	0,91	1,06	0,96	1,12	1,00	exopolygalacturonase / galacturan 1,4-alpha-galacturonidase / pectinase
258687_at	At3g07860	0,87	0,86	0,81	0,92	0,86	0,91	0,76	1,19	expressed protein
258647_at	At3g07870	1,02	0,96	1,11	0,96	1,24	1,04	1,20	1,26	F-box family protein
258637_at	At3g07880	1,07	1,03	1,04	0,93	0,96	1,00	0,93	0,99	Rho GDP-dissociation inhibitor family protein

258636_at	At3g07890	0,88	0,81	1,02	1,14	1,10	1,03	0,99	1,01	RabGAP/TBC domain-containing protein
258648_at	At3g07900	0,91	1,10	1,09	0,99	1,18	0,93	1,13	0,99	expressed protein
258642_at	At3g07910	1,28	1,17	1,04	1,07	1,04	1,10	0,96	0,91	expressed protein
258688_at	At3g07920	1,02	1,08	1,13	1,10	0,93	0,93	0,99	0,83	eukaryotic translation initiation factor 2 subunit 2, putative / eIF-2-beta, puta
258640_at	At3g07930	1,18	1,07	1,04	0,93	1,01	0,95	1,01	1,10	HhH-GPD base excision DNA repair family protein
258689_at	At3g07940	1,05	1,03	1,15	0,92	0,83	1,12	1,05	1,09	zinc finger and C2 domain protein, putative
258638_at	At3g07950	0,86	0,95	1,00	1,25	1,24	0,95	0,79	0,71	rhomboid protein-related
258690_at	At3g07960	0,87	0,86	0,99	0,96	0,99	1,06	0,96	1,00	phosphatidylinositol-4-phosphate 5-kinase family protein
258631_at	At3g07970	1,00	1,11	0,97	0,96	1,14	1,00	1,03	0,87	polygalacturonase, putative / pectinase, putative
258632_s_at	At3g07980	1,05	0,78	1,29	1,04	1,12	0,97	1,06	1,37	protein kinase, putative
258633_at	At3g07990	0,60	0,58	0,50	1,00	1,01	0,68	0,65	0,57	serine carboxypeptidase S10 family protein
258634_at	At3g08000	0,94	1,10	1,10	0,83	0,98	0,89	1,13	1,01	RNA-binding protein, putative
258643_at	At3g08010	1,16	1,36	2,04	0,97	1,04	1,16	1,17	1,12	expressed protein
258635_at	At3g08020	0,88	0,87	0,87	0,77	0,74	1,24	1,44	1,37	PHD finger protein-related
258641_at	At3g08030	1,10	1,11	1,31	0,96	1,02	0,93	0,84	0,89	expressed protein
258646_at	At3g08040	0,90	1,04	0,98	1,33	0,92	0,89	1,06	0,87	MATE efflux family protein
256154_at	At3g08490	1,02	1,18	1,11	1,07	0,99	1,06	0,96	0,91	hypothetical protein
256155_at	At3g08500	0,94	0,95	0,96	0,94	1,20	1,10	0,95	0,96	myb family transcription factor (MYB83)
256156_at	At3g08510	0,88	0,84	0,95	0,92	0,83	0,99	0,94	1,00	phosphoinositide-specific phospholipase C (PLC2)
258666_at	At3g08550	0,91	0,98	0,96	1,07	1,21	0,92	0,85	0,95	elongation defective 1 protein / ELD1 protein
258671_at	At3g08560	0,88	0,98	0,99	1,12	1,02	1,10	0,97	0,94	vacuolar ATP synthase subunit E, putative / V-ATPase E subunit, putative /
258672_at	At3g08570	1,09	0,87	0,85	0,96	1,09	0,92	1,04	1,14	phototropic-responsive protein, putative
258680_at	At3g08580	1,12	1,09	0,96	1,22	0,93	1,00	0,92	0,93	ADP, ATP carrier protein 1, mitochondrial / ADP/ATP translocase 1 / adenir
258679_at	At3g08590	1,35	1,20	1,26	1,30	1,42	1,03	1,04	1,07	2,3-biphosphoglycerate-independent phosphoglycerate mutase, putative / p
258676_at	At3g08600	0,85	0,76	0,72	1,11	1,02	0,93	0,76	0,81	expressed protein
258681_at	At3g08610	1,34	1,21	1,05	1,11	1,02	0,98	1,04	1,04	expressed protein
258673_at	At3g08620	1,30	0,99	1,07	0,99	0,93	1,06	1,10	1,07	KH domain-containing protein
258691_at	At3g08630	0,88	0,70	0,71	1,32	1,28	0,91	0,85	0,82	expressed protein
258692_at	At3g08640	0,84	0,66	0,72	1,06	0,97	0,82	0,75	0,67	alphavirus core protein family
258693_at	At3g08650	0,95	0,90	0,86	0,85	0,87	1,04	0,96	1,07	metal transporter family protein
258694_at	At3g08660	1,04	0,98	0,89	0,99	0,97	0,95	1,00	1,04	phototropic-responsive protein, putative
258663_at	At3g08670	1,08	0,94	0,81	1,03	1,23	0,82	0,83	0,88	expressed protein
258684_at	At3g08680	0,96	0,88	0,85	1,19	1,14	0,88	0,84	0,77	leucine-rich repeat transmembrane protein kinase, putative
258678_at	At3g08690	1,17	1,07	0,96	1,29	1,37	0,99	1,03	1,05	ubiquitin-conjugating enzyme 11 (UBC11)
258664_at	At3g08700	1,02	0,90	0,92	1,15	1,13	0,93	0,86	0,88	ubiquitin-conjugating enzyme, putative
258665_at	At3g08710	0,84	0,86	1,05	1,08	1,16	1,02	0,97	0,98	thioredoxin family protein
258682_at	At3g08720	0,85	0,90	1,19	0,85	0,84	1,21	1,28	1,57	serine/threonine protein kinase (PK19)
258677_at	At3g08730	0,83	0,77	0,95	1,11	0,97	1,15	1,19	1,16	serine/threonine protein kinase (PK1) (PK6)
258674_at	At3g08740	1,00	0,97	1,03	1,00	1,10	0,97	0,96	0,92	elongation factor P (EF-P) family protein
258667_at	At3g08750	1,07	1,04	1,12	0,99	0,85	0,98	1,01	1,17	F-box family protein
258683_at	At3g08760	0,80	1,03	0,95	0,65	0,80	0,96	1,13	1,11	protein kinase family protein
258675_at	At3g08770	2,17	2,27	1,97	2,18	2,28	0,94	0,77	0,83	lipid transfer protein 6 (LTP6)
258668_at	At3g08780	0,83	1,03	0,98	1,19	0,94	0,98	0,87	0,70	expressed protein
258669_at	At3g08800	0,85	1,06	1,02	0,96	1,20	0,85	1,11	1,02	expressed protein
258670_at	At3g08810	1,06	1,06	0,94	0,97	0,92	0,96	0,98	1,18	kelch repeat-containing F-box family protein
258991_at	At3g08820	1,06	1,00	0,93	0,83	0,94	1,07	1,08	0,89	pentatricopeptide (PPR) repeat-containing protein

258990_at	At3g08840	0,73	0,76	0,67	0,72	0,69	0,75	0,79	0,72	D-alanine--D-alanine ligase family
258994_at	At3g08850	0,87	0,86	0,87	0,95	0,78	0,94	0,95	0,97	transducin family protein / WD-40 repeat family protein
258983_at	At3g08860	0,52	0,62	0,75	1,00	1,00	1,42	1,43	0,96	alanine--glyoxylate aminotransferase, putative / beta-alanine-pyruvate amin
258982_at	At3g08870	0,91	1,02	0,96	0,89	1,00	0,98	0,93	0,97	lectin protein kinase, putative
258981_at	At3g08880	0,90	0,84	0,91	0,92	0,93	0,93	0,80	0,82	expressed protein
258988_at	At3g08890	1,29	1,32	1,75	1,05	1,06	0,74	0,90	0,80	expressed protein
258980_at	At3g08900	1,08	0,99	1,02	0,85	0,96	1,04	1,04	1,03	reversibly glycosylated polypeptide-3 (RGP3)
258986_at	At3g08910	1,10	0,96	0,78	1,09	1,13	0,98	0,84	0,86	DNAJ heat shock protein, putative
258989_at	At3g08920	0,84	0,85	0,87	0,66	0,61	0,89	0,87	0,91	rhodanese-like domain-containing protein
258992_at	At3g08930	0,91	0,96	0,83	0,94	1,06	0,85	0,83	0,87	LMBR1 integral membrane family protein
258993_at	At3g08940	1,10	1,14	1,87	0,74	0,64	0,94	0,88	1,17	chlorophyll A-B binding protein (LHCB4.2)
258987_at	At3g08950	1,19	1,23	0,97	0,83	1,05	0,91	1,02	1,02	electron transport SCO1/SenC family protein
258985_at	At3g08960	0,87	1,01	1,08	0,97	0,93	1,01	0,96	0,91	importin beta-2 subunit family protein
258984_at	At3g08970	1,13	1,18	1,04	0,97	1,12	1,23	1,06	1,12	DNAJ heat shock N-terminal domain-containing protein
259199_at	At3g08980	1,04	1,03	1,09	1,01	1,07	0,96	0,86	0,83	signal peptidase I family protein
257522_at	At3g08990	1,18	1,10	0,96	1,21	1,22	0,94	0,98	0,87	yippee family protein
259216_at	At3g09000	0,78	0,72	0,96	0,90	1,05	0,82	0,81	0,95	proline-rich family protein
259213_at	At3g09010	0,81	0,81	0,88	1,07	0,96	1,04	1,17	1,37	protein kinase family protein
259211_at	At3g09020	0,85	0,85	0,95	0,85	0,93	1,14	1,07	0,96	alpha 1,4-glycosyltransferase family protein / glycosyltransferase sugar-binc
259205_at	At3g09030	0,73	0,73	0,84	1,00	1,08	0,78	0,91	0,90	potassium channel tetramerisation domain-containing protein
259206_at	At3g09040	1,04	1,06	1,06	0,79	0,93	1,01	1,01	1,05	pentatricopeptide (PPR) repeat-containing protein
259207_at	At3g09050	1,43	1,56	2,09	0,77	0,69	1,45	1,55	1,10	expressed protein
259208_at	At3g09060	1,10	0,98	1,20	0,88	0,86	0,92	0,94	1,02	pentatricopeptide (PPR) repeat-containing protein
259200_at	At3g09070	1,05	1,05	1,01	0,95	1,39	0,96	1,01	1,05	glycine-rich protein
259201_at	At3g09080	1,06	1,02	1,10	1,02	1,12	0,97	0,98	0,90	transducin family protein / WD-40 repeat family protein
259214_at	At3g09085	1,19	1,40	1,21	1,40	1,37	0,92	1,02	1,12	expressed protein
259215_at	At3g09090	1,00	0,91	0,80	1,02	0,99	0,94	0,88	0,85	defective in exine formation protein (DEX1)
259202_at	At3g09100	0,92	0,91	1,13	0,90	0,93	1,11	1,04	1,19	mRNA capping enzyme family protein
259203_at	At3g09130	0,93	1,08	1,02	1,00	1,08	0,97	0,93	0,78	hypothetical protein
257521_at	At3g09140	0,94	1,04	1,01	1,17	1,09	1,07	0,97	1,04	expressed protein
259210_at	At3g09150	0,89	0,99	1,26	1,09	0,75	1,10	1,02	0,88	phytochromobilin:ferredoxin oxidoreductase, chloroplast / phytochromobilin
259209_at	At3g09160	0,95	0,94	0,97	0,57	0,79	1,27	1,20	1,24	RNA recognition motif (RRM)-containing protein
259204_s_at	At3g09170	0,98	1,08	1,06	1,12	1,05	1,02	0,95	1,00	Ulp1 protease family protein
259212_at	At3g09180	1,05	1,21	1,46	1,04	0,96	1,09	0,96	0,96	expressed protein
259006_at	At3g09200	1,17	1,03	0,97	0,98	0,74	0,89	0,91	0,92	60S acidic ribosomal protein P0 (RPP0B)
259038_at	At3g09210	1,11	0,93	1,06	0,75	0,78	1,17	1,07	0,92	KOW domain-containing transcription factor family protein
259036_at	At3g09220	1,27	1,48	1,10	1,12	0,86	1,59	1,68	1,55	laccase family protein / diphenol oxidase family protein
259026_at	At3g09240	1,00	1,04	1,04	1,07	1,22	0,97	1,11	1,13	protein kinase-related
259039_at	At3g09250	0,93	0,80	0,92	0,89	1,06	0,96	0,88	0,84	expressed protein
259009_at	At3g09260	0,95	0,90	0,81	1,22	1,11	1,07	1,10	0,95	glycosyl hydrolase family 1 protein
259040_at	At3g09270	1,53	1,70	1,78	1,61	1,19	1,28	1,30	1,32	glutathione S-transferase, putative
259027_at	At3g09280	0,97	1,06	1,09	0,94	1,04	1,19	0,98	0,94	expressed protein
259028_at	At3g09290	0,95	0,98	1,04	0,98	0,94	1,12	0,96	0,95	zinc finger (C2H2 type) family protein
259029_at	At3g09300	1,02	0,73	0,80	0,99	1,09	0,85	0,82	0,85	oxysterol-binding family protein
259030_at	At3g09310	0,94	0,76	0,97	1,01	0,95	0,95	0,88	1,07	expressed protein
258978_at	At3g09320	1,02	1,29	1,44	1,03	1,11	0,83	0,98	0,94	zinc finger (DHHC type) family protein

257526_s_at	At3g09330	0,93	1,16	1,04	1,07	1,01	1,25	1,01	0,95 amino acid transporter family protein
259037_at	At3g09350	1,60	1,88	1,58	1,47	1,63	1,27	1,16	1,30 armadillo/beta-catenin repeat family protein
259031_at	At3g09360	1,12	0,91	1,03	0,88	0,88	1,10	1,16	0,99 transcription factor IIB (TFIIB) family protein
259007_at	At3g09370	1,19	1,11	1,00	1,00	1,14	1,28	1,02	1,29 myb family transcription factor (MYB3R3)
259032_at	At3g09380	0,98	1,10	1,06	1,05	1,08	1,06	0,98	0,86 hypothetical protein
259008_at	At3g09390	0,68	0,67	0,66	0,79	0,83	1,01	0,92	0,85 metallothionein protein, putative (MT2A)
259033_at	At3g09410	1,19	0,90	1,22	1,08	1,10	0,93	1,07	1,17 pectinacetylesterase family protein
259034_at	At3g09420	1,21	1,72	1,82	0,81	0,66	1,11	1,57	2,10 pectinacetylesterase family protein
259035_at	At3g09430	0,86	0,87	1,01	0,93	1,01	1,02	0,96	0,91 hypothetical protein
258979_at	At3g09440	1,12	1,23	1,53	1,59	1,56	1,44	1,35	1,35 heat shock cognate 70 kDa protein 3 (HSC70-3) (HSP70-3)
258699_at	At3g09460	1,17	1,16	0,90	1,23	1,26	0,92	0,91	0,82 expressed protein
258705_at	At3g09470	0,58	0,61	0,59	0,73	0,72	0,84	0,75	0,73 expressed protein
258707_at	At3g09480	0,77	0,90	1,01	1,04	0,99	1,24	0,99	0,85 histone H2B, putative
257535_at	At3g09490	0,93	0,97	0,94	1,20	1,40	1,08	1,00	1,06 chloroplast lumen common family protein
258709_at	At3g09500	1,44	1,25	1,26	1,18	1,17	1,03	1,01	0,96 60S ribosomal protein L35 (RPL35A)
258752_at	At3g09520	1,02	0,98	0,93	0,85	0,94	1,14	1,16	1,32 exocyst subunit EXO70 family protein
258753_at	At3g09530	0,93	0,99	1,06	0,90	0,96	1,01	0,98	1,03 exocyst subunit EXO70 family protein
258719_at	At3g09540	0,80	0,72	0,60	1,24	1,17	0,79	0,80	0,71 pectate lyase family protein
258720_at	At3g09550	0,89	0,89	0,81	0,92	1,03	0,97	0,96	0,86 ankyrin repeat family protein
258721_at	At3g09560	1,11	1,17	1,36	0,93	0,75	1,27	1,26	1,26 lipin family protein
258706_at	At3g09570	0,93	0,94	0,89	1,10	1,12	0,94	0,87	0,77 expressed protein
258708_at	At3g09580	1,07	0,89	1,10	0,76	0,78	0,99	0,89	0,80 amine oxidase family protein
258722_at	At3g09590	0,86	1,03	0,84	0,98	1,00	0,95	0,91	0,92 pathogenesis-related protein, putative
258723_at	At3g09600	1,35	1,24	1,46	1,21	1,46	1,48	1,02	0,91 myb family transcription factor
258724_at	At3g09610	1,11	1,10	1,12	1,01	1,02	1,11	1,10	0,92 myb family transcription factor
258725_at	At3g09620	0,94	1,04	0,99	1,05	1,10	1,16	0,92	0,95 DEAD/DEAH box helicase, putative
258715_at	At3g09630	1,38	1,22	1,14	1,33	0,98	0,98	0,95	0,90 60S ribosomal protein L4/L1 (RPL4A)
258695_at	At3g09640	1,11	0,75	1,03	0,98	1,11	1,45	1,06	1,06 L-ascorbate peroxidase 1b (APX1b)
258696_at	At3g09650	0,75	0,83	0,77	0,64	0,68	1,23	1,05	0,91 pentatricopeptide (PPR) repeat-containing protein
258697_at	At3g09660	0,98	1,07	1,09	0,96	1,05	0,92	0,98	1,22 minichromosome maintenance family protein / MCM family protein
257534_at	At3g09670	1,12	1,02	1,07	0,95	1,00	1,11	0,93	0,75 PWWP domain-containing protein
258712_s_at	At3g09680	1,16	1,04	0,97	1,18	1,10	0,98	0,92	0,90 40S ribosomal protein S23 (RPS23A)
258698_at	At3g09690	0,90	0,82	0,97	1,06	1,18	0,86	0,77	0,89 hydrolase, alpha/beta fold family protein
258716_at	At3g09700	1,22	1,04	0,89	1,13	0,91	1,07	0,96	0,87 DNAJ heat shock N-terminal domain-containing protein
258700_at	At3g09710	0,86	0,81	0,96	1,14	1,17	0,87	0,90	1,00 calmodulin-binding family protein
258701_at	At3g09720	0,96	1,13	0,97	1,03	0,86	0,89	0,88	1,06 DEAD/DEAH box helicase, putative
258702_at	At3g09730	1,04	1,08	0,84	1,11	1,10	1,05	0,95	0,95 expressed protein
258713_at	At3g09735	1,14	1,01	0,97	1,08	1,18	1,01	0,96	0,93 DNA-binding S1FA family protein
258717_at	At3g09740	0,86	0,77	0,80	1,13	1,24	0,83	0,84	0,89 syntaxin 71 (SYP71)
258703_at	At3g09750	0,95	1,00	0,98	1,10	1,19	0,99	1,04	0,83 hypothetical protein
258718_at	At3g09760	1,03	1,12	1,10	0,81	0,68	0,96	1,13	0,96 zinc finger (C3HC4-type RING finger) family protein
258714_at	At3g09770	1,13	1,36	1,40	1,15	1,14	1,00	0,86	1,11 zinc finger (C3HC4-type RING finger) family protein
258704_at	At3g09780	0,71	0,77	0,90	0,90	0,87	0,98	0,90	0,86 protein kinase family protein
258711_at	At3g09790	0,98	0,97	1,04	0,96	0,97	1,01	1,02	1,03 polyubiquitin (UBQ8)
258710_s_at	At3g09800	1,17	0,93	0,78	1,38	1,26	1,06	0,90	0,81 clathrin adaptor complex small chain family protein
258655_at	At3g09805	1,33	1,20	0,83	1,37	1,21	1,12	1,04	0,91 isocitrate dehydrogenase, putative / NAD+ isocitrate dehydrogenase, putati

258658_at	At3g09820	1,23	1,06	1,01	1,63	1,38	0,88	0,88	0,94 adenosine kinase 1 (ADK1) / adenosine 5'-phosphotransferase 1
258650_at	At3g09830	0,65	0,74	1,09	1,10	0,94	0,99	1,26	1,55 protein kinase, putative
258649_at	At3g09840	1,00	1,04	1,14	1,12	1,18	0,92	0,95	0,99 cell division cycle protein 48 (CDC48A) (CDC48)
258660_at	At3g09850	0,85	0,94	1,00	0,97	1,12	0,89	0,87	0,90 D111/G-patch domain-containing protein
258654_at	At3g09860	1,11	1,04	1,03	1,05	0,99	0,99	0,95	1,05 expressed protein
258653_at	At3g09870	1,68	1,46	1,07	0,87	1,01	1,44	1,27	0,92 auxin-responsive family protein
258659_at	At3g09880	1,03	0,91	1,05	0,95	0,91	0,92	0,91	0,96 serine/threonine protein phosphatase 2A (PP2A) regulatory subunit B' (B'be
258657_at	At3g09890	1,23	1,01	0,95	1,31	0,86	0,99	0,91	0,75 ankyrin repeat family protein
258656_at	At3g09900	0,98	1,14	1,37	1,64	1,55	1,02	0,92	0,93 Ras-related GTP-binding protein, putative
258652_at	At3g09910	0,84	0,68	0,66	0,98	1,06	0,81	0,96	0,77 Ras-related GTP-binding protein, putative
258651_at	At3g09920	0,82	0,85	0,86	0,87	0,93	0,97	1,00	1,18 phosphatidylinositol-4-phosphate 5-kinase family protein
258940_at	At3g09930	1,00	0,98	1,02	1,02	0,98	0,96	1,03	1,04 GDSL-motif lipase/hydrolase family protein
258941_at	At3g09940	0,84	0,78	0,84	1,36	1,23	0,86	1,11	1,25 monodehydroascorbate reductase, putative
258942_at	At3g09960	0,93	1,05	0,87	0,86	1,19	0,91	0,98	1,22 calcineurin-like phosphoesterase family protein
258883_at	At3g09970	0,95	0,87	0,78	0,77	0,92	1,20	0,88	0,90 calcineurin-like phosphoesterase family protein
258933_at	At3g09980	1,20	1,19	1,49	0,86	0,86	1,17	1,33	1,35 expressed protein
258931_at	At3g10010	0,88	0,79	1,16	1,00	0,80	1,14	1,04	1,16 HhH-GPD base excision DNA repair family protein
258939_at	At3g10020	0,99	1,11	1,49	0,86	1,20	0,77	0,94	1,16 expressed protein
258885_at	At3g10030	0,84	0,90	0,94	0,84	0,90	0,91	0,93	1,14 aspartate/glutamate/uridylate kinase family protein
258930_at	At3g10040	1,34	0,97	1,19	1,70	1,59	0,40	0,51	1,00 expressed protein
258884_at	At3g10050	1,15	0,91	0,98	0,83	0,80	0,99	0,92	0,80 threonine ammonia-lyase / threonine dehydratase / threonine deaminase (
258929_at	At3g10060	0,76	0,69	0,66	1,04	0,98	1,00	0,90	0,93 immunophilin, putative / FKBP-type peptidyl-prolyl cis-trans isomerase, puta
258928_at	At3g10070	0,94	1,01	0,96	0,73	0,83	0,95	0,99	0,94 transcription initiation factor IID (TFIID) subunit A family protein
258938_at	At3g10080	1,89	1,52	1,65	1,90	1,94	0,73	0,66	0,51 germin-like protein, putative
258937_at	At3g10090	1,55	1,39	1,26	1,32	1,42	1,03	0,92	0,90 40S ribosomal protein S28 (RPS28A)
258936_s_at	At3g10100	0,89	1,04	1,08	0,95	1,02	1,03	0,97	0,93 filament protein-related
258935_at	At3g10120	1,05	1,04	0,95	1,00	1,22	1,10	1,30	0,95 expressed protein
258934_at	At3g10140	1,07	1,14	1,53	0,91	0,86	1,06	1,07	1,12 recA family protein
258932_at	At3g10150	0,93	0,93	1,14	1,05	0,96	1,09	0,97	1,10 calcineurin-like phosphoesterase family protein
258927_at	At3g10160	1,12	1,19	0,99	0,93	0,92	1,10	1,22	1,23 dihydrofolate synthetase/folylpolyglutamate synthetase (DHFS/FPGS3)
259145_at	At3g10170	1,06	0,97	1,06	1,24	1,08	0,94	1,06	0,90 kinesin motor protein-related
259144_at	At3g10180	1,09	1,00	1,14	1,04	1,06	0,97	1,07	0,80 kinesin motor protein-related
259143_at	At3g10190	0,94	1,00	0,83	1,11	1,07	0,88	0,88	0,79 calmodulin, putative
259142_at	At3g10200	0,88	0,84	1,03	0,86	0,79	0,92	1,06	0,84 dehydration-responsive protein-related
259152_at	At3g10210	1,07	1,03	1,28	1,16	1,05	0,88	0,79	0,81 expressed protein
259141_at	At3g10220	1,05	1,03	1,00	0,96	0,93	0,95	1,02	1,01 tubulin folding cofactor B
259140_at	At3g10230	0,97	1,12	1,22	0,90	0,77	1,02	1,06	1,07 lycopene beta cyclase (LYC)
259139_at	At3g10240	1,00	1,07	1,05	0,97	1,02	1,06	1,11	1,11 F-box protein-related
259153_at	At3g10250	1,15	1,15	1,49	0,90	1,02	1,06	1,16	1,13 expressed protein
259154_at	At3g10260	0,37	0,32	0,31	0,91	0,89	0,56	0,52	0,53 reticulon family protein
259138_s_at	At3g10270	1,11	1,04	1,04	0,83	0,75	1,09	1,07	1,04 DNA topoisomerase, ATP-hydrolyzing, putative / DNA topoisomerase II, pu
259137_at	At3g10300	0,70	0,58	0,68	0,97	0,72	1,02	1,06	1,07 calcium-binding EF hand family protein
259151_at	At3g10310	1,13	0,96	1,01	1,57	1,28	0,92	1,02	1,18 kinesin motor protein-related
259150_at	At3g10320	0,99	0,96	1,03	1,12	1,15	1,10	1,07	0,98 expressed protein
259155_at	At3g10330	0,96	0,89	1,00	1,04	0,98	0,97	0,93	0,86 transcription initiation factor IIB-2 / general transcription factor TFIIB-2 (TFII
259149_at	At3g10340	1,47	1,43	1,11	1,51	1,31	1,28	1,37	1,35 phenylalanine ammonia-lyase, putative

259148_at	At3g10350	1,14	1,17	1,10	0,80	0,87	1,27	1,25	1,22	anion-transporting ATPase family protein
259147_at	At3g10360	0,92	1,17	1,27	0,84	1,25	0,93	0,92	1,01	pumilio/Puf RNA-binding domain-containing protein
259146_at	At3g10370	1,01	0,76	0,79	0,85	1,09	0,97	0,95	0,89	glycerol-3-phosphate dehydrogenase, putative
259156_at	At3g10380	0,94	0,77	0,86	1,07	1,06	1,04	0,93	0,92	exocyst complex component-related
258944_at	At3g10390	0,87	1,01	0,94	0,83	0,94	1,01	0,93	1,00	amine oxidase family protein / SWIRM domain-containing protein
258943_at	At3g10400	0,87	1,19	0,92	0,93	1,05	0,99	0,98	1,04	RNA recognition motif (RRM)-containing protein
258970_at	At3g10410	0,77	0,69	0,72	1,00	1,02	0,88	0,94	1,08	serine carboxypeptidase III, putative
258925_at	At3g10420	1,34	1,61	1,20	1,08	0,88	1,10	1,20	1,43	sporulation protein-related
258923_at	At3g10450	1,10	0,86	0,94	0,92	0,84	1,32	1,44	1,30	serine carboxypeptidase S10 family protein
258968_at	At3g10460	1,00	0,94	0,97	0,87	0,97	1,04	0,96	1,04	self-incompatibility protein-related
258967_at	At3g10470	1,00	1,04	0,82	0,92	0,98	1,14	1,01	1,00	zinc finger (C2H2 type) family protein
258926_s_at	At3g10490	1,03	0,99	0,89	0,84	0,91	1,00	0,97	0,97	no apical meristem (NAM) family protein
258921_at	At3g10500	1,53	1,70	1,79	0,66	0,76	1,28	2,05	2,20	no apical meristem (NAM) family protein
258920_at	At3g10520	1,76	1,66	1,56	0,57	0,73	1,07	0,95	0,96	non-symbiotic hemoglobin 2 (HB2) (GLB2)
258919_at	At3g10525	0,80	0,75	1,01	1,05	0,96	1,33	1,20	0,99	expressed protein
258965_at	At3g10530	1,11	0,98	0,99	0,98	1,00	1,11	0,96	0,90	transducin family protein / WD-40 repeat family protein
258964_at	At3g10540	1,22	0,98	0,97	1,22	1,43	1,00	0,87	0,83	3-phosphoinositide-dependent protein kinase, putative
258963_at	At3g10550	1,16	1,34	1,79	0,97	1,07	1,27	1,34	1,22	expressed protein
258918_at	At3g10560	0,99	0,90	1,02	0,98	1,18	0,91	1,06	0,86	cytochrome P450, putative
258962_at	At3g10570	0,95	0,93	1,29	1,12	1,07	1,14	0,91	1,21	cytochrome P450, putative
258961_at	At3g10580	0,92	0,83	1,00	1,03	1,36	0,96	0,98	0,98	myb family transcription factor
258960_at	At3g10590	0,98	1,03	1,01	1,17	1,19	1,10	0,82	1,05	myb family transcription factor
258959_at	At3g10600	1,01	1,05	0,99	0,97	1,17	0,99	1,07	0,82	amino acid permease family protein
258922_at	At3g10610	1,34	1,13	1,01	1,35	1,29	0,97	0,91	0,87	40S ribosomal protein S17 (RPS17C)
258924_at	At3g10620	0,52	0,49	0,43	1,06	1,02	0,64	0,66	0,61	diadenosine 5',5'''-P1,P4-tetraphosphate hydrolase, putative
258917_at	At3g10630	1,07	1,01	1,40	1,09	1,30	0,89	0,81	0,81	glycosyl transferase family 1 protein
258915_at	At3g10640	0,93	0,89	0,95	1,06	1,19	0,97	1,15	1,14	SNF7 family protein
258946_at	At3g10650	0,76	0,81	1,01	0,84	0,71	0,94	0,85	0,85	expressed protein
258945_at	At3g10660	1,15	1,00	0,96	1,01	1,18	0,99	1,08	0,91	calcium-dependent protein kinase isoform 2 (CPK2)
258916_at	At3g10670	1,00	0,88	0,85	0,90	0,97	0,99	0,93	0,88	ABC transporter family protein
258969_at	At3g10680	0,99	1,01	0,89	1,01	1,19	1,01	0,97	0,81	heat shock protein-related
258966_at	At3g10690	1,53	1,17	1,08	0,82	0,88	1,09	1,03	1,06	DNA gyrase subunit A family protein
258766_at	At3g10700	1,08	1,02	0,89	0,96	0,88	1,00	0,96	0,86	GHMP kinase family protein
258765_at	At3g10710	1,09	1,04	0,99	1,10	0,94	0,97	0,99	0,91	pectinesterase family protein
258764_at	At3g10720	1,27	1,17	1,45	1,08	1,06	1,20	1,10	1,19	pectinesterase, putative
258773_at	At3g10730	0,94	1,02	0,85	0,96	1,18	0,93	0,83	0,79	sad1/unc-84-like 2 family protein
258774_at	At3g10740	0,91	1,23	1,18	0,94	0,72	0,83	0,90	0,95	glycosyl hydrolase family protein 51
258762_at	At3g10750	1,06	0,98	1,15	1,06	1,23	1,04	0,90	0,85	hypothetical protein
258761_at	At3g10760	0,98	0,86	0,68	1,13	1,34	1,14	0,87	0,74	myb family transcription factor
258771_at	At3g10770	1,13	1,20	1,50	1,02	1,03	1,08	1,06	1,44	expressed protein
258760_at	At3g10780	1,13	1,08	1,17	0,99	0,94	1,00	1,02	0,99	emp24/gp25L/p24 family protein
258759_at	At3g10800	0,94	0,98	1,16	1,04	1,02	1,13	1,15	1,21	bZIP transcription factor family protein
258758_at	At3g10810	0,93	1,02	0,80	0,90	1,10	0,83	0,76	0,74	zinc finger (C3HC4-type RING finger) family protein
258770_at	At3g10830	0,96	0,98	0,98	0,95	0,90	0,85	0,89	0,99	hypothetical protein
257533_at	At3g10840	1,15	1,16	1,44	0,67	0,59	1,29	1,23	1,24	hydrolase, alpha/beta fold family protein
258775_at	At3g10850	0,88	0,80	0,80	1,03	1,11	0,93	0,91	0,92	hydroxyacylglutathione hydrolase, cytoplasmic / glyoxalase II (GLX2-2)

258772_at	At3g10860	1,25	1,11	0,98	1,26	1,30	0,97	0,96	0,96 ubiquinol-cytochrome C reductase complex ubiquinone-binding protein, put
258769_at	At3g10870	1,10	1,18	1,15	0,75	0,87	0,92	0,88	0,88 hydrolase, alpha/beta fold family protein
258768_at	At3g10880	1,04	1,05	1,09	1,06	1,00	0,95	0,95	0,97 hypothetical protein
258767_at	At3g10890	1,08	1,03	1,01	1,06	0,98	1,08	1,08	1,06 (1-4)-beta-mannan endohydrolase, putative
258757_at	At3g10910	1,07	1,17	0,97	1,19	0,92	1,16	1,02	1,22 zinc finger (C3HC4-type RING finger) family protein
256439_at	At3g10920	1,09	0,97	0,91	1,15	1,08	0,98	0,99	1,02 superoxide dismutase (Mn), mitochondrial (SODA) / manganese superoxide
256442_at	At3g10930	1,31	1,24	0,92	0,87	0,59	1,74	2,30	1,39 expressed protein
256441_at	At3g10940	0,69	0,74	0,75	0,95	0,98	0,93	0,97	0,91 protein phosphatase-related
256434_at	At3g10950	1,07	0,90	0,99	0,92	1,01	0,88	0,92	0,92 60S ribosomal protein L37a (RPL37aB)
256443_at	At3g10960	0,79	0,78	0,54	1,14	0,73	1,07	1,26	1,02 xanthine/uracil permease family protein
256440_at	At3g10970	0,85	0,93	1,17	0,80	0,91	1,09	1,15	1,00 haloacid dehalogenase-like hydrolase family protein
256433_at	At3g10980	1,01	1,00	1,08	0,83	0,95	0,93	0,87	0,80 wound-responsive protein-related
256432_at	At3g10990	0,94	1,05	1,06	0,96	1,04	0,90	1,10	0,92 F-box family protein
257579_at	At3g11000	0,92	0,97	1,03	1,22	1,03	1,03	0,99	1,21 expressed protein
256431_s_at	At3g11010	1,07	1,06	0,94	0,89	0,97	1,20	1,08	1,21 disease resistance family protein / LRR family protein
256430_at	At3g11020	1,20	1,25	1,17	0,62	0,68	1,40	1,13	1,15 DRE-binding protein (DREB2B)
256429_at	At3g11040	0,98	0,93	0,96	0,94	1,15	1,04	0,82	1,08 glycosyl hydrolase family 85 protein
256416_at	At3g11050	0,58	0,67	0,44	1,07	1,01	1,07	1,02	0,93 ferritin, putative
256444_at	At3g11060	0,99	1,04	1,11	1,10	1,01	1,03	1,01	1,04 hypothetical protein
256428_at	At3g11080	0,99	0,99	1,01	1,03	0,98	1,26	0,96	1,09 disease resistance family protein
256427_at	At3g11090	1,08	1,26	1,30	1,19	1,42	1,24	1,40	1,31 LOB domain family protein / lateral organ boundaries domain family protein
256413_at	At3g11100	1,00	1,04	0,98	0,98	1,15	0,96	0,95	1,20 expressed protein
256446_at	At3g11110	0,82	0,90	0,95	0,97	1,10	0,97	1,14	0,85 zinc finger (C3HC4-type RING finger) family protein
256438_s_at	At3g11120	1,83	1,23	1,14	1,23	1,55	1,14	1,08	1,00 60S ribosomal protein L41 (RPL41C)
256437_s_at	At3g11130	0,92	0,86	0,87	1,19	1,10	0,85	0,98	0,86 clathrin heavy chain, putative
256436_at	At3g11150	0,94	0,92	0,95	1,08	1,08	0,94	0,96	0,82 expressed protein
256445_at	At3g11160	1,01	0,97	1,14	0,96	1,14	1,08	0,95	1,05 expressed protein
256417_s_at	At3g11170	0,85	0,62	0,71	0,81	0,86	1,19	1,17	1,11 omega-3 fatty acid desaturase, chloroplast (FAD7) (FADD)
256435_at	At3g11180	0,99	0,96	1,04	1,00	1,01	1,04	1,02	0,89 oxidoreductase, 2OG-Fe(II) oxygenase family protein
256414_at	At3g11200	1,14	0,89	0,85	1,27	1,48	0,99	0,91	0,84 PHD finger family protein
256415_at	At3g11210	1,38	1,17	1,43	0,96	1,32	0,97	0,83	0,86 GDSL-motif lipase/hydrolase family protein
256412_at	At3g11220	1,05	0,99	0,93	0,98	0,79	0,87	1,00	1,11 Paxneb protein-related
256256_at	At3g11230	0,85	0,75	0,82	1,11	0,90	1,18	1,26	1,23 yippee family protein
256257_at	At3g11240	1,12	0,97	1,02	1,11	1,33	1,05	0,90	1,19 arginine-tRNA-protein transferase, putative / arginyltransferase, putative / a
256253_at	At3g11250	1,24	1,17	1,01	1,42	1,19	1,02	1,01	0,94 60S acidic ribosomal protein P0 (RPP0C)
256249_at	At3g11270	0,97	0,84	1,01	0,99	1,01	0,92	0,79	0,78 26S proteasome non-ATPase regulatory subunit 7, putative / 26S proteaso
256255_at	At3g11280	1,19	1,08	1,11	1,24	1,30	1,08	1,07	1,17 myb family transcription factor
256254_at	At3g11290	1,24	1,12	1,21	0,92	1,13	1,13	1,13	1,18 expressed protein
256250_at	At3g11320	0,89	0,68	0,82	1,21	1,17	0,97	0,84	0,90 phosphate translocator-related
256251_at	At3g11330	1,21	1,14	1,09	1,13	1,20	1,08	0,96	1,04 leucine-rich repeat family protein
256252_at	At3g11340	0,91	0,92	0,74	1,04	1,13	1,10	1,59	1,07 UDP-glucoronosyl/UDP-glucosyl transferase family protein
259289_at	At3g11350	0,96	0,86	0,89	1,02	0,94	0,90	0,91	0,85 hypothetical protein
259260_at	At3g11370	1,01	1,45	0,89	1,70	1,41	1,02	1,32	1,12 DC1 domain-containing protein
259233_at	At3g11380	0,95	1,02	0,98	0,99	1,03	0,94	1,02	0,96 pentatricopeptide (PPR) repeat-containing protein
259261_at	At3g11390	1,10	1,20	0,98	1,31	1,52	1,06	0,94	0,67 DC1 domain-containing protein
259238_at	At3g11400	1,26	1,06	1,08	1,24	1,26	1,01	0,98	1,10 eukaryotic translation initiation factor 3G / eIF3g

259231_at	At3g11410	0,66	1,16	1,07	0,80	0,75	0,95	1,46	1,49	protein phosphatase 2C, putative / PP2C, putative
259232_at	At3g11420	0,52	0,53	0,55	1,01	1,00	0,91	0,94	1,00	fringe-related protein
259282_at	At3g11430	0,98	1,12	0,93	1,05	0,90	1,16	0,98	1,07	phospholipid/glycerol acyltransferase family protein
259283_at	At3g11440	1,01	0,78	0,82	1,04	0,95	1,06	0,99	1,03	myb family transcription factor (MYB65)
259284_at	At3g11450	1,05	0,90	1,22	0,94	0,96	1,20	1,03	1,37	DNAJ heat shock N-terminal domain-containing protein / cell division protei
259285_at	At3g11460	0,95	1,06	0,95	0,92	0,79	1,02	0,84	0,89	pentatricopeptide (PPR) repeat-containing protein
259286_at	At3g11480	1,02	1,10	1,02	0,97	1,23	1,13	0,90	1,05	S-adenosyl-L-methionine:carboxyl methyltransferase family protein
259287_at	At3g11490	1,11	1,17	1,24	0,96	0,78	1,25	1,16	1,05	rac GTPase activating protein, putative
259288_at	At3g11500	1,48	1,32	1,07	1,53	1,76	0,95	1,00	0,97	small nuclear ribonucleoprotein G, putative / snRNP-G, putative / Sm protei
259239_at	At3g11510	1,31	1,17	1,01	1,20	1,10	0,96	0,90	0,93	40S ribosomal protein S14 (RPS14B)
259290_at	At3g11520	1,19	1,31	1,30	1,60	1,37	1,08	0,86	0,89	cyclin, putative (CYC2)
259236_at	At3g11530	1,18	0,97	0,98	1,36	1,24	0,98	0,94	0,96	vacuolar protein sorting 55 family protein / VPS55 family protein
259259_at	At3g11540	0,94	0,89	0,98	0,91	0,94	0,91	0,91	1,01	gibberellin signal transduction protein (SPINDLY)
259291_at	At3g11550	1,19	1,25	1,10	1,30	1,06	1,00	1,07	1,05	integral membrane family protein
259292_at	At3g11560	0,99	0,95	1,00	0,82	0,81	1,20	1,13	1,20	expressed protein
259293_at	At3g11580	0,95	1,14	0,95	1,04	1,09	1,03	1,04	0,88	DNA-binding protein, putative
259240_at	At3g11590	1,05	1,10	1,12	1,14	1,14	0,96	0,83	0,84	expressed protein
259235_at	At3g11600	1,19	1,17	1,15	1,47	1,42	0,86	0,68	1,08	expressed protein
259234_at	At3g11620	0,88	1,02	1,23	0,89	0,76	0,98	0,98	1,18	expressed protein
259237_at	At3g11630	0,91	0,83	0,78	0,96	0,96	0,97	0,90	0,95	2-cys peroxiredoxin, chloroplast (BAS1)
259101_at	At3g11640	1,05	0,99	0,98	1,09	1,20	1,09	0,97	0,91	expressed protein
259071_at	At3g11650	0,71	0,74	0,90	1,06	1,04	0,93	0,94	0,92	harpin-induced family protein / HIN1 family protein / harpin-responsive famil
259102_at	At3g11660	0,86	1,11	1,06	1,63	1,46	0,94	0,91	0,94	harpin-induced family protein / HIN1 family protein / harpin-responsive famil
259070_at	At3g11670	1,40	1,59	1,68	0,82	0,90	1,18	1,13	1,07	digalactosyldiacylglycerol synthase 1 (DGD1) / MGDG:MGDG galactosyltra
259103_at	At3g11690	0,99	0,82	0,98	0,90	0,97	1,04	0,76	0,84	expressed protein
259072_at	At3g11700	0,97	0,99	0,88	0,99	1,17	1,04	0,92	0,84	beta-Ig-H3 domain-containing protein / fasciclin domain-containing protein
259069_at	At3g11710	1,28	1,06	1,10	1,03	1,02	1,03	1,07	1,04	lysyl-tRNA synthetase, putative / lysine--tRNA ligase, putative
258730_at	At3g11730	1,26	1,08	1,03	1,24	1,04	1,10	1,01	1,02	Ras-related GTP-binding protein, putative
258781_at	At3g11740	0,99	1,09	1,06	1,01	1,04	0,88	0,98	1,01	expressed protein
258726_at	At3g11745	1,12	1,10	1,17	1,24	1,37	1,01	0,97	0,90	expressed protein
258782_at	At3g11750	1,75	1,50	2,14	1,02	1,12	1,24	1,09	1,16	dihydroneopterin aldolase, putative
258783_at	At3g11760	1,09	1,34	0,99	0,86	1,10	0,87	1,00	0,96	expressed protein
258784_at	At3g11770	0,99	1,15	1,19	1,29	1,55	1,01	0,89	0,65	expressed protein
258788_at	At3g11780	1,10	1,08	0,99	1,15	1,12	0,92	0,83	0,84	MD-2-related lipid recognition domain-containing protein / ML domain-conta
258785_at	At3g11790	0,97	0,99	0,95	0,92	1,06	0,98	1,10	0,93	---
258728_at	At3g11800	1,31	0,93	0,95	1,27	1,31	0,98	0,81	0,78	expressed protein
258786_at	At3g11820	0,79	1,00	1,39	0,98	1,10	0,99	1,08	1,31	syntaxin 121 (SYP121) / syntaxin-related protein (SYR1)
258776_at	At3g11830	1,34	1,15	0,95	1,45	1,10	1,01	0,93	0,90	chaperonin, putative
258787_at	At3g11840	0,76	1,44	1,01	0,83	1,35	0,89	1,04	1,12	U-box domain-containing protein
258777_at	At3g11850	1,06	0,92	0,95	0,70	0,82	1,01	0,96	0,87	expressed protein
258778_s_at	At3g11860	1,06	1,02	1,01	0,85	0,73	1,01	0,87	0,98	expressed protein
258779_at	At3g11870	1,11	0,94	1,00	0,96	0,99	0,95	0,95	0,98	protein kinase-related
258731_at	At3g11880	1,03	0,94	0,93	1,01	1,00	0,93	0,98	1,10	expressed protein
258729_at	At3g11900	1,15	1,10	1,15	0,78	0,64	0,88	1,01	1,01	amino acid transporter family protein
258780_at	At3g11910	0,94	0,88	1,02	0,93	0,88	1,06	1,09	1,04	ubiquitin-specific protease, putative
258754_at	At3g11920	0,95	1,08	0,97	0,92	1,16	1,04	0,88	1,33	glutaredoxin-related

258727_at	At3g11930	0,98	0,92	0,91	0,97	1,03	0,72	0,83	0,91 universal stress protein (USP) family protein
258755_at	At3g11950	0,79	0,76	0,72	0,87	0,88	1,03	1,02	1,01 UbiA prenyltransferase family protein
258756_at	At3g11960	1,04	1,22	1,63	0,82	0,66	1,05	1,23	1,29 cleavage and polyadenylation specificity factor (CPSF) A subunit C-termina
256661_at	At3g11964	0,96	0,90	0,87	0,88	0,72	0,99	1,16	1,11 S1 RNA-binding domain-containing protein
256662_at	At3g11980	0,95	1,12	0,98	0,92	0,93	1,00	0,94	0,92 male sterility protein 2 (MS2)
256636_at	At3g12000	0,92	1,06	0,99	0,95	1,19	0,88	1,04	1,15 S-locus related protein SLR1, putative (S1)
256658_at	At3g12010	0,92	0,82	0,94	0,91	0,90	0,95	0,99	0,96 expressed protein
256659_at	At3g12020	0,86	0,81	1,12	0,88	1,03	1,12	1,23	1,67 kinesin motor protein-related
256637_at	At3g12030	0,74	0,77	0,75	1,27	1,56	0,82	0,68	0,78 expressed protein
256664_at	At3g12040	1,00	1,02	1,25	1,00	0,89	1,11	1,10	0,87 DNA-3-methyladenine glycosylase (MAG)
256663_at	At3g12050	1,18	1,22	1,21	1,20	1,21	1,00	1,06	1,04 Aha1 domain-containing protein
256660_at	At3g12060	1,07	1,08	1,14	0,96	1,32	1,13	0,95	1,10 expressed protein
256276_at	At3g12070	1,29	1,32	1,60	1,03	0,87	1,05	1,10	1,11 geranylgeranyl transferase type II beta subunit, putative / RAB geranylgera
256274_at	At3g12080	1,08	0,99	1,10	0,70	0,70	0,95	1,04	0,96 GTP-binding family protein
256273_at	At3g12090	1,51	1,47	1,15	1,20	1,05	0,92	1,43	1,14 senescence-associated family protein
256272_at	At3g12100	1,02	1,16	0,98	0,88	0,84	0,94	1,07	1,12 cation efflux family protein / metal tolerance protein, putative
256275_at	At3g12110	1,48	1,11	1,10	1,67	1,18	0,98	0,94	1,02 actin 11 (ACT11)
256277_at	At3g12120	0,66	0,64	0,66	0,46	0,45	0,80	0,82	0,84 omega-6 fatty acid desaturase, endoplasmic reticulum (FAD2) / delta-12 de
256278_at	At3g12130	1,14	1,02	1,16	1,16	1,03	0,98	1,01	1,05 KH domain-containing protein / zinc finger (CCCH type) family protein
256271_at	At3g12140	1,36	1,50	2,24	0,98	0,96	1,42	1,49	1,56 emsy N terminus domain-containing protein / ENT domain-containing protei
256262_at	At3g12150	1,74	1,63	1,75	1,19	1,18	0,97	0,95	1,24 expressed protein
256261_at	At3g12160	1,03	1,05	1,03	1,04	1,02	1,02	1,02	1,06 Ras-related GTP-binding family protein
256320_at	At3g12170	1,02	1,07	0,97	1,16	1,00	1,08	1,03	0,98 DNAJ heat shock N-terminal domain-containing protein
256286_at	At3g12180	1,43	1,57	1,40	1,60	1,68	0,94	0,90	0,87 cornichon family protein
256287_at	At3g12190	1,03	0,99	0,93	0,98	1,01	1,09	1,01	0,97 hypothetical protein
256291_at	At3g12200	1,07	1,03	1,22	0,85	0,84	1,03	1,01	1,34 protein kinase family protein
256290_at	At3g12203	0,95	1,01	0,92	0,94	1,02	1,04	1,14	1,14 serine carboxypeptidase S10 family protein
256264_at	At3g12210	1,32	1,32	1,26	1,01	0,91	0,94	0,97	0,79 expressed protein
256265_at	At3g12220	1,00	0,97	0,99	1,04	0,98	0,98	1,10	1,01 serine carboxypeptidase S10 family protein
256289_s_at	At3g12230	0,97	0,97	1,02	1,47	1,31	0,99	1,02	0,91 serine carboxypeptidase S10 family protein
256269_at	At3g12250	0,91	1,32	1,50	0,95	0,92	1,13	1,11	0,95 bZIP family transcription factor
256267_at	At3g12260	1,15	1,19	1,09	1,29	1,38	0,95	0,89	0,94 complex 1 family protein / LVR family protein
256288_at	At3g12270	1,41	1,36	1,17	0,96	0,89	1,01	1,01	0,99 protein arginine N-methyltransferase family protein
256268_at	At3g12280	1,01	1,09	1,23	1,07	1,20	0,92	0,96	0,86 retinoblastoma-related protein (RBR1)
256263_at	At3g12290	0,91	0,80	0,85	1,16	1,12	0,84	0,93	0,81 tetrahydrofolate dehydrogenase/cyclohydrolase, putative
256270_at	At3g12300	1,25	1,31	1,49	1,55	1,34	0,93	1,01	0,98 expressed protein
256266_at	At3g12320	2,12	2,07	1,22	1,48	1,16	1,97	1,77	1,24 expressed protein
256230_at	At3g12340	1,13	1,50	1,52	0,86	0,83	1,18	1,17	0,99 immunophilin, putative / FKBP-type peptidyl-prolyl cis-trans isomerase, puta
256236_at	At3g12350	1,05	1,15	0,95	1,34	1,28	1,13	1,00	1,23 F-box family protein
256233_at	At3g12360	0,80	1,07	1,26	0,97	0,93	1,00	1,15	1,71 ankyrin repeat family protein
256234_at	At3g12370	1,42	1,24	1,04	1,18	1,42	0,87	0,88	0,89 ribosomal protein L10 family protein
256242_at	At3g12380	1,05	1,29	1,41	0,82	0,93	1,16	1,17	1,13 actin/actin-like family protein
256241_at	At3g12390	1,21	1,13	1,08	1,50	1,58	0,92	0,94	1,01 nascent polypeptide associated complex alpha chain protein, putative / alph
256238_at	At3g12400	0,81	0,87	1,00	0,94	1,08	0,94	0,95	0,98 tumour susceptibility gene 101 (TSG101) family protein
257585_at	At3g12420	1,11	1,06	1,05	0,96	1,04	0,98	0,98	1,04 ---
256260_at	At3g12440	1,03	0,99	0,98	1,02	1,03	0,95	0,95	0,96 extensin family protein

256259_at	At3g12460	0,99	1,02	1,05	0,86	1,12	0,91	1,00	0,91 hypothetical protein
256239_at	At3g12470	1,02	0,94	0,81	1,04	1,16	1,00	0,94	0,94 expressed protein
256258_at	At3g12480	1,01	1,06	1,31	1,04	0,96	1,16	1,09	1,33 transcription factor, putative
256235_at	At3g12490	0,84	0,83	0,85	1,24	1,32	0,91	1,00	0,98 cysteine protease inhibitor, putative / cystatin, putative
256243_at	At3g12500	1,28	1,62	1,34	2,96	2,18	1,41	1,24	1,19 basic endochitinase
256285_at	At3g12510	1,56	1,38	1,23	1,12	1,33	1,17	1,03	0,94 expressed protein
256244_at	At3g12520	1,11	1,08	1,12	0,86	0,87	1,14	1,20	1,55 sulfate transporter family protein
256284_at	At3g12530	1,27	1,01	1,40	1,30	1,23	1,02	0,93	0,95 DNA replication protein-related
256283_at	At3g12540	0,98	0,97	1,02	0,94	1,16	1,12	1,21	1,06 expressed protein
256282_at	At3g12550	1,10	1,02	1,19	0,85	1,01	1,24	1,16	1,16 XH/XS domain-containing protein / XS zinc finger domain-containing protei
256281_at	At3g12560	1,21	1,07	1,09	0,76	1,33	0,98	0,92	1,05 telomeric DNA-binding protein, putative
256232_at	At3g12570	0,91	0,93	1,12	1,08	1,12	0,95	0,88	1,09 expressed protein
256245_at	At3g12580	1,82	1,82	1,83	1,86	1,68	1,33	1,44	1,34 heat shock protein 70, putative / HSP70, putative
256280_at	At3g12590	1,17	1,19	1,54	1,12	1,09	0,91	1,01	1,32 expressed protein
256240_at	At3g12600	1,23	1,10	1,15	1,20	1,20	0,91	0,96	1,03 MutT/nudix family protein
256237_at	At3g12610	1,68	2,16	2,06	0,94	1,16	0,73	0,58	0,53 DNA-damage-repair/toleration protein, putative (DRT100)
256279_at	At3g12620	0,74	0,57	0,53	0,91	0,96	0,93	0,84	0,92 protein phosphatase 2C family protein / PP2C family protein
256231_at	At3g12630	0,91	1,11	1,46	1,01	1,05	1,00	1,18	1,43 zinc finger (AN1-like) family protein
257703_at	At3g12640	0,99	1,00	1,03	0,86	0,98	1,12	0,98	0,96 RNA recognition motif (RRM)-containing protein
257707_at	At3g12650	0,95	1,07	0,96	1,38	1,35	0,93	0,91	0,91 expressed protein
257691_at	At3g12660	1,06	1,05	1,12	1,01	1,13	0,85	0,78	0,89 fasciclin-like arabinogalactan family protein
257702_at	At3g12670	1,06	1,01	1,09	0,66	0,58	1,00	1,02	1,08 CTP synthase, putative / UTP--ammonia ligase, putative
257695_at	At3g12680	1,06	1,06	1,07	0,84	0,96	1,08	1,02	1,28 floral homeotic protein (HUA1)
257706_at	At3g12685	1,15	1,10	1,50	0,66	0,68	1,30	1,20	1,09 expressed protein
257696_at	At3g12690	0,98	1,04	0,95	0,86	0,89	0,94	0,99	0,99 protein kinase, putative
257697_at	At3g12700	0,88	0,86	0,71	1,37	1,16	0,85	0,83	0,76 aspartyl protease family protein
257701_at	At3g12710	2,12	3,99	5,92	1,31	1,77	0,93	1,31	1,40 methyladenine glycosylase family protein
257704_at	At3g12720	0,96	1,09	1,08	0,93	1,19	0,99	0,99	1,03 myb family transcription factor
257698_at	At3g12730	1,12	1,14	1,20	1,46	1,38	0,87	0,89	0,94 myb family transcription factor
257700_at	At3g12740	0,91	0,86	1,12	0,94	0,91	1,02	1,07	1,31 LEM3 (ligand-effect modulator 3) family protein / CDC50 family protein
257715_at	At3g12750	1,27	1,20	1,14	1,37	1,31	0,87	0,89	0,94 zinc transporter (ZIP1)
257705_at	At3g12760	1,06	1,02	1,15	1,12	1,20	0,91	1,04	1,05 expressed protein
257685_at	At3g12770	1,10	1,01	0,95	0,97	1,05	0,96	0,99	1,02 pentatricopeptide (PPR) repeat-containing protein
257699_at	At3g12780	0,92	0,89	0,83	0,85	0,71	1,12	1,13	1,00 phosphoglycerate kinase, putative
257686_at	At3g12790	1,04	0,93	0,94	0,85	1,11	0,89	0,92	0,83 short-chain dehydrogenase/reductase (SDR) family protein
257687_at	At3g12800	0,88	0,95	0,96	0,96	1,08	0,94	0,89	0,88 short-chain dehydrogenase/reductase (SDR) family protein
257688_at	At3g12810	0,92	0,83	0,97	0,86	0,89	0,94	1,15	1,09 SNF2 domain-containing protein / helicase domain-containing protein
257689_at	At3g12820	1,00	1,00	1,01	1,11	1,09	1,08	1,04	0,95 myb family transcription factor (MYB10)
257690_at	At3g12830	0,80	0,79	1,01	0,89	0,79	1,26	1,18	1,00 auxin-responsive family protein
257692_at	At3g12840	0,93	0,98	0,97	1,12	1,15	1,02	1,01	1,05 hypothetical protein
257693_at	At3g12850	1,19	1,16	0,88	0,87	1,03	0,84	1,08	0,89 COP9 signalosome complex-related / CSN complex-related
257694_at	At3g12860	1,46	1,11	1,06	1,15	1,14	0,97	1,01	1,06 nucleolar protein Nop56, putative
257134_at	At3g12870	1,63	1,08	0,99	1,16	1,36	0,89	0,82	0,94 expressed protein
257136_at	At3g12890	1,13	1,04	0,93	0,97	1,05	1,04	0,97	1,07 expressed protein
257135_at	At3g12900	1,15	1,36	1,10	1,15	0,99	1,10	0,96	0,99 oxidoreductase, 2OG-Fe(II) oxygenase family protein
257846_at	At3g12910	0,93	1,01	0,96	0,97	0,99	1,03	0,96	1,09 expressed protein

257858_at	At3g12920	1,25	1,42	1,46	1,33	1,40	0,94	1,18	1,17	expressed protein
257856_at	At3g12930	1,16	1,11	1,08	0,85	0,71	1,14	0,98	0,85	expressed protein
257851_at	At3g12940	0,85	1,13	0,99	1,08	1,34	0,96	0,81	0,87	expressed protein
257852_at	At3g12950	0,96	0,97	0,87	0,94	1,23	1,13	1,05	1,45	expressed protein
257859_at	At3g12955	1,01	0,98	1,04	1,01	0,98	0,93	0,90	0,94	auxin-responsive protein-related
257853_at	At3g12960	0,91	0,74	0,62	0,87	1,06	1,03	0,97	1,18	expressed protein
257854_at	At3g12980	0,86	0,85	1,15	0,89	0,98	1,07	1,12	1,27	histone acetyltransferase 5 (HAC5)
257857_s_at	At3g12990	1,04	0,96	1,00	0,86	0,99	1,08	0,97	1,21	3' exoribonuclease family protein
257547_at	At3g13000	0,98	1,00	1,19	0,71	0,69	1,10	1,32	1,13	expressed protein
257847_at	At3g13020	0,99	0,98	0,90	0,92	0,98	1,02	0,88	0,86	hAT dimerisation domain-containing protein
257848_at	At3g13030	0,66	0,55	0,55	1,14	0,85	0,81	0,99	0,91	hAT dimerisation domain-containing protein
257855_at	At3g13040	1,00	1,30	1,39	0,90	0,89	1,24	1,37	1,56	myb family transcription factor
257861_at	At3g13050	0,61	0,56	0,68	0,82	0,90	0,87	0,91	1,05	transporter-related
257849_at	At3g13060	0,95	0,96	1,12	1,00	1,17	1,01	1,00	0,98	expressed protein
257860_at	At3g13062	1,01	1,02	1,27	1,06	1,16	1,06	1,02	1,03	expressed protein
257850_at	At3g13065	1,06	0,93	1,03	0,96	0,88	1,07	1,09	1,36	leucine-rich repeat transmembrane protein kinase, putative
257178_at	At3g13070	0,98	0,91	0,90	0,81	0,83	0,99	1,20	1,05	CBS domain-containing protein / transporter associated domain-containing
257184_at	At3g13090	0,98	0,87	0,99	0,98	0,99	1,01	1,07	1,18	ABC transporter, putative
257185_at	At3g13100	1,04	1,18	1,16	1,29	1,17	1,21	1,08	1,51	ABC transporter family protein
257194_at	At3g13110	1,02	1,22	1,15	1,22	1,27	0,82	0,90	0,89	serine O-acetyltransferase (SAT-1)
257190_at	At3g13120	1,03	0,98	0,94	0,83	0,92	0,95	0,92	0,94	30S ribosomal protein S10, chloroplast, putative
257186_at	At3g13130	0,99	0,99	0,99	1,07	1,03	1,24	1,04	0,99	hypothetical protein
257187_at	At3g13140	0,98	1,03	1,17	0,94	0,96	1,05	0,93	0,86	hydroxyproline-rich glycoprotein family protein
257188_at	At3g13150	1,06	0,98	0,95	1,07	0,88	0,93	0,77	0,94	pentatricopeptide (PPR) repeat-containing protein
257193_at	At3g13160	1,34	1,09	0,99	1,02	0,82	1,00	0,89	0,91	pentatricopeptide (PPR) repeat-containing protein
257179_at	At3g13170	1,05	1,04	1,12	1,02	0,96	0,94	0,99	0,88	DNA topoisomerase VIA (SPO11-1)
257191_at	At3g13175	1,23	1,10	0,90	1,27	1,08	0,83	0,77	0,71	expressed protein
257180_at	At3g13180	1,19	1,10	1,09	0,80	1,01	1,17	1,14	1,11	NOL1/NOP2/sun family protein / antitermination NusB domain-containing pı
257181_at	At3g13190	1,45	1,22	1,22	1,01	1,04	1,06	1,26	1,03	myosin heavy chain-related
257192_at	At3g13200	1,15	0,89	0,98	1,18	1,08	1,07	1,03	1,01	Cwf15 / Cwc15 cell cycle control family protein
257182_s_at	At3g13210	1,15	1,11	1,08	1,03	1,07	1,22	0,88	0,97	crooked neck protein, putative / cell cycle protein, putative
257183_at	At3g13220	0,92	0,89	1,09	1,04	1,12	1,01	0,94	0,87	ABC transporter family protein
257189_at	At3g13222	1,01	0,90	1,08	1,10	0,99	0,86	0,87	0,89	expressed protein
257653_at	At3g13225	1,08	1,13	1,23	1,08	0,94	1,06	1,08	1,09	WW domain-containing protein
257659_at	At3g13226	0,85	0,90	0,95	0,99	0,93	0,85	0,97	1,15	regulatory protein RecX family protein
257656_at	At3g13228	0,97	0,95	1,08	1,03	1,21	1,08	0,97	1,00	zinc finger (C3HC4-type RING finger) family protein
257658_at	At3g13230	1,15	0,97	1,03	1,10	0,87	0,97	0,88	0,97	expressed protein
257657_at	At3g13235	1,01	0,92	0,92	0,96	1,03	0,93	0,89	0,88	ubiquitin family protein
257682_at	At3g13240	0,96	0,99	0,96	0,79	0,85	1,09	0,96	0,98	hypothetical protein
257551_at	At3g13270	0,92	1,06	0,81	0,90	1,19	0,97	1,11	1,05	hypothetical protein
257683_at	At3g13280	0,94	1,12	1,06	1,12	1,04	0,92	1,00	0,99	expressed protein
257684_s_at	At3g13290	0,90	0,90	0,90	0,94	0,94	0,99	0,94	0,98	transducin family protein / WD-40 repeat family protein
257654_at	At3g13310	0,90	1,14	1,14	0,79	0,82	0,95	0,93	1,34	DNAJ heat shock N-terminal domain-containing protein
257662_at	At3g13320	0,86	0,81	0,86	1,27	1,16	0,87	0,92	0,99	calcium exchanger (CAX2)
257708_at	At3g13330	0,66	0,59	0,62	0,91	0,89	0,97	0,99	1,02	expressed protein
257661_at	At3g13340	0,78	0,78	0,95	0,89	0,92	1,03	0,92	1,09	WD-40 repeat family protein

257655_at	At3g13350	1,13	0,88	0,85	0,86	0,76	1,10	0,86	0,84	high mobility group (HMG1/2) family protein / ARID/BRIGHT DNA-binding d
257660_at	At3g13360	0,94	0,91	1,12	0,93	1,03	0,93	0,90	0,86	expressed protein
257681_at	At3g13370	1,04	0,92	1,00	0,98	1,04	0,90	1,05	0,99	hypothetical protein
256981_at	At3g13380	0,79	0,61	0,66	0,96	0,97	1,18	1,04	1,41	leucine-rich repeat family protein / protein kinase family protein
256955_at	At3g13390	1,03	1,08	0,97	1,01	1,03	1,05	0,94	0,97	multi-copper oxidase type I family protein
256966_at	At3g13400	0,92	0,94	1,02	1,03	1,19	1,00	0,90	1,13	multi-copper oxidase type I family protein
256956_at	At3g13410	1,03	0,91	0,88	1,00	0,92	0,98	0,99	0,93	expressed protein
256957_at	At3g13420	1,04	1,15	1,13	0,98	0,85	1,07	0,96	0,87	expressed protein
256958_at	At3g13430	0,87	1,06	1,09	1,14	1,02	0,79	1,15	1,51	zinc finger (C3HC4-type RING finger) family protein
256959_at	At3g13440	0,73	0,72	0,80	0,81	0,79	0,96	1,02	1,04	expressed protein
256961_at	At3g13445	1,22	1,13	1,29	1,17	1,45	1,02	0,92	0,95	transcription initiation factor IID-1 (TFIID-1) / TATA-box factor 1 / TATA seq
256965_at	At3g13450	1,01	1,35	1,58	0,85	0,94	1,03	1,16	1,21	2-oxoisovalerate dehydrogenase / 3-methyl-2-oxobutanoate dehydrogenase
256982_at	At3g13460	1,11	1,03	1,09	0,83	0,89	0,99	0,92	0,96	expressed protein
256983_at	At3g13470	1,08	0,94	1,00	0,85	0,77	1,04	1,02	1,01	chaperonin, putative
256984_at	At3g13480	1,01	0,97	1,27	1,05	1,12	1,12	1,02	0,82	expressed protein
256963_at	At3g13490	1,09	0,91	0,92	0,85	0,84	1,10	1,05	1,06	tRNA synthetase class II (D, K and N) family protein
256960_at	At3g13510	0,74	0,97	1,02	1,02	1,07	0,98	0,97	0,95	expressed protein
256964_at	At3g13520	1,05	1,01	0,95	1,33	1,16	0,98	0,98	1,00	arabinogalactan-protein (AGP12)
256985_at	At3g13540	1,15	1,03	1,05	1,01	0,93	1,11	0,94	1,13	myb family transcription factor
256954_at	At3g13550	1,05	1,02	1,05	0,91	1,12	1,10	1,13	1,12	ubiquitin-conjugating enzyme (COP10)
256962_at	At3g13560	1,02	0,96	0,90	1,25	1,10	0,89	0,80	0,78	glycosyl hydrolase family 17 protein
256649_at	At3g13570	1,34	1,35	1,30	1,15	1,00	1,11	1,02	1,16	SC35-like splicing factor, 30a kD (SCL30a)
256648_at	At3g13580	1,75	1,64	1,35	1,64	1,30	1,13	1,00	0,98	60S ribosomal protein L7 (RPL7D)
256646_at	At3g13590	1,05	1,16	1,17	1,26	1,13	0,96	1,09	1,07	DC1 domain-containing protein
256647_at	At3g13610	1,87	2,30	1,91	1,22	1,05	2,71	2,23	1,44	oxidoreductase, 2OG-Fe(II) oxygenase family protein
256650_at	At3g13620	0,90	1,01	1,03	1,01	1,13	0,87	0,94	0,88	amino acid permease family protein
256773_at	At3g13630	1,05	0,98	1,05	1,06	0,89	0,87	1,02	1,13	hypothetical protein
256780_at	At3g13640	1,11	1,01	1,12	1,02	0,94	0,99	1,10	0,97	RNase L inhibitor protein, putative
256781_at	At3g13650	0,92	1,27	0,95	3,00	2,19	0,78	0,92	0,75	disease resistance response protein-related/ dirigent protein-related
256782_at	At3g13660	0,90	1,00	0,98	0,89	0,93	1,12	0,97	0,82	disease resistance response protein-related/ dirigent protein-related
257570_at	At3g13662	0,93	1,15	0,98	1,04	1,30	0,97	0,98	0,84	disease resistance-responsive protein-related / dirigent protein-related
256783_at	At3g13670	1,07	0,94	1,02	0,95	1,05	1,06	1,04	0,98	protein kinase family protein
256789_at	At3g13672	0,87	0,98	1,08	1,09	1,06	0,96	0,98	0,81	seven in absentia (SINA) family protein
256784_at	At3g13674	1,13	1,04	0,87	1,25	1,31	0,71	1,09	0,96	expressed protein
256767_at	At3g13680	1,01	0,96	0,90	1,07	0,99	0,96	0,94	0,83	F-box family protein
256768_at	At3g13682	0,94	1,03	1,05	1,08	1,25	1,03	1,05	0,81	amine oxidase family protein / SWIRM domain-containing protein
256769_at	At3g13690	0,82	1,24	1,60	0,47	0,74	0,96	1,10	1,32	protein kinase family protein
256770_at	At3g13700	1,19	1,19	1,16	1,08	1,04	0,83	0,91	0,83	prenylated rab acceptor (PRA1) family protein
256771_at	At3g13710	1,62	2,03	1,30	0,78	0,80	0,84	0,76	0,72	RNA-binding protein, putative
256785_at	At3g13720	0,91	1,00	0,91	1,01	1,05	0,73	0,72	0,72	prenylated rab acceptor (PRA1) family protein
256788_at	At3g13730	1,23	1,11	1,11	0,94	0,89	0,97	1,08	1,34	cytochrome P450, putative
256786_at	At3g13740	1,05	1,04	1,15	0,75	0,95	1,16	1,02	0,99	URF 4-related
256772_at	At3g13750	0,94	1,84	1,83	0,77	0,75	1,26	1,34	1,32	beta-galactosidase, putative / lactase, putative
256774_at	At3g13760	1,10	1,06	1,04	1,48	1,20	0,93	1,11	1,09	DC1 domain-containing protein
256775_at	At3g13770	0,93	1,01	1,10	0,95	1,16	1,04	0,97	0,97	pentatricopeptide (PPR) repeat-containing protein
256776_at	At3g13772	0,88	0,90	0,95	1,22	1,15	0,92	0,91	0,92	endomembrane protein 70, putative

256777_at	At3g13780	1,06	1,19	1,46	0,93	0,86	1,18	1,01	1,04	expressed protein
256778_at	At3g13782	0,80	0,99	1,12	0,87	1,01	1,23	1,14	1,29	nucleosome assembly protein (NAP) family protein
256779_at	At3g13784	0,97	0,98	1,04	0,99	0,96	0,99	0,91	0,99	beta-fructosidase, putative / beta-fructofuranosidase, putative / cell wall inve
256787_at	At3g13790	1,18	1,15	1,06	1,17	1,06	1,33	1,09	0,98	beta-fructosidase (BFRUCT1) / beta-fructofuranosidase / cell wall invertase
257602_at	At3g13800	1,00	0,96	0,81	1,17	1,03	0,80	0,73	0,70	metallo-beta-lactamase family protein
257610_at	At3g13810	1,10	1,11	0,88	0,85	0,99	1,00	1,20	1,15	zinc finger (C2H2 type) family protein
257603_at	At3g13820	1,04	1,12	1,07	0,91	0,93	0,95	0,95	0,94	F-box family protein
257604_s_at	At3g13830	1,00	0,95	1,05	1,05	1,11	0,98	1,00	1,07	F-box family protein
257605_at	At3g13840	1,04	0,97	0,87	1,07	0,89	1,01	0,92	1,15	scarecrow transcription factor family protein
257609_at	At3g13845	1,36	1,06	0,92	1,25	1,34	0,86	0,91	0,90	expressed protein
257608_at	At3g13860	1,42	1,21	1,06	1,06	0,96	1,04	1,00	1,02	chaperonin, putative
257606_at	At3g13870	1,02	0,86	0,90	1,36	1,24	1,02	0,97	0,91	root hair defective 3 (RHD3)
257607_at	At3g13880	1,08	1,04	1,09	0,94	0,99	1,03	0,89	0,96	pentatricopeptide (PPR) repeat-containing protein
258195_at	At3g13890	0,98	1,01	1,01	1,07	1,04	1,02	0,98	1,22	myb family transcription factor (MYB26)
258200_at	At3g13900	1,19	0,86	1,06	0,91	1,00	0,97	1,00	0,78	haloacid dehalogenase-like hydrolase family protein
258201_at	At3g13910	1,36	1,09	1,01	1,77	1,83	1,07	0,83	0,65	expressed protein
258210_at	At3g13920	1,13	0,95	0,92	0,87	0,88	1,04	0,96	0,93	eukaryotic translation initiation factor 4A-1 / eIF-4A-1
258208_at	At3g13930	0,90	0,77	0,72	1,17	1,12	0,92	0,88	0,98	dihydrolipoamide S-acetyltransferase, putative
258202_at	At3g13940	1,40	0,95	1,06	0,88	0,86	1,08	0,83	0,98	expressed protein
258203_at	At3g13950	0,90	1,04	0,86	1,15	1,01	1,26	1,04	0,97	expressed protein
258204_at	At3g13960	0,98	1,09	1,07	0,84	1,24	1,21	0,80	0,90	expressed protein
258205_at	At3g13970	0,82	0,86	0,98	1,08	1,13	1,13	1,09	1,04	pseudogene, hypothetical protein
258196_at	At3g13980	0,84	1,18	0,89	1,00	1,22	0,77	1,04	1,15	expressed protein
258197_at	At3g14000	0,87	0,81	0,95	0,96	0,96	0,81	0,90	1,00	expressed protein
258206_at	At3g14010	0,84	0,80	0,78	0,90	1,04	1,08	1,03	1,03	hydroxyproline-rich glycoprotein family protein
258198_at	At3g14020	1,01	0,85	0,88	0,84	0,95	0,92	0,86	0,94	CCAAT-binding transcription factor (CBF-B/NF-YA) family protein
258199_at	At3g14030	0,83	0,99	1,00	1,13	1,31	1,12	0,82	0,88	hypothetical protein
258207_at	At3g14050	0,82	1,27	1,06	0,93	0,73	1,20	1,60	1,67	RelA/SpoT protein, putative (RSH2)
258209_at	At3g14060	1,09	1,03	1,02	4,77	5,13	1,27	1,06	1,05	expressed protein
256997_at	At3g14067	1,39	1,35	1,34	1,22	1,54	1,13	1,01	1,04	subtilase family protein
257011_at	At3g14070	0,85	0,83	0,90	1,05	1,09	0,91	0,85	1,02	cation exchanger, putative (CAX9)
257006_at	At3g14075	1,11	1,16	1,26	1,01	1,04	1,01	1,05	1,30	lipase class 3 family protein
257001_at	At3g14080	1,46	1,42	1,48	0,14	0,19	0,90	0,94	0,94	small nuclear ribonucleoprotein, putative / snRNP, putative / Sm protein, pu
257010_at	At3g14090	0,81	0,89	0,84	1,17	1,13	1,06	1,04	1,09	exocyst subunit EXO70 family protein
257002_at	At3g14100	1,01	0,97	0,93	0,98	1,12	0,91	0,82	0,90	oligouridylate-binding protein, putative
257003_at	At3g14110	0,82	0,74	0,77	0,86	0,84	0,88	0,91	0,88	tetratricopeptide repeat (TPR)-containing protein
257000_at	At3g14120	1,14	1,04	1,01	0,74	0,77	0,83	0,92	0,89	expressed protein
257004_s_at	At3g14150	0,72	0,73	0,80	0,80	0,76	1,02	1,29	1,17	(S)-2-hydroxy-acid oxidase, peroxisomal, putative / glycolate oxidase, putat
257009_at	At3g14160	1,00	1,03	1,21	1,02	1,05	1,01	1,00	0,93	oxidoreductase, 2OG-Fe(II) oxygenase family protein
256998_at	At3g14180	0,95	1,10	1,25	1,10	1,10	0,97	0,85	0,97	expressed protein
257005_at	At3g14190	1,24	1,33	1,30	1,54	1,53	0,83	0,96	0,90	expressed protein
256999_at	At3g14200	1,50	1,37	1,15	1,39	1,60	1,40	1,44	1,34	DNAJ heat shock N-terminal domain-containing protein
257007_at	At3g14205	0,92	0,71	0,56	1,07	1,12	1,16	1,21	1,28	phosphoinositide phosphatase family protein
257008_at	At3g14210	0,99	0,76	0,73	0,93	1,07	0,94	0,77	0,69	myrosinase-associated protein, putative
257072_at	At3g14220	0,80	0,75	0,77	1,01	0,98	0,69	0,64	0,59	GDSL-motif lipase/hydrolase family protein
258364_at	At3g14225	0,87	1,03	1,04	0,98	1,11	0,99	1,05	0,89	GDSL-motif lipase/hydrolase family protein

258366_at	At3g14230	0,73	0,68	0,81	1,03	1,18	0,81	0,76	0,79 AP2 domain-containing protein RAP2.2 (RAP2.2)
258368_at	At3g14240	0,93	0,88	0,83	0,88	0,98	0,76	0,68	0,76 subtilase family protein
258360_at	At3g14250	0,99	1,11	1,05	1,01	1,05	0,97	1,04	0,94 zinc finger protein-related
258361_at	At3g14270	0,82	0,79	0,84	0,86	0,90	1,16	0,92	1,01 phosphatidylinositol-4-phosphate 5-kinase family protein
258362_at	At3g14280	1,06	1,03	1,05	1,37	1,40	0,99	0,94	0,97 expressed protein
258373_at	At3g14290	1,16	0,98	0,98	1,17	1,07	1,00	1,03	0,99 20S proteasome alpha subunit E2 (PAE2)
258363_at	At3g14300	0,96	1,17	1,07	0,92	1,09	0,95	0,95	1,00 pectinesterase family protein
258369_at	At3g14310	0,90	0,88	0,92	0,91	0,81	0,81	0,75	0,77 pectinesterase family protein
258354_at	At3g14320	1,04	1,11	1,04	1,13	1,07	1,07	0,94	0,79 zinc finger (C3HC4-type RING finger) family protein
258355_at	At3g14330	0,92	0,87	1,13	0,68	0,81	0,95	0,89	0,79 pentatricopeptide (PPR) repeat-containing protein
258356_at	At3g14340	0,95	0,65	0,81	0,95	1,31	1,06	0,86	0,90 expressed protein
258357_at	At3g14350	0,76	0,72	0,75	1,10	1,22	0,93	0,93	1,08 leucine-rich repeat transmembrane protein kinase, putative
258374_at	At3g14360	1,03	1,02	0,87	0,92	0,88	1,00	1,10	1,08 lipase class 3 family protein
258367_at	At3g14370	1,05	0,98	0,89	1,03	0,98	0,98	1,03	1,05 protein kinase family protein
258358_at	At3g14380	1,02	1,14	1,15	1,30	1,03	0,96	1,13	1,19 integral membrane family protein
258365_s_at	At3g14390	1,32	1,04	0,97	0,88	0,82	1,03	0,95	0,89 diaminopimelate decarboxylase, putative / DAP carboxylase, putative
258370_at	At3g14395	0,91	1,01	1,01	0,99	0,98	0,99	0,95	0,86 expressed protein
258372_at	At3g14400	1,19	0,93	0,90	0,88	1,04	1,05	1,02	0,93 ubiquitin-specific protease 25 (UBP25)
258371_at	At3g14410	0,89	0,76	0,82	1,17	1,34	0,79	0,91	0,87 transporter-related
258359_s_at	At3g14415	1,07	1,05	1,14	0,85	0,95	1,24	1,13	1,20 (S)-2-hydroxy-acid oxidase, peroxisomal, putative / glycolate oxidase, putat
257279_at	At3g14430	0,84	0,86	0,84	1,39	0,89	0,87	0,83	0,89 expressed protein
257280_at	At3g14440	0,59	0,96	1,03	0,74	1,00	1,05	1,04	1,05 9-cis-epoxycarotenoid dioxygenase, putative / neoxanthin cleavage enzyme
257275_at	At3g14450	1,09	0,98	0,85	0,96	0,92	1,03	0,99	0,78 RNA-binding protein, putative
257276_at	At3g14460	0,94	0,96	1,11	0,91	1,01	1,05	1,03	0,96 disease resistance protein (NBS-LRR class), putative
257277_at	At3g14470	0,96	1,06	1,13	0,94	1,00	1,25	0,98	0,86 disease resistance protein (NBS-LRR class), putative
257278_at	At3g14480	0,85	0,96	0,98	0,96	1,05	1,10	1,01	0,96 glycine/proline-rich protein
257557_at	At3g14490	1,05	1,01	1,04	0,95	1,04	0,91	0,99	0,89 terpene synthase/cyclase family protein
257274_at	At3g14510	0,90	0,90	0,91	0,90	1,05	1,03	1,10	0,86 geranylgeranyl pyrophosphate synthase, putative / GGPP synthetase, putat
258116_at	At3g14520	1,02	1,08	1,02	0,89	1,17	1,03	1,13	1,15 terpene synthase/cyclase family protein
258121_s_at	At3g14530	1,16	1,23	1,10	0,97	0,86	1,06	0,94	1,12 geranylgeranyl pyrophosphate synthase, putative / GGPP synthetase, putat
258091_at	At3g14560	1,46	1,32	0,98	1,51	1,10	0,97	1,03	1,06 expressed protein
258122_at	At3g14570	1,55	1,55	1,40	0,84	0,89	0,91	1,36	1,18 glycosyl transferase family 48 protein
258088_at	At3g14580	1,05	0,94	1,05	1,00	1,01	0,94	1,00	0,87 pentatricopeptide (PPR) repeat-containing protein
258092_at	At3g14595	0,71	0,73	0,88	0,83	0,91	0,92	0,95	1,05 expressed protein
258090_at	At3g14600	1,64	1,60	1,58	1,37	1,53	1,09	1,08	1,09 60S ribosomal protein L18A (RPL18aC)
258110_at	At3g14610	0,76	0,88	0,91	1,13	1,10	0,89	0,94	0,87 cytochrome P450, putative
258063_at	At3g14620	0,55	0,58	1,04	0,67	0,74	0,98	1,32	1,49 cytochrome P450, putative
258111_at	At3g14630	0,91	0,93	0,99	0,71	0,84	0,91	1,18	1,39 cytochrome P450, putative
258112_at	At3g14640	1,20	1,03	1,03	0,90	0,85	1,20	1,05	1,02 cytochrome P450, putative
258113_at	At3g14650	1,18	1,23	1,57	0,79	0,64	1,26	1,08	1,16 cytochrome P450, putative
258114_at	At3g14660	0,53	0,51	0,75	0,67	0,83	0,79	0,85	0,93 cytochrome P450, putative
258115_at	At3g14670	1,00	1,04	0,97	0,95	1,03	1,05	0,99	0,94 hypothetical protein
258064_at	At3g14680	0,92	0,97	0,91	1,06	1,05	1,03	1,13	0,77 cytochrome P450, putative
258094_at	At3g14690	1,05	1,21	1,56	1,05	1,14	1,30	1,35	1,40 cytochrome P450, putative
258117_at	At3g14700	1,20	1,32	1,34	1,18	1,12	1,03	1,14	1,19 expressed protein
258118_at	At3g14710	0,90	1,08	1,09	0,93	0,92	1,08	0,96	0,91 F-box family protein

258119_at	At3g14720	1,20	0,87	0,99	1,25	1,16	1,22	1,31	1,58 mitogen-activated protein kinase, putative / MAPK, putative (MPK19)
258120_at	At3g14730	1,14	0,97	1,01	0,97	1,15	0,94	0,88	0,94 pentatricopeptide (PPR) repeat-containing protein
258089_at	At3g14740	1,09	1,13	1,16	1,10	1,14	0,95	0,92	1,14 PHD finger family protein
258093_at	At3g14750	1,24	1,71	1,62	1,15	1,23	1,25	1,09	1,31 expressed protein
256599_at	At3g14760	1,22	1,08	1,01	1,03	1,04	0,85	0,94	1,00 expressed protein
256548_at	At3g14770	1,18	1,32	1,55	1,15	0,83	1,55	1,44	1,18 nodulin MtN3 family protein
256574_at	At3g14780	1,09	1,38	1,28	1,02	1,38	1,02	1,07	1,16 expressed protein
256575_at	At3g14790	1,04	0,87	0,87	1,19	1,25	0,93	0,81	0,75 NAD-dependent epimerase/dehydratase family protein
256545_at	At3g14800	1,13	1,08	1,02	0,90	0,93	1,11	1,00	1,06 hAT-like transposase family (hobo/Ac/Tam3)
256546_at	At3g14820	1,04	0,98	1,02	1,04	0,94	0,99	1,03	0,97 GDSL-motif lipase/hydrolase family protein
256547_at	At3g14840	0,83	0,77	0,91	0,95	0,91	0,87	1,02	1,14 leucine-rich repeat family protein / protein kinase family protein
256600_at	At3g14850	1,21	1,27	1,41	1,10	1,12	1,03	0,92	0,82 expressed protein
256573_at	At3g14860	0,97	0,93	0,90	0,91	1,06	1,03	0,88	0,88 NHL repeat-containing protein
257234_at	At3g14880	0,90	0,83	0,91	1,21	0,94	0,90	0,86	0,82 DNA-binding protein-related
257237_at	At3g14890	1,48	1,04	1,16	1,53	1,27	1,14	1,12	0,99 phosphoesterase
257207_at	At3g14900	1,06	0,96	0,95	0,83	0,85	1,08	0,93	0,76 expressed protein
257208_at	At3g14910	0,72	1,00	0,94	0,82	1,27	0,90	0,77	1,12 expressed protein
257209_at	At3g14920	0,84	0,73	0,83	1,16	1,09	0,94	0,80	0,72 expressed protein
257219_at	At3g14930	0,98	0,90	1,08	0,86	0,93	0,99	0,99	1,00 uroporphyrinogen decarboxylase, putative / UPD, putative
257217_at	At3g14940	0,97	0,92	0,81	1,55	1,73	1,40	1,53	1,40 phosphoenolpyruvate carboxylase, putative / PEP carboxylase, putative
257210_at	At3g14950	1,05	1,03	0,99	1,08	0,95	1,02	0,89	0,91 tetratricopeptide repeat (TPR)-containing protein
257560_at	At3g14960	0,72	0,69	0,74	0,73	0,92	0,96	0,84	0,82 galactosyltransferase family protein
257265_at	At3g14980	0,70	0,82	1,11	0,85	0,74	0,96	0,96	1,04 PHD finger transcription factor, putative
257216_at	At3g14990	0,86	0,90	1,06	0,65	0,75	0,93	1,02	1,10 4-methyl-5(b-hydroxyethyl)-thiazole monophosphate biosynthesis protein, p
257218_at	At3g15000	1,56	1,31	1,42	0,99	1,11	0,96	0,98	1,02 expressed protein
257266_at	At3g15010	0,93	0,80	0,85	0,86	0,91	0,97	0,90	0,98 RNA recognition motif (RRM)-containing protein
257213_at	At3g15020	0,88	0,73	0,66	1,24	1,09	0,87	0,97	1,00 malate dehydrogenase (NAD), mitochondrial, putative
257267_at	At3g15030	0,96	0,79	0,85	0,70	0,84	0,87	0,81	0,86 TCP family transcription factor, putative
257214_at	At3g15040	0,82	0,90	1,20	0,99	1,23	0,89	0,91	1,13 expressed protein
257233_at	At3g15050	0,97	0,94	1,06	0,98	1,26	1,02	1,10	1,09 calmodulin-binding family protein
257235_at	At3g15060	0,80	0,69	0,94	0,93	1,06	0,98	1,06	1,14 Ras-related GTP-binding family protein
257215_at	At3g15070	1,16	1,17	1,45	0,80	1,04	1,13	0,97	1,48 zinc finger (C3HC4-type RING finger) family protein
257211_at	At3g15080	1,37	1,15	0,91	0,97	0,82	1,10	1,16	1,11 exonuclease family protein
257212_at	At3g15090	0,84	1,02	1,30	0,79	0,93	1,25	1,08	1,27 oxidoreductase, zinc-binding dehydrogenase family protein
257236_at	At3g15095	0,64	0,68	0,81	0,63	0,79	0,85	0,77	0,89 expressed protein
256856_at	At3g15110	0,92	0,95	0,88	0,89	0,89	0,96	0,90	0,94 expressed protein
256885_at	At3g15120	0,96	0,83	1,03	0,69	0,81	1,07	1,15	1,12 AAA-type ATPase family protein
256886_at	At3g15130	0,90	0,97	1,06	0,84	0,93	0,92	0,99	0,83 pentatricopeptide (PPR) repeat-containing protein
256858_at	At3g15140	1,33	1,17	1,31	0,77	0,78	0,95	0,96	0,87 exonuclease family protein
256887_at	At3g15150	1,27	1,31	1,36	0,75	1,00	1,02	1,27	1,07 expressed protein
256888_at	At3g15160	0,76	0,79	0,95	0,90	1,06	0,95	1,01	1,06 expressed protein
256857_at	At3g15170	0,84	0,83	0,76	0,91	1,01	0,89	0,92	0,96 cup-shaped cotyledon1 protein / CUC1 protein (CUC1)
256854_at	At3g15180	0,64	0,55	0,62	0,89	0,92	0,89	0,97	1,01 proteasome-related
256855_at	At3g15190	1,12	0,93	0,87	0,89	0,89	0,99	0,98	1,00 chloroplast 30S ribosomal protein S20, putative
256884_at	At3g15200	0,96	0,92	0,83	0,98	0,89	1,46	1,12	1,34 pentatricopeptide (PPR) repeat-containing protein
257053_at	At3g15210	0,92	1,26	1,00	1,10	0,83	1,04	1,27	1,48 ethylene-responsive element-binding factor 4 (ERF4)

257078_at	At3g15220	1,12	1,10	1,07	1,05	1,12	1,10	1,15	1,07 protein kinase, putative
257079_at	At3g15230	1,16	1,26	1,11	1,10	1,05	1,10	1,17	1,02 hypothetical protein
257080_at	At3g15240	1,16	1,04	1,11	1,51	1,40	1,19	1,15	1,16 expressed protein
257049_at	At3g15250	0,97	1,07	0,92	1,00	0,97	1,08	0,94	1,24 expressed protein
257050_at	At3g15260	1,14	1,02	0,91	0,85	0,94	0,87	0,83	0,84 protein phosphatase 2C, putative / PP2C, putative
257051_at	At3g15270	0,97	1,02	0,97	1,01	1,18	0,86	0,97	0,84 squamosa promoter-binding protein-like 5 (SPL5)
257059_at	At3g15280	0,96	0,76	0,71	0,87	0,71	1,15	0,98	0,90 expressed protein
257052_at	At3g15290	0,86	0,78	0,85	0,96	1,10	0,96	0,90	1,02 3-hydroxybutyryl-CoA dehydrogenase, putative
257105_at	At3g15300	0,99	1,10	1,02	1,13	1,19	0,85	1,04	1,05 VQ motif-containing protein
257057_at	At3g15310	1,04	1,00	0,98	0,94	1,05	1,11	1,09	0,97 expressed protein
257056_at	At3g15350	0,60	0,68	0,74	0,72	0,75	0,90	0,99	1,27 glycosyltransferase family 14 protein / core-2/I-branching enzyme family pro
257055_at	At3g15351	1,44	1,18	1,22	0,99	0,81	1,14	1,28	1,46 expressed protein
257058_at	At3g15352	1,17	1,07	1,03	1,23	1,08	1,04	0,98	0,92 cytochrome c oxidase copper chaperone-related
257054_at	At3g15353	1,17	1,11	1,04	1,12	1,07	0,86	0,93	0,92 metallothionein protein, putative
258382_at	At3g15355	1,16	1,36	1,01	0,93	1,11	1,00	0,99	0,91 ubiquitin-conjugating enzyme-related
258397_at	At3g15357	1,12	1,10	1,16	1,73	2,02	0,99	1,00	1,01 expressed protein
258398_at	At3g15360	0,78	0,77	0,79	0,88	0,91	0,99	0,91	0,98 thioredoxin M-type 4, chloroplast (TRX-M4)
258388_at	At3g15370	1,01	1,17	1,12	0,95	1,12	1,02	0,98	0,96 expansin, putative (EXP12)
258403_at	At3g15380	0,78	0,82	0,68	0,76	0,70	0,93	0,94	0,80 choline transporter-related
258389_at	At3g15390	1,07	0,89	1,01	1,11	1,02	1,15	0,97	0,90 expressed protein
258392_at	At3g15400	0,72	0,73	0,74	0,94	1,09	0,92	1,07	1,02 anther development protein, putative
258390_at	At3g15410	0,83	0,73	0,72	1,09	1,24	0,73	0,79	0,92 leucine-rich repeat family protein
258391_at	At3g15420	1,12	1,11	0,92	0,99	1,00	0,93	1,06	1,01 expressed protein
258401_at	At3g15430	0,83	0,93	1,00	1,12	1,06	0,93	1,01	0,96 regulator of chromosome condensation (RCC1) family protein
258383_at	At3g15440	0,77	0,85	0,99	1,00	1,01	0,82	0,99	1,00 expressed protein
258402_at	At3g15450	1,26	2,12	2,06	0,82	0,69	0,92	1,20	1,44 expressed protein
258396_at	At3g15460	1,83	1,02	0,89	1,14	1,24	1,02	1,00	1,03 brix domain-containing protein
258400_at	At3g15470	0,93	0,92	0,90	0,68	0,86	0,94	1,03	1,36 WD-40 repeat family protein
258393_at	At3g15480	0,84	0,75	0,74	1,08	0,99	0,88	0,81	0,81 expressed protein
258384_at	At3g15490	0,98	1,02	1,04	0,95	0,99	0,99	1,05	0,94 expressed protein
258395_at	At3g15500	0,80	1,34	1,15	0,70	0,65	1,06	1,79	2,38 no apical meristem (NAM) family protein (NAC3)
258385_at	At3g15510	1,30	1,44	0,95	1,02	0,86	1,20	0,98	0,90 no apical meristem (NAM) family protein (NAC2)
258386_at	At3g15520	0,91	0,81	0,70	0,64	0,83	0,92	0,99	1,01 peptidyl-prolyl cis-trans isomerase TLP38, chloroplast / thylakoid lumen PPI
258394_at	At3g15530	1,11	1,47	0,82	1,08	1,20	0,89	0,88	0,83 expressed protein
258399_at	At3g15540	1,02	1,18	1,35	0,98	1,04	0,88	1,14	1,13 auxin-responsive protein / indoleacetic acid-induced protein 19 (IAA19)
258387_at	At3g15550	1,04	1,08	1,01	1,13	1,02	0,95	0,96	0,89 expressed protein
257290_at	At3g15560	1,16	1,05	1,10	1,15	1,19	0,97	1,18	0,91 expressed protein
257294_at	At3g15570	1,46	1,69	1,31	0,68	0,72	0,90	1,12	1,03 phototropic-responsive NPH3 family protein
257293_at	At3g15580	1,12	1,08	1,10	0,97	0,94	0,96	1,04	1,08 autophagy 8i (APG8i)
257291_at	At3g15590	1,36	1,31	1,33	0,93	0,94	1,04	1,03	0,98 DNA-binding protein, putative
257292_s_at	At3g15600	0,95	0,97	0,94	1,02	1,00	1,00	0,94	1,06 hypothetical protein
258271_at	At3g15605	0,93	0,93	0,83	0,98	1,12	0,99	0,93	1,02 hypothetical protein
258272_at	At3g15610	1,11	0,97	0,99	1,18	0,98	0,91	0,92	0,95 transducin family protein / WD-40 repeat family protein
258227_at	At3g15620	0,84	0,90	0,98	1,04	1,02	0,99	0,92	1,10 6-4 photolyase (UVR3)
258225_at	At3g15630	0,98	1,43	1,14	0,84	0,97	0,86	0,88	0,83 expressed protein
258274_at	At3g15640	1,08	1,04	0,93	1,38	1,19	0,87	0,88	0,89 cytochrome c oxidase family protein

258270_at	At3g15650	0,88	0,74	0,74	1,37	0,88	1,08	1,22	0,86	phospholipase/carboxylesterase family protein
258273_at	At3g15660	1,15	0,90	0,82	1,23	1,25	0,88	0,91	0,91	glutaredoxin family protein
258224_at	At3g15670	0,34	0,44	0,32	1,18	1,50	1,13	1,04	1,27	late embryogenesis abundant protein, putative / LEA protein, putative
258222_at	At3g15680	1,41	1,58	1,31	1,88	1,34	0,77	0,80	0,91	zinc finger (Ran-binding) family protein
258269_at	At3g15690	0,78	0,70	0,75	0,85	0,92	0,88	0,80	0,83	biotin carboxyl carrier protein of acetyl-CoA carboxylase-related
258276_at	At3g15710	0,79	0,72	0,64	1,05	1,11	0,91	0,93	0,95	signal peptidase, putative
258252_at	At3g15720	1,60	1,74	1,78	0,89	0,77	1,19	0,99	1,09	glycoside hydrolase family 28 protein / polygalacturonase (pectinase) family
258226_at	At3g15730	0,92	0,94	0,96	0,96	0,96	0,90	0,93	0,97	phospholipase D alpha 1 / PLD alpha 1 (PLDALPHA1) (PLD1) / choline pho
258261_at	At3g15740	1,00	1,03	0,99	1,09	1,28	1,14	1,00	0,98	zinc finger (C3HC4-type RING finger) family protein
258275_at	At3g15760	0,99	1,81	1,14	0,72	0,65	1,42	2,12	1,73	expressed protein
258262_at	At3g15770	0,84	1,00	1,33	0,97	1,20	0,95	1,25	1,55	expressed protein
258263_at	At3g15780	1,25	1,12	1,07	0,83	0,83	1,42	1,55	1,45	expressed protein
258264_at	At3g15790	0,80	0,73	0,61	0,90	1,01	1,10	1,09	1,00	methyl-CpG-binding domain-containing protein
258265_at	At3g15800	0,97	0,92	0,97	1,22	1,11	1,14	0,86	0,91	glycosyl hydrolase family 17 protein
258251_at	At3g15810	0,93	1,14	1,53	1,14	1,06	1,18	1,25	1,39	expressed protein
258249_s_at	At3g15820	0,56	0,63	0,84	1,09	0,92	0,92	0,95	1,05	phosphatidic acid phosphatase-related / PAP2-related
258223_at	At3g15840	0,76	0,78	1,01	0,74	0,74	1,22	1,31	1,40	expressed protein
258250_at	At3g15850	1,40	1,24	1,51	1,01	1,00	1,28	1,24	1,35	fatty acid desaturase family protein
258266_at	At3g15860	1,02	1,09	1,00	0,94	0,92	0,97	0,88	0,94	hypothetical protein
258267_at	At3g15870	0,99	1,12	1,04	0,95	1,32	0,99	0,98	0,82	fatty acid desaturase family protein
258268_at	At3g15880	1,03	1,18	1,21	1,09	1,08	1,27	1,26	1,30	WD-40 repeat family protein
257799_at	At3g15890	1,03	1,12	1,03	0,83	1,04	1,04	0,97	1,07	protein kinase family protein
257800_at	At3g15900	0,99	1,01	1,31	0,90	0,85	1,04	1,16	1,31	expressed protein
257795_at	At3g15910	0,98	1,10	1,22	1,01	1,14	1,05	0,82	1,01	hypothetical protein
257796_at	At3g15930	1,07	0,93	1,08	0,95	0,99	1,07	1,08	0,89	pentatricopeptide (PPR) repeat-containing protein
257797_at	At3g15940	0,81	0,89	0,88	1,17	1,05	0,92	0,81	0,76	glycosyl transferase family 1 protein
257798_at	At3g15950	1,28	1,07	0,90	1,29	1,16	1,14	1,54	1,81	DNA topoisomerase-related
258313_at	At3g15970	1,04	1,09	1,09	1,07	1,08	1,16	1,07	0,98	Ran-binding protein 1 domain-containing protein / RanBP1 domain-containi
258331_at	At3g15980	0,97	0,84	0,83	0,89	0,73	0,96	0,90	0,84	coatomer protein complex, subunit beta 2 (beta prime), putative
258287_at	At3g15990	1,03	0,97	1,01	1,05	0,96	0,97	0,90	0,89	sulfate transporter, putative
258333_at	At3g16000	1,02	0,91	1,06	0,73	0,77	1,54	1,45	1,53	matrix-localized MAR DNA-binding protein-related
258334_at	At3g16010	1,24	1,04	0,97	0,93	1,15	0,82	1,09	0,87	pentatricopeptide (PPR) repeat-containing protein
258335_at	At3g16020	1,05	1,08	0,83	1,00	0,93	0,99	0,98	1,05	hypothetical protein
258337_at	At3g16040	0,97	1,03	0,94	0,93	1,05	0,98	0,96	0,97	expressed protein
258336_at	At3g16050	0,96	1,00	1,04	1,53	1,77	0,95	0,92	0,96	stress-responsive protein, putative
258286_at	At3g16060	1,14	1,29	1,59	1,27	1,40	1,11	0,90	0,98	kinesin motor family protein
258284_at	At3g16080	1,42	1,11	1,16	1,10	1,06	1,03	1,05	0,97	60S ribosomal protein L37 (RPL37C)
258328_at	At3g16090	0,91	0,96	0,83	0,92	0,87	1,04	0,97	0,97	zinc finger (C3HC4-type RING finger) family protein
258314_at	At3g16100	1,07	0,94	0,94	0,95	0,97	0,96	0,95	0,89	Ras-related GTP-binding family protein
258329_at	At3g16110	0,99	1,04	0,86	0,99	1,02	1,09	0,99	0,88	thioredoxin family protein
258339_at	At3g16120	0,98	1,15	0,98	0,89	1,04	1,10	1,02	0,98	dynein light chain, putative
258330_at	At3g16130	1,03	1,06	1,03	1,00	1,02	1,05	1,13	0,91	expressed protein
258285_at	At3g16140	0,97	1,00	0,95	0,92	0,96	0,89	0,96	1,07	photosystem I reaction center subunit VI, chloroplast, putative / PSI-H, puta
258338_at	At3g16150	1,54	2,17	1,51	0,61	0,80	0,95	0,98	0,96	L-asparaginase, putative / L-asparagine amidohydrolase, putative
257538_at	At3g16160	0,88	1,01	1,03	1,00	1,19	0,87	1,01	0,82	tesmin/TSO1-like CXC domain-containing protein
258312_at	At3g16170	1,08	1,01	0,96	1,07	1,06	0,96	0,93	0,82	acyl-activating enzyme 13 (AAE13)

258315_at	At3g16175	1,13	1,08	1,00	1,21	1,07	1,08	1,04	0,90	thioesterase family protein
258332_at	At3g16180	0,82	0,72	0,78	0,77	0,82	0,62	0,61	0,58	proton-dependent oligopeptide transport (POT) family protein
258052_at	At3g16190	0,90	0,86	0,95	0,93	1,19	0,86	0,94	0,95	isochorismatase hydrolase family protein
258050_at	At3g16200	0,93	0,81	0,92	1,12	1,38	0,83	0,75	0,83	expressed protein
258049_at	At3g16220	0,64	0,60	0,66	0,77	0,87	0,95	1,02	1,10	expressed protein
258053_at	At3g16230	0,72	0,78	0,87	0,86	1,02	1,00	0,92	0,94	expressed protein
258054_at	At3g16240	1,09	1,11	1,07	1,19	1,10	0,92	0,86	0,84	delta tonoplast integral protein (delta-TIP)
258055_at	At3g16250	1,08	1,04	1,19	0,71	0,76	1,30	1,21	1,27	ferredoxin-related
258051_at	At3g16260	0,99	1,11	1,08	0,70	0,67	1,14	1,18	1,03	metallo-beta-lactamase family protein
259330_at	At3g16270	0,77	0,74	0,83	0,97	1,06	0,99	0,71	0,76	expressed protein
259374_at	At3g16280	1,15	0,96	1,16	0,96	1,02	1,14	1,03	1,20	AP2 domain-containing transcription factor TINY, putative
258048_at	At3g16290	1,02	0,95	0,95	0,84	0,83	1,14	1,18	1,20	FtsH protease, putative
259378_at	At3g16310	1,43	1,27	1,24	1,02	0,91	1,01	0,89	0,93	mitotic phosphoprotein N' end (MPPN) family protein
259376_at	At3g16320	1,00	1,05	1,07	0,98	1,27	1,13	1,09	0,94	cell division cycle family protein / CDC family protein
257517_at	At3g16330	0,90	1,04	0,91	1,33	1,27	0,97	0,94	1,18	expressed protein
259380_at	At3g16340	1,18	1,10	1,26	1,06	0,82	1,04	1,44	1,38	ABC transporter family protein
259379_at	At3g16350	0,67	1,33	0,87	1,20	1,11	0,90	0,99	1,16	myb family transcription factor
259329_at	At3g16360	1,53	1,53	1,39	0,76	1,08	1,03	1,20	0,91	phosphotransfer family protein
259375_at	At3g16370	1,26	1,24	1,26	1,07	0,81	1,00	0,86	0,91	GDSL-motif lipase/hydrolase family protein
259377_at	At3g16380	0,99	1,07	0,97	0,92	0,95	0,84	1,08	0,89	polyadenylate-binding protein, putative / PABP, putative
259381_s_at	At3g16390	0,94	0,94	0,97	0,94	0,97	0,74	0,85	0,82	jacalin lectin family protein
259382_s_at	At3g16430	1,31	0,97	0,99	1,35	1,30	0,96	1,00	0,99	jacalin lectin family protein
259328_at	At3g16440	0,98	1,03	0,79	2,35	2,16	1,17	1,27	1,23	jacalin lectin family protein
259384_at	At3g16450	1,06	0,94	1,04	1,36	1,22	1,03	1,18	1,19	jacalin lectin family protein
259327_at	At3g16460	0,86	0,83	0,84	1,22	1,11	0,95	1,09	1,07	jacalin lectin family protein
259383_at	At3g16470	1,40	1,18	1,15	1,20	0,90	0,99	0,95	0,94	jacalin lectin family protein
259326_at	At3g16480	1,06	0,99	1,02	0,94	0,94	0,97	0,89	0,75	mitochondrial processing peptidase alpha subunit, putative
257229_at	At3g16490	0,97	1,01	0,96	1,11	1,08	1,15	1,07	1,20	calmodulin-binding family protein
257232_at	At3g16500	1,55	1,40	1,17	1,17	0,94	0,95	1,00	1,03	auxin-responsive AUX/IAA family protein
257205_at	At3g16520	1,06	1,49	1,79	1,04	1,19	0,84	0,85	0,76	UDP-glucoronosyl/UDP-glucosyl transferase family protein
257206_at	At3g16530	1,01	0,77	0,77	0,51	0,53	0,74	0,57	0,60	legume lectin family protein
257230_at	At3g16540	1,08	1,00	1,03	1,05	1,05	1,01	1,07	0,80	DegP protease, putative
257231_at	At3g16550	1,03	1,01	0,86	1,15	1,07	1,05	0,95	0,92	DegP protease, putative
258437_at	At3g16560	0,67	0,90	1,19	0,67	0,65	1,23	1,29	1,31	protein phosphatase 2C-related / PP2C-related
258432_at	At3g16570	0,80	0,78	0,76	1,09	1,16	0,75	0,76	0,82	rapid alkalinization factor (RALF) family protein
258431_at	At3g16580	0,93	0,95	0,93	1,10	0,96	1,02	0,96	0,96	F-box family protein
258427_at	At3g16600	0,97	0,92	1,07	0,97	0,90	0,94	0,89	1,08	SNF2 domain-containing protein / helicase domain-containing protein / RIN
258428_at	At3g16610	1,03	0,92	0,99	0,87	1,05	1,01	1,00	0,89	pentatricopeptide (PPR) repeat-containing protein
258429_at	At3g16620	1,02	1,08	1,29	1,01	1,15	0,95	0,97	1,06	chloroplast outer membrane protein, putative
258381_at	At3g16630	1,01	0,97	1,04	1,01	1,03	0,98	0,99	1,01	kinesin motor family protein
258433_at	At3g16640	1,04	1,04	0,99	1,08	1,01	0,90	0,91	0,88	translationally controlled tumor family protein
258380_at	At3g16650	1,32	1,17	1,13	0,87	0,98	1,09	1,19	0,85	PP1/PP2A phosphatases pleiotropic regulator 2 (PRL2)
258418_at	At3g16660	2,06	1,86	1,45	1,19	1,22	1,38	1,29	1,33	expressed protein
258419_at	At3g16670	2,87	2,07	1,59	0,79	0,83	0,78	0,75	0,80	expressed protein
258420_at	At3g16680	1,05	0,96	1,08	1,12	1,03	0,97	1,05	0,94	expressed protein
258421_at	At3g16690	0,81	0,91	0,83	0,88	0,77	1,01	1,18	1,09	nodulin MtN3 family protein

258379_at	At3g16700	0,71	0,60	0,66	0,96	0,84	0,90	0,93	0,97	fumarylacetoacetate hydrolase family protein
258422_at	At3g16710	0,92	1,02	1,03	0,91	0,81	1,11	1,07	0,93	pentatricopeptide (PPR) repeat-containing protein
258436_at	At3g16720	0,84	0,74	0,78	0,83	0,66	0,89	0,93	1,00	zinc finger (C3HC4-type RING finger) family protein
258423_at	At3g16730	0,88	1,00	0,92	0,94	0,94	0,99	0,87	0,84	expressed protein
258435_at	At3g16740	0,93	0,99	1,29	1,15	1,10	0,90	0,85	1,13	F-box family protein
258424_at	At3g16750	1,03	0,98	0,99	0,61	0,73	0,87	1,01	1,21	expressed protein
258425_at	At3g16760	0,89	0,78	0,82	0,98	1,25	0,92	0,78	0,88	tetratricopeptide repeat (TPR)-containing protein
258434_at	At3g16770	1,07	1,00	1,00	0,59	0,70	0,80	0,80	0,82	AP2 domain-containing protein RAP2.3 (RAP2.3)
258410_at	At3g16780	1,49	1,02	1,03	1,28	1,34	1,01	0,94	1,04	60S ribosomal protein L19 (RPL19B)
258430_at	At3g16785	0,85	0,75	0,73	0,94	1,02	1,06	1,00	0,95	phospholipase D zeta1 / PLDzeta1 (PLDP1)
258426_at	At3g16790	0,93	0,73	0,68	0,97	1,12	0,76	0,99	0,71	phospholipase D zeta1 / PLDzeta1 (PLDP1)
257650_at	At3g16800	0,89	1,11	1,41	0,87	0,66	1,32	2,08	2,38	protein phosphatase 2C, putative / PP2C, putative
257652_at	At3g16810	1,32	1,12	1,18	0,96	0,96	1,09	1,08	1,11	pumilio/Puf RNA-binding domain-containing protein
257553_at	At3g16830	1,00	0,93	1,12	0,72	0,82	0,98	0,94	1,16	WD-40 repeat family protein
257648_at	At3g16840	0,92	0,92	0,97	0,88	0,92	0,97	0,93	1,20	DEAD/DEAH box helicase, putative (RH13)
257651_at	At3g16850	1,03	1,00	0,88	0,70	0,82	0,91	0,79	0,85	glycoside hydrolase family 28 protein / polygalacturonase (pectinase) family
257649_at	At3g16855	0,92	0,94	1,04	1,15	1,06	0,91	0,98	1,07	two-component responsive regulator family protein / response regulator fam
256790_at	At3g16857	0,95	0,82	1,10	1,04	1,11	1,03	1,03	1,06	two-component responsive regulator family protein / response regulator fam
256763_at	At3g16860	0,79	0,82	1,01	1,04	0,93	1,13	1,38	1,50	phytochelatin synthetase-related
257571_at	At3g16870	0,90	0,68	0,94	0,84	1,01	0,97	0,87	0,96	zinc finger (GATA type) family protein
257572_at	At3g16880	0,92	1,06	0,95	0,96	1,03	0,95	1,06	1,00	F-box protein-related
257928_at	At3g16890	0,97	0,93	0,94	0,93	0,96	0,97	1,01	1,14	pentatricopeptide (PPR) repeat-containing protein
257880_at	At3g16910	0,99	1,03	1,13	0,80	0,77	1,09	1,07	1,19	AMP-dependent synthetase and ligase family protein
257896_at	At3g16920	1,45	1,39	1,30	0,97	1,03	0,89	0,98	1,09	glycoside hydrolase family 19 protein
257882_at	At3g16930	0,99	1,00	0,86	0,93	1,11	0,93	0,89	1,01	hypothetical protein
257883_at	At3g16940	0,85	1,05	1,37	0,72	0,84	1,12	1,37	1,49	calmodulin-binding protein
257895_at	At3g16950	0,91	0,89	0,78	0,87	0,89	0,87	0,80	0,83	dihydrolipoamide dehydrogenase 1, plastidic / lipoamide dehydrogenase 1 (
257884_at	At3g16960	1,00	0,98	1,07	1,01	0,91	0,96	0,93	0,98	hypothetical protein
257885_at	At3g16970	0,94	0,98	1,02	0,98	1,03	1,02	1,10	0,99	self-incompatibility protein-related
257929_at	At3g16980	1,20	1,21	1,19	1,04	1,04	1,15	0,98	0,95	DNA-directed RNA polymerase II, putative
257888_at	At3g16990	0,88	0,93	1,12	1,19	1,08	0,96	0,92	0,98	TENA/THI-4 family protein
257893_at	At3g17000	1,03	1,06	1,12	1,04	1,07	1,05	0,97	1,09	ubiquitin-conjugating enzyme, putative
257930_at	At3g17010	0,86	1,03	0,87	1,01	1,11	0,84	0,87	0,85	transcriptional factor B3 family protein
257892_at	At3g17020	0,79	0,73	0,77	0,95	1,24	0,84	0,88	0,85	universal stress protein (USP) family protein
257931_at	At3g17030	0,99	1,03	1,10	0,84	0,97	1,01	1,08	0,85	expressed protein
257932_at	At3g17040	1,14	1,70	1,29	0,80	0,77	1,15	1,17	1,35	tetratricopeptide repeat (TPR)-containing protein
257905_at	At3g17050	0,97	0,84	0,96	0,90	1,20	0,95	0,82	1,05	---
257886_at	At3g17060	0,97	1,28	0,99	0,97	0,92	0,97	1,08	0,92	pectinesterase family protein
257889_at	At3g17080	0,97	0,98	1,08	0,99	1,12	0,99	1,01	1,15	self-incompatibility protein-related
257887_at	At3g17090	1,16	1,10	1,05	0,99	1,22	0,90	1,03	1,02	protein phosphatase 2C family protein / PP2C family protein
257894_at	At3g17100	0,96	0,89	0,92	0,80	0,81	0,78	0,75	0,79	expressed protein
257874_at	At3g17110	1,08	1,16	1,01	1,08	1,38	1,22	1,34	1,01	---
257875_at	At3g17120	0,94	1,27	0,84	1,24	0,94	0,87	1,13	1,03	expressed protein
257876_at	At3g17130	1,11	1,07	1,12	1,25	1,18	0,99	0,98	1,12	invertase/pectin methylesterase inhibitor family protein
257877_at	At3g17140	0,92	1,10	0,95	1,12	1,11	1,13	0,96	0,98	invertase inhibitor-related
257878_at	At3g17150	0,78	0,98	0,88	1,06	1,02	1,07	0,96	0,98	invertase/pectin methylesterase inhibitor family protein

257879_at	At3g17160	0,80	0,79	0,79	1,02	1,02	0,84	1,01	1,11	expressed protein
257891_at	At3g17170	0,99	0,93	1,00	0,56	0,60	1,05	1,03	1,11	ribosomal protein S6 family protein (RFC3)
257881_at	At3g17180	0,97	1,08	1,04	1,09	1,02	1,08	1,08	1,15	serine carboxypeptidase S10 family protein
257133_at	At3g17190	1,00	0,95	1,05	0,85	1,02	0,96	0,88	0,97	hypothetical protein
258412_at	At3g17210	0,79	0,71	0,68	1,06	1,28	0,80	0,73	0,68	stable protein 1-related
258465_at	At3g17220	0,92	1,00	1,01	1,07	1,09	0,95	0,95	0,96	invertase/pectin methylesterase inhibitor family protein
258438_at	At3g17230	1,02	0,99	1,07	0,98	1,06	0,97	1,03	0,97	invertase/pectin methylesterase inhibitor family protein
258439_at	At3g17240	0,97	0,80	0,87	1,20	1,22	1,04	0,90	1,02	dihydrolipoamide dehydrogenase 2, mitochondrial / lipoamide dehydrogena
258440_at	At3g17250	0,77	1,16	1,00	0,99	1,08	0,82	1,07	1,05	protein phosphatase 2C-related / PP2C-related
258441_at	At3g17260	0,95	1,06	0,89	1,04	0,99	1,04	0,98	1,04	hAT-like transposase family (hobo/Ac/Tam3)
258411_at	At3g17280	1,05	1,19	1,00	1,09	1,08	1,07	1,07	0,79	F-box family protein
258459_at	At3g17290	0,96	1,14	1,01	1,09	1,00	0,97	1,05	1,11	---
258413_at	At3g17300	1,16	1,16	0,82	1,29	1,14	1,02	0,91	0,75	expressed protein
258416_at	At3g17310	0,83	0,82	0,81	1,11	1,23	0,97	1,01	0,96	methyltransferase family protein
258460_at	At3g17330	1,08	1,28	1,65	1,32	1,23	0,91	1,16	1,46	expressed protein
258461_at	At3g17340	1,05	0,87	0,79	1,16	0,91	0,76	0,92	0,69	importin-related
258462_at	At3g17350	0,95	0,94	1,06	1,27	1,17	0,86	0,90	0,68	expressed protein
258464_at	At3g17360	1,02	0,99	1,03	1,12	1,23	0,99	1,19	1,08	kinesin motor protein-related
258417_at	At3g17365	0,94	1,06	1,27	1,11	1,14	0,86	0,99	0,85	expressed protein
258414_at	At3g17380	1,21	1,13	0,92	1,51	1,26	0,67	0,61	0,43	meprin and TRAF homology domain-containing protein / MATH domain-cor
258415_at	At3g17390	1,01	0,94	1,02	1,00	1,05	1,02	1,01	1,09	S-adenosylmethionine synthetase, putative
258463_at	At3g17410	0,84	0,88	0,84	0,76	0,89	1,02	0,93	0,89	serine/threonine protein kinase, putative
257295_at	At3g17420	1,40	1,28	1,10	1,51	1,18	0,95	1,09	1,28	protein kinase family protein
257238_at	At3g17430	0,90	0,72	0,82	0,98	0,91	0,99	0,91	0,86	phosphate translocator-related
257269_at	At3g17440	1,18	0,95	1,19	0,95	1,09	1,08	1,01	1,10	novel plant SNARE 13 (NPSN13)
257268_at	At3g17450	1,04	1,10	1,03	0,76	0,81	1,17	1,17	1,01	hAT dimerisation domain-containing protein
258404_at	At3g17465	1,30	1,19	1,09	1,10	1,24	0,96	0,91	0,99	ribosomal protein L3 family protein
258375_at	At3g17470	1,20	1,60	1,86	0,77	0,68	1,33	1,42	1,43	RelA/SpoT domain-containing protein / calcium-binding EF-hand family prot
258378_at	At3g17490	1,04	1,02	1,07	1,09	1,16	0,93	1,04	0,96	F-box family protein
258350_at	At3g17510	1,47	1,59	1,32	1,20	0,93	1,57	1,51	1,92	CBL-interacting protein kinase 1 (CIPK1)
258347_at	At3g17520	0,16	0,20	0,31	0,94	0,94	1,04	1,02	0,97	late embryogenesis abundant domain-containing protein / LEA domain-cont
258405_at	At3g17590	1,14	1,18	1,20	1,12	1,19	0,91	0,92	0,93	transcription regulatory protein SNF5, putative (BSH)
258352_at	At3g17600	0,95	1,04	1,02	0,98	1,15	0,91	1,06	0,97	auxin-responsive protein, putative
258349_at	At3g17610	1,54	1,54	1,99	1,28	1,15	1,58	1,48	1,09	bZIP transcription factor family protein / HY5-like protein (HYH)
258406_at	At3g17615	1,23	1,15	0,99	1,19	1,17	0,88	1,04	1,03	rhomboid family protein / zinc finger protein-related
258407_at	At3g17620	1,05	0,96	1,05	1,00	1,23	0,95	1,01	0,76	F-box family protein
258408_at	At3g17630	1,00	1,00	0,90	1,08	0,77	1,02	1,10	0,85	cation/hydrogen exchanger, putative (CHX19)
258409_at	At3g17640	1,25	1,25	1,10	1,09	1,19	0,87	0,86	1,03	leucine-rich repeat family protein
258353_s_at	At3g17650	1,17	1,20	1,10	0,66	0,69	1,20	1,14	1,08	oligopeptide transporter OPT family protein
258376_at	At3g17680	1,34	1,46	1,49	1,13	1,43	0,95	0,92	0,86	expressed protein
258377_at	At3g17690	1,65	1,89	1,37	1,80	1,72	1,09	1,42	1,05	cyclic nucleotide-binding transporter 2 / CNBT2 (CNGC19)
258351_at	At3g17700	0,52	0,48	0,90	0,84	0,59	0,86	1,22	1,39	cyclic nucleotide-binding transporter 1 / CNBT1 (CNGC20)
258348_at	At3g17710	0,68	1,07	1,04	0,85	1,03	0,92	0,97	1,14	F-box family protein
257862_s_at	At3g17720	0,99	0,86	0,97	1,01	1,03	1,00	1,05	1,11	pyridoxal-dependent decarboxylase family protein
257863_at	At3g17730	0,95	0,93	0,95	0,95	0,95	1,12	0,99	1,26	no apical meristem (NAM) family protein
257864_at	At3g17740	1,25	1,39	1,39	0,95	0,96	1,11	1,05	1,09	expressed protein

257865_at	At3g17750	1,01	0,92	1,06	0,81	0,90	0,99	0,92	0,86 protein kinase family protein
257866_at	At3g17770	0,98	1,66	1,86	0,93	1,07	1,31	1,72	1,56 dihydroxyacetone kinase family protein
257867_at	At3g17780	0,89	0,85	0,72	0,96	0,96	0,98	0,83	0,84 expressed protein
258158_at	At3g17790	1,25	1,41	1,38	1,80	1,68	1,32	1,08	0,95 acid phosphatase type 5 (ACP5)
258188_at	At3g17800	0,96	1,22	1,64	1,12	1,20	1,22	1,42	1,36 expressed protein
258162_at	At3g17810	0,86	0,83	0,91	1,53	1,62	0,84	0,89	0,83 dihydroorotate dehydrogenase family protein / dihydroorotate oxidase famil
258160_at	At3g17820	1,16	0,99	1,02	1,30	1,31	0,89	0,95	0,93 glutamine synthetase (GS1)
258220_at	At3g17830	1,18	0,86	0,79	0,78	0,88	1,03	0,92	0,87 DNAJ heat shock family protein
258159_at	At3g17840	0,96	0,90	1,00	1,18	1,14	0,86	0,84	0,93 leucine-rich repeat transmembrane protein kinase, putative
258186_s_at	At3g17850	0,78	0,82	0,97	0,86	0,88	0,91	1,02	1,17 protein kinase, putative
258189_at	At3g17860	0,68	0,76	0,91	0,87	0,86	0,86	0,92	1,00 expressed protein
258187_at	At3g17870	0,94	0,99	1,00	0,79	1,01	1,06	1,08	1,18 tetratricoredoxin (TDX)
258219_at	At3g17880	0,97	0,90	1,01	0,87	0,92	1,01	0,95	1,07 tetratricoredoxin (TDX)
258211_at	At3g17890	1,03	0,87	0,88	0,91	0,98	0,94	0,91	1,05 expressed protein
258165_at	At3g17900	0,90	0,83	1,06	0,87	0,94	0,93	0,81	0,88 expressed protein
258164_at	At3g17910	0,87	0,90	0,90	0,98	1,11	0,96	0,84	0,91 surfeit 1 (SURF1)
258212_at	At3g17920	1,18	1,31	1,65	0,94	0,88	1,20	1,55	1,40 leucine-rich repeat family protein
258161_at	At3g17930	1,09	1,00	1,16	0,83	0,72	1,26	1,21	1,15 expressed protein
258163_at	At3g17940	0,97	0,93	0,94	1,43	1,14	0,85	0,83	0,87 aldose 1-epimerase family protein
258213_at	At3g17950	1,04	1,12	1,03	1,01	0,94	0,77	0,97	0,76 expressed protein
258214_at	At3g17960	0,89	0,85	0,93	0,86	0,75	0,85	0,90	1,11 chloroplast outer membrane translocon subunit, putative
258215_at	At3g17970	0,96	0,88	0,94	0,79	0,70	1,01	0,90	0,75 chloroplast outer membrane translocon subunit, putative
258216_at	At3g17980	1,04	0,89	1,31	0,95	0,96	1,04	1,10	1,00 C2 domain-containing protein
258217_at	At3g17990	0,70	0,74	0,83	0,82	0,81	0,77	0,84	0,67 phosphoethanolamine N-methyltransferase 1 / PEAMT 1 (NMT1)
258218_at	At3g18000	0,73	0,69	0,69	0,67	0,58	0,82	0,88	0,82 phosphoethanolamine N-methyltransferase 1 / PEAMT 1 (NMT1)
257897_at	At3g18030	1,06	1,24	1,42	1,44	1,47	1,00	0,90	0,97 halotolerance protein (HAL3A)
258141_at	At3g18035	0,77	0,69	0,70	0,74	0,74	1,00	0,97	1,02 histone H1/H5 family protein
258123_at	At3g18040	0,89	0,79	0,89	0,86	1,00	0,95	1,02	1,20 mitogen-activated protein kinase, putative / MAPK, putative (MPK9)
258156_at	At3g18050	0,75	0,79	0,91	0,56	0,55	0,78	0,91	0,98 expressed protein
258146_at	At3g18060	1,10	1,07	1,17	1,20	1,23	0,93	0,84	0,82 transducin family protein / WD-40 repeat family protein
258147_at	At3g18070	0,79	0,77	0,74	0,89	1,09	0,98	0,99	0,99 glycosyl hydrolase family 1 protein
258151_at	At3g18080	0,55	0,47	0,55	0,81	0,91	0,76	0,75	0,74 glycosyl hydrolase family 1 protein
258148_s_at	At3g18090	0,91	1,01	1,20	1,13	1,06	1,06	1,00	0,94 DNA-directed RNA polymerase family protein
258157_at	At3g18100	1,22	1,17	1,24	0,85	0,85	1,25	1,24	1,01 myb family transcription factor (MYB4R1)
258149_at	At3g18110	0,97	0,96	0,98	0,63	0,59	1,08	0,99	1,14 pentatricopeptide (PPR) repeat-containing protein
258142_at	At3g18120	0,94	1,08	1,03	1,03	0,96	0,86	0,95	0,95 F-box family protein-related
258155_at	At3g18130	1,49	1,26	1,09	1,06	0,76	1,08	1,08	0,98 guanine nucleotide-binding family protein / activated protein kinase C recep
258150_at	At3g18160	1,21	1,16	1,11	1,05	0,97	1,02	1,11	0,93 peroxin-3 family protein
258153_at	At3g18165	1,18	1,07	1,09	0,95	0,97	1,18	0,92	0,93 expressed protein
258143_at	At3g18170	0,75	0,80	0,89	1,21	1,31	1,07	1,20	1,21 expressed protein
258144_at	At3g18180	0,97	1,03	0,99	1,13	1,25	1,03	0,96	1,02 hypothetical protein
258152_at	At3g18190	1,18	1,13	1,07	1,27	1,01	0,94	0,87	0,95 chaperonin, putative
258145_at	At3g18200	1,10	1,36	1,13	0,95	1,09	1,09	1,05	1,14 nodulin MtN21 family protein
258154_at	At3g18210	1,07	1,16	1,07	1,02	0,93	0,95	1,06	1,05 oxidoreductase, 2OG-Fe(II) oxygenase family protein
258124_at	At3g18215	1,13	1,17	1,04	0,69	0,71	0,90	0,82	0,85 expressed protein
257065_at	At3g18220	1,01	0,92	1,16	0,88	1,30	0,81	1,02	1,09 phosphatidic acid phosphatase family protein / PAP2 family protein

257060_at	At3g18230	0,67	0,69	0,67	1,03	0,92	0,93	0,86	0,77	octicosapeptide/Phox/Bem1p (PB1) domain-containing protein
257063_s_at	At3g18240	1,15	1,20	1,07	1,06	1,20	0,98	0,93	0,94	expressed protein
257061_at	At3g18250	1,77	1,90	1,80	1,68	1,41	1,33	1,27	1,29	expressed protein
257064_at	At3g18260	1,07	1,22	1,10	1,15	1,19	0,89	1,03	0,94	reticulon family protein (RTNLB9)
257067_at	At3g18270	0,79	0,66	0,75	0,78	0,82	0,88	0,87	0,90	mandelate racemase/muconate lactonizing enzyme family protein
257066_at	At3g18280	2,56	1,88	1,27	3,31	2,73	1,11	0,99	0,97	protease inhibitor/seed storage/lipid transfer protein (LTP) family protein
257062_at	At3g18290	1,15	1,35	1,42	1,06	0,87	2,12	1,88	1,73	zinc finger protein-related
257728_at	At3g18295	0,84	0,90	0,98	1,27	1,36	1,06	0,87	0,92	expressed protein
257716_at	At3g18300	0,98	0,74	0,88	1,21	1,37	0,97	1,05	1,25	expressed protein
257721_at	At3g18310	0,94	0,85	0,92	0,90	0,88	0,92	0,91	0,81	expressed protein
257733_at	At3g18350	0,77	0,88	1,14	0,86	1,07	0,98	1,01	1,01	expressed protein
257726_at	At3g18360	0,87	1,01	1,00	0,91	1,24	1,01	0,97	1,10	VQ motif-containing protein
257734_at	At3g18370	0,83	0,66	0,80	0,84	0,80	1,00	0,91	1,00	C2 domain-containing protein
257727_at	At3g18380	0,96	0,99	1,14	1,06	1,01	1,00	0,91	0,98	expressed protein
257717_at	At3g18390	1,17	1,18	1,45	0,68	0,67	1,33	1,39	1,24	expressed protein
257718_at	At3g18400	1,11	1,21	1,19	0,99	1,24	1,02	1,29	1,28	no apical meristem (NAM) family protein
257730_at	At3g18420	1,01	0,69	0,63	0,71	0,76	0,82	0,88	0,82	tetratricopeptide repeat (TPR)-containing protein
257731_at	At3g18430	1,07	1,12	1,14	1,15	1,11	0,96	1,04	1,11	calcium-binding EF hand family protein
257719_at	At3g18440	0,91	0,92	0,91	1,07	1,25	1,15	1,03	1,14	expressed protein
257720_at	At3g18450	0,98	1,04	0,98	1,05	1,01	1,08	0,95	1,10	expressed protein
257550_at	At3g18460	1,02	1,06	1,08	1,04	1,00	0,95	0,81	1,10	hypothetical protein
257732_at	At3g18480	0,87	0,78	0,85	0,95	0,96	1,17	0,99	1,05	CCAAT displacement protein-related / CDP-related
257722_at	At3g18490	0,93	0,99	0,78	1,58	1,44	0,85	0,75	0,64	aspartyl protease family protein
257723_at	At3g18500	1,01	0,89	0,78	0,76	0,60	1,04	0,95	0,75	nocturnin-related
257724_at	At3g18510	1,53	1,16	1,02	0,92	1,05	1,00	0,96	0,87	expressed protein
257729_at	At3g18520	0,95	1,11	1,05	0,99	0,97	0,79	0,88	1,03	histone deacetylase family protein
257808_at	At3g18524	1,12	1,09	1,12	0,99	0,97	1,09	0,85	0,98	zinc finger (DHHC type) family protein
257725_at	At3g18525	1,23	1,21	1,32	0,86	0,95	1,07	1,22	1,01	DNA mismatch repair protein MSH2 (MSH2)
256826_at	At3g18540	0,95	1,10	0,95	1,00	0,97	0,93	0,97	1,12	expressed protein
256799_at	At3g18560	0,92	0,90	0,87	1,18	1,10	0,88	0,95	0,94	expressed protein
256827_at	At3g18570	0,83	0,75	0,79	0,98	1,15	0,79	0,98	1,06	glycine-rich protein / oleosin
256797_at	At3g18600	1,52	1,24	1,10	1,18	0,86	0,97	0,99	1,00	DEAD/DEAH box helicase, putative
256852_at	At3g18610	1,73	1,44	1,27	0,91	1,09	1,04	1,04	1,17	nucleolin, putative
256798_at	At3g18630	1,00	1,01	1,30	1,02	1,10	1,01	0,94	0,98	uracil DNA glycosylase family protein
256853_at	At3g18640	1,01	0,97	1,12	0,85	1,02	1,10	1,00	1,10	zinc finger protein-related
257549_at	At3g18650	1,07	0,98	1,01	0,98	1,08	0,93	1,13	1,12	MADS-box family protein
257757_at	At3g18660	0,97	1,00	1,02	0,92	1,05	1,00	0,99	1,02	glycogenin glucosyltransferase (glycogenin)-related
257806_at	At3g18670	0,88	1,13	1,04	1,02	1,12	1,20	1,01	0,96	ankyrin repeat family protein
257756_at	At3g18680	0,98	0,92	1,04	0,62	0,60	0,92	0,89	0,97	aspartate/glutamate/uridylate kinase family protein
257751_at	At3g18690	0,75	0,79	0,65	1,09	1,02	0,77	0,77	0,99	VQ motif-containing protein
257747_at	At3g18700	1,03	0,95	1,01	1,07	0,91	0,97	0,93	0,90	hypothetical protein
257748_at	At3g18710	0,55	0,83	1,00	0,40	0,68	1,01	1,12	0,93	U-box domain-containing protein
257752_at	At3g18720	1,09	1,00	0,93	1,06	0,97	0,98	1,00	0,72	F-box family protein
257753_at	At3g18740	1,35	1,20	1,16	1,19	1,15	1,00	0,94	0,96	60S ribosomal protein L30 (RPL30C)
257801_at	At3g18750	1,35	1,16	1,06	1,03	1,01	1,09	1,15	1,20	protein kinase family protein
257755_at	At3g18760	1,49	1,21	1,10	1,09	1,26	0,90	0,95	0,93	ribosomal protein S6 family protein

257802_at	At3g18770	1,06	1,02	1,19	0,87	0,99	0,99	0,90	0,81	expressed protein
257749_at	At3g18780	1,20	1,07	1,02	1,07	1,06	1,07	0,93	0,95	actin 2 (ACT2)
257803_at	At3g18790	1,10	0,92	1,14	0,84	0,83	1,17	1,18	1,10	expressed protein
257750_at	At3g18800	1,06	0,97	1,15	0,94	0,94	0,94	0,92	1,00	expressed protein
257804_at	At3g18810	0,98	1,01	1,06	0,99	0,96	1,07	1,10	1,10	protein kinase family protein
257754_at	At3g18820	0,94	0,78	0,75	1,20	1,10	0,99	0,92	0,89	Ras-related GTP-binding protein, putative
257805_at	At3g18830	0,77	0,80	0,72	0,72	0,67	0,86	0,86	0,96	mannitol transporter, putative
256651_at	At3g18840	1,00	1,06	1,19	1,07	1,02	1,07	1,08	0,97	pentatricopeptide (PPR) repeat-containing protein
256652_at	At3g18850	0,88	1,00	0,90	0,85	0,80	0,75	0,84	0,84	phospholipid/glycerol acyltransferase family protein
256657_at	At3g18860	1,12	1,10	1,04	1,06	1,02	1,03	1,01	0,92	transducin family protein / WD-40 repeat family protein
256653_at	At3g18870	1,06	1,00	1,05	0,92	0,96	1,03	0,84	1,05	mitochondrial transcription termination factor-related / mTERF-related
256654_at	At3g18880	0,90	1,13	1,13	1,26	1,30	1,05	0,99	0,99	ribosomal protein S17 family protein
256655_at	At3g18890	0,91	0,89	0,79	0,72	0,80	1,02	0,95	0,99	expressed protein
256656_at	At3g18900	0,94	0,99	1,01	0,93	1,06	0,91	1,04	1,02	expressed protein
256943_at	At3g18910	0,86	0,81	0,94	0,99	0,97	0,78	0,79	0,91	F-box family protein
256948_at	At3g18920	1,03	1,05	1,05	0,91	0,90	1,04	0,94	0,93	zinc finger (C3HC4-type RING finger) family protein
256917_at	At3g18930	1,21	1,18	1,28	0,68	1,05	1,05	0,95	0,84	zinc finger (C3HC4-type RING finger) family protein
256921_at	At3g18940	1,10	0,99	1,14	1,11	1,34	0,85	0,84	0,81	clast3-related
256918_s_at	At3g18960	1,16	1,36	1,60	1,09	1,18	0,89	0,70	0,77	transcriptional factor B3 family protein
256919_at	At3g18970	1,28	1,02	1,03	0,95	0,86	1,12	1,03	0,69	pentatricopeptide (PPR) repeat-containing protein
256920_at	At3g18980	0,79	0,78	0,75	1,03	1,12	0,84	0,92	0,82	F-box family protein
256944_at	At3g18990	0,79	0,72	0,80	0,96	1,10	0,97	0,99	1,02	transcriptional factor B3 family protein
256892_at	At3g19000	1,04	1,16	1,52	0,91	0,79	1,08	1,08	1,10	oxidoreductase, 2OG-Fe(II) oxygenase family protein
256922_at	At3g19010	0,70	0,69	1,15	0,85	0,95	0,97	0,89	0,98	oxidoreductase, 2OG-Fe(II) oxygenase family protein
256945_at	At3g19020	1,01	1,05	0,98	0,97	1,28	1,04	1,01	0,85	leucine-rich repeat family protein / extensin family protein
256891_at	At3g19030	0,96	0,81	0,99	1,01	0,70	1,12	1,13	1,00	expressed protein
256946_at	At3g19040	0,86	0,86	1,28	1,17	1,12	1,05	0,96	1,01	ubiquitin family protein / DNA-binding bromodomain-containing protein
256947_at	At3g19050	1,05	0,92	0,94	1,10	1,03	1,14	1,02	1,02	kinesin motor protein-related
256949_at	At3g19070	0,96	1,09	0,99	1,07	0,94	1,13	0,86	1,01	cell wall protein-related
256950_at	At3g19080	1,02	1,11	1,02	1,13	1,17	0,88	1,05	1,21	SWIB complex BAF60b domain-containing protein
256951_at	At3g19085	0,97	0,92	1,01	0,98	1,20	0,96	1,04	0,97	hypothetical protein
256638_at	At3g19090	0,81	0,97	0,93	0,94	1,50	1,02	0,91	1,17	RNA-binding protein, putative
257024_at	At3g19100	0,88	0,87	1,06	0,88	0,99	0,90	0,96	0,92	calcium-dependent protein kinase, putative / CDPK, putative
257030_at	At3g19110	0,96	0,96	0,94	0,93	1,04	0,91	1,05	1,11	---
257036_at	At3g19120	0,77	0,97	0,77	1,06	1,26	1,01	0,73	0,91	expressed protein
257037_at	At3g19130	0,74	0,64	0,69	1,00	1,27	0,95	0,92	1,00	RNA-binding protein, putative
257032_at	At3g19140	1,00	1,07	0,96	1,03	1,02	0,94	0,90	1,02	hypothetical protein
257039_at	At3g19160	0,87	1,05	0,97	0,94	1,15	0,88	1,04	0,92	adenylate isopentenyltransferase 8 / adenylate dimethylallyltransferase / cy
257033_at	At3g19170	1,01	0,84	0,88	0,75	0,69	1,15	1,21	1,22	peptidase M16 family protein / insulinase family protein
257034_at	At3g19180	1,04	1,08	0,92	0,99	1,03	1,12	0,84	0,96	protein coding
257025_at	At3g19190	0,85	0,94	1,37	0,69	0,69	0,96	1,29	1,67	expressed protein
257026_at	At3g19200	0,90	1,65	1,49	1,23	1,69	0,85	1,04	0,95	hypothetical protein
257027_at	At3g19210	0,94	1,03	0,97	0,92	1,03	1,08	1,02	1,14	DNA repair protein RAD54, putative
257040_at	At3g19220	1,04	1,05	1,02	0,83	0,84	0,99	1,12	0,83	expressed protein
257028_at	At3g19230	0,99	1,03	0,86	0,96	1,02	1,02	1,10	0,99	leucine-rich repeat family protein
257029_at	At3g19240	0,78	1,13	1,50	0,97	0,80	1,22	1,48	1,53	expressed protein

257038_at	At3g19260	0,92	0,83	0,79	1,02	1,13	0,82	0,77	0,75	longevity-assurance (LAG1) family protein
257035_at	At3g19270	1,10	1,10	1,01	1,23	1,21	1,12	0,95	1,01	cytochrome P450 family protein
257031_at	At3g19280	1,19	1,23	1,04	0,90	1,11	1,12	0,94	1,17	glycoprotein 3-alpha-L-fucosyltransferase A / FucTA / core alpha-(1,3)-fuco
258026_at	At3g19290	1,11	1,16	1,28	0,73	0,90	1,26	1,16	1,48	ABA-responsive element-binding protein 2 (AREB2)
258010_at	At3g19300	0,82	1,12	1,06	1,01	0,92	0,92	1,07	0,89	protein kinase family protein
258012_at	At3g19310	1,03	0,97	0,89	1,05	0,96	0,95	1,09	1,21	expressed protein
258013_at	At3g19320	1,00	0,98	1,18	0,99	1,15	0,96	0,93	0,77	leucine-rich repeat family protein
258014_at	At3g19330	1,08	0,93	0,94	0,77	0,86	1,18	0,95	1,10	polyadenylate-binding protein-related / PABP-related
258015_at	At3g19340	1,13	1,09	1,01	0,99	1,06	1,14	1,07	1,05	expressed protein
258016_at	At3g19350	0,85	0,67	0,66	1,03	1,06	1,01	1,01	0,96	polyadenylate-binding protein-related / PABP-related
258024_at	At3g19360	0,77	0,77	0,88	0,99	0,89	0,84	0,92	0,77	zinc finger (CCCH-type) family protein
258017_at	At3g19370	1,40	1,14	1,00	1,14	0,95	1,34	1,39	1,19	expressed protein
258021_at	At3g19380	0,90	0,99	0,87	0,78	0,86	0,78	0,94	0,80	U-box domain-containing protein
258005_at	At3g19390	1,14	1,27	1,39	1,16	1,23	1,13	1,36	1,47	cysteine proteinase, putative / thiol protease, putative
258006_at	At3g19400	0,84	0,87	0,94	0,79	0,97	0,89	0,98	1,09	cysteine proteinase, putative
258007_at	At3g19420	1,20	1,25	1,25	1,01	1,21	1,11	1,11	1,30	expressed protein
258008_at	At3g19430	1,20	1,23	0,85	3,18	3,25	1,16	1,28	1,22	late embryogenesis abundant protein-related / LEA protein-related
258009_at	At3g19440	1,15	1,02	0,92	1,03	0,94	1,01	0,95	0,95	pseudouridine synthase family protein
258023_at	At3g19450	1,51	1,56	1,52	1,21	0,99	1,55	1,28	1,28	cinnamyl-alcohol dehydrogenase (CAD)
258022_at	At3g19460	0,92	0,97	0,78	0,82	0,93	1,11	0,98	0,89	reticulon family protein (RTNLB11)
258019_at	At3g19470	1,15	1,12	1,09	1,05	1,11	0,94	0,93	1,00	F-box family protein
258025_at	At3g19480	1,13	1,04	1,16	0,77	0,80	1,60	1,49	1,52	D-3-phosphoglycerate dehydrogenase, putative / 3-PGDH, putative
258020_at	At3g19490	0,77	0,79	0,76	0,93	1,01	0,87	0,94	0,91	sodium hydrogen antiporter, putative
258018_at	At3g19500	1,00	1,21	1,03	0,98	1,12	1,01	0,97	1,10	ethylene-responsive protein -related
258011_at	At3g19510	1,00	1,00	1,29	0,83	0,94	1,12	1,21	0,96	homeobox protein (HAT 3.1)
258027_at	At3g19515	0,60	0,56	0,54	1,17	1,13	1,04	1,18	1,35	expressed protein
256565_at	At3g19516	0,96	0,81	0,99	1,20	1,08	0,97	0,92	0,99	hypothetical protein
256568_at	At3g19520	1,09	0,90	1,32	1,02	1,14	0,81	0,96	0,96	expressed protein
256566_at	At3g19530	1,03	0,99	1,10	0,97	1,13	1,09	1,03	0,91	hypothetical protein
256570_at	At3g19540	0,92	1,55	1,01	0,96	1,25	0,92	0,93	1,12	expressed protein
256569_at	At3g19550	0,83	0,66	0,83	2,43	2,77	0,84	0,69	0,73	expressed protein
256567_at	At3g19553	0,66	0,57	0,55	0,83	0,85	0,87	0,75	0,86	amino acid permease family protein
257047_at	At3g19570	0,82	0,99	1,12	0,90	1,20	1,03	0,86	0,92	expressed protein
257022_at	At3g19580	0,54	0,81	0,91	0,75	0,73	1,09	1,14	1,49	zinc finger (C2H2 type) protein 2 (AZF2)
257020_at	At3g19590	1,04	0,95	0,84	1,14	0,96	0,97	0,89	0,88	WD-40 repeat family protein / mitotic checkpoint protein, putative
257563_at	At3g19610	0,97	0,93	0,96	0,93	0,98	1,08	1,02	1,08	hypothetical protein
257017_at	At3g19620	0,91	0,86	0,86	0,82	0,84	0,99	0,85	0,84	glycosyl hydrolase family 3 protein
257018_at	At3g19630	1,03	0,88	1,03	1,09	1,21	1,05	0,92	0,91	radical SAM domain-containing protein
257019_at	At3g19640	1,11	1,05	1,09	1,07	1,14	1,09	1,13	1,33	magnesium transporter CorA-like family protein (MRS2-3)
257073_at	At3g19650	1,06	0,78	0,74	0,90	1,31	1,28	1,13	1,32	cyclin-related
257074_at	At3g19660	0,69	0,66	0,57	1,02	1,08	0,95	0,75	0,82	expressed protein
257075_at	At3g19670	1,00	1,18	1,25	0,73	0,81	1,20	1,39	1,58	FF domain-containing protein / WW domain-containing protein
257076_at	At3g19680	1,03	1,76	1,38	0,86	0,80	0,99	1,40	1,68	expressed protein
257077_at	At3g19690	0,94	0,97	0,98	0,95	1,15	0,93	0,96	1,05	pathogenesis-related protein, putative
257043_at	At3g19700	0,92	1,00	1,02	0,95	1,03	1,10	1,07	1,01	leucine-rich repeat transmembrane protein kinase, putative
257021_at	At3g19710	1,54	1,49	1,67	1,21	0,80	0,99	0,96	0,93	branched-chain amino acid aminotransferase, putative / branched-chain am

257044_at	At3g19720	1,01	0,85	0,96	1,05	1,22	1,03	1,09	0,93	dynamin family protein
257045_at	At3g19730	1,06	0,87	0,90	1,17	1,17	0,86	0,88	1,07	dynamin family protein
257046_at	At3g19740	0,88	0,77	0,76	0,95	0,94	0,89	1,08	0,96	AAA-type ATPase family protein
257048_at	At3g19750	0,85	0,97	0,75	0,91	1,10	1,15	0,91	1,09	hypothetical protein
257023_at	At3g19760	1,32	1,21	1,21	0,98	1,05	1,02	1,10	1,03	eukaryotic translation initiation factor 4A, putative / eIF-4A, putative / DEAD
257989_at	At3g19770	0,90	1,19	1,26	1,23	1,17	0,97	1,27	1,25	vacuolar sorting protein 9 domain-containing protein / VPS9 domain-contair
257961_at	At3g19780	0,91	1,07	0,99	0,98	1,01	0,95	0,97	0,98	expressed protein
257962_at	At3g19790	1,23	1,31	1,19	1,32	1,14	1,09	1,13	1,20	hypothetical protein
257966_at	At3g19800	1,10	1,23	1,38	0,86	0,77	1,20	1,12	1,25	expressed protein
257937_at	At3g19810	1,07	0,90	0,96	0,69	0,81	1,01	0,92	1,10	expressed protein
257938_at	At3g19820	0,88	0,86	0,86	1,00	0,85	0,70	0,68	0,74	cell elongation protein / DWARF1 / DIMINUTO (DIM)
257963_at	At3g19840	1,29	1,07	1,34	0,73	0,76	1,16	1,22	1,31	FF domain-containing protein / WW domain-containing protein
257964_at	At3g19850	1,24	1,43	0,90	0,52	0,86	1,12	0,87	1,00	phototropic-responsive NPH3 family protein
257990_at	At3g19860	1,16	1,31	1,33	0,97	0,94	1,11	1,11	1,77	basic helix-loop-helix (bHLH) family protein
257991_at	At3g19870	0,88	0,88	1,19	0,82	0,88	0,95	0,89	0,96	expressed protein
257992_at	At3g19880	0,98	0,97	0,97	1,03	1,01	1,01	0,93	0,93	F-box family protein
257993_at	At3g19890	0,97	1,05	1,09	1,04	1,00	1,09	0,97	0,97	F-box family protein
257965_at	At3g19900	0,87	0,90	0,86	0,92	0,80	1,08	0,96	0,97	expressed protein
257967_at	At3g19910	0,89	0,99	1,09	1,00	1,09	1,02	0,97	1,06	zinc finger (C3HC4-type RING finger) family protein
257994_at	At3g19920	0,65	0,89	0,64	0,89	0,88	0,98	0,99	1,10	expressed protein
257939_at	At3g19930	0,80	0,90	0,84	0,97	0,85	0,81	0,77	0,86	sugar transport protein (STP4)
257995_at	At3g19940	0,86	1,03	0,94	1,04	0,97	0,97	0,92	1,02	sugar transporter, putative
257996_at	At3g19950	1,06	0,94	0,87	0,98	0,92	1,05	0,94	1,18	zinc finger (C3HC4-type RING finger) family protein
256623_at	At3g19960	0,80	0,73	0,91	0,79	0,76	1,08	1,04	1,24	myosin (ATM)
256627_at	At3g19970	0,98	1,33	1,21	1,05	1,06	1,14	1,44	1,41	expressed protein
256629_at	At3g19980	1,21	1,17	1,27	1,16	1,01	1,08	1,03	0,95	serine/threonine protein phosphatase (STPP)
256624_at	At3g19990	0,92	0,99	1,19	0,89	1,10	0,89	0,91	1,17	expressed protein
256628_at	At3g20000	1,05	1,00	0,93	1,17	1,10	0,94	0,98	1,01	porin family protein
256625_at	At3g20010	1,62	1,57	1,69	1,03	1,04	1,31	1,36	1,18	SNF2 domain-containing protein / helicase domain-containing protein / RIN
256626_at	At3g20015	0,61	0,72	0,70	0,83	0,84	0,80	0,78	0,84	aspartyl protease family protein
257116_at	At3g20020	1,04	1,18	1,18	0,84	1,06	1,11	1,00	0,83	protein arginine N-methyltransferase family protein
257123_at	At3g20030	0,92	1,21	0,92	1,01	1,04	0,96	0,97	1,06	F-box family protein
257124_at	At3g20040	1,02	1,00	0,85	1,04	1,11	0,91	0,96	0,98	hexokinase, putative
257125_at	At3g20050	1,23	1,12	1,01	1,28	1,20	0,88	0,95	0,96	T-complex protein 1 alpha subunit / TCP-1-alpha / chaperonin (CCT1)
257126_at	At3g20060	0,98	0,90	1,24	1,15	1,25	0,86	0,92	1,02	ubiquitin-conjugating enzyme 19 (UBC19)
257127_at	At3g20070	1,14	1,16	1,37	0,96	0,87	1,09	1,12	1,11	expressed protein
257128_at	At3g20080	0,93	0,99	0,86	1,08	1,05	0,89	0,98	0,94	cytochrome P450 family protein
257142_at	At3g20090	0,95	0,86	0,99	0,99	0,96	0,96	1,01	0,97	cytochrome P450 family protein
257129_at	At3g20100	0,77	0,70	0,75	0,73	0,83	1,05	0,97	0,90	cytochrome P450 family protein
257143_at	At3g20110	0,89	0,88	0,57	1,03	1,04	1,03	0,96	0,98	cytochrome P450 family protein
257112_at	At3g20120	0,96	0,95	1,19	0,69	0,94	1,04	1,16	1,04	cytochrome P450 family protein
257113_at	At3g20130	0,98	0,95	0,85	0,95	0,91	1,03	0,94	0,93	cytochrome P450 family protein
257114_at	At3g20140	0,91	1,04	0,92	1,01	1,16	1,05	1,09	0,87	cytochrome P450 family protein
257115_at	At3g20150	1,12	1,11	1,00	1,26	1,21	0,87	1,00	1,11	kinesin motor family protein
257117_at	At3g20160	1,02	1,04	0,95	1,24	1,14	0,91	1,02	1,12	geranylgeranyl pyrophosphate synthase, putative / GGPP synthetase, putat
257118_at	At3g20180	1,03	1,05	0,95	1,19	1,24	1,01	0,99	1,02	hypothetical protein

257119_at	At3g20190	1,03	1,07	0,97	0,94	0,93	1,01	1,08	0,93	leucine-rich repeat transmembrane protein kinase, putative
257120_at	At3g20200	1,10	1,23	1,30	0,89	1,12	1,06	1,06	1,06	protein kinase family protein
257130_at	At3g20210	0,20	0,12	0,05	0,58	0,67	0,90	0,99	0,92	vacuolar processing enzyme, putative / asparaginyl endopeptidase, putative
257121_at	At3g20220	1,01	0,98	1,07	0,95	1,04	0,98	1,06	0,89	auxin-responsive protein, putative
257132_at	At3g20230	1,11	1,07	1,16	1,21	1,04	0,97	0,97	1,14	50S ribosomal protein L18 family
257131_at	At3g20240	1,07	0,85	0,87	0,89	1,10	0,93	0,89	0,87	mitochondrial substrate carrier family protein
257122_at	At3g20250	0,74	0,87	0,89	0,80	0,77	1,04	1,13	1,32	pumilio/Puf RNA-binding domain-containing protein
257663_at	At3g20260	0,96	1,03	1,04	1,07	1,17	0,92	0,88	0,86	expressed protein
257666_at	At3g20270	1,01	1,03	1,10	1,22	1,28	1,15	1,16	1,27	lipid-binding serum glycoprotein family protein
257622_at	At3g20280	0,94	1,00	0,98	0,95	0,88	1,03	0,93	1,04	PHD finger family protein
257677_at	At3g20290	1,02	0,97	0,94	0,94	0,88	0,88	1,04	1,20	calcium-binding EF hand family protein
257672_at	At3g20300	0,56	0,60	0,77	0,66	0,71	0,91	0,87	0,99	expressed protein
257675_at	At3g20310	0,77	1,03	0,66	1,04	0,90	1,00	1,06	1,15	ethylene-responsive element-binding family protein
257676_at	At3g20320	0,99	0,85	0,87	1,00	0,91	0,97	0,90	0,93	mce-related family protein
257680_at	At3g20330	1,11	0,91	0,95	0,99	0,99	0,94	0,90	0,84	aspartate carabmoyltransferase, chloroplast / aspartate transcarbamylase /
257670_at	At3g20340	0,72	1,13	1,02	0,74	0,99	1,15	1,27	1,14	expressed protein
257673_at	At3g20370	1,38	1,04	0,87	1,83	1,69	0,89	0,98	1,04	meprin and TRAF homology domain-containing protein / MATH domain-cor
257674_at	At3g20390	1,06	0,94	0,83	1,19	1,00	0,81	0,86	0,87	endoribonuclease L-PSP family protein
257664_at	At3g20400	1,01	1,03	1,00	0,93	0,89	0,85	1,22	1,26	hypothetical protein
257621_at	At3g20410	0,67	0,63	0,76	0,81	1,12	0,84	0,79	0,79	calmodulin-domain protein kinase isoform 9 (CPK9)
257678_at	At3g20420	1,00	1,02	1,02	0,93	0,87	1,06	1,03	1,05	ribonuclease III family protein
257665_at	At3g20430	1,54	1,86	3,03	1,28	2,09	1,14	1,45	1,52	expressed protein
257667_at	At3g20440	0,97	0,92	0,99	0,87	0,76	0,89	0,93	1,00	glycoside hydrolase family 13 protein
257671_at	At3g20450	1,05	1,06	0,81	1,18	1,05	0,87	0,94	1,22	expressed protein
257668_at	At3g20460	1,01	0,97	0,93	1,12	0,99	1,02	0,89	1,10	sugar transporter, putative
257679_at	At3g20470	1,24	1,65	1,41	1,43	1,10	0,94	1,05	0,99	pseudogene, glycine-rich protein
257669_at	At3g20475	1,06	0,94	0,97	0,94	1,09	1,01	0,96	0,93	DNA mismatch repair MutS family protein
257094_at	At3g20480	1,03	0,81	0,96	0,94	1,00	1,00	0,85	1,05	tetraacyldisaccharide 4'-kinase family protein
257086_at	At3g20490	1,12	1,05	1,16	1,07	1,07	1,14	1,00	0,94	expressed protein
257087_at	At3g20500	0,73	0,66	0,82	0,89	0,93	0,99	1,00	1,13	calcineurin-like phosphoesterase family protein
257088_at	At3g20510	0,75	0,73	0,68	1,04	1,06	0,71	0,70	0,75	expressed protein
257089_at	At3g20520	0,96	0,96	0,75	0,75	0,87	0,97	1,18	1,18	glycerophosphoryl diester phosphodiesterase family protein
257090_at	At3g20530	0,99	0,99	0,87	0,92	1,16	1,12	1,02	0,93	protein kinase family protein
257091_at	At3g20540	0,95	1,01	0,88	0,98	0,97	1,01	0,92	0,91	DNA-directed DNA polymerase family protein
257092_at	At3g20550	1,04	1,05	1,15	0,82	0,88	1,11	1,23	1,46	forkhead-associated domain-containing protein / FHA domain-containing pr
257095_at	At3g20560	0,80	0,85	0,79	1,10	1,16	0,92	0,85	0,83	thioredoxin family protein
257093_at	At3g20570	1,27	1,24	0,93	1,38	1,22	0,89	1,06	1,04	plastocyanin-like domain-containing protein
257082_at	At3g20580	0,89	1,12	0,98	0,96	0,95	1,06	1,00	0,99	phytochelatin synthetase-related
257083_s_at	At3g20590	0,61	0,50	0,43	0,66	0,76	0,93	0,88	0,79	non-race specific disease resistance protein, putative
257084_at	At3g20620	1,07	0,85	0,92	1,25	1,01	0,98	0,82	1,10	F-box family protein-related
257085_at	At3g20630	1,14	0,97	1,13	0,96	0,80	1,13	1,07	1,10	ubiquitin-specific protease 14, putative (UBP14)
256696_at	At3g20650	0,93	0,98	1,01	1,01	0,91	0,96	0,99	1,05	mRNA capping enzyme family protein
256697_at	At3g20660	0,82	0,87	0,90	0,89	0,99	0,91	0,93	0,74	organic cation transporter family protein
256666_at	At3g20670	1,07	1,13	1,05	1,10	1,07	1,11	1,32	1,02	histone H2A, putative
256698_at	At3g20680	0,99	1,15	1,25	0,95	1,12	0,97	0,91	1,16	expressed protein
256699_at	At3g20690	0,99	1,08	1,00	0,96	0,97	1,02	0,95	1,00	F-box protein-related

256665_at	At3g20700	1,01	0,96	1,09	1,05	1,03	1,04	1,06	1,07	F-box family protein-related
257574_at	At3g20710	1,04	1,11	1,09	1,04	1,05	1,03	1,11	1,16	F-box protein-related
257973_at	At3g20720	1,05	1,00	1,07	1,05	0,87	1,00	1,02	0,86	expressed protein
257979_at	At3g20730	1,06	0,84	1,25	0,96	1,13	1,21	0,95	0,89	pentatricopeptide (PPR) repeat-containing protein
257987_at	At3g20740	1,08	1,12	1,27	0,79	0,74	1,05	1,02	0,97	fertilization-independent endosperm protein (FIE)
257980_at	At3g20760	0,98	1,06	0,90	0,94	0,90	1,01	1,01	1,07	hypothetical protein
257981_at	At3g20770	0,84	0,83	0,99	0,91	1,07	0,96	0,86	1,02	ethylene-insensitive 3 (EIN3)
257982_at	At3g20780	0,96	0,81	0,80	0,85	1,06	0,93	1,00	0,92	topoisomerase 6 subunit B (TOP6B)
257983_at	At3g20790	0,80	0,90	0,83	1,10	1,15	0,93	0,92	0,89	oxidoreductase family protein
257984_at	At3g20800	0,97	0,91	0,95	0,95	0,85	1,01	0,93	0,98	rcd1-like cell differentiation protein, putative
257985_at	At3g20810	0,54	0,46	0,42	0,54	0,54	0,84	1,09	1,05	transcription factor jumonji (jmjC) domain-containing protein
257974_at	At3g20820	0,98	1,20	1,13	0,99	0,85	0,83	0,77	0,94	leucine-rich repeat family protein
257975_at	At3g20830	0,94	1,02	1,07	0,97	1,10	0,99	0,84	0,90	protein kinase family protein
257976_at	At3g20840	1,07	1,00	1,01	1,23	1,12	1,10	1,02	1,26	ovule development protein, putative
257977_at	At3g20850	0,94	1,10	1,00	0,97	0,97	1,04	0,89	0,98	proline-rich family protein
257978_at	At3g20860	1,39	1,80	1,23	1,13	1,18	1,08	1,40	1,10	protein kinase family protein
257986_at	At3g20865	0,90	1,03	1,17	1,18	1,16	0,84	0,98	1,00	arabinogalactan-protein, putative (AGP)
257988_at	At3g20870	0,97	0,84	0,79	1,12	0,90	0,90	0,94	0,89	metal transporter family protein
257544_at	At3g20880	0,98	1,10	0,93	0,91	1,03	0,88	0,98	0,95	zinc finger (C2H2 type) protein (WIP4)
256800_at	At3g20900	0,96	1,04	0,97	1,03	1,14	0,95	0,91	0,97	expressed protein
256806_at	At3g20910	0,98	1,16	1,07	0,84	1,08	0,99	0,90	0,99	CCAAT-binding transcription factor (CBF-B/NF-YA) family protein
256804_at	At3g20920	1,03	0,94	0,89	1,04	1,07	0,99	0,85	0,93	translocation protein-related
256805_at	At3g20930	1,05	0,94	0,89	0,83	0,70	1,24	1,07	1,00	RNA recognition motif (RRM)-containing protein
256801_at	At3g20940	1,01	0,93	0,71	1,21	1,20	0,76	0,91	0,82	cytochrome P450 family protein
256802_at	At3g20950	0,97	0,77	0,83	0,98	1,04	0,96	1,12	1,14	cytochrome P450 family protein
256803_at	At3g20960	0,96	0,91	1,06	1,03	1,07	1,03	0,95	0,92	cytochrome P450 family protein
256807_at	At3g20970	1,02	1,10	1,12	1,05	1,02	0,92	1,01	1,04	nitrogen fixation NifU-like family protein
256973_at	At3g20980	1,03	0,97	1,05	0,94	1,02	1,06	0,95	1,13	hypothetical protein
256974_at	At3g20990	1,05	0,99	1,15	0,89	0,98	1,06	0,96	0,98	---
256975_at	At3g21000	0,86	1,04	0,82	1,01	1,11	0,94	1,11	0,99	expressed protein
256976_at	At3g21020	1,08	1,05	1,08	0,85	1,11	1,10	0,90	1,05	---
256977_at	At3g21040	0,93	0,90	0,98	1,02	0,88	1,03	0,96	0,99	---
256979_at	At3g21055	1,05	0,93	0,94	1,02	1,09	0,94	0,88	0,90	photosystem II 5 kD protein, putative
256967_at	At3g21060	1,23	1,84	1,99	0,90	0,80	1,20	1,28	1,18	transducin family protein / WD-40 repeat family protein
256968_at	At3g21070	0,51	0,74	1,11	0,72	0,78	1,12	1,48	1,61	ATP-NAD kinase family protein
256969_at	At3g21080	1,09	1,15	1,13	1,34	1,99	0,84	0,84	0,66	ABC transporter-related
256970_at	At3g21090	0,90	1,22	1,16	0,80	0,89	1,02	1,07	1,12	ABC transporter family protein
256971_at	At3g21100	0,88	1,05	0,95	1,05	1,08	0,97	0,89	0,94	RNA recognition motif (RRM)-containing protein
256978_at	At3g21110	1,35	1,18	1,20	1,00	0,82	1,08	1,08	0,99	phosphoribosylamidoimidazole-succinocarboxamide synthase / SAICAR sy
256972_at	At3g21140	1,08	0,96	1,14	0,90	1,01	1,00	0,94	1,19	expressed protein
256952_at	At3g21160	1,06	0,90	0,86	1,17	1,03	1,08	0,99	0,91	mannosyl-oligosaccharide 1,2-alpha-mannosidase, putative
258032_at	At3g21170	1,00	1,10	0,92	1,00	1,08	0,97	0,95	1,21	F-box family protein
258041_at	At3g21175	1,01	0,95	1,09	0,92	0,98	0,90	0,98	0,91	zinc finger (GATA type) family protein
258035_at	At3g21180	1,05	1,06	0,71	1,07	1,04	1,00	0,90	1,10	calcium-transporting ATPase, plasma membrane-type, putative / Ca2+-ATF
258040_at	At3g21190	0,83	0,79	0,71	1,12	0,94	0,84	0,80	0,73	expressed protein
258039_at	At3g21200	0,95	0,96	0,97	0,89	0,90	1,00	0,91	0,95	expressed protein

258036_at	At3g21210	1,00	1,06	0,97	0,98	1,05	1,10	1,09	0,97	universal stress protein (USP) family protein / DC1 domain-containing prote
258046_at	At3g21220	0,76	0,67	0,76	1,16	1,12	0,84	0,78	1,08	mitogen-activated protein kinase kinase (MAPKK), putative (MKK5)
258037_at	At3g21230	0,97	0,83	1,05	2,26	1,68	0,99	1,07	1,23	4-coumarate--CoA ligase, putative / 4-coumaroyl-CoA synthase, putative (4
258047_at	At3g21240	1,15	1,13	1,22	1,42	1,19	1,06	1,35	1,11	4-coumarate--CoA ligase 2 / 4-coumaroyl-CoA synthase 2 (4CL2)
258033_at	At3g21250	0,63	0,54	0,71	0,91	0,76	1,01	1,27	1,14	ABC transporter family protein
258038_at	At3g21260	1,37	1,88	1,62	0,72	0,56	0,86	1,27	1,31	glycolipid transfer protein-related
258044_at	At3g21270	0,91	0,99	1,13	1,05	1,10	0,76	1,10	1,22	Dof-type zinc finger domain-containing protein (ADOF2)
258045_at	At3g21280	0,89	0,75	0,85	0,93	0,92	1,07	0,92	0,93	ubiquitin-specific protease 7, putative (UBP7)
258043_at	At3g21290	0,90	0,80	1,05	0,76	0,82	1,09	1,05	1,12	dentin sialophosphoprotein-related
258034_at	At3g21300	1,12	0,99	0,84	0,64	0,66	0,94	0,92	0,84	RNA methyltransferase family protein
258042_at	At3g21310	1,20	1,25	1,23	1,22	0,98	0,95	0,97	0,82	expressed protein
256811_at	At3g21340	1,08	1,01	0,93	1,00	0,97	1,06	0,98	1,17	leucine-rich repeat protein kinase, putative
256812_at	At3g21350	1,24	1,13	1,25	0,99	1,11	0,91	0,89	0,86	RNA polymerase transcriptional regulation mediator-related
256813_at	At3g21360	1,09	1,09	1,12	0,83	0,98	1,05	1,02	1,18	expressed protein
256814_at	At3g21370	0,03	0,03	0,05	0,39	0,43	0,93	0,99	1,31	glycosyl hydrolase family 1 protein
256815_at	At3g21380	0,09	0,09	0,16	0,46	0,39	0,98	1,00	1,03	jacalin lectin family protein
256819_at	At3g21390	1,38	1,56	1,44	1,35	0,98	1,28	1,28	1,22	mitochondrial substrate carrier family protein
256816_at	At3g21400	0,97	1,21	1,02	1,30	1,69	1,04	1,01	1,14	expressed protein
256817_at	At3g21410	1,02	1,06	0,91	1,01	1,40	1,07	0,91	1,03	F-box family protein (FBW1)
256818_at	At3g21420	0,93	0,98	1,10	1,77	1,88	1,02	0,98	0,98	oxidoreductase, 2OG-Fe(II) oxygenase family protein
256808_at	At3g21430	1,07	1,26	1,75	0,81	0,75	1,19	1,24	1,51	expressed protein
256809_at	At3g21440	1,00	1,17	1,07	0,99	0,83	1,19	1,10	0,93	myb family transcription factor
256810_at	At3g21465	1,01	0,99	0,96	1,04	0,93	1,05	0,93	0,85	expressed protein
258174_at	At3g21470	0,90	0,94	1,04	0,90	0,98	1,08	0,94	0,92	pentatricopeptide (PPR) repeat-containing protein
258180_at	At3g21480	0,95	1,04	1,01	0,75	1,08	0,98	0,93	0,92	transcription activation domain-interacting protein-related
258182_at	At3g21500	0,95	1,05	0,99	0,95	1,08	1,03	1,06	0,91	1-deoxy-D-xylulose 5-phosphate synthase, putative / 1-deoxyxylulose-5-ph
258184_at	At3g21510	1,41	1,27	1,20	1,90	1,43	1,34	1,13	1,19	two-component phosphorelay mediator 3 (HP3)
257540_at	At3g21520	1,23	1,19	1,29	1,25	1,31	1,21	1,08	0,97	expressed protein
258166_at	At3g21540	1,20	1,01	1,16	0,85	0,80	0,89	0,93	0,86	transducin family protein / WD-40 repeat family protein
258183_at	At3g21550	1,85	1,57	1,11	1,67	1,47	1,02	0,82	0,85	expressed protein
258167_at	At3g21560	1,03	0,77	0,93	0,89	0,75	0,88	0,83	1,00	UDP-glucosyltransferase, putative
258168_at	At3g21570	0,95	1,01	1,22	1,03	1,00	1,03	0,93	0,97	expressed protein
258185_at	At3g21580	0,92	0,76	0,94	0,84	0,76	0,91	0,86	0,76	expressed protein
258169_at	At3g21590	0,88	0,98	1,01	1,08	1,02	0,90	0,99	1,00	senescence/dehydration-associated protein-related
258170_at	At3g21600	1,12	0,87	0,83	1,11	1,03	1,00	0,99	1,05	senescence/dehydration-associated protein-related
258171_at	At3g21610	1,04	1,01	1,01	1,34	1,05	0,91	0,98	1,00	expressed protein
258172_at	At3g21620	0,90	0,96	1,03	0,86	1,07	0,95	1,03	0,92	early-responsive to dehydration protein-related / ERD protein-related
258173_at	At3g21630	0,70	0,67	0,63	1,00	1,03	0,75	0,91	0,83	protein kinase family protein
258175_at	At3g21640	1,20	1,11	1,08	0,98	1,01	1,21	1,06	1,14	FKBP-type peptidyl-prolyl cis-trans isomerase family protein
258176_at	At3g21650	0,81	1,02	1,53	1,13	1,16	0,97	1,04	1,38	serine/threonine protein phosphatase 2A (PP2A) regulatory subunit B', puta
258177_at	At3g21660	0,89	0,75	0,90	0,92	1,24	0,95	1,00	1,00	UBX domain-containing protein
258181_at	At3g21670	1,38	1,31	1,52	1,09	1,02	1,60	1,69	1,62	nitrate transporter (NTP3)
258178_at	At3g21680	1,01	1,12	0,92	1,05	1,08	1,04	0,92	1,01	expressed protein
258179_at	At3g21690	0,91	0,96	1,04	0,75	0,69	1,12	1,07	1,03	MATE efflux family protein
257951_at	At3g21700	0,95	0,98	0,92	0,96	0,99	1,05	0,87	0,91	expressed protein
257946_at	At3g21710	1,58	1,72	1,78	1,03	0,84	1,37	1,33	1,44	expressed protein

257947_at	At3g21720	0,18	0,20	0,15	0,37	0,47	1,68	1,92	1,18	isocitrate lyase, putative
257948_at	At3g21740	1,29	1,27	1,37	0,86	1,00	0,83	1,01	0,96	expressed protein
257949_at	At3g21750	0,65	0,73	0,79	0,89	0,97	0,75	0,81	0,90	UDP-glucoronosyl/UDP-glucosyl transferase family protein
257954_at	At3g21760	0,63	0,94	0,97	0,74	0,75	0,99	0,88	0,88	UDP-glucoronosyl/UDP-glucosyl transferase family protein
257952_at	At3g21770	1,06	1,08	0,91	1,75	1,41	1,12	1,24	1,22	peroxidase 30 (PER30) (P30) (PRXR9)
257950_at	At3g21780	0,80	0,93	0,95	0,95	0,95	1,00	0,86	0,94	UDP-glucoronosyl/UDP-glucosyl transferase family protein
257940_at	At3g21790	0,66	0,58	0,64	0,72	0,96	0,89	0,89	0,94	UDP-glucoronosyl/UDP-glucosyl transferase family protein
257941_at	At3g21800	0,91	0,96	1,04	0,97	0,95	1,09	1,02	0,86	UDP-glucoronosyl/UDP-glucosyl transferase family protein
257955_at	At3g21810	1,31	1,41	1,02	0,91	0,98	1,20	1,42	1,59	zinc finger (CCCH-type) family protein
257942_at	At3g21830	1,02	1,02	1,03	0,95	1,06	0,87	1,04	0,96	E3 ubiquitin ligase SCF complex subunit SKP1/ASK1 (At8), putative
257943_at	At3g21840	1,04	1,04	0,90	1,04	1,04	0,97	0,96	0,94	E3 ubiquitin ligase SCF complex subunit SKP1/ASK1 (At7), putative
257944_at	At3g21850	0,93	0,98	1,04	0,97	1,08	0,93	1,03	0,95	E3 ubiquitin ligase SCF complex subunit SKP1/ASK1 (At9), putative
257945_at	At3g21860	0,93	1,02	0,95	0,98	0,97	1,03	1,08	1,15	E3 ubiquitin ligase SCF complex subunit SKP1/ASK1 (At10), putative
257953_at	At3g21865	0,88	0,85	0,98	1,00	1,05	0,90	0,90	0,84	expressed protein
256894_at	At3g21870	1,97	2,60	2,19	1,28	1,50	1,27	1,48	1,43	cyclin family protein
257262_at	At3g21890	0,93	0,98	1,02	1,40	1,32	1,02	0,98	0,99	zinc finger (B-box type) family protein
257261_s_at	At3g21930	1,06	0,99	1,05	0,93	1,02	1,00	0,98	1,22	pollen coat receptor kinase, putative
257254_at	At3g21950	0,78	0,71	0,77	0,91	0,86	0,93	0,72	0,83	S-adenosyl-L-methionine:carboxyl methyltransferase family protein
257255_at	At3g21960	1,04	0,97	0,97	0,99	0,94	0,97	0,91	1,08	receptor-like protein kinase-related
257256_at	At3g21970	1,00	0,99	1,00	1,14	1,12	1,01	1,11	0,80	receptor-like protein kinase-related
257558_s_at	At3g22000	1,04	1,02	0,90	0,94	0,99	0,97	1,06	1,27	receptor-like protein kinase-related
257257_at	At3g22020	1,01	1,10	0,97	1,06	1,08	0,97	1,21	1,07	receptor-like protein kinase-related
257258_at	At3g22040	0,91	1,05	0,93	1,11	1,14	0,94	0,92	1,09	receptor-like protein kinase-related
257559_at	At3g22050	0,92	1,04	1,10	0,91	1,13	1,02	1,03	1,00	receptor-like protein kinase-related
257264_at	At3g22060	0,94	0,76	0,60	1,04	0,82	1,12	0,72	0,59	receptor protein kinase-related
257263_at	At3g22070	0,91	0,93	1,00	1,00	0,83	0,89	0,93	0,87	proline-rich family protein
257259_at	At3g22090	1,05	0,99	0,95	1,03	1,04	1,02	1,00	0,92	expressed protein
257260_at	At3g22104	1,19	1,24	1,23	0,66	0,71	1,05	1,33	0,95	phototropic-responsive NPH3 protein-related
256795_at	At3g22110	1,11	1,09	1,16	1,29	1,33	1,05	0,91	0,89	20S proteasome alpha subunit C (PAC1) (PRC9)
256825_at	At3g22120	0,84	0,79	0,75	1,10	1,12	0,76	0,72	0,63	protease inhibitor/seed storage/lipid transfer protein (LTP) family protein
256824_at	At3g22130	0,86	0,93	1,03	1,04	1,07	0,93	1,03	1,13	---
256791_at	At3g22140	0,95	0,88	1,03	0,89	0,97	1,04	1,08	1,13	---
256792_at	At3g22150	0,88	1,17	1,04	0,66	0,55	1,36	1,42	1,23	pentatricopeptide (PPR) repeat-containing protein
256793_at	At3g22160	0,70	0,94	0,86	1,24	1,28	0,90	0,94	1,09	VQ motif-containing protein
256820_at	At3g22170	1,06	0,91	1,16	0,93	0,97	1,10	0,97	1,18	far-red impaired responsive protein, putative
256821_at	At3g22180	0,93	1,18	1,16	1,03	1,03	0,93	0,95	1,16	zinc finger (DHHC type) family protein
256822_at	At3g22190	0,80	0,94	1,18	0,84	0,72	0,92	0,89	0,90	calmodulin-binding family protein
256765_at	At3g22200	0,76	0,83	0,97	1,00	0,89	1,02	0,96	1,03	4-aminobutyrate aminotransferase / gamma-amino-N-butyrate transaminas
256796_at	At3g22210	0,81	0,81	0,99	0,74	0,88	1,05	0,88	1,02	expressed protein
256823_at	At3g22220	1,12	1,08	1,24	0,91	1,08	1,08	1,02	1,04	hAT dimerisation domain-containing protein
256794_at	At3g22230	1,63	1,20	1,22	1,36	1,41	1,00	0,96	0,84	60S ribosomal protein L27 (RPL27B)
256766_at	At3g22231	0,27	0,33	0,40	1,49	1,12	6,09	4,54	11,00	expressed protein
256617_at	At3g22240	1,31	1,71	1,44	1,24	1,21	1,84	1,82	1,58	expressed protein
256615_at	At3g22250	0,89	1,15	1,11	0,89	1,02	0,97	1,18	1,27	UDP-glucoronosyl/UDP-glucosyl transferase family protein
256616_at	At3g22260	0,59	0,57	0,69	0,94	0,97	0,74	0,85	1,29	OTU-like cysteine protease family protein
258443_at	At3g22280	0,90	0,89	0,80	1,06	1,15	0,88	1,15	0,83	expressed protein

258448_at	At3g22290	1,17	0,89	0,77	1,20	1,04	0,95	0,97	0,87	expressed protein
258454_at	At3g22300	1,00	0,84	0,99	1,03	1,20	0,88	0,80	0,79	40S ribosomal protein S10, mitochondrial (RPS10)
258449_s_at	At3g22310	1,22	1,18	1,19	0,94	0,84	0,98	1,11	1,07	DEAD box RNA helicase, putative (RH9)
258453_at	At3g22320	1,19	1,13	1,01	0,97	0,93	0,99	1,03	0,97	DNA-directed RNA polymerase, putative
258450_at	At3g22340	1,10	1,13	0,97	1,04	1,21	1,06	0,94	0,89	---
257537_at	At3g22350	0,94	1,12	0,94	1,12	0,84	1,08	1,11	0,83	F-box family protein
258451_at	At3g22360	0,86	1,09	1,08	1,05	1,10	1,09	1,01	1,15	alternative oxidase 1b, mitochondrial (AOX1B)
258452_at	At3g22370	1,30	1,22	1,35	0,68	0,74	1,24	1,31	1,28	alternative oxidase 1a, mitochondrial (AOX1A)
258444_at	At3g22380	1,07	1,10	0,98	0,73	0,85	1,05	0,99	1,03	expressed protein
258445_at	At3g22400	1,03	0,98	1,00	0,94	1,01	0,88	0,99	0,92	lipoxygenase, putative
258456_at	At3g22420	1,37	1,76	2,10	1,17	1,29	1,58	1,93	2,34	protein kinase family protein
258457_at	At3g22422	1,01	1,03	1,22	1,12	1,11	1,01	1,02	0,86	imidazoleglycerol-phosphate dehydratase 1 (IGPD1)
258446_at	At3g22430	1,08	1,08	1,03	0,86	0,76	1,06	1,08	0,93	expressed protein
258458_at	At3g22435	0,86	0,94	1,04	0,93	0,90	0,98	0,94	0,96	XS domain-containing protein
258455_at	At3g22440	1,37	1,23	1,19	1,37	1,31	1,00	1,01	1,02	hydroxyproline-rich glycoprotein family protein
258447_at	At3g22450	1,11	0,93	1,09	0,92	1,02	1,00	0,98	0,88	expressed protein
256930_at	At3g22460	0,75	0,81	0,86	0,78	0,70	1,11	1,39	1,55	cysteine synthase, putative / O-acetylserine (thiol)-lyase, putative / O-acetyl
256936_at	At3g22480	1,34	1,23	1,26	1,53	1,84	1,06	0,88	0,99	prefoldin-related KE2 family protein
256931_at	At3g22490	0,83	0,95	1,01	0,81	0,97	0,96	0,90	1,01	late embryogenesis abundant protein, putative / LEA protein, putative
256938_at	At3g22500	1,05	0,93	0,96	0,93	1,05	1,17	0,83	1,38	late embryogenesis abundant protein (ECP31) / LEA protein
256932_at	At3g22520	1,26	1,07	1,18	0,98	1,00	1,23	0,92	0,83	expressed protein
256934_at	At3g22530	0,87	1,03	0,75	1,37	1,36	1,02	1,04	0,82	expressed protein
256926_at	At3g22540	0,92	0,96	1,21	1,04	0,97	0,90	1,03	0,98	expressed protein
256927_at	At3g22550	1,10	1,11	0,68	1,04	0,94	1,10	1,08	0,95	senescence-associated protein-related
256935_at	At3g22570	0,82	0,66	0,55	1,23	0,94	0,98	1,06	1,02	protease inhibitor/seed storage/lipid transfer protein (LTP) family protein
256928_at	At3g22590	0,91	0,92	0,89	1,02	1,20	1,08	0,84	0,97	RNA pol II accessory factor Cdc73 family protein
256933_at	At3g22600	1,50	1,56	1,21	1,75	1,68	1,86	1,83	1,96	protease inhibitor/seed storage/lipid transfer protein (LTP) family protein
256929_at	At3g22610	1,00	1,06	1,02	0,94	0,97	1,17	0,92	0,91	---
256937_at	At3g22620	1,42	1,59	1,10	1,25	0,91	1,44	1,59	1,58	protease inhibitor/seed storage/lipid transfer protein (LTP) family protein
256939_at	At3g22630	1,05	1,11	1,03	1,03	0,99	0,93	0,92	0,89	20S proteasome beta subunit D (PBD1) (PRGB)
258327_at	At3g22640	0,85	0,84	0,76	0,92	1,43	1,04	0,87	1,02	cupin family protein
258344_at	At3g22650	1,02	0,95	1,14	0,98	1,01	1,01	1,01	0,87	F-box family protein
258316_at	At3g22660	1,17	1,14	1,16	1,01	0,77	1,10	0,96	0,96	rRNA processing protein-related
258317_at	At3g22670	0,85	0,93	0,97	0,99	0,88	0,93	1,00	0,88	pentatricopeptide (PPR) repeat-containing protein
258318_at	At3g22680	0,83	0,95	1,22	1,08	1,17	0,82	0,79	0,93	expressed protein
258346_at	At3g22690	1,06	1,05	1,08	1,02	1,00	1,03	0,97	0,93	pentatricopeptide (PPR) repeat-containing protein
258319_at	At3g22700	0,96	1,01	0,98	1,05	1,03	1,00	0,98	0,90	F-box family protein
258320_at	At3g22710	0,89	1,15	1,09	1,00	1,00	0,95	0,95	1,13	F-box family protein
258322_at	At3g22740	1,22	1,20	1,63	1,28	1,02	0,88	1,11	0,95	homocysteine S-methyltransferase 3 (HMT-3)
258323_at	At3g22750	0,76	0,89	0,85	0,94	0,87	0,99	0,86	0,79	protein kinase, putative
258326_at	At3g22760	0,97	1,16	1,05	0,81	1,14	1,08	1,08	0,90	CXC domain containing TSO1-like protein 1 (SOL1)
258340_at	At3g22770	0,99	1,00	0,85	0,97	0,97	0,94	0,98	0,91	F-box family protein-related
258324_at	At3g22780	1,19	1,06	0,96	1,12	0,96	1,08	0,91	0,84	CXC domain protein (TSO1)
258341_at	At3g22790	0,95	0,89	0,90	1,02	1,25	1,00	0,83	1,04	kinase interacting family protein
258342_at	At3g22800	0,95	1,25	0,92	1,16	1,23	0,97	0,95	0,92	leucine-rich repeat family protein / extensin family protein
258343_at	At3g22810	1,10	0,98	0,93	0,77	1,03	0,93	1,21	1,01	expressed protein

258325_at	At3g22830	1,28	1,07	1,04	1,01	1,03	1,16	1,06	1,10	heat shock transcription factor family protein
258321_at	At3g22840	1,23	1,40	1,35	1,18	1,42	1,48	1,49	1,17	chlorophyll A-B binding family protein / early light-induced protein (ELIP)
258345_at	At3g22845	1,20	0,90	0,92	1,39	1,17	1,08	0,91	0,86	emp24/gp25L/p24 protein-related
256829_at	At3g22850	1,10	1,05	0,92	1,78	1,70	1,17	1,01	1,10	expressed protein
256830_at	At3g22860	1,03	1,12	0,88	0,95	0,93	1,11	1,08	1,17	eukaryotic translation initiation factor 3 subunit 8, putative / eIF3c, putative
256831_at	At3g22870	0,98	1,04	1,04	0,94	0,92	1,09	1,00	0,83	F-box family protein
256832_at	At3g22880	0,82	0,79	0,67	1,33	1,10	0,88	0,96	0,81	meiotic recombination protein, putative
256835_at	At3g22890	1,24	1,09	1,14	1,02	0,85	0,93	1,00	1,00	sulfate adenylyltransferase 1 / ATP-sulfurylase 1 (APS1)
256837_at	At3g22900	1,19	1,35	1,28	0,97	1,16	0,85	0,96	0,85	RNA polymerase Rpb7 N-terminal domain-containing protein
256833_at	At3g22910	1,46	1,19	0,84	1,41	1,14	1,74	1,79	1,53	calcium-transporting ATPase, plasma membrane-type, putative / Ca(2+)-AT
256834_at	At3g22920	0,85	0,95	1,04	1,06	0,98	0,89	0,98	0,88	peptidyl-prolyl cis-trans isomerase, putative / cyclophilin, putative / rotamas
256839_at	At3g22930	0,92	0,72	0,61	1,22	1,15	0,87	1,06	0,94	calmodulin, putative
256859_at	At3g22940	0,80	0,92	0,82	0,97	1,11	1,00	1,02	1,01	F-box family protein-related
256838_at	At3g22950	1,01	0,93	1,13	1,22	1,29	1,06	0,88	0,88	ADP-ribosylation factor, putative
256836_at	At3g22960	0,88	0,80	0,78	0,80	0,75	0,89	0,88	0,84	pyruvate kinase, putative
256828_at	At3g22970	1,22	1,13	0,92	0,63	0,99	1,03	1,03	1,02	expressed protein
257758_at	At3g22980	0,89	0,79	0,91	0,81	0,89	0,94	0,86	1,04	elongation factor Tu family protein
257770_at	At3g22990	1,25	1,13	1,41	1,06	1,02	1,10	1,08	1,12	expressed protein
257771_at	At3g23000	0,71	0,87	0,88	0,62	0,69	1,05	1,08	1,02	CBL-interacting protein kinase 7 (CIPK7)
257764_at	At3g23010	1,06	0,92	1,00	0,96	0,99	0,97	1,02	0,98	disease resistance family protein / LRR family protein
257765_at	At3g23020	1,01	0,90	0,93	0,83	0,73	0,96	1,07	0,78	pentatricopeptide (PPR) repeat-containing protein
257766_at	At3g23030	1,98	2,62	1,92	1,20	1,19	1,62	2,02	1,70	auxin-responsive protein / indoleacetic acid-induced protein 2 (IAA2)
257767_at	At3g23040	1,10	1,11	0,90	0,96	0,93	1,07	1,01	0,93	hypothetical protein
257769_at	At3g23050	1,24	1,17	1,22	1,13	1,20	0,92	0,85	0,90	auxin-responsive protein / indoleacetic acid-induced protein 7 (IAA7)
257768_at	At3g23060	1,04	1,00	1,04	0,94	1,12	1,07	1,12	0,99	zinc finger (C3HC4-type RING finger) family protein
257759_at	At3g23070	1,02	0,93	1,09	0,70	0,56	1,14	1,34	1,31	expressed protein
257772_at	At3g23080	0,90	1,21	1,95	0,70	0,69	0,94	1,02	0,99	expressed protein
257760_s_at	At3g23085	0,97	1,02	1,00	1,05	0,96	1,01	1,02	0,85	---
257761_at	At3g23090	1,03	0,85	1,23	1,03	1,16	1,10	1,19	1,10	expressed protein
257762_at	At3g23100	1,07	1,02	1,07	0,93	1,04	0,99	0,99	0,95	double strand break repair protein, putative (XRCC4)
257763_s_at	At3g23110	0,95	1,08	0,91	1,05	1,06	1,20	1,06	0,95	disease resistance family protein
257915_at	At3g23130	0,91	0,98	0,98	1,02	0,94	0,89	0,99	1,00	superman protein (SUP) / zinc finger (C2H2 type) family protein
257922_at	At3g23150	1,00	0,97	0,94	0,76	0,89	0,63	0,56	0,66	ethylene receptor, putative (ETR2)
257923_at	At3g23160	0,70	0,75	0,70	0,91	1,07	0,81	0,93	1,09	expressed protein
257925_at	At3g23170	1,05	0,81	0,92	0,91	1,12	0,66	0,80	0,83	expressed protein
257924_at	At3g23190	1,08	1,25	0,88	1,81	1,56	1,06	1,18	1,06	lesion inducing protein-related
257545_at	At3g23200	0,63	0,68	0,76	0,91	0,66	0,98	1,00	0,89	expressed protein
257916_at	At3g23210	0,98	0,96	0,97	1,05	1,18	0,83	0,97	1,00	basic helix-loop-helix (bHLH) family protein
257917_at	At3g23220	0,94	1,03	0,95	0,84	0,96	0,94	1,02	0,92	ethylene-responsive element-binding protein, putative
257918_at	At3g23230	1,00	1,13	0,97	0,52	1,08	0,87	0,97	1,20	ethylene-responsive factor, putative
257927_at	At3g23240	0,91	0,94	1,18	0,81	1,08	1,14	0,93	1,05	ethylene-responsive factor 1 / ethylene response factor 1 (ERF1)
257919_at	At3g23250	1,42	2,82	1,09	1,08	1,42	2,01	2,15	1,61	myb family transcription factor (MYB15)
257920_at	At3g23260	1,04	0,79	0,69	0,89	0,80	1,07	0,97	1,13	F-box family protein
257921_at	At3g23270	0,95	0,88	0,98	0,96	1,06	0,97	1,01	0,99	regulator of chromosome condensation (RCC1) family protein
257926_at	At3g23280	0,82	0,75	0,80	1,00	1,03	0,87	0,91	0,95	zinc finger (C3HC4-type RING finger) family protein / ankyrin repeat family
256942_at	At3g23290	1,49	1,69	1,31	1,01	1,16	1,00	0,98	0,84	expressed protein

258288_at	At3g23295	1,70	1,47	1,43	1,07	1,15	0,99	0,92	0,92	expressed protein
258298_at	At3g23300	1,08	0,87	0,83	1,20	1,15	0,97	0,80	0,78	dehydration-responsive protein-related
258291_at	At3g23310	1,24	1,08	1,30	0,86	0,83	1,11	1,09	1,66	protein kinase, putative
258297_at	At3g23325	1,05	0,83	0,67	1,26	1,00	0,93	0,81	0,73	splicing factor, putative
258292_at	At3g23330	1,27	1,07	0,95	0,92	0,72	1,09	1,02	0,98	pentatricopeptide (PPR) repeat-containing protein
258300_at	At3g23340	1,29	1,43	1,91	0,87	0,89	1,68	1,94	1,88	casein kinase, putative
258294_at	At3g23350	1,01	1,08	0,94	0,98	0,98	0,88	0,97	0,91	expressed protein
258296_at	At3g23390	1,44	1,18	1,06	1,22	1,23	0,98	1,01	0,98	60S ribosomal protein L36a/L44 (RPL36aA)
258295_at	At3g23400	0,83	0,96	1,01	1,02	1,00	1,07	1,03	1,06	plastid-lipid associated protein PAP / fibrillin family protein
258299_at	At3g23410	0,96	1,25	1,34	0,66	0,75	1,43	2,30	2,39	alcohol oxidase-related
258293_at	At3g23430	1,33	2,17	2,04	1,11	1,12	0,92	1,54	1,34	phosphate transporter, putative (PHO1)
258289_at	At3g23450	1,00	0,99	0,96	0,92	1,06	1,04	1,10	0,91	---
258290_at	At3g23460	1,05	1,01	0,96	1,07	1,27	1,04	1,09	0,96	cyclopropane fatty acid synthase-related
257175_s_at	At3g23470	0,96	1,07	1,08	1,19	0,95	0,75	0,99	1,02	cyclopropane-fatty-acyl-phospholipid synthase family protein
257177_at	At3g23490	1,02	1,04	0,97	1,09	1,18	1,00	1,04	1,03	cyanate lyase family
257176_s_at	At3g23510	1,06	0,87	0,78	1,10	0,89	1,12	1,17	1,03	cyclopropane fatty acid synthase, putative / CPA-FA synthase, putative
258125_s_at	At3g23520	0,92	0,97	0,84	1,01	0,90	1,28	1,00	1,26	cyclopropane fatty acid synthase, putative / CPA-FA synthase, putative
258099_at	At3g23540	1,07	1,18	1,50	1,06	1,12	1,19	1,13	1,30	expressed protein
258100_at	At3g23550	0,96	1,17	1,51	0,96	1,01	0,75	0,77	0,94	MATE efflux family protein
258107_at	At3g23560	0,94	0,91	0,83	1,09	0,79	0,93	0,89	0,91	MATE efflux family protein
258108_at	At3g23570	1,19	1,14	1,03	1,12	1,01	1,11	1,14	1,16	dienelactone hydrolase family protein
258106_at	At3g23580	1,35	1,04	1,22	1,06	0,89	1,14	1,23	1,18	ribonucleoside-diphosphate reductase small chain / ribonucleotide reductas
258101_at	At3g23590	0,99	0,69	0,73	1,10	0,94	1,06	1,08	0,91	expressed protein
258102_at	At3g23600	0,97	0,82	0,79	1,11	1,12	0,98	0,95	1,05	dienelactone hydrolase family protein
258105_at	At3g23605	0,89	0,98	1,03	1,25	1,19	1,04	1,10	1,11	UBX domain-containing protein
258095_at	At3g23610	0,98	1,00	0,79	0,73	0,88	0,84	0,94	1,04	dual specificity protein phosphatase (DsPTP1)
258104_at	At3g23620	1,07	0,86	0,85	1,01	0,97	0,99	0,86	0,77	brix domain-containing protein
258103_at	At3g23630	1,07	1,05	1,26	1,27	1,04	1,28	1,05	0,88	adenylate isopentenyltransferase 7 / cytokinin synthase (IPT7)
258109_at	At3g23640	0,72	0,74	0,80	0,82	0,75	0,93	0,97	0,91	glycosyl hydrolase family 31 protein
258096_at	At3g23650	1,02	1,03	1,02	0,92	1,05	1,00	1,02	0,96	protein kinase-related
258097_at	At3g23660	1,22	1,04	1,15	0,97	1,11	0,90	0,85	0,83	transport protein, putative
258098_at	At3g23670	1,12	0,83	1,08	1,16	1,12	0,88	0,95	1,01	phragmoplast-associated kinesin-related protein, putative
257195_at	At3g23680	0,99	1,04	0,98	1,00	0,96	1,07	1,01	0,94	F-box family protein-related
257198_at	At3g23690	0,99	0,77	0,74	1,12	1,26	0,88	0,88	0,80	basic helix-loop-helix (bHLH) family protein
257172_at	At3g23700	1,04	1,04	1,09	0,66	0,70	1,16	1,15	1,08	S1 RNA-binding domain-containing protein
257199_at	At3g23710	1,16	1,12	1,40	0,94	0,90	0,94	1,03	0,99	chloroplast inner membrane import protein Tic22, putative
257200_at	At3g23720	1,02	0,96	1,03	0,95	1,03	1,00	1,08	0,99	hypothetical protein
257203_at	At3g23730	1,39	1,00	1,10	1,09	1,28	0,86	0,77	0,78	xyloglucan:xyloglucosyl transferase, putative / xyloglucan endotransglycosy
257201_at	At3g23740	1,09	1,10	1,16	0,97	1,02	1,15	1,14	1,00	expressed protein
257202_at	At3g23750	0,87	0,81	0,86	0,99	1,02	0,95	1,01	1,21	leucine-rich repeat family protein / protein kinase family protein
257171_at	At3g23760	1,02	0,98	0,93	0,85	0,90	0,89	0,81	0,76	expressed protein
257170_at	At3g23770	0,97	0,83	1,06	0,92	1,11	0,89	1,02	0,99	glycosyl hydrolase family 17 protein
257196_at	At3g23790	0,94	0,93	0,88	0,87	1,03	1,18	0,99	0,93	AMP-binding protein, putative
257197_at	At3g23800	1,08	1,20	1,01	2,44	1,95	0,86	1,00	1,30	selenium-binding family protein
257204_at	At3g23805	1,22	1,17	0,96	1,39	1,39	0,99	0,93	0,83	rapid alkalinization factor (RALF) family protein
257173_at	At3g23810	1,21	1,09	1,03	1,84	1,22	0,87	0,80	0,75	adenosylhomocysteinase, putative / S-adenosyl-L-homocysteine hydrolase

256865_at	At3g23820	0,92	0,79	0,68	1,13	1,08	0,95	0,83	0,81	NAD-dependent epimerase/dehydratase family protein
256890_at	At3g23830	1,28	1,08	0,99	1,31	1,36	1,01	1,06	0,90	glycine-rich RNA-binding protein, putative
256860_at	At3g23840	0,78	0,92	0,84	0,87	0,95	0,83	0,85	1,02	transferase family protein
256912_at	At3g23850	0,98	0,99	0,97	1,02	1,13	1,00	0,91	1,19	hypothetical protein
256913_at	At3g23870	1,09	1,30	1,24	1,21	1,01	1,00	1,47	1,38	permease-related
256914_at	At3g23880	1,38	1,67	1,36	0,77	0,93	1,21	1,13	0,94	F-box family protein
256864_at	At3g23890	1,67	1,24	1,09	1,42	1,52	0,95	1,10	1,03	DNA topoisomerase, ATP-hydrolyzing / DNA topoisomerase II / DNA gyras
256915_at	At3g23900	1,14	1,01	1,19	0,83	1,03	1,18	1,21	1,27	RNA recognition motif (RRM)-containing protein
256902_s_at	At3g23910	0,82	0,81	0,88	1,05	1,09	1,00	1,00	0,93	expressed protein
256861_at	At3g23920	1,19	1,76	3,65	1,13	1,46	1,35	1,45	1,60	beta-amylase, putative / 1,4-alpha-D-glucan maltohydrolase, putative
257567_at	At3g23930	1,08	1,04	1,11	1,06	1,01	1,03	1,11	1,00	expressed protein
256862_at	At3g23940	1,23	1,06	1,00	0,80	0,68	0,91	0,89	0,93	dehydratase family
256903_at	At3g23960	0,97	1,11	1,06	0,99	1,01	1,00	1,08	0,78	F-box family protein
257568_s_at	At3g23970	1,01	0,95	1,00	0,84	0,96	0,98	1,04	0,99	F-box family protein
256904_at	At3g23980	0,96	0,89	1,15	0,99	1,05	1,00	1,11	1,28	dentin sialophosphoprotein-related
256905_at	At3g23990	1,42	1,23	1,13	1,26	1,18	1,07	1,07	1,06	chaperonin (CPN60) (HSP60)
256906_at	At3g24000	1,13	1,22	1,19	0,80	0,82	1,02	1,02	1,16	pentatricopeptide (PPR) repeat-containing protein
256889_at	At3g24010	1,16	1,12	1,03	1,04	0,98	1,16	1,16	1,11	PHD finger family protein
256907_at	At3g24030	0,82	0,74	1,00	1,01	1,23	0,80	0,84	1,00	hydroxyethylthiazole kinase family protein
256908_at	At3g24040	0,82	0,80	1,07	1,12	1,27	0,89	0,90	0,77	glycosyltransferase family 14 protein / core-2/I-branching enzyme family pro
256916_at	At3g24050	0,74	0,88	0,77	1,00	0,74	0,97	0,94	1,40	GATA transcription factor 1 (GATA-1)
256909_at	At3g24060	0,86	1,05	0,96	1,06	0,97	1,11	1,09	0,95	self-incompatibility protein-related
256863_at	At3g24070	0,67	0,75	1,00	0,87	0,79	1,01	1,06	1,01	zinc knuckle (CCHC-type) family protein
256910_at	At3g24080	1,01	0,72	0,80	0,93	1,19	0,92	1,04	1,25	KRR1 family protein
256911_at	At3g24090	0,77	0,81	1,04	1,02	0,99	0,85	0,90	0,95	glucosamine--fructose-6-phosphate aminotransferase (isomerizing), putativ
257239_at	At3g24100	1,39	1,17	1,19	1,28	1,35	0,98	0,95	0,90	four F5 family protein / 4F5 family protein
257245_at	At3g24110	1,01	0,89	1,02	0,96	1,05	1,02	0,95	0,91	calcium-binding EF hand family protein
257250_at	At3g24120	1,15	1,34	1,72	1,17	1,20	1,06	1,09	1,41	myb family transcription factor
257246_at	At3g24130	0,95	1,04	0,99	0,99	1,11	0,96	0,93	1,10	pectinesterase family protein
257247_at	At3g24140	0,94	1,15	1,01	0,89	0,94	1,18	1,17	1,15	basic helix-loop-helix (bHLH) family protein
257248_at	At3g24150	0,90	0,80	1,08	1,13	1,20	1,04	1,16	1,13	expressed protein
257251_at	At3g24160	0,99	1,01	0,90	1,17	1,07	0,96	0,92	0,94	expressed protein
257252_at	At3g24170	1,63	1,46	1,31	2,15	1,78	1,31	1,27	1,30	glutathione reductase, putative
257249_at	At3g24180	0,86	0,87	0,77	1,06	1,19	1,05	1,02	1,10	expressed protein
257253_at	At3g24190	1,15	1,04	1,08	0,80	0,88	1,62	1,48	1,63	ABC1 family protein
257240_at	At3g24200	1,14	1,01	1,08	0,88	0,94	1,04	1,00	0,92	monooxygenase family protein
257241_at	At3g24210	1,17	1,00	1,09	1,07	1,12	0,94	0,91	1,03	ankyrin repeat family protein
257242_at	At3g24220	1,03	1,01	1,08	1,04	1,11	0,96	1,10	0,82	9-cis-epoxycarotenoid dioxygenase, putative / neoxanthin cleavage enzyme
257243_at	At3g24230	0,98	0,97	1,06	0,98	0,97	0,88	1,00	0,98	pectate lyase family protein
257244_at	At3g24240	1,28	1,13	1,01	1,33	1,40	1,08	1,33	1,14	leucine-rich repeat transmembrane protein kinase, putative
256645_at	At3g24250	1,07	1,02	0,99	0,85	1,02	0,97	0,96	1,05	glycine-rich protein
257156_at	At3g24260	0,89	1,01	0,99	1,14	1,14	0,98	1,13	1,01	hypothetical protein
257561_at	At3g24270	1,03	1,05	1,07	0,99	1,06	0,89	1,03	1,05	hypothetical protein
257161_at	At3g24280	1,06	0,97	0,92	0,99	0,95	1,03	1,03	1,02	expressed protein
257162_s_at	At3g24290	0,89	1,06	0,98	1,07	1,05	1,02	0,96	1,00	ammonium transporter, putative
257163_at	At3g24310	0,96	0,96	0,91	1,05	1,05	0,99	0,98	1,05	myb family transcription factor

257169_at	At3g24315	0,87	0,92	1,06	0,92	1,03	1,01	0,99	1,11	sec20 family protein
257164_at	At3g24320	0,90	0,94	1,14	0,72	0,64	1,03	1,16	1,13	DNA mismatch repair MutS family (MSH1)
257165_at	At3g24330	1,02	1,06	0,89	0,96	0,93	1,13	0,90	1,15	glycosyl hydrolase family 17 protein
257167_at	At3g24340	1,10	1,03	0,97	0,92	1,03	0,95	0,91	0,91	SNF2 domain-containing protein / helicase domain-containing protein
257157_at	At3g24350	0,85	0,80	0,92	1,06	1,24	1,00	0,86	0,94	syntaxin, putative (SYP32)
257158_at	At3g24360	0,90	1,11	0,96	0,87	1,04	1,05	1,04	0,94	enoyl-CoA hydratase/isomerase family protein
257159_at	At3g24400	1,04	1,05	1,03	0,91	0,98	1,00	1,03	1,12	---
257160_at	At3g24410	0,88	0,81	1,06	0,84	1,06	1,13	1,15	1,07	---
257168_at	At3g24430	1,19	1,07	1,02	0,92	0,77	1,21	1,23	1,29	expressed protein
256618_at	At3g24440	1,01	1,08	0,89	0,99	1,31	1,02	1,30	1,25	fibronectin type III domain-containing protein
256621_at	At3g24450	1,48	1,28	1,09	1,40	1,37	1,03	0,98	1,02	copper-binding family protein
256619_at	At3g24470	1,07	1,03	1,13	0,93	0,98	1,19	1,05	1,08	TMS membrane family protein / tumour differentially expressed (TDE) famil
256620_at	At3g24480	0,98	0,98	0,87	0,88	1,23	1,00	1,11	0,83	leucine-rich repeat family protein / extensin family protein
258126_at	At3g24490	0,77	0,76	0,73	0,92	0,77	0,96	0,99	1,11	expressed protein
258138_at	At3g24492	1,02	1,10	1,06	0,99	1,25	0,96	0,99	0,75	DNA mismatch repair protein MSH6-2 (MSH7)
258133_at	At3g24500	1,59	1,57	1,35	1,48	1,76	1,37	1,19	1,27	ethylene-responsive transcriptional coactivator, putative
258140_at	At3g24503	1,03	1,08	1,07	0,76	0,65	1,18	1,21	1,34	aldehyde dehydrogenase (ALDH1a)
258135_at	At3g24506	1,15	1,07	1,05	0,91	1,20	1,06	0,86	0,79	expressed protein
258130_at	At3g24510	0,97	0,87	0,95	0,94	0,94	1,04	1,09	0,99	expressed protein
258137_at	At3g24515	0,98	1,01	1,03	0,93	0,84	1,19	0,99	1,06	ubiquitin-conjugating enzyme, putative
258139_at	At3g24520	0,85	1,26	1,12	0,76	0,90	1,12	1,14	1,10	heat shock transcription factor family protein
258134_at	At3g24530	0,91	0,83	1,34	1,09	1,19	0,90	1,18	1,58	AAA-type ATPase family protein / ankyrin repeat family protein
258131_at	At3g24540	1,05	0,96	1,01	1,06	1,29	0,91	1,05	1,08	protein kinase family protein
258132_at	At3g24550	0,85	0,86	1,20	0,92	0,94	1,10	1,44	1,72	protein kinase family protein
258136_at	At3g24560	1,13	1,26	1,25	0,71	0,87	1,09	1,18	1,01	expressed protein
258127_at	At3g24570	1,05	1,04	1,01	1,21	1,10	0,97	0,85	0,85	peroxisomal membrane 22 kDa family protein
258128_at	At3g24590	0,98	0,85	1,06	0,82	0,97	1,01	1,03	0,90	signal peptidase I family protein
258129_at	At3g24620	1,06	0,86	0,88	0,96	1,14	1,04	1,03	0,94	expressed protein
256896_at	At3g24630	0,94	1,01	1,10	0,93	1,07	0,82	1,04	0,94	hypothetical protein
256897_at	At3g24640	0,93	0,94	1,01	1,03	1,04	1,01	0,87	1,13	hypothetical protein
256898_at	At3g24650	0,91	0,95	0,93	0,91	1,03	1,09	1,03	0,87	abscisic acid-insensitive protein 3 (ABI3)
256899_at	At3g24660	0,82	1,05	0,94	0,95	0,82	0,98	0,91	0,88	leucine-rich repeat transmembrane protein kinase, putative
256900_at	At3g24670	0,96	0,86	1,02	1,51	1,71	1,08	1,05	1,01	pectate lyase family protein
256895_at	At3g24680	0,98	0,93	1,09	1,02	1,34	0,92	0,91	1,05	zinc finger protein, putative
257166_at	At3g24710	0,96	0,94	1,06	0,91	1,10	1,10	0,99	1,11	hypothetical protein
257618_at	At3g24720	0,93	0,91	1,04	1,07	1,02	1,06	1,20	1,26	protein kinase family protein
257594_at	At3g24740	0,87	0,90	0,94	0,83	1,04	1,02	1,05	1,14	expressed protein
257595_at	At3g24750	1,01	0,98	1,07	0,94	1,02	0,97	1,04	0,91	expressed protein
257596_at	At3g24760	0,86	0,97	0,93	0,93	0,96	0,95	0,94	0,84	F-box family protein
257600_at	At3g24770	1,19	1,27	1,11	1,45	1,22	1,03	0,82	0,87	CLE41, putative
257555_at	At3g24780	0,90	0,98	1,09	0,97	1,27	1,05	0,99	0,91	hypothetical protein
257597_at	At3g24790	0,97	1,03	1,01	0,94	0,98	1,01	0,97	1,00	protein kinase family protein
257598_at	At3g24800	1,36	1,14	1,31	1,31	1,35	1,02	0,92	1,05	PRT1 protein (PRT1)
257619_at	At3g24810	1,07	1,07	1,01	1,23	1,24	1,06	1,10	0,94	kip-related protein 5 (KRP5) / cyclin-dependent kinase inhibitor 5 (ICK5)
257601_at	At3g24820	1,00	0,94	1,03	1,00	1,11	1,13	0,95	0,96	BSD domain-containing protein
257599_at	At3g24830	1,15	1,20	1,14	1,33	1,22	1,00	0,98	0,91	60S ribosomal protein L13A (RPL13aB)

257593_at	At3g24840	1,14	0,94	0,83	1,19	1,51	1,08	0,78	0,65	SEC14 cytosolic factor, putative / phosphoglyceride transfer protein, putativ
257620_at	At3g24850	1,12	0,94	0,89	1,01	1,05	0,92	1,12	1,09	hypothetical protein
257590_s_at	At3g24870	0,83	1,01	1,30	0,65	0,69	1,11	1,07	1,56	expressed protein
257554_at	At3g24890	1,09	1,05	1,06	1,48	0,97	0,95	0,97	0,89	synaptobrevin-related
257591_at	At3g24900	1,10	1,00	1,21	0,87	1,09	1,05	1,01	1,24	disease resistance family protein / LRR family protein
257592_at	At3g24954	1,08	1,06	0,98	0,92	1,11	0,95	1,01	1,02	leucine-rich repeat family protein
257099_s_at	At3g24982	0,95	0,98	0,92	1,01	1,01	1,03	1,03	0,92	leucine-rich repeat family protein, 5' fragment
257100_at	At3g25010	0,89	0,97	1,00	0,95	1,10	1,02	1,09	0,99	disease resistance family protein
257101_at	At3g25020	1,06	1,03	0,97	1,09	1,03	1,44	1,14	1,14	disease resistance family protein
257104_at	At3g25040	1,33	1,20	1,20	1,03	1,03	1,12	1,00	0,99	ER lumen protein retaining receptor, putative / HDEL receptor, putative
257102_at	At3g25050	1,01	1,13	0,98	0,85	1,04	1,02	1,05	0,98	xyloglucan:xyloglucosyl transferase, putative / xyloglucan endotransglycosy
257103_at	At3g25060	0,92	1,04	0,92	1,03	1,04	1,07	0,88	0,75	pentatricopeptide (PPR) repeat-containing protein
257868_at	At3g25070	0,86	0,84	0,92	0,81	0,91	0,99	1,02	1,21	RPM1-interacting protein 4 (RIN4)
257839_at	At3g25080	1,01	0,98	1,05	0,96	1,24	0,97	0,92	0,76	hypothetical protein
257812_at	At3g25090	0,85	1,11	1,09	1,08	1,04	1,01	1,11	1,33	F-box family protein-related
257813_at	At3g25100	0,92	1,10	1,10	1,28	1,14	0,99	0,88	0,81	cell division control protein-related
257814_at	At3g25110	1,21	1,07	0,88	1,09	1,31	0,87	0,97	0,97	acyl-(acyl carrier protein) thioesterase / acyl-ACP thioesterase / oleoyl-(acyl
257818_at	At3g25120	1,12	0,92	0,82	1,00	1,21	0,96	0,96	0,86	mitochondrial import inner membrane translocase subunit Tim17/Tim22/Tim
257815_at	At3g25130	1,08	0,93	1,11	0,96	0,99	1,09	1,00	1,06	expressed protein
257816_at	At3g25140	0,90	0,71	0,74	0,96	1,17	0,93	0,89	0,92	glycosyl transferase family 8 protein
257817_at	At3g25150	1,13	1,03	1,11	1,09	1,19	0,90	0,95	1,13	nuclear transport factor 2 (NTF2) family protein / RNA recognition motif (RR
257869_at	At3g25160	0,99	1,06	0,98	1,17	1,14	0,81	1,01	1,04	ER lumen protein retaining receptor family protein
257819_at	At3g25165	0,94	1,02	0,95	1,05	1,13	0,88	0,95	1,09	rapid alkalinization factor (RALF) family protein
257821_at	At3g25170	1,01	0,98	1,01	1,06	0,93	0,93	1,01	1,02	rapid alkalinization factor (RALF) family protein
257835_at	At3g25180	0,96	0,89	0,92	1,40	1,54	1,05	0,76	0,74	cytochrome P450 family protein
257836_at	At3g25185	0,88	0,88	1,05	0,98	1,03	0,97	1,00	1,05	---
257823_at	At3g25190	1,16	1,38	1,51	1,19	1,07	0,67	0,70	0,95	nodulin, putative
257837_at	At3g25200	1,00	0,94	0,98	0,95	0,94	0,96	1,03	1,04	hypothetical protein
257838_at	At3g25210	0,86	0,91	0,92	1,08	0,91	0,96	0,99	0,81	pentatricopeptide (PPR) repeat-containing protein
257820_at	At3g25220	1,33	1,05	1,00	1,29	1,29	1,10	0,96	0,91	FK506-binding protein 2-1 (FKBP15-1) / immunophilin / peptidyl-prolyl cis-tr
257822_at	At3g25230	1,31	1,14	1,08	1,30	1,12	1,10	1,19	1,16	peptidyl-prolyl cis-trans isomerase / FK506-binding protein (ROF1)
257840_at	At3g25250	1,08	1,01	1,28	0,78	1,02	1,65	1,06	1,25	protein kinase family protein
257841_at	At3g25260	0,83	0,88	0,65	0,67	0,81	0,99	0,95	1,07	proton-dependent oligopeptide transport (POT) family protein
257811_at	At3g25280	0,97	1,20	1,14	1,19	1,08	0,93	0,91	0,93	proton-dependent oligopeptide transport (POT) family protein
257824_at	At3g25290	0,97	1,18	0,89	1,30	1,29	0,85	0,96	0,79	auxin-responsive family protein
257956_at	At3g25400	0,87	1,03	0,96	1,08	1,48	0,92	1,26	1,23	expressed protein
257908_at	At3g25410	1,11	1,15	1,45	0,70	0,74	1,27	1,26	1,55	bile acid:sodium symporter family protein
257934_at	At3g25420	0,95	0,91	1,03	1,03	1,01	0,95	0,89	0,93	serine carboxypeptidase S10 family protein
257935_at	At3g25440	1,05	1,09	1,04	1,05	1,11	0,96	0,97	0,97	group II intron splicing factor CRS1-related
257936_at	At3g25450	1,01	1,00	1,03	0,91	0,93	0,88	1,08	0,88	---
257907_at	At3g25470	0,88	0,84	0,74	0,97	0,89	0,96	0,98	1,00	bacterial hemolysin-related
257909_at	At3g25480	0,93	0,85	0,79	0,77	0,94	1,03	0,98	0,90	rhodanese-like domain-containing protein
257957_at	At3g25490	0,88	0,95	0,93	0,96	1,01	1,08	1,06	0,94	wall-associated kinase, putative
257912_at	At3g25500	0,88	0,63	0,59	1,12	1,56	0,84	0,79	0,77	formin homology 2 domain-containing protein / FH2 domain-containing prot
257958_at	At3g25510	0,96	1,03	1,08	1,00	1,16	0,92	1,07	1,04	disease resistance protein (TIR-NBS-LRR class), putative
257906_at	At3g25520	1,37	1,17	1,14	1,03	1,00	1,04	1,04	1,09	60S ribosomal protein L5

257911_at	At3g25530	0,77	0,71	0,74	0,99	1,03	0,96	0,93	0,99	6-phosphogluconate dehydrogenase NAD-binding domain-containing prote
257913_at	At3g25540	1,16	1,32	1,11	1,00	1,09	1,00	0,96	0,95	longevity-assurance (LAG1) family protein
257914_at	At3g25545	1,10	1,01	1,11	0,93	0,95	0,81	0,76	1,09	expressed protein
257959_at	At3g25560	1,07	1,12	1,18	0,82	0,98	1,11	1,19	1,20	protein kinase family protein
257960_at	At3g25570	0,89	0,98	0,84	1,20	1,03	0,95	1,28	1,09	adenosylmethionine decarboxylase family protein
257910_at	At3g25580	1,00	0,81	1,03	1,16	1,10	1,07	0,92	0,79	thioredoxin-related
257933_at	At3g25585	0,98	0,89	0,72	1,20	1,04	1,10	1,01	0,88	aminoalcoholphosphotransferase, putative
256755_at	At3g25600	1,03	1,15	1,01	1,14	1,02	1,06	1,11	1,01	calmodulin, putative
256756_at	At3g25610	0,93	1,40	1,26	0,91	0,93	1,20	1,61	1,67	haloacid dehalogenase-like hydrolase family protein
256757_at	At3g25620	0,99	1,16	1,26	0,79	0,90	1,19	1,29	1,03	ABC transporter family protein
256758_at	At3g25630	0,94	1,01	0,99	0,96	1,05	0,99	0,94	1,14	---
256759_at	At3g25640	0,99	1,01	1,11	1,76	1,32	0,93	1,01	1,11	expressed protein
256760_at	At3g25650	1,05	1,07	1,03	0,97	1,09	0,95	1,14	1,10	Skp1 family protein
256762_at	At3g25655	0,93	1,02	0,89	1,14	1,19	1,33	1,10	1,13	expressed protein
256728_at	At3g25660	0,80	0,73	0,69	0,99	0,83	1,00	0,95	1,04	glutamyl-tRNA(Gln) amidotransferase, putative
256761_at	At3g25670	0,91	0,97	0,98	0,85	0,95	0,91	0,87	0,88	leucine-rich repeat family protein
256729_at	At3g25680	1,15	1,23	1,12	0,94	0,75	1,10	1,01	1,24	expressed protein
256754_at	At3g25690	0,91	0,90	0,90	0,80	0,68	1,16	1,30	1,40	hydroxyproline-rich glycoprotein family protein
257642_at	At3g25710	1,64	2,08	1,04	2,00	2,52	1,00	1,26	1,15	basic helix-loop-helix (bHLH) family protein
257639_at	At3g25720	0,94	0,88	1,01	1,04	1,01	1,03	0,99	0,82	hypothetical protein
257643_at	At3g25730	0,67	0,78	0,87	1,12	1,00	1,12	1,37	1,37	AP2 domain-containing transcription factor, putative
257646_at	At3g25740	0,87	0,68	1,09	0,88	0,95	1,07	1,14	1,33	metallopeptidase M24 family protein
257640_at	At3g25750	1,00	0,94	1,09	1,03	1,08	0,98	1,06	0,88	F-box family protein
257641_s_at	At3g25760	0,83	0,77	0,88	1,25	1,17	0,99	0,93	0,88	early-responsive to dehydration stress protein (ERD12)
257644_at	At3g25780	1,01	1,06	1,64	1,77	1,62	1,09	1,32	1,44	allene oxide cyclase, putative / early-responsive to dehydration protein, puta
257645_at	At3g25790	0,89	0,85	0,93	1,10	0,99	0,97	1,00	1,16	myb family transcription factor
259408_at	At3g25800	0,90	0,90	0,92	0,98	1,04	0,95	0,89	0,94	serine/threonine protein phosphatase 2A (PP2A) 65 KDa regulatory subunit
257647_at	At3g25805	0,87	0,77	0,73	0,73	0,51	0,96	0,93	0,82	expressed protein
257637_at	At3g25810	0,96	1,03	1,02	0,96	0,93	1,07	1,02	1,08	myrcene/ocimene synthase, putative
257638_at	At3g25820	0,88	0,97	0,91	0,99	0,60	0,76	1,36	1,33	---
256994_s_at	At3g25830	0,37	0,38	0,48	0,68	0,61	0,74	1,45	1,81	myrcene/ocimene synthase, putative
256995_at	At3g25840	1,13	0,99	1,20	0,86	0,83	0,96	1,25	1,48	protein kinase family protein
256996_at	At3g25850	1,01	0,96	1,12	0,96	1,05	0,95	1,15	0,82	DC1 domain-containing protein
258083_at	At3g25855	1,03	0,95	0,94	1,04	1,31	1,05	0,95	0,80	expressed protein
258086_at	At3g25860	0,79	0,71	0,71	0,91	0,93	0,76	0,78	0,75	dihydrolipoamide S-acetyltransferase (LTA2)
258078_at	At3g25870	0,52	0,66	0,86	1,32	1,36	1,01	0,95	0,95	expressed protein
258073_at	At3g25880	0,96	0,82	0,92	0,99	0,86	0,98	0,94	1,01	auxin-resistance protein, putative
258074_at	At3g25890	0,68	0,61	0,77	0,72	0,83	0,69	0,61	0,74	AP2 domain-containing transcription factor, putative
258075_at	At3g25900	1,47	1,57	1,59	1,55	1,17	0,92	1,08	1,12	homocysteine S-methyltransferase 1 (HMT-1)
258082_at	At3g25905	1,10	0,97	1,10	0,96	1,09	0,89	0,94	0,79	CLE27, putative
258061_at	At3g25910	1,13	1,07	1,13	1,03	1,06	1,12	0,96	1,19	expressed protein
258076_at	At3g25920	0,94	0,87	0,84	0,93	0,95	1,02	0,98	1,01	50S ribosomal protein L15, chloroplast (CL15)
258080_at	At3g25930	1,36	1,49	1,17	1,26	1,00	1,29	1,33	1,10	universal stress protein (USP) family protein
258079_at	At3g25940	1,14	1,01	1,11	1,21	1,02	1,03	0,90	1,05	transcription factor S-II (TFIIS) domain-containing protein
257541_at	At3g25950	0,91	0,99	0,97	1,16	1,04	0,97	0,99	1,00	hypothetical protein
258065_at	At3g25960	1,05	1,01	0,94	1,01	0,96	1,15	1,18	1,07	pyruvate kinase, putative

258066_at	At3g25970	0,96	1,07	1,02	0,97	0,93	0,97	0,97	1,02	pentatricopeptide (PPR) repeat-containing protein
258067_at	At3g25980	1,22	1,34	1,12	2,23	1,98	0,81	0,75	0,72	mitotic spindle checkpoint protein, putative (MAD2)
258068_at	At3g25990	1,14	0,85	1,10	0,97	1,65	1,22	0,91	1,08	DNA-binding protein GT-1-related
258062_at	At3g26000	1,14	1,16	0,98	1,12	0,73	1,09	1,00	1,05	F-box family protein
258069_at	At3g26010	0,92	0,99	0,93	1,09	0,97	1,03	0,97	0,95	F-box family protein
258084_at	At3g26020	1,00	0,82	0,92	0,89	1,00	1,06	0,83	0,97	serine/threonine protein phosphatase 2A (PP2A) regulatory subunit B', puta
258060_at	At3g26030	1,12	1,01	1,08	1,33	0,97	1,05	0,86	0,83	serine/threonine protein phosphatase 2A (PP2A) regulatory subunit B', puta
258070_at	At3g26040	0,90	0,68	1,04	0,83	1,22	1,05	0,95	0,96	transferase family protein
257542_at	At3g26050	1,12	1,04	0,91	0,91	0,93	1,00	1,02	0,98	expressed protein
258087_at	At3g26060	0,86	0,75	0,74	0,73	0,76	0,92	0,98	1,00	peroxiredoxin Q, putative
258071_s_at	At3g26070	1,11	1,06	1,10	0,84	0,88	1,11	1,07	0,94	plastid-lipid associated protein PAP / fibrillin family protein
258081_at	At3g26085	1,30	1,26	1,17	0,71	0,62	1,42	1,57	1,47	CAAX amino terminal protease family protein
258072_at	At3g26090	0,75	0,63	1,02	0,99	1,20	0,90	0,77	0,98	expressed protein
258085_at	At3g26100	0,69	0,70	0,78	1,11	1,03	0,91	1,00	1,19	regulator of chromosome condensation (RCC1) family protein
258077_at	At3g26110	1,04	1,08	1,04	0,98	0,93	0,93	0,99	0,92	expressed protein
257012_at	At3g26120	1,01	0,99	1,19	1,07	0,92	0,92	0,92	0,93	RNA-binding protein, putative
257633_at	At3g26125	1,03	1,06	1,03	1,08	1,17	1,04	0,98	0,88	cytochrome P450, putative
257552_at	At3g26130	0,97	1,05	0,96	1,01	0,98	0,98	1,00	0,94	glycosyl hydrolase family 5 protein / cellulase family protein
257629_at	At3g26140	1,06	1,13	1,20	0,92	1,20	0,99	0,91	0,93	glycosyl hydrolase family 5 protein / cellulase family protein
257630_at	At3g26150	0,94	1,10	1,08	0,90	1,02	0,99	0,94	1,09	cytochrome P450 71B16, putative (CYP71B16)
257631_at	At3g26160	0,96	0,96	0,88	0,98	0,97	0,96	1,08	0,92	cytochrome P450 family protein
257634_s_at	At3g26170	0,81	0,89	1,05	0,89	1,19	1,11	1,29	1,28	cytochrome P450 71B19, putative (CYP71B19)
257632_at	At3g26190	0,90	0,93	1,00	1,01	1,05	1,00	0,91	1,01	cytochrome P450 71B21, putative (CYP71B21)
257636_at	At3g26200	1,55	1,44	1,22	1,09	0,91	0,54	0,86	0,81	cytochrome P450 71B22, putative (CYP71B22)
257623_at	At3g26210	1,15	1,27	1,41	0,95	0,96	1,21	1,08	1,02	cytochrome P450 71B23, putative (CYP71B23)
257624_at	At3g26220	0,70	0,89	1,00	0,96	0,95	1,00	1,09	0,93	cytochrome P450 family protein
257625_at	At3g26230	0,61	0,61	0,69	1,12	1,06	1,25	1,00	0,94	cytochrome P450 family protein
257626_s_at	At3g26240	1,29	1,02	1,08	0,99	0,99	1,02	1,05	0,94	DC1 domain-containing protein
257627_at	At3g26270	1,05	0,91	0,98	1,04	1,06	0,97	0,97	0,99	cytochrome P450 71B25, putative (CYP71B25)
257635_at	At3g26280	1,04	1,03	1,09	0,93	1,05	1,17	1,17	1,07	cytochrome P450 family protein
257628_at	At3g26290	1,11	1,08	1,05	1,09	1,27	0,89	1,06	0,78	cytochrome P450 71B26, putative (CYP71B26)
256870_at	At3g26300	0,77	0,73	0,69	0,82	0,92	0,90	1,01	1,13	cytochrome P450 family protein
256873_at	At3g26310	0,76	0,80	0,88	0,96	0,91	1,04	1,11	0,97	cytochrome P450 family protein
256874_at	At3g26320	1,05	0,94	0,97	0,85	1,03	1,15	1,00	1,13	cytochrome P450 71B36, putative (CYP71B36)
256875_at	At3g26330	1,02	0,98	1,06	1,27	1,21	0,94	0,99	1,09	cytochrome P450 family protein
257569_at	At3g26350	0,94	1,10	0,88	0,99	1,02	1,13	1,16	1,30	expressed protein
256876_at	At3g26360	1,08	0,94	0,81	0,95	1,24	1,07	0,99	0,76	ribosomal protein-related
256879_at	At3g26370	0,93	0,81	0,81	0,85	1,00	0,87	0,85	0,92	expressed protein
256866_at	At3g26380	0,81	0,68	0,72	0,92	0,97	0,91	0,98	0,96	glycosyl hydrolase family protein 27 / alpha-galactosidase family protein / m
256867_at	At3g26390	0,99	0,94	0,95	1,04	1,07	1,12	0,98	1,23	hypothetical protein
256868_at	At3g26400	0,85	0,82	0,64	1,12	0,98	0,99	0,86	0,94	eukaryotic translation initiation factor 4B, putative/ eIF-4B, putative
256881_at	At3g26410	1,23	1,33	1,45	1,13	1,27	0,96	1,00	0,80	expressed protein
256882_at	At3g26420	0,86	0,86	0,84	0,97	1,03	0,87	0,87	0,91	glycine-rich RNA-binding protein
256869_at	At3g26430	1,14	0,64	0,75	0,78	1,09	0,94	0,95	0,82	glycine-rich RNA-binding protein
256883_at	At3g26440	1,39	1,62	1,39	1,25	1,44	1,31	1,54	1,58	expressed protein
256880_at	At3g26450	1,34	1,14	1,18	1,03	0,82	1,01	1,03	1,18	major latex protein-related / MLP-related

256878_at	At3g26460	1,21	1,16	0,89	1,15	0,76	1,07	1,17	1,03 major latex protein-related / MLP-related
256877_at	At3g26470	1,55	1,29	1,16	1,51	1,30	1,14	1,24	1,15 expressed protein
256871_at	At3g26480	0,97	1,10	1,16	0,94	0,99	0,96	1,04	1,00 transducin family protein / WD-40 repeat family protein
256872_at	At3g26490	1,11	1,16	1,05	0,85	0,72	1,04	0,88	1,15 phototropic-responsive NPH3 family protein
257615_at	At3g26510	3,02	2,39	1,76	1,03	0,82	1,00	1,12	0,97 octicosapeptide/Phox/Bem1p (PB1) domain-containing protein
257313_at	At3g26520	1,21	1,12	1,11	1,34	1,37	0,98	0,93	0,93 tonoplast intrinsic protein, putative
257616_at	At3g26540	0,99	0,91	0,92	0,89	0,96	1,11	1,14	0,98 pentatricopeptide (PPR) repeat-containing protein
257617_at	At3g26550	1,05	0,98	1,08	1,02	1,13	0,95	1,04	1,34 DC1 domain-containing protein
257310_at	At3g26560	1,31	1,26	1,41	0,96	0,87	1,06	1,01	0,94 ATP-dependent RNA helicase, putative
257311_at	At3g26570	1,30	1,39	1,29	1,08	1,12	1,23	1,12	1,10 phosphate transporter family protein
257611_at	At3g26580	0,76	0,77	1,00	0,63	0,77	0,92	0,98	1,12 expressed protein
257314_at	At3g26590	0,99	0,92	0,75	1,14	1,08	1,38	1,29	1,13 MATE efflux family protein
257312_at	At3g26595	0,97	0,89	1,03	1,07	1,22	1,10	0,94	0,97 MATE efflux family protein
257612_at	At3g26600	0,61	0,82	0,95	0,96	1,15	0,81	0,89	0,92 armadillo/beta-catenin repeat family protein
257613_at	At3g26610	0,98	1,05	1,07	0,90	1,09	1,14	1,02	1,03 polygalacturonase, putative / pectinase, putative
257614_at	At3g26614	1,19	1,10	1,04	1,07	0,96	1,13	0,87	0,89 ---
261204_s_at	At3g26618	1,19	1,08	1,00	1,05	1,01	1,02	0,84	0,90 eukaryotic release factor 1 family protein / eRF1 family protein
257548_s_at	At3g26620	0,98	0,90	0,95	0,89	1,03	0,97	0,97	0,82 LOB domain protein, putative / lateral organ boundaries domain protein, pu
257827_at	At3g26630	1,11	1,04	1,28	1,20	1,49	1,14	0,93	0,94 pentatricopeptide (PPR) repeat-containing protein
257833_at	At3g26640	1,02	1,04	1,00	1,08	1,07	0,91	0,91	1,00 transducin family protein / WD-40 repeat family protein
257807_at	At3g26650	1,06	1,00	1,06	0,93	0,89	1,07	1,09	1,04 glyceraldehyde 3-phosphate dehydrogenase A, chloroplast (GAPA) / NADF
257828_at	At3g26670	0,87	0,81	0,96	1,06	0,95	0,98	0,92	1,01 expressed protein
257829_at	At3g26680	0,67	0,78	0,86	0,80	0,80	0,92	0,95	0,88 DNA cross-link repair protein-related
257830_at	At3g26690	0,64	0,62	0,65	0,78	0,78	0,85	1,06	1,29 MutT/nudix family protein
257825_at	At3g26700	1,05	0,92	0,96	1,05	1,03	1,01	0,92	0,84 protein kinase family protein
257831_at	At3g26710	1,17	1,12	1,23	0,92	0,78	1,21	1,23	1,27 expressed protein
257834_at	At3g26720	0,83	0,81	0,93	0,92	1,04	0,88	0,97	0,85 glycosyl hydrolase family 38 protein
257826_at	At3g26730	1,02	1,06	0,98	0,67	0,79	1,08	1,08	0,96 zinc finger (C3HC4-type RING finger) family protein
257832_at	At3g26740	1,01	1,33	2,28	0,78	0,79	1,18	1,10	1,54 light responsive protein-related
258310_at	At3g26744	0,92	0,95	1,02	1,01	0,76	0,91	0,90	0,94 basix helix-loop-helix (bHLH) family protein
258283_at	At3g26750	0,94	1,02	0,95	1,11	1,31	0,76	0,87	1,01 expressed protein
258253_at	At3g26760	1,19	1,46	1,07	2,04	1,47	0,95	0,97	1,05 short-chain dehydrogenase/reductase (SDR) family protein
258257_at	At3g26770	1,08	1,08	1,07	1,39	1,36	0,91	1,05	1,14 short-chain dehydrogenase/reductase (SDR) family protein
258254_at	At3g26780	1,35	1,07	0,77	0,94	0,89	0,75	0,90	0,91 phosphoglycerate/bisphosphoglycerate mutase family protein
258258_at	At3g26790	1,06	1,02	1,01	1,01	1,10	0,95	0,98	0,94 transcriptional regulator (FUSCA3)
258255_at	At3g26800	0,95	0,96	1,04	0,93	0,81	1,08	1,06	1,02 expressed protein
258311_at	At3g26810	1,08	1,00	1,00	0,99	1,06	0,97	0,92	0,98 transport inhibitor response protein, putative
258277_at	At3g26830	1,08	1,03	1,04	0,83	0,83	1,69	1,77	1,67 cytochrome P450 71B15, putative (CYP71B15)
258259_s_at	At3g26840	1,14	0,93	1,05	1,03	1,23	1,18	1,10	1,19 esterase/lipase/thioesterase family protein
258260_at	At3g26850	1,29	1,08	1,07	1,06	1,11	1,28	1,00	0,97 expressed protein
258278_at	At3g26860	0,95	0,99	1,00	1,01	1,06	0,97	1,04	0,96 self-incompatibility protein-related
258279_at	At3g26870	0,96	1,13	1,03	0,98	0,94	1,00	0,93	0,97 self-incompatibility protein-related
258280_at	At3g26880	0,97	1,02	0,95	0,98	0,99	1,05	0,94	1,13 self-incompatibility protein-related
258256_at	At3g26890	0,96	0,96	1,29	0,68	0,86	1,22	1,41	1,76 expressed protein
258281_at	At3g26900	0,91	0,77	0,81	0,84	0,80	0,91	0,98	0,99 shikimate kinase family protein
258282_at	At3g26910	0,68	1,03	1,08	0,66	0,87	0,90	1,26	1,58 hydroxyproline-rich glycoprotein family protein

257013_at	At3g26920	1,01	0,96	0,92	1,08	1,08	0,92	0,94	1,11	F-box family protein
257014_at	At3g26930	1,07	1,06	1,00	0,86	1,03	0,92	0,92	1,08	hypothetical protein
256980_at	At3g26932	0,99	1,11	1,06	0,97	1,11	1,10	1,06	1,05	double-stranded RNA-binding domain (DsRBD)-containing protein
257779_at	At3g26940	1,04	1,05	1,13	0,87	0,98	1,08	1,08	1,02	protein kinase family protein
257783_at	At3g26950	0,93	0,86	0,76	1,07	1,06	1,04	1,03	0,98	expressed protein
257793_at	At3g26960	0,69	0,47	0,59	1,16	1,59	0,72	1,05	0,94	expressed protein
257784_at	At3g26970	0,89	0,74	1,02	1,09	1,18	1,13	1,11	1,17	ubiquitin family protein
257785_at	At3g26980	0,82	0,76	1,06	1,05	1,19	0,98	1,14	1,23	ubiquitin family protein
257786_at	At3g26990	1,00	1,05	1,05	0,92	0,90	1,03	0,89	1,06	expressed protein
257787_at	At3g27000	0,99	0,94	0,77	1,11	1,26	1,16	0,97	0,98	actin-related protein 2 (ARP2)
257788_at	At3g27010	0,96	0,89	0,96	1,15	0,99	0,92	0,90	1,22	TCP family transcription factor, putative
257789_at	At3g27020	0,79	0,91	0,85	0,73	0,78	1,00	0,83	0,84	oligopeptide transporter OPT family protein
257794_at	At3g27050	1,23	1,29	1,20	1,07	0,97	1,25	1,37	1,02	expressed protein
257809_at	At3g27060	2,93	3,45	3,13	2,11	1,52	1,27	1,21	1,24	ribonucleoside-diphosphate reductase small chain, putative / ribonucleotide
257810_at	At3g27070	0,96	0,95	1,07	0,99	1,24	1,08	1,05	1,24	mitochondrial import receptor subunit TOM20-1 / translocase of outer meml
257792_at	At3g27080	1,09	0,88	0,91	1,08	1,08	0,95	0,82	0,77	mitochondrial import receptor subunit TOM20-3 / translocase of outer mem
257790_at	At3g27090	1,08	1,11	1,10	0,94	1,04	1,05	1,01	1,00	expressed protein
257780_at	At3g27100	0,76	0,77	0,75	1,08	1,18	0,95	0,87	0,76	expressed protein
257791_at	At3g27110	0,69	0,64	0,56	0,79	0,81	0,97	1,09	1,10	peptidase M48 family protein
257781_at	At3g27120	0,95	1,08	0,91	1,11	1,11	0,91	1,06	0,85	spastin ATPase, putative
257782_at	At3g27130	1,10	1,14	0,98	1,04	1,06	1,02	0,94	0,96	hypothetical protein
256750_at	At3g27150	1,04	1,11	1,18	1,29	1,28	1,30	1,15	1,00	kelch repeat-containing F-box family protein
256753_at	At3g27160	0,98	0,90	0,91	0,88	0,96	1,08	1,01	1,03	ribosomal protein S21 family protein
256751_at	At3g27170	1,79	0,86	0,88	1,88	1,88	1,35	1,08	1,04	chloride channel protein (CLC-b)
256752_at	At3g27180	1,14	1,04	0,96	0,90	1,02	0,98	0,97	0,90	expressed protein
257174_at	At3g27190	1,18	1,07	1,02	1,32	1,32	0,91	0,97	1,00	uracil phosphoribosyltransferase, putative / UMP pyrophosphorylase, putati
257151_at	At3g27200	1,60	1,62	1,51	1,47	1,10	1,21	1,18	1,32	plastocyanin-like domain-containing protein
257154_at	At3g27210	0,72	1,27	1,50	1,14	1,24	1,14	1,60	1,78	expressed protein
257153_at	At3g27220	1,19	0,97	1,16	1,10	1,53	0,56	0,49	0,58	kelch repeat-containing protein
257150_at	At3g27230	0,75	0,64	0,64	1,23	1,15	0,79	0,72	0,64	expressed protein
257148_at	At3g27240	1,24	1,09	1,05	1,02	1,00	0,88	0,91	0,85	cytochrome c1, putative
257146_at	At3g27260	0,99	1,01	0,97	0,80	1,02	1,08	1,19	1,29	DNA-binding bromodomain-containing protein
257147_at	At3g27270	0,89	1,04	1,05	1,30	0,90	0,97	1,00	1,00	expressed protein
257149_at	At3g27280	1,94	1,59	1,32	1,41	1,18	0,95	0,93	0,97	prohibitin, putative
257155_at	At3g27290	1,00	0,94	0,93	0,88	1,19	0,78	0,99	1,09	F-box family protein-related
257144_at	At3g27300	0,80	0,72	0,70	0,94	1,00	1,00	0,97	0,99	glucose-6-phosphate 1-dehydrogenase / G6PD (ACG9)
257152_at	At3g27310	1,03	1,13	1,22	1,16	0,88	1,02	1,03	0,95	expressed protein
257145_at	At3g27320	1,00	1,04	1,21	0,85	1,16	0,90	0,85	0,94	expressed protein
257709_at	At3g27325	1,03	0,98	0,92	1,48	1,09	0,83	0,95	0,73	expressed protein
257740_at	At3g27330	1,32	1,12	0,97	1,14	1,13	0,92	0,96	1,19	zinc finger (C3HC4-type RING finger) family protein
257741_at	At3g27340	0,94	0,81	0,77	0,87	0,79	1,01	1,04	0,84	expressed protein
257710_at	At3g27350	1,70	1,37	0,94	1,20	1,25	1,20	1,12	1,16	expressed protein
257714_at	At3g27360	0,98	0,97	1,11	1,38	1,36	1,02	1,12	0,66	histone H3
257742_at	At3g27370	1,08	0,98	1,03	1,17	0,86	0,98	0,99	1,01	hypothetical protein
257713_at	At3g27380	1,42	1,19	1,15	1,15	1,06	0,98	0,86	0,92	succinate dehydrogenase, iron-sulphur subunit, mitochondrial (SDH2-1)
257743_at	At3g27390	0,89	0,78	0,79	0,83	0,97	0,90	0,85	0,87	expressed protein

257735_at	At3g27400	1,28	1,28	1,08	1,61	1,55	0,90	0,82	0,80	pectate lyase family protein
257736_at	At3g27410	1,08	1,06	0,96	0,91	1,08	1,08	1,00	0,98	expressed protein
257712_at	At3g27420	1,15	1,25	1,35	0,82	1,02	1,13	1,15	1,47	expressed protein
257711_at	At3g27430	1,11	1,03	0,98	1,09	1,23	0,91	0,93	0,89	20S proteasome beta subunit B (PBB1)
257737_at	At3g27440	0,89	0,80	1,09	0,94	1,19	1,00	1,16	1,01	uracil phosphoribosyltransferase, putative / UMP pyrophosphorylase, putati
257738_at	At3g27450	1,16	0,99	1,05	1,14	1,25	0,97	0,90	0,83	---
257739_at	At3g27470	1,14	1,39	1,01	1,02	0,92	1,17	1,38	1,15	expressed protein
258028_at	At3g27473	0,80	0,55	0,46	1,01	1,03	1,00	1,07	0,99	DC1 domain-containing protein
258031_s_at	At3g27490	1,00	0,92	0,95	1,04	1,07	1,06	1,03	1,04	DC1 domain-containing protein
257997_at	At3g27500	0,95	0,96	1,00	1,02	1,08	1,00	0,94	1,03	DC1 domain-containing protein
257998_at	At3g27510	0,94	0,97	1,07	0,99	1,12	0,93	1,06	0,89	DC1 domain-containing protein
257969_at	At3g27520	0,98	0,94	1,04	0,95	1,09	0,90	0,95	1,11	expressed protein
257971_at	At3g27530	1,27	1,01	1,19	0,89	0,97	1,08	1,20	1,43	vesicle tethering family protein
257999_at	At3g27540	1,04	2,63	1,62	1,18	1,19	1,71	1,79	1,78	glycosyl transferase family 17 protein
257968_at	At3g27550	1,00	1,05	1,01	0,95	1,17	1,09	0,89	0,99	group II intron splicing factor CRS1-related
257972_at	At3g27560	0,83	0,82	0,75	0,91	0,89	1,10	1,11	1,26	protein kinase (ATN1)
257970_at	At3g27570	0,86	0,84	0,76	0,91	0,90	1,07	0,95	1,00	expressed protein
258029_at	At3g27580	0,96	1,00	1,07	1,38	1,29	1,03	1,08	1,11	protein kinase, putative
258030_at	At3g27590	0,95	1,06	0,93	1,05	1,19	1,22	1,00	0,84	hypothetical protein
258228_at	At3g27610	0,81	0,81	0,71	0,82	0,90	0,85	0,89	0,78	expressed protein
258235_at	At3g27620	1,29	1,22	1,50	0,97	1,18	0,91	1,21	0,89	alternative oxidase 1c, mitochondrial (AOX1C)
258242_at	At3g27640	0,94	1,14	1,04	1,07	1,02	1,05	1,09	0,93	transducin family protein / WD-40 repeat family protein
258241_at	At3g27650	1,11	0,90	1,12	1,00	1,13	1,01	1,35	1,41	LOB domain protein 25 / lateral organ boundaries domain protein 25 (LBD2
258240_at	At3g27660	0,52	0,58	0,42	1,03	0,74	1,10	1,06	0,88	glycine-rich protein / oleosin
258238_at	At3g27670	0,87	0,93	1,09	0,98	0,70	1,04	0,96	1,12	expressed protein
258239_at	At3g27690	0,92	0,88	1,68	0,91	0,98	1,18	1,05	1,42	chlorophyll A-B binding protein (LHCB2:4)
258229_at	At3g27700	1,03	1,02	0,97	1,07	1,17	1,24	0,98	0,93	RNA recognition motif (RRM)-containing protein
258230_s_at	At3g27710	0,98	1,07	1,01	1,07	1,17	1,00	0,99	0,83	zinc finger protein-related
258231_at	At3g27730	0,92	1,02	1,04	1,12	0,96	0,93	1,13	0,90	ATP-dependent DNA helicase, putative
258243_at	At3g27740	1,39	1,19	1,09	1,13	0,85	0,99	1,01	1,04	carbamoyl-phosphate synthase (glutamine-hydrolyzing) (CARA) / glutamine
258232_at	At3g27750	1,44	1,25	1,16	1,03	1,21	1,06	0,86	0,90	expressed protein
258244_at	At3g27770	0,65	0,60	0,67	1,18	1,07	0,77	0,79	0,88	expressed protein
258233_at	At3g27780	0,90	1,03	0,90	1,01	0,91	0,89	1,02	0,87	myb family transcription factor (MYB118)
258234_at	At3g27785	1,00	0,96	0,98	1,01	1,06	0,91	1,08	1,09	myb family transcription factor (MYB118)
258236_at	At3g27800	0,97	0,99	1,03	0,96	1,09	1,00	1,01	0,96	hypothetical protein
258237_at	At3g27810	1,00	1,05	1,08	1,05	0,96	1,05	0,97	0,89	myb family transcription factor (MYB3) (MYB21)
257220_at	At3g27812	1,06	0,90	1,03	0,97	1,08	1,09	1,06	1,04	myb family transcription factor (MYB3) (MYB21)
257227_at	At3g27820	1,11	1,08	1,24	0,88	0,75	1,21	1,32	1,28	monodehydroascorbate reductase, putative
257223_at	At3g27840	1,05	0,95	0,96	1,00	1,05	1,09	0,96	0,90	50S ribosomal protein L12-2, chloroplast (CL12-B)
257225_s_at	At3g27850	0,98	0,93	0,93	0,91	1,02	1,00	0,98	0,95	50S ribosomal protein L12-1, chloroplast (CL12-A)
257224_at	At3g27870	1,03	1,15	1,06	0,89	0,78	1,00	1,14	1,14	haloacid dehalogenase-like hydrolase family protein
257226_at	At3g27880	0,66	0,76	1,07	1,19	1,22	0,89	0,78	0,87	expressed protein
257228_at	At3g27890	1,02	1,00	1,00	1,16	1,11	1,08	0,90	0,85	NADPH-dependent FMN reductase family protein
257221_at	At3g27920	1,01	1,01	0,95	1,02	1,16	1,13	1,03	1,16	trichome differentiation protein / GLABROUS1 protein (GL1)
257222_at	At3g27925	1,03	1,07	1,36	0,72	0,76	1,14	1,18	1,32	DegP protease, putative
256851_at	At3g27930	0,97	1,09	1,15	0,91	1,10	1,00	1,02	1,00	expressed protein

256847_at	At3g27950	1,07	1,07	1,06	1,09	1,16	0,91	0,98	1,16	early nodule-specific protein, putative
256848_at	At3g27960	0,66	0,71	0,69	1,10	1,30	1,01	0,98	0,89	kinesin light chain-related
256846_at	At3g27980	0,96	0,99	0,98	1,01	1,08	1,03	1,03	0,98	pectinesterase family protein
256849_at	At3g27990	0,99	1,00	1,03	1,12	0,98	1,01	1,03	0,95	---
256850_at	At3g28000	1,05	0,93	1,05	0,99	1,06	0,96	1,02	1,01	---
257271_at	At3g28007	0,96	0,95	1,07	1,18	1,17	0,93	0,97	0,82	nodulin MtN3 family protein
257296_at	At3g28020	0,87	1,03	0,87	0,95	1,10	1,03	0,97	0,77	hypothetical protein
257273_at	At3g28030	0,84	0,87	0,96	0,85	1,02	1,23	0,97	1,04	UV hypersensitive protein (UVH3) / DNA-repair protein, putative
257297_at	At3g28040	0,94	0,90	1,27	0,98	0,77	0,81	0,75	0,92	leucine-rich repeat transmembrane protein kinase, putative
257299_at	At3g28050	0,73	0,81	0,89	0,75	0,58	0,92	0,90	1,04	nodulin MtN21 family protein
257300_at	At3g28080	1,90	2,20	1,85	0,72	0,78	1,31	1,27	1,30	nodulin MtN21 family protein
257556_at	At3g28090	0,98	1,18	1,01	0,96	1,02	1,17	1,16	1,07	nodulin MtN21 family protein
257308_at	At3g28120	0,98	1,06	0,97	1,00	1,03	0,95	1,03	0,97	hypothetical protein
257272_at	At3g28130	0,78	0,96	0,93	0,82	0,76	0,95	1,19	1,07	nodulin MtN21 family protein
257270_at	At3g28140	0,92	1,09	1,04	1,30	1,05	0,97	1,03	0,98	calmodulin-binding protein
257309_at	At3g28150	0,90	0,88	0,96	0,99	1,24	1,02	1,08	0,96	expressed protein
257298_at	At3g28155	1,05	1,11	1,14	0,96	1,01	1,04	1,27	1,13	hypothetical protein
257068_at	At3g28160	0,96	1,03	1,10	0,94	1,05	0,98	1,04	0,89	---
257069_at	At3g28170	0,88	1,10	1,04	1,09	1,08	1,09	1,05	0,94	hypothetical protein
257071_at	At3g28180	1,29	1,13	1,31	1,07	1,04	1,03	1,22	1,31	glycosyl transferase family 2 protein
257070_at	At3g28190	1,04	1,04	0,98	0,98	0,99	0,96	1,15	1,10	hypothetical protein
256578_at	At3g28200	0,87	0,90	0,87	0,90	1,17	0,98	0,80	0,80	peroxidase, putative
256576_at	At3g28210	0,74	0,92	1,07	0,70	0,82	1,62	1,82	1,69	zinc finger (AN1-like) family protein
256577_at	At3g28220	1,62	1,38	1,10	0,99	1,05	1,60	1,46	1,26	meprin and TRAF homology domain-containing protein / MATH domain-cor
256630_at	At3g28230	0,99	1,00	1,00	0,95	0,94	0,96	0,96	1,11	hypothetical protein
256634_s_at	At3g28240	1,00	0,95	1,02	0,98	1,10	0,94	1,03	0,96	hypothetical protein
256635_at	At3g28260	0,90	0,95	1,10	0,96	0,98	1,01	1,00	0,83	hypothetical protein
256603_at	At3g28270	0,89	1,02	1,57	0,86	0,72	1,09	1,28	0,92	expressed protein
257576_at	At3g28280	1,14	1,02	1,18	0,97	1,10	0,95	1,12	1,05	hypothetical protein
256601_s_at	At3g28290	1,02	0,94	1,11	0,54	0,59	1,19	1,23	1,00	integrin-related protein 14a
256602_at	At3g28310	1,07	1,24	2,08	0,87	1,02	0,78	0,82	1,01	expressed protein
256631_at	At3g28320	1,15	1,30	2,18	0,97	0,95	0,96	1,03	1,12	hypothetical protein
256632_at	At3g28330	0,87	0,84	1,10	1,04	1,27	1,04	1,16	0,86	F-box family protein-related
256633_at	At3g28340	1,09	0,74	0,69	0,92	0,99	1,17	1,07	1,01	galactinol synthase, putative
257899_at	At3g28345	0,83	0,94	0,75	0,91	0,80	0,90	0,93	1,16	ABC transporter family protein
257871_at	At3g28350	0,93	0,98	1,02	0,91	0,93	1,06	1,14	0,94	hypothetical protein
257872_at	At3g28360	0,98	0,93	0,96	0,98	1,04	0,92	0,91	1,16	ABC transporter family protein
257873_at	At3g28380	1,01	0,98	1,01	1,08	0,93	0,96	1,05	1,04	P-glycoprotein, putative
257842_at	At3g28390	0,99	1,00	1,02	1,12	1,17	1,01	0,96	0,77	P-glycoprotein, putative
257843_at	At3g28400	0,92	0,96	0,98	0,96	0,93	1,01	0,96	1,02	---
257546_s_at	At3g28410	1,06	1,03	1,02	0,98	1,09	0,99	0,94	1,07	F-box family protein
257900_at	At3g28420	1,11	1,20	1,04	1,06	0,89	1,04	1,01	0,90	expressed protein
257845_at	At3g28430	1,09	1,09	1,19	1,09	1,15	1,11	0,95	1,08	expressed protein
257901_at	At3g28440	0,92	0,94	1,11	0,90	1,15	0,98	1,12	1,06	hypothetical protein
257902_at	At3g28450	0,73	0,68	0,75	1,01	1,02	0,99	0,86	0,92	leucine-rich repeat transmembrane protein kinase, putative
257903_at	At3g28460	0,84	0,84	0,99	1,04	1,02	0,91	0,97	1,01	expressed protein

257904_at	At3g28470	0,97	1,07	1,03	0,99	1,13	0,96	0,94	0,94	myb family transcription factor (MYB35)
257844_at	At3g28480	1,00	0,88	0,84	1,09	1,09	1,11	0,84	0,80	oxidoreductase, 2OG-Fe(II) oxygenase family protein
257870_at	At3g28490	0,95	1,01	0,98	0,95	1,07	1,07	0,99	1,00	oxidoreductase, 2OG-Fe(II) oxygenase family protein
256597_at	At3g28500	1,76	1,88	2,10	1,07	1,51	0,98	0,97	0,80	60S acidic ribosomal protein P2 (RPP2C)
256593_at	At3g28510	1,10	1,00	1,03	1,06	0,96	1,12	0,99	0,87	AAA-type ATPase family protein
256594_at	At3g28520	0,92	0,98	0,81	1,02	0,96	1,12	1,06	0,98	AAA-type ATPase family protein
256595_x_at	At3g28530	0,72	0,52	0,54	1,07	0,85	0,90	0,94	0,71	gypsy-like retrotransposon family (Athila)
256596_at	At3g28540	0,99	0,84	1,00	1,75	1,40	1,47	1,17	1,21	AAA-type ATPase family protein
257041_at	At3g28550	0,95	0,99	0,75	1,29	1,24	1,19	1,11	1,01	proline-rich extensin-like family protein
256987_at	At3g28560	0,90	0,92	1,13	0,99	1,03	1,00	0,96	1,14	hypothetical protein
256989_at	At3g28580	1,36	1,27	1,06	1,04	1,14	1,20	0,97	1,26	AAA-type ATPase family protein
256990_at	At3g28590	0,95	0,97	1,01	0,84	1,00	0,91	1,03	1,10	hypothetical protein
256991_at	At3g28600	1,10	1,57	1,43	1,71	1,92	1,47	1,19	1,16	AAA-type ATPase family protein
257564_at	At3g28610	1,07	1,03	1,02	1,09	1,12	1,05	1,09	1,09	AAA-type ATPase family protein
257565_at	At3g28620	1,29	1,06	0,98	1,08	1,01	0,95	1,12	1,13	zinc finger (C3HC4-type RING finger) family protein
256992_at	At3g28630	0,98	0,98	1,10	1,06	1,04	1,06	1,11	0,73	expressed protein
257042_s_at	At3g28640	0,95	0,98	1,12	0,75	0,97	0,97	1,16	1,27	pentatricopeptide (PPR) repeat-containing protein
256993_at	At3g28670	1,01	0,96	0,90	0,93	1,02	1,02	0,96	1,00	expressed protein
257015_at	At3g28680	0,95	1,02	0,96	0,97	1,12	1,04	0,92	0,93	prolylcarboxypeptidase-related
257016_at	At3g28690	0,85	0,99	1,00	0,78	0,74	1,04	1,01	0,88	protein kinase, putative
256986_at	At3g28700	0,83	0,76	0,82	1,43	1,41	0,75	0,71	0,70	expressed protein
256988_s_at	At3g28715	1,10	0,97	0,91	0,94	0,99	1,00	0,96	0,97	H+-transporting two-sector ATPase, putative
256579_at	At3g28720	0,86	0,75	0,77	1,27	1,18	1,04	0,83	0,86	expressed protein
256590_at	At3g28730	1,20	1,23	1,59	0,91	1,06	1,04	1,15	1,25	structure-specific recognition protein 1 / high mobility group protein / HMG p
256589_at	At3g28740	0,66	1,01	0,72	0,67	0,46	0,77	1,16	0,87	cytochrome P450 family protein
256584_at	At3g28750	1,04	0,88	0,83	0,94	1,07	1,05	1,14	1,15	expressed protein
256585_at	At3g28760	0,80	0,86	0,77	0,80	0,60	1,10	1,06	1,09	expressed protein
256586_at	At3g28770	1,04	0,96	0,94	1,10	1,03	1,03	0,99	1,10	expressed protein
256587_at	At3g28780	1,03	0,97	0,96	1,03	1,02	1,08	0,91	1,02	glycine-rich protein
256588_at	At3g28790	1,07	1,04	0,94	1,03	1,19	0,94	1,03	1,00	expressed protein
256580_s_at	At3g28810	1,00	1,09	0,95	0,94	1,06	0,94	0,95	1,02	hypothetical protein
256581_at	At3g28830	1,00	1,04	0,94	0,86	1,09	0,91	1,04	0,95	expressed protein
256582_at	At3g28840	1,01	1,08	0,98	1,07	1,00	1,01	0,98	0,87	expressed protein
256583_at	At3g28850	0,57	0,87	0,76	0,83	0,84	1,08	1,18	1,13	glutaredoxin family protein
257137_at	At3g28860	1,01	1,01	1,13	0,68	0,72	0,90	0,84	0,97	multidrug resistance P-glycoprotein, putative
257138_at	At3g28870	1,01	1,03	1,05	0,93	1,02	1,04	1,02	0,93	hypothetical protein
257139_at	At3g28890	1,06	1,08	1,10	1,00	1,06	0,94	1,11	1,13	leucine-rich repeat family protein
257141_at	At3g28900	1,22	0,93	0,95	1,31	1,27	0,93	0,95	0,99	60S ribosomal protein L34 (RPL34C)
257140_at	At3g28910	0,75	1,05	0,79	0,72	0,82	0,93	1,26	0,90	myb family transcription factor (MYB30)
256622_at	At3g28920	0,68	0,63	0,83	0,63	0,78	0,77	0,86	0,89	zinc finger homeobox family protein / ZF-HD homeobox family protein
258002_at	At3g28930	0,97	0,90	0,90	0,91	1,11	0,87	0,72	0,84	avrRpt2-induced AIG2 protein (AIG2)
258000_at	At3g28940	0,84	0,85	0,94	0,88	1,00	0,87	0,91	0,92	avirulence-responsive protein, putative / avirulence induced gene (AIG) pro
258001_at	At3g28950	0,76	0,75	0,67	1,15	1,18	0,89	0,86	0,72	avirulence-responsive protein-related / avirulence induced gene (AIG) prote
257543_at	At3g28960	0,76	0,98	1,00	0,94	0,92	1,06	0,95	0,82	amino acid transporter family protein
258057_at	At3g28970	0,86	0,82	1,15	0,85	1,04	1,14	1,08	1,59	expressed protein
258058_at	At3g28980	0,97	1,00	1,14	1,07	1,05	0,94	0,92	1,14	expressed protein

258056_at	At3g29010	1,32	1,33	1,36	0,90	1,25	1,13	1,23	1,07	expressed protein
258004_at	At3g29020	0,98	1,00	1,05	1,06	1,01	0,96	1,05	0,96	myb family transcription factor (MYB110)
258003_at	At3g29030	1,05	0,97	1,00	0,93	1,21	0,84	0,83	0,86	expansin, putative (EXP5)
258059_at	At3g29035	1,13	1,09	1,10	0,93	0,99	1,67	1,36	1,05	no apical meristem (NAM) family protein
257106_at	At3g29060	1,08	1,07	1,02	0,99	1,00	0,96	1,05	0,74	EXS family protein / ERD1/XPR1/SYG1 family protein
257107_at	At3g29070	1,02	1,01	1,07	1,12	0,95	0,87	0,99	1,05	emp24/gp25L/p24 family protein
258245_at	At3g29075	0,96	0,78	0,77	1,46	1,43	1,06	1,09	1,33	glycine-rich protein
258190_at	At3g29080	1,03	1,02	1,08	0,91	1,07	0,92	1,08	0,97	hypothetical protein
258193_at	At3g29090	0,96	1,18	1,21	0,87	0,73	0,96	1,04	0,97	pectinesterase family protein
258191_at	At3g29100	1,33	1,47	1,61	0,94	0,85	0,88	1,09	0,85	vesicle transport v-SNARE 13 (VTI13) / vesicle soluble NSF attachment prc
258192_at	At3g29110	0,99	1,00	0,91	0,95	1,01	0,99	0,97	1,18	terpene synthase/cyclase family protein
258246_s_at	At3g29120	0,97	1,02	1,02	0,95	1,01	1,11	0,93	0,95	---
258247_at	At3g29130	1,15	1,14	1,00	1,13	1,21	1,18	1,06	1,05	expressed protein
258248_at	At3g29140	0,90	0,94	1,04	0,85	1,16	1,02	1,00	1,08	hypothetical protein
257539_at	At3g29150	0,98	0,99	1,07	0,99	1,08	1,04	0,96	1,03	expressed protein
258221_at	At3g29160	1,29	1,27	1,41	1,23	1,27	1,09	1,08	0,97	Snf1-related protein kinase (KIN11)
258194_at	At3g29170	0,86	0,68	0,92	1,08	0,83	0,97	1,01	0,94	expressed protein
256747_at	At3g29180	0,85	0,85	0,83	0,69	0,82	1,02	0,91	1,03	expressed protein
257773_at	At3g29185	1,15	1,09	1,09	0,71	0,61	1,06	1,05	1,00	expressed protein
257776_at	At3g29190	0,95	1,16	0,99	1,18	1,20	1,00	1,00	0,97	terpene synthase/cyclase family protein
257746_at	At3g29200	1,11	1,36	1,57	0,96	0,76	1,33	1,49	1,25	chorismate mutase, chloroplast (CM1)
257777_x_at	At3g29210	0,99	0,96	1,04	0,81	1,15	1,11	1,12	0,95	hypothetical protein
257778_at	At3g29220	1,09	1,01	1,03	1,08	0,91	1,03	1,11	0,97	hypothetical protein
257744_at	At3g29230	0,83	0,67	0,82	0,75	0,69	1,02	0,95	0,74	pentatricopeptide (PPR) repeat-containing protein
257745_at	At3g29240	1,26	1,59	2,18	0,96	1,03	1,18	1,13	1,38	expressed protein
257774_at	At3g29250	0,95	0,86	0,65	2,18	1,53	1,06	1,15	1,25	short-chain dehydrogenase/reductase (SDR) family protein
257775_at	At3g29260	0,89	0,88	0,93	1,04	1,32	0,96	1,03	0,95	short-chain dehydrogenase/reductase (SDR) family protein
257575_at	At3g29265	0,99	1,01	1,14	1,02	0,93	1,04	1,00	0,88	hypothetical protein
256611_at	At3g29270	0,86	0,95	0,97	0,84	0,87	0,95	0,93	1,16	expressed protein
256612_at	At3g29280	0,96	0,94	0,92	0,78	0,93	0,95	1,02	0,95	expressed protein
256613_at	At3g29290	0,92	0,89	0,91	0,80	0,86	1,12	0,92	0,90	pentatricopeptide (PPR) repeat-containing protein
256614_at	At3g29300	0,97	1,03	1,03	1,10	1,03	1,07	0,88	0,97	hypothetical protein
256764_at	At3g29310	0,90	0,92	1,00	0,76	0,93	0,87	0,95	0,85	calmodulin-binding protein-related
256746_at	At3g29320	0,62	0,58	0,56	0,69	0,68	0,91	0,91	0,97	glucan phosphorylase, putative
256740_at	At3g29330	0,92	0,87	0,85	1,13	1,00	0,94	0,95	0,99	expressed protein
256744_at	At3g29350	0,88	0,90	0,79	1,17	0,86	1,05	1,09	1,02	two-component phosphorelay mediator 1 (HP1)
256745_at	At3g29360	0,92	0,84	0,90	1,17	1,31	1,06	0,93	0,91	UDP-glucose 6-dehydrogenase, putative
256743_at	At3g29370	0,88	1,00	1,09	0,78	0,87	0,97	0,91	0,94	expressed protein
256741_at	At3g29375	1,11	1,25	1,12	0,57	0,45	1,03	0,99	0,76	XH domain-containing protein
256742_at	At3g29380	1,04	0,96	1,06	1,03	1,01	0,97	0,90	0,92	transcription factor IIB (TFIIB) family protein
256734_at	At3g29390	1,21	1,12	1,22	0,63	0,73	1,09	1,36	1,44	hydroxyproline-rich glycoprotein family protein
256735_at	At3g29400	0,63	0,64	0,69	0,78	0,88	0,84	0,68	0,88	exocyst subunit EXO70 family protein
256736_at	At3g29410	1,37	1,43	1,27	1,20	1,06	1,02	0,93	1,01	terpene synthase/cyclase family protein
256737_at	At3g29420	0,90	1,01	1,02	1,10	0,98	1,00	1,10	0,97	geranylgeranyl pyrophosphate synthase-related / GGPP synthetase-related
256738_at	At3g29430	0,95	1,01	0,97	0,89	0,96	1,02	1,02	1,09	geranylgeranyl pyrophosphate synthase, putative / GGPP synthetase, putat
256739_s_at	At3g29460	1,03	0,91	0,94	0,88	1,13	0,97	0,97	1,11	---

255972_at	At3g29500	1,02	1,04	1,08	1,04	0,88	1,04	1,00	0,88	copia-like retrotransposon family
255971_at	At3g29510	0,97	1,01	0,95	0,98	0,90	0,98	0,90	1,02	---
255722_at	At3g29570	1,01	1,03	1,02	0,99	1,15	0,99	1,10	1,24	hypothetical protein
255723_at	At3g29575	0,93	1,02	0,88	0,72	0,79	1,06	0,88	0,96	expressed protein
256924_at	At3g29590	0,95	0,94	1,05	0,93	0,95	1,10	1,04	0,88	transferase family protein
256925_at	At3g29600	1,01	1,05	0,98	0,94	1,08	0,99	0,94	0,97	hypothetical protein
257566_x_at	At3g29610	0,91	0,99	0,86	1,01	0,96	1,01	0,97	0,99	hypothetical protein
256953_at	At3g29630	1,06	1,03	0,95	1,01	1,13	0,94	1,05	1,01	glycosyltransferase family protein
256923_at	At3g29635	1,06	1,07	0,94	1,00	1,12	1,04	0,84	0,83	transferase family protein
256604_at	At3g29640	1,01	0,97	1,04	1,05	1,07	1,02	0,92	1,07	hypothetical protein
257284_at	At3g29650	0,91	1,03	0,97	1,01	1,05	1,03	1,03	0,94	---
257287_at	At3g29660	0,94	0,97	0,99	0,98	1,05	1,07	1,01	0,96	hypothetical protein
257288_at	At3g29670	0,94	1,03	0,93	0,93	1,08	0,93	1,06	1,01	transferase family protein
257289_at	At3g29690	0,98	1,00	1,02	0,94	0,96	1,04	1,07	0,96	transferase-related
257281_s_at	At3g29720	1,03	1,11	0,98	0,88	1,10	1,07	0,88	0,81	transferase family protein
257282_at	At3g29732	1,03	0,96	0,97	0,96	1,01	0,97	0,95	1,00	---
257283_at	At3g29750	0,99	0,96	0,99	0,99	1,05	0,89	1,07	0,87	hypothetical protein
257285_at	At3g29760	1,02	1,10	1,02	0,73	0,68	1,08	1,29	1,83	NLI interacting factor (NIF) family protein
257286_s_at	At3g29763	0,94	1,07	1,04	0,95	0,87	1,09	0,99	0,92	hAT dimerisation domain-containing protein
256564_at	At3g29770	0,88	0,81	0,96	1,13	0,76	0,81	0,81	0,76	hydrolase, alpha/beta fold family protein
256562_at	At3g29775	1,04	1,05	0,93	0,94	0,96	1,01	1,05	0,90	---
256563_at	At3g29780	0,91	0,91	0,97	1,06	1,07	0,93	1,10	1,00	expressed protein
245222_at	At3g29787	0,94	0,95	0,99	0,90	1,16	0,94	1,03	1,16	---
245223_at	At3g29791	0,88	0,96	1,06	0,96	1,07	1,04	0,84	0,86	expressed protein
257347_at	At3g29792	1,03	1,07	0,93	1,08	1,08	1,05	0,94	1,00	---
245224_at	At3g29796	1,01	1,09	1,01	0,98	1,00	0,94	1,09	1,31	hypothetical protein
245225_at	At3g29800	0,95	1,19	1,03	1,01	1,20	1,02	0,96	0,97	AAA-type ATPase family
245228_at	At3g29810	1,09	0,83	0,84	1,58	1,66	1,02	0,94	0,82	phytochelatin synthetase family protein / COBRA cell expansion protein CO
245226_at	At3g29970	0,45	0,58	0,86	1,19	2,56	1,95	1,66	1,29	germination protein-related
245227_s_at	At3g30140	1,19	1,16	1,16	0,87	1,37	0,91	0,77	0,81	GTP-binding family protein
257577_at	At3g30150	0,93	1,08	1,03	0,94	1,01	1,01	1,02	0,97	hypothetical protein
257578_x_at	At3g30160	1,05	1,05	0,90	1,75	1,17	0,94	0,97	1,02	hypothetical protein
256598_at	At3g30180	1,77	1,08	1,02	0,96	0,92	1,03	1,05	1,11	cytochrome P450, putative
257301_at	At3g30190	1,00	0,99	1,04	1,00	1,07	1,04	1,04	1,02	hypothetical protein
257306_at	At3g30200	1,01	0,93	1,02	1,07	0,99	1,03	1,06	1,00	fructose-6-phosphate 2-kinase-related / fructose-2,6-bisphosphatase-relate
257307_at	At3g30210	0,95	0,94	0,99	1,03	1,24	1,01	1,06	1,19	myb family transcription factor (MYB121)
257302_at	At3g30220	1,04	0,98	0,90	0,99	0,97	1,09	0,93	0,98	hypothetical protein
257303_at	At3g30230	0,94	1,03	1,01	0,90	1,17	0,85	0,87	0,73	myosin heavy chain-related
257304_at	At3g30240	1,05	0,89	1,08	0,93	1,01	0,88	1,00	1,08	hypothetical protein
257305_at	At3g30250	1,04	0,92	1,09	1,19	1,06	1,13	1,00	0,99	hypothetical protein
256730_at	At3g30260	0,91	0,85	0,92	1,00	1,10	1,06	0,95	0,89	---
256703_at	At3g30270	1,01	1,02	0,91	0,99	1,02	0,90	1,00	1,11	MADS-box protein (AGL79)
256704_at	At3g30280	0,95	1,08	0,98	0,85	1,03	1,07	1,01	1,11	transferase family protein
256705_at	At3g30290	1,05	0,99	0,86	0,86	1,03	0,88	1,08	0,91	cytochrome P450 family protein
256706_at	At3g30300	0,83	0,99	1,25	0,83	0,86	0,97	1,17	1,22	expressed protein
256707_at	At3g30310	1,14	0,93	1,09	1,02	1,05	0,96	1,12	0,95	hypothetical protein

256708_at	At3g30320	0,96	1,02	1,01	1,03	0,98	1,01	1,02	0,91	hypothetical protein
256709_at	At3g30330	1,00	0,99	1,17	1,03	0,97	1,06	1,11	0,88	---
256731_at	At3g30340	1,08	1,04	0,90	1,01	0,97	0,96	0,98	1,02	nodulin MtN21 family protein
256710_at	At3g30350	0,94	1,06	1,05	0,93	1,22	1,06	1,11	0,98	expressed protein
256732_at	At3g30360	0,96	0,93	0,94	0,94	1,00	1,06	1,02	0,94	hypothetical protein
256733_at	At3g30370	0,91	0,90	1,07	1,07	0,91	1,05	1,08	1,11	hypothetical protein
256702_at	At3g30380	0,91	0,76	0,94	0,89	0,91	1,13	1,16	1,05	expressed protein
256711_at	At3g30390	0,83	0,82	0,83	1,00	0,96	1,03	0,92	0,98	amino acid transporter family protein
256748_x_at	At3g30396	0,84	0,98	0,94	0,89	0,92	0,98	1,10	0,92	---
256749_at	At3g30400	1,00	1,04	1,07	0,93	0,97	1,11	0,94	1,25	---
257110_x_at	At3g30440	1,12	1,04	1,09	0,91	1,01	0,95	0,99	0,90	pseudogene, Ulp1 protease family
257111_x_at	At3g30450	0,96	0,98	0,99	1,11	1,00	1,01	1,06	1,10	hypothetical protein
257081_at	At3g30460	1,03	0,92	1,06	0,98	1,05	0,97	1,21	0,86	zinc finger (C3HC4-type RING finger) family protein
257562_at	At3g30480	1,02	1,07	1,09	0,94	1,00	0,95	1,06	0,95	hypothetical protein
257108_x_at	At3g30490	1,04	0,93	1,08	1,00	1,01	0,98	0,93	1,01	hypothetical protein
257109_s_at	At3g30500	0,97	0,97	1,02	1,13	0,97	1,01	0,92	0,95	pseudogene, hypothetical protein
258301_at	At3g30510	0,96	1,04	0,93	1,06	0,97	1,06	1,01	0,94	hypothetical protein
258306_at	At3g30530	1,01	1,06	0,96	0,92	1,10	1,06	1,06	1,14	bZIP transcription factor family protein
258763_s_at	At3g30540	0,99	1,02	0,98	0,98	1,09	0,86	1,11	0,93	(1-4)-beta-mannan endohydrolase, putative
258307_x_at	At3g30560	0,97	1,00	0,93	0,94	1,01	1,14	1,00	0,95	hypothetical protein
258308_at	At3g30570	0,82	1,06	1,05	0,92	1,02	1,01	0,97	0,86	---
258309_at	At3g30585	0,97	0,92	0,95	0,99	1,04	1,09	1,04	1,14	---
258302_at	At3g30610	0,85	0,94	1,01	0,96	1,34	1,07	1,08	0,93	hypothetical protein
258303_at	At3g30620	0,96	1,01	0,97	0,98	0,99	1,00	0,95	1,09	---
258304_at	At3g30640	1,03	1,01	1,02	0,97	1,00	0,93	1,04	1,03	Ulp1 protease family protein
258305_at	At3g30650	1,01	0,98	1,20	0,96	1,02	1,04	0,92	1,00	expressed protein
256893_x_at	At3g30660	0,96	0,97	0,95	0,98	1,19	1,06	1,01	1,03	hypothetical protein
257898_s_at	At3g30670	1,00	1,03	1,05	0,98	1,00	0,93	1,08	0,99	CACTA-like transposase family (Ptta/En/Spm)
256941_at	At3g30710	1,08	0,83	0,91	1,07	1,02	0,96	0,94	0,96	hypothetical protein
256940_at	At3g30720	1,03	1,05	0,94	0,70	0,78	0,65	0,50	0,72	expressed protein
256571_at	At3g30730	0,95	0,98	1,09	0,92	1,02	1,07	1,04	0,97	hypothetical protein
256572_at	At3g30740	0,98	1,04	1,11	1,08	1,09	0,98	0,97	0,87	---
257316_at	At3g30750	0,97	1,05	0,96	1,02	0,93	1,00	0,93	0,99	hypothetical protein
257315_at	At3g30775	1,59	1,55	1,47	0,92	1,10	1,11	1,26	1,37	proline oxidase, mitochondrial / osmotic stress-responsive proline dehydrog
256901_at	At3g30800	1,04	0,99	1,07	0,93	1,04	1,11	1,08	0,96	---
257098_at	At3g30810	0,90	0,98	0,95	1,02	1,07	1,07	1,03	0,92	hypothetical protein
257096_at	At3g30820	1,04	0,93	0,96	0,99	0,95	1,07	1,04	0,94	hypothetical protein
257097_at	At3g30830	1,02	0,93	1,00	1,00	0,97	1,05	0,95	0,94	---
256608_at	At3g30840	1,07	1,05	1,00	1,09	1,08	1,09	1,11	1,15	hypothetical protein
256609_at	At3g30843	0,95	1,05	0,97	0,94	1,09	1,10	1,10	0,94	hypothetical protein
256610_at	At3g30845	0,98	1,06	1,08	0,94	0,99	1,04	1,02	1,04	hypothetical protein
257346_at	At3g30846	0,96	0,97	1,01	0,93	0,98	1,05	1,13	1,16	---
255974_at	At3g31023	0,89	1,01	0,92	0,93	1,02	1,13	1,03	0,99	---
256558_at	At3g31300	0,94	0,98	0,93	1,05	0,96	1,08	1,03	0,97	hypothetical protein
256549_at	At3g31310	0,94	0,94	1,13	1,01	0,99	1,06	1,12	0,93	hypothetical protein
256550_at	At3g31314	0,96	1,02	0,95	0,88	0,97	0,95	1,03	0,93	CACTA-like transposase family (Ptta/En/Spm)

256551_at	At3g31320	1,00	1,02	0,98	0,97	1,12	0,99	1,01	0,98 hypothetical protein
256561_s_at	At3g31330	0,99	1,01	1,02	0,95	1,04	1,12	1,06	0,98 hypothetical protein
256559_at	At3g31340	1,01	0,96	1,00	0,93	1,06	0,89	0,99	1,02 ---
256552_at	At3g31350	0,96	0,93	1,16	0,98	1,14	0,89	1,06	0,96 hypothetical protein
256553_at	At3g31360	1,01	0,97	0,90	0,99	1,12	1,30	0,99	1,08 ---
256554_at	At3g31380	1,01	1,00	0,90	0,97	1,02	1,01	1,12	0,89 ---
256555_at	At3g31390	1,02	1,06	1,04	0,92	1,10	0,97	1,14	1,04 ---
256556_at	At3g31403	0,94	0,91	1,08	1,09	0,95	0,93	1,04	1,09 ---
256557_at	At3g31410	0,94	0,95	0,90	1,01	1,07	1,00	0,89	1,07 hypothetical protein
256560_s_at	At3g31415	0,91	1,08	0,93	0,92	1,09	0,92	1,05	0,87 terpene synthase/cyclase family protein
256003_at	At3g31430	0,87	1,06	0,94	0,89	1,14	1,03	0,96	1,01 hypothetical protein
256004_at	At3g31460	0,85	0,87	1,06	1,05	1,11	0,98	1,09	1,03 ---
255970_s_at	At3g31540	0,95	0,98	0,96	0,99	0,90	0,93	0,87	0,89 hypothetical protein
263531_s_at	At3g31900	0,94	1,07	1,03	0,99	1,04	0,95	1,01	1,16 pseudogene, similar to Hypothetical protein
256591_at	At3g31910	0,96	0,96	0,91	1,02	0,95	1,11	1,00	0,92 hypothetical protein
256592_at	At3g31915	1,04	1,08	1,04	0,98	1,10	0,99	1,12	1,06 hypothetical protein
265916_at	At3g31920	0,97	1,06	0,92	1,04	1,17	0,99	0,98	0,91 ---
256842_at	At3g31930	0,98	1,00	1,02	0,98	1,08	0,91	0,98	1,04 hypothetical protein
256843_at	At3g31940	0,97	0,99	0,95	1,00	1,16	1,03	1,01	1,01 hypothetical protein
256844_s_at	At3g31955	0,99	0,91	0,96	0,94	1,12	0,95	1,06	1,09 CACTA-like transposase family (Ptta/En/Spm)
256845_x_at	At3g31970	1,03	1,10	0,95	0,94	1,25	0,80	1,11	1,34 ---
256840_x_at	At3g32000	0,96	0,99	1,02	1,06	1,08	0,95	0,95	1,00 ---
256841_x_at	At3g32020	0,92	0,94	1,02	0,91	1,05	1,02	1,04	1,27 hypothetical protein
256684_at	At3g32040	0,99	0,95	0,92	1,09	1,00	0,96	1,06	1,05 geranylgeranyl pyrophosphate synthase, putative / GGPP synthetase, putat
256692_at	At3g32050	0,93	1,04	1,11	0,95	1,08	0,94	1,09	0,93 hypothetical protein
256693_at	At3g32060	0,98	0,95	0,94	1,09	1,04	1,04	1,03	1,04 ---
256694_at	At3g32080	0,95	1,00	1,01	0,98	1,20	1,17	0,95	1,04 hypothetical protein
256695_at	At3g32090	1,00	1,08	1,02	0,81	1,20	0,89	0,97	0,86 transcription factor-related
256685_at	At3g32100	1,03	0,98	1,02	0,96	1,06	1,12	0,95	0,98 hypothetical protein
256686_at	At3g32110	0,98	1,00	1,00	1,06	0,96	1,06	1,02	1,02 ---
256687_x_at	At3g32120	0,96	1,00	0,96	0,98	1,09	1,03	0,96	1,01 hypothetical protein
256688_at	At3g32130	1,02	0,89	0,94	0,93	1,00	0,83	1,05	1,02 hypothetical protein
256691_at	At3g32140	0,98	0,94	0,97	1,01	0,87	1,01	0,99	1,08 hypothetical protein
256689_at	At3g32150	1,10	0,95	1,06	0,96	0,97	0,94	0,96	0,97 hypothetical protein
256690_at	At3g32160	0,98	1,01	0,87	0,90	1,10	0,86	0,79	1,13 hypothetical protein
256667_at	At3g32180	0,93	0,97	0,93	0,91	1,07	1,13	0,90	1,21 hypothetical protein
256668_at	At3g32190	1,02	0,93	1,06	0,89	1,08	0,92	0,92	0,97 hypothetical protein
256669_s_at	At3g32200	0,90	0,95	1,00	1,03	1,18	0,95	1,07	1,04 hypothetical protein
256670_at	At3g32220	1,01	0,92	0,99	1,01	1,10	0,89	1,03	1,00 ---
256639_at	At3g32250	0,89	1,04	0,95	0,96	1,04	1,07	1,01	1,01 ---
256640_at	At3g32260	0,98	1,03	1,04	1,10	0,95	0,98	1,06	1,07 hypothetical protein
256641_at	At3g32270	1,10	1,07	1,01	0,99	0,97	0,83	1,18	0,90 hypothetical protein
256642_at	At3g32280	1,03	0,93	0,95	0,99	1,08	1,08	1,02	1,02 hypothetical protein
256643_at	At3g32290	1,04	0,84	0,94	0,98	1,02	0,90	0,89	0,91 ---
256644_at	At3g32300	1,03	1,02	1,05	0,97	1,10	1,07	0,88	0,99 ---
255975_at	At3g32350	0,87	1,01	0,80	1,08	1,03	1,00	0,98	1,11 ---

255973_at	At3g32400	0,86	1,08	1,10	0,91	0,95	1,07	0,93	1,07	formin homology 2 domain-containing protein / FH2 domain-containing prot
256538_x_at	At3g32900	0,92	1,02	0,98	0,97	0,99	1,01	1,08	0,97	Ulp1 protease family protein
256539_at	At3g32910	0,93	1,07	1,15	1,10	0,99	0,97	0,97	0,93	hypothetical protein
256540_at	At3g32917	1,02	1,01	1,05	0,92	1,01	0,90	1,00	1,07	gypsy-like retrotransposon family
265941_s_at	At3g32920	0,97	0,73	0,84	1,00	0,82	1,02	0,89	0,77	recA family protein
256607_at	At3g32930	0,95	1,02	1,00	0,68	0,59	1,02	1,03	1,05	expressed protein
256605_at	At3g32940	0,80	0,94	0,96	0,75	1,03	0,85	0,91	0,90	expressed protein
256606_at	At3g32960	0,97	0,87	0,87	1,03	1,19	1,09	0,98	0,92	hypothetical protein
259024_at	At3g33064	1,01	0,97	0,97	0,97	0,98	0,96	0,93	0,93	hypothetical protein
257345_s_at	At3g33066	0,99	1,06	1,06	1,05	1,23	0,92	0,86	0,81	---
259025_at	At3g33073	1,03	1,08	1,00	0,93	0,84	0,89	0,99	1,02	hypothetical protein
245203_at	At3g33080	1,06	0,91	0,99	1,04	0,99	0,97	0,98	1,05	hypothetical protein
259242_at	At3g33520	0,63	0,66	0,69	0,79	0,77	0,84	0,89	0,84	actin-related protein 6 (ARP6)
259241_at	At3g33530	0,93	0,80	0,86	1,03	1,06	0,90	0,83	0,86	transducin family protein / WD-40 repeat family protein
252845_at	At3g42050	0,98	0,93	0,94	0,94	0,99	0,98	0,96	0,94	vacuolar ATP synthase subunit H family protein
252835_at	At3g42060	0,98	1,03	0,90	0,88	1,09	1,00	0,90	0,97	myosin heavy chain-related
252836_at	At3g42070	0,81	1,03	0,94	1,01	1,07	0,95	0,92	0,93	hypothetical protein
252837_at	At3g42080	1,00	0,97	0,94	0,99	1,02	1,10	1,01	0,91	hypothetical protein
252838_at	At3g42090	1,00	1,02	0,94	1,00	0,99	1,03	1,00	1,01	hypothetical protein
252839_at	At3g42100	1,03	1,03	1,12	1,08	1,10	1,18	0,96	0,99	AT hook motif-containing protein-related
252841_at	At3g42110	0,95	1,01	1,08	0,81	1,02	0,98	0,98	1,06	hypothetical protein
252842_at	At3g42120	1,01	0,98	1,03	1,06	1,01	1,10	0,96	1,02	hypothetical protein
252843_at	At3g42130	0,92	0,95	0,97	0,97	1,09	1,04	1,03	1,04	glycine-rich protein
252844_at	At3g42140	0,86	1,17	0,91	0,93	1,29	1,03	1,13	1,03	hypothetical protein
252789_s_at	At3g42150	1,12	0,97	1,05	1,23	1,56	0,89	0,85	0,97	expressed protein
252846_at	At3g42160	0,96	1,04	0,91	1,01	1,02	0,95	1,03	1,12	pectinesterase-related
252847_at	At3g42170	0,83	0,88	1,11	0,97	0,89	0,91	0,86	0,93	hAT-like transposase family (hobo/Ac/Tam3)
252848_at	At3g42190	1,05	1,11	0,94	0,99	0,92	0,94	0,92	1,11	hypothetical protein
252790_at	At3g42210	1,00	0,93	0,90	0,97	0,94	1,00	0,96	0,90	---
252791_at	At3g42220	0,97	1,05	0,97	0,93	1,03	1,01	1,00	0,94	---
252792_at	At3g42240	1,01	1,09	0,96	1,01	1,10	1,01	1,02	1,09	hypothetical protein
252793_at	At3g42250	0,97	1,01	0,99	0,96	1,04	1,08	1,02	0,94	hypothetical protein
252794_at	At3g42260	0,97	0,87	0,94	0,90	1,06	0,92	0,95	1,07	---
252795_at	At3g42270	0,97	1,09	0,96	0,92	1,17	1,01	0,98	1,02	---
252796_at	At3g42290	0,98	1,00	1,03	1,02	0,98	1,11	0,96	0,96	---
252797_at	At3g42300	0,84	1,01	0,93	0,93	1,35	0,92	1,01	0,81	hypothetical protein
252798_at	At3g42310	1,04	0,89	0,97	1,08	1,05	1,00	0,96	0,98	hypothetical protein
252799_at	At3g42320	0,99	0,94	1,04	0,89	1,09	0,97	0,89	1,10	hypothetical protein
252800_at	At3g42330	1,00	1,04	0,87	0,97	1,00	1,12	0,96	1,07	hypothetical protein
252801_at	At3g42340	1,04	0,92	1,09	1,02	1,03	0,95	0,91	1,03	hypothetical protein
252802_at	At3g42350	1,05	0,99	0,94	0,94	1,07	1,04	1,15	1,01	hypothetical protein
252803_at	At3g42360	0,97	1,03	0,88	0,99	0,95	1,02	0,91	1,16	---
252804_at	At3g42370	0,97	0,99	1,01	0,93	0,97	0,92	1,22	0,88	---
252805_at	At3g42380	0,94	0,95	1,02	0,93	1,03	0,95	1,08	1,08	hypothetical protein
252806_at	At3g42390	1,04	0,98	1,07	0,93	0,99	1,05	1,02	1,13	hypothetical protein
252807_at	At3g42400	0,98	0,95	0,94	0,91	1,08	0,92	0,96	1,05	hypothetical protein

252808_at	At3g42420	1,00	0,94	0,79	0,92	1,02	1,06	1,14	1,00	---
252809_at	At3g42430	1,00	1,02	0,98	0,92	1,03	0,99	1,01	0,94	hypothetical protein
252810_at	At3g42440	1,00	0,95	1,17	1,01	1,08	1,02	0,97	0,77	hypothetical protein
252811_at	At3g42460	0,90	0,88	1,06	0,99	1,06	1,10	0,95	0,99	hypothetical protein
252812_at	At3g42480	1,11	0,95	0,97	0,98	0,89	1,07	1,01	0,84	hypothetical protein
252813_at	At3g42500	0,91	1,00	1,09	1,10	0,96	1,26	1,00	0,85	hypothetical protein
252814_at	At3g42530	1,11	0,91	1,00	0,97	1,06	1,01	1,03	1,02	Ulp1 protease family
252815_at	At3g42540	0,94	1,05	1,04	1,00	0,94	0,98	0,99	1,15	hypothetical protein
252816_at	At3g42550	0,89	0,97	0,98	0,95	1,10	1,09	1,04	1,10	aspartyl protease family protein
257890_s_at	At3g42570	0,34	0,40	0,85	0,64	1,02	0,41	0,53	0,84	peroxidase, putative
252817_at	At3g42580	0,99	1,00	1,04	1,00	1,13	1,08	1,01	0,99	Ulp1 protease family protein
252818_at	At3g42590	0,91	1,05	0,99	0,87	1,07	0,99	1,07	1,12	hypothetical protein
252819_at	At3g42600	1,09	0,94	1,04	0,99	1,07	1,04	0,96	0,98	hypothetical protein
252759_at	At3g42630	1,04	0,99	1,21	0,97	1,17	0,78	1,07	0,91	pentatricopeptide (PPR) repeat-containing protein
252820_at	At3g42640	1,07	0,93	1,47	0,92	0,89	1,19	1,16	1,27	ATPase, plasma membrane-type, putative / proton pump, putative
252785_at	At3g42660	1,44	1,51	1,79	1,25	1,04	1,04	0,86	0,73	transducin family protein / WD-40 repeat family protein
252786_at	At3g42670	1,26	1,28	1,09	0,94	0,92	0,93	0,90	1,19	SNF2 domain-containing protein / helicase domain-containing protein
252787_at	At3g42680	1,02	0,90	0,98	0,98	1,01	0,93	0,90	1,01	hypothetical protein
252788_at	At3g42690	0,99	1,01	1,29	0,92	0,96	1,14	1,06	0,88	Ulp1 protease family protein
252757_at	At3g42700	1,10	1,00	0,92	0,92	1,03	1,12	1,15	0,92	hypothetical protein
252758_at	At3g42710	0,95	0,94	0,97	1,21	1,09	0,98	1,10	1,11	---
252763_at	At3g42725	1,42	1,39	0,93	1,23	1,00	0,97	1,04	0,96	expressed protein
252760_x_at	At3g42730	0,99	1,02	0,90	0,83	1,06	0,91	0,89	0,99	Ulp1 protease family protein
252761_at	At3g42770	1,09	1,14	0,92	0,90	1,00	1,13	0,97	0,96	F-box family protein
252762_at	At3g42780	0,95	0,99	0,92	0,88	1,15	0,88	1,01	1,41	hypothetical protein
252764_at	At3g42790	1,05	1,07	1,10	0,99	1,02	1,00	1,05	1,18	PHD finger family protein
252765_at	At3g42800	1,06	0,98	1,03	1,14	0,99	0,86	1,02	1,06	expressed protein
252766_at	At3g42820	0,96	1,10	1,12	1,00	1,00	1,10	1,02	0,96	hypothetical protein
252767_at	At3g42830	1,07	0,94	1,12	1,10	1,09	0,98	0,99	0,92	ring-box protein Roc1/Rbx1/Hrt1, putative
252768_at	At3g42840	0,94	1,05	0,92	1,01	1,08	1,08	0,94	0,96	---
252769_at	At3g42850	0,96	1,00	1,04	0,95	0,92	0,89	1,03	0,88	galactokinase, putative
252770_at	At3g42860	1,22	1,03	1,06	0,84	1,01	1,04	0,91	0,98	zinc knuckle (CCHC-type) family protein
252771_at	At3g42880	0,92	0,99	0,99	1,07	1,15	0,99	1,01	0,99	leucine-rich repeat transmembrane protein kinase, putative
252772_at	At3g42900	0,98	1,01	0,95	0,88	1,18	1,03	0,87	1,00	---
252773_at	At3g42910	0,92	0,93	0,99	0,87	0,91	0,92	1,04	0,86	expressed protein
252774_at	At3g42920	0,98	1,10	1,11	1,09	1,00	0,99	0,99	1,04	hypothetical protein
252775_s_at	At3g42930	1,05	0,98	1,00	0,97	0,99	0,98	1,04	0,98	---
252776_at	At3g42940	0,93	1,07	0,93	1,00	0,93	0,89	1,13	0,84	expressed protein
252781_at	At3g42950	0,67	0,63	0,67	1,03	1,36	0,77	0,77	0,87	glycoside hydrolase family 28 protein / polygalacturonase (pectinase) family
252780_at	At3g42960	1,09	0,99	0,96	0,89	1,14	1,01	1,03	1,00	alcohol dehydrogenase (ATA1)
252777_at	At3g42970	0,99	0,94	0,97	0,98	0,92	1,04	1,01	0,92	hypothetical protein
252778_at	At3g42980	0,99	1,01	0,88	1,00	1,00	0,91	1,00	0,94	hypothetical protein
252779_at	At3g42990	0,97	1,05	0,94	0,99	1,09	1,09	0,98	1,04	hypothetical protein
252782_at	At3g43010	1,01	0,99	0,98	1,06	1,03	1,02	0,99	0,99	pseudogene, hypothetical protein
252783_at	At3g43020	0,92	1,00	0,98	1,01	0,95	1,08	1,07	0,94	pseudogene, hypothetical protein
252784_at	At3g43040	1,06	1,02	1,03	1,00	0,95	1,02	1,00	1,04	pseudogene, hypothetical protein

252725_at	At3g43050	0,93	0,97	1,07	0,92	1,07	1,00	0,96	1,08	---
252726_at	At3g43060	0,95	1,07	0,97	1,10	0,89	1,04	1,10	1,22	---
252727_s_at	At3g43070	0,97	1,07	1,00	1,00	1,04	1,01	0,96	0,87	hypothetical protein
252728_at	At3g43080	1,01	0,98	0,94	0,95	1,12	1,03	1,00	1,16	---
252729_at	At3g43100	0,94	1,00	1,02	1,03	1,13	1,01	1,05	1,01	hypothetical protein
252730_at	At3g43110	0,93	1,00	0,84	0,97	1,02	0,96	1,02	0,90	expressed protein
252733_at	At3g43120	0,95	1,07	0,92	0,95	1,03	0,96	1,02	1,08	auxin-responsive protein-related
252731_at	At3g43130	0,99	1,07	1,03	1,10	1,17	0,97	1,02	1,07	---
252732_at	At3g43140	1,01	0,98	1,10	0,95	1,04	0,97	1,03	0,95	hypothetical protein
246341_x_at	At3g43150	0,94	0,91	0,99	1,03	0,96	0,97	0,96	0,97	hypothetical protein
252734_at	At3g43160	1,07	0,96	0,96	0,92	0,94	1,01	1,20	1,17	expressed protein
252735_at	At3g43170	0,98	1,04	0,92	1,02	0,84	1,03	1,00	0,95	hypothetical protein
252746_at	At3g43190	0,44	0,52	0,64	1,41	1,51	0,94	1,11	1,06	sucrose synthase, putative / sucrose-UDP glucosyltransferase, putative
252736_at	At3g43210	0,89	0,96	1,18	1,06	0,93	0,89	0,94	1,00	kinesin motor family protein (NACK2)
252737_at	At3g43220	1,03	1,06	1,14	0,92	1,04	1,11	1,14	1,44	phosphoinositide phosphatase family protein
252745_at	At3g43230	0,97	1,03	1,18	0,71	0,95	1,06	1,12	1,48	zinc finger (FYVE type) family protein
252738_at	At3g43240	1,01	0,89	0,99	1,03	0,89	0,98	0,93	0,93	ARID/BRIGHT DNA-binding domain-containing protein
252739_at	At3g43250	1,59	1,29	0,78	1,40	1,49	1,38	1,42	1,34	cell cycle control protein-related
252740_at	At3g43270	1,21	1,04	1,19	1,20	1,05	1,06	0,97	0,83	pectinesterase family protein
252741_at	At3g43280	0,99	1,03	1,04	1,01	0,99	1,05	0,99	0,98	hypothetical protein
252742_at	At3g43290	0,97	1,06	0,91	1,07	1,29	1,07	0,83	0,86	hypothetical protein
252743_at	At3g43300	0,99	0,86	0,95	0,96	0,91	0,91	0,95	0,95	guanine nucleotide exchange family protein
252744_at	At3g43310	1,12	1,02	1,03	0,95	1,05	1,03	1,10	1,02	---
252747_at	At3g43320	0,94	0,97	1,11	0,89	1,16	0,88	0,91	0,76	hypothetical protein
252748_at	At3g43330	1,15	1,03	0,98	1,05	1,09	0,97	0,96	0,92	hypothetical protein
261343_s_at	At3g43350	0,98	0,98	0,93	1,02	0,95	0,99	0,97	1,09	hypothetical protein
252749_at	At3g43390	1,00	0,92	0,92	0,95	1,25	1,06	1,03	1,06	hypothetical protein
264991_s_at	At3g43400	1,33	1,21	1,14	0,68	0,96	1,19	0,94	1,02	phagocytosis and cell motility protein ELMO1-related
252750_at	At3g43420	0,94	1,06	0,97	1,03	0,95	0,99	0,97	1,03	hypothetical protein
252751_at	At3g43430	1,13	1,61	1,32	1,76	1,21	0,83	1,21	1,16	zinc finger (C3HC4-type RING finger) family protein
264414_s_at	At3g43460	0,93	1,09	0,92	0,92	1,00	0,93	0,98	1,05	pseudogene, Ulp1 protease family
252752_s_at	At3g43480	1,03	1,04	1,07	0,99	1,10	1,09	1,00	1,05	hypothetical protein
252753_at	At3g43500	0,97	1,07	0,95	0,94	1,09	1,07	0,95	0,92	hypothetical protein
252754_at	At3g43510	1,01	1,02	0,86	0,71	0,98	1,08	1,23	1,18	copia-like retrotransposon family
252723_at	At3g43520	1,17	1,09	0,92	1,21	1,16	0,87	0,88	0,86	expressed protein
252755_at	At3g43530	0,94	1,00	0,89	1,06	1,10	1,10	1,12	0,96	hypothetical protein
252724_at	At3g43540	0,95	1,02	0,79	0,94	0,89	1,21	1,24	1,20	expressed protein
252756_s_at	At3g43550	1,01	0,91	0,95	1,00	0,98	0,98	1,03	0,89	GDSL-motif lipase, putative
252722_at	At3g43560	0,91	1,00	0,99	0,99	0,92	0,95	1,10	1,05	hypothetical protein
246328_at	At3g43590	1,02	1,07	1,12	0,88	0,79	1,00	1,00	0,96	zinc knuckle (CCHC-type) family protein
246330_at	At3g43600	0,91	0,79	0,88	1,23	1,01	1,16	0,87	1,00	aldehyde oxidase, putative
246329_at	At3g43610	1,19	1,30	1,58	0,85	0,87	1,02	1,04	1,09	tubulin family protein
252694_at	At3g43630	0,81	0,90	1,00	1,06	0,96	1,01	0,96	1,18	nodulin, putative
252695_s_at	At3g43640	0,96	1,00	0,97	1,03	1,08	0,99	0,96	1,00	---
252696_at	At3g43650	1,01	1,04	0,91	0,98	1,05	0,96	1,00	1,02	---
252697_at	At3g43660	0,92	1,07	1,04	0,90	0,98	1,06	1,03	1,19	nodulin, putative

252698_at	At3g43670	0,74	0,71	0,74	0,54	0,47	0,55	0,55	0,50	copper amine oxidase, putative
252699_at	At3g43680	0,95	1,14	1,00	0,95	1,14	1,02	1,00	1,25	hypothetical protein
252700_at	At3g43690	0,98	1,17	0,90	0,97	1,02	1,08	0,94	0,78	copia-like retrotransposon family protein
252701_at	At3g43700	1,07	1,33	1,17	1,01	0,88	1,10	1,02	1,14	speckle-type POZ protein-related
252711_at	At3g43720	1,18	0,90	0,87	1,04	0,86	0,85	0,78	0,85	protease inhibitor/seed storage/lipid transfer protein (LTP) family protein
252702_at	At3g43730	1,01	1,00	0,91	1,10	1,05	1,04	0,98	1,02	---
252703_at	At3g43740	0,98	0,91	0,82	1,03	0,97	0,95	0,99	0,85	leucine-rich repeat family protein
252704_at	At3g43750	0,96	1,04	1,06	1,03	1,01	1,05	0,97	0,93	zinc finger (C3HC4-type RING finger) family protein
252705_at	At3g43760	0,93	0,97	1,10	1,08	1,11	0,99	0,90	0,96	hypothetical protein
252706_at	At3g43770	1,03	0,99	0,92	0,96	1,05	1,06	1,00	0,93	hypothetical protein
252707_at	At3g43790	0,72	0,68	0,64	1,07	1,09	0,89	1,02	0,99	transporter-related
252712_at	At3g43800	1,20	1,06	0,85	0,91	0,86	1,16	1,19	1,21	glutathione S-transferase, putative
252713_at	At3g43810	1,05	0,91	0,89	1,36	1,20	0,93	0,93	0,92	calmodulin-7 (CAM7)
252708_at	At3g43820	1,00	0,92	1,03	0,99	0,96	0,96	0,99	1,00	---
252709_at	At3g43840	0,95	0,97	0,89	1,02	0,92	1,01	1,04	1,10	expressed protein
252710_at	At3g43860	0,97	0,98	1,09	0,87	1,07	1,11	1,04	1,02	glycosyl hydrolase family 9 protein
252714_x_at	At3g43880	1,03	1,03	0,93	1,01	1,06	1,09	1,00	0,84	hypothetical protein
252715_x_at	At3g43900	1,01	1,02	0,91	0,97	0,91	1,00	1,06	1,16	hypothetical protein
252716_at	At3g43920	1,11	0,80	1,02	0,81	0,96	0,93	0,91	1,07	ribonuclease III family protein
252717_at	At3g43930	1,23	1,12	1,27	1,05	1,19	0,93	1,02	1,00	expressed protein
252718_at	At3g43940	0,98	1,02	1,05	1,03	1,11	1,09	1,01	1,02	hypothetical protein
252719_at	At3g43950	0,94	1,03	0,97	1,03	1,11	1,03	1,04	0,94	hypothetical protein
252692_at	At3g43960	1,28	0,81	0,98	1,06	0,97	0,63	0,74	0,79	cysteine proteinase, putative
252720_at	At3g43970	1,09	0,99	1,27	0,92	1,02	0,83	0,94	1,03	hypothetical protein
252721_at	At3g43990	0,95	0,94	0,99	1,05	1,12	0,93	1,07	1,05	bromo-adjacent homology (BAH) domain-containing protein
252687_at	At3g44000	1,05	1,04	0,97	0,89	1,07	1,06	0,96	1,04	---
252693_s_at	At3g44010	1,47	1,22	1,10	1,46	1,29	0,99	0,95	0,95	40S ribosomal protein S29 (RPS29A)
252688_at	At3g44020	1,04	0,96	1,02	1,03	0,88	1,10	1,03	1,18	thylakoid lumenal P17.1 protein
252689_at	At3g44030	0,98	0,99	0,93	0,99	0,96	1,01	0,99	0,91	pseudogene, similar to OSJNBb0043H09.1
252690_at	At3g44040	1,04	0,99	0,88	0,89	1,09	0,94	0,88	0,99	---
252691_at	At3g44050	1,33	1,09	0,94	1,17	1,12	1,00	0,95	0,88	kinesin motor protein-related
252663_at	At3g44070	0,96	1,05	1,02	1,06	0,89	1,11	0,98	0,99	expressed protein
252664_at	At3g44080	1,04	1,04	0,97	0,96	0,99	1,02	1,08	1,03	F-box family protein
252669_at	At3g44100	1,04	0,98	0,85	1,20	1,15	0,97	0,94	0,90	MD-2-related lipid recognition domain-containing protein / ML domain-conta
252670_at	At3g44110	1,22	1,12	1,29	1,13	1,16	1,09	1,08	1,11	DNAJ heat shock protein, putative (J3)
252665_at	At3g44140	0,95	1,09	1,00	1,01	1,08	1,05	1,18	1,13	hypothetical protein
252666_at	At3g44180	0,92	1,01	1,12	1,14	1,08	0,98	0,96	1,08	syntaxin-related family protein
252671_at	At3g44190	0,82	0,89	1,22	1,12	1,12	1,11	1,25	1,29	pyridine nucleotide-disulphide oxidoreductase family protein
252667_at	At3g44200	0,86	0,86	0,89	1,07	1,38	0,97	0,96	0,96	protein kinase family protein
252668_at	At3g44210	1,04	0,97	1,05	1,03	0,98	1,10	1,02	1,01	hypothetical protein
252672_at	At3g44230	0,92	1,00	1,03	0,94	1,07	0,90	0,93	0,92	hypothetical protein
252673_at	At3g44240	1,05	0,91	0,90	0,98	0,97	0,93	0,91	1,07	CCR4-NOT transcription complex protein, putative
252674_at	At3g44250	0,96	1,03	1,00	1,02	1,18	1,03	1,04	1,22	cytochrome P450 family protein
252679_at	At3g44260	0,86	1,08	1,10	0,94	0,84	0,97	1,24	1,40	CCR4-NOT transcription complex protein, putative
252675_at	At3g44270	1,04	0,98	1,08	0,96	1,27	0,86	0,85	0,90	---
252676_at	At3g44280	1,12	0,90	0,85	1,17	1,27	1,02	0,98	0,88	expressed protein

252678_s_at	At3g44300	1,12	1,01	1,21	1,10	1,24	0,95	0,93	0,91	nitrilase 2 (NIT2)
252677_at	At3g44320	0,95	0,95	0,91	1,48	1,46	1,02	1,17	0,97	nitrilase 3 (NIT3)
252680_at	At3g44330	1,02	0,87	0,79	1,15	1,21	1,03	0,87	0,85	expressed protein
252662_at	At3g44340	0,87	0,89	1,01	0,97	1,04	0,99	1,03	0,91	sec23/sec24 transport family protein
252681_at	At3g44350	0,76	0,94	0,92	1,04	1,15	1,76	0,99	0,96	no apical meristem (NAM) family protein
252682_at	At3g44370	1,09	0,97	1,15	0,97	1,15	1,07	1,06	0,90	expressed protein
252683_at	At3g44380	0,86	0,81	0,98	0,91	0,93	0,72	0,90	0,85	expressed protein
252684_at	At3g44400	0,96	0,92	0,97	1,19	0,98	1,32	1,33	1,00	disease resistance protein (TIR-NBS-LRR class), putative
252685_at	At3g44410	0,93	1,00	1,07	0,91	1,05	0,98	0,93	0,86	---
252686_at	At3g44420	0,99	0,97	1,07	1,00	0,95	1,01	1,03	1,10	---
252659_at	At3g44430	0,71	0,72	0,55	0,82	0,98	0,91	0,82	0,87	expressed protein
252660_at	At3g44440	1,00	0,93	1,17	0,87	1,08	0,97	1,12	0,96	hypothetical protein
252661_at	At3g44450	1,03	0,90	1,10	0,69	0,69	1,03	1,05	1,04	expressed protein
252645_at	At3g44460	0,96	1,05	1,08	0,98	1,05	0,99	0,98	1,01	basic leucine zipper transcription factor (BZIP67)
252631_s_at	At3g44470	0,97	0,95	1,00	0,96	1,04	1,02	0,98	0,92	Mutator-like transposase family
252632_s_at	At3g44480	2,25	1,29	1,07	0,91	0,97	1,00	1,09	0,90	disease resistance protein (TIR-NBS-LRR class), putative
252633_x_at	At3g44490	1,17	1,08	1,10	0,89	0,96	1,06	0,94	0,99	histone deacetylase-related / HD-related
252634_x_at	At3g44500	0,97	1,02	0,88	0,83	1,00	0,93	0,95	1,01	Ulp1 protease family protein
252635_at	At3g44510	1,02	1,05	0,98	1,12	1,08	1,24	1,08	0,97	expressed protein
252636_at	At3g44520	1,02	1,09	1,06	1,02	1,20	1,37	1,06	1,14	esterase/lipase/thioesterase family protein
252637_at	At3g44530	0,92	1,00	0,95	0,96	1,05	1,16	1,00	0,93	transducin family protein / WD-40 repeat family protein
252638_at	At3g44540	1,50	1,36	1,42	0,96	0,81	1,45	1,81	1,78	acyl CoA reductase, putative
252639_at	At3g44550	1,14	1,05	0,80	0,97	0,65	1,36	1,40	1,77	acyl CoA reductase, putative
252640_at	At3g44560	0,95	1,10	1,01	0,95	1,06	1,05	0,99	1,11	acyl CoA reductase, putative
252641_at	At3g44570	0,92	0,89	0,91	0,97	0,95	1,01	0,96	0,97	hypothetical protein
252642_at	At3g44580	1,02	0,97	1,01	0,91	0,96	0,96	1,03	1,00	hypothetical protein
252643_at	At3g44590	1,13	0,98	0,87	1,36	1,17	0,91	0,95	0,90	60S acidic ribosomal protein P2 (RPP2D)
252644_at	At3g44600	1,19	1,04	1,20	0,83	0,81	0,95	1,02	0,99	peptidyl-prolyl cis-trans isomerase cyclophilin-type family protein
252646_at	At3g44610	0,84	0,79	0,97	0,75	0,87	0,91	1,13	0,84	protein kinase family protein
252647_at	At3g44620	0,92	0,84	0,79	0,78	0,82	1,03	1,00	1,01	low molecular weight phosphotyrosine protein phosphatase family protein
252648_at	At3g44630	1,05	0,89	0,93	1,03	0,75	1,45	2,16	2,25	disease resistance protein RPP1-WsB-like (TIR-NBS-LRR class), putative
252649_at	At3g44680	1,33	0,99	1,16	0,89	1,10	1,02	0,97	1,00	histone deacetylase, putative
252650_at	At3g44690	1,14	1,01	1,03	0,99	0,92	0,97	1,03	1,04	expressed protein
252651_at	At3g44700	0,92	1,03	0,99	1,05	1,04	0,88	0,79	0,95	expressed protein
252652_at	At3g44720	1,15	1,11	0,88	1,26	1,16	1,12	1,27	1,49	prephenate dehydratase family protein
252653_at	At3g44730	1,04	1,32	1,16	0,91	1,19	0,99	1,00	0,91	kinesin motor protein-related
252624_at	At3g44735	0,95	0,72	0,65	0,80	0,89	0,85	0,78	0,61	phytosulfokines-related
252654_at	At3g44740	1,05	1,14	1,41	1,03	0,93	1,07	1,07	0,92	tRNA synthetase class II (G, H, P and S) family protein
252625_at	At3g44750	1,55	1,25	1,06	1,15	1,16	1,12	1,07	1,08	histone deacetylase, putative (HD2A)
252655_at	At3g44760	1,02	0,99	1,06	0,87	1,10	0,94	1,13	1,05	hypothetical protein
252656_at	At3g44770	0,99	1,06	0,98	1,08	1,05	0,98	1,09	1,11	hypothetical protein
252657_at	At3g44780	1,03	1,04	1,04	1,18	1,01	1,01	1,00	1,04	hypothetical protein
252658_at	At3g44800	0,89	1,11	0,99	1,06	1,13	1,00	0,99	0,95	meprin and TRAF homology domain-containing protein / MATH domain-cor
246331_at	At3g44820	0,81	0,88	0,89	0,87	0,93	0,94	0,97	1,02	phototropic-responsive NPH3 family protein
246332_at	At3g44830	0,98	1,07	1,06	0,90	1,19	0,99	0,93	1,02	lecithin:cholesterol acyltransferase family protein / LACT family protein
246333_at	At3g44840	0,95	0,89	1,01	1,04	0,96	1,04	0,95	0,94	S-adenosyl-L-methionine:carboxyl methyltransferase family protein

246334_at	At3g44850	1,25	1,01	0,94	0,99	0,97	0,92	1,11	1,16	protein kinase-related
246340_s_at	At3g44860	1,00	1,14	1,03	1,40	1,03	1,01	0,86	0,76	S-adenosyl-L-methionine:carboxyl methyltransferase family protein
246335_at	At3g44880	0,66	0,61	0,72	0,92	0,88	0,92	0,91	0,94	Rieske (2Fe-2S) domain-containing protein
246339_at	At3g44890	0,96	0,79	0,79	0,82	0,95	0,99	0,95	1,01	50S ribosomal protein L9, chloroplast (CL9)
246336_at	At3g44900	1,09	0,99	0,95	1,00	0,97	1,02	0,92	0,94	cation/hydrogen exchanger, putative (CHX4)
246337_at	At3g44910	0,90	1,08	0,96	0,98	1,07	1,05	0,96	1,07	cation/hydrogen exchanger, putative (CHX12)
246338_s_at	At3g44930	1,03	0,96	0,96	1,08	1,05	1,01	1,16	0,98	cation/hydrogen exchanger, putative (CHX11)
252626_at	At3g44940	0,96	0,95	0,90	0,98	1,16	0,88	0,87	0,77	expressed protein
252627_at	At3g44950	1,02	1,01	1,05	0,92	1,07	1,01	1,25	1,07	glycine-rich protein
252628_at	At3g44960	1,08	0,99	1,01	0,97	1,01	0,90	0,87	0,97	expressed protein
252629_at	At3g44970	1,79	2,20	2,13	1,33	0,99	1,72	3,41	4,11	cytochrome P450 family protein
252630_at	At3g44980	0,84	1,02	1,10	1,00	1,26	1,10	1,08	1,15	hypothetical protein
252607_at	At3g44990	0,69	1,07	0,63	0,83	0,79	1,16	1,31	1,06	xyloglucan:xyloglucosyl transferase, putative / xyloglucan endotransglycosy
252600_at	At3g45000	0,97	1,09	0,93	1,09	1,04	0,96	0,96	0,97	SNF7 family protein
252606_at	At3g45010	1,14	1,01	0,92	1,33	1,29	0,91	0,89	1,01	serine carboxypeptidase III, putative
252601_s_at	At3g45030	1,47	1,23	1,19	1,16	1,05	1,02	1,01	1,03	40S ribosomal protein S20 (RPS20A)
252602_at	At3g45040	0,64	0,83	1,24	0,93	0,98	0,99	1,19	1,61	phosphatidate cytidylyltransferase family protein
252603_at	At3g45050	1,04	0,89	0,84	0,95	1,08	1,05	0,96	0,88	expressed protein
252604_at	At3g45060	0,94	1,07	0,96	1,03	0,96	1,08	1,01	0,90	high-affinity nitrate transporter, putative
252605_s_at	At3g45070	0,98	0,94	0,98	1,17	0,99	1,03	1,00	1,01	sulfotransferase family protein
252608_at	At3g45090	1,02	0,90	1,13	1,10	1,25	1,17	0,91	0,92	2-phosphoglycerate kinase-related
252617_at	At3g45100	0,79	0,90	0,93	0,93	0,97	0,90	0,92	1,00	N-acetylglucosaminyl-phosphatidylinositol biosynthetic protein, putative
252609_at	At3g45110	0,95	0,94	1,01	1,01	1,03	0,94	1,02	1,11	hypothetical protein
252610_x_at	At3g45120	0,87	1,14	1,06	1,06	1,04	1,12	0,95	1,16	hypothetical protein
252611_at	At3g45130	1,11	1,07	0,97	0,87	1,29	0,94	0,97	1,18	cycloartenol synthase, putative / 2,3-epoxysqualene--cycloartenol cyclase,
252618_at	At3g45140	0,66	0,52	0,59	1,45	1,25	0,86	0,97	0,88	lipoxygenase (LOX2)
252612_at	At3g45160	1,12	1,47	1,39	1,47	1,84	0,97	1,02	0,76	expressed protein
252613_at	At3g45190	0,84	0,80	1,04	0,89	1,03	1,13	0,98	1,04	SIT4 phosphatase-associated family protein
252619_at	At3g45210	1,06	0,78	0,88	1,28	1,23	1,15	1,12	1,05	expressed protein
252614_at	At3g45220	1,01	1,02	0,94	1,05	1,10	1,12	1,03	1,05	serpin, putative / serine protease inhibitor, putative
252615_at	At3g45230	0,95	0,86	0,73	1,00	0,89	0,86	0,66	0,78	hydroxyproline-rich glycoprotein family protein
252616_at	At3g45240	1,12	1,07	0,96	0,87	0,97	1,15	1,29	0,98	protein kinase family protein
252620_at	At3g45250	0,95	1,02	1,02	0,98	0,97	0,98	0,99	0,95	---
252573_at	At3g45260	1,01	1,05	0,89	0,91	0,91	0,93	0,86	0,83	zinc finger (C2H2 type) family protein
252621_at	At3g45270	1,00	0,94	0,93	0,97	1,07	1,00	0,96	0,76	---
252571_at	At3g45280	0,89	1,00	0,95	0,93	1,06	1,00	0,94	1,02	syntaxin 72 (SYP72)
252572_at	At3g45290	0,85	1,02	0,93	1,01	1,04	0,95	1,04	1,05	seven transmembrane MLO family protein / MLO-like protein 3 (MLO3)
252570_at	At3g45300	0,96	1,11	1,43	0,82	0,85	0,98	1,18	1,36	isovaleryl-CoA-dehydrogenase (IVD)
252622_at	At3g45310	0,88	0,82	0,75	1,03	1,03	0,92	1,02	0,93	cysteine proteinase, putative
252623_at	At3g45320	1,11	1,02	1,13	1,00	1,00	0,93	0,93	0,88	hypothetical protein
252596_at	At3g45330	1,01	1,00	0,95	1,06	1,02	0,95	1,20	0,76	lectin protein kinase family protein
252597_at	At3g45360	0,97	0,94	0,90	0,88	1,00	1,04	1,00	1,00	pseudogene, hypothetical protein
252598_at	At3g45380	0,98	0,88	0,95	1,03	1,00	1,07	1,08	1,06	hypothetical protein
252599_at	At3g45390	1,01	1,05	0,99	1,09	1,06	1,07	0,98	0,92	lectin protein kinase family protein
252568_at	At3g45410	1,01	1,07	1,10	0,90	0,94	1,06	1,09	1,00	lectin protein kinase family protein
252569_at	At3g45420	1,06	0,83	0,94	0,88	1,08	1,02	0,99	0,74	lectin protein kinase family protein

252574_at	At3g45430	1,02	1,11	0,99	0,88	0,82	0,99	1,02	0,88	lectin protein kinase family protein
252575_at	At3g45440	0,90	0,94	1,03	0,91	0,99	0,83	1,01	0,81	lectin protein kinase family protein
252576_s_at	At3g45450	1,25	0,98	0,91	0,99	1,02	1,22	0,86	0,82	Clp amino terminal domain-containing protein
252577_at	At3g45460	1,00	1,14	0,97	0,98	1,02	1,03	1,01	1,04	hypothetical protein
252578_at	At3g45480	0,94	0,99	1,07	0,96	0,97	0,89	1,00	1,01	zinc finger (C3HC4-type RING finger) family protein
252580_at	At3g45490	1,00	1,08	0,92	0,98	1,03	0,96	0,99	0,95	hypothetical protein
252581_at	At3g45500	0,97	1,02	1,00	1,01	1,02	1,02	1,05	1,09	hypothetical protein
252579_at	At3g45510	0,87	1,01	1,10	1,06	0,94	0,99	0,99	0,84	zinc finger (C3HC4-type RING finger) family protein
252582_at	At3g45530	1,00	1,08	1,03	1,07	1,24	1,19	1,03	1,09	DC1 domain-containing protein
252583_at	At3g45540	0,98	0,98	1,01	0,96	0,98	1,03	0,95	0,85	zinc finger (C3HC4-type RING finger) family protein
252584_at	At3g45560	1,09	0,96	1,05	0,83	1,05	0,99	1,06	0,95	zinc finger (C3HC4-type RING finger) family protein
252585_s_at	At3g45580	1,01	0,99	0,96	1,02	1,10	0,95	1,06	0,94	zinc finger (C3HC4-type RING finger) family protein
252593_at	At3g45590	0,98	0,95	1,04	1,01	0,84	0,85	0,98	1,04	tRNA-splicing endonuclease, putative
252591_at	At3g45600	0,94	1,04	0,94	1,16	1,26	1,00	0,88	0,85	senescence-associated family protein
252586_at	At3g45610	1,00	1,06	1,04	0,89	1,06	1,05	0,96	0,92	Dof-type zinc finger domain-containing protein
252587_at	At3g45620	0,75	0,82	0,75	0,89	1,17	1,06	0,89	0,97	transducin family protein / WD-40 repeat family protein
252588_at	At3g45630	1,00	1,09	1,17	0,75	0,92	1,04	1,05	1,28	RNA recognition motif (RRM)-containing protein
252592_at	At3g45640	0,89	1,18	2,18	0,88	0,91	1,12	1,53	1,73	mitogen-activated protein kinase, putative / MAPK, putative (MPK3)
252589_s_at	At3g45650	1,27	1,30	1,15	0,86	0,61	1,61	1,73	2,00	proton-dependent oligopeptide transport (POT) family protein
252590_at	At3g45670	0,87	1,05	0,96	0,84	1,05	0,97	1,03	0,89	protein kinase-related
252594_at	At3g45680	1,02	1,16	0,78	0,84	1,22	1,14	1,00	1,04	proton-dependent oligopeptide transport (POT) family protein
252595_at	At3g45690	0,91	0,83	0,74	1,09	1,01	0,97	0,92	0,84	proton-dependent oligopeptide transport (POT) family protein
252536_at	At3g45700	0,86	0,99	0,99	0,96	1,08	1,06	1,32	1,09	proton-dependent oligopeptide transport (POT) family protein
252537_at	At3g45710	1,04	1,08	0,93	1,38	0,99	1,02	1,21	1,07	proton-dependent oligopeptide transport (POT) family protein
252538_at	At3g45720	0,89	1,04	1,06	0,93	1,00	0,98	1,08	1,19	proton-dependent oligopeptide transport (POT) family protein
252539_at	At3g45730	1,42	1,53	1,53	0,46	0,59	1,08	1,08	1,10	expressed protein
252540_at	At3g45740	1,17	1,07	1,03	0,79	0,84	0,97	1,02	0,99	hydrolase family protein / HAD-superfamily protein
252541_at	At3g45750	1,12	1,03	0,93	0,83	0,88	1,24	0,98	0,98	expressed protein
252542_at	At3g45770	0,81	0,80	0,75	0,77	0,94	0,94	0,95	0,91	oxidoreductase, zinc-binding dehydrogenase family protein
252543_at	At3g45780	1,40	1,23	1,77	0,71	0,62	1,11	1,15	1,21	protein kinase / nonphototropic hypocotyl protein 1 (NPH1) / phototropin
257448_s_at	At3g45800	1,14	1,07	1,00	0,96	1,11	1,00	1,10	1,07	hypothetical protein
252544_at	At3g45810	0,97	1,01	0,99	0,94	1,15	0,88	0,94	0,96	ferric reductase-like transmembrane component family protein
252545_at	At3g45820	1,03	1,26	1,10	1,04	1,17	0,83	0,81	0,95	hypothetical protein
252546_at	At3g45830	0,72	0,79	0,79	0,93	1,00	1,02	0,98	1,35	expressed protein
252547_at	At3g45840	0,95	1,05	1,30	1,02	0,96	0,96	1,05	1,17	DC1 domain-containing protein
252548_at	At3g45850	0,91	1,12	1,18	0,93	0,86	1,13	1,28	1,44	kinesin motor protein-related
252549_at	At3g45860	0,84	1,05	1,19	0,89	0,76	1,50	1,14	1,31	receptor-like protein kinase, putative
252550_at	At3g45870	0,98	1,01	0,86	1,07	0,99	0,88	0,88	1,01	integral membrane family protein / nodulin MtN21-related
252551_at	At3g45880	1,07	0,88	0,91	0,99	0,88	1,04	0,84	1,09	hypothetical protein
252552_at	At3g45900	1,16	1,59	1,34	1,04	0,62	1,13	1,06	1,09	expressed protein
252553_at	At3g45910	1,07	1,09	0,94	1,06	1,20	1,03	1,00	1,04	hypothetical protein
252554_s_at	At3g45920	1,02	1,12	1,11	0,89	1,05	0,99	0,94	0,93	receptor protein kinase-related
252562_s_at	At3g45930	1,05	0,97	0,81	1,28	1,60	1,08	0,83	0,68	histone H4
252555_at	At3g45940	1,00	0,98	1,02	0,82	1,02	0,96	0,96	0,88	alpha-xylosidase, putative
252556_at	At3g45950	1,03	1,06	1,11	1,02	0,97	1,05	0,96	0,93	splicing factor-related
252557_at	At3g45960	1,48	1,64	1,82	1,83	1,66	0,76	1,11	1,21	expansin family protein (EXPL3)

252563_at	At3g45970	0,84	0,90	2,05	1,00	1,28	1,07	1,11	1,38	expansin family protein (EXPL1)
252561_at	At3g45980	0,97	0,94	0,89	1,11	1,28	0,85	0,87	0,81	histone H2B
252558_at	At3g45990	0,95	1,05	1,08	0,96	1,15	0,92	1,03	0,97	actin-depolymerizing factor, putative
252565_at	At3g46000	0,85	0,71	0,74	1,00	1,03	0,81	0,88	0,85	actin-depolymerizing factor, putative (ADF2)
252564_at	At3g46010	1,08	0,88	0,86	1,13	1,13	0,93	0,88	0,96	actin-depolymerizing factor 1 (ADF1)
252559_at	At3g46020	1,21	0,95	1,32	1,13	1,15	1,05	0,89	0,91	RNA-binding protein, putative
252560_at	At3g46030	0,87	0,71	0,76	1,03	1,22	0,86	0,80	0,63	histone H2B, putative
252566_at	At3g46040	1,25	1,26	1,09	1,23	1,16	1,02	1,00	1,01	40S ribosomal protein S15A (RPS15aD)
252514_at	At3g46060	1,12	0,91	0,95	1,46	1,78	0,88	0,89	0,95	Ras-related protein (ARA-3) / small GTP-binding protein, putative
252567_at	At3g46070	1,00	0,98	0,97	0,93	1,05	1,00	1,09	1,08	zinc finger (C2H2 type) family protein
252516_at	At3g46100	1,15	0,99	1,10	0,98	0,89	1,19	1,34	1,23	histidyl-tRNA synthetase / histidine--tRNA ligase
252533_at	At3g46110	0,59	0,94	0,87	0,67	1,07	0,82	0,91	1,13	expressed protein
252534_at	At3g46130	0,85	1,08	0,95	1,08	1,01	1,05	1,09	0,80	myb family transcription factor (MYB48)
252535_at	At3g46150	0,86	0,96	1,14	1,18	1,11	0,87	1,02	1,23	hypothetical protein
252505_at	At3g46170	1,10	0,93	0,96	0,96	0,94	0,95	1,00	0,95	short-chain dehydrogenase/reductase (SDR) family protein
252506_at	At3g46180	1,06	0,88	1,05	1,22	1,21	0,90	1,01	1,13	UDP-galactose/UDP-glucose transporter-related
252507_at	At3g46200	1,34	1,43	1,25	1,00	1,08	1,10	1,09	1,02	MutT/nudix family protein
252508_at	At3g46210	1,46	1,20	1,16	0,89	0,79	1,19	1,13	1,01	3' exoribonuclease family domain 1-containing protein
252513_at	At3g46220	1,03	0,96	1,06	1,07	1,01	1,02	1,09	0,97	expressed protein
252515_at	At3g46230	3,19	3,70	2,76	1,49	2,15	1,49	1,32	1,22	17.4 kDa class I heat shock protein (HSP17.4-CI)
252509_at	At3g46240	0,95	0,97	0,97	1,06	1,20	1,02	1,18	0,96	protein kinase-related
252510_at	At3g46270	0,97	0,98	1,13	1,42	1,28	1,09	0,99	1,11	receptor protein kinase-related
252511_at	At3g46280	0,73	0,91	0,68	3,20	1,98	1,32	0,79	0,80	protein kinase-related
252512_at	At3g46290	0,88	0,60	0,56	0,75	0,88	0,89	0,73	0,75	protein kinase, putative
252517_at	At3g46340	0,99	1,03	1,01	0,92	1,08	0,91	0,90	0,97	leucine-rich repeat protein kinase, putative
252518_at	At3g46350	1,06	0,94	1,05	0,97	1,09	1,00	1,00	0,87	leucine-rich repeat protein kinase, putative
252519_at	At3g46360	0,86	0,94	0,96	1,00	1,01	0,91	1,13	0,97	hypothetical protein
252520_at	At3g46370	1,03	0,97	1,14	0,69	0,81	2,22	2,19	1,97	leucine-rich repeat protein kinase, putative
252521_at	At3g46380	0,95	1,08	0,80	0,93	1,02	1,03	1,19	0,96	hypothetical protein
252522_at	At3g46390	1,05	1,05	1,01	0,96	1,08	0,98	0,98	1,03	hypothetical protein
252523_at	At3g46400	0,99	1,04	0,99	1,04	1,10	0,94	1,04	1,05	leucine-rich repeat protein kinase, putative
252524_at	At3g46430	1,28	1,09	0,92	1,38	1,69	1,17	0,98	0,98	expressed protein
252527_at	At3g46440	1,00	0,91	0,81	1,14	1,06	1,10	1,05	1,02	NAD-dependent epimerase/dehydratase family protein
252525_at	At3g46450	0,73	0,85	0,92	0,96	1,08	1,05	0,93	1,21	SEC14 cytosolic factor family protein / phosphoglyceride transfer family pro
252528_at	At3g46460	1,16	0,98	1,15	1,16	1,24	0,88	0,90	1,04	ubiquitin-conjugating enzyme 13 (UBC13)
252526_at	At3g46480	0,98	1,07	0,99	0,88	1,18	0,91	0,99	0,99	oxidoreductase, 2OG-Fe(II) oxygenase family protein
252529_at	At3g46490	0,57	0,53	0,54	1,07	0,95	0,91	1,02	1,04	oxidoreductase, 2OG-Fe(II) oxygenase family protein
252530_at	At3g46500	0,77	0,81	0,63	0,98	1,01	1,09	1,17	1,12	oxidoreductase, 2OG-Fe(II) oxygenase family protein
252486_at	At3g46510	1,07	1,09	1,12	1,11	1,08	0,94	0,86	0,91	armadillo/beta-catenin repeat family protein / U-box domain-containing fami
252531_at	At3g46520	1,06	0,98	0,96	0,96	1,17	0,95	0,89	1,01	actin 12 (ACT12)
252485_at	At3g46530	0,99	1,06	1,03	0,65	0,92	1,29	1,08	1,09	disease resistance protein, RPP13-like (CC-NBS class), putative
252478_at	At3g46540	0,94	0,90	1,01	1,15	1,55	0,96	0,94	0,80	epsin N-terminal homology (ENTH) domain-containing protein / clathrin ass
252479_at	At3g46560	1,47	1,14	1,04	1,22	1,15	0,95	0,99	0,92	mitochondrial import inner membrane translocase (TIM9)
252532_at	At3g46570	0,97	1,02	0,95	0,91	1,19	1,21	1,03	0,84	glycosyl hydrolase family 17 protein
252480_at	At3g46580	1,10	1,08	1,20	1,26	1,09	0,97	0,88	0,88	methyl-CpG-binding domain-containing protein
252504_at	At3g46590	0,96	1,16	1,01	0,55	0,56	1,10	1,18	1,28	telomere repeat-binding protein, putative

252483_at	At3g46600	1,03	1,18	1,21	0,82	0,80	0,97	0,90	1,09	scarecrow transcription factor family protein
252473_s_at	At3g46610	0,91	0,67	0,71	0,70	0,61	0,97	1,11	0,82	pentatricopeptide (PPR) repeat-containing protein
252474_at	At3g46620	0,88	0,88	0,88	0,66	0,61	1,24	1,32	1,31	zinc finger (C3HC4-type RING finger) family protein
252481_at	At3g46630	0,98	1,10	1,24	0,88	0,86	1,03	0,83	0,86	expressed protein
252475_s_at	At3g46640	0,75	0,78	0,83	0,54	0,75	0,95	1,06	1,09	myb family transcription factor
252476_at	At3g46650	0,97	1,08	0,92	1,00	1,21	0,95	1,09	1,13	UDP-glucoronosyl/UDP-glucosyl transferase family protein
252487_at	At3g46660	0,32	0,41	0,86	1,08	0,97	0,93	0,95	0,98	UDP-glucoronosyl/UDP-glucosyl transferase family protein
252482_at	At3g46670	0,47	0,64	0,95	1,19	0,99	1,29	1,47	1,23	UDP-glucoronosyl/UDP-glucosyl transferase family protein
252477_at	At3g46680	0,89	1,00	0,84	0,97	0,90	1,11	0,94	1,20	UDP-glucoronosyl/UDP-glucosyl transferase family protein
252484_at	At3g46690	1,02	0,89	0,97	0,95	0,97	0,91	1,02	1,39	UDP-glucoronosyl/UDP-glucosyl transferase family protein
252488_at	At3g46700	0,76	0,88	0,82	0,91	0,90	1,09	1,08	1,02	UDP-glucoronosyl/UDP-glucosyl transferase family protein
252489_at	At3g46710	1,12	1,03	1,01	0,92	1,01	1,00	1,05	1,06	disease resistance protein (CC-NBS-LRR class), putative
252490_at	At3g46720	0,93	1,10	0,99	1,06	1,07	1,03	1,13	1,20	UDP-glucoronosyl/UDP-glucosyl transferase family protein
252491_at	At3g46730	1,03	1,02	0,93	0,93	0,81	1,18	0,90	1,04	disease resistance protein (CC-NBS class), putative
252492_at	At3g46740	1,16	1,09	1,11	0,86	0,77	1,04	0,95	0,94	chloroplast outer envelope protein, putative
252493_at	At3g46750	1,04	1,02	0,98	1,11	1,01	1,03	1,00	0,96	hypothetical protein
252494_at	At3g46760	0,95	1,09	1,10	1,00	1,04	1,03	0,98	0,99	protein kinase family protein
252495_at	At3g46770	0,95	1,06	1,06	0,89	0,84	0,99	0,94	1,12	transcriptional factor B3 family protein
252441_at	At3g46780	0,69	0,64	0,70	0,61	0,61	0,91	0,93	1,05	expressed protein
252496_at	At3g46790	0,91	0,91	0,84	0,90	0,75	0,96	0,83	0,99	pentatricopeptide (PPR) repeat-containing protein
252497_at	At3g46800	1,10	1,21	0,96	1,22	1,15	1,07	1,04	0,88	DC1 domain-containing protein
252498_at	At3g46810	1,04	0,96	1,02	1,16	1,16	0,93	0,97	0,83	DC1 domain-containing protein
252471_at	At3g46820	1,01	1,03	1,02	0,94	0,92	0,99	0,88	0,80	serine/threonine protein phosphatase PP1 isozyme 5 (TOPP5) / phosphopr
252472_at	At3g46830	0,88	0,72	0,75	1,69	1,41	0,84	0,79	0,74	Ras-related protein (RAB11A) / small GTP-binding protein, putative
252499_s_at	At3g46840	0,95	1,01	0,86	0,93	0,94	0,95	0,95	0,92	subtilase family protein
252500_at	At3g46860	0,99	1,02	1,07	1,10	1,00	0,98	1,12	0,85	serine protease inhibitor, potato inhibitor I-type family protein
252501_at	At3g46880	1,08	0,95	0,98	1,48	1,26	1,01	1,03	0,99	expressed protein
252502_at	At3g46900	1,64	1,67	1,38	2,08	1,68	1,65	1,22	1,32	copper transporter, putative
252503_at	At3g46910	0,97	0,99	0,94	0,95	0,97	0,96	0,94	1,12	hypothetical protein
252469_at	At3g46920	0,97	0,97	0,97	0,96	0,94	1,17	0,99	1,15	protein kinase family protein
252470_at	At3g46930	0,79	1,41	1,83	1,15	2,01	1,15	1,53	1,29	protein kinase family protein
252442_at	At3g46940	1,27	1,14	1,00	1,59	1,36	0,93	0,88	0,83	deoxyuridine 5'-triphosphate nucleotidohydrolase family
252443_at	At3g46960	1,10	1,32	1,14	0,93	0,90	1,15	1,22	1,28	DEAD/DEAH box helicase, putative
252468_at	At3g46970	0,50	0,45	0,56	0,71	0,60	0,91	0,95	0,87	starch phosphorylase, putative
252444_at	At3g46980	0,88	0,90	1,10	0,75	0,72	0,97	0,96	1,07	transporter-related
252445_at	At3g47000	0,50	0,54	0,51	0,77	0,84	0,87	0,88	0,95	glycosyl hydrolase family 3 protein
252446_at	At3g47010	0,96	0,86	0,86	0,82	0,80	0,80	0,74	0,74	glycosyl hydrolase family 3 protein
252447_at	At3g47040	0,85	1,20	0,94	1,13	1,04	0,91	1,09	0,83	glycosyl hydrolase family 3 protein
252448_at	At3g47050	0,92	1,05	0,87	0,97	1,13	0,86	1,03	1,18	glycosyl hydrolase family 3 protein
252449_at	At3g47060	1,17	1,50	1,45	0,91	0,89	1,14	1,08	1,25	FtsH protease, putative
252463_at	At3g47070	1,23	1,11	1,09	0,96	1,07	1,00	0,93	0,95	expressed protein
252467_at	At3g47080	0,57	0,62	0,75	0,86	0,77	0,93	0,84	0,93	expressed protein
252450_s_at	At3g47090	1,14	1,08	0,94	1,24	0,76	1,30	1,17	1,50	leucine-rich repeat transmembrane protein kinase, putative
252451_at	At3g47100	1,03	1,08	0,97	0,77	1,04	1,00	0,89	1,10	expressed protein
252452_at	At3g47110	0,95	0,92	0,97	1,25	1,02	1,05	1,08	0,95	leucine-rich repeat transmembrane protein kinase, putative
252453_at	At3g47120	0,92	1,01	1,03	0,87	1,08	1,00	1,15	1,03	RNA recognition motif (RRM)-containing protein

252454_at	At3g47130	1,04	1,03	0,90	1,15	0,96	0,89	0,86	1,15	F-box family protein-related
252455_at	At3g47140	1,00	1,02	0,95	0,94	1,09	0,92	1,01	0,96	F-box family protein-related
252464_at	At3g47160	1,58	1,98	1,27	0,78	0,77	1,09	1,11	1,13	expressed protein
252456_at	At3g47170	0,93	1,18	0,96	1,03	1,11	1,05	0,91	0,89	transferase family protein
252457_at	At3g47180	1,14	1,38	1,08	1,16	1,62	1,10	0,97	0,70	zinc finger (C3HC4-type RING finger) family protein
252458_at	At3g47210	0,96	1,11	1,04	1,01	0,83	1,12	1,23	0,94	expressed protein
252459_s_at	At3g47220	1,12	1,08	1,18	0,92	1,00	0,96	1,00	0,79	phosphoinositide-specific phospholipase C family protein
252460_at	At3g47230	1,03	0,91	0,98	0,94	1,10	0,94	0,83	1,13	expressed protein
252461_at	At3g47240	1,02	0,93	0,94	0,90	1,02	1,01	1,00	1,01	hypothetical protein
252462_at	At3g47250	0,94	0,99	1,03	0,85	0,81	1,02	0,93	0,93	expressed protein
252465_x_at	At3g47260	0,94	1,10	1,03	0,95	1,04	1,07	0,91	1,02	Ulp1 protease family protein
252466_x_at	At3g47270	0,98	1,08	1,03	0,96	1,11	0,89	1,10	1,05	hypothetical protein
252412_at	At3g47295	0,96	1,33	1,38	1,57	1,53	0,88	0,87	1,04	expressed protein
252434_s_at	At3g47310	1,15	0,99	1,00	1,02	0,97	1,06	0,90	0,86	---
252435_at	At3g47320	1,21	1,16	1,16	0,85	1,11	1,06	1,09	1,18	Mutator-like transposase family
252436_x_at	At3g47330	1,01	1,00	1,09	1,05	0,99	0,96	0,95	1,37	pseudogene, hypothetical protein
252415_at	At3g47340	1,26	1,87	2,05	0,81	0,70	1,00	0,97	0,99	asparagine synthetase 1 (glutamine-hydrolyzing) / glutamine-dependent as
252413_at	At3g47370	1,31	1,07	0,98	1,40	1,35	0,94	0,92	0,88	40S ribosomal protein S20 (RPS20B)
252437_at	At3g47380	1,02	1,07	1,05	1,27	1,38	1,16	1,11	1,18	invertase/pectin methylesterase inhibitor family protein
252438_at	At3g47390	1,07	0,84	0,67	0,97	0,98	0,98	0,79	0,67	cytidine/deoxycytidylate deaminase family protein
252439_at	At3g47400	1,08	1,11	1,19	1,13	1,25	0,96	1,01	0,86	pectinesterase family protein
252414_at	At3g47420	1,46	1,70	0,85	1,00	1,10	1,04	0,97	1,01	glycerol-3-phosphate transporter, putative / glycerol 3-phosphate permease
252411_at	At3g47430	1,38	1,72	2,02	0,76	0,74	1,48	1,46	1,26	peroxisomal biogenesis factor 11 family protein / PEX11 family protein
252440_at	At3g47440	0,90	1,04	0,94	0,96	0,93	1,05	0,86	1,06	major intrinsic family protein / MIP family protein
252410_at	At3g47450	1,12	1,05	1,03	0,82	0,80	1,09	1,00	1,03	expressed protein
252416_at	At3g47460	0,98	1,12	1,00	1,36	1,18	1,06	1,23	1,13	SMC2-like condensin, putative
252430_at	At3g47470	0,97	0,95	1,11	0,89	0,91	1,03	1,01	1,15	chlorophyll A-B binding protein 4, chloroplast / LHCI type III CAB-4 (CAB4)
252417_at	At3g47480	1,44	1,19	1,18	1,22	1,38	1,18	1,03	1,09	calcium-binding EF hand family protein
252418_at	At3g47490	1,15	1,10	1,10	0,76	0,85	1,06	1,03	1,05	HNH endonuclease domain-containing protein
252429_at	At3g47500	1,21	1,33	1,19	0,76	1,11	1,27	1,31	1,26	Dof-type zinc finger domain-containing protein
252419_at	At3g47510	1,56	1,65	1,72	1,20	1,24	1,18	1,61	1,39	expressed protein
252407_at	At3g47520	0,99	0,87	0,87	1,22	1,03	0,94	0,86	0,83	malate dehydrogenase (NAD), chloroplast (MDH)
252420_at	At3g47530	1,02	1,00	1,12	0,96	0,93	1,01	0,98	1,17	pentatricopeptide (PPR) repeat-containing protein
252421_at	At3g47540	0,95	1,14	1,19	0,83	0,96	0,99	1,05	1,23	chitinase, putative
252422_at	At3g47550	0,78	1,01	1,69	0,94	0,96	1,02	1,18	1,61	zinc finger (C3HC4-type RING finger) family protein
252433_at	At3g47560	1,21	1,19	1,54	0,74	0,65	1,28	1,29	1,34	esterase/lipase/thioesterase family protein
252378_at	At3g47570	1,11	1,04	1,17	0,94	0,69	1,24	1,06	1,11	leucine-rich repeat transmembrane protein kinase, putative
252423_at	At3g47590	1,10	0,89	0,86	1,15	1,00	1,04	1,03	0,98	esterase/lipase/thioesterase family protein
252408_at	At3g47600	1,04	1,13	0,90	0,82	0,87	1,02	0,79	0,89	myb family transcription factor (MYB94)
252424_at	At3g47610	1,27	1,62	1,59	1,20	1,13	1,17	0,95	1,41	expressed protein
252425_at	At3g47620	1,04	0,87	0,79	1,01	1,04	0,87	0,84	0,76	TCP family transcription factor, putative
252426_at	At3g47630	1,06	1,05	1,48	0,92	1,10	1,36	1,06	1,31	expressed protein
252427_at	At3g47640	1,61	1,84	1,38	0,95	0,68	1,63	1,24	1,11	basic helix-loop-helix (bHLH) family protein
252409_at	At3g47650	1,20	1,09	0,99	0,93	0,81	1,12	1,02	0,98	bundle-sheath defective protein 2 family / bsd2 family
252428_at	At3g47660	0,94	1,03	1,10	0,85	1,11	0,95	1,02	1,00	regulator of chromosome condensation (RCC1) family protein
252406_at	At3g47670	0,75	0,73	0,52	1,08	0,98	0,84	0,64	0,62	invertase/pectin methylesterase inhibitor family protein

252432_at	At3g47680	1,03	1,03	1,05	0,78	0,83	1,18	1,11	1,07	expressed protein
252431_at	At3g47700	0,98	0,93	1,06	1,08	0,81	0,91	1,15	1,28	chromosome structural maintenance protein-related
252379_at	At3g47730	0,84	0,79	0,88	0,80	0,76	1,06	1,06	1,33	ABC transporter family protein
252380_at	At3g47740	1,00	1,04	0,96	1,07	0,98	0,98	0,92	0,92	ABC transporter family protein
252381_s_at	At3g47750	0,89	0,91	0,89	0,92	0,89	0,92	1,09	0,87	ABC transporter family protein
252382_at	At3g47770	1,19	0,96	1,04	1,03	1,18	1,07	1,00	1,12	ABC transporter family protein
252383_at	At3g47780	1,00	1,21	0,91	0,93	1,05	1,17	1,01	1,13	ABC transporter family protein
252384_at	At3g47790	1,00	1,04	1,10	0,97	0,90	1,06	1,02	1,10	ABC transporter family protein
252387_at	At3g47800	0,81	1,02	1,09	1,15	1,34	0,78	0,87	0,84	aldose 1-epimerase family protein
252385_at	At3g47810	0,87	0,82	0,78	1,24	1,02	0,94	0,90	0,94	calcineurin-like phosphoesterase family protein
252389_at	At3g47833	1,06	0,96	0,88	1,35	1,40	0,86	0,86	0,82	expressed protein
252390_at	At3g47836	1,49	1,31	0,99	1,15	0,99	1,04	0,94	0,89	expressed protein
252386_at	At3g47840	0,85	1,01	1,04	0,77	1,05	0,98	1,13	0,76	pentatricopeptide (PPR) repeat-containing protein
252388_at	At3g47850	1,15	1,19	1,12	1,02	1,06	1,07	0,93	0,86	expressed protein
252391_at	At3g47860	0,50	0,52	0,56	0,55	0,60	0,75	0,81	0,80	apolipoprotein D-related
252392_s_at	At3g47890	0,77	0,85	0,98	0,90	0,63	1,17	0,94	1,05	ubiquitin carboxyl-terminal hydrolase-related
252393_at	At3g47910	0,87	0,98	1,06	1,00	0,97	1,09	0,97	1,12	expressed protein
252376_at	At3g47930	0,94	0,88	0,82	0,85	0,99	0,96	0,86	0,84	L-galactono-1,4-lactone dehydrogenase, putative
252394_at	At3g47940	0,88	1,03	0,74	1,16	0,95	1,09	1,15	0,97	DNAJ heat shock protein, putative
252395_at	At3g47950	1,11	0,85	1,24	1,04	0,92	1,01	0,80	1,02	ATPase, plasma membrane-type, putative / proton pump, putative
252377_at	At3g47960	0,56	0,55	0,87	0,91	0,94	0,95	1,07	1,00	proton-dependent oligopeptide transport (POT) family protein
252396_at	At3g47970	0,99	0,93	0,93	1,06	1,00	1,03	0,93	1,06	integral membrane HPP family protein
252397_at	At3g47980	0,93	1,07	0,75	1,03	0,91	0,87	1,18	0,90	integral membrane HPP family protein
252398_at	At3g47990	1,41	1,31	1,66	0,88	1,03	1,29	1,46	1,88	zinc finger (C3HC4-type RING finger) family protein
252372_at	At3g48000	0,82	0,80	0,79	0,88	0,89	0,99	0,93	0,99	aldehyde dehydrogenase (ALDH2)
252399_at	At3g48010	1,05	0,95	0,99	0,95	1,02	1,11	0,93	0,96	cyclic nucleotide-regulated ion channel, putative (CNGC16)
252400_at	At3g48020	0,86	1,05	0,85	1,26	1,14	0,97	1,11	1,10	expressed protein
252401_at	At3g48030	1,04	0,86	1,06	1,01	1,06	1,03	1,17	1,42	hypoxia-responsive family protein / zinc finger (C3HC4-type RING finger) fa
252375_at	At3g48040	1,10	1,09	1,62	0,98	0,91	1,01	1,14	1,12	Rac-like GTP-binding protein (ARAC8)
252402_s_at	At3g48050	0,79	0,82	1,12	0,93	0,85	1,05	0,95	1,04	bromo-adjacent homology (BAH) domain-containing protein
252371_at	At3g48070	1,09	1,25	1,53	0,94	0,67	1,15	1,13	1,34	expressed protein
252403_at	At3g48080	1,06	0,88	0,94	1,07	0,94	1,19	1,14	0,99	lipase class 3 family protein / disease resistance protein-related
252373_at	At3g48090	1,08	1,11	1,67	1,06	1,18	1,07	1,44	1,39	disease resistance protein (EDS1)
252374_at	At3g48100	1,08	2,08	1,48	0,91	1,00	1,11	1,48	1,92	two-component responsive regulator / response regulator 5 (ARR5) / respo
252404_at	At3g48110	0,77	0,69	0,67	0,70	0,65	1,04	1,07	1,03	aminoacyl-t-RNA synthetase, putative
252405_at	At3g48120	0,87	0,72	0,76	1,07	0,98	1,13	1,24	1,34	expressed protein
252347_at	At3g48130	1,88	1,86	1,66	1,04	1,25	1,09	1,02	0,80	---
252348_at	At3g48140	1,41	1,22	1,17	1,28	1,31	1,06	0,99	0,99	senescence-associated protein, putative
252349_at	At3g48150	1,07	1,15	1,22	1,10	1,10	0,95	1,03	0,81	cell division cycle family protein / CDC family protein
252354_at	At3g48170	0,91	0,97	0,97	0,92	0,88	1,01	1,03	0,94	betaine-aldehyde dehydrogenase, putative
252352_at	At3g48185	1,37	1,14	0,93	1,56	1,21	0,89	0,93	0,79	expressed protein
252350_at	At3g48190	0,70	0,82	0,77	0,72	0,71	0,90	1,24	1,10	ataxia-telangiectasia mutated protein (Atm)
252353_at	At3g48200	0,85	0,75	0,73	0,73	0,82	1,11	1,10	1,01	expressed protein
252351_at	At3g48210	1,01	0,83	1,01	0,95	1,12	0,94	0,89	0,84	expressed protein
252355_at	At3g48250	1,13	1,05	0,77	1,00	0,84	0,88	0,91	0,72	pentatricopeptide (PPR) repeat-containing protein
252365_at	At3g48350	0,89	0,71	0,75	1,05	0,83	0,84	0,84	0,84	cysteine proteinase, putative

252367_at	At3g48360	1,02	1,90	1,57	0,50	0,30	1,16	2,61	2,47	speckle-type POZ protein-related
252356_at	At3g48370	0,97	1,07	1,01	0,91	0,96	1,01	1,01	1,11	---
252357_at	At3g48410	1,07	1,18	1,29	1,28	1,32	1,10	1,00	0,83	hydrolase, alpha/beta fold family protein
252366_at	At3g48420	0,83	0,72	0,68	0,79	0,80	1,17	1,14	1,26	haloacid dehalogenase-like hydrolase family protein
252358_at	At3g48425	1,34	1,39	1,45	1,14	1,02	1,06	1,04	0,90	endonuclease/exonuclease/phosphatase family protein
252359_at	At3g48440	0,90	0,92	0,97	0,97	1,22	1,09	0,86	0,82	zinc finger (CCCH-type) family protein
252364_at	At3g48450	1,16	1,30	1,02	1,10	1,11	1,24	1,31	1,25	nitrate-responsive NOI protein, putative
252363_at	At3g48460	1,49	1,38	1,56	1,51	1,89	0,89	0,93	1,04	GDSL-motif lipase/hydrolase family protein
252360_at	At3g48480	0,99	1,15	1,12	1,15	0,94	1,02	1,17	0,93	hypothetical protein
252361_at	At3g48490	1,10	1,10	1,20	1,17	1,44	1,07	0,89	1,02	expressed protein
252362_at	At3g48500	1,28	1,23	1,34	0,68	0,60	1,20	1,07	1,28	expressed protein
252321_at	At3g48510	0,79	0,91	0,96	1,00	0,88	0,85	1,05	1,06	expressed protein
252368_at	At3g48520	0,82	0,84	0,70	0,89	0,73	1,11	1,58	1,39	cytochrome P450 family protein
252323_at	At3g48530	0,81	0,93	1,29	1,02	1,01	0,85	0,91	0,89	CBS domain-containing protein
252369_at	At3g48540	0,98	1,07	1,08	1,19	1,14	0,91	0,83	0,87	cytidine/deoxycytidylate deaminase family protein
252322_at	At3g48550	1,08	1,11	1,04	1,06	1,23	1,08	0,96	0,98	expressed protein
252325_at	At3g48560	1,17	1,18	1,09	0,92	0,83	1,02	0,98	0,95	acetolactate synthase, chloroplast / acetohydroxy-acid synthase (ALS)
252324_at	At3g48565	1,02	0,80	0,61	1,14	1,09	0,92	0,86	0,70	protein transport protein SEC61 gamma subunit, putative
252328_at	At3g48570	1,05	0,76	0,75	1,08	1,24	0,93	0,85	0,77	protein transport protein SEC61 gamma subunit, putative
252320_at	At3g48580	0,86	0,65	0,34	0,94	1,21	1,02	0,77	0,73	xyloglucan:xyloglucosyl transferase, putative / xyloglucan endotransglycosy
252370_at	At3g48600	1,10	1,31	0,96	1,11	1,09	1,01	0,87	1,25	SWIB complex BAF60b domain-containing protein
252343_at	At3g48610	1,09	0,94	0,92	1,06	1,07	0,87	0,82	0,86	phosphoesterase family protein
252344_s_at	At3g48620	0,92	0,90	1,03	0,80	0,90	0,87	0,99	1,03	chloroplast outer membrane protein-related
252345_at	At3g48640	0,96	1,26	1,55	1,18	0,92	1,23	1,11	1,30	expressed protein
252346_at	At3g48650	1,03	1,07	0,97	0,85	0,92	1,51	1,07	0,88	pseudogene, At14a-related protein
252326_at	At3g48680	1,28	1,18	1,06	1,03	1,02	0,91	0,91	0,85	bacterial transferase hexapeptide repeat-containing protein
252315_at	At3g48690	0,64	0,73	0,77	0,89	0,87	0,82	0,84	0,91	expressed protein
252316_at	At3g48700	1,35	1,23	1,36	2,07	2,05	1,26	1,08	0,89	expressed protein
252319_at	At3g48710	1,21	1,02	1,28	1,00	1,00	1,12	1,58	1,31	expressed protein
252317_at	At3g48720	1,22	1,57	1,18	1,29	0,92	1,15	1,11	1,19	transferase family protein
252318_at	At3g48730	0,89	0,81	0,90	0,79	0,81	1,00	0,97	0,90	glutamate-1-semialdehyde 2,1-aminomutase 2 (GSA 2) / glutamate-1-semia
252327_at	At3g48740	1,14	1,00	0,94	0,64	0,50	0,84	0,84	0,84	nodulin MtN3 family protein
252337_at	At3g48750	1,05	1,06	1,07	1,20	1,33	0,87	0,81	0,83	cell division control protein 2 homolog A (CDC2A)
252329_at	At3g48760	0,67	0,63	0,81	0,88	0,93	0,96	0,87	1,14	zinc finger (DHHC type) family protein
252330_at	At3g48770	1,22	1,03	1,19	0,97	1,02	0,99	1,10	0,89	hypothetical protein
252331_s_at	At3g48790	0,94	0,68	0,89	1,09	1,30	0,92	0,87	1,08	serine C-palmitoyltransferase, putative
252332_at	At3g48810	0,90	0,97	1,11	0,87	1,21	0,96	1,08	1,12	pentatricopeptide (PPR) repeat-containing protein
252333_at	At3g48830	0,97	1,04	0,96	0,96	0,99	0,92	0,95	1,08	polynucleotide adenylyltransferase family protein / RNA recognition motif (F
252334_at	At3g48850	1,04	1,27	1,03	0,99	1,03	1,12	1,16	1,44	mitochondrial phosphate transporter, putative
252335_at	At3g48860	0,74	0,76	0,96	0,86	0,94	0,96	0,84	0,91	expressed protein
252336_at	At3g48880	1,03	0,95	0,95	0,78	1,21	0,96	0,99	0,98	F-box family protein
252338_at	At3g48890	1,17	1,03	0,96	1,09	1,11	0,98	0,96	0,94	cytochrome b5 domain-containing protein
252339_at	At3g48910	1,03	1,07	1,13	1,05	1,08	1,01	1,06	1,10	expressed protein
252340_at	At3g48920	1,04	1,11	1,07	1,02	1,14	1,08	0,95	1,07	myb family transcription factor (MYB45)
252297_at	At3g48930	1,39	1,14	0,93	1,21	0,97	0,96	0,96	0,91	40S ribosomal protein S11 (RPS11A)
252341_at	At3g48940	1,02	1,03	1,14	1,05	1,20	0,91	0,90	0,98	remorin family protein

252342_at	At3g48950	0,88	1,08	1,01	0,99	0,96	1,03	0,98	1,02	glycoside hydrolase family 28 protein / polygalacturonase (pectinase) family
252283_at	At3g48960	1,03	1,07	1,20	1,08	1,03	1,02	0,94	1,30	60S ribosomal protein L13 (RPL13C)
252296_at	At3g48970	1,36	1,63	1,34	1,19	1,13	1,19	1,04	0,86	copper-binding family protein
252293_at	At3g48990	0,65	0,67	0,81	0,89	0,85	0,96	0,99	0,90	AMP-dependent synthetase and ligase family protein
252292_at	At3g49000	1,69	1,60	2,17	0,89	0,90	1,38	1,32	1,19	RNA polymerase III subunit RPC82 family protein
252294_at	At3g49010	1,23	1,10	1,05	1,09	0,96	0,99	1,00	0,99	60S ribosomal protein L13 (RPL13B) / breast basic conserved protein 1-rela
252284_at	At3g49020	0,94	1,14	1,03	0,99	0,99	0,97	1,14	0,92	F-box family protein
252285_at	At3g49050	0,90	0,74	0,83	0,97	0,81	1,18	1,13	1,07	lipase class 3 family protein / calmodulin-binding heat-shock protein, putativ
252298_at	At3g49060	1,36	1,76	1,76	1,05	0,99	1,13	1,15	1,10	protein kinase family protein / U-box domain-containing protein
252286_at	At3g49070	0,97	1,01	1,07	1,17	1,11	0,98	0,93	1,13	expressed protein
252287_at	At3g49080	1,08	0,95	0,82	1,05	1,17	0,88	0,89	0,83	ribosomal protein S9 family protein
252288_at	At3g49090	1,03	1,01	0,98	1,12	0,87	0,93	1,00	0,90	ribosomal protein S9 family protein
252295_at	At3g49100	0,96	0,85	0,77	1,40	1,49	0,92	0,82	0,96	signal recognition particle 9 kDa protein, putative / SRP9, putative
252291_s_at	At3g49120	0,70	0,75	0,93	0,96	1,29	0,85	0,82	1,00	peroxidase 33 (PER33) (P33) (PRXCA) / neutral peroxidase C (PERC)
252289_at	At3g49130	1,25	1,14	1,06	0,92	1,12	1,17	1,01	0,95	hypothetical protein
252290_at	At3g49140	1,04	0,96	1,03	0,76	0,73	1,19	1,21	1,37	pentatricopeptide (PPR) repeat-containing protein
252299_at	At3g49150	0,95	1,04	1,13	0,97	0,97	0,95	0,89	0,93	F-box family protein
252300_at	At3g49160	0,96	1,05	1,19	0,58	0,57	0,67	0,85	0,85	pyruvate kinase family protein
252301_at	At3g49170	0,85	1,00	0,95	1,12	0,96	0,95	0,94	0,91	pentatricopeptide (PPR) repeat-containing protein
252252_at	At3g49180	1,26	1,03	1,29	1,00	1,06	0,94	0,79	1,02	transducin family protein / WD-40 repeat family protein
252302_at	At3g49190	1,04	0,95	0,95	1,10	1,05	0,97	1,08	0,88	condensation domain-containing protein
252303_at	At3g49210	1,01	0,99	1,30	1,27	1,33	1,08	1,15	1,31	expressed protein
252255_at	At3g49220	1,08	1,04	1,14	1,29	1,31	0,91	0,94	0,92	pectinesterase family protein
252304_at	At3g49230	1,00	0,91	1,10	1,02	0,91	0,91	1,01	1,08	hypothetical protein
252305_at	At3g49240	1,15	0,78	0,90	0,92	0,99	1,01	0,97	0,94	pentatricopeptide (PPR) repeat-containing protein
252254_at	At3g49250	0,98	1,07	1,05	1,06	1,11	1,13	0,93	0,94	expressed protein
252280_at	At3g49260	0,93	0,86	0,99	0,81	0,78	1,03	0,94	1,01	calmodulin-binding family protein
252306_at	At3g49270	1,02	1,11	0,94	1,01	0,99	0,98	1,03	1,28	expressed protein
252307_at	At3g49280	1,05	0,93	0,99	1,02	0,99	0,89	1,04	0,99	hypothetical protein
252253_at	At3g49300	0,91	1,12	0,94	1,02	1,11	1,00	1,11	0,98	proline-rich family protein
252308_at	At3g49310	0,89	0,64	0,59	0,97	1,29	0,91	0,80	0,79	expressed protein
252281_at	At3g49320	1,51	1,43	1,24	0,94	0,83	0,92	0,92	0,79	expressed protein
252309_at	At3g49340	0,96	0,87	1,03	1,01	0,95	0,92	1,05	1,12	cysteine proteinase, putative
252310_at	At3g49350	0,64	0,74	1,03	1,33	1,27	0,90	1,13	1,60	RabGAP/TBC domain-containing protein
252282_at	At3g49360	1,23	1,16	1,37	1,37	1,44	0,83	0,96	0,86	glucosamine/galactosamine-6-phosphate isomerase family protein
252311_at	At3g49370	0,77	0,99	1,05	0,83	0,89	0,83	1,06	0,89	calcium-dependent protein kinase, putative / CDPK, putative
252312_at	At3g49380	0,95	0,98	0,86	1,11	1,08	0,99	0,91	1,06	calmodulin-binding family protein
252313_at	At3g49390	1,02	0,90	1,11	1,04	0,91	1,03	0,98	0,88	RNA-binding protein, putative
252314_at	At3g49400	1,11	1,40	1,26	0,72	0,82	1,19	1,38	1,08	transducin family protein / WD-40 repeat family protein
252271_s_at	At3g49420	1,05	0,93	0,90	1,03	0,99	1,01	0,90	1,03	Got1-like family protein
252256_at	At3g49430	1,14	1,13	1,14	1,02	0,99	0,99	1,05	1,06	pre-mRNA splicing factor, putative
252257_at	At3g49440	0,96	0,88	1,06	1,03	1,06	1,26	1,03	1,02	F-box family protein-related
252258_at	At3g49450	0,96	1,01	1,04	0,93	1,07	1,04	0,97	1,14	F-box family protein
252259_at	At3g49460	1,04	1,11	0,93	1,02	0,97	0,97	0,98	0,91	60S acidic ribosomal protein-related
252277_at	At3g49470	1,06	1,09	1,32	0,89	0,85	1,15	1,07	1,31	nascent polypeptide-associated complex (NAC) domain-containing protein
252260_at	At3g49480	0,96	1,00	1,04	0,99	1,00	0,95	0,99	0,90	hypothetical protein

252276_at	At3g49490	0,99	0,90	1,07	0,87	0,92	1,03	1,13	1,11	expressed protein
252261_at	At3g49500	0,69	0,71	0,75	0,88	0,80	0,71	0,69	0,73	RNA-dependent RNA polymerase (SDE1)
252262_at	At3g49510	0,95	1,01	1,03	1,03	1,13	0,96	0,97	1,12	F-box family protein
252263_at	At3g49520	0,92	1,10	1,01	0,97	0,96	1,06	1,08	0,81	F-box family protein
252278_at	At3g49530	0,87	1,48	1,28	0,88	0,93	1,21	1,50	1,59	no apical meristem (NAM) family protein
252270_at	At3g49560	1,12	1,04	1,08	0,88	0,96	1,06	1,02	1,04	mitochondrial import inner membrane translocase subunit Tim17/Tim22/Tim
252269_at	At3g49580	1,00	1,10	0,98	0,87	0,99	1,06	1,04	1,20	expressed protein
252264_at	At3g49590	0,97	1,03	1,12	0,66	0,89	0,97	0,91	1,15	expressed protein
252275_at	At3g49600	0,88	0,85	1,17	0,79	0,72	0,96	1,09	1,02	ubiquitin-specific protease 26 (UBP26)
252265_at	At3g49620	0,54	0,54	1,18	0,66	0,63	0,64	0,75	0,79	2-oxoacid-dependent oxidase, putative (DIN11)
252266_at	At3g49630	0,83	0,98	1,02	1,06	1,17	0,95	1,00	0,68	2-oxoacid-dependent oxidase, putative
252267_at	At3g49640	1,46	1,04	1,11	1,14	1,02	1,01	1,01	1,41	nitrogen regulation family protein
252268_at	At3g49650	1,17	1,24	1,04	0,86	1,05	0,96	0,88	0,98	kinesin motor protein-related
252273_at	At3g49660	1,39	1,55	1,17	1,08	1,24	1,16	1,03	1,00	transducin family protein / WD-40 repeat family protein
252272_at	At3g49670	0,78	0,76	0,66	0,67	0,66	0,86	0,76	0,66	leucine-rich repeat transmembrane protein kinase, putative
252274_at	At3g49680	1,18	1,21	1,19	0,94	0,85	0,97	1,06	0,94	branched-chain amino acid aminotransferase 3 / branched-chain amino aci
252233_at	At3g49690	0,80	0,95	0,96	0,94	1,11	0,97	1,07	1,00	myb family transcription factor
252279_at	At3g49700	1,01	1,05	0,97	0,85	0,89	0,97	0,92	0,86	1-aminocyclopropane-1-carboxylate synthase, putative / ACC synthase, pu
252245_at	At3g49710	1,11	1,04	0,95	0,97	0,71	0,92	0,82	1,24	pentatricopeptide (PPR) repeat-containing protein
252231_at	At3g49720	0,71	0,67	0,75	1,12	1,22	0,96	0,87	0,91	expressed protein
252246_at	At3g49730	1,21	1,17	1,25	0,80	1,01	1,10	1,18	1,04	pentatricopeptide (PPR) repeat-containing protein
252247_at	At3g49740	1,01	0,90	0,92	1,01	1,02	1,07	1,14	0,77	pentatricopeptide (PPR) repeat-containing protein
252248_at	At3g49750	1,00	1,16	1,01	1,13	1,17	0,98	0,90	0,88	leucine-rich repeat family protein
252232_at	At3g49760	1,03	1,01	0,95	0,97	0,96	1,03	0,88	0,97	bZIP transcription factor family protein
252249_at	At3g49770	1,24	1,14	1,03	0,96	1,14	1,05	0,95	0,94	hypothetical protein
252234_at	At3g49780	1,02	0,90	1,06	0,87	0,93	1,44	1,08	1,27	phytosulfokines 3 (PSK3)
252250_at	At3g49790	1,27	1,09	1,11	0,81	0,83	0,78	0,99	0,96	expressed protein
252230_at	At3g49810	0,70	0,73	0,65	0,73	0,81	0,88	0,99	0,89	U-box domain-containing protein
252251_at	At3g49820	0,86	0,95	0,97	0,97	1,06	0,91	0,99	1,01	hypothetical protein
252221_at	At3g49830	0,93	1,04	0,93	0,93	1,05	0,90	1,07	1,24	DNA helicase-related
252222_at	At3g49840	1,26	1,26	1,06	0,95	1,28	0,97	0,98	0,90	proline-rich family protein
252223_at	At3g49850	1,06	1,02	1,11	0,79	0,80	1,01	0,98	0,73	myb family transcription factor
252224_at	At3g49860	1,02	0,84	0,91	1,45	1,50	1,02	1,00	1,10	ADP-ribosylation factor, putative
252225_at	At3g49870	0,93	1,05	0,94	1,06	0,97	0,89	0,96	0,96	ADP-ribosylation factor, putative
252226_at	At3g49880	0,89	1,08	1,13	1,07	1,02	1,00	0,88	1,07	glycosyl hydrolase family protein 43
252229_at	At3g49890	1,09	0,87	1,06	1,01	1,07	0,94	1,00	0,91	expressed protein
252227_at	At3g49900	0,96	0,87	1,01	0,98	0,94	1,06	1,00	0,86	BTB/POZ domain-containing protein
252235_at	At3g49910	1,47	1,26	1,20	1,31	1,29	1,01	0,97	0,96	60S ribosomal protein L26 (RPL26A)
252228_at	At3g49920	0,99	0,84	0,91	1,02	0,96	0,98	0,85	1,11	porin, putative
252236_at	At3g49930	0,93	0,84	0,86	0,98	1,17	0,94	0,88	0,99	zinc finger (C2H2 type) family protein
252220_at	At3g49940	1,62	1,36	1,05	1,13	1,37	0,90	1,25	1,30	LOB domain protein 38 / lateral organ boundaries domain protein 38 (LBD3
252237_at	At3g49950	0,97	1,04	0,90	1,07	0,99	1,04	0,96	0,75	scarecrow transcription factor family protein
252238_at	At3g49960	1,27	1,20	1,02	1,33	1,16	1,05	1,01	1,00	peroxidase, putative
252239_at	At3g49990	1,02	0,90	1,10	1,01	0,94	1,02	0,88	1,00	expressed protein
252192_at	At3g50000	0,87	0,82	0,89	1,22	1,24	0,93	0,89	0,84	casein kinase II alpha chain 2
252240_at	At3g50010	1,19	0,92	0,88	0,89	1,13	1,00	1,22	0,95	DC1 domain-containing protein

252241_at	At3g50050	1,56	1,17	1,06	0,75	1,20	1,19	1,12	1,09 aspartyl protease family protein
252193_at	At3g50060	1,21	1,24	0,91	0,74	0,62	1,17	1,58	1,15 myb family transcription factor
252189_at	At3g50070	1,06	1,06	1,22	1,19	1,17	0,93	0,90	0,95 cyclin family protein
252242_at	At3g50080	0,91	0,93	0,98	1,23	0,96	0,93	0,96	1,12 F-box family protein (FBL16)
252194_at	At3g50110	0,91	1,10	0,94	0,98	1,19	0,88	0,93	0,96 phosphatase-related
252243_at	At3g50120	0,95	1,02	1,08	0,97	1,05	0,85	1,02	0,94 expressed protein
252244_at	At3g50130	0,97	0,94	0,99	0,97	1,08	0,99	1,00	0,88 expressed protein
252217_at	At3g50140	1,01	0,95	0,94	1,07	0,80	0,91	0,87	0,82 expressed protein
252218_at	At3g50150	0,96	1,01	0,93	0,91	1,01	0,90	1,00	0,95 expressed protein
252219_at	At3g50160	0,99	0,96	1,03	1,06	1,05	1,01	1,06	1,01 hypothetical protein
252190_at	At3g50170	0,97	1,02	1,04	1,00	1,05	0,94	0,91	1,04 hypothetical protein
252191_at	At3g50180	0,95	0,90	1,11	0,93	0,97	1,02	0,98	0,91 hypothetical protein
252195_at	At3g50190	0,91	1,00	1,02	1,04	0,95	1,02	0,99	1,02 expressed protein
252196_at	At3g50200	0,92	0,99	1,07	0,97	1,12	0,99	1,07	1,26 expressed protein
252213_at	At3g50210	1,03	0,86	0,83	0,85	0,93	0,83	0,98	1,00 2-oxoacid-dependent oxidase, putative
252211_at	At3g50220	0,93	1,09	1,09	0,96	1,18	0,95	0,93	0,90 expressed protein
252197_at	At3g50230	1,04	0,98	0,94	1,12	1,03	0,97	0,93	0,88 leucine-rich repeat transmembrane protein kinase, putative
252215_at	At3g50240	0,78	0,84	1,92	0,73	0,61	1,46	1,86	3,37 kinesin motor protein-related
252198_x_at	At3g50250	0,91	0,91	0,92	1,07	1,00	0,92	1,12	1,20 hypothetical protein
252214_at	At3g50260	0,81	1,29	1,21	1,07	0,95	1,21	1,50	2,24 AP2 domain-containing transcription factor, putative
252199_at	At3g50270	0,61	0,50	0,82	0,90	1,05	0,69	0,74	0,71 transferase family protein
252200_at	At3g50280	0,73	0,81	0,92	1,07	0,94	0,72	1,08	1,30 transferase family protein
252201_at	At3g50290	1,04	0,91	1,07	1,06	1,08	1,01	1,13	0,88 ---
252202_at	At3g50300	0,98	1,01	1,03	1,22	1,18	1,02	1,13	0,96 transferase family protein
252212_at	At3g50310	0,93	1,09	0,97	0,91	1,02	0,91	0,85	0,87 protein kinase-related
252203_at	At3g50320	0,97	1,00	0,93	1,07	0,91	1,00	0,92	0,98 hypothetical protein
252204_at	At3g50340	0,75	0,97	0,89	1,04	1,16	1,09	0,91	0,87 expressed protein
252205_at	At3g50350	1,05	0,82	0,92	1,25	1,08	0,95	0,85	1,30 expressed protein
252206_at	At3g50360	1,01	0,74	0,76	1,09	1,17	1,03	1,09	1,06 caltractin / centrin
252207_at	At3g50370	0,93	0,78	0,74	0,78	0,68	0,93	0,89	0,92 expressed protein
252208_at	At3g50380	1,29	0,84	1,10	0,87	0,78	1,40	1,32	1,31 expressed protein
252209_at	At3g50400	1,17	1,31	1,10	0,96	0,93	1,11	1,10	0,85 GDSL-motif lipase/hydrolase family protein
252210_at	At3g50410	0,88	1,24	1,07	1,09	1,30	0,94	0,92	0,90 Dof-type zinc finger domain-containing protein
252216_at	At3g50420	1,06	0,94	0,96	0,95	0,91	1,01	1,02	0,96 pentatricopeptide (PPR) repeat-containing protein
252157_at	At3g50430	0,87	1,11	1,20	0,90	0,95	0,92	1,00	1,09 expressed protein
252168_at	At3g50440	0,63	0,82	1,21	1,14	1,65	0,61	0,64	0,64 hydrolase, alpha/beta fold family protein
252170_at	At3g50480	1,03	1,18	1,04	1,05	1,19	1,17	1,07	1,26 broad-spectrum mildew resistance RPW8 family protein
252166_at	At3g50500	0,69	0,64	0,70	0,91	0,90	0,87	0,80	0,88 protein kinase, putative
252169_at	At3g50520	1,07	0,87	1,00	1,09	0,98	1,11	0,97	1,09 phosphoglycerate/bisphosphoglycerate mutase family protein
252158_at	At3g50530	1,15	1,18	1,13	0,89	0,93	1,07	1,08	1,25 calcium-dependent protein kinase, putative / CDPK, putative
252159_at	At3g50540	0,99	1,03	0,99	0,89	1,07	0,96	1,10	1,08 hypothetical protein
252165_at	At3g50550	1,20	1,00	0,80	1,01	1,36	1,06	1,05	0,73 expressed protein
252167_at	At3g50560	1,31	1,51	1,42	1,37	1,29	0,86	1,07	1,26 short-chain dehydrogenase/reductase (SDR) family protein
252160_at	At3g50570	0,97	1,00	1,05	1,20	1,01	1,03	0,79	1,02 hydroxyproline-rich glycoprotein family protein
252161_at	At3g50580	1,18	1,17	0,95	1,01	0,88	1,00	1,01	1,04 proline-rich family protein
252162_at	At3g50590	0,79	0,90	0,79	1,32	0,99	0,83	0,88	0,73 transducin family protein / WD-40 repeat family protein

252171_at	At3g50600	0,84	0,78	0,78	1,03	1,10	0,85	0,89	0,86	transducin family protein / WD-40 repeat family protein
252163_at	At3g50610	1,00	1,06	0,95	1,02	1,18	1,03	1,02	1,15	hypothetical protein
252164_at	At3g50620	1,06	0,92	0,91	1,05	1,00	0,96	0,96	0,90	nodulation protein-related
252180_at	At3g50630	1,00	1,04	1,06	0,97	0,94	1,13	1,09	1,02	kip-related protein 2 (KRP2) / cyclin-dependent kinase inhibitor 2 (ICK2) / c
252172_at	At3g50640	1,10	1,30	1,02	1,19	1,65	1,19	1,00	0,93	expressed protein
252173_at	At3g50650	0,80	0,78	1,03	1,30	1,15	0,74	0,71	1,01	scarecrow-like transcription factor 7 (SCL7)
252184_at	At3g50660	0,99	1,20	1,12	1,03	0,64	0,87	0,91	0,90	steroid 22-alpha-hydroxylase (CYP90B1) (DWF4)
252182_at	At3g50670	1,30	1,09	1,03	0,92	0,91	1,00	1,11	1,31	U1 small nuclear ribonucleoprotein 70 (U1-70k)
252181_at	At3g50685	0,72	0,74	0,85	0,96	0,87	0,91	0,76	0,77	expressed protein
252174_at	At3g50690	1,09	1,14	1,02	0,87	0,89	0,89	1,02	1,23	leucine-rich repeat family protein
252175_at	At3g50700	1,05	1,11	0,82	0,54	0,57	0,87	0,98	1,02	zinc finger (C2H2 type) family protein
252176_at	At3g50720	0,96	1,09	1,10	0,89	0,80	0,90	0,97	0,86	protein kinase, putative
252177_at	At3g50730	0,97	1,22	1,18	0,89	0,74	1,04	0,91	0,93	protein kinase, putative
252183_at	At3g50740	0,90	1,02	0,88	0,58	0,50	0,82	0,66	0,53	UDP-glucoronosyl/UDP-glucosyl transferase family protein
252178_at	At3g50750	0,70	0,88	0,94	0,68	0,82	0,69	0,83	0,95	brassinosteroid signalling positive regulator-related
252179_at	At3g50760	1,01	0,94	0,82	1,77	1,42	0,91	0,77	0,94	glycosyl transferase family 8 protein
252136_at	At3g50770	1,70	1,69	1,55	0,46	0,66	1,39	1,11	1,18	calmodulin-related protein, putative
252185_at	At3g50780	1,00	0,82	0,76	1,04	1,00	1,00	0,81	0,85	expressed protein
252132_at	At3g50790	0,89	0,80	0,85	0,86	0,99	0,96	0,99	0,96	late embryogenesis abundant protein, putative / LEA protein, putative
252186_at	At3g50810	0,94	1,11	0,96	0,93	0,98	1,10	1,11	0,69	integral membrane protein, putative
252130_at	At3g50820	1,11	1,13	1,15	0,99	1,13	1,21	1,08	1,15	oxygen-evolving enhancer protein, chloroplast, putative / 33 kDa subunit of
252135_at	At3g50830	0,93	0,80	0,72	1,10	0,80	1,05	0,84	0,86	stress-responsive protein, putative
252187_at	At3g50850	0,86	1,04	1,08	1,03	1,15	1,08	0,97	1,01	expressed protein
252188_at	At3g50860	1,02	0,93	0,75	1,22	1,42	0,86	0,92	0,79	clathrin adaptor complex small chain family protein
252128_at	At3g50870	0,99	1,04	1,01	1,05	0,85	0,93	0,94	0,97	zinc finger (GATA type) family protein
252154_at	At3g50880	0,94	0,84	1,01	1,25	1,40	1,02	0,92	0,96	HhH-GPD base excision DNA repair family protein
252129_at	At3g50890	1,07	1,08	1,03	0,86	1,10	1,08	1,04	0,92	zinc finger homeobox family protein / ZF-HD homeobox family protein
252133_at	At3g50900	0,83	0,88	0,86	0,90	0,80	0,73	0,70	1,07	expressed protein
252134_at	At3g50910	0,88	0,74	0,93	0,98	1,15	1,04	1,08	1,25	expressed protein
252155_at	At3g50920	1,08	1,02	1,19	1,05	0,87	0,92	1,04	1,09	phosphatidic acid phosphatase-related / PAP2-related
252131_at	At3g50930	1,79	2,33	1,24	1,31	2,05	1,48	1,57	1,32	AAA-type ATPase family protein
252156_at	At3g50940	1,41	1,55	1,18	1,00	1,02	1,24	1,01	1,05	AAA-type ATPase family protein
252126_at	At3g50950	0,82	1,02	1,64	0,92	0,90	1,22	1,35	1,63	disease resistance protein (CC-NBS-LRR class), putative
252127_at	At3g50960	1,14	1,12	1,02	0,90	0,72	1,03	0,96	0,91	expressed protein
252102_at	At3g50970	0,96	1,08	1,52	1,22	1,03	1,30	1,39	1,34	dehydrin xero2 (XERO2) / low-temperature-induced protein LTI30 (LTI30)
252137_at	At3g50980	0,59	0,61	0,60	1,03	0,97	0,98	0,98	1,02	dehydrin, putative
252138_at	At3g50990	1,05	1,19	1,02	1,00	0,96	0,91	1,09	1,13	peroxidase, putative
252095_at	At3g51000	0,74	1,02	0,97	1,24	1,35	0,83	0,96	1,07	epoxide hydrolase, putative
252124_at	At3g51010	1,42	1,33	1,24	1,20	1,19	0,97	0,91	0,91	expressed protein
252119_at	At3g51030	1,89	1,38	1,24	1,97	1,90	0,86	0,97	0,97	thioredoxin H-type 1 (TRX-H-1)
252125_at	At3g51040	0,89	0,93	0,99	0,85	1,23	0,99	0,98	0,98	expressed protein
252139_at	At3g51050	0,89	0,83	0,66	1,26	1,13	0,98	0,83	0,86	FG-GAP repeat-containing protein
252140_at	At3g51070	0,93	1,15	0,99	0,99	0,99	0,94	0,90	0,97	dehydration-responsive protein-related
252097_at	At3g51090	1,25	1,22	1,27	1,84	2,38	0,94	1,08	1,06	expressed protein
252141_at	At3g51100	1,15	1,07	1,10	1,26	1,36	1,08	0,95	1,18	expressed protein
252100_at	At3g51110	0,70	0,86	1,01	0,79	0,95	1,01	0,88	0,78	crooked neck protein, putative / cell cycle protein, putative

252142_at	At3g51120	0,89	0,90	1,03	0,94	0,88	1,10	1,12	1,13	zinc finger (CCCH-type) family protein
252120_at	At3g51130	0,96	0,94	1,04	1,06	1,01	1,05	1,00	1,28	expressed protein
252122_at	At3g51140	1,10	1,02	1,00	1,04	0,87	0,99	0,92	0,96	expressed protein
252143_at	At3g51150	0,92	0,93	1,01	0,96	0,83	1,19	1,22	1,16	kinesin motor family protein
252121_at	At3g51160	0,80	0,64	0,71	1,19	1,14	0,92	0,78	0,84	GDP-D-mannose-4,6-dehydratase (MUR1)
252096_at	At3g51180	0,77	0,72	0,97	1,08	1,05	0,93	0,80	0,94	zinc finger (CCCH-type) family protein
252144_at	At3g51190	0,95	1,05	1,13	1,06	1,09	0,95	0,96	0,91	60S ribosomal protein L8 (RPL8B)
252145_at	At3g51200	0,84	1,02	1,11	1,05	0,91	0,90	0,92	0,90	auxin-responsive family protein
252146_at	At3g51210	0,95	1,07	0,98	1,03	1,19	0,96	1,14	0,98	flavonol 3-sulfotransferase-related
252123_at	At3g51240	1,32	0,94	0,88	1,41	1,31	1,16	1,27	1,25	naringenin 3-dioxygenase / flavanone 3-hydroxylase (F3H)
252099_at	At3g51250	1,00	1,10	1,06	1,31	1,20	1,04	1,06	1,09	senescence/dehydration-associated protein-related
252094_at	At3g51260	1,18	1,12	1,13	1,21	1,13	1,01	0,88	0,93	20S proteasome alpha subunit D (PAD1)
252147_at	At3g51270	0,99	0,90	1,14	0,91	1,11	0,99	0,94	1,03	RIO1 family protein
252148_at	At3g51280	1,37	1,26	0,94	1,33	1,08	1,00	0,96	0,77	male sterility MS5, putative
252149_at	At3g51290	0,85	0,85	0,98	0,69	0,87	0,94	0,94	0,95	proline-rich family protein
252101_at	At3g51300	1,04	0,92	1,00	1,01	0,82	0,96	0,93	1,22	Rac-like GTP-binding protein (ARAC11) / Rho-like GTP-binding protein (RC
252150_at	At3g51310	0,92	0,89	0,84	1,10	1,06	0,94	1,01	0,96	vacuolar protein sorting-associated protein 35 family protein / VPS35 family
252151_at	At3g51320	1,06	1,01	1,11	1,05	1,05	0,92	0,99	0,82	pentatricopeptide (PPR) repeat-containing protein
252098_at	At3g51330	1,25	1,12	0,92	1,11	1,25	1,19	1,35	1,21	aspartyl protease family protein
252152_at	At3g51350	0,96	1,03	1,12	0,98	0,98	1,17	1,00	1,05	aspartyl protease family protein
252153_at	At3g51360	0,99	0,97	1,03	1,00	1,02	1,07	0,91	0,84	aspartyl protease family protein
252067_at	At3g51370	0,87	0,77	0,93	0,78	0,86	1,01	0,96	0,95	protein phosphatase 2C, putative / PP2C, putative
252091_at	At3g51390	0,97	0,97	1,03	0,90	0,87	0,97	0,93	0,90	zinc finger (DHHC type) family protein
252118_at	At3g51400	0,73	1,03	0,96	1,02	1,00	0,81	0,64	0,78	expressed protein
252103_at	At3g51410	1,07	1,07	1,00	1,13	1,14	0,99	0,98	0,98	expressed protein
252092_at	At3g51420	0,88	0,89	1,00	0,82	0,70	1,02	1,02	1,05	strictosidine synthase family protein
252117_at	At3g51430	0,55	0,58	0,64	1,00	1,19	0,82	0,72	0,63	strictosidine synthase, putative (YLS2)
252068_at	At3g51440	0,62	0,52	0,56	1,26	1,34	0,73	0,65	0,65	strictosidine synthase family protein
252114_at	At3g51450	0,46	0,41	0,40	0,90	0,94	0,80	0,83	0,81	strictosidine synthase family protein
252104_at	At3g51460	1,04	1,04	0,95	0,94	1,12	0,90	1,06	0,88	phosphoinositide phosphatase family protein
252105_at	At3g51470	0,70	0,81	0,91	1,15	1,03	1,03	0,78	0,75	protein phosphatase 2C, putative / PP2C, putative
252106_at	At3g51480	0,97	1,06	1,06	1,02	1,11	0,94	0,96	1,05	glutamate receptor family protein (GLR3.6)
252107_at	At3g51490	0,77	1,02	0,86	1,01	1,04	0,98	0,97	1,26	sugar transporter family protein
252093_at	At3g51500	1,45	1,48	1,38	0,92	1,01	1,29	1,21	1,37	expressed protein
252116_at	At3g51510	0,96	0,98	1,09	0,84	0,94	1,04	0,94	0,99	expressed protein
252064_at	At3g51520	1,06	1,29	1,15	0,94	0,97	1,13	1,11	1,05	diacylglycerol acyltransferase family
252108_at	At3g51530	1,45	1,40	1,40	0,78	0,87	0,99	1,42	1,20	F-box family protein
252109_at	At3g51540	1,03	1,04	1,03	1,12	1,23	0,98	0,97	1,24	expressed protein
252066_at	At3g51550	0,87	0,80	0,90	0,94	0,91	1,05	0,93	0,97	protein kinase family protein
252110_at	At3g51560	0,97	0,95	1,08	0,92	0,98	0,96	1,01	0,95	disease resistance protein (TIR-NBS-LRR class), putative
252111_at	At3g51570	0,87	1,00	1,04	0,90	0,98	1,15	1,05	1,05	disease resistance protein (TIR-NBS-LRR class), putative
252112_at	At3g51580	0,95	0,78	0,78	0,99	1,04	0,98	0,94	1,07	expressed protein
252063_at	At3g51590	0,99	1,11	1,03	1,05	1,19	1,09	0,98	0,86	lipid transfer protein, putative
252115_at	At3g51600	1,27	1,06	1,11	1,94	1,98	0,84	0,73	0,73	nonspecific lipid transfer protein 5 (LTP5)
252065_at	At3g51610	1,11	1,06	0,94	0,94	1,02	0,99	0,88	0,89	expressed protein
252113_at	At3g51620	0,98	0,82	0,78	1,17	1,33	0,80	0,84	0,77	expressed protein

252079_at	At3g51630	0,89	0,85	1,01	1,16	1,20	1,02	0,93	1,05	protein kinase family protein
252069_s_at	At3g51640	0,93	0,86	0,93	0,79	1,09	0,97	1,01	0,98	expressed protein
252076_at	At3g51660	0,86	0,72	0,82	0,70	0,75	1,19	1,13	0,92	macrophage migration inhibitory factor family protein / MIF family protein
252080_at	At3g51670	1,33	1,39	1,26	1,30	1,13	1,29	1,27	1,27	SEC14 cytosolic factor family protein / phosphoglyceride transfer family pro
252070_at	At3g51680	1,15	1,01	0,86	1,20	1,30	1,14	1,19	1,03	short-chain dehydrogenase/reductase (SDR) family protein
252071_at	At3g51690	0,96	0,92	0,96	1,00	0,94	0,91	1,02	1,02	hypothetical protein
252074_at	At3g51700	0,95	1,05	1,02	1,00	1,02	1,08	1,09	0,99	expressed protein
252072_at	At3g51710	1,05	1,08	0,93	0,85	0,94	1,03	1,11	0,96	curculin-like (mannose-binding) lectin family protein / PAN domain-containir
252077_at	At3g51720	1,55	1,19	1,20	1,33	1,10	1,04	0,87	1,00	expressed protein
252075_at	At3g51730	1,17	1,43	1,36	1,14	0,99	1,14	1,19	1,27	saposin B domain-containing protein
252078_at	At3g51740	1,04	0,96	0,99	1,12	1,45	0,90	0,97	0,91	leucine-rich repeat transmembrane protein kinase, putative
252073_at	At3g51750	1,10	0,96	0,75	0,94	0,93	1,21	1,00	1,12	expressed protein
246297_at	At3g51760	0,95	1,27	1,29	0,97	1,26	0,99	0,93	0,84	hypothetical protein
246298_at	At3g51770	0,78	0,78	0,79	0,87	1,41	0,84	0,85	0,78	tetratricopeptide repeat (TPR)-containing protein
246306_at	At3g51780	1,09	0,93	1,08	0,93	1,02	1,03	1,06	0,94	BAG domain-containing protein
246309_at	At3g51790	0,84	0,79	0,79	1,40	1,15	0,92	0,85	0,81	cytochrome c biogenesis protein CcmE family
246307_at	At3g51800	1,17	1,07	1,11	0,96	0,99	1,12	0,99	0,97	metallopeptidase M24 family protein
246299_at	At3g51810	0,84	0,88	0,90	0,89	1,11	1,08	1,09	0,96	Em-like protein GEA1 (EM1)
246308_at	At3g51820	0,86	0,82	0,83	0,61	0,70	1,05	1,09	1,15	chlorophyll synthetase, putative
246300_at	At3g51830	0,83	0,98	0,95	0,80	0,84	1,00	1,20	1,21	phosphoinositide phosphatase family protein
246304_at	At3g51840	0,81	0,83	0,97	0,89	0,98	0,91	0,95	0,99	short-chain acyl-CoA oxidase
246301_at	At3g51850	0,91	0,72	0,92	0,85	0,93	0,95	0,97	1,02	calcium-dependent protein kinase, putative / CDPK, putative
246302_at	At3g51860	0,45	0,60	0,79	1,08	0,68	1,14	1,69	1,58	cation exchanger, putative (CAX3)
246303_at	At3g51870	0,89	0,67	0,75	0,94	0,63	1,05	1,05	0,98	mitochondrial substrate carrier family protein
246311_at	At3g51880	1,08	1,04	1,04	0,90	1,06	1,23	1,32	1,44	high mobility group protein alpha (HMGalpha) / HMG protein alpha
246305_at	At3g51890	0,78	0,80	0,96	0,88	0,97	1,02	0,94	1,14	expressed protein
246310_at	At3g51895	1,19	1,15	1,19	1,84	1,57	1,01	1,12	0,95	sulfate transporter (ST1)
252081_at	At3g51910	1,25	0,86	0,82	1,19	1,07	1,07	1,34	1,08	heat shock transcription factor family protein
252037_at	At3g51920	0,87	0,81	1,16	1,15	0,98	1,06	0,93	0,98	calmodulin-9 (CAM9)
252082_at	At3g51940	1,07	1,08	1,01	0,89	1,05	0,93	1,04	0,99	expressed protein
252033_at	At3g51950	1,11	1,57	1,39	0,72	0,68	1,13	1,50	1,61	zinc finger (CCCH-type) family protein / RNA recognition motif (RRM)-conta
252083_at	At3g51960	0,92	0,91	0,96	0,87	0,80	1,06	0,93	1,12	bZIP family transcription factor
252084_at	At3g51970	0,54	0,69	0,92	1,01	1,14	0,88	0,83	0,78	long-chain-alcohol O-fatty-acyltransferase family protein / wax synthase fan
252042_at	At3g51990	0,96	0,76	0,85	1,04	1,19	0,97	1,00	0,95	protein kinase family protein
252085_s_at	At3g52000	0,98	0,97	1,04	1,05	0,99	0,99	1,03	1,10	serine carboxypeptidase S10 family protein
252086_at	At3g52030	1,20	1,15	1,23	0,86	0,83	1,11	1,16	1,18	F-box family protein / WD-40 repeat family protein
252034_at	At3g52040	1,25	0,94	0,96	1,12	1,21	1,22	1,01	0,85	expressed protein
252040_at	At3g52060	0,99	1,14	0,98	0,61	0,75	1,03	0,86	0,89	expressed protein
252036_at	At3g52070	0,81	1,11	1,00	1,22	0,81	0,74	0,84	0,91	expressed protein
252087_at	At3g52080	1,00	1,02	1,09	0,91	0,96	0,96	0,98	0,94	cation/hydrogen exchanger, putative (CHX28)
252041_at	At3g52090	1,36	1,24	1,04	1,25	1,29	0,99	0,94	0,95	DNA-directed RNA polymerase II 13.6 kDa subunit (RPB13.6)
252088_at	At3g52100	0,89	1,00	0,89	0,88	0,87	0,77	0,91	0,85	expressed protein
252089_at	At3g52110	1,34	1,21	1,26	1,15	1,20	0,88	0,97	0,85	expressed protein
252038_at	At3g52120	0,75	0,62	0,63	0,96	0,96	0,74	0,80	0,69	SWAP (Suppressor-of-White-APricot)/surp domain-containing protein / D11
252090_at	At3g52130	1,01	0,97	0,99	1,03	1,12	1,05	1,04	1,05	protease inhibitor/seed storage/lipid transfer protein (LTP) family protein
252031_at	At3g52140	1,27	1,12	1,16	0,83	0,83	0,99	0,94	1,08	tetratricopeptide repeat (TPR)-containing protein

252032_at	At3g52150	0,98	0,90	0,99	0,85	0,86	1,02	1,01	1,02	RNA recognition motif (RRM)-containing protein
252039_at	At3g52155	1,06	0,98	0,90	1,02	0,88	1,09	0,90	0,94	expressed protein
252035_at	At3g52160	0,93	1,03	1,02	1,02	1,05	1,05	0,95	1,24	beta-ketoacyl-CoA synthase family protein
256675_at	At3g52170	0,92	0,82	0,93	0,80	0,63	0,85	0,89	0,80	expressed protein
256676_at	At3g52180	0,48	0,46	0,55	0,61	0,71	0,91	1,04	1,05	protein tyrosine phosphatase/kinase interaction sequence protein (PTPKIS1
256677_at	At3g52190	0,95	1,12	0,98	1,12	1,00	1,06	0,98	0,89	transducin family protein / WD-40 repeat family protein
256682_at	At3g52200	1,20	0,93	0,91	1,16	1,06	1,07	1,07	1,06	dihydrolipoamide S-acetyltransferase, putative
256726_at	At3g52210	0,98	0,99	1,10	1,06	1,21	1,03	0,97	0,87	mRNA capping enzyme family protein
256683_at	At3g52220	1,24	1,25	1,69	1,03	1,15	1,16	1,06	1,18	expressed protein
256680_at	At3g52230	1,35	1,31	1,37	1,01	1,24	1,14	1,02	1,10	expressed protein
256727_at	At3g52240	0,75	0,79	0,97	0,77	1,10	0,93	0,86	0,93	expressed protein
256700_at	At3g52260	0,82	0,91	0,92	0,69	0,80	0,86	0,97	1,06	pseudouridine synthase family protein
256701_at	At3g52270	0,95	1,00	0,92	0,95	1,04	0,99	1,05	1,17	hypothetical protein
256671_at	At3g52290	1,03	1,07	1,01	1,21	1,02	0,98	1,09	1,07	calmodulin-binding family protein
256679_at	At3g52300	1,27	1,08	0,98	1,12	1,00	1,00	1,01	0,91	ATP synthase D chain-related
256672_at	At3g52310	1,34	0,97	1,05	1,10	1,20	0,94	1,19	1,10	ABC transporter family protein
256681_at	At3g52340	0,87	0,76	0,71	1,14	1,12	1,07	1,08	1,05	sucrose-phosphatase 2 (SPP2)
256674_at	At3g52360	1,33	1,40	1,06	1,34	1,24	1,29	1,21	0,99	expressed protein
256673_at	At3g52370	0,92	0,88	0,77	1,36	1,17	0,84	0,73	0,84	beta-Ig-H3 domain-containing protein / fasciclin domain-containing protein
256678_at	At3g52380	0,79	0,63	0,61	0,61	0,57	1,06	0,92	0,84	33 kDa ribonucleoprotein, chloroplast, putative / RNA-binding protein cp33,
252043_at	At3g52390	0,96	0,92	0,86	0,92	0,91	1,25	1,09	1,07	tatD-related deoxyribonuclease family protein
252053_at	At3g52400	0,86	1,03	1,60	0,97	0,86	1,22	1,40	1,59	syntaxin, putative (SYP122)
252044_at	At3g52420	1,34	1,17	1,27	1,32	1,48	0,97	0,85	0,75	outer envelope membrane protein, putative
252060_at	At3g52430	1,05	1,19	2,15	1,22	1,23	1,15	1,26	1,47	phytoalexin-deficient 4 protein (PAD4)
252045_at	At3g52450	0,88	0,95	0,91	0,92	0,96	1,25	1,27	1,11	U-box domain-containing protein
252046_at	At3g52460	1,00	0,97	0,96	1,02	1,04	0,95	0,97	1,10	hydroxyproline-rich glycoprotein family protein
252058_at	At3g52470	0,80	0,80	0,71	1,02	1,34	0,98	0,88	0,93	harpin-induced family protein / HIN1 family protein / harpin-responsive famil
252057_at	At3g52480	0,96	1,16	1,05	0,86	0,89	0,86	0,78	0,77	expressed protein
252047_at	At3g52490	0,89	1,13	1,12	0,79	0,87	0,86	1,24	0,97	heat shock protein-related
252048_at	At3g52500	0,87	0,70	0,71	1,01	1,19	0,89	0,69	0,61	aspartyl protease family protein
252049_at	At3g52510	1,03	1,14	1,01	0,91	1,07	1,14	0,96	0,98	F-box family protein-related
252054_at	At3g52540	1,00	0,96	1,01	0,98	1,06	1,07	1,04	0,91	ovate family protein
252050_at	At3g52550	0,98	1,04	1,08	0,97	1,00	0,93	1,00	1,08	hypothetical protein
252059_at	At3g52560	1,19	1,04	1,03	1,26	1,30	0,82	0,85	0,79	ubiquitin-conjugating enzyme family protein
252051_at	At3g52570	0,96	0,84	0,91	1,07	1,10	0,99	0,92	0,93	expressed protein
252055_at	At3g52580	1,45	1,21	1,05	1,40	1,06	0,97	1,01	0,97	40S ribosomal protein S14 (RPS14C)
252056_at	At3g52590	1,47	1,23	1,15	1,45	1,30	0,98	0,96	0,95	ubiquitin extension protein 1 (UBQ1) / 60S ribosomal protein L40 (RPL40B)
252052_at	At3g52600	0,99	0,91	0,98	0,90	1,04	1,01	1,00	0,82	beta-fructosidase, putative / beta-fructofuranosidase, putative
252008_at	At3g52610	0,94	0,98	0,96	1,05	0,98	0,87	0,93	0,89	expressed protein
252061_at	At3g52620	0,99	1,00	0,95	0,91	1,00	0,99	1,02	0,90	hypothetical protein
252062_at	At3g52640	1,15	1,04	0,95	1,12	1,08	1,07	1,11	0,98	nicastrin-related
252028_at	At3g52650	1,07	0,99	0,92	1,13	1,17	1,08	1,00	0,95	nicastrin-related
252029_s_at	At3g52660	1,08	1,05	1,04	0,87	0,92	0,93	1,06	1,23	RNA recognition motif (RRM)-containing protein
252030_at	At3g52690	0,99	0,98	1,03	0,98	1,02	1,03	1,06	1,06	hypothetical protein
251999_at	At3g52700	0,93	1,11	0,94	0,99	1,01	1,00	0,89	1,06	hypothetical protein
252000_at	At3g52710	0,67	0,80	0,64	1,02	0,78	0,85	0,72	0,86	expressed protein

252011_at	At3g52720	1,53	1,75	2,23	1,58	1,29	1,02	1,10	0,97	carbonic anhydrase family protein
252012_at	At3g52730	1,35	1,14	0,99	1,21	1,22	1,00	0,95	0,90	ubiquinol-cytochrome C reductase UQCRX/QCR9-like family protein
252010_at	At3g52740	1,20	1,11	1,11	1,19	1,05	1,16	1,11	1,32	expressed protein
252001_at	At3g52750	1,07	0,93	0,99	0,76	0,73	1,07	1,05	1,01	chloroplast division protein, putative
252002_at	At3g52760	0,76	0,87	0,74	1,05	0,86	0,83	0,72	0,62	integral membrane Yip1 family protein
252003_at	At3g52770	0,98	1,00	0,94	0,97	0,99	1,00	0,91	0,96	expressed protein
252004_at	At3g52780	1,01	0,94	0,90	0,98	0,79	0,92	1,11	0,94	purple acid phosphatase (PAP20)
252009_at	At3g52800	0,94	1,32	1,46	1,01	1,08	0,89	1,29	1,43	zinc finger (AN1-like) family protein
252005_at	At3g52810	0,93	1,07	1,07	1,06	1,10	0,99	1,00	0,94	purple acid phosphatase (PAP21)
252006_at	At3g52820	0,96	1,02	0,95	1,06	1,16	0,97	1,03	1,10	purple acid phosphatase (PAP22)
252007_at	At3g52830	1,00	1,00	1,01	0,99	1,05	1,07	1,02	0,90	hypothetical protein
251996_at	At3g52840	1,24	1,54	1,66	1,05	0,93	1,05	1,09	1,18	beta-galactosidase, putative / lactase, putative
252027_at	At3g52850	0,88	0,81	0,90	0,92	0,99	1,12	1,01	1,02	vacuolar sorting receptor, putative
252013_at	At3g52860	1,18	1,13	1,11	1,16	1,12	1,07	0,99	1,13	expressed protein
252014_at	At3g52870	0,90	1,12	1,97	1,02	1,14	1,09	1,12	1,30	calmodulin-binding family protein
252024_at	At3g52880	1,16	1,09	1,05	1,18	1,03	1,08	1,10	1,05	monodehydroascorbate reductase, putative
251994_at	At3g52890	1,05	1,04	1,14	0,84	1,13	1,04	0,90	1,07	protein kinase (KIPK)
252025_at	At3g52900	1,61	1,91	1,82	1,29	1,45	1,11	1,20	1,05	expressed protein
252015_at	At3g52910	1,06	0,95	1,13	0,92	0,98	1,03	0,92	0,88	expressed protein
252023_at	At3g52920	1,01	0,98	1,09	1,06	0,96	1,09	1,01	1,12	expressed protein
252022_at	At3g52930	1,17	1,00	0,96	1,38	1,30	0,92	0,91	0,95	fructose-bisphosphate aldolase, putative
251995_at	At3g52940	0,97	0,98	0,76	1,16	0,86	0,89	0,86	0,85	C-14 sterol reductase / delta(14)-sterol reductase / FACKEL (FK)
252016_at	At3g52950	0,83	0,76	0,68	0,88	1,19	0,99	1,03	0,88	CBS domain-containing protein / octicosapeptide/Phox/Bemp1 (PB1) doma
251993_at	At3g52960	0,81	0,74	0,68	0,97	1,00	0,88	0,83	0,82	peroxiredoxin type 2, putative
252017_at	At3g52970	1,14	1,00	1,08	0,95	0,89	1,05	0,97	0,87	cytochrome P450 family protein
252018_at	At3g53000	0,85	0,85	0,89	0,85	1,10	0,80	0,93	1,02	F-box family protein / SKP1 interacting partner 3-related
251997_at	At3g53020	1,44	1,20	1,09	1,38	1,37	1,01	0,99	1,00	60S ribosomal protein L24 (RPL24B)
252026_at	At3g53030	0,91	0,94	1,02	0,89	0,93	0,87	0,93	0,98	protein kinase family protein
252019_at	At3g53040	0,41	0,53	0,80	0,67	0,57	1,14	1,05	0,95	late embryogenesis abundant protein, putative / LEA protein, putative
252020_at	At3g53060	0,91	0,98	0,92	0,97	0,94	1,04	0,99	0,95	E3 ubiquitin ligase SCF complex subunit SKP1/ASK1 (At6), putative
252021_at	At3g53070	1,02	0,99	1,02	1,09	0,96	0,94	1,11	0,98	beta-galactosidase-related
251981_at	At3g53080	1,03	1,18	1,04	0,97	0,88	1,08	0,84	0,74	galactose-binding lectin family protein
251998_at	At3g53090	1,08	1,24	1,19	0,89	0,78	1,00	1,24	1,13	HECT-domain-containing protein / ubiquitin-transferase family protein / IQ c
251968_at	At3g53100	0,84	0,82	0,64	1,78	1,67	0,63	0,73	0,70	GDSL-motif lipase/hydrolase family protein
251989_at	At3g53110	1,07	0,87	0,80	0,90	0,91	1,03	0,93	0,93	DEAD/DEAH box helicase, putative
251969_at	At3g53130	0,82	0,79	0,82	0,83	0,82	1,08	1,02	0,88	cytochrome P450 family protein
251979_at	At3g53140	1,46	1,35	1,75	0,81	0,89	1,68	1,81	1,89	cytochrome P450 family protein
251970_at	At3g53150	1,33	1,46	1,05	1,16	1,00	1,10	1,06	1,10	UDP-glucoronosyl/UDP-glucosyl transferase family protein
251971_at	At3g53160	1,05	0,94	1,15	0,84	0,99	1,21	1,09	1,10	UDP-glucoronosyl/UDP-glucosyl transferase family protein
251972_at	At3g53170	1,09	1,04	1,26	0,77	0,87	1,05	1,17	0,90	pentatricopeptide (PPR) repeat-containing protein
251973_at	At3g53180	0,81	0,71	0,77	1,10	1,02	0,79	0,81	0,81	glutamine synthetase, putative
251982_at	At3g53190	1,27	1,19	1,41	1,61	1,19	0,87	0,84	0,78	pectate lyase family protein
251974_at	At3g53200	1,00	1,02	1,05	1,04	1,06	0,95	0,93	1,04	myb family transcription factor (MYB27)
251983_at	At3g53210	0,85	0,81	0,98	0,93	0,78	0,90	1,14	1,05	nodulin MtN21 family protein
251985_at	At3g53220	1,61	1,15	1,42	0,97	0,86	1,20	0,97	0,82	thioredoxin family protein
251975_at	At3g53230	1,19	1,21	1,64	1,25	1,15	0,99	1,28	1,59	cell division cycle protein 48, putative / CDC48, putative

251976_at	At3g53240	0,78	0,76	0,67	0,89	1,02	0,72	0,85	0,98 leucine-rich repeat family protein
251977_at	At3g53250	1,03	1,00	0,93	0,85	0,87	0,92	1,03	1,02 auxin-responsive family protein
251984_at	At3g53260	1,25	1,14	1,51	1,25	1,09	1,25	1,30	1,23 phenylalanine ammonia-lyase 2 (PAL2)
251980_at	At3g53270	1,31	1,13	1,24	0,89	0,91	1,13	1,33	1,73 expressed protein
251987_at	At3g53280	1,02	0,85	0,64	1,17	1,39	1,04	0,90	0,73 cytochrome P450 71B5 (CYP71B5)
251978_at	At3g53290	0,90	1,00	0,98	0,98	1,07	0,98	0,94	1,03 cytochrome P450, putative
251988_at	At3g53300	0,96	0,99	1,03	0,98	1,11	1,02	1,16	0,91 cytochrome P450 family protein
251986_at	At3g53310	0,66	0,62	0,48	0,93	0,99	0,71	0,80	0,66 transcriptional factor B3 family protein
251990_at	At3g53320	1,02	1,04	1,11	1,06	1,01	1,06	0,99	0,96 expressed protein
251991_at	At3g53340	1,03	0,90	0,91	1,33	1,21	1,00	0,92	1,09 CCAAT-box binding transcription factor, putative
251992_at	At3g53350	1,05	0,87	0,97	0,86	0,92	1,10	1,13	1,13 myosin heavy chain-related
251965_at	At3g53360	1,05	1,06	1,16	0,83	0,82	1,18	1,10	1,24 pentatricopeptide (PPR) repeat-containing protein
251964_at	At3g53370	1,07	0,88	0,76	1,19	1,00	0,94	0,84	0,86 DNA-binding S1FA family protein
251966_at	At3g53380	0,96	1,01	1,01	0,85	0,95	0,76	0,89	0,91 lectin protein kinase family protein
251967_at	At3g53390	1,20	1,15	1,25	0,80	0,84	1,18	1,24	1,10 transducin family protein / WD-40 repeat family protein
251937_at	At3g53400	0,64	0,56	0,43	0,82	0,85	0,85	0,78	0,78 expressed protein
251959_at	At3g53410	0,64	0,68	0,64	1,21	0,99	0,94	0,91	0,65 zinc finger (C3HC4-type RING finger) family protein
251962_at	At3g53420	1,38	1,37	1,46	2,01	1,95	1,05	1,02	0,89 plasma membrane intrinsic protein 2A (PIP2A) / aquaporin PIP2.1 (PIP2.1)
251938_at	At3g53430	1,38	1,16	1,02	1,23	1,12	1,05	0,97	1,02 60S ribosomal protein L12 (RPL12B)
251939_at	At3g53440	0,97	0,97	0,98	1,08	1,16	0,82	0,89	0,95 expressed protein
251940_at	At3g53450	0,98	1,09	0,89	0,99	0,85	1,03	1,04	0,99 hypothetical protein
251956_at	At3g53460	0,76	0,80	1,09	0,68	0,65	0,98	0,85	0,86 29 kDa ribonucleoprotein, chloroplast / RNA-binding protein cp 29
251941_at	At3g53470	0,84	0,90	1,10	1,06	1,04	1,05	0,98	1,03 expressed protein
251942_at	At3g53480	0,89	0,82	0,69	0,61	0,56	1,60	2,36	2,00 ABC transporter family protein
251943_at	At3g53500	1,43	1,55	1,59	0,87	1,06	1,16	1,10	1,11 zinc knuckle (CCHC-type) family protein
251944_at	At3g53510	0,97	1,00	0,93	0,97	1,07	1,02	1,06	0,90 ABC transporter family protein
251945_at	At3g53520	0,97	0,83	0,81	1,08	1,18	0,99	0,76	0,85 NAD-dependent epimerase/dehydratase family protein
251946_at	At3g53540	1,09	1,13	1,05	0,83	1,31	1,08	1,10	1,16 expressed protein
251947_at	At3g53550	1,11	0,95	1,25	1,05	0,97	0,83	0,94	1,17 hypothetical protein
251958_at	At3g53560	1,10	0,89	0,92	0,88	0,98	0,97	0,87	0,84 chloroplast lumen common family protein
251963_at	At3g53570	0,85	1,04	1,00	0,82	1,16	0,91	1,02	0,93 protein kinase (AFC1) (AME2)
251948_at	At3g53580	1,24	1,18	1,01	1,03	0,89	0,93	0,89	0,89 diaminopimelate epimerase family protein
251949_at	At3g53590	1,01	1,11	1,06	1,02	1,07	0,97	0,99	0,95 leucine-rich repeat transmembrane protein kinase, putative
251950_at	At3g53600	1,12	1,08	0,82	1,28	1,14	1,28	1,07	1,09 zinc finger (C2H2 type) family protein
251960_at	At3g53610	1,03	0,98	0,87	1,38	1,38	0,89	0,93	0,83 Ras-related GTP-binding protein, putative
251961_at	At3g53620	1,26	1,11	1,16	1,51	1,43	0,95	0,96	0,99 inorganic pyrophosphatase, putative (soluble) / pyrophosphate phospho-hy
251951_s_at	At3g53630	1,77	1,43	1,34	0,97	0,96	0,97	1,04	0,97 hypothetical protein
251952_at	At3g53650	0,96	0,96	1,00	1,16	1,36	1,17	0,88	0,94 histone H2B, putative
251953_at	At3g53660	1,01	0,98	0,95	1,12	0,81	0,87	0,90	0,90 expressed protein
251954_at	At3g53670	1,17	1,12	1,06	1,00	1,13	0,94	0,87	0,94 expressed protein
251955_at	At3g53680	1,15	1,28	1,16	0,94	0,94	1,08	1,32	1,23 PHD finger transcription factor, putative
251957_at	At3g53690	1,02	1,00	1,05	0,99	1,10	1,01	0,96	0,87 zinc finger (C3HC4-type RING finger) family protein
251936_at	At3g53700	1,15	1,05	1,13	0,76	0,64	0,82	0,74	0,83 pentatricopeptide (PPR) repeat-containing protein
251905_at	At3g53710	1,03	1,02	1,15	1,05	0,92	0,93	0,85	0,95 ARF GAP-like zinc finger-containing protein ZIGA2 (ZIGA2)
251906_at	At3g53720	0,85	0,96	1,07	0,62	0,92	1,12	1,44	1,59 cation/hydrogen exchanger, putative (CHX20)
251924_at	At3g53730	0,86	0,78	0,60	1,00	1,07	0,92	0,81	0,65 histone H4

251926_at	At3g53740	1,49	1,26	1,14	1,07	1,11	1,07	1,00	0,95	60S ribosomal protein L36 (RPL36B)
251907_at	At3g53760	1,03	1,05	1,46	0,85	1,06	0,96	1,03	1,05	tubulin family protein
251908_at	At3g53770	0,96	1,04	1,03	0,88	0,97	1,01	1,18	1,13	late embryogenesis abundant protein-related / LEA protein-related
251930_at	At3g53780	0,87	0,77	0,90	1,12	0,82	0,98	0,92	1,03	rhomboid family protein
251909_at	At3g53790	1,04	1,06	0,93	1,01	1,10	0,90	0,94	1,01	telomere-binding protein, putative
251919_at	At3g53800	0,92	1,05	1,28	0,64	0,65	1,01	1,04	1,31	armadillo/beta-catenin repeat family protein
251910_at	At3g53810	0,57	0,73	0,82	1,05	0,89	0,79	0,75	1,20	lectin protein kinase, putative
251911_at	At3g53820	0,97	1,18	1,08	0,99	1,05	0,97	1,07	0,85	zinc finger (C2H2 type) family protein
251912_at	At3g53840	1,17	1,02	0,91	1,06	1,09	1,00	1,01	1,05	protein kinase family protein
251923_at	At3g53880	1,04	0,92	1,00	0,96	1,38	1,04	0,89	1,01	aldo/keto reductase family protein
251921_at	At3g53890	1,40	1,15	1,13	1,50	1,53	1,03	0,97	0,90	40S ribosomal protein S21 (RPS21B)
251920_at	At3g53900	0,89	0,77	0,69	0,90	0,85	0,94	0,93	0,95	uracil phosphoribosyltransferase, putative / UMP pyrophosphorylase, putati
251913_at	At3g53910	1,00	0,96	0,97	1,00	1,03	0,90	1,02	1,02	malate dehydrogenase-related
251929_at	At3g53920	1,05	1,19	1,48	0,69	0,64	1,45	1,71	2,16	RNA polymerase sigma subunit SigC (sigC) / sigma factor 3 (SIG3)
251914_at	At3g53930	1,38	1,17	1,46	1,15	0,92	1,24	1,27	2,47	protein kinase family protein
251915_at	At3g53940	1,31	1,26	1,54	1,00	0,91	1,11	0,97	1,07	mitochondrial substrate carrier family protein
251931_at	At3g53950	0,80	0,98	0,95	1,01	0,83	1,07	0,87	0,96	glyoxal oxidase-related
251916_at	At3g53960	0,68	0,83	1,38	0,80	0,54	1,13	1,46	1,67	proton-dependent oligopeptide transport (POT) family protein
251917_at	At3g53970	1,09	1,14	1,04	1,25	1,24	0,81	0,92	0,96	proteasome inhibitor-related
251928_at	At3g53980	1,62	1,83	1,57	2,81	2,38	1,82	2,29	2,01	protease inhibitor/seed storage/lipid transfer protein (LTP) family protein
251927_at	At3g53990	0,86	0,75	0,78	1,01	1,04	0,94	0,91	0,97	universal stress protein (USP) family protein
251925_at	At3g54000	0,69	1,95	1,32	0,81	0,76	1,42	1,58	1,50	expressed protein
251932_at	At3g54010	0,94	0,86	0,85	1,02	0,99	1,04	1,07	1,12	peptidyl-prolyl cis-trans isomerase, putative / FK506-binding protein, putativ
251922_at	At3g54030	0,85	0,68	0,78	1,03	1,08	0,94	0,85	0,99	protein kinase family protein
251918_at	At3g54040	1,72	1,68	1,48	1,58	1,29	1,90	1,24	0,96	photoassimilate-responsive protein-related
251885_at	At3g54050	0,98	0,96	0,98	0,79	0,79	1,19	1,09	1,13	fructose-1,6-bisphosphatase, putative / D-fructose-1,6-bisphosphate 1-phos
251933_at	At3g54060	0,97	1,08	0,98	1,13	1,25	1,01	1,09	1,05	hypothetical protein
251934_at	At3g54070	1,02	1,22	1,34	0,94	1,08	1,23	1,06	1,20	ankyrin repeat family protein
251889_at	At3g54080	0,89	0,79	0,74	1,44	1,35	0,81	0,81	0,70	expressed protein
251935_at	At3g54090	1,09	0,98	1,02	0,67	0,72	1,00	0,93	0,97	pfkB-type carbohydrate kinase family protein
251901_at	At3g54100	1,02	1,15	1,25	0,92	1,05	1,03	1,15	1,32	expressed protein
251902_at	At3g54110	0,89	0,84	0,75	0,98	1,11	0,91	0,89	0,88	plant uncoupling mitochondrial protein (PUMP)
251903_at	At3g54120	0,95	0,90	1,19	1,10	1,22	0,96	0,88	1,04	reticulon family protein (RTNLB12)
251904_at	At3g54130	0,91	0,91	0,82	0,82	0,88	0,99	1,04	1,29	josephin family protein
251882_at	At3g54140	0,73	0,64	0,69	0,97	0,86	0,82	0,80	0,78	proton-dependent oligopeptide transport (POT) family protein
251884_at	At3g54150	0,93	1,24	1,31	1,09	1,21	1,05	1,22	1,58	embryo-abundant protein-related
251887_at	At3g54170	1,12	1,05	1,03	1,05	1,20	1,06	1,21	1,37	FKBP12 interacting protein (FIP37)
251888_at	At3g54190	1,07	1,06	1,06	0,83	1,06	1,15	1,03	0,98	expressed protein
251879_at	At3g54200	0,61	0,79	0,82	0,90	0,84	0,80	0,78	0,84	expressed protein
251883_at	At3g54210	1,08	1,01	0,97	0,75	0,77	1,01	1,00	0,88	ribosomal protein L17 family protein
251890_at	At3g54220	0,98	1,11	0,85	0,97	0,91	0,95	0,82	0,93	scarecrow transcription factor, putative
251873_at	At3g54230	1,15	1,04	1,05	0,93	1,02	1,10	1,27	1,47	zinc finger protein-related / D111/G-patch domain-containing protein / RNA
251874_at	At3g54240	0,99	1,20	0,93	0,95	1,02	1,30	0,94	0,91	hydrolase, alpha/beta fold family protein
251881_at	At3g54250	1,04	0,93	0,76	0,99	1,31	1,13	1,16	1,25	mevalonate diphosphate decarboxylase, putative
251886_at	At3g54260	1,07	1,42	1,36	1,48	1,86	0,58	0,90	1,00	expressed protein
251875_at	At3g54270	0,87	1,04	0,98	1,05	0,93	0,96	0,98	0,94	sucrose-phosphatase 3 (SPP3)

251876_at	At3g54280	0,97	1,14	1,74	0,96	0,84	1,12	1,25	1,39	SNF2 domain-containing protein / helicase domain-containing protein
251880_at	At3g54290	0,79	0,84	0,81	1,01	1,06	0,86	0,93	0,93	expressed protein
251877_at	At3g54300	0,97	0,85	1,02	0,97	1,02	1,04	0,91	0,94	synaptobrevin family protein
251878_at	At3g54310	0,99	1,02	0,99	1,03	0,91	0,93	1,11	1,12	hypothetical protein
251891_at	At3g54320	0,65	0,91	0,90	0,90	1,02	0,89	0,92	1,18	ovule development protein, putative
251892_at	At3g54330	0,60	0,78	0,86	1,21	0,95	0,87	1,16	0,92	ovule development protein, putative
251898_at	At3g54340	1,03	0,94	0,98	1,07	1,01	1,00	0,96	0,84	floral homeotic protein APETALA3 (AP3)
251866_at	At3g54350	1,00	1,00	1,13	0,92	0,89	1,10	1,10	1,04	forkhead-associated domain-containing protein / FHA domain-containing pr
251897_at	At3g54360	0,80	0,78	0,86	0,80	0,81	0,95	0,91	0,99	expressed protein
251893_at	At3g54370	1,10	1,05	1,13	1,12	1,04	0,95	1,01	1,15	SAC3/GANP family protein
251894_at	At3g54380	1,18	1,08	1,20	1,08	1,00	1,02	0,90	1,21	SAC3/GANP family protein
251896_at	At3g54390	1,21	1,01	1,12	1,20	1,14	0,97	1,10	1,12	expressed protein
251899_at	At3g54400	1,30	1,13	1,06	1,83	1,49	0,72	0,67	0,64	aspartyl protease family protein
251895_at	At3g54420	1,26	0,97	0,83	1,67	1,12	1,17	0,97	1,19	class IV chitinase (CHIV)
251900_at	At3g54430	0,87	0,83	0,95	0,86	0,99	0,90	0,99	1,26	lateral root primordium (LRP) protein-related
251867_at	At3g54480	0,71	0,88	0,88	1,01	1,02	0,83	0,93	0,93	SKP1 interacting partner 5 (SKIP5)
251868_at	At3g54490	1,00	1,15	1,04	0,96	0,97	1,22	1,07	0,91	eukaryotic rpb5 RNA polymerase subunit family protein
251869_at	At3g54500	2,22	1,88	2,12	1,08	1,02	1,68	1,72	1,64	expressed protein
251870_at	At3g54510	1,18	1,23	1,21	1,03	1,06	1,24	1,10	0,98	early-responsive to dehydration protein-related / ERD protein-related
251871_at	At3g54520	0,98	1,00	1,01	1,01	0,99	1,07	1,00	0,95	hypothetical protein
251845_at	At3g54540	1,07	0,94	1,01	0,97	1,03	1,04	0,88	0,95	ABC transporter family protein
251846_at	At3g54560	1,28	1,17	0,97	2,24	1,87	0,98	1,01	1,00	histone H2A.F/Z
251872_at	At3g54570	1,07	0,93	0,93	0,88	1,00	1,06	1,05	1,10	calmodulin-binding protein-related
251842_at	At3g54580	1,06	1,05	0,88	1,00	1,69	1,01	1,07	0,93	proline-rich extensin-like family protein
251843_x_at	At3g54590	1,03	1,17	1,01	1,54	1,45	1,34	1,06	0,99	proline-rich extensin-like family protein
251849_at	At3g54610	1,04	1,17	1,16	0,87	0,74	1,31	0,94	1,06	histone acetyltransferase (GCN5)
251848_at	At3g54620	0,89	1,00	1,47	1,05	0,92	1,01	1,04	1,22	bZIP transcription factor family protein
251844_at	At3g54630	1,21	1,18	1,11	1,22	1,48	0,91	0,97	0,93	expressed protein
251847_at	At3g54640	1,18	1,11	1,20	1,11	1,08	1,00	0,97	0,98	tryptophan synthase, alpha subunit (TSA1)
251850_at	At3g54650	1,02	1,10	0,96	0,88	1,01	0,88	0,76	0,80	F-box family protein (FBL17)
251860_at	At3g54660	1,15	1,10	1,13	0,89	0,95	1,16	1,24	1,26	gluthatione reductase, chloroplast
251851_at	At3g54670	0,85	0,86	1,13	0,95	0,94	1,12	1,30	1,42	structural maintenance of chromosomes (SMC) family protein
251859_at	At3g54680	0,83	0,81	1,03	1,19	0,96	0,91	0,99	0,88	proteophosphoglycan-related
251855_at	At3g54690	0,80	0,82	0,76	0,95	0,97	0,82	0,72	0,79	sugar isomerase (SIS) domain-containing protein / CBS domain-containing
251856_at	At3g54720	1,01	1,13	1,27	0,41	0,56	1,14	1,14	1,13	glutamate carboxypeptidase, putative (AMP1)
251852_at	At3g54750	1,12	1,39	1,20	1,31	1,30	0,88	0,88	0,90	expressed protein
251857_at	At3g54770	0,88	1,25	0,97	1,29	1,18	0,99	0,83	1,08	RNA recognition motif (RRM)-containing protein
251853_at	At3g54790	0,96	0,95	1,33	0,89	0,84	0,99	1,01	0,98	armadillo/beta-catenin repeat family protein / U-box domain-containing prot
251854_at	At3g54800	1,00	1,00	1,00	1,08	1,27	0,97	1,10	1,02	pleckstrin homology (PH) domain-containing protein / lipid-binding START d
251861_at	At3g54810	0,69	0,97	1,61	0,99	0,86	1,12	1,52	1,67	zinc finger (GATA type) family protein
251858_at	At3g54820	1,35	1,24	0,99	2,99	2,76	0,90	0,87	0,90	aquaporin, putative
251817_at	At3g54840	1,05	0,99	0,95	0,99	1,15	1,03	0,99	0,92	Rab GTPase (ARA6)
251862_at	At3g54850	1,21	0,90	0,94	1,14	0,98	1,09	0,93	1,14	armadillo/beta-catenin repeat family protein / U-box domain-containing fami
251818_at	At3g54860	0,97	0,88	0,84	1,00	0,83	0,89	1,02	0,87	vacuolar protein sorting protein, putative
251863_at	At3g54870	1,09	0,91	0,97	1,03	1,01	1,02	0,99	1,26	armadillo/beta-catenin repeat family protein / kinesin motor family protein
251814_at	At3g54890	1,05	1,06	1,08	0,90	0,91	0,99	0,99	1,04	chlorophyll A-B binding protein / LHCI type I (CAB)

251815_at	At3g54900	1,30	1,15	1,20	1,08	1,23	1,16	0,86	0,90	CAX-interacting protein 1 (CAXIP1)
251864_at	At3g54920	1,09	1,08	1,40	1,13	1,33	0,80	0,82	0,97	pectate lyase, putative / powdery mildew susceptibility protein (PMR6)
251865_at	At3g54930	1,21	1,27	1,24	0,82	1,12	1,06	1,27	0,80	serine/threonine protein phosphatase 2A (PP2A) regulatory subunit B', puta
251838_at	At3g54940	0,13	0,16	0,26	0,65	0,82	1,03	1,06	1,12	cysteine proteinase, putative
251839_at	At3g54950	0,89	1,42	1,09	1,81	2,27	1,04	0,88	0,77	patatin-related
251840_at	At3g54960	0,97	0,91	0,74	1,20	1,37	0,89	0,90	0,77	thioredoxin family protein
251812_at	At3g54970	0,88	0,94	1,18	0,81	0,82	0,89	0,94	0,92	expressed protein
251841_at	At3g54980	0,92	1,07	1,01	0,85	1,11	0,89	0,93	0,80	pentatricopeptide (PPR) repeat-containing protein
251811_at	At3g54990	0,92	0,85	0,94	0,83	0,92	1,01	1,20	1,10	AP2 domain-containing transcription factor, putative
251813_at	At3g55000	1,02	1,01	1,14	1,21	1,05	1,08	0,93	0,99	tonneau family protein
251816_at	At3g55005	1,08	1,07	1,23	1,18	1,36	1,03	0,85	0,95	tonneau 1b (TON1b)
251830_at	At3g55010	1,14	1,02	0,99	0,84	0,78	0,98	0,95	0,90	phosphoribosylformylglycinamidine cyclo-ligase, chloroplast / phosphoribos
251829_at	At3g55020	1,00	0,91	0,92	1,01	0,45	1,04	1,07	0,94	RabGAP/TBC domain-containing protein
251819_at	At3g55030	1,40	1,40	1,33	1,22	1,17	0,93	0,98	1,16	phosphatidylglycerolphosphate synthase, putative
251820_at	At3g55040	0,87	1,05	1,06	1,02	0,98	1,13	1,21	1,22	In2-1 protein, putative
251821_at	At3g55050	0,78	0,65	0,65	0,98	0,85	0,99	1,02	1,02	serine/threonine protein phosphatase 2C (PP2C6)
251822_at	At3g55060	1,04	0,95	1,01	1,11	1,09	1,10	1,04	0,89	expressed protein
251828_at	At3g55070	0,90	0,95	1,11	1,06	1,12	1,03	0,87	1,18	expressed protein
251823_at	At3g55080	0,64	0,76	0,76	0,63	0,74	0,89	0,90	0,83	SET domain-containing protein
251824_at	At3g55090	1,17	1,09	0,99	1,18	1,03	1,03	1,07	1,10	ABC transporter family protein
251825_at	At3g55100	0,88	0,95	1,04	0,98	0,91	1,04	1,10	1,18	ABC transporter family protein
251826_at	At3g55110	1,55	1,15	1,14	1,07	1,11	1,31	1,10	0,81	ABC transporter family protein
251827_at	At3g55120	1,26	1,17	1,18	1,09	0,99	1,05	0,97	1,01	chalcone-flavanone isomerase / chalcone isomerase (CHI)
251785_at	At3g55130	1,11	1,09	0,93	1,03	0,92	1,19	0,86	0,68	ABC transporter family protein
251831_at	At3g55140	1,03	0,95	1,01	1,29	1,05	0,87	0,73	0,79	pectate lyase family protein
251832_at	At3g55150	0,98	0,98	0,67	1,02	0,94	1,10	0,84	0,66	exocyst subunit EXO70 family protein
251833_at	At3g55160	0,98	1,23	1,18	0,87	0,86	1,13	0,96	0,85	expressed protein
251834_at	At3g55170	1,07	1,00	1,03	1,28	1,48	1,00	0,97	0,85	60S ribosomal protein L35 (RPL35C)
251835_at	At3g55180	0,99	1,03	1,06	1,05	1,06	1,01	1,15	0,94	esterase/lipase/thioesterase family protein
251836_at	At3g55190	0,93	0,94	0,98	1,01	1,10	0,98	0,85	0,98	esterase/lipase/thioesterase family protein
251837_s_at	At3g55200	1,19	1,15	1,17	0,92	0,73	0,97	0,95	0,90	splicing factor, putative
251810_at	At3g55250	0,99	0,86	1,05	0,92	0,74	1,10	0,94	0,88	expressed protein
251782_at	At3g55260	0,73	0,68	0,64	1,09	1,08	0,84	0,70	0,67	glycosyl hydrolase family 20 protein
251786_at	At3g55270	0,79	0,80	1,03	0,72	0,84	0,98	0,93	1,06	MAP kinase phosphatase (MKP1)
251783_at	At3g55280	1,44	1,09	1,08	1,42	1,33	1,08	0,99	1,02	60S ribosomal protein L23A (RPL23aB)
251779_at	At3g55300	0,99	0,97	0,94	1,04	0,98	0,98	1,03	0,94	---
251780_s_at	At3g55310	0,75	0,67	0,71	1,18	1,19	0,82	0,82	0,91	short-chain dehydrogenase/reductase (SDR) family protein
251781_at	At3g55320	0,74	0,66	0,75	0,80	0,82	1,04	0,94	1,10	ABC transporter family protein
251784_at	At3g55330	1,22	1,09	1,03	0,80	0,88	1,21	1,15	1,09	photosystem II reaction center PsbP family protein
251803_at	At3g55350	1,10	0,86	0,86	1,10	1,13	0,80	0,78	0,79	expressed protein
251796_at	At3g55360	0,94	0,92	0,86	1,09	0,98	0,81	0,77	0,77	3-oxo-5-alpha-steroid 4-dehydrogenase family protein / steroid 5-alpha-redu
251806_at	At3g55370	1,29	1,22	1,14	1,30	1,30	1,07	1,04	1,20	Dof-type zinc finger domain-containing protein
251802_at	At3g55380	1,14	1,08	1,19	1,25	1,14	1,02	0,83	0,97	ubiquitin-conjugating enzyme 14 (UBC14)
251795_at	At3g55390	0,71	0,77	1,01	1,00	0,76	0,74	0,74	0,97	integral membrane family protein
251807_at	At3g55400	0,81	0,73	0,73	0,81	0,87	1,00	1,03	1,01	methionyl-tRNA synthetase / methionine--tRNA ligase / MetRS (cpMetRS)
251787_at	At3g55410	0,97	0,81	0,76	0,89	0,91	0,98	0,91	0,85	2-oxoglutarate dehydrogenase E1 component, putative / oxoglutarate deca

251788_at	At3g55420	1,01	0,81	0,68	1,13	1,01	0,77	0,73	0,81	expressed protein
251804_at	At3g55430	0,83	0,83	1,04	0,95	1,12	1,01	1,05	1,14	glycosyl hydrolase family 17 protein / beta-1,3-glucanase, putative
251801_at	At3g55440	0,91	0,91	0,86	1,25	1,21	0,96	0,97	0,94	triosephosphate isomerase, cytosolic, putative
251789_at	At3g55450	0,73	0,83	0,87	0,77	0,85	0,92	0,99	0,99	protein kinase, putative
251798_at	At3g55460	1,06	0,99	1,01	1,14	0,92	0,98	0,94	0,92	SC35-like splicing factor, 30 kD (SCL30)
251790_at	At3g55470	1,03	0,84	0,73	1,33	1,06	0,93	0,87	0,93	C2 domain-containing protein
251808_at	At3g55480	0,75	0,67	0,67	1,01	0,81	0,99	0,93	0,73	adaptin family protein
251791_at	At3g55500	0,94	0,92	0,79	0,85	1,16	0,79	0,66	0,52	expansin, putative (EXP16)
251800_at	At3g55510	1,25	1,04	0,89	0,95	0,89	0,95	0,99	0,78	expressed protein
251799_at	At3g55520	0,89	0,82	0,95	0,89	0,93	0,93	1,04	1,05	immunophilin, putative / FKBP-type peptidyl-prolyl cis-trans isomerase, puta
251805_at	At3g55530	0,98	0,90	0,92	0,78	0,87	1,07	1,12	0,96	zinc finger (C3HC4-type RING finger) family protein
251792_at	At3g55550	0,95	1,03	0,91	1,05	0,95	1,07	1,03	0,94	lectin protein kinase, putative
251797_at	At3g55560	1,07	1,02	0,93	0,92	1,01	1,18	0,99	0,94	DNA-binding protein-related
251793_at	At3g55580	0,96	1,12	0,86	0,97	0,88	1,00	0,99	1,08	regulator of chromosome condensation (RCC1) family protein
251794_at	At3g55590	1,02	1,01	0,97	0,85	1,07	1,03	1,02	0,82	GDP-mannose pyrophosphorylase, putative
251809_at	At3g55600	0,76	0,59	0,74	1,18	1,39	0,97	0,93	0,85	expressed protein
251760_at	At3g55605	1,23	1,10	1,27	1,34	1,28	0,96	0,88	0,64	mitochondrial glycoprotein family protein / MAM33 family protein
251775_s_at	At3g55610	0,79	1,00	1,17	0,84	1,01	1,21	1,21	1,28	delta 1-pyrroline-5-carboxylate synthetase A / P5CS A (P5CS1)
251776_at	At3g55620	1,14	1,11	1,00	0,89	0,86	1,09	0,98	0,92	eukaryotic translation initiation factor 6, putative / eIF-6, putative
251759_at	At3g55630	1,43	1,41	1,56	0,77	0,68	1,61	2,09	2,19	dihydrofolate synthetase/folylpolyglutamate synthetase (DHFS/FPGS4)
251757_at	At3g55640	0,56	0,58	0,82	1,03	0,90	0,80	0,92	1,11	mitochondrial substrate carrier family protein
251777_s_at	At3g55650	0,92	0,94	1,00	0,99	0,99	0,95	1,00	1,10	pyruvate kinase, putative
251778_at	At3g55660	1,15	1,14	1,11	1,29	1,18	1,05	0,87	0,83	expressed protein
251747_at	At3g55670	0,99	0,88	0,88	0,97	1,11	1,03	1,12	1,14	hypothetical protein
251748_at	At3g55680	1,01	1,06	0,99	1,02	0,96	0,93	0,92	1,07	invertase/pectin methylesterase inhibitor family protein
251749_at	At3g55690	0,81	0,93	0,98	0,87	0,78	1,02	0,90	1,04	expressed protein
251761_at	At3g55700	0,96	1,19	1,08	0,99	1,16	1,07	1,06	1,05	UDP-glucoronosyl/UDP-glucosyl transferase family protein
251750_at	At3g55710	1,06	1,23	1,30	0,94	0,75	1,34	1,31	1,34	UDP-glucoronosyl/UDP-glucosyl transferase family protein
251751_at	At3g55720	0,98	1,01	1,09	1,13	1,27	1,08	1,10	0,98	expressed protein
251763_at	At3g55730	1,04	0,99	1,06	0,97	1,20	0,92	0,98	1,00	myb family transcription factor (MYB109)
251752_at	At3g55740	0,99	0,86	0,87	0,68	0,68	1,22	1,20	1,32	proline transporter 2 (ProT2)
251753_at	At3g55760	0,86	0,67	1,00	0,82	0,99	0,91	0,90	1,02	expressed protein
251758_at	At3g55770	0,82	0,73	0,72	1,12	1,08	0,81	0,86	0,92	LIM domain-containing protein
251754_at	At3g55780	0,88	0,96	1,02	1,01	0,95	0,96	0,99	0,80	glycosyl hydrolase family 17 protein
251755_at	At3g55790	1,17	1,07	0,97	1,01	0,84	1,07	0,98	1,04	expressed protein
251762_at	At3g55800	1,18	1,11	1,22	0,87	0,93	1,27	1,20	1,16	sedoheptulose-1,7-bisphosphatase, chloroplast / sedoheptulose-bisphosph
251756_at	At3g55820	0,97	0,94	1,07	1,01	0,87	1,04	0,87	1,10	hypothetical protein
251764_at	At3g55830	0,94	0,85	0,99	0,98	1,31	0,98	0,91	0,88	glycosyltransferase family protein 47
251774_at	At3g55840	1,07	1,26	1,12	1,07	1,12	1,17	1,57	1,60	expressed protein
251717_at	At3g55850	0,47	0,42	0,47	0,83	0,75	0,75	0,85	0,96	amidohydrolase family protein
251716_at	At3g55870	0,94	1,08	0,99	0,95	0,92	1,00	0,93	0,88	anthranilate synthase, alpha subunit, putative
251765_at	At3g55880	0,92	1,01	1,08	0,93	1,25	0,97	0,93	0,90	---
251743_at	At3g55890	0,88	1,05	1,29	1,09	0,87	0,99	1,22	1,22	yippee family protein
251766_at	At3g55910	0,96	0,89	1,06	0,85	0,84	1,10	1,09	1,16	expressed protein
251772_at	At3g55920	0,87	0,67	0,67	0,95	0,93	0,90	0,83	0,92	peptidyl-prolyl cis-trans isomerase, putative / cyclophilin, putative / rotamas
251767_at	At3g55930	1,09	1,03	1,05	0,97	0,96	1,00	0,94	1,30	RNA splicing factor-related

251768_at	At3g55940	1,13	1,03	1,13	1,17	0,88	0,87	1,16	1,15	phosphoinositide-specific phospholipase C, putative
251769_at	At3g55950	0,82	0,61	0,62	1,41	0,94	1,00	0,83	0,96	protein kinase family protein
251773_at	At3g55960	1,11	1,27	1,91	1,19	1,17	1,00	1,13	1,34	NLI interacting factor (NIF) family protein
251770_at	At3g55970	0,94	0,93	1,16	0,76	0,50	1,16	0,93	1,11	oxidoreductase, 2OG-Fe(II) oxygenase family protein
251745_at	At3g55980	0,92	1,63	2,06	0,80	0,86	1,23	2,30	1,96	zinc finger (CCCH-type) family protein
251771_at	At3g56000	0,86	0,94	0,92	1,06	0,70	0,87	1,00	1,21	glycosyl transferase family 2 protein
251744_at	At3g56010	0,99	1,07	1,20	0,96	0,62	0,95	0,97	0,84	expressed protein
251741_at	At3g56040	1,29	1,13	1,18	0,65	0,81	1,35	1,14	1,31	expressed protein
251742_at	At3g56050	0,82	0,93	1,04	0,79	0,75	0,88	0,97	1,02	protein kinase family protein
251746_at	At3g56060	0,94	0,81	0,75	0,72	0,83	0,97	0,84	0,85	glucose-methanol-choline (GMC) oxidoreductase family protein
251740_at	At3g56070	1,99	1,64	1,49	1,00	0,99	1,07	0,98	0,85	peptidyl-prolyl cis-trans isomerase, putative / cyclophilin, putative / rotamas
251713_at	At3g56080	1,11	1,05	1,16	0,51	0,89	1,35	1,55	1,21	dehydration-responsive protein-related
251735_at	At3g56090	0,90	0,88	1,18	0,82	0,90	0,62	0,73	0,73	ferritin, putative
251718_at	At3g56100	0,96	1,10	0,99	1,00	1,14	0,90	0,91	1,08	leucine-rich repeat transmembrane protein kinase, putative
251732_at	At3g56110	0,74	0,77	0,70	1,02	1,17	0,85	0,70	0,62	prenylated rab acceptor (PRA1) family protein
251712_at	At3g56120	1,35	1,13	1,06	1,11	0,90	1,23	1,00	0,96	Met-10+ like family protein
251736_at	At3g56130	1,17	1,00	0,82	1,00	0,89	0,96	0,87	0,83	biotin/lipoyl attachment domain-containing protein
251719_at	At3g56140	0,97	1,01	1,10	0,72	0,83	1,27	1,16	1,12	expressed protein
251738_at	At3g56150	1,12	1,11	1,17	0,95	0,83	1,04	1,01	0,98	eukaryotic translation initiation factor 3 subunit 8 / eIF3 p110 / eIF3c / p105
251720_at	At3g56160	0,89	0,93	1,20	0,92	0,88	0,80	0,88	0,89	expressed protein
251739_at	At3g56170	0,56	0,53	0,69	1,26	1,16	0,91	0,75	0,75	Ca(2+)-dependent nuclease
251721_s_at	At3g56190	1,00	0,81	0,85	1,03	1,08	0,85	0,90	0,98	alpha-soluble NSF attachment protein 2 / alpha-SNAP2 / ASNAP2
251722_at	At3g56200	0,71	0,60	0,63	1,13	0,97	0,95	0,92	1,04	amino acid transporter family protein
251723_at	At3g56230	1,01	1,07	1,11	1,03	1,06	0,92	1,08	0,82	speckle-type POZ protein-related
251733_at	At3g56240	1,64	1,64	1,56	1,41	1,26	0,96	1,07	1,03	copper homeostasis factor / copper chaperone (CCH) (ATX1)
251724_at	At3g56250	0,95	1,00	1,05	0,84	1,06	0,99	1,03	0,94	expressed protein
251725_at	At3g56260	0,95	1,20	0,80	1,29	1,33	1,06	0,98	0,84	expressed protein
251734_at	At3g56270	1,08	1,14	1,14	1,08	1,05	1,07	1,03	0,90	expressed protein
251726_at	At3g56280	0,95	1,07	0,96	1,01	1,07	0,93	0,92	0,96	---
251727_at	At3g56290	2,42	2,46	2,37	1,45	1,20	1,86	1,63	1,69	expressed protein
251728_at	At3g56300	0,95	1,02	1,07	1,10	1,01	1,09	1,02	0,90	tRNA synthetase class I (C) family protein
251729_at	At3g56310	0,82	0,86	0,88	1,09	1,15	0,90	0,91	0,87	alpha-galactosidase, putative / melibiase, putative / alpha-D-galactoside ga
251730_at	At3g56330	0,90	0,88	1,03	0,91	0,74	1,05	0,86	0,86	N2,N2-dimethylguanosine tRNA methyltransferase family protein
251737_at	At3g56340	1,31	1,18	1,14	1,22	1,20	0,98	0,92	0,91	40S ribosomal protein S26 (RPS26C)
251731_at	At3g56350	0,32	0,31	0,35	0,71	0,67	0,92	1,00	1,04	superoxide dismutase (Mn), putative / manganese superoxide dismutase, p
251704_at	At3g56360	1,02	1,28	1,32	0,92	0,87	1,31	1,67	2,15	expressed protein
251714_at	At3g56370	0,80	0,75	0,62	0,73	0,76	0,79	0,75	0,65	leucine-rich repeat transmembrane protein kinase, putative
251709_at	At3g56380	0,98	0,99	0,94	0,92	1,00	0,93	1,03	1,02	two-component responsive regulator / response regulator 17 (ARR17)
251715_at	At3g56390	1,00	0,91	1,01	0,94	0,92	0,98	0,97	0,91	hypothetical protein
251705_at	At3g56400	0,95	1,24	1,05	1,46	1,35	2,00	1,36	1,24	WRKY family transcription factor
251684_at	At3g56410	0,62	0,48	0,75	0,88	0,79	1,10	1,09	1,22	expressed protein
251685_at	At3g56430	1,33	1,29	1,01	1,05	0,73	1,17	0,93	0,97	expressed protein
251686_at	At3g56440	0,96	0,89	1,09	0,67	1,03	1,09	0,94	1,26	WD-40 repeat protein family
251687_at	At3g56460	0,88	0,81	1,00	1,03	0,91	0,93	0,95	0,91	oxidoreductase, zinc-binding dehydrogenase family protein
251688_at	At3g56480	1,42	1,36	1,47	1,20	1,31	0,92	0,99	0,83	myosin heavy chain-related
251707_at	At3g56490	1,64	1,51	1,40	1,38	1,33	0,93	0,89	0,97	zinc-binding protein, putative / protein kinase C inhibitor, putative

251689_at	At3g56500	1,03	1,08	1,02	1,03	1,36	0,88	0,94	0,81	serine-rich protein-related
251690_at	At3g56510	1,31	1,00	1,20	1,00	1,06	1,05	1,02	1,26	TBP-binding protein, putative
251691_at	At3g56520	0,88	1,05	1,08	0,97	1,09	1,03	0,93	0,93	no apical meristem (NAM) family protein
251692_s_at	At3g56540	1,03	1,02	1,10	1,01	0,99	1,06	0,97	1,07	serine carboxypeptidase S10 family protein
251693_at	At3g56550	1,12	1,06	1,01	0,93	1,04	1,04	1,05	0,82	pentatricopeptide (PPR) repeat-containing protein
251694_s_at	At3g56560	1,05	1,02	1,02	1,01	0,95	0,94	1,07	0,95	no apical meristem (NAM) protein-related
251708_at	At3g56580	0,92	0,92	0,90	1,12	1,16	0,91	0,67	0,86	zinc finger (C3HC4-type RING finger) family protein
251695_at	At3g56585	0,86	0,64	0,67	1,03	1,42	0,95	0,79	0,62	hydroxyproline-rich glycoprotein family protein
251696_at	At3g56590	1,02	0,99	0,97	0,95	1,04	0,96	1,10	1,14	---
251697_at	At3g56600	0,95	0,74	0,96	0,97	1,16	1,04	0,99	0,81	phosphatidylinositol 3- and 4-kinase family protein
251698_at	At3g56610	0,86	0,94	0,95	1,01	1,08	1,07	1,00	0,85	expressed protein
251706_at	At3g56620	1,15	1,03	0,94	1,25	1,21	0,86	0,83	0,92	integral membrane family protein / nodulin MtN21-related
251699_at	At3g56630	1,30	1,28	1,41	0,80	1,03	1,37	1,17	1,11	cytochrome P450, putative
251700_at	At3g56640	1,02	1,10	0,93	1,12	1,01	0,81	0,99	0,71	exocyst complex subunit Sec15-like family protein
251701_at	At3g56650	1,44	1,40	1,18	0,69	0,83	1,47	1,44	1,32	thylakoid lumenal 20 kDa protein
251702_at	At3g56660	0,92	1,12	0,99	0,82	1,01	1,01	0,99	1,08	bZIP transcription factor family protein
251703_at	At3g56670	1,11	0,94	0,99	0,89	1,05	1,11	1,02	1,09	hypothetical protein
246319_at	At3g56680	0,88	0,91	1,07	0,79	0,79	1,02	0,89	1,08	expressed protein
246295_at	At3g56690	1,03	1,17	1,22	0,80	0,84	0,98	1,03	1,04	calmodulin-binding protein
246342_at	At3g56700	1,08	1,04	0,89	0,92	0,89	0,97	0,98	0,97	male sterility protein, putative
246293_at	At3g56710	0,83	0,82	0,96	0,86	0,72	0,74	1,05	0,85	sigA-binding protein
246343_at	At3g56720	1,18	1,33	2,00	0,87	0,91	1,18	1,22	1,59	expressed protein
246344_at	At3g56730	1,13	0,90	1,10	1,29	1,04	0,90	0,87	0,90	expressed protein
246318_at	At3g56740	1,01	1,10	1,31	1,03	0,78	0,99	0,99	0,91	ubiquitin-associated (UBA)/TS-N domain-containing protein
246296_at	At3g56750	0,79	0,69	0,94	0,83	0,84	0,95	0,88	0,85	expressed protein
246345_at	At3g56760	0,96	0,96	1,03	0,83	0,78	0,99	0,96	1,10	calcium-dependent protein kinase, putative / CDPK, putative
246290_at	At3g56800	1,04	0,85	0,80	1,21	1,23	0,96	0,98	0,95	calmodulin-2/3/5 (CAM3)
246346_at	At3g56810	0,96	1,03	1,09	0,84	0,97	0,89	0,93	0,84	expressed protein
246291_at	At3g56820	1,02	0,92	1,30	0,99	0,94	0,83	0,82	0,91	expressed protein
246347_at	At3g56830	1,02	1,11	1,20	1,18	1,15	0,96	0,99	1,49	expressed protein
246348_at	At3g56840	1,15	1,06	1,14	0,87	1,00	1,00	0,95	0,92	FAD-dependent oxidoreductase family protein
246314_at	At3g56850	0,99	1,23	1,09	0,96	1,07	0,88	0,90	1,00	ABA-responsive element-binding protein 3 (AREB3)
246292_at	At3g56860	1,10	1,01	1,01	0,91	0,78	0,98	1,06	1,01	UBP1 interacting protein 2a (UBA2a)
246315_at	At3g56870	1,05	1,09	1,07	1,11	1,43	0,97	1,05	1,10	hypothetical protein
246289_at	At3g56880	0,79	0,92	0,77	0,78	0,87	0,90	0,93	0,87	VQ motif-containing protein
246316_at	At3g56890	1,07	0,91	0,93	0,94	1,11	1,01	1,04	1,01	F-box family protein-related
246317_at	At3g56900	1,27	0,87	0,90	1,21	0,94	0,83	0,74	0,72	aladin-related / adracalin-related
246294_at	At3g56910	1,04	0,90	0,89	1,01	0,97	1,00	0,95	1,00	expressed protein
251710_at	At3g56930	1,17	0,96	0,97	1,26	1,25	1,22	0,87	0,99	zinc finger (DHHC type) family protein
251664_at	At3g56940	1,25	1,32	1,56	0,87	0,86	1,08	1,03	1,03	dicarboxylate diiron protein, putative (Crd1)
251661_at	At3g56950	1,11	1,10	1,02	1,30	1,24	1,00	0,88	0,90	small basic membrane integral family protein
251711_at	At3g56960	1,15	0,90	1,03	0,91	0,93	1,00	1,07	1,01	phosphatidylinositol-4-phosphate 5-kinase family protein
251677_at	At3g56980	3,78	2,75	1,67	2,89	1,47	5,74	1,63	1,40	basic helix-loop-helix (bHLH) family protein
251678_at	At3g56990	1,25	1,17	1,44	0,87	0,93	1,14	1,30	1,29	glycine-rich protein
251657_at	At3g57000	1,52	1,39	1,81	0,97	0,89	1,14	1,00	1,23	nucleolar essential protein-related
251668_at	At3g57010	0,99	0,87	0,65	1,84	1,10	0,97	1,05	1,16	strictosidine synthase family protein

251658_at	At3g57020	0,78	0,88	0,93	1,15	1,05	1,23	1,24	1,21	strictosidine synthase family protein
251679_at	At3g57030	1,21	1,16	1,00	1,17	1,02	0,95	0,78	0,76	strictosidine synthase family protein
251665_at	At3g57040	2,09	1,51	1,33	0,80	1,05	1,19	1,47	1,09	two-component responsive regulator / response reactor 4 (RR4)
251666_at	At3g57050	0,96	1,02	1,02	0,93	0,98	0,96	0,96	0,85	cystathionine beta-lyase, chloroplast / beta-cystathionase / cysteine lyase (
251680_at	At3g57060	1,18	1,06	1,16	0,98	1,18	1,03	0,98	0,89	non-SMC condensin subunit, XCAP-D2/Cnd1 family protein
251663_at	At3g57070	0,97	0,78	0,67	1,06	1,15	0,94	0,82	1,17	glutaredoxin family protein
251662_at	At3g57080	1,22	1,01	1,39	0,97	1,06	1,06	1,04	0,95	eukaryotic rpb5 RNA polymerase subunit family protein
251659_at	At3g57090	1,07	0,97	1,04	0,95	1,05	1,11	1,02	1,09	expressed protein
251681_at	At3g57100	0,93	1,06	0,88	1,17	0,93	0,97	1,04	1,03	hypothetical protein
251682_s_at	At3g57110	0,99	1,09	1,02	0,95	0,94	0,95	0,92	1,01	hypothetical protein
251683_at	At3g57120	0,73	0,80	0,92	0,86	0,97	0,92	0,93	0,91	protein kinase family protein
251653_at	At3g57130	1,17	1,06	1,06	0,89	1,07	0,92	1,09	0,89	ankyrin repeat family protein / BTB/POZ domain-containing protein
251654_at	At3g57140	0,98	1,00	0,97	1,00	1,00	0,90	0,97	0,97	patatin-related
251667_at	At3g57150	1,25	1,09	1,06	1,26	1,26	0,96	1,00	0,98	dyskerin, putative / nucleolar protein NAP57, putative
251655_at	At3g57160	1,12	1,07	0,91	1,23	1,22	0,91	0,89	0,94	Clone asmbl_10911 unknown mRNA sequence
251660_at	At3g57165	0,98	0,94	0,87	1,33	1,11	1,01	1,00	0,99	expressed protein
251656_at	At3g57170	1,07	0,94	1,05	0,95	0,89	0,99	1,32	1,37	N-acetylglucosaminyl transferase component family protein / Gpi1 family pr
251669_at	At3g57180	1,09	0,92	0,84	0,60	0,61	1,08	1,00	1,00	expressed protein
251670_at	At3g57190	1,70	2,62	3,08	0,64	0,77	1,50	1,53	1,24	peptide chain release factor, putative
251671_at	At3g57210	1,02	1,04	0,94	0,96	0,97	0,97	1,04	0,94	hypothetical protein
251626_at	At3g57220	0,82	0,71	0,65	0,96	0,78	0,76	0,80	0,73	UDP-GlcNAc:dolichol phosphate N-acetylglucosamine-1-phosphate transfe
251672_at	At3g57230	1,30	1,44	1,23	0,97	1,04	1,12	1,09	1,14	MADS-box protein (AGL16)
251673_at	At3g57240	0,98	1,04	0,93	0,89	0,77	1,09	1,09	1,30	beta-1,3-glucanase (BG3)
251674_at	At3g57250	1,07	0,93	0,97	0,99	1,03	0,97	1,00	0,94	emsy N terminus domain-containing protein / ENT domain-containing protei
251625_at	At3g57260	0,49	0,42	0,55	0,86	0,95	14,10	8,00	15,62	glycosyl hydrolase family 17 protein
251627_at	At3g57270	1,04	1,06	0,88	1,08	1,00	1,09	0,97	1,07	glycosyl hydrolase family 17 protein
251624_at	At3g57280	0,78	0,77	0,86	0,80	0,78	0,79	0,84	0,81	expressed protein
251628_at	At3g57290	1,15	1,01	0,98	0,98	1,17	0,99	0,95	0,97	eukaryotic translation initiation factor 3E / eIF3e (TIF3E1)
251675_at	At3g57300	0,78	0,63	0,93	0,86	0,83	1,00	1,02	1,28	transcriptional activator, putative
251676_at	At3g57320	1,35	1,21	1,09	1,16	1,08	0,91	1,01	0,84	expressed protein
251649_at	At3g57330	1,14	0,93	1,10	0,91	1,02	0,95	1,07	1,10	calcium-transporting ATPase, plasma membrane-type, putative / Ca2+-ATF
251622_at	At3g57340	1,01	0,92	0,98	1,26	1,17	0,95	0,91	0,90	DNAJ heat shock N-terminal domain-containing protein
251650_at	At3g57360	0,97	0,94	1,19	1,07	1,26	0,85	0,93	1,06	expressed protein
251651_at	At3g57370	1,06	0,96	0,90	1,00	0,98	1,13	0,83	0,88	transcription factor IIB (TFIIB) family protein
251652_at	At3g57380	1,05	1,11	1,14	0,87	0,93	1,19	0,99	0,98	expressed protein
251623_at	At3g57390	1,07	1,06	1,53	0,90	1,09	0,95	1,04	1,08	MADS-box protein (AGL18)
251629_at	At3g57410	1,07	0,96	1,01	1,13	1,35	0,95	0,86	0,85	villin 3 (VLN3)
251630_at	At3g57420	0,97	0,83	0,97	0,98	0,93	0,85	0,78	0,91	expressed protein
251631_at	At3g57430	1,29	0,99	0,83	0,73	0,65	1,16	1,01	0,75	pentatricopeptide (PPR) repeat-containing protein
251632_at	At3g57440	0,95	0,94	0,98	0,93	1,33	0,95	1,02	0,84	hypothetical protein
251640_at	At3g57450	0,89	0,84	0,93	1,13	0,98	1,02	1,04	1,08	expressed protein
251633_at	At3g57460	0,95	0,91	0,97	1,03	0,74	1,02	1,18	1,10	expressed protein
251641_at	At3g57470	0,82	0,68	0,84	0,82	0,57	0,92	0,99	1,21	peptidase M16 family protein / insulinase family protein
251634_at	At3g57480	0,86	0,77	0,89	1,22	1,06	0,89	0,96	1,00	zinc finger (C2H2 type, AN1-like) family protein
251638_at	At3g57490	1,04	0,72	0,76	0,87	1,10	0,80	0,82	0,62	40S ribosomal protein S2 (RPS2D)
251635_at	At3g57510	1,06	1,08	0,96	1,03	1,03	0,95	0,91	1,04	endo-polygalacturonase (ADPG1)

251642_at	At3g57520	0,94	0,94	1,72	0,66	0,63	1,06	1,04	1,34 alkaline alpha galactosidase, putative
251636_at	At3g57530	0,96	1,47	1,58	0,78	0,91	1,30	1,87	1,59 calcium-dependent protein kinase, putative / CDPK, putative
251644_at	At3g57540	0,75	0,82	1,05	1,18	1,07	0,85	1,17	1,10 remorin family protein
251643_at	At3g57550	0,95	0,94	1,27	1,33	1,20	0,98	1,05	1,57 guanylate kinase 2 (GK-2)
251639_at	At3g57560	0,99	0,88	0,85	0,91	0,97	0,83	0,77	0,69 aspartate/glutamate/uridylate kinase family protein
251637_at	At3g57570	0,99	0,94	1,01	0,96	0,83	1,07	1,16	0,96 expressed protein
251598_at	At3g57600	0,87	0,99	0,95	1,01	0,94	0,90	0,92	0,97 AP2 domain-containing transcription factor, putative
251599_at	At3g57610	1,30	1,23	1,15	0,98	0,93	1,04	1,06	1,01 adenylosuccinate synthetase (ADSS)
251595_at	At3g57620	0,90	1,02	1,00	0,94	1,08	1,08	0,98	0,84 glyoxal oxidase-related
251594_at	At3g57630	0,74	0,89	0,59	0,96	0,82	0,85	0,86	0,83 exostosin family protein
251596_at	At3g57650	0,86	0,80	0,79	1,08	1,14	0,89	0,80	0,88 acyl-CoA:1-acylglycerol-3-phosphate acyltransferase, putative
251593_at	At3g57660	1,27	1,13	1,06	0,84	0,70	0,96	1,01	0,97 DNA-directed RNA polymerase family protein
251592_at	At3g57670	0,99	0,94	0,96	0,98	0,93	1,05	0,96	0,85 zinc finger (C2H2 type) protein (WIP2)
251591_at	At3g57680	1,11	1,13	1,14	0,96	1,05	1,06	0,89	0,98 peptidase S41 family protein
251590_at	At3g57690	0,90	1,05	1,09	0,89	1,09	1,00	1,02	0,94 arabinogalactan-protein, putative (AGP23)
251621_at	At3g57700	0,61	0,65	0,78	1,01	0,99	0,82	0,75	0,75 protein kinase, putative
251600_at	At3g57710	0,91	0,84	0,73	0,96	1,01	0,96	0,93	0,97 protein kinase family protein
251648_at	At3g57720	0,82	0,95	0,86	0,99	0,94	1,00	0,95	0,96 protein kinase, putative
251597_at	At3g57750	0,82	1,01	1,04	0,93	0,92	0,91	0,87	0,87 protein kinase, putative
251603_at	At3g57760	0,50	0,62	0,78	1,15	1,03	0,90	1,08	1,21 protein kinase family protein
251647_at	At3g57770	0,52	0,29	0,37	0,76	0,83	0,82	0,66	0,70 protein kinase, putative
251646_at	At3g57780	0,69	0,46	0,43	0,82	0,75	0,81	0,78	0,88 expressed protein
251602_at	At3g57785	0,99	0,90	0,74	1,00	1,05	1,03	0,88	0,74 expressed protein
251645_at	At3g57790	0,64	0,52	0,70	0,86	0,98	0,86	0,74	0,82 glycoside hydrolase family 28 protein / polygalacturonase (pectinase) famil
251601_at	At3g57795	1,44	2,00	2,25	0,57	0,90	1,05	1,11	1,12 basic helix-loop-helix (bHLH) family protein
251558_at	At3g57810	1,26	1,19	1,81	0,90	1,07	1,02	1,00	1,21 OTU-like cysteine protease family protein
251604_at	At3g57820	1,04	0,98	0,98	1,03	1,02	1,00	1,07	0,96 ---
251605_at	At3g57830	0,96	1,03	1,11	0,84	0,77	1,12	0,88	0,89 leucine-rich repeat transmembrane protein kinase, putative
251606_at	At3g57840	0,98	0,96	1,05	0,97	0,95	1,00	0,99	1,02 self-incompatibility protein-related
251607_at	At3g57850	0,95	1,05	1,03	0,99	1,12	1,12	1,07	1,04 hypothetical protein
251608_at	At3g57860	1,09	1,14	1,06	1,05	1,13	0,81	0,93	0,90 expressed protein
251561_at	At3g57870	1,13	1,07	1,18	1,28	1,17	1,04	0,96	0,98 ubiquitin-conjugating enzyme, putative
251563_at	At3g57880	0,81	0,96	1,08	1,06	0,98	1,04	1,08	1,09 C2 domain-containing protein
251562_at	At3g57890	0,84	0,78	0,79	0,97	1,10	0,94	0,95	0,97 tubulin-specific chaperone C-related
251609_at	At3g57900	1,03	1,05	1,08	0,91	0,98	0,95	0,95	0,93 hypothetical protein
251560_at	At3g57920	1,08	1,06	0,99	0,96	1,10	1,15	0,86	1,04 squamosa promoter-binding protein, putative
251610_at	At3g57930	0,68	0,72	1,15	0,84	1,05	1,24	1,34	1,80 expressed protein
251611_at	At3g57940	1,50	1,02	0,97	0,93	0,81	1,17	0,96	1,41 expressed protein
251612_at	At3g57950	0,97	1,06	1,05	1,10	1,13	0,98	1,07	0,98 expressed protein
251613_at	At3g57960	0,94	1,11	1,01	1,13	0,81	0,99	1,01	0,72 emsy N terminus domain-containing protein / ENT domain-containing protei
251614_at	At3g57970	0,95	1,03	1,05	0,96	1,06	1,04	1,16	1,06 emsy N terminus domain-containing protein / ENT domain-containing protei
251615_at	At3g57980	1,03	1,02	0,96	0,91	1,15	1,11	0,95	0,96 DNA-binding bromodomain-containing protein
251616_at	At3g57990	0,84	0,81	0,84	0,62	0,73	0,94	0,81	0,76 expressed protein
251617_at	At3g58000	1,09	0,98	0,88	1,03	1,04	1,18	0,91	1,02 VQ motif-containing protein
251559_at	At3g58010	0,79	0,72	0,79	0,87	0,76	1,07	1,06	1,03 expressed protein
251618_at	At3g58020	0,99	1,05	0,98	0,92	0,96	0,96	0,97	1,15 DNAJ heat shock N-terminal domain-containing protein

251589_at	At3g58040	1,21	1,24	1,22	1,07	1,05	0,95	0,98	1,15 seven in absentia (SINA) family protein
251619_at	At3g58050	1,06	1,04	1,14	0,95	0,81	1,10	1,06	1,33 expressed protein
251620_at	At3g58060	0,97	0,89	0,88	0,95	0,92	1,11	1,02	1,00 cation efflux family protein / metal tolerance protein, putative (MTPc3)
251586_at	At3g58070	0,94	1,08	0,97	1,00	0,96	1,09	0,90	0,94 zinc finger (C2H2 type) family protein
251587_at	At3g58080	1,06	0,91	1,01	0,98	1,04	0,94	0,97	0,84 hypothetical protein
251588_at	At3g58090	0,95	0,87	1,02	0,92	1,03	0,86	0,92	0,83 pseudogene, disease resistance response protein-related
251574_at	At3g58100	0,89	0,84	0,83	0,91	1,12	0,90	0,78	0,80 glycosyl hydrolase family protein 17
251575_at	At3g58120	1,88	1,83	1,86	1,05	1,14	0,92	1,18	1,53 bZIP transcription factor family protein
251573_at	At3g58140	1,11	1,02	0,95	0,88	0,75	1,19	1,11	1,00 phenylalanyl-tRNA synthetase class IIc family protein
251564_at	At3g58160	1,14	1,16	1,49	0,73	0,83	1,31	1,53	1,58 myosin heavy chain, putative
251579_at	At3g58170	0,90	0,88	0,96	0,97	0,96	0,98	0,95	0,95 Bet1-like SNARE 1-1 / Bet1 / Sft1-like SNARE 14a / BS14a (BET11)
251578_at	At3g58180	1,06	0,96	0,89	1,04	1,08	0,95	0,94	0,89 PBS lyase HEAT-like repeat-containing protein
251565_at	At3g58190	0,97	1,09	1,03	1,05	0,98	0,92	1,12	0,85 LOB domain protein 29 / lateral organ boundaries domain protein 29 (LBD2
251576_at	At3g58200	1,00	1,13	1,02	1,04	1,01	1,08	1,07	0,96 meprin and TRAF homology domain-containing protein / MATH domain-cor
251566_at	At3g58210	0,93	1,07	1,02	0,98	0,93	1,08	1,04	0,91 meprin and TRAF homology domain-containing protein / MATH domain-cor
251567_at	At3g58230	1,03	0,98	0,99	0,87	0,89	1,06	1,09	1,11 hypothetical protein
251568_at	At3g58280	0,96	1,04	0,95	1,03	0,99	1,00	1,04	0,96 hypothetical protein
251569_at	At3g58290	1,00	1,05	0,99	1,01	1,00	0,92	0,92	1,16 meprin and TRAF homology domain-containing protein / MATH domain-cor
251570_at	At3g58300	1,00	0,98	0,82	0,96	1,05	0,92	0,97	1,21 hypothetical protein
251571_at	At3g58330	0,97	0,96	1,11	0,91	1,04	0,95	0,99	1,10 hypothetical protein
251577_at	At3g58350	1,14	0,99	0,92	1,02	1,13	0,91	0,90	0,92 meprin and TRAF homology domain-containing protein / MATH domain-cor
251572_at	At3g58390	0,92	0,95	1,09	1,01	1,08	0,98	1,02	0,92 eukaryotic release factor 1 family protein / eRF1 family protein
251580_at	At3g58450	0,41	0,39	0,45	0,65	0,62	0,91	1,10	1,16 universal stress protein (USP) family protein
251533_at	At3g58460	0,95	0,76	0,75	0,86	0,92	0,90	0,86	0,92 rhomboid family protein / ubiquitin-associated (UBA)/TS-N domain-containi
251537_at	At3g58470	1,36	1,50	1,79	0,94	1,30	0,92	0,88	0,91 expressed protein
251534_at	At3g58510	0,97	0,95	0,95	1,03	1,19	0,85	1,03	0,93 DEAD box RNA helicase, putative (RH11)
251530_at	At3g58520	1,01	0,98	0,97	0,96	1,04	0,91	0,88	0,68 expressed protein
251532_at	At3g58530	0,84	0,68	1,02	0,95	1,01	1,05	0,99	0,91 F-box family protein-related
251535_at	At3g58540	0,99	0,90	0,95	0,96	0,97	0,99	1,05	0,98 expressed protein
251531_at	At3g58550	1,40	1,52	1,14	1,21	1,45	1,00	0,98	0,98 protease inhibitor/seed storage/lipid transfer protein (LTP) family protein
251581_at	At3g58560	1,23	1,22	1,14	1,11	1,05	1,06	0,97	0,98 endonuclease/exonuclease/phosphatase family protein
251529_at	At3g58570	0,94	1,06	1,22	0,94	0,88	1,02	1,01	1,07 DEAD box RNA helicase, putative
251582_at	At3g58580	1,20	1,17	1,43	1,23	1,13	1,17	0,98	1,05 endonuclease/exonuclease/phosphatase family protein
251583_at	At3g58590	0,92	1,30	1,06	0,87	0,83	1,01	0,93	0,94 pentatricopeptide (PPR) repeat-containing protein
251528_at	At3g58600	0,94	0,88	1,06	1,02	1,03	1,03	1,01	1,35 expressed protein
251536_at	At3g58610	1,09	0,98	1,01	0,98	0,85	0,95	0,90	0,99 ketol-acid reductoisomerase
251584_at	At3g58620	0,85	0,72	1,29	1,04	1,18	0,92	0,88	1,04 tetratricopeptide repeat (TPR)-containing protein
251585_at	At3g58630	0,85	0,74	0,66	1,00	1,07	0,96	0,85	0,92 expressed protein
251526_at	At3g58640	0,86	0,98	1,15	0,98	0,91	0,96	1,06	1,02 protein kinase family protein
251527_at	At3g58650	1,29	1,41	1,16	1,09	1,20	0,84	1,00	0,89 expressed protein
251538_at	At3g58660	1,15	0,94	1,01	1,02	1,01	0,97	0,91	0,95 60S ribosomal protein-related
251554_at	At3g58670	0,96	0,92	0,99	1,01	1,34	0,88	0,95	0,93 expressed protein
251551_at	At3g58680	0,89	0,84	0,79	0,81	0,88	0,97	1,01	1,09 ethylene-responsive transcriptional coactivator, putative
251539_at	At3g58690	0,77	0,78	0,93	0,83	0,90	0,92	0,91	0,99 protein kinase family protein
251552_at	At3g58700	1,20	1,08	0,96	1,49	1,43	0,88	0,87	0,88 60S ribosomal protein L11 (RPL11B)
251553_at	At3g58710	0,70	0,77	0,68	1,04	0,91	0,82	0,96	0,93 WRKY family transcription factor

251557_at	At3g58730	0,92	0,85	0,78	1,06	1,03	0,87	0,88	0,81	vacuolar ATP synthase subunit D (VATD) / V-ATPase D subunit / vacuolar
251540_at	At3g58740	0,82	0,96	0,88	0,99	1,17	1,03	0,92	0,97	citrate synthase, glyoxysomal, putative
251541_at	At3g58750	0,72	0,74	0,80	0,84	1,07	0,85	0,81	0,80	citrate synthase, glyoxysomal, putative
251542_at	At3g58760	0,83	0,71	0,71	0,84	0,92	0,89	0,94	0,93	ankyrin protein kinase, putative
251543_at	At3g58770	1,00	0,94	0,93	0,94	0,88	0,91	0,95	0,86	expressed protein
251555_at	At3g58780	1,00	1,00	0,94	0,84	1,04	1,06	0,89	0,96	agamous-like MADS box protein AGL1 / shatterproof 1 (AGL1) (SHP1)
251544_at	At3g58790	0,80	1,04	0,85	1,82	1,40	0,85	0,95	0,97	glycosyl transferase family 8 protein
251550_at	At3g58800	0,97	0,98	1,06	1,39	1,38	0,92	0,75	0,88	expressed protein
251545_at	At3g58810	0,99	0,96	0,82	0,98	1,33	1,19	0,86	1,02	zinc transporter, putative
251546_at	At3g58830	0,86	0,72	0,81	0,69	0,60	0,90	0,90	0,73	haloacid dehalogenase (HAD) superfamily protein
251556_at	At3g58840	1,11	1,15	0,97	1,06	1,13	1,07	1,27	1,18	expressed protein
251547_at	At3g58860	0,87	0,99	1,02	0,93	1,04	1,10	0,98	1,17	F-box family protein
251548_at	At3g58870	0,98	1,12	1,01	0,98	0,97	1,04	0,97	1,02	---
251549_at	At3g58890	1,01	1,01	0,96	0,95	0,90	1,03	0,84	0,80	syntaxin-related family protein
251508_at	At3g58970	0,77	0,73	0,94	0,85	1,16	0,96	0,91	1,12	magnesium transporter CorA-like family protein
251523_at	At3g58980	0,93	0,96	0,87	0,89	0,94	1,00	0,98	0,85	expressed protein
251524_at	At3g58990	1,47	2,80	3,39	1,91	2,32	0,84	0,80	0,83	aconitase C-terminal domain-containing protein
251525_at	At3g59000	0,97	1,06	1,06	0,82	0,87	0,94	0,95	1,01	F-box family protein
251509_at	At3g59010	0,87	1,03	0,95	1,03	1,14	0,95	0,82	0,99	pectinesterase family protein
251495_at	At3g59020	1,06	1,04	1,15	1,07	1,06	0,98	0,96	1,03	importin beta-2 subunit family protein
251504_at	At3g59030	1,04	1,02	1,01	0,94	1,11	0,93	0,96	0,94	transparent testa 12 protein (TT12) / multidrug transporter-like protein
251496_at	At3g59040	0,96	1,01	1,07	0,83	0,75	1,15	1,16	1,16	pentatricopeptide (PPR) repeat-containing protein
251505_at	At3g59050	1,08	1,02	1,38	1,53	1,34	0,96	0,95	1,14	amine oxidase family protein
251497_at	At3g59060	1,72	2,67	1,87	1,16	1,22	1,23	1,38	1,63	basic helix-loop-helix (bHLH) family protein
251498_at	At3g59070	1,01	0,98	0,87	1,02	1,01	1,01	0,98	1,19	auxin-responsive protein, putative
251507_at	At3g59080	0,85	0,63	0,62	0,67	0,88	0,99	1,02	1,10	aspartyl protease family protein
251506_at	At3g59090	1,03	1,09	1,07	0,90	1,02	1,21	1,02	1,11	expressed protein
251499_at	At3g59100	1,11	1,18	1,35	1,08	1,25	1,01	1,22	1,00	glycosyl transferase family 48 protein
251500_at	At3g59110	0,98	1,30	1,69	0,74	0,64	0,88	1,10	1,21	protein kinase family protein
251501_at	At3g59120	0,90	0,96	1,00	1,15	1,14	0,91	0,91	0,99	DC1 domain-containing protein
251502_at	At3g59130	0,91	1,01	1,07	0,99	0,93	0,99	1,03	0,82	DC1 domain-containing protein
251503_at	At3g59140	0,59	0,55	0,58	0,80	0,68	1,08	1,26	1,30	ABC transporter family protein
251510_at	At3g59170	1,00	0,97	0,96	1,07	1,10	0,93	1,08	0,92	F-box family protein
251511_at	At3g59180	0,96	0,90	0,96	0,94	1,06	1,00	1,00	0,94	hypothetical protein
251512_at	At3g59190	1,08	1,13	1,07	1,00	1,27	1,15	1,07	0,89	F-box family protein
251513_at	At3g59220	0,93	1,01	0,90	1,14	1,04	1,01	1,10	1,21	pirin, putative
251514_at	At3g59260	1,09	0,88	0,95	0,88	1,11	1,03	0,97	1,08	pirin, putative
251515_at	At3g59270	1,04	0,89	0,74	0,94	1,30	1,01	1,01	1,25	syntaxin-related family protein
251492_at	At3g59280	1,07	0,96	0,98	0,89	1,02	1,05	1,09	1,12	signaling molecule-related
251468_at	At3g59290	0,90	0,86	0,80	0,86	0,98	1,01	0,88	0,88	epsin N-terminal homology (ENTH) domain-containing protein
251493_at	At3g59300	0,88	1,02	0,76	0,87	0,84	1,03	0,97	0,97	expressed protein
251516_s_at	At3g59310	0,56	0,69	0,83	0,63	0,50	0,80	1,28	1,36	expressed protein
251466_at	At3g59340	1,03	0,93	0,63	1,13	1,10	1,11	0,96	0,94	expressed protein
251494_at	At3g59350	0,64	0,68	1,10	0,86	0,94	1,03	0,99	1,24	serine/threonine protein kinase, putative
251465_at	At3g59360	0,96	0,90	0,88	1,12	0,96	0,88	0,77	0,88	nucleotide-sugar transporter family protein
251517_at	At3g59370	0,97	0,99	0,79	1,12	1,33	1,11	1,08	0,99	expressed protein

251464_at	At3g59380	1,13	1,18	1,29	1,29	1,11	0,89	0,91	1,03	farnesyltransferase alpha subunit, putative / FTA, putative / protein farnesyl
251518_at	At3g59390	1,26	1,15	1,14	1,05	0,92	0,91	1,15	1,07	expressed protein
251519_at	At3g59400	1,27	1,21	0,95	1,19	1,14	1,17	0,96	1,17	expressed protein
251520_at	At3g59410	0,77	0,74	0,66	0,79	0,72	0,91	1,06	1,01	protein kinase family protein
251521_at	At3g59420	0,74	0,87	0,79	0,80	0,86	0,90	0,84	0,93	receptor protein kinase, putative (ACR4)
251522_at	At3g59430	1,08	1,08	1,17	0,87	1,02	0,95	0,93	0,89	expressed protein
251488_at	At3g59440	1,01	1,08	0,95	1,10	1,21	0,99	0,99	0,94	calcium-binding protein, putative
251489_at	At3g59460	1,05	1,13	1,13	1,06	0,94	1,04	1,00	1,08	hypothetical protein
251467_at	At3g59470	1,06	1,23	1,16	0,87	0,92	1,00	1,04	1,40	far-red impaired responsive family protein / FAR1 family protein
251491_at	At3g59480	1,11	1,41	1,08	0,97	0,81	1,11	1,12	1,01	pfkB-type carbohydrate kinase family protein
251490_at	At3g59490	0,87	0,92	1,23	0,81	0,92	0,97	0,83	1,05	expressed protein
251469_at	At3g59530	0,87	0,94	0,74	0,97	1,19	1,15	1,07	0,96	strictosidine synthase family protein
251486_at	At3g59540	1,47	1,18	1,08	1,38	1,26	1,00	1,00	0,91	60S ribosomal protein L38 (RPL38B)
251485_at	At3g59550	1,36	1,19	1,02	1,21	1,28	1,03	0,93	1,12	cohesion family protein SYN3 (SYN3)
251470_at	At3g59560	0,84	0,84	0,83	0,88	0,90	0,97	1,01	0,87	RabGAP/TBC domain-containing protein
251471_at	At3g59570	0,90	0,88	0,93	1,04	0,91	1,01	1,09	0,90	RabGAP/TBC domain-containing protein
251472_at	At3g59580	0,96	0,95	0,81	0,84	0,86	0,95	1,04	0,87	RWP-RK domain-containing protein
251473_at	At3g59610	0,93	0,99	1,01	0,98	1,11	1,04	0,90	1,00	F-box family protein / jacalin lectin family protein
251474_at	At3g59630	1,21	1,15	1,14	0,92	1,10	1,17	1,04	1,13	diphthamide synthesis DPH2 family protein
251484_at	At3g59640	0,94	0,94	1,25	0,97	0,98	1,05	0,94	1,02	glycine-rich protein
251483_at	At3g59650	1,14	1,03	0,80	1,22	1,20	1,05	0,84	0,88	mitochondrial ribosomal protein L51/S25/CI-B8 family protein
251475_at	At3g59660	1,19	1,19	1,12	0,88	1,04	1,07	1,34	1,32	C2 domain-containing protein / GRAM domain-containing protein
251476_at	At3g59670	1,37	1,33	0,89	1,17	0,75	1,31	1,16	0,78	expressed protein
251477_at	At3g59680	1,18	1,20	1,02	1,05	1,05	1,01	1,04	0,98	expressed protein
251478_at	At3g59690	0,97	1,15	1,03	0,85	1,20	0,99	1,08	1,17	calmodulin-binding family protein
251479_at	At3g59700	0,70	0,51	0,65	1,24	1,13	0,89	0,92	1,01	lectin protein kinase, putative
251480_at	At3g59710	1,03	0,87	0,97	0,96	1,19	0,93	1,05	0,97	short-chain dehydrogenase/reductase (SDR) family protein
251481_at	At3g59730	0,94	1,03	1,05	0,98	1,18	1,03	1,01	0,90	receptor lectin kinase, putative
251482_s_at	At3g59740	1,02	0,83	0,89	0,92	0,89	1,00	1,50	1,19	receptor lectin kinase 3 (lecRK3)
251487_at	At3g59760	0,99	0,71	0,76	1,12	0,95	1,00	1,04	0,88	cysteine synthase, mitochondrial, putative / O-acetylserine (thiol)-lyase, put
251460_at	At3g59770	0,88	0,77	0,75	1,01	1,11	0,92	0,92	0,80	sacI homology domain-containing protein / WW domain-containing protein
251461_at	At3g59780	0,97	0,87	0,82	0,75	0,78	1,20	1,10	1,18	expressed protein
251462_at	At3g59790	1,02	0,97	0,96	0,91	1,08	1,09	0,95	1,06	mitogen-activated protein kinase, putative / MAPK, putative (MPK10)
251463_at	At3g59800	1,19	0,90	1,14	0,94	1,04	1,18	1,00	1,12	expressed protein
251447_at	At3g59810	1,39	1,26	1,25	1,26	1,48	1,14	0,97	1,04	small nuclear ribonucleoprotein F, putative / U6 snRNA-associated Sm-like
251432_at	At3g59820	1,17	0,86	0,87	0,83	0,78	1,01	1,07	1,25	calcium-binding mitochondrial protein-related
251433_at	At3g59830	0,90	0,69	0,99	1,04	1,03	1,05	0,80	1,09	ankyrin protein kinase, putative
251446_at	At3g59840	1,20	0,91	1,01	1,36	1,28	0,78	0,75	0,77	expressed protein
251448_at	At3g59845	1,06	1,04	1,00	1,10	0,93	0,95	1,13	0,88	NADP-dependent oxidoreductase, putative
251434_at	At3g59850	0,99	1,03	1,16	1,11	1,28	1,00	1,06	1,05	polygalacturonase, putative / pectinase, putative
251435_at	At3g59860	0,99	0,93	1,03	0,93	0,99	1,05	0,90	0,94	Mutator-like transposase family
251445_at	At3g59870	1,01	0,82	0,83	0,96	0,92	1,09	1,03	1,00	expressed protein
251436_at	At3g59900	1,09	1,04	1,39	1,28	1,52	0,81	0,73	1,06	expressed protein
251437_at	At3g59910	1,11	1,05	1,01	0,92	0,92	1,15	1,02	1,04	expressed protein
251449_at	At3g59920	1,07	0,92	0,94	1,09	1,30	0,91	0,92	0,97	Rab GDP dissociation inhibitor (GDI2)
251438_s_at	At3g59930	0,89	0,58	0,33	0,92	0,93	0,58	0,61	0,66	expressed protein

251443_at	At3g59940	0,92	0,98	0,83	0,70	0,60	0,99	1,08	1,08	kelch repeat-containing F-box family protein
251439_at	At3g59950	1,09	1,00	1,26	0,93	0,87	1,08	1,02	1,22	autophagy 4b (APG4b)
251442_at	At3g59980	0,94	0,85	0,79	0,75	0,76	1,07	0,95	0,88	tRNA-binding region domain-containing protein
251444_at	At3g59990	1,26	1,16	1,25	0,99	1,02	1,08	1,04	1,10	methionyl aminopeptidase, putative / methionine aminopeptidase, putative /
251440_at	At3g60010	0,98	1,08	1,06	1,10	1,04	1,08	0,97	1,01	E3 ubiquitin ligase SCF complex subunit SKP1/ASK1 (At13), putative
251441_at	At3g60020	0,95	0,98	1,04	0,96	1,04	1,00	1,03	0,93	E3 ubiquitin ligase SCF complex subunit SKP1/ASK1 (At5), putative
251450_at	At3g60030	0,93	0,84	0,99	1,01	0,92	0,99	0,97	1,12	squamosa promoter-binding protein-like 12 (SPL12)
251451_s_at	At3g60040	0,98	1,07	0,96	0,97	1,10	1,11	1,02	1,04	F-box family protein
251452_at	At3g60060	0,97	1,00	0,88	0,97	0,96	1,07	0,98	1,03	hypothetical protein
251453_at	At3g60070	1,22	1,17	0,99	1,08	0,92	1,25	1,14	1,12	lactose permease-related
251454_at	At3g60080	0,96	0,91	1,09	1,55	1,43	1,00	0,88	0,97	zinc finger (C3HC4-type RING finger) family protein
251455_at	At3g60100	1,02	1,11	0,86	0,97	1,00	1,01	0,96	0,85	citrate synthase, mitochondrial, putative
251430_at	At3g60110	0,99	1,01	1,03	0,91	0,99	1,05	1,09	1,02	DNA-binding bromodomain-containing protein
251456_at	At3g60120	1,11	1,24	1,16	0,97	0,94	1,31	1,08	0,91	glycosyl hydrolase family 1 protein
251427_at	At3g60130	0,95	0,90	1,00	1,12	1,05	0,85	0,90	0,84	glycosyl hydrolase family 1 protein / beta-glucosidase, putative (YLS1)
251428_at	At3g60140	0,85	1,29	2,21	0,77	0,72	2,28	2,23	1,64	glycosyl hydrolase family 1 protein
251457_s_at	At3g60160	1,10	1,23	1,08	0,90	0,82	1,03	1,13	1,14	ABC transporter family protein
251458_at	At3g60170	1,03	1,02	0,99	0,90	1,14	0,95	1,04	1,19	---
251426_at	At3g60180	0,88	0,88	0,89	1,00	0,92	1,03	1,02	1,03	uridylate kinase, putative / uridine monophosphate kinase, putative / UMP k
251429_at	At3g60190	0,92	0,94	0,94	0,96	0,80	1,00	1,05	1,05	dynamin-like protein E (DL1E)
251459_at	At3g60200	0,76	0,84	0,78	0,97	1,24	0,85	0,77	0,94	expressed protein
251425_at	At3g60210	1,27	1,18	1,04	0,86	0,98	1,07	0,98	0,99	chloroplast chaperonin 10, putative
251412_at	At3g60220	0,91	1,05	0,90	1,06	0,95	1,00	0,90	0,91	zinc finger (C3HC4-type RING finger) family protein (ATL4)
251431_at	At3g60240	0,97	0,88	1,01	0,83	0,99	1,02	1,01	1,06	MIF4G domain-containing protein / MA3 domain-containing protein
251409_at	At3g60245	1,16	1,09	1,00	1,16	1,05	1,00	0,98	1,00	60S ribosomal protein L37a (RPL37aC)
251411_at	At3g60250	1,02	0,93	1,21	1,03	1,15	0,91	1,05	1,16	casein kinase II beta chain, putative (CKB3)
251406_at	At3g60260	0,68	0,67	1,01	1,02	1,16	0,91	1,08	1,42	phagocytosis and cell motility protein ELMO1-related
251401_at	At3g60270	1,22	1,20	1,45	1,11	1,14	0,89	1,03	0,98	uclacyanin, putative
251410_at	At3g60280	0,97	1,01	1,03	0,87	0,94	1,12	1,01	0,83	uclacyanin 3 (UCC3)
251402_at	At3g60290	0,78	0,62	0,71	0,65	0,59	0,84	0,81	0,88	oxidoreductase, 2OG-Fe(II) oxygenase family protein
251403_at	At3g60300	0,99	1,09	1,11	0,74	0,99	1,05	0,97	1,24	RWD domain-containing protein
251404_at	At3g60310	1,03	1,11	1,09	0,92	0,94	0,95	0,99	0,99	expressed protein
251413_at	At3g60320	1,00	1,21	1,58	0,85	0,79	0,99	0,98	1,01	expressed protein
251405_at	At3g60330	1,16	1,09	1,14	1,10	1,31	1,23	1,00	0,89	ATPase, plasma membrane-type, putative / proton pump, putative
251408_at	At3g60340	0,93	0,99	1,03	0,82	0,96	0,96	1,02	1,04	palmitoyl protein thioesterase family protein
251407_at	At3g60350	1,01	0,88	1,06	0,70	0,86	0,95	0,93	0,91	armadillo/beta-catenin repeat family protein / F-box family protein
251371_at	At3g60360	1,27	1,02	1,17	0,74	1,07	1,04	0,92	1,19	expressed protein
251414_at	At3g60370	1,10	0,97	0,98	0,98	0,83	1,17	0,94	0,96	immunophilin / FKBP-type peptidyl-prolyl cis-trans isomerase family protein
251415_at	At3g60380	0,81	0,81	0,80	0,65	0,89	1,01	0,83	0,85	expressed protein
251374_at	At3g60390	1,38	1,35	1,06	0,83	1,05	1,15	1,00	1,07	homeobox-leucine zipper protein 3 (HAT3) / HD-ZIP protein 3
251416_at	At3g60400	0,90	1,22	0,92	1,21	1,05	0,95	0,94	0,88	mitochondrial transcription termination factor-related / mTERF-related
251375_at	At3g60410	0,86	0,94	1,15	0,89	1,03	1,08	1,10	1,29	expressed protein
251400_at	At3g60420	0,64	0,51	0,81	0,72	0,88	0,97	0,92	1,08	expressed protein
251417_at	At3g60430	0,91	1,03	0,95	1,06	0,91	1,06	1,15	1,00	hypothetical protein
251418_at	At3g60440	0,93	0,97	0,85	0,95	0,98	1,14	0,97	0,70	expressed protein
251370_at	At3g60450	1,60	1,20	1,27	1,97	1,53	1,03	1,11	1,28	expressed protein

251419_at	At3g60470	1,06	0,89	0,95	1,16	0,96	1,00	1,07	1,24	hypothetical protein
251369_at	At3g60480	1,06	0,99	0,99	1,07	0,95	1,05	1,00	1,02	expressed protein
251420_at	At3g60490	0,96	1,04	0,99	0,94	1,27	0,95	0,95	1,10	AP2 domain-containing transcription factor TINY, putative
251421_at	At3g60510	0,87	0,91	1,14	0,87	0,81	0,92	0,95	0,87	enoyl-CoA hydratase/isomerase family protein
251372_at	At3g60520	0,66	0,87	1,06	0,78	0,83	0,75	0,92	0,93	expressed protein
251373_at	At3g60530	0,77	1,09	1,15	0,76	0,83	0,53	0,68	0,71	zinc finger (GATA type) family protein
251422_at	At3g60540	1,00	0,82	0,74	1,02	1,04	1,07	0,83	0,86	sec61beta family protein
251423_at	At3g60550	1,03	1,05	1,07	1,03	0,96	1,07	1,02	0,84	cyclin family protein
251424_at	At3g60560	0,97	0,99	0,99	0,93	1,07	0,96	1,05	0,94	hypothetical protein
251397_at	At3g60570	0,98	1,07	1,04	0,97	1,08	0,99	0,90	0,89	beta-expansin, putative (EXPB5)
251398_at	At3g60580	1,04	0,95	1,04	0,98	1,14	1,00	1,05	0,99	zinc finger (C2H2 type) family protein
251399_at	At3g60590	0,96	1,03	1,33	0,95	0,89	1,11	0,95	1,28	expressed protein
251338_at	At3g60600	0,89	0,84	0,99	1,05	1,30	0,90	0,88	0,93	vesicle-associated membrane protein, putative / VAMP, putative
251343_at	At3g60620	0,99	0,99	0,83	0,99	1,01	1,09	0,88	0,77	phosphatidate cytidylyltransferase family protein
251376_at	At3g60630	0,80	0,76	0,93	0,93	1,21	0,82	0,82	0,95	scarecrow transcription factor family protein
251393_at	At3g60640	1,32	1,31	1,41	0,92	1,14	1,02	1,11	1,07	autophagy 8g (APG8g)
251377_at	At3g60650	1,05	1,01	0,95	1,15	1,04	0,97	0,94	1,00	expressed protein
251378_at	At3g60660	1,07	1,05	1,05	1,11	1,15	0,89	0,87	0,90	expressed protein
251379_at	At3g60680	0,97	0,94	0,82	1,03	0,99	0,88	0,85	0,98	expressed protein
251342_at	At3g60690	0,68	0,84	0,93	0,68	0,68	1,18	0,99	1,30	auxin-responsive family protein
251380_at	At3g60700	1,16	1,13	0,99	0,93	0,86	1,09	0,91	0,96	expressed protein
251381_at	At3g60720	1,18	1,07	0,97	1,10	0,89	1,08	1,07	1,00	receptor-like protein kinase-related
251382_at	At3g60730	0,95	1,02	1,05	1,04	1,04	0,98	0,96	0,98	pectinesterase family protein
251383_at	At3g60740	0,72	0,81	0,81	1,11	0,74	0,93	0,89	0,79	tubulin folding cofactor D
251396_at	At3g60750	0,90	0,86	0,95	0,87	0,80	1,15	1,07	1,09	transketolase, putative
251384_at	At3g60760	0,88	1,05	1,86	0,84	1,02	0,91	1,21	1,32	hypothetical protein
251341_at	At3g60770	1,20	1,12	1,10	1,12	1,15	0,98	0,94	0,95	40S ribosomal protein S13 (RPS13A)
251339_at	At3g60780	1,01	0,98	1,02	0,98	0,98	0,96	1,07	0,92	expressed protein
251385_at	At3g60790	1,04	0,81	1,00	1,10	1,11	0,92	0,89	1,24	F-box protein-related
251386_at	At3g60800	0,83	0,78	0,89	1,12	1,06	0,94	0,90	1,02	zinc finger (DHHC type) family protein
251387_at	At3g60810	0,63	0,56	0,56	0,83	0,96	0,84	0,93	0,87	expressed protein
251337_at	At3g60820	1,18	1,03	0,98	1,12	1,11	0,97	0,92	0,97	20S proteasome beta subunit F1 (PBF1)
251340_at	At3g60830	1,29	1,38	1,30	1,04	0,97	1,11	0,99	0,99	actin-related protein 7 (ARP7)
251388_at	At3g60840	1,21	1,22	1,18	1,03	1,12	0,93	1,01	0,89	microtubule associated protein (MAP65/ASE1) family protein
251389_at	At3g60850	0,83	0,93	0,85	0,79	1,00	0,86	0,99	1,05	expressed protein
251390_at	At3g60860	0,98	0,88	1,02	0,97	1,18	0,93	0,90	0,91	guanine nucleotide exchange family protein
251392_at	At3g60880	1,15	1,03	1,00	0,91	0,93	0,97	0,98	1,05	dihydrodipicolinate synthase 1 (DHDPS1) (DHDPS) (DHPS1)
251394_at	At3g60900	1,02	1,01	0,78	1,16	1,06	0,92	0,89	0,82	fasciclin-like arabinogalactan-protein (FLA10)
251391_at	At3g60910	0,82	0,80	0,77	1,07	1,09	0,78	0,74	0,89	expressed protein
251344_at	At3g60920	0,96	0,97	1,08	1,03	1,22	1,13	0,89	1,11	beige/BEACH domain-containing protein
251345_at	At3g60940	1,05	0,92	0,98	1,06	1,16	0,80	0,88	0,81	expressed protein
251346_at	At3g60980	1,37	1,18	1,17	1,23	1,56	0,89	0,79	0,76	pentatricopeptide (PPR) repeat-containing protein
251347_at	At3g61000	0,98	0,78	0,83	0,84	0,71	0,98	1,29	0,99	glycosyl hydrolase family protein 85
251348_at	At3g61010	0,98	1,01	1,00	0,99	0,90	0,96	1,12	1,15	glycosyl hydrolase family protein 85
251349_s_at	At3g61020	0,73	0,54	0,58	0,95	1,14	0,88	0,97	0,97	expressed protein
251350_at	At3g61040	0,92	1,06	1,05	1,07	0,98	1,15	1,03	0,97	cytochrome P450 family protein

251351_s_at	At3g61050	0,95	0,86	0,97	1,07	1,25	1,03	0,89	0,90	C2 domain-containing protein
251356_at	At3g61060	1,13	1,61	1,36	0,69	0,60	1,57	2,11	1,63	F-box family protein / lectin-related
251352_at	At3g61070	0,68	0,76	0,86	0,83	0,85	0,83	0,86	0,86	peroxisomal biogenesis factor 11 family protein / PEX11 family protein
251353_at	At3g61080	0,58	0,56	0,42	0,70	0,57	0,87	0,86	0,89	fructosamine kinase family protein
251354_at	At3g61090	1,00	1,04	1,17	1,05	1,14	1,07	1,04	0,97	expressed protein
251355_at	At3g61100	1,65	1,38	1,30	1,03	1,02	1,00	0,98	0,85	expressed protein
251357_at	At3g61110	1,54	1,30	1,19	1,52	1,65	0,98	0,98	0,95	40S ribosomal protein S27 (ARS27A)
251312_at	At3g61120	1,01	1,03	1,08	0,99	0,97	0,95	0,99	0,93	MADS-box protein (AGL13)
251308_at	At3g61130	0,86	0,63	0,64	0,97	1,05	0,96	0,77	0,82	glycosyl transferase family 8 protein
251311_at	At3g61140	1,17	1,12	1,20	0,97	1,06	1,06	0,98	1,05	COP9 signalosome complex subunit 1 / CSN complex subunit 1 (CSN1) / C
251310_at	At3g61150	0,82	0,76	0,65	0,78	0,80	0,81	0,75	0,67	homeobox-leucine zipper family protein / homeodomain GLABRA2 like prot
251358_at	At3g61160	0,57	0,98	1,10	0,70	0,62	1,00	1,09	1,06	shaggy-related protein kinase beta / ASK-beta (ASK2)
251359_at	At3g61170	0,86	1,01	0,91	0,93	1,13	1,08	1,03	0,99	pentatricopeptide (PPR) repeat-containing protein
251314_at	At3g61180	0,93	1,06	1,11	0,83	0,86	1,01	1,00	1,03	zinc finger (C3HC4-type RING finger) family protein
251336_at	At3g61190	1,24	1,36	1,08	1,10	1,47	1,80	1,54	1,22	BON1-associated protein 1 (BAP1)
251307_at	At3g61200	1,19	1,07	0,92	1,04	0,84	1,06	0,88	0,87	thioesterase family protein
251360_at	At3g61210	1,98	1,88	2,18	1,21	1,57	0,97	1,02	1,39	embryo-abundant protein-related
251309_at	At3g61220	1,62	1,67	1,83	1,05	0,74	1,62	1,70	1,37	short-chain dehydrogenase/reductase (SDR) family protein
251361_at	At3g61230	0,94	1,07	0,97	0,93	1,20	1,11	0,97	1,02	LIM domain-containing protein
251362_at	At3g61240	1,21	1,16	1,24	1,14	1,18	1,01	0,90	1,00	DEAD/DEAH box helicase, putative (RH12)
251363_at	At3g61250	1,21	0,97	1,02	1,20	1,33	1,09	0,78	0,91	myb family transcription factor (MYB17)
251306_at	At3g61260	0,84	0,86	0,84	0,79	0,92	1,04	1,09	1,25	DNA-binding family protein / remorin family protein
251364_at	At3g61300	0,94	1,00	0,99	1,13	1,05	1,13	0,90	0,98	C2 domain-containing protein
251365_at	At3g61310	0,98	1,10	0,93	1,21	1,16	0,78	0,73	0,80	DNA-binding family protein
251366_at	At3g61340	0,97	0,99	1,03	0,96	1,13	1,09	0,94	1,04	F-box family protein
251367_at	At3g61350	0,85	0,89	0,87	1,05	1,31	0,96	1,00	0,83	SKP1 interacting partner 4 (SKIP4)
251313_at	At3g61360	0,90	1,23	1,00	1,00	1,15	0,88	0,94	0,99	pentatricopeptide (PPR) repeat-containing protein
251368_at	At3g61380	1,21	1,66	1,17	1,67	0,98	1,08	1,64	0,90	expressed protein
251334_at	At3g61390	1,00	1,03	1,04	1,76	1,36	1,05	1,08	1,06	U-box domain-containing protein
251335_at	At3g61400	1,10	0,99	0,98	0,98	1,08	0,96	1,00	1,14	2-oxoglutarate-dependent dioxygenase, putative
251315_at	At3g61410	1,02	1,07	0,98	1,03	0,98	0,99	0,86	0,89	hypothetical protein
251324_at	At3g61430	1,26	1,38	1,60	2,11	1,91	1,00	0,92	0,97	plasma membrane intrinsic protein 1A (PIP1A) / aquaporin PIP1.1 (PIP1.1)
251322_at	At3g61440	1,23	1,17	1,26	1,31	1,45	0,96	0,94	0,94	cysteine synthase, putative / O-acetylserine (thiol)-lyase, putative / O-acetyl
251329_at	At3g61450	1,09	1,12	1,10	0,95	1,16	1,05	0,99	1,11	syntaxin 73 (SYP73)
251321_at	At3g61460	0,98	1,06	0,92	1,07	1,07	0,90	0,86	0,99	zinc finger (C3HC4-type RING finger) family protein (BRH1)
251325_s_at	At3g61470	0,92	0,90	0,95	0,87	0,85	1,00	0,98	0,97	chlorophyll A-B binding protein (LHCA2)
251316_s_at	At3g61480	0,89	0,85	1,04	0,78	0,94	1,01	0,95	0,85	expressed protein
251317_at	At3g61490	1,21	1,27	1,26	2,29	1,47	0,88	0,93	1,01	glycoside hydrolase family 28 protein / polygalacturonase (pectinase) family
251320_at	At3g61530	1,04	1,01	1,03	0,85	0,90	0,90	1,03	0,90	ketopantoate hydroxymethyltransferase family protein
251327_at	At3g61540	0,88	0,74	0,96	0,99	0,94	1,01	0,96	0,97	peptidase family protein
251330_at	At3g61550	0,92	0,93	0,91	0,84	0,75	0,94	0,90	0,91	zinc finger (C3HC4-type RING finger) family protein
251318_at	At3g61570	1,02	0,87	1,07	0,94	0,88	1,18	1,28	1,49	intracellular protein transport protein USO1-related
251323_at	At3g61580	0,96	0,98	1,22	1,06	1,00	0,97	0,84	1,00	delta-8 sphingolipid desaturase (SLD1)
251326_at	At3g61590	0,89	0,99	0,91	1,09	1,11	0,72	0,93	0,84	F-box family protein
251328_at	At3g61600	1,07	1,20	1,31	1,07	1,01	1,02	1,03	1,33	BTB/POZ domain-containing protein
251319_at	At3g61610	1,19	1,12	1,19	1,79	1,80	1,04	0,83	0,78	aldose 1-epimerase family protein

251288_at	At3g61620	1,11	0,92	1,32	0,96	0,96	1,08	0,95	0,90	exonuclease RRP41 (RRP41)
251282_at	At3g61630	1,67	1,27	1,51	0,92	1,10	1,27	0,92	0,94	AP2 domain-containing transcription factor, putative
251281_at	At3g61640	0,85	0,74	0,79	1,05	0,95	1,05	1,18	1,40	arabinogalactan-protein (AGP20)
251331_s_at	At3g61650	1,16	1,03	1,14	0,96	0,83	0,94	0,91	0,84	tubulin gamma-1 chain / gamma-1 tubulin (TUBG1)
251332_at	At3g61670	0,91	0,93	1,11	0,87	1,06	0,98	1,10	1,42	expressed protein
251333_at	At3g61690	0,92	1,02	1,18	0,67	0,80	1,04	1,15	1,20	expressed protein
251274_at	At3g61700	0,73	0,73	0,77	0,79	0,99	0,91	1,01	1,05	expressed protein
251286_at	At3g61710	1,35	1,36	1,38	1,16	0,98	1,13	1,07	1,26	autophagy protein Apg6 family
251275_at	At3g61720	1,02	1,12	1,03	1,09	1,11	1,02	0,99	0,85	C2 domain-containing protein
251276_at	At3g61740	1,24	1,21	1,01	0,78	0,97	0,95	1,06	1,04	PHD finger family protein (ATX3)
251277_at	At3g61760	0,96	0,91	0,82	1,03	0,89	0,74	0,85	0,91	dynamin-like protein B (DL1B)
251285_at	At3g61770	1,12	0,93	0,95	1,12	0,70	1,01	0,96	0,86	expressed protein
251278_at	At3g61780	1,35	1,28	1,34	0,84	0,57	1,08	1,04	1,20	expressed protein
251283_at	At3g61790	0,94	0,91	0,93	1,27	1,05	0,88	0,80	0,84	seven in absentia (SINA) family protein
251279_at	At3g61800	0,80	0,82	0,92	0,88	1,22	1,16	1,05	1,11	expressed protein
251280_at	At3g61810	0,97	1,03	1,08	0,96	0,98	0,99	1,07	1,21	glycosyl hydrolase family 17 protein
251287_at	At3g61820	1,09	1,16	1,27	1,53	1,46	0,65	0,62	0,62	aspartyl protease family protein
251289_at	At3g61830	0,71	0,95	1,25	0,89	1,10	0,94	1,20	1,01	transcriptional factor B3 family protein / auxin-responsive factor AUX/IAA-re
251284_at	At3g61840	1,03	0,98	0,98	1,10	1,17	0,99	0,97	0,85	expressed protein
251290_at	At3g61850	1,08	1,30	1,01	1,28	1,21	1,16	1,16	1,20	Dof zinc finger protein DAG1 / Dof affecting germination 1 (DAG1) / transcri
251242_at	At3g61860	1,19	1,22	1,46	0,94	0,92	1,11	1,14	1,26	arginine/serine-rich splicing factor RSP31 (RSP31)
251243_at	At3g61870	1,06	1,06	1,03	0,56	0,64	0,83	0,82	0,83	expressed protein
251301_at	At3g61880	1,23	1,22	1,19	0,99	1,09	1,03	0,95	1,07	cytochrome P450, putative
251272_at	At3g61890	0,84	0,86	1,01	1,14	1,02	1,15	1,25	1,02	homeobox-leucine zipper protein 12 (HB-12) / HD-ZIP transcription factor 1
251291_at	At3g61900	0,96	1,14	0,91	1,18	1,11	1,12	0,91	0,91	auxin-responsive family protein
251303_at	At3g61910	0,97	1,04	0,91	0,89	1,03	0,90	0,94	0,94	no apical meristem (NAM) family protein
251292_at	At3g61920	0,98	1,02	1,02	1,11	1,09	1,01	0,90	1,07	expressed protein
251293_at	At3g61930	1,38	1,53	1,25	0,94	1,06	1,01	0,93	0,92	expressed protein
251294_at	At3g61940	1,05	1,00	0,93	1,00	1,09	1,04	0,98	0,92	zinc transporter, putative
251299_at	At3g61950	0,97	0,95	1,00	0,95	1,13	0,92	0,94	1,11	basic helix-loop-helix (bHLH) family protein
251273_at	At3g61960	1,30	1,45	1,38	0,93	1,07	1,22	1,15	1,16	protein kinase family protein
251302_at	At3g61970	0,88	0,96	1,12	0,96	0,96	0,84	0,96	0,90	DNA-binding protein, putative
251300_at	At3g61980	0,82	0,75	0,79	0,94	1,06	1,01	1,13	1,38	serine protease inhibitor, Kazal-type family protein
251304_at	At3g61990	0,74	0,79	0,72	1,74	1,22	1,05	1,06	1,12	O-methyltransferase family 3 protein
251295_at	At3g62000	0,84	0,81	0,93	0,84	0,92	1,09	1,05	1,00	O-methyltransferase family 3 protein
251296_at	At3g62010	0,75	0,84	1,33	0,89	0,78	0,95	1,50	1,58	expressed protein
251297_at	At3g62020	1,33	1,20	1,20	1,43	1,49	0,81	0,87	0,90	germin-like protein (GLP10)
251305_at	At3g62030	1,00	0,89	0,90	0,87	0,91	1,02	1,01	1,06	peptidyl-prolyl cis-trans isomerase, chloroplast / cyclophilin / rotamase / cyc
251298_at	At3g62040	1,23	1,33	1,12	1,69	1,48	0,80	1,16	0,97	haloacid dehalogenase-like hydrolase family protein
251271_at	At3g62050	1,06	1,04	1,01	0,92	1,09	0,93	1,02	0,87	expressed protein
251244_at	At3g62060	1,32	1,18	1,06	1,20	1,12	0,86	0,98	0,78	pectinacetylesterase family protein
251262_at	At3g62080	0,88	0,92	1,03	0,93	0,73	1,00	1,09	1,10	SNF7 family protein
251245_at	At3g62090	0,89	1,02	0,89	0,98	1,03	1,10	1,03	0,94	basic helix-loop-helix (bHLH) protein, putative
251246_at	At3g62100	1,01	1,12	1,03	0,97	1,01	0,99	0,92	1,00	auxin-responsive protein, putative
251261_at	At3g62110	0,89	0,61	0,72	0,79	0,70	0,81	0,74	0,54	glycoside hydrolase family 28 protein / polygalacturonase (pectinase) family
251264_at	At3g62120	1,21	1,13	1,02	1,07	0,91	1,07	1,04	1,03	tRNA synthetase class II (G, H, P and S) family protein

251260_at	At3g62130	1,35	1,51	1,53	0,93	0,90	1,01	1,07	1,09	epimerase-related
251247_at	At3g62140	1,33	1,41	1,24	1,15	1,20	1,17	1,01	1,24	expressed protein
251248_at	At3g62150	0,77	0,73	1,05	0,75	0,80	0,90	0,87	0,91	multidrug resistant (MDR) ABC transporter, putative
251249_at	At3g62160	1,40	1,59	1,28	1,23	1,31	0,77	0,95	0,99	transferase family protein
251258_at	At3g62170	0,99	0,91	0,98	1,05	1,05	0,97	1,00	1,11	pectinesterase family protein
251250_at	At3g62180	1,02	1,07	1,05	1,00	1,08	1,04	0,92	1,05	invertase/pectin methylesterase inhibitor family protein
251263_at	At3g62190	1,53	1,24	1,17	1,06	1,04	1,08	1,09	1,04	DNAJ heat shock N-terminal domain-containing protein
251251_at	At3g62220	0,85	0,84	0,97	1,04	1,09	1,07	1,23	1,43	serine/threonine protein kinase, putative
251252_at	At3g62230	0,99	0,83	0,82	0,99	0,99	1,03	1,11	0,91	F-box family protein
251253_at	At3g62240	1,07	1,10	1,02	0,88	0,91	1,00	1,15	0,90	zinc finger (C2H2 type) family protein
251259_at	At3g62260	0,88	1,90	1,34	0,94	0,81	1,20	2,09	1,75	protein phosphatase 2C, putative / PP2C, putative
251254_at	At3g62270	0,65	0,56	0,40	1,07	0,92	1,01	1,31	1,17	anion exchange family protein
251255_at	At3g62280	0,93	1,04	1,02	1,03	1,38	0,95	1,02	1,03	GDSL-motif lipase/hydrolase family protein
251257_at	At3g62290	0,98	0,97	1,00	1,01	1,20	0,88	0,92	0,89	ADP-ribosylation factor
251256_at	At3g62300	1,02	1,06	1,36	0,99	1,00	0,91	0,93	1,03	agenet domain-containing protein
251265_at	At3g62310	1,64	1,53	1,29	0,94	0,71	1,15	1,14	0,95	RNA helicase, putative
251266_at	At3g62320	0,92	1,00	0,82	0,88	1,20	0,98	0,96	0,93	hypothetical protein
251267_at	At3g62330	0,78	0,73	0,87	1,06	1,30	0,93	1,04	1,24	zinc knuckle (CCHC-type) family protein
251268_at	At3g62350	0,96	0,99	1,06	0,99	1,04	1,09	0,88	1,16	hypothetical protein
251269_at	At3g62360	1,02	0,92	0,80	0,97	1,08	0,94	0,88	0,94	expressed protein
251270_at	At3g62370	0,94	0,80	0,94	0,84	1,19	0,90	0,96	0,96	expressed protein
251236_at	At3g62380	0,98	1,09	0,90	0,93	1,13	0,95	0,97	1,15	hypothetical protein
251219_at	At3g62390	1,02	1,06	1,24	0,88	0,97	1,12	0,97	1,21	expressed protein
251220_at	At3g62400	1,29	1,13	1,16	1,21	1,11	0,98	1,01	0,96	expressed protein
251218_at	At3g62410	0,95	0,77	0,76	0,81	0,67	1,31	1,17	1,21	CP12 domain-containing protein
251237_at	At3g62420	0,87	0,82	0,79	0,80	0,77	1,03	1,19	1,35	bZIP transcription factor family protein
251238_at	At3g62430	0,89	0,93	0,95	0,98	1,21	0,95	1,06	1,00	F-box family protein
251239_at	At3g62440	1,01	1,07	0,94	1,05	0,86	1,05	1,00	1,09	F-box family protein
251240_at	At3g62450	1,04	0,93	0,86	1,04	1,45	0,91	0,81	0,67	expressed protein
251241_s_at	At3g62460	2,79	2,67	1,85	0,98	0,98	1,00	0,85	0,88	expressed protein
251211_s_at	At3g62470	0,83	0,81	0,95	0,87	1,03	0,99	1,02	1,13	pentatricopeptide (PPR) repeat-containing protein
251212_at	At3g62480	1,09	1,03	0,87	0,95	1,11	1,01	1,07	1,02	hypothetical protein
251213_at	At3g62490	0,93	0,96	0,93	0,99	1,03	1,10	1,04	1,00	expressed protein
251214_at	At3g62500	1,03	0,92	0,97	0,70	0,83	0,97	1,22	0,99	expressed protein
251215_at	At3g62510	1,01	1,03	0,97	0,86	0,95	1,22	1,32	1,12	protein disulfide isomerase-related
251216_at	At3g62520	0,98	1,00	0,88	0,98	1,13	0,98	1,05	0,88	---
251221_at	At3g62550	1,15	1,87	2,45	0,92	0,66	0,96	1,53	1,66	universal stress protein (USP) family protein
251217_at	At3g62560	1,30	1,20	1,17	1,23	1,30	1,07	0,96	1,02	GTP-binding protein, putative
251222_at	At3g62580	1,01	0,90	0,79	1,28	1,29	0,93	0,92	0,88	expressed protein
251191_at	At3g62590	0,93	0,94	0,92	1,33	1,23	1,14	1,09	1,08	lipase class 3 family protein
251182_at	At3g62600	1,17	0,97	0,98	1,38	1,28	1,03	0,98	1,17	DNAJ heat shock family protein
251223_at	At3g62610	0,91	0,92	1,07	0,96	1,04	1,00	1,14	1,15	myb family transcription factor
251224_at	At3g62620	1,33	1,31	1,44	0,89	1,47	1,23	0,92	1,05	sucrose-phosphatase-related
251183_at	At3g62630	0,91	0,96	0,97	0,82	1,11	0,99	0,84	1,16	expressed protein
251180_at	At3g62640	0,99	1,00	0,89	0,99	1,09	1,03	0,90	1,09	expressed protein
251189_at	At3g62650	0,89	0,97	1,00	0,92	0,95	0,86	0,87	1,04	expressed protein

251225_at	At3g62660	0,83	0,77	0,65	0,69	0,68	0,83	0,76	0,94	glycosyl transferase family 8 protein
251226_at	At3g62680	1,59	1,00	0,81	1,59	1,44	1,09	0,93	1,07	proline-rich family protein
251190_at	At3g62690	1,01	1,23	1,00	0,69	0,88	1,01	0,96	1,03	zinc finger (C3HC4-type RING finger) family protein (ATL5)
251227_at	At3g62700	0,85	0,82	0,98	0,83	0,68	1,13	1,11	1,09	glutathione-conjugate transporter, putative
251228_at	At3g62710	0,90	1,06	0,94	0,98	1,05	1,03	1,03	1,00	glycosyl hydrolase family 3 protein
251192_at	At3g62720	0,83	0,79	0,86	0,99	0,92	0,98	1,03	1,26	galactosyl transferase GMA12/MNN10 family protein
251188_at	At3g62730	1,12	0,91	0,92	1,21	1,12	1,03	1,03	0,97	expressed protein
251229_at	At3g62740	0,98	0,99	0,96	0,94	0,85	0,99	1,32	1,15	glycosyl hydrolase family 1 protein
251230_at	At3g62750	0,75	0,64	0,70	0,76	0,82	0,90	0,95	1,03	glycosyl hydrolase family 1 protein
251231_at	At3g62760	0,93	1,03	0,96	1,44	1,05	1,02	0,93	1,02	glutathione S-transferase, putative
251187_at	At3g62770	1,16	1,14	1,37	1,08	0,87	1,03	1,07	1,46	transport protein-related
251232_at	At3g62780	0,80	0,74	0,70	1,07	0,93	0,89	0,81	1,05	C2 domain-containing protein
251186_at	At3g62790	1,25	1,20	0,97	1,30	1,36	1,04	0,87	0,88	NADH-ubiquinone oxidoreductase-related
251233_at	At3g62800	1,11	1,26	1,16	1,10	0,95	1,03	1,01	1,02	double-stranded RNA-binding domain (DsRBD)-containing protein
251210_at	At3g62810	1,25	1,16	1,15	1,43	1,68	0,88	0,88	0,81	complex 1 family protein / LVR family protein
251181_at	At3g62820	0,78	0,76	0,65	0,90	0,63	0,72	0,72	0,68	invertase/pectin methylesterase inhibitor family protein
251184_at	At3g62840	1,26	0,95	0,89	1,01	1,08	0,93	0,98	0,94	small nuclear ribonucleoprotein D2, putative / snRNP core protein D2, putat
251235_at	At3g62860	1,47	2,04	2,53	1,64	2,08	0,89	1,17	1,48	esterase/lipase/thioesterase family protein
251185_at	At3g62870	1,58	1,21	1,14	1,13	1,03	1,04	0,98	1,01	60S ribosomal protein L7A (RPL7aB)
251208_at	At3g62880	1,67	1,74	2,17	1,06	1,05	1,01	1,09	1,20	mitochondrial import inner membrane translocase subunit Tim17/Tim22/Tim
251209_at	At3g62890	1,05	0,94	0,97	0,93	1,01	1,02	0,90	1,02	pentatricopeptide (PPR) repeat-containing protein
251193_at	At3g62910	1,32	1,15	1,12	0,68	0,89	1,12	1,19	1,24	peptide chain release factor, putative
251194_at	At3g62920	0,98	0,87	0,90	1,19	1,13	0,94	1,04	1,02	expressed protein
251195_at	At3g62930	2,03	1,35	1,11	1,22	1,52	0,89	1,06	0,92	glutaredoxin family protein
251149_at	At3g62940	1,68	1,50	1,60	1,27	1,12	1,21	1,07	1,30	OTU-like cysteine protease family protein
251196_at	At3g62950	1,24	1,07	1,03	1,50	0,96	1,06	1,10	0,96	glutaredoxin family protein
251197_at	At3g62960	1,04	1,16	1,34	1,45	1,70	0,72	0,76	0,74	glutaredoxin family protein
251198_at	At3g62970	1,00	1,03	1,28	0,78	0,75	0,96	1,06	1,15	zinc finger (C3HC4-type RING finger) family protein
251199_at	At3g62980	1,40	1,44	1,54	0,91	1,01	0,95	0,98	1,22	transport inhibitor response 1 (TIR1) (FBL1)
251153_at	At3g63000	1,02	1,23	1,11	0,86	1,07	0,89	0,89	0,99	NPL4 family protein
251200_at	At3g63010	0,91	1,11	1,16	1,05	1,23	0,93	0,93	1,12	expressed protein
251201_at	At3g63020	1,00	0,89	0,85	0,82	0,89	0,92	0,94	1,06	hypothetical protein
251202_at	At3g63040	0,99	0,91	0,77	0,95	1,03	0,99	1,05	0,97	expressed protein
251207_at	At3g63050	1,05	0,92	1,03	1,07	1,03	1,12	1,00	1,09	expressed protein
251156_at	At3g63060	0,89	0,98	1,07	0,79	0,99	1,05	1,01	0,88	circadian clock coupling factor, putative
251203_at	At3g63070	1,02	1,07	1,20	0,78	1,04	1,13	1,27	1,28	PWWP domain-containing protein
251205_at	At3g63080	1,09	0,89	0,94	1,21	1,16	1,01	0,90	1,03	glutathione peroxidase, putative
251206_at	At3g63090	0,85	0,78	0,92	1,27	1,03	0,93	0,74	0,70	expressed protein
251204_at	At3g63100	1,12	1,04	1,05	1,20	1,20	0,88	1,05	1,01	glycine-rich protein
251154_at	At3g63110	0,91	1,00	1,00	0,89	0,97	0,91	1,03	0,95	adenylate isopentenyltransferase 3 / cytokinin synthase (IPT3)
251150_at	At3g63120	1,27	1,22	1,47	1,15	1,31	1,00	0,95	1,01	cyclin family protein
251152_at	At3g63130	1,33	1,31	1,25	1,20	1,70	1,00	0,85	0,77	RAN GTPase activating protein 1 (RanGAP1)
251157_at	At3g63140	0,80	0,71	0,79	0,82	0,70	1,10	1,01	1,11	mRNA-binding protein, putative
251158_at	At3g63150	0,81	0,79	0,80	0,81	0,91	0,96	0,98	0,86	GTP-binding protein-related
251155_at	At3g63160	0,66	0,58	0,57	0,54	0,62	0,95	0,90	0,94	expressed protein
251151_at	At3g63170	0,74	0,73	0,72	0,96	0,96	0,82	0,78	0,80	expressed protein

251148_at	At3g63180	1,06	1,34	1,45	0,89	1,07	0,96	0,94	1,36	expressed protein
251172_at	At3g63190	0,81	0,74	0,71	0,86	0,96	0,95	0,99	0,98	ribosome recycling factor, chloroplast, putative / ribosome releasing factor,
251174_at	At3g63200	1,81	1,22	0,95	1,60	1,51	0,77	0,99	1,18	patatin-related
251169_at	At3g63210	1,20	1,09	0,93	0,79	0,84	1,19	1,17	0,93	expressed protein
251171_at	At3g63220	0,87	0,87	1,01	0,88	0,97	0,86	0,86	1,22	kelch repeat-containing F-box family protein
251159_at	At3g63230	0,91	1,08	1,01	1,02	1,20	0,89	0,97	1,00	senescence-associated protein-related
251160_at	At3g63240	0,97	0,78	0,96	0,71	0,97	1,01	0,84	0,94	endonuclease/exonuclease/phosphatase family protein
251175_at	At3g63250	0,93	1,02	1,07	0,98	1,01	0,89	0,93	0,97	homocysteine S-methyltransferase 2 (HMT-2)
251170_at	At3g63260	0,95	0,85	0,90	1,10	1,04	0,98	0,97	0,97	protein kinase, putative (MRK1)
251173_at	At3g63270	1,02	0,99	0,83	1,14	0,93	0,95	0,86	0,91	expressed protein
251161_at	At3g63290	0,86	0,90	0,92	0,90	1,11	1,01	0,96	1,16	expressed protein
251162_at	At3g63300	1,11	1,13	0,95	0,85	1,10	1,06	1,09	1,15	expressed protein
251163_at	At3g63310	0,83	0,93	0,85	1,23	1,01	0,95	0,81	1,00	expressed protein
251164_s_at	At3g63320	0,93	0,98	0,93	0,96	0,97	0,96	1,13	1,01	protein phosphatase 2C-related / PP2C-related
251165_at	At3g63330	1,01	1,05	0,92	0,84	0,78	0,96	1,04	1,03	protein kinase family protein
251166_at	At3g63350	1,04	1,00	0,76	0,99	0,95	0,96	0,83	1,17	heat shock transcription factor family protein
251167_at	At3g63360	1,08	1,10	0,96	1,00	1,01	1,04	0,90	0,93	defensin-related
251168_at	At3g63370	0,93	0,99	1,13	1,05	0,95	1,11	0,99	0,90	pentatricopeptide (PPR) repeat-containing protein
251176_at	At3g63380	0,94	1,27	1,10	0,88	0,97	2,46	1,12	1,31	calcium-transporting ATPase, plasma membrane-type, putative / Ca(2+)-AT
251117_at	At3g63390	1,04	1,11	1,19	1,01	1,24	0,92	0,81	0,82	expressed protein
251177_at	At3g63400	1,21	1,38	1,62	0,73	0,97	1,15	1,28	1,27	peptidyl-prolyl cis-trans isomerase cyclophilin-type family protein
251118_at	At3g63410	0,98	0,88	0,91	0,74	0,72	1,08	0,96	1,04	chloroplast inner envelope membrane protein, putative (APG1)
251121_at	At3g63420	0,66	0,64	0,87	0,92	1,02	0,80	0,79	0,89	heterotrimeric G protein gamma-subunit (AGG1)
251178_at	At3g63440	1,08	1,18	0,98	0,92	1,06	1,20	1,25	1,02	FAD-binding domain-containing protein / cytokinin oxidase family protein
251179_at	At3g63460	1,12	1,08	1,03	1,22	1,34	0,85	0,89	0,85	WD-40 repeat family protein
251116_at	At3g63470	1,14	0,99	0,95	1,07	1,16	1,06	1,25	1,23	serine carboxypeptidase, putative
251147_at	At3g63480	0,93	1,01	1,05	1,04	1,05	1,01	1,19	1,00	kinesin heavy chain, putative
251120_at	At3g63490	0,91	0,91	0,91	0,89	0,96	0,98	1,00	1,09	ribosomal protein L1 family protein
251145_at	At3g63500	0,76	0,88	1,10	0,91	0,92	0,90	1,02	1,19	expressed protein
251119_at	At3g63510	0,90	0,86	0,73	0,78	0,94	1,02	1,07	1,08	nitrogen regulation family protein
251146_at	At3g63520	0,98	0,88	0,86	0,79	0,76	1,23	1,25	1,23	9-cis-epoxycarotenoid dioxygenase / neoxanthin cleavage enzyme / NCED
256248_at	At3g66652	1,17	1,04	1,19	0,80	0,87	1,12	1,05	1,02	fip1 motif-containing protein
256247_at	At3g66656	1,08	0,96	1,11	0,95	1,04	1,04	0,95	0,95	MADS-box family protein
256246_at	At3g66658	1,04	0,97	1,07	1,16	1,26	0,98	0,86	0,91	betaine-aldehyde dehydrogenase, putative
255724_at	At4g00010	1,09	1,26	1,15	0,95	1,07	1,02	0,99	1,05	expressed protein
255693_s_at	At4g00020	1,21	1,14	1,12	1,12	1,01	1,03	1,06	1,18	BRCA2 repeat-containing protein
255710_at	At4g00030	1,58	1,19	1,59	1,00	1,02	1,16	1,05	1,07	plastid-lipid associated protein PAP / fibrillin family protein
255703_at	At4g00040	1,40	1,17	1,03	0,87	0,96	1,29	1,42	1,51	chalcone and stilbene synthase family protein
255694_at	At4g00050	2,44	1,92	1,39	1,17	1,05	1,53	1,30	1,18	basic helix-loop-helix (bHLH) family protein
257488_s_at	At4g00070	0,97	1,02	1,12	1,02	0,97	1,07	1,09	1,09	zinc finger protein-related
255695_at	At4g00080	0,96	1,17	0,96	2,22	2,20	1,05	1,02	1,42	invertase/pectin methylesterase inhibitor family protein
255711_at	At4g00090	1,46	1,33	1,55	1,07	0,96	1,15	1,18	1,39	transducin family protein / WD-40 repeat family protein
255706_at	At4g00100	1,40	1,28	1,09	1,06	1,12	1,03	0,92	0,91	40S ribosomal protein S13 (RPS13A)
255696_at	At4g00110	0,76	0,77	0,63	1,11	1,09	0,85	0,85	0,71	NAD-dependent epimerase/dehydratase family protein
255697_at	At4g00130	0,99	1,07	0,97	0,94	0,97	1,07	0,98	1,13	hypothetical protein
255698_at	At4g00150	0,92	0,83	1,09	0,60	0,76	0,96	0,97	1,19	scarecrow-like transcription factor 6 (SCL6)

255705_at	At4g00160	0,98	0,98	0,95	1,06	1,07	0,95	0,92	1,10 F-box family protein
255704_at	At4g00170	1,18	1,11	1,18	0,91	0,91	1,02	1,12	1,12 vesicle-associated membrane family protein / VAMP family protein
255709_at	At4g00180	0,87	0,80	0,80	0,90	0,97	0,86	0,96	1,01 axial regulator YABBY3 (YABBY3)
255699_at	At4g00190	1,04	1,09	0,96	0,88	1,09	1,06	0,98	0,82 pectinesterase family protein
255700_at	At4g00200	0,97	0,97	0,94	1,26	1,16	0,93	1,02	1,04 DNA-binding family protein
255701_at	At4g00220	1,02	1,16	1,00	0,96	1,12	1,00	1,10	0,92 LOB domain protein 30 / lateral organ boundaries domain protein 30 (LBD3
255702_at	At4g00230	0,91	0,88	0,82	1,24	1,25	0,75	0,64	0,53 subtilisin-like serine endopeptidase (XSP1)
255707_at	At4g00231	0,90	1,21	0,87	0,66	1,02	1,02	1,01	0,90 ataxin-related
255708_at	At4g00232	0,98	0,88	1,12	0,96	0,87	1,23	1,04	1,00 hypothetical protein
255713_s_at	At4g00234	0,89	0,86	0,89	1,04	0,68	1,03	0,92	1,07 expressed protein
255668_s_at	At4g00238	1,02	0,97	0,96	1,22	1,23	0,83	0,99	0,97 DNA-binding storekeeper protein-related
255667_at	At4g00240	0,91	0,91	0,69	1,09	1,12	0,99	0,92	0,93 phospholipase D beta 2 / PLD beta 2 (PLDBETA2) / PLDdelta1
265908_at	At4g00270	1,02	0,90	0,89	0,90	1,14	1,12	0,93	1,08 DNA-binding storekeeper protein-related
255712_at	At4g00280	0,95	0,96	1,00	1,10	1,02	1,14	0,96	1,14 hypothetical protein
255714_at	At4g00300	1,00	0,81	0,80	0,88	1,17	0,94	0,87	0,88 fringe-related protein
255672_at	At4g00310	1,07	0,92	1,04	0,87	0,95	0,98	0,85	0,89 expressed protein
255715_s_at	At4g00320	1,67	1,66	1,56	0,99	1,12	1,01	0,98	1,13 F-box family protein
255716_at	At4g00330	0,79	0,76	0,83	1,04	1,13	0,98	0,92	1,03 protein kinase family protein
255670_at	At4g00335	1,28	1,44	1,39	0,81	0,80	0,99	1,04	1,13 zinc finger (C3HC4-type RING finger) family protein
255673_at	At4g00340	0,95	0,93	0,86	0,84	1,03	0,80	0,92	0,96 S-locus glycoprotein family protein / curculin-like (mannose-binding) lectin fa
255717_at	At4g00350	0,94	1,11	1,11	0,90	1,20	1,02	0,96	0,92 MATE efflux family protein
255671_at	At4g00355	1,01	1,08	1,32	1,01	1,19	1,02	0,95	1,23 expressed protein
255690_at	At4g00360	0,82	0,78	0,91	1,01	0,82	0,81	0,69	0,92 cytochrome P450, putative
255691_at	At4g00370	1,12	1,25	1,26	0,91	1,02	1,22	1,24	1,08 sugar transporter family protein
255666_at	At4g00390	1,00	1,08	0,94	1,02	1,13	0,93	1,06	0,85 expressed protein
255692_at	At4g00400	0,77	0,85	0,88	0,80	0,74	0,91	0,88	0,86 phospholipid/glycerol acyltransferase family protein
255662_at	At4g00410	0,96	0,77	0,92	0,93	0,67	0,98	0,77	0,74 phospholipid/glycerol acyltransferase family protein
255669_at	At4g00416	1,11	0,99	1,00	0,89	0,98	1,01	0,92	1,12 methyl-CpG-binding domain-containing protein
255663_at	At4g00420	1,19	1,26	1,37	1,13	1,18	0,98	0,96	0,90 double-stranded RNA-binding domain (DsRBD)-containing protein
255674_at	At4g00430	1,01	1,13	1,32	1,38	1,37	0,92	0,92	0,96 plasma membrane intrinsic protein, putative
255664_at	At4g00440	0,86	0,95	0,88	1,33	1,22	0,86	0,91	1,00 expressed protein
255665_at	At4g00450	0,87	0,81	1,12	0,72	0,67	0,99	0,92	1,24 expressed protein
255757_at	At4g00460	1,07	1,02	0,86	1,12	1,00	0,91	1,00	0,95 expressed protein
255675_at	At4g00480	0,83	1,04	1,07	1,23	1,10	0,79	0,90	0,93 myc-related transcription factor (MYC1)
255676_at	At4g00490	1,27	1,17	1,13	0,84	0,70	1,41	1,28	1,25 beta-amylase, putative / 1,4-alpha-D-glucan maltohydrolase, putative
255677_at	At4g00500	1,11	1,08	1,10	0,91	1,04	1,12	0,87	1,00 lipase class 3 family protein / calmodulin-binding heat-shock protein-related
255678_at	At4g00510	1,09	0,95	1,16	0,84	0,88	1,07	0,95	0,64 cyclic nucleotide-binding domain-containing protein
255679_at	At4g00520	1,03	0,99	0,98	0,90	1,08	0,92	0,85	0,77 acyl-CoA thioesterase family protein
255680_at	At4g00530	1,06	1,01	1,22	0,98	1,14	1,07	1,05	0,72 hypothetical protein
255681_at	At4g00550	1,01	0,92	1,13	0,90	1,07	1,05	0,92	1,09 UDP-galactose:MGDG galactosyltransferase 2 / digalactosyldiacylglycerol s
255682_at	At4g00560	0,72	0,74	0,73	0,91	0,80	0,76	0,73	0,94 methionine adenosyltransferase regulatory beta subunit-related
255683_at	At4g00570	0,78	0,62	0,67	1,23	0,97	0,75	0,75	0,87 malate oxidoreductase, putative
255633_at	At4g00585	1,14	0,95	0,91	1,20	1,32	0,93	0,86	0,90 expressed protein
255684_at	At4g00590	0,90	0,93	0,93	0,94	1,07	0,95	0,99	0,93 asparaginase 2 family protein
255685_s_at	At4g00600	2,05	1,58	1,48	0,99	0,96	0,93	0,92	0,83 tetrahydrofolate dehydrogenase/cyclohydrolase, putative
255686_at	At4g00630	0,74	0,81	0,75	0,72	0,74	1,03	0,94	1,00 K+ efflux antiporter, putative (KEA2)

255687_at	At4g00640	0,74	0,71	0,73	0,91	0,75	0,98	0,97	0,86	hypothetical protein
255634_at	At4g00650	1,03	0,97	0,92	0,95	1,06	1,00	1,08	0,82	FRIGIDA protein
255688_at	At4g00660	0,97	1,01	1,13	1,06	1,11	0,88	0,97	1,04	DEAD/DEAH box helicase, putative
255689_at	At4g00670	0,85	0,75	0,52	0,94	1,08	1,23	1,23	1,14	remorin family protein
255632_at	At4g00680	0,97	0,89	0,78	1,47	1,58	1,28	1,11	1,08	actin-depolymerizing factor, putative
255661_at	At4g00690	1,00	1,09	1,13	0,94	1,25	0,94	1,03	0,97	Ulp1 protease family protein
255630_at	At4g00700	0,93	1,09	0,88	1,03	1,07	1,10	1,00	1,04	C2 domain-containing protein
255631_at	At4g00710	0,91	1,03	1,16	1,22	1,52	0,94	0,97	1,06	protein kinase family protein
255635_at	At4g00720	1,02	0,94	1,02	0,80	0,86	0,92	0,84	0,93	shaggy-related protein kinase theta / ASK-theta (ASK8)
255636_at	At4g00730	0,71	0,70	0,68	0,71	0,78	0,91	0,84	0,87	anthocyaninless2 (ANL2)
255638_at	At4g00740	0,94	1,01	1,18	1,08	1,16	0,92	0,84	0,87	dehydration-responsive protein-related
255637_at	At4g00750	0,93	0,89	1,04	0,79	0,94	1,09	0,97	1,13	dehydration-responsive family protein
255660_at	At4g00755	1,15	1,44	1,60	1,06	1,29	1,09	1,07	0,96	F-box family protein
255639_at	At4g00760	0,86	0,92	0,81	0,92	0,78	1,01	1,13	1,23	two-component responsive regulator family protein / response regulator fam
255658_at	At4g00770	0,96	0,99	1,05	0,88	0,96	1,08	0,93	0,92	expressed protein
255626_at	At4g00780	1,20	1,15	1,50	1,24	1,39	0,84	0,99	0,95	meprin and TRAF homology domain-containing protein / MATH domain-cor
255640_at	At4g00790	1,04	1,17	1,31	0,90	0,76	1,15	1,19	1,30	expressed protein
255641_at	At4g00800	1,09	1,08	1,25	0,99	0,85	1,11	1,05	1,07	expressed protein
255657_at	At4g00810	1,51	1,30	1,14	1,10	1,05	1,03	1,00	0,97	60S acidic ribosomal protein P1 (RPP1B)
255642_at	At4g00820	1,08	1,11	1,18	1,08	1,46	0,89	0,97	1,09	calmodulin-binding protein-related
255598_at	At4g00830	1,07	1,00	0,94	1,10	0,98	1,00	0,98	0,93	RNA recognition motif (RRM)-containing protein
255643_at	At4g00840	0,94	0,95	0,92	1,09	0,70	1,04	0,95	0,80	zinc finger (DHHC type) family protein
255628_at	At4g00850	1,04	0,99	1,33	0,84	1,13	0,98	0,93	0,97	SSXT family protein
255629_at	At4g00860	1,35	1,12	0,98	1,22	1,31	1,02	1,01	0,93	stress-related ozone-induced protein (OZI1) / stress-related ozone-respons
255644_at	At4g00870	1,01	1,09	0,96	1,11	1,03	1,11	0,91	1,05	basic helix-loop-helix (bHLH) family protein
255645_at	At4g00880	1,01	0,79	0,62	0,86	0,79	0,86	0,84	0,91	auxin-responsive family protein
255646_at	At4g00890	0,99	1,05	1,00	1,03	1,29	0,91	0,92	1,11	proline-rich family protein
255659_at	At4g00895	0,89	0,82	0,77	0,87	0,90	1,03	0,87	0,74	ATP synthase delta chain-related
255647_at	At4g00900	1,14	1,08	1,03	1,28	1,25	1,00	0,99	1,01	calcium-transporting ATPase 2, endoplasmic reticulum-type (ECA2)
255648_at	At4g00910	1,02	0,94	0,96	1,00	1,10	1,03	0,91	0,97	expressed protein
255649_at	At4g00920	1,00	0,95	0,97	1,00	1,00	0,98	1,06	0,81	COP1-interacting protein-related
255650_s_at	At4g00930	1,18	0,83	0,95	0,73	0,73	1,04	1,27	1,02	COP1-interacting protein 4.1 (CIP4.1)
255651_at	At4g00940	0,98	0,96	1,03	0,95	0,97	0,91	0,99	1,10	Dof-type zinc finger domain-containing protein
255652_at	At4g00950	1,25	1,08	1,09	0,75	0,80	0,96	0,86	0,73	expressed protein
255627_at	At4g00955	1,11	0,85	0,73	1,02	1,20	1,10	1,06	0,91	expressed protein
255653_at	At4g00960	1,04	0,88	0,98	0,97	1,02	0,97	0,87	1,09	protein kinase family protein
255654_at	At4g00970	0,56	0,38	0,68	0,86	0,70	0,87	1,29	0,99	protein kinase family protein
255655_at	At4g00980	1,19	1,23	0,94	0,86	1,01	0,93	0,92	1,05	zinc knuckle (CCHC-type) family protein
255656_at	At4g00990	1,07	1,27	1,67	0,82	0,92	1,05	1,21	1,30	transcription factor jumonji (jmjC) domain-containing protein
255599_at	At4g01010	0,78	0,77	1,16	1,15	1,17	0,99	1,25	1,28	cyclic nucleotide-regulated ion channel, putative (CNGC13)
255602_at	At4g01026	1,24	1,52	1,72	1,33	1,19	1,12	1,07	1,03	expressed protein
255601_at	At4g01030	0,90	0,92	1,03	0,84	1,02	1,00	0,94	0,89	pentatricopeptide (PPR) repeat-containing protein
255603_at	At4g01040	0,99	0,95	0,89	0,95	1,01	0,96	0,90	0,93	glycosyl hydrolase family 18 protein
255572_at	At4g01050	1,09	1,00	1,03	0,81	0,78	1,08	0,98	1,09	hydroxyproline-rich glycoprotein family protein
255622_at	At4g01070	0,83	0,97	1,08	1,00	0,94	0,85	1,01	0,91	UDP-glucoronosyl/UDP-glucosyl transferase family protein
255604_at	At4g01080	1,00	1,03	0,91	0,91	0,78	0,92	0,99	1,12	expressed protein

255605_at	At4g01090	0,72	0,86	1,11	0,80	0,82	1,19	1,56	1,93	extra-large G-protein-related
255570_at	At4g01100	1,12	1,16	1,10	1,01	0,95	1,01	0,95	0,93	mitochondrial substrate carrier family protein
255606_at	At4g01110	0,95	0,99	1,05	1,01	0,99	1,13	1,00	0,98	hypothetical protein
255625_at	At4g01120	1,09	1,01	1,48	1,03	1,41	0,86	0,95	1,43	G-box binding factor 2 (GBF2)
255607_at	At4g01130	0,43	0,47	0,51	0,62	0,69	0,81	0,73	0,60	acetylesterase, putative
255608_at	At4g01140	1,04	0,93	0,84	1,28	0,85	0,90	0,96	0,98	expressed protein
255567_at	At4g01150	0,95	0,92	0,88	0,94	1,00	0,96	0,92	0,93	expressed protein
255609_s_at	At4g01180	0,99	1,02	1,06	0,98	1,02	1,01	0,99	1,03	XH/XS domain-containing protein
255610_at	At4g01190	0,90	0,96	0,89	1,01	1,30	1,20	0,83	0,94	phosphatidylinositol-4-phosphate 5-kinase family protein
255611_at	At4g01210	0,93	0,78	0,99	0,84	1,01	0,97	1,06	1,04	glycosyltransferase family protein 1
255571_at	At4g01220	0,85	0,77	0,90	0,85	1,29	0,87	0,72	0,86	expressed protein
255612_at	At4g01240	1,04	1,00	0,88	1,04	1,17	1,10	0,95	0,87	hypothetical protein
255568_at	At4g01250	0,89	1,00	0,77	0,83	0,87	0,97	1,22	1,57	WRKY family transcription factor
255613_at	At4g01270	0,98	1,04	0,95	0,99	1,08	1,04	0,97	0,89	zinc finger (C3HC4-type RING finger) family protein
255614_at	At4g01280	1,12	1,17	1,28	0,86	1,25	0,89	0,88	1,08	myb family transcription factor
255615_at	At4g01290	0,90	0,84	0,86	0,91	1,00	0,97	0,99	0,94	expressed protein
255616_at	At4g01300	0,89	0,81	0,81	0,99	1,00	1,03	0,84	0,77	expressed protein
255623_at	At4g01310	1,08	0,96	0,98	0,92	0,95	1,07	0,99	1,01	ribosomal protein L5 family protein
255569_at	At4g01320	1,01	0,81	0,79	1,13	1,11	0,96	0,86	0,91	CAAX protease, putative (STE24)
255617_at	At4g01330	1,15	1,60	1,81	0,85	0,95	1,00	1,25	1,22	protein kinase family protein
255618_at	At4g01340	1,10	1,04	0,92	0,92	0,91	0,99	1,06	1,06	CHP-rich zinc finger protein-related
255619_s_at	At4g01350	0,88	0,82	1,01	0,85	0,75	1,08	1,37	0,97	DC1 domain-containing protein
255624_at	At4g01370	1,13	1,01	0,85	1,13	1,07	1,10	0,98	0,84	mitogen-activated protein kinase, putative / MAPK, putative (MPK4)
255620_at	At4g01380	1,01	1,02	1,03	1,15	1,04	1,08	1,02	0,93	plastocyanin-like domain-containing protein
255621_at	At4g01390	1,07	1,24	1,08	0,95	0,93	0,85	0,89	0,89	meprin and TRAF homology domain-containing protein / MATH domain-cor
255573_at	At4g01400	0,92	0,93	0,97	0,77	1,00	0,87	0,73	0,73	pentatricopeptide (PPR) repeat-containing protein
255577_at	At4g01410	0,74	1,06	0,85	1,14	1,33	0,73	0,80	0,88	harpin-induced family protein / HIN1 family protein / harpin-responsive famil
255574_at	At4g01420	0,98	1,03	0,96	1,00	1,10	1,05	0,93	0,97	calcineurin B-like protein 5 (CBL5)
255575_at	At4g01430	0,67	0,60	0,53	1,16	1,10	0,93	1,15	0,97	nodulin MtN21 family protein
255576_at	At4g01440	1,00	1,19	1,01	0,93	0,84	0,92	0,91	1,06	nodulin MtN21 family protein
255578_at	At4g01450	1,24	1,07	1,06	1,08	0,87	0,90	1,02	1,13	nodulin MtN21 family protein
255579_at	At4g01460	0,97	0,93	0,93	0,90	1,05	1,12	1,08	0,89	basic helix-loop-helix (bHLH) family protein
255580_at	At4g01470	0,97	1,18	0,91	0,99	1,05	0,89	0,85	0,86	major intrinsic family protein / MIP family protein
255587_at	At4g01480	1,12	1,12	0,79	2,04	1,48	0,93	0,96	0,94	inorganic pyrophosphatase, putative (soluble) / pyrophosphate phospho-hy
255581_at	At4g01490	0,98	1,05	0,94	1,06	0,91	0,98	1,09	0,84	---
255582_at	At4g01500	1,04	1,02	1,06	1,05	1,31	0,99	0,96	1,18	DNA-binding protein, putative
255583_at	At4g01510	0,63	0,54	0,60	0,94	0,67	0,91	1,00	0,77	transmembrane ARV1-like family protein
255584_at	At4g01530	0,98	1,01	0,97	1,07	1,07	0,95	1,00	0,95	hypothetical protein
255585_at	At4g01550	0,77	0,79	1,04	0,76	0,95	0,83	0,99	1,12	no apical meristem (NAM) family protein
255586_at	At4g01560	1,21	1,07	1,01	0,87	1,06	0,99	0,90	1,06	brix domain-containing protein
255588_at	At4g01570	0,92	0,70	0,86	0,79	0,80	0,96	0,83	1,00	pentatricopeptide (PPR) repeat-containing protein
255536_at	At4g01575	0,92	0,93	0,77	1,15	1,21	1,13	1,16	1,08	serine protease inhibitor, Kazal-type family protein
255589_s_at	At4g01590	1,21	1,03	1,08	1,03	1,19	1,04	0,98	0,79	expressed protein
255590_at	At4g01610	0,54	0,52	0,49	0,99	0,88	0,96	0,97	1,02	cathepsin B-like cysteine protease, putative
255591_at	At4g01630	1,05	1,31	1,15	1,20	1,19	1,20	1,13	1,06	expansin, putative (EXP17)
255592_s_at	At4g01640	0,98	1,02	0,95	0,99	1,05	1,13	1,02	1,17	S locus F-box-related / SLF-related

255593_at	At4g01650	0,86	0,75	0,84	0,93	0,81	0,91	0,92	0,76	expressed protein
255594_at	At4g01660	1,05	0,88	1,05	1,25	1,07	1,18	0,98	1,08	ABC1 family protein
255538_at	At4g01680	1,42	1,35	1,08	1,07	1,28	0,63	0,92	1,04	myb family transcription factor (MYB55)
255537_at	At4g01690	1,04	0,93	1,02	0,83	0,88	1,17	0,96	0,94	protoporphyrinogen oxidase (PPOX)
255595_at	At4g01700	0,80	0,79	0,91	2,94	2,69	1,61	0,98	1,03	chitinase, putative
255539_at	At4g01710	0,97	0,93	1,00	1,27	1,41	0,89	0,80	0,84	actin polymerization factor protein-related
255596_at	At4g01720	1,49	1,61	1,14	1,15	1,12	1,11	1,20	1,06	WRKY family transcription factor
255597_at	At4g01730	1,05	1,06	1,06	1,30	1,54	0,91	0,85	0,74	zinc finger (DHHC type) family protein
255563_at	At4g01740	1,00	0,92	1,10	1,00	0,99	1,02	1,03	1,04	DC1 domain-containing protein
255564_s_at	At4g01750	0,77	0,65	0,74	1,00	0,80	0,91	0,84	1,08	expressed protein
255565_at	At4g01760	0,95	1,02	0,92	1,09	1,13	0,95	1,04	1,09	DC1 domain-containing protein
255566_s_at	At4g01780	1,15	0,89	0,76	1,02	1,09	1,10	1,09	1,17	XH/XS domain-containing protein / XS zinc finger domain-containing protei
255535_at	At4g01790	1,11	1,17	1,10	1,11	1,34	1,07	0,93	1,02	ribosomal protein L7Ae/L30e/S12e/Gadd45 family protein / ribonuclease P-
255540_at	At4g01800	0,91	0,76	0,84	0,68	0,67	1,20	1,22	1,37	preprotein translocase secA subunit, putative
255555_at	At4g01810	0,93	1,02	1,02	1,03	0,99	0,95	0,82	0,83	protein transport protein-related
255541_s_at	At4g01820	0,96	1,00	0,99	1,05	1,08	0,91	0,94	0,84	multidrug resistance P-glycoprotein, putative
255551_at	At4g01840	0,98	1,00	1,01	1,13	1,02	1,01	1,22	0,97	outward rectifying potassium channel, putative (KCO5)
255552_at	At4g01850	0,96	0,92	0,95	0,91	1,00	0,89	0,95	0,96	S-adenosylmethionine synthetase 2 (SAM2)
255542_at	At4g01860	0,88	0,86	0,82	0,83	0,88	0,83	0,83	1,13	transducin family protein / WD-40 repeat family protein
255543_at	At4g01870	0,52	0,53	0,70	0,53	0,53	0,81	0,72	1,00	tolB protein-related
255544_at	At4g01880	0,79	0,97	0,87	0,84	0,85	0,82	0,89	0,87	expressed protein
255545_at	At4g01890	0,96	0,97	1,07	1,04	1,16	1,11	0,93	0,77	glycoside hydrolase family 28 protein / polygalacturonase (pectinase) family
255554_at	At4g01895	1,00	0,94	0,95	1,30	1,48	0,85	0,85	0,75	expressed protein
255558_at	At4g01900	0,78	0,68	0,62	0,92	0,97	0,73	0,79	0,77	P II nitrogen sensing protein (GLB I)
255546_at	At4g01910	0,95	0,98	0,96	0,92	0,98	0,89	1,01	0,88	DC1 domain-containing protein
255547_at	At4g01920	1,03	0,99	1,01	1,18	1,09	1,23	1,00	0,93	DC1 domain-containing protein
255548_at	At4g01930	0,91	1,10	1,06	1,13	1,18	1,11	0,92	0,98	DC1 domain-containing protein
255556_at	At4g01940	0,87	0,85	0,92	0,89	0,99	1,06	0,93	0,88	nitrogen fixation NifU-like family protein
255549_at	At4g01950	0,73	0,91	1,48	0,88	0,49	1,18	2,06	2,83	phospholipid/glycerol acyltransferase family protein
255553_at	At4g01960	1,40	1,37	1,83	1,45	1,37	1,21	0,94	1,11	expressed protein
255550_at	At4g01970	0,92	0,91	0,70	0,93	0,97	0,90	0,91	0,95	galactinol-raffinose galactosyltransferase, putative
255557_at	At4g01990	1,14	1,12	0,94	1,07	0,95	1,03	0,77	0,89	pentatricopeptide (PPR) repeat-containing protein
255559_at	At4g02010	1,00	0,96	0,88	0,71	0,98	0,90	0,78	0,97	protein kinase family protein
255510_at	At4g02020	1,04	1,03	1,29	0,88	0,70	1,09	1,22	1,00	zeste-like protein 1 (EZA1)
255560_at	At4g02030	1,13	0,93	1,02	1,02	1,11	1,07	1,01	0,97	expressed protein
255561_at	At4g02050	1,10	1,14	1,01	1,22	0,97	1,16	1,05	1,12	sugar transporter, putative
255513_at	At4g02060	1,60	1,31	1,52	1,82	1,53	1,10	0,98	0,93	prolifera protein (PRL) / DNA replication licensing factor Mcm7 (MCM7)
255562_at	At4g02070	0,89	1,02	1,09	0,94	1,00	0,95	1,05	1,10	DNA mismatch repair protein MSH6-1 (MSH6-1) (AGAA.3)
255511_at	At4g02075	1,97	1,41	1,08	0,89	1,10	1,40	1,48	0,96	zinc finger (C3HC4-type RING finger) family protein
255505_at	At4g02080	0,94	0,90	0,99	1,20	1,34	0,93	0,87	0,91	GTP-binding protein (SAR1A)
255528_at	At4g02090	1,10	0,67	1,21	1,11	0,99	0,98	0,97	1,00	expressed protein
255529_at	At4g02120	1,24	1,28	1,59	0,86	0,91	1,15	1,46	1,71	CTP synthase, putative / UTP--ammonia ligase, putative
255506_at	At4g02130	0,79	0,69	0,89	0,83	0,94	0,92	0,97	1,17	glycosyl transferase family 8 protein
255530_at	At4g02140	0,97	0,87	1,19	0,81	0,88	0,99	0,69	0,94	expressed protein
255507_at	At4g02150	1,17	1,00	0,98	1,18	1,06	0,96	0,97	0,92	importin alpha-2 subunit
255531_at	At4g02160	0,94	1,25	1,15	0,84	1,16	1,14	0,88	1,00	hypothetical protein

255532_at	At4g02170	1,25	1,05	0,90	0,80	0,85	1,49	1,12	1,10	expressed protein
255533_at	At4g02180	1,03	0,94	1,05	1,18	1,02	1,06	1,13	1,06	DC1 domain-containing protein
255534_at	At4g02190	0,91	0,93	1,09	0,93	1,06	1,09	0,98	1,05	DC1 domain-containing protein
255512_at	At4g02195	0,84	0,79	0,98	0,87	1,02	1,09	1,22	1,28	syntaxin 42 (SYP42) / TLG2b
255504_at	At4g02200	0,94	1,49	1,76	0,71	0,92	0,95	1,06	0,82	drought-responsive family protein
255509_at	At4g02210	1,35	1,86	1,33	0,84	1,11	1,26	1,19	1,06	expressed protein
255508_at	At4g02220	0,97	0,81	0,87	0,83	0,82	0,92	0,87	0,87	zinc finger (MYND type) family protein / programmed cell death 2 C-termina
255520_at	At4g02230	1,55	1,68	1,68	1,21	1,31	1,10	1,01	1,13	60S ribosomal protein L19 (RPL19C)
255514_s_at	At4g02240	1,00	0,96	1,00	1,06	1,05	0,96	1,04	1,02	MADS-box family protein
255515_at	At4g02250	0,93	0,98	1,05	0,98	1,08	0,93	1,03	1,04	invertase/pectin methylesterase inhibitor family protein
255522_at	At4g02260	0,85	0,98	0,88	0,58	0,60	1,20	1,21	1,16	RelA/SpoT protein, putative (RSH1)
255516_at	At4g02270	1,52	1,23	1,20	2,00	2,27	0,95	1,12	0,94	pollen Ole e 1 allergen and extensin family protein
255521_at	At4g02280	0,40	0,41	0,28	0,99	0,84	0,73	0,72	0,78	sucrose synthase, putative / sucrose-UDP glucosyltransferase, putative
255517_at	At4g02290	1,07	1,05	0,87	1,01	1,04	0,92	0,94	0,70	glycosyl hydrolase family 9 protein
255518_at	At4g02300	1,00	0,95	0,99	0,91	1,06	0,92	0,93	1,17	pectinesterase family protein
255519_at	At4g02310	1,03	0,95	1,18	0,90	1,09	0,99	1,13	1,37	kelch repeat-containing F-box family protein
255523_at	At4g02320	0,96	1,12	0,95	0,86	1,14	0,89	0,94	1,06	pectinesterase family protein
255524_at	At4g02330	1,28	0,99	1,19	1,63	1,37	1,03	0,85	0,71	pectinesterase family protein
255525_at	At4g02340	0,81	0,68	0,80	0,81	0,86	0,88	0,78	1,02	epoxide hydrolase, putative
255526_at	At4g02350	0,78	0,77	0,90	1,01	1,19	0,94	0,91	0,81	exocyst complex subunit Sec15-like family protein
255527_at	At4g02360	0,97	0,63	0,74	0,78	1,00	0,86	0,79	0,86	expressed protein
255477_at	At4g02370	0,78	0,79	0,72	0,96	0,83	0,84	0,72	0,74	expressed protein
255479_at	At4g02380	0,80	0,89	1,03	1,09	1,07	0,95	1,09	1,80	late embryogenesis abundant 3 family protein / LEA3 family protein
255500_at	At4g02390	1,81	1,53	1,94	0,75	0,72	1,06	1,31	1,40	poly (ADP-ribose) polymerase / NAD(+) ADP-ribosyltransferase / poly(ADP-
255501_at	At4g02400	1,13	1,08	0,93	0,93	0,84	1,13	0,94	1,04	U3 ribonucleoprotein (Utp) family protein
255502_at	At4g02410	0,53	0,54	0,56	0,52	0,38	1,07	1,49	1,10	lectin protein kinase family protein
255503_at	At4g02420	0,72	0,81	0,99	0,95	1,10	0,97	1,01	0,97	lectin protein kinase, putative
255472_at	At4g02430	0,97	0,88	0,86	0,87	1,15	1,08	1,11	1,08	pre-mRNA splicing factor, putative / SR1 protein, putative
255478_at	At4g02440	0,85	0,79	0,81	0,91	1,06	0,88	0,92	0,87	F-box family protein
255473_at	At4g02450	1,23	1,04	1,08	1,13	1,28	0,93	1,04	1,01	glycine-rich protein
255481_at	At4g02460	1,15	1,20	1,03	0,84	0,65	1,11	1,04	1,18	DNA mismatch repair protein, putative
255474_at	At4g02470	0,92	0,98	1,17	0,81	0,81	0,97	1,07	1,05	AAA-type ATPase family protein
255475_at	At4g02480	0,96	0,97	1,08	1,00	0,84	0,96	1,14	0,97	AAA-type ATPase family protein
255480_at	At4g02485	1,19	1,28	1,29	1,01	0,85	1,03	1,04	1,05	oxidoreductase, 2OG-Fe(II) oxygenase family protein
255476_at	At4g02490	1,04	1,01	1,02	0,97	1,12	0,80	1,00	0,94	---
255483_at	At4g02500	0,82	0,76	0,71	1,17	1,01	0,85	0,76	0,74	galactosyl transferase GMA12/MNN10 family protein
255482_at	At4g02510	0,85	0,84	0,91	0,72	0,63	0,94	0,90	0,94	chloroplast outer membrane protein, putative
255440_at	At4g02530	0,98	0,80	0,80	0,90	0,93	1,07	1,07	1,15	chloroplast thylakoid lumen protein
255484_at	At4g02540	0,98	0,85	0,94	0,89	0,80	1,11	1,59	2,08	DC1 domain-containing protein
255485_at	At4g02550	0,83	0,95	0,90	0,86	1,06	0,95	0,79	0,89	expressed protein
255444_at	At4g02560	1,09	1,15	1,08	1,15	1,09	1,24	1,25	1,33	homeobox protein LUMINIDEPENDENS (LD)
255441_at	At4g02570	1,10	0,98	1,04	0,95	1,02	1,04	1,03	1,09	cullin family protein
255442_at	At4g02580	1,20	1,05	1,03	1,17	1,23	1,08	0,95	0,96	NADH-ubiquinone oxidoreductase 24 kDa subunit, putative
255497_at	At4g02590	1,22	0,97	1,08	1,17	0,86	1,01	1,13	1,53	basic helix-loop-helix (bHLH) family protein
255486_at	At4g02600	0,62	0,59	0,59	0,81	0,92	0,89	0,94	0,90	seven transmembrane MLO family protein / MLO-like protein 1 (MLO1)
255487_at	At4g02610	1,12	1,13	1,23	1,31	1,35	0,94	1,18	0,87	tryptophan synthase, alpha subunit, putative

255498_at	At4g02620	1,22	1,06	0,95	1,17	1,36	0,94	0,92	0,93	vacuolar ATPase subunit F family protein
255488_at	At4g02630	0,80	0,70	0,61	1,12	1,22	1,12	1,09	0,76	protein kinase family protein
255496_at	At4g02640	0,96	0,72	0,85	1,10	1,28	0,94	0,96	0,97	bZIP transcription factor family protein
255489_at	At4g02650	1,01	0,97	1,06	1,08	1,12	1,02	0,85	1,23	epsin N-terminal homology (ENTH) domain-containing protein / clathrin ass
255490_at	At4g02660	1,00	1,08	1,01	0,99	0,96	1,25	1,31	1,10	WD-40 repeat family protein / beige-related
255491_at	At4g02670	0,95	1,00	1,20	1,07	1,05	0,97	1,17	0,99	zinc finger (C2H2 type) family protein
255492_at	At4g02680	1,19	0,96	0,90	0,95	0,88	1,02	0,95	0,94	tetratricopeptide repeat (TPR)-containing protein
255493_at	At4g02690	0,89	0,90	1,15	1,02	0,80	1,05	0,93	1,08	hypothetical protein
255443_at	At4g02700	0,90	1,02	0,85	0,87	0,92	0,95	0,85	0,89	sulfate transporter
255494_at	At4g02710	1,13	1,14	0,99	0,97	1,03	1,09	1,11	1,05	kinase interacting family protein
255495_at	At4g02720	1,26	1,20	1,63	1,17	1,10	1,05	1,08	1,41	expressed protein
255499_at	At4g02730	1,24	1,06	0,89	1,16	1,56	0,95	0,84	1,06	transducin family protein / WD-40 repeat family protein
255445_at	At4g02740	1,00	0,88	0,97	1,19	0,96	1,01	1,15	1,02	F-box family protein
255446_at	At4g02750	1,17	0,88	1,37	0,80	0,94	0,86	0,96	1,06	pentatricopeptide (PPR) repeat-containing protein
255457_at	At4g02770	0,95	0,92	1,00	0,90	0,90	0,96	0,89	0,86	photosystem I reaction center subunit II, chloroplast, putative / photosystem
255461_at	At4g02780	0,96	1,00	1,00	0,98	1,03	0,97	1,01	1,08	copalyl diphosphate synthase / CPS / ent-kaurene synthetase A (GA1)
255447_at	At4g02790	1,13	1,20	1,22	0,64	0,61	1,13	1,16	1,12	GTP-binding family protein
255460_at	At4g02800	1,49	1,32	1,19	1,34	1,29	1,09	0,93	0,91	expressed protein
255448_at	At4g02810	0,97	1,02	1,01	1,00	1,13	1,00	1,05	0,88	expressed protein
255449_at	At4g02820	1,21	1,13	1,35	1,07	0,93	1,14	0,94	1,12	pentatricopeptide (PPR) repeat-containing protein
255458_at	At4g02840	1,41	1,25	1,13	1,37	1,31	1,11	0,97	0,92	small nuclear ribonucleoprotein D1, putative / snRNP core protein D1, putat
255450_at	At4g02850	1,03	0,96	0,77	0,92	1,16	0,92	0,89	0,84	phenazine biosynthesis PhzC/PhzF family protein
255451_at	At4g02860	0,79	0,64	0,67	1,20	1,25	0,89	0,83	0,89	phenazine biosynthesis PhzC/PhzF family protein
255452_at	At4g02880	0,85	0,94	1,11	0,79	0,78	1,01	1,21	1,34	expressed protein
255459_at	At4g02890	0,96	0,98	1,34	0,92	0,98	1,03	1,03	1,24	polyubiquitin (UBQ14)
255453_at	At4g02900	1,09	1,12	0,93	1,04	0,93	1,09	1,03	1,00	early-responsive to dehydration protein-related / ERD protein-related
255454_at	At4g02910	1,10	0,91	1,06	1,00	1,08	0,94	0,97	0,96	hypothetical protein
255456_at	At4g02920	1,00	1,10	1,36	0,89	0,78	1,10	1,17	1,77	expressed protein
255455_at	At4g02930	1,39	1,14	0,94	1,20	1,10	0,91	1,02	0,96	elongation factor Tu, putative / EF-Tu, putative
255462_at	At4g02940	0,84	0,93	0,87	1,04	1,03	0,91	0,93	1,19	oxidoreductase, 2OG-Fe(II) oxygenase family protein
255463_at	At4g02960	0,98	1,06	0,91	0,87	1,12	1,05	0,97	0,98	---
255464_s_at	At4g02970	1,04	1,10	0,88	0,92	1,10	0,98	0,98	1,00	ubiquitin family protein
255412_at	At4g02980	0,91	0,92	0,84	0,97	1,17	0,95	0,90	0,74	auxin-binding protein 1 (ABP1)
255465_at	At4g02990	1,04	0,90	1,27	0,58	0,58	0,97	0,92	0,87	mitochondrial transcription termination factor family protein / mTERF family
255466_at	At4g03000	1,01	1,01	1,16	0,90	0,95	1,20	0,94	1,01	expressed protein
255467_at	At4g03010	0,81	0,75	0,95	1,01	0,87	0,83	0,78	0,72	leucine-rich repeat family protein
255468_at	At4g03020	0,82	0,71	0,70	0,90	1,06	1,05	0,89	0,82	transducin family protein / WD-40 repeat family protein
255469_at	At4g03030	0,80	0,82	0,85	0,71	0,68	0,93	0,90	0,76	kelch repeat-containing F-box family protein
255470_at	At4g03040	1,00	1,08	1,11	0,95	0,98	1,21	0,95	0,94	hypothetical protein
255471_at	At4g03050	0,96	1,11	1,17	0,98	1,02	0,92	1,00	1,00	2-oxoglutarate-dependent dioxygenase, putative (AOP3)
255437_at	At4g03060	1,42	1,43	1,52	1,44	1,05	0,97	1,15	0,95	2-oxoglutarate-dependent dioxygenase, putative (AOP2)
255438_at	At4g03070	1,45	1,49	1,19	1,08	1,11	1,38	1,32	1,01	2-oxoglutarate-dependent dioxygenase (AOP1.2)
255439_at	At4g03080	0,81	0,73	0,76	0,82	0,82	0,99	0,97	0,97	kelch repeat-containing serine/threonine phosphoesterase family protein
255409_at	At4g03090	1,03	0,99	1,08	0,93	0,91	0,96	1,03	1,14	expressed protein
255410_at	At4g03100	1,34	1,50	1,18	1,35	1,34	0,98	0,92	0,96	rac GTPase activating protein, putative
255411_at	At4g03110	0,90	0,84	0,92	1,10	0,98	1,09	1,06	1,23	RNA-binding protein, putative

255431_at	At4g03120	1,23	1,36	1,30	1,07	1,28	1,16	1,00	0,96	proline-rich family protein
255413_at	At4g03140	0,93	1,11	1,15	1,18	1,28	0,92	0,87	0,98	short-chain dehydrogenase/reductase (SDR) family protein
255436_at	At4g03150	0,95	0,88	1,54	1,03	1,23	1,05	0,84	0,90	expressed protein
255404_at	At4g03153	0,95	0,95	1,00	1,14	1,01	1,01	1,06	1,06	kinase interacting family protein
255414_at	At4g03156	1,04	0,95	1,04	1,02	1,02	0,99	0,98	0,97	small GTPase-related
255415_at	At4g03160	0,97	0,86	1,06	1,03	1,07	0,92	1,02	0,98	hypothetical protein
255416_at	At4g03170	1,00	1,00	1,04	1,05	1,11	1,07	1,15	1,08	hypothetical protein
255434_at	At4g03180	1,23	0,94	1,10	1,15	0,98	1,09	1,09	1,03	expressed protein
255417_at	At4g03190	1,17	1,08	0,93	0,67	0,93	0,81	0,76	0,93	F-box family protein (FBL18)
255418_at	At4g03200	0,68	0,72	0,78	0,76	0,70	0,98	0,97	0,96	expressed protein
255402_at	At4g03205	1,05	0,98	0,77	0,92	0,85	0,96	1,38	1,24	coproporphyrinogen III oxidase, putative / coproporphyrinogenase, putative
255433_at	At4g03210	1,77	1,48	1,31	1,25	1,25	0,90	0,82	0,70	xyloglucan:xyloglucosyl transferase, putative / xyloglucan endotransglycosy
255419_at	At4g03230	1,07	0,98	0,94	1,09	1,05	0,92	1,19	0,94	S-locus lectin protein kinase family protein
255420_at	At4g03240	0,91	0,99	0,85	0,89	1,11	0,83	0,97	0,79	frataxin protein-related
255421_at	At4g03260	1,00	1,11	1,18	0,89	0,77	1,30	1,47	1,49	leucine-rich repeat family protein
255422_at	At4g03270	1,04	1,06	0,98	0,96	1,01	0,97	0,94	0,94	cyclin family protein
255435_at	At4g03280	1,00	0,89	0,92	0,90	0,94	1,01	1,00	0,98	cytochrome B6-F complex iron-sulfur subunit, chloroplast / Rieske iron-sulfu
255423_at	At4g03290	1,05	1,06	0,98	0,95	1,09	1,01	1,02	0,98	calcium-binding protein, putative
255430_at	At4g03320	1,39	1,43	1,38	1,23	1,07	1,18	1,01	1,17	chloroplast protein import component-related
255432_at	At4g03330	1,00	0,98	1,17	1,08	1,10	0,96	0,96	1,02	syntaxin, putative (SYP123)
255424_at	At4g03340	1,01	0,86	0,80	0,93	0,97	1,00	0,91	1,04	glycosyltransferase family 14 protein / core-2/I-branching enzyme family pro
255425_s_at	At4g03360	0,94	0,97	0,88	1,00	1,02	1,12	0,94	0,94	ubiquitin family protein
255426_at	At4g03370	1,00	0,93	1,00	1,02	1,03	1,03	1,02	0,94	ubiquitin family protein
255427_at	At4g03380	1,01	1,00	1,04	0,91	0,93	0,93	1,01	1,08	expressed protein
255428_at	At4g03390	1,00	0,74	0,67	1,11	0,94	1,17	1,04	1,12	leucine-rich repeat transmembrane protein kinase, putative
255403_at	At4g03400	1,59	0,96	1,22	0,79	1,10	1,05	1,02	0,77	auxin-responsive GH3 family protein
255429_at	At4g03410	0,73	0,64	0,66	0,84	1,14	1,02	1,06	0,96	peroxisomal membrane protein-related
255382_at	At4g03430	1,62	1,49	1,49	0,96	0,96	1,27	1,26	1,52	pre-mRNA splicing factor-related
255405_s_at	At4g03440	1,04	1,01	1,05	0,90	0,83	1,02	1,09	0,97	ankyrin repeat family protein
255406_at	At4g03450	1,02	1,06	1,14	1,03	1,03	0,98	0,94	1,13	ankyrin repeat family protein
255407_at	At4g03480	0,91	0,96	0,95	0,83	1,06	0,97	0,94	0,91	ankyrin repeat family protein
255408_at	At4g03490	1,04	0,89	0,95	1,09	1,09	0,95	1,08	1,03	ankyrin repeat family protein
255377_at	At4g03500	1,06	1,03	0,95	1,10	1,05	0,91	1,05	0,87	ankyrin repeat family protein
255381_at	At4g03510	1,65	1,42	0,99	0,52	0,67	0,91	1,10	1,28	zinc finger (C3HC4-type RING finger) family protein (RMA1)
255379_at	At4g03520	0,86	0,86	0,87	0,86	0,87	0,94	0,91	0,89	thioredoxin M-type 2, chloroplast (TRX-M2)
264691_s_at	At4g03530	1,01	0,94	0,96	1,02	0,97	1,11	0,95	1,00	---
255378_at	At4g03550	0,93	0,74	0,69	1,12	0,81	0,89	0,86	0,81	glycosyl transferase family 48 protein
255380_at	At4g03560	0,91	0,87	0,86	0,86	0,80	0,98	0,98	1,06	two-pore calcium channel (TPC1)
255383_at	At4g03570	0,95	1,01	0,99	1,05	0,81	0,99	1,01	1,20	hypothetical protein
255384_at	At4g03580	0,95	1,13	0,98	1,01	1,13	0,89	1,01	1,01	hypothetical protein
255401_at	At4g03600	0,88	0,98	1,03	0,92	1,00	0,99	0,96	1,13	expressed protein
255385_at	At4g03610	1,10	1,12	1,12	1,05	0,93	0,87	1,07	1,04	phosphonate metabolism protein-related
255386_at	At4g03620	1,01	1,06	0,97	1,09	1,01	1,08	0,99	0,97	myosin heavy chain-related
255387_at	At4g03630	1,00	1,01	1,02	1,08	0,99	1,03	0,98	0,97	root nodule development protein-related
255388_at	At4g03640	0,92	0,94	0,94	1,07	0,97	0,93	0,88	0,99	hypothetical protein
255389_at	At4g03650	1,03	1,02	1,05	1,02	1,00	0,95	1,02	1,05	---

255390_at	At4g03660	1,01	1,06	1,00	1,10	1,00	1,01	1,05	0,91	hypothetical protein
255391_at	At4g03670	0,99	1,05	0,93	0,98	1,02	1,06	0,89	0,93	hypothetical protein
255392_at	At4g03680	1,02	1,02	1,11	0,97	1,00	0,94	0,93	0,91	hypothetical protein
255393_at	At4g03690	0,96	1,04	1,02	0,98	1,00	0,91	0,94	0,98	hypothetical protein
255394_at	At4g03700	1,02	0,97	1,07	1,11	1,09	1,02	1,04	0,93	pseudogene, replication protein-related
255395_at	At4g03710	1,00	0,99	0,93	0,93	0,99	1,08	0,94	1,01	---
255396_at	At4g03720	0,92	1,00	1,05	1,00	0,92	1,05	1,01	0,95	---
255397_at	At4g03730	0,87	1,01	1,03	0,89	1,04	0,98	1,02	1,01	---
255398_at	At4g03740	1,02	0,92	0,94	1,00	0,99	1,00	0,96	1,04	hypothetical protein
255399_at	At4g03750	0,93	1,06	0,96	0,99	0,99	1,14	1,02	0,87	hypothetical protein
255400_at	At4g03760	1,08	1,00	1,03	0,77	0,97	0,87	0,79	1,01	---
255374_at	At4g03770	0,96	1,05	1,00	0,95	0,94	0,95	0,99	1,02	---
255375_at	At4g03780	1,06	1,00	0,98	1,05	1,01	0,93	0,97	0,98	---
255376_x_at	At4g03790	1,18	0,99	0,97	0,99	1,06	0,97	0,85	0,78	---
255346_at	At4g03800	0,94	1,04	0,96	0,90	1,03	1,16	0,95	0,95	---
255347_at	At4g03810	1,04	0,97	1,00	0,96	1,05	0,97	0,96	0,97	---
255348_at	At4g03820	0,90	0,87	0,53	0,83	0,85	0,75	0,67	0,56	expressed protein
255349_at	At4g03830	0,99	0,98	0,95	0,99	1,04	1,01	0,96	1,03	myosin heavy chain-related
255350_at	At4g03840	0,99	0,97	0,98	0,99	1,03	0,97	1,00	1,01	---
255351_x_at	At4g03850	1,06	1,03	0,94	0,96	1,03	0,99	1,05	1,06	---
255352_at	At4g03860	0,97	1,07	0,92	0,95	1,06	0,91	1,01	1,07	---
255353_at	At4g03870	0,93	0,99	1,00	0,90	1,14	0,90	1,01	1,14	---
255354_at	At4g03880	0,97	1,00	1,06	1,03	1,13	0,96	0,97	1,11	---
255355_at	At4g03890	0,96	0,90	0,97	0,85	1,02	0,94	0,91	0,94	hypothetical protein
255356_x_at	At4g03900	0,96	1,05	0,99	1,02	1,05	1,01	1,03	0,88	---
255357_at	At4g03930	0,96	0,90	0,92	0,93	1,13	1,06	1,02	1,06	pectin methylesterase, putative
255358_at	At4g03940	0,94	1,04	1,08	0,98	0,96	1,06	0,95	0,86	expressed protein
255359_at	At4g03950	1,00	0,97	1,16	1,05	1,07	0,98	1,08	0,93	glucose-6-phosphate/phosphate translocator, putative
255360_at	At4g03960	1,07	0,96	1,28	1,25	1,50	1,02	0,84	0,87	tyrosine specific protein phosphatase family protein
263310_s_at	At4g03970	0,96	0,88	0,94	1,03	0,99	0,99	1,06	0,79	Ulp1 protease family protein
255361_at	At4g03990	1,10	0,98	1,05	0,96	1,02	1,05	0,91	1,00	hypothetical protein
255362_at	At4g04000	0,89	0,95	1,12	1,06	0,94	0,98	0,91	1,17	---
255363_x_at	At4g04010	0,88	1,03	1,18	1,01	1,00	1,03	1,08	0,94	pseudogene, Ulp1 protease family
255364_s_at	At4g04020	0,94	0,76	0,84	0,96	1,11	0,95	0,84	0,87	plastid-lipid associated protein PAP, putative / fibrillin, putative
255365_at	At4g04040	1,15	1,35	1,47	0,86	0,85	1,17	1,24	1,20	pyrophosphate--fructose-6-phosphate 1-phosphotransferase beta subunit, [illegible]
255366_x_at	At4g04050	1,02	1,00	1,04	1,03	1,08	1,08	1,08	1,14	---
255367_at	At4g04060	1,01	1,11	1,07	1,02	1,21	0,98	1,02	1,09	---
255368_at	At4g04070	1,09	1,03	1,01	1,00	0,94	1,01	0,96	1,01	hypothetical protein
255369_at	At4g04080	0,99	1,11	1,01	0,98	0,88	0,87	0,94	1,05	iron-sulfur cluster assembly complex protein, putative
255370_at	At4g04100	0,99	0,94	1,02	0,97	0,97	0,90	0,91	0,96	---
255371_at	At4g04110	0,91	0,93	0,99	0,99	0,96	1,05	1,16	1,13	Toll-Interleukin-Resistance (TIR) domain-containing protein
255372_at	At4g04120	0,88	1,08	1,07	1,13	1,12	1,00	1,08	0,83	---
255373_s_at	At4g04130	0,90	1,03	0,97	0,95	1,14	1,02	0,96	1,05	Ulp1 protease family protein
255314_at	At4g04150	0,99	1,08	1,01	0,93	1,15	1,05	0,91	1,00	---
255315_at	At4g04160	0,94	0,98	1,02	1,01	1,11	1,06	0,92	1,17	pseudogene, hypothetical protein
255316_at	At4g04170	1,00	0,94	0,97	1,05	1,05	1,04	0,96	1,13	---

255317_at	At4g04180	1,05	0,91	1,04	0,86	0,98	0,78	0,88	0,86	AAA-type ATPase family protein
255318_at	At4g04190	0,94	0,98	1,25	0,98	1,00	0,93	0,99	0,86	expressed protein
255325_at	At4g04210	0,83	0,80	0,91	0,94	0,84	0,96	1,01	0,89	UBX domain-containing protein
255319_at	At4g04220	1,32	1,05	1,04	0,86	1,00	0,99	0,99	0,87	disease resistance family protein
255320_at	At4g04230	0,88	1,08	0,94	0,96	0,88	0,91	1,01	0,93	---
255321_at	At4g04260	1,08	1,21	1,01	0,94	0,91	0,96	0,80	0,70	bromo-adjacent homology (BAH) domain-containing protein
255322_at	At4g04270	1,05	0,97	0,95	1,20	1,16	1,11	1,13	0,89	---
255323_at	At4g04280	1,00	0,93	1,06	0,98	1,02	1,17	1,15	1,15	---
255324_s_at	At4g04290	0,98	0,93	1,08	0,94	1,03	0,94	0,94	1,02	---
255326_at	At4g04300	1,09	0,97	0,99	1,05	0,97	1,03	0,98	1,08	---
255327_at	At4g04320	0,92	0,99	1,30	1,10	1,11	1,02	1,04	1,07	malonyl-CoA decarboxylase family protein
255331_at	At4g04330	0,56	0,69	0,88	0,58	0,65	1,09	1,15	1,20	expressed protein
255332_at	At4g04340	0,57	0,59	0,73	0,70	0,69	0,84	0,93	1,03	early-responsive to dehydration protein-related / ERD protein-related
255328_at	At4g04350	1,22	1,16	1,03	0,68	0,68	1,35	1,25	1,30	leucyl-tRNA synthetase, putative / leucine--tRNA ligase, putative
255329_at	At4g04370	0,89	1,01	1,03	0,77	0,85	1,09	0,93	1,09	pentatricopeptide (PPR) repeat-containing protein
255330_s_at	At4g04380	1,00	1,04	1,10	0,96	1,14	0,99	1,07	1,06	---
255333_at	At4g04410	0,99	1,01	1,02	0,97	0,97	0,85	0,91	0,67	---
255334_at	At4g04420	0,97	0,95	0,99	1,10	1,02	0,97	0,98	1,04	---
255335_at	At4g04430	0,95	1,03	0,96	0,98	0,96	0,95	1,01	0,93	---
255336_at	At4g04440	0,95	1,00	1,07	1,03	1,14	1,09	0,94	0,92	---
255337_at	At4g04450	0,95	1,08	0,96	0,88	1,17	1,00	1,01	1,01	WRKY family transcription factor
255345_at	At4g04460	0,76	0,99	0,59	1,00	1,08	0,91	1,07	0,75	aspartyl protease family protein
255338_at	At4g04470	1,04	0,98	0,93	1,28	1,18	1,04	1,00	1,05	peroxisomal membrane protein 22 kDa (PMP22)
255339_at	At4g04480	0,99	0,96	1,02	1,01	1,07	0,94	0,84	0,91	hypothetical protein
255340_at	At4g04490	0,96	1,02	1,06	1,01	1,04	1,26	1,15	1,19	protein kinase family protein
255341_at	At4g04500	0,90	1,08	0,96	0,96	0,98	1,05	0,92	1,04	protein kinase family protein
255342_at	At4g04510	0,97	0,97	0,91	0,85	1,04	0,93	1,09	1,23	protein kinase family protein
255343_at	At4g04530	1,02	0,98	1,12	1,09	1,03	1,00	1,01	0,88	Ulp1 protease family protein (snoR29)
255344_s_at	At4g04540	0,86	0,79	1,15	0,86	0,69	0,96	1,11	1,25	protein kinase family protein
255311_at	At4g04550	0,95	1,03	1,00	0,91	0,99	0,97	1,05	0,99	---
255312_at	At4g04560	0,96	1,06	1,01	1,03	1,01	1,04	0,87	0,89	---
255313_at	At4g04590	1,00	0,90	1,01	1,07	0,93	1,08	1,09	0,93	---
255282_at	At4g04600	1,09	0,89	1,07	0,99	1,03	0,91	0,90	1,08	---
255284_at	At4g04610	1,76	1,26	0,86	0,76	0,90	1,07	1,34	1,24	5'-adenylylsulfate reductase (APR1) / PAPS reductase homolog (PRH19)
255283_at	At4g04620	1,27	1,29	1,28	0,96	1,02	1,00	1,03	0,92	autophagy 8b (APG8b)
255285_at	At4g04630	0,88	0,90	1,00	0,89	0,98	0,85	0,97	1,18	expressed protein
255290_at	At4g04640	1,02	0,96	1,00	0,82	0,90	1,01	1,04	1,02	ATP synthase gamma chain 1, chloroplast (ATPC1)
255286_at	At4g04650	0,79	1,00	0,94	1,06	0,95	1,05	1,01	0,96	hypothetical protein
255287_at	At4g04660	0,92	0,92	1,05	0,96	1,04	0,94	0,97	0,98	---
255288_at	At4g04670	1,12	1,01	0,82	0,91	0,81	0,88	0,81	0,92	Met-10+ like family protein / kelch repeat-containing protein
255289_at	At4g04690	0,92	1,05	0,96	0,87	1,16	0,98	0,94	0,93	F-box family protein (FBX15)
255291_at	At4g04700	0,90	1,02	0,90	1,10	1,12	0,98	0,99	1,11	calcium-dependent protein kinase, putative / CDPK, putative
255292_s_at	At4g04710	1,08	0,97	1,17	0,79	1,03	1,15	1,09	1,06	calcium-dependent protein kinase, putative / CDPK, putative
255293_at	At4g04730	0,98	0,99	1,06	1,00	0,98	0,97	1,09	0,87	hypothetical protein
255306_at	At4g04740	1,22	1,42	2,07	0,77	0,88	1,19	1,26	1,29	calcium-dependent protein kinase, putative / CDPK, putative
255294_at	At4g04750	1,02	0,91	0,76	0,84	1,07	0,87	1,09	0,85	sugar transporter family protein

255295_at	At4g04760	0,81	1,01	0,92	0,96	0,94	0,96	1,04	0,90	sugar transporter family protein
255305_at	At4g04770	1,09	1,04	1,00	0,62	0,49	0,71	0,68	0,62	ATP-binding-cassette transporter (ABC1)
255296_at	At4g04790	0,95	0,89	1,02	0,82	0,81	0,89	0,89	0,86	pentatricopeptide (PPR) repeat-containing protein
255301_at	At4g04800	1,10	1,31	1,33	2,00	1,79	1,03	0,98	1,07	methionine sulfoxide reductase domain-containing protein / SeIR domain-co
255297_x_at	At4g04820	0,93	1,01	1,01	0,84	1,09	0,97	1,07	1,00	pseudogene, hypothetical protein
255302_at	At4g04830	1,18	1,25	1,14	1,00	0,96	0,83	1,08	1,07	methionine sulfoxide reductase domain-containing protein / SeIR domain-co
255298_at	At4g04840	1,77	1,39	1,47	1,27	1,02	0,93	0,95	0,98	methionine sulfoxide reductase domain-containing protein / SeIR domain-co
255304_at	At4g04850	1,25	1,32	1,24	0,92	0,94	1,48	1,41	1,56	K+ efflux antiporter, putative (KEA3)
255303_at	At4g04860	0,94	0,97	1,02	1,01	1,17	0,90	0,93	0,99	Der1-like family protein / degradation in the ER-like family protein
255300_at	At4g04870	0,83	0,80	0,73	1,10	1,06	0,90	0,96	0,93	CDP-alcohol phosphatidyltransferase family protein
255299_at	At4g04880	1,33	1,44	1,63	0,98	0,96	1,28	1,09	1,29	adenosine/AMP deaminase family protein
255277_at	At4g04890	0,90	0,89	1,00	0,78	0,73	0,88	0,91	0,95	homeobox-leucine zipper protein protodermal factor 2 (PDF2)
255307_at	At4g04900	0,93	0,94	1,01	1,06	1,07	0,95	0,95	1,15	p21-rho-binding domain-containing protein
255308_at	At4g04910	1,13	1,09	0,86	1,16	1,07	1,07	0,94	0,89	AAA-type ATPase family protein
255309_at	At4g04920	1,12	1,12	1,13	0,93	0,82	1,06	1,14	1,16	expressed protein
255276_at	At4g04930	0,94	1,08	0,93	0,91	1,06	0,88	0,96	1,02	fatty acid desaturase family protein
255278_at	At4g04940	1,22	0,92	0,94	1,00	1,08	1,03	0,90	0,75	transducin family protein / WD-40 repeat family protein
255279_at	At4g04950	1,17	0,96	0,89	1,09	1,00	1,05	1,06	0,84	thioredoxin family protein
255310_at	At4g04955	1,06	1,08	0,81	0,85	1,14	0,83	0,83	0,70	amidohydrolase family protein
255280_at	At4g04960	0,80	0,84	0,90	1,07	0,88	0,86	0,94	1,01	lectin protein kinase, putative
255281_at	At4g04970	1,00	1,06	1,00	0,74	0,65	0,99	0,89	0,90	callose synthase, putative / 1,3-beta-glucan synthase, putative
255251_at	At4g04980	0,96	0,99	1,02	0,96	0,94	1,07	1,04	0,94	hydroxyproline-rich glycoprotein family protein
255252_at	At4g04990	1,38	1,46	1,57	0,98	0,90	1,18	1,42	1,40	expressed protein
255253_at	At4g05000	0,93	0,87	0,98	1,14	0,98	0,95	0,92	0,95	vacuolar protein sorting-associated protein 28 family protein / VPS28 family
255259_at	At4g05020	0,67	0,63	0,62	1,02	0,93	1,04	1,00	0,94	NADH dehydrogenase-related
255254_at	At4g05030	0,87	0,92	0,98	1,08	1,20	1,05	0,91	1,06	heavy-metal-associated domain-containing protein
255260_at	At4g05040	1,01	0,88	1,07	0,76	0,73	0,89	0,96	0,97	ankyrin repeat family protein
255257_at	At4g05050	0,90	0,82	1,09	0,99	1,11	0,93	1,01	1,19	polyubiquitin (UBQ11)
255258_at	At4g05060	1,04	1,05	1,14	1,11	0,97	0,95	0,92	0,94	vesicle-associated membrane family protein / VAMP family protein
255255_at	At4g05070	0,96	0,90	1,00	0,51	0,58	1,08	1,23	1,16	expressed protein
255256_at	At4g05080	0,98	1,00	0,92	1,02	0,96	1,02	1,00	0,91	F-box family protein
255249_at	At4g05090	1,05	1,06	1,17	0,88	0,78	1,18	1,08	1,03	inositol monophosphatase family protein
255250_at	At4g05100	0,96	0,98	0,94	1,82	1,14	1,47	1,50	1,44	myb family transcription factor (MYB74)
255261_s_at	At4g05110	0,84	0,60	0,46	1,33	0,89	0,99	0,82	0,75	equilibrative nucleoside transporter, putative (ENT6)
255262_at	At4g05140	0,96	0,99	1,04	0,88	0,99	0,99	1,05	0,91	equilibrative nucleoside transporter family protein
255221_at	At4g05150	0,75	0,74	0,79	0,68	0,56	0,87	0,84	0,98	octicosapeptide/Phox/Bem1p (PB1) domain-containing protein
255263_at	At4g05160	0,93	0,88	0,95	0,95	0,99	1,09	0,99	1,07	4-coumarate--CoA ligase, putative / 4-coumaroyl-CoA synthase, putative
255264_at	At4g05170	0,95	1,05	0,99	1,04	1,10	1,00	1,12	0,90	basic helix-loop-helix (bHLH) family protein
255248_at	At4g05180	0,91	0,89	0,93	0,95	1,00	0,93	0,94	0,92	oxygen-evolving enhancer protein 3, chloroplast, putative (PSBQ2)
255265_at	At4g05190	1,39	1,38	1,48	1,86	1,56	0,96	0,86	0,79	kinesin-like protein A, putative
255266_at	At4g05200	1,02	1,55	0,96	0,95	0,84	0,97	1,09	1,04	protein kinase family protein
255267_at	At4g05210	1,08	0,86	0,81	0,85	0,93	1,05	1,10	1,26	bacterial transferase hexapeptide repeat-containing protein
255268_at	At4g05230	0,92	1,09	0,99	1,03	1,01	0,99	0,94	1,08	ubiquitin family protein
255269_at	At4g05240	1,03	0,98	1,04	0,97	1,09	0,97	1,08	0,80	ubiquitin family protein
255270_at	At4g05250	1,03	1,00	0,93	0,88	1,05	1,13	1,15	0,97	ubiquitin family protein
255271_at	At4g05260	0,99	0,89	1,09	0,87	1,11	0,90	0,92	0,84	ubiquitin family protein

255272_at	At4g05270	0,96	1,08	1,01	0,86	1,14	1,06	0,97	1,06	ubiquitin family protein
255273_at	At4g05280	1,00	1,00	0,96	0,97	1,03	1,03	1,06	1,09	Ulp1 protease family protein
255274_at	At4g05300	0,94	0,86	0,89	0,88	1,18	0,92	1,06	0,94	hypothetical protein
255275_at	At4g05310	1,09	0,99	0,93	0,88	1,05	0,88	0,97	0,99	ubiquitin family protein
255220_at	At4g05320	0,98	0,97	1,08	1,02	0,90	1,11	0,99	1,12	polyubiquitin (UBQ10) (SEN3)
255232_at	At4g05330	1,07	0,88	0,96	0,84	0,77	1,04	1,02	1,10	zinc finger and C2 domain protein, putative
255222_at	At4g05340	0,99	1,08	1,01	1,01	1,06	1,05	1,06	0,97	hypothetical protein
255223_at	At4g05370	1,61	1,80	1,43	0,99	1,01	1,07	0,97	0,89	hypothetical protein
255230_at	At4g05390	0,84	0,71	0,76	1,64	1,59	0,85	0,84	0,81	ferredoxin--NADP(+) reductase, putative / adrenodoxin reductase, putative
255224_at	At4g05400	1,05	0,88	0,83	1,27	1,14	0,87	0,87	0,95	expressed protein
255225_at	At4g05410	1,26	1,15	1,01	1,04	0,86	1,04	0,91	0,91	transducin family protein / WD-40 repeat family protein
255226_at	At4g05420	1,19	0,98	1,11	0,89	0,87	1,03	0,95	0,94	UV-damaged DNA-binding protein, putative
255227_at	At4g05440	1,27	1,09	1,08	0,93	0,95	1,08	0,87	0,85	temperature sensing protein-related
255233_at	At4g05450	1,13	1,12	1,03	1,22	1,07	1,03	0,96	0,92	adrenodoxin-like ferredoxin 2
255231_at	At4g05460	1,10	1,14	1,17	0,92	1,21	0,94	0,85	1,13	F-box family protein (FBL20)
255228_at	At4g05470	0,99	0,96	0,90	1,01	0,94	1,06	0,99	0,94	F-box family protein (FBL21)
255229_at	At4g05490	1,04	1,02	1,05	0,92	1,05	1,04	1,10	0,85	F-box family protein (FBL22)
255234_at	At4g05500	1,03	1,02	0,99	0,96	1,06	0,94	0,94	1,21	---
255235_at	At4g05510	0,95	1,07	0,87	0,97	1,29	1,08	1,01	0,85	---
255236_at	At4g05520	1,28	1,28	1,52	1,37	1,35	0,89	0,97	0,96	calcium-binding EF hand family protein
255240_at	At4g05530	0,88	0,78	0,70	1,28	1,05	0,91	0,79	0,84	short-chain dehydrogenase/reductase (SDR) family protein
255237_at	At4g05550	0,86	1,04	1,07	0,96	1,04	0,95	1,08	1,07	---
255238_at	At4g05570	1,05	1,00	0,94	0,93	1,21	0,95	0,94	0,98	hypothetical protein
255239_at	At4g05580	1,03	0,97	1,01	1,03	1,02	1,06	1,08	0,97	hypothetical protein
255243_at	At4g05590	1,27	1,00	1,04	1,76	1,42	0,94	0,95	0,89	expressed protein
255241_at	At4g05600	1,01	1,01	0,99	1,05	0,97	0,99	1,06	0,98	---
255242_at	At4g05610	0,94	1,03	0,92	1,05	1,11	1,07	0,93	0,97	---
255244_at	At4g05620	0,83	0,97	1,12	1,04	0,92	0,97	0,93	0,86	F-box family protein
255245_at	At4g05630	1,08	1,08	0,99	0,99	1,08	0,96	1,03	1,03	expressed protein
255246_at	At4g05640	0,93	1,01	0,96	0,81	1,15	1,00	0,85	1,00	hypothetical protein
255247_at	At4g07330	1,00	1,01	0,91	0,91	0,95	0,94	1,02	0,99	---
255188_at	At4g07340	1,05	1,02	1,00	0,98	1,01	1,04	0,97	0,84	replication protein-related
255189_at	At4g07350	0,99	1,03	1,12	0,90	1,05	1,11	0,95	1,11	hypothetical protein
255190_x_at	At4g07360	1,01	0,88	1,05	0,97	0,96	1,04	1,02	1,02	---
255191_at	At4g07370	1,05	0,97	1,16	1,05	1,08	1,03	1,03	1,02	---
255192_at	At4g07380	0,96	1,00	1,01	1,05	1,06	0,95	1,04	0,87	hypothetical protein
255199_at	At4g07390	1,05	1,00	1,00	1,12	1,13	0,82	0,92	0,89	PQ-loop repeat family protein / transmembrane family protein
255193_at	At4g07400	0,77	0,97	1,07	0,77	0,99	0,99	1,03	0,95	F-box family protein (FBL8) (FBL24)
255194_at	At4g07410	1,00	0,88	0,86	0,94	0,69	1,09	1,00	0,99	transducin family protein / WD-40 repeat family protein
255195_at	At4g07420	0,93	0,97	0,99	0,94	1,05	0,98	0,98	1,06	---
255196_x_at	At4g07430	1,06	1,01	1,03	0,93	0,99	1,04	1,06	1,02	hypothetical protein
255197_x_at	At4g07450	0,96	1,02	0,94	1,06	1,02	1,07	0,93	0,82	hypothetical protein
255198_at	At4g07460	0,98	1,01	0,95	1,10	0,99	1,11	0,99	0,91	hypothetical protein
255200_at	At4g07480	1,01	1,03	0,97	0,98	0,98	1,02	0,91	1,06	hypothetical protein
255201_at	At4g07490	1,03	1,00	0,96	0,97	1,00	1,03	0,99	1,08	---
255202_at	At4g07500	0,97	0,98	0,99	0,84	1,07	1,06	1,07	0,92	hypothetical protein

255203_at	At4g07510	0,94	1,16	0,91	1,09	1,01	1,02	0,90	0,95	hypothetical protein
255207_at	At4g07515	1,07	1,06	0,92	1,04	1,02	0,95	1,04	1,00	expressed protein
255204_at	At4g07530	0,92	0,97	0,95	0,87	1,01	1,03	0,93	1,16	hypothetical protein
255205_at	At4g07540	0,91	0,98	1,03	1,03	1,09	1,05	0,99	0,87	---
255206_at	At4g07550	1,02	1,03	1,05	1,06	1,12	1,11	1,05	0,97	---
255208_at	At4g07580	1,00	0,95	0,93	0,99	0,99	1,02	1,00	0,92	---
255209_at	At4g07590	0,99	1,01	1,16	0,97	0,99	0,91	1,08	0,98	---
255210_at	At4g07600	1,13	0,97	0,99	1,00	0,97	1,01	1,01	1,04	---
255211_at	At4g07610	0,95	1,01	0,95	1,01	1,02	0,97	1,09	0,97	---
255212_at	At4g07620	1,00	1,07	0,99	1,01	1,09	0,94	1,00	0,91	---
255213_at	At4g07640	0,96	1,01	0,99	0,98	1,14	0,97	1,09	1,04	---
255214_at	At4g07650	0,94	1,05	0,98	0,85	1,04	1,01	0,95	0,87	pseudogene, hypothetical protein
255215_at	At4g07660	1,02	0,97	1,09	0,94	0,92	0,99	0,98	1,08	---
255216_s_at	At4g07670	0,91	0,90	0,88	1,08	1,12	0,95	1,24	1,39	protease-associated (PA) domain-containing protein
255217_s_at	At4g07680	1,05	0,98	1,05	0,95	1,10	1,07	1,02	1,02	pseudogene, hypothetical protein
255218_at	At4g07700	1,03	1,04	0,99	0,91	0,92	1,04	1,00	1,03	gypsy-like retrotransposon family (Athila)
255219_at	At4g07710	0,89	0,97	0,90	0,99	0,97	0,92	0,93	0,88	---
255184_at	At4g07730	0,99	1,04	1,01	0,97	1,10	1,02	1,05	1,12	---
255185_at	At4g07740	0,95	1,03	1,02	0,90	0,99	1,02	1,03	0,98	hypothetical protein
255186_at	At4g07750	0,94	0,85	0,73	0,94	1,20	1,04	1,08	1,09	---
255187_s_at	At4g07760	1,05	0,96	1,04	1,05	1,11	0,95	1,06	1,02	---
255156_at	At4g07780	0,97	0,96	1,04	0,93	1,02	0,95	0,95	0,92	---
255157_at	At4g07790	1,09	1,04	1,21	1,03	1,01	1,02	1,10	1,09	---
255158_s_at	At4g07800	0,96	0,93	1,00	1,01	1,07	1,02	0,97	1,00	hypothetical protein
255159_at	At4g07810	0,90	0,92	0,95	0,96	0,98	1,08	1,02	1,11	---
255160_at	At4g07820	0,89	1,13	1,09	1,02	0,88	1,28	1,24	1,17	pathogenesis-related protein, putative
255161_at	At4g07830	0,94	1,01	0,99	1,06	1,02	0,98	1,04	0,93	---
255162_at	At4g07840	0,99	0,92	0,97	0,99	1,01	0,93	1,09	1,04	---
255163_at	At4g07850	1,01	0,97	0,96	0,93	1,09	0,97	1,07	1,03	---
255164_at	At4g07880	0,88	0,97	1,05	0,89	1,07	1,10	0,88	0,89	---
255165_at	At4g07890	0,97	0,95	1,00	1,13	0,93	1,11	0,93	0,87	---
255166_at	At4g07900	1,02	1,08	1,06	0,96	0,94	1,10	1,07	0,90	---
255167_at	At4g07920	0,92	1,08	1,02	0,89	1,18	1,00	0,99	1,03	---
255168_at	At4g07930	0,98	0,96	0,92	1,01	1,08	0,93	1,04	0,99	---
255169_x_at	At4g07940	0,93	0,91	0,91	0,95	0,97	0,97	1,02	0,96	hypothetical protein
255176_s_at	At4g07950	1,12	0,91	0,88	0,94	1,06	0,75	0,85	0,66	DNA-directed RNA polymerase III family protein
255175_at	At4g07960	0,96	0,63	0,81	1,23	1,22	0,89	0,95	0,89	glycosyl transferase family 2 protein
255170_at	At4g07970	1,02	0,98	1,06	1,03	0,97	1,04	0,92	0,94	---
255171_at	At4g07990	0,88	0,73	0,78	0,97	1,01	1,11	1,00	0,99	DNAJ heat shock N-terminal domain-containing protein
255172_x_at	At4g08000	1,01	1,06	1,05	0,99	1,03	1,02	0,96	1,08	---
255173_at	At4g08020	0,94	0,99	0,97	1,03	1,02	1,03	1,06	1,05	---
255174_at	At4g08030	1,08	0,99	1,07	1,07	1,02	0,88	1,00	1,02	---
255177_at	At4g08040	1,94	3,25	1,65	1,14	1,13	1,57	1,41	0,98	1-aminocyclopropane-1-carboxylate synthase, putative / ACC synthase, pu
255178_at	At4g08050	1,00	1,02	0,90	0,91	1,08	1,05	0,93	0,99	---
266586_s_at	At4g08060	1,02	1,00	0,98	0,91	1,05	0,98	0,94	1,02	---
255179_at	At4g08090	1,02	0,97	1,02	0,97	1,12	1,06	1,03	1,08	---

255180_at	At4g08100	0,98	1,02	0,94	0,95	1,06	1,00	1,04	0,92	---
255181_at	At4g08110	0,88	0,97	1,10	0,91	0,88	0,93	1,24	1,18	CACTA-like transposase family (Ptta/En/Spm)
255182_at	At4g08130	0,97	1,06	1,08	1,07	1,04	1,01	0,93	1,03	hypothetical protein
255183_at	At4g08140	1,08	1,02	0,88	1,12	1,14	1,02	0,91	1,00	hypothetical protein
255149_at	At4g08150	1,18	1,30	1,55	0,90	0,84	1,12	1,59	1,28	homeobox protein knotted-1 like 1 (KNAT1)
255150_at	At4g08160	1,19	1,29	1,20	1,10	1,16	1,15	1,24	1,54	glycosyl hydrolase family 10 protein / carbohydrate-binding domain-containi
255132_at	At4g08170	0,73	0,90	1,50	1,22	1,30	1,00	1,54	1,96	inositol 1,3,4-trisphosphate 5/6-kinase family protein
255151_at	At4g08180	0,77	0,76	0,75	1,31	1,50	0,87	0,79	0,96	oxysterol-binding family protein
255152_at	At4g08190	1,00	0,98	1,00	0,96	1,05	1,07	0,98	1,05	Ras-related GTP-binding protein, putative
255153_at	At4g08210	0,97	0,88	0,90	0,97	0,89	1,16	0,95	1,01	pentatricopeptide (PPR) repeat-containing protein
255154_at	At4g08220	1,13	1,23	1,08	1,14	1,01	0,98	0,86	1,00	---
255155_at	At4g08230	1,15	1,10	0,93	1,00	1,04	1,06	1,05	1,03	glycine-rich protein
255130_at	At4g08240	0,98	1,05	1,20	1,61	1,31	0,98	0,94	1,01	expressed protein
255125_at	At4g08250	1,03	0,90	1,13	1,08	1,10	0,96	0,83	0,99	scarecrow transcription factor family protein
255126_at	At4g08270	1,01	0,98	1,06	0,98	1,02	1,16	0,96	1,12	hypothetical protein
255131_at	At4g08280	0,97	0,84	0,78	1,00	1,08	0,83	0,86	0,84	expressed protein
255129_at	At4g08290	1,15	0,99	0,77	1,67	1,11	0,80	0,85	0,73	nodulin MtN21 family protein
255127_at	At4g08300	1,05	1,02	0,83	1,23	1,07	0,79	0,76	0,70	nodulin MtN21 family protein
255128_at	At4g08310	1,36	1,08	1,28	1,11	1,25	1,44	1,45	1,34	expressed protein
255133_at	At4g08320	1,11	0,96	1,06	0,92	0,94	0,95	0,87	0,95	tetratricopeptide repeat (TPR)-containing protein
255134_at	At4g08330	0,91	0,97	0,89	0,84	1,03	1,13	0,86	1,09	expressed protein
255135_at	At4g08340	0,97	1,07	1,08	1,02	1,04	1,14	0,99	0,88	Ulp1 protease family protein
255136_s_at	At4g08350	0,95	0,92	0,97	0,85	0,81	0,99	0,94	0,95	KOW domain-containing transcription factor family protein
255137_at	At4g08370	1,00	1,08	0,98	1,02	0,96	1,11	0,92	1,05	proline-rich extensin-like family protein
255138_at	At4g08380	0,94	1,11	0,93	0,96	0,99	1,06	0,89	0,99	proline-rich extensin-like family protein
255142_at	At4g08390	0,91	0,85	0,87	0,94	0,86	0,57	0,68	0,56	L-ascorbate peroxidase, stromal (sAPX)
255139_s_at	At4g08400	1,03	1,00	0,97	1,11	1,12	0,89	1,10	0,96	proline-rich extensin-like family protein
255140_x_at	At4g08410	1,01	1,17	1,11	1,05	1,19	0,96	0,87	0,96	proline-rich extensin-like family protein
255141_at	At4g08420	1,04	0,94	1,08	0,90	0,99	1,00	0,93	1,00	---
255143_at	At4g08430	0,93	1,10	0,94	0,95	1,04	1,01	1,09	1,00	Ulp1 protease family protein
255145_at	At4g08440	1,05	1,04	0,91	0,98	0,95	1,13	1,13	1,06	---
255146_at	At4g08450	1,09	0,94	1,04	1,11	1,00	1,03	1,05	0,98	disease resistance protein (TIR-NBS-LRR class), putative
255147_at	At4g08460	1,02	0,89	0,92	0,99	0,99	1,00	0,89	1,02	expressed protein
255148_at	At4g08470	0,94	0,97	1,18	0,95	1,19	1,06	1,05	0,93	mitogen-activated protein kinase, putative
255121_at	At4g08480	1,28	1,25	1,12	0,95	0,96	0,96	1,22	1,03	mitogen-activated protein kinase, putative
255122_at	At4g08490	0,93	0,98	0,95	1,05	1,00	0,96	0,97	1,06	---
255095_at	At4g08500	0,75	0,82	1,25	0,88	1,03	0,94	1,23	1,21	mitogen-activated protein kinase kinase, putative
255123_at	At4g08530	1,07	0,97	0,93	0,96	0,99	1,00	1,02	0,99	protease inhibitor/seed storage/lipid transfer protein (LTP) family protein
255124_at	At4g08560	1,00	0,95	1,01	1,01	1,01	1,01	1,11	1,00	pumilio/Puf RNA-binding domain-containing protein
255093_s_at	At4g08580	1,26	1,09	1,25	0,82	0,96	1,24	1,22	1,64	microfibrillar-associated protein-related
255094_at	At4g08590	1,17	1,25	1,22	1,29	1,17	1,07	1,05	0,92	zinc finger (C3HC4-type RING finger) family protein
255096_at	At4g08600	0,93	1,03	1,11	1,14	1,04	1,08	1,01	0,91	hypothetical protein
255097_at	At4g08610	0,98	1,11	1,08	1,06	1,02	0,84	1,05	1,02	hypothetical protein
255105_at	At4g08620	0,91	0,92	1,08	1,15	1,19	0,98	0,92	1,05	sulfate transporter
255098_at	At4g08640	0,95	1,09	0,99	0,95	1,01	1,02	0,96	1,00	hypothetical protein
255099_at	At4g08650	0,97	0,90	0,93	0,98	0,97	1,08	0,99	0,95	---

255100_at	At4g08660	0,97	0,96	1,07	0,95	0,98	0,98	1,01	1,08	---
255101_at	At4g08670	1,00	1,07	0,83	1,07	1,12	1,01	0,91	1,09	protease inhibitor/seed storage/lipid transfer protein (LTP) family protein
255102_s_at	At4g08680	0,94	0,99	1,00	1,03	1,11	1,00	1,12	1,13	---
255104_at	At4g08685	1,44	1,11	1,14	2,05	1,49	0,78	0,82	0,84	pollen Ole e 1 allergen and extensin family protein
255103_at	At4g08690	0,97	0,98	1,14	1,12	1,15	1,04	1,00	1,07	SEC14 cytosolic factor family protein / phosphoglyceride transfer family pro
255117_s_at	At4g08700	0,90	0,89	1,02	0,82	0,93	0,99	1,09	1,21	purine permease family protein
255106_at	At4g08710	1,03	0,98	0,95	0,95	0,94	1,08	1,02	1,07	hypothetical protein
255107_at	At4g08730	0,96	1,00	0,99	1,15	1,03	1,13	0,93	1,21	hypothetical protein
255108_at	At4g08740	0,90	1,05	1,16	1,02	1,12	0,99	0,82	0,89	hypothetical protein
255109_s_at	At4g08760	1,00	0,99	1,03	0,90	1,00	1,01	0,95	0,93	RNA recognition motif (RRM)-containing protein
255110_at	At4g08770	1,49	1,84	1,60	1,84	1,64	1,45	1,81	1,51	peroxidase, putative
255111_at	At4g08780	1,42	1,66	1,41	1,95	1,59	0,99	1,11	1,29	peroxidase, putative
255118_at	At4g08790	0,78	0,84	0,96	0,85	0,74	0,90	0,76	0,93	nitrilase, putative
255112_at	At4g08800	1,01	0,99	1,15	0,95	1,03	0,93	1,01	1,00	protein kinase, putative
255113_at	At4g08820	0,91	0,99	1,01	1,08	0,96	0,96	0,97	1,21	hypothetical protein
255114_at	At4g08830	1,03	0,98	1,06	0,96	1,23	0,83	0,92	1,17	---
255115_at	At4g08840	0,92	0,98	0,96	1,06	1,03	0,91	1,13	0,98	pumilio/Puf RNA-binding domain-containing protein
255116_at	At4g08850	0,93	0,75	0,76	0,71	0,60	1,29	1,02	1,05	leucine-rich repeat family protein / protein kinase family protein
255065_s_at	At4g08870	0,93	1,00	0,93	1,45	1,30	1,14	1,00	0,86	arginase, putative
255119_at	At4g08880	1,04	0,98	1,02	1,03	1,08	0,91	0,95	1,02	Ulp1 protease family protein
255120_x_at	At4g08890	1,06	0,94	0,92	1,18	0,99	1,23	1,03	0,95	Mutator-like transposase family
255068_at	At4g08920	1,26	1,52	1,74	0,73	0,78	1,01	1,00	1,14	cryptochrome 1 apoprotein (CRY1) / flavin-type blue-light photoreceptor (H'
255061_at	At4g08930	0,85	0,80	0,96	1,05	1,09	0,98	1,10	1,30	thioredoxin-related
255062_at	At4g08940	0,88	0,99	0,90	0,75	1,04	1,04	1,04	0,97	expressed protein
255064_at	At4g08950	1,18	1,07	0,97	1,05	0,95	1,03	1,21	1,61	phosphate-responsive protein, putative (EXO)
255067_at	At4g08960	0,90	0,92	1,09	1,06	1,00	0,90	0,90	0,97	phosphotyrosyl phosphatase activator (PTPA) family protein
255063_at	At4g08970	0,97	0,88	1,03	1,04	1,00	1,04	0,76	1,07	---
255066_at	At4g08980	1,12	1,26	1,45	1,14	1,34	1,07	1,24	1,34	F-box family protein (FBW2)
255069_s_at	At4g08990	1,16	1,15	1,15	0,97	0,92	1,08	0,98	1,12	DNA (cytosine-5-)-methyltransferase, putative
255079_s_at	At4g09000	0,87	0,81	0,81	1,39	1,26	0,92	0,87	0,95	14-3-3 protein GF14 phi (GRF4)
255078_at	At4g09010	0,77	0,67	0,81	0,80	0,87	1,02	1,04	1,14	L-ascorbate peroxidase, chloroplast, putative
255070_at	At4g09020	0,55	0,47	0,50	0,73	0,78	0,82	0,84	0,83	isoamylase, putative / starch debranching enzyme, putative
255080_at	At4g09030	1,24	1,30	1,05	1,32	1,16	0,97	0,91	1,02	arabinogalactan-protein (AGP10)
255071_at	At4g09050	1,15	1,14	1,23	0,76	0,86	1,06	1,09	0,77	RNA recognition motif (RRM)-containing protein
255072_at	At4g09080	1,13	1,00	1,06	1,00	0,96	0,93	0,99	1,06	chloroplast outer membrane protein, putative
255073_at	At4g09090	1,02	0,94	1,10	1,13	1,09	0,99	0,98	1,11	glycosyl hydrolase family protein 17
255074_at	At4g09100	1,08	1,19	0,96	1,20	1,17	1,05	0,98	0,99	zinc finger (C3HC4-type RING finger) family protein
255075_at	At4g09110	0,99	1,11	1,05	1,03	1,10	0,99	1,03	0,95	zinc finger (C3HC4-type RING finger) family protein
255076_at	At4g09130	1,03	1,10	0,99	1,05	1,17	1,05	0,93	1,17	zinc finger (C3HC4-type RING finger) family protein
255081_at	At4g09140	1,12	1,03	1,25	1,04	1,00	0,93	0,86	1,05	DNA mismatch repair protein MLH1 (MLH1)
255077_at	At4g09150	0,93	0,79	1,05	0,93	0,86	1,09	1,07	1,31	T-complex protein 11
255082_at	At4g09160	0,82	0,75	0,83	0,83	0,87	0,89	0,84	1,04	SEC14 cytosolic factor family protein / phosphoglyceride transfer family pro
255083_s_at	At4g09230	0,92	1,03	0,88	1,02	1,05	0,96	0,95	0,92	pseudogene, hypothetical protein
255084_s_at	At4g09270	0,99	1,03	1,12	0,94	1,20	1,01	0,88	1,00	hypothetical protein
255085_at	At4g09290	0,95	0,86	1,06	0,92	0,98	0,93	0,89	0,82	hypothetical protein
255086_at	At4g09300	0,93	0,96	1,02	1,02	1,08	0,94	0,98	1,04	expressed protein

255089_at	At4g09320	1,47	1,23	1,19	1,26	1,08	1,04	1,00	0,99 nucleoside diphosphate kinase 1 (NDK1)
255087_at	At4g09330	1,42	1,30	1,32	1,06	1,00	1,17	1,19	1,17 SPla/RYanodine receptor (SPRY) domain-containing protein
255088_at	At4g09350	1,52	1,57	1,63	0,84	0,80	1,70	1,62	0,97 DNAJ heat shock N-terminal domain-containing protein
255090_at	At4g09360	1,03	1,13	1,07	0,95	1,11	1,02	0,96	0,90 disease resistance protein (NBS-LRR class), putative
255091_at	At4g09390	0,92	1,07	1,02	1,01	1,07	1,01	0,99	0,96 hypothetical protein
255092_s_at	At4g09400	0,97	0,96	1,03	1,19	0,99	1,02	0,99	0,90 hypothetical protein
255058_s_at	At4g09410	0,96	1,09	0,99	1,03	1,13	1,08	0,97	0,85 Mutator-like transposase family
255059_at	At4g09420	1,05	1,07	1,05	0,90	0,73	1,02	0,83	0,84 disease resistance protein (TIR-NBS class), putative
255060_at	At4g09430	0,95	1,18	0,83	1,01	1,04	1,04	1,07	1,08 disease resistance protein (TIR-NBS-LRR class), putative
255037_at	At4g09460	1,67	1,44	1,14	1,29	1,54	1,07	1,00	1,12 myb family transcription factor
255029_x_at	At4g09470	0,98	1,01	1,09	0,99	1,09	1,04	1,10	1,02 hypothetical protein
255030_at	At4g09480	0,87	0,99	0,96	0,94	1,02	0,93	0,92	0,99 copia-like retrotransposon family
255031_at	At4g09490	1,15	1,15	1,04	0,91	1,02	0,94	0,88	0,84 RNase H domain-containing protein
255032_at	At4g09500	0,96	0,98	0,97	1,00	0,99	0,99	0,89	0,98 glycosyltransferase family protein
255038_at	At4g09510	0,98	0,80	0,95	1,41	1,03	0,76	0,84	0,83 beta-fructofuranosidase, putative / invertase, putative / saccharase, putative
255033_at	At4g09520	1,15	1,07	1,02	0,93	1,33	0,87	0,86	0,94 2,3-biphosphoglycerate-independent phosphoglycerate mutase family prote
255034_at	At4g09540	0,97	1,01	1,10	0,98	1,09	1,09	1,05	0,99 ---
255035_at	At4g09550	1,07	0,88	0,80	1,17	1,07	0,97	0,82	0,83 expressed protein
255036_at	At4g09560	0,80	1,09	1,03	0,87	0,81	0,91	0,75	1,00 protease-associated zinc finger (C3HC4-type RING finger) family protein
255039_at	At4g09570	1,23	1,05	1,13	0,97	0,91	1,08	1,05	0,98 calcium-dependent protein kinase, putative / CDPK, putative
255040_at	At4g09580	1,05	0,93	1,11	0,89	0,91	0,98	1,01	0,96 expressed protein
255048_at	At4g09600	0,29	0,31	0,46	0,42	0,30	0,95	0,99	1,03 gibberellin-regulated protein 3 (GASA3) / gibberellin-responsive protein 3
255049_at	At4g09610	0,88	0,85	0,79	0,82	0,91	0,86	0,99	1,07 gibberellin-regulated protein 2 (GASA2) / gibberellin-responsive protein 2
255041_at	At4g09620	0,98	1,12	1,61	0,79	0,72	1,04	1,34	1,28 expressed protein
255042_at	At4g09630	0,92	0,85	0,77	0,90	1,10	0,92	0,88	0,87 expressed protein
255043_at	At4g09640	0,95	1,00	1,16	1,06	0,96	0,85	0,89	0,84 expressed protein
255046_at	At4g09650	1,16	1,06	1,09	0,90	0,94	0,98	0,93	0,89 ATP synthase delta chain, chloroplast, putative / H(+)-transporting two-sect
255047_at	At4g09670	1,05	1,05	1,24	1,02	1,05	0,98	0,93	1,04 oxidoreductase family protein
255044_at	At4g09680	1,03	1,07	1,10	0,78	0,75	1,20	1,22	1,08 expressed protein
255045_at	At4g09690	0,96	0,88	0,93	0,96	1,01	1,04	1,05	0,91 DC1 domain-containing protein
255050_at	At4g09700	1,05	1,04	1,05	1,06	0,95	1,03	1,07	1,08 hypothetical protein
255051_at	At4g09710	1,04	1,04	0,92	0,95	0,91	0,99	1,10	0,95 ---
255052_at	At4g09720	0,88	0,82	0,82	1,25	1,32	0,86	1,05	0,92 Ras-related GTP-binding protein, putative
255053_at	At4g09730	1,28	1,22	1,43	0,73	0,75	1,18	0,96	1,22 DEAD/DEAH box helicase, putative
255054_s_at	At4g09740	1,01	1,02	1,28	0,87	1,00	1,09	1,00	1,16 glycosyl hydrolase family 9 protein
254998_at	At4g09760	0,94	1,00	0,96	1,08	0,95	1,74	1,57	1,65 choline kinase, putative
255000_at	At4g09800	1,36	1,27	1,25	1,23	1,39	0,95	0,99	0,93 40S ribosomal protein S18 (RPS18C)
255055_at	At4g09810	1,12	0,98	0,89	0,96	0,69	1,16	1,14	1,42 transporter-related
255056_at	At4g09820	0,91	0,96	0,95	0,99	1,00	1,06	1,18	1,12 basic helix-loop-helix (bHLH) family protein
254999_at	At4g09830	1,17	1,18	1,17	1,09	1,07	0,99	0,98	1,07 expressed protein
255057_at	At4g09840	1,02	0,98	1,06	0,99	0,93	1,02	1,01	1,05 expressed protein
255023_at	At4g09850	1,03	0,92	0,97	1,06	0,98	1,01	0,99	1,04 expressed protein
255024_at	At4g09860	1,00	1,06	1,02	0,91	1,12	0,97	0,96	0,96 hypothetical protein
255027_at	At4g09880	0,85	0,97	0,93	1,02	1,15	0,90	0,99	1,22 hypothetical protein
255028_at	At4g09890	0,88	0,99	1,06	0,73	0,92	0,95	1,11	0,93 expressed protein
255025_at	At4g09900	0,84	0,96	1,19	0,71	0,66	0,99	1,14	1,21 hydrolase, alpha/beta fold family protein

255026_at	At4g09910	0,86	1,18	1,35	0,62	0,52	1,12	1,01	0,90	hydrolase, alpha/beta fold family protein
255001_at	At4g09930	0,96	0,96	1,03	0,96	1,04	0,95	1,05	0,89	avirulence-responsive family protein / avirulence induced gene (AIG1) famil
255002_at	At4g09940	1,09	0,88	1,01	0,95	0,99	1,05	0,90	1,11	avirulence-responsive family protein / avirulence induced gene (AIG1) famil
255003_at	At4g09950	1,05	1,15	1,05	1,16	1,23	1,04	1,27	1,00	avirulence-responsive family protein / avirulence induced gene (AIG1) famil
255014_at	At4g09960	1,05	1,05	0,93	1,00	1,00	1,01	1,00	1,03	MADS-box protein (AGL11)
255004_at	At4g09970	0,97	0,93	1,03	0,89	1,01	0,91	0,96	0,94	expressed protein
255015_at	At4g09980	0,91	0,83	0,86	1,13	0,87	0,93	0,89	0,86	methyltransferase MT-A70 family protein
255005_at	At4g09990	1,09	1,02	1,11	1,37	1,25	0,98	0,99	1,16	expressed protein
255013_at	At4g10000	0,74	0,79	0,85	0,86	1,00	0,89	0,89	0,93	expressed protein
255006_at	At4g10010	1,05	0,91	1,05	0,93	1,09	1,07	1,02	1,02	protein kinase family protein
255007_at	At4g10020	0,94	1,00	0,83	0,80	1,03	1,05	1,07	0,96	short-chain dehydrogenase/reductase (SDR) family protein
255012_at	At4g10030	0,95	0,85	0,82	0,80	0,81	0,93	0,79	0,91	hydrolase, alpha/beta fold family protein
255011_at	At4g10040	1,27	0,93	0,80	1,35	1,36	0,90	0,94	0,90	cytochrome c, putative
255010_at	At4g10050	0,91	0,93	0,83	1,08	0,93	1,01	1,02	1,04	hydrolase, alpha/beta fold family protein
255008_at	At4g10060	1,71	1,93	2,43	0,85	0,77	1,38	1,52	1,75	expressed protein
255009_at	At4g10070	1,14	1,10	1,29	0,87	1,01	1,20	1,10	1,12	KH domain-containing protein
255018_at	At4g10090	1,51	1,19	1,15	1,07	1,29	1,18	1,07	1,12	expressed protein
255017_at	At4g10100	1,09	0,99	0,95	1,08	1,36	0,98	0,85	0,83	molybdenum cofactor synthesis family protein
255016_at	At4g10120	2,26	1,83	2,00	1,04	0,85	1,98	1,92	2,20	sucrose-phosphate synthase, putative
255801_at	At4g10130	0,81	1,04	0,91	1,01	1,10	0,81	0,95	1,00	DNAJ heat shock N-terminal domain-containing protein
255810_at	At4g10140	0,88	0,77	0,80	1,03	1,23	0,83	0,81	0,83	expressed protein
255802_s_at	At4g10150	1,04	1,02	0,90	1,14	0,93	1,12	1,08	0,86	zinc finger (C3HC4-type RING finger) family protein
255803_at	At4g10170	0,83	0,79	0,78	0,93	0,82	0,81	0,88	0,74	synaptobrevin-related family protein
255799_at	At4g10180	0,92	0,96	0,91	0,81	0,88	1,05	1,00	0,91	light-mediated development protein 1 / deetiolated1 (DET1)
255800_at	At4g10200	0,97	1,11	0,94	1,00	0,97	1,04	0,91	1,00	hAT dimerisation domain-containing protein / transposase-related
255019_at	At4g10210	0,93	0,90	0,95	1,04	1,25	1,00	1,04	1,10	hypothetical protein
255804_at	At4g10220	1,01	1,04	1,00	0,98	0,96	0,97	1,01	0,99	hypothetical protein
255805_at	At4g10240	0,98	1,07	1,05	0,97	0,98	0,96	1,01	1,00	zinc finger (B-box type) family protein
255811_at	At4g10250	1,00	1,06	1,12	0,92	0,99	1,01	1,12	1,13	22.0 kDa ER small heat shock protein (HSP22.0-ER)
255806_at	At4g10260	0,95	1,02	1,14	0,91	1,00	0,88	0,55	0,49	pfkB-type carbohydrate kinase family protein
255807_at	At4g10270	1,92	1,15	0,97	1,93	2,04	0,38	0,38	0,81	wound-responsive family protein
255808_at	At4g10280	1,55	1,30	1,14	1,38	1,16	1,13	0,89	0,84	expressed protein
255809_at	At4g10300	1,07	0,97	0,97	0,83	0,95	1,21	1,16	1,14	expressed protein
255812_at	At4g10310	1,02	0,99	0,99	0,77	0,65	1,09	1,06	1,03	sodium transporter (HKT1)
255020_at	At4g10320	1,03	0,98	1,00	0,85	1,04	1,03	0,99	0,96	isoleucyl-tRNA synthetase, putative / isoleucine--tRNA ligase, putative
255021_at	At4g10330	1,03	1,18	1,17	1,17	0,96	0,87	1,19	1,19	glycine-rich protein
254970_at	At4g10340	0,99	0,96	1,04	0,94	0,89	1,00	0,99	1,00	chlorophyll A-B binding protein CP26, chloroplast / light-harvesting complex
255022_at	At4g10350	0,92	0,99	1,10	1,20	1,25	0,84	0,92	0,96	no apical meristem (NAM) family protein
254969_at	At4g10360	1,08	0,98	0,93	1,07	0,87	1,18	1,09	1,09	expressed protein
254995_at	At4g10370	1,00	1,05	1,06	1,00	0,97	0,93	0,96	0,96	DC1 domain-containing protein
254971_at	At4g10380	0,64	0,68	0,60	1,07	1,30	0,89	1,06	1,01	major intrinsic family protein / MIP family protein
254996_at	At4g10390	0,89	1,12	0,86	1,94	1,61	0,81	0,97	1,08	protein kinase family protein
254997_s_at	At4g10400	1,29	1,18	0,92	1,01	1,09	1,27	1,01	0,96	F-box family protein
254967_at	At4g10410	0,92	1,06	1,17	1,10	0,99	1,03	1,07	0,92	hypothetical protein
254968_at	At4g10420	0,97	1,06	0,87	0,93	1,13	1,04	0,88	0,97	hypothetical protein
254972_at	At4g10440	1,00	1,00	0,87	1,09	1,21	0,86	0,96	0,88	dehydration-responsive family protein

254980_at	At4g10450	1,48	1,14	1,04	0,98	1,05	1,00	0,85	0,78	60S ribosomal protein L9 (RPL90D)
254973_at	At4g10460	0,93	1,06	0,95	1,01	1,04	0,89	0,96	1,01	---
254982_at	At4g10470	1,04	1,10	1,15	0,69	0,73	1,14	0,92	0,94	expressed protein
254981_at	At4g10480	1,17	1,05	0,95	1,08	1,13	1,03	0,96	1,00	nascent polypeptide associated complex alpha chain protein, putative / alph
254974_at	At4g10490	0,95	0,96	1,06	0,93	0,88	1,03	1,09	0,98	oxidoreductase, 2OG-Fe(II) oxygenase family protein
254975_at	At4g10500	1,47	2,13	2,00	1,84	1,94	2,04	1,85	1,93	oxidoreductase, 2OG-Fe(II) oxygenase family protein
254976_at	At4g10510	1,06	1,00	1,20	1,02	1,08	1,05	0,94	0,97	subtilase family protein
254977_s_at	At4g10520	0,97	0,96	1,00	0,94	1,21	0,97	0,95	1,00	subtilase family protein
254978_at	At4g10540	1,01	1,02	1,04	0,96	0,97	0,91	0,99	1,06	subtilase family protein
254979_at	At4g10550	1,02	0,89	0,91	0,96	1,10	1,14	0,94	1,00	subtilase family protein
254983_at	At4g10560	0,97	0,92	0,92	1,14	1,12	1,07	1,09	1,23	DC1 domain-containing protein
254985_x_at	At4g10580	1,06	1,03	0,96	0,85	0,91	0,96	1,04	1,28	gypsy-like retrotransposon family
254984_s_at	At4g10590	1,03	1,05	1,30	0,90	0,77	1,05	0,94	1,04	ubiquitin carboxyl-terminal hydrolase family protein
254990_at	At4g10610	1,02	1,11	1,36	0,99	0,97	0,90	0,90	0,94	RNA-binding protein, putative
254991_at	At4g10620	1,18	1,16	1,12	1,05	1,05	0,90	0,86	1,14	expressed protein
254986_at	At4g10640	1,02	1,02	1,11	0,93	1,26	1,31	1,00	1,08	calmodulin-binding family protein
254988_at	At4g10670	1,04	1,05	0,95	1,08	1,16	1,00	1,10	1,12	transcription elongation factor-related
254989_at	At4g10690	0,96	1,12	1,14	1,08	1,13	1,07	1,05	0,97	---
254987_s_at	At4g10700	0,96	1,10	0,91	0,92	1,18	1,00	0,98	0,95	hypothetical protein
254992_at	At4g10710	1,13	1,08	1,28	0,94	0,97	1,13	1,17	1,12	transcriptional regulator-related
254993_at	At4g10730	0,98	1,00	1,06	0,84	0,85	1,06	1,19	1,15	protein kinase family protein
254937_at	At4g10750	0,90	0,92	0,96	1,16	1,05	0,78	0,82	0,73	HpcH/HpaI aldolase family protein
254994_at	At4g10760	1,18	1,06	1,17	0,91	1,11	1,22	0,88	0,84	methyltransferase MT-A70, putative
254938_at	At4g10770	0,78	0,85	1,02	0,87	0,75	1,10	1,28	1,47	oligopeptide transporter OPT family protein
254935_at	At4g10780	1,02	0,99	1,04	1,00	1,12	1,01	1,02	0,99	disease resistance protein (CC-NBS-LRR class), putative
254936_at	At4g10790	1,24	1,27	1,65	1,01	1,03	1,18	0,86	0,88	UBX domain-containing protein
254939_at	At4g10800	1,12	0,96	1,11	1,11	1,06	1,03	1,04	1,04	expressed protein
254950_at	At4g10810	1,05	1,01	0,92	1,28	1,32	0,87	0,79	0,87	expressed protein
254940_at	At4g10820	1,03	1,06	0,99	1,00	1,04	1,08	0,95	1,02	F-box family protein
254941_s_at	At4g10830	0,96	1,03	1,01	1,05	1,07	0,91	0,95	1,08	---
254951_at	At4g10840	0,98	0,89	0,93	1,08	1,35	0,89	0,81	0,90	kinesin light chain-related
254960_at	At4g10845	0,99	1,05	1,01	1,00	1,05	1,08	1,06	1,00	hypothetical protein
254956_at	At4g10850	0,85	0,93	0,97	0,89	0,90	0,97	1,05	0,83	nodulin MtN3 family protein
254942_at	At4g10880	0,97	1,12	0,97	1,00	1,02	0,99	1,01	0,98	hypothetical protein
254943_at	At4g10890	0,93	1,12	1,01	1,05	1,13	1,12	1,01	1,23	expressed protein
254954_at	At4g10910	0,87	1,00	0,94	0,83	1,01	0,98	0,93	0,87	expressed protein
254955_at	At4g10920	1,72	1,39	1,48	1,08	1,20	1,22	1,10	1,12	transcriptional coactivator p15 (PC4) family protein (KELP)
254953_at	At4g10925	1,04	1,19	1,41	0,94	1,01	0,99	0,93	0,92	F-box family protein
254944_at	At4g10930	1,39	1,39	2,54	0,80	0,89	1,45	1,75	1,61	expressed protein
254945_at	At4g10940	1,37	1,40	1,68	0,87	0,93	1,29	1,43	1,21	PHD finger family protein
254946_at	At4g10950	0,97	1,01	0,95	1,04	1,06	0,84	1,08	1,09	GDSL-motif lipase/hydrolase family protein
254959_at	At4g10955	0,94	0,91	1,13	1,03	1,07	0,87	0,80	0,99	lipase class 3 family protein
254952_at	At4g10960	0,79	0,65	0,73	1,12	1,01	0,91	0,83	1,13	lipase class 3 family protein
254957_at	At4g10970	0,80	0,85	1,09	1,06	1,12	0,83	1,01	1,38	expressed protein
254947_at	At4g10990	0,92	0,83	0,85	0,91	1,09	1,05	0,95	1,10	---
254948_at	At4g11000	0,82	0,72	0,84	0,87	1,00	0,81	0,86	1,18	ankyrin repeat family protein

254958_at	At4g11010	1,01	0,82	0,80	1,22	1,06	0,92	0,86	0,86	nucleoside diphosphate kinase 3, mitochondrial (NDK3)
254949_at	At4g11020	0,97	1,00	0,97	1,17	0,98	1,11	0,99	0,98	expressed protein
254961_at	At4g11030	1,02	0,90	1,09	1,03	1,10	1,11	1,03	1,09	long-chain-fatty-acid--CoA ligase, putative / long-chain acyl-CoA synthetase
254962_at	At4g11050	1,04	0,92	0,90	1,17	0,94	1,02	1,16	1,32	endo-1,4-beta-glucanase, putative / cellulase, putative
254963_at	At4g11060	1,30	1,17	1,18	1,10	0,98	0,99	0,90	0,91	single-strand-binding family protein
254964_at	At4g11080	1,13	1,12	1,15	1,08	1,19	1,14	1,15	1,14	high mobility group (HMG1/2) family protein
254965_at	At4g11090	0,87	0,93	0,87	1,04	1,26	0,80	0,77	1,01	expressed protein
254911_at	At4g11100	0,97	0,93	1,06	0,91	0,90	1,19	1,01	1,18	expressed protein
254966_at	At4g11110	0,89	1,07	1,43	0,79	0,76	1,12	1,03	1,25	WD-40 repeat family protein / phytochrome A-related
254932_at	At4g11120	1,23	1,06	0,86	1,05	1,15	1,00	0,95	1,00	translation elongation factor Ts (EF-Ts), putative
254933_at	At4g11130	1,10	1,07	1,21	0,82	0,80	0,84	0,99	1,10	RNA-dependent RNA polymerase, putative
254934_at	At4g11140	0,93	0,88	1,07	0,90	1,02	1,10	0,91	0,85	AP2 domain-containing transcription factor, putative
254903_at	At4g11150	1,11	0,94	0,90	1,26	1,18	0,99	0,93	0,98	vacuolar ATP synthase subunit E / V-ATPase E subunit / vacuolar proton pu
254904_at	At4g11160	1,06	0,90	0,94	1,07	0,95	0,99	1,05	0,96	translation initiation factor IF-2, mitochondrial, putative
254905_at	At4g11170	0,97	0,95	0,92	1,03	1,11	1,13	0,99	1,12	disease resistance protein (TIR-NBS-LRR class), putative
254910_at	At4g11175	1,07	0,93	1,10	1,00	0,87	1,09	0,86	0,74	translation initiation factor IF-1, chloroplast, putative
254906_at	At4g11180	0,96	1,06	1,00	0,97	1,02	0,92	0,93	0,92	disease resistance-responsive family protein / dirigent family protein
254907_at	At4g11190	1,25	0,98	0,59	2,91	1,61	0,96	1,02	0,89	disease resistance-responsive family protein / dirigent family protein
254908_at	At4g11200	0,96	1,04	1,04	1,01	1,06	1,03	1,05	0,99	hypothetical protein
254909_at	At4g11210	0,95	0,98	0,78	0,90	0,78	0,99	1,07	1,18	disease resistance-responsive family protein / dirigent family protein
254920_at	At4g11220	1,20	1,14	1,16	1,19	1,12	0,97	1,03	1,00	reticulon family protein (RTNLB2)
254912_at	At4g11230	0,89	1,13	0,95	0,98	1,04	1,10	0,94	0,93	respiratory burst oxidase, putative / NADPH oxidase, putative
254923_at	At4g11240	1,03	1,03	1,13	0,87	1,06	0,97	0,85	0,94	serine/threonine protein phosphatase PP1 isozyme 6 (PP1BG) (TOPP6)
254918_at	At4g11260	1,03	0,93	0,95	1,03	1,00	1,03	0,97	0,98	phosphatase-related
254913_at	At4g11270	0,82	0,74	0,81	0,82	0,89	0,91	0,91	0,81	transducin family protein / WD-40 repeat family protein
254926_at	At4g11280	1,18	4,49	2,73	0,69	0,45	1,99	4,93	3,34	1-aminocyclopropane-1-carboxylate synthase 6 / ACC synthase 6 (ACS6)
254914_at	At4g11290	1,36	1,52	1,25	1,70	1,60	1,04	0,99	0,74	peroxidase, putative
254921_at	At4g11300	0,66	0,92	0,94	0,75	0,99	0,88	0,97	1,11	expressed protein
254915_s_at	At4g11310	0,41	0,42	0,36	0,79	0,67	0,85	0,93	0,67	cysteine proteinase, putative
254924_at	At4g11330	0,99	0,80	1,01	0,85	0,97	0,96	0,97	0,98	mitogen-activated protein kinase, putative / MAPK, putative (MPK5)
254916_at	At4g11340	1,12	1,32	1,22	1,21	1,12	1,01	1,06	0,89	Toll-Interleukin-Resistance (TIR) domain-containing protein
254917_at	At4g11350	0,93	0,99	0,93	1,08	1,37	0,98	0,83	1,14	fringe-related protein
254919_at	At4g11360	0,84	1,33	1,16	0,76	0,91	0,86	0,99	1,22	zinc finger (C3HC4-type RING finger) family protein (RHA1b)
254922_at	At4g11370	0,89	1,07	0,85	1,17	0,90	1,09	1,04	1,11	zinc finger (C3HC4-type RING finger) family protein
254925_at	At4g11380	1,00	0,94	0,97	1,34	1,15	0,93	0,88	0,80	beta-adaptin, putative
254927_at	At4g11400	0,95	0,95	0,94	1,00	1,06	1,05	0,94	1,00	ARID/BRIGHT DNA-binding domain-containing protein / ELM2 domain-cont
254928_at	At4g11410	0,93	0,78	0,67	1,00	0,97	1,16	0,97	0,95	short-chain dehydrogenase/reductase (SDR) family protein
254873_at	At4g11420	1,11	0,95	1,10	0,91	0,88	1,04	1,05	1,25	eukaryotic translation initiation factor 3 subunit 10 / eIF-3 theta / eIF3a (TIF3
254929_at	At4g11440	1,09	0,99	0,92	1,09	1,11	1,13	0,96	0,86	mitochondrial substrate carrier family protein
254930_at	At4g11450	1,00	1,03	1,04	0,91	1,05	1,02	1,10	0,88	expressed protein
254931_at	At4g11460	1,01	1,02	1,10	0,89	1,02	0,86	0,86	1,00	protein kinase family protein
254897_at	At4g11470	0,97	1,00	0,97	0,99	0,92	0,93	0,91	1,17	protein kinase family protein
254898_at	At4g11480	0,92	0,93	1,20	0,95	1,06	0,95	0,99	1,02	protein kinase family protein
254899_at	At4g11490	1,04	0,97	0,90	1,01	1,06	1,02	1,04	1,21	protein kinase family protein
254900_at	At4g11510	0,97	1,04	0,86	0,94	1,17	0,94	0,97	0,99	expressed protein
254901_at	At4g11530	1,08	1,01	1,05	0,87	0,88	1,29	0,96	0,86	protein kinase family protein

254902_at	At4g11550	0,97	0,98	1,18	1,06	1,14	0,93	1,00	1,04	DC1 domain-containing protein
254872_at	At4g11560	0,96	1,06	1,21	0,95	1,04	1,12	1,15	1,15	bromo-adjacent homology (BAH) domain-containing protein
254874_at	At4g11570	0,85	0,98	1,03	0,77	0,78	1,13	1,24	1,41	haloacid dehalogenase-like hydrolase family protein
254875_at	At4g11590	0,98	1,21	1,00	0,94	1,07	1,04	0,96	0,94	F-box family protein
254890_at	At4g11600	0,70	0,75	0,87	0,94	0,94	1,04	1,12	1,15	glutathione peroxidase, putative
254876_at	At4g11610	0,99	1,10	0,77	0,94	1,09	0,93	1,18	0,91	C2 domain-containing protein
254877_at	At4g11640	0,97	0,93	1,05	1,14	1,06	1,09	1,10	0,93	serine racemase, putative
254889_at	At4g11650	1,24	1,82	1,49	2,06	1,88	2,20	1,57	1,41	osmotin-like protein (OSM34)
254878_at	At4g11660	1,06	1,28	1,23	0,98	1,02	1,24	1,13	0,94	heat shock factor protein 7 (HSF7) / heat shock transcription factor 7 (HSTF
254879_at	At4g11670	0,69	0,74	0,69	1,08	1,06	0,75	0,89	0,82	expressed protein
254880_at	At4g11690	0,99	0,91	1,02	0,99	1,11	0,99	0,96	0,88	pentatricopeptide (PPR) repeat-containing protein
254881_at	At4g11700	1,01	0,98	1,10	0,94	1,08	0,98	1,02	1,08	hypothetical protein
254882_s_at	At4g11710	0,89	0,95	0,91	1,03	0,78	1,09	0,98	1,06	non-LTR retrotransposon family (LINE)
254883_at	At4g11720	1,16	0,96	1,04	1,00	1,04	0,98	1,11	0,96	hypothetical protein
254884_at	At4g11730	1,01	1,05	1,00	0,95	1,12	1,14	0,97	0,96	ATPase, plasma membrane-type, putative / proton pump, putative
254891_at	At4g11740	0,97	1,02	1,35	1,10	1,00	1,07	0,97	1,15	ara4-interacting protein, putative (SAY1)
254885_at	At4g11750	0,97	1,19	1,12	0,91	0,96	0,93	1,05	0,93	kelch repeat-containing protein
254886_at	At4g11760	0,99	1,08	1,00	0,88	0,96	1,04	1,06	0,95	expressed protein
254887_at	At4g11770	0,92	0,95	0,98	0,91	1,02	1,01	0,93	0,94	kelch repeat-containing F-box family protein
254888_at	At4g11780	1,09	0,96	1,04	0,95	1,01	0,96	1,00	0,95	hypothetical protein
254844_at	At4g11790	1,44	1,07	1,38	1,01	1,11	1,30	1,03	1,12	Ran-binding protein 1 domain-containing protein / RanBP1 domain-containi
254892_at	At4g11800	1,01	1,04	0,86	1,03	1,10	0,92	1,01	1,00	calcineurin-like phosphoesterase family protein
254845_at	At4g11820	1,05	0,89	0,79	0,85	0,85	0,96	0,82	0,80	hydroxymethylglutaryl-CoA synthase / HMG-CoA synthase / 3-hydroxy-3-m
254893_at	At4g11830	0,87	0,63	0,66	0,79	0,71	0,79	0,87	0,98	phospholipase D gamma 2 / PLD gamma 2 (PLDGAMMA2)
254846_at	At4g11835	1,12	0,91	0,87	1,09	1,05	0,98	0,90	1,03	phospholipase D gamma 2 / PLD gamma 2 (PLDGAMMA2)
254894_at	At4g11840	1,01	0,88	1,10	0,95	1,05	1,10	1,26	1,26	phospholipase D gamma 3 / PLD gamma 3 (PLDGAMMA3)
254847_at	At4g11850	0,89	0,87	0,96	0,86	0,89	0,94	0,91	0,84	phospholipase D gamma 1 / PLD gamma 1 (PLDGAMMA1)
254843_at	At4g11860	1,30	1,31	1,26	0,92	0,94	1,26	1,04	1,47	expressed protein
254895_at	At4g11870	1,03	1,02	1,01	0,92	1,15	0,95	0,93	0,89	hypothetical protein
254896_at	At4g11880	1,03	0,95	0,84	0,94	1,01	1,02	1,07	0,93	MADS-box protein (AGL14)
254869_at	At4g11890	0,86	0,79	0,92	1,43	1,45	0,96	0,88	0,83	protein kinase family protein
254870_at	At4g11900	0,57	0,61	0,60	0,71	0,49	1,47	1,49	1,17	S-locus lectin protein kinase family protein
254871_at	At4g11920	0,97	0,90	1,13	0,92	1,10	1,08	0,95	1,15	WD-40 repeat family protein
254840_at	At4g11930	0,92	1,09	1,11	1,01	1,15	0,93	0,93	1,02	hypothetical protein
254841_at	At4g11940	1,01	0,96	1,15	0,90	0,93	0,93	0,93	0,95	hypothetical protein
254842_at	At4g11950	1,04	0,86	0,90	1,03	1,00	0,84	1,10	0,98	hypothetical protein
254848_at	At4g11960	1,00	0,97	0,96	0,90	0,88	0,80	0,93	1,03	expressed protein
254849_at	At4g11970	1,08	0,99	1,17	0,99	0,90	1,02	1,22	1,14	YT521-B-like family protein
254863_at	At4g11980	0,94	0,86	1,00	0,94	0,88	0,90	1,02	1,00	MutT/nudix family protein
254850_at	At4g12000	0,76	0,75	0,97	1,31	1,02	1,28	1,42	1,73	expressed protein
254851_at	At4g12010	1,02	0,87	0,75	0,70	0,99	1,07	1,13	1,12	disease resistance protein (TIR-NBS-LRR class), putative
254852_at	At4g12020	0,95	1,01	0,97	0,96	1,09	1,07	1,14	1,19	protein kinase family protein
254862_at	At4g12030	1,26	1,42	1,86	1,30	0,79	1,17	1,42	1,44	bile acid:sodium symporter family protein
254861_at	At4g12040	0,84	1,05	1,74	1,39	1,53	1,07	1,23	1,63	zinc finger (AN1-like) family protein
254859_at	At4g12060	1,21	0,98	1,10	1,01	1,09	1,00	1,01	0,93	Clp amino terminal domain-containing protein
254858_at	At4g12070	0,83	0,85	1,50	0,98	1,28	0,93	0,86	1,04	expressed protein

254853_at	At4g12080	1,06	1,09	0,89	1,48	1,44	0,93	1,51	1,67	DNA-binding family protein
254860_at	At4g12110	1,07	1,00	1,07	1,19	1,23	0,97	0,85	0,73	sterol desaturase family protein
254857_at	At4g12120	0,69	0,70	0,73	0,69	0,90	0,93	0,88	1,11	cytokinesis-related Sec1 protein, putative
254854_at	At4g12130	0,88	0,96	0,74	1,00	1,02	0,90	0,79	0,77	glycine cleavage T family protein / aminomethyl transferase family protein
254855_s_at	At4g12140	0,98	0,86	0,97	1,01	1,00	0,96	0,93	1,15	zinc finger (C3HC4-type RING finger) family protein
254856_at	At4g12160	1,02	0,94	1,04	1,05	1,08	1,04	0,92	0,73	---
254864_at	At4g12180	0,95	1,00	0,92	1,12	1,05	1,10	1,04	1,08	---
254865_s_at	At4g12190	1,03	0,94	1,09	0,95	0,92	0,98	0,94	0,82	zinc finger (C3HC4-type RING finger) family protein
254866_at	At4g12220	1,05	0,95	1,07	1,01	0,89	1,04	0,95	1,00	hypothetical protein
254811_at	At4g12230	0,92	0,83	0,90	1,03	1,14	0,85	0,81	0,83	esterase/lipase/thioesterase family protein
254867_at	At4g12240	0,83	0,96	0,87	0,89	0,85	0,81	0,97	0,81	zinc finger (C2H2 type) family protein
254812_at	At4g12250	0,92	0,66	0,66	1,29	1,09	1,06	0,85	0,86	NAD-dependent epimerase/dehydratase family protein
254868_at	At4g12270	0,96	1,05	1,02	1,03	1,03	0,99	0,97	1,10	copper amine oxidase family protein
254833_s_at	At4g12280	0,37	0,36	0,51	0,76	0,81	0,76	0,88	0,91	copper amine oxidase family protein
254834_at	At4g12300	1,10	1,14	1,10	1,61	1,23	1,23	0,82	1,08	cytochrome P450 family protein
254835_s_at	At4g12310	1,02	1,40	1,36	1,06	1,07	1,34	1,45	1,10	cytochrome P450, putative
254836_at	At4g12330	1,03	0,99	0,95	1,27	1,09	0,96	1,04	1,00	cytochrome P450 family protein
254814_at	At4g12340	1,07	0,97	0,90	1,05	1,16	0,99	0,95	1,03	expressed protein
254813_at	At4g12350	1,00	1,01	1,08	1,08	0,93	1,06	1,00	0,98	transcription factor, putative (MYB42)
254837_at	At4g12360	0,95	0,94	1,02	0,99	1,02	0,97	0,98	1,13	protease inhibitor/seed storage/lipid transfer protein (LTP) family protein
254838_at	At4g12380	0,84	1,04	1,06	1,02	1,03	0,96	0,93	0,70	hypothetical protein
254810_at	At4g12390	1,29	1,34	0,90	1,27	1,20	0,99	1,10	1,15	invertase/pectin methylesterase inhibitor family protein
254839_at	At4g12400	1,70	1,26	1,28	1,36	2,01	1,33	1,54	1,64	stress-inducible protein, putative
254809_at	At4g12410	0,83	1,04	0,91	1,24	1,13	0,88	1,00	1,04	auxin-responsive family protein
254815_at	At4g12420	1,19	1,02	0,98	0,96	1,07	0,92	0,94	0,96	multi-copper oxidase, putative (SKU5)
254806_at	At4g12430	1,04	1,20	0,93	0,99	1,03	0,86	0,99	0,93	trehalose-6-phosphate phosphatase, putative
254816_at	At4g12440	1,23	1,33	1,22	1,07	1,05	1,04	0,98	0,86	adenine phosphoribosyltransferase, putative
254817_at	At4g12460	0,91	0,98	1,17	0,84	0,91	1,09	1,22	1,60	oxysterol-binding family protein
254818_at	At4g12470	1,61	1,30	1,28	3,06	2,24	0,76	0,67	0,72	protease inhibitor/seed storage/lipid transfer protein (LTP) family protein
254805_at	At4g12480	0,89	0,78	0,77	3,99	2,12	0,83	0,68	0,72	protease inhibitor/seed storage/lipid transfer protein (LTP) family protein
254832_at	At4g12490	1,17	1,11	1,27	5,83	2,59	1,56	2,54	2,92	protease inhibitor/seed storage/lipid transfer protein (LTP) family protein
254819_at	At4g12500	1,03	1,02	1,03	5,75	1,54	2,08	2,07	2,57	protease inhibitor/seed storage/lipid transfer protein (LTP) family protein
254820_s_at	At4g12510	1,07	1,02	0,67	1,48	1,27	0,92	0,95	0,99	protease inhibitor/seed storage/lipid transfer protein (LTP) family protein
254829_at	At4g12530	0,98	1,04	1,00	1,20	1,24	0,94	1,01	0,95	protease inhibitor/seed storage/lipid transfer protein (LTP) family protein
254821_at	At4g12540	1,02	0,99	0,98	0,96	1,07	1,08	0,91	0,91	expressed protein
254828_at	At4g12550	0,63	0,54	0,36	2,17	2,43	1,32	1,25	1,21	protease inhibitor/seed storage/lipid transfer protein (LTP) family protein
254822_at	At4g12570	1,04	1,05	1,21	0,78	1,04	0,96	0,93	1,02	ubiquitin-protein ligase, putative
254823_at	At4g12580	1,13	1,03	0,93	1,03	0,98	1,06	0,95	0,70	expressed protein
254830_at	At4g12590	1,01	0,94	0,99	1,17	1,12	0,93	0,88	0,93	expressed protein
254831_at	At4g12600	1,72	1,39	1,15	1,03	1,05	1,04	0,94	0,98	ribosomal protein L7Ae/L30e/S12e/Gadd45 family protein
254824_s_at	At4g12620	1,09	1,22	1,06	1,34	0,86	0,96	0,92	1,02	replication control protein, putative
254825_at	At4g12630	1,06	1,09	1,14	1,08	1,07	0,95	0,85	0,77	RNA recognition motif (RRM)-containing protein
254826_at	At4g12640	0,99	1,05	1,20	1,01	1,22	1,04	0,70	0,84	RNA recognition motif (RRM)-containing protein
254827_at	At4g12650	0,96	0,85	0,85	1,12	1,09	0,84	0,81	0,79	endomembrane protein 70, putative
254787_at	At4g12690	1,39	1,28	1,06	0,95	0,88	1,29	1,15	1,00	expressed protein
254807_at	At4g12700	1,12	1,17	1,14	1,05	0,90	1,20	0,97	1,17	expressed protein

254784_at	At4g12720	1,16	1,29	2,08	1,23	1,27	1,10	1,30	1,73	MutT/nudix family protein
254785_at	At4g12730	1,15	0,96	0,85	1,70	1,43	0,82	0,67	0,67	fasciclin-like arabinogalactan-protein (FLA2)
254808_at	At4g12740	0,87	0,98	1,00	0,93	0,92	0,87	1,07	1,14	adenine-DNA glycosylase-related / MYH-related
254778_at	At4g12750	0,94	1,21	1,12	0,55	0,63	0,97	1,08	1,56	expressed protein
254779_at	At4g12760	1,14	0,97	1,30	0,74	0,88	1,35	1,75	1,74	expressed protein
254780_s_at	At4g12770	1,04	0,91	0,77	0,93	1,01	0,88	0,92	0,93	auxilin-related
254788_at	At4g12790	0,72	0,69	0,67	1,04	1,03	0,84	1,03	1,12	ATP-binding family protein
254790_at	At4g12800	0,97	1,00	1,00	0,98	0,99	1,00	0,95	0,95	photosystem I reaction center subunit XI, chloroplast (PSI-L) / PSI subunit \
254783_at	At4g12830	1,26	1,30	1,42	0,65	0,58	1,83	1,67	1,65	hydrolase, alpha/beta fold family protein
254781_at	At4g12840	1,00	1,02	0,96	0,98	1,02	0,91	1,13	0,90	expressed protein
254782_at	At4g12860	1,04	0,91	0,82	1,01	1,09	0,94	1,02	1,31	calcium-binding protein, putative
254789_at	At4g12880	0,96	0,80	0,78	1,66	1,36	0,72	0,64	0,63	plastocyanin-like domain-containing protein
254786_at	At4g12890	1,08	1,15	1,10	1,09	0,96	0,93	0,89	1,09	gamma interferon responsive lysosomal thiol reductase family protein / GIL'
254791_at	At4g12910	0,72	0,71	0,60	0,75	0,77	1,01	1,24	1,16	serine carboxypeptidase S10 family protein
254792_at	At4g12920	1,13	0,90	1,02	0,89	1,20	1,15	1,03	0,86	aspartyl protease family protein
254793_at	At4g12930	0,94	0,91	1,01	0,96	1,25	0,88	1,09	0,98	hypothetical protein
254777_at	At4g12960	1,02	0,96	0,99	1,06	1,05	0,95	0,92	1,12	gamma interferon responsive lysosomal thiol reductase family protein / GIL'
254794_at	At4g12970	1,39	1,22	1,42	0,74	0,91	1,24	1,05	1,05	expressed protein
254746_at	At4g12980	3,08	3,52	2,18	0,53	0,45	1,78	2,13	1,83	auxin-responsive protein, putative
254795_at	At4g12990	0,94	0,96	1,04	1,04	0,89	0,89	1,15	0,93	expressed protein
254796_at	At4g13000	1,12	0,99	1,13	0,95	1,05	0,96	0,99	0,95	protein kinase family protein
254804_at	At4g13010	1,13	1,06	1,15	1,04	0,92	1,20	1,14	1,27	oxidoreductase, zinc-binding dehydrogenase family protein
254747_at	At4g13020	0,76	0,86	0,94	0,70	0,84	0,94	1,23	1,27	serine/threonine protein kinase (MHK)
254797_at	At4g13030	1,29	1,27	1,54	0,85	0,84	1,42	1,84	1,38	expressed protein
254798_at	At4g13050	0,92	0,94	0,95	0,79	0,77	0,98	0,82	0,76	acyl-(acyl carrier protein) thioesterase, putative / acyl-ACP thioesterase, pu
254799_at	At4g13060	0,91	1,01	1,03	1,03	1,15	1,01	0,97	1,17	F-box family protein-related
254800_at	At4g13070	1,03	1,02	0,95	1,03	0,93	0,90	0,88	0,94	group II intron splicing factor CRS1-related
254801_at	At4g13080	0,98	1,12	0,98	0,92	0,80	0,95	0,92	0,90	xyloglucan:xyloglucosyl transferase, putative / xyloglucan endotransglycosy
254802_at	At4g13090	0,97	1,02	1,07	1,02	0,93	1,05	0,93	1,03	xyloglucan:xyloglucosyl transferase, putative / xyloglucan endotransglycosy
254803_at	At4g13100	1,46	1,07	1,09	0,93	0,96	1,09	1,04	1,09	zinc finger (C3HC4-type RING finger) family protein
254748_at	At4g13120	0,94	0,92	0,93	0,84	1,16	0,89	0,93	0,97	---
254749_at	At4g13130	1,11	0,90	1,01	1,01	0,89	1,07	1,07	0,88	DC1 domain-containing protein
254750_at	At4g13140	0,81	0,68	0,82	0,58	0,62	1,04	1,05	0,95	expressed protein
254751_at	At4g13150	0,92	0,91	0,98	0,98	0,78	0,99	0,95	0,90	expressed protein
254752_at	At4g13160	1,05	0,91	0,91	0,79	0,97	0,97	1,17	0,99	expressed protein
254763_at	At4g13170	1,22	1,06	0,96	1,09	1,04	1,03	0,94	0,94	60S ribosomal protein L13A (RPL13aC)
254759_at	At4g13180	0,72	0,87	1,01	0,73	0,65	0,91	0,88	0,97	short-chain dehydrogenase/reductase (SDR) family protein
254753_at	At4g13190	0,99	0,99	1,04	0,86	1,18	1,08	0,92	0,77	protein kinase family protein
254761_at	At4g13195	0,83	0,88	0,92	1,00	1,08	0,93	0,98	1,09	expressed protein
254760_at	At4g13200	0,69	0,56	0,65	0,94	0,89	0,89	0,80	0,84	expressed protein
254754_at	At4g13210	1,07	0,91	0,97	1,07	1,07	0,96	0,92	1,04	pectate lyase family protein
254755_at	At4g13220	1,21	1,10	1,07	0,94	0,66	1,04	0,94	0,90	expressed protein
254762_at	At4g13230	0,97	0,98	1,17	1,10	1,12	1,04	0,83	0,79	late embryogenesis abundant domain-containing protein / LEA domain-cont
254756_at	At4g13235	1,32	1,29	1,01	1,50	1,37	1,26	1,42	1,30	hypothetical protein
254757_at	At4g13240	0,96	0,91	0,97	0,94	1,02	1,06	0,95	0,98	hypothetical protein
254764_at	At4g13250	0,71	0,86	1,14	0,43	0,43	0,72	0,76	0,81	short-chain dehydrogenase/reductase (SDR) family protein

254758_at	At4g13260	0,89	0,83	0,93	0,96	1,16	0,94	1,00	0,83	flavin-containing monooxygenase / FMO (YUCCA2)
254765_at	At4g13270	1,00	0,89	0,84	1,06	1,03	1,08	0,95	0,92	expressed protein
254766_at	At4g13280	0,85	0,88	0,60	0,86	0,72	0,93	1,03	0,79	terpene synthase/cyclase family protein
254767_s_at	At4g13290	0,93	0,90	0,75	0,84	0,92	1,05	1,28	1,21	cytochrome P450 71A19, putative (CYP71A19)
254768_at	At4g13320	0,99	0,98	0,88	0,88	0,90	0,91	0,90	1,30	expressed protein
254769_at	At4g13330	0,83	0,62	0,70	1,12	1,17	0,90	0,84	1,08	expressed protein
254770_at	At4g13340	0,99	0,90	0,71	0,87	0,98	1,20	0,91	0,89	leucine-rich repeat family protein / extensin family protein
254744_at	At4g13345	0,94	1,04	1,13	0,84	0,76	1,11	1,48	1,50	TMS membrane family protein / tumour differentially expressed (TDE) famil
254776_at	At4g13360	0,77	0,79	0,76	1,09	1,12	0,88	0,91	0,89	enoyl-CoA hydratase/isomerase family protein
254771_at	At4g13380	0,99	1,04	0,93	1,04	1,01	0,91	0,93	0,97	heavy-metal-associated domain-containing protein
254772_at	At4g13390	1,01	0,95	0,98	1,10	1,03	0,99	1,08	0,92	proline-rich extensin-like family protein
254773_at	At4g13410	0,81	0,89	0,93	1,13	1,17	1,04	1,00	1,04	glycosyl transferase family 2 protein
254743_at	At4g13420	0,99	1,04	0,90	0,89	1,16	1,00	1,06	0,92	potassium transporter (HAK5)
254742_at	At4g13430	1,19	1,05	1,06	0,97	0,82	0,98	0,95	0,90	aconitase family protein / aconitate hydratase family protein
254774_at	At4g13440	1,02	0,96	1,29	1,08	1,32	0,94	0,92	0,96	calcium-binding EF hand family protein
254775_at	At4g13450	0,89	1,15	1,00	0,93	0,99	1,00	0,90	0,85	universal stress protein (USP) family protein
254745_at	At4g13460	0,74	0,70	0,95	0,93	1,09	0,88	0,83	0,88	SET domain-containing protein (SUVH9)
254714_at	At4g13480	0,96	1,03	0,89	0,99	1,00	0,99	1,03	1,03	myb family transcription factor (MYB79)
254723_at	At4g13510	0,74	0,79	0,89	0,75	0,78	0,92	0,84	0,82	ammonium transporter 1, member 1 (AMT1.1)
254721_at	At4g13520	1,37	1,24	1,07	1,38	1,44	0,93	0,88	0,92	expressed protein
254722_at	At4g13530	1,09	1,21	1,54	0,89	0,85	1,27	1,34	1,37	expressed protein
254715_at	At4g13550	0,71	0,75	0,72	0,87	0,67	1,01	1,13	1,21	lipase class 3 family protein
254716_at	At4g13560	0,75	0,79	0,74	0,88	0,82	0,76	0,85	0,89	late embryogenesis abundant domain-containing protein / LEA domain-cont
254717_at	At4g13570	0,96	0,96	0,92	1,00	1,00	0,99	1,10	1,29	histone H2A, putative
254718_at	At4g13580	1,42	1,17	0,87	2,24	1,39	1,09	1,22	1,15	disease resistance-responsive family protein
254719_at	At4g13600	1,10	1,30	0,84	1,11	1,08	0,92	1,03	0,96	glycosyl hydrolase family protein 17
254720_at	At4g13610	0,91	1,05	1,15	1,07	1,00	1,15	0,97	1,30	DNA (cytosine-5-)-methyltransferase, putative
254724_at	At4g13630	0,76	0,84	0,86	0,90	0,95	0,85	0,90	1,01	expressed protein
254682_at	At4g13640	0,95	1,05	1,68	0,75	0,96	0,89	1,06	1,39	myb family transcription factor
254725_at	At4g13650	0,95	1,19	1,06	0,81	0,91	1,08	0,93	1,17	pentatricopeptide (PPR) repeat-containing protein
254726_at	At4g13660	1,78	1,57	1,76	2,91	2,14	1,23	1,05	1,01	pinoresinol-lariciresinol reductase, putative
254727_at	At4g13670	0,88	0,93	1,01	0,91	1,01	1,02	1,01	1,04	peptidoglycan-binding domain-containing protein
254728_at	At4g13690	1,09	0,96	1,11	1,01	1,14	0,97	0,99	1,10	hypothetical protein
254729_at	At4g13700	1,00	1,00	1,13	0,85	1,05	1,00	1,01	1,05	serine/threonine protein phosphatase family protein
254689_at	At4g13710	0,92	1,00	1,03	1,01	1,07	0,94	0,93	0,81	pectate lyase family protein
254686_at	At4g13720	0,77	0,65	0,61	0,93	0,92	0,80	0,73	0,76	inosine triphosphate pyrophosphatase, putative / HAM1 family protein
254730_at	At4g13730	0,95	1,00	1,04	0,99	0,93	1,07	0,95	0,80	RabGAP/TBC domain-containing protein
254731_at	At4g13740	1,01	0,97	0,88	0,99	1,10	1,08	0,84	0,83	expressed protein
254732_at	At4g13750	0,80	0,92	0,83	0,73	0,82	0,85	0,75	0,95	expressed protein
254733_at	At4g13760	1,04	1,04	0,98	1,03	0,99	1,15	0,99	1,09	glycoside hydrolase family 28 protein / polygalacturonase (pectinase) family
254687_at	At4g13770	1,56	1,52	2,13	1,12	1,17	0,97	0,95	0,82	cytochrome P450 family protein
254734_at	At4g13780	1,17	1,07	1,03	1,12	0,98	0,96	0,91	0,95	methionine--tRNA ligase, putative / methionyl-tRNA synthetase, putative / M
254685_at	At4g13790	0,96	1,01	0,92	1,02	0,91	1,04	1,07	0,89	auxin-responsive protein, putative
254683_at	At4g13800	0,82	0,77	0,84	0,70	0,72	1,70	1,38	1,00	permease-related
254735_at	At4g13810	1,07	1,02	1,10	0,73	1,03	1,34	1,10	1,01	disease resistance family protein / LRR family protein
254736_at	At4g13820	1,07	1,09	1,08	1,17	1,27	1,07	0,95	0,96	disease resistance family protein / LRR family protein

254688_at	At4g13830	1,10	1,35	1,26	0,65	0,66	1,03	1,28	1,60 DNAJ heat shock N-terminal domain-containing protein (J20)
254737_at	At4g13840	1,26	1,17	1,23	1,17	1,12	0,87	0,82	0,85 transferase family protein
254684_at	At4g13850	1,14	1,02	0,96	1,14	1,07	0,86	0,99	0,92 glycine-rich RNA-binding protein (GRP2)
254738_at	At4g13860	0,94	0,98	0,83	0,92	1,11	1,20	1,15	1,35 glycine-rich RNA-binding protein, putative
254739_at	At4g13880	0,98	1,01	0,97	0,90	1,08	0,97	1,01	0,93 leucine-rich repeat family protein
254740_s_at	At4g13890	1,34	1,00	0,91	1,39	1,14	1,04	0,95	0,87 glycine hydroxymethyltransferase, putative / serine hydroxymethyltransfera
254741_s_at	At4g13900	1,13	0,82	0,79	1,37	1,53	1,41	1,23	1,33 pseudogene, similar to NL0D
245356_at	At4g13940	1,05	1,04	1,00	0,95	0,91	0,95	0,93	0,95 adenosylhomocysteinase / S-adenosyl-L-homocysteine hydrolase / AdoHcy
245310_at	At4g13950	0,94	0,86	0,95	1,32	1,48	0,86	0,68	0,64 rapid alkalinization factor (RALF) family protein
245652_at	At4g13960	0,96	0,98	1,02	0,96	1,05	0,99	1,05	1,05 F-box family protein
245283_at	At4g13980	1,05	0,97	1,05	0,93	1,21	0,86	0,98	1,08 heat shock transcription factor family protein
245653_at	At4g13985	0,95	1,02	1,00	1,02	1,02	1,02	0,88	1,00 F-box family protein
245619_at	At4g13990	0,95	0,96	1,05	1,12	1,04	1,05	0,88	0,76 exostosin family protein
245316_at	At4g14000	1,04	1,20	1,35	1,13	1,09	0,93	0,89	0,89 expressed protein
245386_at	At4g14010	1,25	1,41	1,23	1,82	1,84	0,77	0,81	0,70 rapid alkalinization factor (RALF) family protein
245385_at	At4g14020	1,41	1,37	1,40	2,60	3,43	0,82	0,78	0,79 rapid alkalinization factor (RALF) family protein
245285_s_at	At4g14040	1,10	1,04	1,20	0,92	0,90	0,83	0,95	0,85 selenium-binding protein, putative
245620_at	At4g14050	1,15	0,98	1,06	0,97	0,96	0,91	0,91	0,95 pentatricopeptide (PPR) repeat-containing protein
245267_at	At4g14060	0,83	0,92	0,83	1,64	1,25	0,79	0,75	0,64 major latex protein-related / MLP-related
245621_at	At4g14070	0,90	0,83	0,75	0,88	0,74	1,31	1,19	1,22 AMP-binding protein, putative
245622_at	At4g14080	0,93	1,07	1,19	0,97	1,04	1,04	0,94	0,98 glycosyl hydrolase family 17 protein / anther-specific protein (A6)
245624_at	At4g14090	0,88	0,96	1,03	1,07	0,84	1,00	1,11	0,89 UDP-glucoronosyl/UDP-glucosyl transferase family protein
245326_at	At4g14100	1,05	0,99	1,17	0,95	1,02	1,00	0,99	0,92 expressed protein
245623_s_at	At4g14103	1,13	0,97	1,09	0,90	1,02	1,09	1,17	1,01 F-box family protein
245255_at	At4g14110	1,29	1,13	1,27	1,33	1,25	1,04	0,98	1,10 COP9 signalosome subunit, putative / CSN subunit, putative (CSN8)
245325_at	At4g14130	1,53	1,42	1,47	1,60	1,96	0,72	0,61	0,76 xyloglucan:xyloglucosyl transferase, putative / xyloglucan endotransglycosy
245259_at	At4g14150	1,07	1,02	1,07	1,02	1,13	1,07	0,96	0,90 phragmoplast-associated kinesin-related protein (PAKRP1)
245625_at	At4g14160	0,89	0,81	0,90	0,86	0,92	1,00	0,87	0,85 transport protein, putative
245595_at	At4g14170	0,94	1,00	0,86	1,00	1,09	1,00	0,84	0,90 pentatricopeptide (PPR) repeat-containing protein
245596_at	At4g14180	1,06	1,08	0,87	1,07	1,06	1,03	1,08	0,89 expressed protein
245597_at	At4g14190	0,92	0,80	0,87	0,77	0,79	0,99	0,89	0,90 pentatricopeptide (PPR) repeat-containing protein
245598_at	At4g14200	1,04	0,98	1,11	1,16	1,32	0,99	1,04	1,13 expressed protein
245284_at	At4g14210	1,23	1,12	1,15	0,86	0,77	1,24	1,18	1,07 phytoene dehydrogenase, chloroplast / phytoene desaturase (PDS)
245599_at	At4g14220	0,78	0,94	0,86	0,98	0,94	0,98	0,87	1,10 zinc finger (C3HC4-type RING finger) family protein
245600_at	At4g14230	0,86	0,84	0,89	0,83	1,09	0,94	0,84	0,94 CBS domain-containing protein-related
245601_at	At4g14240	1,02	1,11	1,14	1,25	1,15	1,10	1,06	0,95 expressed protein
245602_at	At4g14270	1,00	1,28	1,26	0,90	1,04	0,95	0,83	0,82 expressed protein
245603_at	At4g14280	1,01	1,04	1,05	0,98	1,10	0,89	1,08	1,06 hypothetical protein
245604_at	At4g14290	0,91	1,04	1,07	1,01	1,25	0,97	1,06	1,07 expressed protein
245605_at	At4g14300	1,26	1,35	1,31	0,99	0,87	1,10	1,11	1,14 heterogeneous nuclear ribonucleoprotein, putative / hnRNP, putative
245606_at	At4g14310	0,93	1,16	1,13	1,08	1,28	1,11	1,00	1,08 ---
245311_at	At4g14320	1,77	1,53	1,29	1,36	1,45	1,04	1,00	0,98 60S ribosomal protein L36a/L44 (RPL36aB)
245607_at	At4g14330	1,14	1,02	0,84	1,11	1,40	0,91	0,86	0,99 phragmoplast-associated kinesin-related protein 2 (PAKRP2)
245294_at	At4g14340	1,04	1,12	1,41	1,04	1,40	0,99	0,92	0,96 casein kinase I (CKI1)
245608_at	At4g14350	0,87	0,84	1,30	0,92	1,15	1,03	1,20	1,52 protein kinase family protein
245274_at	At4g14360	0,87	0,74	0,77	0,98	1,03	0,94	0,90	0,90 dehydration-responsive protein-related

245329_at	At4g14365	1,15	1,02	1,71	1,67	1,82	1,10	0,92	1,08	zinc finger (C3HC4-type RING finger) family protein / ankyrin repeat family p
245609_at	At4g14370	0,92	0,98	0,99	0,96	1,02	1,04	1,06	0,99	disease resistance protein (TIR-NBS-LRR class), putative
245610_at	At4g14380	1,06	1,10	1,04	1,03	1,15	1,05	1,01	0,96	expressed protein
245261_at	At4g14385	0,93	0,94	0,92	1,10	1,04	1,10	0,99	1,16	expressed protein
245611_at	At4g14390	0,84	1,05	0,98	1,13	0,98	0,98	1,00	1,16	ankyrin repeat family protein
245265_at	At4g14400	0,89	1,15	0,85	1,00	0,97	1,17	1,04	0,99	ankyrin repeat family protein
245331_at	At4g14410	1,14	1,11	1,33	1,07	1,48	0,96	1,04	1,39	basic helix-loop-helix (bHLH) family protein
245340_at	At4g14420	1,22	1,15	1,03	1,29	1,10	0,99	0,83	0,87	lesion inducing protein-related
245359_at	At4g14430	0,91	0,91	1,26	1,19	1,23	1,06	0,92	0,90	enoyl-CoA hydratase/isomerase family protein
245612_at	At4g14440	0,98	0,97	0,89	1,63	1,86	0,64	0,60	0,60	enoyl-CoA hydratase/isomerase family protein
245613_at	At4g14450	0,91	0,91	0,89	0,96	1,02	1,09	0,98	0,95	Bet1-like SNARE 1-2 / Bet1 / Sft1-like SNARE 14b / BS14b (BET12)
245614_at	At4g14460	1,02	0,97	0,96	1,04	1,08	0,85	1,00	0,92	---
245328_at	At4g14465	1,02	0,90	0,94	1,10	1,09	0,98	0,99	1,16	DNA-binding protein-related
245615_at	At4g14470	0,85	1,10	0,92	0,99	1,06	1,10	0,89	1,00	---
245616_at	At4g14480	0,71	1,06	1,09	0,90	0,92	0,83	0,95	0,83	protein kinase family protein
245617_at	At4g14490	1,06	1,10	1,01	1,15	0,98	0,95	1,15	1,11	forkhead-associated domain-containing protein / FHA domain-containing pr
245269_at	At4g14500	0,86	1,09	1,26	0,86	0,80	0,92	1,04	1,15	expressed protein
245618_at	At4g14510	0,92	0,98	0,86	0,79	0,71	1,11	1,06	1,08	expressed protein
245591_at	At4g14530	0,93	1,09	1,07	0,99	1,00	0,98	0,97	1,16	hypothetical protein
245592_at	At4g14540	0,99	0,95	1,04	1,01	0,92	0,93	0,96	0,85	CCAAT-box binding transcription factor subunit B (NF-YB) (HAP3) (AHAP3
245593_at	At4g14550	1,34	1,50	1,81	1,09	0,98	1,60	1,37	1,20	auxin-responsive AUX/IAA family protein
245397_at	At4g14560	1,88	1,51	1,21	0,73	0,90	1,23	1,22	1,00	auxin-responsive protein / indoleacetic acid-induced protein 1 (IAA1)
245594_at	At4g14570	0,98	0,75	0,82	1,00	1,01	0,90	0,88	0,98	acylaminoacyl-peptidase-related
245563_at	At4g14580	0,98	1,22	1,12	1,07	0,92	1,01	1,33	1,28	CBL-interacting protein kinase 4 (CIPK4)
245564_at	At4g14590	1,10	1,03	0,98	0,90	1,01	1,07	0,84	1,25	expressed protein
245312_at	At4g14600	1,08	0,98	1,18	1,01	1,04	0,92	0,92	0,86	expressed protein
245565_at	At4g14605	1,17	1,02	1,01	0,81	0,73	1,15	0,83	0,91	mitochondrial transcription termination factor-related / mTERF-related
245566_at	At4g14610	0,90	0,85	0,90	1,12	0,91	1,17	0,94	0,96	---
245333_at	At4g14615	1,31	1,22	0,96	0,97	1,01	0,95	0,99	1,05	expressed protein
245567_at	At4g14630	1,97	2,07	2,04	1,53	1,25	2,78	2,26	1,26	germin-like protein (GLP9)
245257_at	At4g14640	0,99	0,99	1,05	0,89	0,92	1,02	1,02	0,84	calmodulin-8 (CAM8)
245568_at	At4g14650	0,87	1,04	1,04	1,01	1,09	0,96	1,27	0,94	expressed protein
245569_at	At4g14660	1,25	0,95	0,88	0,77	0,83	0,93	1,01	1,00	RNA polymerase Rpb7 N-terminal domain-containing protein
245570_at	At4g14670	0,93	1,01	0,86	1,12	1,11	0,87	0,92	1,02	heat shock protein 101, putative / HSP101, putative
245254_at	At4g14680	1,10	1,17	1,42	0,95	0,69	0,89	0,98	1,05	sulfate adenylyltransferase 3 / ATP-sulfurylase 3 (APS3)
245306_at	At4g14690	1,46	1,49	1,08	1,14	1,21	1,25	1,41	1,27	chlorophyll A-B binding family protein / early light-induced protein, putative
245571_at	At4g14695	1,07	0,99	0,93	1,04	0,98	0,99	1,10	1,00	expressed protein
245373_s_at	At4g14716	0,84	0,82	0,87	0,93	1,03	1,00	1,00	0,96	iron-deficiency-responsive protein, putative
245572_at	At4g14720	0,79	1,12	1,22	0,87	1,05	0,90	0,84	0,86	expressed protein
245573_at	At4g14730	0,98	0,98	1,15	0,91	1,14	1,04	0,97	1,02	transmembrane protein-related
245574_at	At4g14750	1,08	1,13	1,04	0,99	1,12	0,95	1,00	1,02	calmodulin-binding family protein
245575_at	At4g14760	1,09	1,05	1,11	0,87	0,89	1,20	1,27	1,35	M protein repeat-containing protein
245576_at	At4g14770	1,32	1,28	1,14	1,15	1,10	0,85	0,96	1,00	tesmin/TSO1-like CXC domain-containing protein
245577_at	At4g14780	0,94	1,19	0,89	0,89	0,94	0,84	1,03	0,93	protein kinase, putative
245578_at	At4g14790	0,95	0,88	0,65	0,98	0,93	1,05	1,03	0,87	ATP-dependent RNA helicase, mitochondrial (SUV3)
245315_at	At4g14800	1,08	0,97	0,91	1,27	1,30	0,89	0,85	0,89	20S proteasome beta subunit D2 (PBD2) (PRCGA)

245579_at	At4g14810	0,85	0,97	1,04	1,04	1,00	1,11	1,09	1,04	hypothetical protein
245322_at	At4g14815	0,91	1,03	1,10	1,01	0,91	1,10	1,01	0,97	protease inhibitor/seed storage/lipid transfer protein (LTP) family protein
245580_at	At4g14820	1,04	1,02	1,02	0,89	0,93	0,93	1,11	1,24	pentatricopeptide (PPR) repeat-containing protein
245581_at	At4g14840	1,01	1,07	1,21	0,81	0,98	1,11	1,01	1,03	expressed protein
245582_at	At4g14850	0,96	1,01	1,11	0,79	0,98	0,98	0,98	1,04	pentatricopeptide (PPR) repeat-containing protein
245396_at	At4g14870	1,17	1,06	1,01	0,92	0,86	1,15	0,95	1,10	expressed protein
245286_at	At4g14880	0,82	0,80	0,78	0,90	0,82	0,84	0,97	0,93	cysteine synthase / O-acetylserine (thiol)-lyase / O-acetylserine sulfhydrylas
245347_at	At4g14890	1,07	1,07	1,30	1,12	0,98	0,93	0,76	0,72	ferredoxin family protein
245398_at	At4g14900	1,20	1,12	1,14	1,30	1,36	0,85	0,87	1,03	hydroxyproline-rich glycoprotein family protein
245287_at	At4g14910	1,09	1,01	1,23	0,98	0,96	1,13	0,92	0,96	imidazoleglycerol-phosphate dehydratase, putative
245583_at	At4g14920	0,84	1,15	1,26	0,99	0,91	1,11	1,03	1,23	PHD finger transcription factor, putative
245330_at	At4g14930	1,21	1,61	1,89	1,06	1,11	1,20	1,28	1,34	acid phosphatase survival protein SurE, putative
245584_at	At4g14940	1,30	1,15	1,13	0,86	1,04	0,89	1,05	1,08	copper amine oxidase, putative
245273_at	At4g14950	0,89	0,92	1,05	1,26	1,37	0,93	0,97	1,07	expressed protein
245270_at	At4g14960	0,86	0,82	0,76	0,86	0,93	0,82	0,81	0,86	tubulin alpha-6 chain (TUA6)
245366_at	At4g14965	1,11	1,04	0,90	0,88	0,98	1,13	1,13	1,01	cytochrome b5 domain-containing protein
245585_at	At4g14970	1,03	0,96	1,04	1,07	0,98	1,00	1,10	0,85	hypothetical protein
245586_at	At4g14980	1,00	0,98	0,92	1,15	1,17	1,02	1,02	0,98	DC1 domain-containing protein
245282_at	At4g14990	1,53	1,68	1,52	1,53	1,47	1,09	1,05	1,08	expressed protein
245372_at	At4g15000	1,49	1,20	1,15	1,26	1,13	1,05	0,93	0,90	60S ribosomal protein L27 (RPL27C)
245298_at	At4g15010	1,13	1,08	1,30	1,08	1,30	0,99	1,08	1,08	mitochondrial substrate carrier family protein
245587_at	At4g15020	0,93	0,90	0,92	0,86	0,86	1,00	0,83	1,20	expressed protein
245588_at	At4g15030	1,02	0,67	1,05	0,94	0,96	1,16	1,31	1,05	expressed protein
245589_at	At4g15040	1,00	1,05	0,96	0,87	0,92	0,92	1,00	0,87	subtilase family protein
264461_s_at	At4g15050	0,96	0,97	0,88	1,19	0,95	1,01	1,07	0,87	expressed protein
245590_at	At4g15060	0,96	0,94	1,02	1,01	1,15	1,03	0,90	1,09	F-box protein-related
248692_s_at	At4g15070	0,98	0,84	0,99	0,98	0,85	1,05	1,00	0,97	DC1 domain-containing protein
245256_at	At4g15090	0,79	0,67	0,71	0,82	0,74	1,25	1,21	1,29	far-red impaired response protein (FAR1) / far-red impaired responsive prot
245292_at	At4g15093	1,19	1,05	0,91	1,43	1,15	0,88	0,87	0,87	catalytic LigB subunit of aromatic ring-opening dioxygenase family
245531_at	At4g15100	0,86	0,84	0,73	1,22	1,11	0,96	0,93	1,20	serine carboxypeptidase S10 family protein
245532_at	At4g15110	0,91	0,79	0,84	0,74	0,67	1,08	1,02	1,03	cytochrome P450 97B3, putative (CYP97B3)
245363_at	At4g15120	1,06	1,15	1,07	1,04	1,09	1,24	1,10	1,00	VQ motif-containing protein
245533_at	At4g15130	1,00	0,88	1,42	0,78	0,88	1,15	1,31	1,94	cholinephosphate cytidylyltransferase, putative / phosphorylcholine transfer
245309_at	At4g15140	1,06	1,07	1,08	1,42	1,43	0,79	0,74	0,73	expressed protein
245534_at	At4g15150	1,03	0,89	1,00	1,08	0,99	1,07	1,02	1,08	glycine-rich protein
245535_at	At4g15160	1,07	1,08	1,16	1,04	1,31	1,00	0,92	1,07	protease inhibitor/seed storage/lipid transfer protein (LTP) family protein
245536_at	At4g15180	0,89	0,90	1,15	0,75	0,92	0,87	1,01	0,94	SET domain-containing protein
245537_at	At4g15190	0,97	0,91	1,01	1,05	1,07	1,04	1,06	0,92	---
245538_at	At4g15200	1,08	1,11	0,98	1,04	0,97	1,04	0,97	0,84	formin homology 2 domain-containing protein / FH2 domain-containing prot
245275_at	At4g15210	0,92	0,77	0,48	5,63	3,93	1,12	0,89	0,64	beta-amylase (BMY1) / 1,4-alpha-D-glucan maltohydrolase
245539_at	At4g15220	1,03	0,94	1,08	1,00	1,08	0,88	0,98	0,97	ABC transporter family protein
245540_at	At4g15230	1,35	1,35	1,29	0,86	0,56	1,30	2,04	1,84	ABC transporter family protein
245541_at	At4g15240	0,97	0,95	0,97	0,87	1,20	1,13	1,11	0,75	fringe-related protein
245542_at	At4g15250	0,94	0,96	1,05	1,17	1,12	0,88	1,01	1,02	zinc finger (B-box type) family protein
245543_at	At4g15260	0,74	0,64	0,83	0,94	0,99	0,73	0,75	0,72	UDP-glucoronosyl/UDP-glucosyl transferase family protein
245544_at	At4g15270	0,78	0,74	0,72	1,03	1,11	1,15	1,03	0,95	glucosyltransferase-related

245545_at	At4g15280	0,91	1,14	0,83	1,02	1,42	1,11	0,88	1,05	UDP-glucoronosyl/UDP-glucosyl transferase family protein
245546_at	At4g15290	0,92	1,08	0,93	1,00	1,16	1,18	1,18	1,05	cellulose synthase family protein
245547_at	At4g15300	0,98	1,04	0,94	1,09	1,02	1,16	1,07	1,09	cytochrome P450 family protein
245548_at	At4g15310	0,87	1,16	0,91	1,01	0,93	1,02	0,90	0,89	cytochrome P450-related
245549_at	At4g15320	0,96	0,92	1,09	1,03	1,16	1,09	1,02	1,25	cellulose synthase family protein
245550_at	At4g15330	0,86	0,75	0,71	1,14	0,94	1,08	0,92	1,30	cytochrome P450 family protein
245258_at	At4g15340	0,88	0,87	0,82	1,46	1,02	1,04	1,28	1,06	pentacyclic triterpene synthase (04C11)
245551_at	At4g15350	1,00	0,96	0,88	1,09	1,22	0,93	1,02	0,90	cytochrome P450 family protein
245552_at	At4g15360	0,99	1,02	0,99	1,15	1,04	0,99	0,93	0,97	cytochrome P450 family protein
245553_at	At4g15370	1,02	1,21	1,13	2,72	1,87	0,95	1,16	1,08	pentacyclic triterpene synthase, putative
245554_at	At4g15380	0,95	0,92	0,94	0,93	0,97	0,98	1,05	0,92	cytochrome P450 family protein
245555_at	At4g15390	0,18	0,22	0,13	1,01	0,83	0,76	0,72	0,65	transferase family protein
245556_at	At4g15400	0,97	1,00	0,97	1,02	0,94	0,95	1,08	0,98	transferase family protein
245557_at	At4g15410	1,22	1,18	1,27	1,22	1,38	1,15	1,03	1,05	UBX domain-containing protein
245248_at	At4g15415	1,06	1,06	0,95	0,89	0,86	1,13	0,92	0,96	serine/threonine protein phosphatase 2A (PP2A) regulatory subunit B' (B'ga
245313_at	At4g15420	1,15	1,06	1,17	1,24	0,94	1,19	1,02	1,27	PRLI-interacting factor K
245558_at	At4g15430	1,19	1,16	0,98	0,91	1,02	0,94	1,07	1,11	early-responsive to dehydration protein-related / ERD protein-related
245253_at	At4g15440	0,69	0,72	0,84	1,00	1,08	0,94	0,93	1,00	hydroperoxide lyase (HPL1)
245559_at	At4g15460	0,96	1,09	1,00	0,98	1,05	1,15	0,94	0,88	glycine-rich protein
245289_at	At4g15470	0,97	0,87	0,80	1,12	1,07	0,99	0,91	0,93	expressed protein
245344_at	At4g15475	0,90	1,25	1,91	0,90	0,89	1,12	1,09	1,31	F-box family protein (FBL4)
245560_at	At4g15480	0,91	1,04	0,85	1,00	1,02	1,01	0,98	1,12	UDP-glucoronosyl/UDP-glucosyl transferase family protein
245352_at	At4g15490	0,52	0,56	0,84	0,79	1,02	0,73	0,74	1,00	UDP-glucoronosyl/UDP-glucosyl transferase family protein
245561_at	At4g15500	0,88	0,93	0,76	0,92	1,09	0,92	0,85	1,02	UDP-glucoronosyl/UDP-glucosyl transferase family protein
245368_at	At4g15510	1,27	1,21	1,36	0,85	0,90	1,33	1,14	1,03	photosystem II reaction center PsbP family protein
245562_at	At4g15520	1,15	1,06	1,25	1,03	1,08	1,04	0,91	0,86	tRNA/rRNA methyltransferase (SpoU) family protein
245528_at	At4g15530	0,90	1,16	1,35	0,66	0,66	1,00	1,16	1,19	pyruvate phosphate dikinase family protein
245321_at	At4g15545	1,16	1,22	1,36	1,04	1,12	1,15	1,08	1,03	expressed protein
245277_at	At4g15550	0,95	0,93	1,05	0,69	0,72	0,75	1,09	0,99	UDP-glucose:indole-3-acetate beta-D-glucosyltransferase (IAGLU)
245281_at	At4g15560	1,08	1,25	1,41	0,91	0,90	1,14	1,26	1,22	1-deoxy-D-xylulose 5-phosphate synthase, putative / 1-deoxyxylulose-5-ph
245529_at	At4g15570	1,16	1,34	1,35	0,96	0,90	1,12	1,07	1,04	tRNA-splicing endonuclease positive effector-related
245530_at	At4g15580	0,96	1,05	1,26	0,99	1,01	0,88	0,91	1,00	---
245500_at	At4g15590	0,89	1,04	1,07	0,97	1,00	1,02	1,00	0,91	---
245317_at	At4g15610	0,83	0,86	0,86	0,98	0,89	1,08	1,16	1,14	integral membrane family protein
245501_at	At4g15620	0,90	0,84	1,24	1,38	1,26	0,96	0,90	0,97	integral membrane family protein
245304_at	At4g15630	0,75	0,61	0,60	1,09	0,61	0,97	0,86	0,70	integral membrane family protein
245502_at	At4g15640	1,26	1,07	1,22	1,08	1,12	0,94	0,94	1,02	expressed protein
245503_at	At4g15650	0,98	0,95	0,95	1,00	1,01	0,96	0,95	1,01	protein kinase-related
245504_at	At4g15660	1,09	0,96	0,97	0,63	0,52	1,43	1,89	1,89	glutaredoxin family protein
245392_at	At4g15680	1,11	1,57	1,07	1,96	3,01	0,96	1,06	0,99	glutaredoxin family protein
245505_at	At4g15690	1,04	1,26	1,09	1,67	1,75	0,79	0,88	0,91	glutaredoxin family protein
245506_at	At4g15700	1,05	1,14	1,23	1,47	3,07	1,24	1,23	1,41	glutaredoxin family protein
245507_at	At4g15710	1,03	0,99	1,12	0,96	0,98	0,87	1,19	1,06	hypothetical protein
245508_at	At4g15720	0,99	0,92	1,03	0,88	0,85	1,05	1,02	0,88	pentatricopeptide (PPR) repeat-containing protein
245509_at	At4g15730	0,99	1,07	1,10	1,08	1,17	1,01	0,98	0,88	expressed protein
245510_at	At4g15740	0,97	1,04	0,81	0,88	1,03	1,00	1,02	1,05	C2 domain-containing protein

245371_at	At4g15750	0,93	0,97	0,98	1,18	1,03	1,03	0,83	1,32	invertase/pectin methylesterase inhibitor family protein
245511_at	At4g15760	1,00	1,01	0,98	1,04	1,15	1,03	0,92	1,01	C2 domain-containing protein
245512_at	At4g15770	1,18	0,99	0,95	0,97	0,86	0,95	0,90	0,78	60S ribosome subunit biogenesis protein, putative
245513_at	At4g15780	1,27	1,22	1,16	0,91	1,03	0,99	0,77	0,85	synaptobrevin-related family protein
245364_at	At4g15790	1,14	1,11	1,21	1,26	1,19	1,11	0,92	0,92	expressed protein
245334_at	At4g15800	0,95	0,80	0,81	1,11	0,99	0,94	0,88	1,03	rapid alkalinization factor (RALF) family protein
245514_at	At4g15805	0,81	1,05	0,96	1,20	0,88	1,00	0,89	0,70	chloroplast outer membrane protein, putative
245515_at	At4g15810	1,03	0,90	0,88	0,75	0,90	0,98	1,01	1,02	chloroplast outer membrane protein, putative
245516_at	At4g15820	1,12	1,10	1,23	1,15	0,97	1,19	1,14	1,02	wound-responsive protein-related
245343_at	At4g15830	2,23	1,86	1,49	2,17	2,12	0,72	0,97	0,72	expressed protein
245517_at	At4g15840	1,01	1,13	1,24	0,72	0,76	1,08	0,97	1,22	expressed protein
245518_at	At4g15850	1,19	1,01	1,11	1,09	0,92	0,93	0,79	0,77	DEAD/DEAH box helicase, putative
245519_at	At4g15860	1,00	0,98	0,95	1,19	0,88	1,03	0,96	0,88	---
245520_at	At4g15870	0,98	1,03	0,96	0,95	0,94	0,98	1,06	1,11	terpene synthase/cyclase family protein
245521_at	At4g15880	1,23	1,12	1,16	0,90	0,99	1,19	1,08	1,03	Ulp1 protease family protein
245522_at	At4g15890	0,96	1,10	0,89	0,98	1,03	0,94	0,93	1,04	expressed protein
245358_at	At4g15900	1,44	1,33	1,27	1,12	1,40	0,99	0,87	0,94	PP1/PP2A phosphatases pleiotropic regulator 1 (PRL1)
245523_at	At4g15910	1,28	1,24	1,12	1,24	1,52	0,88	1,00	1,07	drought-responsive protein / drought-induced protein (Di21)
245524_at	At4g15920	1,02	0,85	0,87	0,94	1,01	1,02	0,94	0,83	nodulin MtN3 family protein
245525_at	At4g15930	1,16	1,18	1,17	1,24	1,21	1,04	0,98	0,99	dynein light chain, putative
245332_at	At4g15940	0,93	0,81	0,79	1,04	1,24	0,91	0,85	0,83	fumarylacetoacetate hydrolase family protein
245526_at	At4g15950	1,03	0,96	1,08	1,07	1,30	0,94	0,96	0,94	expressed protein
245527_at	At4g15960	0,99	0,89	1,15	0,99	1,09	0,79	0,81	1,30	epoxide hydrolase-related
245369_at	At4g15975	0,96	0,94	0,91	1,00	0,99	0,71	0,95	1,08	zinc finger (C3HC4-type RING finger) family protein
245468_at	At4g15980	0,95	1,05	1,08	1,02	0,98	1,06	0,86	1,23	pectinesterase family protein
245353_at	At4g16000	1,17	1,03	0,99	1,42	1,19	1,03	0,95	1,11	expressed protein
245469_at	At4g16010	1,01	1,03	1,00	0,94	1,05	1,00	1,02	1,13	---
245470_at	At4g16020	0,97	1,00	0,90	0,87	1,21	0,90	1,01	1,00	---
245471_at	At4g16030	0,97	0,96	0,91	0,92	1,06	1,08	1,02	1,01	60S ribosomal protein L19, putative
245472_at	At4g16040	0,96	0,91	1,08	1,04	1,02	1,12	0,92	0,89	hypothetical protein
245473_at	At4g16050	1,00	1,06	0,93	0,90	1,03	1,08	1,13	1,02	expressed protein
245474_at	At4g16060	0,66	0,64	0,63	1,13	1,15	0,75	0,67	0,71	expressed protein
245475_at	At4g16080	0,90	0,92	1,02	0,87	1,05	1,01	1,04	0,96	hypothetical protein
245476_at	At4g16090	0,93	0,87	1,02	0,95	0,87	1,06	1,01	1,02	hypothetical protein
245295_at	At4g16100	1,28	1,03	0,58	1,09	0,93	0,97	0,85	0,87	expressed protein
245477_at	At4g16110	0,83	0,78	0,71	1,21	1,13	1,01	0,90	1,00	two-component responsive regulator family protein / response regulator fan
245339_at	At4g16120	0,96	0,84	1,04	1,25	1,16	0,88	0,82	0,94	phytochelatin synthetase-related
245478_at	At4g16130	0,86	0,86	0,89	0,90	0,67	1,06	1,16	1,10	GHMP kinase family protein
245479_at	At4g16140	0,83	0,52	0,49	1,17	0,96	1,02	0,81	0,78	proline-rich family protein
245216_at	At4g16143	0,89	0,84	0,81	1,01	0,96	0,92	0,86	0,83	importin alpha-2, putative (IMPA-2)
245319_at	At4g16146	0,41	0,43	0,57	0,56	0,63	0,97	1,02	1,02	expressed protein
245480_at	At4g16150	1,04	1,04	1,24	1,14	1,23	0,93	0,89	0,95	calmodulin-binding protein
245291_at	At4g16155	1,00	0,80	0,78	0,82	0,82	0,93	0,95	1,02	dihydrolipoamide dehydrogenase 2, plastidic / lipoamide dehydrogenase 2
245335_at	At4g16160	0,41	0,44	0,23	0,63	0,51	0,87	0,99	1,03	mitochondrial import inner membrane translocase subunit Tim17/Tim22/Tim
245481_at	At4g16170	0,92	0,87	0,81	0,74	0,75	0,95	0,92	0,95	expressed protein
245482_at	At4g16180	1,03	0,85	1,00	0,88	0,97	0,90	0,90	0,99	expressed protein

245483_at	At4g16190	0,81	0,85	0,99	0,75	0,86	1,06	1,21	1,23	cysteine proteinase, putative
245484_at	At4g16210	0,71	0,71	0,73	1,22	1,22	0,82	0,80	0,75	enoyl-CoA hydratase/isomerase family protein
245485_at	At4g16230	1,01	0,95	0,93	1,12	1,14	0,99	1,09	0,98	GDSL-motif lipase/hydrolase family protein
245486_x_at	At4g16240	1,09	1,08	1,09	1,42	1,69	0,92	1,07	1,25	hypothetical protein
245487_at	At4g16250	0,74	0,80	1,09	0,89	0,91	0,89	0,84	0,84	phytochrome D (PHYD)
245393_at	At4g16260	1,06	1,39	1,05	1,21	0,74	0,71	0,74	0,81	glycosyl hydrolase family 17 protein
245367_at	At4g16265	0,91	0,92	0,77	1,36	1,20	0,90	0,85	0,65	DNA-directed RNA polymerase II, putative
245488_at	At4g16270	0,91	1,17	1,09	0,80	1,29	1,09	1,03	0,98	peroxidase 40 (PER40) (P40)
245489_at	At4g16280	1,19	1,15	1,55	0,97	1,05	1,12	1,19	1,60	flowering time control protein / FCA gamma (FCA)
245490_at	At4g16310	0,83	0,94	1,09	0,81	0,88	1,01	1,18	1,08	amine oxidase family protein / SWIRM domain-containing protein
245491_at	At4g16320	0,85	0,87	1,07	0,75	0,81	0,90	1,09	1,41	---
245360_at	At4g16330	0,94	1,02	1,02	0,85	0,90	0,93	0,95	0,96	oxidoreductase, 2OG-Fe(II) oxygenase family protein
245492_at	At4g16340	0,82	0,86	0,82	1,15	0,92	1,01	1,06	0,77	adapter protein SPIKE1 (SPK1)
245300_at	At4g16350	0,99	1,04	0,85	1,02	0,94	1,15	0,90	0,82	calcineurin B-like protein 6 (CBL6)
245260_at	At4g16360	0,80	0,80	0,75	1,01	0,98	0,91	0,80	0,73	5'-AMP-activated protein kinase beta-2 subunit, putative
245296_at	At4g16370	1,87	2,10	2,67	1,38	1,15	2,11	2,09	2,09	oligopeptide transporter OPT family protein
245493_at	At4g16380	0,91	0,94	1,17	1,07	1,02	1,01	0,96	0,89	---
245494_at	At4g16390	0,85	0,65	0,71	0,75	0,77	0,91	0,89	0,82	chloroplastic RNA-binding protein P67, putative
245495_at	At4g16400	1,01	1,13	1,09	0,95	1,01	1,21	0,94	1,19	expressed protein
245388_at	At4g16410	1,06	0,88	0,85	0,74	0,69	1,09	0,95	0,97	expressed protein
245288_at	At4g16420	1,11	1,20	1,38	0,77	0,75	1,09	1,19	1,40	transcriptional adaptor (ADA2b)
245271_at	At4g16430	0,90	0,96	0,92	1,08	0,95	0,94	0,83	0,97	basic helix-loop-helix (bHLH) family protein
245496_at	At4g16440	1,03	1,03	1,50	1,01	1,08	1,12	1,19	1,65	iron hydrogenase family protein
245338_at	At4g16442	0,83	0,78	0,76	1,12	0,93	0,91	0,80	0,76	integral membrane family protein
245320_at	At4g16444	0,91	0,88	0,75	0,79	0,95	0,97	0,85	0,92	expressed protein
245341_at	At4g16447	0,94	1,13	0,91	1,08	1,22	1,05	1,08	1,15	expressed protein
245394_at	At4g16450	1,41	1,27	1,17	1,26	1,11	1,02	0,98	0,96	expressed protein
245497_at	At4g16460	1,10	0,99	1,01	0,99	1,05	1,12	1,10	1,10	hypothetical protein
245498_at	At4g16470	1,00	0,96	1,08	0,93	1,01	1,00	1,16	1,05	pentatricopeptide (PPR) repeat-containing protein
245499_at	At4g16480	0,96	0,94	1,04	0,79	0,73	0,92	0,89	0,91	sugar transporter family protein
245290_at	At4g16490	1,03	1,29	1,46	1,34	1,27	0,96	0,99	1,15	armadillo/beta-catenin repeat family protein
245323_at	At4g16500	0,75	0,69	0,67	0,95	1,16	0,85	0,79	0,83	cysteine protease inhibitor family protein / cystatin family protein
245297_at	At4g16510	1,14	0,96	1,08	0,71	0,89	0,92	0,89	0,91	YbaK/prolyl-tRNA synthetase-related
245336_at	At4g16515	1,22	1,62	1,81	1,10	1,66	0,83	0,88	0,99	expressed protein
245391_at	At4g16520	1,00	1,16	1,26	0,78	0,81	1,12	1,31	1,36	autophagy 8f (APG8f)
245262_at	At4g16563	0,54	0,51	0,44	0,84	0,56	0,92	0,76	0,67	aspartyl protease family protein
245337_at	At4g16566	0,94	1,12	1,06	1,27	1,15	0,99	0,89	0,91	histidine triad family protein / HIT family protein
245268_at	At4g16570	0,73	0,78	0,86	0,89	0,97	0,81	0,90	1,02	protein arginine N-methyltransferase-related
245465_at	At4g16590	1,01	1,05	0,92	0,99	1,13	1,00	0,92	1,18	glucosyltransferase-related
245466_at	At4g16600	0,99	1,00	0,99	1,11	1,04	1,06	1,16	0,91	glycogenin glucosyltransferase (glycogenin)-related
245467_at	At4g16610	0,97	1,02	0,99	1,00	1,05	0,97	0,93	0,89	zinc finger (C2H2 type) family protein
245436_at	At4g16620	0,87	1,02	0,97	1,00	1,14	1,12	1,02	1,02	integral membrane family protein / nodulin MtN21-related
245437_at	At4g16630	1,08	1,03	1,14	1,07	0,98	1,21	0,99	1,09	DEAD/DEAH box helicase, putative (RH28)
245345_at	At4g16640	1,02	0,97	0,97	0,93	0,94	0,97	0,96	1,01	matrix metalloproteinase, putative
245438_at	At4g16650	0,97	0,85	0,82	1,01	0,87	0,92	0,83	0,94	expressed protein
245293_at	At4g16660	1,27	0,90	0,75	1,01	0,94	1,18	1,11	1,28	heat shock protein 70, putative / HSP70, putative

245439_at	At4g16670	0,97	1,30	1,00	0,97	0,80	0,98	1,17	0,97	expressed protein
245440_at	At4g16680	1,53	1,43	1,41	0,96	0,87	1,75	1,96	1,85	RNA helicase, putative
245349_at	At4g16690	0,71	0,84	1,02	0,70	0,81	1,42	1,29	1,33	esterase/lipase/thioesterase family protein
245441_at	At4g16700	1,25	1,03	1,14	1,08	1,16	0,95	0,94	0,99	phosphatidylserine decarboxylase
245442_at	At4g16710	0,96	0,96	1,08	0,99	1,03	1,19	1,30	1,64	glycosyltransferase family protein 28
245342_at	At4g16720	1,20	1,06	1,02	1,01	1,00	0,90	0,96	0,85	60S ribosomal protein L15 (RPL15A)
245443_at	At4g16730	1,04	1,03	1,01	1,12	1,00	1,08	1,01	0,89	terpene synthase/cyclase family protein
245444_at	At4g16740	0,97	1,06	0,99	0,97	0,90	0,94	1,00	1,12	terpene synthase/cyclase family protein
245314_at	At4g16745	1,08	1,16	1,17	1,09	0,92	0,97	0,85	1,16	exostosin family protein
245445_at	At4g16750	0,93	0,99	0,92	0,87	1,06	0,96	1,03	1,05	DRE-binding transcription factor, putative
245249_at	At4g16760	0,87	0,82	0,76	1,10	1,05	0,85	0,86	0,85	acyl-CoA oxidase (ACX1)
245307_at	At4g16770	0,70	0,72	0,66	1,04	1,08	0,91	0,86	0,86	oxidoreductase, 2OG-Fe(II) oxygenase family protein
245276_at	At4g16780	1,23	1,98	1,35	0,88	0,84	1,13	1,15	1,23	homeobox-leucine zipper protein 4 (HAT4) / HD-ZIP protein 4
245384_at	At4g16790	1,15	1,03	1,02	1,73	2,02	0,96	1,02	0,99	hydroxyproline-rich glycoprotein family protein
245446_at	At4g16800	1,32	0,99	0,92	1,06	1,21	0,98	1,10	1,08	enoyl-CoA hydratase, putative
245447_at	At4g16820	1,18	1,02	1,11	0,67	0,67	1,20	1,39	1,19	lipase class 3 family protein
245350_at	At4g16830	1,03	0,99	1,00	1,07	1,07	0,99	0,95	1,03	nuclear RNA-binding protein (RGGA)
245370_at	At4g16840	0,76	0,65	0,62	1,21	1,45	0,97	0,81	0,78	expressed protein
245280_at	At4g16845	0,85	0,82	1,02	1,05	1,24	1,05	0,96	1,09	vernalization 2 protein (VRN2)
245327_at	At4g16850	0,96	0,98	1,01	0,96	0,83	0,81	0,88	0,74	expressed protein
245448_at	At4g16860	0,88	1,01	1,02	0,65	0,51	1,27	2,45	2,06	disease resistance protein (TIR-NBS-LRR class), putative
245449_at	At4g16870	1,11	0,88	0,83	0,66	0,60	1,79	2,07	2,00	---
245450_at	At4g16880	1,01	1,02	0,99	1,06	0,86	0,81	1,26	1,02	disease resistance protein-related
245451_at	At4g16890	0,92	1,04	1,53	0,88	0,69	1,04	1,42	1,42	disease resistance protein (TIR-NBS-LRR class), putative
245453_at	At4g16900	1,00	1,06	1,05	0,83	0,92	1,15	0,92	1,08	disease resistance protein (TIR-NBS-LRR class), putative
245452_at	At4g16910	1,06	1,01	0,95	0,96	1,07	0,97	1,07	0,91	---
245454_at	At4g16920	0,93	1,18	1,22	0,94	1,08	0,90	1,01	1,14	disease resistance protein (TIR-NBS-LRR class), putative
245455_at	At4g16930	1,02	0,95	1,00	1,06	0,98	0,96	0,89	0,94	disease resistance protein (TIR-NBS-LRR class), putative
245456_at	At4g16950	0,90	1,07	1,63	0,68	0,53	1,18	1,43	1,70	disease resistance protein (TIR-NBS-LRR class), putative
245457_s_at	At4g16960	1,06	1,18	1,11	1,17	0,98	1,15	1,49	1,09	disease resistance protein (TIR-NBS-LRR class), putative
245458_at	At4g16970	0,93	1,29	0,98	0,94	1,19	0,96	0,97	0,81	protein kinase family protein
245459_at	At4g16980	0,97	1,00	0,93	1,00	0,72	1,01	0,83	0,92	---
245318_at	At4g16985	1,12	1,08	0,90	1,27	0,93	1,08	0,86	0,93	arabinogalactan-protein family
245460_at	At4g16990	1,21	1,22	1,16	1,22	1,03	1,18	1,26	1,22	disease resistance protein (TIR-NBS class), putative
245461_at	At4g17000	1,06	0,91	1,17	1,18	1,24	0,96	1,05	0,90	hypothetical protein
245303_at	At4g17010	1,03	1,02	1,15	1,24	1,49	1,06	0,98	0,81	expressed protein
245462_at	At4g17020	1,06	1,26	1,22	0,91	0,90	1,08	1,12	1,26	transcription factor-related
245463_at	At4g17030	0,84	0,88	0,95	1,79	1,58	0,79	0,77	0,75	expansin-related
245400_at	At4g17040	0,95	0,92	0,99	1,01	0,96	1,01	1,02	1,11	ATP-dependent Clp protease proteolytic subunit, putative
245464_at	At4g17050	0,99	0,85	0,84	0,98	1,05	0,93	0,90	0,86	expressed protein
245430_at	At4g17060	0,84	0,88	0,87	0,88	1,00	1,05	0,89	1,06	expressed protein
245266_at	At4g17070	1,04	0,93	0,99	1,47	1,21	0,92	0,80	0,94	expressed protein
245431_at	At4g17080	1,30	1,10	1,17	0,96	1,30	0,94	1,03	1,03	MORN (Membrane Occupation and Recognition Nexus) repeat-containing [illegible]
245346_at	At4g17090	0,76	0,90	1,37	0,60	0,61	1,28	1,35	1,29	beta-amylase (CT-BMY) / 1,4-alpha-D-glucan maltohydrolase
245432_at	At4g17100	0,82	0,79	0,88	0,87	0,81	0,88	0,89	0,92	serine protease protein -related, expressed pseudogene
245433_at	At4g17110	0,52	0,58	0,53	0,63	0,80	0,86	0,87	0,87	expressed protein

245434_at	At4g17120	0,57	0,51	0,55	0,81	0,82	0,92	0,83	0,82	expressed protein
245435_at	At4g17130	1,08	1,07	1,08	0,90	0,77	0,93	1,06	0,98	hypothetical protein
245405_at	At4g17150	0,80	0,86	1,08	1,10	1,11	0,97	1,09	1,05	expressed protein
245406_at	At4g17160	0,97	0,99	0,91	0,97	1,35	1,03	0,87	1,08	Ras-related GTP-binding protein, putative
245407_at	At4g17170	1,09	0,89	0,89	0,95	1,13	0,93	0,90	0,94	Rab2-like GTP-binding protein (RAB2)
245408_at	At4g17180	0,79	0,82	1,05	0,84	1,14	0,94	0,75	0,76	glycosyl hydrolase family 17 protein
245301_at	At4g17190	0,95	0,76	0,77	1,07	1,01	0,89	0,91	0,84	farnesyl pyrophosphate synthetase 2 (FPS2) / FPP synthetase 2 / farnesyl
245409_at	At4g17200	1,11	0,90	0,99	1,01	1,10	0,96	1,01	1,02	F-box family protein
245305_at	At4g17215	1,08	1,22	1,10	0,94	1,02	1,02	1,10	0,96	expressed protein
245410_at	At4g17220	1,00	1,01	0,94	0,85	0,90	0,91	1,13	1,11	expressed protein
245247_at	At4g17230	0,60	1,19	1,37	0,75	0,97	1,11	1,41	1,26	scarecrow-like transcription factor 13 (SCL13)
245411_at	At4g17240	0,72	0,74	0,87	1,01	1,20	0,91	0,75	0,74	expressed protein
245264_at	At4g17245	0,79	0,89	0,78	1,21	1,22	0,71	0,76	0,79	zinc finger (C3HC4-type RING finger) family protein
245272_at	At4g17250	0,72	0,74	1,05	0,71	0,87	1,04	1,06	0,94	expressed protein
245324_at	At4g17260	0,91	0,87	1,11	1,49	1,76	0,81	0,63	0,75	L-lactate dehydrogenase, putative
245279_at	At4g17270	0,90	1,07	1,13	1,27	1,38	0,95	0,91	0,97	Mo25 family protein
245412_at	At4g17280	1,12	1,42	1,06	1,21	1,23	0,96	1,05	1,10	auxin-responsive family protein
245413_at	At4g17300	0,97	0,93	0,89	0,72	0,74	1,08	1,10	1,10	asparaginyl-tRNA synthetase, chloroplast, mitochondrial / asparagine-tRNA
245414_at	At4g17310	1,03	0,90	0,81	0,99	1,02	0,95	0,85	1,00	expressed protein
245415_at	At4g17330	0,85	0,91	0,92	1,01	1,05	1,05	1,08	1,10	agenet domain-containing protein
245399_at	At4g17340	1,27	1,15	0,97	4,96	4,15	1,07	0,91	0,88	major intrinsic family protein / MIP family protein
245416_at	At4g17350	1,07	1,17	1,05	1,33	1,34	0,93	0,98	1,15	expressed protein
245417_at	At4g17360	1,16	1,11	1,22	0,93	0,80	1,48	1,39	1,51	formyltetrahydrofolate deformylase, putative
245418_at	At4g17370	0,85	0,88	1,17	0,68	0,69	1,44	1,52	1,73	oxidoreductase family protein
245419_at	At4g17380	0,97	0,99	1,07	0,96	0,92	0,94	1,01	0,94	DNA mismatch repair MutS family protein
245355_at	At4g17390	1,35	1,26	1,11	0,99	0,85	1,01	0,95	0,89	60S ribosomal protein L15 (RPL15B)
245420_at	At4g17410	0,97	0,75	0,96	0,97	1,23	1,07	0,87	1,01	expressed protein
245395_at	At4g17420	1,10	1,07	1,19	1,21	1,48	1,04	0,91	0,95	expressed protein
245421_at	At4g17430	1,04	1,03	1,00	1,16	1,06	0,93	0,91	1,07	expressed protein
245362_at	At4g17460	1,70	1,99	1,24	0,75	0,99	1,01	1,31	1,42	homeobox-leucine zipper protein 1 (HAT1) / HD-ZIP protein 1
245422_at	At4g17470	0,45	0,34	0,39	1,69	1,45	0,55	0,41	0,46	palmitoyl protein thioesterase family protein
245389_at	At4g17480	1,06	0,90	1,01	0,98	1,14	1,04	0,96	1,00	palmitoyl protein thioesterase family protein
245423_at	At4g17483	1,14	0,83	1,10	0,99	1,05	0,84	0,76	1,06	palmitoyl protein thioesterase family protein
245308_at	At4g17486	1,02	0,99	1,46	1,10	1,16	0,89	0,87	1,07	expressed protein
245250_at	At4g17490	0,95	1,12	1,46	0,66	0,63	1,02	1,35	1,75	ethylene-responsive element-binding protein, putative
245252_at	At4g17500	0,84	0,91	0,92	0,77	0,69	0,87	1,04	0,93	ethylene-responsive element-binding protein 1 (ERF1) / EREBP-2 protein
245424_at	At4g17505	1,04	0,97	1,07	1,11	1,19	1,01	1,03	1,02	expressed protein
245425_at	At4g17510	1,28	1,14	1,14	1,11	1,24	0,90	0,89	0,78	ubiquitin carboxyl-terminal hydrolase, putative / ubiquitin thiolesterase, puta
245387_at	At4g17520	1,12	0,98	0,92	0,87	1,03	0,95	1,06	1,14	nuclear RNA-binding protein, putative
245299_at	At4g17530	0,99	0,83	0,85	1,27	1,20	1,08	1,08	1,01	Ras-related GTP-binding protein, putative
245426_at	At4g17540	1,29	0,89	1,09	0,85	0,87	1,55	1,35	1,43	expressed protein
245427_at	At4g17550	0,63	0,67	0,56	1,10	0,89	0,84	0,80	0,77	transporter-related
245357_at	At4g17560	1,11	0,91	0,98	0,89	0,91	1,08	1,02	0,99	ribosomal protein L19 family protein
245428_at	At4g17570	0,93	0,94	1,01	0,89	0,69	1,14	1,12	1,08	zinc finger (GATA type) family protein
245429_at	At4g17580	0,89	1,02	1,08	0,99	1,10	1,00	0,99	0,92	Bax inhibitor-1 family protein / BI-1 family protein
245402_at	At4g17585	0,93	0,97	0,85	0,92	1,03	1,16	0,90	1,07	hypothetical protein

245403_at	At4g17590	0,81	0,92	0,96	1,12	1,03	0,97	1,04	1,04 nucleolar protein-related
245354_at	At4g17600	1,05	1,01	1,14	0,77	0,67	1,14	1,02	0,93 lil3 protein
245404_at	At4g17610	0,92	0,94	0,68	0,84	0,73	0,90	0,96	0,99 tRNA/rRNA methyltransferase (SpoU) family protein
245251_at	At4g17615	0,54	0,90	0,83	0,81	1,10	1,02	1,45	1,22 calcineurin B-like protein 1 (CBL1)
245374_at	At4g17620	1,11	1,03	1,13	0,85	0,90	1,10	1,16	1,08 glycine-rich protein
245351_at	At4g17640	0,94	0,87	0,92	0,93	0,93	1,04	0,93	1,05 casein kinase II beta chain, putative
245390_at	At4g17650	0,88	0,97	1,04	1,09	0,95	0,86	0,78	0,93 aromatic-rich family protein
245375_at	At4g17660	1,05	1,11	1,06	0,95	1,07	1,05	0,99	1,20 protein kinase, putative
245401_at	At4g17670	1,22	1,31	0,76	0,99	1,21	0,94	0,64	0,44 senescence-associated protein-related
245376_at	At4g17690	1,09	0,92	0,91	1,09	1,00	0,92	1,04	0,93 peroxidase, putative
245302_at	At4g17695	1,27	1,00	1,18	1,23	0,89	1,09	0,98	0,96 myb family transcription factor (KAN3)
245377_at	At4g17700	0,94	1,12	1,10	0,99	0,98	1,13	0,96	0,93 hypothetical protein
245378_at	At4g17710	1,15	1,27	1,19	0,98	1,04	0,95	0,82	0,95 homeobox-leucine zipper family protein / lipid-binding START domain-conta
245365_at	At4g17720	0,97	0,83	1,20	1,27	1,26	1,17	0,96	0,95 RNA recognition motif (RRM)-containing protein
245278_at	At4g17730	0,76	0,73	0,76	0,80	0,97	0,90	0,86	0,92 syntaxin 23 (SYP23) / PEP12-like protein
245263_at	At4g17740	1,07	0,88	0,85	0,77	0,91	1,09	1,04	0,99 C-terminal processing protease, putative
245379_at	At4g17750	1,08	1,07	0,96	0,94	1,11	1,28	1,11	1,00 heat shock factor protein 1 (HSF1) / heat shock transcription factor 1 (HSTF
245380_at	At4g17760	1,02	1,36	1,10	0,91	0,97	0,87	0,88	1,19 expressed protein
245348_at	At4g17770	1,10	1,33	1,15	1,11	0,85	0,93	0,94	0,91 glycosyl transferase family 20 protein / trehalose-phosphatase family protei
245381_at	At4g17785	0,98	1,00	0,94	0,87	1,15	1,06	0,93	0,88 myb family transcription factor (MYB39)
245361_at	At4g17790	1,09	1,23	1,21	0,96	1,16	0,98	1,01	0,91 expressed protein
245382_at	At4g17800	0,89	0,87	0,68	0,77	0,71	1,05	1,53	1,66 DNA-binding protein-related
245383_at	At4g17810	0,82	0,83	0,82	0,77	0,69	1,27	1,03	1,19 zinc finger (C2H2 type) family protein
254690_at	At4g17830	1,19	1,00	0,95	1,00	1,04	1,07	0,99	0,91 peptidase M20/M25/M40 family protein
254691_at	At4g17840	0,82	0,90	1,14	0,77	0,76	1,20	1,32	1,38 expressed protein
254692_at	At4g17860	1,00	1,10	1,05	0,89	1,07	0,99	1,12	0,86 hypothetical protein
254705_at	At4g17870	1,04	0,76	0,69	1,39	1,09	0,99	0,87	0,94 expressed protein
254693_at	At4g17880	0,95	0,93	0,81	0,99	1,09	0,99	0,97	1,18 basic helix-loop-helix (bHLH) family protein
254709_at	At4g17890	0,91	0,77	0,87	1,03	1,03	0,90	0,83	0,82 human Rev interacting-like family protein / hRIP family protein
254708_at	At4g17895	1,05	1,10	1,21	0,98	0,78	1,01	0,98	0,92 ubiquitin-specific protease 20, putative (UBP20)
254694_at	At4g17900	1,23	1,48	1,11	0,70	0,84	1,02	1,23	1,27 zinc-binding family protein
254695_at	At4g17910	1,10	1,09	0,93	0,92	1,04	0,91	1,07	0,91 zinc finger (C3HC4-type RING finger) family protein / pentatricopeptide (PP
254696_at	At4g17930	1,01	0,98	0,98	0,93	1,00	1,01	1,00	1,04 hypothetical protein
254702_at	At4g17940	1,03	0,87	0,85	1,02	1,09	0,90	0,82	0,88 expressed protein
254706_at	At4g17950	0,77	0,80	1,21	1,08	1,05	0,90	0,95	1,21 DNA-binding family protein
254703_at	At4g17960	0,98	0,79	0,96	0,87	1,13	0,92	1,03	0,94 expressed protein
254697_at	At4g17970	0,85	1,25	2,43	0,75	1,04	1,13	1,89	2,77 expressed protein
254698_at	At4g17980	1,05	1,02	0,90	0,99	1,28	1,07	0,90	0,91 no apical meristem (NAM) family protein
254699_at	At4g17990	1,02	1,05	0,96	0,93	1,18	1,07	1,09	1,09 hypothetical protein
254700_at	At4g18000	0,92	1,08	0,96	1,06	1,04	1,00	0,93	0,81 hypothetical protein
254707_at	At4g18010	0,67	1,43	1,52	0,92	0,72	1,13	2,11	1,94 inositol polyphosphate 5-phosphatase II (IP5PII)
254704_at	At4g18020	0,98	1,09	1,16	0,96	0,79	1,00	1,03	1,20 pseudo-response regulator 2 (APRR2) (TOC2)
254701_at	At4g18030	0,85	0,72	0,78	1,23	1,25	0,83	0,91	1,00 dehydration-responsive family protein
254654_at	At4g18040	1,17	0,94	0,86	1,14	0,93	1,02	0,96	0,95 eukaryotic translation initiation factor 4E 1 / eIF-4E1 / mRNA cap-binding pr
254710_at	At4g18050	0,86	0,88	1,00	0,90	0,99	0,78	1,25	1,42 ABC transporter family protein
254711_at	At4g18060	0,95	0,85	1,13	0,96	1,05	0,95	0,98	1,06 SH3 domain-containing protein 3 (SH3P3)

254656_at	At4g18070	0,64	0,63	0,86	1,12	1,02	0,96	1,05	1,09 expressed protein
254712_at	At4g18080	0,85	1,03	0,98	0,96	1,17	0,94	1,00	0,92 hypothetical protein
254713_at	At4g18090	0,96	0,91	1,07	0,90	1,10	0,99	1,11	0,96 hypothetical protein
254655_s_at	At4g18100	1,41	1,12	1,10	1,13	1,11	0,96	1,00	0,95 60S ribosomal protein L32 (RPL32A)
254679_at	At4g18120	0,86	0,81	0,97	0,92	0,82	1,07	1,20	1,11 RNA recognition motif (RRM)-containing protein
254680_at	At4g18130	0,62	0,55	0,68	0,77	0,73	1,02	0,97	0,88 phytochrome E (PHYE)
254681_at	At4g18140	1,09	0,87	0,96	1,20	0,74	1,04	1,05	1,58 NLI interacting factor (NIF) family protein
254651_at	At4g18160	0,91	0,98	1,02	1,05	1,03	0,92	1,09	0,92 outward rectifying potassium channel, putative (KCO6)
254652_at	At4g18170	1,08	1,13	1,02	1,24	1,72	1,04	1,21	1,23 WRKY family transcription factor
254653_at	At4g18180	0,95	1,14	1,12	1,11	1,14	0,99	1,01	0,94 glycoside hydrolase family 28 protein / polygalacturonase (pectinase) family
254657_s_at	At4g18210	0,74	1,11	1,09	0,89	0,91	1,07	1,49	1,23 purine permease family protein
254658_at	At4g18230	0,86	0,88	0,92	0,83	0,53	1,01	0,98	1,07 expressed protein
254659_at	At4g18240	0,78	0,71	0,84	0,75	0,79	1,19	1,20	1,25 starch synthase-related protein
254660_at	At4g18250	0,99	1,06	1,02	1,06	1,09	1,13	0,98	1,05 receptor serine/threonine kinase, putative
254661_at	At4g18260	1,06	1,02	1,09	1,00	1,03	1,14	1,12	0,74 cytochrome B561-related
254662_at	At4g18270	0,77	0,88	0,75	0,72	0,82	1,04	1,08	1,08 glycosyl transferase family 4 protein
254667_at	At4g18280	0,86	0,88	0,83	1,31	1,43	1,05	1,09	0,95 glycine-rich cell wall protein-related
254663_at	At4g18290	1,04	0,92	0,96	0,89	0,84	0,88	0,90	0,82 inward rectifying potassium channel (KAT2)
254664_at	At4g18300	1,05	0,91	1,11	0,91	1,09	1,02	0,97	1,15 eIF4-gamma/eIF5/eIF2-epsilon domain-containing protein
254666_at	At4g18330	1,17	1,33	1,32	1,00	0,90	1,00	1,07	0,80 eukaryotic translation initiation factor 2 subunit 3, putative / eIF2S3, putative
254665_at	At4g18340	1,17	1,19	0,99	0,87	0,82	0,77	1,33	1,28 glycosyl hydrolase family 17 protein
254668_at	At4g18350	1,02	0,87	1,03	0,92	0,92	0,99	0,90	1,32 9-cis-epoxycarotenoid dioxygenase, putative / neoxanthin cleavage enzyme
254630_at	At4g18360	0,96	1,11	1,03	1,02	0,85	1,50	1,69	1,20 (S)-2-hydroxy-acid oxidase, peroxisomal, putative / glycolate oxidase, putat
254669_at	At4g18370	1,03	1,02	0,96	0,77	0,77	1,17	1,12	1,03 protease HhoA, chloroplast (SPPA) (HHOA)
254622_at	At4g18375	1,10	1,07	1,11	1,09	1,03	1,01	0,89	0,91 KH domain-containing protein
254670_at	At4g18390	0,67	0,80	0,73	0,96	0,69	0,92	0,75	0,83 TCP family transcription factor, putative
254627_at	At4g18395	0,96	1,08	0,99	0,91	0,91	1,06	0,94	0,89 expressed protein
254626_at	At4g18400	0,84	0,85	0,79	0,90	0,87	0,94	0,87	1,00 expressed protein
254671_at	At4g18410	1,06	1,13	0,91	0,88	1,16	1,13	1,02	1,03 ---
254672_at	At4g18420	1,17	1,19	1,00	0,91	1,02	0,98	0,91	1,15 hypothetical protein
254629_at	At4g18425	1,54	1,28	0,99	1,69	2,02	1,25	1,11	1,09 expressed protein
254673_at	At4g18430	1,04	1,02	1,10	1,11	1,10	1,04	1,09	1,30 Ras-related GTP-binding protein, putative
256461_s_at	At4g18440	1,15	0,96	0,87	0,99	0,77	0,95	0,88	0,64 adenylosuccinate lyase, putative / adenylosuccinase, putative
254674_at	At4g18450	0,93	0,98	0,99	1,06	1,33	1,09	0,94	0,96 ethylene-responsive factor, putative
254676_at	At4g18460	0,86	0,81	0,85	1,02	1,06	0,78	1,06	0,85 D-Tyr-tRNA(Tyr) deacylase family protein
254675_at	At4g18465	0,99	0,99	1,18	0,95	0,94	0,95	1,08	0,89 RNA helicase, putative
254625_at	At4g18470	1,15	1,08	1,06	0,99	0,96	1,13	1,07	0,99 negative regulator of systemic acquired resistance (SNI1)
254623_at	At4g18480	1,23	1,02	1,09	0,84	0,82	1,16	1,07	1,01 magnesium-chelatase subunit chlI, chloroplast / Mg-protoporphyrin IX chela
254677_at	At4g18490	0,91	1,07	0,95	0,97	0,97	0,85	1,17	0,82 hypothetical protein
254678_at	At4g18500	0,99	0,98	1,11	1,02	1,07	0,97	0,96	0,95 hypothetical protein
254644_at	At4g18510	0,93	0,76	0,77	1,35	1,63	0,88	1,07	0,98 Clavata3 / ESR-Related-2 (CLE2)
254645_at	At4g18520	0,84	0,68	0,88	0,76	0,59	0,78	0,73	0,71 pentatricopeptide (PPR) repeat-containing protein
254646_at	At4g18530	0,64	0,61	0,63	0,88	0,74	0,84	1,11	1,17 expressed protein
254647_at	At4g18540	0,99	1,00	1,11	1,07	1,10	0,90	1,00	1,36 expressed protein
254648_at	At4g18550	1,19	1,27	1,19	1,11	1,10	0,94	1,00	1,11 lipase class 3 family protein
254650_at	At4g18560	0,92	1,06	0,97	0,86	1,14	0,92	0,89	1,05 ---

254649_at	At4g18570	1,11	1,12	1,16	0,85	0,79	0,96	0,97	0,86	proline-rich family protein
254624_at	At4g18580	0,86	0,95	0,88	1,06	1,17	0,97	0,93	1,05	expressed protein
254620_at	At4g18590	1,19	1,09	0,98	1,15	1,11	0,91	0,94	0,81	expressed protein
254628_at	At4g18593	1,38	1,24	1,54	0,86	1,03	1,05	1,11	1,34	dual specificity protein phosphatase-related
254621_at	At4g18600	0,75	0,69	0,80	0,84	0,88	0,88	0,82	0,85	expressed protein
254631_at	At4g18610	0,94	0,93	0,83	1,14	1,14	1,04	0,99	1,61	expressed protein
254632_at	At4g18630	1,04	1,03	0,95	1,09	1,16	1,09	0,86	0,78	expressed protein
254633_at	At4g18640	1,11	0,88	0,88	0,99	0,97	0,92	1,25	1,04	leucine-rich repeat transmembrane protein kinase, putative
254634_at	At4g18650	0,92	0,99	0,94	1,20	0,93	1,02	0,94	0,93	transcription factor-related
254635_at	At4g18670	1,03	0,94	1,02	0,95	1,09	1,05	0,93	0,87	leucine-rich repeat family protein / extensin family protein
254636_at	At4g18700	0,73	0,62	0,74	1,17	1,14	0,97	0,93	0,99	CBL-interacting protein kinase 12 (CIPK12)
254616_at	At4g18710	1,08	1,25	1,45	0,99	1,12	1,07	0,98	1,29	shaggy-related protein kinase eta / ASK-eta (ASK7)
254637_at	At4g18720	0,97	1,12	1,01	0,99	0,99	1,02	1,06	0,93	transcription elongation factor-related
254617_s_at	At4g18730	1,19	1,10	1,10	1,00	0,98	0,96	0,97	0,95	60S ribosomal protein L11 (RPL11C)
254638_at	At4g18740	1,05	0,80	1,23	0,82	0,74	0,92	1,08	1,16	expressed protein
254639_at	At4g18750	1,00	0,79	0,95	0,80	0,85	1,15	1,09	1,00	pentatricopeptide (PPR) repeat-containing protein
254619_at	At4g18770	0,96	0,96	1,09	0,89	1,09	1,02	1,11	0,88	myb family transcription factor (MYB98)
254618_at	At4g18780	1,14	1,51	1,13	1,22	0,89	1,12	1,07	1,02	cellulose synthase, catalytic subunit (IRX1)
254640_at	At4g18790	0,91	1,09	1,02	1,02	1,13	1,11	1,08	1,03	NRAMP metal ion transporter 5, putative (NRAMP5)
254641_at	At4g18800	0,96	0,83	0,74	1,60	1,87	0,84	0,82	0,87	Ras-related GTP-binding family protein
254642_at	At4g18810	0,89	0,90	0,82	0,82	0,78	1,39	1,33	1,33	expressed protein
254643_at	At4g18820	0,83	0,79	1,05	0,79	0,86	0,96	1,09	1,23	expressed protein
254588_at	At4g18840	1,03	1,05	1,08	0,78	0,98	1,00	1,15	0,93	pentatricopeptide (PPR) repeat-containing protein
254589_at	At4g18850	1,02	1,06	0,91	1,06	1,08	0,96	0,90	0,99	hypothetical protein
254590_at	At4g18860	0,89	1,06	0,91	0,99	0,99	1,04	1,04	1,02	expressed protein
254591_at	At4g18870	0,99	1,06	0,95	0,98	1,09	1,12	0,96	1,23	heat shock transcription factor family protein
254592_at	At4g18880	0,73	1,20	0,93	0,71	0,66	1,29	1,67	1,41	heat shock transcription factor 21 (HSF21)
254610_at	At4g18890	0,79	0,68	0,59	0,99	0,79	1,03	1,18	1,40	brassinosteroid signalling positive regulator-related
254593_s_at	At4g18900	1,05	0,89	0,93	0,87	0,86	1,00	0,86	0,94	transducin family protein / WD-40 repeat family protein
254608_at	At4g18910	0,81	0,72	0,59	1,23	0,92	0,84	0,74	0,68	aquaglyceroporin / NOD26-like major intrinsic protein 2 (NLM2)
254607_at	At4g18920	0,76	0,93	0,91	1,06	1,17	1,01	0,95	1,02	expressed protein
254594_at	At4g18930	0,86	0,80	0,82	1,38	1,46	0,87	0,85	0,82	cyclic phosphodiesterase
254605_at	At4g18950	0,80	1,22	1,87	1,16	1,15	1,23	1,79	1,96	ankyrin protein kinase, putative
254595_at	At4g18960	1,01	1,01	0,96	0,95	0,98	0,96	0,97	0,91	floral homeotic protein AGAMOUS (AG)
254609_at	At4g18970	1,53	1,21	1,10	0,95	0,84	0,99	0,89	0,90	GDSL-motif lipase/hydrolase family protein
254596_at	At4g18975	1,04	1,04	0,92	1,01	1,00	1,16	0,96	0,88	pentatricopeptide (PPR) repeat-containing protein
254597_at	At4g18980	0,99	0,96	1,05	1,01	1,16	0,98	0,92	0,89	expressed protein
254598_at	At4g18990	0,90	0,92	1,09	1,03	1,01	1,13	0,98	0,90	xyloglucan:xyloglucosyl transferase, putative / xyloglucan endotransglycosy
254599_at	At4g19000	1,01	1,16	1,04	0,94	0,99	1,15	1,03	0,97	IWS1 C-terminus family protein
254600_at	At4g19010	0,90	0,92	0,95	0,80	0,83	0,88	0,92	1,12	4-coumarate--CoA ligase family protein / 4-coumaroyl-CoA synthase family
254601_at	At4g19020	1,01	0,85	0,89	1,25	1,17	0,92	0,98	0,93	chromomethylase 2 (CMT2)
254606_at	At4g19030	1,12	1,29	0,95	1,24	0,89	0,96	1,09	1,09	major intrinsic family protein / MIP family protein
254602_at	At4g19040	1,03	0,89	0,94	0,98	0,97	1,02	0,98	0,95	pleckstrin homology (PH) domain-containing protein / lipid-binding START d
254603_at	At4g19050	0,99	0,86	0,80	1,05	1,17	0,99	0,86	0,92	mob1/phocein family protein
254604_at	At4g19070	1,16	1,10	1,22	1,02	0,90	1,08	1,14	1,17	cadmium-responsive protein / cadmium induced protein (AS8)
254611_at	At4g19095	1,01	0,99	1,14	0,93	1,09	1,04	0,91	0,84	expressed protein

254612_at	At4g19100	1,15	1,04	1,93	0,89	0,82	1,09	1,03	1,02	expressed protein
254560_at	At4g19110	1,30	1,01	0,89	0,88	1,03	1,03	1,06	1,00	protein kinase, putative
254563_at	At4g19120	0,82	0,78	0,83	0,77	1,19	0,81	0,79	0,78	early-responsive to dehydration stress protein (ERD3)
254565_at	At4g19130	1,03	0,96	1,00	1,04	1,15	1,00	0,97	0,94	replication protein-related
254557_at	At4g19140	0,75	0,71	0,86	0,89	0,89	0,97	1,00	1,16	expressed protein
254613_at	At4g19150	1,05	1,06	1,05	1,09	1,14	1,21	1,16	1,25	ankyrin repeat family protein
254561_at	At4g19160	0,84	0,74	0,70	0,88	0,94	0,74	0,83	0,96	expressed protein
254564_at	At4g19170	0,95	0,96	1,06	0,61	0,41	1,51	1,96	1,70	9-cis-epoxycarotenoid dioxygenase, putative / neoxanthin cleavage enzyme
254558_at	At4g19185	1,02	0,94	0,79	1,04	0,98	0,87	0,88	0,98	integral membrane family protein
254614_at	At4g19190	1,03	0,98	0,96	0,71	1,26	1,03	0,95	1,09	zinc knuckle (CCHC-type) family protein
254559_at	At4g19200	0,82	0,70	0,82	1,04	1,04	0,85	0,98	1,08	proline-rich family protein
254615_at	At4g19210	0,99	0,79	0,81	0,91	0,91	0,82	0,88	0,90	RNase L inhibitor protein, putative
254556_at	At4g19220	1,00	1,02	0,87	0,80	1,24	1,01	1,09	1,02	pentatricopeptide (PPR) repeat-containing protein
254562_at	At4g19230	0,70	1,32	0,94	0,72	0,77	0,92	1,09	0,97	cytochrome P450 family protein
254566_at	At4g19240	1,00	1,10	0,89	1,09	1,11	0,97	1,11	0,89	expressed protein
254567_s_at	At4g19260	1,07	1,04	1,06	1,00	0,98	0,98	1,01	0,87	kelch repeat-containing protein
254568_x_at	At4g19300	1,01	0,97	0,95	0,91	1,12	0,91	0,97	0,89	hypothetical protein
254569_s_at	At4g19320	1,07	0,98	0,97	1,01	0,93	1,08	0,91	0,89	hypothetical protein
254570_s_at	At4g19330	0,94	1,04	0,94	1,11	1,01	1,18	1,07	1,09	kelch repeat-containing protein
254576_at	At4g19350	1,30	1,16	1,02	1,06	1,06	1,06	1,05	0,92	expressed protein
254571_at	At4g19370	1,15	1,24	1,31	0,92	0,84	0,97	0,89	0,96	hypothetical protein
254572_at	At4g19380	2,28	1,59	1,15	0,91	0,97	1,20	1,00	1,03	alcohol oxidase-related
254580_at	At4g19390	0,67	0,62	0,71	0,81	0,68	0,97	0,95	0,74	expressed protein
254579_at	At4g19400	0,79	0,68	0,91	1,04	1,20	1,16	1,17	1,15	expressed protein
254578_at	At4g19410	0,78	0,81	0,75	0,86	0,82	0,88	0,87	0,88	pectinacetylesterase, putative
254573_at	At4g19420	1,33	1,23	1,37	1,29	1,15	0,89	1,21	1,35	pectinacetylesterase family protein
254574_at	At4g19430	1,02	0,92	0,85	1,23	1,11	0,97	1,00	1,03	expressed protein
254581_at	At4g19440	0,91	0,96	1,06	1,07	1,05	0,93	1,01	1,09	pentatricopeptide (PPR) repeat-containing protein
254577_at	At4g19450	0,93	0,88	2,00	0,94	1,17	1,00	1,18	1,24	nodulin-related
254575_at	At4g19460	0,92	0,95	0,93	1,16	1,14	0,99	0,99	0,83	glycosyl transferase family 1 protein
254582_at	At4g19470	0,99	0,87	0,99	0,84	0,91	1,03	1,04	0,99	disease resistance protein-related
254583_at	At4g19480	0,95	1,04	1,02	1,11	1,05	1,01	0,97	0,89	hypothetical protein
254584_at	At4g19490	0,90	0,98	0,84	0,83	1,07	0,96	0,93	0,84	expressed protein
254585_at	At4g19500	0,95	1,07	1,01	0,69	0,72	1,30	1,15	1,32	disease resistance protein (TIR-NBS-LRR class), putative
254586_at	At4g19510	0,97	0,91	0,90	0,71	0,62	1,15	1,31	1,24	disease resistance protein (TIR-NBS-LRR class), putative
254587_at	At4g19520	1,02	0,86	0,98	0,88	0,92	1,32	1,61	1,63	disease resistance protein (TIR-NBS-LRR class), putative
254553_at	At4g19530	0,82	0,80	1,34	0,59	0,46	1,05	1,12	1,32	disease resistance protein (TIR-NBS-LRR class), putative
254529_at	At4g19540	0,94	0,67	0,75	0,83	0,73	1,00	0,89	0,82	expressed protein
254554_at	At4g19550	1,06	0,95	0,99	1,00	0,96	1,24	1,07	0,92	expressed protein
254555_at	At4g19570	0,99	0,92	1,12	1,01	1,03	1,01	0,96	1,08	DNAJ heat shock N-terminal domain-containing protein
254525_at	At4g19580	0,89	1,04	0,98	1,07	1,10	0,87	0,95	0,83	DNAJ heat shock N-terminal domain-containing protein
254526_at	At4g19600	0,89	0,65	0,68	1,02	1,11	0,96	0,98	0,93	cyclin family protein
254527_at	At4g19610	1,17	1,18	2,03	0,77	0,74	1,07	1,24	1,56	RNA recognition motif (RRM)-containing protein
254528_at	At4g19620	0,99	1,02	1,05	1,09	1,02	0,98	1,02	1,06	hypothetical protein
254530_at	At4g19640	0,90	0,88	0,95	1,08	1,07	1,09	1,04	1,15	Ras-related GTP-binding protein, putative
254531_at	At4g19650	1,02	1,04	1,07	0,95	0,97	0,96	0,99	1,28	mitochondrial transcription termination factor-related / mTERF-related

254532_at	At4g19660	0,86	1,20	0,99	0,69	0,91	1,26	1,27	1,07 ankyrin repeat family protein / BTB/POZ domain-containing protein
254533_at	At4g19670	1,13	1,42	1,34	0,86	1,23	1,01	0,95	1,18 zinc finger (C3HC4-type RING finger) family protein
254534_at	At4g19680	1,10	1,20	0,99	1,11	1,15	1,01	0,95	0,99 iron-responsive transporter (IRT2)
254550_at	At4g19690	2,69	5,08	2,60	3,19	1,59	4,88	3,03	3,32 iron-responsive transporter (IRT1)
254535_at	At4g19710	1,20	0,94	0,96	0,67	0,70	1,13	1,13	1,02 bifunctional aspartate kinase/homoserine dehydrogenase, putative / AK-HS
254536_at	At4g19720	0,99	1,11	1,18	0,98	0,96	0,91	1,02	0,92 glycosyl hydrolase family 18 protein
254537_at	At4g19730	0,92	1,02	1,07	0,96	0,94	1,12	0,97	1,08 glycosyl hydrolase family 18 protein
254538_at	At4g19740	0,99	0,99	1,09	1,07	0,95	1,06	1,07	1,02 glycosyl hydrolase family 18 protein
254539_s_at	At4g19750	1,02	1,05	0,90	0,99	0,88	0,96	0,94	0,94 glycosyl hydrolase family 18 protein
254540_s_at	At4g19770	0,96	0,86	0,96	1,11	1,06	0,98	0,91	1,01 glycosyl hydrolase family 18 protein
254541_at	At4g19780	1,01	0,95	0,96	1,04	1,04	1,05	1,02	1,13 ---
254542_s_at	At4g19790	0,96	1,05	0,93	0,92	1,06	1,02	0,97	1,07 ---
254543_at	At4g19810	0,24	0,20	0,13	0,52	0,48	1,15	1,05	1,01 glycosyl hydrolase family 18 protein
254544_at	At4g19820	1,05	1,03	0,92	1,35	1,12	1,08	0,89	0,96 glycosyl hydrolase family 18 protein
254545_at	At4g19830	0,96	1,01	0,91	1,29	1,16	1,08	0,96	0,96 immunophilin / FKBP-type peptidyl-prolyl cis-trans isomerase family protein
254551_at	At4g19840	1,10	0,99	0,94	1,09	0,99	0,94	1,00	0,98 lectin-related
254546_at	At4g19850	1,21	1,02	0,94	1,01	0,97	0,94	1,05	1,03 lectin-related
254547_at	At4g19860	1,23	1,65	1,56	0,79	0,97	1,07	1,12	1,69 lecithin:cholesterol acyltransferase family protein / LACT family protein
254548_at	At4g19870	0,87	1,12	1,39	0,95	1,01	0,98	0,92	0,93 kelch repeat-containing F-box family protein
254549_at	At4g19880	0,70	0,84	1,01	0,75	0,88	0,90	0,98	0,94 glutathione S-transferase-related
254552_at	At4g19900	0,83	0,95	1,01	0,88	0,90	1,02	0,96	0,94 glycosyl transferase-related
254518_at	At4g19920	0,86	1,05	1,04	1,15	1,02	0,91	0,86	0,94 Toll-Interleukin-Resistance (TIR) domain-containing protein
254519_at	At4g19940	0,99	0,96	0,91	1,02	1,12	0,89	0,96	0,96 F-box family protein
254520_at	At4g19960	0,76	0,68	0,98	0,82	0,75	1,29	1,52	1,79 potassium transporter family protein
254522_at	At4g19980	0,92	1,02	0,94	1,32	1,53	1,08	1,09	0,88 expressed protein
254505_at	At4g19985	1,08	0,96	0,90	1,08	1,07	1,01	0,94	0,85 GCN5-related N-acetyltransferase (GNAT) family protein
254523_at	At4g19990	0,88	0,91	0,88	0,96	1,05	0,96	1,11	1,04 far-red impaired responsive family protein / FAR1 family protein
254524_at	At4g20000	0,78	1,11	0,93	1,35	1,38	1,07	1,16	1,27 VQ motif-containing protein
254503_at	At4g20010	1,04	0,92	0,98	0,75	0,82	0,98	0,96	0,92 expressed protein
254493_at	At4g20020	1,33	1,26	1,41	1,05	1,12	1,02	0,86	0,92 expressed protein
254504_at	At4g20030	1,18	1,02	1,08	1,12	0,99	0,86	0,91	0,81 RNA recognition motif (RRM)-containing protein
254494_at	At4g20050	1,01	1,03	0,95	0,86	1,00	0,96	0,93	0,89 expressed protein
254495_at	At4g20060	1,25	1,08	1,02	0,93	0,99	1,06	1,21	0,95 expressed protein
254496_at	At4g20070	0,91	0,83	0,80	0,91	0,95	0,92	0,92	0,92 peptidase M20/M25/M40 family protein
254497_at	At4g20080	0,93	1,05	0,94	0,99	1,04	1,10	0,99	1,01 C2 domain-containing protein
254498_at	At4g20090	0,93	1,06	0,96	0,81	0,89	0,86	0,96	1,06 pentatricopeptide (PPR) repeat-containing protein
254499_at	At4g20100	1,09	1,00	0,90	0,90	0,99	1,07	0,91	1,01 PQ-loop repeat family protein / transmembrane family protein
254500_at	At4g20110	0,81	1,14	0,99	1,15	0,99	1,26	1,50	1,53 vacuolar sorting receptor, putative
254501_at	At4g20120	0,88	0,81	0,81	0,72	0,73	1,13	1,03	1,07 ribulose-1,5 bisphosphate carboxylase/oxygenase large subunit N-methyltra
254502_at	At4g20130	0,97	0,79	0,85	0,67	0,73	1,05	1,03	1,06 ribulose-1,5 bisphosphate carboxylase/oxygenase large subunit N-methyltra
254506_at	At4g20140	0,69	1,02	1,00	0,71	0,62	1,27	1,13	0,98 leucine-rich repeat transmembrane protein kinase, putative
254462_at	At4g20150	1,27	1,21	1,14	1,34	1,37	1,01	0,95	1,04 expressed protein
254507_at	At4g20160	0,92	0,92	0,98	1,00	1,01	0,96	1,02	0,98 expressed protein
254508_at	At4g20170	0,75	0,71	0,80	0,97	0,82	1,03	1,26	1,33 expressed protein
254509_at	At4g20200	0,99	0,96	1,04	1,04	1,07	0,99	0,98	0,97 terpene synthase/cyclase family protein
254510_at	At4g20210	0,93	0,96	0,89	1,23	0,99	0,97	1,10	1,03 terpene synthase/cyclase family protein

254511_at	At4g20220	0,92	1,04	1,15	0,97	1,07	0,90	1,04	1,01 hypothetical protein
254512_at	At4g20230	0,91	0,77	0,90	1,08	1,16	0,90	1,00	1,06 terpene synthase/cyclase family protein
254513_at	At4g20240	0,91	1,08	1,04	0,97	0,94	0,92	1,03	1,02 cytochrome P450, putative
254514_at	At4g20250	0,89	1,00	0,97	0,97	1,11	0,92	1,06	0,95 hypothetical protein
254492_at	At4g20260	0,88	0,84	0,88	0,96	1,07	0,98	0,96	1,03 DREPP plasma membrane polypeptide family protein
254515_at	At4g20270	0,99	1,21	0,94	0,72	0,91	0,92	1,05	1,18 leucine-rich repeat transmembrane protein kinase, putative
254463_at	At4g20280	1,52	1,38	1,46	1,01	0,95	1,28	1,00	1,08 transcription initiation factor IID (TFIID) 28 kDa subunit (TAFII-28) family pro
254516_at	At4g20290	0,95	1,04	1,11	1,02	0,99	0,92	1,04	0,90 expressed protein
254491_at	At4g20300	0,99	0,74	0,85	1,01	1,25	0,99	0,94	1,05 expressed protein
254517_at	At4g20310	0,98	1,44	1,10	0,77	0,96	0,95	0,99	1,00 peptidase M50 family protein / sterol-regulatory element binding protein (SF
254490_at	At4g20320	2,03	1,46	1,52	1,16	1,71	1,40	2,13	1,84 CTP synthase, putative / UTP--ammonia ligase, putative
254478_at	At4g20330	1,07	0,90	0,96	1,03	1,05	1,08	0,97	0,98 transcription initiation factor-related
254479_at	At4g20350	0,96	1,13	1,06	0,95	1,01	1,01	0,97	1,10 expressed protein
254480_at	At4g20360	1,06	0,93	0,89	0,93	0,81	1,02	1,04	0,97 elongation factor Tu / EF-Tu (TUFA)
254482_at	At4g20370	0,91	1,13	0,91	0,91	0,90	1,03	0,96	1,16 twin sister of FT protein (TSF) / TFL1 like protein
254477_at	At4g20380	0,87	1,20	1,44	1,10	1,22	1,16	1,24	1,55 zinc finger protein (LSD1)
254474_at	At4g20390	0,93	0,91	0,87	0,91	0,93	0,95	1,04	1,26 integral membrane family protein
254464_at	At4g20400	1,01	1,04	0,87	0,97	0,94	0,93	1,00	1,00 transcription factor jumonji (jmj) family protein / zinc finger (C5HC2 type) far
254476_at	At4g20410	0,95	0,91	1,02	1,08	1,12	0,88	0,89	1,00 gamma-soluble NSF attachment protein / gamma-SNAP
254465_at	At4g20420	1,04	0,94	1,02	1,12	0,96	0,98	0,91	0,90 tapetum-specific protein-related
254466_at	At4g20430	1,35	1,09	1,00	0,86	1,19	0,80	0,87	1,01 subtilase family protein
254475_at	At4g20440	1,11	1,04	1,02	0,97	0,97	1,02	0,96	1,03 small nuclear ribonucleoprotein associated protein B, putative / snRNP-B, p
254467_at	At4g20450	0,92	1,06	0,98	1,08	0,98	1,04	1,03	1,03 leucine-rich repeat protein kinase, putative
254468_at	At4g20460	0,92	0,92	0,72	1,01	0,98	1,40	1,25	0,94 NAD-dependent epimerase/dehydratase family protein
254469_at	At4g20470	0,86	1,15	0,95	0,98	1,07	1,05	1,03	1,00 hypothetical protein
254481_at	At4g20480	1,01	1,09	1,09	0,92	0,93	1,35	1,60	2,14 expressed protein
254470_at	At4g20490	1,19	0,98	1,06	0,91	1,06	0,93	0,98	1,03 ---
254471_s_at	At4g20500	1,02	1,02	1,08	0,93	1,16	1,07	0,96	0,98 expressed protein
254472_at	At4g20510	1,01	1,06	0,96	1,01	1,06	0,94	0,98	1,05 expressed protein
254473_s_at	At4g20530	0,95	1,02	1,04	0,95	1,03	0,94	0,89	0,95 hypothetical protein
254483_at	At4g20740	0,75	0,90	0,94	0,84	0,97	0,87	0,92	0,62 pentatricopeptide (PPR) repeat-containing protein
254484_at	At4g20750	1,18	1,06	1,10	0,93	1,10	1,09	1,02	0,94 short-chain dehydrogenase/reductase (SDR) family protein
254485_at	At4g20760	1,16	1,25	1,17	1,05	0,97	1,13	1,09	1,10 short-chain dehydrogenase/reductase (SDR) family protein
254486_at	At4g20770	1,03	0,99	0,84	0,92	1,04	0,94	1,11	1,06 pentatricopeptide (PPR) repeat-containing protein
254487_at	At4g20780	0,77	0,65	0,66	0,76	1,04	0,86	0,89	0,90 calcium-binding protein, putative
254488_at	At4g20790	0,98	0,98	0,97	1,03	0,98	1,04	1,01	1,06 leucine-rich repeat family protein
254489_at	At4g20800	0,97	1,08	0,89	1,03	1,10	0,97	0,97	0,94 FAD-binding domain-containing protein
254430_at	At4g20820	0,97	0,99	1,06	1,18	0,96	1,03	0,87	0,94 FAD-binding domain-containing protein
254432_at	At4g20830	1,10	0,98	0,90	0,98	0,83	1,07	0,96	0,85 FAD-binding domain-containing protein
254431_at	At4g20840	0,88	0,84	0,82	0,63	0,85	1,15	0,94	0,88 FAD-binding domain-containing protein
254433_at	At4g20850	0,84	0,75	0,78	0,87	0,75	1,05	1,10	1,21 subtilase family protein
254447_at	At4g20860	0,62	0,65	0,71	0,80	1,03	1,15	0,89	0,94 FAD-binding domain-containing protein
254448_at	At4g20870	0,93	1,05	0,89	1,38	1,25	0,96	0,86	0,95 fatty acid hydroxylase, putative
254434_at	At4g20880	0,74	0,72	0,74	0,90	0,95	0,86	0,89	0,77 ethylene-responsive nuclear protein / ethylene-regulated nuclear protein (E
254446_at	At4g20890	0,79	0,68	0,68	0,87	0,98	0,89	0,88	0,85 tubulin beta-9 chain (TUB9)
254435_at	At4g20900	1,04	0,90	1,10	1,00	1,14	0,94	1,05	0,95 male sterility MS5 / pollenless 3

254449_at	At4g20910	1,21	1,06	1,15	0,94	1,12	1,12	0,92	0,84	double-stranded RNA binding protein-related / DsRBD protein-related
254445_at	At4g20930	0,55	0,64	0,66	0,78	0,86	0,89	0,90	0,96	3-hydroxyisobutyrate dehydrogenase, putative
254436_at	At4g20940	0,98	0,85	1,02	1,04	0,93	1,06	1,01	1,05	leucine-rich repeat family protein
254437_s_at	At4g20950	0,98	0,77	1,06	0,93	0,64	0,97	0,93	0,86	---
254444_at	At4g20960	0,63	0,57	0,67	0,88	0,90	0,86	0,80	0,67	cytidine/deoxycytidylate deaminase family protein
254438_at	At4g20980	1,27	0,95	0,88	1,13	0,81	1,08	0,94	0,81	eukaryotic translation initiation factor 3 subunit 7, putative / eIF-3 zeta, puta
254439_s_at	At4g20990	0,91	0,86	1,23	1,08	1,09	0,97	0,96	1,48	carbonic anhydrase family protein
254440_at	At4g21020	0,52	0,59	0,68	0,64	0,71	1,02	1,07	0,95	late embryogenesis abundant domain-containing protein / LEA domain-cont
254441_at	At4g21050	1,05	0,88	1,06	0,97	0,96	0,99	1,02	1,04	Dof-type zinc finger domain-containing protein
254442_at	At4g21060	1,13	1,14	1,18	1,20	1,08	0,97	1,20	1,21	galactosyltransferase family protein
254443_at	At4g21070	1,58	1,26	1,49	1,09	1,27	1,01	1,08	0,93	BRCT domain-containing protein / zinc finger (C3HC4-type RING finger) far
254450_at	At4g21080	1,00	1,08	1,08	1,12	1,07	0,93	0,93	0,97	Dof-type zinc finger domain-containing protein
254451_at	At4g21090	1,07	0,93	0,99	1,04	1,04	1,04	1,00	1,04	adrenodoxin-like ferredoxin 1
254452_at	At4g21100	1,47	1,13	1,08	0,83	0,88	0,97	0,98	0,82	UV-damaged DNA-binding protein, putative
254429_at	At4g21105	1,34	1,17	1,01	1,42	1,15	1,05	1,00	0,91	expressed protein
254399_at	At4g21110	1,33	1,18	1,28	1,09	1,00	1,08	1,07	1,19	G10 family protein
254453_at	At4g21120	1,08	0,98	0,79	1,09	0,92	0,97	1,08	0,92	amino acid permease family protein
254454_at	At4g21130	0,98	1,18	0,99	0,99	1,03	0,94	1,02	1,12	transducin family protein / WD-40 repeat family protein
254455_at	At4g21140	1,02	1,03	0,92	1,07	0,86	0,95	0,77	0,98	expressed protein
254456_at	At4g21150	0,89	0,89	0,82	1,13	0,93	0,91	0,86	0,77	ribophorin II (RPN2) family protein
254428_at	At4g21160	1,08	0,91	1,01	1,07	0,92	1,13	1,03	0,99	zinc finger and C2 domain protein (ZAC)
254457_at	At4g21170	1,05	0,83	0,77	0,84	0,93	0,90	1,01	0,82	pentatricopeptide (PPR) repeat-containing protein
254458_at	At4g21180	1,02	0,88	0,80	0,87	0,74	1,09	1,03	1,10	DNAJ heat shock N-terminal domain-containing protein / sec63 domain-cor
254427_at	At4g21190	1,15	1,17	1,19	0,64	0,56	1,23	1,25	1,36	pentatricopeptide (PPR) repeat-containing protein
254459_at	At4g21200	0,82	0,66	0,55	0,92	0,91	0,91	0,66	0,79	oxidoreductase, 2OG-Fe(II) oxygenase family protein
254460_at	At4g21210	1,06	0,89	0,92	0,83	1,00	1,18	1,02	1,04	expressed protein
254461_at	At4g21230	0,73	0,68	0,67	1,01	0,96	0,98	0,99	0,88	protein kinase family protein
254426_at	At4g21240	0,91	1,03	1,06	0,99	1,03	1,02	1,02	1,07	F-box family protein
254400_at	At4g21270	1,16	1,12	1,33	1,18	1,31	1,05	1,01	0,94	kinesin-like protein A (KATA)
254398_at	At4g21280	1,06	1,04	1,20	0,99	0,97	1,00	0,98	1,00	oxygen-evolving enhancer protein 3, chloroplast, putative (PSBQ1) (PSBQ)
254401_at	At4g21300	0,91	1,01	0,90	0,93	1,02	0,95	1,01	1,07	pentatricopeptide (PPR) repeat-containing protein
254402_at	At4g21310	1,23	1,40	1,40	1,14	1,58	0,84	0,98	1,04	expressed protein
254414_at	At4g21320	1,13	1,11	1,09	0,82	1,02	1,10	1,00	1,04	(2R)-phospho-3-sulfolactate synthase-related
254403_at	At4g21323	1,09	0,99	1,06	0,97	1,09	1,05	0,98	1,05	subtilase family protein
254415_at	At4g21326	0,94	0,82	0,78	1,08	1,00	0,78	0,86	0,72	subtilase family protein
254404_at	At4g21340	1,09	0,98	0,87	1,01	0,95	1,02	1,09	1,09	ethylene-responsive protein-related
254405_at	At4g21350	0,83	0,83	0,96	0,89	1,14	0,84	0,96	0,78	U-box domain-containing protein
254406_at	At4g21360	1,06	0,93	0,99	1,05	1,18	1,00	0,98	1,13	---
254407_at	At4g21370	1,01	0,97	1,05	0,88	1,17	1,14	0,92	0,83	S-locus protein kinase, putative
254416_at	At4g21380	1,12	0,93	0,99	0,91	0,82	1,16	1,02	1,24	S-locus protein kinase, putative (ARK3)
254408_at	At4g21390	0,88	0,74	0,96	1,24	1,16	1,05	0,99	1,37	S-locus lectin protein kinase family protein
254409_at	At4g21400	0,53	0,36	0,44	1,06	0,98	0,95	1,19	1,13	protein kinase family protein
254410_at	At4g21410	1,07	0,78	0,84	1,07	1,01	0,80	0,87	0,80	protein kinase family protein
254411_at	At4g21420	1,07	0,94	1,05	0,97	0,94	0,95	1,03	0,98	---
254412_at	At4g21430	0,89	0,94	1,16	0,92	1,01	0,94	0,92	0,82	transcription factor jumonji (jmjC) domain-containing protein
254413_at	At4g21440	0,98	0,93	0,92	0,93	1,05	1,06	1,10	0,90	myb family transcription factor (MYB102)

254425_at	At4g21450	1,04	1,06	1,05	1,03	1,06	0,95	1,02	1,07	vesicle-associated membrane family protein / VAMP family protein
254417_at	At4g21470	1,18	1,27	1,44	1,19	1,19	1,17	1,08	1,11	riboflavin kinase/FAD synthetase family protein
254418_at	At4g21480	0,79	0,89	1,00	1,13	0,99	0,78	0,80	0,86	glucose transporter, putative
254419_at	At4g21490	1,01	0,97	0,97	1,05	1,09	1,06	0,96	0,97	pyridine nucleotide-disulphide oxidoreductase family protein
254420_at	At4g21500	0,88	1,04	1,02	1,11	1,04	0,86	0,90	0,77	expressed protein
254424_at	At4g21510	1,54	1,32	0,94	0,62	0,84	0,99	1,10	1,18	F-box family protein
254421_at	At4g21550	1,02	0,99	1,07	0,95	0,91	0,89	1,05	0,75	transcriptional factor B3 family protein
254422_at	At4g21560	0,85	0,89	1,01	1,12	0,96	1,09	1,14	1,19	vacuolar protein sorting-associated protein 28 family protein / VPS28 family
254393_at	At4g21580	0,67	0,64	0,62	0,96	0,80	0,95	0,99	0,98	oxidoreductase, zinc-binding dehydrogenase family protein
254391_at	At4g21590	0,91	0,85	0,77	1,05	1,32	0,97	0,95	0,99	bifunctional nuclease, putative
254392_at	At4g21600	1,19	0,95	0,61	1,66	1,53	0,96	1,04	1,17	bifunctional nuclease, putative
254423_at	At4g21610	1,02	0,91	0,83	1,21	0,93	0,99	0,90	0,89	zinc finger protein, putative
254372_at	At4g21620	0,99	0,86	0,85	1,37	1,17	0,89	0,91	0,83	glycine-rich protein
254394_at	At4g21630	0,90	0,93	1,02	1,04	1,21	0,90	1,07	1,01	subtilase family protein
254395_at	At4g21640	1,01	1,05	0,99	0,98	1,10	0,89	1,08	0,98	subtilase family protein
254377_at	At4g21650	0,94	0,63	0,59	1,34	1,14	0,72	0,65	0,72	subtilase family protein
254396_at	At4g21680	0,89	0,91	0,49	1,05	0,80	1,18	1,38	1,22	proton-dependent oligopeptide transport (POT) family protein
254397_at	At4g21690	0,94	0,98	1,10	0,92	1,06	1,04	1,02	1,04	gibberellin 3 beta-hydroxylase family protein
254367_at	At4g21700	0,72	0,64	0,82	0,96	0,84	0,98	0,89	0,73	hypothetical protein
254368_at	At4g21710	1,02	0,87	0,92	0,97	0,91	0,93	0,99	1,19	DNA-directed RNA polymerase II 135 kDa polypeptide / RNA polymerase II
254369_at	At4g21720	1,03	1,01	0,89	0,90	0,94	1,03	0,82	0,89	expressed protein
254373_at	At4g21730	1,32	1,43	1,32	0,79	0,91	1,02	1,05	1,30	---
254370_at	At4g21750	0,93	1,02	1,12	0,77	0,74	0,90	0,82	0,95	L1 specific homeobox gene (ML1) / ovule-specific homeobox protein A20
254371_at	At4g21760	1,05	1,01	1,10	1,05	1,20	1,21	1,09	1,00	glycosyl hydrolase family 1 protein
254374_at	At4g21780	1,05	1,10	1,06	1,07	1,37	1,04	0,97	1,07	expressed protein
254376_at	At4g21790	0,76	0,68	0,65	1,17	0,98	0,87	0,93	0,98	transmembrane protein-related (TOM1)
254375_at	At4g21800	1,25	1,17	1,24	1,07	1,12	1,14	1,06	1,10	ATP-binding family protein
254378_at	At4g21810	1,04	1,32	1,35	0,94	0,87	1,03	1,14	0,93	Der1-like family protein / degradation in the ER-like family protein
254379_at	At4g21820	0,92	1,01	1,13	1,07	1,08	0,93	1,17	1,01	calmodulin-binding family protein
254385_s_at	At4g21830	1,00	1,00	0,92	1,62	1,37	1,07	1,36	1,36	methionine sulfoxide reductase domain-containing protein / SeIR domain-co
254387_at	At4g21850	1,19	1,10	1,02	1,29	1,19	0,90	0,93	0,90	methionine sulfoxide reductase domain-containing protein / SeIR domain-co
254388_at	At4g21860	0,93	0,90	0,79	1,06	1,00	1,00	0,90	0,95	methionine sulfoxide reductase domain-containing protein / SeIR domain-co
254380_at	At4g21865	0,98	0,76	0,90	1,17	1,02	0,90	0,93	0,96	expressed protein
254384_at	At4g21870	0,92	0,48	0,74	0,85	0,94	1,18	1,19	1,41	26.5 kDa class P-related heat shock protein (HSP26.5-P)
254381_at	At4g21880	0,89	1,02	1,12	1,04	1,09	0,95	0,89	0,75	pentatricopeptide (PPR) repeat-containing protein
254382_at	At4g21890	0,84	0,91	0,97	0,87	1,04	1,06	0,93	0,92	expressed protein
254389_s_at	At4g21910	0,60	0,56	0,46	0,92	0,89	0,93	1,00	1,03	MATE efflux family protein
254390_at	At4g21940	1,23	1,51	2,39	1,04	1,09	2,15	2,58	1,85	calcium-dependent protein kinase, putative / CDPK, putative
254383_at	At4g21950	0,94	1,02	0,98	0,88	1,08	1,02	1,03	1,05	hypothetical protein
254386_at	At4g21960	0,96	1,01	1,04	1,00	1,03	0,76	0,76	0,78	peroxidase 42 (PER42) (P42) (PRXR1)
254346_at	At4g21980	1,37	1,42	1,22	1,38	1,47	1,12	1,04	1,14	autophagy 8a (APG8a)
254343_at	At4g21990	0,98	1,09	0,78	0,42	0,59	0,93	1,04	1,43	5'-adenylylsulfate reductase (APR3) / PAPS reductase homolog (PRH26)
254345_at	At4g22000	1,49	1,13	1,25	0,86	0,95	1,00	1,13	1,17	expressed protein
254363_at	At4g22010	0,88	0,84	0,81	1,24	1,29	0,89	0,83	0,82	multi-copper oxidase type I family protein
254364_at	At4g22020	1,04	1,02	0,93	1,14	1,01	1,03	1,00	1,25	---
254365_at	At4g22030	1,05	1,09	1,07	1,03	1,10	0,86	1,01	1,20	F-box family protein

254366_s_at	At4g22040	0,96	1,10	1,08	1,06	1,02	0,98	0,95	1,06	---
254336_at	At4g22050	1,01	1,02	0,99	2,25	2,76	1,05	1,05	0,99	aspartyl protease family protein
254337_at	At4g22060	0,96	1,08	1,07	0,95	0,97	1,04	1,03	0,90	---
254347_at	At4g22070	0,96	0,97	0,94	1,11	0,97	1,06	0,98	0,98	WRKY family transcription factor
254338_s_at	At4g22080	1,04	0,97	0,95	1,13	1,11	0,94	1,03	0,86	pectate lyase family protein
254339_at	At4g22100	0,88	0,94	0,94	0,98	0,95	1,03	1,09	0,89	glycosyl hydrolase family 1 protein
254344_at	At4g22110	1,15	1,12	1,18	1,03	0,91	1,09	1,30	1,17	alcohol dehydrogenase, putative
254340_at	At4g22120	0,95	1,11	1,07	1,10	0,86	0,93	0,85	0,98	early-responsive to dehydration protein-related / ERD protein-related
254341_at	At4g22130	0,97	1,03	0,96	1,24	1,35	0,82	0,92	1,02	protein kinase family protein
254342_at	At4g22140	0,99	1,02	1,20	0,88	1,06	0,97	0,88	1,08	PHD finger family protein / bromo-adjacent homology (BAH) domain-contai
254348_at	At4g22150	0,86	0,82	0,78	1,22	1,23	0,91	0,90	0,97	UBX domain-containing protein
254362_at	At4g22160	0,98	0,98	0,83	0,97	1,18	0,85	0,77	0,85	expressed protein
254356_at	At4g22190	0,82	0,96	1,00	0,93	1,26	0,78	0,99	1,20	expressed protein
254305_at	At4g22200	1,31	1,23	1,03	0,63	0,87	1,00	1,22	0,94	potassium channel protein 2 (AKT2) (AKT3)
254361_at	At4g22212	1,18	0,91	0,70	1,65	1,02	0,96	1,45	1,31	expressed protein
254357_at	At4g22220	1,31	1,20	1,12	1,09	1,26	1,10	1,02	1,08	iron-sulfur cluster assembly complex protein, putative
254349_at	At4g22250	0,95	0,90	0,88	0,77	0,98	0,93	1,03	1,02	zinc finger (C3HC4-type RING finger) family protein
254335_at	At4g22260	0,75	0,70	0,62	0,91	0,89	0,92	0,98	0,96	alternative oxidase, putative / immutans protein (IM)
254304_at	At4g22270	0,81	0,75	0,76	1,08	1,03	1,11	1,08	1,19	expressed protein
254350_at	At4g22280	2,23	1,74	1,35	1,13	1,13	0,96	1,11	1,11	F-box family protein
254351_at	At4g22300	1,03	0,87	0,83	1,21	1,23	0,74	0,84	0,81	phospholipase/carboxylesterase family protein
254358_at	At4g22310	1,28	1,17	1,04	1,25	1,33	1,00	0,97	0,98	expressed protein
254352_at	At4g22320	0,96	0,82	1,03	1,11	1,40	0,96	0,98	0,91	expressed protein
254306_at	At4g22330	1,02	0,75	0,98	1,19	1,13	0,91	0,90	0,88	alkaline phytoceramidase family / aPHC family
254360_at	At4g22340	0,94	0,94	0,93	1,00	0,95	0,95	1,09	0,98	phosphatidate cytidylyltransferase, putative / CDP-diglyceride synthetase, p
254353_s_at	At4g22350	1,28	1,06	1,09	0,84	0,93	1,04	1,04	1,10	ubiquitin carboxyl-terminal hydrolase family protein
254359_at	At4g22360	0,78	1,18	1,32	1,35	1,51	0,98	1,22	1,12	SWIB complex BAF60b domain-containing protein
254354_at	At4g22370	1,06	1,09	1,10	1,04	0,93	0,98	1,03	0,94	---
254355_at	At4g22380	1,11	1,05	1,00	0,95	0,90	1,02	0,89	0,85	ribosomal protein L7Ae/L30e/S12e/Gadd45 family protein
254307_at	At4g22400	1,07	1,01	1,05	1,01	1,09	0,95	0,99	1,47	expressed protein
254308_at	At4g22410	1,05	1,13	0,86	1,04	0,90	1,14	0,93	1,11	ubiquitin carboxyl-terminal hydrolase family protein
254309_s_at	At4g22420	0,98	0,97	1,00	1,20	1,08	1,08	1,01	1,16	F-box family protein-related
254310_at	At4g22430	0,94	1,19	0,92	0,94	1,07	0,97	1,12	0,96	hypothetical protein
254311_at	At4g22440	0,99	0,94	1,04	1,05	1,03	1,03	0,97	0,81	hypothetical protein
254312_s_at	At4g22450	0,84	0,82	1,01	0,99	1,11	1,07	0,95	0,88	transposase IS4 family protein
254313_at	At4g22460	1,00	1,05	0,85	1,19	1,22	0,87	0,94	1,16	protease inhibitor/seed storage/lipid transfer protein (LTP) family protein
254314_at	At4g22470	0,67	0,66	0,42	1,69	1,60	0,85	0,57	0,52	protease inhibitor/seed storage/lipid transfer protein (LTP) family protein
254315_at	At4g22480	0,86	0,94	0,96	0,89	0,99	0,94	1,01	1,00	---
254327_at	At4g22490	1,32	1,19	1,11	0,72	0,75	1,22	1,23	1,38	protease inhibitor/seed storage/lipid transfer protein (LTP) family protein
254316_at	At4g22500	0,77	0,92	1,10	0,83	1,12	0,98	0,98	1,25	---
254317_at	At4g22510	1,14	1,21	0,96	0,99	0,90	0,89	1,00	0,97	hypothetical protein
254318_at	At4g22530	0,90	1,07	0,93	0,89	0,98	0,92	0,93	0,84	embryo-abundant protein-related
254329_at	At4g22540	0,96	1,26	1,35	0,68	0,72	0,95	1,16	1,16	oxysterol-binding family protein
254319_at	At4g22560	1,04	0,97	0,89	1,02	0,94	0,90	1,08	1,06	expressed protein
254328_at	At4g22570	1,62	1,34	1,48	1,50	1,04	0,94	1,14	1,13	adenine phosphoribosyltransferase, putative
254320_at	At4g22580	0,62	0,60	0,62	0,92	1,01	0,68	0,62	0,83	exostosin family protein

254321_at	At4g22590	0,91	1,02	1,02	1,04	1,05	1,17	1,43	1,56 trehalose-6-phosphate phosphatase, putative
254322_at	At4g22600	1,02	1,09	0,94	0,94	1,14	1,01	1,07	0,95 hypothetical protein
254326_at	At4g22610	0,96	1,17	0,96	1,51	1,21	1,08	1,41	1,46 protease inhibitor/seed storage/lipid transfer protein (LTP) family protein
254323_at	At4g22620	0,89	1,15	0,99	2,86	1,37	1,57	1,25	1,05 auxin-responsive family protein
254324_at	At4g22640	1,05	1,06	1,06	1,14	1,10	0,95	0,96	1,04 expressed protein
254325_at	At4g22650	0,91	1,08	0,96	0,96	1,22	1,02	0,93	0,83 hypothetical protein
254275_at	At4g22670	1,21	1,18	1,08	1,19	1,12	0,92	1,03	1,09 tetratricopeptide repeat (TPR)-containing protein
254277_at	At4g22680	1,02	1,09	1,02	1,08	1,11	0,91	0,95	1,11 myb family transcription factor (MYB85)
254330_at	At4g22700	1,06	0,99	0,91	0,92	0,98	0,92	0,95	0,83 LOB domain family protein / lateral organ boundaries domain family protein
254331_s_at	At4g22710	0,95	1,01	0,98	0,90	0,80	0,87	0,80	0,86 cytochrome P450 family protein
254273_at	At4g22720	1,19	1,09	1,18	0,99	1,00	1,10	0,94	0,99 glycoprotease M22 family protein
254332_at	At4g22730	0,99	0,99	0,99	0,90	1,09	0,91	0,94	1,06 leucine-rich repeat transmembrane protein kinase, putative
254278_at	At4g22740	1,35	1,29	1,65	1,00	1,20	1,17	1,14	1,23 glycine-rich protein
254279_at	At4g22750	0,85	0,77	0,81	0,78	0,91	0,93	0,87	0,81 zinc finger (DHHC type) family protein
254333_at	At4g22753	0,64	0,79	0,93	1,37	1,31	1,28	1,15	0,89 sterol desaturase family protein
254280_at	At4g22756	0,79	0,73	0,74	0,98	0,96	0,69	0,89	0,67 sterol desaturase family protein
254334_at	At4g22760	0,86	0,93	1,06	0,95	1,07	0,91	0,96	1,04 pentatricopeptide (PPR) repeat-containing protein
254274_at	At4g22770	0,84	0,93	0,95	1,04	0,64	0,88	0,98	1,21 DNA-binding family protein
254300_at	At4g22780	0,78	2,28	1,86	0,91	0,83	1,26	1,81	1,33 ACT domain-containing protein (ACR7)
254301_at	At4g22790	0,82	0,88	0,83	0,97	0,92	0,82	0,95	0,97 MATE efflux family protein
254302_at	At4g22800	1,01	1,06	0,98	1,02	1,01	0,97	1,03	0,92 hypothetical protein
254276_at	At4g22820	0,86	0,90	1,08	1,00	1,37	0,87	1,08	1,16 zinc finger (AN1-like) family protein
254303_at	At4g22830	0,81	0,74	0,74	1,05	1,08	0,85	0,78	0,77 expressed protein
254281_at	At4g22840	1,15	1,25	0,98	1,32	1,16	0,90	0,88	0,83 bile acid:sodium symporter family protein
254272_at	At4g22850	1,17	1,05	1,05	1,18	1,00	0,99	0,93	0,86 expressed protein
254282_at	At4g22860	1,16	1,26	1,12	1,07	1,24	1,11	1,01	0,92 expressed protein
254283_s_at	At4g22870	1,07	1,34	1,24	0,93	1,09	1,16	1,14	1,13 leucoanthocyanidin dioxygenase, putative / anthocyanidin synthase, putativ
254298_at	At4g22890	0,90	0,90	1,00	0,93	0,90	1,18	1,18	1,16 expressed protein
254284_at	At4g22910	0,98	0,87	0,69	0,84	1,08	0,93	0,86	0,78 WD-40 repeat family protein
254299_at	At4g22920	0,81	1,03	1,28	0,80	0,94	1,06	1,15	1,17 expressed protein
254267_at	At4g22930	1,08	1,02	0,87	1,13	1,05	1,02	0,95	0,98 dihydroorotase, mitochondrial / DHOase (PYR4)
254285_at	At4g22940	0,96	1,00	1,05	1,13	0,92	0,95	0,89	0,97 protein kinase family protein
254286_at	At4g22950	0,95	1,02	0,96	0,95	1,01	0,89	1,15	1,13 MADS-box protein (AGL19)
254287_at	At4g22960	1,22	1,06	1,18	0,98	1,00	0,96	1,05	1,10 hypothetical protein
254288_at	At4g22970	1,05	1,06	0,99	0,84	0,80	1,05	1,08	1,01 peptidase C50 family protein
254289_at	At4g22980	0,44	0,82	0,85	0,69	1,03	0,94	1,02	0,92 expressed protein
254290_at	At4g23000	1,08	1,22	1,04	1,02	1,04	1,12	1,08	1,21 calcineurin-like phosphoesterase family protein
254291_at	At4g23010	0,69	0,68	0,84	1,33	1,18	0,91	0,95	0,99 UDP-galactose transporter-related
254292_at	At4g23030	0,77	0,66	0,82	0,98	0,88	0,85	1,01	1,11 MATE efflux protein-related
254268_at	At4g23040	0,89	1,01	1,13	0,80	1,01	0,83	1,04	1,08 UBX domain-containing protein
254269_at	At4g23050	0,57	0,49	0,57	0,70	0,70	1,02	1,04	1,29 protein kinase, putative
254293_at	At4g23060	0,53	1,09	1,27	0,92	0,92	1,03	1,34	1,42 calmodulin-binding family protein
254294_at	At4g23070	0,66	0,66	0,87	0,87	0,70	1,01	1,18	0,96 rhomboid family protein
254295_at	At4g23080	0,99	1,00	0,95	0,90	1,14	0,96	1,09	1,32 expressed protein
254296_at	At4g23090	1,04	1,15	1,04	1,08	0,89	1,06	0,84	0,99 hypothetical protein
254270_at	At4g23100	0,97	0,84	0,86	0,89	0,77	0,88	0,91	0,92 glutamate-cysteine ligase / gamma-glutamylcysteine synthetase (GSH1)

254297_at	At4g23110	0,91	0,98	1,08	1,05	1,00	0,99	1,11	1,02	hypothetical protein
254266_at	At4g23130	0,90	0,81	0,98	0,88	0,97	1,07	1,06	1,08	receptor-like protein kinase 6 (RLK6)
254265_s_at	At4g23140	0,71	0,97	0,87	0,95	0,97	1,80	1,29	1,27	receptor-like protein kinase 5 (RLK5)
254271_at	At4g23150	0,86	0,94	1,00	1,06	0,91	1,33	1,14	1,15	protein kinase family protein
254256_at	At4g23180	0,53	0,63	1,35	0,54	0,59	0,93	1,31	1,82	receptor-like protein kinase 4, putative (RLK4)
254241_at	At4g23190	0,65	0,77	1,33	0,77	0,80	1,14	1,39	2,02	protein kinase family protein
254242_at	At4g23200	0,51	0,43	0,71	0,89	0,93	0,88	0,86	0,94	protein kinase family protein
254243_at	At4g23210	1,22	1,12	1,07	1,03	1,10	0,95	0,99	0,91	protein kinase family protein
254255_at	At4g23220	0,65	0,61	0,94	0,85	0,89	1,21	1,03	1,16	protein kinase family protein
254244_at	At4g23230	0,85	0,95	1,07	0,91	1,09	0,94	1,17	1,01	protein kinase family protein
254245_at	At4g23240	1,20	1,05	1,08	1,30	0,98	1,06	1,05	1,04	protein kinase family protein
254246_at	At4g23250	1,03	0,95	1,24	1,02	1,24	1,06	0,89	0,75	protein kinase family protein
254247_at	At4g23260	0,82	0,69	0,74	0,73	0,69	1,36	1,57	1,66	protein kinase family protein
254248_at	At4g23270	0,73	0,73	1,05	0,69	0,80	0,95	1,20	1,28	protein kinase family protein
254249_at	At4g23280	0,95	0,93	1,13	0,88	1,11	1,13	1,17	1,04	protein kinase, putative
254250_at	At4g23290	0,95	0,88	1,03	1,05	1,07	1,06	1,07	0,93	protein kinase family protein
254251_at	At4g23300	0,93	1,11	1,18	0,91	1,09	1,07	1,31	1,47	protein kinase family protein
254252_at	At4g23310	0,83	1,12	1,05	1,06	1,18	1,12	0,96	1,15	receptor-like protein kinase, putative
254253_at	At4g23320	0,98	0,87	0,94	1,00	1,12	0,98	1,07	1,05	protein kinase family protein
254254_at	At4g23330	0,88	0,86	1,13	1,21	1,02	1,03	1,12	0,93	eukaryotic translation initiation factor-related
254257_s_at	At4g23350	0,97	0,99	0,85	0,95	1,09	1,08	0,97	0,88	expressed protein
254239_at	At4g23400	0,81	0,80	0,83	1,01	0,88	0,76	0,68	0,76	major intrinsic family protein / MIP family protein
254258_at	At4g23410	1,25	1,19	1,20	1,58	1,13	0,81	0,96	0,86	senescence-associated family protein
254259_s_at	At4g23420	0,89	0,79	0,75	1,03	1,02	1,01	1,13	1,05	short-chain dehydrogenase/reductase (SDR) family protein
254260_at	At4g23440	0,66	0,81	1,02	0,77	1,09	0,82	0,87	0,93	expressed protein
254210_at	At4g23450	1,04	0,97	1,00	0,93	1,03	1,29	0,96	1,21	zinc finger (C3HC4-type RING finger) family protein
254261_at	At4g23460	0,81	0,70	0,67	1,02	1,22	0,90	0,89	0,89	beta-adaptin, putative
254262_at	At4g23480	0,81	0,70	0,72	0,96	1,14	0,94	0,92	1,02	hydroxyproline-rich glycoprotein family protein
254209_at	At4g23490	0,97	1,09	0,94	1,27	0,97	0,98	0,90	0,82	fringe-related protein
254263_at	At4g23493	1,24	1,11	1,02	1,32	1,13	1,05	0,93	1,01	expressed protein
254240_at	At4g23496	1,03	1,13	0,87	1,08	0,94	0,90	1,03	1,06	expressed protein
254264_at	At4g23510	1,03	0,90	0,88	1,43	1,30	0,96	1,13	1,19	disease resistance protein (TIR class), putative
254237_at	At4g23520	1,00	0,92	1,05	1,04	1,08	0,90	0,93	1,06	cysteine proteinase, putative
254238_at	At4g23540	1,18	0,94	0,89	0,88	0,84	1,20	0,96	1,04	expressed protein
254211_at	At4g23570	1,04	1,06	1,05	1,04	1,09	1,06	1,20	1,31	phosphatase-related
254212_at	At4g23580	1,01	1,11	1,09	1,00	1,20	1,19	1,00	0,81	kelch repeat-containing F-box family protein
254213_at	At4g23590	1,01	1,01	0,95	1,05	1,18	0,99	1,03	0,93	aminotransferase class I and II family protein
254232_at	At4g23600	0,36	0,33	0,40	2,41	2,19	0,55	0,56	0,57	coronatine-responsive tyrosine aminotransferase / tyrosine transaminase
254229_at	At4g23610	1,05	1,02	0,83	1,15	1,13	1,04	0,94	1,04	expressed protein
254228_at	At4g23620	1,56	1,54	1,37	1,54	1,57	1,03	0,97	0,90	50S ribosomal protein-related
254227_at	At4g23630	1,14	1,04	0,95	1,09	1,03	0,96	0,89	0,91	reticulon family protein (RTNLB1)
254214_at	At4g23640	0,84	0,84	0,81	1,10	1,14	0,99	0,94	0,85	potassium transporter / tiny root hair 1 protein (TRH1)
254224_at	At4g23650	0,68	0,66	0,67	0,99	1,14	0,99	0,93	0,93	calcium-dependent protein kinase, putative / CDPK, putative
254230_at	At4g23660	1,02	0,95	1,01	1,13	1,27	1,01	0,95	0,85	UbiA prenyltransferase family protein
254225_at	At4g23670	0,62	0,55	0,57	1,02	0,96	0,83	0,76	0,77	major latex protein-related / MLP-related
254234_at	At4g23680	1,83	1,51	1,37	1,61	1,47	1,36	1,23	1,40	major latex protein-related / MLP-related

254226_at	At4g23690	1,45	1,27	1,05	2,36	1,84	1,17	1,23	1,17	disease resistance-responsive family protein / dirigent family protein
254215_at	At4g23700	1,11	1,42	1,28	0,93	0,97	1,18	1,04	1,13	cation/hydrogen exchanger, putative (CHX17)
254216_at	At4g23710	1,07	0,89	0,69	1,23	1,14	0,96	0,85	0,97	vacuolar ATP synthase subunit G 2 (VATG2) / V-ATPase G subunit 2 (VAG
254217_at	At4g23720	0,98	1,05	0,97	0,99	1,00	0,94	0,93	0,91	expressed protein
254223_at	At4g23730	1,29	1,55	1,88	2,02	2,41	1,07	1,20	1,19	aldose 1-epimerase family protein
254218_at	At4g23740	0,95	0,95	0,95	0,90	1,18	1,13	1,09	1,20	leucine-rich repeat transmembrane protein kinase, putative
254235_at	At4g23750	0,86	0,97	0,80	0,79	0,87	0,86	0,84	0,72	AP2 domain-containing transcription factor, putative
254219_at	At4g23760	1,09	1,12	1,18	1,06	1,05	0,98	1,06	0,97	expressed protein
254220_at	At4g23780	1,03	0,98	0,96	1,02	1,02	1,17	1,01	0,90	hypothetical protein
254233_at	At4g23800	2,05	1,47	1,36	1,35	1,59	1,16	1,12	1,08	high mobility group (HMG1/2) family protein
254231_at	At4g23810	0,83	1,13	0,84	0,61	0,59	1,56	0,97	0,92	WRKY family transcription factor
254221_at	At4g23820	1,00	0,92	0,87	0,89	0,83	0,71	0,64	0,63	glycoside hydrolase family 28 protein / polygalacturonase (pectinase) famil
254222_at	At4g23830	1,07	1,16	1,00	1,06	1,03	1,00	0,93	0,84	leucine-rich repeat family protein
254236_at	At4g23840	1,11	0,96	1,32	0,80	1,08	1,11	0,88	0,90	leucine-rich repeat family protein
254192_at	At4g23850	0,97	0,84	0,78	1,14	1,03	1,04	1,10	1,04	long-chain-fatty-acid--CoA ligase / long-chain acyl-CoA synthetase
254177_at	At4g23860	1,11	1,43	1,52	1,20	1,25	1,12	0,97	0,99	PHD finger protein-related
254193_at	At4g23870	0,66	0,74	0,83	0,64	0,69	1,03	0,81	0,80	expressed protein
254178_at	At4g23880	0,74	0,90	1,13	0,59	0,93	1,19	1,00	0,87	expressed protein
254190_at	At4g23885	2,22	1,83	1,62	1,75	1,56	1,32	1,30	1,25	expressed protein
254187_at	At4g23890	0,93	1,11	1,39	0,72	0,87	1,11	1,07	1,17	expressed protein
254191_at	At4g23900	0,94	0,90	0,78	1,25	1,16	0,86	0,88	0,77	nucleoside diphosphate kinase 4 (NDK4)
254179_at	At4g23910	0,94	0,86	1,16	1,01	0,95	0,88	0,92	1,02	expressed protein
254188_at	At4g23920	0,61	0,58	0,68	0,99	0,90	0,81	0,94	0,77	UDP-glucose 4-epimerase, putative / UDP-galactose 4-epimerase, putative
254180_at	At4g23930	0,90	0,89	1,07	1,04	1,04	0,92	0,87	0,81	expressed protein
254181_at	At4g23940	1,07	1,07	0,93	0,84	0,87	1,27	1,07	1,12	FtsH protease, putative
254182_at	At4g23950	1,06	1,03	1,10	1,01	0,86	0,93	1,03	0,89	expressed protein
254183_at	At4g23960	0,94	0,92	0,99	1,06	1,13	0,96	1,01	0,87	F-box family protein
254184_at	At4g23970	0,94	0,82	0,88	0,97	1,07	1,13	1,05	1,06	hypothetical protein
254194_at	At4g23980	1,25	1,14	1,17	0,99	1,45	1,08	1,06	0,74	auxin-responsive factor (ARF9)
254185_at	At4g23990	1,16	1,19	1,17	0,85	0,65	0,99	1,09	0,85	cellulose synthase family protein
254189_at	At4g24000	0,83	0,97	0,88	0,90	1,06	1,12	0,97	0,82	cellulose synthase family protein
254186_at	At4g24010	0,96	1,05	0,96	0,98	1,10	1,02	0,95	1,01	cellulose synthase family protein
254195_at	At4g24020	0,90	0,94	1,13	0,75	0,77	1,07	1,09	1,30	RWP-RK domain-containing protein
254196_at	At4g24030	0,87	0,98	0,88	0,97	0,88	0,91	0,85	1,02	hypothetical protein
254197_at	At4g24040	0,43	0,44	0,40	0,70	0,63	0,64	0,66	0,79	glycosyl hydrolase family protein 37 / trehalase, putative
254175_at	At4g24050	1,25	1,85	1,84	0,96	0,65	1,06	1,23	1,09	short-chain dehydrogenase/reductase (SDR) family protein
254207_at	At4g24060	1,72	1,99	2,91	0,96	0,88	1,00	1,64	1,86	Dof-type zinc finger domain-containing protein
254198_at	At4g24090	1,11	0,95	1,13	0,76	0,65	1,12	1,04	0,97	expressed protein
254199_at	At4g24100	1,09	0,97	1,00	0,93	1,20	0,93	1,04	1,19	protein kinase family protein
254200_at	At4g24110	0,42	0,61	0,67	0,60	0,84	0,61	0,60	0,75	expressed protein
254174_at	At4g24120	1,16	1,07	1,03	1,24	1,01	0,60	0,68	0,52	transporter, putative
254201_at	At4g24130	1,08	0,99	0,81	1,06	0,91	0,95	0,83	0,76	expressed protein
254202_at	At4g24140	1,04	1,28	0,88	1,20	0,96	0,77	0,73	0,81	hydrolase, alpha/beta fold family protein
254203_at	At4g24150	1,08	0,96	1,03	1,07	1,08	1,08	1,09	1,33	expressed protein
254204_at	At4g24160	0,90	1,11	1,47	0,87	0,88	1,21	1,42	1,62	hydrolase, alpha/beta fold family protein
254205_at	At4g24170	0,99	1,00	0,95	1,00	1,17	1,03	1,02	1,14	kinesin motor family protein

254208_at	At4g24175	1,09	1,01	1,16	0,86	0,71	0,99	1,01	0,88 expressed protein
254206_at	At4g24180	1,01	0,95	1,22	1,03	1,14	1,12	1,09	1,05 pathogenesis-related thaumatin family protein
254166_at	At4g24190	1,25	1,16	1,16	1,38	1,22	1,05	1,04	1,25 shepherd protein (SHD) / clavata formation protein, putative
254176_at	At4g24200	0,79	0,84	0,99	0,91	0,90	1,03	0,83	1,10 expressed protein
254160_at	At4g24210	0,69	0,82	0,87	1,22	1,48	0,79	0,82	0,86 F-box family protein / SLEEPY1 protein
254157_at	At4g24220	0,68	0,70	0,76	1,03	0,94	0,94	0,87	0,80 expressed protein
254159_at	At4g24240	0,89	1,01	0,91	0,75	1,05	1,04	0,70	0,85 WRKY family transcription factor
254168_at	At4g24250	1,07	1,11	1,05	0,88	1,24	0,98	1,01	0,96 seven transmembrane MLO family protein / MLO-like protein 13 (MLO13)
254146_at	At4g24260	0,90	1,00	1,05	0,99	1,13	1,02	0,91	0,92 endo-1,4-beta-glucanase, putative / cellulase, putative
254147_at	At4g24270	1,34	1,18	1,15	0,77	0,97	1,12	0,99	1,21 RNA recognition motif (RRM)-containing protein
254148_at	At4g24280	1,22	1,02	1,01	0,80	0,77	1,11	1,11	1,18 heat shock protein 70, putative / HSP70, putative
254169_at	At4g24290	0,71	0,72	0,80	0,92	1,06	0,94	0,92	0,87 expressed protein
254149_at	At4g24300	0,92	1,06	1,03	1,01	1,14	0,93	0,98	1,06 hypothetical protein
254165_at	At4g24330	1,02	0,91	0,93	1,14	1,09	0,97	0,88	0,89 expressed protein
254163_s_at	At4g24340	0,78	0,69	0,59	1,07	0,91	0,80	0,90	1,05 phosphorylase family protein
254150_at	At4g24350	0,77	0,60	0,65	0,89	0,91	0,79	0,74	1,00 phosphorylase family protein
254161_at	At4g24370	0,67	0,84	0,83	1,20	1,30	0,96	0,79	0,72 expressed protein
254158_at	At4g24380	0,79	1,52	1,65	0,82	1,05	1,37	2,07	1,69 expressed protein
254151_at	At4g24390	1,06	1,36	1,13	0,80	0,85	0,81	0,97	1,53 F-box family protein (FBX14)
254167_at	At4g24400	0,96	0,89	1,20	1,06	1,11	1,08	1,03	1,18 CBL-interacting protein kinase 8 (CIPK8)
254152_at	At4g24410	1,05	0,93	1,08	0,90	0,99	0,96	0,98	1,07 expressed protein
254170_at	At4g24430	1,08	1,23	1,33	1,18	1,22	1,00	1,13	1,37 expressed protein
254162_at	At4g24440	1,13	0,98	1,15	1,14	1,09	0,96	0,98	0,89 transcription initiation factor IIA gamma chain / TFIIA-gamma (TFIIA-S)
254153_at	At4g24450	1,30	1,13	1,39	1,15	1,19	0,84	1,19	1,07 starch excess protein-related
254154_at	At4g24460	0,82	0,80	0,75	0,97	0,89	0,93	0,76	0,76 expressed protein
254164_at	At4g24470	1,02	1,13	1,15	1,01	1,04	1,05	1,01	0,95 zinc finger (GATA type) protein ZIM (ZIM)
254155_at	At4g24480	0,82	0,90	0,85	0,76	0,77	0,96	1,05	1,19 serine/threonine protein kinase, putative
254156_at	At4g24490	1,09	1,01	1,13	1,03	1,09	1,08	0,93	0,87 geranylgeranyl transferase alpha subunit-related / RAB geranylgeranyltrans
254124_at	At4g24500	1,06	1,01	0,95	0,89	1,10	1,01	0,99	0,95 hydroxyproline-rich glycoprotein family protein
254122_at	At4g24510	1,25	1,05	1,37	1,04	1,24	0,88	0,76	0,82 eceriferum protein (CER2)
254127_at	At4g24520	0,67	0,64	0,65	0,77	0,82	0,90	0,85	0,93 NADPH-cytochrome p450 reductase, putative / NADPH-ferrihemoprotein re
254171_at	At4g24530	1,05	1,05	0,80	1,15	0,98	0,82	0,88	1,06 expressed protein
254130_at	At4g24540	0,97	0,98	0,96	1,59	1,36	0,64	0,72	0,55 MADS-box family protein
254172_at	At4g24550	0,90	0,87	0,94	1,08	1,01	1,03	0,98	1,04 clathrin adaptor complexes medium subunit family protein
254128_at	At4g24560	0,79	0,97	1,30	0,89	1,13	1,12	1,02	1,76 ubiquitin-specific protease 16, putative (UBP16)
254120_at	At4g24570	0,87	0,77	1,00	0,92	0,99	0,96	1,01	1,03 mitochondrial substrate carrier family protein
254173_at	At4g24580	0,94	1,04	0,92	1,08	1,01	1,10	1,15	1,09 pleckstrin homology (PH) domain-containing protein-related / RhoGAP dom
254139_at	At4g24600	1,07	1,11	1,01	1,01	0,99	0,97	1,05	0,97 hypothetical protein
254140_at	At4g24610	1,07	1,19	1,31	0,91	1,08	0,97	0,83	0,79 expressed protein
254141_at	At4g24620	0,82	0,86	0,81	0,93	0,99	0,97	1,00	1,01 glucose-6-phosphate isomerase, putative
254142_at	At4g24630	0,92	1,02	1,18	0,83	0,98	1,06	0,91	1,11 zinc finger (DHHC type) family protein
254123_at	At4g24640	1,00	1,05	0,92	0,88	1,13	0,96	1,01	1,08 invertase/pectin methylesterase inhibitor family protein
254129_at	At4g24650	0,96	0,95	1,03	1,12	1,10	0,96	0,90	1,26 adenylate isopentenyltransferase 4 / cytokinin synthase (IPT4)
254132_at	At4g24660	1,03	1,03	1,03	1,14	0,78	0,80	0,72	0,92 zinc finger homeobox family protein / ZF-HD homeobox family protein
254125_at	At4g24670	0,72	0,92	1,07	0,96	0,86	0,91	0,90	1,00 alliinase family protein
254143_at	At4g24680	0,94	0,87	1,07	0,90	0,88	1,02	1,02	1,16 expressed protein

254144_at	At4g24690	1,01	1,04	0,96	0,81	0,73	1,10	1,09	0,97 ubiquitin-associated (UBA)/TS-N domain-containing protein / octicosapeptic
254145_at	At4g24700	1,43	1,31	1,27	1,08	1,26	1,09	0,97	1,00 expressed protein
254115_at	At4g24710	0,94	0,97	1,20	0,95	1,06	0,92	1,07	1,15 AAA-type ATPase family protein
254116_at	At4g24730	0,80	0,80	1,00	1,11	1,14	0,88	1,05	1,09 calcineurin-like phosphoesterase family protein
254131_at	At4g24740	1,08	1,10	1,46	0,93	1,04	1,17	1,53	1,75 protein kinase (AFC2)
254117_at	At4g24750	0,76	0,74	0,69	0,79	0,71	0,98	1,01	1,11 expressed protein
254121_at	At4g24760	1,28	1,28	1,31	1,23	1,20	1,22	1,01	1,28 expressed protein
254126_at	At4g24770	1,12	1,06	0,99	0,80	0,85	1,03	1,07	0,97 31 kDa ribonucleoprotein, chloroplast, putative / RNA-binding protein RNP-'
254119_at	At4g24780	1,56	1,44	1,15	0,92	1,21	1,14	1,20	1,51 pectate lyase family protein
254118_at	At4g24790	0,97	1,09	0,97	0,95	1,08	1,08	1,10	1,01 expressed protein
254089_at	At4g24800	1,03	1,40	1,66	1,01	1,21	1,06	1,09	1,00 MA3 domain-containing protein
254133_at	At4g24810	1,09	1,15	1,00	0,59	0,65	1,23	1,07	1,17 ABC1 family protein
254086_at	At4g24820	1,03	0,94	1,06	0,86	1,14	0,90	0,93	0,86 26S proteasome regulatory subunit, putative (RPN7)
254134_at	At4g24830	1,21	1,07	1,15	1,05	0,97	0,89	0,93	0,95 arginosuccinate synthase family
254087_at	At4g24840	0,95	0,81	1,03	1,24	0,87	0,98	0,99	0,95 expressed protein
254135_at	At4g24850	1,02	1,03	1,21	0,96	1,02	0,96	1,10	1,11 AAA-type ATPase family protein
254136_at	At4g24860	0,95	0,97	0,96	1,12	1,14	1,05	1,00	0,96 AAA-type ATPase family protein
254088_at	At4g24880	1,30	1,17	1,07	0,95	1,08	0,99	0,82	0,80 expressed protein
254111_at	At4g24890	0,99	1,08	1,02	1,23	1,13	1,18	1,09	1,01 calcineurin-like phosphoesterase family protein
254113_at	At4g24900	1,36	1,35	1,08	0,82	0,96	1,32	1,64	1,44 expressed protein
254083_at	At4g24920	1,04	0,79	0,74	1,18	1,13	1,00	0,90	0,91 protein transport protein SEC61 gamma subunit, putative
254137_at	At4g24930	1,45	1,19	1,06	1,00	0,96	1,28	1,18	1,03 thylakoid lumenal 17.9 kDa protein, chloroplast
254084_at	At4g24940	1,07	0,91	1,08	1,01	0,98	1,00	0,97	0,98 SUMO activating enzyme 1a (SAE1a)
254138_at	At4g24950	0,97	0,96	1,01	1,00	1,00	1,00	1,03	1,18 hypothetical protein
254085_at	At4g24960	1,20	0,90	1,18	1,45	1,30	1,20	1,12	1,18 ABA-responsive protein (HVA22d)
254112_at	At4g24970	0,96	0,92	0,85	0,87	0,87	0,91	0,95	1,26 ATP-binding region, ATPase-like domain-containing protein
254114_at	At4g24980	0,98	1,08	1,15	1,04	1,02	0,94	1,02	1,02 nodulin MtN21 family protein
254106_at	At4g24990	0,65	0,73	0,77	1,30	1,18	0,98	0,90	1,03 ubiquitin family protein
254101_at	At4g25000	0,85	1,00	0,96	0,99	1,09	1,09	0,93	1,02 alpha-amylase, putative / 1,4-alpha-D-glucan glucanohydrolase, putative
254090_at	At4g25010	0,94	0,97	0,94	0,95	0,83	0,99	1,07	0,93 nodulin MtN3 family protein
254100_at	At4g25020	1,40	1,31	1,04	1,46	1,74	1,16	1,08	1,29 KOW domain-containing protein / D111/G-patch domain-containing protein
254103_at	At4g25030	0,69	0,72	0,81	0,79	0,91	0,71	0,79	0,84 expressed protein
254104_at	At4g25040	1,02	1,04	1,14	1,06	0,87	1,09	1,01	0,87 integral membrane family protein
254102_at	At4g25050	1,22	1,06	1,00	0,90	0,92	1,07	1,00	1,04 acyl carrier family protein / ACP family protein
254091_at	At4g25060	1,13	1,03	0,94	0,93	1,09	0,95	0,87	1,13 expressed protein
254105_at	At4g25080	1,14	1,14	1,30	0,84	0,75	1,11	1,19	1,33 magnesium-protoporphyrin O-methyltransferase, putative / magnesium-prot
254092_at	At4g25090	1,16	0,97	0,94	1,04	1,09	1,03	1,04	1,07 respiratory burst oxidase, putative / NADPH oxidase, putative
254098_at	At4g25100	0,96	0,90	0,95	0,83	0,90	0,55	0,60	0,57 superoxide dismutase (Fe), chloroplast (SODB) / iron superoxide dismutase
254093_at	At4g25110	1,05	0,95	0,88	1,13	0,90	0,92	0,95	1,15 latex-abundant family protein (AMC2) / caspase family protein
254094_at	At4g25120	1,00	0,90	0,99	1,05	0,79	0,99	1,03	0,86 UvrD/REP helicase family protein
254099_at	At4g25130	1,04	0,96	0,91	1,00	0,95	1,06	1,01	1,03 peptide methionine sulfoxide reductase, putative
254095_at	At4g25140	0,07	0,06	0,03	0,44	0,43	1,01	1,11	1,04 glycine-rich protein / oleosin
254096_at	At4g25150	1,15	1,15	1,22	1,32	2,01	0,92	0,94	0,71 acid phosphatase, putative
254097_at	At4g25160	1,01	1,07	1,00	1,08	1,13	0,86	1,07	1,05 protein kinase family protein
254057_at	At4g25170	0,56	0,60	0,69	0,68	0,74	0,96	1,08	1,12 expressed protein
254059_at	At4g25200	2,04	1,75	1,37	1,02	1,27	1,22	0,96	0,89 23.6 kDa mitochondrial small heat shock protein (HSP23.6-M)

254058_at	At4g25210	0,95	0,79	0,96	0,95	0,94	1,05	1,05	1,05 expressed protein
254107_at	At4g25220	1,11	0,90	0,95	0,81	1,08	1,01	0,99	0,71 transporter, putative
254108_at	At4g25230	0,88	0,80	0,78	0,89	0,94	0,90	0,98	0,96 zinc finger (C3HC4-type RING finger) family protein
254109_at	At4g25240	1,34	1,20	0,95	1,21	0,93	0,99	1,04	0,80 multi-copper oxidase type I family protein
254056_at	At4g25250	0,99	1,03	0,98	0,95	0,99	1,07	0,93	0,89 invertase/pectin methylesterase inhibitor family protein
254110_at	At4g25260	0,86	0,68	0,58	1,01	0,86	0,63	0,51	0,41 invertase/pectin methylesterase inhibitor family protein
254051_at	At4g25270	0,99	0,91	0,97	0,74	0,88	0,99	0,86	1,28 pentatricopeptide (PPR) repeat-containing protein
254052_at	At4g25280	0,89	0,76	0,71	1,08	1,11	1,06	1,11	1,25 adenylate kinase family protein
254053_s_at	At4g25300	0,81	0,63	0,97	0,98	1,15	0,97	1,06	1,01 oxidoreductase, 2OG-Fe(II) oxygenase family protein
254054_at	At4g25320	1,11	0,86	0,74	1,04	0,87	1,02	0,84	0,92 DNA-binding protein-related
254055_at	At4g25330	0,99	1,04	1,03	1,02	1,15	1,01	0,94	0,93 expressed protein
254076_at	At4g25340	1,79	1,25	1,47	1,07	0,90	1,11	1,12	1,22 immunophilin-related / FKBP-type peptidyl-prolyl cis-trans isomerase-relate
254060_at	At4g25350	0,76	0,83	0,82	0,69	0,75	1,02	1,03	1,28 EXS family protein / ERD1/XPR1/SYG1 family protein
254061_at	At4g25360	0,80	0,84	0,88	0,94	1,12	1,01	0,91	1,02 expressed protein
254072_at	At4g25370	0,90	0,78	0,81	1,15	1,18	0,90	0,92	1,02 Clp amino terminal domain-containing protein
254062_at	At4g25380	0,98	1,11	1,06	0,86	0,88	0,95	0,96	1,12 zinc finger (AN1-like) family protein
254063_at	At4g25390	0,58	0,64	0,91	0,74	0,78	0,98	1,18	1,38 protein kinase family protein
254064_at	At4g25410	0,96	0,86	0,91	0,93	0,85	0,88	1,16	1,33 basix helix-loop-helix (bHLH) family protein
254065_at	At4g25420	1,01	0,98	0,98	0,97	1,12	1,06	0,94	0,77 gibberellin 20-oxidase
254070_at	At4g25430	1,04	0,92	0,91	1,07	1,03	1,03	1,02	1,01 hypothetical protein
254069_at	At4g25440	1,07	1,13	1,12	1,04	1,12	1,12	1,22	0,99 MutT/nudix family protein
254068_at	At4g25450	1,10	1,11	1,18	0,90	0,96	1,08	1,21	1,07 ABC transporter family protein
254067_at	At4g25460	1,10	1,24	1,05	1,00	0,84	1,09	1,09	1,13 ABC transporter family protein
254075_at	At4g25470	1,10	1,37	1,06	1,00	0,65	1,24	1,27	0,97 DRE-binding protein (DREB1C) / CRT/DRE-binding factor 2 (CBF2)
254066_at	At4g25480	0,78	0,91	0,93	0,65	0,89	1,04	0,89	1,01 DRE-binding protein (DREB1A) / CRT/DRE-binding factor 3 (CBF3)
254074_at	At4g25490	0,46	0,91	0,87	0,29	0,56	0,87	1,11	1,01 DRE-binding protein (DREB1B) / CRT/CRE-binding factor 1 (CBF1) / transc
254073_at	At4g25500	1,15	0,97	1,17	0,82	0,85	1,04	1,11	1,09 arginine/serine-rich splicing factor RSP40 (RSP40)
254071_at	At4g25510	1,00	1,00	1,03	0,98	0,98	1,02	1,08	1,07 hypothetical protein
245237_at	At4g25520	0,92	0,98	1,52	0,87	0,86	1,00	1,19	1,18 transcriptional co-regulator family protein
245239_at	At4g25530	0,93	0,89	0,72	0,97	1,29	1,11	1,01	0,88 homeodomain protein (FWA)
245236_at	At4g25540	1,14	1,05	1,09	0,93	1,20	1,08	0,91	0,99 DNA mismatch repair protein MSH3 (MSH3)
245235_at	At4g25550	0,97	0,84	0,79	0,99	1,06	0,86	0,85	0,86 expressed protein
245234_at	At4g25560	0,94	0,99	1,10	1,11	1,17	0,93	0,91	1,05 myb family transcription factor (MYB18)
245238_at	At4g25570	0,99	0,96	0,96	1,13	1,05	0,99	0,92	0,96 cytochrome B561 family protein
245233_at	At4g25580	0,89	1,13	1,29	0,76	0,72	1,13	0,95	1,08 stress-responsive protein-related
245232_at	At4g25590	1,12	1,08	0,89	1,03	1,11	0,96	0,99	1,13 actin-depolymerizing factor, putative
245231_at	At4g25600	0,96	0,89	1,05	0,89	0,98	1,00	0,96	0,93 ShTK domain-containing protein
245230_at	At4g25610	1,05	1,03	1,14	1,08	1,11	1,05	1,10	1,11 zinc finger (C2H2 type) family protein
245229_at	At4g25620	0,89	1,27	1,25	0,71	0,64	0,92	1,28	1,28 hydroxyproline-rich glycoprotein family protein
254080_at	At4g25630	1,10	0,95	0,73	1,61	1,31	1,00	0,94	0,88 fibrillarin 2 (FIB2)
254077_at	At4g25640	1,02	0,96	0,99	0,86	0,74	1,12	1,20	1,29 MATE efflux family protein
254021_at	At4g25650	0,87	0,99	1,06	0,64	0,76	1,14	1,29	1,46 Rieske (2Fe-2S) domain-containing protein
254081_at	At4g25660	1,05	0,94	1,17	1,07	1,01	1,17	1,00	1,36 expressed protein
254050_s_at	At4g25670	0,80	0,80	0,86	1,13	1,15	0,78	0,81	0,83 expressed protein
254048_at	At4g25680	1,03	0,73	0,84	1,27	1,18	0,89	0,78	0,97 expressed protein
254020_at	At4g25700	1,74	1,80	1,88	1,51	1,40	1,67	1,41	1,57 beta-carotene hydroxylase

254078_at	At4g25710	1,02	0,85	1,16	0,84	0,93	0,81	0,95	0,94	kelch repeat-containing F-box family protein
254082_at	At4g25720	0,85	0,74	0,84	1,19	1,11	0,93	1,00	0,85	glutamine cyclotransferase family protein
254079_at	At4g25730	1,26	1,02	1,06	0,89	1,09	1,07	1,00	1,10	FtsJ-like methyltransferase family protein
254049_at	At4g25740	1,15	1,09	0,97	1,26	1,34	0,96	0,90	0,85	40S ribosomal protein S10 (RPS10A)
254022_at	At4g25750	0,98	0,99	0,94	1,02	1,01	0,85	0,87	0,79	ABC transporter family protein
254037_at	At4g25760	1,03	0,94	0,98	0,94	1,13	1,00	0,97	1,13	expressed protein
254023_at	At4g25770	0,85	0,81	0,77	0,95	0,94	0,92	0,88	0,96	expressed protein
254024_at	At4g25780	0,85	1,08	0,99	0,89	0,92	0,84	1,16	0,97	pathogenesis-related protein, putative
254025_at	At4g25790	0,99	0,77	0,77	1,18	1,18	1,11	1,08	1,03	allergen V5/Tpx-1-related family protein
254026_at	At4g25800	0,89	1,02	0,95	0,97	0,98	0,84	1,02	0,88	calmodulin-binding protein
254042_at	At4g25810	1,30	1,16	1,25	1,30	1,33	1,15	1,25	1,68	xyloglucan:xyloglucosyl transferase, putative / xyloglucan endotransglycosy
254044_at	At4g25820	1,42	1,07	1,02	1,47	2,38	1,03	1,38	1,27	xyloglucan:xyloglucosyl transferase / xyloglucan endotransglycosylase / en
254041_at	At4g25830	0,68	0,64	0,73	1,01	0,99	0,67	0,66	0,71	integral membrane family protein
254027_at	At4g25835	0,61	0,64	0,85	0,86	0,79	0,82	0,82	0,88	AAA-type ATPase family protein
254039_at	At4g25840	0,93	0,89	0,92	1,44	1,18	0,89	0,83	0,84	haloacid dehalogenase-like hydrolase family protein
254028_s_at	At4g25860	1,01	1,02	1,09	1,10	1,12	1,00	0,87	0,94	oxysterol-binding family protein
254029_at	At4g25870	0,88	0,77	0,70	0,90	1,11	1,08	0,83	0,72	expressed protein
254045_at	At4g25880	1,10	1,12	1,20	0,91	0,86	1,02	1,13	1,11	pumilio/Puf RNA-binding domain-containing protein
254030_at	At4g25890	1,41	1,25	1,07	1,38	1,46	1,09	0,96	0,92	60S acidic ribosomal protein P3 (RPP3A)
254040_at	At4g25900	1,03	1,00	0,89	1,00	0,93	1,11	0,97	0,92	aldose 1-epimerase family protein
254038_at	At4g25910	1,30	1,71	2,48	1,30	1,44	1,17	1,26	1,96	nitrogen fixation protein, putative
254031_at	At4g25920	1,02	1,04	1,00	0,93	1,05	1,01	0,89	0,87	expressed protein
254032_at	At4g25940	0,72	0,76	0,96	0,80	0,89	0,91	1,04	1,04	epsin N-terminal homology (ENTH) domain-containing protein
254033_at	At4g25950	1,01	1,01	1,02	0,98	0,93	0,96	0,97	1,09	vacuolar ATP synthase, putative / V-ATPase, putative / vacuolar proton pur
254034_at	At4g25960	1,05	1,04	1,23	0,93	0,79	1,02	0,89	1,15	multidrug resistance P-glycoprotein, putative
254035_at	At4g25970	0,61	0,56	0,58	0,94	0,84	0,82	0,87	0,89	phosphatidylserine decarboxylase, putative
254036_at	At4g25980	0,93	0,73	0,72	1,00	1,24	1,05	0,81	0,79	cationic peroxidase, putative
254043_at	At4g25990	1,16	0,90	0,96	0,97	0,98	0,87	0,89	1,00	expressed protein
253991_at	At4g26000	1,21	0,93	1,08	0,95	1,22	1,00	1,00	1,02	KH domain-containing protein
253998_at	At4g26010	1,16	0,97	1,05	1,41	1,46	0,91	1,06	0,93	peroxidase, putative
254046_at	At4g26020	1,04	0,99	0,97	1,07	1,04	1,00	0,97	1,03	expressed protein
254047_at	At4g26040	1,07	0,94	0,97	1,05	1,14	1,25	1,21	1,18	hypothetical protein
254013_at	At4g26050	0,92	1,08	1,12	1,14	1,20	1,06	1,00	1,10	leucine-rich repeat family protein
253992_at	At4g26060	0,69	0,72	0,76	1,20	1,09	0,96	0,96	1,13	expressed protein
253993_at	At4g26070	0,77	0,67	0,70	0,97	0,87	0,90	0,79	0,89	mitogen-activated protein kinase kinase (MAPKK) (MKK1) (MEK1)
253994_at	At4g26080	1,45	1,72	1,43	1,54	1,41	1,24	1,31	1,39	protein phosphatase 2C ABI1 / PP2C ABI1 / abscisic acid-insensitive 1 (AB
253997_at	At4g26090	0,78	1,03	0,83	0,63	1,16	0,84	1,15	1,04	disease resistance protein RPS2 (CC-NBS-LRR class), putative
253995_at	At4g26100	0,91	0,95	1,46	0,99	1,15	0,95	0,91	1,00	casein kinase, putative
253996_at	At4g26110	1,77	1,29	1,10	1,13	1,08	1,12	1,09	1,11	nucleosome assembly protein (NAP), putative
254014_at	At4g26120	1,23	1,16	1,16	1,02	1,14	1,21	1,01	1,09	ankyrin repeat family protein / BTB/POZ domain-containing protein
253989_at	At4g26130	0,52	0,43	0,49	1,07	1,26	0,74	0,68	0,70	expressed protein
254015_at	At4g26140	1,43	1,45	1,34	0,81	1,04	1,16	1,12	1,25	beta-galactosidase, putative / lactase, putative
254016_at	At4g26150	1,13	0,88	0,98	0,66	0,93	1,23	1,12	1,12	zinc finger (GATA type) family protein
253990_at	At4g26160	1,04	1,06	1,07	0,98	1,28	1,07	0,94	0,86	thioredoxin family protein
254017_at	At4g26170	0,97	1,03	1,02	1,00	0,93	1,01	1,05	1,08	expressed protein
254018_at	At4g26180	1,15	1,15	0,97	0,85	1,08	1,02	1,12	1,06	mitochondrial substrate carrier family protein

254019_at	At4g26190	0,98	0,91	0,98	0,85	0,93	1,25	1,50	1,32	expressed protein
253999_at	At4g26200	1,19	1,16	0,67	1,60	1,23	1,05	1,08	0,84	1-aminocyclopropane-1-carboxylate synthase, putative / ACC synthase, put
253986_at	At4g26210	1,14	1,00	0,93	1,04	0,99	1,01	0,96	0,97	mitochondrial ATP synthase g subunit family protein
253985_at	At4g26220	0,97	0,90	1,05	1,38	1,17	0,80	1,05	0,92	caffeoyl-CoA 3-O-methyltransferase, putative
254012_at	At4g26230	1,35	1,23	1,25	1,26	1,21	1,11	0,97	1,05	60S ribosomal protein L31 (RPL31B)
254010_at	At4g26240	0,93	0,86	0,86	1,02	0,85	1,02	0,91	1,04	expressed protein
254000_at	At4g26250	0,89	0,96	0,98	1,17	1,02	1,08	0,94	1,02	galactinol synthase, putative
254001_at	At4g26260	1,89	1,96	3,29	0,58	0,87	1,07	1,40	1,33	expressed protein
253987_at	At4g26270	0,83	0,81	0,69	0,94	1,01	1,14	0,94	0,94	phosphofructokinase family protein
254002_at	At4g26280	1,03	0,85	1,04	0,92	1,05	1,11	0,99	1,20	sulfotransferase family protein
254003_at	At4g26290	0,99	1,06	1,10	1,00	1,10	0,95	1,01	0,99	expressed protein
254004_at	At4g26300	1,17	1,05	0,96	0,98	0,84	0,98	1,03	1,01	arginyl-tRNA synthetase, putative / arginine--tRNA ligase, putative
253988_at	At4g26310	1,00	0,98	1,06	0,95	0,89	0,86	0,93	0,85	elongation factor P (EF-P) family protein
253957_at	At4g26320	0,85	0,87	0,75	1,39	0,95	1,00	1,32	0,90	arabinogalactan-protein (AGP13)
254005_at	At4g26330	0,97	1,23	1,12	0,91	1,10	0,91	0,98	1,04	subtilase family protein
254006_at	At4g26340	0,89	0,99	0,90	0,91	1,00	0,99	0,94	1,00	F-box family protein
254007_s_at	At4g26360	1,13	1,18	1,02	0,91	1,05	1,07	0,95	0,91	hypothetical protein
254011_at	At4g26370	0,98	1,04	1,22	0,74	0,82	1,04	0,96	0,92	antitermination NusB domain-containing protein
254008_at	At4g26380	1,03	1,13	0,84	1,02	1,10	0,99	0,92	1,23	DC1 domain-containing protein
254009_at	At4g26390	0,93	0,95	0,94	0,95	1,04	0,91	0,95	0,93	pyruvate kinase, putative
253958_at	At4g26400	1,02	1,30	1,49	1,26	1,18	1,16	1,06	1,76	zinc finger (C3HC4-type RING finger) family protein
253959_at	At4g26410	1,08	0,88	0,93	0,92	1,04	0,90	0,92	0,90	expressed protein
253960_at	At4g26420	1,02	1,11	0,82	0,97	1,14	0,96	1,07	1,07	S-adenosyl-L-methionine:carboxyl methyltransferase family protein
253969_at	At4g26430	1,29	1,43	1,74	1,32	0,80	1,01	1,01	1,15	COP9 signalosome subunit 6 / CSN subunit 6 (CSN6B)
253961_at	At4g26440	0,98	0,95	0,98	1,00	0,90	1,05	0,95	1,12	WRKY family transcription factor
253962_at	At4g26460	0,94	0,97	0,95	0,96	1,14	0,96	0,96	0,84	hypothetical protein
253963_at	At4g26470	1,09	1,30	0,98	1,15	1,22	0,92	1,04	1,07	calcium-binding EF hand family protein
253964_at	At4g26480	0,66	0,69	0,70	1,01	0,92	0,91	0,93	0,92	KH domain-containing protein
253965_at	At4g26490	0,78	0,71	0,97	1,19	1,38	0,93	1,01	1,22	expressed protein
253972_at	At4g26500	0,83	0,81	0,95	1,02	1,13	0,96	0,79	0,82	BolA-like family protein / Fe-S metabolism associated domain-containing pr
253970_at	At4g26510	1,11	0,92	1,05	1,08	0,92	0,97	0,95	0,91	uracil phosphoribosyltransferase / UMP pyrophosphorylase (UPT1)
253966_at	At4g26520	1,05	0,97	0,58	0,77	0,81	1,27	1,00	1,05	fructose-bisphosphate aldolase, cytoplasmic
253971_at	At4g26530	0,96	0,93	1,12	0,47	0,53	1,46	1,45	1,60	fructose-bisphosphate aldolase, putative
253974_at	At4g26540	1,03	0,96	1,04	0,84	0,91	0,95	1,03	0,81	protein kinase family protein
253967_at	At4g26550	0,89	0,98	1,12	1,09	1,16	0,89	0,82	0,91	expressed protein
253973_at	At4g26555	0,93	0,91	0,82	0,75	0,72	1,02	1,04	1,00	immunophilin / FKBP-type peptidyl-prolyl cis-trans isomerase family protein
253968_at	At4g26560	1,05	1,13	1,18	1,34	1,02	1,05	1,01	0,96	calcineurin B-like protein, putative
253982_at	At4g26570	1,04	0,84	0,74	1,42	1,17	0,94	0,92	0,76	calcineurin B-like protein 3 (CBL3)
253984_at	At4g26590	0,95	1,00	1,02	0,94	1,03	0,88	1,10	1,03	oligopeptide transporter OPT family protein
253975_at	At4g26600	1,34	1,34	1,23	0,93	0,79	1,02	1,14	1,15	nucleolar protein, putative
253976_at	At4g26610	0,98	1,04	1,07	1,09	1,05	0,77	0,86	0,86	protein kinase, putative
253980_at	At4g26620	0,93	0,99	0,99	1,28	1,01	1,10	0,85	0,89	sucrase-related
253977_at	At4g26630	1,04	0,90	1,00	0,76	0,86	1,38	1,52	1,71	expressed protein
253983_at	At4g26640	1,12	1,16	1,11	0,90	0,97	0,90	0,88	1,02	WRKY family transcription factor
253926_at	At4g26650	0,90	0,86	0,90	1,00	0,97	0,96	0,82	0,89	RNA recognition motif (RRM)-containing protein
253978_at	At4g26660	1,08	1,28	1,08	1,18	1,15	0,97	0,85	1,00	expressed protein

253981_at	At4g26670	0,77	0,78	0,82	0,79	0,69	0,89	0,80	0,85	mitochondrial import inner membrane translocase subunit Tim17/Tim22/Tim
253979_at	At4g26680	0,94	1,09	0,95	1,06	1,06	0,94	0,99	0,88	pentatricopeptide (PPR) repeat-containing protein
253925_at	At4g26690	0,75	0,68	0,64	1,02	1,10	0,95	0,93	0,86	glycerophosphoryl diester phosphodiesterase family protein
253956_at	At4g26700	0,74	0,76	0,91	0,99	1,02	0,99	1,09	1,21	fimbrin-like protein (FIM1)
253927_at	At4g26710	1,09	1,08	0,94	1,18	0,98	1,09	0,90	0,94	ATP synthase subunit H family protein
253928_at	At4g26720	1,30	1,36	1,47	0,87	0,83	1,15	1,02	1,04	serine/threonine protein phosphatase PP-X isozyme 1 (PPX1)
253929_s_at	At4g26730	1,07	1,08	1,07	1,07	1,06	0,90	1,07	0,97	expressed protein
253930_at	At4g26740	0,62	0,57	0,70	0,79	0,74	1,10	1,05	1,38	embryo-specific protein 1 (ATS1)
253953_at	At4g26750	0,72	0,69	1,01	1,03	1,05	0,97	0,87	1,09	hydroxyproline-rich glycoprotein family protein
253947_at	At4g26760	0,96	0,92	0,80	1,04	1,14	0,96	0,88	0,91	microtubule associated protein (MAP65/ASE1) family protein
253931_at	At4g26770	1,00	0,98	1,09	0,95	1,05	0,96	0,97	0,88	phosphatidate cytidylyltransferase, putative / CDP-diglyceride synthetase, p
253949_at	At4g26780	1,07	0,91	0,82	1,00	0,92	0,96	0,85	0,68	co-chaperone grpE family protein
253946_at	At4g26790	0,98	1,10	1,06	1,17	1,02	1,11	1,02	1,08	GDSL-motif lipase/hydrolase family protein
253932_at	At4g26800	0,94	0,91	1,02	0,91	1,05	1,06	0,90	0,90	pentatricopeptide (PPR) repeat-containing protein
253933_at	At4g26820	1,00	1,00	0,99	1,01	1,15	1,11	1,08	1,01	hypothetical protein
253934_at	At4g26830	1,04	0,92	0,87	0,99	1,04	0,96	0,93	0,82	glycosyl hydrolase family 17 protein
253952_at	At4g26840	1,30	1,21	1,13	1,02	0,96	1,04	1,05	1,04	ubiquitin-like protein (SMT3)
253922_at	At4g26850	1,09	1,22	1,03	0,90	0,70	1,26	1,25	1,15	expressed protein
253951_at	At4g26860	1,45	1,30	1,14	1,10	1,01	1,31	1,08	1,16	alanine racemase family protein
253935_at	At4g26870	1,05	0,97	1,17	1,19	1,11	0,88	0,94	0,96	aspartyl-tRNA synthetase, putative / aspartate--tRNA ligase, putative
253936_at	At4g26880	1,12	1,10	1,04	1,18	1,16	1,07	1,10	1,01	stigma-specific Stig1 family protein
253937_at	At4g26890	1,02	0,68	0,89	1,29	0,91	1,20	1,01	0,81	protein kinase family protein
253921_at	At4g26900	1,13	0,91	0,87	0,83	0,87	0,94	1,01	0,96	imidazole glycerol phosphate synthase hisHF, chloroplast / IGP synthase / I
253950_at	At4g26910	0,99	0,92	0,97	1,09	1,18	0,86	0,89	0,86	2-oxoacid dehydrogenase family protein
253938_at	At4g26920	0,93	1,05	1,07	0,82	1,23	1,12	1,03	0,82	hypothetical protein
253939_at	At4g26930	0,89	1,01	0,94	0,98	0,95	1,02	0,95	0,96	myb family transcription factor (MYB97)
253948_at	At4g26940	1,04	1,30	1,13	1,41	1,30	0,77	0,94	0,88	galactosyltransferase family protein
253940_at	At4g26950	0,98	0,95	1,03	1,01	1,07	0,99	1,08	1,08	expressed protein
253954_at	At4g26970	0,79	0,75	0,74	1,14	1,08	0,87	0,87	0,88	aconitate hydratase, cytoplasmic, putative / citrate hydro-lyase/aconitase, p
253941_at	At4g26980	1,32	1,36	1,68	1,00	1,20	1,15	0,94	0,88	expressed protein
253955_at	At4g27000	1,39	1,29	1,21	0,86	1,03	1,03	1,09	1,07	RNA-binding protein 45 (RBP45), putative
253942_at	At4g27010	1,10	1,00	0,90	0,76	0,87	0,86	1,00	0,90	expressed protein
253943_at	At4g27030	1,47	1,34	1,45	0,97	0,90	1,85	1,42	1,26	expressed protein
253944_at	At4g27040	1,21	1,07	1,28	1,00	0,95	1,05	1,10	1,27	expressed protein
253945_at	At4g27050	1,02	1,05	1,59	0,77	0,89	1,00	1,18	1,27	F-box family protein
253923_at	At4g27060	1,05	1,18	1,08	1,11	0,94	0,88	0,89	0,93	expressed protein
253898_s_at	At4g27070	0,80	0,82	0,94	0,81	0,84	0,93	0,87	0,86	tryptophan synthase, beta subunit 2 (TSB2)
253899_at	At4g27080	0,94	0,82	0,79	1,21	0,95	0,91	0,88	0,81	thioredoxin family protein
253901_at	At4g27090	1,47	1,24	1,12	1,28	1,13	1,09	1,05	0,95	60S ribosomal protein L14 (RPL14B)
253924_at	At4g27110	0,98	1,05	1,08	1,02	0,97	1,03	1,06	1,12	phytochelatin synthetase-related
253897_at	At4g27120	0,67	0,69	0,69	1,00	1,06	1,06	1,11	1,12	expressed protein
253900_at	At4g27130	0,85	0,77	0,74	0,80	0,86	1,00	0,93	0,89	eukaryotic translation initiation factor SUI1, putative
253904_at	At4g27140	0,54	0,27	0,13	0,28	0,37	0,87	0,80	0,66	2S seed storage protein 1 / 2S albumin storage protein / NWMU1-2S albur
253894_at	At4g27150	0,64	0,42	0,11	0,79	0,92	0,96	1,05	1,02	2S seed storage protein 2 / 2S albumin storage protein / NWMU2-2S albur
253895_at	At4g27160	0,72	0,59	0,16	0,66	0,52	0,91	0,96	0,81	2S seed storage protein 3 / 2S albumin storage protein / NWMU2-2S albur
253902_at	At4g27170	0,92	0,92	0,29	0,91	0,92	0,99	1,03	1,06	2S seed storage protein 4 / 2S albumin storage protein / NWMU2-2S albur

253903_at	At4g27180	1,03	1,06	1,08	1,02	0,92	1,08	1,25	1,41	kinesin-like protein B (KATB)
253896_at	At4g27190	1,04	1,05	0,97	0,88	1,16	1,04	0,90	1,05	disease resistance protein (NBS-LRR class), putative
253905_s_at	At4g27200	0,92	1,08	1,04	0,95	1,01	0,89	1,07	1,01	---
253906_at	At4g27220	0,99	0,97	1,07	1,12	0,99	0,98	1,02	0,89	disease resistance protein (NBS-LRR class), putative
253920_at	At4g27230	0,90	1,05	1,11	1,12	0,87	0,87	0,82	1,04	histone H2A, putative
253916_at	At4g27240	1,03	0,92	0,98	1,00	1,10	0,87	0,87	1,06	zinc finger (C2H2 type) family protein
253907_at	At4g27250	1,01	0,92	0,96	0,95	0,90	1,03	1,01	0,93	dihydroflavonol 4-reductase family / dihydrokaempferol 4-reductase family
253908_at	At4g27260	0,92	1,66	1,78	0,93	1,41	0,92	1,04	0,93	auxin-responsive GH3 family protein
253909_at	At4g27270	0,91	0,75	0,81	1,39	1,21	0,99	0,99	0,99	quinone reductase family protein
253915_at	At4g27280	1,10	1,17	1,38	1,09	1,02	1,18	1,50	1,71	calcium-binding EF hand family protein
253910_at	At4g27290	1,03	1,02	1,08	1,03	0,94	1,21	1,10	1,09	S-locus protein kinase, putative
253911_at	At4g27300	0,91	1,08	1,56	0,97	0,78	1,03	1,18	1,46	S-locus protein kinase, putative
253918_at	At4g27320	1,12	1,09	1,05	1,18	1,11	0,99	0,92	0,85	universal stress protein (USP) family protein
253862_at	At4g27330	1,07	1,03	0,96	0,92	0,96	0,99	1,00	0,81	sporocyteless (SPL)
253912_at	At4g27340	1,24	1,12	1,30	0,68	0,83	1,07	1,19	1,08	Met-10+ like family protein
253919_at	At4g27350	1,00	1,20	1,57	1,38	1,35	1,22	1,31	1,95	expressed protein
253913_at	At4g27370	1,04	1,12	1,34	0,91	0,82	1,07	1,33	1,22	myosin family protein
253917_at	At4g27380	1,19	0,94	0,75	1,16	0,94	1,17	0,96	0,80	expressed protein
253893_at	At4g27390	0,89	0,93	0,94	0,80	0,85	0,94	0,98	0,89	expressed protein
253914_at	At4g27400	1,14	1,33	1,31	1,64	1,59	0,96	1,01	0,74	late embryogenesis abundant protein-related / LEA protein-related
253872_at	At4g27410	0,66	1,33	1,00	0,78	0,81	0,82	1,29	1,31	no apical meristem (NAM) family protein (RD26)
253863_at	At4g27420	1,00	1,12	0,83	1,05	0,99	1,10	1,01	0,96	ABC transporter family protein
253876_at	At4g27430	0,91	0,93	1,21	0,88	0,87	0,86	0,99	1,39	COP1-interacting protein 7 (CIP7)
253877_at	At4g27435	1,21	1,06	0,84	1,01	0,89	0,93	1,01	0,79	expressed protein
253871_at	At4g27440	0,84	0,75	0,82	0,57	0,50	0,77	0,78	0,80	protochlorophyllide reductase B, chloroplast / PCR B / NADPH-protochlorop
253874_at	At4g27450	0,98	1,47	1,65	1,37	1,57	0,66	0,51	0,58	expressed protein
253864_at	At4g27460	0,66	0,79	0,98	0,90	0,73	0,71	0,89	0,89	CBS domain-containing protein
253865_at	At4g27470	0,99	1,38	1,21	0,85	1,05	1,02	1,02	0,94	zinc finger (C3HC4-type RING finger) family protein
253866_at	At4g27480	0,93	0,90	1,34	1,03	1,20	0,94	1,14	1,52	glycosyltransferase family 14 protein / core-2/I-branching enzyme family prc
253867_at	At4g27490	0,99	0,73	0,97	0,97	1,10	0,92	0,89	1,04	3' exoribonuclease family domain 1-containing protein
253868_at	At4g27500	0,86	0,73	0,92	0,97	0,95	1,02	1,09	1,24	expressed protein
253869_at	At4g27510	1,08	0,91	1,06	1,07	1,04	0,98	1,04	1,00	expressed protein
253875_at	At4g27520	0,72	0,72	0,61	0,94	0,99	1,12	0,90	0,91	plastocyanin-like domain-containing protein
253870_at	At4g27530	0,98	0,89	1,00	0,74	0,91	1,05	0,97	0,99	expressed protein
253873_at	At4g27540	0,73	0,88	1,00	1,03	0,92	0,87	0,87	0,82	prenylated rab acceptor (PRA1) protein-related
253878_at	At4g27550	0,97	1,02	1,10	1,07	1,03	1,02	1,14	1,05	alpha, alpha-trehalose-phosphate synthase, UDP-forming, putative / trehalc
253879_s_at	At4g27570	1,04	0,92	1,10	2,42	1,76	1,06	1,06	0,87	glycosyltransferase family protein
253831_at	At4g27580	0,98	0,87	0,90	0,95	1,13	1,06	0,95	1,07	expressed protein
253880_at	At4g27590	0,99	1,22	0,98	1,14	1,10	0,97	1,02	0,92	copper-binding protein-related
253858_at	At4g27600	1,11	1,12	1,26	0,64	0,68	1,12	1,19	1,08	pfkB-type carbohydrate kinase family protein
253892_at	At4g27620	0,74	0,62	0,88	0,75	0,84	0,95	0,82	0,86	expressed protein
262869_s_at	At4g27630	0,89	0,98	0,92	0,82	0,84	0,95	1,00	1,05	expressed protein
253881_at	At4g27640	1,22	1,13	1,07	0,94	0,77	0,92	0,85	0,80	importin beta-2 subunit family protein
253882_at	At4g27650	0,88	0,71	0,71	1,27	1,02	1,00	1,14	1,19	pelota (PEL1)
253830_at	At4g27652	0,70	0,56	0,66	1,06	0,79	0,97	1,22	0,95	expressed protein
253832_at	At4g27654	0,75	0,81	0,84	1,11	0,43	0,92	1,22	1,20	expressed protein

253859_at	At4g27657	0,97	0,72	1,09	1,47	0,80	0,94	1,00	1,13 expressed protein
253883_at	At4g27660	1,04	0,91	0,89	1,02	0,94	0,91	0,78	0,90 expressed protein
253884_at	At4g27670	1,19	1,33	1,12	0,99	1,30	1,06	1,08	1,38 25.3 kDa small heat shock protein, chloroplast precursor (HSP25.3-P)
253861_at	At4g27680	1,08	1,01	1,39	1,35	1,65	0,99	0,98	1,11 MSP1 protein, putative / intramitochondrial sorting protein, putative
253885_at	At4g27690	1,06	1,41	1,62	0,99	1,01	1,06	1,06	1,03 vacuolar protein sorting-associated protein 26, putative / VPS26, putative
253860_at	At4g27700	1,15	1,21	1,39	0,89	0,93	1,18	0,99	1,05 rhodanese-like domain-containing protein
253886_at	At4g27710	0,95	0,96	0,95	1,00	1,17	1,02	0,96	0,99 cytochrome P450 family protein
253891_at	At4g27720	0,75	0,71	0,80	1,20	0,99	0,97	0,94	0,94 expressed protein
253887_at	At4g27730	0,99	0,90	0,84	1,25	1,22	0,85	0,61	0,63 oligopeptide transporter OPT family protein
253888_at	At4g27750	0,88	0,83	0,94	0,85	1,20	0,90	0,90	0,87 expressed protein
253889_at	At4g27760	1,02	1,15	1,10	0,98	0,69	1,00	0,93	0,93 oxidoreductase, forever young (FEY3)
253840_at	At4g27780	0,89	0,86	0,86	0,96	1,28	0,96	1,04	1,14 acyl-CoA binding protein 2 (ACBP2)
253833_at	At4g27790	1,08	0,95	0,95	0,96	0,98	1,01	0,79	1,11 calcium-binding EF hand family protein
253834_at	At4g27800	0,70	0,74	0,82	0,61	0,74	1,08	1,25	1,38 protein phosphatase 2C PPH1 / PP2C PPH1 (PPH1)
253835_at	At4g27820	1,51	1,52	1,21	0,87	0,76	1,13	1,20	0,92 glycosyl hydrolase family 1 protein
253841_at	At4g27830	0,73	0,68	0,91	1,03	1,04	0,95	1,05	1,28 glycosyl hydrolase family 1 protein
253836_at	At4g27840	0,81	0,66	0,65	1,02	1,00	0,99	0,73	0,89 expressed protein
253837_at	At4g27850	1,04	1,25	1,01	1,04	1,08	1,02	0,90	1,13 proline-rich family protein
253842_at	At4g27860	0,74	0,67	0,68	1,21	0,93	0,82	0,91	1,24 integral membrane family protein
253838_at	At4g27880	0,99	1,09	0,95	0,93	0,95	1,00	0,97	1,18 seven in absentia (SINA) family protein
253839_at	At4g27890	0,97	0,99	0,97	1,19	1,19	1,19	0,93	0,93 nuclear movement family protein
253854_at	At4g27900	0,72	0,59	0,69	0,69	1,12	1,05	0,92	1,07 expressed protein
253843_at	At4g27910	0,93	0,98	1,27	1,03	0,95	1,03	1,24	1,10 PHD finger protein-related / SET domain-containing protein (TX4)
253844_at	At4g27930	0,98	1,06	0,93	0,90	1,02	1,04	0,93	0,94 hypothetical protein
253824_at	At4g27940	0,97	0,95	1,28	1,12	1,00	1,30	1,38	1,29 mitochondrial substrate carrier family protein
253826_s_at	At4g27960	1,04	0,93	0,93	1,16	1,08	0,96	0,99	0,95 ubiquitin-conjugating enzyme 10 (UBC10)
253828_at	At4g27970	1,14	1,19	1,30	1,57	1,63	1,10	1,18	0,89 C4-dicarboxylate transporter/malic acid transport family protein
253845_at	At4g27980	0,97	1,02	1,13	1,01	0,96	0,99	0,94	1,03 expressed protein
253857_at	At4g27990	0,84	0,80	0,77	0,97	1,00	0,94	0,78	0,75 YGGT family protein
253846_at	At4g28000	0,94	0,97	1,16	0,82	1,10	0,90	0,92	0,81 AAA-type ATPase family protein
253847_at	At4g28010	1,00	0,88	1,03	1,01	0,91	1,11	1,12	0,94 pentatricopeptide (PPR) repeat-containing protein
253848_at	At4g28020	1,10	1,22	1,13	0,94	0,87	1,02	1,06	0,93 expressed protein
253825_at	At4g28025	1,17	1,09	1,00	0,88	0,81	1,17	1,06	1,12 expressed protein
253823_at	At4g28030	1,04	0,91	1,00	0,75	0,87	0,92	0,93	0,93 GCN5-related N-acetyltransferase (GNAT) family protein
253829_at	At4g28040	1,15	1,29	1,68	0,74	1,15	0,87	0,95	1,04 nodulin MtN21 family protein
253855_at	At4g28050	0,83	0,87	0,79	1,08	1,10	0,73	0,67	0,74 senescence-associated protein, putative
253849_at	At4g28080	0,81	0,89	1,06	0,59	0,59	1,17	1,01	0,99 expressed protein
253827_at	At4g28085	0,81	0,70	0,69	0,98	1,00	0,84	0,83	1,00 expressed protein
253850_at	At4g28090	0,99	0,89	1,07	1,05	1,03	1,08	1,08	1,31 multi-copper oxidase type I family protein
253856_at	At4g28100	1,14	1,06	1,12	1,08	1,19	0,86	0,77	0,75 expressed protein
253851_at	At4g28110	0,97	0,99	1,04	0,92	1,09	1,00	1,04	0,93 myb family transcription factor (MYB41)
253852_at	At4g28120	0,97	0,99	1,13	1,02	1,01	0,93	0,94	0,94 diacylglycerol kinase accessory domain-containing protein
253853_at	At4g28130	0,97	1,05	0,95	1,06	1,06	0,95	0,92	1,04 diacylglycerol kinase accessory domain-containing protein
253799_at	At4g28140	0,88	0,94	0,96	2,71	2,84	1,43	1,07	1,02 AP2 domain-containing transcription factor, putative
253813_at	At4g28150	1,03	0,94	1,03	1,46	1,06	1,15	1,09	1,06 expressed protein
253800_at	At4g28160	1,14	0,98	1,10	1,12	1,13	1,06	1,01	0,96 hydroxyproline-rich glycoprotein family protein

253801_at	At4g28170	0,95	1,00	1,14	1,01	0,92	1,03	0,94	0,87	hypothetical protein
253802_at	At4g28180	1,00	1,03	0,99	0,73	1,03	1,00	0,92	0,74	expressed protein
253811_at	At4g28190	0,99	0,73	0,58	1,14	1,06	0,71	0,80	0,68	expressed protein
253803_at	At4g28200	1,21	1,08	1,09	1,03	0,90	1,08	1,01	0,99	expressed protein
253816_at	At4g28210	1,02	0,91	0,91	0,71	0,70	0,91	0,93	0,77	expressed protein
253810_at	At4g28220	0,79	0,78	0,79	0,83	0,92	0,93	0,92	0,89	NADH dehydrogenase-related
253804_at	At4g28230	1,11	1,02	0,87	2,03	1,51	0,99	0,87	0,92	expressed protein
253812_at	At4g28240	1,26	1,05	1,06	0,99	1,24	1,04	1,04	1,19	wound-responsive protein-related
253815_at	At4g28250	1,25	1,14	0,74	2,30	1,89	0,84	0,93	1,03	beta-expansin, putative (EXPB3)
253805_at	At4g28260	1,12	1,15	1,17	0,99	0,88	1,05	1,03	1,06	expressed protein
253806_at	At4g28270	1,90	1,76	1,42	0,91	0,81	1,17	1,26	1,38	zinc finger (C3HC4-type RING finger) family protein
253807_at	At4g28280	0,85	0,97	1,22	1,04	1,18	1,01	0,93	0,79	expressed protein
253814_at	At4g28290	2,60	2,30	1,82	2,04	1,80	1,04	1,39	1,40	expressed protein
253808_at	At4g28300	1,23	1,40	1,68	1,74	2,12	0,99	1,05	1,12	hydroxyproline-rich glycoprotein family protein
253817_at	At4g28310	1,15	1,12	0,97	1,49	1,27	0,86	0,97	0,97	expressed protein
253809_at	At4g28320	0,92	1,08	1,07	1,21	1,32	0,98	0,95	0,76	glycosyl hydrolase family 5 protein / cellulase family protein
253818_at	At4g28330	0,86	0,90	0,82	1,15	0,90	0,79	1,05	0,98	expressed protein
253819_at	At4g28350	1,02	0,86	0,98	0,86	0,77	1,07	0,95	1,12	lectin protein kinase family protein
253773_s_at	At4g28360	1,16	0,98	1,26	0,83	1,02	1,01	1,10	1,08	ribosomal protein L22 family protein
253820_at	At4g28370	0,79	1,05	1,11	0,96	1,13	0,92	1,02	1,36	zinc finger (C3HC4-type RING finger) family protein
253821_at	At4g28380	0,94	1,05	0,87	1,02	1,06	0,96	1,05	1,14	leucine-rich repeat family protein
253776_at	At4g28390	0,84	0,90	0,91	0,89	0,93	0,91	0,85	0,99	ADP, ATP carrier protein, mitochondrial, putative / ADP/ATP translocase, p
253772_at	At4g28395	0,89	1,08	0,94	0,93	1,17	1,04	0,92	1,01	lipid transfer protein, putative
253780_at	At4g28400	0,78	0,91	1,20	0,83	1,03	1,13	1,20	1,30	protein phosphatase 2C, putative / PP2C, putative
253822_at	At4g28410	0,93	0,98	1,21	1,32	1,39	1,05	1,13	0,80	aminotransferase-related
253795_at	At4g28420	1,01	1,11	1,07	1,01	0,87	1,01	1,07	1,02	aminotransferase, putative
253771_at	At4g28430	1,01	1,02	0,93	1,06	1,20	1,03	0,96	1,02	reticulon family protein
253775_at	At4g28440	1,38	1,20	0,98	1,15	1,04	1,10	0,98	0,94	DNA-binding protein-related
253777_at	At4g28450	1,24	0,96	1,02	0,94	1,10	0,94	0,93	0,91	transducin family protein / WD-40 repeat family protein
253796_at	At4g28460	1,05	1,51	1,08	0,83	1,07	1,20	1,23	1,09	hypothetical protein
253797_at	At4g28470	0,96	0,93	1,01	1,02	0,95	1,00	1,06	0,99	26S proteasome regulatory subunit, putative
253778_at	At4g28480	1,29	1,18	1,21	1,21	1,47	1,09	0,98	1,16	DNAJ heat shock family protein
253779_at	At4g28490	0,78	0,75	0,87	0,77	0,87	0,90	0,84	0,94	leucine-rich repeat transmembrane protein kinase, putative
253798_at	At4g28500	0,93	1,17	0,92	1,04	1,06	1,05	1,06	0,96	no apical meristem (NAM) family protein
253767_at	At4g28520	0,02	0,05	0,03	0,28	0,25	1,07	0,94	1,28	12S seed storage protein, putative / cruciferin, putative
253774_at	At4g28530	0,96	1,07	0,97	0,90	0,97	1,06	0,95	0,85	no apical meristem (NAM) family protein
253770_at	At4g28540	0,98	0,82	0,91	0,95	0,95	0,98	0,81	0,97	casein kinase, putative
253768_at	At4g28550	0,97	0,94	1,12	1,03	1,36	1,05	1,21	1,12	RabGAP/TBC domain-containing protein
253769_at	At4g28560	0,94	1,12	1,01	1,04	1,10	0,95	0,80	0,88	leucine-rich repeat family protein (fragment)
253789_at	At4g28570	0,84	0,87	0,88	0,97	0,93	1,06	1,00	1,27	alcohol oxidase-related
253781_at	At4g28580	0,94	1,05	1,03	0,97	1,16	1,02	1,04	1,05	magnesium transporter CorA-like family protein (MRS2-6)
253782_at	At4g28590	0,96	0,89	1,12	0,75	0,88	1,04	0,97	1,03	expressed protein
253783_at	At4g28600	0,95	0,81	1,04	1,01	1,07	1,04	0,95	0,89	calmodulin-binding protein
253784_at	At4g28610	1,13	1,28	1,12	0,94	1,02	1,19	1,17	1,41	myb family transcription factor, putative / phosphate starvation response reg
253785_s_at	At4g28620	0,97	1,00	0,87	0,87	1,02	0,91	0,99	1,00	ABC transporter family protein
253791_at	At4g28640	0,99	1,13	1,15	0,96	1,01	0,97	1,03	0,99	auxin-responsive protein / indoleacetic acid-induced protein 11 (IAA11)

253786_at	At4g28650	1,03	1,05	0,99	1,22	1,08	1,04	0,96	1,01 leucine-rich repeat transmembrane protein kinase, putative
253790_at	At4g28660	1,34	1,18	1,20	1,00	1,06	1,51	1,16	1,10 photosystem II reaction centre W (PsbW) family protein
253787_at	At4g28670	0,95	1,10	0,97	0,92	0,92	0,90	1,00	1,15 protein kinase family protein
253788_at	At4g28680	1,18	0,95	1,23	1,05	0,91	1,05	1,06	1,11 tyrosine decarboxylase, putative
253792_at	At4g28700	1,00	1,05	1,01	0,88	0,97	0,92	1,13	1,13 ammonium transporter, putative
253737_at	At4g28703	0,76	0,92	1,08	0,90	0,97	0,67	0,77	0,96 expressed protein
253740_at	At4g28706	0,58	0,57	0,52	0,83	0,92	0,87	0,99	0,94 pfkB-type carbohydrate kinase family protein
253793_at	At4g28710	0,81	0,71	0,86	0,99	0,74	0,87	1,01	1,04 myosin heavy chain, putative
253794_at	At4g28720	0,93	0,96	1,08	1,00	1,02	0,79	1,02	0,94 flavin-containing monooxygenase family protein / FMO family protein
253765_at	At4g28740	1,02	1,07	1,15	0,70	0,65	1,35	1,33	1,23 expressed protein
253738_at	At4g28750	0,99	0,94	0,96	0,84	0,93	0,97	0,95	0,94 photosystem I reaction center subunit IV, chloroplast, putative / PSI-E, puta
253739_at	At4g28760	0,80	1,03	1,70	0,91	1,15	0,84	1,05	1,28 expressed protein
253766_at	At4g28770	0,98	0,94	0,97	1,14	1,12	0,92	0,94	0,91 expressed protein
253736_at	At4g28780	1,34	1,17	1,03	1,37	1,06	1,14	0,88	0,85 GDSL-motif lipase/hydrolase family protein
253760_at	At4g28810	1,03	1,00	0,91	1,03	1,18	1,00	1,03	1,12 expressed protein
253761_at	At4g28820	1,04	1,02	1,08	1,03	1,26	1,08	0,95	1,00 zinc finger (HIT type) family protein
253762_at	At4g28830	1,25	1,33	1,54	0,95	1,00	1,14	1,09	1,08 expressed protein
253763_at	At4g28850	1,01	1,16	1,03	0,94	1,20	0,97	0,94	1,04 xyloglucan:xyloglucosyl transferase, putative / xyloglucan endotransglycosy
253764_s_at	At4g28860	1,13	1,27	1,83	1,01	0,85	1,06	0,95	1,05 casein kinase, putative
253741_at	At4g28890	0,79	0,85	0,83	1,12	1,20	0,87	1,07	1,10 zinc finger (C3HC4-type RING finger) family protein
253742_at	At4g28900	1,02	1,00	0,97	1,03	0,97	1,01	0,85	0,97 ---
253752_at	At4g28910	0,84	0,75	0,80	1,14	1,33	0,82	0,83	0,86 expressed protein
[truncated: 741,965 more chars]
