# Supplementary material for: Arabidopsis thaliana LSM proteins function in mRNA splicing and degradation
Source: Nucleic Acids Res. 2013 Apr 24;41(12):6232–49. doi: 10.1093/nar/gkt296 (PMC3695525; doi:10.1093/nar/gkt296)
Supplement: Supplementary Data [file supp_gkt296_nar-03286-a-2012-File012.pdf]

mutant *sad1/lsm5*

Transcript ID	stabilization fold	Gene Title
At5g62520	4,20	zinc finger protein-related
At1g80840	4,11	SIN-like family protein
At4g11280	3,81	disease resistance protein RPM1 (CC-NBS-LRR class), putative
At2g38470	3,61	S-locus protein kinase, putative
At1g30135	3,57	disease resistance protein (TIR-NBS-LRR class), putative
At5g03350	3,30	radical SAM domain-containing protein
At4g22780	2,91	pentatricopeptide (PPR) repeat-containing protein
At1g78070	2,90	transducin family protein / WD-40 repeat family protein
At5g10100	2,88	auxin-responsive protein / indoleacetic acid-induced protein 7 (IAA7)
At1g24330	2,86	expressed protein
At3g54000	2,83	expressed protein
At2g32150	2,73	leucine-rich repeat protein kinase, putative
At1g43800	2,64	exonuclease family protein
At1g02390	2,63	mitogen-activated protein kinase kinase (MAPKK), putative (MKK8)
At2g14610	2,58	zinc finger (B-box type) family protein
At5g62470	2,57	hypothetical protein
At4g31800	2,56	calmodulin-2/3/5 (CAM3)
At3g27540	2,54	mitochondrial transcription termination factor family protein / mTERF family protein
At5g62570	2,54	SET domain-containing protein
At2g34600	2,53	autophagy 8e (APG8e)
At5g45340	2,51	actin-depolymerizing factor 4 (ADF4)
At4g36410	2,45	pentatricopeptide (PPR) repeat-containing protein
At4g28520	2,43	phototropic-responsive NPH3 family protein
At5g25280	2,41	cytochrome P450 family protein
At2g26190	2,37	expressed protein
At5g63770	2,30	omega-3 fatty acid desaturase, endoplasmic reticulum (FAD3)
At4g32020	2,30	UMUC-like DNA repair family protein
At1g05510	2,26	protein kinase family protein
At1g17380	2,21	protein kinase family protein
At1g69480	2,16	PAZ domain-containing protein / piwi domain-containing protein
At3g62260	2,16	DGCR14-related
At4g18010	2,14	pectinesterase family protein
At5g13700	2,13	multi-copper oxidase type I family protein
At2g24100	2,13	aldehyde oxidase, putative
At2g35715	2,10	homeobox-leucine zipper protein 2 (HAT2) / HD-ZIP protein 2
At1g63090	2,09	histone deacetylase-related / HD-related
At1g74430	2,09	succinyl-CoA ligase (GDP-forming) beta-chain, mitochondrial, putative / succinyl-CoA synthetase
At4g34410	2,08	membrane protein, putative
At1g61660	2,04	peptide chain release factor, putative
At4g23060	2,04	MATE efflux family protein
At1g66090	2,04	metal-transporting P-type ATPase, putative (PAA1)
At5g22920	2,04	40S ribosomal protein S4 (RPS4A)
At1g49840	2,04	chloroplast outer membrane protein, putative
At5g56870	2,02	serine/threonine protein kinase, putative
At1g53870	2,01	protein kinase family protein
At4g17230	2,01	homeobox protein SHOOT MERISTEMLESS (STM)
At1g52890	2,01	cinnamyl-alcohol dehydrogenase family / CAD family
At4g27410	2,01	expressed protein
At5g26920	2,00	glycosyl hydrolase family 17 protein
At4g25490	2,00	expressed protein
At5g41100	2,00	leucine-rich repeat family protein
At3g23250	1,99	member of the R2R3 factor gene family.
At3g16350	1,97	expressed protein
At1g15740	1,96	transferase family protein
At3g13750	1,95	transmembrane protein, putative
At5g63790	1,94	phosphatidate cytidylyltransferase family protein
At3g48100	1,93	serine carboxypeptidase S10 family protein
At5g67480	1,93	peptidase M3 family protein / thimet oligopeptidase family protein
At4g24380	1,93	10-formyltetrahydrofolate biosynthetic process, folic acid and derivative biosynthetic process
At1g75230	1,93	expressed protein
At2g01670	1,92	syntaxin 121 (SYP121) / syntaxin-related protein (SYR1)
At1g53885	1,91	Dof-type zinc finger domain-containing protein
At1g19960	1,91	DC1 domain-containing protein
At3g58990	1,91	hypothetical protein
At1g16670	1,89	calmodulin-domain protein kinase isoform 9 (CPK9)
At3g11840	1,89	Encodes a U-box-domain-containing E3 ubiquitin ligase that acts as a negative regulator of PAMP
At1g28260	1,89	PUR alpha-1 protein
At4g19230	1,88	MATE efflux family protein
At4g22980	1,88	hypothetical protein
At3g12710	1,88	synbindin, putative
At4g39890	1,88	pseudogene, F-box protein (SKP1 interacting partner 3-related)
At2g18050	1,87	serine carboxypeptidase S10 family protein

At1g01720	1,87	expressed protein
At3g48360	1,86	auxin-responsive family protein
At2g01420	1,85	DNA-directed RNA polymerase, mitochondrial (RPOMT)
At5g54510	1,83	universal stress protein (USP) family protein
At3g19200	1,83	rac GTPase activating protein, putative
At3g15760	1,82	cyanate lyase family
At1g61065	1,82	expressed protein
At5g52750	1,81	protein kinase, putative
At1g61340	1,81	expressed protein
At2g25250	1,80	expressed protein
At2g03240	1,80	short-chain dehydrogenase/reductase (SDR) family protein
At1g60140	1,80	SGS domain-containing protein
At2g23320	1,80	C2 domain-containing protein
At4g27260	1,80	F-box family protein
At1g70420	1,80	cation exchanger, putative (CAX10)
At5g41810	1,78	fringe-related protein
At3g46930	1,78	calmodulin, putative
At1g15670	1,78	Galactose oxidase/kelch repeat superfamily protein
At2g05160	1,77	senescence-associated family protein
At5g65140	1,77	F-box family protein
At3g55980	1,76	expressed protein
At3g27210	1,76	pathogenesis-related thaumatin family protein
At1g43160	1,76	pseudo-response regulator 2 (APRR2) (TOC2)
At3g11410	1,75	expressed protein
At1g15045	1,75	cytosine methyltransferase, putative
At1g56220	1,75	cysteine proteinase inhibitor-related
At5g61900	1,75	DNAJ heat shock N-terminal domain-containing protein
At1g74590	1,74	GTP-binding protein-related
At1g18010	1,73	aspartate aminotransferase, chloroplast / transaminase A (ASP5) (AAT1)
At2g35710	1,73	GATA transcription factor 3, putative (GATA-3)
At5g24590	1,73	phototropic-responsive NPH3 family protein
At1g22710	1,73	acyl-activating enzyme 12 (AAE12)
At3g61160	1,73	hypothetical protein
At5g10550	1,73	gamma interferon responsive lysosomal thiol reductase family protein / GILT family protein
At2g39400	1,73	matrix-localized MAR DNA-binding protein-related
At5g56880	1,72	bacterial transferase hexapeptide repeat-containing protein
At5g14420	1,72	disease resistance protein (NBS-LRR class), putative
At5g41080	1,72	expressed protein
At3g19680	1,72	AP2 domain-containing transcription factor, putative
At5g58720	1,71	dihydrolipoamide dehydrogenase 1, mitochondrial / lipoamide dehydrogenase 1 (MTLPD1)
At1g01140	1,71	expressed protein
At1g63030	1,71	cysteine proteinase, putative
At3g01830	1,71	DegP2 protease (DEGP2)
At4g36550	1,70	NADH-ubiquinone oxidoreductase chain 3, putative
At2g36750	1,70	eukaryotic translation initiation factor 3 subunit 10 / eIF-3 theta / eIF3a (TIF3A1)
At3g49530	1,70	ribitol kinase, putative
At2g38790	1,70	serine/threonine protein phosphatase 2A (PP2A) regulatory subunit B’, putative
At1g51090	1,70	outer envelope membrane protein, putative
At1g70740	1,70	protein kinase family protein
At5g10120	1,69	transferase family protein
At5g59490	1,69	Haloacid dehalogenase-like hydrolase (HAD) superfamily protein
At3g17770	1,69	Ras-related GTP-binding family protein
At1g28330	1,69	short-chain dehydrogenase/reductase (SDR) family protein
At2g40270	1,68	cyclin, putative
At5g50760	1,68	mitochondrial substrate carrier family protein
At3g19540	1,68	kinesin motor family protein (NACK2)
At3g15450	1,68	phospholipase D gamma 2 / PLD gamma 2 (PLDGAMMA2)
At4g35985	1,68	Senescence/dehydration-associated protein-related
At5g45310	1,68	zinc finger (C3HC4-type RING finger) family protein
At1g10640	1,68	DJ-1 family protein / protease-related
At1g34180	1,68	WRKY family transcription factor
At5g06300	1,67	SNF2 domain-containing protein / helicase domain-containing protein
At5g48900	1,67	peptidyl-prolyl cis-trans isomerase / cyclophilin (CYP2) / rotamase
At1g32450	1,67	expressed protein
At3g15500	1,67	ribosomal protein S7 family protein
At4g17615	1,67	clathrin coat assembly protein, putative
At1g73800	1,67	Snf1-related protein kinase (KIN10) (SKIN10)
At1g77210	1,67	pentatricopeptide (PPR) repeat-containing protein
At1g69260	1,67	expressed protein
At1g69890	1,66	WRKY family transcription factor
At3g06500	1,66	kelch repeat-containing F-box family protein
At4g35180	1,66	COP1-interacting protein-related
At5g65660	1,66	ethylene-responsive protein, putative
At3g06070	1,65	expressed protein
At2g01620	1,65	glycosyl transferase family 2 protein

At1g08920	1,65	peroxidase, putative
At4g35480	1,65	sulfate transporter (ST1)
At1g14040	1,64	dehydration-responsive family protein
At2g26980	1,64	homeodomain transcription factor (KNAT7)
At3g14440	1,63	hesB-like domain-containing protein
At4g18880	1,63	expressed protein
At3g23430	1,63	expressed protein
At2g29320	1,63	pathogenesis-related thaumatin family protein
At2g23030	1,63	zinc finger (C2H2 type) family protein (ZAT10) / salt-tolerance zinc finger protein (STZ)
At1g70590	1,63	kelch repeat-containing protein / serine/threonine phosphoesterase family protein
At3g62550	1,62	Adenine nucleotide alpha hydrolases-like superfamily protein
At1g68580	1,62	no apical meristem (NAM) family protein
At1g79700	1,62	long-chain-fatty-acid--CoA ligase, putative / long-chain acyl-CoA synthetase, putative
At1g02310	1,61	Glycosyl hydrolase superfamily protein
At1g74290	1,61	hypothetical protein
At1g64590	1,61	MADS-box protein (AGL79)
At2g22190	1,61	kinesin motor family protein
At2g42760	1,61	no apical meristem (NAM) family protein
At4g16780	1,61	expressed protein
At5g50570	1,61	transcription factor, putative / E2F-like repressor E2L2 (E2L2)
At5g01790	1,60	zinc finger (C3HC4-type RING finger) family protein
At2g39660	1,59	protease inhibitor/seed storage/lipid transfer protein (LTP) family protein
At1g31650	1,59	wound-responsive protein-related
At3g50260	1,59	cysteine synthase / O-acetylserine (thiol)-lyase / O-acetylserine sulfhydrylase (OAS1)
At4g02200	1,59	laccase family protein / diphenol oxidase family protein
At2g05940	1,59	expressed protein
At2g25460	1,59	syntaxin 42 (SYP42) / TLG2b
At3g54950	1,59	C2 domain-containing protein
At2g02310	1,59	dual specificity protein phosphatase family protein
At1g76640	1,59	auxin-responsive protein / indoleacetic acid-induced protein 20 (IAA20)
At5g01300	1,59	anion exchange family protein
At3g46110	1,58	ATP-dependent DNA helicase, putative
At5g42050	1,58	pentatricopeptide (PPR) repeat-containing protein
At1g28050	1,58	B-box type zinc finger protein with CCT domain
At4g11360	1,58	hypothetical protein
At4g31940	1,58	myb family transcription factor (KAN2)
At2g30520	1,58	RNA polymerase sigma subunit SigD (sigD) / sigma-like factor (SIG4)
At5g22570	1,58	epsin N-terminal homology (ENTH) domain-containing protein / clathrin assembly protein-related
At3g17710	1,58	F-box and associated interaction domains-containing protein
At5g46050	1,58	potassium channel tetramerisation domain-containing protein
At1g47400	1,58	PRLI-interacting factor L, putative
At1g72100	1,57	expressed protein
At1g13940	1,57	expressed protein
At5g41410	1,57	homeodomain protein required for ovule identity
At1g14370	1,57	expressed protein
At1g70290	1,57	hypothetical protein
At1g75800	1,57	protein kinase family protein
At2g03550	1,57	pyridine nucleotide-disulphide oxidoreductase family protein
At1g67970	1,57	histone acetyltransferase (GCN5)
At3g20340	1,57	nascent polypeptide-associated complex (NAC) domain-containing protein
At1g03090	1,57	hypothetical protein
At3g59060	1,56	Ku70-binding family protein
At5g16030	1,56	choline transporter-related
At4g32030	1,56	cell division cycle protein 48-related / CDC48-related
At1g20823	1,55	zinc finger (C2H2 type) family protein
At5g14700	1,55	expressed protein
At1g12610	1,55	Encodes a member of the DREB subfamily A-1 of ERF/AP2 transcription factor family (DDF1).
At1g03790	1,55	F-box protein-related
At2g40170	1,55	chloroplast thylakoid lumen protein
At5g18600	1,55	cysteine synthase, chloroplast / O-acetylserine (thiol)-lyase / O-acetylserine sulfhydrylase / cpACS1
At1g34110	1,55	basic helix-loop-helix (bHLH) protein (RAP-1)
At1g22985	1,55	hypothetical protein
At3g07310	1,55	expressed protein
At3g44990	1,55	UDP-glucoronosyl/UDP-glucosyl transferase family protein
At3g28740	1,55	exopolygalacturonase / galacturan 1,4-alpha-galacturonidase / pectinase
At2g41140	1,55	XH/XS domain-containing protein / XS zinc finger domain-containing protein
At3g14050	1,55	acetolactate synthase small subunit, putative
At5g25630	1,55	acyl-CoA binding family protein
At1g72920	1,55	Rac-like GTP-binding protein (ARAC8)
At1g11210	1,54	pentatricopeptide (PPR) repeat-containing protein
At1g60800	1,54	esterase/lipase/thioesterase family protein
At1g30810	1,54	myb family transcription factor
At5g15130	1,54	DNA-binding S1FA family protein
At4g30350	1,54	expressed protein
At3g57190	1,54	endo-1,4-beta-glucanase, putative / cellulase, putative

At1g21000	1,54	expressed protein
At5g53890	1,54	expressed protein
At5g18630	1,54	zinc finger (C2H2 type) family protein
At1g28370	1,54	thioredoxin family protein
At2g25200	1,54	pale cress protein (PAC)
At2g22880	1,54	protein kinase family protein
At1g68050	1,54	pectinesterase family protein
At1g59740	1,54	expressed protein
At5g35370	1,54	serine/threonine protein kinase, putative
At1g33720	1,53	disease resistance protein (TIR-NBS-LRR class), putative
At5g12230	1,53	8-oxoguanine-DNA glycosylase (OGG1)
At3g57530	1,53	cold-shock DNA-binding family protein / glycine-rich protein (GRP2)
At5g13220	1,53	expressed protein
At1g78460	1,53	hypothetical protein
At2g48130	1,53	zinc finger (GATA type) family protein
At5g42440	1,53	60S ribosomal protein L10A (RPL10aB)
At1g20650	1,52	ubiquitin-conjugating enzyme 11 (UBC11)
At3g28850	1,52	60S ribosomal protein L24 (RPL24B)
At2g31980	1,52	F-box family protein
At3g60140	1,52	hypothetical protein
At1g73480	1,52	expansin-related
At4g05200	1,52	plastocyanin-like domain-containing protein
At5g59520	1,52	PHD finger family protein
At4g18950	1,52	citrate synthase, mitochondrial, putative
At1g78280	1,52	myb family transcription factor
At3g17250	1,52	expressed protein
At4g22360	1,52	no apical meristem (NAM) family protein
At3g13690	1,52	kelch repeat-containing F-box family protein
At1g80450	1,51	expressed protein
At3g25610	1,51	expressed protein
At1g73760	1,51	ARF GAP-like zinc finger-containing protein ZiGA4 (ZIGA4)
At3g21760	1,51	acyl-protein thioesterase-related
At3g18710	1,51	DNA replication licensing factor, putative
At1g70530	1,51	expressed protein
At1g67530	1,51	expressed protein
At1g77460	1,51	chaperonin (CPN60) (HSP60)
At4g39770	1,51	haloacid dehalogenase-like hydrolase (HAD) superfamily protein
At4g36040	1,51	expressed protein
At2g45810	1,50	agenet domain-containing protein
At3g26910	1,50	zinc finger (C2H2 type) family protein
At1g69850	1,50	xanthine/uracil/vitamin C permease family protein
At1g07135	1,50	peroxiredoxin type 2, putative
At5g59960	1,50	expressed protein
At4g14480	1,50	glycosyl hydrolase family 3 protein
At5g47240	1,50	hypothetical protein
At2g07701	1,50	calmodulin-binding protein
At5g14880	1,50	Potassium transporter family protein
At1g77450	1,50	nitrate-responsive NOI protein, putative
At4g27450	1,50	aluminium induced protein with YGL and LRDR motifs
At3g21060	1,50	expressed protein
At2g30020	1,50	splicing factor PWI domain-containing protein
At4g18210	1,50	phosphoinositide phosphatase family protein
At3g24520	1,49	hypothetical protein
At2g30500	1,49	cytochrome P450 family protein
At3g19580	1,49	expressed protein
At1g77000	1,49	expressed protein
At1g14340	1,49	expressed protein
At1g75860	1,49	hypothetical protein
At5g64900	1,49	expressed protein
At3g06380	1,49	expressed protein
At1g19180	1,49	expressed protein
At1g22930	1,49	zinc finger protein CONSTANS-LIKE 2 (COL2)
At1g11670	1,49	late embryogenesis abundant group 1 domain-containing protein / LEA group 1 domain
At3g47340	1,48	PHD finger family protein
At4g31875	1,48	disease resistance protein (CC-NBS-LRR class), putative
At2g31945	1,48	expressed protein
At1g17810	1,48	zinc finger (GATA type) family protein
At2g18370	1,48	WD-40 repeat protein family
At3g23920	1,48	superman protein (SUP) / zinc finger (C2H2 type) family protein
At4g24050	1,48	hypothetical protein
At1g03380	1,48	expressed protein
At5g54170	1,48	glycosyl hydrolase family 38 protein
At4g17970	1,48	acyl-(acyl carrier protein) thioesterase/acyl-ACP thioesterase/oleoyl-(acyl-carrier protein) hydrolase
At4g20310	1,48	hypothetical protein
At5g39050	1,48	expressed protein

At2g41640	1,48	expressed protein
At1g59910	1,48	cyclic nucleotide-regulated ion channel / cyclic nucleotide-gated channel (CNGC6)
At2g22330	1,47	kelch repeat-containing F-box family protein
At2g02370	1,47	CBL-interacting protein kinase 21, putative (CIPK21)
At4g11650	1,47	heavy-metal-associated domain-containing protein
At2g43330	1,47	expressed protein
At4g30390	1,47	glutathione S-transferase, putative
At5g45110	1,47	sucrose transporter, putative / sucrose-proton symporter, putative
At2g22770	1,47	cytochrome P450 71A16, putative (CYP71A16)
At4g20140	1,47	serine/threonine protein phosphatase 2A (PP2A) 55 kDa regulatory subunit B
At3g03990	1,47	profilin 5 (PRO5) (PRF3)
At2g25900	1,47	protein phosphatase 2C-related / PP2C-related
At5g05140	1,47	F-box family protein
At3g03210	1,46	expressed protein
At1g25500	1,46	cleavage stimulation factor, putative
At2g46550	1,46	expressed protein
At2g41230	1,46	17.8 kDa class I heat shock protein (HSP17.8-CI)
At2g29300	1,46	expressed protein
At5g04100	1,46	trypsin inhibitor, putative
At1g75810	1,46	expressed protein
At1g27770	1,46	calcium-dependent protein kinase, putative / CDPK, putative
At1g27690	1,46	hypothetical protein
At2g43060	1,46	expressed protein
At5g46710	1,46	PLATZ transcription factor family protein
At1g69790	1,46	protein serine/threonine kinase activity
At5g66070	1,46	GDP-mannose pyrophosphorylase, putative
At1g68340	1,46	methylmalonate-semialdehyde dehydrogenase, putative
At2g21080	1,46	zinc finger (C3HC4-type RING finger) family protein
At1g22370	1,46	arginyl-tRNA synthetase, putative / arginine--tRNA ligase, putative
At5g45690	1,45	expressed protein
At1g14550	1,45	transporter-related
At3g12250	1,45	gypsy-like retrotransposon family
At5g11070	1,45	pseudogene, zinc knuckle (CCHC type) protein family
At4g38400	1,45	expressed protein
At5g40420	1,45	nitrogen fixation NifU-like family protein
At2g02450	1,45	zinc finger (C3HC4-type RING finger) family protein
At3g21070	1,45	proline-rich family protein
At2g24740	1,45	NAD-dependent epimerase/dehydratase family protein
At4g24110	1,45	esterase/lipase/thioesterase family protein
At3g15630	1,45	aspartyl protease family protein
At5g64240	1,45	cysteine protease inhibitor family protein / cystatin family protein
At2g30070	1,45	CTP synthase, putative / UTP--ammonia ligase, putative
At1g19050	1,45	aspartyl protease family protein
At5g02290	1,45	expressed protein
At5g27920	1,45	zinc finger (C2H2 type) family protein
At5g53570	1,45	40S ribosomal protein S25, putative
At2g02710	1,45	U5 small nuclear ribonucleoprotein helicase, putative
At1g73540	1,45	expressed protein
At3g19240	1,45	hypothetical protein
At4g10500	1,45	hypothetical protein
At1g03610	1,45	disease resistance protein (TIR-NBS-LRR class), putative
At5g02580	1,44	protein disulfide isomerase, putative
At2g22350	1,44	expressed protein
At1g08630	1,44	oxidoreductase, 2OG-Fe(II) oxygenase family protein
At2g15830	1,44	cinnamyl-alcohol dehydrogenase (CAD)
At1g08720	1,44	hypothetical protein
At2g38940	1,44	CDP-diacylglycerol-inositol 3-phosphatidyltransferase, putative/phosphatidylinositol synthase
At1g07280	1,44	plastocyanin-like domain-containing protein
At2g28930	1,44	zinc finger (C3HC4-type RING finger) family protein
At1g32120	1,44	SEC14 cytosolic factor, putative
At5g67440	1,44	hypothetical protein
At1g68190	1,44	expressed protein
At4g36540	1,44	mob1/phocein family protein
At1g30040	1,44	expressed protein
At1g24430	1,44	bZIP transcription factor family protein
At1g49470	1,44	expressed protein
At2g36950	1,44	expressed protein
At5g63130	1,44	tetrahydrofolate dehydrogenase/cyclohydrolase, putative
At3g28600	1,43	DC1 domain-containing protein
At5g16830	1,43	acyl carrier protein, chloroplast, putative / ACP, putative
At1g23080	1,43	expressed protein
At5g62090	1,43	S-adenosyl-L-methionine:carboxyl methyltransferase family protein
At4g28460	1,43	expressed protein
At2g36640	1,43	syntaxin-related family protein
At1g75710	1,43	patatin, putative

At5g44120	1,43	40S ribosomal protein S25 (RPS25A)
At2g41710	1,43	transporter-related
At5g63450	1,43	40S ribosomal protein S11 (RPS11B)
At1g26420	1,43	SKP1 interacting partner 6 (SKIP6)
At1g01490	1,43	F-box family protein
At4g01410	1,43	expansin, putative (EXP2)
At3g11370	1,43	pathogenesis-related protein, putative
At1g72950	1,43	expressed protein
At1g43640	1,42	calmodulin-binding family protein
At1g55270	1,42	dynamin-like protein E (DL1E)
At3g61060	1,42	transcription activation domain-interacting protein-related
At4g25620	1,42	leucine-rich repeat transmembrane protein kinase, putative
At3g43430	1,42	beta-fructosidase, putative / beta-fructofuranosidase, putative
At2g36690	1,42	expressed protein
At1g73500	1,42	dihydrolipoamide S-acetyltransferase (LTA2)
At5g66050	1,42	expressed protein
At5g61590	1,42	expressed protein
At3g47040	1,42	expressed protein
At3g09420	1,42	Ras-related GTP-binding protein, putative
At5g44770	1,42	magnesium transporter CorA-like family protein
At5g48850	1,42	expressed protein
At2g36650	1,42	alanine-glyoxylate aminotransferase, putative/beta-alanine-pyruvate aminotransferase, putative/AG
At1g78340	1,42	ribosomal protein L22 family protein
At4g20000	1,42	hypothetical protein
At2g39570	1,42	Sas10/U3 ribonucleoprotein (Utp) family protein
At5g12440	1,42	major latex protein-related / MLP-related
At3g07350	1,42	hypothetical protein
At5g26200	1,42	GTP-binding family protein
At5g62710	1,42	transporter, putative
At4g33666	1,42	translocon-associated protein beta (TRAPB) family protein
At3g51400	1,42	pentatricopeptide (PPR) repeat-containing protein
At3g54770	1,41	pentatricopeptide (PPR) repeat-containing protein
At1g72450	1,41	dolichyl-phosphate beta-D-mannosyltransferase, putative / dolichol-phosphate mannosyltransferase
At2g15120	1,41	expressed protein
At2g14250	1,41	hydroxyproline-rich glycoprotein family protein
At4g15680	1,41	pectate lyase family protein
At1g09575	1,41	fip1 motif-containing protein
At3g50410	1,41	F-box family protein
At2g42830	1,41	trehalose-6-phosphate phosphatase, putative
At2g16680	1,41	expressed protein
At5g26230	1,41	zinc finger (Ran-binding) family protein
At4g33050	1,41	hypothetical protein
At3g16150	1,41	inositol monophosphatase family protein
At3g54810	1,41	auxin-responsive family protein
At3g02340	1,41	RING/U-box superfamily protein
At3g58590	1,41	26S proteasome regulatory subunit, putative
At5g11610	1,41	40S ribosomal protein S28 (RPS28C)
At4g34150	1,41	galactinol synthase, putative
At1g23870	1,41	expressed protein
At3g60530	1,41	cathepsin-related
At1g04790	1,41	RING/U-box superfamily protein
At3g13980	1,41	expressed protein
At3g51950	1,41	zinc finger (C3HC4-type RING finger) family protein
At1g21410	1,41	expressed protein
At2g29310	1,41	expressed protein
At1g21090	1,41	glycosyl transferase family 8 protein
At5g52120	1,41	ubiquitin fusion degradation UFD1 family protein
At5g57630	1,41	expressed protein
At3g16520	1,41	hypothetical protein
At1g49180	1,41	scarecrow-like transcription factor 14 (SCL14)
At1g76900	1,41	WD-40 repeat family protein / phytochrome A-related
At5g66330	1,41	cytochrome P450 family protein
At2g03890	1,40	Phosphoinositide kinase which undergo autophosphorylation and phosphorylate serine/threonine
At3g54320	1,40	ubiquitin activating enzyme 1 (UBA1)
At1g55110	1,40	hydroxyproline-rich glycoprotein family protein
At5g52430	1,40	phospholipase/carboxylesterase family protein
At4g14720	1,40	expressed protein
At4g31550	1,40	DC1 domain-containing protein
At2g27690	1,40	heat shock cognate 70 kDa protein 3 (HSC70-3) (HSP70-3)
At3g06490	1,40	cyclin, putative
At4g20110	1,40	protein kinase family protein
At3g52800	1,40	monosaccharide transporter, putative
At5g57050	1,40	peptidoglycan-binding LysM domain-containing protein
At5g07330	1,40	leucine-rich repeat transmembrane protein kinase, putative
At5g53750	1,40	expressed protein

At5g57360	1,40	protein kinase, putative
At1g22810	1,40	protein phosphatase 2C, putative / PP2C, putative
At3g28910	1,40	origin recognition complex subunit 6 family protein (ORC6)
At1g01240	1,40	FAD-binding domain-containing protein / cytokinin oxidase family protein
At5g65640	1,40	transcriptional factor B3 family protein
At4g11300	1,40	zinc finger (GATA type) family protein
At5g27520	1,40	ferredoxin--NADP(+) reductase, putative / adrenodoxin reductase, putative
At2g39830	1,40	2-on-2 hemoglobin (GLB3)
At3g03750	1,40	glycine-rich protein
At1g20100	1,40	metallo-beta-lactamase family protein
At4g01330	1,39	pentatricopeptide (PPR) repeat-containing protein
At1g74330	1,39	sucrose transporter / sucrose-proton symporter (SUC1)
At3g57795	1,39	60S ribosomal protein L37a (RPL37aB)
At4g31730	1,39	transcriptional factor B3 family protein / auxin-responsive factor AUX/IAA-related
At4g29100	1,39	dihydropterin pyrophosphokinase, putative / dihydropteroate synthase, putative / DHPS, putative
At1g28360	1,39	agenet domain-containing protein
At5g52420	1,39	expressed protein
At3g51000	1,39	heavy-metal-associated domain-containing protein
At1g18460	1,39	pseudo-response regulator, putative / timing of CAB expression 1-like protein, putative
At1g68140	1,39	pseudogene, CHP-rich zinc finger protein, putative
At3g62860	1,39	alpha/beta-Hydrolases superfamily protein
At1g64390	1,39	2,3-diketo-5-methylthio-1-phosphopentane phosphatase family
At5g55700	1,39	disease resistance protein (CC-NBS-LRR class), putative
At2g15580	1,39	acyl-activating enzyme 14 (AAE14)
At5g50770	1,39	vacuoleless1 (VCL1)
At4g19660	1,39	plasma membrane intrinsic protein 1A (PIP1A) / aquaporin PIP1.1 (PIP1.1) (AQ1)
At2g16390	1,39	thioesterase family protein
At2g39250	1,39	DEAD box RNA helicase, putative
At3g03470	1,39	member of CYP89A
At4g27470	1,39	GCN5-related N-acetyltransferase (GNAT) family protein
At1g49000	1,39	expressed protein
At1g55280	1,39	disease resistance protein (TIR-NBS-LRR class), putative
At3g14910	1,39	calcium-transporting ATPase, plasma membrane-type, putative/Ca2+-ATPase, putative ACA10
At1g80160	1,39	adenylate kinase, chloroplast, putative / ATP-AMP transphosphorylase, putative
At5g20120	1,38	transferase family protein
At5g66690	1,38	expressed protein
At5g51990	1,38	expressed protein
At3g03190	1,38	calmodulin-binding family protein
At1g53090	1,38	60S ribosomal protein L22-2 (RPL22B)
At5g06610	1,38	protein of unknown function
At5g34900	1,38	expressed protein
At2g18890	1,38	purine permease-related
At1g01650	1,38	hypothetical protein
At5g24030	1,38	expressed protein
At4g20380	1,38	beta-expansin, putative (EXPB1)
At5g01030	1,38	hypothetical protein
At1g27120	1,38	DNAJ heat shock N-terminal domain-containing protein
At4g37890	1,38	betaine-aldehyde dehydrogenase, putative
At1g08230	1,38	5’ nucleotidase family protein
At5g39670	1,38	aspartate/glutamate/uridylate kinase family protein
At4g02210	1,38	expressed protein
At4g16970	1,38	proline-rich family protein
At4g15475	1,38	expressed protein
At1g66480	1,38	expressed protein
At1g42990	1,38	basic helix-loop-helix (bHLH) family protein
At4g37260	1,38	expressed protein
At5g01880	1,38	expressed protein
At3g13650	1,38	3’-5’ exonuclease domain-containing protein/helicase and RNase D C-terminal domain-containing
At2g14080	1,38	DNAJ heat shock N-terminal domain-containing protein
At3g21260	1,38	transcriptional factor B3 family protein
At3g47295	1,38	farnesyl-diphosphate farnesyltransferase 1 / squalene synthase 1 (SQS1)
At1g07260	1,38	casein kinase, putative
At5g40150	1,38	anion-transporting ATPase family protein
At5g18650	1,38	Ras-related GTP-binding protein, putative
At4g32350	1,38	expressed protein
At2g07740	1,38	nucleolar RNA-associated family protein / Nrap family protein
At4g14920	1,38	6-phosphogluconate dehydrogenase family protein
At4g12310	1,37	expressed protein
At3g06020	1,37	mitochondrial import inner membrane translocase subunit Tim17/Tim22/Tim23 family protein
At2g04690	1,37	sec23/sec24 transport family protein
At1g59620	1,37	myb family transcription factor (MYB103)
At5g51980	1,37	small nuclear ribonucleoprotein U2B, putative / spliceosomal protein, putative
At5g50450	1,37	Dof-type zinc finger domain-containing protein
At3g04860	1,37	chloroplast inner envelope membrane protein, putative (APG1)
At5g08240	1,37	St12p protein (ST12p) / SEC12p protein, putative

At3g14750	1,37	basic helix-loop-helix (bHLH) family protein
At3g46670	1,37	MATE efflux family protein
At5g16820	1,37	proton-dependent oligopeptide transport (POT) family protein
At2g25490	1,37	protein phosphatase 2C, putative / PP2C, putative
At1g05340	1,37	bZIP protein
At4g34760	1,37	GCN5-related N-acetyltransferase (GNAT) family protein / nuclear shuttle interacting protein (NSI)
At3g61380	1,37	lipid transfer protein 3 (LTP3)
At2g37580	1,37	glycine-rich protein
At4g39950	1,37	SNF2 domain-containing protein / helicase domain-containing protein
At4g35770	1,37	zinc finger (C3HC4-type RING finger) family protein / ankyrin repeat family protein
At2g23560	1,37	oxysterol-binding family protein
At3g05650	1,37	(2R)-phospho-3-sulfolactate synthase-related
At3g04350	1,37	BON1-associated protein (BAP1)-related
At2g45930	1,37	expressed protein
At3g61360	1,37	kinesin-related protein (MKRP2)
At2g46400	1,37	transcriptional factor B3 family protein
At5g56590	1,37	MutT/nudix family protein
At5g14130	1,37	Peroxidase superfamily protein
At5g25840	1,37	expressed protein
At5g64330	1,37	15.7 kDa class I-related small heat shock protein-like (HSP15.7-CI)
At3g19920	1,37	expressed protein
At1g67980	1,37	expressed protein
At5g23050	1,37	geranylgeranyl transferase alpha subunit-related
At3g60400	1,37	expressed protein
At5g16120	1,37	DegP protease, putative
At1g56010	1,37	F-box family protein
At5g58860	1,37	disease resistance-responsive protein-related / dirigent protein-related
At1g80830	1,37	LOB domain protein 12 / lateral organ boundaries domain protein 12 (LBD12)
At4g23140	1,37	mitochondrial substrate carrier family protein
At5g15240	1,37	hypothetical protein
At1g21100	1,37	calcineurin-like phosphoesterase family protein
At4g23810	1,37	lectin protein kinase, putative
At3g10400	1,36	encodes a U12-type spliceosomal protein U11/U12-31K
At2g19120	1,36	WWE domain-containing protein / ceo protein, putative (CEO)
At1g18970	1,36	thylakoid assembly protein, putative
At2g22660	1,36	peroxisomal biogenesis factor 11 family protein / PEX11 family protein
At2g28085	1,36	MATE efflux family protein
At3g15210	1,36	interferon-related developmental regulator family protein / IFRD protein family
At3g45900	1,36	hypothetical protein
At1g73150	1,36	expressed protein
At5g51560	1,36	expressed protein
At3g52070	1,36	dehydratase family
At1g28480	1,36	auxin-responsive family protein
At5g48180	1,36	hypothetical protein
At1g25550	1,36	RNase H domain-containing protein
At4g37410	1,36	phospholipase D zeta1 / PLDzeta1 (PLDP1)
At1g11310	1,36	SWIB complex BAF60b domain-containing protein / plus-3 domain-containing protein
At1g12710	1,36	expressed protein
At3g42140	1,36	CHP-rich zinc finger protein, putative
At1g73750	1,36	thioredoxin family protein
At4g24800	1,36	hypothetical protein
At4g33710	1,36	cytochrome P450 79A2 (CYP79A2)
At5g36250	1,36	expressed protein
At3g19300	1,36	short-chain dehydrogenase/reductase (SDR) family protein
At4g09560	1,36	hypothetical protein
At1g05575	1,36	ankyrin repeat family protein
At2g22420	1,36	pectinesterase family protein
At5g49360	1,36	expressed protein
At1g22550	1,36	hypothetical protein
At1g79370	1,36	disease resistance protein (CC-NBS-LRR class), putative
At1g20080	1,36	expressed protein
At5g11420	1,36	60S ribosomal protein L4/L1 (RPL4A)
At4g39090	1,36	enoyl-CoA hydratase/isomerase family protein
At2g21820	1,36	zinc finger (C3HC4-type RING finger) family protein
At3g10740	1,36	expressed protein
At1g71910	1,36	heat shock transcription factor family protein
At2g46750	1,36	expressed protein
At3g21090	1,36	DEAD/DEAH box helicase family protein / pentatricopeptide (PPR) repeat-containing protein
At1g19380	1,36	expressed protein
At3g22160	1,36	glycoside hydrolase family 28 protein / polygalacturonase (pectinase) family protein
At1g21450	1,35	disease resistance protein (CC-NBS-LRR class), putative
At3g63380	1,35	expressed protein
At3g19970	1,35	pseudogene, hypothetical protein
At2g03090	1,35	ADP-ribosylation factor, putative
At2g36670	1,35	KRR1 family protein

At1g70840	1,35	expressed protein
At2g23840	1,35	expressed protein
At5g60170	1,35	kelch repeat-containing F-box family protein
At1g42365	1,35	expressed protein
At2g38640	1,35	zinc finger (C3HC4-type RING finger) family protein
At3g17120	1,35	expressed protein
At2g46050	1,35	expressed protein
At1g32640	1,35	hypothetical protein
At4g23310	1,35	oxidoreductase, 2OG-Fe(II) oxygenase family protein
At1g13195	1,35	mitochondrial import receptor subunit TOM20-4 / translocase of outer membrane 20 kDa subunit 4
At5g55650	1,35	transducin family protein / WD-40 repeat family protein
At5g59780	1,35	leucine-rich repeat transmembrane protein kinase, putative
At4g00231	1,35	regulator of chromosome condensation (RCC1) family protein
At5g37710	1,35	expressed protein
At3g02210	1,35	thioredoxin family protein
At5g50820	1,35	UDP-glucoronosyl/UDP-glucosyl transferase family protein
At2g36000	1,35	cytochrome P450 family protein
At2g41705	1,35	4-coumarate--CoA ligase, putative / 4-coumaroyl-CoA synthase, putative
At5g56980	1,35	no apical meristem (NAM) family protein
At5g54080	1,35	hydrolase, alpha/beta fold family protein
At2g22760	1,35	SPL1-Related3 protein (SPL1R3)
At1g06570	1,34	transcription regulatory protein SNF2, putative
At4g16670	1,34	expressed protein
At2g22860	1,34	expressed protein
At3g23080	1,34	expressed protein
At3g16560	1,34	leucine-rich repeat family protein
At1g78890	1,34	ATP-dependent Clp protease ATP-binding subunit ClpX1 (CLPX)
At1g72030	1,34	kinase interacting family protein
At2g21510	1,34	hypothetical protein
At5g35930	1,34	sulfate transporter
At5g25160	1,34	bZIP transcription factor family protein
At5g57340	1,34	expressed protein
At3g61830	1,34	mitochondrial substrate carrier family protein
At5g52400	1,34	dihydroneopterin aldolase, putative
At4g32940	1,34	eukaryotic translation initiation factor 2B family protein / eIF-2B family protein
At3g12360	1,34	protein kinase family protein
At1g79970	1,34	proline-rich family protein
At5g44930	1,34	leucine-rich repeat family protein / protein kinase family protein
At1g49230	1,34	Ran-binding protein 1, putative / RanBP1, putative
At1g78995	1,34	CBL-interacting protein kinase 23 (CIPK23)
At2g43590	1,34	zinc finger (CCCH-type) family protein
At4g20470	1,34	malate oxidoreductase, putative
At2g14450	1,34	expressed protein
At2g17850	1,34	expressed protein
At3g07650	1,34	expressed protein
At1g67520	1,34	DNAJ heat shock N-terminal domain-containing protein / S-locus protein, putative
At4g19860	1,34	short-chain dehydrogenase/reductase (SDR) family protein
At4g36030	1,34	adenylate isopentenyltransferase 3 / cytokinin synthase (IPT3)
At1g77110	1,34	PHD finger transcription factor, putative
At5g14390	1,34	xyloglucan:xyloglucosyl transferase, putative / xyloglucan endotransglycosylase, putative
At1g62990	1,34	hypothetical protein
At2g26290	1,34	expressed protein
At2g46060	1,34	kinesin-like protein B (KATB)
At1g07350	1,34	late embryogenesis abundant protein-related / LEA protein-related
At4g14930	1,34	transcriptional factor B3 family protein
At3g59110	1,34	myosin heavy chain-related
At4g15310	1,34	major intrinsic family protein / MIP family protein
At1g13700	1,34	ubiquitin-specific protease 2 (UBP2)
At1g78270	1,34	expressed protein
At3g02840	1,34	ARM repeat superfamily protein
At5g65630	1,33	gibberellin 20-oxidase family protein
At2g23690	1,33	unknown protein
At2g21180	1,33	60S ribosomal protein L31 (RPL31A)
At2g30750	1,33	dihydrofolate synthetase/folylpolyglutamate synthetase (DHFS/FPGS4)
At5g45060	1,33	hypothetical protein
At3g26600	1,33	pentatricopeptide (PPR) repeat-containing protein
At5g56340	1,33	argonaute protein (AGO1)
At5g01720	1,33	expressed protein
At5g07700	1,33	expressed protein
At5g41680	1,33	expressed protein
At5g09940	1,33	oligouridylate-binding protein, putative
At3g51860	1,33	cinnamoyl-CoA reductase-related
At5g17960	1,33	tRNA synthetase class I (C) family protein
At2g19130	1,33	ethylene-responsive calmodulin-binding protein, putative (SR1)
At2g02590	1,33	expressed protein

At4g28370	1,33	hypothetical protein
At3g20310	1,33	expressed protein
At1g79410	1,33	terpene synthase/cyclase family protein
At1g21590	1,33	hypothetical protein
At1g78690	1,33	expressed protein
At2g30510	1,33	PAC motif-containing protein
At1g80320	1,33	myb family transcription factor
At3g51490	1,33	pentatricopeptide (PPR) repeat-containing protein
At1g73680	1,33	Encodes an alpha dioxygenase
At5g21170	1,33	expressed protein
At3g20470	1,33	cytochrome P450, putative
At5g47910	1,33	TCP family transcription factor, putative
At5g08535	1,33	pseudogene, similar to putative helicase
At2g25160	1,33	AP2 domain-containing transcription factor TINY, putative
At3g13450	1,33	pentatricopeptide (PPR) repeat-containing protein
At3g22150	1,33	mitochondrial substrate carrier family protein
At4g33300	1,33	tonneau 1b (TON1b)
At5g22690	1,33	serine/threonine protein kinase, putative
At3g51760	1,33	expressed protein
At3g03670	1,33	pseudogene, hypothetical protein
At3g60520	1,33	glycosyl transferase family 8 protein
At4g27690	1,33	expressed protein
At2g39920	1,33	expressed protein
At3g19770	1,33	NHL repeat-containing protein
At3g54150	1,33	S-adenosyl-L-methionine-dependent methyltransferases superfamily protein
At3g01180	1,33	S-locus protein kinase, putative
At1g56520	1,33	alpha-trehalose-phosphate synthase, UDP-forming, putative/trehalose-6-phosphate synthase, putati
At1g26390	1,33	cellulose synthase family protein
At1g10340	1,33	sulfotransferase family protein
At5g22290	1,33	zinc finger homeobox family protein / ZF-HD homeobox family protein
At1g27100	1,33	pentatricopeptide (PPR) repeat-containing protein
At5g18490	1,33	protease inhibitor, putative (DR4)
At4g17760	1,33	NAD-dependent epimerase/dehydratase family protein
At5g49730	1,33	cohesion family protein SYN2 (SYN2)
At5g40460	1,33	expressed protein
At1g07705	1,33	FAD-binding domain-containing protein
At3g17470	1,33	heavy-metal-associated domain-containing protein
At5g61000	1,33	dimethyladenosine transferase, putative
At1g21610	1,33	disease resistance protein (TIR-NBS-LRR class), putative
At4g16515	1,33	expressed protein
At3g54260	1,33	phosphoesterase family protein
At3g12340	1,33	leucine-rich repeat transmembrane protein kinase, putative
At2g47770	1,33	transducin family protein / WD-40 repeat family protein
At1g65360	1,32	zinc finger (B-box type) family protein
At1g48320	1,32	DC1 domain-containing protein
At2g05720	1,32	basic helix-loop-helix (bHLH) family protein
At1g50040	1,32	expressed protein
At5g58150	1,32	expressed protein
At1g74450	1,32	serine/threonine protein phosphatase PP2A-2 catalytic subunit (PP2A2)
At1g29690	1,32	zinc finger (C3HC4-type RING finger) family protein
At1g64405	1,32	lysine and histidine specific transporter, putative
At1g17840	1,32	leucine-rich repeat transmembrane protein kinase, putative
At1g68740	1,32	DNA (cytosine-5-)-methyltransferase, putative
At5g38100	1,32	tetratricopeptide repeat (TPR)-containing protein
At3g12940	1,32	dehydration-responsive family protein
At1g74710	1,32	phosphatidylinositol-4-phosphate 5-kinase family protein
At4g18780	1,32	intracellular protein transport protein USO1-related
At3g45640	1,32	cystathionine gamma-synthase, chloroplast, putative / O-succinylhomoserine (Thiol)-lyase, putative
At1g55640	1,32	hypothetical protein
At1g80110	1,32	expressed protein
At3g15530	1,32	F-box family protein (FBW1)
At4g37790	1,32	exocyst subunit EXO70 family protein
At1g23890	1,32	basic helix-loop-helix (bHLH) family protein
At1g72360	1,32	expressed protein
At4g25910	1,32	ethylene-responsive factor, putative
At1g19690	1,32	two-component responsive regulator / response reactor 4 (RR4)
At3g23030	1,32	pentatricopeptide (PPR) repeat-containing protein
At3g17060	1,32	Vps52/Sac2 family protein
At3g26740	1,32	transcription factor jumonji (jmjC) domain-containing protein
At1g28520	1,32	methionyl aminopeptidase, putative / methionine aminopeptidase, putative / peptidase M, putative
At1g27990	1,32	hydroxyproline-rich glycoprotein family protein
At3g21870	1,32	expressed protein
At5g10820	1,32	aquaporin, putative
At4g02160	1,32	expressed protein
At5g24490	1,32	zinc finger protein (PRAF1) / regulator of chromosome condensation (RCC1) family protein

At2g42350	1,32	expressed protein
At1g64170	1,32	phytochelatin synthetase-related
At3g13510	1,32	protein kinase family protein
At5g05410	1,32	pectate lyase family protein
At3g48640	1,32	zinc finger protein-related
At1g79960	1,32	CBL-interacting protein kinase 19 (CIPK19)
At1g63480	1,32	MADS-box family protein
At1g07870	1,32	scarecrow transcription factor family protein
At2g15420	1,32	amino acid permease, putative (AUX1)
At1g73220	1,32	expressed protein
At1g69050	1,31	serpin family protein / serine protease inhibitor family protein
At1g05470	1,31	cyclic nucleotide-regulated ion channel, putative
At3g22800	1,31	ERF domain protein 9 (ERF9)
At4g33130	1,31	AAA-type ATPase family protein
At5g35790	1,31	Ulp1 protease family protein
At5g38910	1,31	AP2 domain-containing transcription factor, putative
At1g78310	1,31	expressed protein
At3g45160	1,31	hydrolase, alpha/beta fold family protein
At2g22060	1,31	molybdopterin biosynthesis CNX1 protein / molybdenum cofactor biosynthesis enzyme CNX1
At2g16660	1,31	serine/threonine protein kinase (MHK)
At3g54330	1,31	expressed protein
At5g61570	1,31	actin polymerization factor protein-related
At1g34420	1,31	expressed protein
At1g68620	1,31	expressed protein
At5g24380	1,31	coatomer protein complex, subunit beta 2 (beta prime), putative
At2g18970	1,31	ubiquitin-specific protease 4 (UBP4)
At1g10170	1,31	copper transporter 1 (COPT1)
At3g05120	1,31	nucleolar protein gar2-related
At1g52140	1,31	glycosyl hydrolase family 5 protein / cellulase family protein
At1g72670	1,31	phospholipid/glycerol acyltransferase family protein
At4g26090	1,31	hypothetical protein
At2g28440	1,31	mevalonate diphosphate decarboxylase, putative
At5g54200	1,31	UVB-resistance protein (UVR8)
At3g23410	1,31	thylakoid lumenal 17.9 kDa protein, chloroplast
At2g33830	1,31	expressed protein
At4g39100	1,31	hypothetical protein
At3g01970	1,31	expressed protein
At4g16260	1,31	gypsy-like retrotransposon family (Athila)
At3g50440	1,31	scarecrow transcription factor, putative
At1g53100	1,31	expressed protein
At4g04460	1,31	hypothetical protein
At5g25350	1,31	zinc finger (C3HC4-type RING finger) family protein
At5g49150	1,31	expressed protein
At1g67830	1,31	expressed protein
At1g77990	1,31	hypothetical protein
At3g20030	1,31	pentatricopeptide (PPR) repeat-containing protein
At4g32105	1,31	prephenate dehydratase family protein
At3g25090	1,31	hypothetical protein
At4g36010	1,31	expressed protein
At5g61300	1,31	ribosomal protein L1 family protein
At1g47940	1,31	Clavata3 / ESR-Related-6 (CLE6)
At2g22550	1,31	expressed protein
At5g24070	1,31	cytochrome P450-related
At5g49390	1,31	expansin, putative (EXP5)
At4g30870	1,31	Encodes an Arabidopsis homolog of the endonuclease MSU81
At2g04880	1,31	DegP protease, putative
At4g22540	1,31	glycosyl hydrolase family 9 protein
At5g64780	1,31	transcription factor, putative
At1g13250	1,31	leucine-rich repeat protein kinase, putative
At5g45800	1,31	geranylgeranyl pyrophosphate synthase, putative / GGPP/ farnesyltranstransferase, putative
At2g31870	1,30	phytochrome A signal transduction 1 (PAT1)
At5g47730	1,30	ubiquinol-cytochrome C reductase complex 7.8 kDa protein, putative / mitochondrial hinge protein
At2g30480	1,30	MATE efflux family protein
At2g12250	1,30	glutamate receptor family protein (GLR3.7) (GLR5)
At2g36770	1,30	DNA-binding family protein / remorin family protein
At1g74950	1,30	myb family transcription factor
At5g56160	1,30	expressed protein
At1g49450	1,30	myb family transcription factor (MYB66) / werewolf (WER)
At5g27420	1,30	expressed protein
At2g31050	1,30	glycosyl hydrolase family 1 protein
At2g24120	1,30	auxin-responsive factor (ARF7)
At3g58790	1,30	60S acidic ribosomal protein P3 (RPP3A)
At5g50360	1,30	expressed protein
At4g33000	1,30	cytochrome b5 domain-containing protein
At1g67910	1,30	expressed protein

At1g05590	1,30	hypothetical protein
At1g72520	1,30	endonuclease/exonuclease/phosphatase family protein / calcium-binding EF hand family protein
At5g62130	1,30	expressed protein
At2g17470	1,30	hypothetical protein
At4g15805	1,30	esterase/lipase/thioesterase family protein
At5g63190	1,30	AICARFT/IMPCHase bienzyme family protein
At5g47070	1,30	leucine-rich repeat family protein
At4g13450	1,30	hypothetical protein
At3g22240	1,30	phosphatidylinositol-4-phosphate 5-kinase family protein
At1g13740	1,30	pentatricopeptide (PPR) repeat-containing protein
At5g11870	1,30	hypothetical protein
At3g45120	1,30	dehydration-responsive protein-related
At1g55310	1,30	transporter-related
At3g45040	1,30	glutathione S-transferase-related
At3g13040	1,30	calmodulin-binding family protein
At1g32560	1,30	translation elongation factor Ts (EF-Ts), putative
At4g33960	1,30	transcriptional factor B3 family protein
At5g56100	1,30	metallo-beta-lactamase family protein
At3g05640	1,30	myb family transcription factor
At2g24550	1,30	N’-5’-phosphoribosyl-formimino-5-aminoimidazole-4-carboxamide ribonucleotide isomerase
At1g16680	1,30	major latex protein-related / MLP-related
At5g46370	1,30	protein kinase-related
At2g33580	1,30	LysM-containing receptor-like kinase
At2g38905	1,30	myb family transcription factor
At3g22250	1,30	UDP-Glycosyltransferase superfamily protein
At1g63740	1,30	DEAD box RNA helicase, putative
At3g02885	1,30	myb family transcription factor (MYB63)
At1g18740	1,30	zinc finger (C2H2 type) family protein
At1g75730	1,30	DEAD/DEAH box helicase, putative
At1g30860	1,30	adenylylsulfate kinase, putative
At5g58430	1,30	KH domain-containing protein / zinc finger (CCCH type) family protein
At5g27350	1,30	protein tyrosine phosphatase-like protein, putative (PAS2)
At1g51990	1,30	phenylalanine ammonia-lyase 1 (PAL1)
At3g11660	1,30	K+ efflux antiporter, putative (KEA6)
At1g29160	1,30	zinc finger (CCCH-type) family protein
At5g61250	1,30	expressed protein
At2g36840	1,30	myb family transcription factor (MYB49)
At5g11650	1,30	expressed protein
At2g20145	1,30	D111/G-patch domain-containing protein
At3g56400	1,30	glucose-6-phosphate isomerase, cytosolic (PGIC)
At1g10350	1,30	inorganic phosphate transporter (PHT1) (PT1)

mutant *lsm1a lsm1b*

Transcript ID	stabilization fold	Gene Title
At3g29970	2,15	DIE2/ALG10 family
At4g15700	2,09	transducin family protein / WD-40 repeat family protein
At3g23230	2,06	cation exchanger, putative (CAX11)
At1g64380	1,95	zinc finger (C3HC4-type RING finger) family protein / BRCT domain-containing protein
At1g60030	1,93	chloroplast protein import component-related
At4g25490	1,92	expressed protein
At5g15580	1,91	UDP-glucose 6-dehydrogenase, putative
At1g61800	1,89	calmodulin-binding family protein
At4g26090	1,84	hypothetical protein
At2g18050	1,84	serine carboxypeptidase S10 family protein
At1g59620	1,82	myb family transcription factor (MYB103)
At1g66500	1,80	Pre-mRNA cleavage complex II
At4g19190	1,78	expressed protein
At4g34760	1,76	GCN5-related N-acetyltransferase (GNAT) family protein / nuclear shuttle interacting protein (NSI)
At3g46930	1,75	calmodulin, putative
At3g56080	1,75	permease, putative
At5g20820	1,74	UDP-glucoronosyl/UDP-glucosyl transferase family protein
At3g12560	1,74	expressed protein
At5g25190	1,71	beta-(1,2)-xylosyltransferase (XYLT)
At5g35370	1,71	serine/threonine protein kinase, putative
At3g25990	1,71	superoxide dismutase (Mn), mitochondrial (SODA) / manganese superoxide dismutase (MSD1)
At3g18710	1,70	DNA replication licensing factor, putative
At4g37290	1,68	hypothetical protein
At3g54580	1,68	hypothetical protein
At4g32020	1,68	UMUC-like DNA repair family protein
At1g30370	1,67	expressed protein
At3g19850	1,67	zinc finger (C3HC4-type RING finger) family protein
At5g25570	1,66	expressed protein
At3g62620	1,66	eukaryotic translation initiation factor 1A, putative / eIF-1A, putative / eIF-4C, putative
At5g03690	1,65	pentatricopeptide (PPR) repeat-containing protein
At5g67420	1,64	subtilase family protein
At5g22220	1,64	expressed protein
At5g47380	1,63	ubiquitin interaction motif-containing protein
At3g20430	1,63	hypothetical protein
At4g27900	1,63	hypothetical protein
At1g12820	1,63	proton-dependent oligopeptide transport (POT) family protein
At3g51770	1,63	expressed protein
At5g25520	1,63	F-box family protein
At3g11840	1,62	Encodes a U-box-domain-containing E3 ubiquitin ligase that acts as a negative regulator PAMP
At4g25820	1,62	expressed protein
At2g33170	1,61	expressed protein
At4g16270	1,61	glycine-rich protein (GRP19)
At1g77850	1,61	MATE efflux protein-related
At2g42140	1,61	GDSL-motif lipase/hydrolase family protein
At5g28530	1,61	beta-galactosidase, putative / lactase, putative
At5g23680	1,60	expressed protein
At5g50550	1,60	S-adenosyl-L-methionine:carboxyl methyltransferase family protein
At5g23080	1,60	disease resistance protein (TIR-NBS-LRR class), putative
At5g38700	1,60	expressed protein
At3g42570	1,60	hypothetical protein
At1g02330	1,60	dienelactone hydrolase family protein
At2g01990	1,60	eukaryotic translation initiation factor 2 family protein / eIF-2 family protein
At3g19090	1,60	expressed protein
At2g41330	1,59	expressed protein
At5g48850	1,59	expressed protein
At1g78070	1,59	transducin family protein / WD-40 repeat family protein
At1g12280	1,59	glycogenin glucosyltransferase (glycogenin)-related
At1g68780	1,59	hypothetical protein
At3g50050	1,59	expressed protein
At1g55830	1,58	expressed protein
At4g33560	1,58	40S ribosomal protein S29 (RPS29A)
At3g06590	1,58	cytochrome P450, putative
At3g53540	1,58	cytochrome P450 family protein
At3g46110	1,58	ATP-dependent DNA helicase, putative
At5g02890	1,58	plastocyanin-like domain-containing protein
At3g22970	1,58	expressed protein
At3g30140	1,57	flavin-containing monooxygenase family protein / FMO family protein
At3g50930	1,57	hypothetical protein
At1g53540	1,57	geranylgeranyl pyrophosphate synthase, putative / GGPP synthetase, putative
At5g25580	1,57	kelch repeat-containing F-box family protein
At1g06210	1,57	expressed protein
At1g78270	1,57	expressed protein

At4g38000	1,57	hypothetical protein
At1g76530	1,56	proton-dependent oligopeptide transport (POT) family protein
At1g74430	1,56	succinyl-CoA ligase (GDP-forming) beta-chain, mitochondrial, putative / succinyl-CoA synthetase
At3g57795	1,56	60S ribosomal protein L37a (RPL37aB)
At2g47520	1,56	expressed protein
At2g43000	1,56	AMP-dependent synthetase and ligase family protein
At4g39570	1,56	UTP-glucose-1-phosphate uridylyltransferase, putative/UDP-glucose pyrophosphorylase, putative
At2g07722	1,56	mechanosensitive ion channel domain-containing protein / MS ion channel domain
At4g23880	1,56	MADS-box family protein
At5g67480	1,56	peptidase M3 family protein / thimet oligopeptidase family protein
At1g24400	1,56	expressed protein
At1g69880	1,56	subtilase family protein
At2g43050	1,56	expressed protein
At3g13690	1,56	kelch repeat-containing F-box family protein
At1g18880	1,56	ribosome-binding factor A family protein
At4g00231	1,55	regulator of chromosome condensation (RCC1) family protein
At5g67280	1,55	dynamin family protein
At2g36400	1,55	Growth regulating factor encoding transcription activator
At3g14910	1,55	calcium-transporting ATPase, plasma membrane-type, putative / Ca2+-ATPase, putative ACA10
At3g48880	1,55	cyclin family protein
At1g74120	1,55	F-box family protein
At3g22640	1,55	expressed protein
At3g18930	1,55	NLI interacting factor (NIF) family protein
At5g47430	1,55	DWNN domain, a CCHC-type zinc finger
At2g32100	1,54	calcium-dependent protein kinase, putative / CDPK, putative
At4g19220	1,54	60S acidic ribosomal protein P3 (RPP3B)
At4g28040	1,54	LIM domain-containing protein
At1g19530	1,54	zinc knuckle (CCHC-type) family protein
At2g31180	1,54	molybdenum cofactor sulfurase (LOS5) (ABA3)
At1g66850	1,54	ubiquitin-conjugating enzyme, putative
At5g15120	1,54	UDP-D-glucuronate carboxy-lyase-related
At2g18500	1,54	EXS family protein / ERD1/XPR1/SYG1 family protein
At1g14930	1,54	phosphatidylinositol 4-kinase, putative
At3g03630	1,54	amidase family protein
At4g15680	1,54	pectate lyase family protein
At5g22470	1,54	NAD+ ADP-ribosyltransferases
At4g19120	1,54	DENN (AEX-3) domain-containing protein
At1g68620	1,54	expressed protein
At5g51050	1,54	expressed protein
At3g56440	1,53	cytochrome P450, putative
At1g12710	1,53	expressed protein
At1g22335	1,53	GPI-anchor transamidase, putative
At5g63630	1,53	MADS-box protein AGL27-II (AGL27) / MADS affecting flowering 1 (MAF1)
At2g41820	1,53	hypothetical protein
At1g06760	1,53	myb family transcription factor (MYB122)
At1g26970	1,53	basic helix-loop-helix (bHLH) family protein
At5g47390	1,53	SET domain-containing protein (SUVH2)
At5g59350	1,53	expressed protein
At1g70150	1,53	cytidylyltransferase domain-containing protein
At1g27690	1,53	hypothetical protein
At4g27260	1,52	F-box family protein
At4g01220	1,52	hypothetical protein
At4g25150	1,52	RNA recognition motif (RRM)-containing protein
At5g49440	1,52	expressed protein
At5g14390	1,52	xyloglucan:xyloglucosyl transferase, putative / xyloglucan endotransglycosylase, putative
At1g30080	1,52	hypothetical protein
At2g36485	1,52	expressed protein
At3g04710	1,52	expressed protein
At1g04310	1,52	cation/hydrogen exchanger, putative (CHX18)
At1g10850	1,52	DEAD box RNA helicase, putative (RH20)
At1g68930	1,52	tetraacyldisaccharide 4'-kinase family protein
At2g42870	1,52	terpene synthase/cyclase family protein
At5g23610	1,52	hypothetical protein
At1g10020	1,52	acyl-(acyl-carrier-protein) desaturase, putative / stearoyl-ACP desaturase, putative
At1g51090	1,52	outer envelope membrane protein, putative
At1g04230	1,52	ankyrin repeat family protein
At5g28300	1,51	expressed protein
At2g41730	1,51	ankyrin repeat family protein / BTB/POZ domain-containing protein
At4g36490	1,51	cinnamoyl-CoA reductase family
At5g38850	1,51	RNA-binding protein, putative
At1g43730	1,51	hypothetical protein
At2g24630	1,51	protein kinase, putative
At2g20180	1,51	CBS domain-containing protein / octicosapeptide/Phox/Bemp1 (PB1) domain-containing protein
At1g03350	1,51	hydroxyproline-rich glycoprotein family protein
At1g65240	1,50	expressed protein

At4g22980	1,50	hypothetical protein
At4g16515	1,50	expressed protein
At2g22430	1,50	expressed protein
At1g80630	1,50	GRAM domain-containing protein / ABA-responsive protein-related
At5g62900	1,50	Ras-related GTP-binding protein, putative
At5g50170	1,50	transducin family protein / WD-40 repeat family protein
At5g50450	1,50	Dof-type zinc finger domain-containing protein
At5g43490	1,50	ferredoxin, putative
At1g14890	1,50	glutamine synthetase, putative
At4g26920	1,49	glutaredoxin family protein
At1g66550	1,49	diphthamide synthesis DPH2 family protein
At1g68050	1,49	pectinesterase family protein
At4g26260	1,49	calcineurin-like phosphoesterase family protein
At1g07050	1,49	tetratricopeptide repeat (TPR)-containing protein
At1g15330	1,49	invertase/pectin methylesterase inhibitor family protein
At2g26710	1,49	MATE efflux family protein
At3g07520	1,49	dual specificity protein phosphatase (DsPTP1)
At3g21080	1,49	actin 12 (ACT12)
At1g11400	1,49	expressed protein
At2g15695	1,49	NOT2/NOT3/NOT5 family protein
At5g66820	1,49	pyruvate kinase, putative
At2g46690	1,49	cyclic nucleotide-regulated ion channel / cyclic nucleotide-gated channel (CNGC1)
At1g63670	1,49	CCR4-NOT transcription complex protein, putative
At5g61450	1,49	glycosyl transferase family 17 protein
At5g35670	1,49	chloroplast outer membrane translocon subunit, putative
At1g73070	1,49	expressed protein
At2g20800	1,49	oxidoreductase, zinc-binding dehydrogenase family protein
At1g13110	1,48	actin/actin-like family protein
At1g63820	1,48	hypothetical protein
At5g40130	1,48	monooxygenase, putative (MO2)
At3g13960	1,48	MutT/nudix family protein
At3g10360	1,48	PHD finger family protein
At5g20240	1,48	S-adenosyl-methionine-sterol-C-methyltransferase
At5g18680	1,48	haloacid dehalogenase-like hydrolase family protein
At3g18220	1,48	transcriptional factor B3 family protein / auxin-responsive factor AUX/IAA-related
At1g11210	1,48	pentatricopeptide (PPR) repeat-containing protein
At5g18530	1,48	lectin protein kinase family protein
At5g50410	1,48	expressed protein
At3g32090	1,48	DNA mismatch repair MutS family protein
At4g33070	1,48	late embryogenesis abundant protein, putative / LEA protein, putative
At3g53720	1,48	pseudogene, Ulp1 protease family
At4g20320	1,48	expressed protein
At1g30040	1,48	expressed protein
At3g45130	1,48	hypothetical protein
At1g62050	1,48	transcription initiation factor IID-2 (TFIID-2) / TATA-box factor 2 / TATA sequence (TBP2)
At5g46850	1,48	oxidoreductase, 2OG-Fe(II) oxygenase family protein
At5g48650	1,48	expressed protein
At5g43990	1,48	S-locus lectin protein kinase family protein
At3g02340	1,48	RING/U-box superfamily protein
At1g63310	1,47	ferric-chelate reductase, putative
At5g23460	1,47	hypothetical protein
At2g21210	1,47	cytochrome P450, putative
At5g03470	1,47	COP9 signalosome subunit 6 / CSN subunit 6 (CSN6A)
At1g67855	1,47	expressed protein
At4g12400	1,47	histone H2B, putative
At2g16460	1,47	expressed protein
At5g22750	1,47	ADP-ribosylation factor
At3g26040	1,47	dehydration-induced protein (ERD15)
At4g23980	1,47	Encodes auxin response factor 9 (ARF9)
At1g04000	1,47	expressed protein
At1g66160	1,47	F-box family protein
At5g35870	1,47	expressed protein
At1g79410	1,47	terpene synthase/cyclase family protein
At3g19650	1,47	cyclin-related
At5g16960	1,47	protein kinase, putative
At5g65540	1,46	MFP1 attachment factor, putative
At1g33060	1,46	glutathione S-transferase, putative
At3g47500	1,46	ubiquitin-conjugating enzyme family protein
At5g01260	1,46	expressed protein
At1g09575	1,46	fip1 motif-containing protein
At1g73570	1,46	HCP-like superfamily protein
At2g24840	1,46	expressed protein
At3g45680	1,46	auxin-responsive factor-related
At1g61810	1,46	NLI interacting factor (NIF) family protein
At4g37260	1,46	expressed protein

At5g48900	1,46	peptidyl-prolyl cis-trans isomerase / cyclophilin (CYP2) / rotamase
At5g01200	1,46	vesicle-associated membrane protein, putative / VAMP, putative
At4g39410	1,46	member of WRKY Transcription Factor; Group II-c
At5g61590	1,46	expressed protein
At5g48070	1,46	cation efflux family protein
At2g20470	1,46	integral membrane family protein
At5g66440	1,46	rapid alkalinization factor (RALF) family protein
At4g01280	1,46	expressed protein
At2g38060	1,46	expressed protein
At1g02540	1,46	expressed protein
At4g34210	1,45	pathogenesis-related protein, putative
At3g01610	1,45	wall-associated kinase, putative
At3g09070	1,45	RNA recognition motif (RRM)-containing protein
At3g50440	1,45	scarecrow transcription factor, putative
At4g38840	1,45	expressed protein
At1g23000	1,45	protein kinase-related
At3g42300	1,45	NAD-dependent epimerase/dehydratase family protein
At5g50030	1,45	29 kDa ribonucleoprotein, chloroplast / RNA-binding protein cp 29
At5g18600	1,45	cysteine synthase, chloroplast / O-acetylserine (thiol)-lyase / O-acetylserine sulfhydrylase (OASB)
At1g01760	1,45	expressed protein
At1g52680	1,45	transcriptional factor B3 family protein
At2g02850	1,45	WRKY family transcription factor
At3g51040	1,45	leucine-rich repeat transmembrane protein kinase, putative
At5g42140	1,45	myb family transcription factor (MYB113)
At5g46490	1,45	hydrolase, alpha/beta fold family protein
At2g29580	1,45	phosphoinositide-specific phospholipase C (PLC1)
At4g30960	1,45	expressed protein
At5g65640	1,44	transcriptional factor B3 family protein
At2g34570	1,44	haloacid dehalogenase-like hydrolase family protein
At2g04490	1,44	CCR4-NOT transcription complex protein, putative
At2g13840	1,44	myb family transcription factor
At1g68490	1,44	phototropic-responsive NPH3 family protein
At1g43886	1,44	Met-10+ like family protein
At3g21480	1,44	expressed protein (SWP1)
At1g43930	1,44	AAA-type ATPase family protein
At3g46230	1,44	sterol 4-alpha-methyl-oxidase 2 (SMO2)
At3g60360	1,44	leucine-rich repeat family protein, 5' fragment
At2g04240	1,44	E3 ubiquitin ligase SCF complex subunit SKP1/ASK1 (At10), putative
At5g52190	1,44	DC1 domain-containing protein
At3g50770	1,44	expressed protein
At1g13260	1,44	auxin-responsive factor, putative
At5g07530	1,44	NAD-dependent epimerase/dehydratase family protein
At4g36780	1,44	terpene synthase/cyclase family protein
At5g35830	1,44	ankyrin repeat family protein / BTB/POZ domain-containing protein
At3g53880	1,44	60S acidic ribosomal protein P1 (RPP1A)
At5g18770	1,44	GDSL-motif lipase/hydrolase family protein
At1g58100	1,44	cyclin delta-2 (CYCD2)
At4g19670	1,43	cold-shock DNA-binding family protein
At1g06970	1,43	desulfhydrase family
At1g07350	1,43	late embryogenesis abundant protein-related / LEA protein-related
At1g53400	1,43	tRNA pseudouridine synthase family protein
At2g15870	1,43	cytochrome P450 71B19, putative (CYP71B19)
At5g44770	1,43	magnesium transporter CorA-like family protein
At5g56110	1,43	expressed protein
At5g20480	1,43	expressed protein
At5g58360	1,43	expressed protein
At1g48260	1,43	RNA recognition motif (RRM)-containing protein
At5g64640	1,43	expressed protein
At2g33490	1,43	ribosomal protein S17 family protein
At5g35330	1,43	heavy-metal-associated domain-containing protein / copper chaperone (CCH)-related
At2g46200	1,43	cytochrome P450, putative
At4g32430	1,43	Pentatricopeptide repeat (PPR) superfamily protein
At2g19890	1,43	expressed protein
At2g19470	1,43	kinesin-like protein (FRA1)
At1g69810	1,43	expressed protein
At4g09520	1,43	kinesin motor protein-related
At3g57440	1,43	expressed protein
At3g63130	1,43	exocyst complex component-related
At1g22310	1,43	amidase family protein
At3g52240	1,43	arabinogalactan-protein (AGP12)
At4g23440	1,42	hypothetical protein
At5g64470	1,42	pentatricopeptide (PPR) repeat-containing protein
At2g35290	1,42	pentatricopeptide (PPR) repeat-containing protein
At3g53570	1,42	lil3 protein
At4g24250	1,42	PAPA-1-like family protein / zinc finger (HIT type) family protein

At2g34440	1,42	DNA topoisomerase I
At2g24570	1,42	DEAD box RNA helicase, putative
At1g49620	1,42	sodium proton exchanger, putative (NHX5)
At1g68480	1,42	expressed protein
At1g53180	1,42	zinc finger (CCCH-type) family protein
At2g47020	1,42	PRLI-interacting factor, putative
At3g29210	1,42	cupin family protein
At4g26150	1,42	40S ribosomal protein S27 (RPS27D)
At4g31830	1,42	14-3-3 protein GF14 mu (GRF9)
At2g18540	1,42	RNA recognition motif (RRM)-containing protein
At1g75030	1,42	pfkB-type carbohydrate kinase family protein
At1g53940	1,42	haloacid dehalogenase-like hydrolase family protein
At1g52880	1,42	NADH-ubiquinone oxidoreductase 51 kDa subunit, mitochondrial, putative
At1g15360	1,42	zinc finger (C3HC4-type RING finger) family protein
At4g33490	1,41	chaperonin, putative
At3g62370	1,41	hypothetical protein
At4g13810	1,41	mitochondrial substrate carrier family protein
At1g50940	1,41	expressed protein
At3g43400	1,41	RNA recognition motif (RRM)-containing protein
At5g14180	1,41	expressed protein
At2g36650	1,41	alanine--glyoxylate aminotransferase, putative / beta-alanine-pyruvate aminotransferase, putative
At2g38960	1,41	expressed protein
At3g46530	1,41	pentacyclic triterpene synthase, putative
At5g10210	1,41	expressed protein
At2g44430	1,41	mitogen-activated protein kinase, putative / MAPK, putative (MPK11)
At3g28500	1,41	protein kinase family protein
At5g32470	1,41	expressed protein
At2g10880	1,41	MATE efflux family protein
At3g59690	1,41	leucine-rich repeat family protein
At3g01110	1,41	expressed protein
At1g06320	1,41	myb family transcription factor
At5g17260	1,41	expressed protein
At1g66610	1,41	far-red impaired responsive protein, putative
At4g27750	1,41	serine/threonine protein phosphatase 2A (PP2A) regulatory subunit B’ (B’beta)
At3g16360	1,41	Per1-like protein-related
At1g21820	1,41	ATP-dependent Clp protease proteolytic subunit (ClpP3)
At5g04670	1,41	speckle-type POZ protein-related
At4g05640	1,41	oxidoreductase-related
At4g30790	1,41	RWP-RK domain-containing protein
At1g09795	1,41	expressed protein
At1g48360	1,41	DNA replication licensing factor, putative
At5g14930	1,41	zinc finger protein-related
At1g63590	1,41	hypothetical protein
At3g22760	1,41	expressed protein
At4g24110	1,41	esterase/lipase/thioesterase family protein
At1g28050	1,41	B-box type zinc finger protein with CCT domain
At1g56180	1,41	expressed protein
At4g28180	1,41	NLI interacting factor (NIF) family protein
At5g50600	1,41	mitochondrial import inner membrane translocase subunit Tim17/Tim22/Tim23 family protein
At4g12010	1,41	hypothetical protein
At5g61160	1,41	agamous-like MADS box protein AGL5 / floral homeodomain transcription factor (AGL5)
At1g48960	1,41	guanine nucleotide-binding protein beta subunit (GB1) / GTP-binding protein beta subunit (AGB1)
At4g24240	1,40	expressed protein
At5g67190	1,40	defender against cell death 2 (DAD2)
At3g26430	1,40	PHD finger family protein (MMD1)
At1g13400	1,40	DC1 domain-containing protein
At5g45610	1,40	succinyl-CoA ligase (GDP-forming) alpha-chain, mitochondrial, putative/succinyl-CoA synthetase
At4g34950	1,40	no apical meristem (NAM) family protein
At5g08090	1,40	hydrolase, alpha/beta fold family protein
At2g33980	1,40	structural maintenance of chromosomes (SMC) family protein
At1g22970	1,40	haloacid dehalogenase-like hydrolase family protein
At2g37585	1,40	protein kinase family protein
At3g47180	1,40	tetratricopeptide repeat (TPR)-containing protein
At4g03400	1,40	Encodes a GH3-related gene involved in red light-specific hypocotyl elongation
At4g30560	1,40	aminotransferase class I and II family protein
At2g10390	1,40	heavy-metal-associated protein-related
At5g42690	1,40	fibrillarin 2 (FIB2)
At4g21990	1,40	vacuolar ATP synthase subunit G 2 (VATG2) / V-ATPase G subunit 2 (VAG2)
At5g36250	1,40	expressed protein
At5g25590	1,40	C2 domain-containing protein
At1g71770	1,40	calcium-transporting ATPase, plasma membrane-type, putative / Ca2+-ATPase, putative (ACA9)
At5g01210	1,40	hypothetical protein
At3g30610	1,40	DNAJ heat shock N-terminal domain-containing protein / cell division protein-related
At5g46540	1,40	ubiquitin extension protein, putative / 40S ribosomal protein S27A (RPS27aA)
At1g25400	1,40	expressed protein

At4g32780	1,40	DNAJ heat shock N-terminal domain-containing protein / sec63 domain-containing protein
At2g40130	1,40	sensory transduction histidine kinase-related
At4g17160	1,40	pentatricopeptide (PPR) repeat-containing protein
At4g40080	1,40	MADS-box family protein
At3g62450	1,40	expressed protein
At5g11430	1,40	expressed protein
At3g27220	1,39	glycine hydroxymethyltransferase, putative / serine hydroxymethyltransferase, putative
At5g02000	1,39	sec23/sec24 transport family protein
At1g47750	1,39	pyruvate kinase, putative
At2g19670	1,39	serine/threonine protein kinase, putative
At1g33055	1,39	hydroxyproline-rich glycoprotein family protein
At3g61800	1,39	expressed protein
At3g24480	1,39	pseudouridylate synthase TruB family protein
At1g44222	1,39	aquaglyceroporin / NOD26-like major intrinsic protein 2 (NLM2)
At5g40060	1,39	expressed protein
At1g54340	1,39	copper amine oxidase, putative
At1g61010	1,39	acyl-CoA oxidase, putative
At5g38340	1,39	hypothetical protein
At5g47520	1,39	RAB GTPase homolog A5A (RABA5a)
At1g20310	1,39	expressed protein
At1g60670	1,39	RNA recognition motif (RRM)-containing protein
At4g15280	1,39	tubulin folding cofactor C / Porcino (POR)
At3g48810	1,39	zinc finger (C3HC4-type RING finger) family protein
At1g02050	1,39	auxin-responsive protein, putative
At4g21310	1,39	expressed protein
At3g43510	1,39	DNAJ heat shock N-terminal domain-containing protein
At2g12550	1,39	ubiquitin-associated (UBA)/TS-N domain-containing protein
At3g25500	1,39	F-box family protein / SKP1 interacting partner 3-related
At1g72180	1,39	FAD-binding domain-containing protein
At3g59270	1,39	pyruvate kinase, putative
At2g38420	1,39	methyl-CpG-binding domain-containing protein
At1g18670	1,39	glycosyl hydrolase family 5 protein / cellulase family protein
At5g57080	1,39	expressed protein
At4g16020	1,39	RNA-binding protein, putative
At2g36840	1,39	myb family transcription factor (MYB49)
At4g18170	1,39	zinc finger protein-related
At1g13570	1,39	peptidyl-prolyl cis-trans isomerase cyclophilin-type family protein
At5g40520	1,39	expressed protein
At5g52530	1,39	prohibitin
At1g32610	1,39	expressed protein
At3g46640	1,39	small nuclear ribonucleoprotein D2, putative / snRNP core protein D2, putative / Sm protein D2
At3g50640	1,39	CCAAT-box binding transcription factor (LEC1)
At5g62640	1,39	expressed protein
At2g27200	1,39	glutaredoxin family protein
At4g20430	1,39	sulfotransferase family protein
At2g13690	1,39	hydroxyproline-rich glycoprotein family protein
At1g09460	1,39	aminoacylase, putative / N-acyl-L-amino-acid amidohydrolase, putative
At2g31990	1,39	calcineurin-like phosphoesterase family protein
At2g20150	1,38	expressed protein
At2g36010	1,38	expressed protein
At5g15710	1,38	H+-transporting two-sector ATPase, C subunit family protein
At3g15870	1,38	cadmium/zinc-transporting ATPase, putative (HMA1)
At2g25350	1,38	lactoylglutathione lyase family protein / glyoxalase I family protein
At4g03190	1,38	avirulence-responsive family protein / avirulence induced gene (AIG1) family protein
At4g14410	1,38	expressed protein
At4g17970	1,38	acyl-(acyl carrier protein) thioesterase/acyl-ACP thioesterase/oleoyl-(acyl-carrier protein)hydrolas
At4g02010	1,38	chlorophyllase (CLH2)
At3g09960	1,38	WRKY family transcription factor
At3g10020	1,38	expressed protein
At2g12150	1,38	small ubiquitin-like modifier 2 (SUMO)
At2g05370	1,38	G-box binding factor 4 (GBF4)
At3g11210	1,38	ammonium transporter, putative
At3g42140	1,38	CHP-rich zinc finger protein, putative
At4g32740	1,38	RNA helicase, putative
At3g58470	1,38	transferase family protein
At3g29010	1,38	expressed protein
At3g21410	1,38	expressed protein
At4g02160	1,38	expressed protein
At3g51640	1,38	serine/threonine protein kinase, putative
At2g23770	1,38	cathepsin B-like cysteine protease, putative
At3g60380	1,38	leucine-rich repeat family protein / extensin family protein (LRX1)
At5g08565	1,38	F-box family protein
At3g20410	1,38	glycerophosphoryl diester phosphodiesterase family protein
At1g54160	1,38	expressed protein
At4g22200	1,38	Dof-type zinc finger domain-containing protein

At4g37820	1,38	coatomer protein complex, subunit beta 2 (beta prime), putative
At5g64090	1,38	zinc finger (Ran-binding) family protein
At4g08940	1,38	rcd1-like cell differentiation protein, putative
At2g20160	1,38	expressed protein
At3g09160	1,38	pseudogene, hypothetical protein
At5g47800	1,38	VQ motif-containing protein
At2g29050	1,38	glycosyl hydrolase family 1 protein
At4g32070	1,38	hypothetical protein
At5g17890	1,38	zinc finger (NF-X1 type) family protein
At2g03170	1,38	DNA-binding family protein
At4g31150	1,38	alpha-N-acetylglucosaminidase family / NAGLU family
At1g76405	1,38	cytidine/deoxycytidylate deaminase family protein
At5g59360	1,38	protein transport protein sec61, putative
At2g24000	1,38	heat shock protein 70, putative / HSP70, putative
At1g34420	1,38	expressed protein
At4g13190	1,38	Ulp1 protease family protein
At1g06710	1,38	histone deacetylase, putative (HD2A)
At2g40640	1,38	protein kinase family protein
At5g39350	1,38	expressed protein
At4g25480	1,38	myb family transcription factor (MYB110)
At1g55810	1,38	F-box family protein
At3g06520	1,38	zinc finger (CCCH-type) family protein / RNA recognition motif (RRM)-containing protein
At5g43740	1,38	DNA-directed RNA polymerase, putative
At2g40350	1,38	myb-related transcription factor (CCA1)
At1g80340	1,38	epoxide hydrolase, putative
At4g33720	1,38	Ras-related GTP-binding protein, putative
At5g51560	1,38	expressed protein
At3g14080	1,38	disease resistance-responsive family protein
At5g14090	1,38	ataxin-related
At5g27670	1,37	phospholipase/carboxylesterase family protein
At5g62280	1,37	expressed protein
At1g11170	1,37	KH domain-containing protein
At2g15670	1,37	expressed protein
At3g25400	1,37	protein kinase family protein
At2g24430	1,37	expressed protein
At1g17665	1,37	tropinone reductase, putative / tropine dehydrogenase, putative
At5g49710	1,37	F-box family protein
At3g54930	1,37	expressed protein
At4g13120	1,37	hypothetical protein
At3g32940	1,37	expressed protein
At2g44420	1,37	DRE-binding protein (DREB2A)
At3g56585	1,37	expressed protein
At5g04510	1,37	armadillo/beta-catenin repeat family protein / F-box family protein
At2g32800	1,37	disease resistance protein (CC-NBS-LRR class), putative / PRM1 homolog, putative
At3g12060	1,37	expressed protein
At3g19200	1,37	rac GTPase activating protein, putative
At1g08940	1,37	ankyrin repeat family protein (EMB506)
At4g15240	1,37	HpcH/HpaI aldolase family protein
At3g14340	1,37	expressed protein
At2g13440	1,37	exocyst subunit EXO70 family protein
At2g37310	1,37	calmodulin-binding family protein
At2g41900	1,37	oligopeptide transporter OPT family protein
At3g29140	1,37	vacuolar protein sorting-associated protein 26, putative / VPS26, putative
At2g28740	1,37	auxin efflux carrier family protein
At4g38930	1,37	zinc finger (B-box type) family protein
At3g63240	1,37	MATE efflux family protein
At2g15830	1,37	cinnamyl-alcohol dehydrogenase (CAD)
At4g01120	1,37	expressed protein
At2g24160	1,37	esterase/lipase/thioesterase family protein
At2g01150	1,37	armadillo/beta-catenin repeat family protein / U-box domain-containing protein
At3g55500	1,37	glutamine synthetase (GS1)
At2g47440	1,37	expressed protein
At5g18990	1,37	non-SMC condensin subunit, XCAP-D2/Cnd1 family protein
At4g34540	1,37	GCN5-related N-acetyltransferase (GNAT) family protein
At3g05710	1,37	UV hypersensitive protein (UVH3) / DNA-repair protein, putative
At3g26960	1,37	Pollen Ole e 1 allergen and extensin family protein
At2g31270	1,37	myb family transcription factor (MYB78)
At3g58970	1,37	expressed protein
At1g16510	1,37	expressed protein
At2g35510	1,37	expressed protein
At1g28030	1,36	bZIP family transcription factor
At1g02700	1,36	RNA recognition motif (RRM)-containing protein
At5g25475	1,36	nucleotide-sugar transporter family protein
At4g38260	1,36	expressed protein
At5g59720	1,36	hypothetical protein

At5g57500	1,36	heat shock factor protein 2 (HSF2) / heat shock transcription factor 2 (HSTF2)
At3g62320	1,36	hypothetical protein
At1g77460	1,36	chaperonin (CPN60) (HSP60)
At4g22820	1,36	disease resistance protein (TIR-NBS-LRR class), putative
At2g46410	1,36	RNA-binding protein, putative
At2g42990	1,36	thioredoxin family protein
At4g20840	1,36	squamosa promoter-binding protein-like 4 (SPL4)
At5g58050	1,36	heavy-metal-associated domain-containing protein
At3g18360	1,36	expressed protein
At5g32460	1,36	leucine-rich repeat family protein / protein kinase family protein
At1g49975	1,36	hypothetical protein
At3g02800	1,36	Encodes an atypical dual-specificity phosphatase
At1g26270	1,36	expressed protein
At2g32390	1,36	kelch repeat-containing F-box family protein
At2g06200	1,36	quinolinate phosphoribosyl transferase family protein
At1g05400	1,36	thioredoxin M-type 3, chloroplast (TRX-M3)
At5g24130	1,36	permease-related
At5g64510	1,36	CER1 protein, putative
At3g14780	1,36	lysine decarboxylase family protein
At2g39540	1,36	auxin-responsive protein / indoleacetic acid-induced protein 16 (IAA16)
At1g71690	1,36	myb family transcription factor
At1g69050	1,36	serpin family protein / serine protease inhibitor family protein
At5g24580	1,36	avirulence-responsive family protein / avirulence induced gene (AIG1) family protein
At3g27320	1,36	phototropic-responsive NPH3 family protein
At1g06540	1,36	calmodulin-binding protein
At5g61360	1,36	expressed protein
At1g17160	1,36	hypothetical protein
At2g36020	1,36	heat shock protein 70, putative / HSP70, putative
At1g56090	1,36	F-box protein-related
At4g20780	1,36	40S ribosomal protein S27 (RPS27A)
At5g10550	1,36	gamma interferon responsive lysosomal thiol reductase family protein / GILT family protein
At1g27285	1,36	kinesin motor protein-related
At5g27610	1,36	auxin-responsive protein, putative
At5g07710	1,36	3’ exoribonuclease family domain 1-containing protein
At3g20120	1,36	protein phosphatase 2C family protein / PP2C family protein
At4g03410	1,36	zinc finger (C3HC4-type RING finger) family protein
At1g63650	1,36	expressed protein
At5g04630	1,36	malate dehydrogenase, glyoxysomal, putative
At3g21660	1,36	transducin family protein / WD-40 repeat family protein
At5g06760	1,36	Encodes LEA4-5, a member of the Late Embryogenesis Abundant (LEA) proteins
At4g23840	1,36	protein kinase-related
At4g10640	1,36	hexokinase, putative
At3g14440	1,36	hesB-like domain-containing protein
At2g01080	1,36	glycosyl hydrolase family 14 protein
At2g23510	1,36	myb family transcription factor
At5g05800	1,36	mitochondrial substrate carrier family protein
At1g54960	1,36	expressed protein
At3g27290	1,36	hypothetical protein
At1g48370	1,36	hypothetical protein
At4g22500	1,36	protein kinase family protein
At1g12290	1,36	late embryogenesis abundant group 1 domain-containing protein / LEA group 1
At4g17180	1,36	40S ribosomal protein S15A (RPS15aE)
At1g27360	1,36	expressed protein
At1g04540	1,35	nodulin MtN21 family protein
At3g60490	1,35	F-box family protein
At3g58810	1,35	pectinesterase family protein
At1g67220	1,35	heavy-metal-associated domain-containing protein / copper chaperone (CCH)-related
At5g43670	1,35	expressed protein
At1g52710	1,35	heat shock transcription factor family protein
At1g09750	1,35	12S seed storage protein (CRB)
At5g14500	1,35	60S acidic ribosomal protein P2 (RPP2D)
At4g02730	1,35	germin-like protein, putative
At4g00850	1,35	short-chain dehydrogenase/reductase (SDR) family protein
At2g41340	1,35	protein kinase family protein
At4g00820	1,35	oxidoreductase, 2OG-Fe(II) oxygenase family protein
At1g18010	1,35	aspartate aminotransferase, chloroplast / transaminase A (ASP5) (AAT1)
At3g54720	1,35	protein kinase family protein
At1g20640	1,35	auxin-responsive family protein
At5g39520	1,35	expressed protein
At4g22190	1,35	expressed protein
At5g47280	1,35	transferase family protein
At3g47840	1,35	pentatricopeptide (PPR) repeat-containing protein
At2g31630	1,35	expressed protein
At5g64370	1,35	ABC transporter family protein
At5g09250	1,35	glycosyl transferase family 1 protein

At5g63390	1,35	expressed protein
At3g49120	1,35	immunophilin, putative / FKBP-type peptidyl-prolyl cis-trans isomerase, putative
At4g05300	1,35	RNA-binding protein, putative
At2g32160	1,35	expressed protein
At5g16540	1,35	carotenoid isomerase, putative
At4g17615	1,35	clathrin coat assembly protein, putative
At1g77740	1,35	AAA-type ATPase family protein / vacuolar sorting protein-related
At1g77140	1,35	expressed protein
At3g42900	1,35	expressed protein
At4g17080	1,35	leucine-rich repeat family protein
At4g04955	1,35	Encodes an allantoinase which is involved in allantoin degradation and assimilation
At5g01400	1,35	hypothetical protein
At4g29530	1,35	expressed protein
At3g62630	1,35	adenylate isopentenyltransferase 8 / adenylate dimethylallyltransferase / cytokinin synthase (IPT8)
At3g52890	1,35	heavy-metal-associated domain-containing protein
At2g30340	1,35	electron transfer flavoprotein-ubiquinone oxidoreductase family protein
At2g41770	1,35	SC35-like splicing factor, 33 kD (SCL33)
At3g50550	1,35	unknown protein
At2g30620	1,35	microtubule-associated protein
At2g20940	1,35	expressed protein
At2g02480	1,35	expressed protein
At5g65490	1,35	oxidoreductase-related
At4g39070	1,35	Encodes BZS1, a brassinosteroids-regulated BZR1 target (BRBT) gene
At5g04500	1,35	myb family transcription factor (MYB17)
At4g30935	1,35	calmodulin-binding protein
At4g31500	1,35	expressed protein
At3g03930	1,35	hypothetical protein
At3g46540	1,35	phosphofructokinase family protein
At3g20340	1,35	nascent polypeptide-associated complex (NAC) domain-containing protein
At4g21510	1,35	hypothetical protein
At2g45170	1,35	phosphoglycerate/bisphosphoglycerate mutase family protein
At3g55880	1,35	phosphatidylinositol-4-phosphate 5-kinase family protein
At2g01760	1,35	U1 small nuclear ribonucleoprotein 70 (U1-70k)
At3g12710	1,35	synbindin, putative
At5g50560	1,35	geranylgeranyl pyrophosphate synthase, putative / GGPP synthetase, putative / farnesyltranstransfe
At2g20040	1,35	expressed protein
At2g35990	1,35	mitogen-activated protein kinase, putative / MAPK, putative (MPK15)
At3g52950	1,35	transducin family protein / WD-40 repeat family protein
At4g12920	1,34	late embryogenesis abundant domain-containing protein / LEA domain-containing protein
At4g14340	1,34	nodulin MtN21 family protein
At1g10320	1,34	hypothetical protein
At3g01830	1,34	DegP2 protease (DEGP2)
At1g60140	1,34	SGS domain-containing protein
At2g32600	1,34	zinc finger (C3HC4-type RING finger) family protein
At2g29660	1,34	transporter-related
At1g14070	1,34	hypothetical protein
At1g29680	1,34	prefoldin, putative
At3g02610	1,34	brix domain-containing protein
At2g27410	1,34	integral membrane protein, putative / sugar transporter family protein
At5g38040	1,34	pectinesterase family protein
At3g60300	1,34	eukaryotic translation initiation factor 6, putative / eIF-6, putative
At2g30470	1,34	expressed protein
At5g52710	1,34	glucosamine/galactosamine-6-phosphate isomerase family protein
At1g30860	1,34	adenylylsulfate kinase, putative
At1g51780	1,34	expressed protein
At5g52230	1,34	aspartyl protease family protein
At3g46480	1,34	DNAJ heat shock N-terminal domain-containing protein
At2g13675	1,34	two-component phosphorelay mediator 3 (HP3)
At1g07410	1,34	RAB GTPase homolog A2B (RABA2b)
At1g31010	1,34	coproporphyrinogen III oxidase, putative / coproporphyrinogenase, putative / coprogen oxidase
At3g62280	1,34	hypothetical protein
At1g12480	1,34	phosphoribulokinase/uridine kinase-related
At2g16060	1,34	no apical meristem (NAM) family protein
At1g44478	1,34	leucine-rich repeat family protein
At1g38950	1,34	tudor domain-containing protein / nuclease family protein
At3g49840	1,34	ethylene-responsive element-binding protein 1 (ERF1) / EREBP-2 protein
At4g31750	1,34	abscisic acid-responsive HVA22 family protein
At1g80190	1,34	flavin-containing monooxygenase family protein / FMO family protein
At1g65000	1,34	phosphatidylserine decarboxylase, putative
At4g04690	1,34	auxin-responsive family protein
At3g61190	1,34	hypothetical protein
At3g43230	1,34	expressed protein
At3g26170	1,34	expressed protein
At1g75730	1,34	DEAD/DEAH box helicase, putative
At1g33480	1,34	chlorophyll A-B binding protein CP26, chloroplast / light-harvesting complex II protein 5 (LHCB5)

At5g57180	1,34	transport protein, putative
At3g47100	1,34	cell wall protein precursor, putative
At1g05310	1,34	calcium-binding EF hand family protein
At3g17050	1,34	sugar transporter family protein
At2g38710	1,34	response to salt stress
At2g39705	1,34	leucine-rich repeat family protein / protein kinase family protein
At3g02260	1,34	F-box family protein-related
At1g68680	1,34	protein disulfide isomerase-related
At4g25530	1,34	kelch repeat-containing F-box family protein
At4g04450	1,34	pyridoxine 5'-phosphate oxidase-related
At2g18510	1,34	expressed protein
At3g55830	1,34	sugar transporter family protein
At5g67580	1,34	transducin family protein / WD-40 repeat family protein
At1g29200	1,34	DNA-binding protein GT-1-related
At3g49590	1,34	expressed protein
At1g08030	1,34	hypothetical protein
At4g27780	1,34	subtilisin-like serine protease-related
At1g47270	1,34	F-box family protein
At4g25220	1,34	autophagy 8c (APG8c)
At3g22810	1,34	DEAD/DEAH box helicase, putative
At4g38810	1,34	AP2 domain-containing transcription factor, putative
At1g50040	1,34	expressed protein
At3g15150	1,34	expressed protein
At4g12570	1,34	self-incompatibility protein-related
At3g01660	1,34	expressed protein
At5g46550	1,34	cytochrome P450 family protein
At5g66985	1,34	amino acid transporter family protein
At4g28000	1,34	expressed protein
At2g40820	1,34	expressed protein
At5g40330	1,34	expressed protein
At4g21370	1,34	hypothetical protein
At5g57140	1,34	acyl-(acyl-carrier-protein) desaturase, putative / stearoyl-ACP desaturase, putative
At1g73350	1,34	enoyl-CoA hydratase/isomerase family protein
At1g18160	1,34	gibberellin response modulator (GAI) (RGA2) / gibberellin-responsive modulator
At1g29820	1,33	CACTA-like transposase family (Ptta/En/Spm)
At1g29350	1,33	galactosyltransferase family protein
At1g31690	1,33	leucine-rich repeat transmembrane protein kinase, putative
At3g04030	1,33	expressed protein
At1g14660	1,33	eukaryotic translation initiation factor SUI1, putative
At3g19570	1,33	RNase P subunit p30 family protein
At5g50360	1,33	expressed protein
At5g49210	1,33	serine/threonine protein kinase (RKF2)
At3g23240	1,33	auxin-responsive family protein
At5g07450	1,33	importin beta-2 subunit family protein
At4g05510	1,33	membrane protein, putative
At2g11650	1,33	ubiquitin-conjugating enzyme, putative
At1g47870	1,33	heat shock transcription factor family protein
At1g11220	1,33	sugar-porter family protein 2 (SFP2)
At5g05060	1,33	hypothetical protein
At2g06005	1,33	exocyst subunit EXO70 family protein
At5g51240	1,33	expressed protein
At5g28280	1,33	pre-mRNA cleavage complex family protein
At4g29800	1,33	iron-deficiency-responsive protein, putative
At2g01910	1,33	transferase family protein
At4g05270	1,33	glutaredoxin family protein
At1g20670	1,33	armadillo/beta-catenin repeat family protein / F-box family protein
At4g00690	1,33	pentatricopeptide (PPR) repeat-containing protein
At5g49330	1,33	hypothetical protein
At5g52740	1,33	membrane protein, putative
At3g24440	1,33	phenylalanyl-tRNA synthetase class IIc family protein
At1g67710	1,33	DC1 domain-containing protein
At1g74140	1,33	short-chain dehydrogenase/reductase (SDR) family protein
At3g63070	1,33	expressed protein
At1g15530	1,33	glucose-methanol-choline (GMC) oxidoreductase family protein
At1g49810	1,33	hypothetical protein
At5g08420	1,33	heavy-metal-associated domain-containing protein
At4g24820	1,33	expressed protein
At1g33590	1,33	transcriptional factor B3 family protein
At4g00350	1,33	glycosyl hydrolase family 1 protein
At1g43640	1,33	calmodulin-binding family protein
At1g65440	1,33	expressed protein
At5g52390	1,33	ABC transporter family protein
At1g04580	1,33	senescence-associated protein-related
At2g20500	1,33	phosphate-responsive protein, putative
At2g46780	1,33	expressed protein

At1g19050	1,33	aspartyl protease family protein
At1g07020	1,33	zinc finger (C3HC4-type RING finger) family protein
At5g53100	1,33	myb family transcription factor (MYB36)
At5g55600	1,33	expressed protein
At5g12020	1,33	basic helix-loop-helix (bHLH) family protein
At2g28440	1,33	mevalonate diphosphate decarboxylase, putative
At1g32700	1,33	6-4 photolyase (UVR3)
At5g35660	1,33	expressed protein
At5g10490	1,33	expressed protein
At1g73220	1,33	expressed protein
At5g27390	1,33	protein kinase family protein
At4g11300	1,33	zinc finger (GATA type) family protein
At2g31130	1,33	SEUSS transcriptional co-regulator
At3g58670	1,33	KOW domain-containing transcription factor family protein
At3g10580	1,33	short-chain dehydrogenase/reductase (SDR) family protein
At1g76850	1,33	expressed protein
At5g12050	1,33	ATP-dependent DNA helicase, putative
At5g49580	1,33	expressed protein
At2g32210	1,33	beta-amylase (CT-BMY) / 1,4-alpha-D-glucan maltohydrolase
At5g18750	1,33	expressed protein
At1g04090	1,33	expressed protein
At4g18560	1,33	U3 ribonucleoprotein (Utp) family protein
At4g37890	1,33	betaine-aldehyde dehydrogenase, putative
At3g63400	1,33	cytochrome P450, putative
At5g13600	1,33	expressed protein
At1g25280	1,33	expressed protein
At2g23100	1,33	centroradialis protein, putative (CEN)
At4g34660	1,33	hypothetical protein
At5g09230	1,33	glycosyltransferase family protein 8
At1g72390	1,33	expressed protein
At3g54250	1,33	glycerophosphoryl diester phosphodiesterase family protein
At1g54770	1,33	expressed protein
At1g76110	1,33	Dof-type zinc finger domain-containing protein
At1g56310	1,33	diacylglycerol kinase family protein
At2g06160	1,33	calcium-dependent protein kinase 19 (CDPK19)
At1g30210	1,33	pentatricopeptide (PPR) repeat-containing protein
At5g51390	1,33	transducin family protein / WD-40 repeat family protein
At3g06370	1,33	expressed protein
At2g15720	1,32	auxin transport protein, putative (PIN3)
At1g35750	1,32	expressed protein
At3g21790	1,32	gibberellin-regulated protein 1 (GASA1) / gibberellin-responsive protein 1
At1g78820	1,32	SET domain-containing protein (ASHH1)
At5g20610	1,32	glutathione S-transferase, putative
At5g08740	1,32	expressed protein
At3g44830	1,32	expressed protein
At5g02470	1,32	GTP-binding protein (SAR1A)
At1g58120	1,32	subtilase family protein
At4g02210	1,32	expressed protein
At5g67460	1,32	AP2 domain-containing transcription factor, putative
At5g13310	1,32	auxin-responsive protein, putative
At3g44270	1,32	expressed protein
At3g06140	1,32	armadillo/beta-catenin repeat family protein / kinesin motor family protein
At1g18170	1,32	glycosyl hydrolase family 1 protein
At4g17785	1,32	expressed protein
At1g32040	1,32	zinc finger (B-box type) family protein
At2g29790	1,32	F-box family protein / SKP1 interacting partner 3-related
At4g31670	1,32	60S ribosomal protein L29 (RPL29A)
At1g07980	1,32	pentatricopeptide (PPR) repeat-containing protein
At3g49310	1,32	hypothetical protein
At1g11320	1,32	no apical meristem (NAM) family protein
At3g42950	1,32	hypothetical protein
At4g07920	1,32	protease-associated zinc finger (C3HC4-type RING finger) family protein
At1g18710	1,32	hydroxyproline-rich glycoprotein family protein
At4g00300	1,32	calcium-dependent protein kinase, putative / CDPK, putative
At4g02320	1,32	60S ribosomal protein L7 (RPL7D)
At1g14260	1,32	hypothetical protein
At1g72950	1,32	expressed protein
At5g13620	1,32	protein kinase family protein
At4g27670	1,32	F-box family protein
At5g56980	1,32	no apical meristem (NAM) family protein
At1g80740	1,32	expressed protein
At5g37380	1,32	expressed protein
At5g42900	1,32	guanine nucleotide exchange family protein
At1g05770	1,32	vacuolar ATP synthase, putative / V-ATPase, putative / vacuolar proton pump, putative
At3g23250	1,32	Member of the R2R3 factor gene family.

At2g17940	1,32	expressed protein
At2g42080	1,32	calmodulin-related protein, putative
At2g36480	1,32	2-oxoglutarate-dependent dioxygenase, putative
At1g35995	1,32	calcium-dependent protein kinase, putative / CDPK, putative
At4g14020	1,32	amino acid transporter family protein
At4g02430	1,32	expressed protein
At4g28550	1,32	inward rectifying potassium channel (KAT1)
At5g05290	1,32	zinc finger (C2H2 type) family protein
At3g31970	1,32	DEAD/DEAH box helicase, putative
At1g69760	1,32	cell division control protein CDC6, putative
At5g65140	1,32	F-box family protein
At1g05250	1,32	glucosamine/galactosamine-6-phosphate isomerase family protein
At1g16705	1,32	glycoprotease M22 family protein
At3g57040	1,32	protein phosphatase 2C, putative / PP2C, putative
At5g11530	1,32	Involved in regulating reproductive development
At2g19310	1,32	no apical meristem (NAM) family protein
At5g01160	1,32	light-mediated development protein 1 / deetiolated1 (DET1)
At2g28660	1,32	hypothetical protein
At5g01670	1,32	expansin, putative (EXP11)
At3g30350	1,32	leucine-rich repeat protein kinase, putative
At5g24280	1,32	pseudogene, pentatricopeptide (PPR) repeat-containing protein
At5g59080	1,32	transporter-related
At2g31170	1,32	basic helix-loop-helix (bHLH) family protein
At4g18410	1,32	poly (ADP-ribose) polymerase family protein
At1g36150	1,32	pectinesterase family protein
At5g07200	1,32	COP9 signalosome subunit 5B / CSN subunit 5B (CSN5B) / c-JUN coactivator protein AJH1
At5g20760	1,32	glycosyl transferase family 2 protein
At5g04270	1,32	expressed protein
At4g39840	1,32	HNH endonuclease domain-containing protein
At4g36440	1,32	expressed protein
At1g02620	1,32	pre-mRNA splicing factor, putative / SR1 protein, putative
At1g23540	1,32	pectate lyase family protein
At5g26696	1,32	expressed protein
At2g40680	1,32	expressed protein
At3g05510	1,32	expressed protein
At2g21490	1,32	alcohol dehydrogenase, putative
At2g46680	1,32	preprotein translocase secA subunit, putative
At3g06830	1,32	inorganic phosphate transporter (PHT3)
At3g12790	1,32	3-hydroxybutyryl-CoA dehydrogenase, putative
At1g01920	1,32	mitochondrial substrate carrier family protein
At4g23740	1,32	myb family transcription factor (MYB46)
At1g55315	1,32	expressed protein
At3g43390	1,32	transcription initiation factor IID (TFIID) subunit A family protein
At4g14230	1,32	repair endonuclease family protein
At4g05460	1,32	cyclin, putative (SDS)
At1g80590	1,32	transducin family protein / WD-40 repeat family protein
At5g49560	1,32	zinc finger (MYND type) family protein
At1g57670	1,32	glycosyl hydrolase family 79 N-terminal domain-containing protein
At1g69160	1,32	high mobility group (HMG1/2) family protein
At1g17460	1,32	myb family transcription factor
At5g64780	1,32	transcription factor, putative
At2g14650	1,32	calcium-binding EF hand family protein
At1g61380	1,32	expressed protein
At3g26910	1,32	zinc finger (C2H2 type) family protein
At1g10230	1,32	hypothetical protein
At4g17460	1,31	expressed protein
At5g52630	1,31	expressed protein
At2g24640	1,31	expressed protein
At3g13270	1,31	expressed protein
At1g40710	1,31	expressed protein
At2g17590	1,31	L-ascorbate peroxidase, chloroplast, putative
At3g02910	1,31	hydrolase, alpha/beta fold family protein
At5g07540	1,31	expressed protein
At5g06400	1,31	expressed protein
At2g16590	1,31	signal recognition particle receptor alpha subunit family protein
At5g66050	1,31	expressed protein
At1g56145	1,31	terpene synthase/cyclase family protein
At2g26190	1,31	expressed protein
At1g68240	1,31	myosin heavy chain-related
At1g32560	1,31	translation elongation factor Ts (EF-Ts), putative
At3g59080	1,31	F-box family protein-related
At3g05100	1,31	early-responsive to dehydration protein-related / ERD protein-related
At4g19660	1,31	plasma membrane intrinsic protein 1A (PIP1A) / aquaporin PIP1.1 (PIP1.1) (AQ1)
At2g21610	1,31	expressed protein
At3g14520	1,31	aminotransferase, putative

At5g47950	1,31	expressed protein
At1g16440	1,31	expressed protein
At3g52470	1,31	glycosyl hydrolase family 18 protein
At3g56500	1,31	14-3-3 protein GF14 psi (GRF3) (RCI1)
At3g06440	1,31	nodulin MtN21 family protein
At2g24230	1,31	plastocyanin-like domain-containing protein
At2g03320	1,31	zinc finger (C2H2 type) family protein (ZAT11)
At2g19060	1,31	phagocytosis and cell motility protein ELMO1-related
At2g17900	1,31	glucosamine/galactosamine-6-phosphate isomerase-related
At3g19020	1,31	expressed protein
At2g03890	1,31	Phosphoinositide kinase which undergo autophosphorylation and phosphorylate serine/threonine
At3g03870	1,31	disease resistance protein (CC-NBS-LRR class), putative
At2g29920	1,31	pseudogene, protein phosphatase 2C
At5g04450	1,31	AMP-binding protein, putative
At4g26160	1,31	Encodes a member of the thioredoxin family protein
At5g02910	1,31	hypothetical protein
At1g08810	1,31	putative transcription factor of the R2R3-MYB gene family.
At4g38400	1,31	expressed protein
At1g48760	1,31	peroxidase 27 (PER27) (P27) (PRXR7)
At3g04620	1,31	leucine-rich repeat transmembrane protein kinase, putative
At3g24680	1,31	zinc finger (C3HC4-type RING finger) family protein
At3g44730	1,31	eukaryotic translation initiation factor 2B family protein / eIF-2B family protein
At3g62910	1,31	ncodes a plastid-localized ribosome release factor 1 that is essential in chloroplast development
At4g27190	1,31	armadillo/beta-catenin repeat family protein
At4g34220	1,31	beta-expansin, putative (EXBP2)
At5g57160	1,31	fasciclin-like arabinogalactan-protein (FLA12)
At1g11080	1,31	syntaxin, putative
At1g26260	1,31	hydrolase, alpha/beta fold family protein
At4g36410	1,31	pentatricopeptide (PPR) repeat-containing protein
At1g14440	1,31	epsin N-terminal homology (ENTH) domain-containing protein / clathrin assembly protein-related
At1g24540	1,31	thaumatin-like protein, putative
At1g06160	1,31	lipase, putative
At5g48250	1,31	exocyst subunit EXO70 family protein
At2g27990	1,31	protein kinase family protein
At1g55550	1,31	glutamate receptor family protein (GLR2.6)
At2g21070	1,31	nodulin MtN3 family protein
At3g24506	1,31	expressed protein
At5g37030	1,31	polyadenylate-binding protein, putative / PABP, putative
At2g17820	1,31	pfkB-type carbohydrate kinase family protein
At5g19300	1,31	pentatricopeptide (PPR) repeat-containing protein
At5g04180	1,31	protein kinase family protein
At1g77750	1,31	dihydrodipicolinate synthase 1 (DHDPS1) (DHDPS) (DHPS1)
At2g31350	1,31	chromatin protein family
At1g17300	1,31	glutathione peroxidase, putative
At4g39720	1,31	galactosyl transferase GMA12/MNN10 family protein
At3g05640	1,31	myb family transcription factor
At2g14580	1,31	expressed protein
At2g12900	1,31	serine carboxypeptidase S28 family protein
At2g21230	1,31	hypothetical protein
At1g51450	1,31	RabGAP/TBC domain-containing protein
At4g24780	1,31	myb family transcription factor (MYB116)
At4g13420	1,31	expressed protein
At4g29140	1,31	zinc finger (C3HC4-type RING finger) family protein
At3g12460	1,31	family II extracellular lipase 4 (EXL4)
At4g12120	1,31	glycosyl hydrolase family 1 protein
At1g58170	1,31	expressed protein
At1g67510	1,31	nodulin, putative
At5g15840	1,31	dihydrodipicolinate reductase family protein
At2g16380	1,31	zinc finger (DHHC type) family protein
At1g01500	1,31	VQ motif-containing protein
At1g79790	1,31	vernalization 2 protein (VRN2)
At2g35770	1,31	embryogenesis-associated protein-related
At5g32440	1,31	F-box family protein
At3g05370	1,31	galactinol synthase, putative
At4g05570	1,31	exostosin family protein
At1g24600	1,31	beta-ketoacyl-CoA synthase family protein
At2g35210	1,31	ankyrin repeat family protein / BTB/POZ domain-containing protein
At3g26140	1,31	dehydrin, putative
At5g48260	1,30	expressed protein
At1g27640	1,30	DNA-directed RNA polymerase, chloroplast (RPOPT)
At5g06570	1,30	CHP-rich zinc finger protein, putative
At2g29490	1,30	expressed protein
At5g53540	1,30	transporter-related
At5g05220	1,30	holocarboxylase synthetase 2 (HCS2.d)
At1g78170	1,30	hypothetical protein

At1g30090	1,30	oxidoreductase, 2OG-Fe(II) oxygenase family protein
At4g12070	1,30	expressed protein
At1g42650	1,30	cytochrome P450, putative
At3g05030	1,30	fringe-related protein
At3g45620	1,30	Pep3/Vps18/deep orange family protein
At1g36900	1,30	protease inhibitor/seed storage/lipid transfer protein (LTP) family protein
At3g12950	1,30	calreticulin 1 (CRT1)
At2g24350	1,30	sulfotransferase family protein
At3g24780	1,30	chaperonin, putative
At5g65360	1,30	C2 domain-containing protein
At4g02200	1,30	laccase family protein / diphenol oxidase family protein
At5g56140	1,30	glucose-methanol-choline (GMC) oxidoreductase family protein
At1g27430	1,30	protein kinase family protein
At5g24490	1,30	zinc finger protein (PRAF1) / regulator of chromosome condensation (RCC1) family protein
At1g74250	1,30	peptidyl-prolyl cis-trans isomerase cyclophilin-type family protein
At4g27140	1,30	protein kinase family protein
At5g55550	1,30	dihydrodipicolinate synthase 2 (DHDPS2)
At1g21780	1,30	leucine-rich repeat transmembrane protein kinase, putative
At1g27570	1,30	SEC14 cytosolic factor family protein / phosphoglyceride transfer family protein
At1g62820	1,30	hypothetical protein
At3g21400	1,30	plastocyanin-like domain-containing protein
At5g50335	1,30	imidazoleglycerol-phosphate dehydratase 1 (IGPD1)
At3g15520	1,30	expressed protein
At1g73380	1,30	disease resistance response protein-related/ dirigent protein-related
At1g56020	1,30	hypothetical protein
At3g54980	1,30	germin-like protein, putative
At1g10920	1,30	expressed protein
At5g19420	1,30	thioredoxin family protein
At3g21020	1,30	copper homeostasis factor, putative / copper chaperone, putative (CCH)
At3g06030	1,30	histone deacetylase family protein (HDA18)
At5g11780	1,30	snRNA activating complex family protein
At2g01100	1,30	polyadenylate-binding protein, putative / PABP, putative
At2g33820	1,30	calmodulin, putative
At1g70060	1,30	ribonuclease 3 (RNS3)
At2g47930	1,30	DNAJ heat shock N-terminal domain-containing protein (J11)
At1g62970	1,30	ubiquitin carboxyl-terminal hydrolase family 1 protein
At1g67120	1,30	expressed protein
At3g46570	1,30	MADS-box protein (AGL38)
At4g13980	1,30	pentatricopeptide (PPR) repeat-containing protein
At4g01400	1,30	Hypothetical protein, complete cds, clone: RAFL16-43-P18
At3g51760	1,30	expressed protein
At5g17800	1,30	germin-like protein (GLP8)
At4g12930	1,30	expressed protein
At1g56670	1,30	translation initiation factor IF-1, chloroplast, putative
At1g73360	1,30	expressed protein
At1g10170	1,30	copper transporter 1 (COPT1)
At5g39890	1,30	expressed protein
At3g43320	1,30	universal stress protein (USP) family protein
At4g33240	1,30	hypothetical protein
At3g17020	1,30	Adenine nucleotide alpha hydrolases-like superfamily protein
At2g31830	1,30	hypothetical protein
At2g37640	1,30	mitogen-activated protein kinase, putative / MAPK, putative (MPK16)
At4g31875	1,30	disease resistance protein (CC-NBS-LRR class), putative
At3g28200	1,30	expressed protein
At1g08620	1,30	expressed protein
At3g26360	1,30	subtilase family protein
At2g48080	1,30	expressed protein
At3g06760	1,30	expressed protein
At3g16150	1,30	inositol monophosphatase family protein
At3g61210	1,30	invertase/pectin methylesterase inhibitor family protein
At3g47660	1,30	WD-40 repeat family protein / katanin p80 subunit, putative
At5g59700	1,30	expressed protein
At3g19540	1,30	kinesin motor family protein (NACK2)
At4g33150	1,30	expressed protein
At2g30320	1,30	expressed protein
At5g65090	1,30	expressed protein
At5g65570	1,30	hypothetical protein
At3g29030	1,30	hypothetical protein
At3g25250	1,30	hydrolase, alpha/beta fold family protein
At1g04990	1,30	subtilase family protein
At1g35260	1,30	expressed protein
At1g59750	1,30	glutamine amidotransferase class-I domain-containing protein
At1g66920	1,30	phosphate translocator-related
At1g05410	1,30	AAA-type ATPase family protein
At3g16320	1,30	Ulp1 protease family protein

At2g29090	1,30	Rieske (2Fe-2S) domain-containing protein
At4g17980	1,30	expressed protein (APS2)
At1g53350	1,30	F-box family protein
At4g37710	1,30	two-component responsive regulator family protein / response regulator family protein
At5g38400	1,30	expressed protein
At1g31930	1,30	lysine decarboxylase family protein
At4g17960	1,30	terpene synthase/cyclase family protein
At5g67260	1,30	aminoacylase, putative / N-acyl-L-amino-acid amidohydrolase, putative
At5g48485	1,30	expressed protein
At2g18120	1,30	pyruvate dehydrogenase (lipoamide) kinase (PDHK)
At1g49190	1,30	urophorphyrin III methylase (UPM1)
At5g67530	1,30	prephenate dehydratase family protein
At3g47320	1,30	gibberellin 2-oxidase / GA2-oxidase (GA2OX3)
At5g27450	1,30	protease inhibitor/seed storage/lipid transfer protein (LTP) family protein
At1g12460	1,30	hypothetical protein
At5g19090	1,30	expressed protein
At1g78410	1,30	expressed protein
At5g16680	1,30	expressed protein
At5g07580	1,30	leucine-rich repeat transmembrane protein kinase, putative
At1g06580	1,30	inosine-uridine preferring nucleoside hydrolase family protein
At4g04710	1,30	hypothetical protein
At3g44200	1,30	FAD-binding domain-containing protein
At1g05740	1,30	lectin protein kinase, putative

mutant *lsm8*

Transcript ID	stabilization fold	Gene Title
At5g25280	2,52	cytochrome P450 family protein
At4g11280	2,48	disease resistance protein RPM1 (CC-NBS-LRR class), putative
At1g78070	2,48	transducin family protein / WD-40 repeat family protein
At5g62520	2,30	zinc finger protein-related
At1g79310	2,28	ARID/BRIGHT DNA-binding domain-containing protein / ELM2 domain-containing protein
At3g48360	2,26	auxin-responsive family protein
At1g43160	2,24	pseudo-response regulator 2 (APRR2) (TOC2)
At5g40910	2,24	latex-abundant family protein (AMC1) / caspase family protein
At5g43270	2,14	kelch repeat-containing F-box family protein
At5g63770	2,13	omega-3 fatty acid desaturase, endoplasmic reticulum (FAD3)
At2g34600	2,06	autophagy 8e (APG8e)
At1g61820	2,02	expressed protein
At1g08920	2,01	peroxidase, putative
At2g32150	2,01	leucine-rich repeat protein kinase, putative
At3g44970	1,98	hypothetical protein
At5g06300	1,98	SNF2 domain-containing protein / helicase domain-containing protein
At3g25830	1,96	eukaryotic translation initiation factor 3 subunit 2 / TGF-beta receptor interacting protein 1 / eIF3i
At2g20670	1,95	lipin family protein
At2g40180	1,95	leucine-rich repeat family protein / protein kinase family protein
At5g28080	1,95	adenylate kinase family protein
At1g80840	1,94	SIN-like family protein
At4g16860	1,93	expressed protein
At3g55980	1,87	expressed protein
At5g66900	1,86	prenylated rab acceptor (PRA1) protein-related
At4g18010	1,86	pectinesterase family protein
At5g62570	1,86	SET domain-containing protein
At1g12610	1,81	Encodes a member of the DREB subfamily A-1 of ERF/AP2 transcription factor family (DDF1)
At1g07150	1,81	expressed protein
At1g72510	1,79	expressed protein
At2g38470	1,79	S-locus protein kinase, putative
At2g05510	1,79	expressed protein
At1g56510	1,78	expressed protein
At3g25820	1,78	suppressor of lin-12-like protein-related / sel-1 protein-related
At5g60890	1,77	histone acetyltransferase 4 (HAC4)
At5g08350	1,76	serine carboxypeptidase S10 family protein
At2g35715	1,76	homeobox-leucine zipper protein 2 (HAT2) / HD-ZIP protein 2
At1g13350	1,75	endo-1,4-beta-glucanase / cellulase (CEL2)
At2g25900	1,75	protein phosphatase 2C-related / PP2C-related
At5g54730	1,75	bZIP transcription factor family protein
At4g31800	1,75	calmodulin-2/3/5 (CAM3)
At1g61340	1,74	expressed protein
At4g01950	1,74	zinc finger (DHHC type) family protein
At3g62260	1,74	DGCR14-related
At4g18340	1,73	pectate lyase family protein
At4g29140	1,71	zinc finger (C3HC4-type RING finger) family protein
At5g63970	1,71	sugar transporter, putative
At2g01880	1,70	sugar transporter family protein
At3g15500	1,70	ribosomal protein S7 family protein
At4g29950	1,70	multi-copper oxidase, putative (SKU5)
At1g12710	1,69	expressed protein
At5g66210	1,69	pentatricopeptide (PPR) repeat-containing protein
At5g02230	1,68	expressed protein
At4g17970	1,68	acyl-(acyl carrier protein) thioesterase/acyl-ACP thioesterase/oleoyl-(acyl-carrier protein) hydrolase
At2g40000	1,68	ortholog of sugar beet HS1 PRO-1 2 (HSPRO2)
At2g31945	1,67	expressed protein
At5g54690	1,67	RNA polymerase Rpb7 N-terminal domain-containing protein
At3g23430	1,67	expressed protein
At5g52050	1,67	hypothetical protein
At5g46470	1,67	expressed protein
At2g24100	1,66	aldehyde oxidase, putative
At5g46510	1,66	calmodulin-2/3/5 (CAM5) (TCH1)
At4g34410	1,66	membrane protein, putative
At1g33700	1,66	expressed protein
At5g40340	1,66	expressed protein
At5g45340	1,66	actin-depolymerizing factor 4 (ADF4)
At3g07340	1,66	hypothetical protein
At5g65630	1,64	gibberellin 20-oxidase family protein
At4g24060	1,64	multi-copper oxidase type I family protein
At4g12490	1,63	expressed protein
At4g12080	1,63	AT-hook motif nuclear-localized protein 1 (AHL1)
At4g29780	1,63	expressed protein
At2g35710	1,62	GATA transcription factor 3, putative (GATA-3)

At1g58170	1,62	expressed protein
At3g23410	1,62	thylakoid lumenal 17.9 kDa protein, chloroplast
At3g07350	1,61	hypothetical protein
At4g18050	1,61	expressed protein
At3g10500	1,61	F-box family protein (FBL19)
At2g18440	1,61	bacterial transferase hexapeptide repeat-containing protein
At2g22770	1,60	cytochrome P450 71A16, putative (CYP71A16)
At5g66320	1,60	hypothetical protein
At1g55020	1,60	serine/threonine protein kinase family protein
At1g61560	1,60	mitochondrial substrate carrier family protein
At3g59310	1,60	F-box family protein
At3g62550	1,59	Adenine nucleotide alpha hydrolases-like superfamily protein
At3g26200	1,59	MADS-box family protein
At5g04370	1,59	NAD-dependent epimerase/dehydratase family protein
At5g38140	1,59	pentatricopeptide (PPR) repeat-containing protein
At1g33720	1,59	disease resistance protein (TIR-NBS-LRR class), putative
At2g21510	1,59	hypothetical protein
At4g27410	1,58	expressed protein
At1g11380	1,58	cytochrome P450, putative
At1g60270	1,58	short-chain dehydrogenase/reductase (SDR) family protein
At3g16800	1,58	vacuolar sorting receptor, putative
At2g20625	1,58	glutaredoxin family protein
At1g11210	1,58	pentatricopeptide (PPR) repeat-containing protein
At2g25720	1,57	calcium-dependent protein kinase, putative / CDPK, putative
At1g63880	1,57	cation/hydrogen exchanger, putative (CHX24)
At1g53440	1,57	protein phosphatase 2C family protein / PP2C family protein
At2g22980	1,57	protein kinase, putative
At5g65660	1,57	ethylene-responsive protein, putative
At4g15230	1,57	pentatricopeptide (PPR) repeat-containing protein
At3g62010	1,57	ERF domain protein 12 (ERF12)
At3g06500	1,57	kelch repeat-containing F-box family protein
At1g74810	1,56	myb family transcription factor (MYB108)
At1g62990	1,56	hypothetical protein
At1g56120	1,56	cytidine/deoxycytidylate deaminase family protein
At5g63480	1,56	chaperonin, putative
At5g54040	1,56	pentatricopeptide (PPR) repeat-containing protein
At2g42890	1,56	ankyrin repeat family protein
At1g30135	1,56	disease resistance protein (TIR-NBS-LRR class), putative
At2g30040	1,56	transcription regulator NOT2/NOT3/NOT5 family protein
At1g03620	1,56	serine carboxypeptidase S10 family protein
At5g43180	1,56	superoxide dismutase (Cu-Zn) (SODCC) / copper/zinc superoxide dismutase (CSD1)
At1g76410	1,56	ATL8; zinc ion binding
At3g12090	1,55	alpha, alpha-trehalose-phosphate synthase, UDP-forming, putative/trehalose-6-phosphate synthase
At4g37610	1,55	pumilio/Puf RNA-binding domain-containing protein
At4g08170	1,55	ribosomal protein S6 family protein
At4g16880	1,55	DNA replication helicase, putative
At4g39770	1,55	Haloacid dehalogenase-like hydrolase (HAD) superfamily protein
At3g05320	1,55	DNAJ heat shock N-terminal domain-containing protein
At4g37410	1,54	phospholipase D zeta1 / PLDzeta1 (PLDP1)
At3g11410	1,54	expressed protein
At1g70290	1,54	hypothetical protein
At3g54260	1,54	phosphoesterase family protein
At5g59780	1,54	leucine-rich repeat transmembrane protein kinase, putative
At5g62920	1,54	eukaryotic translation initiation factor 2 subunit 2, putative / eIF-2-beta, putative
At1g03520	1,53	DNA-binding protein-related
At3g13674	1,53	cation/hydrogen exchanger, putative (CHX9)
At5g15790	1,53	malate dehydrogenase, cytosolic, putative
At1g53180	1,53	zinc finger (CCCH-type) family protein
At5g67440	1,53	hypothetical protein
At5g59080	1,53	transporter-related
At5g52750	1,52	protein kinase, putative
At3g61380	1,52	lipid transfer protein 3 (LTP3)
At5g09980	1,52	elicitor peptide 4 precursor (PROPEP4)
At4g22212	1,52	expressed protein
At5g04790	1,52	expressed protein
At2g28085	1,52	MATE efflux family protein
At4g20320	1,52	expressed protein
At1g74400	1,52	pyruvate kinase, putative
At1g73540	1,52	expressed protein
At5g66070	1,52	GDP-mannose pyrophosphorylase, putative
At1g11670	1,51	late embryogenesis abundant group 1 domain-containing protein / LEA group 1
At5g67080	1,51	galactosyltransferase family protein
At5g64000	1,51	expressed protein
At2g28110	1,51	F-box family protein (FBW2)
At1g17990	1,51	DNA repair protein RAD23, putative

At5g10695	1,51	nucleoside diphosphate kinase 4 (NDK4)
At3g07360	1,51	calmodulin-binding protein
At2g30020	1,51	splicing factor PWI domain-containing protein
At4g31000	1,51	thiazole biosynthetic enzyme, chloroplast (ARA6) (THI1) (THI4)
At4g36830	1,50	beta-1,2-N-acetylglucosaminyltransferase II
At4g24380	1,50	10-formyltetrahydrofolate biosynthetic process, folic acid and derivative biosynthetic process
At1g44910	1,50	amine oxidase family protein
At5g24470	1,50	FtsH protease, putative
At3g14570	1,50	pinoresinol-lariciresinol reductase, putative
At2g03280	1,50	Kin17 DNA-binding protein-related
At3g28740	1,50	exopolygalacturonase / galacturan 1,4-alpha-galacturonidase / pectinase
At3g15760	1,50	cyanate lyase family
At1g32450	1,50	expressed protein
At1g72700	1,50	pseudogene, hypothetical protein
At1g62300	1,50	RNA helicase, putative
At1g22550	1,50	hypothetical protein
At1g58842	1,49	ABC transporter family protein
At2g42870	1,49	terpene synthase/cyclase family protein
At3g50280	1,49	hypothetical protein
At2g28305	1,49	expressed protein
At3g59740	1,49	glycosyl hydrolase family 1 protein
At1g14480	1,49	transducin family protein / WD-40 repeat family protein
At1g76900	1,49	WD-40 repeat family protein / phytochrome A-related
At1g14120	1,49	hypothetical protein
At1g72920	1,49	Rac-like GTP-binding protein (ARAC8)
At3g44630	1,49	leucine-rich repeat transmembrane protein kinase, putative
At1g49840	1,49	chloroplast outer membrane protein, putative
At1g07135	1,49	peroxiredoxin type 2, putative
At5g65280	1,49	beta-fructofuranosidase, putative / invertase, putative / saccharase, putative / beta-fructosidase
At5g66400	1,49	expressed protein
At1g63030	1,49	cysteine proteinase, putative
At5g13180	1,49	auxin-responsive protein, putative
At5g56160	1,48	expressed protein
At3g21260	1,48	transcriptional factor B3 family protein
At3g53480	1,48	prolifera protein (PRL) / DNA replication licensing factor Mcm7 (MCM7)
At4g30460	1,48	NADH-ubiquinone oxidoreductase-related
At5g61900	1,48	DNAJ heat shock N-terminal domain-containing protein
At2g21060	1,48	disease resistance-responsive family protein / dirigent family protein
At3g23870	1,48	transducin family protein / WD-40 repeat family protein
At1g78290	1,48	secretory carrier membrane protein (SCAMP) family protein
At3g51860	1,48	cinnamoyl-CoA reductase-related
At4g37400	1,48	shaggy-related protein kinase kappa, putative / ASK-kappa, putative
At4g00970	1,48	leucine-rich repeat transmembrane protein kinase, putative
At3g02390	1,48	expressed protein
At5g62770	1,48	actin-depolymerizing factor, putative
At5g25110	1,48	expressed protein
At1g80440	1,48	F-box family protein
At1g10480	1,48	ethylene-responsive element-binding family protein
At1g33055	1,47	hydroxyproline-rich glycoprotein family protein
At1g69890	1,47	WRKY family transcription factor
At5g22700	1,47	NOL1/NOP2/sun family protein
At2g44500	1,47	AP2 domain-containing transcription factor, putative
At5g01670	1,47	expansin, putative (EXP11)
At3g43430	1,47	beta-fructosidase, putative / beta-fructofuranosidase, putative
At5g60200	1,47	hydrolase, alpha/beta fold family protein
At3g26960	1,47	Pollen Ole e 1 allergen and extensin family protein
At4g35985	1,46	Senescence/dehydration-associated protein-related
At5g13700	1,46	multi-copper oxidase type I family protein
At1g77210	1,46	pentatricopeptide (PPR) repeat-containing protein
At1g05560	1,46	nucleolar protein, putative
At2g48110	1,46	leucine-rich repeat transmembrane protein kinase, putative
At4g18950	1,46	citrate synthase, mitochondrial, putative
At1g09460	1,46	aminoacylase, putative / N-acyl-L-amino-acid amidohydrolase, putative
At2g03530	1,46	KOW domain-containing protein / D111/G-patch domain-containing protein
At5g41080	1,46	expressed protein
At4g35440	1,46	5'-AMP-activated protein kinase beta-1 subunit-related
At1g49230	1,46	Ran-binding protein 1, putative / RanBP1, putative
At5g58700	1,46	amino acid transporter family protein
At2g36120	1,46	bHLH family protein
At2g01520	1,46	PHD finger protein-related
At2g23810	1,46	F-box family protein
At1g17380	1,46	protein kinase family protein
At1g77450	1,46	nitrate-responsive NOI protein, putative
At2g18370	1,46	WD-40 repeat protein family
At1g22190	1,46	expressed protein

At4g17800	1,46	protease inhibitor/seed storage/lipid transfer protein (LTP) family protein
At3g45960	1,46	NADH dehydrogenase-related
At5g24290	1,45	pentatricopeptide (PPR) repeat-containing protein
At4g01680	1,45	small nuclear ribonucleoprotein F, putative / snRNP-F, putative / Sm protein F, putative
At3g62040	1,45	basic helix-loop-helix (bHLH) family protein
At3g11340	1,45	glutathione S-transferase, putative
At2g41640	1,45	expressed protein
At3g52800	1,45	monosaccharide transporter, putative
At5g56870	1,45	serine/threonine protein kinase, putative
At3g21270	1,45	wound-responsive protein-related
At5g56750	1,45	cytochrome P450, putative
At1g61065	1,45	unknown function
At5g15190	1,44	expressed protein
At5g60800	1,44	pentatricopeptide (PPR) repeat-containing protein
At3g20400	1,44	esterase/lipase/thioesterase family protein
At4g15550	1,44	pentatricopeptide (PPR) repeat-containing protein
At5g14130	1,44	Peroxidase superfamily protein
At3g13430	1,44	RabGAP/TBC domain-containing protein
At1g26250	1,44	xyloglucan:xyloglucosyl transferase, putative / xyloglucan endotransglycosylase, putative (XTR7)
At3g52490	1,44	proton-dependent oligopeptide transport (POT) family protein
At3g57530	1,44	cold-shock DNA-binding family protein / glycine-rich protein (GRP2)
At1g76640	1,44	auxin-responsive protein / indoleacetic acid-induced protein 20 (IAA20)
At4g02540	1,44	hypothetical protein
At5g25630	1,44	acyl-CoA binding family protein
At1g64060	1,44	expressed protein
At2g26290	1,44	expressed protein
At1g66480	1,44	expressed protein
At1g05470	1,44	cyclic nucleotide-regulated ion channel, putative
At4g03205	1,44	expressed protein
At1g29280	1,44	pentatricopeptide (PPR) repeat-containing protein
At5g58720	1,44	dihydrolipoamide dehydrogenase 1, mitochondrial / lipoamide dehydrogenase 1 (MTLPD1)
At2g17975	1,44	Ras-related GTP-binding protein, putative
At2g36690	1,44	expressed protein
At1g69580	1,44	ethylene-responsive protein, putative
At5g37710	1,43	expressed protein
At4g22780	1,43	pentatricopeptide (PPR) repeat-containing protein
At2g18480	1,43	PWWP domain-containing protein
At5g50720	1,43	expressed protein
At1g21920	1,43	glycosyl hydrolase family 18 protein
At1g68580	1,43	no apical meristem (NAM) family protein
At2g28830	1,43	expressed protein
At4g37970	1,43	expressed protein
At3g51530	1,43	synbindin, putative
At3g05670	1,43	expressed protein
At1g56670	1,43	translation initiation factor IF-1, chloroplast, putative
At2g03240	1,43	short-chain dehydrogenase/reductase (SDR) family protein
At1g52890	1,43	cinnamyl-alcohol dehydrogenase family / CAD family
At4g24450	1,43	immunophilin / FKBP-type peptidyl-prolyl cis-trans isomerase family protein
At5g05790	1,43	NADH-ubiquinone oxidoreductase 20 kDa subunit, mitochondrial
At1g22870	1,43	transferase family protein
At1g75860	1,43	hypothetical protein
At5g41100	1,43	leucine-rich repeat family protein
At1g29090	1,43	transducin family protein / WD-40 repeat family protein
At1g52070	1,43	flavin-containing monooxygenase family protein / FMO family protein
At4g29690	1,43	peptidyl-prolyl cis-trans isomerase / cyclophilin-40 (CYP40) / rotamase
At1g61100	1,43	DC1 domain-containing protein / PHD finger protein-related
At2g25690	1,43	homeodomain-containing protein
At5g22920	1,43	40S ribosomal protein S4 (RPS4A)
At5g52120	1,42	ubiquitin fusion degradation UFD1 family protein
At4g08150	1,42	prolyl oligopeptidase family protein
At3g32270	1,42	acyl-CoA dehydrogenase-related
At2g31880	1,42	meprin and TRAF homology domain-containing protein / MATH domain-containing protein
At1g17340	1,42	phosphatidic acid phosphatase family protein / PAP2 family protein
At2g29380	1,42	pentatricopeptide (PPR) repeat-containing protein
At1g49520	1,42	glycosyl hydrolase family 17 protein
At1g17060	1,42	pentatricopeptide (PPR) repeat-containing protein
At3g48520	1,42	glycosyl hydrolase family 1 protein
At5g40450	1,42	expressed protein
At2g45910	1,42	GDSL-motif lipase/hydrolase family protein
At2g01460	1,42	expressed protein
At5g10550	1,42	gamma interferon responsive lysosomal thiol reductase family protein / GILT family protein
At5g11070	1,42	pseudogene, zinc knuckle (CCHC type) protein family
At2g01670	1,42	syntaxin 121 (SYP121) / syntaxin-related protein (SYR1)
At3g17700	1,42	MADS-box family protein
At1g76650	1,42	expressed protein

At5g14700	1,42	expressed protein
At1g16510	1,42	expressed protein
At3g56710	1,42	cationic peroxidase, putative
At2g38300	1,41	protease inhibitor/seed storage/lipid transfer protein (LTP) family protein
At2g30070	1,41	CTP synthase, putative / UTP--ammonia ligase, putative
At4g17615	1,41	clathrin coat assembly protein, putative
At1g53430	1,41	Leucine-rich repeat transmembrane protein kinase
At2g38760	1,41	kinetochore protein-related
At5g09460	1,41	expressed protein
At1g50600	1,41	early-responsive to dehydration protein-related / ERD protein-related
At1g32690	1,41	F-box family protein-related
At5g37500	1,41	origin recognition complex subunit 3-related / ORC3-related
At5g20820	1,41	UDP-glucoronosyl/UDP-glucosyl transferase family protein
At4g30470	1,41	calcium-dependent protein kinase isoform 6 (CPK6)
At4g23180	1,41	14-3-3 protein, putative / grf15, putative
At5g07270	1,41	expressed protein
At5g63370	1,41	1-aminocyclopropane-1-carboxylate synthase, putative / ACC synthase, putative
At3g48710	1,41	AIR synthase-related family protein
At5g48570	1,41	glucose-6-phosphate 1-dehydrogenase, putative / G6PD, putative
At2g14680	1,41	calpain-type cysteine protease family
At3g12710	1,41	synbindin, putative
At5g27950	1,41	expressed protein
At5g07580	1,41	leucine-rich repeat transmembrane protein kinase, putative
At3g19680	1,41	AP2 domain-containing transcription factor, putative
At5g64900	1,41	expressed protein
At1g63700	1,41	auxin-responsive protein, putative
At2g24120	1,41	auxin-responsive factor (ARF7)
At1g34280	1,41	expressed protein
At3g09420	1,41	Ras-related GTP-binding protein, putative
At1g32840	1,41	transcriptional factor B3 family protein
At1g74390	1,40	expressed protein
At5g24030	1,40	expressed protein
At1g67110	1,40	phosphate transporter family protein
At2g33790	1,40	cytochrome P450 family protein
At1g24330	1,40	expressed protein
At3g27210	1,40	pathogenesis-related thaumatin family protein
At1g21000	1,40	expressed protein
At4g25620	1,40	leucine-rich repeat transmembrane protein kinase, putative
At3g26910	1,40	zinc finger (C2H2 type) family protein
At5g04250	1,40	expressed protein
At1g54510	1,40	LOB domain protein 16 / lateral organ boundaries domain protein 16 (LBD16)
At4g18210	1,40	phosphoinositide phosphatase family protein
At5g13290	1,40	exonuclease RRP41 (RRP41)
At4g37260	1,40	expressed protein
At1g12950	1,40	senescence-associated protein-related
At2g39220	1,40	expressed protein
At1g23030	1,40	yippee family protein
At2g32140	1,40	pentatricopeptide (PPR) repeat-containing protein
At5g67480	1,39	peptidase M3 family protein / thimet oligopeptidase family protein
At1g53450	1,39	expressed protein
At1g69810	1,39	expressed protein
At1g70750	1,39	protein kinase family protein
At1g15740	1,39	transferase family protein
At2g40110	1,39	expressed protein
At5g66800	1,39	expressed protein
At3g03200	1,39	PHD finger family protein
At1g19050	1,39	aspartyl protease family protein
At1g25470	1,39	aldehyde dehydrogenase (ALDH2)
At2g17110	1,39	hypothetical protein
At1g49830	1,39	ankyrin repeat family protein
At3g49940	1,39	terpene synthase/cyclase family protein
At5g28560	1,39	SET domain-containing protein / YDG/SRA domain-containing protein
At1g30760	1,39	holocarboxylase synthetase 1 (HCS1)
At1g59910	1,39	cyclic nucleotide-regulated ion channel / cyclic nucleotide-gated channel (CNGC6)
At5g05140	1,39	F-box family protein
At2g40460	1,39	zinc finger (C3HC4-type RING finger) family protein
At1g07705	1,39	FAD-binding domain-containing protein
At5g48110	1,39	protein kinase, putative
At2g21780	1,39	guanylate-binding family protein
At1g51280	1,39	RNA polymerase sigma subunit SigF (sigF) / sigma-like factor (SIG6)
At5g24380	1,39	coatomer protein complex, subunit beta 2 (beta prime), putative
At3g48190	1,39	DNA-directed RNA polymerase family protein
At5g17640	1,39	receptor-like protein CLAVATA2 (CLV2)
At1g69260	1,38	expressed protein
At2g29300	1,38	expressed protein

At3g31970	1,38	DEAD/DEAH box helicase, putative
At3g42630	1,38	E3 ubiquitin ligase SCF complex subunit SKP1/ASK1 (At7), putative
At1g75960	1,38	leucine zipper factor-related
At5g09880	1,38	pseudogene, hypothetical protein
At4g02410	1,38	transcription initiation factor-related
At4g25780	1,38	expansin, putative (EXP12)
At3g16340	1,38	glycine-rich protein
At1g04240	1,38	glycosyl hydrolase family 3 protein
At3g57540	1,38	RNA recognition motif (RRM)-containing protein
At5g46730	1,38	expressed protein
At5g58980	1,38	expressed protein
At5g54510	1,38	universal stress protein (USP) family protein
At5g59820	1,38	3-oxo-5-alpha-steroid 4-dehydrogenase, putative / steroid 5-alpha-reductase, putative
At5g54660	1,38	expressed protein
At2g02710	1,38	U5 small nuclear ribonucleoprotein helicase, putative
At5g50130	1,38	proton-dependent oligopeptide transport (POT) family protein
At5g58120	1,38	WD-40 repeat family protein
At5g60170	1,38	kelch repeat-containing F-box family protein
At2g20145	1,38	D111/G-patch domain-containing protein
At2g36080	1,38	chaperone protein dnaJ-related
At1g79270	1,37	transferase family protein
At5g40380	1,37	cytochrome P450 family protein
At5g49520	1,37	expressed protein
At2g43600	1,37	mitochondrial substrate carrier family protein
At2g23690	1,37	expressed protein
At2g19780	1,37	RelA/SpoT domain-containing protein / calcium-binding EF-hand family protein
At5g62100	1,37	glutaredoxin, putative
At1g02050	1,37	auxin-responsive protein, putative
At3g25400	1,37	protein kinase family protein
At1g64610	1,37	avirulence-responsive family protein / avirulence induced gene (AIG1) family protein
At4g18490	1,37	halotolerance protein (HAL3A)
At1g15080	1,37	auxin-responsive protein / indoleacetic acid-induced protein 19 (IAA19)
At5g62090	1,37	S-adenosyl-L-methionine:carboxyl methyltransferase family protein
At2g35000	1,37	NLI interacting factor (NIF) family protein
At4g19420	1,37	elongation factor Tu / EF-Tu (TUFA)
At5g59220	1,37	Ras-related protein (ARA-4) / small GTP-binding protein, putative
At2g25490	1,37	protein phosphatase 2C, putative / PP2C, putative
At4g10330	1,37	elongation factor 1B-gamma, putative / eEF-1B gamma, putative
At5g67100	1,37	sodium/dicarboxylate cotransporter, putative
At1g62830	1,37	hydroxyproline-rich glycoprotein family protein
At3g45640	1,37	cystathionine gamma-synthase, chloroplast, putative / O-succinylhomoserine (Thiol)-lyase, putative
At4g26090	1,37	hypothetical protein
At1g27770	1,37	calcium-dependent protein kinase, putative / CDPK, putative
At1g62320	1,37	pentatricopeptide (PPR) repeat-containing protein
At3g47510	1,37	leucine-rich repeat transmembrane protein kinase, putative
At1g77000	1,37	expressed protein
At1g72360	1,37	expressed protein
At5g07000	1,37	hypothetical protein
At5g66170	1,37	multi-copper oxidase type I family protein
At1g12880	1,36	membrane bound O-acyl transferase (MBOAT) family protein / wax synthase-related
At1g31120	1,36	protein kinase family protein
At3g06070	1,36	expressed protein
At4g15710	1,36	expressed protein
At2g02310	1,36	dual specificity protein phosphatase family protein
At1g80630	1,36	GRAM domain-containing protein / ABA-responsive protein-related
At1g60200	1,36	protein phosphatase 2C, putative / PP2C, putative
At5g23130	1,36	expressed protein
At1g23390	1,36	transducin family protein / WD-40 repeat family protein
At5g23680	1,36	expressed protein
At3g04200	1,36	auxin-responsive protein, putative
At1g11000	1,36	DEAD/DEAH box helicase, putative
At5g57340	1,36	expressed protein
At1g75250	1,36	pseudouridine synthase family protein
At4g18460	1,36	thioredoxin H-type 1 (TRX-H-1)
At5g49450	1,36	copper-binding family protein
At2g47260	1,36	ATP-dependent Clp protease proteolytic subunit (ClpP1)
At2g22390	1,36	glycerophosphoryl diester phosphodiesterase family protein
At5g03380	1,36	oligopeptide transporter OPT family protein
At2g07722	1,36	mechanosensitive ion channel domain-containing protein / MS ion channel domain
At5g28140	1,36	pyridine nucleotide-disulphide oxidoreductase family protein
At2g33390	1,36	hypothetical protein
At4g16890	1,36	pentatricopeptide (PPR) repeat-containing protein
At2g24550	1,36	N’-5’-phosphoribosyl-formimino-5-aminoimidazole-4-carboxamide ribonucleotide isomerase
At2g22020	1,36	unknown protein
At1g68120	1,36	GDP-4-keto-6-deoxy-D-mannose-3,5-epimerase-4-reductase (GER1)

At2g22860	1,36	expressed protein
At1g11960	1,36	ethylene-responsive factor, putative
At1g80700	1,36	expressed protein
At2g04795	1,35	DNA-binding protein-related
At5g48000	1,35	expressed protein
At4g18640	1,35	transducin family protein / WD-40 repeat family protein
At5g59860	1,35	protein phosphatase 2C, putative / PP2C, putative
At4g15830	1,35	expressed protein
At3g14620	1,35	putative cytochrome P450
At3g48090	1,35	cytochrome b5 domain-containing protein
At5g41810	1,35	fringe-related protein
At1g50740	1,35	actin 1 (ACT1)
At2g28070	1,35	dehydrin xero2 (XERO2) / low-temperature-induced protein LTI30 (LTI30)
At5g24590	1,35	phototropic-responsive NPH3 family protein
At5g48090	1,35	shrunken seed protein (SSE1)
At1g77360	1,35	C2 domain-containing protein
At1g20190	1,35	inositol polyphosphate 6-/3-/5-kinase 2a (IPK2a)
At3g28910	1,35	origin recognition complex subunit 6 family protein (ORC6)
At3g50060	1,35	deoxyhypusine synthase
At3g15950	1,35	LIM domain-containing protein
At3g13980	1,35	expressed protein
At2g27500	1,35	histone H2A, putative
At3g54810	1,35	auxin-responsive family protein
At1g04220	1,35	DNA-binding family protein
At5g01040	1,35	hypothetical protein
At3g47980	1,35	DC1 domain-containing protein / UV-B light-insensitive protein, putative
At4g32880	1,35	DC1 domain-containing protein
At3g01580	1,35	expressed protein
At5g65570	1,35	hypothetical protein
At3g55840	1,34	peroxin-3 family protein
At2g38320	1,34	emsy N terminus domain-containing protein / ENT domain-containing protein
At3g61060	1,34	transcription activation domain-interacting protein-related
At4g25820	1,34	expressed protein
At1g32780	1,34	hypothetical protein
At3g25570	1,34	DNA repair protein RAD23, putative
At5g57140	1,34	acyl-(acyl-carrier-protein) desaturase, putative / stearoyl-ACP desaturase, putative
At2g40140	1,34	myb family transcription factor
At5g08370	1,34	alpha-galactosidase 2 (AGAL2)
At5g46410	1,34	SWIM zinc finger family protein
At4g40000	1,34	DC1 domain-containing protein
At5g38280	1,34	Ulp1 protease family protein
At2g26360	1,34	hydroxyproline-rich glycoprotein family protein
At3g27620	1,34	heavy-metal-associated domain-containing protein
At3g02100	1,34	proton-dependent oligopeptide transport (POT) family protein
At3g62740	1,34	expressed protein
At3g25610	1,34	expressed protein
At1g70740	1,34	protein kinase family protein
At4g08110	1,34	hypothetical protein
At1g74430	1,34	succinyl-CoA ligase (GDP-forming) beta-chain, mitochondrial, putative / succinyl-CoA synthetase
At3g55940	1,34	RNA recognition motif (RRM)-containing protein
At1g21910	1,34	Ras-related GTP-binding protein, putative
At1g77270	1,34	beta-glucosidase (PSR3.2)
At4g28290	1,34	cyclin, putative
At4g15975	1,34	expressed protein
At5g05730	1,34	S-adenosyl-L-methionine:carboxyl methyltransferase family protein
At1g49660	1,34	galactinol synthase, putative
At1g64660	1,34	hypothetical protein
At4g33000	1,33	cytochrome b5 domain-containing protein
At3g27650	1,33	disease resistance protein (TIR-NBS-LRR class), putative
At3g19190	1,33	2-oxoglutarate-dependent dioxygenase (AOP1.2)
At1g78550	1,33	AP2 domain-containing transcription factor, putative
At5g16000	1,33	expressed protein
At3g16010	1,33	calcium-transporting ATPase 4, endoplasmic reticulum-type (ECA4)
At1g57670	1,33	glycosyl hydrolase family 79 N-terminal domain-containing protein
At2g37950	1,33	hAT dimerisation domain-containing protein
At1g74660	1,33	protein kinase family protein
At1g16040	1,33	nucleosome assembly protein (NAP), putative
At1g02220	1,33	S-adenosyl-L-methionine:carboxyl methyltransferase family protein
At4g32350	1,33	expressed protein
At2g39980	1,33	expressed protein
At3g54330	1,33	expressed protein
At3g57170	1,33	leucine-rich repeat protein kinase, putative
At3g14050	1,33	acetolactate synthase small subunit, putative
At1g32920	1,33	lipase-related
At2g06230	1,33	pathogen-responsive alpha-dioxygenase, putative

At1g70850	1,33	lactose permease-related
At5g50450	1,33	Dof-type zinc finger domain-containing protein
At4g28330	1,33	ankyrin repeat family protein
At1g17190	1,33	phosphofructokinase family protein
At1g72140	1,33	expressed protein
At3g48100	1,33	serine carboxypeptidase S10 family protein
At4g37770	1,33	expressed protein
At2g33170	1,33	expressed protein
At4g38680	1,33	DC1 domain-containing protein
At4g26320	1,33	expansin, putative (EXP22)
At1g79520	1,33	expressed protein
At1g72520	1,33	endonuclease/exonuclease/phosphatase family protein / calcium-binding EF hand family protein
At4g36040	1,33	expressed protein
At5g18130	1,33	expressed protein
At3g60760	1,33	auxin-responsive protein, putative
At4g15660	1,33	sterol desaturase family protein
At3g42370	1,33	unknown protein
At1g72500	1,33	expressed protein
At2g22010	1,33	lecithin:cholesterol acyltransferase family protein / LACT family protein
At3g51950	1,33	zinc finger (C3HC4-type RING finger) family protein
At1g22570	1,33	expressed protein
At1g24030	1,33	Protein kinase superfamily protein
At5g42200	1,33	pentatricopeptide (PPR) repeat-containing protein
At4g33985	1,33	50S ribosomal protein L12-1, chloroplast (CL12-A)
At4g17490	1,33	2-oxoacid-dependent oxidase, putative
At1g19270	1,33	disease resistance protein-related
At1g79910	1,33	dihydrolipoamide S-acetyltransferase, putative
At5g47720	1,33	uracil phosphoribosyltransferase, putative / UMP pyrophosphorylase, putative / UPRTase, putative
At3g21070	1,33	proline-rich family protein
At5g58690	1,33	60S ribosomal protein L10 (RPL10B)
At5g66300	1,33	ammonium transporter 1, member 1 (AMT1.1)
At1g75230	1,33	expressed protein
At4g13345	1,33	expressed protein
At2g16340	1,33	expressed protein
At5g52760	1,33	expressed protein
At3g46930	1,33	calmodulin, putative
At4g27654	1,32	disease resistance protein (NBS-LRR class), putative
At2g22300	1,32	expansin-related
At1g27730	1,32	auxin-responsive family protein
At2g21890	1,32	expressed protein
At5g60220	1,32	aspartyl protease family protein
At1g10650	1,32	brassinosteroid signalling positive regulator, putative
At4g35970	1,32	zinc finger (C3HC4-type RING finger) family protein
At4g18530	1,32	expressed protein
At5g43380	1,32	lesion inducing protein-related
At4g14650	1,32	photosystem I reaction center subunit II, chloroplast, putative/photosystem I 20 kDa subunit, putative
At1g80170	1,32	glycosyl hydrolase family protein 17
At2g22330	1,32	kelch repeat-containing F-box family protein
At5g26734	1,32	RNA recognition motif (RRM)-containing protein
At5g17240	1,32	expressed protein
At3g05220	1,32	AMP-dependent synthetase and ligase family protein
At1g78280	1,32	myb family transcription factor
At1g10060	1,32	glycosyl hydrolase family 17 protein
At2g32660	1,32	hypothetical protein
At5g51350	1,32	nucleoside diphosphate kinase 3, mitochondrial (NDK3)
At5g02290	1,32	expressed protein
At3g23470	1,32	transferase family protein
At5g66640	1,32	zinc finger (C3HC4-type RING finger) family protein
At4g14580	1,32	xyloglucan:xyloglucosyl transferase, putative / xyloglucan endotransglycosylase, putative
At3g62860	1,32	alpha/beta-Hydrolases superfamily protein
At1g15940	1,32	glycine-rich protein
At5g14940	1,32	glycosyl hydrolase family protein 17
At4g25410	1,32	protein kinase family protein
At5g04300	1,32	phosphoglycerate/bisphosphoglycerate mutase family protein
At1g63930	1,32	auxin-responsive family protein
At2g13370	1,32	hypothetical protein
At2g15070	1,32	Ulp1 protease family protein
At1g75800	1,32	protein kinase family protein
At3g06210	1,32	expressed protein
At3g07280	1,32	photosystem II core complex proteins psbY, chloroplast (PSBY) / L-arginine metabolising enzyme
At1g03905	1,32	expressed protein
At5g19530	1,32	UbiA prenyltransferase family protein
At2g24530	1,32	integral membrane transporter family protein
At3g61000	1,32	hAT dimerisation domain-containing protein
At1g18570	1,31	expressed protein

At5g50530	1,31	emp24/gp25L/p24 protein-related
At3g24530	1,31	zinc finger (C2H2 type) family protein (ZFP3)
At1g67720	1,31	sterol desaturase family protein
At3g15770	1,31	dehydration-responsive protein-related
At5g01510	1,31	glycine-rich RNA-binding protein
At5g15460	1,31	ABC transporter family protein
At3g04640	1,31	protein kinase family protein
At5g46480	1,31	Ras-related GTP-binding protein, putative
At1g23710	1,31	fibronectin type III domain-containing protein
At5g18630	1,31	zinc finger (C2H2 type) family protein
At1g02670	1,31	zinc finger (C3HC4-type RING finger) family protein
At1g68050	1,31	pectinesterase family protein
At1g67870	1,31	hypothetical protein
At1g74840	1,31	myb family transcription factor (GLK2)
At3g17770	1,31	Ras-related GTP-binding family protein
At4g37540	1,31	2-oxoglutarate-dependent dioxygenase, putative (AOP2)
At5g01380	1,31	expressed protein
At1g21450	1,31	disease resistance protein (CC-NBS-LRR class), putative
At5g18970	1,31	hypothetical protein
At1g18330	1,31	homeobox-leucine zipper protein 22 (HAT22) / HD-ZIP protein 22
At4g13020	1,31	DNAJ heat shock N-terminal domain-containing protein
At4g26260	1,31	calcineurin-like phosphoesterase family protein
At5g24150	1,31	protease inhibitor, putative
At2g35930	1,31	emsy N terminus domain-containing protein / ENT domain-containing protein
At1g70530	1,31	expressed protein
At5g52510	1,31	expressed protein
At5g18600	1,31	cysteine synthase, chloroplast / O-acetylserine (thiol)-lyase / O-acetylserine sulfhydrylase (OASB)
At5g37230	1,31	expressed protein
At4g11950	1,31	glycosyl transferase family 48 protein
At1g29690	1,31	zinc finger (C3HC4-type RING finger) family protein
At5g67550	1,31	expressed protein
At2g02850	1,31	WRKY family transcription factor
At2g29490	1,31	expressed protein
At4g30490	1,31	Ulp1 protease family protein
At3g24550	1,31	pectinesterase family protein
At4g23060	1,31	MATE efflux family protein
At3g22280	1,31	leucine-rich repeat family protein
At1g19180	1,31	expressed protein
At2g17030	1,31	thioredoxin-related
At3g06940	1,31	hypothetical protein
At3g17250	1,31	expressed protein
At2g36450	1,31	hypothetical protein
At3g19770	1,31	NHL repeat-containing protein
At2g23420	1,31	galactosyltransferase family protein
At5g04010	1,31	exportin 1, putative
At5g45110	1,31	sucrose transporter, putative / sucrose-proton symporter, putative
At4g35720	1,31	UDP-glucoronosyl/UDP-glucosyl transferase family protein
At1g56310	1,30	diacylglycerol kinase family protein
At4g07670	1,30	hypothetical protein
At1g62570	1,30	calcium-transporting ATPase, putative
At4g08500	1,30	leucine-rich repeat family protein / protein kinase family protein
At1g56145	1,30	terpene synthase/cyclase family protein
At5g51190	1,30	PHD finger transcription factor, putative
At3g15540	1,30	MATE efflux family protein
At1g64620	1,30	expressed protein
At3g16790	1,30	ovule development protein, putative
At3g08800	1,30	pentatricopeptide (PPR) repeat-containing protein
At4g24740	1,30	expressed protein
At1g56700	1,30	cyclin delta-3 (CYCD3)
At4g04830	1,30	disease resistance protein (TIR-NBS-LRR class), putative
At5g63830	1,30	expressed protein
At2g39240	1,30	adenylosuccinate synthetase (ADSS)
At2g23540	1,30	beta-amylase, putative / 1,4-alpha-D-glucan maltohydrolase, putative
At4g18880	1,30	expressed protein
At1g09060	1,30	basic helix-loop-helix (bHLH) family protein
At4g01090	1,30	hypothetical protein
At1g06440	1,30	expressed protein
At3g15450	1,30	phospholipase D gamma 2 / PLD gamma 2 (PLDGAMMA2)
At1g29820	1,30	CACTA-like transposase family (Ptta/En/Spm)
At3g22810	1,30	DEAD/DEAH box helicase, putative
At5g63790	1,30	phosphatidate cytidylyltransferase family protein
At5g01770	1,30	single-strand-binding family protein
At2g01530	1,30	dentin sialophosphoprotein-related
At3g25840	1,30	glycosyl hydrolase family 38 protein
At4g37590	1,30	ABC transporter family protein

At3g14630	1,30	splicing factor RSZ33 (RSZ33)
At1g64460	1,30	expressed protein
At4g26220	1,30	S-adenosyl-L-methionine-dependent methyltransferases superfamily protein
At1g76360	1,30	hypothetical protein
At3g62270	1,30	appr-1-p processing enzyme family protein
At1g10010	1,30	short vegetative phase protein (SVP)
At1g69080	1,30	DNAJ heat shock N-terminal domain-containing protein
At5g58787	1,30	C2 domain-containing protein
At1g19770	1,30	phytochrome kinase substrate-related
At1g20100	1,30	metallo-beta-lactamase family protein
At5g64240	1,30	cysteine protease inhibitor family protein / cystatin family protein
At4g22610	1,30	auxin-responsive GH3 family protein
At1g21410	1,30	expressed protein
At2g29600	1,30	protein kinase family protein
At2g02370	1,30	CBL-interacting protein kinase 21, putative (CIPK21)
At5g61150	1,30	hypothetical protein
At5g07100	1,30	expressed protein
At5g60290	1,30	ABC transporter family protein
At4g39950	1,30	SNF2 domain-containing protein / helicase domain-containing protein
At1g13880	1,30	glycine-rich protein
At3g17120	1,30	expressed protein
At3g55630	1,30	GCN5-related N-acetyltransferase (GNAT) family protein
At2g19120	1,30	WWE domain-containing protein / ceo protein, putative (CEO)
At2g39110	1,30	2-oxoglutarate-dependent dioxygenase, putative
At3g17690	1,30	pentatricopeptide (PPR) repeat-containing protein
At5g28520	1,30	expressed protein
At1g05460	1,30	Encodes a protein with similarity to RNA helicases
At4g28720	1,30	Auxin biosynthetic gene regulated by RVE1
At2g39420	1,30	F-box family protein (FBX14)
At3g06020	1,30	mitochondrial import inner membrane translocase subunit Tim17/Tim22/Tim23 family protein
At1g67530	1,30	expressed protein
At4g13030	1,30	pentatricopeptide (PPR) repeat-containing protein
At2g44480	1,30	no apical meristem (NAM) family protein
At2g22930	1,30	ABA-responsive element-binding protein 2 (AREB2)
